# Microsatellite Genome-Wide Database Development for the Commercial Blackhead Seabream (*Acanthopagrus schlegelii*)

**DOI:** 10.3390/genes14030620

**Published:** 2023-03-01

**Authors:** Xinhui Luo, Lichun Zhang, Songlin Chen

**Affiliations:** 1 Institute of Animal Biotechnology, Jilin Academy of Agricultural Sciences, Changchun 130033, China; 2 Key Laboratory for Sustainable Development of Marine Fisheries, Ministry of Agriculture, Yellow Sea Fisheries Research Institute, Chinese Academy of Fishery Sciences, Qingdao 266071, China; 3 Key Laboratory for Marine Fishery Biotechnology and Genetic Breeding, Laboratory for Marine Fisheries Science and Food Production Processes, Pilot National Laboratory for Marine Science and Technology, Qingdao 266071, China

**Keywords:** microsatellite database, genome-wide, whole genome resequencing, *Acanthopagrus schlegelii*

## Abstract

Simple sequence repeats (SSRs), the markers with the highest polymorphism and co-dominance degrees, offer a crucial genetic research resource. Limited SSR markers in blackhead seabream have been reported. The availability of the blackhead seabream genome assembly provided the opportunity to carry out genome-wide identification for all microsatellite markers, and bioinformatic analyses open the way for developing a microsatellite genome-wide database in blackhead seabream. In this study, a total of 412,381 SSRs were identified in the 688.08 Mb genome by Krait software. Whole-genome sequences (10×) of 42 samples were aligned against the reference genome and genotyped using the HipSTR tools by comparing and counting repeat number variation across the SSR loci. A total of 156,086 SSRs with a 2–4 bp repeat were genotyped by HipSTR tools, which accounted for 55.78% of the 2–4 bp SSRs in the reference genome. High accuracy of genotyping was observed by comparing HipSTR tools and PCR amplification. A set of 109,131 loci with a number of alleles ≥ 3 and with a number of genotyped individuals ≥ 6 were reserved to constitute the polymorphic SSR database. Fifty-one polymorphic SSR loci were identified through PCR amplification. This strategy to develop polymorphic SSR markers not only obtained a large set of polymorphic SSRs but also eliminated the need for laborious experimental screening. SSR markers developed in this study may facilitate blackhead seabream research, which lays a certain foundation for further gene tagging and genetic linkage analysis, such as marker-assisted selection, genetic mapping, as well as comparative genomic analysis.

## 1. Introduction

Blackhead seabream (*Acanthopagrus schlegelii*) mainly distributes on the West Pacific coast from Japan and Korea, to the East China Sea. As the ecological and commercial importance, this species has been cultured successfully in China as well as in Japan. Notably, concern over the rapid decline in blackhead seabream stocks is growing because the wild and cultured population declined from 2000–2015, according to the Fisheries Statistical Yearbook of Taiwan [1]. As a result, various measures have been taken to recover the blackhead seabream populations in Japan and some areas of China, which included fishery supplementation of the open waters and fishing restrictions [2,3]. The development of molecular markers is needed to establish sustainable conservation strategies aimed at maintaining the species' genetic diversity.

Simple sequence repeats (SSRs), also known as microsatellite markers, are DNA sequences consisting of short, consecutively repeated mono-, di-, tri-, tetra-, penta-, or hexa-nucleotide motifs. SSRs are distributed ubiquitously throughout the genomes of eukaryotes, being one of the most informative molecular markers for population genetic studies due to their high frequency, wide distribution, polymorphism, and codominance in genomes [4]. Microsatellite markers have been used as genetic tools in fish for linkage map construction [5,6], assessment of populations’ genetic diversity [7], parentage determination [8], and a genome-wide association study [9]. 

So far, previous studies have focused on the development of a limited number of SSRs markers of blackhead seabream. Mao et al. and Yang et al. [10,11] obtained eight and thirteen polymorphic loci from the enriched genomic library, respectively. Liu et al. [12] tested 16 polymorphic microsatellite loci in the species of blackhead seabream by cross-species amplification. Forty-nine highly polymorphic microsatellite markers were developed based on specific-locus amplified fragment sequencing (SLAF-seq) technology [13]. Wide availability and the reasonable costs of the next-generation sequencing (NGS) technology and application of the genome survey tools have greatly facilitated the exploration of the genomic information and development of molecular markers for fishes. Recently, some tools have been developed for the identification, analysis, and extraction of SSR markers by using NGS data (lobSTR [14], STRait Razor [15], toaSTR [16], and HipSTR [17]). 

The limited number of molecular markers hindered the genetic structure analysis and resource conservation of blackhead seabream. In this study, a larger set of genome-wide polymorphic SSR markers were identified by whole genome resequencing of 42 samples and HipSTR software, validating a subset of markers following PCR amplification and electrophoresis. The present study is the first comprehensive report on mining SSR markers in the blackhead seabream genome. Identifying a large set of SSR markers using this approach provides a cost and resource-efficient alternative to the traditional ones, which involves random testing of primer pairs designed from the genome or transcriptome sequences. The large set of polymorphic microsatellite loci identified in this study will contribute to the investigation of genetic diversity and stock enhancement monitoring in blackhead seabream and its closely related fishes. 

## 2. Materials and Methods

### 2.1. Genome-Wide SSRs Analysis of the Blackhead Seabream

The reference genome sequences of blackhead seabream were downloaded from the GigaScience Database (http://gigadb.org/dataset/100409, accessed on 26 October 2022) [18]. Microsatellite motif identification and its flanking regions were performed using the Krait v.1.3.3 software [19]. The search criteria consisted of the following parameters: 12, 6, 5, 4, 3, and 3 repeats for mono-, di-, tri-, tetra-, penta-, and hexanucleotide motifs, respectively. Compound SSRs were defined as those with an interval of less than 100 bp between two repeat motifs. For simplicity, those repeats with unit patterns being circular permutations and reverse complements were grouped as one type. For instance, AGG denotes AGG, GGA, GAG, CCT, CTC, and TCC in different reading frames or on the complementary strand. Relative frequency (SSRs per megabase pair (Mbp)) and relative density (SSRs in base pairs (bp) per Mb) were used for comparisons between different repeat types.

### 2.2. Genome Resequencing and Construction of Polymorphic SSR Database

Forty-two individuals of blackhead seabream were collected from the east coast of China, including thirty-two cultured individuals from Jiangsu, Zhejiang, Fujian, and Guangdong provinces, and ten wild individuals from the South China Sea. DNA for whole genome resequencing was extracted from the fin tissue of 42 individuals using the TIANamp Marine Animals DNA Kit (Tiangen, Cat. DP324, Beijing, China). Genomic libraries were constructed using the TruSeq Library Construction Kit (Illumina Inc., San Diego, CA, USA) with an insert size of approximately 350 bp, sequenced as 2 × 150 bp paired-end reads on the Illumina NovaSeq 6000 machine, with an average mean depth of 10× per sample by LC-BIO Co., Ltd. (Hangzhou, China). The Illumina NGS platform specifies that high-quality reads should have a Q20 of at least 90% and a Q30 of at least 85%.

The reference genome assembly and the whole genome resequencing data provided opportunities for identifying polymorphic microsatellite loci rapidly. Initially, the paired-end reads of each of the 42 samples were mapped to the reference genome using BWA v.0.7.17 (mem option). Next, a bed file was prepared for each of the SSR loci that contained the scaffold name, start and end positions of SSR loci, motif length, the number of repeat units in the reference sequence, and the SSR locus ID. The aligned bam files of the 42 samples, the bed file containing the SSR regions in the reference genome, and the reference SSR scaffold were used to genotype by the HipSTR program.

The HipSTR program was run using Mode 1 (De novo stutter estimation and SSR calling with de novo allele generation). The HipSTR-generated VCF files with SSR calls were filtered for low-quality calls using the HipSTR option: min call qual: 0.9; max call flank indel: 0.15; max call stutter: 0.15; min call allele bias: −2; min call strand bias: −2. Furthermore, the genotype calls were filtered for monomorphic and high missing rates among the samples and non-reference alleles on less than six samples. Microsatellite loci with the number of alleles ≥ 3 and the number of genotyped individuals ≥ 6 were reserved to constitute the polymorphic SSR database.

### 2.3. Microsatellite Marker Analysis

First, a (GT) n-enriched genomic library was constructed by the FIASCO (Fast Isolation by AFLP of Sequences Containing Repeats) protocol [20], and 30 polymorphic loci screened from 30 cultured individuals were deposited on GenBank (GU206877–GU206906). In addition, 21 loci with at least 3 alleles were randomly selected for polymorphism verification according to the polymorphic SSR database.

Genomic DNA was extracted from the fin tissue of 30 individuals from five different populations and then diluted to 50 ng/μL. Fifty-one primer pairs were designed by Primer Premier 5.0 software. Nested PCRs were performed in the three-primer system multiplexes according to the protocol described by Schuelke [21]. Briefly, an M13F-forward primer with the 5′ tail sequence of 5′-TGTAAAACGACGGCCAGT-3′ was used in combination with an M13F primer labeled with a fluorescent dye at the 5′ end. Briefly, 20 µL PCR reactions containing 1 µL of genomic DNA (50 ng/µL), 10 µL of 2× Flash PCR MasterMix (Dye) (CoWin Biotech, Cat. CW3009H, Beijing, China), 4.5 µL of dd H_2_O, 2 µL of fluorescent-labeled M13F primer (10 µM) (5′-FAM, 5′-HEX.) (Azenta Co., Ltd., Suzhou, China), 0.5 µL of M13F-forward primer (10 µM), and 2 µL of reverse primer (10 µM) were carried out. A BIO-RAD T100 thermal cycler was used for denaturation with 3 min at 94 °C; then 30 cycles at 94 °C for 30 s, 50–60 °C (Table 1) for 40 s, and 72 °C for 40 s, followed by another 8 cycles at 94 °C for 30 s, 53 °C for 45 s, and 72 °C for 45 s, followed by a final extension of 10 min at 72 °C. PCR products were electrophoresed on an ABI3730xl DNA analyzer (Life Technologies, Carlsbad, CA, USA) or on 6% denaturing polyacrylamide gels using silver staining.

### 2.4. Data Analysis

The raw results of the samples’ genotyping were examined with GeneMarker HID v.1.90 Software [22]. The allelic diversity and genetic variation parameters, including the number of different alleles (Na), the index of observed heterozygosity (Ho), expected heterozygosity (He), and Hardy–Weinberg equilibrium (HWE), were calculated by GenAlEx v. 6.5 software [23]. The possibility of scoring errors, allelic dropout, and null alleles were performed using the Micro-Checker v.2.2.3 software [24]. The polymorphic information contents (PIC) were calculated by the PowerMarker v.3.25 software [25]. Spearman's correlation analysis was used to find out the correlation between the number of microsatellites and scaffold length by IBM SPSS Statistics v.26.0.0 software. 

## 3. Results

### 3.1. Identification of Genome-Wide SSRs 

A total of 412,381 microsatellite markers were identified in the 688.08 Mb genome of blackhead seabream; these loci were distributed on 31,359 scaffolds, and compound SSRs (56,667) constituted 13.74% of the total SSRs. The total length of the identified SSRs was 8,513,865 bp, accounting for 1.24 % of the reference genome length. The average length of SSR blocks was 20.65 bp, and the relative abundance and density were 628.17 loci/Mb and 12,968.89 bp/Mb, respectively. 

**Figure 1:**
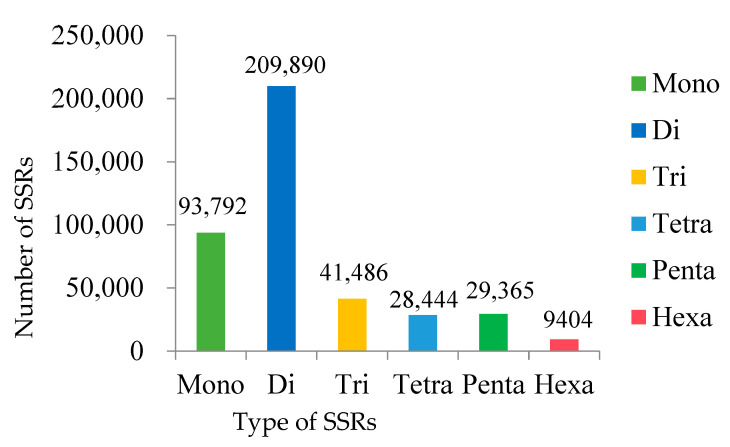
Distribution of SSRs in the reference genome of blackhead seabream.

**Figure 2:**
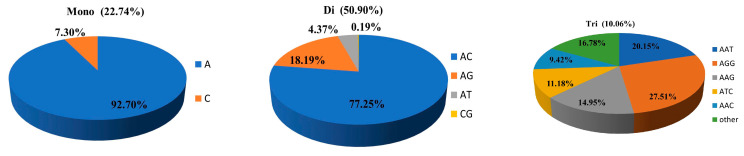

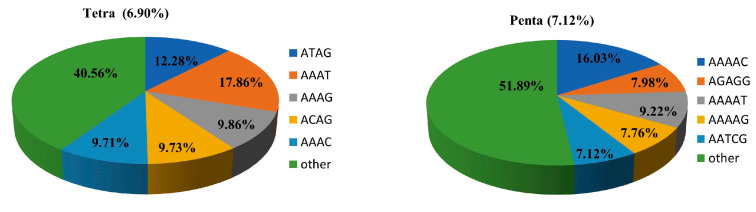
Frequency distribution of different repeat motifs in the reference genome of blackhead seabream.

The dinucleotides were the most abundant (209,890) with a proportion of 50.90%, followed by mononucleotides (93,792; 22.74%), trinucleotides (41,486; 10.06%), pentanucleotides (29,365; 7.12%), tetranucleotides (28,444; 6.90%), and hexanucleotides (9404; 2.28%) (Figure 1 and Figure 2). The highest frequency and density were di- (319.72 loci/Mb, 7238.89 bp/Mb), followed by mono- (142.87 loci/Mb, 2179.84 bp/Mb), tri- (63.19 loci/Mb, 1422.2 bp/Mb), tetra- (43.33 loci/Mb, 1045.27 bp/Mb), penta- (44.73 loci/Mb, 801.71 bp/Mb), and hexa-nucleotides (14.32 loci/Mb, 280.98 bp/Mb) (Supplementary Table S1).

Among the mononucleotide types, A predominated heavily (92.70%) (Figure 2). In dinucleotide SSR loci, repetitive microsatellites AC (77.25%) was the most abundant repeat motif, and CG was the least abundant (0.19%). AGG was the most abundant trinucleotide motif (27.51%), followed by AAT (20.15%), AAG (14.95%), ATC (11.18%), and AAC (9.42%). For tetranucleotide repeats, the most frequent motif was AAAT (17.86%), followed by ATAG (12.28%), AAAG (9.86%), ACAG (9.73%), and AAAC (9.71%). The two main types of pentonucleotide motifs were AAAAC (16.03%) and AAAAT (9.22%). Hexanucleotide SSR loci contained more motif types, each with a relatively small percentage (Supplementary Table S2). The AC motif was the type with the highest number of repeats, microsatellite length, proportion of repeat types, average length, and relative abundance density. The longest and highest number of SSR present at a locus was a string of 825 AGG repeats, with 2475 bp in scaffold 129. 

**Figure 3:**
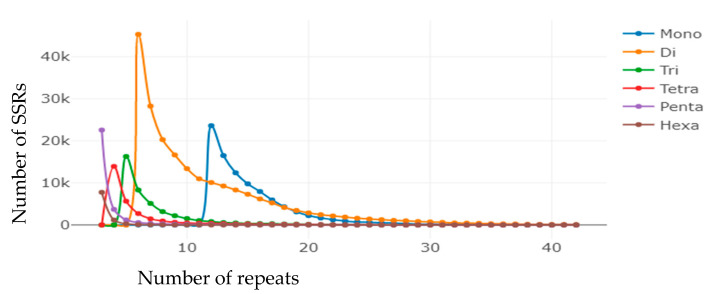
Distribution of SSR repeat number for each SSR type in blackhead seabream.

The copy number of mononucleotide repeats was mostly concentrated in 12–23 times, accounting for 95.63% (89,696) of the total number of mono-nucleotide SSRs, of which 12 repeats accounted for 25.16% (23,600) (Figure 3). The copy number of dinucleotide repeats ranged from 6 to 20 times, which accounted for 91.37% (191,786) of the total dinucleotide SSRs, and 6 repeat types were the most abundant and accounted for 21.60% (45,329). The copy number of trinucleotide repeats was mainly concentrated in 5–12 times, accounting for 92.67% (38,444) of the total number of trinucleotide SSRs, of which 5 repeats were the most, accounting for 39.24% (16,281) of the total. The copy number of tetranucleotide repeats was largely concentrated in 4–10 times, accounting for 90.70% (25,798) of the total number of tetranucleotide SSRs, of which 4 repeats were the most and accounted for 48.95% (13,923) of the total number. The copy number of pentanucleotide repeats was mostly concentrated in 3–6 times, accounting for 95.54% (28,054) of the total number of pentanucleotide SSRs, of which 3 repeats accounted for 76.87% (22,574). The 3–6 times copy number of hexanucleotide repeats dominated heavily, which accounted for 98.90% (9301) of the total hexanucleotide SSRs. The number of three repeats was the largest, accounting for 82.60% (7767) of the total number of hexanucleotide SSRs (Supplementary Table S3).

### 3.2. Identification of Genome-Wide Polymorphic SSRs

Whole genome resequencing of the 42 samples using Illumina sequencing technology generated an average depth of 10× per individual and an average genome coverage of 90.61%, assuming a genome size of 688.08 Mbp. The sequencing depth of 42 samples ranged from 7.74 to 12.60, with an average depth of 9.06. The Q20 values were greater than 95.60%, with an average GC content of 42.43%.

A batch of 156,086 matched the criteria set with a 2–4 bp repeat SSR via HipSTR to call genotype from 42 samples, which accounted for 55.78% of the 2–4 bp SSR in the reference genome (Supplementary Table S5). We evaluated the coverage of 156,086 SSR loci in the whole genome. Briefly, 156,086 SSR loci were distributed among 117 of a total of 31,359 scaffolds (634.17 Mbp), which accounted for 92.10% of the genome size. Examination of Spearman’s correlation coefficients suggested that the number of microsatellites and scaffold length were highly correlated. (Spearman’s correlation test, r = 1.0, *p* < 0.001) (Supplementary Table S4). We calculated the maximum length of SSRs identified by HipSTR, in which the longest motif for a dinucleotide was (TG)_38_ loci, which was located at scaffold 88, start point 1,576,858, and the longest motif length was 86 bp. The proportion of dinucleotide motifs with lengths ≤ 86 bp in the genome was 99.84% (209,563). The longest trinucleotide motifs were (AGA)_22_ loci, which were located at scaffold 88, start point 1,598,852, and a motif length of 93 bp. The proportion of trinucleotide motifs with repeat length ≤ 93 bp in the genome was 99.81% (41,407). (TAGA)_19_ and (TCTA)_20_ were the longest motifs for a tetranucleotide with lengths of 104 bp, which were located at scaffold 11 start point 2,335,928, and scaffold 67 start point 5,444,193, respectively. The proportion of tetranucleotide motif length with ≤104 bp in the genome was 99.74% (28,371). These results indicated that the reference genome SSR for the 2–4 bases has been identified based on the paired-end sequencing data and HipSTR tools. 

A total of 119,347 dinucleotide repeats were successfully called, accounting for 56.86% of the total dinucleotide repeats in the reference genome (Supplementary Table S5). Moreover, 113,226 dinucleotide repeats with copy numbers ranging from 6 to 20 repeats accounted for 94.87% of the total number of dinucleotide repeats successfully called. Due to the increase in the number of repeats in dinucleotides, the ratio of microsatellite loci successfully called accounted for 55.05% to 62.79% of the repeats, and the ratio of different repeats was similar, with an average of 59.04%. A total of 22,161 trinucleotide repeats were successfully called, accounting for 53.42% of all trinucleotide repeats in the reference genome. Moreover, 21,477 trinucleotide repeats with copy numbers ranging from 5 to 12 repeats accounted for 96.91% of all trinucleotide repeats called successfully. The ratio of called microsatellite loci decreased as the number of repeat units increased, accounting for 41.43% to 58.97% of corresponding repeats, with an average of 55.87%. The SSRs with tetranucleotide repeats were found in 14,578, accounting for 51.25% of all tetranucleotide repeats. The total number of SSR markers, 13,873, ranging from 4 to 10, accounted for 95.16% of all tetranucleotide repeats called successfully. Along with the increase in repeat times, the ratio of called microsatellite loci decreased slightly, accounting for 47.23% to 56.96% of corresponding repeats, with an average of 53.76%. In general, the average proportions of successful calls of di-, tri-, and tetra-nucleotides were 59.04%, 55.87%, and 53.76%, respectively.

For each SSR locus, the number of genotyped individuals ranged from 1 to 42 (Supplementary Table S6). A total of 13,505 (8.65%) loci with the number of genotyped individuals ≤ 5. The number of loci increased as the number of genotyped individuals ranged from 6 to 18. When the number of genotyped individuals was 19 to 20, the number of microsatellite loci was 6783 and 6701. When the number of genotyped individuals ranged from 21 to 42, the number of microsatellite loci showed a downward trend. There were 1804 (1.16%) loci with ≥35 genotyped individuals, including 2 loci with 42 genotyped individuals. The average number of genotyped individuals per locus was 17.62.

Among 156,086 SSRs, the number of alleles ranged from 1 to 27, and 21 loci with at least 3 alleles were selected randomly and validated among 30 individuals by PCR amplification and capillary electrophoresis. The results showed that the number of alleles per locus ranged from 3 to 15, with a mean of 8.48 alleles per locus (Table 2). The polymorphism rate for each SSR marker was 100%. We tested the matching degree of the number of alleles between PCR (Na-PCR) and HipSTR (Na-HipSTR) of 87 known polymorphic SSRs (Supplementary Table S7) [26]. A total of 78 polymorphic loci were located in the genome; 58 were matched to HipSTR-Na; the remaining loci failed to be matched due to base mutations in the flanking regions around the motifs or the absence of the SSR locus in the database. Five polymorphic loci with Na-PCR values = 2 had Na-HipSTR values of 2, 3, 2, 13, and 2, respectively. Two polymorphic loci with Na-PCR values = 3 had Na-HipSTR values of 4 and 2, respectively. Fifty-one polymorphic loci with Na-PCR values ≥ 4 had Na-HipSTR values of ≥3; of which 26 SSR loci with the number of genotyped individuals ≥ 5, the Na-HipSTR values ranged from 5 to 18. These results suggested that conventional PCR protocols and HipSTR tools had a high consistency in identifying microsatellite polymorphisms. 

In order to verify the accuracy of HipSTR tools for genotyping SSRs, electrophoretic analysis results of the same individuals in 22 pairs of primers were compared to HipSTR (Supplementary Table S8). PCR and capillary electrophoresis determined that 146 were heterozygotes and 67 were homozygotes in the 213 genotype. We found that the 191 genotype of the HipSTR was in agreement with PCR. The accuracy of HipSTR was determined to be 89.67%. We calculated the polymorphic information content (PIC) of 63 microsatellite loci, with the Na-HipSTR values ranging from 3 to 7. The PIC values ranged from 0.3539 to 0.7936. Fifty-two out of the sixty-three markers were highly polymorphic (PIC > 0.5), and the remaining eleven polymorphic loci were of medium polymorphism degree (0.25 ≤ PIC < 0.5) (Supplementary Table S9).

Finally, a set of 109,131 loci with the number of alleles ≥ 3 and the number of genotyped individuals ≥ 6 was reserved to constitute the polymorphic SSR database and classified according to the number of alleles and the type of duplicated repeats. The average number of Na-HipSTR alleles at these loci was 6.92, and the average number of genotyped individuals was 17.65. Among these loci, the motif types of microsatellites included 80.46% (87,807) dinucleotides, 12.40% (13,537) trinucleotides, and 7.14% (7787) tetranucleotides. Moreover, 17,040 SSRs with 3 alleles, 27,232 with the number of alleles 4 to 5, and 64,859 with the number of alleles ≥ 6, respectively, accounted for 15.61%, 24.95%, and 59.43% of the polymorphic SSR database. The full SSR database can be found in the Supplementary Information (Supplementary Table S10), including ID, scaffold number, starting point, repeating unit, number of genotyped individuals, and number of alleles at the SSR locus.

### 3.3. Identification of Polymorphic SSRs Using PCR Amplification

In this study, a total of 51 microsatellite markers were developed by PCR amplification from 30 individuals, of which 30 loci were identified among 83 sequences, which were sequenced from 148 positive clones from a (GT)n-enriched genomic library. The remaining 21 polymorphic loci with at least 3 alleles were validated by 21 potential microsatellite loci randomly selected from the database, with a polymorphism ratio of 100%. The number of alleles per locus (Na) ranged from 2 to 15 (mean 6.84); observed heterozygosity (Ho) ranged from 0.0667 to 0.9000 (mean 0.5636), and expected heterozygosity (He) ranged from 0.0655 to 0.9122 (mean 0.6642) (Table 2). The PIC values ranged from 0.0624 to 0.8896 (mean 0.6224). Forty-one out of the fifty-one markers were highly polymorphic loci (PIC > 0.5), five markers were of medium polymorphism degree (0.25 ≤ PIC < 0.5), and five markers were low polymorphism sites (PIC < 0.25). (Table 2). Botstein et al. [27] reported that markers with He > 0.6 and PIC > 0.5 were the most reliable for population genetic studies and molecular breeding programs. Based on this information, these 40 polymorphic markers (highlighted in gray) could have sufficient discriminating power to distinguish among individuals and breeds. 15 loci (acse121, acs199, acse54, acs22, acse93, acs4, AS1, AS4, AS5, AS9, AS10, AS14, AS25, AS28, and AS36) departed from the HWE, and the remaining 36 loci were in the HWE (*p* > 0.05; Table 2). This was likely due to null alleles presence in 12 loci (acse121, acse54, acs22, acse93, acs4, AS1, AS2, AS4, AS5, AS9, AS19, AS25) detected with MICRO-CHECKER software (*p* < 0.05). No evidence for stuttering or allelic dropout was found in any of the loci (*p* > 0.05). 

## 4. Discussion

In this study, a total of 412,381 SSRs were identified and accounted for 1.24% of the reference genome length. The genome-wide SSR content was similar to that of *Takifugu rubripes* (0.77%), *Takifugu flavidus* (0.73%) [28], and *Monopterus albus* (1.03%) [29]. Among different types of repeats, A/T motifs were considerably more common than G/C motifs (see Figure 2). Schorderet and Gartler [30] suggested this phenomenon might result from cytosine conversion to thymine. AC was the most abundant repeat category, and CG was the last, consistent with *Sander lucioperca* [31] and *Lateolabrax maculatus* [32]. AGG was the predominant trinucleotide type, which is similar to *Cyclopterus lumpus* [33], while in *L. maculatus* and many teleost fishes [34], the most common motif was ATT, and in catfish [35], ATA and TTA motifs dominate. There were also differences in the types of repeats in tetranucleotide microsatellites among the different species. In *Gadus macrocephalus* [36], CACG was the dominant repeat type, and AAAT and ATAG were the most abundant in blackhead seabream. These differences suggested that the predominant repeat motif in fishes might be species specific.

Bhattarai et al. [37] identified a final set of the 5986 polymorphic SSR loci using the HipSTR program by comparing and counting repeat number variation across the SSR loci of the whole-genome sequence data (30×) among 21 spinach plants. A total of 12,549 SSR loci were genotyped successfully from resequencing data (20×) of 20 brown-eared pheasant individuals using lobSTR software [38]. Eight hundred and seventy-one polymorphic SSRs were screened across the transcriptome of twelve individuals using the PSR tool in swamp eel [30]. Valle-Silva et al. [39] evaluated the concordance of the HipSTR, STRait Razor, and ToaSTR tools for SSR genotype calling via NGS data, and they found that the three tools present high allele calling accuracy (greater than 97%). Although several bioinformatics tools are available for SSRs analysis, in this study we chose to use HipSTR for the following reasons: (1) it was developed for calling microsatellites specifically from Illumina data; (2) it can process hundreds of samples at once; (3) it provides accurate genotype calling; and (4) it is able to manage differently diploid and haploid genotypes. In this study, we have genotyped SSRs across the genome using whole-genome sequences (10×) of 42 samples using HipSTR tools. A total of 109,131 polymorphic SSR loci were genotyped with different numbers of genotyped individuals; this approach provides a way to develop a large number of microsatellite markers. Identification of a large set of polymorphic SSR markers will support genetics and breeding research in blackhead seabream. 

Presently, polymerase chain reaction (PCR) is considered the gold standard method to investigate short tandem repeats, and the resulting amplicons can be processed by several molecular technologies. HipSTR accuracy was tested by comparing calls from 118 PCR WGS samples to capillary electrophoresis data, reporting about 98.8% consistency between the two datasets. Rocca et al. [40] evaluated the accuracy of the bioinformatics tool HipSTR in detecting and quantifying CAG repeats within the androgen receptor gene of 228 infertile men. Their findings showed that the bioinformatics tool HipSTR is 100% accurate in detecting infertile men, and HipSTR was more accurate than Sanger in genotyping normal karyotype men. Halman et al. [41] performed a comparison of four SSR genotyping tools for genotyping accuracy on 433 samples and around a million genotypes. As a result, HipSTR exhibited the lowest heterozygous error rate at low coverage. In this study, we evaluated the concordance of the HipSTR bioinformatic tool and PCR for SSR genotyping, and high concordance was observed, but low sequencing depth of samples, base mutations of SSR flanking sequences, library construction, and the PCR process may contribute to the drop in the correct call rates. Our results indicated that increases in the depth of sequencing and the length of DNA fragments can improve genotyping accuracy. Currently, some studies have reported on genotyping multiple samples using bioinformatic tools to study population structures in species. Halman et al. [42] used HipSTR to call 22 SSR marker genotypes from 2504 samples obtained from 26 human populations, and the results of the evaluation of the obtained genetic data by AMOVA, principal component analysis, and clustering analysis corroborated the reliability of this SSR dataset. Han et al. [43] genotyped SSRs using HipSTR in more than 3000 *Plasmodium falciparum* and 174 *Plasmodium vivax* to study *Plasmodium* genetic diversity, population structures, and genomic signatures of selection. HipSTR provides a mutually validated approach for traditional PCR development of microsatellites, a combination of bioinformatics and high-throughput techniques that will improve the effective use of microsatellite markers in genetic analysis.

## 5. Conclusions

In this study, a total of 412,381 SSRs were identified based on the reference genome of blackhead seabream. A large number of microsatellite markers (156,086) were mined using HipSTR by comparing sequence variants among 42 resequencing data. A set of 109,131 loci with the number of genotyped individuals ≥ 6 and the number of alleles ≥ 3 was reserved to constitute the polymorphic SSR database. Fifty-one polymorphic SSRs were identified using the PCR protocol. This is the first study to demonstrate SSR genotyping using HipSTR on target NGS data and evaluate its accuracy in blackhead seabream. Identified in our study, highly polymorphic SSR markers provide a resourceful database for genetic, genomic, and evolutionary biology studies of this species.

## Figures and Tables

**Table 1:** Distribution of the number of alleles for each SSR type in the polymorphic SSR database.

Alleles	Di-	Tri-	Tetra-	Total
3	11,626	3339	2075	17,040 (15.61)%
4, 5	19,917	4791	2524	27,232 (24.95%)
≥6	56,264	5407	3188	64,859 (59.43%)
Total	87,807 (80.16%)	13,537 (12.40%)	7787 (7.14%)	109,131 (100%)

**Table 2:** Characterization of 51 polymorphic microsatellite markers of blackhead seabream.

Loci	Primer Left Sequence	Primer Right Sequence	Ta	Product Size (bp)	Motif	Na	Ho	He	PIC	P-HWE
acs36	CCAAACCTGAGCACGACA	TAACACCTACACCCATCCCT	53	214–238	(GT)_10_	6	0.7857	0.8006	0.7550	0.5013
acs146	CATTTCTGGGCAAACAAGC	GGGTGGATAATCACACAGGG	53	252–270	(CA)_6_	5	0.7692	0.7217	0.6608	0.4491
acs168	GCATCTGGCAGCGTTTATC	TAGATACAGGGTTTTATTGTTTCC	53	233–263	(TG)_10_	5	0.4483	0.6346	0.6176	0.2949
acs12	TTCTCTTGGGCTTAGGATG	GTGACAGAGGTAAACTGAGGT	50	179–183	(GT)_7_	2	0.1333	0.1266	0.1167	0.6956
acs145	TTAGGTCAGGTCGGAGCACG	CAAAACGGGGTCATCAGAGC	54	196–220	(AC)_20_	8	0.9000	0.8452	0.8096	0.2175
acs133	AATGGTCGCTTCTCCTCC	TCACTGTCTTACTTGTATTGACG	53	214–241	(CTC)_7_	7	0.8000	0.7977	0.7513	0.4107
acs201	CCTATGTATGCCTGTCACTC	TGTGTAACTCAATATGATAGAAC	52	226–240	(GT)_9_	6	0.7586	0.6824	0.6352	0.4065
acs173	CCAGAGAGAGAAGTGAGGGGA	TGCAGGGCAGCTCTAACCT	53	248–251	(TGA)_6_	2	0.0667	0.0655	0.0624	0.8502
acs28	ACAGTGAGAGGCTTTGGTGG	ATGGGTTTGTCAGGATGAGC	52	189–195	(GT)_7_	3	0.5333	0.6305	0.5478	0.2352
acse37	ACACTAAGACAACTCAAGCAAC	CACCATTGTTTGTTTGAGC	51	228–234	(CAA)_4_	2	0.2000	0.1831	0.1638	0.5428
acse121	GCCGAAATAAACCAGATGATG	TTGTGACGGGAATGAGAGAC	52	210–238	(CA)_10_	6	0.5667	0.7610	0.7056	0.0011 *
acs199	GCGGAAGCCTTTGATTGATG	CAGTTGGAGCATCGGAGCAG	53	186–216	(GT)_15_	6	0.5600	0.7461	0.6870	0.0000 *
acs107	CTGGAATGTTACTGGAGCC	CCACAAATACACTACAATAGAAA	50	190–210	(AC)_22_	4	0.6000	0.5949	0.5319	0.6639
acse85	TTGCTGACCCATTGTGAAGT	AATAACTCCTCGCACCAGACT	52	178–205	(GCTGT)_3_	7	0.7931	0.8469	0.8100	0.9946
acse86	GTGCTGAGGGTCTTCGTTT	CTCCCACTGAGTCCAGGTAA	52	179–185	(AC)_10_	2	0.4000	0.4520	0.3457	0.5839
acse54	CAGCAGATCGGATCAGAAC	TGCGAGAACATCACAATAAAC	52	175–201	(AC)_24_	7	0.3214	0.8084	0.7694	0.0000 *
acs22	GCTGCCAGGGTGCAGTTTCG	TCTGTCCATCCCGCCCGTCT	54	182–197	(GAA)_4_	4	0.3103	0.6528	0.5822	0.0001 *
acs16	GACATTTTACAGCAGACAACC	TTCTTTATCCACAGAGCAGG	50	245–275	(CA)_18_	9	0.5357	0.6318	0.5976	0.2055
acse34	GTGGTTTACAAGGTGTGGCT	TGTTATTGTGTCACCCTGTCC	50	164–178	(GT)_9_	6	0.8333	0.7299	0.6816	0.0596
acs151	AGCAGACTGACAAGAGCACCT	AGATAGGTGGGCTGCGAG	54	234–238	(CA)_6_	2	0.0714	0.0701	0.0665	0.8446
acse93	GACAGCAACAATAATCATAA	CGTTTCACTGCCATCTG	50	286–304	(CA)_8_	6	0.2667	0.7684	0.7139	0.0000 *
acs4	CGTACATTCTGTCAAAGCCA	AAAGGAGAAGTGCCGAGC	52	202–238	(CA)_15_	10	0.5862	0.8536	0.8214	0.0042 *
acs75	TGTTATCCGACCTCAATCTC	TCTCACCATCACCTCCCA	51	227–245	(AC)_13_	8	0.8889	0.8637	0.8298	0.7215
acse101	AGGTATGTGGCTTGGTGTTG	TTGGCTCCATAGCACATTG	52	230–260	(GT)_9_	6	0.6552	0.7901	0.7412	0.1989
acse138	TGTGAGAAAGGAAGAATGAG	TGCTGGGAAACTACAATAAC	50	185–205	(GA)_12_	7	0.7333	0.7463	0.6910	0.5044
acse141	GGGCACGAGCAGTGATGTAG	CCATGTGTCTGCTATTTGCAAT	50	250–280	(TG)_9_	8	0.6667	0.7983	0.7585	0.1691
acs3	TCGTGGAGAAAGGAAGAG	CAGAAGGAGACATTTGAACT	50	183–201	(GT)_18_	7	0.8077	0.8205	0.7790	0.5272
acs78	ATCCAGTGGCAACAGAGGTC	TGACCCATCCCCAATCCC	53	215–219	(GT)_11_	2	0.2000	0.1831	0.1638	0.5428
acse165	TACGCTTCAAACGGAGACTT	GGGAACATTATTTGATTGGC	53	220–232	(GT)_7_	7	0.2610	0.2830	0.2708	0.3550
acse76	ACAACAATGCTACCAACA	AAACAAAACCTTTCCACT	50	220–292	(GAA)_15_	11	0.7500	0.8416	0.8080	0.3155
AS1	GTCCTTGTTGTAATGAGGGTTT	TCTTCATGTGTGGGGGCT	60	159–183	(AC)_17_	11	0.5313	0.8011	0.7638	0.0029 *
AS2	CGTGTTTATGTGGGTGTG	ACACGGACAAAGTCACAGT	53	146–184	(AG)_20_	15	0.7813	0.9122	0.8896	0.1611
AS3	TGGACCGTATTGAAACCTGT	CAACATCAAGGCAGCAGAGT	59	153–163	(TG)_10_	4	0.4063	0.3914	0.3516	0.9632
AS4	CAACTACCTGTTCTCTGTGTCCT	CAAAATGTGCCATGACGAC	55	272–294	(TG)_18_	12	0.5625	0.9107	0.8877	0.0000 *
AS5	TCTGTGAGAATGAGACGGAGTA	TGAGGTTTGCGAATGTGG	57	271–293	(AC)_13_	11	0.4375	0.7386	0.7081	0.0000 *
AS8	CAGTCTCCAGAGTGCGATAAC	AAGAAACAGGTGTCTCGTGAA	57	244–258	(GT)_8_	6	0.8125	0.7490	0.6960	0.5973
AS9	GCCGCAACTAACAAAGAG	ACATACAGAATACATAAACGACAG	55	198–216	(GT)_14_	8	0.3330	0.7460	0.7045	0.0000 *
AS10	ACACACTGGTCATTCGGTAA	TAGCGATAAAGAGGCACAAC	57	270–298	(GT)_14_	13	0.8438	0.9023	0.8779	0.0000 *
AS14	AGAAAGGGCGGTCTGATG	GATTGACACCATCCCAGAACT	59	214–246	(GT)_14_	14	0.5333	0.6531	0.6301	0.0000 *
AS15	TTGATTACTGTTCTCGTCTT	ATACAATACAAAACGATTACAA	53	205–215	(TG)_13_	6	0.8000	0.7062	0.6644	0.0669
AS19	AGCAGGAGATTCATGTGAGTC	ACTGCCCATGCTAACTGTTA	57	224–238	(TG)_18_	9	0.6875	0.8681	0.8374	0.0899
AS20	TTTATGTAACAAAGTGGATTTC	CAGTGCTGAGTTGGTACAAT	53	225–231	(AC)_12_	4	0.3667	0.3486	0.3206	0.1141
AS25	GTTTTCAATGTAAAGTCGGTCA	AATCGCCCAATCTGTAAGTG	57	252–280	(CA)_17_	12	0.3750	0.8309	0.7996	0.0000 *
AS26	AGCAGATGGTCGATGTTG	TTTGTTAGAGCGAAGAAGTG	53	137–143	(AC)_8_	4	0.5625	0.6424	0.5666	0.1208
AS27	CACAATGAGTTGCTGGAGGT	TTCACAGTCCCAAAGCACA	59	228–256	(CA)_12_	11	0.7333	0.8311	0.7977	0.5160
AS28	ACCGTGGATGTGTTTCGTCA	GCTTGGTAGTATTCAGGCTCTCAG	60	157–169	(CA)_11_	8	0.6563	0.7128	0.6696	0.0136 *
AS29	TTTATTGGAACACAGGGATT	GTTTCATTTTGACCATTTACC	57	195–211	(AC)_17_	8	0.8000	0.7768	0.7269	0.5920
AS31	CAGCGGCGTCTCACCTTG	GCCCGTTCAGCCTTCTCA	60	253–265	(CA)_10_	5	0.4063	0.5432	0.4757	0.2522
AS34	GCTACAGCCGCCCCACT	GGATTCAGGAAGATTGATTTGC	60	205–217	(AGAA)_6_	3	0.6667	0.6266	0.5391	0.6575
AS35	GGCTTAGGTGGAGCGTTT	GAGCGTGTTCCCAGTCATTA	57	225–253	(AGAA)_8_	8	0.7000	0.8260	0.7877	0.8708
AS36	GCAGCCTGAGCCTGGAGATA	TTAGACCGTAGAGTGTCACCGAAG	60	262–286	(GAAA)_4_	6	0.5450	0.6280	0.5707	0.0000 *

Ta: annealing temperature; Na: number of alleles; Ho: observed heterozygosity; He: expected heterozygosity; PIC: polymorphic information content; P-HWE: Hardy–Weinberg probability test; * indicates significant deviation from Hardy–Weinberg proportions after Bonferroni correction (*p* < 0.05).

## Data Availability

The data presented in this study are available upon request from the corresponding author.

## Supplementary Materials

The following supporting information can be downloaded at: https://www.mdpi.com/article/10.3390/genes14030620/s1, Supplementary Table S1. The number, length, frequency, and density of six different types of perfect SSRs; Supplementary Table S2. Frequency distribution of motif types of hexanucleotide SSR loci in the reference genome of blackhead seabream; Supplementary Table S3. Distribution of main SSR repeat number for each SSR type in blackhead seabream; Supplementary Table S4. Distribution of called microsatellite loci on the scaffold of the genome; Supplementary Table S5. Distribution of repeats of called microsatellite loci; Supplementary Table S6. Distribution of genotyped individuals of called microsatellite loci; Supplementary Table S7. The alleles number of 58 known polymorphic SSRs identified by PCR and HipSTR; Supplementary Table S8. Comparison of genotyping consistency between PCR and HipSTR; Supplementary Table S9. The polymorphic information content of 63 sites called by HipSTR; Supplementary Table S10. Detailed information of the full SSR database.
