# Supplementary material for: Microsatellite Genome-Wide Database Development for the Commercial Blackhead Seabream (Acanthopagrus schlegelii)
Source: Genes (Basel). 2023 Mar 1;14(3):620. doi: 10.3390/genes14030620 (PMC10048070; doi:10.3390/genes14030620)
Supplement: Supplementary file 1 [file Table S10. Detailed information of the full SSRs database.pdf › Table S10. Detailed information of the full SSRs database.pdf]

Table S10. Detailed information of the full SSRs database

(The indicators are on page 2,140th.)

ID	SCAF	START	END	TYPE	REPEAT	LEN	MOTIF	N	Na
3	1	21312	21338	3	9	27	ATT	13	3
5	1	30921	30954	2	17	34	AC	18	8
10	1	56994	57009	4	4	16	CAAA	27	5
17	1	76103	76116	2	7	14	TG	24	5
19	1	83229	83244	2	8	16	AC	12	8
26	1	92192	92219	2	14	28	CA	6	8
32	1	100385	100410	2	13	26	AC	12	6
33	1	100747	100758	2	6	12	TG	19	3
35	1	101894	101905	2	6	12	AT	8	3
39	1	111797	111810	2	7	14	GT	31	3
43	1	115807	115818	2	6	12	GT	11	7
45	1	120099	120110	2	6	12	AC	29	3
51	1	125463	125478	2	8	16	AC	25	5
52	1	126168	126181	2	7	14	CA	16	9
53	1	126235	126256	2	11	22	CA	23	9
59	1	130459	130470	2	6	12	CA	26	3
60	1	130600	130623	2	12	24	AC	8	11
64	1	131207	131218	2	6	12	TG	18	6
65	1	133154	133179	2	13	26	GT	15	6
67	1	135541	135562	2	11	22	GT	7	10
68	1	135664	135703	2	20	40	AC	16	8
75	1	161497	161512	2	8	16	AC	26	4
76	1	162014	162047	2	17	34	CA	9	11
77	1	162164	162193	2	15	30	CA	12	7
78	1	168665	168684	4	5	20	ATTT	18	5
82	1	172771	172790	2	10	20	TG	25	9
86	1	182032	182051	4	5	20	AAAC	31	3
87	1	182735	182766	2	16	32	AG	11	6
88	1	184552	184587	2	18	36	CA	17	12
96	1	201896	201907	2	6	12	CA	26	5
103	1	213472	213497	2	13	26	CA	11	7
104	1	217188	217223	2	18	36	TG	17	12
105	1	217576	217593	2	9	18	TG	22	9
110	1	222122	222151	2	15	30	AC	14	13
111	1	224675	224690	2	8	16	CA	21	6
114	1	234043	234075	3	11	33	TCT	26	7
115	1	236709	236738	2	15	30	CA	10	4
123	1	255249	255260	2	6	12	AT	18	3
136	1	267553	267567	3	5	15	GCG	13	4
138	1	272069	272084	2	8	16	GT	27	9
139	1	278636	278663	4	7	28	TTCT	19	7
147	1	286296	286315	4	5	20	TGAC	31	4
151	1	292530	292549	2	10	20	GT	24	5
152	1	294301	294324	2	12	24	TG	14	8
157	1	300270	300297	4	7	28	GAAT	31	4
158	1	305690	305719	2	15	30	TG	15	10
159	1	306563	306584	2	11	22	GT	16	4
160	1	308111	308126	4	4	16	AATA	15	14

161	1	309975	309989	3	5	15	CTT	6	4
167	1	320388	320399	2	6	12	AC	24	5
168	1	328547	328566	2	10	20	AC	8	9
173	1	329144	329161	2	9	18	TG	23	4
174	1	329409	329426	2	9	18	AC	19	4
176	1	333103	333126	4	6	24	AAAG	23	3
179	1	343107	343128	2	11	22	TG	20	8
180	1	343470	343485	2	8	16	AC	15	11
181	1	343681	343724	2	22	44	AC	7	9
186	1	346271	346300	2	15	30	TG	13	11
187	1	346912	346933	2	11	22	GT	10	11
191	1	351039	351058	2	10	20	TG	13	5
192	1	352195	352218	2	12	24	AC	21	8
199	1	354793	354814	2	11	22	CA	14	5
203	1	359011	359022	2	6	12	CA	25	6
209	1	360168	360193	2	13	26	CA	17	6
210	1	360340	360379	2	20	40	CA	19	10
211	1	361866	361889	4	6	24	CCAT	22	3
215	1	365072	365083	2	6	12	TG	24	3
216	1	365402	365421	2	10	20	GT	22	11
220	1	367422	367439	2	9	18	CA	17	6
221	1	369181	369192	2	6	12	AC	22	5
224	1	369909	369928	4	5	20	TGTA	23	3
238	1	380677	380730	2	27	54	CA	12	8
243	1	390523	390534	2	6	12	CA	19	9
244	1	390688	390703	2	8	16	TG	18	9
245	1	390962	390995	2	17	34	CA	12	10
250	1	394174	394209	2	18	36	TG	11	6
261	1	406526	406543	2	9	18	AC	15	12
270	1	411405	411428	2	12	24	GT	24	6
271	1	413112	413126	3	5	15	CTC	20	3
275	1	417463	417496	2	17	34	AC	6	5
277	1	417680	417699	2	10	20	AC	6	4
278	1	421668	421685	2	9	18	GT	20	6
281	1	426015	426034	2	10	20	TG	16	9
282	1	426134	426151	2	9	18	GT	18	4
284	1	426338	426377	2	20	40	TG	10	13
285	1	430019	430066	4	12	48	ATCC	20	7
300	1	450347	450360	2	7	14	CA	14	9
302	1	454479	454500	2	11	22	TG	11	7
304	1	459066	459080	3	5	15	CTC	28	3
308	1	475965	475984	4	5	20	GTGA	17	3
310	1	476041	476080	2	20	40	AG	10	12
311	1	476148	476159	2	6	12	GT	26	3
313	1	478475	478489	3	5	15	AAG	21	5
315	1	481527	481576	2	25	50	AC	11	10
316	1	481796	481811	2	8	16	TG	12	3
319	1	482416	482435	2	10	20	GT	20	10
320	1	482592	482607	2	8	16	AC	29	4
322	1	487955	487982	2	14	28	TG	19	10
325	1	491294	491309	2	8	16	TG	26	5

330	1	500649	500676	2	14	28	AC	11	6
331	1	501696	501727	2	16	32	TG	8	10
332	1	512529	512540	2	6	12	AC	31	3
338	1	523584	523603	2	10	20	AC	13	8
339	1	523798	523839	2	21	42	CA	13	15
341	1	524004	524021	2	9	18	GT	8	3
342	1	524321	524352	2	16	32	GT	8	6
345	1	524672	524693	2	11	22	TG	10	9
347	1	525306	525353	2	24	48	TG	9	11
348	1	527012	527039	2	14	28	AC	11	6
350	1	527154	527165	2	6	12	GT	12	7
351	1	527272	527305	2	17	34	TC	10	5
353	1	528759	528774	2	8	16	AG	25	3
354	1	531579	531598	4	5	20	CTTT	28	9
355	1	532175	532190	2	8	16	TG	14	7
356	1	532191	532202	2	6	12	AG	9	3
357	1	535065	535102	2	19	38	AC	10	11
358	1	536110	536141	2	16	32	GT	17	8
359	1	536295	536322	2	14	28	GT	13	10
361	1	536513	536544	2	16	32	TG	9	10
362	1	536711	536734	2	12	24	TG	18	9
363	1	538246	538260	3	5	15	ATG	21	4
364	1	538433	538452	2	10	20	TG	22	7
367	1	543392	543413	2	11	22	CA	20	9
375	1	549463	549486	2	12	24	GT	18	9
384	1	558749	558776	2	14	28	TC	17	7
390	1	568627	568648	2	11	22	GT	19	21
394	1	569748	569797	2	25	50	CA	7	10
397	1	572026	572039	2	7	14	TC	16	4
402	1	579708	579719	2	6	12	GT	32	3
403	1	583425	583436	2	6	12	AC	29	3
404	1	583567	583580	2	7	14	GT	27	3
405	1	583996	584025	2	15	30	CA	8	7
407	1	585563	585580	2	9	18	CA	19	5
408	1	585873	585922	2	25	50	GT	11	13
418	1	592227	592268	2	21	42	GT	7	8
422	1	595440	595453	2	7	14	CA	21	9
423	1	595456	595469	2	7	14	CA	23	9
424	1	595870	595885	2	8	16	AG	22	3
426	1	596041	596088	2	24	48	GT	14	14
428	1	599113	599128	2	8	16	CA	29	4
429	1	601106	601133	2	14	28	CA	14	15
431	1	601933	601958	2	13	26	GT	19	20
434	1	603723	603738	2	8	16	CA	11	5
435	1	603740	603755	2	8	16	AC	15	8
436	1	603834	603853	2	10	20	GT	11	6
439	1	607192	607231	2	20	40	CA	13	15
440	1	607488	607513	2	13	26	AC	12	8
442	1	610530	610551	2	11	22	AC	20	6
443	1	610887	610922	2	18	36	TC	16	11
444	1	611473	611502	2	15	30	AC	8	9

445	1	618305	618318	2	7	14	TC	28	4
446	1	618411	618424	2	7	14	TG	21	3
449	1	622142	622169	2	14	28	AC	16	9
457	1	631698	631717	4	5	20	TCTT	14	9
462	1	635919	635950	2	16	32	TG	16	18
463	1	636110	636149	2	20	40	GT	15	15
464	1	636411	636432	2	11	22	GT	15	13
466	1	636521	636536	2	8	16	TG	8	5
467	1	636542	636557	2	8	16	GT	11	6
468	1	640127	640153	3	9	27	CTC	22	9
471	1	642189	642232	2	22	44	CA	9	8
479	1	653952	653967	2	8	16	GT	12	4
481	1	654361	654382	2	11	22	TG	13	7
484	1	658943	658974	2	16	32	AC	17	8
485	1	661951	661972	2	11	22	AC	17	7
487	1	664254	664283	2	15	30	AC	6	9
490	1	666251	666262	2	6	12	CT	15	8
494	1	668303	668332	2	15	30	AG	16	12
495	1	668915	668940	2	13	26	GT	20	11
497	1	672023	672036	2	7	14	TG	28	5
498	1	677042	677053	2	6	12	TG	15	3
506	1	695721	695744	2	12	24	AC	24	6
510	1	700081	700096	2	8	16	CA	22	12
511	1	700097	700112	2	8	16	CT	18	10
519	1	705073	705087	3	5	15	CCT	14	3
520	1	705112	705129	3	6	18	TTA	10	3
522	1	709649	709664	2	8	16	CA	24	9
523	1	710061	710098	2	19	38	CA	14	11
525	1	711504	711541	2	19	38	AC	12	10
529	1	714212	714245	2	17	34	GT	16	12
531	1	715170	715191	2	11	22	GT	16	7
532	1	716201	716214	2	7	14	TG	24	3
534	1	719494	719523	2	15	30	TG	11	11
535	1	719642	719695	2	27	54	TG	7	7
536	1	720032	720055	2	12	24	TG	14	11
544	1	724192	724217	2	13	26	GT	17	9
546	1	724313	724328	2	8	16	CA	30	4
548	1	724687	724720	2	17	34	GT	13	10
549	1	724854	724873	2	10	20	TG	8	3
550	1	725426	725447	2	11	22	AC	21	4
551	1	725851	725866	4	4	16	CCTC	7	3
552	1	726036	726047	2	6	12	AC	12	8
553	1	726198	726209	2	6	12	TG	23	3
555	1	727247	727270	2	12	24	CA	8	8
558	1	729659	729676	2	9	18	GT	25	5
561	1	729989	730006	2	9	18	TG	13	7
562	1	730067	730082	2	8	16	TG	18	5
563	1	730217	730234	2	9	18	GT	26	4
565	1	732099	732114	2	8	16	GT	31	3
566	1	733004	733053	2	25	50	CA	9	11
568	1	734293	734324	2	16	32	TG	8	8

570	1	734454	734469	2	8	16	GT	25	3
572	1	736877	736894	2	9	18	GA	13	3
576	1	738867	738890	2	12	24	GT	15	5
577	1	739049	739066	2	9	18	TG	17	11
578	1	739222	739241	2	10	20	AC	19	12
580	1	742361	742374	2	7	14	CT	16	3
587	1	742696	742709	2	7	14	TG	8	3
593	1	744503	744552	2	25	50	AC	9	8
595	1	744782	744795	2	7	14	AC	25	3
596	1	748861	748872	2	6	12	AC	13	5
597	1	748901	748914	2	7	14	AC	8	6
598	1	749087	749102	2	8	16	CA	21	4
599	1	749160	749179	2	10	20	TG	18	5
601	1	749402	749429	2	14	28	GT	18	9
602	1	750601	750616	2	8	16	GA	17	4
604	1	754180	754195	2	8	16	TG	24	6
605	1	754197	754208	2	6	12	GT	23	5
606	1	754325	754342	2	9	18	CA	18	7
607	1	754535	754552	2	9	18	TG	12	3
609	1	754960	754979	2	10	20	TG	12	7
611	1	755314	755333	2	10	20	AC	25	6
612	1	756070	756117	2	24	48	TG	7	8
613	1	756279	756300	2	11	22	TG	8	4
616	1	756579	756610	2	16	32	AC	8	12
620	1	758759	758770	2	6	12	CA	21	4
623	1	759928	759939	2	6	12	AG	16	4
632	1	763766	763785	2	10	20	AC	11	9
635	1	764185	764222	2	19	38	TG	8	9
636	1	764402	764413	2	6	12	TG	7	5
637	1	764427	764442	2	8	16	GT	9	3
639	1	766800	766827	2	14	28	AC	24	6
641	1	767183	767194	2	6	12	CA	15	4
644	1	768457	768478	2	11	22	AC	6	8
646	1	769113	769124	2	6	12	GA	17	3
647	1	769357	769400	2	22	44	GT	6	8
648	1	771201	771214	2	7	14	AC	29	9
650	1	773198	773211	2	7	14	CA	8	5
651	1	773469	773502	2	17	34	AC	8	12
652	1	773930	773941	2	6	12	CA	27	4
655	1	775826	775845	2	10	20	TG	26	13
656	1	775846	775857	2	6	12	AG	7	5
659	1	776311	776328	2	9	18	CA	13	5
660	1	776440	776453	2	7	14	TG	17	8
662	1	776635	776656	2	11	22	TG	15	7
665	1	778100	778121	2	11	22	TG	21	7
667	1	779156	779171	4	4	16	AAAT	8	5
669	1	779773	779806	2	17	34	CA	22	4
670	1	779929	779940	2	6	12	TG	22	4
671	1	780616	780627	2	6	12	AC	19	4
672	1	780863	780908	2	23	46	AG	12	7
674	1	782009	782034	2	13	26	AC	18	4

676	1	787990	788003	2	7	14	TG	24	6
677	1	788863	788876	2	7	14	TG	23	4
678	1	789051	789070	2	10	20	CA	15	7
680	1	790671	790686	2	8	16	GT	24	3
681	1	790797	790808	2	6	12	TC	26	3
682	1	790809	790822	2	7	14	AC	27	3
683	1	790933	790948	2	8	16	TG	22	13
684	1	790950	790963	2	7	14	GT	18	9
685	1	793888	793899	2	6	12	AC	15	3
687	1	794774	794787	2	7	14	AC	20	4
689	1	795892	795911	2	10	20	GT	16	3
692	1	799039	799054	4	4	16	CCTG	30	5
693	1	799282	799299	2	9	18	AC	24	6
694	1	799403	799414	2	6	12	AC	24	4
695	1	799420	799431	2	6	12	CA	19	5
696	1	801541	801552	2	6	12	CA	11	4
697	1	801555	801566	2	6	12	CA	14	5
700	1	803200	803233	2	17	34	GT	6	7
701	1	803498	803543	2	23	46	GT	14	9
703	1	805608	805621	2	7	14	AC	28	7
704	1	807951	807976	2	13	26	GT	15	9
705	1	808063	808078	2	8	16	CA	18	4
706	1	808260	808293	2	17	34	GT	9	10
707	1	809580	809615	2	18	36	TG	11	10
710	1	811486	811497	2	6	12	GT	32	3
715	1	812281	812308	2	14	28	AC	13	13
717	1	814607	814642	2	18	36	TG	7	10
718	1	815054	815069	2	8	16	TG	17	10
722	1	816305	816316	2	6	12	AC	22	4
724	1	817443	817472	2	15	30	AC	9	12
725	1	820471	820498	2	14	28	GT	11	13
729	1	822006	822031	2	13	26	TG	13	8
732	1	822157	822174	2	9	18	CA	26	10
734	1	822677	822692	2	8	16	AC	16	3
735	1	822921	822934	2	7	14	AC	17	12
736	1	823075	823090	2	8	16	CA	18	4
737	1	823864	823889	2	13	26	AC	12	11
739	1	824000	824031	2	16	32	TG	8	9
741	1	825226	825241	2	8	16	TG	26	5
750	1	828117	828138	2	11	22	TG	13	7
752	1	828681	828692	2	6	12	TG	22	7
753	1	830624	830649	2	13	26	GT	13	11
755	1	831179	831196	2	9	18	GT	18	5
756	1	831318	831353	2	18	36	GT	6	7
757	1	832654	832693	4	10	40	TCTG	14	11
758	1	833471	833484	2	7	14	CA	14	7
759	1	833487	833500	2	7	14	CA	9	4
761	1	834944	834961	2	9	18	GT	18	8
762	1	835258	835293	4	9	36	TTCT	14	11
766	1	838215	838232	2	9	18	AC	12	9
767	1	838467	838494	2	14	28	AC	12	6

768	1	838685	838706	2	11	22	GT	10	10
773	1	847894	847917	2	12	24	GT	16	9
777	1	849721	849740	2	10	20	AC	18	4
779	1	851981	852002	2	11	22	CA	20	11
781	1	853717	853736	2	10	20	AC	21	6
783	1	853965	853982	2	9	18	CA	18	7
784	1	854057	854070	2	7	14	CA	32	4
786	1	856209	856230	2	11	22	AC	18	10
787	1	856367	856384	2	9	18	AC	16	8
789	1	857129	857148	2	10	20	GT	20	5
792	1	858973	858988	2	8	16	AC	11	9
793	1	859516	859527	2	6	12	CA	27	10
794	1	863111	863140	2	15	30	AC	12	11
795	1	863636	863683	2	24	48	CA	12	8
796	1	863805	863826	2	11	22	AC	14	4
799	1	866055	866084	2	15	30	TG	15	8
805	1	869070	869089	2	10	20	AC	20	6
807	1	873557	873574	3	6	18	CTC	23	4
810	1	878308	878337	2	15	30	AC	16	13
811	1	878557	878572	2	8	16	GT	17	11
814	1	879912	879937	2	13	26	TG	9	7
816	1	881227	881252	2	13	26	CA	17	9
818	1	884246	884295	2	25	50	CA	13	16
821	1	887961	887982	2	11	22	CA	21	8
822	1	888072	888087	2	8	16	GC	27	11
823	1	888088	888101	2	7	14	GT	10	7
824	1	890295	890328	2	17	34	TG	14	8
826	1	891301	891328	2	14	28	TG	18	11
829	1	892206	892235	2	15	30	TG	10	8
830	1	892375	892390	2	8	16	AC	22	3
832	1	900668	900685	3	6	18	ATT	9	4
833	1	900686	900700	3	5	15	AGT	11	4
834	1	901364	901389	2	13	26	GT	12	9
835	1	904815	904830	2	8	16	AC	16	4
836	1	905072	905091	2	10	20	AC	18	4
846	1	914287	914316	2	15	30	GT	13	10
848	1	915494	915509	2	8	16	CA	24	6
851	1	922260	922279	2	10	20	GA	23	4
855	1	925447	925460	2	7	14	AC	10	3
864	1	934760	934787	2	14	28	TG	18	6
867	1	944933	944956	2	12	24	AC	12	7
868	1	948754	948787	2	17	34	AC	16	7
870	1	952866	952886	3	7	21	ATT	13	6
872	1	953730	953749	2	10	20	CA	23	12
883	1	957927	957952	2	13	26	CA	11	6
885	1	967779	967794	2	8	16	TG	27	6
887	1	970968	970997	3	10	30	CTC	23	5
890	1	971400	971429	2	15	30	GA	19	10
892	1	974422	974435	2	7	14	CA	22	3
895	1	982987	983020	2	17	34	GA	8	9
896	1	983120	983145	2	13	26	AC	19	7

911	1	1001346	1001367	2	11	22	TG	15	10
912	1	1001431	1001448	2	9	18	TG	26	9
919	1	1009737	1009758	2	11	22	TG	12	6
922	1	1013832	1013863	2	16	32	AC	7	9
925	1	1021744	1021755	2	6	12	GT	31	3
926	1	1023344	1023363	4	5	20	GACA	30	3
931	1	1027600	1027631	2	16	32	CA	18	12
936	1	1041728	1041741	2	7	14	TG	29	3
944	1	1045590	1045603	2	7	14	CA	17	4
948	1	1048217	1048231	3	5	15	TTA	6	5
954	1	1061219	1061236	2	9	18	GT	15	4
956	1	1062165	1062192	2	14	28	AC	12	13
958	1	1064608	1064621	2	7	14	TG	24	9
959	1	1065172	1065201	2	15	30	AG	8	10
960	1	1065603	1065618	4	4	16	TTTA	23	3
962	1	1068489	1068506	2	9	18	AC	24	6
974	1	1072885	1072896	2	6	12	GT	13	5
975	1	1073021	1073048	2	14	28	TG	13	11
976	1	1073429	1073442	2	7	14	GA	24	3
981	1	1076317	1076344	2	14	28	GT	20	10
982	1	1076695	1076715	3	7	21	TTC	15	4
989	1	1097427	1097438	2	6	12	AC	21	5
991	1	1107657	1107674	2	9	18	AC	25	4
994	1	1113306	1113321	2	8	16	AC	14	9
998	1	1114604	1114625	2	11	22	CA	8	9
999	1	1115088	1115105	3	6	18	CTG	23	5
1000	1	1124382	1124420	3	13	39	CTC	14	7
1003	1	1125834	1125869	2	18	36	TG	23	8
1005	1	1128659	1128684	2	13	26	CA	11	5
1009	1	1138126	1138139	2	7	14	AC	17	4
1014	1	1141957	1141994	2	19	38	CA	12	8
1032	1	1163822	1163839	3	6	18	TGT	21	4
1043	1	1177804	1177815	2	6	12	GA	18	3
1046	1	1193636	1193667	2	16	32	GT	15	11
1049	1	1197541	1197566	2	13	26	AC	7	5
1050	1	1200415	1200438	4	6	24	AAAT	17	9
1076	1	1210850	1210861	2	6	12	GT	26	5
1078	1	1211314	1211334	3	7	21	AGA	20	3
1079	1	1211433	1211468	2	18	36	CT	10	9
1086	1	1234761	1234790	2	15	30	AC	19	8
1087	1	1236381	1236402	2	11	22	TG	19	11
1094	1	1254053	1254080	2	14	28	AC	8	8
1099	1	1267067	1267090	2	12	24	CA	18	9
1101	1	1273014	1273029	2	8	16	AC	15	3
1103	1	1276156	1276189	2	17	34	CA	14	13
1106	1	1276622	1276637	2	8	16	AC	28	3
1107	1	1279601	1279615	3	5	15	ATA	19	3
1114	1	1284876	1284891	2	8	16	AC	23	9
1115	1	1287235	1287264	2	15	30	CA	20	17
1118	1	1296517	1296530	2	7	14	GT	29	4
1124	1	1311668	1311681	2	7	14	CA	21	4

1129	1	1317729	1317744	4	4	16	CACG	7	5
1134	1	1326860	1326879	2	10	20	AC	14	8
1136	1	1333071	1333114	2	22	44	GT	7	8
1137	1	1333212	1333235	2	12	24	AC	15	16
1141	1	1333570	1333581	2	6	12	GT	13	7
1146	1	1335821	1335836	4	4	16	GGTC	28	6
1147	1	1339611	1339628	2	9	18	TG	15	9
1151	1	1344848	1344875	2	14	28	TC	19	11
1152	1	1344960	1344977	3	6	18	AGC	13	4
1153	1	1345509	1345524	2	8	16	GT	17	10
1154	1	1345527	1345538	2	6	12	GT	11	7
1155	1	1346037	1346060	2	12	24	AC	15	7
1162	1	1352648	1352685	2	19	38	CA	9	9
1164	1	1362957	1362990	2	17	34	TG	12	7
1165	1	1367557	1367568	2	6	12	AT	15	4
1169	1	1372886	1372905	2	10	20	CA	16	9
1172	1	1377129	1377140	2	6	12	CA	26	6
1175	1	1377531	1377552	2	11	22	AC	22	13
1176	1	1379025	1379050	2	13	26	CA	6	6
1177	1	1379153	1379192	2	20	40	AC	6	10
1178	1	1379655	1379674	2	10	20	CA	14	5
1180	1	1380560	1380593	2	17	34	CA	9	5
1186	1	1386818	1386829	2	6	12	AC	22	3
1189	1	1389138	1389165	4	7	28	CATC	21	4
1195	1	1403597	1403626	2	15	30	TG	13	8
1199	1	1406034	1406057	4	6	24	GATG	28	4
1201	1	1407797	1407816	2	10	20	CA	18	6
1202	1	1408079	1408098	2	10	20	TG	18	3
1207	1	1414643	1414672	2	15	30	GT	22	13
1208	1	1415710	1415723	2	7	14	AC	25	3
1209	1	1418870	1418883	2	7	14	AC	25	3
1210	1	1419088	1419121	2	17	34	TG	8	9
1211	1	1419322	1419335	2	7	14	GT	11	3
1215	1	1422551	1422582	2	16	32	TG	12	6
1217	1	1424003	1424044	2	21	42	AC	8	9
1221	1	1429338	1429369	2	16	32	CA	17	13
1230	1	1441139	1441168	3	10	30	GAG	14	4
1233	1	1441623	1441643	3	7	21	TGG	20	7
1234	1	1442157	1442172	4	4	16	GTGC	28	6
1235	1	1442173	1442184	2	6	12	GT	28	4
1237	1	1448756	1448769	2	7	14	AC	28	5
1247	1	1458596	1458625	2	15	30	GT	10	5
1249	1	1460770	1460781	2	6	12	AC	18	3
1253	1	1465214	1465243	2	15	30	GT	6	6
1255	1	1465540	1465565	2	13	26	GT	21	7
1256	1	1465728	1465753	2	13	26	AC	14	3
1258	1	1466854	1466867	2	7	14	GT	16	6
1262	1	1479795	1479824	2	15	30	CA	13	10
1266	1	1486393	1486406	2	7	14	TG	20	7
1268	1	1489713	1489724	2	6	12	TG	23	5
1273	1	1497727	1497740	2	7	14	TG	26	4

1274	1	1497766	1497785	2	10	20	GT	19	4
1275	1	1498705	1498720	2	8	16	AC	28	7
1286	1	1516214	1516228	3	5	15	AGC	19	4
1289	1	1518763	1518788	2	13	26	CT	15	5
1293	1	1521207	1521236	3	10	30	CCT	28	6
1294	1	1522314	1522334	3	7	21	GAG	13	4
1296	1	1526916	1526939	2	12	24	CA	20	7
1299	1	1527086	1527109	2	12	24	TG	13	7
1300	1	1527726	1527755	2	15	30	CA	10	5
1301	1	1530477	1530488	2	6	12	CA	11	6
1305	1	1535152	1535165	2	7	14	AC	24	4
1313	1	1538353	1538364	2	6	12	TG	19	3
1314	1	1538368	1538379	2	6	12	GT	10	3
1315	1	1539954	1539973	2	10	20	GT	16	12
1316	1	1540023	1540046	4	6	24	TGTC	17	3
1318	1	1543611	1543632	2	11	22	CA	11	8
1320	1	1551229	1551246	2	9	18	CA	21	5
1321	1	1552172	1552191	2	10	20	CA	20	5
1324	1	1554331	1554360	2	15	30	AC	15	11
1325	1	1554710	1554725	2	8	16	GT	26	14
1329	1	1556800	1556831	2	16	32	CT	8	7
1331	1	1557069	1557086	2	9	18	AC	11	5
1333	1	1557976	1557993	2	9	18	AT	21	10
1334	1	1558051	1558066	4	4	16	AAAT	22	9
1335	1	1558615	1558628	2	7	14	CA	22	3
1342	1	1564275	1564290	2	8	16	CA	12	4
1347	1	1580828	1580843	2	8	16	AC	30	3
1348	1	1582290	1582301	2	6	12	TG	19	8
1349	1	1583245	1583266	2	11	22	TC	18	4
1352	1	1588839	1588858	2	10	20	GT	26	9
1354	1	1589073	1589088	2	8	16	CT	8	3
1359	1	1591681	1591698	2	9	18	CA	27	10
1360	1	1592622	1592636	3	5	15	GCG	31	9
1362	1	1593960	1593979	4	5	20	AAAG	23	5
1369	1	1624071	1624084	2	7	14	GT	13	10
1372	1	1627990	1628004	3	5	15	AGC	23	3
1377	1	1633804	1633817	2	7	14	TG	27	10
1381	1	1645171	1645182	2	6	12	GT	24	4
1386	1	1660266	1660279	2	7	14	TG	20	12
1388	1	1664004	1664025	2	11	22	AC	7	4
1389	1	1666240	1666259	2	10	20	TG	7	8
1398	1	1686134	1686157	2	12	24	GT	15	3
1404	1	1712526	1712549	3	8	24	ACT	29	5
1416	1	1725490	1725507	2	9	18	TG	25	7
1419	1	1727254	1727273	2	10	20	AC	21	9
1424	1	1728876	1728893	3	6	18	CCT	22	5
1426	1	1729465	1729494	2	15	30	AC	19	9
1428	1	1730891	1730926	2	18	36	AC	7	8
1429	1	1735302	1735335	2	17	34	TC	17	6
1430	1	1736721	1736748	2	14	28	CA	19	10
1431	1	1736875	1736908	2	17	34	TG	13	14

1434	1	1739407	1739422	2	8	16	GT	22	9
1435	1	1739424	1739441	2	9	18	TG	22	9
1437	1	1740388	1740407	4	5	20	TTAT	16	3
1443	1	1751321	1751342	2	11	22	CA	18	6
1444	1	1751682	1751707	2	13	26	TG	30	6
1446	1	1753163	1753186	4	6	24	CTGT	21	4
1450	1	1771136	1771151	4	4	16	AAAT	17	6
1457	1	1786757	1786770	2	7	14	AT	15	9
1474	1	1833251	1833274	2	12	24	CA	12	7
1476	1	1835058	1835077	2	10	20	TC	20	4
1477	1	1835782	1835793	2	6	12	AC	24	4
1478	1	1836879	1836894	2	8	16	GT	17	4
1484	1	1858385	1858400	4	4	16	CAGA	32	4
1488	1	1862317	1862340	3	8	24	GGA	20	4
1491	1	1864262	1864287	2	13	26	TG	10	8
1503	1	1889135	1889146	2	6	12	AC	22	13
1504	1	1889148	1889163	2	8	16	CA	20	14
1506	1	1894244	1894267	2	12	24	CA	16	7
1507	1	1902282	1902301	2	10	20	TG	22	11
1509	1	1902798	1902824	3	9	27	GAG	20	8
1510	1	1903224	1903237	2	7	14	AT	14	9
1512	1	1910456	1910485	3	10	30	TGA	24	6
1521	1	1928017	1928031	3	5	15	CTT	33	3
1528	1	1949865	1949888	4	6	24	TGAT	25	3
1538	1	2019167	2019202	3	12	36	TGA	18	6
1539	1	2023387	2023416	2	15	30	AC	17	6
1540	1	2032763	2032783	3	7	21	TTA	8	3
1541	1	2038543	2038554	2	6	12	CA	29	3
1548	1	2053485	2053520	2	18	36	GT	15	9
1550	1	2057545	2057558	2	7	14	TG	18	3
1553	1	2066991	2067020	3	10	30	ATT	8	4
1554	1	2067343	2067366	2	12	24	CA	19	5
1564	1	2100862	2100877	2	8	16	AC	17	4
1566	1	2113973	2113990	3	6	18	ATT	20	4
1576	1	2117296	2117323	2	14	28	CA	9	5
1577	1	2118479	2118498	2	10	20	CA	31	8
1578	1	2118723	2118750	2	14	28	AC	16	4
1592	1	2128853	2128866	2	7	14	AC	23	5
1596	1	2132049	2132072	2	12	24	TG	15	11
1605	1	2140113	2140124	2	6	12	TG	26	3
1609	1	2145781	2145792	2	6	12	GT	17	6
1610	1	2146116	2146133	2	9	18	TC	13	4
1612	1	2146964	2146987	3	8	24	TCC	12	4
1613	1	2147158	2147175	2	9	18	AT	6	6
1614	1	2148433	2148448	2	8	16	CT	23	7
1615	1	2148608	2148623	2	8	16	TC	22	4
1618	1	2153427	2153448	2	11	22	AC	16	6
1625	1	2166078	2166095	2	9	18	TC	18	5
1630	1	2173815	2173832	3	6	18	ATG	22	4
1631	1	2179236	2179253	2	9	18	AC	23	8
1632	1	2197147	2197164	2	9	18	AC	24	6

1634	1	2197556	2197571	2	8	16	GT	15	3
1635	1	2199998	2200009	2	6	12	AC	30	3
1638	1	2208271	2208302	2	16	32	CA	18	9
1639	1	2209918	2209947	2	15	30	TG	19	12
1640	1	2210736	2210773	2	19	38	GT	16	14
1641	1	2210899	2210932	2	17	34	CA	13	9
1642	1	2211274	2211293	2	10	20	CA	15	6
1645	1	2221701	2221718	3	6	18	GGA	32	5
1655	1	2238372	2238393	2	11	22	AC	17	17
1658	1	2240087	2240104	2	9	18	CA	17	10
1675	1	2307710	2307729	4	5	20	TTTA	14	7
1677	1	2309933	2309950	3	6	18	GTT	30	3
1697	1	2367112	2367131	4	5	20	AACA	18	6
1699	1	2369408	2369434	3	9	27	TCC	28	4
1702	1	2372722	2372753	2	16	32	TG	9	11
1706	1	2373749	2373768	2	10	20	GT	22	6
1707	1	2374159	2374176	2	9	18	TC	11	5
1715	1	2381145	2381159	3	5	15	AAC	19	4
1716	1	2381515	2381534	4	5	20	ACCA	19	6
1723	1	2390554	2390579	2	13	26	CA	11	14
1725	1	2392365	2392404	2	20	40	CA	19	22
1728	1	2399397	2399420	2	12	24	AG	18	7
1737	1	2421235	2421262	2	14	28	CA	13	11
1748	1	2434078	2434107	2	15	30	CA	12	9
1749	1	2437299	2437324	2	13	26	AC	28	11
1750	1	2438224	2438239	4	4	16	ATTT	23	5
1752	1	2438703	2438742	2	20	40	AC	11	10
1753	1	2439000	2439019	2	10	20	TG	20	7
1760	1	2452974	2452989	2	8	16	TC	15	4
1761	1	2453260	2453297	2	19	38	AC	13	15
1765	1	2457113	2457134	2	11	22	GT	18	6
1771	1	2468811	2468832	2	11	22	TG	22	7
1774	1	2472456	2472479	2	12	24	TG	6	4
1780	1	2481555	2481568	2	7	14	CA	26	4
1781	1	2481796	2481813	2	9	18	CA	30	5
1784	1	2482942	2482953	2	6	12	TG	10	6
1785	1	2483801	2483816	2	8	16	TG	14	4
1786	1	2485156	2485173	2	9	18	TG	21	5
1805	1	2516501	2516512	2	6	12	AG	19	4
1806	1	2516573	2516592	2	10	20	CA	19	9
1816	1	2523970	2523983	2	7	14	CA	24	3
1818	1	2524253	2524282	2	15	30	CA	13	14
1820	1	2524531	2524550	4	5	20	GGAT	28	6
1826	1	2532446	2532466	3	7	21	CAG	26	11
1827	1	2534271	2534282	2	6	12	GT	27	3
1837	1	2558453	2558464	2	6	12	AC	8	5
1839	1	2570587	2570601	3	5	15	TGC	26	3
1840	1	2572831	2572850	2	10	20	CT	14	5
1842	1	2578845	2578866	2	11	22	CA	18	10
1847	1	2580620	2580649	2	15	30	AC	9	7
1851	1	2592276	2592299	2	12	24	AC	8	7

1852	1	2592384	2592411	2	14	28	CA	6	6
1853	1	2601607	2601640	2	17	34	GT	7	9
1859	1	2628414	2628429	2	8	16	TG	13	10
1863	1	2655273	2655286	2	7	14	AT	24	3
1875	1	2694467	2694486	2	10	20	AC	20	5
1876	1	2694665	2694685	3	7	21	TTC	26	4
1877	1	2696577	2696592	2	8	16	AT	18	4
1888	1	2707272	2707289	2	9	18	TG	31	5
1899	1	2731456	2731471	2	8	16	AC	22	3
1913	1	2756093	2756116	4	6	24	TTTA	16	4
1917	1	2761884	2761899	2	8	16	TG	14	4
1938	1	2806349	2806386	2	19	38	CA	9	7
1945	1	2816785	2816826	2	21	42	GT	8	5
1948	1	2822247	2822274	2	14	28	GT	17	7
1950	1	2824864	2824879	2	8	16	AG	25	8
1951	1	2825990	2826015	2	13	26	CA	12	5
1956	1	2839826	2839847	2	11	22	CA	17	6
1957	1	2840169	2840194	2	13	26	CT	11	5
1959	1	2840638	2840667	2	15	30	AG	13	11
1965	1	2844226	2844247	2	11	22	CA	23	4
1967	1	2846879	2846906	2	14	28	GT	21	14
1980	1	2868922	2868941	2	10	20	CA	16	10
1981	1	2870512	2870527	2	8	16	GT	14	8
1982	1	2870868	2870881	2	7	14	CA	24	10
1994	1	2894768	2894779	2	6	12	GT	26	4
1996	1	2895829	2895858	3	10	30	AGA	30	11
2000	1	2897991	2898002	2	6	12	TC	15	6
2002	1	2899510	2899521	2	6	12	CA	17	9
2017	1	2916740	2916754	3	5	15	CGG	24	4
2021	1	2925213	2925250	2	19	38	AC	15	7
2022	1	2925431	2925452	2	11	22	AC	12	5
2026	1	2931312	2931326	3	5	15	TTC	28	3
2029	1	2937673	2937706	2	17	34	TC	11	9
2031	1	2939675	2939696	2	11	22	AC	20	9
2034	1	2948773	2948806	2	17	34	TG	22	12
2039	1	2967704	2967723	2	10	20	TG	14	18
2041	1	2971654	2971677	2	12	24	TG	12	8
2049	1	2992202	2992217	2	8	16	TG	27	5
2050	1	2995334	2995349	2	8	16	TG	21	4
2052	1	2998364	2998389	2	13	26	TC	26	6
2059	1	3011884	3011907	2	12	24	CA	16	5
2065	1	3015743	3015758	2	8	16	GT	17	3
2070	1	3024126	3024145	2	10	20	AC	20	11
2084	1	3050834	3050848	3	5	15	GTG	27	3
2087	1	3077394	3077409	4	4	16	ATTT	21	6
2091	1	3086288	3086311	3	8	24	AAT	16	4
2096	1	3106533	3106550	3	6	18	TTA	15	7
2107	1	3139142	3139171	2	15	30	TG	6	4
2114	1	3148481	3148514	2	17	34	AC	14	12
2122	1	3156690	3156703	2	7	14	AT	14	6
2124	1	3160634	3160669	2	18	36	GT	17	15

2128	1	3167639	3167664	2	13	26	TG	16	4
2131	1	3169641	3169678	2	19	38	AC	16	11
2135	1	3170775	3170796	2	11	22	CA	22	7
2140	1	3184212	3184225	2	7	14	AC	13	11
2148	1	3200224	3200238	3	5	15	CTT	10	5
2152	1	3205286	3205303	3	6	18	TAT	19	3
2157	1	3213616	3213635	2	10	20	TG	15	6
2159	1	3219856	3219871	2	8	16	GT	9	5
2162	1	3221775	3221798	4	6	24	TGGA	24	5
2167	1	3231587	3231606	4	5	20	CATC	27	4
2168	1	3232175	3232208	2	17	34	TG	8	9
2169	1	3232501	3232524	2	12	24	GT	16	7
2172	1	3234434	3234451	2	9	18	TG	10	6
2173	1	3235133	3235178	2	23	46	CA	19	8
2180	1	3246610	3246629	4	5	20	GTTG	27	3
2181	1	3259612	3259639	2	14	28	AC	10	11
2186	1	3282012	3282059	2	24	48	TG	8	9
2189	1	3291139	3291156	2	9	18	TC	17	4
2194	1	3308046	3308061	2	8	16	TG	19	4
2197	1	3313086	3313107	2	11	22	GT	22	4
2199	1	3317184	3317201	3	6	18	CCT	19	4
2200	1	3317256	3317270	3	5	15	ATT	23	8
2203	1	3320584	3320609	2	13	26	GT	12	12
2207	1	3323224	3323245	2	11	22	GT	6	4
2216	1	3342817	3342838	2	11	22	CA	22	8
2221	1	3363652	3363669	2	9	18	AT	16	7
2224	1	3369992	3370009	2	9	18	TG	20	16
2232	1	3384668	3384681	2	7	14	GT	27	3
2233	1	3387866	3387877	2	6	12	GT	23	6
2235	1	3395430	3395453	4	6	24	TAGG	26	6
2237	1	3397933	3397946	2	7	14	AG	22	4
2245	1	3415522	3415535	2	7	14	GT	15	4
2246	1	3421928	3421975	2	24	48	TG	6	7
2249	1	3428553	3428570	2	9	18	GA	21	4
2253	1	3431333	3431356	3	8	24	CAG	22	4
2254	1	3435177	3435194	2	9	18	TG	7	5
2255	1	3437859	3437894	2	18	36	GT	13	13
2256	1	3438953	3438986	2	17	34	GA	15	10
2266	1	3442648	3442674	3	9	27	GCA	28	6
2268	1	3444555	3444580	2	13	26	AC	17	13
2269	1	3447016	3447027	2	6	12	AC	20	4
2276	1	3457120	3457137	2	9	18	TG	28	8
2277	1	3458461	3458481	3	7	21	GGA	22	4
2279	1	3460111	3460125	3	5	15	CCG	22	4
2281	1	3460425	3460458	2	17	34	TG	15	10
2288	1	3478751	3478774	2	12	24	AC	24	8
2289	1	3478936	3478951	2	8	16	AC	24	6
2290	1	3479081	3479094	2	7	14	AC	22	5
2291	1	3479096	3479107	2	6	12	CA	24	6
2293	1	3479358	3479381	2	12	24	GA	13	7
2294	1	3481262	3481279	2	9	18	TG	26	4

2295	1	3486655	3486666	2	6	12	TA	23	3
2298	1	3490368	3490387	4	5	20	TTCT	6	4
2301	1	3492263	3492280	2	9	18	GT	17	4
2302	1	3493538	3493549	2	6	12	CA	23	9
2303	1	3500343	3500366	2	12	24	AC	18	8
2307	1	3505415	3505442	2	14	28	GT	13	8
2312	1	3511664	3511697	2	17	34	TG	15	20
2315	1	3513707	3513732	2	13	26	AC	14	9
2318	1	3520962	3520976	3	5	15	GCT	19	4
2319	1	3521665	3521680	4	4	16	ATCT	26	4
2324	1	3529513	3529530	2	9	18	CA	12	12
2325	1	3530003	3530018	4	4	16	TATT	24	4
2327	1	3530922	3530936	3	5	15	CGC	24	6
2328	1	3530939	3530953	3	5	15	GCT	25	6
2329	1	3532449	3532464	2	8	16	GA	22	6
2332	1	3541580	3541600	3	7	21	GCA	19	3
2337	1	3552028	3552042	3	5	15	CAG	27	8
2338	1	3553674	3553685	2	6	12	GT	24	4
2339	1	3559719	3559732	2	7	14	TG	19	3
2353	1	3572079	3572098	2	10	20	TC	10	7
2356	1	3572245	3572262	2	9	18	CT	6	6
2363	1	3584138	3584157	2	10	20	CA	15	4
2364	1	3585810	3585823	2	7	14	TG	26	6
2371	1	3588142	3588186	3	15	45	TCC	7	6
2382	1	3594498	3594531	2	17	34	GT	12	10
2384	1	3602043	3602066	2	12	24	TG	28	9
2385	1	3603958	3603969	2	6	12	CA	17	7
2386	1	3615059	3615078	4	5	20	ATTT	7	4
2387	1	3615179	3615204	2	13	26	AC	14	11
2389	1	3615700	3615715	4	4	16	AAAT	13	11
2394	1	3620570	3620585	4	4	16	CAGA	34	3
2395	1	3621225	3621245	3	7	21	TGA	20	3
2399	1	3626297	3626310	2	7	14	CA	27	7
2400	1	3630876	3630897	2	11	22	AC	11	7
2411	1	3649536	3649557	2	11	22	TG	27	12
2413	1	3654193	3654208	2	8	16	TC	16	6
2420	1	3659580	3659597	3	6	18	CTG	25	3
2421	1	3659654	3659677	4	6	24	TTTA	9	6
2424	1	3663447	3663468	2	11	22	TG	21	10
2425	1	3663713	3663726	2	7	14	CT	24	4
2427	1	3666307	3666336	2	15	30	TG	7	10
2433	1	3668519	3668538	4	5	20	CCTC	14	5
2435	1	3670336	3670349	2	7	14	TG	21	3
2436	1	3673531	3673554	3	8	24	GGA	14	4
2437	1	3673871	3673884	2	7	14	CA	12	6
2439	1	3674185	3674200	2	8	16	TG	10	3
2440	1	3674312	3674329	2	9	18	TG	8	9
2441	1	3674522	3674563	2	21	42	AC	11	15
2447	1	3676892	3676915	4	6	24	GGAT	19	5
2452	1	3689054	3689069	2	8	16	TG	27	5
2454	1	3692034	3692047	2	7	14	CA	13	5

2462	1	3706809	3706838	2	15	30	AC	7	5
2463	1	3707157	3707180	2	12	24	TG	15	9
2466	1	3715583	3715618	2	18	36	TG	15	7
2469	1	3721438	3721467	3	10	30	TGA	27	3
2473	1	3723086	3723105	2	10	20	TC	16	4
2474	1	3723427	3723458	2	16	32	GT	12	10
2475	1	3725215	3725234	4	5	20	GTCA	32	3
2489	1	3767997	3768008	2	6	12	AG	27	4
2492	1	3775377	3775408	2	16	32	GT	10	16
2493	1	3776096	3776119	2	12	24	TG	11	7
2494	1	3779012	3779037	2	13	26	AC	8	5
2497	1	3795260	3795287	2	14	28	TG	18	14
2500	1	3802673	3802704	2	16	32	TG	13	13
2506	1	3810393	3810418	2	13	26	TG	19	18
2508	1	3813608	3813625	3	6	18	CAG	20	3
2510	1	3817724	3817759	2	18	36	TG	8	8
2512	1	3818592	3818609	2	9	18	TG	21	11
2522	1	3821199	3821214	2	8	16	AG	19	3
2526	1	3822503	3822522	2	10	20	AC	17	5
2527	1	3822887	3822900	2	7	14	AC	13	3
2528	1	3823051	3823064	2	7	14	AC	20	6
2530	1	3829602	3829617	2	8	16	TG	20	12
2537	1	3840882	3840909	4	7	28	ATTT	12	4
2545	1	3856722	3856745	4	6	24	ATAG	9	5
2553	1	3872405	3872419	3	5	15	CAC	20	4
2554	1	3873163	3873189	3	9	27	CAA	21	5
2570	1	3908059	3908072	2	7	14	GA	19	4
2571	1	3909021	3909036	2	8	16	TC	25	4
2609	1	3952892	3952913	2	11	22	GT	15	6
2610	1	3953078	3953103	2	13	26	TG	15	6
2612	1	3958238	3958249	2	6	12	GT	34	3
2615	1	3959898	3959915	2	9	18	TG	25	5
2616	1	3960791	3960812	2	11	22	GT	22	13
2617	1	3963421	3963458	2	19	38	AC	10	9
2625	1	3989208	3989223	4	4	16	TTTA	22	4
2627	1	3990448	3990461	2	7	14	CA	19	7
2628	1	3994125	3994142	2	9	18	AC	20	14
2629	1	3994499	3994524	2	13	26	CA	6	9
2630	1	4001269	4001314	2	23	46	CA	13	9
2631	1	4001617	4001666	2	25	50	TC	8	7
2633	1	4003521	4003536	2	8	16	TA	7	6
2635	1	4003776	4003795	2	10	20	AG	13	4
2640	1	4013568	4013585	2	9	18	GT	26	10
2642	1	4017610	4017625	2	8	16	CA	23	3
2644	1	4023166	4023177	2	6	12	CA	19	4
2648	1	4040594	4040615	2	11	22	AG	23	6
2651	1	4041536	4041561	2	13	26	AC	15	11
2659	1	4059465	4059500	3	12	36	AAT	17	10
2660	1	4062999	4063028	2	15	30	TG	12	12
2663	1	4063962	4063979	2	9	18	CA	7	5
2664	1	4064494	4064507	2	7	14	TG	21	5

2668	1	4065336	4065349	2	7	14	GT	23	7
2671	1	4069188	4069199	2	6	12	CA	28	11
2674	1	4071885	4071916	2	16	32	GT	9	9
2677	1	4078372	4078399	4	7	28	CAGA	23	7
2679	1	4081760	4081775	2	8	16	CA	10	9
2683	1	4086397	4086420	2	12	24	CA	21	10
2685	1	4093260	4093274	3	5	15	AGC	32	3
2686	1	4095035	4095076	2	21	42	GT	7	10
2688	1	4095214	4095253	2	20	40	GT	7	11
2693	1	4098095	4098108	2	7	14	TG	19	3
2694	1	4098277	4098300	2	12	24	TG	22	9
2697	1	4099951	4099968	3	6	18	CCA	31	4
2698	1	4100222	4100243	2	11	22	GT	25	11
2700	1	4104093	4104114	2	11	22	CA	23	16
2707	1	4118487	4118500	2	7	14	TA	18	8
2715	1	4137376	4137393	3	6	18	GCA	23	4
2716	1	4140382	4140407	2	13	26	TG	22	7
2718	1	4142895	4142914	4	5	20	AGTA	18	4
2727	1	4161522	4161537	2	8	16	GT	23	10
2728	1	4161803	4161838	2	18	36	TG	22	10
2729	1	4162351	4162374	3	8	24	CTC	18	5
2731	1	4165969	4165980	2	6	12	AC	22	6
2739	1	4195922	4195945	3	8	24	CCT	28	5
2741	1	4209708	4209731	2	12	24	TG	12	10
2744	1	4223986	4224013	2	14	28	GT	20	6
2750	1	4231717	4231756	2	20	40	AC	11	9
2756	1	4245954	4245971	2	9	18	GT	14	5
2758	1	4249467	4249481	3	5	15	GAG	23	3
2777	1	4294666	4294701	2	18	36	AG	22	4
2779	1	4304593	4304618	2	13	26	CA	11	8
2782	1	4309449	4309466	2	9	18	GT	14	8
2784	1	4314190	4314207	2	9	18	TG	15	5
2785	1	4317865	4317892	2	14	28	CT	17	12
2790	1	4335501	4335527	3	9	27	CAG	13	10
2795	1	4350649	4350664	2	8	16	TG	24	4
2796	1	4350807	4350826	2	10	20	GT	24	12
2802	1	4370843	4370857	3	5	15	CTG	23	4
2805	1	4381960	4381980	3	7	21	TTG	18	4
2808	1	4383592	4383635	2	22	44	CA	8	8
2811	1	4389606	4389629	2	12	24	AC	20	8
2814	1	4396129	4396168	2	20	40	GT	10	7
2815	1	4397847	4397866	2	10	20	CA	21	7
2819	1	4406330	4406351	2	11	22	CA	25	8
2821	1	4414206	4414245	4	10	40	TCTA	19	8
2824	1	4421631	4421646	2	8	16	CA	27	10
2825	1	4421845	4421858	2	7	14	TC	13	10
2830	1	4428947	4428964	2	9	18	CA	17	11
2834	1	4432350	4432375	2	13	26	CA	26	11
2836	1	4434275	4434290	2	8	16	GA	8	4
2839	1	4437289	4437322	2	17	34	GT	15	15
2840	1	4440960	4440993	2	17	34	CA	12	8

2841	1	4441312	4441343	2	16	32	TG	7	9
2842	1	4441524	4441537	2	7	14	CA	15	5
2843	1	4443262	4443282	3	7	21	CCT	17	13
2846	1	4443463	4443478	2	8	16	AG	14	13
2848	1	4444532	4444555	4	6	24	ATGG	21	7
2849	1	4445885	4445923	3	13	39	TGT	17	10
2850	1	4446186	4446217	2	16	32	CA	15	7
2854	1	4454437	4454466	2	15	30	TG	10	8
2860	1	4470490	4470522	3	11	33	ATT	11	3
2864	1	4476215	4476234	2	10	20	AT	17	4
2868	1	4494370	4494381	2	6	12	CT	29	4
2869	1	4499898	4499945	2	24	48	GT	9	7
2876	1	4509578	4509599	2	11	22	CA	16	14
2878	1	4512890	4512907	2	9	18	CT	20	4
2884	1	4518394	4518415	2	11	22	CA	16	8
2889	1	4528685	4528698	2	7	14	GT	15	4
2890	1	4530039	4530066	2	14	28	CA	14	9
2898	1	4544077	4544120	2	22	44	CA	21	10
2899	1	4544313	4544330	2	9	18	CA	6	4
2902	1	4545383	4545396	2	7	14	GT	18	7
2903	1	4545507	4545520	2	7	14	GA	24	3
2904	1	4545825	4545844	4	5	20	AAAT	28	10
2905	1	4547014	4547033	2	10	20	GT	21	8
2906	1	4547691	4547714	2	12	24	TG	12	8
2910	1	4549613	4549636	3	8	24	CAT	26	6
2911	1	4554297	4554326	2	15	30	TG	18	7
2912	1	4556343	4556358	2	8	16	TG	27	3
2915	1	4559356	4559391	2	18	36	CA	12	11
2917	1	4559595	4559606	2	6	12	GT	11	6
2918	1	4559608	4559627	4	5	20	CGTG	6	6
2920	1	4561481	4561502	2	11	22	CA	13	10
2922	1	4563726	4563755	2	15	30	GT	9	4
2923	1	4563880	4563909	2	15	30	CA	20	11
2925	1	4564329	4564346	2	9	18	AC	15	11
2930	1	4567946	4567969	2	12	24	AC	16	12
2932	1	4568501	4568528	2	14	28	TG	16	13
2933	1	4568941	4568954	2	7	14	AC	26	3
2935	1	4569503	4569516	2	7	14	GT	20	5
2938	1	4577521	4577556	2	18	36	TG	8	11
2939	1	4578757	4578780	2	12	24	AC	8	9
2943	1	4584337	4584380	2	22	44	GT	10	8
2945	1	4587171	4587192	2	11	22	CA	15	10
2946	1	4589773	4589806	2	17	34	AC	8	9
2949	1	4593630	4593647	2	9	18	TG	15	12
2952	1	4595542	4595553	2	6	12	AG	28	4
2955	1	4596690	4596705	2	8	16	AC	21	13
2956	1	4596706	4596719	2	7	14	AG	19	9
2957	1	4596829	4596850	2	11	22	AC	18	12
2962	1	4606675	4606706	2	16	32	TG	10	10
2965	1	4610488	4610499	2	6	12	TG	25	5
2968	1	4612759	4612784	2	13	26	AC	7	7

2971	1	4616772	4616795	2	12	24	TG	10	6
2973	1	4617110	4617133	2	12	24	AC	14	7
2975	1	4620353	4620367	3	5	15	AAG	21	3
2976	1	4620841	4620864	2	12	24	AC	14	8
2978	1	4621086	4621109	2	12	24	CA	21	12
2979	1	4624059	4624086	2	14	28	CA	9	11
2980	1	4625083	4625120	2	19	38	AC	10	12
2981	1	4628080	4628117	2	19	38	CA	9	9
2989	1	4636117	4636131	3	5	15	GCT	30	4
2991	1	4643668	4643717	2	25	50	AC	7	10
2994	1	4643862	4643879	2	9	18	TC	22	4
2995	1	4644341	4644355	3	5	15	TTG	26	4
3002	1	4652356	4652377	2	11	22	CA	13	9
3003	1	4652857	4652872	2	8	16	CA	20	4
3004	1	4653692	4653711	2	10	20	CA	20	8
3005	1	4656913	4656944	2	16	32	TC	6	8
3009	1	4657579	4657598	2	10	20	GT	19	4
3025	1	4671238	4671255	3	6	18	AAC	27	3
3027	1	4672174	4672189	2	8	16	CT	25	4
3028	1	4672295	4672326	2	16	32	GT	14	11
3029	1	4672430	4672457	2	14	28	AC	10	9
3030	1	4672626	4672681	2	28	56	GT	7	12
3031	1	4672813	4672826	2	7	14	CA	25	7
3034	1	4676583	4676606	2	12	24	CA	18	11
3035	1	4676977	4676994	2	9	18	CA	21	8
3040	1	4679033	4679054	2	11	22	GT	7	9
3044	1	4683516	4683543	2	14	28	GA	11	5
3045	1	4683645	4683686	2	21	42	AC	13	11
3052	1	4686472	4686503	2	16	32	AC	15	11
3055	1	4688119	4688136	2	9	18	GT	21	9
3057	1	4693434	4693445	2	6	12	CA	21	3
3058	1	4693447	4693458	2	6	12	AC	19	3
3061	1	4699263	4699276	2	7	14	CA	21	4
3062	1	4702273	4702288	2	8	16	TG	20	6
3063	1	4703152	4703167	2	8	16	CA	7	4
3065	1	4703555	4703602	2	24	48	TG	9	14
3067	1	4704273	4704290	2	9	18	CA	18	9
3068	1	4704293	4704306	2	7	14	CA	19	7
3069	1	4704697	4704708	2	6	12	GA	22	3
3070	1	4704810	4704833	2	12	24	AC	14	12
3071	1	4705042	4705065	2	12	24	TG	19	10
3077	1	4707264	4707291	2	14	28	AG	19	14
3078	1	4707346	4707371	2	13	26	CA	12	10
3079	1	4707553	4707564	2	6	12	CA	7	8
3080	1	4707567	4707584	2	9	18	CA	14	9
3081	1	4707715	4707732	2	9	18	TG	23	7
3082	1	4708068	4708111	2	22	44	TG	7	9
3084	1	4708524	4708539	2	8	16	CT	27	5
3085	1	4709378	4709395	2	9	18	CA	28	8
3087	1	4711432	4711459	4	7	28	GTCT	24	4
3088	1	4714199	4714216	2	9	18	AC	21	7

3093	1	4717244	4717261	3	6	18	GTT	25	3
3095	1	4718243	4718262	2	10	20	CA	28	7
3099	1	4727429	4727452	2	12	24	TG	6	8
3101	1	4728676	4728687	2	6	12	GT	22	5
3103	1	4729641	4729668	2	14	28	TG	13	7
3104	1	4731639	4731658	2	10	20	AC	21	6
3107	1	4734939	4734956	2	9	18	AC	6	3
3108	1	4735124	4735141	2	9	18	AC	8	3
3109	1	4738633	4738658	2	13	26	TG	14	6
3111	1	4742883	4742902	2	10	20	GT	24	7
3113	1	4743044	4743077	2	17	34	CA	17	5
3120	1	4758910	4758933	2	12	24	AC	21	7
3122	1	4760549	4760580	2	16	32	CA	7	6
3124	1	4763877	4763909	3	11	33	AAC	22	7
3126	1	4776094	4776135	2	21	42	TG	11	9
3131	1	4787346	4787363	3	6	18	AAC	20	3
3137	1	4796181	4796204	4	6	24	TCTT	24	3
3138	1	4797039	4797060	2	11	22	AC	15	8
3140	1	4801478	4801493	2	8	16	GT	17	6
3143	1	4815850	4815877	2	14	28	CA	13	11
3146	1	4836602	4836627	2	13	26	AC	23	6
3157	1	4864308	4864327	4	5	20	TGTT	27	9
3165	1	4875652	4875667	4	4	16	AAAC	26	3
3167	1	4884409	4884426	3	6	18	CTT	9	10
3168	1	4885215	4885238	2	12	24	AC	18	15
3172	1	4888339	4888350	2	6	12	TG	29	3
3173	1	4891608	4891639	4	8	32	TATC	18	6
3174	1	4894947	4894974	2	14	28	GT	7	7
3176	1	4896679	4896700	2	11	22	GT	25	10
3179	1	4902303	4902317	3	5	15	GCT	13	3
3180	1	4905898	4905912	3	5	15	TTG	28	3
3181	1	4910874	4910885	2	6	12	AT	21	3
3194	1	4948248	4948273	2	13	26	GT	12	8
3214	1	4988270	4988281	2	6	12	AC	24	3
3216	1	4992628	4992645	3	6	18	GGA	21	4
3220	1	4995716	4995729	2	7	14	CA	21	7
3221	1	4996771	4996800	2	15	30	AC	8	9
3231	1	5017197	5017222	2	13	26	TG	23	12
3237	1	5025931	5025946	2	8	16	GT	20	4
3238	1	5034365	5034376	2	6	12	GT	18	4
3241	1	5034901	5034926	2	13	26	CA	9	5
3242	1	5035080	5035095	2	8	16	GT	22	5
3243	1	5035559	5035578	2	10	20	CT	7	7
3246	1	5036124	5036153	2	15	30	TG	17	9
3247	1	5036535	5036550	2	8	16	CA	15	5
3257	1	5040836	5040853	2	9	18	GA	19	6
3258	1	5040977	5040994	2	9	18	TG	20	10
3261	1	5070064	5070075	2	6	12	GT	29	3
3268	1	5097595	5097609	3	5	15	TCA	29	3
3274	1	5125253	5125270	2	9	18	CA	22	7
3276	1	5136309	5136328	2	10	20	CA	21	11

3277	1	5136397	5136412	4	4	16	GTGC	22	27
3282	1	5141433	5141448	4	4	16	GGAT	32	3
3292	1	5165838	5165853	2	8	16	TG	16	10
3295	1	5171360	5171373	2	7	14	TG	24	5
3301	1	5185548	5185561	2	7	14	CA	25	3
3303	1	5193406	5193433	2	14	28	TG	19	16
3304	1	5200165	5200210	2	23	46	CA	6	5
3306	1	5200487	5200498	2	6	12	CA	27	3
3308	1	5205338	5205357	2	10	20	AC	13	10
3314	1	5219317	5219348	4	8	32	GATA	12	9
3315	1	5221214	5221241	2	14	28	GT	20	8
3321	1	5231792	5231815	3	8	24	AAG	23	16
3322	1	5235861	5235884	2	12	24	AC	20	5
3323	1	5236916	5236930	3	5	15	GAT	35	3
3328	1	5288122	5288147	2	13	26	AC	15	14
3330	1	5290816	5290851	2	18	36	AC	7	8
3333	1	5295470	5295490	3	7	21	TAT	18	13
3335	1	5297067	5297096	2	15	30	AC	17	13
3338	1	5306226	5306245	4	5	20	ATCC	27	3
3339	1	5308173	5308184	2	6	12	AC	24	5
3342	1	5321306	5321325	2	10	20	TA	6	5
3350	1	5335880	5335895	4	4	16	TTTA	15	8
3353	1	5344246	5344261	2	8	16	AC	23	6
3356	1	5354406	5354421	2	8	16	GT	24	3
3362	1	5360899	5360916	2	9	18	TC	21	6
3364	1	5361449	5361462	2	7	14	TC	27	5
3368	1	5366042	5366057	4	4	16	CTTT	24	3
3369	1	5367185	5367214	2	15	30	GT	11	8
3370	1	5368116	5368127	2	6	12	AC	25	13
3373	1	5381450	5381489	2	20	40	AC	10	15
3374	1	5382067	5382094	2	14	28	GT	12	18
3379	1	5390815	5390832	2	9	18	AC	20	8
3383	1	5401540	5401557	2	9	18	AG	29	6
3385	1	5403563	5403577	3	5	15	AAC	26	3
3386	1	5405741	5405764	2	12	24	TG	21	13
3391	1	5413925	5413948	2	12	24	TG	12	9
3394	1	5424258	5424275	2	9	18	TG	8	9
3395	1	5424277	5424294	2	9	18	GT	13	13
3396	1	5426223	5426238	2	8	16	TG	18	4
3399	1	5429323	5429344	2	11	22	GT	22	7
3404	1	5438191	5438217	3	9	27	CCT	24	7
3407	1	5450551	5450566	2	8	16	TC	27	4
3411	1	5461030	5461049	4	5	20	CTAT	23	4
3412	1	5467158	5467189	4	8	32	ATGT	21	11
3416	1	5471215	5471244	2	15	30	TG	6	6
3424	1	5486553	5486570	2	9	18	AC	16	6
3425	1	5486748	5486761	2	7	14	TG	29	3
3427	1	5486923	5486940	2	9	18	AC	24	10
3428	1	5487658	5487679	2	11	22	TG	14	9
3432	1	5490996	5491023	2	14	28	CA	21	7
3434	1	5493406	5493425	2	10	20	TG	11	3

3441	1	5497347	5497378	4	8	32	ATCC	22	4
3442	1	5499421	5499434	2	7	14	CA	20	4
3443	1	5499473	5499486	2	7	14	AC	15	8
3448	1	5504888	5504903	2	8	16	CT	26	5
3449	1	5507353	5507376	2	12	24	GT	11	9
3452	1	5515490	5515515	2	13	26	TG	21	10
3454	1	5517188	5517209	2	11	22	TG	19	9
3455	1	5517395	5517406	2	6	12	AC	24	5
3456	1	5517674	5517707	2	17	34	TG	8	10
3458	1	5517909	5517935	3	9	27	GAG	20	6
3463	1	5529861	5529886	2	13	26	AC	19	5
3464	1	5530121	5530148	2	14	28	CA	15	10
3472	1	5540320	5540331	2	6	12	AC	18	5
3473	1	5543905	5543920	2	8	16	TA	7	3
3474	1	5551251	5551268	2	9	18	GT	22	5
3479	1	5563915	5563928	2	7	14	AC	27	4
3481	1	5565123	5565166	2	22	44	TG	11	13
3482	1	5567573	5567590	2	9	18	CA	19	6
3483	1	5569887	5569908	2	11	22	TG	10	10
3485	1	5573895	5573920	2	13	26	AC	8	8
3486	1	5574265	5574280	2	8	16	TG	7	4
3489	1	5590205	5590216	2	6	12	TG	28	6
3490	1	5591747	5591766	4	5	20	AAAG	25	3
3491	1	5593355	5593368	2	7	14	AC	27	3
3492	1	5595589	5595608	2	10	20	GA	19	5
3493	1	5598585	5598608	2	12	24	CA	12	8
3494	1	5598734	5598751	2	9	18	AC	21	8
3495	1	5602304	5602327	2	12	24	TG	21	9
3496	1	5602419	5602448	2	15	30	CA	11	15
3500	1	5606044	5606069	2	13	26	TG	11	14
3508	1	5616805	5616830	2	13	26	CA	23	4
3510	1	5621043	5621056	2	7	14	CA	27	16
3511	1	5621282	5621297	2	8	16	CA	20	10
3512	1	5621300	5621317	2	9	18	CA	20	8
3519	1	5645856	5645899	2	22	44	AC	7	10
3527	1	5659359	5659384	2	13	26	GT	26	5
3533	1	5663294	5663305	2	6	12	TC	21	3
3534	1	5664885	5664902	2	9	18	TC	27	6
3538	1	5673590	5673610	3	7	21	TGT	22	4
3539	1	5679946	5679963	2	9	18	AT	7	3
3549	1	5706839	5706862	3	8	24	CTG	24	5
3557	1	5736761	5736814	2	27	54	GA	6	8
3561	1	5753817	5753831	3	5	15	CTG	16	4
3563	1	5754136	5754159	3	8	24	GGA	20	5
3566	1	5771846	5771871	2	13	26	AC	21	10
3569	1	5780212	5780223	2	6	12	CA	11	6
3570	1	5781032	5781047	2	8	16	TG	15	6
3571	1	5781160	5781173	2	7	14	CA	11	4
3573	1	5781278	5781313	2	18	36	CA	8	8
3575	1	5785180	5785203	4	6	24	TTGT	18	3
3584	1	5802930	5802959	2	15	30	AC	9	10

3585	1	5805244	5805271	2	14	28	GT	19	12
3586	1	5816545	5816556	2	6	12	TG	20	3
3591	1	5819510	5819523	2	7	14	CA	17	7
3599	1	5832177	5832204	2	14	28	GT	6	9
3601	1	5832885	5832912	2	14	28	TG	21	11
3603	1	5833334	5833357	3	8	24	AGG	20	4
3604	1	5839727	5839766	2	20	40	CA	20	9
3605	1	5840918	5840932	3	5	15	AGC	27	5
3609	1	5848925	5848954	2	15	30	GT	19	8
3610	1	5853275	5853296	2	11	22	GT	13	4
3611	1	5862036	5862053	2	9	18	CT	23	4
3612	1	5862261	5862272	2	6	12	TG	13	8
3613	1	5862275	5862288	2	7	14	TG	18	9
3614	1	5873191	5873202	2	6	12	CT	28	3
3616	1	5875632	5875653	2	11	22	AC	21	4
3618	1	5876295	5876318	2	12	24	CA	12	7
3619	1	5876614	5876625	2	6	12	CA	24	3
3624	1	5879017	5879036	2	10	20	AC	13	5
3626	1	5882819	5882830	2	6	12	GC	22	4
3627	1	5883024	5883041	2	9	18	AC	24	5
3630	1	5883680	5883694	3	5	15	TGC	26	5
3631	1	5883830	5883856	3	9	27	TGC	22	5
3632	1	5884155	5884178	3	8	24	GCG	23	8
3635	1	5887927	5887938	2	6	12	GT	21	8
3641	1	5890772	5890789	2	9	18	GT	23	3
3644	1	5892093	5892122	3	10	30	AGG	6	5
3645	1	5892656	5892669	2	7	14	AC	18	7
3646	1	5893061	5893072	2	6	12	AG	27	9
3649	1	5899393	5899410	2	9	18	GT	19	4
3650	1	5902043	5902058	4	4	16	GTTG	32	3
3652	1	5904250	5904265	4	4	16	GAGG	7	4
3660	1	5910487	5910518	2	16	32	TG	14	6
3664	1	5930839	5930864	2	13	26	GT	14	12
3666	1	5932608	5932621	2	7	14	CT	33	3
3667	1	5932739	5932758	4	5	20	CTGT	27	3
3673	1	5949142	5949168	3	9	27	TTC	24	4
3675	1	5965721	5965738	3	6	18	TGT	21	4
3681	1	5978110	5978145	2	18	36	AC	8	9
3685	1	5984080	5984094	3	5	15	TGT	17	5
3686	1	5984457	5984468	2	6	12	TC	25	3
3693	1	5999923	5999943	3	7	21	AGC	19	5
3700	1	6008248	6008263	4	4	16	TTGT	29	3
3713	1	6037257	6037280	3	8	24	AGG	21	10
3714	1	6037453	6037486	2	17	34	AC	13	8
3718	1	6056796	6056825	3	10	30	TTA	6	4
3723	1	6067980	6068003	3	8	24	ATT	21	5
3732	1	6099015	6099032	2	9	18	TG	21	6
3752	1	6130978	6130998	3	7	21	GCA	27	5
3753	1	6138018	6138043	2	13	26	GT	18	11
3754	1	6138261	6138292	2	16	32	GT	8	9
3756	1	6138441	6138460	2	10	20	CA	17	5

3758	1	6138768	6138793	2	13	26	AG	10	3
3761	1	6144127	6144152	2	13	26	AC	25	11
3762	1	6146087	6146106	2	10	20	AC	16	5
3767	1	6150043	6150058	2	8	16	GT	27	8
3773	1	6164509	6164532	2	12	24	AC	7	3
3780	1	6183281	6183296	2	8	16	AG	20	5
3785	1	6202539	6202558	2	10	20	GA	24	7
3788	1	6213204	6213227	2	12	24	GA	18	5
3793	1	6215030	6215059	2	15	30	GT	19	8
3795	1	6222273	6222290	2	9	18	AG	22	3
3799	1	6230836	6230859	3	8	24	AAG	27	4
3805	1	6236464	6236481	2	9	18	AC	14	10
3806	1	6238771	6238790	4	5	20	TTTC	31	4
3815	1	6267191	6267204	2	7	14	TG	15	5
3816	1	6267206	6267217	2	6	12	GT	15	4
3817	1	6268959	6268976	2	9	18	CA	15	6
3821	1	6276915	6276936	2	11	22	TG	17	10
3824	1	6277270	6277309	2	20	40	TG	19	10
3826	1	6277568	6277589	2	11	22	GT	6	6
3827	1	6280326	6280337	2	6	12	GT	27	11
3829	1	6282688	6282709	2	11	22	TG	23	6
3832	1	6284742	6284757	2	8	16	TC	33	3
3833	1	6288070	6288091	2	11	22	TG	18	5
3835	1	6290771	6290798	2	14	28	CA	18	13
3837	1	6294756	6294795	2	20	40	GT	11	10
3838	1	6295496	6295507	2	6	12	TG	29	5
3843	1	6306730	6306745	4	4	16	AAAT	15	4
3851	1	6315355	6315386	2	16	32	CA	8	5
3858	1	6327587	6327598	2	6	12	GT	29	4
3863	1	6334978	6334997	2	10	20	TC	26	5
3864	1	6339646	6339683	2	19	38	TG	16	10
3869	1	6342425	6342448	2	12	24	GT	17	4
3871	1	6346404	6346423	2	10	20	CA	8	3
3875	1	6351283	6351296	2	7	14	GT	20	6
3878	1	6360583	6360604	2	11	22	AG	20	4
3879	1	6365355	6365382	2	14	28	AC	15	12
3883	1	6373979	6374012	2	17	34	AC	12	8
3884	1	6376163	6376176	2	7	14	GT	18	6
3888	1	6382663	6382680	3	6	18	CAG	29	3
3893	1	6395616	6395637	2	11	22	CT	26	9
3894	1	6404132	6404153	2	11	22	CT	15	7
3895	1	6407710	6407724	3	5	15	TGC	23	3
3898	1	6412784	6412799	2	8	16	AC	11	5
3899	1	6412858	6412891	2	17	34	TG	8	8
3911	1	6448177	6448194	2	9	18	AC	18	5
3912	1	6462786	6462807	2	11	22	GT	6	6
3917	1	6469486	6469503	2	9	18	CA	20	4
3920	1	6482931	6482950	2	10	20	TG	30	19
3922	1	6493402	6493433	2	16	32	AC	8	12
3923	1	6494725	6494736	2	6	12	TC	23	6
3924	1	6494740	6494751	2	6	12	CA	23	7

3926	1	6496989	6497008	2	10	20	AC	17	6
3931	1	6499368	6499381	2	7	14	TC	10	5
3932	1	6499382	6499393	2	6	12	AC	9	5
3934	1	6499500	6499519	2	10	20	CA	10	7
3936	1	6501088	6501107	4	5	20	TTAT	13	3
3937	1	6501796	6501813	3	6	18	GGT	8	5
3938	1	6502580	6502613	2	17	34	GT	12	10
3939	1	6502786	6502807	2	11	22	GT	7	7
3941	1	6504716	6504735	4	5	20	AAAT	15	4
3943	1	6506836	6506853	2	9	18	GT	13	9
3944	1	6511842	6511853	2	6	12	CA	30	7
3945	1	6512085	6512108	2	12	24	GT	25	7
3955	1	6531496	6531521	2	13	26	TG	19	7
3962	1	6536961	6536972	2	6	12	GT	15	9
3963	1	6536975	6536992	2	9	18	GT	16	8
3967	1	6546654	6546675	2	11	22	GT	11	12
3968	1	6546824	6546845	2	11	22	GT	21	8
3969	1	6548211	6548226	2	8	16	TG	21	11
3971	1	6548418	6548439	2	11	22	TG	21	12
3973	1	6548666	6548687	2	11	22	TG	23	9
3974	1	6548886	6548901	2	8	16	TG	19	3
3978	1	6551336	6551367	2	16	32	GT	14	10
3979	1	6551766	6551817	2	26	52	CA	7	10
3987	1	6564241	6564255	3	5	15	TAC	24	4
3990	1	6565155	6565193	3	13	39	CAT	17	20
3992	1	6567974	6567989	4	4	16	AAAG	15	6
3995	1	6573051	6573066	2	8	16	TG	25	9
3996	1	6574580	6574591	2	6	12	CA	25	3
4000	1	6580463	6580474	2	6	12	AC	19	5
4001	1	6580477	6580488	2	6	12	AC	24	4
4004	1	6588086	6588105	2	10	20	CA	8	5
4005	1	6588201	6588218	2	9	18	CA	14	9
4006	1	6588361	6588387	3	9	27	CCT	13	5
4007	1	6588520	6588539	4	5	20	CTCC	6	3
4008	1	6588681	6588692	2	6	12	TC	18	5
4009	1	6588695	6588710	2	8	16	TC	15	4
4011	1	6591889	6591903	3	5	15	TCC	25	4
4014	1	6598138	6598149	2	6	12	AC	23	3
4018	1	6599368	6599379	2	6	12	TC	22	4
4032	1	6629757	6629771	3	5	15	CAA	36	3
4040	1	6637255	6637266	2	6	12	TG	15	3
4044	1	6638864	6638881	2	9	18	GT	19	5
4053	1	6658302	6658351	2	25	50	TG	18	14
4061	1	6665533	6665554	2	11	22	GT	20	4
4062	1	6665738	6665767	2	15	30	GT	10	5
4063	1	6665893	6665916	2	12	24	TG	11	7
4080	1	6669386	6669401	2	8	16	TC	22	4
4084	1	6677020	6677041	2	11	22	AC	17	6
4085	1	6677164	6677177	2	7	14	AC	20	9
4089	1	6687184	6687197	2	7	14	CT	13	4
4090	1	6687199	6687216	2	9	18	TC	15	6

4096	1	6692063	6692086	2	12	24	AC	13	11
4100	1	6694943	6694976	2	17	34	AG	15	13
4101	1	6695399	6695414	2	8	16	GT	16	3
4103	1	6696714	6696725	2	6	12	AG	10	9
4104	1	6696883	6696918	2	18	36	TG	12	11
4107	1	6699174	6699199	2	13	26	CT	9	9
4111	1	6700499	6700512	2	7	14	TG	13	8
4112	1	6700698	6700713	2	8	16	GT	19	6
4113	1	6701132	6701173	2	21	42	CA	12	13
4114	1	6701302	6701317	2	8	16	AC	14	3
4119	1	6721317	6721350	2	17	34	CA	9	8
4120	1	6721866	6721893	2	14	28	CA	19	9
4122	1	6722014	6722037	2	12	24	GT	20	10
4123	1	6722370	6722393	2	12	24	GT	14	6
4124	1	6722519	6722542	3	8	24	AAC	16	9
4133	1	6732520	6732531	2	6	12	TG	29	3
4134	1	6732582	6732621	2	20	40	GT	6	4
4135	1	6732731	6732746	2	8	16	GT	19	3
4137	1	6732936	6732951	2	8	16	TG	24	5
4138	1	6732999	6733024	2	13	26	TG	19	10
4139	1	6733447	6733470	2	12	24	AG	16	5
4141	1	6736972	6737001	2	15	30	GT	8	11
4142	1	6737089	6737116	2	14	28	AG	17	9
4145	1	6745792	6745809	3	6	18	AGG	14	6
4148	1	6746241	6746267	3	9	27	TCT	26	5
4150	1	6748622	6748643	2	11	22	GT	20	9
4152	1	6748783	6748802	2	10	20	AC	13	8
4153	1	6750802	6750825	2	12	24	CA	13	7
4157	1	6751915	6751960	2	23	46	TG	12	12
4161	1	6756526	6756540	3	5	15	TCC	13	4
4162	1	6756892	6756909	3	6	18	TCC	9	5
4164	1	6757710	6757723	2	7	14	CA	18	3
4166	1	6758107	6758138	2	16	32	GT	13	8
4168	1	6762480	6762497	2	9	18	AC	19	6
4169	1	6762759	6762774	4	4	16	AAAT	24	4
4172	1	6765370	6765385	4	4	16	TGAC	31	4
4176	1	6769707	6769734	2	14	28	GA	7	5
4178	1	6770258	6770271	2	7	14	GT	19	8
4180	1	6770642	6770655	2	7	14	CA	22	3
4183	1	6770905	6770922	2	9	18	GT	12	9
4184	1	6771225	6771246	2	11	22	CA	21	7
4185	1	6771361	6771386	2	13	26	TG	12	8
4186	1	6772897	6772912	2	8	16	TG	20	6
4187	1	6773128	6773153	2	13	26	CA	11	8
4191	1	6776415	6776466	2	26	52	TG	7	9
4192	1	6776594	6776608	3	5	15	GAG	13	9
4197	1	6778823	6778850	2	14	28	TG	12	4
4203	1	6781944	6781955	2	6	12	GA	7	5
4204	1	6782108	6782119	2	6	12	GA	12	6
4207	1	6785822	6785833	2	6	12	GA	12	5
4210	1	6786196	6786223	2	14	28	GT	19	9

4213	1	6792390	6792409	2	10	20	GT	15	6
4214	1	6792640	6792671	4	8	32	GTCT	14	6
4225	1	6806824	6806839	2	8	16	GT	9	8
4226	1	6806841	6806856	2	8	16	TG	9	7
4246	1	6819293	6819310	2	9	18	CA	15	6
4247	1	6820757	6820772	2	8	16	CA	20	6
4248	1	6820912	6820937	2	13	26	CA	11	8
4250	1	6821403	6821420	2	9	18	TG	15	6
4252	1	6823003	6823016	2	7	14	TG	24	3
4253	1	6823646	6823669	2	12	24	TG	11	9
4258	1	6825837	6825872	2	18	36	AC	17	10
4260	1	6828152	6828171	2	10	20	AG	15	5
4266	1	6829410	6829429	2	10	20	CA	13	6
4269	1	6831364	6831389	2	13	26	GT	20	9
4270	1	6831497	6831516	2	10	20	GT	16	11
4271	1	6831894	6831933	2	20	40	CA	20	10
4273	1	6835892	6835912	3	7	21	CCT	17	3
4277	1	6840537	6840556	2	10	20	CA	13	12
4279	1	6845762	6845795	2	17	34	GT	19	8
4280	1	6849883	6849898	2	8	16	TG	26	5
4281	1	6850317	6850328	2	6	12	GT	22	5
4282	1	6850544	6850557	2	7	14	GT	23	8
4284	1	6852946	6852963	2	9	18	GA	21	10
4285	1	6853322	6853343	2	11	22	TG	17	12
4287	1	6855981	6856028	2	24	48	CA	11	8
4290	1	6862541	6862564	2	12	24	AC	7	7
4294	1	6866171	6866192	2	11	22	TG	22	11
4298	1	6880072	6880103	2	16	32	AC	7	9
4300	1	6886471	6886490	4	5	20	TTTA	12	3
4302	1	6895034	6895047	2	7	14	AT	15	5
4307	1	6920130	6920149	4	5	20	CATT	22	4
4313	1	6925551	6925598	4	12	48	TCTT	9	6
4319	1	6930348	6930371	4	6	24	TTTG	22	5
4324	1	6938281	6938306	2	13	26	TG	20	11
4326	1	6943913	6943930	3	6	18	TCC	29	3
4329	1	6971679	6971693	3	5	15	AAT	14	4
4330	1	6972824	6972838	3	5	15	CTG	28	3
4336	1	7000062	7000093	2	16	32	AC	15	14
4339	1	7005471	7005496	2	13	26	CA	12	8
4342	1	7008641	7008654	2	7	14	AC	22	4
4344	1	7008803	7008814	2	6	12	AC	33	3
4345	1	7013960	7013975	2	8	16	CT	26	5
4355	1	7042029	7042049	3	7	21	GAG	23	4
4368	1	7071335	7071354	2	10	20	TG	15	5
4373	1	7076957	7076982	2	13	26	CA	19	7
4379	1	7095555	7095566	2	6	12	AT	20	10
4380	1	7097838	7097849	2	6	12	AC	22	3
4386	1	7111668	7111682	3	5	15	TTG	19	10
4389	1	7113622	7113637	2	8	16	GT	24	9
4391	1	7122570	7122581	2	6	12	AT	27	3
4397	1	7138692	7138725	2	17	34	GT	9	7

4398	1	7138777	7138796	2	10	20	GT	17	11
4399	1	7139053	7139078	2	13	26	AC	19	17
4413	1	7183130	7183153	2	12	24	AC	8	9
4419	1	7196798	7196809	2	6	12	CT	19	4
4425	1	7228192	7228235	2	22	44	CA	16	7
4426	1	7232217	7232268	4	13	52	TTTC	18	7
4428	1	7244421	7244432	2	6	12	AC	21	4
4429	1	7246420	7246431	2	6	12	GT	25	9
4449	1	7293625	7293651	3	9	27	AAC	13	5
4450	1	7294726	7294761	4	9	36	AAAC	19	3
4451	1	7295623	7295646	3	8	24	TAA	21	5
4453	1	7302941	7302970	2	15	30	TG	12	8
4454	1	7302999	7303018	2	10	20	TG	10	3
4462	1	7344660	7344675	2	8	16	CA	27	4
4463	1	7345006	7345031	2	13	26	GT	23	9
4467	1	7351530	7351547	2	9	18	GT	17	13
4476	1	7384997	7385020	4	6	24	ATTT	16	12
4484	1	7400042	7400057	2	8	16	AC	28	3
4489	1	7409564	7409587	2	12	24	TG	20	13
4493	1	7415907	7415954	2	24	48	AC	9	10
4494	1	7419071	7419082	2	6	12	AC	24	10
4502	1	7454593	7454618	2	13	26	GT	23	8
4504	1	7461030	7461050	3	7	21	CTC	27	3
4507	1	7470655	7470670	2	8	16	AG	13	4
4515	1	7494670	7494695	2	13	26	CA	17	7
4521	1	7501615	7501630	2	8	16	CA	20	5
4525	1	7512873	7512888	4	4	16	ATTT	17	6
4529	1	7518469	7518486	2	9	18	TG	13	5
4533	1	7520632	7520655	4	6	24	TTTA	25	4
4541	1	7537845	7537884	4	10	40	TCTT	15	6
4543	1	7539417	7539430	2	7	14	CA	23	5
4551	1	7552944	7552955	2	6	12	CG	21	9
4552	1	7552956	7552975	2	10	20	CA	21	11
4554	1	7555972	7555995	4	6	24	GTTG	19	3
4561	1	7566752	7566775	2	12	24	GT	19	5
4584	1	7603086	7603101	2	8	16	GT	21	8
4598	1	7628236	7628271	2	18	36	AC	20	7
4609	1	7642887	7642908	2	11	22	CA	9	8
4610	1	7643056	7643071	2	8	16	CA	18	10
4612	1	7655389	7655408	2	10	20	AC	13	7
4615	1	7663392	7663409	2	9	18	CA	12	7
4621	1	7678732	7678746	3	5	15	TGT	26	3
4622	1	7688199	7688222	2	12	24	GT	8	10
4624	1	7690133	7690164	2	16	32	AC	11	5
4634	1	7701628	7701642	3	5	15	AAT	21	3
4639	1	7707800	7707833	2	17	34	GT	9	12
4642	1	7708550	7708575	2	13	26	GT	16	11
4643	1	7708817	7708838	2	11	22	TG	17	9
4649	1	7720836	7720871	3	12	36	TAA	10	8
4650	1	7724447	7724461	3	5	15	CTC	13	5
4651	1	7724469	7724483	3	5	15	TCC	14	4

4658	1	7734052	7734075	3	8	24	AAT	6	4
4659	1	7735224	7735253	2	15	30	CA	16	9
4673	1	7797117	7797130	2	7	14	AC	16	4
4675	1	7798182	7798215	2	17	34	CA	18	19
4678	1	7798902	7798916	3	5	15	AAG	29	4
4679	1	7802034	7802061	2	14	28	AC	20	5
4682	1	7806738	7806773	4	9	36	TATC	19	9
4685	1	7810859	7810878	4	5	20	AGAC	29	3
4687	1	7814884	7814913	2	15	30	TG	9	9
4689	1	7819958	7819973	2	8	16	GT	16	7
4690	1	7819974	7819987	2	7	14	GA	14	4
4692	1	7833216	7833236	3	7	21	ATT	13	6
4696	1	7845658	7845690	3	11	33	GTT	21	8
4698	1	7850079	7850098	2	10	20	TC	27	4
4699	1	7850099	7850118	4	5	20	GTTC	9	5
4701	1	7856028	7856045	3	6	18	GGA	29	4
4702	1	7856453	7856473	3	7	21	GGA	23	4
4715	1	7886442	7886457	2	8	16	AC	22	5
4721	1	7902417	7902428	2	6	12	GT	21	6
4740	1	7934651	7934678	4	7	28	CAGG	25	4
4742	1	7945505	7945528	3	8	24	CCT	14	4
4744	1	7946303	7946318	2	8	16	TG	20	3
4758	1	7964806	7964821	2	8	16	TG	14	3
4759	1	7964849	7964864	2	8	16	GA	11	7
4760	1	7964981	7965000	2	10	20	AC	18	5
4761	1	7967568	7967591	2	12	24	AC	8	9
4764	1	7970061	7970075	3	5	15	TGC	18	3
4769	1	7981152	7981169	2	9	18	TG	19	5
4772	1	7984178	7984213	3	12	36	TCA	29	9
4774	1	7987980	7987993	2	7	14	GA	29	3
4782	1	8004231	8004246	2	8	16	GT	10	8
4783	1	8004662	8004687	2	13	26	TG	13	8
4785	1	8005525	8005536	2	6	12	GT	15	12
4787	1	8005819	8005834	2	8	16	TG	19	8
4792	1	8028740	8028755	2	8	16	AC	20	4
4793	1	8029347	8029364	3	6	18	AAT	22	3
4802	1	8061792	8061803	2	6	12	TG	23	4
4810	1	8067090	8067110	3	7	21	TTC	25	7
4811	1	8071697	8071756	4	15	60	ACTA	9	5
4818	1	8096087	8096101	3	5	15	AGG	21	4
4823	1	8126732	8126761	2	15	30	GT	22	11
4829	1	8138034	8138051	2	9	18	AG	20	4
4830	1	8138954	8138969	2	8	16	TG	19	6
4835	1	8156503	8156522	2	10	20	CA	9	7
4836	1	8156706	8156737	4	8	32	GTCA	22	6
4837	1	8157071	8157134	2	32	64	GT	7	9
4842	1	8169181	8169198	3	6	18	CCT	22	3
4847	1	8177486	8177513	4	7	28	ATTT	6	4
4855	1	8191044	8191061	2	9	18	TG	9	9
4856	1	8191063	8191076	2	7	14	GT	8	7
4866	1	8207456	8207477	2	11	22	TG	13	8

4867	1	8207598	8207621	2	12	24	GT	17	9
4870	1	8214325	8214350	2	13	26	CA	14	13
4871	1	8214451	8214472	2	11	22	TC	16	5
4875	1	8216395	8216412	2	9	18	TG	26	7
4878	1	8225017	8225030	2	7	14	GT	26	5
4883	1	8249557	8249580	3	8	24	GTA	13	8
4887	1	8281594	8281650	3	19	57	ACT	18	10
4888	1	8284958	8284977	4	5	20	TTAT	23	11
4889	1	8285516	8285529	2	7	14	GT	16	11
4890	1	8286091	8286116	2	13	26	TG	16	7
4891	1	8287889	8287918	2	15	30	GT	10	11
4903	1	8316561	8316576	2	8	16	AG	23	4
4917	1	8369036	8369050	3	5	15	CAT	16	7
4919	1	8372545	8372562	3	6	18	CCT	16	6
4922	1	8374309	8374338	2	15	30	GT	16	13
4929	1	8381967	8381986	2	10	20	TG	26	7
4931	1	8384018	8384044	3	9	27	TCT	23	8
4939	1	8399767	8399787	3	7	21	TCT	23	4
4948	1	8409325	8409338	2	7	14	TG	14	12
4951	1	8409674	8409691	2	9	18	CA	12	10
4952	1	8409860	8409891	2	16	32	CA	23	8
4953	1	8410362	8410389	2	14	28	CA	18	10
4954	1	8411106	8411123	3	6	18	TGG	17	6
4968	1	8444729	8444752	3	8	24	GGA	29	5
4970	1	8452911	8452934	4	6	24	ATCT	15	11
4973	1	8460218	8460232	3	5	15	AAC	25	5
4978	1	8464854	8464871	2	9	18	AC	17	11
4982	1	8476671	8476686	2	8	16	GT	15	8
4983	1	8476689	8476706	2	9	18	GT	19	11
4987	1	8490945	8490962	2	9	18	AT	23	8
4990	1	8497191	8497204	2	7	14	AT	23	5
4991	1	8500142	8500162	3	7	21	GCA	27	10
5008	1	8529993	8530036	2	22	44	GT	11	14
5009	1	8533434	8533449	2	8	16	GT	10	5
5013	1	8539046	8539057	2	6	12	CA	26	4
5015	1	8540070	8540091	2	11	22	GT	9	8
5021	1	8548840	8548863	2	12	24	AG	13	4
5027	1	8561977	8561988	2	6	12	AC	30	3
5032	1	8577572	8577607	2	18	36	AG	6	5
5051	1	8608647	8608662	2	8	16	AC	19	3
5055	1	8610408	8610441	2	17	34	AC	10	11
5061	1	8630166	8630183	2	9	18	CT	26	5
5083	1	8676022	8676039	2	9	18	AC	21	3
5088	1	8682759	8682776	2	9	18	TC	20	4
5093	1	8692800	8692815	2	8	16	GT	21	7
5097	1	8695179	8695212	2	17	34	TG	17	9
5099	1	8697447	8697488	2	21	42	AC	9	8
5101	1	8700983	8700998	4	4	16	AGAC	23	5
5107	1	8707017	8707040	2	12	24	TG	18	11
5109	1	8710028	8710099	4	18	72	ATCT	19	8
5112	1	8717987	8718000	2	7	14	GT	20	7

5117	1	8724880	8724897	2	9	18	GT	24	7
5118	1	8724918	8724931	2	7	14	GT	6	3
5119	1	8729784	8729801	3	6	18	GCT	21	9
5120	1	8729802	8729816	3	5	15	GTG	19	9
5122	1	8730540	8730569	2	15	30	TG	19	18
5123	1	8731090	8731111	2	11	22	TG	15	18
5124	1	8731279	8731292	2	7	14	TG	21	8
5126	1	8738408	8738435	2	14	28	CA	22	10
5128	1	8743612	8743631	2	10	20	TG	9	8
5133	1	8760204	8760217	2	7	14	GT	27	3
5134	1	8760898	8760949	2	26	52	AC	9	11
5136	1	8773087	8773100	2	7	14	GA	21	3
5137	1	8773263	8773276	2	7	14	AC	18	4
5140	1	8779802	8779816	3	5	15	CCA	30	3
5141	1	8783255	8783284	2	15	30	AC	21	19
5143	1	8789524	8789538	3	5	15	CCT	10	8
5144	1	8790972	8790989	2	9	18	AC	20	6
5145	1	8791121	8791138	2	9	18	AG	18	5
5146	1	8791337	8791360	2	12	24	GT	16	7
5148	1	8795005	8795042	2	19	38	CA	11	10
5150	1	8797378	8797401	2	12	24	TG	8	7
5153	1	8805982	8806005	2	12	24	TG	24	9
5160	1	8821768	8821785	3	6	18	TGT	28	3
5167	1	8859263	8859284	2	11	22	TC	21	6
5174	1	8866881	8866904	2	12	24	TG	20	14
5187	1	8883992	8884003	2	6	12	TG	16	4
5189	1	8896150	8896179	2	15	30	GT	30	9
5204	1	8934838	8934861	4	6	24	AGAC	19	7
5209	1	8945710	8945730	3	7	21	ATG	24	3
5210	1	8949811	8949854	2	22	44	CT	7	8
5225	1	8969783	8969802	2	10	20	CA	20	14
5226	1	8973598	8973615	2	9	18	AG	23	3
5228	1	8980698	8980709	2	6	12	AC	31	3
5231	1	8989939	8989952	2	7	14	GT	21	6
5235	1	8991756	8991785	3	10	30	AAG	24	10
5240	1	9009089	9009108	2	10	20	TG	13	6
5245	1	9015066	9015095	2	15	30	AC	18	6
5246	1	9017136	9017161	2	13	26	TG	17	8
5248	1	9023005	9023031	3	9	27	CCA	26	3
5253	1	9036404	9036423	4	5	20	AAAT	32	4
5256	1	9043439	9043454	2	8	16	AC	26	6
5265	1	9053648	9053659	2	6	12	CT	18	3
5280	1	9084364	9084387	2	12	24	TG	9	4
5283	1	9089372	9089386	3	5	15	AAT	16	5
5285	1	9094181	9094196	4	4	16	AAAT	27	4
5289	1	9097140	9097169	2	15	30	AG	15	10
5296	1	9114537	9114551	3	5	15	TCG	29	5
5301	1	9123815	9123834	2	10	20	GT	17	15
5307	1	9128018	9128035	2	9	18	CA	9	9
5312	1	9139681	9139698	2	9	18	AC	15	9
5313	1	9140328	9140341	2	7	14	CA	30	4

5324	1	9168866	9168883	2	9	18	GT	14	9
5332	1	9183779	9183792	2	7	14	GA	18	5
5334	1	9191916	9191927	2	6	12	AG	18	3
5336	1	9194421	9194464	2	22	44	TG	16	15
5339	1	9201889	9201904	2	8	16	CT	18	7
5353	1	9219314	9219337	2	12	24	GT	7	6
5354	1	9219625	9219640	4	4	16	AAAC	6	4
5360	1	9230315	9230332	2	9	18	AC	14	12
5363	1	9240456	9240475	4	5	20	TTTA	23	4
5377	1	9274031	9274045	3	5	15	TAT	16	4
5381	1	9282706	9282726	3	7	21	TCT	25	5
5383	1	9286110	9286139	3	10	30	TGA	25	6
5386	1	9291593	9291632	2	20	40	CA	16	13
5392	1	9311614	9311625	2	6	12	GT	20	4
5393	1	9334170	9334185	2	8	16	GT	24	4
5394	1	9336950	9336970	3	7	21	CAA	23	4
5400	1	9354716	9354735	2	10	20	TG	21	4
5401	1	9360198	9360233	2	18	36	TG	13	13
5403	1	9369934	9369945	2	6	12	AT	28	3
5407	1	9375304	9375315	2	6	12	GA	24	5
5412	1	9377234	9377284	3	17	51	TTG	13	8
5420	1	9393284	9393323	2	20	40	AG	6	6
5426	1	9415605	9415616	2	6	12	CA	27	3
5427	1	9417792	9417809	2	9	18	AC	26	6
5430	1	9425346	9425359	2	7	14	GT	29	5
5435	1	9439398	9439421	2	12	24	CA	20	6
5441	1	9443695	9443750	2	28	56	GT	8	8
5453	1	9461995	9462006	2	6	12	AC	23	5
5454	1	9462010	9462023	2	7	14	CA	17	5
5465	1	9483037	9483050	2	7	14	GT	6	3
5466	1	9483129	9483152	2	12	24	TG	23	10
5469	1	9487297	9487312	4	4	16	ATTT	22	14
5475	1	9499510	9499531	2	11	22	TC	21	4
5479	1	9510222	9510243	2	11	22	GA	23	3
5487	1	9545406	9545433	2	14	28	TG	19	6
5489	1	9545522	9545545	2	12	24	GA	13	6
5491	1	9552251	9552264	2	7	14	TG	25	5
5493	1	9559313	9559328	2	8	16	CA	28	5
5499	1	9582102	9582115	2	7	14	AG	19	5
5503	1	9594408	9594435	2	14	28	GT	24	6
5508	1	9611333	9611372	2	20	40	AG	21	12
5516	1	9643240	9643265	2	13	26	CA	13	8
5526	1	9659307	9659324	2	9	18	TG	14	3
5528	1	9659911	9659926	2	8	16	TG	18	5
5536	1	9673540	9673559	2	10	20	TG	18	9
5537	1	9673679	9673700	2	11	22	TG	9	8
5541	1	9679214	9679225	2	6	12	AC	25	3
5550	1	9700454	9700468	3	5	15	CTG	27	3
5553	1	9702262	9702285	2	12	24	TG	19	4
5554	1	9712475	9712488	2	7	14	TG	8	10
5575	1	9772416	9772437	2	11	22	GT	7	7

5581	1	9782545	9782574	2	15	30	CA	6	8
5584	1	9791545	9791562	2	9	18	AC	12	3
5589	1	9807442	9807462	3	7	21	ATC	24	8
5590	1	9807682	9807705	4	6	24	TTTG	26	5
5591	1	9808301	9808312	2	6	12	AG	20	5
5593	1	9810899	9810918	2	10	20	GA	29	5
5594	1	9812315	9812330	2	8	16	GA	13	3
5599	1	9812947	9812961	3	5	15	TCC	17	7
5603	1	9820130	9820157	2	14	28	TG	17	8
5605	1	9831694	9831708	3	5	15	TTG	33	3
5618	1	9871042	9871073	2	16	32	GT	9	9
5620	1	9875106	9875117	2	6	12	GT	33	4
5621	1	9881842	9881873	2	16	32	CA	10	10
5628	1	9894429	9894454	2	13	26	TG	13	11
5630	1	9895943	9895958	2	8	16	CA	21	6
5632	1	9906618	9906686	3	23	69	AGA	18	16
5634	1	9917967	9917990	2	12	24	AC	16	9
5635	1	9920443	9920470	2	14	28	AC	11	8
5640	1	9927210	9927224	3	5	15	CAA	11	3
5644	1	9938456	9938487	4	8	32	TATC	9	7
5645	1	9939473	9939512	2	20	40	TG	8	7
5653	1	9982582	9982599	2	9	18	GT	10	7
5654	1	9982600	9982617	2	9	18	TG	11	7
5664	1	10016058	10016075	2	9	18	AC	20	6
5665	1	10016960	10016995	4	9	36	TTCT	13	9
5666	1	10018141	10018152	2	6	12	TA	14	4
5669	1	10024799	10024822	4	6	24	AATG	25	4
5671	1	10028904	10028918	3	5	15	CTG	31	4
5673	1	10031366	10031380	3	5	15	GCA	26	7
5674	1	10032721	10032736	2	8	16	AC	24	5
5678	1	10047352	10047363	2	6	12	GT	19	4
5680	1	10048840	10048859	2	10	20	CA	10	7
5687	1	10064525	10064562	2	19	38	CT	7	8
5691	1	10078811	10078831	3	7	21	AGC	32	5
5696	1	10082329	10082346	2	9	18	GT	13	7
5703	1	10107798	10107817	2	10	20	TG	14	3
5704	1	10121909	10121928	4	5	20	TTTG	23	10
5718	1	10146653	10146688	2	18	36	TG	6	7
5721	1	10166570	10166605	2	18	36	TG	9	10
5724	1	10167896	10167921	2	13	26	TG	19	8
5729	1	10191948	10191971	4	6	24	ATTT	13	3
5733	1	10198520	10198539	4	5	20	TTGT	19	4
5734	1	10218708	10218723	4	4	16	AACA	26	3
5736	1	10221360	10221391	2	16	32	AG	28	6
5739	1	10226830	10226843	2	7	14	AT	13	7
5746	1	10243663	10243680	3	6	18	CCT	15	4
5747	1	10245910	10245921	2	6	12	CA	26	6
5750	1	10252437	10252456	4	5	20	TGAA	23	3
5752	1	10253897	10253936	4	10	40	GAAA	21	6
5760	1	10287196	10287215	4	5	20	ACAG	29	4
5774	1	10305737	10305766	2	15	30	TG	12	10

5777	1	10312554	10312565	2	6	12	TG	30	4
5778	1	10313556	10313571	2	8	16	GT	18	6
5782	1	10320694	10320719	2	13	26	GT	8	7
5783	1	10321169	10321188	2	10	20	GT	22	5
5784	1	10321299	10321314	2	8	16	AC	22	7
5785	1	10321506	10321529	2	12	24	AC	18	4
5789	1	10322899	10322912	2	7	14	GT	11	6
5794	1	10324807	10324818	2	6	12	GT	30	3
5800	1	10329933	10329950	3	6	18	CCT	32	4
5806	1	10335858	10335909	2	26	52	TG	6	9
5812	1	10346965	10346990	2	13	26	AC	21	6
5815	1	10351617	10351630	2	7	14	CA	18	3
5821	1	10359602	10359619	3	6	18	TCC	19	3
5830	1	10391300	10391319	4	5	20	AATG	20	3
5833	1	10394223	10394240	2	9	18	GT	11	7
5834	1	10394243	10394260	2	9	18	GT	9	6
5839	1	10405024	10405037	2	7	14	TG	22	4
5840	1	10405190	10405203	2	7	14	GT	23	4
5844	1	10411103	10411126	3	8	24	CAG	22	6
5845	1	10413003	10413014	2	6	12	GT	28	3
5848	1	10421280	10421351	4	18	72	AGAT	19	9
5852	1	10423600	10423617	3	6	18	TTG	7	4
5853	1	10426168	10426183	2	8	16	GA	26	6
5855	1	10427051	10427062	2	6	12	TG	31	3
5859	1	10435466	10435504	3	13	39	GAA	21	12
5860	1	10436968	10437001	2	17	34	AC	11	8
5861	1	10441837	10441866	3	10	30	CCT	12	4
5863	1	10442435	10442460	2	13	26	CA	16	5
5864	1	10442536	10442559	2	12	24	GT	15	8
5871	1	10456594	10456607	2	7	14	AC	30	4
5875	1	10472184	10472201	2	9	18	CA	26	4
5876	1	10474999	10475038	2	20	40	CA	12	11
5880	1	10480456	10480473	3	6	18	TGT	20	4
5889	1	10542249	10542263	3	5	15	GCA	22	4
5890	1	10547375	10547408	2	17	34	CA	10	8
5899	1	10554785	10554802	2	9	18	AC	27	4
5902	1	10560462	10560475	2	7	14	GT	25	4
5906	1	10564469	10564482	2	7	14	TG	19	3
5911	1	10584971	10584982	2	6	12	TG	23	6
5912	1	10592288	10592307	4	5	20	TATC	21	3
5914	1	10598450	10598463	2	7	14	CA	31	3
5922	1	10627904	10627927	3	8	24	TTC	19	7
5924	1	10631090	10631119	2	15	30	TG	13	5
5936	1	10661560	10661586	3	9	27	TAA	12	10
5949	1	10698327	10698344	3	6	18	TGA	13	3
5977	1	10757388	10757429	2	21	42	AC	13	8
5980	1	10764930	10764951	2	11	22	CA	11	13
5982	1	10765571	10765588	2	9	18	GT	22	9
5983	1	10766878	10766895	2	9	18	AC	27	3
5984	1	10767800	10767823	4	6	24	CATA	11	3
5985	1	10769431	10769450	2	10	20	TG	23	4

5989	1	10775329	10775350	2	11	22	AC	10	9
5991	1	10776163	10776188	2	13	26	TG	12	9
5992	1	10776483	10776508	2	13	26	GT	9	10
5994	1	10779696	10779707	2	6	12	TC	30	3
5995	1	10779914	10779929	2	8	16	CA	26	7
5996	1	10782065	10782088	2	12	24	GT	28	4
5998	1	10787647	10787667	3	7	21	GAT	16	4
6002	1	10791433	10791450	2	9	18	GT	8	5
6003	1	10798488	10798520	3	11	33	CAT	24	10
6004	1	10802659	10802670	2	6	12	TA	18	5
6006	1	10808798	10808812	3	5	15	TTG	24	7
6007	1	10817477	10817500	4	6	24	TCTG	24	4
6016	1	10835893	10835906	2	7	14	CT	28	12
6017	1	10835907	10835920	2	7	14	CA	16	6
6018	1	10836028	10836049	2	11	22	TG	13	8
6024	1	10839430	10839441	2	6	12	TG	33	8
6028	1	10862635	10862656	2	11	22	TG	16	6
6032	1	10869214	10869225	2	6	12	GC	7	4
6033	1	10871358	10871387	2	15	30	TG	9	12
6034	1	10878018	10878029	2	6	12	AC	26	4
6036	1	10880916	10880939	2	12	24	TG	14	10
6037	1	10881078	10881099	2	11	22	CA	16	12
6044	1	10885672	10885698	3	9	27	AGA	26	11
6048	1	10887192	10887203	2	6	12	TC	18	5
6050	1	10887721	10887742	2	11	22	GT	26	10
6054	1	10890096	10890115	2	10	20	TA	8	8
6057	1	10892378	10892395	2	9	18	GT	6	4
6058	1	10892846	10892865	2	10	20	GT	20	6
6061	1	10894424	10894435	2	6	12	GT	22	11
6071	1	10927199	10927226	2	14	28	GT	15	8
6072	1	10931267	10931282	2	8	16	GT	19	3
6078	1	10955626	10955641	4	4	16	CACG	18	6
6090	1	10988672	10988691	2	10	20	AC	14	12
6091	1	10988933	10988952	2	10	20	CA	22	7
6092	1	10990671	10990697	3	9	27	CAT	25	4
6093	1	10992948	10992971	2	12	24	CA	18	3
6095	1	10995080	10995093	2	7	14	CT	32	3
6096	1	10996736	10996763	2	14	28	TG	9	10
6097	1	10996862	10996879	2	9	18	CA	15	5
6099	1	10997437	10997460	4	6	24	ATAA	16	4
6100	1	11000493	11000512	4	5	20	AGAC	11	6
6105	1	11013851	11013862	2	6	12	AT	7	3
6107	1	11018779	11018802	2	12	24	TG	9	4
6108	1	11018883	11018918	2	18	36	GT	10	11
6109	1	11019711	11019728	3	6	18	AAC	17	7
6111	1	11026878	11026927	2	25	50	AC	7	10
6121	1	11048540	11048563	2	12	24	AC	14	14
6131	1	11087411	11087428	2	9	18	AC	10	4
6134	1	11098292	11098309	3	6	18	AAT	11	10
6135	1	11098770	11098785	4	4	16	AAAC	20	4
6142	1	11112443	11112457	3	5	15	CAG	29	7

6143	1	11113756	11113767	2	6	12	TA	22	5
6146	1	11119674	11119699	2	13	26	GA	13	8
6150	1	11130368	11130391	4	6	24	GAAT	10	3
6155	1	11142215	11142228	2	7	14	CA	21	3
6158	1	11149677	11149694	3	6	18	TTG	10	7
6160	1	11158593	11158616	4	6	24	AAAC	23	4
6162	1	11159121	11159160	2	20	40	GT	11	10
6165	1	11167718	11167737	4	5	20	CATA	31	3
6173	1	11178514	11178543	2	15	30	CA	12	11
6191	1	11215079	11215092	2	7	14	GT	16	11
6192	1	11218825	11218856	2	16	32	AC	13	12
6194	1	11221803	11221817	3	5	15	GCA	21	3
6212	1	11261933	11261944	2	6	12	AC	8	3
6215	1	11267406	11267423	2	9	18	AG	17	4
6219	1	11269624	11269635	2	6	12	TG	30	3
6221	1	11271008	11271023	2	8	16	GT	15	12
6244	1	11324189	11324200	2	6	12	TG	17	3
6248	1	11336724	11336741	2	9	18	GT	19	6
6249	1	11337060	11337083	2	12	24	GT	17	8
6253	1	11340400	11340413	2	7	14	GT	23	4
6254	1	11340621	11340635	3	5	15	AGG	23	3
6256	1	11348496	11348525	2	15	30	GT	13	13
6259	1	11353584	11353599	2	8	16	AG	11	8
6261	1	11353729	11353744	4	4	16	TTGT	20	4
6264	1	11356576	11356607	2	16	32	CT	13	12
6267	1	11359858	11359872	3	5	15	GAG	17	5
6269	1	11360152	11360169	2	9	18	GT	7	6
6271	1	11360533	11360552	2	10	20	TG	14	10
6272	1	11360891	11360910	2	10	20	AG	9	9
6282	1	11368054	11368065	2	6	12	AC	17	10
6283	1	11368156	11368187	2	16	32	GT	17	13
6284	1	11368322	11368369	2	24	48	AC	10	10
6291	1	11393291	11393316	2	13	26	GT	13	17
6294	1	11407956	11407975	2	10	20	TG	19	6
6295	1	11408208	11408235	2	14	28	TG	11	10
6296	1	11410041	11410070	2	15	30	TG	7	10
6307	1	11417857	11417882	2	13	26	CA	13	7
6309	1	11418142	11418185	2	22	44	GT	15	12
6314	1	11433030	11433045	2	8	16	CA	23	6
6316	1	11435682	11435696	3	5	15	CCT	19	3
6317	1	11436437	11436456	2	10	20	GT	21	12
6319	1	11436824	11436837	2	7	14	AC	19	4
6321	1	11437624	11437637	2	7	14	GT	18	11
6323	1	11441040	11441063	2	12	24	TG	11	12
6329	1	11454888	11454908	3	7	21	TCC	26	9
6331	1	11456273	11456296	2	12	24	TG	20	11
6332	1	11459332	11459343	2	6	12	TG	28	3
6335	1	11462748	11462763	2	8	16	AC	22	5
6344	1	11506924	11506939	2	8	16	GT	22	12
6345	1	11506942	11506961	4	5	20	GTGC	25	15
6347	1	11508405	11508420	2	8	16	CA	19	13

6350	1	11516629	11516660	2	16	32	TG	6	8
6352	1	11517725	11517744	4	5	20	GTTT	21	5
6360	1	11538788	11538808	3	7	21	AAG	23	6
6361	1	11543681	11543694	2	7	14	AC	12	3
6365	1	11548982	11549001	2	10	20	GT	13	12
6367	1	11551476	11551491	4	4	16	GACA	24	4
6370	1	11562862	11562903	2	21	42	GT	7	9
6371	1	11562993	11563016	2	12	24	TG	9	9
6374	1	11565933	11565950	2	9	18	TG	19	4
6377	1	11570710	11570733	2	12	24	TG	15	7
6379	1	11576110	11576137	2	14	28	CA	13	7
6387	1	11587787	11587802	4	4	16	TATT	28	4
6397	1	11596521	11596544	2	12	24	TG	17	6
6398	1	11596691	11596710	2	10	20	TG	17	5
6400	1	11598787	11598816	2	15	30	AC	15	6
6406	1	11603731	11603746	4	4	16	ACAA	22	3
6407	1	11604128	11604145	2	9	18	TG	17	9
6408	1	11604530	11604541	2	6	12	CA	22	5
6410	1	11606952	11606973	2	11	22	AC	15	13
6413	1	11610531	11610563	3	11	33	CTC	28	7
6415	1	11613660	11613685	2	13	26	GT	17	6
6416	1	11615853	11615876	2	12	24	TG	19	8
6417	1	11621229	11621244	2	8	16	GT	20	4
6422	1	11634619	11634630	2	6	12	GT	30	3
6424	1	11639371	11639388	3	6	18	TGA	26	3
6425	1	11639498	11639511	2	7	14	AC	8	3
6426	1	11639770	11639785	2	8	16	TC	27	4
6427	1	11642953	11642992	4	10	40	TCTT	15	7
6429	1	11645991	11646028	2	19	38	GT	11	14
6438	1	11664404	11664431	4	7	28	TCAA	17	7
6439	1	11665268	11665283	2	8	16	GT	11	6
6444	1	11676601	11676616	2	8	16	CA	10	10
6445	1	11679006	11679033	2	14	28	GT	9	5
6446	1	11679182	11679201	2	10	20	CA	22	9
6450	1	11682603	11682626	4	6	24	AACC	15	7
6452	1	11684472	11684489	2	9	18	TG	15	3
6453	1	11684601	11684614	2	7	14	CA	16	13
6454	1	11685010	11685027	2	9	18	GA	22	5
6455	1	11685869	11685886	2	9	18	AC	27	5
6456	1	11686066	11686085	2	10	20	AC	21	7
6459	1	11690349	11690370	2	11	22	CA	25	5
6461	1	11690509	11690530	2	11	22	AC	20	12
6470	1	11695642	11695685	4	11	44	ATGG	20	7
6474	1	11706645	11706658	2	7	14	CA	12	9
6475	1	11706808	11706823	2	8	16	TG	10	9
6476	1	11706964	11706991	2	14	28	GT	10	5
6477	1	11710006	11710017	2	6	12	GA	25	3
6478	1	11710135	11710162	2	14	28	GT	11	12
6479	1	11715043	11715066	2	12	24	TG	18	5
6482	1	11719691	11719712	2	11	22	AC	20	8
6485	1	11725746	11725787	2	21	42	AC	6	6

6486	1	11725891	11725908	2	9	18	TC	19	6
6492	1	11727811	11727844	2	17	34	GT	9	6
6497	1	11738016	11738055	2	20	40	AC	11	12
6499	1	11741153	11741170	2	9	18	CA	19	6
6508	1	11748363	11748386	2	12	24	TC	20	6
6509	1	11753388	11753402	3	5	15	TCT	20	4
6513	1	11754167	11754178	2	6	12	AG	18	4
6514	1	11755647	11755686	4	10	40	GATA	21	11
6520	1	11767773	11767786	2	7	14	AC	10	8
6527	1	11784040	11784069	2	15	30	CT	12	10
6540	1	11811701	11811716	2	8	16	GT	18	6
6543	1	11812025	11812036	2	6	12	AC	20	10
6545	1	11812468	11812489	2	11	22	CA	14	4
6546	1	11814283	11814300	2	9	18	GT	26	8
6547	1	11814421	11814446	2	13	26	GT	18	8
6555	1	11819408	11819428	3	7	21	TCC	22	4
6556	1	11820781	11820794	2	7	14	AC	27	5
6577	1	11844615	11844628	2	7	14	AC	21	3
6578	1	11847274	11847287	2	7	14	TA	25	6
6581	1	11852813	11852824	2	6	12	AC	23	3
6587	1	11858611	11858637	3	9	27	TGC	27	4
6592	1	11866538	11866573	2	18	36	GT	13	9
6593	1	11866768	11866782	3	5	15	CTC	22	4
6594	1	11876046	11876061	4	4	16	ATTC	23	3
6611	1	11933151	11933170	2	10	20	GT	24	7
6613	1	11937041	11937066	2	13	26	TG	18	8
6615	1	11944497	11944516	4	5	20	AGAA	27	4
6616	1	11945549	11945580	2	16	32	TG	19	14
6620	1	11951165	11951186	2	11	22	CA	17	10
6640	1	11972400	11972419	2	10	20	GA	7	5
6645	1	11978940	11978975	2	18	36	AC	6	6
6665	1	12020723	12020740	2	9	18	AC	9	5
6666	1	12020814	12020828	3	5	15	CAG	24	3
6677	1	12042861	12042899	3	13	39	ATC	22	13
6683	1	12047732	12047765	2	17	34	CT	10	10
6685	1	12051279	12051296	2	9	18	TC	19	3
6696	1	12065132	12065145	2	7	14	TG	17	7
6699	1	12065901	12065924	3	8	24	GTT	22	5
6705	1	12094207	12094226	2	10	20	TA	16	6
6709	1	12102686	12102699	2	7	14	AC	13	5
6718	1	12113025	12113040	4	4	16	TTTG	19	4
6719	1	12113128	12113151	4	6	24	TATT	17	4
6727	1	12137506	12137529	4	6	24	AAAC	20	4
6728	1	12140226	12140245	2	10	20	AC	17	14
6738	1	12154371	12154402	2	16	32	CA	7	10
6742	1	12159045	12159066	2	11	22	CA	16	7
6743	1	12159120	12159145	2	13	26	CA	19	13
6744	1	12162213	12162232	4	5	20	AAAC	25	4
6748	1	12168259	12168270	2	6	12	GT	23	17
6755	1	12174510	12174531	2	11	22	AT	7	7
6756	1	12177184	12177199	4	4	16	CAGA	23	3

6759	1	12181003	12181030	2	14	28	GT	9	7
6763	1	12201591	12201611	3	7	21	CTG	34	4
6778	1	12217664	12217691	2	14	28	AC	10	11
6781	1	12218929	12218946	3	6	18	ATT	16	4
6785	1	12219578	12219591	2	7	14	TG	15	8
6790	1	12224669	12224696	2	14	28	TG	14	9
6801	1	12236272	12236286	3	5	15	CCT	24	4
6810	1	12253268	12253295	2	14	28	GT	25	9
6811	1	12255150	12255161	2	6	12	CT	27	7
6813	1	12257950	12257965	4	4	16	GACT	15	6
6814	1	12257966	12257985	4	5	20	GGCT	16	7
6816	1	12260593	12260608	2	8	16	CA	14	13
6817	1	12260611	12260624	2	7	14	CA	15	11
6825	1	12276019	12276050	2	16	32	TC	8	7
6829	1	12282442	12282453	2	6	12	GT	35	3
6830	1	12286281	12286296	2	8	16	AC	26	3
6840	1	12309394	12309413	2	10	20	TG	19	6
6841	1	12314042	12314059	2	9	18	CA	19	4
6861	1	12368663	12368690	2	14	28	TG	14	9
6864	1	12369806	12369833	2	14	28	GT	6	4
6865	1	12375172	12375199	2	14	28	CA	6	7
6867	1	12375784	12375803	2	10	20	TG	7	7
6869	1	12378663	12378676	2	7	14	AC	18	5
6873	1	12384591	12384614	3	8	24	CTG	20	3
6884	1	12389199	12389234	2	18	36	TG	20	11
6908	1	12476335	12476358	2	12	24	TC	13	9
6915	1	12487227	12487260	2	17	34	AC	9	13
6918	1	12491631	12491646	4	4	16	TCTT	25	7
6938	1	12542170	12542187	2	9	18	AC	17	13
6939	1	12542573	12542610	2	19	38	CA	13	10
6945	1	12552828	12552841	2	7	14	CA	17	3
6956	1	12573987	12573998	2	6	12	AC	22	6
6965	1	12610387	12610404	3	6	18	AAT	24	8
6970	1	12622063	12622078	2	8	16	CA	18	7
6982	1	12643440	12643453	2	7	14	AG	19	5
6983	1	12644003	12644017	3	5	15	AAT	9	3
7010	1	12696268	12696281	2	7	14	TG	22	15
7011	1	12696283	12696296	2	7	14	GT	16	12
7018	1	12701308	12701323	4	4	16	GGTT	24	6
7023	1	12721740	12721754	3	5	15	TCC	17	3
7024	1	12723815	12723836	2	11	22	AC	9	9
7027	1	12732186	12732205	4	5	20	AAAT	19	6
7029	1	12741903	12741918	4	4	16	AAAG	25	3
7032	1	12743761	12743776	2	8	16	GA	25	4
7035	1	12762374	12762401	4	7	28	AAGA	18	7
7043	1	12789750	12789763	2	7	14	AT	18	5
7046	1	12803166	12803180	3	5	15	AAG	24	5
7054	1	12832836	12832855	2	10	20	AC	21	5
7059	1	12842379	12842390	2	6	12	GT	17	5
7064	1	12852309	12852324	4	4	16	TTTG	20	5
7066	1	12854471	12854482	2	6	12	AC	7	6

7095	1	12883142	12883173	2	16	32	GT	14	6
7096	1	12883258	12883277	2	10	20	GT	17	4
7097	1	12893584	12893603	2	10	20	AC	21	4
7102	1	12904957	12904978	2	11	22	GT	23	8
7108	1	12911384	12911409	2	13	26	AG	18	5
7115	1	12959757	12959786	2	15	30	AC	8	5
7123	1	12995860	12995880	3	7	21	CTG	6	6
7130	1	13004221	13004236	2	8	16	AG	20	4
7140	1	13045718	13045739	2	11	22	GT	16	10
7146	1	13072164	13072178	3	5	15	AAT	10	3
7161	1	13116924	13116943	2	10	20	AC	18	6
7166	1	13132411	13132424	2	7	14	AT	8	3
7185	1	13159708	13159737	2	15	30	AC	9	12
7189	1	13177156	13177173	2	9	18	AG	21	5
7194	1	13178448	13178473	2	13	26	CA	11	6
7196	1	13178649	13178666	2	9	18	GA	19	3
7204	1	13183856	13183877	2	11	22	GA	12	6
7205	1	13183913	13183928	2	8	16	GA	17	3
7207	1	13187005	13187020	4	4	16	AAAC	30	4
7240	1	13236790	13236805	2	8	16	AC	26	3
7252	1	13266880	13266906	3	9	27	CTC	24	4
7253	1	13267506	13267517	2	6	12	GA	32	3
7262	1	13292740	13292754	3	5	15	CCT	30	3
7263	1	13292921	13292964	2	22	44	AC	9	8
7269	1	13305980	13305994	3	5	15	CCT	7	5
7270	1	13306827	13306844	3	6	18	AAG	25	3
7287	1	13330914	13330925	2	6	12	CA	22	4
7288	1	13332011	13332034	2	12	24	TC	18	8
7323	1	13384363	13384376	2	7	14	GT	21	3
7333	1	13394446	13394465	2	10	20	TG	23	5
7335	1	13399011	13399036	2	13	26	TC	13	7
7337	1	13399080	13399091	2	6	12	AC	13	8
7348	1	13417947	13417967	3	7	21	ATT	14	4
7349	1	13420231	13420282	4	13	52	TATC	18	9
7350	1	13420463	13420477	3	5	15	AAC	26	4
7351	1	13420607	13420662	4	14	56	AGAT	15	12
7352	1	13422011	13422025	3	5	15	TTG	21	5
7353	1	13427369	13427389	3	7	21	TGA	26	8
7359	1	13436890	13436907	3	6	18	TAA	12	8
7374	1	13457359	13457398	2	20	40	AC	10	12
7376	1	13461384	13461399	2	8	16	AC	19	7
7385	1	13479644	13479669	2	13	26	AC	10	7
7386	1	13481348	13481368	3	7	21	CAG	28	8
7401	1	13493149	13493178	2	15	30	GT	10	13
7406	1	13508038	13508053	2	8	16	CA	19	4
7409	1	13511293	13511308	4	4	16	AAAT	22	4
7413	1	13520450	13520465	2	8	16	TG	16	5
7417	1	13525505	13525516	2	6	12	AC	33	3
7424	1	13547082	13547095	2	7	14	TG	28	3
7425	1	13549541	13549552	2	6	12	TG	17	5
7437	1	13564386	13564401	2	8	16	AC	16	3

7442	1	13573084	13573095	2	6	12	TA	16	8
7444	1	13574646	13574663	3	6	18	AAC	21	12
7446	1	13575011	13575026	4	4	16	CAAA	21	10
7456	1	13600575	13600595	3	7	21	AGG	13	3
7457	1	13602563	13602574	2	6	12	CA	33	4
7458	1	13602941	13602964	2	12	24	GT	27	3
7463	1	13613867	13613882	2	8	16	CA	27	4
7483	1	13657252	13657269	2	9	18	GT	29	5
7488	1	13664476	13664490	3	5	15	GCC	23	3
7495	1	13698591	13698614	3	8	24	TGA	29	9
7507	1	13723861	13723881	3	7	21	AAG	21	3
7516	1	13733053	13733067	3	5	15	TTC	21	3
7518	1	13734778	13734797	4	5	20	TTTC	16	6
7520	1	13738962	13738979	2	9	18	TC	30	6
7521	1	13743090	13743107	2	9	18	TG	20	7
7522	1	13744053	13744070	2	9	18	TC	23	4
7529	1	13758995	13759014	2	10	20	CA	17	11
7532	1	13759157	13759168	2	6	12	GA	20	7
7533	1	13759226	13759241	2	8	16	GA	22	3
7535	1	13771809	13771838	2	15	30	CA	20	7
7544	1	13797429	13797460	4	8	32	CTTT	24	5
7547	1	13807125	13807140	2	8	16	TG	14	4
7554	1	13823735	13823768	2	17	34	AC	8	13
7555	1	13823939	13823962	4	6	24	CAAA	27	5
7557	1	13832030	13832047	2	9	18	GT	27	7
7563	1	13840414	13840439	2	13	26	TG	14	9
7566	1	13846116	13846127	2	6	12	TC	28	6
7569	1	13850497	13850522	2	13	26	GT	17	10
7570	1	13851901	13851916	2	8	16	AC	22	5
7578	1	13869233	13869244	2	6	12	GA	26	3
7580	1	13881150	13881181	2	16	32	CA	8	7
7583	1	13883407	13883418	2	6	12	AC	24	8
7584	1	13883421	13883434	2	7	14	AC	22	8
7591	1	13888064	13888093	2	15	30	GT	12	7
7593	1	13889562	13889579	2	9	18	AG	17	6
7600	1	13897505	13897528	2	12	24	GA	26	5
7602	1	13899599	13899618	4	5	20	TTAT	17	8
7606	1	13907467	13907478	2	6	12	TG	25	3
7623	1	13944490	13944507	2	9	18	AC	16	4
7625	1	13953824	13953837	2	7	14	AC	21	5
7629	1	13962965	13962978	2	7	14	CA	22	5
7647	1	13981693	13981710	3	6	18	TGG	27	6
7651	1	13984548	13984573	2	13	26	TG	14	6
7653	1	13984655	13984682	2	14	28	AG	10	10
7659	1	14000870	14000929	2	30	60	TG	13	9
7665	1	14007646	14007661	2	8	16	TA	12	3
7667	1	14014708	14014731	3	8	24	AGC	14	5
7673	1	14033952	14033975	2	12	24	AC	26	5
7675	1	14037190	14037204	3	5	15	GCT	30	3
7676	1	14056661	14056674	2	7	14	TG	29	3
7679	1	14067931	14067960	2	15	30	AC	10	10

7681	1	14076080	14076101	2	11	22	CA	21	25
7685	1	14085319	14085333	3	5	15	GGA	25	5
7689	1	14099180	14099194	3	5	15	TGT	21	3
7693	1	14103590	14103607	3	6	18	CCT	17	7
7694	1	14104288	14104305	2	9	18	TG	13	7
7699	1	14110575	14110596	2	11	22	TG	17	7
7704	1	14117628	14117641	2	7	14	TG	20	3
7710	1	14127209	14127224	4	4	16	TTTA	32	4
7718	1	14140003	14140036	2	17	34	AC	10	8
7720	1	14140518	14140547	2	15	30	AC	16	10
7721	1	14143715	14143754	2	20	40	CT	19	5
7727	1	14156449	14156462	2	7	14	TG	22	8
7731	1	14165753	14165770	2	9	18	CA	17	6
7734	1	14175642	14175659	3	6	18	CAG	29	3
7743	1	14195443	14195463	3	7	21	GAT	31	4
7744	1	14197663	14197702	4	10	40	TCTT	14	8
7755	1	14224163	14224190	2	14	28	GT	20	7
7758	1	14227805	14227818	2	7	14	AC	27	3
7768	1	14243341	14243362	2	11	22	GA	6	6
7770	1	14246663	14246712	2	25	50	TG	8	12
7771	1	14248684	14248715	2	16	32	GT	15	13
7786	1	14292057	14292080	2	12	24	TG	17	13
7788	1	14292888	14292902	3	5	15	ACT	23	3
7796	1	14305375	14305404	2	15	30	TG	6	7
7797	1	14305790	14305833	2	22	44	CA	13	11
7798	1	14306700	14306737	2	19	38	CA	12	13
7802	1	14309919	14309936	2	9	18	CA	25	10
7803	1	14310474	14310499	2	13	26	AC	20	10
7804	1	14311019	14311057	3	13	39	CTT	9	10
7810	1	14312918	14312935	3	6	18	AAG	15	4
7813	1	14316157	14316168	2	6	12	AG	13	8
7819	1	14318154	14318183	2	15	30	GT	15	12
7822	1	14320637	14320684	3	16	48	GAT	18	8
7824	1	14323829	14323849	3	7	21	ATG	11	5
7826	1	14324432	14324459	2	14	28	AC	12	16
7836	1	14341022	14341057	2	18	36	CT	8	7
7837	1	14347651	14347672	2	11	22	GT	14	8
7838	1	14347732	14347771	2	20	40	TG	10	11
7844	1	14362563	14362582	2	10	20	AC	19	10
7854	1	14372435	14372458	2	12	24	CA	14	5
7855	1	14372647	14372666	2	10	20	CA	19	5
7858	1	14375118	14375131	2	7	14	GT	28	5
7861	1	14386702	14386733	2	16	32	TG	11	10
7864	1	14393612	14393639	2	14	28	GT	17	7
7866	1	14396751	14396780	2	15	30	TG	12	9
7871	1	14402230	14402257	2	14	28	AC	10	6
7877	1	14436882	14436905	2	12	24	TG	21	6
7880	1	14444089	14444118	3	10	30	GAG	21	7
7881	1	14445458	14445469	2	6	12	GT	22	3
7886	1	14450612	14450643	2	16	32	CA	19	8
7887	1	14453363	14453382	2	10	20	AC	24	4

7889	1	14455219	14455246	2	14	28	AC	20	8
7892	1	14461287	14461330	2	22	44	AC	11	12
7894	1	14462077	14462096	2	10	20	GT	16	6
7896	1	14464816	14464829	2	7	14	GT	11	4
7900	1	14467827	14467844	2	9	18	CA	10	9
7902	1	14473232	14473245	2	7	14	TG	23	4
7911	1	14499087	14499104	2	9	18	AC	26	4
7913	1	14506312	14506326	3	5	15	TCG	30	6
7925	1	14517129	14517152	4	6	24	TCCT	25	4
7930	1	14524473	14524484	2	6	12	AC	28	5
7931	1	14524688	14524703	2	8	16	CA	11	3
7933	1	14525533	14525546	2	7	14	TG	26	5
7946	1	14557418	14557441	2	12	24	TG	12	9
7948	1	14570243	14570260	2	9	18	GA	18	9
7956	1	14575821	14575852	2	16	32	CA	14	9
7958	1	14577092	14577115	3	8	24	CAG	26	4
7971	1	14591473	14591500	2	14	28	GT	6	3
7974	1	14592346	14592359	2	7	14	GT	23	5
7978	1	14596120	14596135	4	4	16	TCTG	23	4
7982	1	14603010	14603025	2	8	16	GA	12	5
7983	1	14603033	14603044	2	6	12	AG	8	8
7984	1	14603049	14603060	2	6	12	AG	16	8
7988	1	14607753	14607770	2	9	18	AC	26	6
7989	1	14608224	14608253	3	10	30	GTT	23	10
7991	1	14609685	14609702	3	6	18	TTG	24	3
8000	1	14621123	14621152	2	15	30	CT	9	8
8002	1	14621688	14621731	2	22	44	CT	9	10
8004	1	14625270	14625281	2	6	12	TG	16	5
8009	1	14635103	14635114	2	6	12	GT	22	3
8016	1	14639731	14639746	2	8	16	TC	24	3
8017	1	14642139	14642156	2	9	18	GT	20	8
8019	1	14647324	14647338	3	5	15	TCA	25	3
8021	1	14652359	14652374	4	4	16	AAAT	19	8
8023	1	14653537	14653554	3	6	18	AAG	25	5
8024	1	14654333	14654344	2	6	12	TC	25	3
8026	1	14656745	14656759	3	5	15	ACA	22	5
8029	1	14658249	14658272	3	8	24	GCT	24	8
8031	1	14668226	14668281	2	28	56	GT	9	12
8032	1	14669858	14669869	2	6	12	TA	15	4
8040	1	14678505	14678520	2	8	16	GT	26	7
8042	1	14684867	14684882	2	8	16	TG	19	7
8044	1	14688879	14688908	3	10	30	CAC	23	4
8049	1	14693985	14694014	2	15	30	TG	20	5
8050	1	14694444	14694461	2	9	18	TG	23	5
8051	1	14695579	14695608	2	15	30	CA	13	5
8058	1	14700814	14700843	2	15	30	AC	8	8
8067	1	14711285	14711302	3	6	18	AGA	19	4
8068	1	14711820	14711845	2	13	26	AC	19	8
8069	1	14712281	14712310	2	15	30	CT	17	10
8072	1	14724789	14724816	2	14	28	TG	13	7
8073	1	14728040	14728059	2	10	20	TC	13	4

8076	1	14728707	14728724	2	9	18	TC	11	3
8082	1	14736169	14736184	4	4	16	GAAA	13	6
8083	1	14736699	14736732	2	17	34	AC	18	7
8084	1	14736926	14736941	4	4	16	GTCT	27	6
8085	1	14737518	14737532	3	5	15	TTA	6	3
8086	1	14739185	14739204	4	5	20	ATAG	32	3
8087	1	14739654	14739679	2	13	26	TG	13	7
8088	1	14739991	14740014	2	12	24	AG	22	7
8101	1	14750664	14750683	4	5	20	CTAG	20	3
8118	1	14790192	14790207	2	8	16	GT	15	5
8119	1	14790347	14790370	3	8	24	CTG	20	3
8124	1	14791624	14791639	4	4	16	AACA	27	3
8133	1	14809901	14809926	2	13	26	GT	15	9
8135	1	14813738	14813753	2	8	16	GA	15	11
8136	1	14813755	14813768	2	7	14	AG	19	13
8140	1	14822646	14822661	2	8	16	TA	14	5
8144	1	14824908	14824923	2	8	16	GT	19	9
8145	1	14825058	14825089	2	16	32	TG	19	8
8146	1	14825574	14825618	3	15	45	CTT	22	9
8148	1	14829014	14829040	3	9	27	GAG	29	5
8150	1	14832151	14832166	4	4	16	ATTT	10	3
8157	1	14840174	14840213	2	20	40	AC	7	9
8158	1	14842307	14842322	4	4	16	TTTA	25	5
8164	1	14853181	14853192	2	6	12	TC	29	4
8183	1	14877484	14877515	2	16	32	CA	12	7
8185	1	14878105	14878124	4	5	20	GGTT	27	3
8189	1	14883120	14883131	2	6	12	CT	23	3
8190	1	14887001	14887028	2	14	28	TG	9	7
8191	1	14893786	14893813	2	14	28	AC	13	15
8197	1	14904919	14904932	2	7	14	TG	12	7
8198	1	14904935	14904952	2	9	18	TG	8	4
8205	1	14915321	14915356	3	12	36	TGA	13	6
8206	1	14921464	14921489	2	13	26	TG	11	9
8208	1	14922211	14922234	2	12	24	TG	17	11
8210	1	14923839	14923858	4	5	20	CAGC	24	5
8212	1	14924426	14924437	2	6	12	CA	33	3
8213	1	14925099	14925124	2	13	26	GT	9	8
8215	1	14929009	14929024	4	4	16	ATTT	19	4
8217	1	14932039	14932058	4	5	20	TCCC	12	5
8218	1	14932577	14932590	2	7	14	TG	22	4
8219	1	14932943	14932972	2	15	30	TG	6	8
8220	1	14942097	14942116	4	5	20	CAGG	26	4
8226	1	14960983	14960994	2	6	12	TG	29	3
8232	1	14966479	14966504	2	13	26	TG	19	8
8233	1	14968683	14968696	2	7	14	TG	24	6
8234	1	14972190	14972207	2	9	18	TG	17	4
8235	1	14973836	14973875	2	20	40	CA	7	8
8236	1	14976595	14976606	2	6	12	AC	23	6
8237	1	14976925	14976936	2	6	12	AC	31	3
8239	1	14984784	14984813	2	15	30	CA	15	7
8246	1	15000858	15000873	4	4	16	TTTG	30	3

8248	1	15002178	15002195	2	9	18	GT	25	3
8258	1	15010579	15010602	2	12	24	AG	9	8
8265	1	15017691	15017710	2	10	20	GT	18	6
8274	1	15046787	15046816	3	10	30	TCC	8	5
8280	1	15051235	15051258	4	6	24	GTTA	13	5
8288	1	15065659	15065670	2	6	12	AC	22	3
8293	1	15070597	15070635	3	13	39	ATC	13	7
8294	1	15078321	15078336	4	4	16	AACA	21	3
8300	1	15088738	15088755	3	6	18	AAC	24	3
8312	1	15103375	15103396	2	11	22	GA	15	6
8313	1	15105760	15105773	2	7	14	GA	26	3
8314	1	15105802	15105813	2	6	12	GT	23	4
8319	1	15114504	15114519	2	8	16	TG	25	8
8328	1	15124986	15124999	2	7	14	TG	20	6
8331	1	15127768	15127783	2	8	16	GT	26	9
8333	1	15131039	15131052	2	7	14	CA	22	4
8339	1	15133007	15133026	2	10	20	TG	18	14
8344	1	15142758	15142773	4	4	16	CTCG	23	3
8353	1	15150179	15150190	2	6	12	CA	12	4
8354	1	15150193	15150210	2	9	18	CA	17	8
8356	1	15150346	15150357	2	6	12	CA	19	8
8358	1	15151566	15151581	2	8	16	AC	21	7
8359	1	15151584	15151595	2	6	12	AC	21	6
8360	1	15151710	15151727	2	9	18	TG	23	3
8365	1	15160321	15160348	2	14	28	GT	6	5
8369	1	15163734	15163761	2	14	28	AG	17	10
8371	1	15164809	15164822	2	7	14	TG	20	9
8372	1	15165047	15165070	4	6	24	AAAG	15	6
8373	1	15166005	15166018	2	7	14	CA	29	4
8375	1	15172257	15172280	4	6	24	GTTT	30	6
8380	1	15182365	15182376	2	6	12	TG	21	3
8381	1	15183054	15183067	2	7	14	TC	24	3
8384	1	15189510	15189521	2	6	12	TG	17	9
8395	1	15207268	15207281	2	7	14	GT	18	5
8399	1	15209804	15209817	2	7	14	AG	15	3
8400	1	15212167	15212178	2	6	12	GT	14	4
8401	1	15212552	15212572	3	7	21	TTC	12	9
8402	1	15212925	15212942	2	9	18	GT	8	8
8403	1	15213893	15213930	2	19	38	GT	16	13
8404	1	15214431	15214448	2	9	18	TG	18	10
8410	1	15217551	15217574	2	12	24	AC	26	7
8412	1	15220254	15220277	2	12	24	TG	10	3
8426	1	15235623	15235644	2	11	22	AC	20	3
8427	1	15236576	15236590	3	5	15	AGC	34	3
8433	1	15243932	15243961	2	15	30	CA	6	6
8438	1	15259411	15259442	2	16	32	CA	15	6
8440	1	15261400	15261420	3	7	21	CTG	25	7
8446	1	15265555	15265582	2	14	28	AG	16	7
8459	1	15293388	15293405	2	9	18	AG	15	4
8465	1	15304559	15304570	2	6	12	GT	25	6
8466	1	15304757	15304771	3	5	15	CCA	7	4

8467	1	15305279	15305312	2	17	34	TC	9	9
8468	1	15306858	15306877	2	10	20	AC	16	5
8470	1	15310587	15310601	3	5	15	GCG	25	8
8473	1	15312096	15312113	2	9	18	GT	17	4
8478	1	15317340	15317357	2	9	18	CA	6	3
8479	1	15317438	15317457	2	10	20	CA	13	4
8486	1	15328735	15328750	2	8	16	CA	22	8
8491	1	15333972	15333985	2	7	14	CA	20	6
8492	1	15334955	15334974	2	10	20	AT	17	12
8499	1	15360229	15360252	2	12	24	GT	15	8
8503	1	15369054	15369075	2	11	22	AC	12	9
8508	1	15384896	15384931	4	9	36	ATAG	13	12
8509	1	15390659	15390692	2	17	34	CA	8	9
8514	1	15401315	15401330	2	8	16	AC	22	4
8516	1	15405919	15405936	3	6	18	ATT	28	3
8520	1	15425968	15425981	2	7	14	AG	36	3
8528	1	15455612	15455627	2	8	16	GT	25	3
8534	1	15481125	15481138	2	7	14	GA	28	5
8535	1	15485404	15485437	2	17	34	CA	14	9
8542	1	15502923	15502937	3	5	15	GCT	28	3
8546	1	15508749	15508760	2	6	12	GT	13	6
8547	1	15508888	15508913	2	13	26	AC	9	9
8548	1	15510508	15510528	3	7	21	TCC	16	3
8558	1	15514285	15514314	2	15	30	GT	12	8
8559	1	15514491	15514502	2	6	12	TG	15	3
8562	1	15515867	15515887	3	7	21	CAA	28	3
8563	1	15518629	15518646	2	9	18	GT	27	6
8565	1	15521407	15521422	2	8	16	AC	25	5
8574	1	15540115	15540170	2	28	56	GT	7	8
8576	1	15546329	15546343	3	5	15	AAT	13	8
8584	1	15593134	15593145	2	6	12	GT	20	3
8591	1	15607479	15607492	2	7	14	CA	21	3
8593	1	15610791	15610820	2	15	30	AC	6	7
8603	1	15624872	15624892	3	7	21	CAG	26	3
8605	1	15625123	15625138	2	8	16	AC	10	5
8606	1	15625518	15625549	2	16	32	AC	9	10
8609	1	15630346	15630357	2	6	12	GA	32	4
8614	1	15647791	15647810	2	10	20	TG	13	10
8617	1	15654617	15654636	4	5	20	ACAG	18	7
8619	1	15660343	15660388	2	23	46	TG	9	9
8620	1	15660536	15660555	2	10	20	AC	19	4
8630	1	15677457	15677480	2	12	24	GT	17	7
8635	1	15679631	15679645	3	5	15	TGA	25	4
8636	1	15679649	15679663	3	5	15	TGA	29	6
8637	1	15682211	15682230	2	10	20	GT	9	7
8641	1	15684896	15684913	2	9	18	AC	11	4
8649	1	15691545	15691560	2	8	16	CA	18	4
8665	1	15717643	15717660	2	9	18	TG	16	14
8666	1	15719360	15719375	2	8	16	AC	16	14
8669	1	15726013	15726034	2	11	22	TG	19	5
8670	1	15727297	15727312	2	8	16	AC	24	3

8671	1	15734763	15734774	2	6	12	CA	30	4
8677	1	15739639	15739656	3	6	18	GAG	21	6
8678	1	15740706	15740747	2	21	42	GT	19	9
8682	1	15757911	15757925	3	5	15	CAT	22	3
8689	1	15791084	15791113	2	15	30	CA	7	9
8693	1	15801799	15801813	3	5	15	TTG	26	3
8694	1	15802477	15802500	2	12	24	GT	10	6
8696	1	15806139	15806156	2	9	18	TG	20	4
8710	1	15818121	15818140	2	10	20	GA	20	4
8717	1	15826162	15826173	2	6	12	GT	20	5
8719	1	15827060	15827075	2	8	16	CA	14	3
8720	1	15827084	15827095	2	6	12	AC	20	3
8726	1	15836218	15836237	2	10	20	TG	7	3
8727	1	15836309	15836320	2	6	12	AG	24	3
8730	1	15839289	15839306	2	9	18	AC	22	3
8735	1	15846803	15846814	2	6	12	AG	26	3
8739	1	15848131	15848148	3	6	18	TGC	20	8
8740	1	15848325	15848336	2	6	12	AC	22	3
8741	1	15848772	15848789	3	6	18	CCT	12	6
8742	1	15848885	15848922	2	19	38	GT	12	10
8744	1	15851620	15851679	3	20	60	AGA	6	6
8747	1	15853418	15853429	2	6	12	GT	21	5
8748	1	15854515	15854532	2	9	18	AT	10	6
8751	1	15857033	15857044	2	6	12	CA	13	12
8754	1	15857456	15857467	2	6	12	GT	22	4
8755	1	15857474	15857487	2	7	14	GT	24	5
8757	1	15857541	15857552	2	6	12	AG	17	6
8758	1	15859153	15859170	2	9	18	GT	19	7
8759	1	15859173	15859186	2	7	14	GT	8	4
8767	1	15866040	15866055	2	8	16	CT	14	5
8768	1	15866139	15866152	2	7	14	CT	18	3
8774	1	15874372	15874385	2	7	14	TG	24	3
8776	1	15877443	15877468	2	13	26	AC	11	6
8779	1	15878435	15878460	2	13	26	GT	18	5
8783	1	15891282	15891301	2	10	20	AC	19	6
8784	1	15892610	15892625	2	8	16	AG	22	6
8791	1	15915025	15915056	2	16	32	GT	16	12
8796	1	15925121	15925134	2	7	14	GT	24	12
8800	1	15929011	15929038	2	14	28	AC	14	8
8803	1	15938007	15938026	4	5	20	ATTT	8	7
8805	1	15943376	15943411	2	18	36	AC	9	6
8810	1	15951210	15951229	2	10	20	AC	17	4
8812	1	15956184	15956195	2	6	12	GA	23	6
8814	1	15956785	15956810	2	13	26	GT	21	7
8815	1	15961724	15961751	2	14	28	CA	17	14
8820	1	15969633	15969646	2	7	14	AG	27	4
8825	1	15983905	15983919	3	5	15	AGC	28	4
8830	1	15993095	15993124	2	15	30	TG	10	9
8835	1	16006271	16006330	4	15	60	TCTA	15	8
8839	1	16017241	16017266	2	13	26	CA	13	7
8841	1	16022274	16022291	2	9	18	CA	9	4

8856	1	16061408	16061431	4	6	24	ATTT	14	6
8858	1	16065889	16065924	2	18	36	CA	15	11
8878	1	16091441	16091462	2	11	22	TG	9	4
8879	1	16095254	16095267	2	7	14	AG	22	4
8882	1	16097780	16097793	2	7	14	AC	24	12
8885	1	16100562	16100583	2	11	22	GT	12	6
8893	1	16109030	16109055	2	13	26	TG	10	9
8894	1	16109253	16109292	2	20	40	AC	14	8
8896	1	16109541	16109570	3	10	30	TCC	18	8
8900	1	16117379	16117398	2	10	20	CA	32	7
8901	1	16117557	16117580	2	12	24	GT	16	6
8904	1	16120240	16120257	3	6	18	GAG	29	6
8907	1	16121891	16121906	2	8	16	CT	29	9
8917	1	16126990	16127019	3	10	30	CCT	17	4
8921	1	16131182	16131213	4	8	32	ATCC	20	7
8925	1	16140693	16140707	3	5	15	CCT	26	3
8929	1	16157095	16157116	2	11	22	TC	28	6
8931	1	16160693	16160706	2	7	14	AG	21	3
8947	1	16207511	16207540	3	10	30	GAT	29	14
8949	1	16213055	16213072	3	6	18	ATA	19	4
8950	1	16213890	16213903	2	7	14	CA	24	5
8952	1	16214880	16214891	2	6	12	AC	20	3
8956	1	16220247	16220262	4	4	16	CTTG	25	3
8957	1	16221431	16221442	2	6	12	AT	16	11
8958	1	16224440	16224466	3	9	27	GAT	24	5
8960	1	16227516	16227531	2	8	16	TG	31	5
8968	1	16238802	16238849	4	12	48	AGAA	15	9
8971	1	16241410	16241425	2	8	16	AG	18	3
8973	1	16242563	16242577	3	5	15	AGA	19	17
8974	1	16242581	16242595	3	5	15	AGA	19	18
8975	1	16242982	16243001	4	5	20	GACA	22	4
8977	1	16243421	16243434	2	7	14	GT	21	5
8989	1	16255999	16256028	3	10	30	TGA	21	4
9000	1	16275178	16275197	2	10	20	TG	17	6
9002	1	16275885	16275900	2	8	16	AG	25	5
9005	1	16281487	16281502	2	8	16	GT	27	8
9006	1	16281712	16281737	2	13	26	GT	9	8
9011	1	16292159	16292170	2	6	12	GT	23	4
9012	1	16292719	16292746	2	14	28	TG	12	5
9013	1	16296216	16296229	2	7	14	GT	29	3
9014	1	16296381	16296400	2	10	20	TG	23	7
9015	1	16297422	16297443	2	11	22	TG	20	6
9016	1	16298830	16298855	2	13	26	CA	15	6
9018	1	16300281	16300294	2	7	14	CA	26	3
9021	1	16304623	16304646	2	12	24	CA	11	7
9024	1	16311523	16311536	2	7	14	AC	28	5
9026	1	16311771	16311792	2	11	22	TG	9	5
9030	1	16317485	16317506	2	11	22	GT	16	7
9031	1	16318023	16318050	2	14	28	TG	20	11
9032	1	16318206	16318231	2	13	26	CA	15	11
9033	1	16318372	16318395	3	8	24	TTC	16	5

9037	1	16326761	16326796	2	18	36	CA	8	11
9040	1	16337998	16338018	3	7	21	CAA	19	6
9048	1	16359376	16359389	2	7	14	TC	10	8
9068	1	16384791	16384808	2	9	18	CA	10	8
9070	1	16387388	16387403	2	8	16	GT	19	3
9071	1	16389245	16389256	2	6	12	GT	31	3
9074	1	16397980	16397995	2	8	16	AC	20	6
9077	1	16402267	16402278	2	6	12	AG	39	3
9078	1	16408850	16408864	3	5	15	GAT	21	3
9081	1	16415996	16416031	2	18	36	GT	8	7
9087	1	16420245	16420278	2	17	34	AG	19	9
9088	1	16420742	16420757	2	8	16	TG	24	6
9091	1	16425598	16425613	4	4	16	TTTA	10	4
9093	1	16438762	16438785	3	8	24	TTA	12	10
9110	1	16457272	16457287	2	8	16	AC	26	5
9111	1	16461647	16461670	2	12	24	GT	22	11
9117	1	16477408	16477421	2	7	14	AC	7	4
9118	1	16477534	16477551	2	9	18	CT	14	9
9123	1	16481345	16481356	2	6	12	GT	17	4
9124	1	16483894	16483913	2	10	20	TG	17	13
9130	1	16493482	16493511	2	15	30	GT	11	7
9131	1	16494649	16494676	4	7	28	TTCA	24	4
9134	1	16497995	16498009	3	5	15	TCT	19	3
9138	1	16503536	16503557	2	11	22	GT	22	7
9139	1	16503683	16503716	2	17	34	CA	9	9
9142	1	16508061	16508076	2	8	16	CA	20	15
9143	1	16508361	16508418	2	29	58	AC	14	11
9151	1	16518038	16518057	4	5	20	AGAC	29	3
9156	1	16527034	16527051	2	9	18	TA	10	3
9166	1	16547182	16547207	2	13	26	TG	8	9
9170	1	16549766	16549781	2	8	16	GT	10	6
9172	1	16553053	16553080	4	7	28	CATC	18	8
9173	1	16554860	16554885	2	13	26	GT	17	7
9175	1	16562877	16562903	3	9	27	TCA	23	4
9185	1	16580918	16580929	2	6	12	TA	23	3
9190	1	16589391	16589406	2	8	16	CA	12	6
9193	1	16589511	16589534	2	12	24	AC	16	5
9195	1	16592414	16592431	2	9	18	TG	22	7
9196	1	16592590	16592607	2	9	18	AC	17	13
9200	1	16597935	16597946	2	6	12	TC	25	3
9201	1	16601282	16601303	2	11	22	TC	16	8
9202	1	16602346	16602363	3	6	18	CTG	27	3
9208	1	16610171	16610188	2	9	18	GA	22	6
9211	1	16618519	16618540	2	11	22	TG	15	6
9213	1	16619422	16619436	3	5	15	CTT	16	5
9217	1	16623708	16623719	2	6	12	CT	21	3
9221	1	16625680	16625691	2	6	12	AG	26	5
9230	1	16629934	16629949	4	4	16	AACA	26	4
9234	1	16634779	16634804	2	13	26	AC	13	7
9238	1	16653008	16653025	2	9	18	TG	28	3
9240	1	16654650	16654667	2	9	18	CA	17	8

9243	1	16656698	16656709	2	6	12	AC	28	3
9248	1	16670120	16670141	2	11	22	GT	10	7
9252	1	16676291	16676308	2	9	18	AC	21	5
9256	1	16685846	16685869	2	12	24	TC	15	5
9263	1	16713232	16713249	2	9	18	CA	11	8
9282	1	16740052	16740072	3	7	21	GCA	23	5
9285	1	16742192	16742219	2	14	28	AC	18	7
9286	1	16742712	16742727	4	4	16	CTGA	27	3
9287	1	16746655	16746690	2	18	36	AC	19	8
9288	1	16748108	16748121	2	7	14	GT	21	4
9289	1	16748222	16748237	2	8	16	GT	14	11
9290	1	16748240	16748257	2	9	18	GT	14	10
9294	1	16755228	16755247	2	10	20	AG	27	4
9295	1	16757601	16757628	2	14	28	CA	9	10
9296	1	16760362	16760385	2	12	24	AC	15	7
9306	1	16770061	16770080	2	10	20	CA	16	9
9307	1	16770195	16770212	3	6	18	AGA	13	3
9318	1	16789876	16789889	2	7	14	CA	22	3
9319	1	16790105	16790120	4	4	16	ACAG	30	3
9323	1	16792367	16792378	2	6	12	TG	20	4
9324	1	16794718	16794733	4	4	16	AGGA	33	4
9325	1	16797053	16797082	2	15	30	TG	21	8
9329	1	16799986	16800011	2	13	26	CT	15	10
9332	1	16800199	16800222	2	12	24	TG	12	4
9338	1	16802776	16802791	2	8	16	AC	23	8
9339	1	16802877	16802890	2	7	14	TG	17	3
9340	1	16803016	16803043	2	14	28	TG	14	6
9341	1	16803126	16803141	2	8	16	AC	21	11
9345	1	16817398	16817409	2	6	12	TG	32	3
9346	1	16819481	16819492	2	6	12	CT	29	3
9347	1	16821477	16821488	2	6	12	AC	29	7
9349	1	16821891	16821918	2	14	28	CA	9	7
9352	1	16825486	16825501	2	8	16	CA	22	12
9355	1	16831066	16831077	2	6	12	AG	15	3
9359	1	16834733	16834744	2	6	12	TC	23	3
9360	1	16834842	16834861	2	10	20	CA	12	4
9362	1	16836484	16836497	2	7	14	TG	22	3
9365	1	16837225	16837244	2	10	20	CA	15	12
9368	1	16838559	16838580	2	11	22	AC	20	7
9369	1	16840549	16840564	2	8	16	TC	26	5
9370	1	16840715	16840726	2	6	12	TG	24	5
9376	1	16846913	16846936	2	12	24	AC	17	5
9385	1	16855984	16856016	3	11	33	ACT	26	7
9387	1	16856902	16856913	2	6	12	GT	15	9
9388	1	16856915	16856934	2	10	20	TG	11	9
9390	1	16857009	16857026	2	9	18	GA	24	4
9393	1	16859276	16859301	2	13	26	AC	19	8
9399	1	16860142	16860171	2	15	30	GT	12	5
9410	1	16866879	16866896	2	9	18	AG	18	3
9411	1	16867256	16867271	4	4	16	TTTC	20	3
9413	1	16868694	16868717	2	12	24	GA	9	5

9414	1	16868789	16868806	3	6	18	TGG	8	4
9417	1	16871787	16871802	4	4	16	CAGG	31	3
9419	1	16872328	16872345	2	9	18	GT	10	10
9422	1	16872758	16872775	2	9	18	AC	12	10
9423	1	16872778	16872789	2	6	12	AC	13	13
9425	1	16873969	16873980	2	6	12	GT	29	3
9426	1	16874150	16874189	2	20	40	AC	20	8
9430	1	16875915	16875930	2	8	16	TG	6	5
9436	1	16877575	16877590	2	8	16	AC	14	4
9438	1	16879188	16879201	2	7	14	TG	24	4
9439	1	16879285	16879330	2	23	46	GT	7	6
9440	1	16879434	16879457	2	12	24	CA	6	4
9441	1	16879498	16879531	2	17	34	CA	9	7
9444	1	16879849	16879864	2	8	16	TG	26	4
9451	1	16887441	16887478	2	19	38	CA	8	10
9452	1	16887593	16887614	2	11	22	AC	15	8
9453	1	16887788	16887815	2	14	28	CA	18	10
9457	1	16890522	16890551	2	15	30	GT	9	12
9459	1	16892462	16892473	2	6	12	AT	24	4
9462	1	16892860	16892889	3	10	30	AGG	9	3
9464	1	16893459	16893470	2	6	12	AC	24	3
9466	1	16893610	16893636	3	9	27	AGG	12	5
9468	1	16895821	16895856	2	18	36	GT	17	7
9476	1	16914711	16914728	2	9	18	TG	12	6
9481	1	16939351	16939382	4	8	32	TTAT	18	6
9505	1	16970929	16970948	4	5	20	GACA	27	3
9506	1	16976737	16976754	3	6	18	CCT	28	3
9518	1	16986607	16986624	2	9	18	CA	17	4
9521	1	16989244	16989257	2	7	14	TG	14	4
9522	1	16989969	16989984	4	4	16	AAAT	20	3
9529	1	17000804	17000819	2	8	16	GT	22	4
9533	1	17002345	17002364	2	10	20	GT	15	4
9546	1	17007845	17007862	2	9	18	GT	9	12
9548	1	17008550	17008583	2	17	34	GT	16	12
9556	1	17012844	17012855	2	6	12	CT	26	5
9558	1	17023785	17023798	2	7	14	TC	28	4
9559	1	17024027	17024042	4	4	16	TCAC	23	5
9564	1	17036841	17036888	2	24	48	CA	8	11
9569	1	17052531	17052580	2	25	50	AC	12	7
9573	1	17059335	17059356	2	11	22	AC	15	10
9574	1	17059998	17060018	3	7	21	CCT	28	5
9577	1	17061391	17061404	2	7	14	TG	9	7
9579	1	17062198	17062215	2	9	18	AT	7	8
9584	1	17067585	17067616	2	16	32	TC	9	8
9587	1	17069330	17069347	2	9	18	TA	11	7
9588	1	17069585	17069602	3	6	18	CCT	7	5
9597	1	17070919	17070932	2	7	14	CA	22	3
9606	1	17074470	17074481	2	6	12	TG	20	5
9607	1	17074484	17074499	2	8	16	TG	19	5
9610	1	17076079	17076098	2	10	20	CA	10	5
9611	1	17076142	17076159	3	6	18	AGC	9	4

9612	1	17077304	17077324	3	7	21	CCT	8	4
9614	1	17077530	17077561	2	16	32	AG	22	7
9615	1	17077926	17077941	4	4	16	CAGA	18	3
9621	1	17079167	17079182	2	8	16	CA	31	4
9641	1	17102137	17102164	2	14	28	AC	15	7
9643	1	17105452	17105469	3	6	18	TCA	23	4
9646	1	17108158	17108193	3	12	36	GAG	15	11
9648	1	17113145	17113176	2	16	32	CA	22	14
9675	1	17190735	17190768	2	17	34	TG	11	7
9678	1	17219879	17219902	2	12	24	GT	22	9
9680	1	17223080	17223101	2	11	22	TG	29	6
9682	1	17224310	17224333	2	12	24	GT	15	8
9688	1	17234747	17234768	2	11	22	TG	16	6
9692	1	17248951	17248974	2	12	24	CA	9	3
9693	1	17249673	17249688	2	8	16	TG	32	3
9696	1	17256519	17256556	2	19	38	AC	14	11
9698	1	17259551	17259566	2	8	16	GT	25	3
9699	1	17261464	17261475	2	6	12	GT	28	3
9701	1	17264676	17264691	4	4	16	TTCT	15	6
9707	1	17284999	17285016	2	9	18	AC	24	4
9709	1	17286936	17286951	2	8	16	CA	29	5
9710	1	17299188	17299227	2	20	40	TG	14	10
9714	1	17315581	17315600	2	10	20	AC	14	8
9715	1	17315756	17315773	2	9	18	CA	26	6
9716	1	17319373	17319400	2	14	28	TG	19	7
9726	1	17331736	17331763	4	7	28	TGCC	24	4
9728	1	17332861	17332872	2	6	12	GT	25	4
9733	1	17338493	17338506	2	7	14	GA	15	4
9734	1	17340022	17340043	2	11	22	TC	13	7
9736	1	17340104	17340117	2	7	14	GT	17	5
9739	1	17340436	17340451	2	8	16	GT	15	3
9741	1	17341620	17341635	2	8	16	TG	22	9
9767	1	17366249	17366276	2	14	28	TC	13	8
9773	1	17373407	17373426	2	10	20	TC	13	4
9774	1	17373427	17373438	2	6	12	AC	21	7
9775	1	17375379	17375392	2	7	14	TG	15	4
9779	1	17377507	17377538	2	16	32	CA	19	11
9780	1	17377619	17377632	2	7	14	AC	26	5
9785	1	17381996	17382021	2	13	26	GT	14	5
9786	1	17382162	17382173	2	6	12	CA	27	3
9787	1	17382517	17382534	2	9	18	AC	21	8
9788	1	17384233	17384244	2	6	12	CA	31	3
9790	1	17385073	17385094	2	11	22	TG	16	6
9792	1	17386488	17386499	2	6	12	TA	8	3
9796	1	17391165	17391188	4	6	24	CTGG	28	3
9797	1	17392735	17392755	3	7	21	TCT	10	4
9800	1	17397819	17397830	2	6	12	AC	19	3
9801	1	17397832	17397847	2	8	16	CA	22	5
9802	1	17398220	17398239	4	5	20	CTGC	19	6
9803	1	17400990	17401005	2	8	16	AC	20	4
9804	1	17402824	17402867	2	22	44	AC	11	12

9810	1	17409435	17409454	2	10	20	CA	23	10
9814	1	17418235	17418254	2	10	20	TG	22	4
9816	1	17425167	17425178	2	6	12	AG	27	3
9817	1	17428561	17428576	4	4	16	CGTG	28	3
9820	1	17431619	17431630	2	6	12	CA	27	3
9821	1	17432857	17432882	2	13	26	GT	18	7
9824	1	17436425	17436436	2	6	12	AG	11	3
9827	1	17438054	17438075	2	11	22	TG	27	5
9828	1	17438232	17438257	2	13	26	AC	19	9
9829	1	17438458	17438469	2	6	12	GT	16	7
9833	1	17443611	17443640	3	10	30	GAG	23	8
9836	1	17444094	17444119	2	13	26	AC	9	7
9838	1	17445271	17445292	2	11	22	AG	20	4
9839	1	17445387	17445404	2	9	18	GT	28	3
9840	1	17445517	17445546	2	15	30	GT	9	4
9843	1	17446480	17446499	2	10	20	GA	24	9
9844	1	17446500	17446519	2	10	20	GT	21	7
9847	1	17448780	17448793	2	7	14	CT	20	5
9848	1	17449481	17449494	2	7	14	AC	28	6
9849	1	17450357	17450390	2	17	34	TG	11	9
9850	1	17450909	17450924	2	8	16	GT	17	8
9853	1	17453580	17453607	2	14	28	TG	15	8
9854	1	17454248	17454269	2	11	22	GT	12	12
9855	1	17457666	17457679	2	7	14	CA	17	6
9862	1	17461475	17461506	2	16	32	GT	13	10
9863	1	17461711	17461724	2	7	14	TG	18	4
9864	1	17461765	17461796	2	16	32	GT	13	8
9865	1	17461969	17461984	2	8	16	CA	8	3
9870	1	17466124	17466139	4	4	16	CTGG	16	3
9876	1	17471109	17471144	2	18	36	TG	13	9
9886	1	17477228	17477239	2	6	12	TG	18	4
9892	1	17479045	17479062	2	9	18	CA	21	8
9895	1	17481891	17481918	2	14	28	GT	10	9
9899	1	17483457	17483472	4	4	16	AAAG	8	3
9900	1	17483572	17483593	2	11	22	GT	18	8
9905	1	17491470	17491493	2	12	24	AC	22	12
9907	1	17492063	17492078	4	4	16	TTCC	30	4
9908	1	17492198	17492227	2	15	30	CA	18	12
9910	1	17494354	17494369	2	8	16	AG	11	6
9912	1	17495179	17495204	2	13	26	AC	12	13
9914	1	17495357	17495374	2	9	18	AC	16	5
9918	1	17496682	17496699	2	9	18	TC	23	4
9920	1	17499399	17499426	2	14	28	GT	14	10
9925	1	17509030	17509055	2	13	26	AC	12	5
9927	1	17510414	17510445	2	16	32	CA	18	10
9931	1	17512729	17512748	2	10	20	CT	11	5
9932	1	17514545	17514572	2	14	28	CA	18	10
9934	1	17521222	17521235	2	7	14	TG	23	4
9936	1	17522269	17522284	2	8	16	GT	20	7
9938	1	17522417	17522446	2	15	30	TG	11	7
9939	1	17522576	17522595	2	10	20	TC	30	3

9940	1	17522930	17522949	2	10	20	TG	10	11
9941	1	17523117	17523162	2	23	46	TG	6	8
9942	1	17525679	17525700	2	11	22	TG	19	11
9946	1	17531014	17531027	2	7	14	CA	26	5
9947	1	17531145	17531172	2	14	28	CA	22	10
9949	1	17535269	17535296	2	14	28	CT	7	9
9952	1	17535787	17535808	2	11	22	GT	10	4
9957	1	17536223	17536236	2	7	14	GT	29	10
9958	1	17536798	17536829	2	16	32	GT	18	9
9959	1	17536970	17536987	2	9	18	TG	15	7
9968	1	17542932	17542943	2	6	12	TG	9	6
9971	1	17549214	17549229	2	8	16	CT	27	4
9976	1	17554869	17554884	2	8	16	CA	19	10
9979	1	17557544	17557555	2	6	12	TA	7	7
9982	1	17564470	17564493	2	12	24	TC	19	5
9988	1	17570177	17570194	3	6	18	CCA	26	4
9996	1	17575977	17575988	2	6	12	CT	23	6
9997	1	17575989	17576000	2	6	12	CA	15	3
9998	1	17576133	17576146	2	7	14	TG	18	12
10003	1	17577994	17578009	2	8	16	CT	22	3
10006	1	17581114	17581139	2	13	26	CA	14	8
10008	1	17581966	17581981	4	4	16	TTTG	23	4
10011	1	17591421	17591456	2	18	36	GT	7	10
10012	1	17594334	17594353	2	10	20	TG	10	7
10016	1	17609608	17609621	2	7	14	CT	14	3
10024	1	17632667	17632684	2	9	18	TG	16	9
10025	1	17632980	17632993	2	7	14	CT	23	3
10027	1	17633346	17633375	2	15	30	TG	18	12
10029	1	17635725	17635742	2	9	18	AC	24	3
10030	1	17635983	17636002	2	10	20	AC	23	5
10040	1	17644020	17644040	3	7	21	CCT	17	3
10041	1	17644306	17644333	2	14	28	AC	19	18
10046	1	17646006	17646029	2	12	24	TG	16	7
10058	1	17661024	17661059	2	18	36	TG	16	15
10064	1	17669439	17669462	2	12	24	CA	15	9
10072	1	17679428	17679449	2	11	22	GA	20	6
10073	1	17680135	17680154	4	5	20	TTAT	19	4
10078	1	17691261	17691288	2	14	28	TG	10	8
10080	1	17692772	17692795	3	8	24	AGC	23	10
10085	1	17700252	17700273	2	11	22	GT	17	6
10086	1	17700828	17700839	2	6	12	TG	29	6
10088	1	17712893	17712908	2	8	16	TG	24	6
10089	1	17714792	17714807	2	8	16	TG	27	6
10106	1	17746101	17746120	2	10	20	GT	13	11
10108	1	17748220	17748234	3	5	15	AAT	27	4
10112	1	17753479	17753500	2	11	22	GT	26	7
10113	1	17754866	17754885	2	10	20	TG	23	8
10116	1	17760397	17760420	4	6	24	GGAT	23	5
10121	1	17773250	17773271	2	11	22	CA	15	9
10126	1	17788786	17788821	2	18	36	TC	17	11
10148	1	17845416	17845430	3	5	15	GGT	25	4

10150	1	17846810	17846845	4	9	36	GAAG	15	9
10151	1	17849513	17849528	2	8	16	AC	21	4
10154	1	17851814	17851825	2	6	12	CA	25	3
10158	1	17859512	17859545	2	17	34	CA	17	11
10170	1	17868771	17868782	2	6	12	TA	9	5
10171	1	17869049	17869080	2	16	32	TG	8	9
10175	1	17887226	17887253	2	14	28	CA	11	10
10176	1	17897843	17897854	2	6	12	CT	19	4
10177	1	17898142	17898157	2	8	16	AC	21	6
10181	1	17902449	17902466	2	9	18	CA	14	3
10183	1	17902508	17902527	2	10	20	AC	14	11
10187	1	17902935	17902958	2	12	24	GA	6	6
10189	1	17904307	17904324	2	9	18	CA	22	3
10194	1	17907242	17907289	2	24	48	TG	8	12
10195	1	17907472	17907495	3	8	24	AGG	19	6
10198	1	17911520	17911531	2	6	12	CA	24	4
10199	1	17913549	17913584	3	12	36	GAT	19	12
10204	1	17919621	17919638	2	9	18	AC	22	9
10206	1	17922318	17922357	2	20	40	AC	9	13
10211	1	17938950	17938993	4	11	44	ATCA	18	6
10213	1	17942802	17942829	2	14	28	CT	11	10
10218	1	17953800	17953815	2	8	16	AC	15	3
10219	1	17955204	17955233	2	15	30	AC	6	6
10221	1	17957634	17957672	3	13	39	TCC	15	4
10223	1	17958164	17958175	2	6	12	CA	29	4
10227	1	17964701	17964716	2	8	16	TC	22	3
10229	1	17967071	17967088	2	9	18	TC	25	3
10233	1	17968863	17968876	2	7	14	AC	20	3
10234	1	17969041	17969072	2	16	32	TC	8	5
10236	1	17969400	17969417	2	9	18	TG	26	4
10237	1	17970283	17970312	2	15	30	CA	9	8
10238	1	17970461	17970496	2	18	36	AC	14	6
10239	1	17970605	17970622	2	9	18	GT	25	10
10240	1	17970882	17970903	2	11	22	AC	20	6
10243	1	17975799	17975816	2	9	18	TG	19	5
10244	1	17976871	17976902	2	16	32	TG	13	10
10251	1	17981635	17981656	2	11	22	CT	18	7
10253	1	17982091	17982110	2	10	20	CA	16	9
10254	1	17983673	17983696	2	12	24	CA	15	6
10269	1	17997543	17997554	2	6	12	TC	19	6
10271	1	17999851	17999882	4	8	32	TAGA	21	11
10272	1	18001349	18001366	2	9	18	GT	14	5
10273	1	18001624	18001644	3	7	21	GCA	21	4
10275	1	18002496	18002513	3	6	18	AAC	23	3
10277	1	18003383	18003397	3	5	15	ACA	19	4
10280	1	18012348	18012359	2	6	12	TG	18	3
10287	1	18026105	18026120	2	8	16	TC	10	3
10288	1	18028197	18028218	2	11	22	GT	15	8
10289	1	18031876	18031887	2	6	12	GA	21	4
10290	1	18032604	18032615	2	6	12	GT	17	3
10291	1	18033054	18033073	4	5	20	GTTG	16	4

10296	1	18041580	18041621	2	21	42	TG	14	11
10299	1	18049280	18049295	2	8	16	GT	29	3
10300	1	18053248	18053261	2	7	14	TC	19	4
10305	1	18068204	18068224	3	7	21	ATT	9	4
10306	1	18074122	18074133	2	6	12	TC	24	3
10320	1	18107170	18107185	2	8	16	AC	20	3
10322	1	18110613	18110632	2	10	20	AG	26	7
10328	1	18118807	18118826	2	10	20	GT	25	6
10332	1	18133678	18133695	2	9	18	CT	29	7
10333	1	18133780	18133793	2	7	14	TG	26	3
10334	1	18137561	18137596	2	18	36	TG	8	10
10337	1	18142907	18142918	2	6	12	CA	18	5
10338	1	18143083	18143106	3	8	24	GAG	9	4
10339	1	18143362	18143375	2	7	14	GA	15	3
10342	1	18145804	18145823	2	10	20	AC	19	8
10343	1	18145998	18146015	2	9	18	CT	17	7
10344	1	18146610	18146623	2	7	14	TG	17	4
10346	1	18151066	18151079	2	7	14	CA	19	13
10347	1	18151819	18151848	3	10	30	GAG	6	5
10355	1	18160401	18160418	3	6	18	ACA	21	11
10356	1	18163042	18163053	2	6	12	TG	19	3
10357	1	18163106	18163129	2	12	24	TG	6	7
10358	1	18163575	18163590	2	8	16	TC	22	5
10362	1	18169171	18169185	3	5	15	TCC	16	4
10368	1	18172290	18172313	3	8	24	GGA	24	6
10369	1	18174624	18174635	2	6	12	TG	21	6
10370	1	18174639	18174650	2	6	12	GT	18	6
10376	1	18180226	18180245	2	10	20	AC	26	8
10378	1	18183266	18183281	2	8	16	GT	20	6
10379	1	18184205	18184220	2	8	16	TG	22	7
10380	1	18184339	18184352	2	7	14	TG	17	3
10381	1	18184377	18184392	2	8	16	TG	23	4
10382	1	18185025	18185040	2	8	16	CA	22	4
10384	1	18187933	18187948	2	8	16	GT	20	4
10385	1	18189773	18189787	3	5	15	TTG	26	4
10390	1	18194604	18194641	2	19	38	AC	7	8
10391	1	18195033	18195044	2	6	12	GT	25	5
10392	1	18195229	18195246	2	9	18	CA	19	11
10394	1	18199538	18199553	2	8	16	AC	29	4
10395	1	18199922	18199935	2	7	14	GT	20	4
10401	1	18202746	18202769	2	12	24	TG	16	8
10404	1	18205421	18205438	2	9	18	AC	24	8
10405	1	18205578	18205609	2	16	32	AC	6	11
10406	1	18205795	18205808	2	7	14	GA	24	3
10412	1	18206960	18206975	2	8	16	CA	27	3
10414	1	18208972	18209016	3	15	45	TCA	21	14
10417	1	18211040	18211067	2	14	28	AC	21	14
10419	1	18213421	18213436	2	8	16	GT	23	11
10421	1	18213919	18213938	2	10	20	GT	29	6
10429	1	18226507	18226534	2	14	28	TG	13	9
10430	1	18228886	18228901	2	8	16	GT	26	6

10431	1	18229341	18229376	2	18	36	TG	18	15
10432	1	18230875	18230894	2	10	20	TG	21	6
10436	1	18235281	18235294	2	7	14	AG	31	7
10440	1	18236696	18236729	2	17	34	TC	8	11
10442	1	18238537	18238564	2	14	28	GT	17	10
10443	1	18240219	18240246	2	14	28	AC	24	7
10447	1	18243121	18243141	3	7	21	AAG	23	3
10449	1	18243698	18243724	3	9	27	AAG	6	4
10452	1	18244429	18244452	2	12	24	GA	17	11
10456	1	18244911	18244930	2	10	20	CT	20	7
10457	1	18245281	18245292	2	6	12	TC	19	6
10458	1	18245411	18245431	3	7	21	TTG	18	9
10459	1	18245483	18245494	2	6	12	GA	23	3
10461	1	18245634	18245657	2	12	24	GT	18	7
10464	1	18268001	18268016	2	8	16	AG	26	4
10470	1	18277776	18277789	2	7	14	GT	22	4
10472	1	18278273	18278316	2	22	44	CT	11	7
10475	1	18284189	18284228	2	20	40	AC	16	9
10477	1	18286626	18286639	2	7	14	CT	9	6
10478	1	18294569	18294580	2	6	12	AG	25	6
10480	1	18295651	18295686	2	18	36	CA	10	11
10481	1	18296138	18296169	2	16	32	GT	19	7
10482	1	18296327	18296338	2	6	12	CA	22	6
10483	1	18296340	18296357	2	9	18	AC	22	6
10484	1	18297597	18297636	2	20	40	TG	19	11
10490	1	18316003	18316014	2	6	12	AC	21	3
10495	1	18324803	18324836	2	17	34	TG	13	14
10496	1	18324961	18325002	2	21	42	GT	11	10
10503	1	18332011	18332044	2	17	34	CA	11	12
10504	1	18332260	18332293	2	17	34	CA	11	9
10506	1	18333076	18333087	2	6	12	TG	16	4
10509	1	18335692	18335711	2	10	20	AC	15	11
10511	1	18341222	18341237	4	4	16	TTTC	7	6
10513	1	18341347	18341370	3	8	24	TTG	24	8
10514	1	18341891	18341920	2	15	30	AC	16	11
10515	1	18342262	18342275	2	7	14	GA	23	4
10517	1	18343013	18343026	2	7	14	GT	23	5
10525	1	18344573	18344587	3	5	15	GAG	14	4
10526	1	18344591	18344605	3	5	15	GAG	17	3
10530	1	18345466	18345501	2	18	36	AC	15	9
10532	1	18345824	18345843	2	10	20	TC	27	10
10536	1	18347466	18347492	3	9	27	AGA	23	7
10540	1	18356980	18356999	2	10	20	AT	7	7
10542	1	18359166	18359186	3	7	21	AGA	25	9
10543	1	18363401	18363418	2	9	18	GA	11	8
10545	1	18363876	18363887	2	6	12	GT	13	10
10546	1	18363892	18363905	2	7	14	GT	16	10
10548	1	18365797	18365812	2	8	16	TG	16	7
10553	1	18370247	18370268	2	11	22	CA	13	8
10556	1	18373124	18373141	2	9	18	TC	23	4
10557	1	18373687	18373716	3	10	30	GGA	22	6

10559	1	18374262	18374277	2	8	16	CA	24	4
10560	1	18374509	18374522	2	7	14	TG	26	6
10562	1	18374767	18374780	2	7	14	CA	16	6
10564	1	18374972	18375011	2	20	40	CA	10	10
10567	1	18376341	18376356	2	8	16	TC	22	4
10569	1	18380132	18380165	2	17	34	TC	12	8
10572	1	18381167	18381186	2	10	20	AC	23	10
10576	1	18387702	18387723	2	11	22	TG	11	8
10577	1	18387864	18387903	2	20	40	TG	7	10
10578	1	18388560	18388581	2	11	22	GT	10	4
10579	1	18388757	18388768	2	6	12	TG	30	3
10580	1	18389173	18389186	2	7	14	TG	21	6
10582	1	18397259	18397276	2	9	18	TG	25	9
10583	1	18398900	18398929	2	15	30	TG	12	9
10585	1	18405296	18405307	2	6	12	TG	32	4
10587	1	18411204	18411225	2	11	22	AC	17	3
10589	1	18415222	18415247	2	13	26	AC	7	7
10590	1	18415367	18415382	2	8	16	AC	18	9
10594	1	18434806	18434841	2	18	36	GT	14	10
10597	1	18454394	18454405	2	6	12	AT	19	6
10600	1	18485277	18485290	2	7	14	TC	15	5
10608	1	18508916	18508937	2	11	22	GT	16	7
10609	1	18514027	18514042	2	8	16	TG	21	6
10611	1	18518472	18518485	2	7	14	AC	11	3
10615	1	18523069	18523080	2	6	12	AC	26	3
10616	1	18530335	18530349	3	5	15	TCT	27	6
10617	1	18530950	18530967	3	6	18	CCT	26	4
10618	1	18531397	18531444	2	24	48	AC	22	15
10622	1	18535268	18535291	2	12	24	GT	18	6
10623	1	18538553	18538580	2	14	28	CA	14	12
10624	1	18538657	18538668	2	6	12	CA	29	4
10625	1	18541757	18541768	2	6	12	CT	6	5
10630	1	18551347	18551372	2	13	26	GT	21	13
10634	1	18558567	18558592	2	13	26	AC	16	14
10637	1	18560523	18560548	2	13	26	AC	8	12
10639	1	18562890	18562904	3	5	15	TGG	22	6
10641	1	18564276	18564299	3	8	24	CTC	34	5
10642	1	18564506	18564529	2	12	24	GT	23	7
10649	1	18572192	18572221	2	15	30	GT	7	4
10650	1	18573832	18573855	4	6	24	AAAT	21	4
10651	1	18574765	18574784	2	10	20	CT	25	8
10654	1	18579501	18579516	2	8	16	CA	19	17
10655	1	18583166	18583177	2	6	12	CA	23	3
10657	1	18588711	18588736	2	13	26	CA	22	13
10661	1	18590805	18590836	2	16	32	AG	17	8
10664	1	18596723	18596744	2	11	22	TG	23	9
10665	1	18598826	18598837	2	6	12	GA	32	5
10669	1	18605800	18605811	2	6	12	TC	23	3
10670	1	18606722	18606742	3	7	21	GGA	27	9
10672	1	18607268	18607295	2	14	28	GT	21	12
10676	1	18618410	18618441	2	16	32	CA	17	9

10678	1	18621198	18621227	2	15	30	TG	11	9
10679	1	18621319	18621360	2	21	42	AC	6	8
10681	1	18621913	18621936	4	6	24	CAAA	22	5
10683	1	18625858	18625885	2	14	28	AC	15	9
10686	1	18628792	18628806	3	5	15	CCT	20	7
10688	1	18629332	18629363	2	16	32	CA	19	13
10699	1	18632065	18632088	3	8	24	CTC	23	7
10700	1	18635015	18635036	2	11	22	CA	23	8
10701	1	18636329	18636340	2	6	12	GA	25	3
10704	1	18638410	18638429	2	10	20	AC	8	10
10705	1	18638584	18638601	2	9	18	TG	26	7
10706	1	18638658	18638690	3	11	33	TCC	23	13
10707	1	18638846	18638863	2	9	18	AC	25	7
10709	1	18642154	18642181	2	14	28	AC	13	6
10713	1	18649654	18649665	2	6	12	AT	27	5
10714	1	18652340	18652354	3	5	15	GAG	9	9
10716	1	18653196	18653211	2	8	16	GT	21	9
10717	1	18653334	18653363	3	10	30	TTC	19	6
10722	1	18657904	18657936	3	11	33	GGA	14	9
10723	1	18658812	18658835	3	8	24	CAG	21	6
10724	1	18659046	18659077	2	16	32	CA	10	8
10727	1	18660129	18660148	2	10	20	CT	9	4
10728	1	18660576	18660597	2	11	22	CA	15	12
10729	1	18661003	18661016	2	7	14	TG	22	13
10733	1	18661554	18661577	3	8	24	GCA	24	6
10736	1	18662056	18662076	3	7	21	GCA	18	4
10738	1	18662876	18662895	2	10	20	TG	19	4
10743	1	18666850	18666867	2	9	18	CA	6	5
10745	1	18668019	18668042	3	8	24	AGG	20	11
10748	1	18669242	18669263	2	11	22	TC	22	7
10755	1	18673842	18673875	2	17	34	GT	7	8
10761	1	18676195	18676224	2	15	30	TG	15	10
10763	1	18677420	18677435	4	4	16	TTTA	18	8
10766	1	18678767	18678784	2	9	18	TA	9	4
10768	1	18679449	18679468	2	10	20	AC	13	7
10769	1	18679669	18679692	2	12	24	GT	17	5
10770	1	18679783	18679800	2	9	18	CA	19	9
10771	1	18679985	18680000	2	8	16	TG	7	3
10775	1	18681556	18681575	2	10	20	TG	17	4
10776	1	18682551	18682576	2	13	26	AC	14	15
10778	1	18683935	18683950	2	8	16	GA	23	10
10783	1	18687249	18687276	2	14	28	TG	11	15
10786	1	18702426	18702443	3	6	18	ATA	16	8
10787	1	18705425	18705438	2	7	14	AT	12	3
10792	1	18715801	18715834	2	17	34	TG	7	11
10794	1	18716867	18716888	2	11	22	GT	7	6
10796	1	18717909	18717929	3	7	21	TTG	13	5
10797	1	18718313	18718326	2	7	14	AC	24	5
10799	1	18722656	18722687	2	16	32	CA	7	8
10800	1	18727794	18727825	2	16	32	AC	21	15
10805	1	18746970	18746985	2	8	16	AC	9	4

10806	1	18747791	18747802	2	6	12	AT	20	3
10809	1	18753161	18753178	2	9	18	TG	21	3
10810	1	18754299	18754325	3	9	27	GCT	21	7
10811	1	18756718	18756743	2	13	26	CA	12	3
10812	1	18756914	18756933	2	10	20	AC	16	4
10818	1	18761112	18761141	2	15	30	AC	13	12
10823	1	18766370	18766401	2	16	32	TG	10	6
10824	1	18768405	18768420	2	8	16	GT	17	11
10825	1	18768554	18768583	2	15	30	CA	17	11
10827	1	18770353	18770367	3	5	15	TCC	23	5
10828	1	18770585	18770602	2	9	18	GT	20	5
10831	1	18779615	18779628	2	7	14	CA	26	7
10832	1	18779942	18779953	2	6	12	GT	24	6
10833	1	18780196	18780222	3	9	27	TCC	19	4
10839	1	18788296	18788311	2	8	16	GT	20	15
10841	1	18794034	18794053	2	10	20	CA	9	12
10846	1	18811896	18811913	2	9	18	AG	26	3
10850	1	18815658	18815677	4	5	20	AAAT	18	5
10855	1	18819742	18819757	2	8	16	CA	32	5
10856	1	18821269	18821284	2	8	16	GT	21	6
10857	1	18825558	18825573	2	8	16	CT	23	5
10858	1	18827717	18827742	2	13	26	GT	11	8
10859	1	18835993	18836012	2	10	20	GT	22	9
10865	1	18841680	18841693	2	7	14	GT	15	3
10867	1	18841883	18841898	2	8	16	CT	26	7
10868	1	18844289	18844321	3	11	33	TGT	20	7
10869	1	18846266	18846283	3	6	18	TCC	28	9
10873	1	18847650	18847675	2	13	26	CA	25	10
10875	1	18849079	18849110	2	16	32	CA	12	6
10883	1	18861117	18861134	2	9	18	CA	21	8
10884	1	18861197	18861212	2	8	16	TC	21	3
10885	1	18861409	18861434	2	13	26	TG	17	11
10887	1	18861567	18861582	2	8	16	AC	20	10
10889	1	18861799	18861818	2	10	20	AC	20	4
10892	1	18862552	18862567	2	8	16	AG	24	9
10895	1	18863967	18864002	3	12	36	GAG	13	7
10896	1	18864806	18864829	2	12	24	AC	18	12
10898	1	18869771	18869792	2	11	22	CA	22	13
10899	1	18869924	18869941	3	6	18	CTC	30	4
10905	1	18876017	18876050	2	17	34	CA	9	7
10907	1	18880602	18880621	4	5	20	AATG	26	5
10917	1	18890726	18890739	2	7	14	AG	13	3
10918	1	18890749	18890760	2	6	12	GA	11	3
10920	1	18891195	18891215	3	7	21	AAG	18	15
10922	1	18891697	18891720	3	8	24	GGA	18	7
10925	1	18892050	18892067	2	9	18	CT	13	12
10928	1	18892777	18892800	2	12	24	CA	14	6
10941	1	18917885	18917910	2	13	26	AC	17	13
10943	1	18918370	18918381	2	6	12	AC	19	6
10944	1	18926483	18926494	2	6	12	GA	27	4
10946	1	18932645	18932665	3	7	21	CCT	15	3

10949	1	18935432	18935449	2	9	18	GT	16	6
10951	1	18936689	18936702	2	7	14	TC	32	6
10952	1	18936894	18936907	2	7	14	CT	18	4
10956	1	18939121	18939146	2	13	26	AC	11	9
10958	1	18939318	18939341	2	12	24	TG	14	11
10959	1	18940511	18940524	2	7	14	AC	21	3
10961	1	18943351	18943370	2	10	20	TG	28	4
10963	1	18944838	18944859	2	11	22	GT	24	9
10964	1	18946370	18946383	2	7	14	GT	22	9
10966	1	18951597	18951620	2	12	24	GT	9	9
10967	1	18951726	18951771	2	23	46	AC	10	11
10970	1	18955124	18955147	2	12	24	AC	20	8
10974	1	18959507	18959536	2	15	30	TC	17	11
10977	1	18962280	18962309	3	10	30	GAG	13	8
10978	1	18962671	18962690	2	10	20	TG	13	9
10979	1	18965890	18965913	2	12	24	GT	18	13
10981	1	18966482	18966495	2	7	14	CT	15	5
10984	1	18969235	18969262	2	14	28	GT	6	8
10986	1	18969648	18969661	2	7	14	TG	20	4
10987	1	18969794	18969835	2	21	42	TG	6	8
10988	1	18969957	18969982	2	13	26	GT	14	10
10993	1	18976598	18976624	3	9	27	CCT	11	5
10994	1	18976812	18976845	2	17	34	CA	10	13
10995	1	18977683	18977697	3	5	15	TCT	23	7
10997	1	18980049	18980068	2	10	20	TG	13	10
10998	1	18981459	18981482	2	12	24	CA	16	14
11000	1	18983270	18983293	2	12	24	AC	10	9
11005	1	18996597	18996618	2	11	22	AC	23	10
11006	1	18996941	18996964	2	12	24	AC	15	10
11009	1	19002520	19002551	2	16	32	CT	13	8
11013	1	19004169	19004184	2	8	16	AC	17	4
11014	1	19004336	19004365	2	15	30	CA	14	9
11015	1	19006518	19006541	2	12	24	GT	21	10
11016	1	19006684	19006717	2	17	34	CA	19	13
11022	1	19008929	19008974	2	23	46	GT	12	12
11023	1	19009116	19009127	2	6	12	TG	16	10
11025	1	19009415	19009441	3	9	27	TGA	27	5
11027	1	19011457	19011483	3	9	27	GTT	24	10
11028	1	19013492	19013506	3	5	15	GAA	19	5
11031	1	19014054	19014068	3	5	15	ATA	20	5
11036	1	19017409	19017452	2	22	44	AC	12	7
11037	1	19017595	19017614	4	5	20	AAAC	25	3
11042	1	19019015	19019044	2	15	30	AC	9	8
11044	1	19019820	19019851	4	8	32	TTAT	16	10
11047	1	19020357	19020384	2	14	28	CA	12	7
11049	1	19020836	19020850	3	5	15	CAC	16	3
11050	1	19021269	19021292	2	12	24	TG	9	8
11053	1	19022969	19022996	2	14	28	GT	10	6
11061	1	19037154	19037179	2	13	26	AC	11	11
11063	1	19038812	19038841	2	15	30	TG	8	8
11068	1	19040708	19040740	3	11	33	GAG	13	5

11070	1	19041020	19041047	2	14	28	CA	11	5
11072	1	19041578	19041593	4	4	16	TTTG	14	4
11076	1	19053749	19053766	2	9	18	AC	23	12
11077	1	19061143	19061156	2	7	14	TC	23	7
11078	1	19061157	19061170	2	7	14	AC	20	6
11082	1	19063320	19063345	2	13	26	GT	14	11
11086	1	19075008	19075043	2	18	36	AC	8	7
11089	1	19078295	19078315	3	7	21	GAA	23	8
11091	1	19078821	19078840	2	10	20	TG	29	5
11094	1	19080531	19080544	2	7	14	TC	20	3
11105	1	19102714	19102745	2	16	32	CA	7	9
11108	1	19121304	19121327	3	8	24	GTT	26	4
11109	1	19122215	19122232	3	6	18	AGA	18	4
11131	1	19128882	19128893	2	6	12	TG	23	7
11132	1	19128969	19128986	2	9	18	AG	18	5
11133	1	19129091	19129108	3	6	18	TTA	23	6
11135	1	19132958	19132971	2	7	14	CT	22	5
11136	1	19133373	19133398	2	13	26	GT	20	8
11139	1	19135528	19135541	2	7	14	TG	22	4
11141	1	19136176	19136203	2	14	28	AC	12	11
11143	1	19138510	19138535	2	13	26	TG	16	11
11146	1	19139468	19139483	2	8	16	CA	25	3
11149	1	19142061	19142074	2	7	14	AC	18	7
11151	1	19142498	19142513	2	8	16	AG	28	3
11152	1	19143130	19143147	2	9	18	TG	20	6
11153	1	19144859	19144872	2	7	14	AC	27	6
11155	1	19150741	19150752	2	6	12	AC	24	4
11156	1	19151054	19151067	2	7	14	AC	31	4
11159	1	19168913	19168933	3	7	21	GAG	24	5
11162	1	19172080	19172097	3	6	18	ACA	26	7
11164	1	19174667	19174692	2	13	26	AC	20	8
11165	1	19174828	19174841	2	7	14	TC	32	3
11166	1	19177681	19177692	2	6	12	TG	26	4
11167	1	19177887	19177902	4	4	16	AAAT	24	5
11170	1	19191356	19191373	3	6	18	GCT	8	3
11171	1	19192298	19192313	2	8	16	CA	14	11
11179	1	19206853	19206874	2	11	22	CT	12	6
11183	1	19213085	19213102	2	9	18	AC	15	9
11184	1	19213212	19213225	2	7	14	TG	19	6
11189	1	19214128	19214143	4	4	16	CTTC	9	4
11192	1	19214987	19215002	4	4	16	TCCT	10	3
11201	1	19215614	19215633	4	5	20	TTCC	11	3
11206	1	19219173	19219208	2	18	36	CA	20	10
11208	1	19223770	19223787	3	6	18	ATC	22	5
11209	1	19224836	19224853	2	9	18	TA	8	3
11212	1	19227345	19227359	3	5	15	CTC	18	4
11213	1	19230550	19230573	4	6	24	TGCG	36	19
11215	1	19233072	19233099	4	7	28	AATG	9	3
11222	1	19252008	19252021	2	7	14	AG	31	4
11226	1	19261178	19261191	2	7	14	TG	28	4
11227	1	19261335	19261356	2	11	22	AC	15	8

11228	1	19262174	19262189	2	8	16	CA	28	10
11231	1	19273916	19273937	2	11	22	AC	16	10
11237	1	19287487	19287506	4	5	20	CCCT	7	4
11248	1	19311141	19311168	2	14	28	GT	19	13
11250	1	19312121	19312140	2	10	20	AG	6	3
11253	1	19313650	19313665	2	8	16	TG	15	13
11257	1	19319674	19319691	2	9	18	CA	16	10
11263	1	19331770	19331796	3	9	27	GAG	25	9
11264	1	19332232	19332247	2	8	16	CA	19	3
11268	1	19334410	19334424	3	5	15	TGT	15	5
11269	1	19334830	19334855	2	13	26	AT	17	10
11271	1	19337150	19337165	2	8	16	AG	15	3
11275	1	19340942	19340971	2	15	30	TC	9	5
11277	1	19341137	19341154	2	9	18	CA	13	4
11279	1	19341264	19341279	2	8	16	CT	24	6
11280	1	19341352	19341373	2	11	22	CA	19	10
11286	1	19346201	19346214	2	7	14	CA	27	3
11294	1	19352757	19352768	2	6	12	CT	17	5
11298	1	19357218	19357238	3	7	21	AGG	9	4
11301	1	19363026	19363039	2	7	14	AC	22	7
11302	1	19363041	19363056	2	8	16	CA	22	5
11303	1	19363702	19363715	2	7	14	TC	17	4
11306	1	19366335	19366355	3	7	21	AGA	27	6
11307	1	19367080	19367093	2	7	14	TC	13	4
11309	1	19369218	19369239	2	11	22	TG	21	15
11310	1	19369764	19369783	2	10	20	AC	29	8
11311	1	19371259	19371282	3	8	24	AAC	30	5
11312	1	19372693	19372707	3	5	15	CTG	31	3
11313	1	19373958	19373993	2	18	36	AC	12	8
11318	1	19380630	19380653	3	8	24	GGT	30	5
11323	1	19387209	19387229	3	7	21	CAT	20	5
11324	1	19389152	19389165	2	7	14	CA	14	3
11325	1	19390032	19390065	2	17	34	CA	13	9
11327	1	19393766	19393781	2	8	16	GT	24	4
11328	1	19395674	19395697	4	6	24	CAAA	18	6
11332	1	19399538	19399551	2	7	14	AC	24	5
11333	1	19400989	19401012	2	12	24	GT	19	7
11336	1	19403772	19403785	2	7	14	CA	24	6
11341	1	19407790	19407809	2	10	20	GA	17	6
11351	1	19415283	19415304	2	11	22	CA	11	12
11352	1	19415460	19415473	2	7	14	AC	24	5
11358	1	19422767	19422784	2	9	18	CA	16	11
11359	1	19422786	19422799	2	7	14	AC	19	12
11361	1	19424936	19424959	2	12	24	GT	23	8
11362	1	19425025	19425038	2	7	14	AC	23	3
11370	1	19429357	19429374	2	9	18	CA	14	5
11371	1	19430397	19430408	2	6	12	CA	21	5
11376	1	19435814	19435831	2	9	18	TC	24	6
11379	1	19439039	19439054	2	8	16	GT	8	3
11380	1	19439055	19439074	2	10	20	GA	13	8
11386	1	19439982	19440045	4	16	64	GATA	18	12

11389	1	19442293	19442306	2	7	14	CT	8	3
11391	1	19443655	19443688	2	17	34	CA	7	6
11392	1	19443821	19443836	2	8	16	CA	17	10
11397	1	19448333	19448360	2	14	28	CA	20	14
11399	1	19449516	19449529	2	7	14	TG	24	5
11401	1	19449728	19449755	4	7	28	ACAA	20	5
11406	1	19452795	19452812	2	9	18	AC	20	9
11419	1	19466887	19466949	3	21	63	GAA	15	14
11423	1	19473342	19473355	2	7	14	AT	23	4
11424	1	19473642	19473667	2	13	26	GT	10	4
11425	1	19477457	19477472	2	8	16	TG	19	3
11427	1	19482476	19482490	3	5	15	AGA	13	4
11428	1	19482983	19483002	2	10	20	CA	13	8
11431	1	19494912	19494941	2	15	30	TG	6	5
11434	1	19504580	19504603	3	8	24	TTG	23	10
11436	1	19504992	19505033	3	14	42	AGT	6	9
11437	1	19505991	19506004	2	7	14	GT	25	3
11438	1	19509421	19509432	2	6	12	CA	29	3
11439	1	19514393	19514406	2	7	14	CA	24	4
11447	1	19523732	19523746	3	5	15	ATT	25	3
11450	1	19525151	19525174	2	12	24	GT	14	14
11452	1	19527365	19527390	2	13	26	GT	13	12
11456	1	19531567	19531587	3	7	21	CAG	29	3
11457	1	19531679	19531690	2	6	12	GT	24	12
11458	1	19531699	19531710	2	6	12	GT	16	7
11462	1	19534095	19534116	2	11	22	GT	18	10
11463	1	19534229	19534246	2	9	18	GT	14	17
11465	1	19534445	19534462	3	6	18	TCT	20	3
11470	1	19541688	19541705	2	9	18	GT	11	6
11471	1	19541707	19541720	2	7	14	TG	10	7
11473	1	19542106	19542123	2	9	18	TG	9	7
11475	1	19542648	19542659	2	6	12	TG	21	5
11476	1	19542820	19542831	2	6	12	GT	27	4
11477	1	19542984	19543007	2	12	24	TG	16	11
11480	1	19546545	19546586	2	21	42	TG	9	8
11482	1	19546959	19547014	2	28	56	GT	8	12
11483	1	19548228	19548259	2	16	32	AC	11	13
11484	1	19548773	19548796	2	12	24	GT	12	10
11486	1	19549903	19549924	2	11	22	AG	20	8
11487	1	19550748	19550763	2	8	16	CA	9	9
11493	1	19552387	19552422	2	18	36	AC	14	8
11494	1	19556001	19556016	2	8	16	TG	13	4
11496	1	19559467	19559488	2	11	22	AC	15	11
11500	1	19562072	19562095	2	12	24	GA	6	6
11504	1	19562656	19562667	2	6	12	AC	17	4
11505	1	19564283	19564306	2	12	24	AC	21	10
11506	1	19565427	19565438	2	6	12	AC	27	4
11508	1	19566262	19566285	2	12	24	GT	10	7
11526	1	19588695	19588710	2	8	16	TG	25	5
11533	1	19603799	19603812	2	7	14	AC	30	3
11534	1	19603947	19603979	3	11	33	CAT	10	4

11544	1	19613259	19613284	2	13	26	CA	10	7
11545	1	19613420	19613445	2	13	26	TG	8	4
11546	1	19613510	19613523	2	7	14	CT	18	5
11547	1	19613561	19613578	2	9	18	CA	25	13
11550	1	19616921	19616946	2	13	26	GT	10	7
11552	1	19617738	19617763	2	13	26	GT	12	9
11554	1	19617947	19617978	2	16	32	CA	7	11
11555	1	19618746	19618787	2	21	42	GT	6	7
11557	1	19619134	19619157	2	12	24	AC	21	8
11558	1	19621086	19621121	2	18	36	AC	9	7
11559	1	19622263	19622277	3	5	15	AAT	22	8
11563	1	19624158	19624181	3	8	24	TTC	19	5
11565	1	19624926	19624947	2	11	22	CA	18	10
11566	1	19625620	19625665	2	23	46	TG	12	11
11567	1	19625809	19625836	2	14	28	TG	15	14
11570	1	19628061	19628080	2	10	20	GT	16	12
11573	1	19629358	19629369	2	6	12	CA	8	5
11574	1	19629478	19629495	2	9	18	CT	13	5
11576	1	19631226	19631239	2	7	14	GT	19	9
11577	1	19631398	19631411	2	7	14	CA	16	7
11582	1	19635473	19635492	2	10	20	GT	18	5
11583	1	19635693	19635714	2	11	22	GA	14	9
11592	1	19636770	19636799	2	15	30	CA	6	6
11593	1	19636936	19636955	2	10	20	GT	16	10
11595	1	19638119	19638130	2	6	12	GT	26	4
11596	1	19638227	19638238	2	6	12	GT	32	3
11598	1	19638394	19638407	2	7	14	CA	18	3
11602	1	19639189	19639214	2	13	26	CA	15	12
11603	1	19639585	19639600	4	4	16	GGTT	28	3
11604	1	19641246	19641265	2	10	20	GA	18	7
11608	1	19645546	19645571	2	13	26	TG	9	9
11611	1	19645763	19645788	2	13	26	TG	9	8
11612	1	19646851	19646886	2	18	36	CA	15	19
11614	1	19648208	19648227	2	10	20	AC	23	13
11615	1	19649684	19649717	2	17	34	GT	15	7
11617	1	19649993	19650012	2	10	20	GT	24	3
11619	1	19650313	19650328	2	8	16	TG	16	3
11620	1	19651011	19651036	2	13	26	TG	15	8
11622	1	19654534	19654553	2	10	20	GT	13	8
11623	1	19656148	19656173	2	13	26	CA	15	6
11624	1	19656319	19656346	2	14	28	TG	13	16
11625	1	19656442	19656455	2	7	14	GT	18	7
11626	1	19656534	19656573	2	20	40	CA	11	9
11627	1	19656717	19656738	2	11	22	TG	17	9
11628	1	19657463	19657474	2	6	12	AG	21	5
11631	1	19661101	19661144	2	22	44	AC	9	10
11632	1	19661238	19661249	2	6	12	TG	25	5
11634	1	19661515	19661530	2	8	16	CT	21	11
11635	1	19661680	19661695	2	8	16	GT	20	7
11636	1	19661762	19661793	2	16	32	TG	18	10
11638	1	19661930	19661943	2	7	14	CA	19	3

11639	1	19662235	19662268	2	17	34	GT	13	8
11640	1	19663783	19663796	2	7	14	TG	19	9
11641	1	19663802	19663817	2	8	16	GT	21	5
11642	1	19663952	19663975	2	12	24	CA	13	6
11643	1	19664262	19664279	2	9	18	AC	19	6
11647	1	19664633	19664656	2	12	24	AC	10	7
11648	1	19664695	19664712	2	9	18	CA	9	3
11651	1	19666814	19666855	2	21	42	CA	8	9
11654	1	19667674	19667701	2	14	28	AC	15	8
11655	1	19667926	19667939	2	7	14	TG	24	7
11656	1	19668042	19668057	2	8	16	AC	8	4
11657	1	19668158	19668169	2	6	12	CA	14	8
11658	1	19668174	19668189	2	8	16	CA	14	8
11659	1	19671065	19671076	2	6	12	CA	24	3
11661	1	19673319	19673356	2	19	38	AC	11	13
11667	1	19677763	19677790	2	14	28	CA	12	8
11669	1	19678878	19678895	2	9	18	TG	18	9
11671	1	19679056	19679069	2	7	14	CA	20	6
11672	1	19679072	19679085	2	7	14	CA	24	9
11673	1	19681460	19681481	2	11	22	AG	29	8
11676	1	19682491	19682534	2	22	44	CA	19	9
11677	1	19682905	19682916	2	6	12	CT	26	3
11684	1	19686395	19686420	2	13	26	GT	12	5
11686	1	19687113	19687130	2	9	18	AC	11	9
11690	1	19688012	19688045	2	17	34	GT	7	5
11693	1	19688460	19688479	2	10	20	CA	11	10
11696	1	19691058	19691073	2	8	16	TC	8	6
11698	1	19691312	19691327	2	8	16	AG	13	5
11699	1	19691650	19691671	2	11	22	TG	17	7
11702	1	19694407	19694440	2	17	34	AC	20	14
11703	1	19694537	19694560	2	12	24	GT	9	8
11704	1	19694810	19694855	2	23	46	GT	8	11
11705	1	19694971	19694990	2	10	20	CT	20	4
11706	1	19697024	19697055	2	16	32	CA	17	11
11707	1	19698907	19698920	2	7	14	CA	18	4
11708	1	19699122	19699151	2	15	30	GT	19	10
11709	1	19699370	19699385	2	8	16	CT	8	4
11710	1	19701128	19701143	2	8	16	TC	13	4
11711	1	19701381	19701396	2	8	16	CT	26	6
11715	1	19703835	19703862	2	14	28	CA	6	6
11719	1	19706737	19706768	2	16	32	TG	21	7
11721	1	19707012	19707045	2	17	34	GT	12	11
11724	1	19707560	19707605	2	23	46	AC	6	8
11725	1	19707685	19707706	2	11	22	AC	12	11
11726	1	19707801	19707822	2	11	22	CA	12	9
11729	1	19708006	19708049	2	22	44	TG	9	10
11730	1	19708698	19708715	2	9	18	AC	25	12
11731	1	19708718	19708733	2	8	16	AC	18	11
11732	1	19708853	19708884	2	16	32	GT	6	9
11737	1	19711199	19711216	2	9	18	CA	23	5
11740	1	19713777	19713804	2	14	28	CA	17	10

11741	1	19714196	19714221	2	13	26	GT	13	7
11747	1	19719619	19719645	3	9	27	GAT	23	6
11748	1	19721968	19721987	2	10	20	CT	16	6
11750	1	19722777	19722806	2	15	30	CA	8	8
11752	1	19723493	19723522	2	15	30	AC	8	10
11758	1	19728992	19729007	2	8	16	TG	24	4
11761	1	19732210	19732221	2	6	12	TG	26	3
11763	1	19732549	19732572	2	12	24	CT	22	8
11764	1	19733516	19733549	2	17	34	CA	15	11
11765	1	19733706	19733727	2	11	22	CA	17	11
11766	1	19737915	19737930	2	8	16	AC	19	6
11770	1	19748635	19748650	4	4	16	AAAG	17	4
11771	1	19749173	19749186	2	7	14	TG	22	3
11772	1	19749397	19749436	2	20	40	AC	10	12
11775	1	19753113	19753170	2	29	58	TG	6	7
11777	1	19754794	19754809	2	8	16	GT	17	7
11778	1	19754976	19754989	2	7	14	TG	6	3
11779	1	19755347	19755370	2	12	24	TG	18	12
11788	1	19763257	19763280	2	12	24	CA	23	13
11789	1	19763420	19763434	3	5	15	GTA	36	3
11790	1	19763644	19763657	2	7	14	CA	24	7
11791	1	19763763	19763786	2	12	24	AC	18	10
11792	1	19764372	19764392	3	7	21	TGG	21	5
11793	1	19764756	19764769	2	7	14	GT	26	3
11794	1	19767714	19767729	2	8	16	CA	16	12
11795	1	19771518	19771537	2	10	20	TG	23	4
11798	1	19777428	19777461	2	17	34	TG	10	10
11800	1	19780105	19780120	2	8	16	CA	29	5
11801	1	19780359	19780374	2	8	16	GA	24	6
11804	1	19788360	19788385	2	13	26	CA	22	13
11808	1	19792007	19792020	2	7	14	GT	15	7
11809	1	19792022	19792035	2	7	14	TG	19	7
11812	1	19797134	19797147	2	7	14	GA	24	3
11814	1	19798731	19798756	2	13	26	AC	14	12
11816	1	19799398	19799411	2	7	14	CA	27	6
11817	1	19799733	19799750	3	6	18	TAT	9	5
11819	1	19802409	19802422	2	7	14	CA	29	7
11821	1	19805941	19805956	2	8	16	GT	24	12
11822	1	19806257	19806278	2	11	22	GT	11	11
11823	1	19807134	19807163	2	15	30	AC	16	6
11826	1	19814154	19814177	3	8	24	TGT	24	8
11832	1	19840507	19840524	2	9	18	AG	28	13
11833	1	19846017	19846028	2	6	12	TG	27	3
11834	1	19847299	19847312	2	7	14	CA	29	5
11835	1	19848555	19848568	2	7	14	TG	18	7
11836	1	19848714	19848729	2	8	16	TG	20	12
11837	1	19850270	19850313	2	22	44	GT	6	9
11838	1	19850445	19850464	2	10	20	CT	7	3
11840	1	19851514	19851545	2	16	32	CA	7	5
11841	1	19852743	19852760	2	9	18	AG	33	4
11842	1	19853506	19853523	2	9	18	AC	19	7

11843	1	19855233	19855254	2	11	22	AC	15	13
11844	1	19857772	19857785	2	7	14	TG	30	5
11846	1	19858340	19858359	2	10	20	CA	21	8
11847	1	19858501	19858528	2	14	28	AC	15	7
11848	1	19862674	19862685	2	6	12	AC	27	3
11849	1	19863171	19863230	2	30	60	GT	6	8
11852	1	19865865	19865876	2	6	12	AC	12	3
11853	1	19866528	19866541	2	7	14	AC	22	3
11856	1	19872546	19872575	2	15	30	CA	9	8
11863	1	19875772	19875809	2	19	38	TC	18	14
11865	1	19877010	19877021	2	6	12	CT	22	3
11866	1	19879145	19879156	2	6	12	CA	25	4
11867	1	19879345	19879366	2	11	22	TG	19	7
11868	1	19881123	19881138	2	8	16	AC	19	8
11870	1	19882956	19882967	2	6	12	CA	25	4
11871	1	19883323	19883337	3	5	15	TGC	23	6
11874	1	19886445	19886464	2	10	20	GT	19	7
11875	1	19886610	19886621	2	6	12	AC	24	3
11876	1	19886864	19886875	2	6	12	AT	8	3
11881	1	19892502	19892519	3	6	18	GAG	28	3
11882	1	19893795	19893806	2	6	12	TG	27	5
11883	1	19896807	19896818	2	6	12	TG	21	3
11889	1	19901886	19901900	3	5	15	GAT	23	4
11893	1	19911184	19911213	2	15	30	CA	10	4
11895	1	19913379	19913396	2	9	18	AC	17	5
11896	1	19914608	19914627	2	10	20	CA	23	9
11897	1	19915699	19915728	2	15	30	TG	24	5
11900	1	19918355	19918386	2	16	32	AC	12	11
11901	1	19921446	19921457	2	6	12	GT	19	3
11902	1	19922593	19922610	2	9	18	AC	19	4
11903	1	19922748	19922761	2	7	14	GT	26	3
11904	1	19922805	19922822	2	9	18	AC	24	4
11905	1	19924138	19924153	2	8	16	AC	26	3
11906	1	19925241	19925272	2	16	32	CA	7	6
11907	1	19925395	19925418	2	12	24	AC	7	5
11908	1	19925634	19925657	2	12	24	CA	17	7
11909	1	19926002	19926013	2	6	12	AC	14	7
11910	1	19926015	19926030	2	8	16	CA	14	4
11913	1	19932390	19932411	2	11	22	TG	7	8
11914	1	19932544	19932579	2	18	36	TG	16	10
11915	1	19935869	19935888	2	10	20	AC	18	13
11920	1	19946935	19946949	3	5	15	CAG	27	3
11925	1	19960537	19960551	3	5	15	AAG	21	3
11926	1	19960569	19960583	3	5	15	GAA	22	5
11928	1	19960773	19960794	2	11	22	CA	17	7
11929	1	19960975	19961002	4	7	28	GTGA	18	8
11930	1	19961052	19961071	4	5	20	TGAG	15	3
11937	1	19992365	19992380	2	8	16	GT	20	4
11939	1	19995988	19996005	2	9	18	GT	7	7
11941	1	19997415	19997428	2	7	14	AC	23	4
11942	1	19999172	19999186	3	5	15	GGA	24	8

11943	1	20006960	20006973	2	7	14	TC	22	16
11944	1	20007284	20007295	2	6	12	CA	16	3
11948	1	20015674	20015688	3	5	15	CGG	26	4
11949	1	20019962	20019979	2	9	18	CA	17	6
11950	1	20025697	20025726	3	10	30	ATC	22	6
11953	1	20036044	20036077	2	17	34	GT	12	9
11954	1	20037678	20037701	3	8	24	CAG	29	10
11959	1	20045034	20045053	4	5	20	AAAC	21	5
11962	1	20047625	20047652	2	14	28	TG	7	8
11966	1	20055341	20055356	2	8	16	CT	16	8
11967	1	20055730	20055744	3	5	15	TTG	25	3
11970	1	20058192	20058203	2	6	12	CT	27	3
11972	1	20064358	20064371	2	7	14	GT	22	3
11973	1	20068411	20068422	2	6	12	GA	11	8
11974	1	20068424	20068441	2	9	18	AG	10	8
11977	1	20069509	20069530	2	11	22	CA	12	7
11986	1	20081922	20081943	2	11	22	CT	25	3
11988	1	20083542	20083557	4	4	16	GAAG	24	3
11991	1	20085192	20085217	2	13	26	TG	13	10
11999	1	20105824	20105837	2	7	14	TC	15	5
12000	1	20105840	20105853	2	7	14	TC	17	6
12002	1	20109319	20109346	2	14	28	TG	19	8
12009	1	20124507	20124532	2	13	26	GT	18	8
12010	1	20127287	20127300	2	7	14	GT	23	3
12012	1	20129217	20129232	2	8	16	CA	29	8
12014	1	20132378	20132421	2	22	44	TG	14	9
12018	1	20136718	20136729	2	6	12	TG	25	5
12019	1	20136732	20136743	2	6	12	TG	31	4
12021	1	20146078	20146105	4	7	28	TGTA	14	3
12024	1	20149397	20149436	2	20	40	GT	7	7
12025	1	20149866	20149889	2	12	24	TG	17	11
12028	1	20154097	20154114	3	6	18	TCC	27	4
12029	1	20160086	20160099	2	7	14	GA	31	3
12030	1	20163327	20163340	2	7	14	CT	32	3
12036	1	20180878	20180917	2	20	40	AC	17	10
12053	1	20203676	20203687	2	6	12	AG	26	6
12054	1	20210473	20210494	2	11	22	TA	14	5
12057	1	20219686	20219700	3	5	15	GAG	21	3
12063	1	20226836	20226857	2	11	22	GT	17	7
12064	1	20227467	20227488	2	11	22	TG	13	9
12065	1	20227699	20227722	3	8	24	CTG	25	10
12066	1	20228252	20228266	3	5	15	AAT	16	4
12074	1	20228808	20228827	4	5	20	TGTT	18	12
12079	1	20233597	20233620	2	12	24	TG	6	8
12081	1	20235442	20235463	2	11	22	GT	16	8
12082	1	20235654	20235671	2	9	18	AC	18	3
12083	1	20235883	20235916	2	17	34	TG	13	14
12085	1	20243643	20243680	2	19	38	CA	12	12
12089	1	20252183	20252194	2	6	12	CA	24	4
12090	1	20255299	20255316	2	9	18	GA	23	10
12091	1	20255738	20255781	2	22	44	TG	11	7

12092	1	20256332	20256349	2	9	18	CT	24	6
12094	1	20261149	20261175	3	9	27	ATC	19	19
12095	1	20262252	20262269	2	9	18	CA	26	6
12096	1	20263424	20263439	2	8	16	CA	24	9
12098	1	20264774	20264793	2	10	20	AC	13	14
12100	1	20269113	20269142	2	15	30	CA	17	13
12102	1	20269964	20270001	2	19	38	AC	9	11
12103	1	20271367	20271382	4	4	16	GACA	22	4
12105	1	20272463	20272510	2	24	48	GT	12	13
12106	1	20275434	20275449	2	8	16	AC	16	7
12107	1	20275598	20275617	2	10	20	TC	23	3
12111	1	20276123	20276138	2	8	16	TC	19	5
12112	1	20276139	20276150	2	6	12	AC	21	5
12113	1	20277471	20277486	2	8	16	CA	29	8
12116	1	20278170	20278199	2	15	30	CA	9	13
12117	1	20278515	20278532	2	9	18	AC	22	5
12118	1	20278811	20278824	2	7	14	AG	16	3
12119	1	20278859	20278882	3	8	24	CCT	11	7
12123	1	20279389	20279404	2	8	16	AC	22	4
12124	1	20281220	20281247	2	14	28	AC	7	6
12133	1	20283922	20283939	2	9	18	AC	17	4
12136	1	20284147	20284166	4	5	20	GGAT	23	10
12137	1	20285926	20285945	2	10	20	AC	19	11
12140	1	20290161	20290182	2	11	22	CA	15	9
12142	1	20291814	20291837	2	12	24	AC	10	10
12143	1	20295772	20295785	2	7	14	AC	14	6
12144	1	20295910	20295931	2	11	22	CA	16	8
12146	1	20296082	20296095	2	7	14	TG	12	6
12147	1	20300156	20300179	2	12	24	CA	16	5
12148	1	20300356	20300387	4	8	32	CCTT	7	3
12149	1	20300553	20300568	2	8	16	AC	17	10
12151	1	20300671	20300690	2	10	20	GT	17	8
12153	1	20300800	20300817	2	9	18	TG	8	11
12157	1	20303155	20303168	2	7	14	AC	16	5
12158	1	20304038	20304055	2	9	18	GT	22	19
12161	1	20308951	20308972	2	11	22	CA	13	9
12162	1	20309942	20309957	2	8	16	GT	14	7
12163	1	20312655	20312674	2	10	20	CA	17	7
12167	1	20327784	20327804	3	7	21	ATC	18	9
12169	1	20334494	20334521	2	14	28	GT	14	5
12173	1	20346922	20346936	3	5	15	ACA	26	8
12174	1	20348015	20348035	3	7	21	CAG	27	3
12175	1	20348262	20348277	2	8	16	GA	19	3
12176	1	20348482	20348511	2	15	30	CA	9	9
12177	1	20348543	20348568	2	13	26	AC	14	12
12179	1	20360959	20360976	2	9	18	GT	17	13
12181	1	20363939	20363959	3	7	21	ATC	26	7
12183	1	20368181	20368198	2	9	18	TG	24	12
12185	1	20380755	20380774	2	10	20	TG	12	4
12188	1	20388517	20388532	2	8	16	TC	22	11
12200	1	20417265	20417294	2	15	30	GT	6	9

12201	1	20417954	20417969	2	8	16	CT	20	3
12202	1	20421812	20421829	3	6	18	TGC	26	7
12204	1	20425213	20425232	2	10	20	GT	22	4
12214	1	20447868	20447895	2	14	28	AC	7	8
12219	1	20454040	20454063	2	12	24	GT	18	10
12220	1	20454360	20454383	3	8	24	TGA	12	5
12222	1	20456281	20456314	2	17	34	TG	14	13
12223	1	20456411	20456424	2	7	14	TC	29	3
12224	1	20457992	20458025	2	17	34	GT	10	11
12225	1	20458086	20458097	2	6	12	TC	23	3
12227	1	20462207	20462224	2	9	18	AC	19	7
12229	1	20467670	20467685	2	8	16	GT	24	8
12235	1	20479134	20479151	2	9	18	CA	19	8
12240	1	20485150	20485164	3	5	15	ATT	11	7
12241	1	20486027	20486044	3	6	18	TGA	24	7
12245	1	20496671	20496682	2	6	12	GT	16	10
12250	1	20501120	20501134	3	5	15	AGC	25	4
12252	1	20503243	20503254	2	6	12	TC	28	4
12253	1	20504232	20504251	2	10	20	GA	21	11
12254	1	20504557	20504588	2	16	32	GT	14	10
12255	1	20512455	20512468	2	7	14	GA	31	3
12258	1	20514398	20514421	3	8	24	CTC	15	7
12259	1	20514649	20514672	3	8	24	AGA	24	3
12276	1	20542297	20542312	2	8	16	AG	20	3
12279	1	20548900	20548932	3	11	33	CTC	22	6
12281	1	20551929	20551961	3	11	33	TGT	28	8
12284	1	20553170	20553209	2	20	40	TG	6	9
12286	1	20554017	20554052	3	12	36	CCT	8	5
12293	1	20574238	20574255	2	9	18	CA	10	5
12295	1	20580350	20580376	3	9	27	AAC	30	11
12297	1	20602121	20602148	2	14	28	CA	21	15
12300	1	20606021	20606036	4	4	16	GGAA	24	5
12303	1	20610231	20610250	2	10	20	AT	12	3
12305	1	20616910	20616923	2	7	14	AG	25	5
12306	1	20616927	20616938	2	6	12	GT	21	4
12307	1	20623283	20623312	2	15	30	AC	11	9
12309	1	20626015	20626042	2	14	28	TG	7	9
12310	1	20626274	20626317	2	22	44	TC	9	12
12312	1	20634250	20634264	3	5	15	TCC	29	3
12314	1	20638778	20638805	2	14	28	CA	13	16
12316	1	20644386	20644412	3	9	27	CCT	11	8
12318	1	20655115	20655146	2	16	32	GT	15	8
12321	1	20661167	20661180	2	7	14	AT	16	4
12327	1	20669477	20669504	2	14	28	GT	16	14
12332	1	20680202	20680222	3	7	21	AGA	27	5
12333	1	20681062	20681083	2	11	22	AG	24	8
12334	1	20681230	20681257	2	14	28	GT	15	10
12335	1	20683710	20683751	3	14	42	TGA	20	11
12336	1	20685127	20685141	3	5	15	GGT	22	3
12338	1	20689687	20689706	2	10	20	TC	6	7
12340	1	20696869	20696884	2	8	16	TA	15	12

12346	1	20702164	20702178	3	5	15	AAC	25	3
12348	1	20706453	20706472	2	10	20	AT	11	7
12352	1	20709465	20709480	2	8	16	TG	15	6
12354	1	20712984	20713010	3	9	27	GAG	25	4
12357	1	20713843	20713857	3	5	15	GCA	28	3
12358	1	20714275	20714292	3	6	18	GAG	12	3
12359	1	20718340	20718357	2	9	18	TC	16	7
12361	1	20726555	20726581	3	9	27	GGA	18	6
12362	1	20726723	20726740	2	9	18	GT	19	13
12366	1	20728797	20728811	3	5	15	TCT	21	5
12370	1	20742174	20742191	2	9	18	TC	25	5
12372	1	20743833	20743860	2	14	28	AC	12	6
12373	1	20743970	20743993	2	12	24	AC	14	11
12374	1	20744671	20744698	2	14	28	GT	12	10
12386	1	20767259	20767290	4	8	32	GATG	28	12
12388	1	20768154	20768174	3	7	21	CCT	9	7
12389	1	20770523	20770537	3	5	15	TCC	20	5
12391	1	20773170	20773197	2	14	28	GT	17	9
12392	1	20775433	20775452	4	5	20	TCTT	22	4
12393	1	20776193	20776230	2	19	38	GT	18	11
12396	1	20779913	20779930	3	6	18	TCT	15	10
12403	1	20787645	20787680	2	18	36	AC	7	9
12404	1	20790820	20790833	2	7	14	AG	24	4
12405	1	20791537	20791552	2	8	16	TG	23	11
12409	1	20806986	20807009	3	8	24	TTC	17	7
12411	1	20822011	20822026	4	4	16	CAGT	29	3
12412	1	20824384	20824398	3	5	15	AGC	26	6
12415	1	20834270	20834283	2	7	14	CT	26	4
12416	1	20834535	20834552	2	9	18	TG	8	7
12417	1	20835029	20835046	3	6	18	CCT	19	6
12418	1	20835891	20835908	3	6	18	GGA	23	5
12422	1	20839632	20839658	3	9	27	AAG	20	10
12431	1	20848112	20848127	4	4	16	AAAG	31	5
12436	1	20854025	20854056	4	8	32	AAAG	17	14
12440	1	20856661	20856681	3	7	21	CCT	21	8
12441	1	20856873	20856890	3	6	18	TCC	20	4
12442	1	20857094	20857126	3	11	33	CTC	19	13
12448	1	20863363	20863380	2	9	18	AC	15	8
12450	1	20863667	20863700	2	17	34	TG	13	9
12451	1	20866177	20866214	2	19	38	AC	13	14
12453	1	20870401	20870414	2	7	14	CA	29	11
12455	1	20872244	20872273	2	15	30	GT	14	10
12460	1	20887012	20887037	2	13	26	AC	13	6
12461	1	20888454	20888481	2	14	28	AC	14	7
12462	1	20888589	20888602	2	7	14	CA	17	8
12463	1	20889004	20889051	4	12	48	CATC	17	14
12467	1	20895115	20895128	2	7	14	CA	13	6
12481	1	20914996	20915007	2	6	12	AC	17	4
12483	1	20921544	20921559	4	4	16	AAAC	14	11
12484	1	20922126	20922143	2	9	18	GT	26	8
12485	1	20922983	20922997	3	5	15	CCT	26	7

12486	1	20923527	20923547	3	7	21	TAA	9	9
12492	1	20939018	20939032	3	5	15	TTA	25	4
12493	1	20944192	20944203	2	6	12	TG	16	7
12506	1	20961277	20961300	2	12	24	CA	17	7
12507	1	20961420	20961441	2	11	22	CA	17	15
12509	1	20962200	20962217	2	9	18	GA	24	5
12510	1	20964142	20964153	2	6	12	TA	22	3
12511	1	20966272	20966287	2	8	16	GA	10	3
12512	1	20966629	20966642	2	7	14	TC	20	5
12518	1	20972855	20972874	2	10	20	CA	12	10
12523	1	20976065	20976085	3	7	21	AGG	19	5
12524	1	20977228	20977247	4	5	20	AAGA	18	5
12527	1	20983204	20983219	2	8	16	GT	16	4
12530	1	20989205	20989232	2	14	28	AC	20	11
12531	1	20989835	20989862	2	14	28	CA	12	6
12534	1	20992526	20992549	3	8	24	AAG	23	7
12535	1	20994002	20994023	2	11	22	TG	10	12
12537	1	20995896	20995921	2	13	26	TG	20	5
12538	1	20996081	20996110	2	15	30	AC	13	16
12541	1	20998522	20998539	3	6	18	CTC	23	7
12542	1	20998736	20998750	3	5	15	GAG	22	8
12551	1	21007890	21007925	2	18	36	AC	11	7
12552	1	21008037	21008074	2	19	38	GT	16	10
12553	1	21008507	21008528	2	11	22	AC	12	10
12554	1	21011057	21011080	2	12	24	AC	10	10
12555	1	21011232	21011253	2	11	22	CA	7	7
12557	1	21011406	21011421	2	8	16	AC	7	5
12558	1	21011424	21011439	2	8	16	AC	11	9
12559	1	21011535	21011549	3	5	15	AGC	21	3
12562	1	21015040	21015060	3	7	21	GCA	21	6
12563	1	21015755	21015790	2	18	36	GT	9	9
12568	1	21019150	21019173	2	12	24	TC	15	5
12569	1	21019464	21019479	4	4	16	TCAG	34	7
12571	1	21022979	21023002	2	12	24	AC	13	11
12574	1	21023493	21023512	2	10	20	CA	12	6
12575	1	21023595	21023626	2	16	32	CA	8	6
12579	1	21026339	21026358	4	5	20	CAGG	36	10
12584	1	21028135	21028158	2	12	24	GT	19	10
12598	1	21038991	21039032	2	21	42	AG	13	8
12600	1	21040001	21040020	4	5	20	AAAT	28	3
12603	1	21040869	21040886	3	6	18	CAG	23	3
12604	1	21042159	21042170	2	6	12	TG	26	5
12606	1	21046237	21046252	2	8	16	GT	15	8
12607	1	21046378	21046409	2	16	32	TG	20	11
12610	1	21049355	21049384	2	15	30	CA	22	6
12611	1	21053337	21053370	2	17	34	GT	9	10
12613	1	21053561	21053572	2	6	12	TG	19	5
12614	1	21053842	21053861	2	10	20	AC	19	19
12615	1	21055903	21055923	3	7	21	GAG	21	5
12616	1	21056094	21056109	2	8	16	GA	24	3
12618	1	21058349	21058368	2	10	20	AC	18	4

12619	1	21059191	21059246	2	28	56	GT	12	12
12620	1	21060833	21060860	2	14	28	TG	17	9
12621	1	21061028	21061059	4	8	32	CGGA	25	15
12622	1	21061144	21061165	2	11	22	AC	14	11
12623	1	21061222	21061233	2	6	12	AC	20	5
12628	1	21063728	21063753	2	13	26	CA	7	9
12629	1	21063915	21063960	2	23	46	CA	7	12
12637	1	21087120	21087133	2	7	14	AG	29	3
12639	1	21088052	21088073	2	11	22	CA	16	8
12643	1	21092003	21092030	2	14	28	CA	20	13
12644	1	21092297	21092318	2	11	22	CT	23	5
12645	1	21092622	21092633	2	6	12	AT	10	7
12646	1	21093222	21093237	2	8	16	TA	10	4
12651	1	21108480	21108495	4	4	16	AATA	28	3
12652	1	21108722	21108741	2	10	20	AC	22	6
12655	1	21114350	21114377	2	14	28	CA	16	16
12656	1	21114647	21114678	2	16	32	GT	11	11
12657	1	21114830	21114841	2	6	12	GT	12	10
12658	1	21114844	21114861	2	9	18	GT	15	10
12661	1	21117830	21117853	2	12	24	CA	11	11
12662	1	21120503	21120546	2	22	44	GT	14	8
12665	1	21123759	21123782	2	12	24	CA	21	7
12666	1	21123962	21123987	2	13	26	CA	13	9
12667	1	21124353	21124380	2	14	28	CA	11	10
12670	1	21132280	21132295	2	8	16	GT	15	7
12673	1	21140774	21140799	2	13	26	CA	11	9
12676	1	21141452	21141481	2	15	30	AC	11	9
12677	1	21141890	21141907	2	9	18	TG	17	5
12678	1	21141937	21141968	2	16	32	TG	19	10
12681	1	21142685	21142706	2	11	22	AC	18	11
12683	1	21143226	21143257	2	16	32	AC	11	7
12686	1	21149349	21149364	2	8	16	TG	20	18
12689	1	21151136	21151150	3	5	15	TGA	24	5
12690	1	21152730	21152753	3	8	24	GAG	15	5
12693	1	21156725	21156740	4	4	16	TTTA	12	3
12696	1	21157829	21157856	2	14	28	GT	8	12
12698	1	21158042	21158065	2	12	24	AC	15	8
12700	1	21158206	21158245	2	20	40	CA	9	10
12710	1	21173213	21173224	2	6	12	CA	25	3
12711	1	21174811	21174826	2	8	16	AC	16	4
12713	1	21180293	21180324	2	16	32	GT	13	13
12716	1	21188700	21188711	2	6	12	GT	19	6
12718	1	21191239	21191252	2	7	14	CA	27	3
12719	1	21191695	21191718	3	8	24	GCA	23	16
12725	1	21204420	21204434	3	5	15	AAC	27	3
12727	1	21207039	21207053	3	5	15	GCT	24	5
12728	1	21209377	21209416	2	20	40	AC	6	10
12729	1	21210116	21210130	3	5	15	AGC	28	4
12734	1	21218266	21218293	2	14	28	GT	22	6
12735	1	21218406	21218437	2	16	32	GT	12	16
12738	1	21219044	21219055	2	6	12	AT	27	4

12741	1	21221893	21221932	2	20	40	AC	7	9
12745	1	21222267	21222298	2	16	32	CA	13	12
12746	1	21222398	21222437	2	20	40	CA	12	15
12749	1	21228650	21228669	4	5	20	TATT	19	10
12758	1	21248655	21248680	2	13	26	TG	15	8
12760	1	21250288	21250299	2	6	12	CA	12	6
12764	1	21256836	21256867	2	16	32	TG	11	9
12769	1	21265955	21265972	2	9	18	GA	15	4
12770	1	21266478	21266523	2	23	46	TG	9	12
12771	1	21267050	21267077	2	14	28	TG	8	8
12772	1	21269198	21269209	2	6	12	CT	16	4
12773	1	21270939	21270958	2	10	20	CA	20	8
12780	1	21284816	21284833	2	9	18	GT	18	9
12785	1	21290427	21290448	2	11	22	GT	18	6
12787	1	21297046	21297059	2	7	14	GT	17	3
12788	1	21297242	21297253	2	6	12	AC	28	8
12791	1	21302687	21302702	2	8	16	GT	14	7
12794	1	21304257	21304278	2	11	22	TG	21	5
12795	1	21304512	21304527	2	8	16	AC	23	4
12796	1	21306421	21306440	2	10	20	TG	18	9
12797	1	21306734	21306745	2	6	12	GT	10	4
12798	1	21307298	21307309	2	6	12	AC	27	3
12805	1	21317435	21317464	2	15	30	CA	11	12
12807	1	21317673	21317700	4	7	28	AAAG	21	7
12810	1	21319710	21319733	2	12	24	AC	23	10
12812	1	21325776	21325799	3	8	24	TCA	30	4
12813	1	21326674	21326701	2	14	28	TG	10	6
12815	1	21331712	21331739	2	14	28	AC	11	9
12817	1	21338808	21338819	2	6	12	TA	27	3
12818	1	21342142	21342187	2	23	46	AC	9	9
12821	1	21345950	21345965	2	8	16	GA	26	3
12827	1	21355973	21355986	2	7	14	AC	26	4
12828	1	21356388	21356425	2	19	38	AC	17	11
12829	1	21356629	21356664	2	18	36	GT	13	12
12831	1	21358469	21358490	2	11	22	CA	24	10
12832	1	21358611	21358650	2	20	40	AC	7	8
12834	1	21359955	21359966	2	6	12	AC	25	3
12835	1	21363896	21363907	2	6	12	TG	22	3
12841	1	21386271	21386292	2	11	22	CA	13	16
12843	1	21393643	21393656	2	7	14	CT	33	3
12844	1	21398099	21398132	2	17	34	CA	11	12
12847	1	21400491	21400518	2	14	28	CT	11	12
12853	1	21420123	21420156	2	17	34	GT	16	9
12860	1	21424150	21424161	2	6	12	TC	16	8
12861	1	21426552	21426589	2	19	38	CA	10	11
12865	1	21429706	21429721	2	8	16	TG	23	8
12866	1	21431664	21431689	2	13	26	TG	13	13
12868	1	21433741	21433755	3	5	15	TGT	26	4
12871	1	21436647	21436658	2	6	12	TA	19	6
12873	1	21439087	21439110	2	12	24	TG	24	8
12874	1	21439223	21439240	2	9	18	TG	24	12

12875	1	21439243	21439256	2	7	14	TG	11	9
12876	1	21444165	21444182	2	9	18	AC	10	10
12879	1	21446678	21446705	2	14	28	TG	8	10
12880	1	21448000	21448025	2	13	26	CA	11	10
12885	1	21468861	21468882	2	11	22	CA	22	8
12887	1	21471078	21471125	3	16	48	AGA	10	14
12894	1	21477359	21477380	2	11	22	GT	16	13
12898	1	21481190	21481233	2	22	44	CA	11	16
12900	1	21482129	21482144	4	4	16	GTGA	30	4
12903	1	21485363	21485388	2	13	26	CA	17	9
12904	1	21486211	21486238	2	14	28	CA	16	17
12906	1	21486359	21486373	3	5	15	GGA	23	5
12909	1	21494079	21494098	2	10	20	TG	15	9
12910	1	21495601	21495616	4	4	16	CCTC	26	7
12916	1	21497617	21497637	3	7	21	AGC	16	3
12918	1	21500223	21500254	2	16	32	GT	18	8
12921	1	21505272	21505289	3	6	18	GAG	29	4
12923	1	21511600	21511631	2	16	32	AC	13	7
12927	1	21515875	21515902	2	14	28	TG	10	10
12931	1	21522664	21522687	2	12	24	GT	11	9
12936	1	21527775	21527795	3	7	21	CCT	13	7
12938	1	21529728	21529769	2	21	42	AC	8	11
12940	1	21530323	21530346	2	12	24	AC	18	9
12941	1	21532932	21532961	2	15	30	TG	20	7
12942	1	21534418	21534439	2	11	22	TG	12	6
12949	1	21545346	21545363	3	6	18	CCT	11	9
12955	1	21559028	21559055	2	14	28	AC	10	10
12956	1	21559104	21559115	2	6	12	TG	21	3
12968	1	21589864	21589875	2	6	12	GT	12	7
12970	1	21592607	21592618	2	6	12	AC	26	4
12971	1	21592923	21592948	2	13	26	AC	15	10
12972	1	21593140	21593155	2	8	16	CA	16	3
12973	1	21593503	21593514	2	6	12	CA	19	6
12974	1	21593842	21593883	2	21	42	TG	19	11
12975	1	21595433	21595444	2	6	12	GT	28	3
12977	1	21595855	21595880	2	13	26	GT	11	9
12981	1	21609266	21609291	2	13	26	AC	23	7
12982	1	21609534	21609553	4	5	20	CTGT	12	10
12983	1	21609555	21609574	4	5	20	TCTT	17	11
12984	1	21609714	21609729	4	4	16	TTTC	10	3
12985	1	21609790	21609817	4	7	28	TTCT	15	8
12986	1	21610249	21610284	2	18	36	AG	18	14
12988	1	21612893	21612908	4	4	16	TCTT	31	3
12990	1	21615866	21615881	4	4	16	ATAC	31	4
13000	1	21625522	21625567	2	23	46	GT	6	9
13012	1	21642118	21642139	2	11	22	CA	19	10
13015	1	21650485	21650498	2	7	14	GT	23	5
13016	1	21650505	21650516	2	6	12	GT	14	3
13017	1	21650663	21650682	2	10	20	TG	6	9
13021	1	21658226	21658245	2	10	20	TG	26	6
13022	1	21658285	21658304	2	10	20	TG	23	10

13025	1	21663989	21664008	2	10	20	GT	16	9
13026	1	21664010	21664029	2	10	20	TG	13	8
13027	1	21664145	21664170	2	13	26	TG	10	16
13028	1	21665117	21665134	3	6	18	TGT	30	5
13032	1	21670229	21670252	2	12	24	GT	10	5
13034	1	21670938	21670949	2	6	12	CA	28	3
13050	1	21694876	21694889	2	7	14	AC	21	4
13053	1	21699318	21699341	4	6	24	ATAA	25	4
13056	1	21704026	21704049	3	8	24	GGA	9	5
13057	1	21705079	21705096	3	6	18	GAG	15	10
13060	1	21706766	21706797	2	16	32	TG	7	9
13064	1	21712856	21712875	2	10	20	TG	13	12
13065	1	21715681	21715714	2	17	34	TG	13	9
13067	1	21716360	21716389	3	10	30	CTG	33	10
13070	1	21720110	21720127	2	9	18	GA	14	4
13071	1	21723384	21723405	2	11	22	TG	16	6
13073	1	21724605	21724619	3	5	15	TGA	27	5
13074	1	21724935	21724952	2	9	18	GT	21	8
13082	1	21736083	21736114	2	16	32	TG	24	12
13083	1	21739009	21739026	2	9	18	TG	15	8
13084	1	21739196	21739209	2	7	14	CA	27	10
13089	1	21751495	21751516	2	11	22	CT	19	11
13092	1	21754532	21754547	2	8	16	AC	9	9
13093	1	21754550	21754561	2	6	12	AC	19	14
13094	1	21754973	21755002	2	15	30	GT	11	12
13104	1	21763825	21763840	2	8	16	GA	15	4
13106	1	21764838	21764855	2	9	18	CA	14	4
13119	1	21785240	21785259	2	10	20	AC	19	12
13122	1	21785372	21785385	2	7	14	TG	16	8
13124	1	21786584	21786601	2	9	18	AC	19	8
13125	1	21786794	21786832	3	13	39	CTT	25	13
13128	1	21789840	21789855	2	8	16	TC	17	7
13133	1	21793697	21793712	2	8	16	GT	24	10
13139	1	21800307	21800340	2	17	34	TG	7	7
13141	1	21805492	21805506	3	5	15	GGT	22	4
13142	1	21807970	21807987	2	9	18	TG	28	5
13143	1	21808714	21808733	4	5	20	TCCA	22	4
13145	1	21814002	21814022	3	7	21	CCT	18	13
13146	1	21814207	21814264	2	29	58	GT	12	10
13148	1	21817955	21817970	2	8	16	TG	11	14
13149	1	21817972	21817987	2	8	16	GT	8	11
13152	1	21822133	21822172	2	20	40	TG	12	13
13153	1	21822308	21822319	2	6	12	AC	22	3
13156	1	21823719	21823752	2	17	34	CA	6	7
13157	1	21823882	21823923	2	21	42	CA	7	12
13159	1	21825375	21825404	2	15	30	TG	9	10
13163	1	21829828	21829857	2	15	30	CA	20	9
13168	1	21833724	21833753	2	15	30	CA	14	8
13171	1	21834324	21834355	2	16	32	AC	10	7
13173	1	21838213	21838262	2	25	50	GT	20	18
13179	1	21841861	21841898	2	19	38	GT	9	9

13180	1	21845700	21845723	3	8	24	TGT	28	12
13184	1	21854358	21854397	2	20	40	GT	8	7
13185	1	21854585	21854598	2	7	14	GA	21	3
13186	1	21854645	21854662	2	9	18	GA	20	9
13190	1	21856719	21856742	2	12	24	GT	15	11
13191	1	21856911	21856936	2	13	26	TG	8	7
13193	1	21857301	21857330	2	15	30	AC	18	12
13203	1	21861497	21861516	2	10	20	AG	9	5
13205	1	21862571	21862590	2	10	20	CA	17	10
13209	1	21865066	21865077	2	6	12	CA	32	3
13211	1	21868066	21868099	2	17	34	GT	9	10
13212	1	21869067	21869096	2	15	30	TG	10	9
13219	1	21875647	21875658	2	6	12	TG	20	5
13224	1	21884176	21884189	2	7	14	AC	22	12
13227	1	21890542	21890559	2	9	18	TA	11	5
13228	1	21891257	21891276	2	10	20	GT	16	8
13234	1	21903090	21903101	2	6	12	CA	21	12
13236	1	21906383	21906412	2	15	30	TG	8	8
13238	1	21910173	21910186	2	7	14	AG	26	3
13242	1	21911931	21911951	3	7	21	AGA	22	4
13243	1	21913415	21913426	2	6	12	AC	30	4
13246	1	21921419	21921430	2	6	12	CA	26	4
13247	1	21922907	21922921	3	5	15	TGC	14	4
13248	1	21924637	21924650	2	7	14	TG	25	6
13252	1	21928785	21928812	2	14	28	CA	16	11
13263	1	21940701	21940732	2	16	32	CA	17	10
13265	1	21940971	21941008	2	19	38	GT	14	10
13275	1	21948090	21948101	2	6	12	CA	16	6
13276	1	21948109	21948120	2	6	12	AC	14	5
13277	1	21948240	21948269	2	15	30	AC	13	10
13280	1	21953229	21953246	2	9	18	AC	23	6
13284	1	21955742	21955769	2	14	28	GT	17	11
13286	1	21956571	21956586	2	8	16	CT	11	6
13287	1	21956606	21956643	2	19	38	AC	12	12
13288	1	21956689	21956702	2	7	14	AC	17	3
13290	1	21957399	21957424	2	13	26	GA	13	6
13291	1	21957489	21957526	2	19	38	CA	10	10
13294	1	21959752	21959777	2	13	26	GT	13	9
13295	1	21959886	21959945	2	30	60	GT	10	12
13296	1	21961640	21961655	2	8	16	AC	20	4
13298	1	21963728	21963757	2	15	30	GT	20	10
13301	1	21965224	21965238	3	5	15	TCC	26	3
13302	1	21965242	21965259	3	6	18	TCC	25	4
13305	1	21967832	21967849	3	6	18	CCT	10	4
13306	1	21967904	21967915	2	6	12	CA	17	5
13311	1	21977324	21977369	2	23	46	CA	13	11
13313	1	21979489	21979508	4	5	20	GAGG	12	5
13315	1	21981853	21981876	2	12	24	TG	24	9
13316	1	21981940	21981951	2	6	12	GT	23	5
13317	1	21982044	21982059	4	4	16	CAGA	15	5
13319	1	21982513	21982538	2	13	26	AC	18	12

13322	1	21983515	21983546	2	16	32	GT	7	10
13324	1	21983647	21983664	3	6	18	TAT	17	5
13325	1	21983794	21983807	2	7	14	GA	18	5
13326	1	21986002	21986019	3	6	18	TCC	11	11
13330	1	21987454	21987471	2	9	18	TG	11	9
13332	1	21987801	21987826	2	13	26	CT	18	12
13333	1	21989838	21989852	3	5	15	CCT	17	7
13334	1	21989915	21989938	2	12	24	CA	8	6
13337	1	21991501	21991516	4	4	16	GACA	31	5
13338	1	21995026	21995047	2	11	22	TG	26	3
13339	1	21996321	21996340	2	10	20	AT	8	9
13343	1	21999018	21999033	4	4	16	AAAT	27	3
13345	1	22000966	22000979	2	7	14	CA	19	8
13346	1	22000981	22000994	2	7	14	AC	20	9
13355	1	22008454	22008467	2	7	14	CA	24	5
13357	1	22008780	22008794	3	5	15	CGG	20	6
13360	1	22010408	22010422	3	5	15	TCC	28	10
13363	1	22011445	22011468	3	8	24	GAG	13	4
13364	1	22013179	22013236	2	29	58	CA	7	9
13365	1	22014725	22014740	2	8	16	AG	23	7
13366	1	22015976	22015999	3	8	24	AAG	6	5
13367	1	22016168	22016203	2	18	36	GT	8	11
13369	1	22016331	22016354	2	12	24	GT	9	10
13370	1	22016396	22016423	2	14	28	TG	11	8
13371	1	22016512	22016549	2	19	38	TG	12	13
13372	1	22016633	22016648	2	8	16	GA	24	6
13373	1	22018001	22018018	3	6	18	GCT	22	3
13374	1	22018212	22018225	2	7	14	AG	9	5
13379	1	22020625	22020666	2	21	42	GT	11	11
13391	1	22031105	22031119	3	5	15	CTT	32	3
13397	1	22031880	22031893	2	7	14	GT	14	9
13398	1	22032701	22032726	2	13	26	GT	13	6
13400	1	22033720	22033755	2	18	36	GT	7	9
13409	1	22038404	22038415	2	6	12	AC	16	6
13413	1	22040317	22040349	3	11	33	AGG	14	8
13418	1	22041617	22041652	2	18	36	GT	9	10
13424	1	22056635	22056676	2	21	42	CA	7	10
13425	1	22056815	22056829	3	5	15	GAA	27	3
13431	1	22059498	22059511	2	7	14	AC	23	4
13438	1	22062045	22062059	3	5	15	AGG	8	4
13440	1	22062702	22062717	2	8	16	TG	12	5
13441	1	22062936	22062950	3	5	15	TCC	12	6
13442	1	22062997	22063014	3	6	18	CCT	14	6
13449	1	22064619	22064648	2	15	30	TG	16	6
13457	1	22066533	22066547	3	5	15	AGG	13	3
13458	1	22066580	22066611	2	16	32	AG	7	5
13459	1	22068310	22068343	2	17	34	CA	13	15
13461	1	22068495	22068506	2	6	12	TG	19	3
13466	1	22069274	22069293	2	10	20	AC	9	11
13469	1	22071593	22071612	2	10	20	CT	27	10
13470	1	22072906	22072921	2	8	16	AC	19	8

13475	1	22075874	22075909	2	18	36	GT	13	15
13476	1	22076018	22076047	2	15	30	GT	9	12
13482	1	22090011	22090028	2	9	18	TC	10	4
13484	1	22091464	22091491	2	14	28	CA	11	8
13485	1	22093401	22093424	2	12	24	AC	18	11
13486	1	22094783	22094804	2	11	22	TG	21	13
13488	1	22096404	22096424	3	7	21	CCT	25	11
13489	1	22096599	22096642	2	22	44	AC	18	18
13490	1	22100421	22100442	2	11	22	CT	8	4
13491	1	22101721	22101738	2	9	18	AC	22	6
13493	1	22106168	22106207	2	20	40	CA	9	12
13496	1	22108933	22108954	2	11	22	TG	9	4
13497	1	22109070	22109081	2	6	12	AG	19	3
13498	1	22109195	22109215	3	7	21	GGA	10	5
13504	1	22113982	22114015	2	17	34	TG	18	15
13505	1	22115487	22115500	2	7	14	GA	32	7
13506	1	22116741	22116778	2	19	38	GT	22	12
13509	1	22117770	22117785	4	4	16	ATCG	31	3
13510	1	22119079	22119099	3	7	21	TAA	24	9
13514	1	22121601	22121616	2	8	16	GA	20	8
13515	1	22121770	22121784	3	5	15	GGT	21	3
13517	1	22122778	22122795	2	9	18	GT	8	11
13536	1	22134476	22134489	2	7	14	AG	29	4
13537	1	22139386	22139403	2	9	18	TG	7	8
13545	1	22143451	22143468	2	9	18	AC	18	5
13546	1	22146670	22146685	4	4	16	AAGG	8	5
13550	1	22148053	22148070	3	6	18	GCC	17	10
13553	1	22148510	22148524	3	5	15	GCT	27	4
13554	1	22150240	22150257	3	6	18	ATG	29	5
13557	1	22150953	22150968	2	8	16	TC	28	3
13562	1	22152971	22152991	3	7	21	TCA	21	14
13564	1	22156004	22156021	3	6	18	AAG	22	5
13565	1	22157421	22157456	2	18	36	TG	7	7
13566	1	22157557	22157584	2	14	28	GT	17	18
13568	1	22159856	22159869	2	7	14	CA	24	5
13571	1	22164489	22164515	3	9	27	TCC	21	6
13576	1	22171574	22171593	4	5	20	GAAA	30	4
13580	1	22173636	22173673	2	19	38	CA	14	11
13581	1	22173931	22173966	3	12	36	CTC	15	7
13592	1	22188533	22188554	2	11	22	CT	10	7
13594	1	22192900	22192935	2	18	36	TG	13	12
13596	1	22196004	22196029	2	13	26	CA	12	10
13600	1	22198655	22198684	2	15	30	CA	7	8
13603	1	22202386	22202413	2	14	28	TG	17	14
13607	1	22207738	22207758	3	7	21	ATG	16	3
13608	1	22212067	22212092	2	13	26	CA	15	11
13612	1	22217096	22217119	3	8	24	GAG	17	11
13614	1	22217542	22217591	2	25	50	CA	10	10
13618	1	22220500	22220531	2	16	32	CA	8	8
13630	1	22225960	22225999	2	20	40	TG	15	10
13631	1	22233559	22233570	2	6	12	CT	22	7

13632	1	22233811	22233846	2	18	36	GT	9	9
13636	1	22243804	22243821	2	9	18	CA	14	9
13642	1	22253473	22253496	3	8	24	CTT	22	6
13651	1	22264422	22264453	2	16	32	AC	18	10
13660	1	22275782	22275807	2	13	26	CA	17	13
13663	1	22277318	22277335	2	9	18	GT	22	11
13664	1	22278483	22278502	2	10	20	GT	11	8
13666	1	22285721	22285744	3	8	24	TTG	22	6
13667	1	22286206	22286229	3	8	24	GCT	34	7
13669	1	22292885	22292914	3	10	30	AGA	15	8
13673	1	22298381	22298420	2	20	40	AC	17	9
13685	1	22367270	22367285	4	4	16	TAGA	21	13
13686	1	22369021	22369047	3	9	27	ATA	14	5
13691	1	22374221	22374268	2	24	48	GT	12	13
13694	1	22376463	22376478	2	8	16	GT	26	5
13695	1	22376981	22377006	2	13	26	GT	20	8
13696	1	22378451	22378480	3	10	30	CCT	16	8
13697	1	22379092	22379107	2	8	16	TG	9	8
13698	1	22379204	22379239	2	18	36	TG	8	11
13699	1	22379286	22379303	2	9	18	CA	12	10
13701	1	22380347	22380362	2	8	16	TG	26	9
13702	1	22383056	22383077	2	11	22	AC	14	7
13703	1	22383476	22383503	2	14	28	AC	24	12
13704	1	22384067	22384092	2	13	26	CA	6	10
13714	1	22398108	22398137	3	10	30	GGA	18	4
13715	1	22399098	22399112	3	5	15	GGC	28	6
13718	1	22400629	22400658	2	15	30	AC	12	13
13720	1	22402242	22402279	2	19	38	TG	8	8
13723	1	22404919	22404964	2	23	46	CA	6	7
13724	1	22405115	22405152	2	19	38	CA	6	8
13725	1	22405271	22405312	2	21	42	GT	10	12
13726	1	22405439	22405458	2	10	20	AC	14	4
13727	1	22405934	22405951	2	9	18	TG	14	11
13728	1	22405954	22405967	2	7	14	TG	14	10
13729	1	22409457	22409486	2	15	30	AC	9	12
13734	1	22416701	22416756	2	28	56	TG	9	9
13737	1	22421076	22421117	2	21	42	AC	10	10
13738	1	22421225	22421254	2	15	30	TC	11	14
13740	1	22421374	22421399	2	13	26	TC	19	9
13741	1	22421533	22421554	2	11	22	TG	19	12
13742	1	22422795	22422826	2	16	32	TG	23	12
13743	1	22425225	22425250	2	13	26	AC	12	8
13744	1	22425392	22425427	2	18	36	GT	17	15
13746	1	22425590	22425607	2	9	18	TG	11	12
13747	1	22430239	22430250	2	6	12	AC	31	3
13750	1	22430801	22430838	2	19	38	AC	10	10
13753	1	22441917	22441934	2	9	18	TG	24	8
13754	1	22443134	22443149	2	8	16	AG	9	3
13758	1	22445220	22445233	2	7	14	AC	15	7
13760	1	22447197	22447226	2	15	30	AC	9	7
13764	1	22449119	22449152	2	17	34	CA	8	7

13765	1	22449967	22449992	2	13	26	AC	11	10
13766	1	22450783	22450814	2	16	32	TG	16	13
13767	1	22450873	22450898	2	13	26	GT	17	13
13770	1	22453799	22453810	2	6	12	GT	30	5
13771	1	22453869	22453880	2	6	12	TC	19	4
13772	1	22462740	22462767	2	14	28	TG	15	11
13773	1	22464116	22464131	2	8	16	AG	30	10
13775	1	22464375	22464389	3	5	15	AAC	18	5
13776	1	22464561	22464602	2	21	42	GT	12	11
13777	1	22465681	22465710	2	15	30	TG	11	10
13782	1	22466537	22466566	2	15	30	AC	13	5
13785	1	22467759	22467776	2	9	18	AG	21	4
13788	1	22468686	22468725	2	20	40	AC	7	8
13789	1	22469233	22469256	2	12	24	AC	10	10
13790	1	22470491	22470512	2	11	22	CA	12	10
13791	1	22470698	22470729	2	16	32	AC	10	10
13794	1	22470876	22470901	2	13	26	AC	16	5
13798	1	22472445	22472474	2	15	30	CA	6	7
13805	1	22475054	22475083	2	15	30	GT	12	12
13811	1	22478460	22478480	3	7	21	CTC	23	6
13812	1	22480951	22480968	2	9	18	CT	21	8
13813	1	22481220	22481249	2	15	30	CA	8	9
13814	1	22482039	22482071	3	11	33	CCT	18	12
13816	1	22482225	22482254	3	10	30	CTC	14	8
13817	1	22483187	22483224	2	19	38	TG	12	10
13818	1	22483581	22483614	2	17	34	AC	10	7
13820	1	22486040	22486061	2	11	22	AC	20	6
13821	1	22486122	22486151	2	15	30	AC	18	13
13824	1	22489183	22489194	2	6	12	GA	24	3
13825	1	22491309	22491344	3	12	36	TCC	17	9
13828	1	22493490	22493539	2	25	50	CA	6	8
13830	1	22493708	22493741	2	17	34	CA	8	13
13832	1	22495052	22495093	2	21	42	CA	6	8
13833	1	22495129	22495146	2	9	18	AC	18	6
13834	1	22498238	22498270	3	11	33	GGA	6	5
13839	1	22507156	22507173	2	9	18	CA	22	11
13841	1	22507308	22507349	2	21	42	TG	9	11
13842	1	22507537	22507556	2	10	20	GT	18	3
13844	1	22507660	22507703	2	22	44	AC	7	8
13847	1	22513826	22513845	2	10	20	CA	19	4
13848	1	22514054	22514071	2	9	18	CA	23	4
13860	1	22515817	22515840	2	12	24	CA	19	11
13861	1	22517816	22517851	3	12	36	TTC	10	3
13863	1	22517991	22518018	2	14	28	CA	12	13
13864	1	22518092	22518113	2	11	22	AC	19	7
13866	1	22518673	22518684	2	6	12	GT	26	3
13867	1	22519846	22519891	2	23	46	GT	11	11
13868	1	22522577	22522602	2	13	26	GT	8	9
13869	1	22523346	22523387	2	21	42	GT	12	10
13870	1	22523969	22524000	4	8	32	CTTC	23	6
13876	1	22529369	22529383	3	5	15	TGA	29	5

13877	1	22532160	22532177	2	9	18	AC	25	8
13881	1	22539860	22539883	3	8	24	TCC	31	7
13882	1	22542791	22542802	2	6	12	AC	25	5
13883	1	22544950	22544973	3	8	24	AAG	16	5
13884	1	22549373	22549408	2	18	36	TG	9	11
13889	1	22552576	22552605	3	10	30	GCT	21	5
13891	1	22553534	22553548	3	5	15	AGC	30	5
13893	1	22553998	22554024	3	9	27	ATC	17	5
13894	1	22554087	22554125	3	13	39	CTT	7	6
13909	2	13550	13561	2	6	12	GT	12	5
13910	2	13590	13605	2	8	16	GT	7	3
13915	2	21769	21794	2	13	26	TG	16	5
13921	2	33796	33811	2	8	16	TA	17	8
13933	2	52373	52388	4	4	16	GGAC	22	4
13936	2	55672	55686	3	5	15	AAC	7	4
13938	2	61240	61272	3	11	33	CTT	9	10
13956	2	117456	117475	4	5	20	ATAG	10	8
13973	2	179689	179700	2	6	12	AG	19	3
13991	2	230345	230396	2	26	52	GT	6	6
13992	2	231418	231433	4	4	16	TTAA	26	4
13998	2	245682	245695	2	7	14	TG	22	4
14009	2	259125	259151	3	9	27	GAA	14	6
14018	2	269453	269466	2	7	14	AC	22	4
14024	2	282282	282297	4	4	16	TATT	26	7
14025	2	283261	283292	2	16	32	CA	10	11
14034	2	303882	303933	4	13	52	ATAG	12	9
14055	2	335737	335754	2	9	18	CA	16	8
14063	2	340002	340013	2	6	12	GT	29	3
14066	2	342635	342649	3	5	15	CAT	9	6
14069	2	344538	344555	2	9	18	AC	18	8
14071	2	349196	349221	2	13	26	TG	14	6
14085	2	374173	374204	2	16	32	GT	14	12
14097	2	386403	386414	2	6	12	TA	19	3
14106	2	400050	400071	2	11	22	TG	16	8
14110	2	405508	405523	2	8	16	TC	16	3
14119	2	416844	416875	2	16	32	AC	10	9
14123	2	421903	421938	2	18	36	TG	19	19
14126	2	432517	432542	2	13	26	CA	23	6
14140	2	457183	457212	2	15	30	TG	9	9
14141	2	457369	457396	2	14	28	AC	20	5
14142	2	460121	460136	2	8	16	TG	14	4
14143	2	460328	460343	2	8	16	CA	20	5
14144	2	460563	460578	2	8	16	GT	21	12
14146	2	461323	461340	2	9	18	GT	17	9
14148	2	467484	467499	2	8	16	GT	24	5
14149	2	467537	467558	2	11	22	TG	13	4
14152	2	473022	473049	2	14	28	TG	8	9
14153	2	474343	474358	4	4	16	AAAT	22	3
14170	2	491074	491093	2	10	20	AC	16	5
14171	2	491266	491301	2	18	36	AC	9	6
14173	2	491536	491561	2	13	26	AC	15	7

14174	2	491884	491903	2	10	20	TG	24	11
14175	2	492416	492431	2	8	16	CA	19	7
14177	2	493529	493550	2	11	22	AC	18	5
14180	2	495426	495441	4	4	16	GATG	32	3
14181	2	496873	496888	2	8	16	TA	7	3
14183	2	511036	511059	2	12	24	AC	21	7
14189	2	512651	512668	3	6	18	ATT	17	12
14192	2	517015	517028	2	7	14	CA	21	3
14199	2	532226	532237	2	6	12	GT	25	4
14202	2	534874	534895	2	11	22	TG	18	6
14205	2	550583	550598	2	8	16	AC	26	3
14220	2	598673	598718	2	23	46	AC	9	10
14221	2	598818	598845	2	14	28	GT	20	8
14226	2	603659	603679	3	7	21	TAA	10	8
14228	2	608734	608781	4	12	48	TGTA	9	6
14229	2	615438	615451	2	7	14	TG	17	6
14231	2	619972	619985	2	7	14	TG	27	3
14236	2	631199	631214	2	8	16	TA	24	7
14244	2	651315	651346	2	16	32	GT	10	9
14246	2	652927	652947	3	7	21	GAT	7	4
14251	2	661647	661664	2	9	18	AC	25	3
14252	2	662492	662517	2	13	26	CA	11	11
14256	2	667747	667770	2	12	24	CA	22	11
14262	2	679324	679338	3	5	15	CAG	29	3
14269	2	695926	695947	2	11	22	TG	17	13
14270	2	698480	698493	2	7	14	TG	13	4
14276	2	711161	711194	2	17	34	AC	14	10
14277	2	714156	714187	4	8	32	TTAT	20	7
14288	2	741603	741632	2	15	30	CA	13	11
14290	2	751407	751424	2	9	18	CA	19	9
14293	2	752550	752569	2	10	20	TC	12	4
14297	2	764491	764505	3	5	15	AAT	13	7
14298	2	764918	764933	4	4	16	AGTG	17	3
14311	2	790002	790015	2	7	14	TG	26	3
14312	2	790183	790194	2	6	12	CA	22	5
14313	2	790197	790210	2	7	14	CA	15	5
14315	2	793006	793029	4	6	24	GATG	26	13
14318	2	797816	797827	2	6	12	TG	11	5
14323	2	808793	808822	2	15	30	CA	8	12
14330	2	819183	819194	2	6	12	AC	23	3
14331	2	819388	819413	2	13	26	GT	18	10
14334	2	832409	832426	2	9	18	TG	19	3
14335	2	833970	833985	4	4	16	AGAC	24	6
14348	2	861516	861563	2	24	48	AG	12	11
14349	2	862399	862420	2	11	22	TG	18	8
14352	2	870413	870434	2	11	22	GT	14	11
14353	2	870949	870964	2	8	16	TG	19	6
14354	2	871087	871098	2	6	12	GT	28	3
14359	2	905291	905320	2	15	30	CA	9	12
14367	2	913753	913776	2	12	24	GT	16	10
14368	2	915336	915363	2	14	28	CA	12	12

14378	2	928042	928057	2	8	16	GT	16	5
14380	2	931585	931602	2	9	18	GT	12	5
14382	2	932782	932801	2	10	20	CA	25	8
14384	2	940445	940456	2	6	12	GT	22	5
14386	2	945189	945216	2	14	28	CA	14	10
14397	2	973203	973238	4	9	36	AAAG	11	11
14399	2	978248	978259	2	6	12	CA	21	6
14402	2	980607	980620	2	7	14	TG	33	3
14403	2	983457	983488	2	16	32	AC	9	7
14408	2	990747	990758	2	6	12	GT	16	5
14409	2	990761	990772	2	6	12	GT	15	6
14414	2	996623	996660	2	19	38	CT	14	8
14419	2	1004376	1004389	2	7	14	AC	18	6
14421	2	1014741	1014770	2	15	30	TG	14	9
14428	2	1044953	1044972	2	10	20	GT	24	11
14429	2	1046071	1046084	2	7	14	AC	21	3
14441	2	1076555	1076580	2	13	26	GT	9	6
14448	2	1097857	1097872	4	4	16	ACAT	25	4
14449	2	1099496	1099513	3	6	18	TCC	21	7
14454	2	1108014	1108025	2	6	12	CA	28	4
14455	2	1108166	1108179	2	7	14	AC	12	3
14464	2	1128837	1128863	3	9	27	TTG	13	8
14465	2	1129531	1129554	2	12	24	GT	8	5
14472	2	1154970	1154987	2	9	18	AC	21	10
14473	2	1157668	1157709	2	21	42	AC	13	17
14474	2	1158208	1158219	2	6	12	CA	25	3
14475	2	1161025	1161050	2	13	26	GT	11	10
14482	2	1163661	1163674	2	7	14	TC	21	6
14483	2	1164191	1164214	2	12	24	CA	16	11
14484	2	1165936	1165965	3	10	30	AGA	16	9
14498	2	1183518	1183533	2	8	16	GT	24	3
14502	2	1201995	1202006	2	6	12	TG	27	3
14512	2	1220213	1220232	2	10	20	CA	11	8
14514	2	1220386	1220407	2	11	22	AC	11	8
14519	2	1230673	1230686	2	7	14	GT	14	4
14521	2	1231277	1231294	2	9	18	AC	12	5
14534	2	1255347	1255364	3	6	18	TCT	34	7
14535	2	1256196	1256237	2	21	42	AC	20	19
14542	2	1265365	1265392	2	14	28	AC	10	9
14545	2	1274596	1274619	2	12	24	AC	18	6
14551	2	1283064	1283099	2	18	36	GT	12	7
14552	2	1283323	1283342	2	10	20	AC	14	10
14557	2	1287706	1287733	2	14	28	CA	11	8
14563	2	1306714	1306739	2	13	26	TG	18	13
14565	2	1309640	1309665	2	13	26	TG	16	10
14566	2	1310018	1310031	2	7	14	TC	11	4
14567	2	1310052	1310063	2	6	12	TC	22	6
14571	2	1316860	1316903	2	22	44	CA	20	12
14574	2	1323613	1323642	3	10	30	GGA	16	4
14576	2	1332886	1332905	4	5	20	ATTT	28	6
14579	2	1337328	1337357	2	15	30	GT	12	9

14580	2	1340146	1340179	2	17	34	AC	18	13
14581	2	1340279	1340294	2	8	16	CA	20	8
14585	2	1343606	1343629	3	8	24	AAC	16	5
14590	2	1348936	1348949	2	7	14	TG	19	5
14599	2	1371318	1371353	4	9	36	ATTG	11	6
14603	2	1381111	1381130	4	5	20	TTTG	20	4
14605	2	1385177	1385190	2	7	14	CA	19	3
14613	2	1398407	1398428	2	11	22	CA	18	9
14614	2	1399300	1399313	2	7	14	AT	22	4
14625	2	1417978	1417989	2	6	12	CA	8	3
14629	2	1434426	1434441	2	8	16	CA	24	3
14631	2	1440871	1440912	2	21	42	TG	9	10
14646	2	1466973	1466984	2	6	12	TG	17	3
14647	2	1473007	1473038	4	8	32	TAGA	9	11
14648	2	1474219	1474246	4	7	28	ATAG	22	6
14651	2	1476091	1476122	2	16	32	TG	12	8
14656	2	1494018	1494043	2	13	26	AT	15	8
14659	2	1497812	1497831	4	5	20	TTTA	13	6
14660	2	1499238	1499251	2	7	14	GT	21	3
14662	2	1507815	1507828	2	7	14	AC	25	4
14663	2	1508094	1508133	2	20	40	GT	13	8
14668	2	1518831	1518874	4	11	44	ACTT	11	8
14669	2	1520618	1520631	2	7	14	GA	23	5
14672	2	1524216	1524229	2	7	14	TG	11	3
14676	2	1531617	1531628	2	6	12	AT	11	6
14677	2	1531850	1531865	4	4	16	AAAT	21	4
14680	2	1543184	1543199	2	8	16	AT	11	5
14681	2	1545454	1545469	2	8	16	TG	6	3
14692	2	1580550	1580573	2	12	24	TG	11	11
14696	2	1585801	1585824	2	12	24	TG	17	7
14701	2	1592282	1592303	2	11	22	TC	23	4
14702	2	1593109	1593156	4	12	48	CAGA	23	13
14706	2	1608176	1608189	2	7	14	TA	8	8
14707	2	1608586	1608615	2	15	30	TG	15	7
14709	2	1610072	1610083	2	6	12	AG	26	3
14714	2	1654109	1654130	2	11	22	GT	11	7
14729	2	1674790	1674847	2	29	58	AC	7	11
14730	2	1676599	1676630	2	16	32	GT	11	6
14734	2	1683699	1683754	4	14	56	TGAT	6	5
14737	2	1688936	1688962	3	9	27	TTG	23	7
14739	2	1691307	1691346	4	10	40	ATAG	19	10
14741	2	1692299	1692318	4	5	20	TTAT	18	3
14743	2	1708292	1708311	2	10	20	AC	23	8
14746	2	1714015	1714034	4	5	20	TTTA	27	5
14759	2	1739661	1739678	3	6	18	AAT	21	5
14760	2	1739870	1739899	2	15	30	GT	17	11
14780	2	1771931	1771958	4	7	28	TGAT	22	15
14787	2	1792650	1792661	2	6	12	GT	19	3
14792	2	1805632	1805687	4	14	56	TCTA	15	11
14798	2	1818012	1818047	2	18	36	AC	10	15
14799	2	1823646	1823675	2	15	30	TG	13	7

14804	2	1832990	1833007	2	9	18	TG	9	13
14809	2	1859426	1859441	4	4	16	TGTA	24	3
14813	2	1883892	1883907	2	8	16	CA	21	6
14814	2	1884231	1884268	2	19	38	AC	6	9
14815	2	1887830	1887844	3	5	15	ATA	10	4
14818	2	1891920	1891939	4	5	20	GGAC	8	5
14827	2	1905603	1905623	3	7	21	AGG	15	4
14829	2	1907939	1907956	2	9	18	TG	13	8
14833	2	1926206	1926217	2	6	12	GA	29	4
14834	2	1929384	1929415	2	16	32	AC	14	6
14835	2	1930819	1930850	2	16	32	TG	17	14
14837	2	1933357	1933374	2	9	18	TG	17	8
14838	2	1934243	1934269	3	9	27	ACA	10	6
14840	2	1959060	1959074	3	5	15	TCC	25	3
14841	2	1961580	1961605	2	13	26	TG	26	16
14842	2	1962564	1962577	2	7	14	TC	26	3
14843	2	1964511	1964532	2	11	22	GT	19	12
14848	2	1984365	1984380	4	4	16	TAAA	29	4
14849	2	1987606	1987635	2	15	30	TG	9	10
14868	2	2047985	2048002	2	9	18	AT	6	4
14870	2	2050356	2050393	2	19	38	AG	6	11
14871	2	2065757	2065772	4	4	16	ATCC	27	5
14881	2	2080632	2080647	2	8	16	TA	10	3
14885	2	2083343	2083360	3	6	18	ATG	16	7
14887	2	2087198	2087227	2	15	30	AC	11	11
14906	2	2158107	2158124	3	6	18	TCC	25	11
14913	2	2171095	2171106	2	6	12	AG	23	3
14925	2	2192906	2192949	2	22	44	CA	8	9
14926	2	2193507	2193518	2	6	12	GT	24	4
14927	2	2193732	2193745	2	7	14	AG	13	5
14928	2	2193747	2193760	2	7	14	GA	6	3
14939	2	2194069	2194086	2	9	18	TC	7	6
14948	2	2200603	2200634	4	8	32	ACAT	6	7
14950	2	2201316	2201329	2	7	14	AC	21	7
14963	2	2211938	2211977	2	20	40	CA	16	9
14965	2	2217086	2217117	4	8	32	CCTG	25	14
14972	2	2220195	2220224	2	15	30	AC	24	10
14973	2	2220429	2220444	2	8	16	CA	19	10
14976	2	2225597	2225612	2	8	16	GT	17	8
14979	2	2226163	2226188	2	13	26	AG	7	5
14991	2	2240580	2240607	2	14	28	CA	13	9
14992	2	2240847	2240888	2	21	42	CA	8	11
14993	2	2242518	2242553	2	18	36	TG	19	8
14995	2	2242863	2242884	2	11	22	AC	16	12
14996	2	2242989	2243004	2	8	16	AC	22	6
14998	2	2250012	2250026	3	5	15	GAG	26	5
14999	2	2250596	2250615	2	10	20	TG	21	5
15001	2	2250797	2250812	2	8	16	CA	20	10
15007	2	2259188	2259221	2	17	34	CA	12	11
15010	2	2262322	2262351	2	15	30	TG	11	15
15012	2	2262464	2262477	2	7	14	CA	25	4

15013	2	2262743	2262762	2	10	20	TG	23	12
15016	2	2264255	2264270	4	4	16	GCAG	27	5
15018	2	2268484	2268511	2	14	28	AC	8	9
15019	2	2268625	2268642	2	9	18	GA	15	5
15020	2	2268683	2268708	2	13	26	CT	23	7
15021	2	2268752	2268769	2	9	18	CA	15	11
15026	2	2273257	2273272	2	8	16	TG	26	7
15027	2	2273454	2273483	2	15	30	AC	11	10
15028	2	2273652	2273663	2	6	12	CT	19	8
15029	2	2273666	2273677	2	6	12	CT	23	8
15030	2	2273801	2273830	2	15	30	TG	15	10
15031	2	2274240	2274255	2	8	16	GT	23	11
15032	2	2274257	2274272	2	8	16	TG	20	11
15034	2	2277524	2277555	2	16	32	TG	17	13
15036	2	2277996	2278009	2	7	14	AG	18	5
15048	2	2286398	2286417	2	10	20	TC	10	3
15049	2	2287035	2287052	2	9	18	AC	17	6
15059	2	2299272	2299283	2	6	12	TG	27	4
15065	2	2301806	2301857	2	26	52	GA	7	4
15069	2	2307030	2307057	2	14	28	AC	26	6
15070	2	2307695	2307712	2	9	18	CA	16	10
15071	2	2313422	2313447	2	13	26	GT	19	9
15073	2	2319006	2319053	2	24	48	AC	8	10
15081	2	2339708	2339737	2	15	30	AC	15	9
15086	2	2355642	2355673	2	16	32	TG	11	8
15093	2	2364134	2364147	2	7	14	GT	25	6
15097	2	2371451	2371464	2	7	14	AC	31	5
15098	2	2375606	2375619	2	7	14	AG	10	8
15111	2	2392151	2392178	2	14	28	CT	12	15
15112	2	2395370	2395405	2	18	36	GT	14	10
15113	2	2395523	2395538	2	8	16	AC	16	3
15114	2	2396663	2396690	2	14	28	CA	19	9
15116	2	2404822	2404845	2	12	24	CA	20	9
15117	2	2405138	2405157	2	10	20	CA	25	3
15120	2	2412388	2412403	2	8	16	TG	19	7
15123	2	2412703	2412726	2	12	24	CA	20	6
15124	2	2412821	2412846	2	13	26	AC	19	7
15138	2	2464869	2464882	2	7	14	TA	6	6
15142	2	2471174	2471187	2	7	14	GT	22	6
15145	2	2476287	2476298	2	6	12	TG	26	3
15151	2	2486530	2486545	2	8	16	GT	13	9
15154	2	2499322	2499349	2	14	28	CA	12	6
15157	2	2501471	2501486	2	8	16	GT	27	4
15166	2	2520272	2520303	2	16	32	AC	13	7
15168	2	2520723	2520754	2	16	32	GA	16	13
15172	2	2530567	2530588	2	11	22	TG	6	3
15174	2	2536923	2536950	4	7	28	AGAC	31	5
15175	2	2537939	2537952	2	7	14	AC	24	6
15176	2	2540502	2540529	2	14	28	TG	17	9
15177	2	2540827	2540840	2	7	14	GT	9	9
15178	2	2540844	2540859	2	8	16	TG	10	7

15179	2	2544578	2544601	2	12	24	AC	24	11
15182	2	2547340	2547360	3	7	21	AAC	7	5
15183	2	2550138	2550155	2	9	18	CA	19	11
15184	2	2550319	2550338	2	10	20	AC	17	4
15187	2	2553076	2553095	2	10	20	CA	20	14
15188	2	2553177	2553220	2	22	44	GT	12	15
15193	2	2559532	2559551	2	10	20	GT	19	7
15196	2	2562232	2562251	2	10	20	GT	16	3
15199	2	2562712	2562735	2	12	24	AC	18	6
15208	2	2570610	2570645	3	12	36	AAG	21	10
15210	2	2571577	2571596	2	10	20	TG	8	8
15216	2	2578450	2578465	2	8	16	AC	23	5
15217	2	2578795	2578814	2	10	20	TC	22	5
15218	2	2579087	2579114	2	14	28	CA	14	7
15219	2	2579250	2579265	2	8	16	TG	15	9
15220	2	2580427	2580450	2	12	24	TG	16	8
15237	2	2596649	2596666	2	9	18	AC	18	3
15248	2	2600408	2600421	2	7	14	CA	20	4
15250	2	2603405	2603446	2	21	42	AC	11	11
15253	2	2611747	2611785	3	13	39	GAT	23	6
15259	2	2616458	2616495	2	19	38	AC	10	10
15266	2	2623429	2623452	2	12	24	CT	24	7
15274	2	2629773	2629794	2	11	22	GT	15	11
15281	2	2636231	2636256	2	13	26	GA	10	11
15282	2	2636420	2636469	2	25	50	AC	9	11
15283	2	2636494	2636517	2	12	24	AC	11	7
15287	2	2641313	2641330	2	9	18	TC	22	7
15288	2	2641331	2641348	2	9	18	AC	26	10
15291	2	2649180	2649197	2	9	18	GT	6	4
15294	2	2649341	2649384	2	22	44	TG	6	8
15295	2	2649581	2649602	2	11	22	AG	12	3
15313	2	2687748	2687771	4	6	24	GAAA	24	8
15324	2	2712864	2712875	2	6	12	TG	18	4
15329	2	2722354	2722401	4	12	48	ATAG	11	13
15331	2	2726042	2726065	2	12	24	CT	13	5
15332	2	2726159	2726174	4	4	16	AGGC	18	3
15333	2	2726317	2726346	2	15	30	CT	13	12
15336	2	2728055	2728072	2	9	18	AT	15	3
15342	2	2739499	2739522	3	8	24	CCT	20	9
15348	2	2748639	2748670	2	16	32	AC	12	5
15350	2	2751272	2751287	2	8	16	AG	31	6
15351	2	2752878	2752893	4	4	16	TTTG	20	3
15352	2	2753820	2753837	2	9	18	TG	22	9
15365	2	2776947	2776970	2	12	24	TG	18	10
15370	2	2791631	2791660	2	15	30	GT	24	9
15381	2	2837283	2837294	2	6	12	GT	30	5
15382	2	2837344	2837357	2	7	14	GT	31	8
15388	2	2848227	2848270	4	11	44	CCAG	10	11
15392	2	2868154	2868193	4	10	40	AAAC	15	6
15394	2	2881711	2881732	2	11	22	GT	20	7
15396	2	2882178	2882197	4	5	20	TGTC	26	8

15397	2	2885148	2885171	4	6	24	TAAA	22	4
15398	2	2890892	2890911	4	5	20	TTTA	18	6
15406	2	2899234	2899259	2	13	26	TG	9	13
15410	2	2901980	2901991	2	6	12	TG	22	4
15423	2	2933069	2933104	2	18	36	GT	11	11
15428	2	2938488	2938501	2	7	14	GT	28	4
15431	2	2940697	2940714	2	9	18	AC	10	6
15432	2	2940717	2940730	2	7	14	AC	15	9
15434	2	2943202	2943215	2	7	14	CA	27	4
15436	2	2943477	2943488	2	6	12	CA	18	4
15446	2	2951924	2951949	2	13	26	GA	12	4
15464	2	2985900	2985923	2	12	24	CA	21	4
15466	2	2993983	2993996	2	7	14	AC	10	3
15471	2	2999891	2999910	2	10	20	CA	14	9
15473	2	3000683	3000700	2	9	18	TG	21	4
15478	2	3015891	3015906	2	8	16	CA	17	5
15485	2	3027071	3027086	2	8	16	TG	20	3
15486	2	3029486	3029499	2	7	14	TG	28	3
15488	2	3038670	3038681	2	6	12	AC	17	9
15494	2	3046721	3046734	2	7	14	TC	22	3
15502	2	3059177	3059208	4	8	32	GACA	24	8
15506	2	3066644	3066663	2	10	20	GT	19	4
15512	2	3075708	3075723	4	4	16	GCAC	23	3
15517	2	3078523	3078536	2	7	14	AC	26	3
15522	2	3086199	3086225	3	9	27	CTC	20	3
15526	2	3099183	3099198	2	8	16	GT	16	7
15531	2	3113387	3113420	2	17	34	AC	11	12
15542	2	3122840	3122857	2	9	18	AG	8	3
15543	2	3122970	3122989	2	10	20	TG	15	8
15546	2	3129425	3129444	2	10	20	GT	24	6
15552	2	3140979	3141002	2	12	24	AC	12	4
15555	2	3147788	3147799	2	6	12	CA	31	3
15569	2	3171517	3171566	2	25	50	CA	11	11
15599	2	3222011	3222024	2	7	14	TG	30	3
15617	2	3246955	3246974	2	10	20	AC	21	6
15618	2	3249198	3249209	2	6	12	GT	26	6
15619	2	3249213	3249228	2	8	16	TG	25	5
15627	2	3268889	3268918	3	10	30	AGA	21	4
15630	2	3275003	3275018	4	4	16	AGGC	23	3
15636	2	3276183	3276200	3	6	18	AAG	16	7
15649	2	3286290	3286307	2	9	18	CT	19	5
15650	2	3290716	3290739	2	12	24	AC	15	8
15654	2	3301185	3301210	2	13	26	TG	17	10
15658	2	3304183	3304194	2	6	12	TC	28	3
15663	2	3310856	3310879	2	12	24	CA	13	11
15665	2	3317974	3317985	2	6	12	CA	28	3
15667	2	3318160	3318179	2	10	20	TG	16	9
15671	2	3325551	3325562	2	6	12	CA	25	4
15676	2	3332487	3332504	2	9	18	CA	14	5
15678	2	3338964	3338991	2	14	28	TG	19	13
15683	2	3347659	3347686	2	14	28	CT	20	12

15706	2	3381023	3381048	2	13	26	GA	13	4
15711	2	3386207	3386240	2	17	34	TG	11	10
15712	2	3386394	3386419	2	13	26	AG	21	7
15716	2	3390929	3390942	2	7	14	AC	26	4
15717	2	3392379	3392414	2	18	36	TG	9	9
15720	2	3401212	3401245	2	17	34	AC	9	11
15721	2	3401325	3401350	2	13	26	AC	17	10
15723	2	3402402	3402437	2	18	36	CA	15	11
15726	2	3406685	3406712	2	14	28	TC	10	6
15727	2	3406844	3406863	4	5	20	GATG	26	3
15751	2	3459356	3459385	3	10	30	ATG	29	5
15755	2	3479891	3479904	2	7	14	AT	25	3
15759	2	3514792	3514813	2	11	22	GT	12	5
15760	2	3515424	3515441	2	9	18	GT	23	5
15761	2	3530487	3530498	2	6	12	GT	24	4
15762	2	3531789	3531809	3	7	21	TGT	22	5
15766	2	3543536	3543565	2	15	30	TG	15	10
15767	2	3543599	3543644	2	23	46	GA	7	13
15769	2	3544194	3544208	3	5	15	CCG	21	3
15770	2	3546460	3546475	4	4	16	ATTT	7	4
15773	2	3550365	3550376	2	6	12	TG	34	3
15776	2	3555426	3555459	2	17	34	TG	16	14
15781	2	3566645	3566658	2	7	14	AC	22	11
15786	2	3580114	3580125	2	6	12	AT	29	3
15797	2	3598756	3598779	2	12	24	GT	20	12
15799	2	3599023	3599038	2	8	16	AC	11	8
15808	2	3613397	3613414	3	6	18	GAA	27	3
15809	2	3613865	3613892	2	14	28	CA	11	10
15811	2	3627457	3627472	2	8	16	TG	20	3
15812	2	3628249	3628262	2	7	14	CA	29	7
15817	2	3640019	3640036	2	9	18	TG	13	4
15820	2	3657000	3657029	2	15	30	CA	12	9
15821	2	3657115	3657132	2	9	18	TG	22	3
15825	2	3664990	3665003	2	7	14	CA	27	5
15830	2	3672453	3672466	2	7	14	GT	18	5
15833	2	3682740	3682759	2	10	20	CT	19	6
15834	2	3683340	3683357	3	6	18	AGC	26	4
15838	2	3693177	3693200	2	12	24	GT	18	13
15849	2	3718639	3718671	3	11	33	TGT	22	10
15850	2	3721443	3721462	2	10	20	AC	16	7
15851	2	3730294	3730323	2	15	30	AC	12	13
15855	2	3731369	3731390	2	11	22	GT	9	9
15856	2	3731475	3731498	2	12	24	GT	19	6
15858	2	3735367	3735384	2	9	18	GA	21	5
15860	2	3738593	3738613	3	7	21	TCC	23	6
15861	2	3740100	3740119	2	10	20	GT	26	10
15863	2	3746826	3746851	2	13	26	GT	13	7
15864	2	3747265	3747280	2	8	16	CT	22	11
15867	2	3749038	3749049	2	6	12	CA	21	7
15868	2	3749438	3749453	2	8	16	GT	24	6
15873	2	3753556	3753577	2	11	22	GT	20	11

15876	2	3760515	3760554	2	20	40	AC	10	10
15877	2	3770097	3770120	4	6	24	ATCC	28	5
15889	2	3795625	3795640	2	8	16	CT	23	4
15895	2	3826512	3826527	4	4	16	AAAC	24	3
15901	2	3833681	3833698	2	9	18	TG	18	5
15907	2	3853667	3853684	2	9	18	GT	13	4
15912	2	3863070	3863101	2	16	32	TG	6	4
15924	2	3874113	3874132	4	5	20	CTGC	18	5
15925	2	3875810	3875835	2	13	26	TG	7	8
15932	2	3888615	3888642	2	14	28	GA	8	8
15936	2	3890924	3890939	2	8	16	CT	29	4
15937	2	3892316	3892329	2	7	14	TG	18	4
15941	2	3893217	3893228	2	6	12	AC	15	3
15942	2	3893266	3893279	2	7	14	TG	14	7
15943	2	3893894	3893905	2	6	12	TG	15	3
15945	2	3894453	3894466	2	7	14	CA	25	4
15946	2	3894887	3894912	2	13	26	GT	12	10
15947	2	3894944	3894957	2	7	14	GA	22	7
15954	2	3899424	3899439	2	8	16	AC	26	6
15961	2	3902163	3902186	3	8	24	TCC	7	3
15962	2	3902427	3902442	2	8	16	AT	10	3
15969	2	3912202	3912229	2	14	28	TG	12	9
15970	2	3912431	3912442	2	6	12	TG	12	4
15972	2	3912680	3912693	2	7	14	AC	23	3
15980	2	3929074	3929103	2	15	30	AC	7	8
15992	2	3958242	3958253	2	6	12	AC	17	4
15996	2	3963452	3963467	2	8	16	GT	10	7
15997	2	3963470	3963487	2	9	18	GT	14	7
15998	2	3964391	3964434	2	22	44	AC	8	8
16004	2	3968078	3968099	2	11	22	TG	20	5
16012	2	3977908	3977923	4	4	16	ATTT	27	3
16013	2	3978921	3978950	2	15	30	TG	13	9
16016	2	3980704	3980727	3	8	24	CAC	19	4
16021	2	3988359	3988390	2	16	32	GA	13	7
16025	2	3991437	3991462	2	13	26	AC	19	9
16026	2	3991681	3991692	2	6	12	GT	22	7
16029	2	3992328	3992341	2	7	14	GT	14	10
16030	2	3992913	3992936	2	12	24	AC	10	10
16034	2	3993325	3993338	2	7	14	CT	14	4
16036	2	3994386	3994403	2	9	18	GA	11	9
16041	2	3996638	3996661	3	8	24	TCT	24	3
16043	2	3998381	3998418	2	19	38	TG	6	8
16054	2	4011601	4011614	2	7	14	TG	24	7
16055	2	4012663	4012682	2	10	20	CA	12	3
16056	2	4013248	4013277	2	15	30	CA	8	9
16061	2	4025361	4025376	2	8	16	TG	24	3
16063	2	4033229	4033240	2	6	12	GA	10	3
16087	2	4058104	4058121	2	9	18	TG	18	5
16088	2	4058177	4058196	4	5	20	GTGC	20	8
16092	2	4066787	4066810	2	12	24	CA	8	6
16110	2	4126759	4126778	2	10	20	CA	18	7

16111	2	4132155	4132168	2	7	14	CA	28	3
16137	2	4164694	4164705	2	6	12	AC	20	4
16139	2	4173673	4173684	2	6	12	TG	25	3
16142	2	4184145	4184162	2	9	18	AC	10	3
16154	2	4204662	4204688	3	9	27	CAG	19	3
16155	2	4204773	4204792	2	10	20	CA	16	7
16159	2	4215375	4215390	2	8	16	AC	21	5
16161	2	4230179	4230208	2	15	30	GA	12	8
16165	2	4234166	4234179	2	7	14	GT	27	4
16170	2	4242814	4242834	3	7	21	AAT	16	3
16172	2	4248857	4248882	2	13	26	TC	20	9
16174	2	4249168	4249183	2	8	16	TC	19	6
16176	2	4252341	4252356	4	4	16	CAGT	26	4
16179	2	4253921	4253952	2	16	32	AC	14	8
16182	2	4254867	4254881	3	5	15	GCA	8	3
16192	2	4274044	4274059	4	4	16	AAAT	28	3
16193	2	4275312	4275341	2	15	30	CA	15	10
16197	2	4279858	4279881	4	6	24	GGAT	21	6
16199	2	4283898	4283915	3	6	18	AGT	26	3
16202	2	4298227	4298238	2	6	12	GT	21	3
16203	2	4298830	4298841	2	6	12	CA	24	3
16208	2	4308060	4308081	2	11	22	AC	13	9
16211	2	4312224	4312235	2	6	12	AC	18	5
16212	2	4312377	4312410	2	17	34	GT	24	18
16216	2	4314485	4314506	2	11	22	CA	20	8
16218	2	4314678	4314695	2	9	18	CA	9	6
16220	2	4317078	4317103	2	13	26	CA	19	11
16222	2	4319353	4319376	2	12	24	AC	12	8
16239	2	4355227	4355248	2	11	22	CT	20	4
16245	2	4364661	4364682	2	11	22	TG	8	9
16246	2	4365215	4365234	2	10	20	GT	15	6
16259	2	4374341	4374354	2	7	14	GT	28	5
16264	2	4377809	4377832	2	12	24	AC	12	12
16267	2	4384365	4384396	2	16	32	TC	10	8
16269	2	4385055	4385070	2	8	16	AC	19	4
16270	2	4385508	4385523	2	8	16	CA	14	3
16272	2	4386235	4386256	2	11	22	CA	10	7
16277	2	4391927	4391942	2	8	16	TC	9	3
16279	2	4392963	4392982	2	10	20	GA	9	4
16280	2	4393275	4393292	2	9	18	AC	12	5
16286	2	4396081	4396104	2	12	24	TG	7	10
16289	2	4396433	4396450	2	9	18	CA	8	3
16290	2	4397444	4397491	2	24	48	CA	14	12
16291	2	4397550	4397573	2	12	24	CT	15	5
16293	2	4397692	4397717	2	13	26	TG	8	9
16294	2	4397837	4397856	2	10	20	TG	17	6
16295	2	4398388	4398413	2	13	26	TG	7	9
16296	2	4398570	4398605	2	18	36	AC	7	6
16297	2	4399000	4399020	3	7	21	CCA	13	3
16298	2	4399520	4399539	2	10	20	GT	12	5
16314	2	4423770	4423781	2	6	12	AG	27	3

16315	2	4425204	4425219	2	8	16	CA	21	9
16317	2	4429478	4429511	2	17	34	CA	12	15
16321	2	4435872	4435889	2	9	18	CA	23	4
16324	2	4440673	4440726	3	18	54	GTA	20	8
16325	2	4444401	4444414	2	7	14	GT	16	3
16327	2	4444923	4444942	2	10	20	TG	22	8
16328	2	4447209	4447264	2	28	56	CA	18	9
16330	2	4449428	4449441	2	7	14	CA	25	3
16332	2	4451881	4451900	2	10	20	TG	12	4
16336	2	4452622	4452637	2	8	16	TG	27	6
16337	2	4452708	4452723	2	8	16	AC	27	4
16338	2	4452819	4452830	2	6	12	AC	26	6
16340	2	4457633	4457648	2	8	16	TC	16	9
16341	2	4457650	4457663	2	7	14	CT	15	8
16343	2	4462688	4462719	2	16	32	TG	13	6
16344	2	4462831	4462851	3	7	21	GGT	8	4
16345	2	4467750	4467779	2	15	30	CA	13	8
16346	2	4470545	4470562	2	9	18	CA	21	8
16352	2	4481274	4481287	2	7	14	TC	21	3
16353	2	4483157	4483180	2	12	24	AC	13	10
16367	2	4493568	4493597	2	15	30	TG	8	7
16368	2	4494017	4494046	2	15	30	TG	11	12
16372	2	4501158	4501179	2	11	22	TC	9	3
16376	2	4507633	4507658	2	13	26	GT	18	7
16395	2	4526525	4526552	2	14	28	AC	10	7
16396	2	4527739	4527752	2	7	14	AC	20	4
16401	2	4528197	4528228	2	16	32	TG	9	5
16402	2	4528303	4528318	2	8	16	GT	17	5
16404	2	4528467	4528500	2	17	34	GT	17	10
16406	2	4532776	4532790	3	5	15	TTG	19	4
16410	2	4534840	4534851	2	6	12	CA	24	4
16411	2	4537513	4537526	2	7	14	TG	10	5
16412	2	4537528	4537539	2	6	12	GT	15	5
16413	2	4537689	4537714	2	13	26	TG	15	10
16425	2	4551731	4551756	2	13	26	CA	8	11
16427	2	4552185	4552202	2	9	18	CT	8	5
16432	2	4555056	4555095	2	20	40	GT	11	8
16435	2	4558638	4558657	2	10	20	CA	23	7
16446	2	4564547	4564558	2	6	12	TG	21	3
16455	2	4569570	4569587	3	6	18	CCT	21	5
16456	2	4571201	4571222	2	11	22	TC	25	7
16460	2	4581977	4582006	2	15	30	TG	10	11
16461	2	4590647	4590660	2	7	14	TG	8	4
16463	2	4592558	4592573	4	4	16	AGAT	23	4
16464	2	4594059	4594074	2	8	16	GT	27	4
16465	2	4598666	4598681	4	4	16	TTTG	15	5
16466	2	4598808	4598827	2	10	20	AC	19	5
16469	2	4599838	4599863	2	13	26	CA	19	16
16475	2	4611023	4611040	3	6	18	AGG	25	3
16476	2	4613949	4613964	2	8	16	CT	23	8
16477	2	4613965	4613978	2	7	14	CA	23	8

16478	2	4614184	4614195	2	6	12	TG	16	4
16479	2	4614308	4614327	2	10	20	TG	10	9
16487	2	4619972	4619987	2	8	16	AC	27	3
16489	2	4623797	4623820	2	12	24	CA	13	13
16493	2	4632476	4632493	3	6	18	TTC	19	4
16495	2	4638257	4638286	2	15	30	TG	17	12
16496	2	4638483	4638497	3	5	15	TGC	34	3
16497	2	4641948	4641961	2	7	14	AG	20	5
16498	2	4642260	4642281	2	11	22	AC	12	5
16500	2	4644488	4644507	2	10	20	CA	17	7
16501	2	4645486	4645501	4	4	16	CAAA	30	5
16508	2	4647524	4647539	4	4	16	AAAC	23	3
16509	2	4650563	4650574	2	6	12	TC	24	4
16513	2	4661424	4661441	3	6	18	GAG	23	4
16537	2	4717104	4717127	3	8	24	CCT	19	3
16553	2	4727551	4727565	3	5	15	GCA	25	3
16554	2	4727813	4727840	4	7	28	TTTG	16	4
16560	2	4736589	4736620	2	16	32	AC	15	12
16565	2	4746934	4746949	4	4	16	AAAT	22	3
16566	2	4746993	4747014	2	11	22	TG	16	12
16574	2	4759597	4759628	2	16	32	TC	9	5
16579	2	4778009	4778053	3	15	45	ATC	23	8
16583	2	4793055	4793070	2	8	16	AC	30	4
16584	2	4793258	4793277	4	5	20	CATC	21	3
16585	2	4794497	4794508	2	6	12	TG	32	3
16596	2	4809577	4809604	2	14	28	CA	13	13
16598	2	4810555	4810566	2	6	12	GT	23	4
16601	2	4829385	4829408	2	12	24	TG	12	7
16603	2	4840441	4840458	3	6	18	TTC	9	4
16604	2	4841531	4841554	2	12	24	AC	21	12
16607	2	4844894	4844907	2	7	14	CA	25	8
16608	2	4846669	4846680	2	6	12	AC	23	6
16618	2	4862179	4862194	2	8	16	TG	22	3
16623	2	4880132	4880145	2	7	14	TG	18	3
16635	2	4894291	4894332	2	21	42	CA	11	8
16636	2	4895389	4895400	2	6	12	TG	25	4
16639	2	4912599	4912628	3	10	30	TCA	24	3
16640	2	4912666	4912680	3	5	15	CAT	24	3
16641	2	4914642	4914677	2	18	36	CA	9	11
16646	2	4929838	4929881	2	22	44	AC	7	9
16648	2	4932435	4932464	2	15	30	CA	16	8
16651	2	4936668	4936681	2	7	14	CA	22	8
16653	2	4939169	4939192	2	12	24	GA	23	8
16660	2	4952622	4952641	2	10	20	TG	9	10
16661	2	4952844	4952867	4	6	24	AGAT	27	8
16664	2	4953638	4953651	2	7	14	TC	17	3
16675	2	4994147	4994180	2	17	34	CA	12	7
16679	2	5009393	5009410	2	9	18	GA	27	5
16680	2	5010420	5010431	2	6	12	GT	13	3
16687	2	5028474	5028493	2	10	20	AC	17	6
16697	2	5048483	5048496	2	7	14	GA	12	10

16700	2	5060188	5060203	4	4	16	GATG	28	3
16701	2	5063904	5063923	4	5	20	ATTT	23	3
16702	2	5064035	5064062	4	7	28	TTTA	14	7
16708	2	5078942	5078973	2	16	32	GT	19	8
16716	2	5087153	5087178	2	13	26	CA	17	13
16720	2	5087963	5087974	2	6	12	AG	13	3
16722	2	5088291	5088306	4	4	16	AAAG	18	4
16724	2	5089545	5089560	4	4	16	TTTC	12	4
16728	2	5097237	5097266	2	15	30	AC	18	8
16732	2	5106866	5106887	2	11	22	AG	23	5
16733	2	5109615	5109632	2	9	18	TG	10	8
16739	2	5124940	5124955	2	8	16	TG	27	5
16740	2	5135337	5135358	2	11	22	CA	6	7
16742	2	5143282	5143303	2	11	22	TG	22	8
16744	2	5153933	5153954	2	11	22	GT	18	5
16755	2	5195202	5195215	2	7	14	TG	25	7
16757	2	5201770	5201785	4	4	16	GATT	28	4
16767	2	5213482	5213501	2	10	20	CA	13	3
16769	2	5214819	5214834	2	8	16	CA	30	8
16776	2	5244544	5244564	3	7	21	TCA	22	6
16783	2	5269035	5269072	2	19	38	AC	15	14
16784	2	5275720	5275743	2	12	24	CA	15	4
16792	2	5285064	5285089	2	13	26	AC	20	8
16796	2	5287459	5287472	2	7	14	AC	14	9
16800	2	5288439	5288478	2	20	40	CT	6	10
16801	2	5288759	5288778	2	10	20	CT	18	7
16804	2	5289586	5289601	4	4	16	TTTA	16	5
16805	2	5289778	5289821	4	11	44	TTCT	16	10
16813	2	5294008	5294023	4	4	16	AAAC	15	4
16818	2	5299424	5299447	3	8	24	GGT	10	5
16827	2	5306743	5306772	3	10	30	GAG	8	7
16829	2	5309283	5309310	2	14	28	GT	11	9
16830	2	5309519	5309539	3	7	21	ACC	9	4
16839	2	5319182	5319201	2	10	20	GT	10	8
16855	2	5336147	5336168	2	11	22	TG	15	11
16867	2	5348117	5348144	2	14	28	CA	20	10
16870	2	5351385	5351412	2	14	28	TG	15	7
16872	2	5356095	5356116	2	11	22	GT	18	6
16874	2	5356307	5356322	2	8	16	GA	27	5
16875	2	5356735	5356754	2	10	20	CA	23	7
16877	2	5357650	5357669	2	10	20	TG	20	7
16883	2	5375530	5375541	2	6	12	GT	14	5
16890	2	5388464	5388481	3	6	18	ATA	11	3
16897	2	5393643	5393658	4	4	16	TTTC	17	7
16898	2	5393941	5393958	2	9	18	TG	22	16
16899	2	5393960	5393975	2	8	16	GT	19	10
16900	2	5394201	5394220	2	10	20	GA	11	7
16901	2	5394407	5394424	3	6	18	CCT	30	3
16904	2	5396374	5396397	3	8	24	CTC	24	9
16905	2	5397816	5397845	3	10	30	AAG	22	8
16906	2	5398215	5398236	2	11	22	GA	16	6

16908	2	5400312	5400343	4	8	32	ATCA	29	4
16912	2	5406105	5406136	2	16	32	GT	16	12
16917	2	5413223	5413237	3	5	15	AAT	19	5
16919	2	5414796	5414807	2	6	12	GT	33	3
16922	2	5421810	5421831	2	11	22	GT	8	5
16934	2	5447597	5447644	4	12	48	CTAT	19	11
16945	2	5475045	5475056	2	6	12	CA	19	6
16946	2	5475059	5475072	2	7	14	CA	17	7
16948	2	5480452	5480477	2	13	26	GT	16	7
16949	2	5480555	5480582	2	14	28	TG	13	6
16950	2	5480789	5480808	2	10	20	TG	8	10
16953	2	5481482	5481523	2	21	42	TG	10	13
16954	2	5482919	5482952	2	17	34	GT	15	13
16959	2	5484410	5484421	2	6	12	TG	26	3
16966	2	5498045	5498080	2	18	36	TC	21	11
16970	2	5516419	5516452	2	17	34	AC	13	6
16975	2	5519989	5520056	4	17	68	TGAT	19	14
16977	2	5533165	5533180	2	8	16	GT	26	6
16979	2	5535581	5535624	2	22	44	AC	8	9
16981	2	5543041	5543070	2	15	30	GT	19	6
16982	2	5543366	5543379	2	7	14	AC	27	6
16985	2	5550399	5550418	2	10	20	AC	9	4
16988	2	5561096	5561115	2	10	20	CA	20	10
16993	2	5576890	5576949	4	15	60	ATCT	9	8
16995	2	5586267	5586294	2	14	28	TG	14	12
16997	2	5591941	5591964	2	12	24	GT	19	11
17004	2	5616325	5616348	4	6	24	CACT	25	4
17016	2	5621336	5621351	2	8	16	GA	10	3
17017	2	5625005	5625022	2	9	18	AC	18	5
17019	2	5633262	5633279	2	9	18	GT	22	8
17022	2	5637611	5637632	2	11	22	TC	16	5
17024	2	5642718	5642739	2	11	22	GT	20	8
17025	2	5646278	5646295	2	9	18	GA	23	4
17028	2	5662341	5662358	3	6	18	AGG	17	4
17036	2	5683664	5683689	2	13	26	TG	19	9
17041	2	5698278	5698303	2	13	26	GT	9	12
17042	2	5699686	5699699	2	7	14	TG	27	3
17051	2	5753296	5753325	2	15	30	TG	18	6
17052	2	5753412	5753439	2	14	28	TG	16	8
17057	2	5768665	5768682	3	6	18	CTG	26	6
17060	2	5778951	5778998	2	24	48	TG	8	10
17061	2	5780785	5780800	2	8	16	AC	30	6
17062	2	5781212	5781225	2	7	14	TG	27	3
17065	2	5786014	5786025	2	6	12	GT	9	4
17066	2	5786028	5786039	2	6	12	GT	15	5
17067	2	5786042	5786053	2	6	12	GT	16	4
17068	2	5786208	5786234	3	9	27	AGA	19	8
17069	2	5786542	5786567	2	13	26	TG	14	8
17070	2	5787914	5787929	2	8	16	TG	16	5
17075	2	5794659	5794684	2	13	26	AC	13	6
17076	2	5794829	5794858	2	15	30	TG	18	15

17086	2	5806975	5806988	2	7	14	TG	30	4
17090	2	5817138	5817155	2	9	18	TG	29	6
17092	2	5826628	5826639	2	6	12	CA	19	3
17099	2	5863599	5863614	2	8	16	TG	25	4
17106	2	5872680	5872697	3	6	18	CTC	24	4
17107	2	5876677	5876712	3	12	36	CTC	15	8
17109	2	5879236	5879262	3	9	27	TTA	13	6
17112	2	5884738	5884755	3	6	18	CTC	22	3
17120	2	5928548	5928565	2	9	18	GT	22	3
17121	2	5929063	5929086	2	12	24	TG	18	7
17122	2	5929606	5929623	3	6	18	AGC	17	3
17130	2	5966688	5966719	2	16	32	GT	15	10
17134	2	5976037	5976051	3	5	15	CTG	30	3
17135	2	5976553	5976594	2	21	42	TG	7	7
17136	2	5978531	5978564	2	17	34	AG	13	6
17137	2	5978973	5978994	2	11	22	CA	18	7
17140	2	5982464	5982478	3	5	15	CGG	25	6
17142	2	5990262	5990293	2	16	32	TG	21	9
17147	2	6007262	6007273	2	6	12	CA	30	5
17149	2	6013650	6013665	4	4	16	GTGC	16	7
17150	2	6013706	6013719	2	7	14	GT	22	7
17153	2	6016740	6016771	2	16	32	CA	13	4
17154	2	6019781	6019808	2	14	28	CT	9	7
17155	2	6019836	6019859	4	6	24	TGTC	12	5
17156	2	6020668	6020681	2	7	14	CA	20	5
17157	2	6022754	6022769	2	8	16	TG	17	8
17158	2	6022772	6022785	2	7	14	TG	16	7
17160	2	6024470	6024484	3	5	15	TCA	30	5
17162	2	6025273	6025284	2	6	12	AC	16	5
17172	2	6045729	6045756	2	14	28	GT	17	12
17178	2	6047878	6047907	2	15	30	GA	19	7
17190	2	6065243	6065258	2	8	16	AC	21	3
17193	2	6067435	6067454	2	10	20	AC	15	9
17197	2	6073194	6073223	3	10	30	CCT	8	6
17203	2	6080555	6080582	2	14	28	GT	11	10
17204	2	6080960	6081011	2	26	52	TG	12	9
17208	2	6085461	6085488	2	14	28	AG	9	7
17210	2	6091039	6091082	2	22	44	GT	11	13
17212	2	6093052	6093077	2	13	26	AC	16	5
17213	2	6103957	6103988	2	16	32	TG	7	9
17216	2	6114238	6114249	2	6	12	AT	9	10
17218	2	6124446	6124461	2	8	16	CT	22	4
17221	2	6126115	6126132	3	6	18	GAT	14	3
17222	2	6126473	6126484	2	6	12	CA	25	5
17228	2	6138315	6138332	2	9	18	CA	22	9
17229	2	6140395	6140418	2	12	24	TG	13	5
17230	2	6140512	6140539	2	14	28	CA	15	5
17239	2	6157011	6157032	2	11	22	AC	22	11
17242	2	6158929	6158944	2	8	16	TG	27	10
17247	2	6162042	6162071	2	15	30	GT	11	9
17256	2	6166706	6166725	2	10	20	CA	12	8

17269	2	6175393	6175406	2	7	14	GT	10	4
17270	2	6175429	6175442	2	7	14	GT	22	4
17272	2	6177129	6177166	2	19	38	GT	15	9
17280	2	6181323	6181338	2	8	16	CA	25	3
17281	2	6183692	6183705	2	7	14	TG	21	5
17283	2	6186748	6186765	2	9	18	AG	15	4
17286	2	6190288	6190311	2	12	24	AG	16	10
17317	2	6223096	6223119	2	12	24	GT	7	4
17319	2	6224440	6224463	2	12	24	GA	9	5
17320	2	6224734	6224755	2	11	22	GT	15	7
17322	2	6226064	6226107	4	11	44	TTCT	12	9
17323	2	6226708	6226725	3	6	18	GGA	16	3
17338	2	6234555	6234586	2	16	32	TG	8	10
17340	2	6234957	6234990	2	17	34	CA	12	12
17341	2	6238252	6238283	2	16	32	CA	8	10
17343	2	6239601	6239620	2	10	20	GT	17	3
17344	2	6242021	6242034	2	7	14	AG	22	5
17354	2	6261219	6261246	2	14	28	GT	17	16
17358	2	6266475	6266489	3	5	15	GTG	15	4
17360	2	6270300	6270333	2	17	34	TC	6	6
17368	2	6274896	6274911	4	4	16	TTTG	18	10
17371	2	6282827	6282840	2	7	14	TA	7	3
17372	2	6284741	6284758	3	6	18	ATT	22	4
17373	2	6287502	6287553	2	26	52	CT	9	11
17374	2	6287597	6287614	2	9	18	CA	14	4
17376	2	6288523	6288537	3	5	15	GGT	26	3
17381	2	6295857	6295876	2	10	20	AC	20	4
17382	2	6297586	6297601	4	4	16	AAAT	30	5
17383	2	6299857	6299868	2	6	12	GT	21	3
17384	2	6300313	6300328	4	4	16	AAAT	11	3
17386	2	6302045	6302078	2	17	34	AC	9	8
17390	2	6308628	6308641	2	7	14	TG	23	4
17392	2	6312724	6312735	2	6	12	TC	31	5
17393	2	6314391	6314402	2	6	12	CA	13	6
17408	2	6329097	6329116	2	10	20	GA	15	4
17413	2	6333246	6333257	2	6	12	AC	6	5
17418	2	6341032	6341047	2	8	16	TG	20	4
17436	2	6376556	6376575	2	10	20	GA	15	9
17440	2	6388413	6388426	2	7	14	TA	18	3
17445	2	6404931	6404951	3	7	21	TGA	20	7
17456	2	6418109	6418124	4	4	16	ATGG	27	3
17458	2	6421494	6421507	2	7	14	CA	20	5
17459	2	6421798	6421809	2	6	12	CA	13	7
17462	2	6424794	6424820	3	9	27	TCA	26	8
17464	2	6425648	6425661	2	7	14	TA	15	12
17467	2	6439267	6439280	2	7	14	TA	19	3
17471	2	6448480	6448491	2	6	12	CA	25	4
17474	2	6456349	6456386	2	19	38	GT	9	7
17483	2	6471593	6471607	3	5	15	AGA	29	3
17485	2	6481317	6481344	2	14	28	GT	24	10
17490	2	6503611	6503642	4	8	32	AGAA	20	4

17491	2	6504494	6504505	2	6	12	TG	20	3
17498	2	6511704	6511715	2	6	12	AT	22	8
17500	2	6529414	6529435	2	11	22	TC	14	6
17505	2	6555911	6555925	3	5	15	ATT	28	3
17514	2	6581188	6581199	2	6	12	CA	17	3
17515	2	6581232	6581246	3	5	15	CCA	23	3
17517	2	6587411	6587430	2	10	20	GT	12	9
17518	2	6587675	6587700	2	13	26	GT	13	5
17519	2	6587752	6587781	3	10	30	AGG	13	5
17520	2	6587870	6587881	2	6	12	AC	18	3
17531	2	6591600	6591617	3	6	18	TGG	20	3
17533	2	6591988	6592023	2	18	36	GT	7	9
17535	2	6592128	6592159	2	16	32	GT	14	7
17536	2	6592317	6592332	4	4	16	GTGC	23	3
17539	2	6595369	6595380	2	6	12	TG	12	9
17540	2	6595676	6595689	2	7	14	AC	18	7
17544	2	6597126	6597161	2	18	36	GA	7	7
17545	2	6597501	6597515	3	5	15	TGC	9	4
17551	2	6598288	6598305	2	9	18	AC	16	7
17559	2	6604772	6604785	2	7	14	GT	21	3
17560	2	6606001	6606014	2	7	14	AC	27	5
17561	2	6606880	6606899	2	10	20	CA	14	10
17563	2	6607283	6607304	2	11	22	TG	16	9
17567	2	6609777	6609791	3	5	15	CCT	21	4
17569	2	6611991	6612006	2	8	16	GT	15	3
17571	2	6614285	6614300	2	8	16	GA	21	3
17572	2	6614566	6614595	2	15	30	TC	6	7
17576	2	6616297	6616320	2	12	24	TC	12	9
17578	2	6616546	6616560	3	5	15	CAA	30	3
17582	2	6619395	6619408	2	7	14	GT	25	6
17584	2	6619710	6619729	2	10	20	AC	17	8
17585	2	6620850	6620899	2	25	50	GT	14	11
17586	2	6621107	6621124	2	9	18	GT	20	4
17587	2	6621264	6621287	2	12	24	GT	9	7
17589	2	6623252	6623277	2	13	26	TG	11	7
17594	2	6635913	6635944	2	16	32	CA	11	6
17596	2	6638363	6638378	2	8	16	AC	27	3
17608	2	6644848	6644867	2	10	20	TG	14	3
17610	2	6645894	6645917	2	12	24	TG	11	10
17611	2	6646015	6646038	2	12	24	AG	8	8
17612	2	6647689	6647706	2	9	18	GT	25	8
17616	2	6650111	6650130	4	5	20	TTAT	26	3
17620	2	6655735	6655746	2	6	12	GT	25	4
17623	2	6660587	6660604	2	9	18	CA	25	10
17629	2	6662393	6662404	2	6	12	AC	13	5
17632	2	6662802	6662822	3	7	21	GAG	16	4
17633	2	6663076	6663087	2	6	12	TG	15	3
17634	2	6663342	6663373	2	16	32	AC	16	10
17635	2	6666385	6666399	3	5	15	TAT	25	4
17638	2	6668930	6668944	3	5	15	CCT	20	3
17652	2	6687529	6687556	2	14	28	CA	7	10

17653	2	6687613	6687639	3	9	27	CTT	11	7
17654	2	6688237	6688250	2	7	14	AC	24	5
17656	2	6693406	6693429	2	12	24	GT	11	6
17658	2	6699073	6699086	2	7	14	TG	19	5
17661	2	6702709	6702722	2	7	14	AC	23	4
17663	2	6708412	6708439	2	14	28	GT	12	10
17671	2	6713293	6713318	2	13	26	GT	21	11
17674	2	6717384	6717401	3	6	18	CCT	7	3
17676	2	6719285	6719310	2	13	26	CA	17	5
17683	2	6724055	6724069	3	5	15	GGC	22	4
17686	2	6724671	6724684	2	7	14	TG	20	5
17687	2	6724857	6724872	2	8	16	TG	25	4
17688	2	6725178	6725193	2	8	16	TC	17	10
17689	2	6725449	6725466	2	9	18	TC	16	9
17690	2	6725469	6725484	2	8	16	TC	21	11
17692	2	6726190	6726213	3	8	24	GAA	28	7
17696	2	6729806	6729829	2	12	24	TC	9	5
17698	2	6730037	6730058	2	11	22	AC	21	4
17699	2	6730302	6730321	2	10	20	TG	23	6
17700	2	6732522	6732565	2	22	44	TC	8	7
17701	2	6732963	6732986	2	12	24	CA	20	6
17703	2	6735457	6735468	2	6	12	TC	14	7
17704	2	6735471	6735484	2	7	14	TC	6	3
17709	2	6738378	6738419	2	21	42	CA	7	9
17710	2	6740007	6740024	2	9	18	CA	23	5
17713	2	6741456	6741483	2	14	28	AC	16	7
17719	2	6743980	6743993	2	7	14	TG	13	9
17722	2	6747140	6747159	2	10	20	GT	15	7
17734	2	6756088	6756117	2	15	30	TC	10	6
17737	2	6758481	6758498	2	9	18	CA	20	8
17740	2	6764322	6764335	2	7	14	AC	26	5
17741	2	6767826	6767839	2	7	14	AG	18	4
17743	2	6770149	6770160	2	6	12	TA	15	3
17744	2	6770717	6770734	2	9	18	CT	26	5
17745	2	6773357	6773370	2	7	14	TG	23	8
17746	2	6773557	6773574	2	9	18	GT	18	5
17747	2	6773602	6773615	2	7	14	TG	17	5
17748	2	6773763	6773778	2	8	16	GT	18	3
17749	2	6774334	6774355	2	11	22	GT	20	6
17750	2	6774510	6774541	2	16	32	TG	15	8
17757	2	6788544	6788555	2	6	12	CA	27	3
17758	2	6788558	6788569	2	6	12	CA	25	3
17762	2	6794539	6794554	2	8	16	TC	14	4
17774	2	6799988	6800005	2	9	18	AC	18	4
17775	2	6800187	6800218	2	16	32	AC	13	8
17776	2	6800615	6800652	2	19	38	AC	13	8
17785	2	6812758	6812781	2	12	24	TG	15	9
17787	2	6813886	6813927	2	21	42	AC	8	10
17788	2	6815387	6815400	2	7	14	CA	7	4
17791	2	6818917	6818950	2	17	34	CA	14	9
17792	2	6819334	6819355	2	11	22	AC	17	11

17793	2	6819892	6819905	2	7	14	GT	20	5
17794	2	6820047	6820076	2	15	30	TG	15	8
17795	2	6820849	6820878	2	15	30	GT	18	16
17797	2	6821556	6821593	2	19	38	AC	11	5
17798	2	6821864	6821885	2	11	22	CA	16	10
17802	2	6825372	6825397	2	13	26	CA	14	6
17803	2	6825452	6825465	2	7	14	CA	26	3
17804	2	6825998	6826043	2	23	46	CA	6	8
17810	2	6830519	6830548	2	15	30	GT	16	7
17815	2	6831440	6831467	2	14	28	CA	18	10
17817	2	6831742	6831769	2	14	28	AC	11	10
17819	2	6831821	6831846	2	13	26	CA	9	9
17821	2	6832449	6832470	2	11	22	AC	15	4
17823	2	6833871	6833906	2	18	36	GT	8	9
17826	2	6836198	6836219	2	11	22	AC	11	3
17833	2	6838918	6838939	2	11	22	AC	20	9
17834	2	6838981	6838998	3	6	18	GGT	32	3
17835	2	6839300	6839315	2	8	16	CA	14	3
17839	2	6841025	6841052	2	14	28	CA	15	10
17841	2	6841569	6841604	2	18	36	GT	7	8
17842	2	6841782	6841797	2	8	16	GT	6	4
17843	2	6841984	6842001	2	9	18	CT	7	5
17844	2	6842002	6842015	2	7	14	CA	7	3
17849	2	6843515	6843534	2	10	20	GA	11	11
17851	2	6844702	6844715	2	7	14	GA	11	6
17856	2	6846562	6846573	2	6	12	AG	26	8
17862	2	6848716	6848737	2	11	22	CA	24	10
17866	2	6851181	6851208	2	14	28	TG	8	9
17870	2	6855817	6855868	2	26	52	GT	6	10
17871	2	6856081	6856112	2	16	32	CA	15	10
17875	2	6863823	6863861	3	13	39	AGG	13	5
17881	2	6875365	6875398	2	17	34	GT	12	6
17883	2	6876554	6876585	2	16	32	TG	11	7
17885	2	6877289	6877314	2	13	26	TG	14	9
17889	2	6878795	6878822	2	14	28	AC	23	11
17892	2	6880394	6880420	3	9	27	CAT	26	5
17893	2	6880960	6880989	2	15	30	TG	15	10
17901	2	6889248	6889267	2	10	20	AG	16	8
17904	2	6894276	6894311	4	9	36	CTGC	13	6
17906	2	6904202	6904231	2	15	30	TG	9	6
17911	2	6915822	6915837	2	8	16	GT	20	3
17914	2	6918084	6918095	2	6	12	AC	18	7
17918	2	6924436	6924455	2	10	20	AC	21	7
17919	2	6924606	6924621	2	8	16	TG	16	4
17925	2	6928195	6928216	2	11	22	GT	10	3
17927	2	6932831	6932844	2	7	14	CA	19	10
17928	2	6932847	6932862	2	8	16	CA	11	9
17930	2	6937384	6937397	2	7	14	GT	25	3
17938	2	6943664	6943677	2	7	14	AG	17	3
17940	2	6944467	6944506	2	20	40	GT	13	9
17941	2	6944654	6944675	2	11	22	TG	6	9

17942	2	6944761	6944772	2	6	12	AC	27	4
17957	2	6970748	6970771	2	12	24	CT	17	8
17962	2	6975317	6975370	2	27	54	CA	9	7
17966	2	6978620	6978643	3	8	24	GAG	7	3
17970	2	6980383	6980398	4	4	16	CAGG	20	3
17972	2	6980466	6980483	2	9	18	AG	17	4
17973	2	6980853	6980878	2	13	26	AG	17	7
17982	2	6984370	6984383	2	7	14	AC	23	3
17987	2	6986687	6986718	2	16	32	TG	10	16
17993	2	6996081	6996104	2	12	24	AC	15	3
17995	2	6998102	6998121	2	10	20	AC	24	5
17996	2	6999182	6999201	2	10	20	CT	24	3
17999	2	7001069	7001084	2	8	16	CA	13	4
18000	2	7001119	7001138	4	5	20	CTTT	15	3
18001	2	7001477	7001488	2	6	12	GT	23	5
18002	2	7001670	7001683	2	7	14	TG	25	4
18003	2	7001798	7001831	2	17	34	CA	9	7
18005	2	7006714	7006743	2	15	30	AC	10	8
18008	2	7007745	7007762	2	9	18	CA	16	8
18009	2	7007855	7007868	2	7	14	AC	22	3
18011	2	7009424	7009449	2	13	26	CA	15	3
18013	2	7011978	7011995	2	9	18	TC	26	3
18016	2	7015163	7015184	2	11	22	AC	14	11
18017	2	7016044	7016055	2	6	12	AC	23	4
18019	2	7017739	7017756	2	9	18	TC	14	9
18020	2	7018328	7018363	4	9	36	TCTA	19	8
18023	2	7022084	7022113	2	15	30	AC	17	10
18024	2	7022234	7022255	2	11	22	CA	18	5
18025	2	7023027	7023050	3	8	24	GCT	14	6
18026	2	7023097	7023150	2	27	54	TG	6	11
18028	2	7025932	7025955	2	12	24	CA	19	5
18030	2	7027318	7027349	2	16	32	GT	22	6
18031	2	7028067	7028088	2	11	22	AC	9	8
18036	2	7030620	7030631	2	6	12	AC	28	3
18038	2	7030680	7030691	2	6	12	TG	21	4
18040	2	7031289	7031320	2	16	32	AG	8	6
18046	2	7036170	7036193	2	12	24	TC	23	7
18049	2	7041375	7041404	2	15	30	CA	7	12
18051	2	7041607	7041652	2	23	46	AC	6	9
18053	2	7043014	7043027	2	7	14	CA	13	5
18055	2	7043182	7043197	2	8	16	GT	14	7
18056	2	7043359	7043379	3	7	21	CAG	20	6
18060	2	7047161	7047174	2	7	14	TC	13	5
18069	2	7053645	7053684	2	20	40	AG	8	7
18071	2	7055629	7055643	3	5	15	TAA	31	3
18087	2	7080668	7080679	2	6	12	TG	23	6
18089	2	7082939	7082950	2	6	12	TG	29	4
18091	2	7085783	7085798	2	8	16	CT	20	4
18101	2	7097811	7097824	2	7	14	TG	11	5
18104	2	7098107	7098136	2	15	30	AC	8	11
18105	2	7102438	7102449	2	6	12	AC	19	3

18106	2	7102453	7102466	2	7	14	AC	18	3
18107	2	7102573	7102600	2	14	28	TC	14	12
18113	2	7111222	7111237	2	8	16	AC	28	4
18116	2	7125723	7125734	2	6	12	AC	24	3
18117	2	7126769	7126782	2	7	14	TG	24	3
18118	2	7126784	7126795	2	6	12	GT	31	3
18119	2	7126989	7127016	4	7	28	ATGG	29	5
18120	2	7127880	7127907	2	14	28	TG	16	10
18122	2	7128648	7128659	2	6	12	GT	28	4
18125	2	7132707	7132724	2	9	18	CT	21	4
18128	2	7135533	7135556	2	12	24	AG	20	7
18129	2	7135594	7135617	2	12	24	GA	22	6
18130	2	7137079	7137098	2	10	20	GT	16	7
18131	2	7141306	7141321	2	8	16	AC	26	4
18132	2	7143488	7143523	2	18	36	AC	20	9
18134	2	7143943	7143960	3	6	18	TCC	17	3
18142	2	7162204	7162217	2	7	14	TC	10	4
18143	2	7162220	7162233	2	7	14	TC	13	5
18145	2	7162455	7162470	2	8	16	TC	12	4
18146	2	7165047	7165066	2	10	20	CT	12	9
18148	2	7165736	7165765	2	15	30	TG	10	15
18149	2	7165892	7165913	2	11	22	TG	15	5
18150	2	7165937	7165950	2	7	14	GT	18	3
18156	2	7174761	7174772	2	6	12	CA	13	4
18158	2	7186406	7186433	2	14	28	AC	18	8
18162	2	7196569	7196582	2	7	14	AC	15	4
18164	2	7196779	7196804	2	13	26	GA	10	9
18165	2	7196888	7196911	2	12	24	TG	14	7
18168	2	7201464	7201485	2	11	22	CA	15	4
18172	2	7215437	7215454	2	9	18	CA	29	4
18173	2	7216895	7216908	2	7	14	GT	21	7
18174	2	7219149	7219166	2	9	18	CA	21	5
18175	2	7219386	7219401	2	8	16	TG	28	4
18176	2	7219473	7219490	2	9	18	AC	23	3
18177	2	7221673	7221684	2	6	12	GT	21	5
18178	2	7231287	7231310	4	6	24	TCTG	27	3
18183	2	7242805	7242834	2	15	30	GT	16	8
18187	2	7251186	7251197	2	6	12	AT	26	3
18191	2	7255812	7255833	2	11	22	CA	12	7
18193	2	7260162	7260173	2	6	12	TC	31	3
18195	2	7262272	7262305	2	17	34	AG	6	6
18196	2	7263616	7263645	2	15	30	CA	7	5
18212	2	7289326	7289345	2	10	20	AC	20	6
18214	2	7294259	7294288	2	15	30	GT	11	10
18215	2	7294667	7294692	2	13	26	TG	15	11
18221	2	7322006	7322029	2	12	24	AG	10	6
18225	2	7327070	7327101	4	8	32	TATG	9	7
18229	2	7335937	7335956	2	10	20	CA	13	9
18230	2	7339444	7339459	2	8	16	GT	23	10
18231	2	7341075	7341092	2	9	18	TG	25	4
18232	2	7358305	7358330	2	13	26	CA	8	5

18243	2	7373409	7373423	3	5	15	GCT	27	4
18248	2	7378077	7378096	2	10	20	CA	22	7
18250	2	7382186	7382209	2	12	24	AT	9	5
18252	2	7391178	7391197	2	10	20	AC	7	8
18253	2	7391237	7391260	3	8	24	AAT	9	8
18260	2	7404766	7404781	2	8	16	AC	17	8
18261	2	7423346	7423367	2	11	22	AC	14	8
18263	2	7425482	7425497	4	4	16	AATG	32	3
18267	2	7441615	7441632	2	9	18	CA	15	6
18269	2	7445056	7445081	2	13	26	CA	15	16
18272	2	7449228	7449255	4	7	28	TTTG	18	6
18273	2	7452783	7452800	2	9	18	AC	13	8
18274	2	7454835	7454854	2	10	20	TG	18	5
18277	2	7462812	7462827	2	8	16	AC	15	9
18278	2	7462829	7462848	2	10	20	CA	19	16
18283	2	7464173	7464188	2	8	16	AC	9	4
18285	2	7466112	7466159	2	24	48	CA	8	7
18291	2	7473391	7473430	2	20	40	AC	12	11
18293	2	7477861	7477882	2	11	22	GT	19	5
18295	2	7480544	7480555	2	6	12	AT	20	3
18296	2	7481998	7482017	4	5	20	ATGG	30	4
18300	2	7491868	7491895	2	14	28	AC	11	5
18307	2	7500403	7500414	2	6	12	CA	30	3
18308	2	7502423	7502437	3	5	15	GCT	28	4
18315	2	7519399	7519412	2	7	14	AT	8	6
18317	2	7523635	7523660	2	13	26	AC	19	11
18318	2	7523856	7523873	2	9	18	CA	16	9
18321	2	7527913	7527928	2	8	16	AC	19	11
18323	2	7540347	7540386	2	20	40	TG	6	5
18326	2	7542398	7542411	2	7	14	AC	28	4
18327	2	7543423	7543438	2	8	16	TG	12	3
18332	2	7549578	7549611	2	17	34	GT	10	9
18333	2	7549831	7549845	3	5	15	GCT	33	3
18338	2	7568155	7568182	2	14	28	GT	13	7
18350	2	7580019	7580044	2	13	26	AC	13	6
18351	2	7582251	7582282	2	16	32	AC	16	8
18387	2	7619045	7619068	2	12	24	CT	7	9
18391	2	7627727	7627742	2	8	16	AC	22	6
18401	2	7657544	7657564	3	7	21	CTA	21	5
18406	2	7664719	7664752	2	17	34	AC	14	10
18413	2	7673817	7673831	3	5	15	TGC	23	3
18418	2	7686596	7686609	2	7	14	TG	14	5
18421	2	7695493	7695512	4	5	20	AAAT	18	3
18426	2	7708342	7708357	2	8	16	GT	19	5
18427	2	7710168	7710185	3	6	18	AAT	7	3
18445	2	7748642	7748679	2	19	38	AC	11	11
18446	2	7750028	7750055	2	14	28	CA	11	7
18447	2	7754766	7754811	2	23	46	GT	8	12
18449	2	7755508	7755523	2	8	16	GT	15	3
18452	2	7763296	7763333	2	19	38	AC	7	8
18458	2	7777937	7777960	2	12	24	CA	26	10

18460	2	7784954	7784993	2	20	40	AC	9	9
18463	2	7788752	7788791	2	20	40	AC	8	8
18464	2	7796154	7796177	2	12	24	GT	18	11
18471	2	7813241	7813286	2	23	46	CA	7	10
18472	2	7814898	7814912	3	5	15	ATA	10	3
18473	2	7816962	7816999	2	19	38	AC	14	12
18476	2	7819037	7819072	2	18	36	GT	6	7
18477	2	7819261	7819276	4	4	16	CACG	6	5
18481	2	7827708	7827723	2	8	16	AG	15	5
18484	2	7830937	7830960	3	8	24	TCA	19	8
18488	2	7832765	7832776	2	6	12	AG	29	4
18491	2	7839897	7839914	2	9	18	AC	26	3
18492	2	7840953	7840968	4	4	16	AAAC	21	4
18519	2	7877179	7877220	2	21	42	AC	15	11
18530	2	7906937	7906948	2	6	12	GT	18	6
18531	2	7906951	7906964	2	7	14	GT	16	4
18534	2	7909852	7909863	2	6	12	CA	29	3
18535	2	7909988	7910025	2	19	38	AC	13	8
18537	2	7914738	7914763	2	13	26	CT	14	12
18539	2	7915866	7915883	2	9	18	CA	23	7
18546	2	7919522	7919539	2	9	18	GT	22	11
18547	2	7919728	7919741	2	7	14	AG	15	3
18550	2	7923739	7923770	2	16	32	AC	8	11
18552	2	7925776	7925787	2	6	12	CA	19	4
18557	2	7926812	7926823	2	6	12	TG	14	3
18558	2	7929980	7929993	2	7	14	AC	29	3
18566	2	7934822	7934833	2	6	12	AC	21	4
18567	2	7935280	7935291	2	6	12	GT	23	5
18571	2	7938098	7938125	2	14	28	AC	11	11
18582	2	7956684	7956701	3	6	18	CAA	24	4
18583	2	7957333	7957362	3	10	30	GCA	15	9
18585	2	7961889	7961904	2	8	16	AC	28	4
18586	2	7962537	7962562	2	13	26	CA	8	5
18589	2	7965561	7965592	2	16	32	CA	11	6
18597	2	7971253	7971270	2	9	18	TG	24	7
18602	2	7975184	7975199	2	8	16	TG	21	6
18603	2	7978551	7978588	2	19	38	TG	9	9
18604	2	7978694	7978727	2	17	34	CA	11	10
18605	2	7978761	7978774	2	7	14	CA	25	3
18607	2	7980690	7980711	2	11	22	AC	17	3
18617	2	7993786	7993835	2	25	50	TG	13	15
18618	2	7993930	7993951	2	11	22	CA	15	7
18628	2	8009086	8009139	2	27	54	TG	6	6
18630	2	8009713	8009726	2	7	14	CA	22	12
18645	2	8019508	8019543	2	18	36	GT	10	10
18651	2	8025867	8025882	2	8	16	AT	7	4
18652	2	8026698	8026717	4	5	20	GACA	28	3
18653	2	8026811	8026834	2	12	24	AC	9	6
18655	2	8027279	8027330	4	13	52	TTTC	26	11
18657	2	8028152	8028189	2	19	38	AC	7	10
18660	2	8031655	8031672	2	9	18	CT	16	5

18663	2	8032890	8032913	4	6	24	AACA	6	3
18670	2	8036880	8036917	2	19	38	GT	11	9
18675	2	8045378	8045403	2	13	26	CA	15	15
18677	2	8050405	8050446	3	14	42	CAT	12	6
18684	2	8061678	8061723	2	23	46	CA	7	11
18685	2	8062845	8062866	2	11	22	AC	16	15
18686	2	8065017	8065036	2	10	20	TG	24	12
18687	2	8066412	8066437	2	13	26	GT	13	7
18688	2	8067101	8067121	3	7	21	ACA	28	3
18690	2	8069307	8069322	2	8	16	TG	19	4
18692	2	8071792	8071806	3	5	15	CAG	31	3
18697	2	8095839	8095868	2	15	30	TG	18	6
18699	2	8102939	8102954	2	8	16	TG	28	7
18703	2	8104907	8104922	2	8	16	CA	24	6
18704	2	8106090	8106105	2	8	16	TG	26	4
18705	2	8106222	8106241	2	10	20	CA	18	12
18710	2	8119681	8119694	2	7	14	AC	20	3
18714	2	8124865	8124882	3	6	18	TAT	13	4
18716	2	8127849	8127869	3	7	21	TTG	26	5
18719	2	8128249	8128270	2	11	22	CA	9	7
18720	2	8130310	8130341	2	16	32	TG	8	9
18721	2	8130822	8130839	2	9	18	GT	19	6
18725	2	8143847	8143864	2	9	18	GT	13	5
18726	2	8144982	8144993	2	6	12	AG	32	3
18727	2	8146753	8146802	2	25	50	CA	6	9
18728	2	8146966	8146977	2	6	12	TG	18	4
18729	2	8146980	8146995	2	8	16	TG	21	6
18735	2	8150186	8150201	2	8	16	GT	13	9
18736	2	8150204	8150219	2	8	16	GT	14	9
18737	2	8150300	8150317	2	9	18	TG	18	5
18742	2	8161053	8161068	2	8	16	AC	19	5
18745	2	8165785	8165802	3	6	18	ATA	23	4
18746	2	8167228	8167245	2	9	18	GT	16	7
18754	2	8169858	8169875	3	6	18	AGG	15	4
18755	2	8171123	8171158	3	12	36	GAG	8	6
18757	2	8177196	8177209	2	7	14	GT	22	5
18761	2	8180879	8180928	2	25	50	CA	11	10
18767	2	8192602	8192615	2	7	14	AC	17	6
18768	2	8192649	8192668	2	10	20	CA	12	8
18770	2	8193571	8193598	2	14	28	CT	18	7
18771	2	8193729	8193764	4	9	36	ACAG	23	5
18772	2	8195747	8195764	2	9	18	CA	19	7
18777	2	8202332	8202349	2	9	18	AC	24	4
18783	2	8205362	8205389	4	7	28	TGTA	8	3
18785	2	8205590	8205619	2	15	30	AC	12	6
18786	2	8206067	8206090	4	6	24	TGGA	27	3
18790	2	8212657	8212674	2	9	18	AC	26	11
18791	2	8215466	8215509	2	22	44	GT	6	6
18792	2	8215702	8215741	2	20	40	TG	15	10
18798	2	8229516	8229527	2	6	12	GT	14	5
18800	2	8229734	8229751	2	9	18	CA	15	4

18801	2	8229911	8229934	2	12	24	CA	12	7
18802	2	8231692	8231737	2	23	46	GA	7	8
18809	2	8240791	8240802	2	6	12	AC	23	5
18813	2	8245549	8245614	2	33	66	CT	8	12
18815	2	8248128	8248139	2	6	12	AT	13	6
18819	2	8256822	8256849	2	14	28	TG	7	6
18820	2	8256979	8257012	2	17	34	CA	10	6
18822	2	8257347	8257370	2	12	24	CA	14	3
18825	2	8258819	8258840	2	11	22	CA	12	6
18826	2	8258935	8258972	2	19	38	AC	10	7
18830	2	8259362	8259379	2	9	18	CA	16	12
18837	2	8280418	8280429	2	6	12	AG	27	3
18838	2	8280500	8280523	2	12	24	TG	15	11
18839	2	8282851	8282866	2	8	16	GT	17	7
18840	2	8293217	8293252	2	18	36	AC	8	9
18845	2	8299617	8299648	2	16	32	AC	14	10
18848	2	8303639	8303653	3	5	15	CAT	22	3
18854	2	8310785	8310808	2	12	24	CA	10	4
18855	2	8310845	8310872	2	14	28	CA	11	9
18861	2	8317607	8317628	2	11	22	CA	19	6
18865	2	8321161	8321175	3	5	15	GCA	25	3
18866	2	8322449	8322478	2	15	30	AC	16	8
18867	2	8323359	8323398	2	20	40	CA	11	9
18874	2	8334954	8334973	2	10	20	GT	22	9
18876	2	8336491	8336524	2	17	34	CA	17	12
18877	2	8337399	8337410	2	6	12	GT	16	15
18878	2	8337412	8337427	2	8	16	TG	6	5
18879	2	8337688	8337705	3	6	18	AAT	13	7
18880	2	8341928	8341941	2	7	14	TC	25	4
18884	2	8346723	8346742	2	10	20	CA	25	6
18885	2	8347117	8347132	2	8	16	GT	15	8
18886	2	8348876	8348893	3	6	18	TCC	15	4
18890	2	8357588	8357602	3	5	15	AGG	26	6
18894	2	8363919	8363966	2	24	48	GT	9	9
18896	2	8365572	8365589	2	9	18	CA	20	8
18898	2	8365989	8366008	2	10	20	AC	8	5
18905	2	8373656	8373667	2	6	12	CT	17	4
18910	2	8378541	8378562	2	11	22	CA	26	15
18915	2	8397869	8397882	2	7	14	CT	10	6
18916	2	8401599	8401614	4	4	16	GTGC	18	4
18917	2	8417057	8417074	2	9	18	TC	17	6
18919	2	8418404	8418419	2	8	16	AC	24	3
18920	2	8418421	8418434	2	7	14	CA	26	4
18922	2	8425454	8425465	2	6	12	AC	26	4
18923	2	8426055	8426070	2	8	16	TG	23	4
18925	2	8441125	8441139	3	5	15	GTT	29	3
18929	2	8470733	8470748	4	4	16	AGGC	7	3
18930	2	8471227	8471258	2	16	32	TG	15	10
18933	2	8476728	8476741	2	7	14	TG	30	5
18934	2	8482420	8482443	2	12	24	TG	17	7
18941	2	8483168	8483183	2	8	16	CA	21	5

18945	2	8485247	8485260	2	7	14	GT	20	4
18946	2	8485684	8485701	2	9	18	GT	19	8
18947	2	8489700	8489713	2	7	14	GT	20	4
18949	2	8497528	8497557	2	15	30	CA	16	14
18956	2	8518106	8518123	2	9	18	AC	15	3
18959	2	8521842	8521859	2	9	18	TC	16	4
18960	2	8522338	8522369	4	8	32	ATGT	19	4
18969	2	8536388	8536411	2	12	24	GA	18	8
18971	2	8541054	8541073	4	5	20	CTGT	22	3
18975	2	8543935	8543972	2	19	38	CA	14	12
18978	2	8557039	8557050	2	6	12	TG	30	4
18984	2	8560748	8560769	2	11	22	GT	25	14
18998	2	8583796	8583823	2	14	28	TG	10	4
19007	2	8594169	8594186	2	9	18	CA	18	7
19017	2	8597623	8597652	2	15	30	GT	16	9
19020	2	8598746	8598767	2	11	22	GT	12	8
19021	2	8599389	8599400	2	6	12	GT	32	3
19023	2	8608451	8608464	2	7	14	TG	19	3
19030	2	8635315	8635336	2	11	22	GT	15	8
19035	2	8648299	8648310	2	6	12	TG	17	6
19036	2	8648312	8648327	2	8	16	GT	21	8
19038	2	8657915	8657926	2	6	12	AC	31	3
19050	2	8689484	8689505	2	11	22	GA	15	6
19051	2	8689604	8689655	2	26	52	TG	10	13
19052	2	8690288	8690319	4	8	32	ATGT	17	7
19053	2	8696989	8697004	2	8	16	TG	23	3
19058	2	8721123	8721162	2	20	40	TG	14	11
19060	2	8724127	8724140	2	7	14	GT	16	4
19075	2	8763966	8764013	2	24	48	CA	13	12
19077	2	8774969	8775000	2	16	32	TC	7	6
19088	2	8804422	8804445	2	12	24	AC	18	7
19090	2	8808817	8808830	2	7	14	GA	28	4
19091	2	8809194	8809205	2	6	12	TG	26	7
19092	2	8809208	8809221	2	7	14	TG	19	6
19094	2	8809779	8809790	2	6	12	AG	29	3
19095	2	8809831	8809850	2	10	20	TC	22	4
19108	2	8841743	8841781	3	13	39	TGT	22	11
19109	2	8842358	8842372	3	5	15	GTT	24	4
19110	2	8842564	8842579	4	4	16	TCCA	24	3
19114	2	8855385	8855411	3	9	27	TAT	13	3
19116	2	8861374	8861419	2	23	46	TG	8	12
19117	2	8863348	8863361	2	7	14	AT	23	3
19120	2	8881798	8881811	2	7	14	AC	28	4
19121	2	8882770	8882789	4	5	20	GTGC	32	5
19129	2	8890878	8890901	3	8	24	CTT	24	4
19135	2	8898257	8898272	2	8	16	TG	18	3
19144	2	8905559	8905573	3	5	15	CCT	24	3
19152	2	8922014	8922025	2	6	12	TG	22	4
19171	2	8948040	8948051	2	6	12	GT	21	3
19172	2	8948226	8948239	2	7	14	CA	28	3
19173	2	8948566	8948577	2	6	12	TG	26	3

19176	2	8962419	8962456	2	19	38	CA	16	13
19180	2	8965185	8965200	4	4	16	GTGC	25	10
19181	2	8965535	8965550	2	8	16	CA	15	11
19183	2	8968071	8968084	2	7	14	GT	21	3
19185	2	8968171	8968214	2	22	44	TG	9	10
19186	2	8972298	8972309	2	6	12	GT	29	3
19189	2	8972538	8972555	3	6	18	CCT	18	3
19195	2	8979416	8979427	2	6	12	TG	28	3
19197	2	8981095	8981122	2	14	28	CA	17	9
19199	2	8984892	8984907	2	8	16	GT	22	3
19202	2	8985807	8985842	2	18	36	TG	15	9
19206	2	8988966	8988985	2	10	20	AC	21	8
19207	2	8989006	8989019	2	7	14	CA	24	3
19208	2	8991229	8991258	2	15	30	CT	15	9
19213	2	8994132	8994157	2	13	26	AC	11	6
19216	2	8996747	8996766	2	10	20	CA	9	5
19217	2	8997014	8997031	3	6	18	ACC	12	3
19219	2	8997090	8997105	2	8	16	AC	16	3
19220	2	8997479	8997502	3	8	24	CTC	16	3
19222	2	8997664	8997703	2	20	40	GA	7	8
19223	2	8997891	8997917	3	9	27	CCT	9	4
19228	2	9001442	9001453	2	6	12	TG	18	3
19234	2	9002465	9002490	2	13	26	GT	14	6
19235	2	9002615	9002638	2	12	24	TC	8	5
19240	2	9005181	9005196	2	8	16	AC	23	11
19241	2	9005255	9005278	4	6	24	CTTC	24	3
19242	2	9005411	9005422	2	6	12	GT	22	5
19243	2	9005427	9005440	2	7	14	GT	23	3
19249	2	9010644	9010655	2	6	12	CA	6	4
19250	2	9010746	9010763	2	9	18	CA	18	10
19251	2	9011036	9011049	2	7	14	TG	23	4
19252	2	9011696	9011723	2	14	28	TG	20	7
19257	2	9015061	9015075	3	5	15	CAG	24	4
19259	2	9016185	9016208	2	12	24	GT	16	11
19263	2	9022645	9022656	2	6	12	CA	20	9
19265	2	9023335	9023349	3	5	15	GGA	16	3
19271	2	9029836	9029847	2	6	12	AT	16	3
19272	2	9030552	9030577	2	13	26	TG	15	5
19273	2	9030686	9030703	2	9	18	TG	18	4
19276	2	9035322	9035336	3	5	15	AGG	27	4
19277	2	9037689	9037734	2	23	46	CA	8	6
19286	2	9045707	9045740	2	17	34	TC	12	12
19294	2	9052264	9052275	2	6	12	AG	18	7
19297	2	9055664	9055683	2	10	20	AG	19	5
19298	2	9058141	9058156	4	4	16	TTCT	15	4
19300	2	9059674	9059691	3	6	18	AAT	12	3
19301	2	9061780	9061799	2	10	20	GT	16	5
19304	2	9062622	9062645	2	12	24	TG	9	10
19305	2	9063070	9063083	2	7	14	AG	19	3
19306	2	9063343	9063372	2	15	30	TG	19	9
19307	2	9063439	9063452	2	7	14	GT	19	5

19310	2	9070340	9070365	2	13	26	GA	9	8
19314	2	9077706	9077723	2	9	18	CA	21	9
19315	2	9079980	9080013	2	17	34	TG	7	8
19317	2	9080137	9080154	2	9	18	CA	22	10
19318	2	9081121	9081142	2	11	22	CA	19	8
19320	2	9081256	9081273	2	9	18	TG	8	5
19321	2	9082499	9082518	2	10	20	TG	16	6
19325	2	9085515	9085526	2	6	12	GA	17	5
19326	2	9085566	9085579	2	7	14	GA	13	3
19328	2	9089526	9089541	2	8	16	CA	16	4
19329	2	9091422	9091459	2	19	38	TG	6	6
19330	2	9091582	9091619	2	19	38	CA	7	4
19338	2	9095872	9095911	2	20	40	TG	10	10
19339	2	9097265	9097284	2	10	20	TG	23	12
19342	2	9104727	9104742	2	8	16	TG	13	8
19343	2	9105070	9105099	2	15	30	GT	27	9
19347	2	9110645	9110670	2	13	26	CT	16	3
19352	2	9122647	9122662	2	8	16	TA	12	5
19359	2	9127169	9127184	2	8	16	AC	21	3
19364	2	9129455	9129474	2	10	20	GT	23	4
19366	2	9131352	9131373	2	11	22	GT	10	5
19367	2	9131570	9131599	2	15	30	TG	21	7
19368	2	9131716	9131735	2	10	20	TG	18	7
19376	2	9139449	9139470	2	11	22	AC	15	6
19379	2	9154300	9154345	2	23	46	CA	7	8
19381	2	9167032	9167047	2	8	16	AC	16	5
19382	2	9167493	9167506	2	7	14	TG	26	3
19388	2	9180349	9180396	2	24	48	TG	8	9
19389	2	9181596	9181617	2	11	22	CA	22	7
19394	2	9197090	9197104	3	5	15	CTG	29	3
19395	2	9200729	9200744	2	8	16	AC	14	7
19396	2	9201281	9201294	2	7	14	GT	29	3
19401	2	9205836	9205862	3	9	27	AAT	12	5
19406	2	9213731	9213752	2	11	22	GT	21	8
19413	2	9221594	9221609	4	4	16	GCAT	27	3
19421	2	9224239	9224254	2	8	16	CA	9	3
19427	2	9225416	9225437	2	11	22	GA	11	8
19429	2	9225864	9225883	2	10	20	GT	19	5
19431	2	9227720	9227753	2	17	34	AC	13	12
19439	2	9234537	9234576	2	20	40	GT	15	10
19440	2	9234749	9234776	2	14	28	AC	12	6
19443	2	9235257	9235282	2	13	26	CA	13	7
19445	2	9241221	9241240	2	10	20	CA	14	5
19449	2	9245951	9245976	2	13	26	CA	10	9
19457	2	9249991	9250002	2	6	12	CA	25	3
19463	2	9257086	9257107	2	11	22	CA	22	8
19464	2	9257969	9258016	2	24	48	TG	7	9
19465	2	9258154	9258165	2	6	12	AC	27	3
19467	2	9261198	9261219	2	11	22	GT	22	9
19468	2	9261729	9261762	2	17	34	CA	9	6
19470	2	9266018	9266029	2	6	12	GT	19	3

19471	2	9267022	9267049	4	7	28	TTTA	12	9
19474	2	9270642	9270661	2	10	20	GT	14	5
19476	2	9277777	9277790	2	7	14	AC	29	5
19480	2	9278585	9278622	2	19	38	GT	11	14
19481	2	9280208	9280231	2	12	24	TG	23	6
19482	2	9281574	9281611	2	19	38	GA	6	9
19483	2	9285117	9285130	2	7	14	AC	19	3
19486	2	9292670	9292703	2	17	34	TG	10	7
19488	2	9292890	9292913	2	12	24	AC	22	4
19499	2	9303422	9303469	2	24	48	TG	7	7
19500	2	9306502	9306513	2	6	12	AT	10	4
19504	2	9319369	9319394	2	13	26	GT	10	11
19508	2	9326078	9326101	2	12	24	AC	20	5
19517	2	9337081	9337104	2	12	24	GT	16	3
19527	2	9342913	9342950	2	19	38	TG	6	5
19529	2	9343155	9343194	2	20	40	TG	6	7
19530	2	9343314	9343345	2	16	32	GT	9	6
19533	2	9346344	9346357	2	7	14	GT	6	3
19534	2	9346359	9346376	2	9	18	TG	13	8
19546	2	9394837	9394848	2	6	12	CA	27	3
19559	2	9419066	9419093	2	14	28	TC	17	6
19571	2	9441287	9441308	2	11	22	AC	13	3
19572	2	9445448	9445477	2	15	30	TG	12	8
19586	2	9469504	9469515	2	6	12	TG	13	3
19590	2	9497499	9497532	2	17	34	TG	9	7
19594	2	9507377	9507427	3	17	51	TAC	6	9
19597	2	9518071	9518098	2	14	28	TG	10	8
19601	2	9543266	9543287	2	11	22	CA	19	14
19604	2	9544719	9544734	4	4	16	CAGG	17	10
19609	2	9567995	9568028	2	17	34	AC	12	7
19610	2	9570965	9570980	2	8	16	TG	27	4
19612	2	9571279	9571294	2	8	16	GT	9	5
19613	2	9573244	9573287	2	22	44	GT	12	14
19617	2	9594077	9594088	2	6	12	AC	29	4
19619	2	9607131	9607150	2	10	20	GT	18	7
19621	2	9612566	9612595	3	10	30	CAA	23	7
19626	2	9626316	9626330	3	5	15	GGT	23	3
19627	2	9628338	9628349	2	6	12	AC	15	3
19633	2	9641289	9641310	2	11	22	CA	16	12
19639	2	9651400	9651414	3	5	15	GCT	23	5
19642	2	9657904	9657915	2	6	12	TA	16	5
19643	2	9658947	9658960	2	7	14	TG	14	7
19648	2	9668744	9668765	2	11	22	CA	11	11
19653	2	9676248	9676265	3	6	18	GAA	27	4
19656	2	9682342	9682353	2	6	12	TC	21	3
19658	2	9682866	9682885	2	10	20	AC	6	6
19666	2	9697659	9697688	2	15	30	TG	12	9
19673	2	9709490	9709503	2	7	14	AG	27	3
19674	2	9713583	9713614	2	16	32	AG	9	7
19676	2	9715244	9715259	2	8	16	TG	24	3
19679	2	9721402	9721425	2	12	24	TG	23	8

19683	2	9729796	9729827	2	16	32	TC	23	8
19684	2	9730212	9730225	2	7	14	TG	23	3
19688	2	9738334	9738351	2	9	18	CA	11	7
19689	2	9739931	9739954	3	8	24	CTG	32	7
19690	2	9742296	9742307	2	6	12	AC	25	4
19695	2	9746360	9746380	3	7	21	CCT	32	7
19704	2	9764541	9764560	2	10	20	AC	27	4
19706	2	9766937	9766990	2	27	54	TG	6	9
19707	2	9769506	9769517	2	6	12	GT	20	4
19708	2	9771349	9771366	2	9	18	CA	31	5
19710	2	9775583	9775606	2	12	24	TG	25	3
19711	2	9776319	9776330	2	6	12	TC	17	6
19712	2	9776332	9776343	2	6	12	CT	20	6
19714	2	9776508	9776521	2	7	14	CA	27	5
19720	2	9784480	9784491	2	6	12	GT	18	3
19723	2	9789104	9789135	4	8	32	CCAT	27	8
19726	2	9791087	9791110	2	12	24	AC	11	11
19729	2	9796039	9796053	3	5	15	TCT	21	3
19730	2	9796227	9796242	2	8	16	GT	11	9
19731	2	9796568	9796581	2	7	14	TG	33	6
19733	2	9798071	9798090	2	10	20	AC	24	4
19734	2	9798239	9798256	2	9	18	TG	26	6
19737	2	9801005	9801018	2	7	14	TG	17	5
19739	2	9803113	9803140	2	14	28	GT	14	15
19741	2	9804792	9804811	2	10	20	GT	7	4
19745	2	9809112	9809133	2	11	22	CA	16	6
19746	2	9809654	9809675	2	11	22	CA	21	7
19748	2	9813036	9813083	2	24	48	CT	8	12
19751	2	9817162	9817217	4	14	56	AGAA	17	14
19755	2	9821087	9821098	2	6	12	AC	19	10
19758	2	9821236	9821263	2	14	28	AC	11	10
19760	2	9821984	9822005	2	11	22	AC	23	5
19765	2	9827743	9827758	2	8	16	TC	17	10
19766	2	9827759	9827776	2	9	18	AC	18	9
19767	2	9828208	9828240	3	11	33	AGG	12	7
19771	2	9834860	9834891	2	16	32	TG	14	9
19773	2	9842950	9842979	2	15	30	CA	6	8
19774	2	9843081	9843094	2	7	14	CT	15	4
19776	2	9845376	9845387	2	6	12	TG	21	3
19777	2	9846558	9846575	2	9	18	AC	15	4
19779	2	9849892	9849915	2	12	24	GT	16	3
19784	2	9858668	9858693	2	13	26	CA	16	10
19788	2	9861631	9861648	2	9	18	GT	7	6
19789	2	9862394	9862411	2	9	18	TG	13	12
19798	2	9880284	9880297	2	7	14	TC	23	4
19814	2	9910768	9910791	4	6	24	TTCC	10	5
19815	2	9912193	9912214	2	11	22	AC	11	8
19817	2	9915762	9915783	2	11	22	TC	17	11
19818	2	9919490	9919505	4	4	16	GACT	30	4
19823	2	9921706	9921731	2	13	26	CA	7	6
19829	2	9928288	9928301	2	7	14	AC	23	5

19830	2	9928467	9928482	2	8	16	TG	22	3
19835	2	9935392	9935411	2	10	20	CA	24	5
19842	2	9940956	9940983	2	14	28	GA	12	14
19844	2	9941359	9941374	2	8	16	AC	20	9
19849	2	9942461	9942480	2	10	20	AG	6	3
19856	2	9945098	9945133	4	9	36	TTCC	23	9
19866	2	9949547	9949564	3	6	18	GCT	25	5
19867	2	9950710	9950721	2	6	12	TA	25	7
19877	2	9953301	9953352	4	13	52	AAAG	11	8
19878	2	9953581	9953606	2	13	26	GT	7	8
19879	2	9954030	9954044	3	5	15	AAT	12	3
19881	2	9954563	9954576	2	7	14	GT	12	3
19886	2	9955529	9955568	2	20	40	TG	19	14
19889	2	9959257	9959268	2	6	12	GA	15	5
19895	2	9964281	9964298	2	9	18	GT	12	7
19897	2	9967234	9967249	2	8	16	TG	17	6
19901	2	9968494	9968529	4	9	36	ATCC	18	7
19906	2	9973503	9973524	2	11	22	CA	8	8
19910	2	9978630	9978641	2	6	12	CA	17	4
19911	2	9978643	9978658	2	8	16	AC	15	5
19913	2	9980873	9980904	2	16	32	CA	9	8
19914	2	9981452	9981475	2	12	24	CA	18	8
19918	2	9986193	9986208	2	8	16	CA	22	4
19919	2	9986352	9986365	2	7	14	CA	23	3
19921	2	9987665	9987704	2	20	40	GA	8	6
19923	2	9989719	9989730	2	6	12	CA	25	3
19925	2	9990085	9990112	2	14	28	CA	18	18
19926	2	9992104	9992121	2	9	18	CT	19	9
19930	2	9992466	9992509	2	22	44	GT	7	11
19931	2	9992813	9992830	2	9	18	CA	22	8
19933	2	9993424	9993441	2	9	18	TG	15	8
19934	2	9993585	9993596	2	6	12	GT	20	8
19939	2	10001296	10001307	2	6	12	GA	24	3
19953	2	10018302	10018325	3	8	24	GAG	10	3
19957	2	10021386	10021399	2	7	14	GT	30	5
19958	2	10021457	10021470	2	7	14	CA	17	3
19964	2	10034651	10034670	4	5	20	AAAC	7	3
19967	2	10036245	10036271	3	9	27	AGC	20	8
19970	2	10038127	10038144	2	9	18	GA	16	6
19971	2	10038334	10038353	2	10	20	GA	6	3
19972	2	10038702	10038721	2	10	20	CT	21	5
19975	2	10041064	10041081	2	9	18	AC	22	7
19976	2	10041503	10041520	2	9	18	GT	20	15
19984	2	10050679	10050694	2	8	16	TC	22	4
19985	2	10051502	10051517	2	8	16	CA	16	11
19986	2	10051565	10051596	2	16	32	AC	10	8
19987	2	10051848	10051865	2	9	18	TG	18	6
19988	2	10051961	10051978	2	9	18	GT	15	8
19989	2	10054016	10054043	2	14	28	GT	11	9
19993	2	10057239	10057260	2	11	22	CA	10	11
19997	2	10066902	10066935	2	17	34	GA	14	8

20005	2	10074002	10074043	2	21	42	CA	19	12
20007	2	10075718	10075745	2	14	28	GA	19	4
20009	2	10082646	10082671	2	13	26	GA	6	5
20020	2	10101831	10101848	2	9	18	AC	20	4
20021	2	10102140	10102169	2	15	30	TG	12	11
20030	2	10110952	10110971	2	10	20	GT	20	10
20031	2	10111114	10111139	2	13	26	TG	19	10
20032	2	10111329	10111360	2	16	32	GT	18	10
20038	2	10115722	10115737	4	4	16	CTGT	26	8
20048	2	10118544	10118559	2	8	16	CT	14	5
20050	2	10119761	10119781	3	7	21	AAT	14	6
20053	2	10121819	10121862	4	11	44	TTGA	13	10
20058	2	10122595	10122609	3	5	15	CCA	21	3
20059	2	10122781	10122804	3	8	24	CAG	27	12
20061	2	10123338	10123355	3	6	18	AGC	20	3
20063	2	10123930	10123943	2	7	14	AC	16	4
20064	2	10124035	10124066	2	16	32	TG	19	11
20075	2	10133142	10133167	2	13	26	CA	21	14
20078	2	10133668	10133693	2	13	26	AG	7	8
20095	2	10148430	10148465	2	18	36	CA	6	11
20096	2	10148629	10148656	2	14	28	CA	14	9
20098	2	10150413	10150424	2	6	12	TC	26	4
20099	2	10150998	10151015	2	9	18	AC	18	6
20100	2	10151099	10151114	2	8	16	AC	24	3
20104	2	10154597	10154624	2	14	28	TG	10	8
20113	2	10163483	10163504	2	11	22	CA	18	5
20115	2	10164055	10164068	2	7	14	AC	24	4
20116	2	10164193	10164220	2	14	28	GT	13	13
20117	2	10164376	10164395	2	10	20	GT	13	10
20119	2	10164624	10164657	2	17	34	AC	16	5
20124	2	10175299	10175312	2	7	14	GT	26	5
20126	2	10181966	10181993	2	14	28	AC	9	8
20127	2	10182528	10182545	2	9	18	GT	19	5
20132	2	10188202	10188221	2	10	20	AC	22	7
20135	2	10189756	10189815	4	15	60	TATC	18	9
20136	2	10190865	10190878	2	7	14	TC	19	6
20142	2	10194816	10194833	2	9	18	AC	9	4
20166	2	10211379	10211400	2	11	22	CA	7	5
20168	2	10214922	10214941	2	10	20	TG	12	10
20169	2	10220053	10220064	2	6	12	TG	12	4
20171	2	10220469	10220492	2	12	24	AC	17	5
20178	2	10228178	10228191	2	7	14	GT	34	6
20182	2	10230925	10230950	2	13	26	CA	19	10
20183	2	10232492	10232507	2	8	16	TG	22	8
20184	2	10232713	10232746	2	17	34	GT	12	11
20185	2	10233564	10233581	2	9	18	GT	22	7
20191	2	10236023	10236058	2	18	36	AC	17	10
20195	2	10241198	10241225	2	14	28	GT	10	9
20196	2	10242225	10242254	2	15	30	AC	8	8
20202	2	10244687	10244714	2	14	28	TG	7	7
20207	2	10247692	10247717	2	13	26	AG	24	8

20214	2	10259226	10259253	2	14	28	GT	11	8
20218	2	10265543	10265558	2	8	16	TG	19	7
20221	2	10266239	10266254	4	4	16	GGAG	25	4
20230	2	10268395	10268414	4	5	20	TTTC	15	9
20232	2	10269101	10269112	2	6	12	AT	16	8
20236	2	10272107	10272142	3	12	36	ATT	6	5
20238	2	10274102	10274125	3	8	24	CAG	25	5
20239	2	10274227	10274247	3	7	21	GCC	25	8
20244	2	10276300	10276329	2	15	30	TG	13	11
20246	2	10277924	10277941	2	9	18	CA	13	5
20247	2	10277986	10278031	2	23	46	CA	7	12
20251	2	10284631	10284644	2	7	14	CA	16	7
20253	2	10286520	10286543	2	12	24	GT	15	4
20257	2	10287095	10287106	2	6	12	TG	23	6
20263	2	10291833	10291864	2	16	32	TG	18	10
20267	2	10292146	10292175	2	15	30	GT	10	7
20270	2	10294383	10294412	2	15	30	GT	10	9
20272	2	10294614	10294639	2	13	26	TG	10	8
20274	2	10299273	10299296	2	12	24	GT	25	16
20280	2	10305291	10305308	3	6	18	ATT	9	3
20281	2	10306135	10306156	2	11	22	GT	24	6
20283	2	10307171	10307188	2	9	18	AC	23	4
20286	2	10309176	10309207	2	16	32	TC	7	7
20288	2	10310972	10310983	2	6	12	GT	24	5
20290	2	10313643	10313656	2	7	14	CA	22	9
20301	2	10328130	10328141	2	6	12	TG	25	5
20305	2	10329971	10329998	2	14	28	CA	20	7
20317	2	10357155	10357172	2	9	18	TG	29	3
20319	2	10361220	10361245	2	13	26	TG	12	8
20323	2	10382481	10382496	4	4	16	GGAT	24	4
20324	2	10383887	10383898	2	6	12	AT	7	4
20331	2	10390237	10390252	4	4	16	AAAC	18	8
20335	2	10398087	10398102	2	8	16	TC	28	5
20338	2	10404866	10404905	2	20	40	GA	6	3
20339	2	10405011	10405022	2	6	12	GT	13	3
20341	2	10406118	10406151	2	17	34	TG	17	13
20345	2	10412603	10412617	3	5	15	CTG	25	3
20346	2	10413387	10413420	2	17	34	TG	8	6
20347	2	10413469	10413488	2	10	20	AC	10	4
20354	2	10427185	10427208	2	12	24	GT	17	5
20355	2	10427358	10427375	2	9	18	CA	13	11
20357	2	10431271	10431308	2	19	38	CT	6	8
20358	2	10431482	10431545	4	16	64	AGAT	12	11
20359	2	10431622	10431653	2	16	32	CA	12	12
20360	2	10434224	10434251	2	14	28	CT	16	13
20386	2	10455261	10455272	2	6	12	CA	25	6
20387	2	10457433	10457446	2	7	14	AC	24	4
20388	2	10464025	10464036	2	6	12	GT	10	3
20390	2	10465668	10465685	2	9	18	AC	19	7
20408	2	10486108	10486135	2	14	28	TG	12	6
20409	2	10492958	10492975	2	9	18	CA	25	7

20410	2	10493138	10493153	2	8	16	CA	17	5
20415	2	10502114	10502143	2	15	30	TG	9	9
20418	2	10503166	10503228	3	21	63	AGA	18	12
20419	2	10506058	10506069	2	6	12	TG	20	3
20421	2	10510941	10510974	2	17	34	GA	9	7
20422	2	10512542	10512559	3	6	18	TGT	25	3
20423	2	10512933	10512958	2	13	26	GT	17	5
20425	2	10514725	10514740	4	4	16	AAAC	22	5
20426	2	10514989	10515018	2	15	30	AC	12	7
20428	2	10515154	10515187	2	17	34	AC	11	12
20434	2	10521559	10521574	4	4	16	AAAC	15	3
20437	2	10522740	10522773	2	17	34	CT	11	6
20438	2	10529225	10529244	2	10	20	CA	19	4
20453	2	10546533	10546554	2	11	22	GT	10	6
20455	2	10552833	10552844	2	6	12	TG	15	3
20458	2	10568366	10568381	4	4	16	GAAA	30	3
20459	2	10571336	10571371	2	18	36	TC	12	7
20462	2	10571762	10571787	2	13	26	CT	10	4
20463	2	10575092	10575127	2	18	36	AC	10	10
20466	2	10575433	10575446	2	7	14	TG	18	4
20471	2	10581298	10581327	2	15	30	CA	18	8
20473	2	10586235	10586248	2	7	14	TG	14	3
20475	2	10586471	10586502	2	16	32	GT	13	10
20476	2	10586615	10586662	4	12	48	AAAG	17	6
20480	2	10595622	10595635	2	7	14	AG	26	6
20481	2	10597141	10597164	2	12	24	TG	14	8
20486	2	10602138	10602153	4	4	16	TGGT	25	3
20490	2	10603488	10603515	2	14	28	AC	14	3
20491	2	10606151	10606184	2	17	34	CA	11	6
20492	2	10606301	10606358	2	29	58	GT	10	12
20497	2	10607602	10607631	2	15	30	CA	24	13
20499	2	10611931	10611956	2	13	26	AC	20	4
20503	2	10612764	10612777	2	7	14	CT	24	4
20504	2	10612916	10612937	2	11	22	GT	18	4
20507	2	10615731	10615762	4	8	32	TTCT	8	6
20510	2	10617288	10617299	2	6	12	CA	19	6
20511	2	10620335	10620350	2	8	16	AC	23	5
20515	2	10622789	10622802	2	7	14	TG	29	3
20516	2	10622919	10622954	2	18	36	TG	14	15
20519	2	10625002	10625027	2	13	26	TG	17	7
20520	2	10627471	10627486	4	4	16	AGAA	19	7
20523	2	10628963	10628980	2	9	18	CT	20	4
20529	2	10638598	10638629	2	16	32	AC	19	10
20534	2	10646808	10646835	2	14	28	AC	16	5
20541	2	10652970	10652999	2	15	30	TG	18	9
20543	2	10654441	10654454	2	7	14	AC	21	3
20544	2	10655837	10655866	2	15	30	AG	14	8
20545	2	10656198	10656215	2	9	18	TG	23	4
20546	2	10656328	10656355	2	14	28	CA	15	6
20547	2	10659316	10659337	2	11	22	CA	20	6
20552	2	10664560	10664583	2	12	24	TG	14	7

20556	2	10667784	10667805	2	11	22	GT	16	6
20559	2	10671343	10671370	2	14	28	CA	16	8
20560	2	10671548	10671567	2	10	20	CA	25	8
20561	2	10671673	10671686	2	7	14	AC	24	9
20569	2	10685679	10685692	2	7	14	CA	28	3
20570	2	10685900	10685915	4	4	16	TTTA	16	4
20571	2	10686949	10686960	2	6	12	GT	28	5
20573	2	10687251	10687274	2	12	24	AC	15	5
20574	2	10687444	10687471	2	14	28	CA	9	5
20576	2	10689760	10689793	2	17	34	CA	14	12
20580	2	10692379	10692404	2	13	26	CA	10	8
20581	2	10692540	10692551	2	6	12	GT	24	7
20586	2	10698367	10698382	2	8	16	CA	24	3
20588	2	10698518	10698533	2	8	16	AC	23	4
20593	2	10703853	10703874	2	11	22	AC	14	9
20594	2	10703973	10703984	2	6	12	TG	33	4
20605	2	10715722	10715737	2	8	16	AC	21	11
20608	2	10715892	10715907	2	8	16	GA	13	3
20613	2	10716322	10716341	2	10	20	CA	24	7
20614	2	10717553	10717580	2	14	28	CA	8	7
20616	2	10717718	10717741	2	12	24	AC	22	9
20622	2	10722477	10722502	2	13	26	AG	6	7
20623	2	10725020	10725033	2	7	14	TA	20	3
20624	2	10725811	10725832	2	11	22	AC	20	6
20625	2	10725910	10725929	2	10	20	TC	8	6
20628	2	10728090	10728111	2	11	22	AC	22	7
20632	2	10733030	10733055	2	13	26	AC	18	6
20634	2	10737382	10737405	3	8	24	CTC	20	5
20635	2	10737791	10737804	2	7	14	AC	30	3
20636	2	10738489	10738508	2	10	20	CA	19	5
20637	2	10738706	10738719	2	7	14	GT	22	4
20638	2	10739044	10739071	2	14	28	TG	11	12
20639	2	10740422	10740457	2	18	36	AC	7	9
20640	2	10741164	10741183	2	10	20	GT	19	18
20641	2	10741652	10741663	2	6	12	AC	24	4
20646	2	10746328	10746351	2	12	24	CA	22	5
20647	2	10751267	10751282	4	4	16	TGTC	31	6
20651	2	10756699	10756718	2	10	20	AC	25	10
20652	2	10757774	10757794	3	7	21	GCT	25	5
20654	2	10759338	10759349	2	6	12	CT	21	3
20656	2	10760067	10760084	2	9	18	CT	10	8
20658	2	10762745	10762756	2	6	12	CA	34	3
20660	2	10764232	10764247	2	8	16	CA	24	3
20664	2	10768936	10768951	4	4	16	TTTG	24	5
20665	2	10772191	10772204	2	7	14	CT	21	7
20667	2	10773086	10773119	2	17	34	GT	6	9
20669	2	10774153	10774178	2	13	26	CT	6	6
20670	2	10774211	10774226	2	8	16	CA	8	4
20684	2	10794582	10794599	2	9	18	GT	8	9
20687	2	10796260	10796274	3	5	15	CCT	15	3
20688	2	10796386	10796400	3	5	15	AGC	22	4

20690	2	10801661	10801678	2	9	18	AC	24	5
20692	2	10802938	10802975	2	19	38	AC	17	15
20693	2	10803415	10803432	2	9	18	AC	11	4
20695	2	10804565	10804592	2	14	28	GT	13	12
20696	2	10804675	10804698	2	12	24	TG	14	11
20709	2	10816505	10816516	2	6	12	GT	26	5
20713	2	10824437	10824469	3	11	33	AGA	15	8
20715	2	10825633	10825656	2	12	24	CA	16	10
20716	2	10828917	10828938	2	11	22	CA	17	11
20717	2	10829062	10829113	2	26	52	GT	7	8
20720	2	10831005	10831038	2	17	34	AC	10	6
20721	2	10831177	10831220	2	22	44	GT	6	8
20723	2	10831564	10831589	2	13	26	GT	13	11
20727	2	10834308	10834331	2	12	24	CA	13	11
20742	2	10855359	10855402	2	22	44	GT	6	10
20745	2	10855723	10855738	2	8	16	AC	24	4
20750	2	10857634	10857659	2	13	26	CA	9	3
20751	2	10858214	10858225	2	6	12	CA	27	4
20752	2	10859390	10859407	2	9	18	GT	17	4
20755	2	10860036	10860053	2	9	18	AC	17	7
20756	2	10860162	10860177	2	8	16	AG	24	3
20758	2	10860778	10860789	2	6	12	AG	23	6
20759	2	10860792	10860805	2	7	14	AG	25	7
20772	2	10867045	10867060	4	4	16	CAAA	24	3
20774	2	10867874	10867895	2	11	22	AG	17	7
20777	2	10872734	10872760	3	9	27	TTG	14	5
20778	2	10873369	10873386	2	9	18	GT	28	4
20779	2	10873493	10873518	2	13	26	AC	12	12
20787	2	10877914	10877949	2	18	36	CA	6	8
20788	2	10880027	10880040	2	7	14	GT	18	11
20790	2	10880327	10880342	2	8	16	CA	24	4
20797	2	10899074	10899091	2	9	18	TG	13	7
20808	2	10907664	10907687	4	6	24	AAAC	18	5
20809	2	10908322	10908333	2	6	12	GA	27	3
20811	2	10908660	10908699	2	20	40	TG	11	9
20820	2	10917086	10917097	2	6	12	CA	23	4
20821	2	10917579	10917620	2	21	42	TG	11	11
20822	2	10921605	10921634	2	15	30	TG	6	7
20823	2	10927702	10927727	2	13	26	AC	11	11
20827	2	10933058	10933077	2	10	20	CA	7	6
20832	2	10943900	10943917	2	9	18	AC	17	6
20833	2	10945491	10945502	2	6	12	TG	21	3
20834	2	10945946	10945961	2	8	16	AC	15	3
20837	2	10952316	10952329	2	7	14	GT	18	4
20841	2	10954856	10954895	2	20	40	GT	9	9
20843	2	10957326	10957359	2	17	34	TG	20	15
20850	2	10969752	10969769	2	9	18	AC	14	9
20853	2	10975086	10975105	2	10	20	GT	27	6
20855	2	10979620	10979655	4	9	36	GACA	20	9
20860	2	10982325	10982352	2	14	28	AC	13	9
20865	2	10986119	10986142	2	12	24	AG	7	7

20867	2	10987389	10987416	2	14	28	GT	13	11
20870	2	10993512	10993539	2	14	28	AC	18	13
20872	2	11002158	11002171	2	7	14	GT	15	5
20874	2	11002275	11002304	2	15	30	AG	11	6
20875	2	11002463	11002474	2	6	12	TG	29	3
20879	2	11008887	11008900	2	7	14	AC	30	5
20883	2	11014316	11014329	2	7	14	TG	13	3
20884	2	11014908	11014928	3	7	21	CAG	31	3
20886	2	11019222	11019253	2	16	32	GT	20	10
20889	2	11020808	11020831	2	12	24	CA	14	5
20893	2	11031426	11031447	2	11	22	CA	20	6
20895	2	11031593	11031610	2	9	18	TG	20	5
20898	2	11034277	11034292	4	4	16	ACAG	26	3
20900	2	11034873	11034892	2	10	20	GT	19	6
20901	2	11036732	11036761	2	15	30	GT	8	12
20906	2	11037301	11037320	2	10	20	GA	6	5
20909	2	11041762	11041783	2	11	22	AC	17	14
20913	2	11050009	11050026	2	9	18	AC	12	12
20915	2	11053815	11053834	4	5	20	TTCA	28	4
20919	2	11058260	11058283	4	6	24	ACAT	22	6
20921	2	11058505	11058544	4	10	40	TCTG	9	9
20922	2	11059889	11059940	2	26	52	GT	8	7
20924	2	11060995	11061026	2	16	32	AC	9	9
20925	2	11061461	11061490	2	15	30	CA	22	8
20926	2	11062207	11062230	2	12	24	TG	17	15
20929	2	11070306	11070325	2	10	20	CA	26	6
20930	2	11070368	11070379	2	6	12	CA	27	3
20933	2	11074229	11074260	2	16	32	CA	9	9
20935	2	11079240	11079273	2	17	34	TG	11	12
20936	2	11081205	11081232	2	14	28	AC	14	16
20951	2	11091338	11091379	2	21	42	GT	15	19
20952	2	11091653	11091664	2	6	12	TG	24	3
20957	2	11098693	11098706	2	7	14	AC	29	6
20961	2	11102003	11102014	2	6	12	CA	28	3
20962	2	11102156	11102187	2	16	32	TG	24	9
20964	2	11102525	11102544	2	10	20	TG	17	9
20968	2	11108125	11108142	2	9	18	AC	26	6
20970	2	11112765	11112810	2	23	46	AC	12	12
20976	2	11119354	11119377	2	12	24	AC	9	5
20977	2	11119519	11119542	2	12	24	CA	14	6
20983	2	11143280	11143307	4	7	28	ATCA	31	5
20984	2	11145180	11145207	2	14	28	GT	24	10
20986	2	11146895	11146914	4	5	20	AAAT	24	3
20990	2	11154361	11154372	2	6	12	GT	13	3
20991	2	11155589	11155606	2	9	18	TG	27	3
20992	2	11164571	11164585	3	5	15	AAT	22	4
20994	2	11176608	11176621	2	7	14	AT	24	8
20997	2	11185247	11185280	2	17	34	GT	9	9
21007	2	11213934	11213945	2	6	12	AC	15	8
21008	2	11214259	11214272	2	7	14	AG	8	3
21011	2	11214880	11214901	2	11	22	GT	10	5

21014	2	11227193	11227204	2	6	12	GT	27	4
21016	2	11227388	11227409	2	11	22	GT	6	7
21017	2	11227922	11227947	2	13	26	AC	12	8
21018	2	11233858	11233883	2	13	26	GT	18	9
21022	2	11235336	11235359	2	12	24	TG	12	4
21023	2	11237923	11237942	2	10	20	TG	14	8
21025	2	11245694	11245707	2	7	14	TG	34	5
21027	2	11245864	11245887	2	12	24	AC	11	8
21030	2	11251569	11251584	2	8	16	AC	26	9
21032	2	11262646	11262657	2	6	12	TC	35	3
21033	2	11263074	11263113	2	20	40	AC	24	9
21037	2	11271709	11271724	4	4	16	TTTC	24	3
21038	2	11271987	11272004	2	9	18	CA	15	8
21039	2	11272696	11272719	4	6	24	AAAC	12	5
21044	2	11294907	11294924	2	9	18	TG	23	7
21047	2	11296419	11296430	2	6	12	TG	28	3
21048	2	11296616	11296627	2	6	12	GT	7	4
21055	2	11302829	11302882	2	27	54	AC	11	10
21056	2	11302959	11302984	2	13	26	CA	8	12
21059	2	11305464	11305489	2	13	26	TG	20	7
21064	2	11314041	11314052	2	6	12	GT	26	7
21065	2	11316093	11316120	2	14	28	AC	13	6
21067	2	11323372	11323393	2	11	22	CA	18	4
21068	2	11323477	11323500	2	12	24	AC	23	11
21072	2	11337645	11337670	2	13	26	CA	11	4
21073	2	11339682	11339735	2	27	54	CA	18	13
21075	2	11352928	11352947	2	10	20	TG	20	4
21076	2	11353023	11353058	2	18	36	GT	12	7
21077	2	11355289	11355308	4	5	20	TATT	12	5
21079	2	11355458	11355485	2	14	28	GA	13	10
21080	2	11355529	11355566	2	19	38	CT	8	9
21081	2	11355943	11355960	2	9	18	CA	17	7
21084	2	11357059	11357086	2	14	28	TG	17	7
21090	2	11364122	11364135	2	7	14	AC	17	6
21091	2	11366421	11366446	2	13	26	GT	16	5
21095	2	11367484	11367503	2	10	20	TA	7	6
21096	2	11371046	11371063	3	6	18	TTG	24	6
21107	2	11388601	11388615	3	5	15	CGG	14	4
21109	2	11389084	11389101	3	6	18	CTG	29	3
21120	2	11414845	11414856	2	6	12	GT	17	3
21122	2	11415017	11415040	2	12	24	CT	17	3
21123	2	11416098	11416113	2	8	16	TC	28	7
21126	2	11421155	11421184	2	15	30	AC	8	11
21127	2	11421435	11421446	2	6	12	TG	17	3
21137	2	11432040	11432053	2	7	14	TG	10	6
21139	2	11434568	11434587	2	10	20	GT	7	8
21140	2	11439885	11439910	2	13	26	AC	18	12
21145	2	11440526	11440541	2	8	16	TC	13	3
21147	2	11445934	11445947	2	7	14	GT	22	5
21153	2	11450683	11450718	2	18	36	AC	9	13
21156	2	11453896	11453915	2	10	20	TG	22	5

21161	2	11468408	11468437	2	15	30	TG	18	13
21174	2	11512967	11512986	4	5	20	TGTT	23	3
21175	2	11513215	11513228	2	7	14	TA	24	3
21178	2	11515120	11515153	2	17	34	TC	7	7
21183	2	11517772	11517785	2	7	14	AC	21	4
21185	2	11518290	11518313	2	12	24	GA	7	6
21186	2	11521999	11522038	2	20	40	GT	17	14
21194	2	11529762	11529791	2	15	30	AC	23	7
21197	2	11531375	11531398	2	12	24	GT	9	6
21201	2	11538671	11538700	2	15	30	GT	15	6
21205	2	11540039	11540070	2	16	32	CA	14	10
21208	2	11547007	11547024	2	9	18	TG	26	11
21210	2	11549411	11549424	2	7	14	TG	25	4
21211	2	11549601	11549626	2	13	26	AC	19	6
21212	2	11549784	11549809	2	13	26	AC	14	8
21216	2	11558452	11558471	2	10	20	AC	25	3
21217	2	11559081	11559096	2	8	16	CA	24	9
21219	2	11559197	11559214	2	9	18	AG	8	7
21222	2	11559368	11559399	2	16	32	GA	14	9
21223	2	11559497	11559522	2	13	26	TG	23	18
21224	2	11559661	11559674	2	7	14	GT	18	6
21227	2	11565420	11565431	2	6	12	TC	17	3
21228	2	11565606	11565637	4	8	32	TGTC	27	11
21230	2	11567797	11567824	2	14	28	CA	6	7
21232	2	11568070	11568099	2	15	30	TG	17	13
21233	2	11570066	11570103	2	19	38	AC	9	9
21234	2	11571949	11571980	2	16	32	CA	9	7
21235	2	11576871	11576886	2	8	16	GT	23	3
21236	2	11577865	11577894	2	15	30	AC	10	8
21241	2	11595769	11595782	2	7	14	AC	8	3
21242	2	11607680	11607695	2	8	16	CA	25	4
21243	2	11612805	11612828	3	8	24	GCT	21	6
21247	2	11619787	11619802	2	8	16	GT	27	6
21248	2	11620656	11620669	2	7	14	TG	9	11
21249	2	11620672	11620683	2	6	12	TG	12	12
21253	2	11626841	11626855	3	5	15	AGC	27	5
21255	2	11628020	11628031	2	6	12	CT	19	4
21260	2	11639549	11639568	4	5	20	AAAG	27	5
21268	2	11662054	11662069	2	8	16	AC	13	7
21269	2	11662072	11662085	2	7	14	AC	18	6
21270	2	11662434	11662447	2	7	14	GT	22	3
21276	2	11666745	11666759	3	5	15	GCA	38	3
21278	2	11673753	11673770	2	9	18	AC	20	6
21279	2	11676927	11676940	2	7	14	GT	30	3
21281	2	11679154	11679199	2	23	46	AC	7	10
21283	2	11679875	11679886	2	6	12	TA	8	3
21289	2	11687014	11687029	2	8	16	TG	7	3
21293	2	11690486	11690503	2	9	18	AG	27	5
21301	2	11703405	11703425	3	7	21	AGG	22	4
21302	2	11703513	11703526	2	7	14	TG	26	4
21305	2	11703896	11703910	3	5	15	TCC	30	5

21308	2	11707340	11707359	4	5	20	TGTT	26	4
21310	2	11708453	11708464	2	6	12	AC	17	3
21312	2	11714629	11714660	2	16	32	AC	14	14
21318	2	11722650	11722665	4	4	16	TTTG	10	4
21320	2	11726147	11726164	3	6	18	TTC	22	4
21323	2	11740565	11740582	2	9	18	TG	28	4
21324	2	11745627	11745658	2	16	32	TC	9	7
21325	2	11745740	11745753	2	7	14	GT	22	5
21328	2	11756365	11756384	2	10	20	TG	17	15
21331	2	11758635	11758648	2	7	14	TG	19	5
21335	2	11777535	11777550	4	4	16	TTTC	13	4
21340	2	11803288	11803309	2	11	22	TG	22	11
21345	2	11816037	11816054	2	9	18	TG	24	5
21346	2	11819226	11819251	2	13	26	TG	19	12
21347	2	11819286	11819311	2	13	26	GT	25	4
21350	2	11822940	11822963	2	12	24	GT	13	5
21352	2	11832466	11832479	2	7	14	CA	22	7
21370	2	11847910	11847939	2	15	30	GT	13	8
21377	2	11862695	11862754	2	30	60	TG	9	13
21379	2	11862924	11862939	2	8	16	TG	9	3
21383	2	11866684	11866695	2	6	12	TG	16	10
21388	2	11876438	11876455	3	6	18	CCT	19	8
21390	2	11877533	11877544	2	6	12	AC	16	4
21391	2	11877562	11877573	2	6	12	CA	14	3
21399	2	11886340	11886351	2	6	12	CA	30	3
21400	2	11892884	11892899	2	8	16	CT	25	6
21403	2	11899887	11899912	2	13	26	TG	16	6
21404	2	11900067	11900092	2	13	26	AC	9	6
21407	2	11902279	11902294	2	8	16	TG	15	3
21409	2	11910300	11910323	2	12	24	AC	19	6
21412	2	11919827	11919848	2	11	22	AT	8	7
21417	2	11925182	11925203	2	11	22	CA	18	7
21418	2	11926675	11926698	2	12	24	GT	27	4
21420	2	11927855	11927876	2	11	22	CA	15	11
21422	2	11929917	11929932	4	4	16	AAAT	26	3
21423	2	11930692	11930725	2	17	34	CA	9	8
21425	2	11931084	11931113	2	15	30	CA	13	10
21426	2	11931136	11931151	2	8	16	CA	19	6
21427	2	11931365	11931380	2	8	16	AC	15	6
21436	2	11945563	11945576	2	7	14	GT	23	6
21437	2	11945813	11945827	3	5	15	CTC	19	6
21438	2	11945829	11945843	3	5	15	TCC	22	9
21440	2	11946025	11946052	2	14	28	AG	14	9
21441	2	11947781	11947798	2	9	18	CA	19	9
21444	2	11950458	11950469	2	6	12	CA	22	5
21446	2	11953372	11953389	3	6	18	TCT	31	3
21447	2	11953456	11953481	2	13	26	CA	10	9
21450	2	11954277	11954288	2	6	12	TC	19	4
21455	2	11959518	11959529	2	6	12	AC	23	3
21456	2	11959779	11959802	2	12	24	AC	11	11
21457	2	11960136	11960149	2	7	14	CA	29	5

21458	2	11960235	11960256	2	11	22	CA	18	6
21468	2	11971897	11971917	3	7	21	TTA	14	4
21471	2	11978519	11978532	2	7	14	CT	25	3
21472	2	11981820	11981843	2	12	24	GT	20	6
21473	2	11981971	11981998	2	14	28	AC	19	11
21476	2	11984469	11984490	2	11	22	CT	22	4
21484	2	11994377	11994394	2	9	18	GT	19	4
21486	2	11997160	11997173	2	7	14	GA	21	6
21487	2	11997211	11997226	2	8	16	AC	23	4
21491	2	12000721	12000760	2	20	40	AC	16	8
21492	2	12001289	12001309	3	7	21	TGC	29	4
21494	2	12001585	12001600	2	8	16	AC	20	10
21512	2	12042609	12042650	2	21	42	TG	9	7
21528	2	12059069	12059082	2	7	14	TG	26	6
21529	2	12062942	12062959	3	6	18	GCT	22	5
21530	2	12063254	12063271	2	9	18	AC	22	4
21534	2	12073746	12073765	4	5	20	TGAC	27	5
21540	2	12086219	12086248	2	15	30	AC	8	8
21545	2	12094032	12094047	2	8	16	TA	25	4
21547	2	12104935	12104972	2	19	38	TG	14	10
21554	2	12124346	12124375	3	10	30	CAT	24	14
21560	2	12177998	12178013	4	4	16	AAAC	22	9
21563	2	12184910	12184927	2	9	18	AC	21	5
21564	2	12185302	12185321	2	10	20	GA	20	8
21565	2	12185500	12185511	2	6	12	CG	21	18
21566	2	12185512	12185525	2	7	14	CA	17	14
21570	2	12191143	12191157	3	5	15	CAG	26	3
21571	2	12191258	12191277	2	10	20	TG	17	7
21573	2	12192505	12192518	2	7	14	GT	15	3
21579	2	12220037	12220060	2	12	24	TG	18	9
21584	2	12231052	12231065	2	7	14	AC	29	4
21588	2	12238578	12238595	2	9	18	TA	8	6
21591	2	12253057	12253070	2	7	14	GT	18	6
21592	2	12255650	12255673	3	8	24	CAG	27	5
21594	2	12256525	12256536	2	6	12	AC	23	14
21595	2	12257391	12257410	2	10	20	TG	20	15
21597	2	12257617	12257631	3	5	15	AAC	25	3
21599	2	12259387	12259436	2	25	50	TG	10	14
21601	2	12266098	12266127	3	10	30	ATC	15	9
21608	2	12287299	12287314	4	4	16	TGGA	9	3
21610	2	12288798	12288815	2	9	18	AC	12	9
21612	2	12289551	12289565	3	5	15	AAC	28	3
21613	2	12289632	12289655	2	12	24	CA	10	8
21618	2	12309747	12309762	2	8	16	AC	7	8
21620	2	12325052	12325069	3	6	18	TAA	26	4
21627	2	12343815	12343830	4	4	16	TTTC	19	4
21634	2	12369171	12369198	2	14	28	AC	20	7
21636	2	12385902	12385931	2	15	30	AC	14	8
21637	2	12388810	12388831	2	11	22	AC	16	3
21661	2	12413202	12413243	2	21	42	AC	8	13
21663	2	12413995	12414022	2	14	28	AC	7	6

21665	2	12414329	12414348	2	10	20	GT	22	17
21666	2	12414545	12414568	2	12	24	CT	25	6
21667	2	12416304	12416371	4	17	68	AGAT	10	10
21668	2	12416560	12416573	2	7	14	TA	23	5
21669	2	12417737	12417752	2	8	16	GT	15	12
21671	2	12419272	12419299	2	14	28	GT	21	12
21686	2	12458205	12458220	2	8	16	AC	29	4
21687	2	12463484	12463501	2	9	18	TC	28	3
21692	2	12472253	12472284	4	8	32	AGAA	23	6
21694	2	12481784	12481797	2	7	14	CT	34	4
21700	2	12493342	12493356	3	5	15	TTG	13	3
21703	2	12499837	12499850	2	7	14	AG	21	5
21707	2	12505478	12505499	2	11	22	TG	22	7
21711	2	12515557	12515574	2	9	18	AC	25	5
21712	2	12517815	12517826	2	6	12	AG	24	5
21722	2	12552853	12552864	2	6	12	CA	23	3
21725	2	12569118	12569131	2	7	14	TC	30	4
21729	2	12576020	12576041	2	11	22	TG	23	6
21732	2	12589037	12589068	2	16	32	GA	12	8
21733	2	12593864	12593875	2	6	12	GT	30	3
21734	2	12597767	12597784	2	9	18	AC	14	7
21738	2	12612061	12612075	3	5	15	TTC	13	5
21743	2	12619535	12619554	4	5	20	TTAT	14	4
21751	2	12624421	12624440	2	10	20	CA	19	12
21759	2	12639414	12639449	2	18	36	CA	12	6
21761	2	12641197	12641220	2	12	24	TG	19	12
21762	2	12643484	12643495	2	6	12	TG	25	3
21764	2	12653055	12653122	4	17	68	GATG	21	11
21767	2	12660232	12660257	2	13	26	GA	20	3
21773	2	12682152	12682167	2	8	16	AT	12	4
21781	2	12703451	12703474	2	12	24	TG	18	13
21788	2	12723067	12723102	2	18	36	CA	12	8
21791	2	12726370	12726383	2	7	14	TA	17	5
21799	2	12740801	12740812	2	6	12	GT	27	4
21802	2	12746692	12746723	2	16	32	CA	6	5
21807	2	12761243	12761256	2	7	14	AG	25	3
21820	2	12789416	12789435	2	10	20	TG	19	7
21821	2	12790389	12790430	2	21	42	CA	8	6
21822	2	12790716	12790749	2	17	34	AC	13	8
21826	2	12795098	12795109	2	6	12	AC	24	7
21827	2	12795115	12795126	2	6	12	CA	23	5
21829	2	12801583	12801594	2	6	12	AT	22	9
21830	2	12801595	12801608	2	7	14	AG	21	7
21832	2	12803611	12803630	2	10	20	CA	22	4
21837	2	12811798	12811821	2	12	24	GT	7	5
21839	2	12818662	12818681	2	10	20	AC	19	7
21842	2	12834730	12834744	3	5	15	CCT	20	4
21843	2	12837197	12837210	2	7	14	GT	15	3
21852	2	12936675	12936698	3	8	24	TGC	24	6
21857	2	12966091	12966117	3	9	27	ACA	19	4
21859	2	12970762	12970777	4	4	16	AAAT	20	4

21860	2	12972340	12972351	2	6	12	AC	25	3
21873	2	12998021	12998050	2	15	30	AC	9	7
21875	2	13001016	13001045	3	10	30	TCA	28	5
21879	2	13004372	13004407	2	18	36	AC	8	9
21881	2	13005414	13005434	3	7	21	GAG	22	3
21884	2	13006968	13006991	2	12	24	TG	23	14
21885	2	13007556	13007585	3	10	30	TCT	25	12
21892	2	13012229	13012266	2	19	38	GT	11	10
21894	2	13016652	13016672	3	7	21	CTC	20	4
21897	2	13017793	13017818	2	13	26	GT	18	6
21898	2	13018188	13018215	2	14	28	TG	19	8
21903	2	13019253	13019290	2	19	38	CA	9	6
21908	2	13020984	13020999	2	8	16	TG	23	4
21912	2	13027423	13027450	2	14	28	AC	13	12
21927	2	13048109	13048124	4	4	16	TTCT	32	4
21931	2	13055447	13055482	2	18	36	GT	8	7
21935	2	13068173	13068198	2	13	26	TG	16	7
21936	2	13068532	13068543	2	6	12	TG	28	3
21939	2	13073426	13073439	2	7	14	TG	28	4
21952	2	13098124	13098137	2	7	14	GT	25	15
21959	2	13117556	13117571	4	4	16	AACA	31	4
21960	2	13123905	13123948	4	11	44	CTTT	16	5
21963	2	13129848	13129867	4	5	20	TTTA	22	3
21971	2	13150953	13150996	4	11	44	TATC	21	8
21977	2	13168839	13168854	4	4	16	AAAT	19	15
21981	2	13189028	13189051	4	6	24	AAAC	28	3
21985	2	13213753	13213780	2	14	28	CT	14	6
21986	2	13217332	13217347	2	8	16	GT	22	5
21988	2	13219182	13219196	3	5	15	TGT	21	6
21991	2	13219345	13219372	4	7	28	ATAG	14	15
21995	2	13227379	13227396	3	6	18	AAC	28	9
21996	2	13227803	13227838	4	9	36	AATA	23	7
21998	2	13228286	13228321	3	12	36	AAT	7	5
21999	2	13228456	13228475	4	5	20	GTTT	18	5
22006	2	13237798	13237825	4	7	28	AAAG	12	5
22007	2	13237946	13237961	2	8	16	AC	17	17
22010	2	13244149	13244174	2	13	26	AG	10	5
22015	2	13261101	13261114	2	7	14	TG	23	4
22017	2	13269873	13269890	3	6	18	GCT	20	5
22018	2	13270469	13270484	2	8	16	GA	20	5
22019	2	13271099	13271110	2	6	12	AG	18	8
22021	2	13278048	13278067	2	10	20	AC	18	9
22023	2	13282642	13282661	2	10	20	TC	19	6
22026	2	13286022	13286043	2	11	22	TG	20	8
22027	2	13286648	13286669	2	11	22	CT	14	5
22033	2	13291643	13291662	2	10	20	TC	19	6
22034	2	13291717	13291750	2	17	34	CT	12	6
22044	2	13303092	13303123	2	16	32	AC	6	7
22049	2	13308151	13308172	2	11	22	TC	22	5
22052	2	13311235	13311258	3	8	24	TCC	22	4
22053	2	13311437	13311448	2	6	12	TG	25	6

22063	2	13319947	13319978	2	16	32	AC	8	9
22070	2	13327249	13327262	2	7	14	TG	29	4
22074	2	13334243	13334256	2	7	14	AC	25	3
22080	2	13339423	13339436	2	7	14	GT	17	6
22081	2	13339460	13339477	2	9	18	GT	17	9
22082	2	13339886	13339901	2	8	16	TG	26	3
22088	2	13346187	13346208	2	11	22	CA	24	5
22090	2	13347916	13347937	2	11	22	GA	19	6
22092	2	13348676	13348717	2	21	42	GA	11	7
22093	2	13348997	13349016	2	10	20	GA	12	5
22097	2	13349804	13349833	2	15	30	AC	15	9
22100	2	13351532	13351557	2	13	26	CT	15	11
22103	2	13354448	13354465	2	9	18	TG	20	6
22105	2	13355033	13355050	2	9	18	GT	19	4
22109	2	13356669	13356686	2	9	18	CT	19	13
22114	2	13359981	13359996	2	8	16	CA	22	5
22115	2	13360417	13360436	2	10	20	TG	19	7
22124	2	13373367	13373381	3	5	15	GGC	19	5
22127	2	13377939	13377974	2	18	36	CA	9	5
22132	2	13380738	13380751	2	7	14	GT	15	4
22133	2	13384233	13384246	2	7	14	AT	11	4
22134	2	13386629	13386650	2	11	22	GA	13	13
22137	2	13388924	13388935	2	6	12	GT	11	9
22138	2	13389495	13389514	2	10	20	GT	18	7
22141	2	13390305	13390336	2	16	32	TG	11	9
22142	2	13391305	13391316	2	6	12	AT	13	5
22143	2	13392161	13392174	2	7	14	TG	10	5
22153	2	13400423	13400434	2	6	12	TG	27	3
22157	2	13402098	13402135	2	19	38	TC	8	6
22163	2	13407334	13407357	2	12	24	GT	27	10
22166	2	13409852	13409865	2	7	14	CA	14	6
22168	2	13413232	13413243	2	6	12	TA	23	3
22182	2	13426782	13426803	2	11	22	GT	21	11
22191	2	13451362	13451393	2	16	32	AC	14	8
22196	2	13456921	13456944	2	12	24	TG	20	5
22197	2	13457122	13457135	2	7	14	AC	6	5
22198	2	13459532	13459561	3	10	30	AGA	21	11
22200	2	13461353	13461370	2	9	18	GT	25	6
22210	2	13473704	13473721	2	9	18	GT	28	3
22224	2	13493622	13493641	4	5	20	AAAG	24	3
22232	2	13498833	13498847	3	5	15	TTC	13	5
22248	2	13550728	13550742	3	5	15	TTG	29	4
22252	2	13567507	13567542	3	12	36	AGA	17	5
22253	2	13573465	13573497	3	11	33	TGT	10	6
22271	2	13612372	13612386	3	5	15	TTA	23	7
22279	2	13624344	13624365	2	11	22	GT	8	9
22281	2	13638841	13638852	2	6	12	CA	34	3
22285	2	13649550	13649564	3	5	15	TTG	21	3
22300	2	13682941	13682956	4	4	16	ACCA	30	3
22312	2	13717266	13717277	2	6	12	TG	26	4
22315	2	13726182	13726205	3	8	24	GGA	23	5

22318	2	13729931	13729948	3	6	18	CTG	26	6
22320	2	13733234	13733245	2	6	12	CT	21	3
22322	2	13736093	13736107	3	5	15	TCC	23	4
22330	2	13742473	13742490	2	9	18	GT	17	6
22334	2	13753470	13753499	2	15	30	TC	19	9
22336	2	13757942	13757957	2	8	16	CA	15	9
22338	2	13760009	13760036	2	14	28	TG	8	10
22348	2	13795251	13795268	2	9	18	TG	20	4
22352	2	13801024	13801045	2	11	22	TG	21	7
22354	2	13815291	13815320	2	15	30	AG	13	13
22370	2	13851073	13851086	2	7	14	CT	26	4
22376	2	13892256	13892287	2	16	32	AC	10	11
22388	2	13923765	13923784	4	5	20	TATG	13	3
22396	2	13942262	13942285	3	8	24	CCA	16	8
22419	2	13971225	13971239	3	5	15	CAC	21	4
22425	2	13986150	13986170	3	7	21	TTC	22	4
22427	2	13987690	13987707	2	9	18	CA	15	9
22428	2	13987888	13987908	3	7	21	CTG	24	8
22436	2	13997645	13997659	3	5	15	AAG	8	4
22438	2	14004736	14004751	4	4	16	TAAA	28	3
22442	2	14014967	14014990	4	6	24	ATCT	21	3
22445	2	14017882	14017905	2	12	24	GT	13	7
22446	2	14018173	14018188	2	8	16	TG	11	5
22459	2	14024662	14024679	2	9	18	TG	16	9
22474	2	14058466	14058492	3	9	27	AAG	29	4
22476	2	14063873	14063900	2	14	28	GT	15	10
22480	2	14079469	14079486	3	6	18	TGC	24	5
22481	2	14081856	14081871	2	8	16	AC	14	8
22484	2	14084636	14084662	3	9	27	TTA	18	7
22488	2	14094857	14094868	2	6	12	AC	20	3
22492	2	14110918	14110941	3	8	24	TTG	27	7
22496	2	14133183	14133204	2	11	22	TG	9	7
22498	2	14138227	14138244	2	9	18	GT	31	8
22505	2	14166231	14166256	2	13	26	CA	15	12
22509	2	14177296	14177331	4	9	36	AAAG	24	6
22510	2	14185566	14185625	4	15	60	GATA	13	11
22519	2	14208716	14208735	2	10	20	GA	12	6
22527	2	14219800	14219829	2	15	30	CT	20	7
22530	2	14227278	14227301	3	8	24	ACC	6	3
22531	2	14228951	14228964	2	7	14	CT	7	3
22534	2	14240017	14240028	2	6	12	AC	29	3
22538	2	14245976	14245990	3	5	15	AGA	20	4
22541	2	14253501	14253515	3	5	15	GCT	25	5
22551	2	14277716	14277733	3	6	18	CAG	18	5
22556	2	14294796	14294829	2	17	34	TG	14	14
22561	2	14300611	14300630	4	5	20	AAAT	30	5
22562	2	14300976	14300993	3	6	18	TAA	8	4
22563	2	14301211	14301242	2	16	32	GT	7	7
22564	2	14301277	14301291	3	5	15	CAG	15	3
22565	2	14301576	14301587	2	6	12	AG	8	8
22567	2	14303884	14303907	3	8	24	TTA	20	9

22568	2	14304033	14304047	3	5	15	AAT	28	3
22571	2	14305546	14305559	2	7	14	AG	26	7
22572	2	14307263	14307298	2	18	36	GT	12	12
22573	2	14307774	14307793	4	5	20	TTCT	20	8
22574	2	14307887	14307910	3	8	24	CTC	14	7
22575	2	14309277	14309290	2	7	14	TC	28	9
22578	2	14313424	14313441	3	6	18	CTG	32	5
22582	2	14318004	14318021	2	9	18	TG	9	5
22588	2	14324389	14324415	3	9	27	GCA	27	11
22589	2	14326252	14326269	2	9	18	CA	17	13
22591	2	14328949	14328982	2	17	34	AC	12	15
22593	2	14329893	14329910	2	9	18	AC	17	9
22596	2	14330802	14330817	2	8	16	GT	22	10
22597	2	14331062	14331083	2	11	22	GT	9	12
22603	2	14332171	14332185	3	5	15	CTG	14	5
22605	2	14332630	14332645	2	8	16	AC	7	7
22607	2	14333026	14333046	3	7	21	GGC	27	5
22609	2	14337457	14337478	2	11	22	AC	9	7
22610	2	14337578	14337595	3	6	18	GCT	27	3
22611	2	14337918	14337931	2	7	14	GT	13	10
22612	2	14337933	14337948	2	8	16	TG	15	10
22614	2	14346874	14346903	2	15	30	GA	24	14
22617	2	14357733	14357746	2	7	14	AC	24	5
22620	2	14368151	14368168	2	9	18	CA	23	11
22622	2	14368440	14368454	3	5	15	TTC	8	3
22628	2	14374053	14374064	2	6	12	CA	31	3
22633	2	14391120	14391139	2	10	20	CA	17	7
22638	2	14402175	14402200	2	13	26	GT	9	10
22641	2	14402825	14402836	2	6	12	AC	30	3
22642	2	14404480	14404507	2	14	28	TG	7	7
22648	2	14415946	14415991	2	23	46	GT	7	8
22653	2	14424047	14424060	2	7	14	TG	14	4
22661	2	14451622	14451643	2	11	22	AC	14	10
22663	2	14456732	14456767	2	18	36	CA	19	9
22668	2	14475856	14475875	2	10	20	AC	19	13
22679	2	14512972	14512995	3	8	24	AGG	28	8
22681	2	14520866	14520891	2	13	26	TG	20	10
22682	2	14520982	14520999	3	6	18	GTG	21	3
22688	2	14530620	14530649	2	15	30	AC	10	11
22694	2	14540001	14540016	2	8	16	TG	24	6
22698	2	14549535	14549554	2	10	20	TG	16	10
22702	2	14554892	14554907	2	8	16	AC	19	3
22703	2	14557358	14557369	2	6	12	CA	25	3
22704	2	14558657	14558682	2	13	26	CA	17	8
22708	2	14565548	14565567	2	10	20	AC	20	4
22725	2	14632811	14632858	2	24	48	GA	6	8
22726	2	14632991	14633008	3	6	18	AAC	23	5
22730	2	14635572	14635597	2	13	26	CA	15	19
22731	2	14635716	14635736	3	7	21	TGC	27	3
22732	2	14636202	14636215	2	7	14	TA	9	4
22734	2	14642484	14642507	2	12	24	CA	7	8

22735	2	14643301	14643315	3	5	15	GCG	31	4
22740	2	14645588	14645621	2	17	34	CA	18	12
22747	2	14662218	14662231	2	7	14	GA	15	5
22748	2	14662359	14662380	2	11	22	GT	16	4
22749	2	14663176	14663189	2	7	14	TG	23	7
22753	2	14666429	14666440	2	6	12	GT	18	7
22754	2	14666442	14666457	2	8	16	TG	20	8
22755	2	14666719	14666768	2	25	50	CA	7	7
22757	2	14667862	14667885	4	6	24	TCTG	27	10
22765	2	14717989	14718004	2	8	16	GT	27	3
22768	2	14723950	14723961	2	6	12	GT	14	5
22769	2	14724795	14724832	2	19	38	TG	12	8
22789	2	14762231	14762250	2	10	20	CA	23	10
22794	2	14768693	14768707	3	5	15	ATC	27	3
22799	2	14779089	14779112	2	12	24	GT	21	16
22802	2	14792415	14792436	2	11	22	GT	21	7
22803	2	14792729	14792752	2	12	24	TG	11	11
22804	2	14797255	14797280	2	13	26	TG	18	9
22809	2	14819302	14819315	2	7	14	GT	20	4
22812	2	14821803	14821822	2	10	20	TG	27	11
22814	2	14822252	14822265	2	7	14	AC	19	4
22820	2	14837015	14837032	3	6	18	GCT	19	5
22824	2	14848943	14848956	2	7	14	GT	26	3
22828	2	14851769	14851786	3	6	18	GCT	20	6
22833	2	14869442	14869453	2	6	12	AC	28	8
22835	2	14872713	14872730	3	6	18	GTT	22	8
22846	2	14882723	14882762	4	10	40	TTTC	18	16
22849	2	14887476	14887491	2	8	16	GT	7	4
22850	2	14888392	14888427	2	18	36	GT	21	8
22851	2	14888568	14888593	2	13	26	TG	19	10
22854	2	14890670	14890683	2	7	14	GT	15	9
22860	2	14896400	14896415	2	8	16	CA	17	19
22867	2	14905425	14905451	3	9	27	TAT	16	8
22875	2	14926544	14926561	2	9	18	GA	18	5
22878	2	14928361	14928388	2	14	28	TG	10	8
22882	2	14940451	14940476	2	13	26	TG	9	4
22883	2	14940765	14940802	2	19	38	AG	6	5
22886	2	14952387	14952400	2	7	14	TG	16	3
22889	2	14955931	14955954	2	12	24	GT	12	13
22892	2	14959039	14959059	3	7	21	AAC	21	3
22896	2	14960123	14960136	2	7	14	TG	23	4
22898	2	14960825	14960850	2	13	26	AC	7	9
22900	2	14963971	14963986	2	8	16	GT	29	6
22901	2	14966653	14966666	2	7	14	CA	26	3
22915	2	14978132	14978161	2	15	30	TG	9	11
22916	2	14979656	14979671	2	8	16	AC	7	3
22922	2	14983518	14983535	2	9	18	TG	11	8
22929	2	14990978	14991017	2	20	40	CT	10	8
22930	2	14991247	14991280	2	17	34	CA	7	9
22931	2	14992544	14992571	2	14	28	AC	14	9
22932	2	14992763	14992778	2	8	16	AC	16	7

22933	2	14994812	14994827	2	8	16	GT	20	3
22935	2	14997100	14997141	2	21	42	GT	9	10
22936	2	14997850	14997871	2	11	22	AC	20	8
22937	2	14998272	14998289	2	9	18	AC	18	6
22938	2	14998405	14998420	2	8	16	TG	24	4
22944	2	15002551	15002566	2	8	16	TG	19	8
22946	2	15005690	15005705	2	8	16	TG	27	13
22947	2	15008109	15008128	2	10	20	AC	11	11
22950	2	15012939	15012968	2	15	30	AC	17	7
22956	2	15021989	15022030	2	21	42	CA	7	8
22959	2	15024121	15024152	2	16	32	AC	9	13
22961	2	15026183	15026206	2	12	24	GT	20	8
22962	2	15029965	15029978	2	7	14	TG	17	5
22963	2	15030149	15030166	2	9	18	AC	21	7
22964	2	15030168	15030181	2	7	14	CA	21	6
22965	2	15030357	15030368	2	6	12	GT	25	3
22966	2	15031210	15031229	2	10	20	GT	22	10
22967	2	15031373	15031394	2	11	22	AC	12	6
22969	2	15037864	15037879	2	8	16	GT	27	5
22973	2	15040179	15040226	2	24	48	TG	7	6
22975	2	15040747	15040768	2	11	22	TG	8	4
22976	2	15040965	15040976	2	6	12	TG	25	4
22986	2	15061528	15061549	2	11	22	TG	22	8
22989	2	15065427	15065442	2	8	16	GT	24	5
22990	2	15065608	15065637	2	15	30	CA	24	10
22991	2	15066922	15066935	2	7	14	TG	33	3
22993	2	15069358	15069377	2	10	20	GT	16	5
22994	2	15069535	15069546	2	6	12	CA	14	7
22996	2	15071544	15071561	2	9	18	TG	11	7
22997	2	15075812	15075875	4	16	64	TAGA	7	7
22998	2	15077205	15077224	4	5	20	AAAC	10	13
23000	2	15078712	15078733	2	11	22	GT	9	9
23003	2	15079917	15079946	2	15	30	CA	13	7
23004	2	15080214	15080243	2	15	30	TG	10	8
23005	2	15080450	15080461	2	6	12	AG	22	4
23006	2	15081185	15081226	2	21	42	TG	12	10
23007	2	15082616	15082639	4	6	24	TTGT	14	9
23010	2	15085010	15085029	2	10	20	GT	18	8
23011	2	15085489	15085526	2	19	38	AC	12	11
23012	2	15085631	15085644	2	7	14	AC	16	9
23014	2	15089954	15089980	3	9	27	AAC	16	9
23016	2	15091016	15091031	2	8	16	AC	25	3
23018	2	15096444	15096463	2	10	20	CT	10	7
23020	2	15096804	15096833	2	15	30	TG	20	10
23022	2	15098416	15098429	2	7	14	CA	34	3
23023	2	15101251	15101282	2	16	32	AG	18	16
23024	2	15101759	15101776	2	9	18	GT	17	8
23028	2	15107415	15107428	2	7	14	TG	24	5
23030	2	15107956	15107991	2	18	36	AC	17	9
23031	2	15108127	15108154	2	14	28	AC	8	5
23035	2	15109250	15109267	3	6	18	CAG	26	7

23038	2	15109670	15109691	2	11	22	TC	17	9
23041	2	15111830	15111853	3	8	24	TGC	23	7
23043	2	15112527	15112540	2	7	14	CA	20	9
23048	2	15115718	15115729	2	6	12	TG	24	5
23058	2	15127697	15127720	2	12	24	TG	12	6
23059	2	15127852	15127883	2	16	32	AC	6	6
23060	2	15128087	15128100	2	7	14	AC	14	5
23067	2	15132307	15132318	2	6	12	TG	16	7
23069	2	15132458	15132477	2	10	20	CT	15	7
23072	2	15133364	15133377	2	7	14	CA	22	3
23073	2	15133940	15133951	2	6	12	TC	23	3
23074	2	15133952	15133969	2	9	18	AC	25	4
23075	2	15134242	15134255	2	7	14	CA	25	8
23076	2	15134403	15134414	2	6	12	AC	25	6
23077	2	15135758	15135773	2	8	16	TC	7	4
23080	2	15136430	15136459	2	15	30	GA	14	12
23082	2	15139974	15139995	2	11	22	TC	18	6
23083	2	15142673	15142704	4	8	32	AGAA	12	8
23084	2	15146722	15146745	4	6	24	CAGA	20	4
23090	2	15157753	15157786	2	17	34	TG	16	12
23094	2	15171470	15171487	3	6	18	TTG	29	4
23095	2	15173942	15173965	2	12	24	GA	12	8
23097	2	15176514	15176543	2	15	30	TG	22	13
23106	2	15195562	15195621	4	15	60	AAAG	13	15
23110	2	15207762	15207775	2	7	14	CA	16	3
23113	2	15211243	15211272	2	15	30	GT	17	6
23119	2	15214875	15214886	2	6	12	AC	23	3
23120	2	15219560	15219597	2	19	38	AC	14	13
23121	2	15219786	15219815	2	15	30	GT	12	10
23126	2	15222555	15222586	2	16	32	CT	10	9
23128	2	15222762	15222785	2	12	24	AC	19	7
23129	2	15223495	15223508	2	7	14	CA	25	3
23140	2	15240630	15240645	4	4	16	GATA	25	6
23144	2	15249053	15249073	3	7	21	TCA	18	3
23170	2	15282214	15282225	2	6	12	CA	23	3
23172	2	15294699	15294724	2	13	26	AG	17	5
23177	2	15322155	15322190	2	18	36	TG	13	10
23187	2	15340259	15340284	2	13	26	GT	10	6
23188	2	15342061	15342078	2	9	18	GT	9	8
23189	2	15343811	15343852	2	21	42	GT	7	8
23194	2	15351851	15351870	2	10	20	CA	19	6
23197	2	15356533	15356552	2	10	20	TG	11	4
23199	2	15365397	15365414	2	9	18	TG	25	8
23200	2	15365417	15365432	2	8	16	TG	20	9
23203	2	15371288	15371305	2	9	18	CA	23	5
23204	2	15371351	15371362	2	6	12	AC	32	4
23205	2	15371987	15372001	3	5	15	ATT	17	9
23206	2	15373513	15373550	2	19	38	AC	11	12
23208	2	15375778	15375795	2	9	18	AC	17	6
23215	2	15383349	15383370	2	11	22	TG	21	8
23216	2	15383488	15383503	2	8	16	TG	19	4

23222	2	15398713	15398736	2	12	24	CA	20	3
23223	2	15399296	15399323	2	14	28	AC	16	12
23225	2	15402049	15402060	2	6	12	GT	27	4
23228	2	15406190	15406215	2	13	26	GT	11	7
23229	2	15407329	15407342	2	7	14	GT	29	5
23230	2	15408758	15408787	2	15	30	TG	10	12
23231	2	15408903	15408942	2	20	40	TC	6	8
23234	2	15409077	15409110	2	17	34	GT	9	9
23235	2	15412419	15412446	2	14	28	TC	14	8
23238	2	15413636	15413655	2	10	20	AG	18	5
23239	2	15414071	15414098	2	14	28	TG	12	12
23243	2	15422754	15422777	2	12	24	CA	12	6
23248	2	15423386	15423409	2	12	24	TG	21	20
23249	2	15423515	15423530	2	8	16	GT	11	4
23252	2	15430114	15430131	2	9	18	AC	11	14
23259	2	15433893	15433912	2	10	20	GT	16	6
23262	2	15440651	15440688	2	19	38	GT	12	8
23264	2	15442028	15442047	2	10	20	CA	10	9
23265	2	15442162	15442173	2	6	12	GT	10	7
23267	2	15442328	15442365	2	19	38	GT	10	10
23268	2	15442485	15442524	2	20	40	GA	9	8
23269	2	15443006	15443025	4	5	20	GTTG	25	4
23272	2	15449460	15449489	2	15	30	CA	7	7
23273	2	15451633	15451646	2	7	14	GA	22	4
23275	2	15454366	15454399	2	17	34	CA	10	11
23276	2	15454464	15454489	2	13	26	AC	15	11
23278	2	15455170	15455185	4	4	16	TCTT	18	5
23280	2	15456441	15456468	2	14	28	GT	18	5
23284	2	15458598	15458619	2	11	22	CA	13	10
23286	2	15458769	15458780	2	6	12	CA	29	7
23287	2	15458909	15458926	2	9	18	CA	18	5
23288	2	15458975	15459004	2	15	30	CA	16	10
23294	2	15465076	15465101	2	13	26	TG	22	9
23295	2	15465211	15465226	2	8	16	TG	12	4
23296	2	15465523	15465534	2	6	12	CA	27	9
23297	2	15465821	15465858	2	19	38	GT	6	8
23298	2	15465935	15465952	2	9	18	CA	10	10
23300	2	15468356	15468379	3	8	24	GCT	13	5
23304	2	15468545	15468562	2	9	18	CT	16	6
23307	2	15476291	15476302	2	6	12	AC	24	6
23312	2	15487063	15487074	2	6	12	AC	25	3
23315	2	15490125	15490154	2	15	30	GT	17	8
23316	2	15490466	15490507	3	14	42	TCC	16	8
23317	2	15490713	15490734	2	11	22	AC	6	5
23318	2	15490856	15490871	4	4	16	GGAA	29	3
23319	2	15491161	15491175	3	5	15	GAG	17	4
23329	2	15494672	15494685	2	7	14	GT	21	6
23330	2	15495049	15495072	2	12	24	AC	15	9
23333	2	15497687	15497702	2	8	16	GT	17	7
23347	2	15508568	15508593	2	13	26	TG	23	8
23349	2	15511786	15511799	2	7	14	AC	24	4

23350	2	15514503	15514514	2	6	12	AC	24	3
23353	2	15518142	15518159	2	9	18	AC	21	4
23354	2	15518255	15518270	2	8	16	GT	27	10
23356	2	15520700	15520723	2	12	24	CA	21	10
23362	2	15525114	15525141	2	14	28	AC	18	12
23371	2	15530024	15530039	4	4	16	AGGA	12	4
23373	2	15530141	15530164	4	6	24	GGAA	9	3
23374	2	15530291	15530314	4	6	24	AGAA	25	5
23378	2	15533145	15533176	2	16	32	TG	14	13
23379	2	15534659	15534682	2	12	24	TG	13	11
23380	2	15534986	15534999	2	7	14	TG	13	5
23382	2	15536676	15536707	2	16	32	TC	15	8
23383	2	15539590	15539605	2	8	16	TG	22	6
23384	2	15539913	15539926	2	7	14	CA	20	3
23385	2	15539928	15539943	2	8	16	AC	21	4
23386	2	15540057	15540078	2	11	22	GT	8	8
23395	2	15543986	15544017	2	16	32	GT	22	6
23400	2	15550102	15550117	2	8	16	CA	16	10
23401	2	15552431	15552442	2	6	12	TG	32	3
23402	2	15553007	15553018	2	6	12	AC	33	3
23403	2	15553244	15553259	4	4	16	TGCA	32	5
23406	2	15559008	15559023	2	8	16	GA	16	3
23407	2	15559482	15559505	2	12	24	TG	15	10
23410	2	15562237	15562248	2	6	12	CT	28	4
23415	2	15571360	15571371	2	6	12	GT	7	3
23421	2	15584453	15584470	3	6	18	CTG	19	4
23425	2	15589969	15589980	2	6	12	AT	28	3
23427	2	15591105	15591130	2	13	26	TG	18	15
23437	2	15595487	15595504	2	9	18	TG	22	4
23450	2	15613199	15613244	2	23	46	GT	6	10
23452	2	15613719	15613738	4	5	20	GTCT	22	4
23453	2	15613781	15613798	2	9	18	AC	24	4
23454	2	15613876	15613911	2	18	36	AC	9	11
23461	2	15625436	15625447	2	6	12	AC	26	7
23462	2	15626942	15626999	2	29	58	GT	8	9
23465	2	15629339	15629362	2	12	24	TG	6	9
23471	2	15640238	15640261	2	12	24	TG	14	9
23477	2	15649818	15649829	2	6	12	AC	24	5
23478	2	15649991	15650008	2	9	18	CA	24	7
23479	2	15650077	15650088	2	6	12	AC	32	3
23480	2	15650587	15650619	3	11	33	TCC	16	4
23481	2	15654512	15654525	2	7	14	AC	23	3
23494	2	15674706	15674727	2	11	22	TG	21	16
23495	2	15677606	15677625	2	10	20	AC	17	6
23496	2	15679617	15679628	2	6	12	CA	31	3
23497	2	15679764	15679799	2	18	36	TC	12	11
23504	2	15689447	15689470	2	12	24	GT	15	9
23506	2	15691861	15691876	2	8	16	TG	24	8
23508	2	15693988	15694015	2	14	28	GT	19	10
23510	2	15694367	15694386	2	10	20	CA	24	6
23511	2	15694854	15694877	2	12	24	GT	9	8

23513	2	15696106	15696143	2	19	38	TG	7	8
23514	2	15696262	15696287	2	13	26	TG	11	11
23518	2	15696689	15696720	2	16	32	GT	12	10
23519	2	15696832	15696845	2	7	14	AC	25	3
23521	2	15699249	15699272	2	12	24	CA	15	13
23522	2	15702655	15702674	2	10	20	TC	30	5
23523	2	15703009	15703024	2	8	16	TG	26	4
23524	2	15703213	15703232	2	10	20	AC	21	6
23526	2	15704782	15704823	2	21	42	AC	8	12
23528	2	15706394	15706441	2	24	48	GT	7	8
23529	2	15706566	15706577	2	6	12	AC	18	8
23531	2	15707979	15707990	2	6	12	TG	22	4
23536	2	15710144	15710161	2	9	18	CA	18	8
23543	2	15715440	15715457	2	9	18	GT	16	4
23545	2	15715771	15715802	2	16	32	AC	20	7
23547	2	15720621	15720638	2	9	18	TG	20	3
23548	2	15720779	15720804	2	13	26	AC	16	6
23552	2	15725123	15725146	4	6	24	ATCC	24	4
23554	2	15725445	15725468	2	12	24	AC	7	8
23555	2	15729445	15729462	2	9	18	GT	26	3
23559	2	15734384	15734397	2	7	14	TG	20	3
23564	2	15738105	15738120	2	8	16	CA	13	3
23566	2	15739544	15739563	4	5	20	CAGA	27	10
23573	2	15744842	15744855	2	7	14	GA	18	5
23579	2	15747069	15747082	2	7	14	TG	16	7
23584	2	15748431	15748456	2	13	26	TG	11	12
23588	2	15749179	15749192	2	7	14	CA	21	6
23590	2	15749384	15749413	2	15	30	GT	6	4
23591	2	15749658	15749673	2	8	16	CA	19	8
23595	2	15751379	15751394	2	8	16	CA	18	7
23596	2	15751627	15751650	2	12	24	TG	15	11
23598	2	15752361	15752378	2	9	18	TG	25	5
23599	2	15754289	15754304	2	8	16	AC	22	4
23600	2	15754525	15754558	2	17	34	GT	6	9
23601	2	15757363	15757382	2	10	20	AG	21	7
23602	2	15757884	15757905	2	11	22	TG	6	5
23603	2	15758059	15758090	2	16	32	CA	16	9
23605	2	15761702	15761721	2	10	20	CA	17	5
23606	2	15762323	15762336	2	7	14	TG	34	3
23608	2	15763654	15763665	2	6	12	CA	23	3
23613	2	15765565	15765578	2	7	14	TG	18	4
23615	2	15765694	15765713	2	10	20	TG	20	5
23617	2	15765996	15766021	2	13	26	TG	16	6
23618	2	15768141	15768162	2	11	22	TG	15	7
23619	2	15768440	15768465	2	13	26	AC	13	10
23620	2	15768947	15768974	2	14	28	AC	17	4
23622	2	15769603	15769630	2	14	28	AC	22	5
23630	2	15779476	15779501	2	13	26	CA	11	7
23634	2	15780367	15780390	2	12	24	AC	12	8
23635	2	15782392	15782403	2	6	12	AC	9	3
23636	2	15782590	15782607	2	9	18	GT	26	10

23637	2	15782877	15782892	2	8	16	GT	20	5
23641	2	15792856	15792869	2	7	14	AC	15	4
23642	2	15800323	15800340	2	9	18	CA	19	4
23643	2	15801382	15801407	2	13	26	GT	13	14
23645	2	15801549	15801568	2	10	20	AC	11	5
23646	2	15801654	15801671	2	9	18	TG	10	8
23648	2	15802750	15802767	2	9	18	TG	26	4
23649	2	15802909	15802940	2	16	32	AC	11	11
23653	2	15806476	15806503	2	14	28	TG	9	8
23654	2	15806868	15806881	2	7	14	GT	15	3
23656	2	15807133	15807152	2	10	20	AC	28	3
23666	2	15820186	15820207	2	11	22	CA	22	7
23667	2	15823197	15823208	2	6	12	GT	25	4
23670	2	15844275	15844304	2	15	30	AC	15	7
23671	2	15845050	15845089	2	20	40	TG	16	10
23683	2	15874171	15874212	2	21	42	TG	10	10
23692	2	15884087	15884101	3	5	15	GAG	30	4
23693	2	15887406	15887421	2	8	16	AC	24	6
23694	2	15890391	15890408	3	6	18	GAG	29	6
23696	2	15893702	15893725	4	6	24	GGAT	25	3
23699	2	15920923	15920936	2	7	14	AC	25	3
23700	2	15931551	15931582	4	8	32	TATC	23	12
23703	2	15935269	15935292	4	6	24	TATT	8	4
23707	2	15938041	15938062	2	11	22	TG	14	8
23709	2	15940055	15940076	2	11	22	TG	25	9
23716	2	15950066	15950085	2	10	20	AC	15	4
23718	2	15960051	15960066	2	8	16	CA	18	8
23722	2	15963889	15963906	2	9	18	AC	21	4
23723	2	15966888	15966911	2	12	24	GT	22	11
23726	2	15969307	15969318	2	6	12	GT	27	4
23727	2	15970428	15970439	2	6	12	GT	19	5
23729	2	15971569	15971595	3	9	27	GAG	16	4
23731	2	15973454	15973468	3	5	15	GCC	20	7
23732	2	15973469	15973483	3	5	15	TCC	21	6
23733	2	15973678	15973698	3	7	21	CCT	18	4
23734	2	15978252	15978273	2	11	22	GT	16	14
23735	2	15981328	15981341	2	7	14	AC	34	3
23741	2	15989714	15989741	2	14	28	AG	17	9
23743	2	16000682	16000705	4	6	24	CCAT	26	7
23755	2	16020516	16020529	2	7	14	CA	28	4
23757	2	16025268	16025288	3	7	21	TTC	10	5
23760	2	16029390	16029403	2	7	14	TG	25	5
23761	2	16033291	16033310	2	10	20	TG	10	5
23764	2	16033490	16033537	2	24	48	AC	15	9
23765	2	16035776	16035827	2	26	52	TG	17	12
23766	2	16041378	16041401	2	12	24	TG	16	9
23767	2	16041544	16041565	2	11	22	AC	25	9
23768	2	16043201	16043232	2	16	32	CT	12	14
23769	2	16043515	16043538	4	6	24	AAAG	17	6
23782	2	16066177	16066192	2	8	16	TG	6	5
23784	2	16069809	16069824	2	8	16	CA	18	8

23787	2	16072854	16072867	2	7	14	TG	32	3
23789	2	16078955	16078990	2	18	36	AC	26	9
23790	2	16079761	16079804	2	22	44	TG	7	9
23793	2	16086631	16086668	2	19	38	TG	7	7
23794	2	16088744	16088755	2	6	12	GT	24	3
23799	2	16106271	16106300	2	15	30	TG	24	10
23802	2	16117676	16117693	2	9	18	AC	25	6
23803	2	16117828	16117845	2	9	18	GT	18	7
23806	2	16121028	16121041	2	7	14	TG	13	3
23807	2	16121376	16121387	2	6	12	TG	22	9
23818	2	16137146	16137159	2	7	14	GT	15	10
23820	2	16143484	16143495	2	6	12	AC	19	11
23821	2	16146568	16146585	2	9	18	GT	19	7
23823	2	16165147	16165164	2	9	18	GA	26	4
23825	2	16165494	16165523	2	15	30	GT	17	11
23835	2	16171024	16171067	4	11	44	TCTA	15	10
23836	2	16175205	16175222	2	9	18	AC	19	7
23839	2	16177055	16177086	2	16	32	GT	16	14
23852	2	16186784	16186811	2	14	28	TG	19	10
23853	2	16187086	16187099	2	7	14	GT	21	4
23857	2	16191515	16191550	2	18	36	AC	7	11
23862	2	16194091	16194108	2	9	18	TG	16	4
23863	2	16194262	16194285	2	12	24	AC	11	12
23865	2	16195450	16195461	2	6	12	TA	14	3
23866	2	16197880	16197899	2	10	20	TG	20	6
23867	2	16198037	16198048	2	6	12	GT	6	3
23868	2	16198613	16198668	2	28	56	AG	14	12
23872	2	16208196	16208207	2	6	12	CA	24	5
23877	2	16213681	16213692	2	6	12	AC	27	3
23878	2	16214867	16214888	2	11	22	TG	13	5
23880	2	16220342	16220357	2	8	16	CT	23	5
23882	2	16222287	16222308	2	11	22	TG	24	7
23892	2	16237968	16237983	2	8	16	CA	18	19
23893	2	16238084	16238103	4	5	20	GTGA	27	5
23900	2	16269655	16269694	2	20	40	GT	14	14
23901	2	16270624	16270647	2	12	24	TG	18	6
23912	2	16284149	16284164	2	8	16	TC	13	6
23919	2	16297434	16297451	2	9	18	TG	17	7
23923	2	16307087	16307122	2	18	36	TG	13	12
23942	2	16318258	16318277	2	10	20	AC	17	4
23943	2	16319237	16319251	3	5	15	CAG	18	3
23946	2	16323353	16323384	2	16	32	AC	15	7
23949	2	16331803	16331822	2	10	20	TC	26	4
23957	2	16345432	16345443	2	6	12	AC	28	3
23958	2	16345847	16345862	2	8	16	CT	12	6
23959	2	16358406	16358435	3	10	30	AAG	18	11
23960	2	16361389	16361408	2	10	20	CA	13	15
23967	2	16383897	16383912	4	4	16	AAAC	22	3
23974	2	16392731	16392752	2	11	22	TG	24	3
23979	2	16405848	16405867	2	10	20	GT	14	4
23981	2	16407677	16407702	2	13	26	CA	17	7

23984	2	16413960	16413995	2	18	36	GT	15	9
23986	2	16417333	16417354	2	11	22	GT	16	4
23988	2	16422796	16422823	2	14	28	CA	19	8
24003	2	16447069	16447094	2	13	26	TC	19	8
24004	2	16450722	16450757	2	18	36	TG	6	7
24005	2	16450879	16450926	2	24	48	AC	7	9
24008	2	16463315	16463328	2	7	14	CA	23	6
24009	2	16463692	16463705	2	7	14	TG	26	5
24010	2	16465992	16466013	2	11	22	TG	25	8
24011	2	16467244	16467269	2	13	26	TG	15	7
24013	2	16469626	16469640	3	5	15	CAG	26	5
24015	2	16478504	16478517	2	7	14	TG	22	3
24016	2	16483427	16483444	2	9	18	GT	26	5
24017	2	16493956	16493977	2	11	22	CA	16	5
24024	2	16499026	16499043	2	9	18	GT	16	10
24025	2	16501295	16501308	2	7	14	AC	33	3
24026	2	16501600	16501649	2	25	50	GT	7	4
24041	2	16514640	16514657	3	6	18	CCT	6	4
24045	2	16515578	16515593	2	8	16	GA	12	6
24048	2	16516435	16516454	2	10	20	CA	14	10
24050	2	16519286	16519306	3	7	21	AGC	22	3
24053	2	16528060	16528079	2	10	20	CT	23	3
24055	2	16529151	16529180	2	15	30	CA	9	7
24056	2	16529651	16529672	2	11	22	AC	14	11
24057	2	16531962	16531999	2	19	38	TG	11	7
24059	2	16534888	16534899	2	6	12	AG	26	4
24060	2	16534951	16534971	3	7	21	TCC	20	3
24062	2	16536741	16536764	3	8	24	AGC	33	6
24063	2	16537834	16537849	2	8	16	CT	24	4
24069	2	16539163	16539174	2	6	12	GT	31	5
24070	2	16541509	16541538	2	15	30	AC	9	7
24072	2	16542500	16542515	4	4	16	TCCT	28	4
24074	2	16547313	16547334	2	11	22	AC	13	9
24076	2	16547523	16547548	2	13	26	CA	18	8
24078	2	16550931	16550950	4	5	20	AAAG	31	6
24080	2	16555145	16555182	2	19	38	CA	14	14
24081	2	16555410	16555429	2	10	20	TG	22	4
24082	2	16562082	16562113	2	16	32	CA	23	10
24090	2	16573477	16573490	2	7	14	GT	30	4
24097	2	16585316	16585335	2	10	20	AC	17	15
24098	2	16585337	16585348	2	6	12	CA	15	14
24107	2	16594390	16594403	2	7	14	TG	27	3
24112	2	16600074	16600087	2	7	14	AC	29	3
24113	2	16600137	16600148	2	6	12	CA	26	3
24114	2	16600512	16600523	2	6	12	GC	27	5
24118	2	16616274	16616297	2	12	24	GT	11	8
24126	2	16622608	16622623	2	8	16	CA	14	9
24134	2	16627567	16627600	2	17	34	AC	11	8
24136	2	16629253	16629264	2	6	12	AG	15	3
24137	2	16629527	16629538	2	6	12	AG	19	3
24138	2	16630085	16630098	2	7	14	TG	25	5

24139	2	16630296	16630329	2	17	34	CA	8	6
24140	2	16630497	16630516	2	10	20	GA	17	6
24141	2	16631024	16631039	2	8	16	GT	21	10
24144	2	16634496	16634515	2	10	20	TG	18	7
24146	2	16634728	16634745	3	6	18	CTC	13	4
24147	2	16636892	16636903	2	6	12	GA	27	3
24148	2	16637629	16637640	2	6	12	GA	11	3
24150	2	16638428	16638451	2	12	24	GT	24	9
24153	2	16638937	16638954	2	9	18	TG	10	3
24155	2	16639295	16639308	2	7	14	CT	19	3
24157	2	16639601	16639612	2	6	12	CA	20	5
24163	2	16643312	16643333	2	11	22	CA	19	9
24164	2	16643436	16643451	2	8	16	TG	13	4
24165	2	16646387	16646400	2	7	14	CT	24	4
24166	2	16646564	16646583	2	10	20	CA	17	10
24174	2	16650078	16650127	2	25	50	AC	12	10
24176	2	16650306	16650323	2	9	18	GT	11	3
24177	2	16650691	16650718	2	14	28	TG	10	6
24178	2	16653008	16653029	2	11	22	TG	13	10
24183	2	16655117	16655144	2	14	28	CA	8	9
24200	2	16671347	16671361	3	5	15	AGA	28	7
24204	2	16674068	16674081	2	7	14	TG	28	6
24207	2	16674571	16674590	2	10	20	CT	23	3
24208	2	16676925	16676960	3	12	36	TCT	16	9
24209	2	16677067	16677112	2	23	46	AC	7	6
24210	2	16677162	16677177	2	8	16	CA	21	3
24212	2	16682695	16682708	2	7	14	TG	21	6
24213	2	16683727	16683746	2	10	20	AC	14	9
24216	2	16686888	16686917	2	15	30	TG	11	12
24219	2	16688108	16688119	2	6	12	CA	20	3
24224	2	16695524	16695551	2	14	28	TG	13	7
24226	2	16696554	16696565	2	6	12	GT	22	4
24231	2	16699871	16699890	2	10	20	CA	8	6
24232	2	16700014	16700041	2	14	28	CT	9	11
24237	2	16703086	16703111	2	13	26	GA	16	5
24238	2	16705102	16705121	2	10	20	TG	21	10
24239	2	16705166	16705185	2	10	20	GT	19	6
24243	2	16709237	16709266	2	15	30	CA	7	7
24245	2	16709495	16709532	2	19	38	TG	16	9
24248	2	16710449	16710462	2	7	14	GT	22	6
24249	2	16710557	16710572	2	8	16	CA	25	5
24254	2	16718169	16718196	2	14	28	CA	20	6
24255	2	16720479	16720502	2	12	24	TG	18	3
24259	2	16723627	16723656	2	15	30	GT	9	7
24264	2	16726210	16726225	2	8	16	CA	14	9
24267	2	16729181	16729224	2	22	44	CA	6	9
24269	2	16729665	16729696	2	16	32	AC	18	7
24271	2	16730922	16730947	2	13	26	CA	9	5
24272	2	16731317	16731338	2	11	22	GT	26	5
24276	2	16740889	16740908	2	10	20	CA	19	6
24277	2	16741530	16741547	2	9	18	CA	17	4

24278	2	16741604	16741625	2	11	22	AC	21	7
24279	2	16742177	16742188	2	6	12	TG	25	5
24286	2	16747013	16747026	2	7	14	CT	25	5
24287	2	16747204	16747229	2	13	26	TG	17	9
24293	2	16749154	16749171	2	9	18	AC	13	5
24299	2	16753912	16753923	2	6	12	TG	19	7
24301	2	16754005	16754030	2	13	26	GT	15	7
24308	2	16759621	16759638	2	9	18	GT	19	4
24311	2	16760635	16760650	2	8	16	CT	17	4
24312	2	16763441	16763462	2	11	22	TG	17	11
24314	2	16764007	16764018	2	6	12	CA	20	4
24315	2	16764020	16764037	2	9	18	AC	20	5
24316	2	16764210	16764223	2	7	14	CT	15	3
24318	2	16764575	16764586	2	6	12	CA	31	3
24323	2	16774186	16774219	2	17	34	TG	7	10
24324	2	16775519	16775538	2	10	20	GT	13	10
24328	2	16778819	16778846	2	14	28	GT	16	11
24329	2	16778964	16778989	2	13	26	GT	13	6
24331	2	16779066	16779091	2	13	26	TG	14	7
24333	2	16783837	16783850	2	7	14	GT	28	7
24334	2	16788584	16788601	2	9	18	CA	18	14
24338	2	16796072	16796083	2	6	12	CA	22	3
24339	2	16799245	16799262	2	9	18	AC	24	6
24341	2	16806133	16806146	2	7	14	TG	24	4
24342	2	16806512	16806537	2	13	26	GT	10	9
24343	2	16808315	16808340	2	13	26	TG	17	14
24345	2	16810655	16810682	2	14	28	GA	9	8
24346	2	16811164	16811185	2	11	22	GT	17	9
24352	2	16813884	16813907	2	12	24	GT	12	5
24355	2	16816288	16816299	2	6	12	TG	27	3
24356	2	16816789	16816812	2	12	24	GT	18	13
24357	2	16818272	16818283	2	6	12	GT	18	4
24361	2	16818560	16818571	2	6	12	TG	23	4
24362	2	16818706	16818737	2	16	32	TG	11	8
24366	2	16819715	16819730	2	8	16	AC	19	6
24372	2	16823563	16823584	2	11	22	CA	9	3
24374	2	16824549	16824570	2	11	22	GT	24	9
24379	2	16837168	16837179	2	6	12	TA	9	4
24380	2	16837678	16837691	2	7	14	GT	6	6
24382	2	16840573	16840598	2	13	26	AC	25	6
24383	2	16840841	16840870	2	15	30	AC	11	11
24384	2	16843184	16843195	2	6	12	GT	27	3
24387	2	16846248	16846261	2	7	14	AC	14	3
24388	2	16846267	16846282	2	8	16	CA	13	6
24389	2	16846756	16846773	3	6	18	ATT	15	5
24393	2	16855312	16855323	2	6	12	AC	20	4
24413	2	16896943	16896954	2	6	12	GT	18	4
24414	2	16896957	16896968	2	6	12	GT	21	3
24415	2	16896993	16897004	2	6	12	GT	22	3
24420	2	16905373	16905400	2	14	28	TG	19	7
24433	2	16927218	16927232	3	5	15	ATT	27	3

24434	2	16928153	16928182	2	15	30	GT	11	5
24435	2	16928359	16928370	2	6	12	GT	21	3
24452	2	16960166	16960197	4	8	32	TCCT	12	7
24453	2	16960474	16960497	4	6	24	TTGT	22	3
24458	2	16986056	16986083	4	7	28	TTTG	21	5
24462	2	16993465	16993502	2	19	38	TG	7	7
24466	2	16998702	16998722	3	7	21	GAA	20	9
24484	2	17040539	17040556	3	6	18	GAT	26	3
24496	2	17056685	17056716	2	16	32	GT	9	10
24505	2	17066226	17066239	2	7	14	GA	27	3
24506	2	17066822	17066845	2	12	24	GT	20	4
24507	2	17068902	17068913	2	6	12	CA	17	4
24508	2	17068916	17068931	2	8	16	TA	11	5
24509	2	17069013	17069046	2	17	34	CT	7	6
24513	2	17075346	17075363	2	9	18	AC	19	4
24514	2	17075454	17075469	2	8	16	TG	19	5
24517	2	17077403	17077440	2	19	38	TG	6	6
24519	2	17080624	17080645	2	11	22	TG	13	9
24520	2	17080711	17080722	2	6	12	CT	20	5
24521	2	17080724	17080739	2	8	16	TC	17	6
24526	2	17084934	17084957	2	12	24	AC	19	8
24532	2	17087457	17087472	4	4	16	CGTG	6	3
24538	2	17095798	17095817	2	10	20	TG	15	13
24539	2	17095819	17095834	2	8	16	GT	11	12
24543	2	17098463	17098480	2	9	18	TG	13	7
24544	2	17098857	17098894	2	19	38	GT	7	11
24547	2	17100035	17100066	2	16	32	GA	16	11
24548	2	17100445	17100468	2	12	24	TC	13	5
24551	2	17100660	17100683	2	12	24	AC	12	10
24554	2	17104620	17104639	2	10	20	GT	15	7
24563	2	17126877	17126890	2	7	14	TG	20	6
24564	2	17127079	17127094	2	8	16	TC	11	9
24569	2	17134315	17134330	2	8	16	CA	9	5
24570	2	17135372	17135401	2	15	30	CA	16	7
24577	2	17145432	17145446	3	5	15	GAG	25	4
24579	2	17154092	17154109	3	6	18	CCT	17	4
24581	2	17154690	17154701	2	6	12	TG	26	3
24584	2	17161306	17161321	4	4	16	GGAG	22	3
24590	2	17172578	17172595	2	9	18	TG	15	5
24591	2	17172626	17172649	3	8	24	CTC	22	4
24601	2	17189774	17189789	2	8	16	CA	23	6
24602	2	17189791	17189804	2	7	14	AC	20	3
24625	2	17244395	17244420	2	13	26	CA	24	11
24626	2	17246091	17246104	2	7	14	GT	24	3
24632	2	17257669	17257702	2	17	34	AC	13	12
24634	2	17261482	17261493	2	6	12	CT	9	9
24638	2	17271649	17271674	2	13	26	GT	11	6
24641	2	17273382	17273397	2	8	16	TG	18	3
24643	2	17275410	17275431	2	11	22	AC	9	7
24644	2	17278128	17278147	2	10	20	AC	18	10
24646	2	17278915	17278944	2	15	30	TG	9	5

24647	2	17281317	17281330	2	7	14	GC	31	5
24651	2	17283193	17283212	2	10	20	TG	16	9
24657	2	17288000	17288023	2	12	24	TG	8	8
24658	2	17288064	17288085	2	11	22	TG	11	4
24660	2	17289280	17289293	2	7	14	TG	25	7
24661	2	17289295	17289306	2	6	12	GT	16	3
24665	2	17297148	17297159	2	6	12	TG	21	6
24667	2	17308193	17308210	2	9	18	CA	6	7
24669	2	17308499	17308522	2	12	24	AC	17	11
24673	2	17313940	17313983	4	11	44	ATAG	10	10
24685	2	17329646	17329671	2	13	26	AC	18	9
24692	2	17341803	17341836	2	17	34	TG	13	10
24695	2	17353512	17353538	3	9	27	TGT	23	8
24702	2	17373656	17373669	2	7	14	CA	18	9
24703	2	17373757	17373780	2	12	24	TG	16	10
24705	2	17381278	17381297	2	10	20	TG	20	6
24707	2	17381520	17381533	2	7	14	CA	14	8
24712	2	17391880	17391909	3	10	30	AGC	25	11
24713	2	17392203	17392220	2	9	18	TG	13	7
24714	2	17392272	17392283	2	6	12	AG	18	4
24718	2	17393110	17393123	2	7	14	TC	24	4
24720	2	17396371	17396384	2	7	14	AG	24	3
24724	2	17404436	17404457	2	11	22	GT	7	7
24726	2	17404593	17404608	4	4	16	GAGG	10	4
24728	2	17406501	17406522	2	11	22	CA	20	11
24730	2	17408248	17408263	2	8	16	AC	27	4
24731	2	17408657	17408684	2	14	28	GT	12	6
24737	2	17412227	17412244	2	9	18	CA	29	3
24739	2	17412601	17412622	2	11	22	GT	20	6
24740	2	17412725	17412746	2	11	22	GT	18	4
24741	2	17413967	17413996	2	15	30	TG	16	9
24742	2	17414197	17414212	2	8	16	CA	24	9
24743	2	17414364	17414379	2	8	16	TG	26	8
24744	2	17415031	17415044	2	7	14	AC	24	7
24745	2	17415199	17415226	2	14	28	AG	14	5
24752	2	17428280	17428295	2	8	16	AT	11	6
24759	2	17439797	17439820	3	8	24	GAG	22	3
24760	2	17440049	17440066	2	9	18	GA	9	7
24773	2	17449402	17449419	2	9	18	CA	16	4
24774	2	17450233	17450258	2	13	26	TC	13	5
24776	2	17450451	17450478	2	14	28	GT	8	7
24777	2	17452412	17452427	2	8	16	GT	26	5
24782	2	17455857	17455874	2	9	18	CA	26	4
24784	2	17458578	17458589	2	6	12	TG	28	4
24785	2	17459160	17459177	2	9	18	TG	24	5
24786	2	17461586	17461597	2	6	12	CT	20	4
24787	2	17461631	17461642	2	6	12	TC	26	3
24790	2	17466721	17466746	2	13	26	AC	9	8
24795	2	17479889	17479920	2	16	32	CA	11	6
24796	2	17481165	17481178	2	7	14	TG	18	4
24797	2	17481549	17481566	2	9	18	GT	17	5

24800	2	17489947	17489982	2	18	36	TG	12	10
24801	2	17496872	17496889	2	9	18	TC	20	6
24802	2	17496891	17496904	2	7	14	CT	20	4
24805	2	17509900	17509917	3	6	18	TCA	24	3
24812	2	17528327	17528342	2	8	16	TG	22	4
24814	2	17535269	17535284	2	8	16	AC	19	3
24815	2	17538599	17538626	2	14	28	CA	7	10
24817	2	17539256	17539267	2	6	12	GT	23	4
24818	2	17539301	17539316	2	8	16	GT	20	4
24819	2	17541190	17541205	2	8	16	AC	13	8
24820	2	17541562	17541583	2	11	22	TG	9	8
24821	2	17543423	17543436	2	7	14	GT	29	4
24824	2	17549441	17549472	2	16	32	TG	16	11
24827	2	17555941	17555958	2	9	18	AG	26	3
24828	2	17556955	17556968	2	7	14	GT	15	3
24829	2	17561061	17561090	2	15	30	TG	16	7
24832	2	17563870	17563895	2	13	26	CA	21	12
24833	2	17564032	17564045	2	7	14	CA	21	4
24838	2	17574527	17574542	2	8	16	AC	23	11
24839	2	17574545	17574560	2	8	16	AC	22	10
24843	2	17578928	17578941	2	7	14	GT	24	4
24844	2	17579083	17579106	2	12	24	GT	6	10
24848	2	17584406	17584429	2	12	24	CA	17	7
24852	2	17592565	17592588	3	8	24	ATG	29	4
24860	2	17600220	17600251	2	16	32	TG	15	11
24862	2	17606870	17606897	2	14	28	TG	12	9
24863	2	17609194	17609209	2	8	16	CA	24	3
24866	2	17612080	17612107	2	14	28	CA	7	7
24868	2	17613330	17613343	2	7	14	TG	27	8
24873	2	17615449	17615474	2	13	26	TG	10	3
24877	2	17620685	17620702	2	9	18	AT	10	4
24878	2	17623779	17623798	2	10	20	TA	6	4
24883	2	17640814	17640837	3	8	24	GAG	29	3
24894	2	17662047	17662058	2	6	12	AC	23	4
24898	2	17676738	17676753	2	8	16	AC	28	5
24900	2	17683498	17683511	2	7	14	GT	23	15
24901	2	17687237	17687250	2	7	14	TG	22	3
24903	2	17689653	17689684	2	16	32	TG	10	10
24905	2	17697733	17697759	3	9	27	CTC	13	8
24908	2	17703093	17703106	2	7	14	CA	27	6
24911	2	17706020	17706045	2	13	26	AC	12	6
24916	2	17714192	17714205	2	7	14	AT	10	6
24917	2	17714338	17714355	2	9	18	TG	11	5
24929	2	17758408	17758440	3	11	33	TCA	28	8
24932	2	17767271	17767300	3	10	30	CCT	25	7
24936	2	17775224	17775238	3	5	15	CCT	25	3
24939	2	17784163	17784218	4	14	56	ATCT	9	5
24940	2	17786528	17786563	3	12	36	TGA	20	7
24941	2	17787067	17787081	3	5	15	CAA	29	5
24943	2	17791607	17791622	2	8	16	CT	28	3
24945	2	17799175	17799192	3	6	18	TTG	29	5

24948	2	17801982	17802001	4	5	20	AAAC	28	9
24949	2	17813235	17813261	3	9	27	CTC	20	4
24957	2	17830049	17830070	2	11	22	GT	13	8
24960	2	17859958	17859975	2	9	18	TG	6	9
24961	2	17864015	17864034	2	10	20	GT	22	5
24963	2	17869305	17869325	3	7	21	CTC	19	5
24964	2	17872675	17872686	2	6	12	TG	9	3
24965	2	17872688	17872703	2	8	16	GT	15	9
24975	2	17900898	17900930	3	11	33	GGA	22	7
24978	2	17909612	17909632	3	7	21	CAG	25	5
24979	2	17910717	17910740	3	8	24	CCT	19	4
25010	2	17950101	17950130	2	15	30	AG	6	5
25015	2	17958698	17958709	2	6	12	AC	16	3
25017	2	17960750	17960763	2	7	14	CA	24	3
25019	2	17962503	17962534	2	16	32	GT	21	14
25024	2	17970719	17970732	2	7	14	GA	17	3
25026	2	17971180	17971195	2	8	16	TG	19	4
25027	2	17971312	17971323	2	6	12	CA	24	5
25028	2	17972125	17972148	2	12	24	TC	12	8
25032	2	17973095	17973108	2	7	14	AC	20	5
25037	2	17990763	17990783	3	7	21	CAG	32	4
25038	2	17998411	17998432	2	11	22	GA	15	5
25039	2	17999157	17999170	2	7	14	CT	13	4
25040	2	18001300	18001321	2	11	22	AG	22	6
25043	2	18012451	18012474	2	12	24	TG	11	10
25045	2	18013251	18013270	4	5	20	ACAG	23	3
25048	2	18015264	18015283	2	10	20	TG	15	5
25063	2	18035051	18035076	2	13	26	TG	7	3
25083	2	18044860	18044909	2	25	50	CA	7	8
25085	2	18045637	18045654	2	9	18	GT	7	3
25089	2	18049835	18049850	2	8	16	TG	23	5
25090	2	18050822	18050869	4	12	48	ACAA	15	13
25094	2	18054667	18054682	2	8	16	GT	11	3
25096	2	18055764	18055784	3	7	21	CAA	25	9
25101	2	18065414	18065439	2	13	26	CA	13	9
25108	2	18079179	18079190	2	6	12	AT	11	5
25110	2	18081149	18081164	2	8	16	AC	23	8
25115	2	18084260	18084275	2	8	16	GT	22	16
25120	2	18088800	18088811	2	6	12	TG	33	3
25125	2	18100138	18100167	2	15	30	AC	23	12
25126	2	18101127	18101140	2	7	14	TG	23	3
25130	2	18107090	18107119	2	15	30	AG	6	5
25131	2	18108393	18108408	2	8	16	GT	19	5
25152	2	18126989	18127003	3	5	15	TCT	18	10
25154	2	18127355	18127366	2	6	12	AC	15	4
25155	2	18127368	18127381	2	7	14	CA	11	5
25156	2	18127630	18127653	2	12	24	GT	22	10
25157	2	18132373	18132392	2	10	20	CA	20	6
25163	2	18138239	18138266	2	14	28	AC	15	8
25166	2	18139902	18139923	2	11	22	GT	17	6
25171	2	18143781	18143794	2	7	14	TG	28	4

25181	2	18156293	18156314	2	11	22	TG	23	8
25184	2	18160958	18160981	2	12	24	TG	17	6
25188	2	18169754	18169777	4	6	24	GTGC	16	12
25191	2	18172549	18172612	4	16	64	ATCT	15	10
25204	2	18184836	18184871	2	18	36	GT	17	4
25205	2	18185214	18185225	2	6	12	TG	16	3
25207	2	18186638	18186663	2	13	26	CA	9	7
25213	2	18192008	18192027	4	5	20	AGCC	19	3
25216	2	18202907	18202924	2	9	18	TG	20	4
25222	2	18208080	18208111	2	16	32	GT	9	11
25224	2	18211285	18211304	2	10	20	TG	18	5
25227	2	18215251	18215262	2	6	12	CA	15	3
25228	2	18217447	18217476	2	15	30	GT	16	7
25229	2	18217732	18217767	2	18	36	GT	6	5
25231	2	18220216	18220235	2	10	20	CA	15	5
25232	2	18221668	18221719	4	13	52	CTTT	14	9
25233	2	18225182	18225203	2	11	22	AC	14	8
25236	2	18226362	18226373	2	6	12	TC	18	4
25242	2	18228686	18228715	2	15	30	TG	10	17
25243	2	18228755	18228766	2	6	12	GT	15	3
25248	2	18234314	18234325	2	6	12	GT	15	6
25249	2	18234327	18234344	2	9	18	TG	15	5
25254	2	18238355	18238378	2	12	24	CT	6	4
25256	2	18241781	18241804	2	12	24	TG	13	9
25257	2	18241983	18241994	2	6	12	TG	20	3
25258	2	18243686	18243699	2	7	14	AG	16	5
25260	2	18247073	18247094	2	11	22	TG	11	4
25262	2	18247433	18247456	2	12	24	TG	10	9
25274	2	18249085	18249111	3	9	27	TCA	13	4
25277	2	18251864	18251875	2	6	12	TC	26	4
25278	2	18253778	18253803	2	13	26	CA	25	5
25279	2	18254921	18254946	2	13	26	AG	28	5
25281	2	18255378	18255407	2	15	30	TG	15	9
25283	2	18258252	18258265	2	7	14	CA	22	8
25285	2	18258660	18258685	2	13	26	AC	25	6
25289	2	18265414	18265433	2	10	20	CA	22	6
25297	2	18276113	18276126	2	7	14	GT	19	6
25298	2	18276128	18276145	2	9	18	TG	22	7
25301	2	18280831	18280842	2	6	12	TG	26	5
25302	2	18281355	18281372	2	9	18	CA	15	14
25317	2	18313531	18313574	2	22	44	AG	8	8
25320	2	18320787	18320808	2	11	22	GT	28	12
25326	2	18336796	18336809	2	7	14	GT	24	6
25327	2	18339043	18339056	2	7	14	AG	18	3
25330	2	18346607	18346630	3	8	24	CTC	12	6
25331	2	18347258	18347273	4	4	16	GTGC	30	7
25334	2	18350054	18350074	3	7	21	GAG	32	5
25337	2	18352652	18352679	2	14	28	GT	17	6
25339	2	18356126	18356137	2	6	12	AC	24	4
25347	2	18382770	18382819	2	25	50	GT	21	9
25358	2	18402281	18402300	2	10	20	TG	8	3

25359	2	18403723	18403754	2	16	32	TG	16	11
25364	2	18419869	18419880	2	6	12	CA	24	3
25365	2	18420873	18420894	2	11	22	CT	15	6
25367	2	18420984	18420995	2	6	12	CA	20	3
25369	2	18421301	18421318	2	9	18	CA	17	10
25371	2	18423932	18423946	3	5	15	AAG	14	6
25377	2	18425039	18425072	2	17	34	AC	16	10
25379	2	18425161	18425184	2	12	24	CA	22	7
25382	2	18430271	18430294	2	12	24	CA	7	7
25384	2	18430948	18430961	2	7	14	TG	27	3
25385	2	18431178	18431201	3	8	24	AGG	23	4
25387	2	18432232	18432253	2	11	22	AC	16	4
25391	2	18439955	18439982	2	14	28	CA	15	7
25394	2	18446594	18446607	2	7	14	TG	20	3
25396	2	18452266	18452281	4	4	16	TTTC	24	3
25411	2	18480689	18480706	2	9	18	AG	16	9
25412	2	18480823	18480866	2	22	44	GT	6	9
25415	2	18486824	18486843	2	10	20	TG	14	10
25417	2	18488509	18488522	2	7	14	TG	15	3
25422	2	18493187	18493218	2	16	32	AC	12	15
25424	2	18494059	18494076	2	9	18	TG	14	8
25429	2	18509453	18509474	2	11	22	GA	21	10
25432	2	18511515	18511544	2	15	30	CA	15	8
25434	2	18515442	18515459	2	9	18	GT	19	6
25436	2	18526179	18526202	2	12	24	GT	6	8
25439	2	18536856	18536879	4	6	24	AATC	14	7
25441	2	18545383	18545400	3	6	18	TTA	18	4
25443	2	18549806	18549849	2	22	44	CA	6	7
25447	2	18560280	18560297	2	9	18	AC	24	8
25457	2	18603771	18603796	2	13	26	CA	9	6
25458	2	18605010	18605031	2	11	22	TG	9	6
25459	2	18611378	18611389	2	6	12	AC	28	4
25468	2	18619657	18619676	2	10	20	GT	24	10
25469	2	18620488	18620537	2	25	50	AC	8	11
25474	2	18627355	18627381	3	9	27	CCT	14	5
25476	2	18634703	18634714	2	6	12	TG	19	12
25477	2	18634716	18634727	2	6	12	GT	17	11
25478	2	18634874	18634903	2	15	30	CT	6	5
25479	2	18635177	18635194	2	9	18	AC	25	7
25480	2	18637004	18637019	2	8	16	AC	28	3
25489	2	18647812	18647833	2	11	22	TG	20	8
25498	2	18663588	18663605	2	9	18	TG	15	4
25499	2	18663691	18663718	2	14	28	GT	13	12
25500	2	18665349	18665369	3	7	21	GAG	16	4
25511	2	18678685	18678702	2	9	18	CA	22	8
25513	2	18678838	18678865	2	14	28	CA	11	4
25517	2	18679565	18679578	2	7	14	GT	20	7
25518	2	18679580	18679591	2	6	12	TG	18	4
25520	2	18680105	18680118	2	7	14	CT	26	5
25521	2	18680120	18680133	2	7	14	TC	26	4
25523	2	18684663	18684682	2	10	20	TG	23	8

25529	2	18689336	18689363	2	14	28	TG	15	9
25532	2	18700851	18700864	2	7	14	AC	19	6
25535	2	18721181	18721195	3	5	15	GAG	22	4
25536	2	18721196	18721210	3	5	15	CAG	17	4
25537	2	18721310	18721327	3	6	18	GAT	20	5
25538	2	18722046	18722066	3	7	21	GAG	16	7
25539	2	18735834	18735849	2	8	16	TG	25	7
25540	2	18737306	18737319	2	7	14	TG	16	8
25545	2	18750415	18750430	2	8	16	AC	26	7
25557	2	18771960	18771983	3	8	24	AAT	20	4
25558	2	18774034	18774051	2	9	18	TC	16	8
25559	2	18785140	18785151	2	6	12	TG	29	4
25563	2	18789812	18789845	2	17	34	CA	9	8
25569	2	18795777	18795812	3	12	36	GCA	31	10
25575	2	18811555	18811566	2	6	12	AG	27	4
25588	2	18847366	18847403	2	19	38	AG	7	6
25601	2	18900583	18900610	2	14	28	GA	9	6
25602	2	18900749	18900766	2	9	18	GA	20	3
25607	2	18914301	18914324	2	12	24	GT	18	10
25608	2	18915389	18915414	2	13	26	AC	11	7
25610	2	18921929	18921952	3	8	24	ACT	13	4
25612	2	18926230	18926243	2	7	14	CA	29	4
25624	2	18939155	18939166	2	6	12	GC	22	9
25625	2	18939167	18939182	2	8	16	AC	22	8
25632	2	18947768	18947799	2	16	32	GT	7	9
25639	2	18964630	18964641	2	6	12	AC	11	4
25658	2	18999533	18999544	2	6	12	AC	31	3
25662	2	19010025	19010045	3	7	21	GGA	24	3
25665	2	19010436	19010465	2	15	30	GA	6	8
25669	2	19011678	19011692	3	5	15	GCA	23	3
25680	2	19045928	19045943	4	4	16	TGTT	13	4
25681	2	19047220	19047231	2	6	12	TG	26	3
25685	2	19053176	19053196	3	7	21	CTT	21	15
25690	2	19067250	19067265	2	8	16	AT	18	5
25693	2	19075866	19075879	2	7	14	AT	10	4
25697	2	19077047	19077064	3	6	18	CTG	20	3
25702	2	19081578	19081599	2	11	22	GA	11	4
25708	2	19086770	19086787	2	9	18	TC	12	4
25709	2	19087263	19087288	2	13	26	CA	21	6
25715	2	19099407	19099418	2	6	12	CA	30	5
25725	2	19110970	19110985	2	8	16	TG	25	5
25726	2	19127822	19127839	2	9	18	CA	25	4
25727	2	19129742	19129753	2	6	12	TG	21	3
25735	2	19148735	19148748	2	7	14	AT	11	4
25738	2	19173449	19173460	2	6	12	GT	27	3
25739	2	19173553	19173572	2	10	20	GT	16	6
25749	2	19202335	19202350	4	4	16	ATTT	32	3
25751	2	19204157	19204180	4	6	24	ATGG	13	4
25752	2	19205821	19205834	2	7	14	GT	31	3
25754	2	19213006	19213045	4	10	40	TTTC	13	5
25765	2	19230072	19230091	2	10	20	AC	13	9

25767	2	19231287	19231300	2	7	14	AC	20	3
25774	2	19235972	19235987	2	8	16	GA	17	9
25775	2	19237856	19237889	2	17	34	AC	18	10
25778	2	19239252	19239267	4	4	16	GTCT	20	3
25779	2	19239408	19239422	3	5	15	GCT	26	11
25783	2	19239953	19239974	2	11	22	AG	11	5
25784	2	19240246	19240265	2	10	20	AC	16	15
25788	2	19241724	19241739	2	8	16	CA	17	8
25790	2	19243288	19243319	2	16	32	AC	12	6
25791	2	19243687	19243704	2	9	18	TG	19	3
25798	2	19254026	19254037	2	6	12	GT	28	3
25801	2	19271640	19271659	2	10	20	AC	22	10
25803	2	19277819	19277850	2	16	32	CA	18	8
25804	2	19278127	19278154	2	14	28	GT	12	9
25807	2	19284708	19284723	2	8	16	TA	17	4
25809	2	19285967	19285981	3	5	15	TTC	21	3
25811	2	19287473	19287492	2	10	20	CA	10	11
25814	2	19288736	19288753	2	9	18	CA	21	12
25817	2	19289356	19289376	3	7	21	CAG	34	10
25821	2	19292538	19292549	2	6	12	TC	36	4
25824	2	19301095	19301108	2	7	14	AC	30	3
25829	2	19315952	19315963	2	6	12	TG	19	6
25838	2	19345604	19345624	3	7	21	TTA	13	3
25842	2	19347034	19347053	4	5	20	TATG	18	3
25844	2	19352506	19352521	4	4	16	TTTC	28	3
25845	2	19360019	19360042	3	8	24	GAT	34	5
25848	2	19372851	19372865	3	5	15	AGG	23	3
25850	2	19373934	19373965	2	16	32	CT	15	16
25854	2	19374844	19374867	4	6	24	AGAA	19	9
25856	2	19377490	19377531	2	21	42	CA	12	11
25857	2	19377680	19377699	2	10	20	TG	23	6
25859	2	19380684	19380698	3	5	15	GTG	35	3
25861	2	19385240	19385251	2	6	12	CA	21	5
25862	2	19387950	19387965	2	8	16	TG	21	5
25864	2	19391295	19391314	2	10	20	AC	8	3
25872	2	19418421	19418448	4	7	28	TATG	25	5
25874	2	19426196	19426223	2	14	28	AC	11	13
25877	2	19427069	19427098	2	15	30	AG	10	7
25878	2	19437224	19437257	2	17	34	TG	7	4
25883	2	19444729	19444752	3	8	24	AGG	20	7
25885	2	19445929	19445948	2	10	20	GA	20	6
25893	2	19452180	19452201	2	11	22	AC	16	5
25894	2	19453548	19453573	2	13	26	TC	12	6
25903	2	19466943	19466958	4	4	16	TTCA	37	3
25910	2	19473667	19473688	2	11	22	CA	26	7
25912	2	19476298	19476329	2	16	32	TG	7	8
25918	2	19483995	19484016	2	11	22	GT	17	8
25919	2	19485749	19485763	3	5	15	GAG	27	4
25921	2	19487180	19487215	2	18	36	GT	12	12
25925	2	19490506	19490517	2	6	12	CA	20	7
25927	2	19494588	19494601	2	7	14	AC	15	4

25933	2	19495758	19495787	2	15	30	AC	19	13
25948	2	19505792	19505813	2	11	22	TG	23	10
25949	2	19507168	19507185	3	6	18	CTG	22	4
25950	2	19508137	19508162	2	13	26	TG	6	9
25951	2	19510572	19510605	2	17	34	TG	7	10
25952	2	19510783	19510798	4	4	16	GAGC	10	4
25953	2	19510954	19510969	2	8	16	TC	17	5
25954	2	19513381	19513398	2	9	18	AG	17	5
25956	2	19513638	19513665	2	14	28	AC	6	9
25959	2	19517913	19517928	2	8	16	AC	22	6
25960	2	19518151	19518172	2	11	22	GT	12	7
25962	2	19522760	19522781	2	11	22	GA	15	7
25965	2	19523257	19523288	2	16	32	CA	14	9
25972	2	19529459	19529470	2	6	12	AC	20	3
25975	2	19531103	19531114	2	6	12	TG	32	3
25976	2	19531302	19531335	2	17	34	TG	12	13
25977	2	19533685	19533710	2	13	26	GT	8	11
25985	2	19541506	19541523	3	6	18	ATG	25	7
25986	2	19543657	19543684	2	14	28	AC	9	10
25987	2	19543890	19543907	2	9	18	TG	14	6
26007	2	19553380	19553397	2	9	18	CA	17	5
26008	2	19553400	19553415	2	8	16	CA	19	7
26012	2	19557089	19557115	3	9	27	AGG	15	5
26026	2	19581373	19581386	2	7	14	AC	26	3
26030	2	19600128	19600141	2	7	14	TC	15	5
26031	2	19600142	19600157	2	8	16	AC	14	4
26033	2	19600356	19600369	2	7	14	GT	20	5
26046	2	19627840	19627855	2	8	16	TC	23	4
26054	2	19638257	19638268	2	6	12	AC	28	3
26057	2	19640698	19640711	2	7	14	TG	32	4
26061	2	19641877	19641902	2	13	26	CA	12	6
26066	2	19643666	19643697	2	16	32	AC	13	9
26067	2	19644369	19644382	2	7	14	AC	26	3
26068	2	19644543	19644560	2	9	18	AC	13	8
26070	2	19644967	19644980	2	7	14	CA	24	3
26071	2	19645033	19645068	2	18	36	CA	17	8
26072	2	19647249	19647269	3	7	21	CTC	30	4
26074	2	19648598	19648613	4	4	16	TGCG	16	5
26075	2	19648668	19648685	3	6	18	CTT	21	4
26079	2	19651106	19651127	2	11	22	AC	21	11
26081	2	19651966	19651989	2	12	24	AC	18	6
26084	2	19653895	19653906	2	6	12	TG	21	3
26085	2	19654027	19654046	2	10	20	AC	15	5
26096	2	19658571	19658604	2	17	34	GA	6	4
26102	2	19659504	19659521	3	6	18	GAG	11	4
26103	2	19659884	19659901	2	9	18	TG	25	4
26106	2	19660517	19660540	2	12	24	TG	9	4
26110	2	19662915	19662942	2	14	28	TG	12	9
26112	2	19665889	19665918	2	15	30	GT	14	10
26115	2	19667572	19667583	2	6	12	GT	27	3
26116	2	19667642	19667661	2	10	20	GT	15	14

26122	2	19669340	19669411	3	24	72	AGA	11	10
26126	2	19670684	19670695	2	6	12	TG	21	8
26134	2	19676464	19676481	2	9	18	GA	22	4
26135	2	19677296	19677315	4	5	20	TTTG	28	5
26137	2	19677935	19677946	2	6	12	AC	31	5
26141	2	19682629	19682652	3	8	24	CAG	16	5
26146	2	19687261	19687282	2	11	22	TG	19	7
26164	2	19694837	19694856	2	10	20	AC	12	5
26167	2	19696640	19696651	2	6	12	TC	25	3
26170	2	19697043	19697057	3	5	15	ACG	23	3
26171	2	19697293	19697316	2	12	24	GT	9	6
26172	2	19697636	19697653	2	9	18	TG	20	9
26176	2	19702771	19702800	2	15	30	TG	11	9
26179	2	19707610	19707631	2	11	22	CA	9	7
26190	2	19722207	19722227	3	7	21	GGA	13	3
26193	2	19728315	19728350	2	18	36	TG	6	6
26195	2	19730701	19730726	2	13	26	CA	17	8
26198	2	19736040	19736071	2	16	32	CA	11	8
26199	2	19737254	19737265	2	6	12	CA	26	5
26200	2	19737387	19737400	2	7	14	CT	26	3
26201	2	19737639	19737676	2	19	38	GT	7	10
26203	2	19739135	19739184	2	25	50	AC	7	9
26204	2	19739634	19739669	4	9	36	GCTG	24	8
26205	2	19745045	19745060	2	8	16	CA	18	5
26206	2	19745092	19745107	2	8	16	CA	14	4
26208	2	19746649	19746676	2	14	28	AC	15	6
26209	2	19746797	19746820	2	12	24	AC	8	10
26211	2	19748913	19748950	2	19	38	CA	10	9
26213	2	19753976	19753999	3	8	24	GGA	13	3
26217	2	19754479	19754500	2	11	22	AG	15	8
26218	2	19754793	19754820	2	14	28	TG	12	8
26227	2	19763245	19763260	2	8	16	CA	15	5
26228	2	19765743	19765756	2	7	14	GT	21	4
26229	2	19765938	19765957	2	10	20	AC	21	8
26230	2	19767463	19767496	2	17	34	GT	16	8
26233	2	19770154	19770165	2	6	12	AG	15	4
26239	2	19775154	19775193	2	20	40	CA	9	6
26240	2	19775744	19775775	2	16	32	TG	7	9
26242	2	19776572	19776589	2	9	18	CT	14	3
26254	2	19782914	19782927	2	7	14	CA	22	3
26259	2	19792268	19792289	2	11	22	TG	18	5
26260	2	19792457	19792470	2	7	14	TG	19	3
26261	2	19794742	19794785	2	22	44	GT	8	10
26263	2	19794966	19794987	2	11	22	TC	12	3
26270	2	19803274	19803285	2	6	12	GT	30	3
26272	2	19803500	19803541	2	21	42	GT	8	10
26283	2	19820422	19820471	2	25	50	AC	10	10
26285	2	19822241	19822256	2	8	16	GA	16	3
26286	2	19825013	19825036	3	8	24	TTA	13	4
26289	2	19832212	19832250	3	13	39	TGC	11	11
26293	2	19846870	19846887	2	9	18	CT	16	5

26301	2	19882566	19882579	2	7	14	GT	18	6
26308	2	19891531	19891548	2	9	18	GT	22	5
26309	2	19892490	19892543	2	27	54	CA	13	12
26310	2	19892676	19892691	2	8	16	CA	14	5
26319	2	19904980	19904991	2	6	12	TG	19	4
26320	2	19904994	19905005	2	6	12	TG	14	5
26321	2	19905093	19905132	2	20	40	TG	10	8
26324	2	19926710	19926759	2	25	50	TG	6	6
26328	2	19933419	19933472	2	27	54	TG	9	10
26331	2	19937502	19937527	2	13	26	CA	15	11
26332	2	19939768	19939791	2	12	24	TG	15	6
26333	2	19940644	19940671	2	14	28	CA	13	12
26334	2	19941111	19941128	2	9	18	CA	23	7
26336	2	19941271	19941282	2	6	12	CA	17	3
26347	2	19952680	19952699	2	10	20	GA	9	8
26348	2	19954793	19954814	2	11	22	AC	7	5
26349	2	19955506	19955517	2	6	12	AC	19	4
26350	2	19955689	19955718	2	15	30	CA	9	12
26356	2	19960520	19960533	2	7	14	GT	27	3
26359	2	19965098	19965113	2	8	16	CA	26	4
26369	2	19982462	19982497	2	18	36	AC	10	7
26372	2	19986947	19986972	2	13	26	GT	20	10
26375	2	19994926	19994955	2	15	30	TG	13	7
26390	2	20018064	20018083	2	10	20	TG	12	5
26395	2	20033890	20033904	3	5	15	AAC	14	4
26417	2	20128280	20128311	2	16	32	AG	6	6
26422	2	20139918	20139933	2	8	16	GT	10	3
26424	2	20140655	20140674	4	5	20	AAAC	22	4
26425	2	20143989	20144006	2	9	18	GT	22	6
26438	2	20176455	20176468	2	7	14	GT	24	6
26453	2	20207664	20207675	2	6	12	GT	25	5
26455	2	20225679	20225714	3	12	36	ATA	11	6
26458	2	20242304	20242355	4	13	52	TTTC	18	10
26459	2	20242509	20242529	3	7	21	CCT	21	6
26461	2	20244044	20244065	2	11	22	CA	13	4
26466	2	20247915	20247928	2	7	14	TG	6	7
26470	2	20252845	20252868	2	12	24	TG	15	7
26473	2	20255512	20255523	2	6	12	TG	6	3
26475	2	20255540	20255551	2	6	12	TG	16	3
26476	2	20255971	20256002	2	16	32	GT	6	8
26478	2	20260682	20260696	3	5	15	AGC	30	3
26482	2	20264959	20264984	2	13	26	AC	13	10
26483	2	20265734	20265757	2	12	24	TG	23	7
26484	2	20267753	20267768	2	8	16	AG	24	3
26485	2	20268521	20268541	3	7	21	GAA	17	10
26503	2	20288940	20288977	2	19	38	TG	7	10
26511	2	20289881	20289894	2	7	14	GA	8	5
26516	2	20290722	20290733	2	6	12	CT	20	4
26517	2	20290735	20290748	2	7	14	TC	22	3
26518	2	20290785	20290810	2	13	26	TG	13	11
26519	2	20290874	20290889	2	8	16	TG	21	10

26527	2	20293387	20293404	2	9	18	GT	13	6
26530	2	20295232	20295247	4	4	16	TTTG	23	5
26531	2	20295424	20295444	3	7	21	TCC	15	5
26536	2	20298834	20298859	2	13	26	GA	14	5
26541	2	20302341	20302354	2	7	14	CA	19	9
26542	2	20305284	20305297	2	7	14	CT	17	7
26543	2	20305299	20305312	2	7	14	TC	14	5
26544	2	20305564	20305577	2	7	14	GT	19	10
26546	2	20310057	20310072	2	8	16	AT	7	4
26549	2	20312848	20312873	2	13	26	TG	18	10
26550	2	20313050	20313111	2	31	62	GT	7	11
26557	2	20313665	20313678	2	7	14	GT	22	5
26559	2	20315448	20315465	2	9	18	GA	18	5
26561	2	20316419	20316432	2	7	14	GC	28	3
26567	2	20323224	20323237	2	7	14	TG	11	4
26569	2	20327161	20327176	4	4	16	AGAA	24	3
26570	2	20327219	20327240	2	11	22	AG	7	5
26572	2	20330261	20330278	2	9	18	CA	18	4
26578	2	20342152	20342163	2	6	12	CA	25	3
26579	2	20342665	20342680	2	8	16	TG	26	7
26586	2	20351939	20351954	2	8	16	AC	10	4
26588	2	20360976	20360990	3	5	15	CTC	29	3
26592	2	20364278	20364291	2	7	14	AC	22	3
26598	2	20367799	20367842	2	22	44	TG	10	8
26599	2	20371998	20372011	2	7	14	TC	33	3
26603	2	20388619	20388632	2	7	14	GT	11	6
26604	2	20391621	20391632	2	6	12	TG	31	3
26605	2	20393689	20393714	2	13	26	GT	14	8
26612	2	20395799	20395816	2	9	18	AC	23	5
26621	2	20420218	20420233	2	8	16	CA	20	7
26628	2	20431151	20431164	2	7	14	TG	29	6
26629	2	20433921	20433936	4	4	16	ATAC	9	3
26630	2	20435473	20435514	2	21	42	CA	15	8
26632	2	20437560	20437579	2	10	20	AG	23	5
26634	2	20440655	20440672	2	9	18	TG	23	4
26637	2	20443671	20443692	2	11	22	GT	24	5
26638	2	20444158	20444173	2	8	16	AC	19	4
26641	2	20447640	20447651	2	6	12	TG	18	3
26648	2	20464415	20464432	2	9	18	TG	21	6
26655	2	20476451	20476470	4	5	20	ACTG	29	4
26656	2	20476622	20476651	2	15	30	TG	14	10
26658	2	20478380	20478401	2	11	22	TG	8	12
26659	2	20478445	20478464	2	10	20	TG	20	8
26660	2	20478572	20478585	2	7	14	CA	22	6
26665	2	20483242	20483269	2	14	28	CA	20	16
26671	2	20504171	20504198	4	7	28	TTCA	22	6
26676	2	20513613	20513632	4	5	20	TGAA	18	7
26677	2	20530119	20530134	2	8	16	CA	14	7
26680	2	20536264	20536277	2	7	14	TG	23	3
26682	2	20537756	20537769	2	7	14	TC	24	3
26683	2	20543211	20543228	2	9	18	CT	17	8

26686	2	20547800	20547835	2	18	36	AC	23	7
26688	2	20553346	20553359	2	7	14	CT	21	4
26690	2	20553471	20553482	2	6	12	AG	19	4
26707	2	20572868	20572901	2	17	34	CT	10	9
26711	2	20608932	20608951	4	5	20	TTTC	16	4
26719	2	20628099	20628130	2	16	32	TG	18	9
26730	2	20656389	20656409	3	7	21	CTT	17	5
26734	2	20663520	20663531	2	6	12	TG	21	4
26735	2	20663567	20663588	2	11	22	GT	13	9
26736	2	20669572	20669585	2	7	14	GA	24	6
26737	2	20672757	20672774	3	6	18	TCC	28	3
26742	2	20683166	20683185	2	10	20	AC	20	7
26743	2	20683322	20683337	2	8	16	AC	20	3
26745	2	20683816	20683837	2	11	22	TG	7	3
26746	2	20685392	20685429	2	19	38	CA	11	7
26747	2	20687511	20687534	2	12	24	CA	26	6
26748	2	20688257	20688272	2	8	16	GT	30	3
26749	2	20689314	20689325	2	6	12	CT	25	3
26750	2	20689328	20689341	2	7	14	CT	22	3
26751	2	20691079	20691102	2	12	24	CA	8	8
26759	2	20703416	20703433	2	9	18	TG	12	7
26763	2	20710791	20710806	2	8	16	CA	21	10
26764	2	20716445	20716458	2	7	14	GT	8	3
26769	2	20719077	20719096	2	10	20	CA	8	8
26774	2	20720093	20720140	2	24	48	TG	9	8
26777	2	20721789	20721802	2	7	14	CA	13	10
26778	2	20727582	20727597	2	8	16	CT	22	3
26779	2	20728045	20728056	2	6	12	AC	21	3
26782	2	20731394	20731407	2	7	14	AC	22	6
26784	2	20734965	20734979	3	5	15	GAA	22	5
26785	2	20741620	20741637	2	9	18	AC	6	11
26786	2	20741792	20741827	2	18	36	CA	11	8
26787	2	20743493	20743506	2	7	14	AC	22	3
26788	2	20743595	20743610	2	8	16	TG	17	4
26789	2	20746601	20746620	4	5	20	CTTC	22	4
26802	2	20765864	20765875	2	6	12	AC	32	6
26806	2	20769426	20769437	2	6	12	GA	19	6
26808	2	20771097	20771108	2	6	12	CA	25	4
26809	2	20775577	20775592	2	8	16	AC	20	4
26810	2	20775801	20775816	2	8	16	AC	10	8
26812	2	20776879	20776900	2	11	22	TG	25	8
26813	2	20777085	20777114	2	15	30	CA	18	13
26814	2	20777904	20777924	3	7	21	AGC	27	8
26816	2	20784878	20784889	2	6	12	CA	27	5
26818	2	20788603	20788620	2	9	18	CT	11	10
26824	2	20796486	20796505	2	10	20	AG	15	4
26826	2	20800400	20800413	2	7	14	TG	9	8
26831	2	20801622	20801641	2	10	20	TG	11	7
26839	2	20806781	20806806	2	13	26	CA	15	5
26840	2	20808748	20808761	2	7	14	AC	26	5
26841	2	20809140	20809169	2	15	30	TG	14	9

26842	2	20809349	20809374	2	13	26	AC	12	5
26850	2	20817616	20817667	2	26	52	CA	6	9
26851	2	20817780	20817809	2	15	30	CA	13	13
26852	2	20821944	20821961	2	9	18	GA	19	5
26853	2	20822117	20822130	2	7	14	GA	21	3
26858	2	20824504	20824517	2	7	14	GT	17	4
26859	2	20828565	20828580	2	8	16	GA	29	11
26861	2	20829222	20829265	2	22	44	AG	17	9
26862	2	20837190	20837207	2	9	18	CA	16	5
26871	2	20842329	20842346	2	9	18	TC	24	5
26873	2	20847152	20847187	2	18	36	TG	18	14
26884	2	20853010	20853039	2	15	30	GT	23	3
26887	2	20853820	20853849	2	15	30	CT	16	10
26888	2	20854043	20854066	2	12	24	CA	16	8
26889	2	20854153	20854178	2	13	26	AC	11	7
26892	2	20854498	20854527	2	15	30	AC	19	9
26893	2	20854620	20854635	2	8	16	CA	12	10
26894	2	20854865	20854886	2	11	22	GA	19	4
26896	2	20856790	20856809	2	10	20	GT	15	8
26901	2	20860718	20860737	2	10	20	TG	19	7
26902	2	20860803	20860824	2	11	22	CA	15	11
26904	2	20860981	20861016	2	18	36	CA	17	11
26909	2	20864835	20864854	2	10	20	TC	11	6
26910	2	20864883	20864898	2	8	16	GT	22	4
26911	2	20865584	20865611	2	14	28	CA	21	14
26912	2	20865771	20865782	2	6	12	AC	26	3
26913	2	20866693	20866712	2	10	20	AG	10	8
26914	2	20866916	20866931	2	8	16	TG	17	11
26915	2	20866933	20866948	2	8	16	GT	14	9
26916	2	20869669	20869688	2	10	20	AC	19	5
26926	2	20880449	20880474	2	13	26	AC	22	5
26941	2	20888802	20888831	2	15	30	GT	20	8
26942	2	20888985	20888998	2	7	14	CA	12	5
26943	2	20889814	20889845	2	16	32	AC	14	9
26946	2	20891982	20892005	2	12	24	GT	11	10
26948	2	20892341	20892362	2	11	22	AC	14	5
26949	2	20892560	20892579	2	10	20	AC	17	4
26950	2	20892985	20893006	2	11	22	TC	13	4
26952	2	20896076	20896089	2	7	14	CA	27	3
26955	2	20898817	20898842	2	13	26	AC	17	11
26960	2	20902254	20902283	2	15	30	CA	13	12
26976	2	20919230	20919251	2	11	22	AC	14	11
26977	2	20920891	20920902	2	6	12	TA	21	5
26981	2	20923190	20923213	2	12	24	AC	15	9
26983	2	20924476	20924491	4	4	16	AGGA	17	3
26986	2	20926493	20926510	2	9	18	AC	6	3
26987	2	20927165	20927192	2	14	28	GA	10	6
26990	2	20928107	20928118	2	6	12	GT	11	4
27001	2	20939060	20939071	2	6	12	TG	26	5
27004	2	20943936	20943949	2	7	14	CA	15	3
27018	2	20968082	20968117	2	18	36	TG	13	9

27022	2	20971348	20971365	2	9	18	GT	10	4
27024	2	20972139	20972158	2	10	20	AG	22	5
27028	2	20977013	20977030	2	9	18	GT	8	6
27030	2	20977486	20977499	2	7	14	GT	18	6
27033	2	20983269	20983288	2	10	20	TG	6	6
27035	2	20983435	20983446	2	6	12	CT	27	3
27038	2	20986329	20986342	2	7	14	GT	23	3
27042	2	20989790	20989809	2	10	20	GT	10	5
27045	2	20993196	20993209	2	7	14	CT	18	4
27047	2	20997080	20997097	2	9	18	AC	13	8
27052	2	21003960	21003980	3	7	21	GGT	21	3
27060	2	21034312	21034331	4	5	20	ATGG	28	10
27064	2	21042569	21042580	2	6	12	TG	24	4
27065	2	21042719	21042734	2	8	16	GT	23	10
27066	2	21044245	21044265	3	7	21	TCA	24	5
27069	2	21047883	21047894	2	6	12	TC	16	3
27075	2	21058007	21058028	2	11	22	CA	19	5
27076	2	21063004	21063021	3	6	18	CCT	18	6
27077	2	21075215	21075242	4	7	28	AAAG	18	13
27078	2	21085729	21085750	2	11	22	AC	12	6
27086	2	21092658	21092672	3	5	15	GTT	22	3
27087	2	21093778	21093793	2	8	16	AC	26	4
27088	2	21094112	21094131	4	5	20	ATCT	27	4
27089	2	21094314	21094365	4	13	52	TAGA	23	12
27090	2	21095352	21095363	2	6	12	GA	25	4
27091	2	21095713	21095730	3	6	18	AGC	25	6
27092	2	21096692	21096712	3	7	21	CAG	22	5
27095	2	21099457	21099476	2	10	20	GT	20	5
27096	2	21099731	21099752	2	11	22	GT	9	4
27101	2	21101007	21101022	2	8	16	GT	21	3
27109	2	21130236	21130247	2	6	12	AC	17	8
27110	2	21130340	21130359	2	10	20	AC	18	10
27111	2	21131992	21132003	2	6	12	AC	34	3
27112	2	21132108	21132123	2	8	16	CA	17	7
27129	2	21151468	21151481	2	7	14	GA	15	4
27130	2	21151609	21151626	2	9	18	TG	19	3
27133	2	21153200	21153217	2	9	18	TG	19	7
27138	2	21155586	21155605	2	10	20	CA	24	9
27142	2	21156076	21156097	2	11	22	CT	11	7
27143	2	21156128	21156143	2	8	16	CT	14	3
27144	2	21156478	21156491	2	7	14	TG	17	4
27145	2	21156493	21156508	2	8	16	GT	11	6
27148	2	21165899	21165914	2	8	16	TG	21	4
27152	2	21166997	21167020	3	8	24	CTC	20	3
27155	2	21171435	21171460	2	13	26	CT	17	4
27156	2	21172421	21172442	2	11	22	TC	21	7
27157	2	21172632	21172651	2	10	20	CA	19	9
27158	2	21176629	21176642	2	7	14	GT	17	4
27162	2	21177912	21177935	2	12	24	TG	24	14
27163	2	21178087	21178102	4	4	16	ACTG	32	3
27174	2	21183859	21183876	2	9	18	CA	13	3

27182	2	21189925	21189942	2	9	18	TG	13	5
27185	2	21192038	21192067	2	15	30	CA	19	14
27191	2	21197655	21197666	2	6	12	GT	22	8
27192	2	21197871	21197888	2	9	18	GT	21	6
27194	2	21199990	21200009	2	10	20	GA	15	5
27196	2	21200391	21200411	3	7	21	CTG	26	4
27198	2	21203113	21203140	2	14	28	AG	12	5
27200	2	21203438	21203451	2	7	14	AC	13	3
27201	2	21203561	21203586	2	13	26	CT	13	5
27202	2	21203638	21203657	2	10	20	AC	10	5
27208	2	21210164	21210177	2	7	14	TC	12	3
27215	2	21220416	21220429	2	7	14	AG	10	4
27216	2	21220430	21220447	2	9	18	TG	8	4
27218	2	21220471	21220482	2	6	12	GA	27	3
27221	2	21225500	21225525	2	13	26	TG	14	10
27228	2	21229348	21229369	2	11	22	TG	21	8
27234	2	21232499	21232514	2	8	16	CA	23	8
27235	2	21232872	21232903	2	16	32	TG	10	10
27242	2	21234072	21234095	2	12	24	CA	11	12
27243	2	21234714	21234749	2	18	36	GT	6	5
27261	2	21249696	21249711	2	8	16	CT	19	5
27263	2	21249753	21249768	2	8	16	CT	29	4
27264	2	21249956	21250003	3	16	48	TTG	22	7
27275	2	21291638	21291665	2	14	28	AC	9	8
27278	2	21313270	21313285	4	4	16	CAAT	22	3
27292	2	21329022	21329036	3	5	15	TGC	8	3
27295	2	21335439	21335478	4	10	40	TGGA	19	11
27296	2	21338535	21338550	4	4	16	AAAT	14	3
27299	2	21345354	21345399	2	23	46	TG	27	4
27303	2	21351560	21351577	3	6	18	GAA	21	3
27311	2	21375105	21375120	4	4	16	AGCT	28	3
27317	2	21394794	21394811	2	9	18	TG	21	11
27320	2	21399954	21399980	3	9	27	TTC	23	4
27323	2	21403649	21403662	2	7	14	TG	25	3
27326	2	21415740	21415753	2	7	14	TG	30	4
27327	2	21416434	21416445	2	6	12	GT	27	3
27331	2	21429361	21429372	2	6	12	TG	26	13
27332	2	21429536	21429550	3	5	15	GCA	24	4
27339	2	21438776	21438793	2	9	18	AC	18	8
27342	2	21440560	21440573	2	7	14	TG	29	5
27345	2	21441988	21442023	3	12	36	CAA	14	8
27347	2	21445528	21445539	2	6	12	TA	15	5
27354	2	21453351	21453374	2	12	24	AG	23	8
27359	2	21462980	21462997	2	9	18	GA	6	3
27360	2	21468238	21468253	2	8	16	AC	18	5
27364	2	21469479	21469500	2	11	22	TC	12	3
27366	2	21470329	21470344	2	8	16	GT	15	4
27369	2	21472183	21472196	2	7	14	TC	20	6
27376	2	21486825	21486840	2	8	16	CA	8	5
27393	2	21552129	21552142	2	7	14	TG	21	5
27416	2	21595055	21595082	2	14	28	CA	14	5

27419	2	21598823	21598858	3	12	36	ATT	8	4
27429	2	21627892	21627919	2	14	28	AC	17	7
27435	3	3615	3632	3	6	18	ATA	15	6
27437	3	21534	21565	2	16	32	CA	11	13
27440	3	26028	26039	2	6	12	GT	19	17
27443	3	26325	26357	3	11	33	ACC	10	9
27445	3	32647	32668	2	11	22	TG	19	9
27450	3	36668	36687	2	10	20	TG	25	3
27451	3	36906	36923	2	9	18	CA	21	4
27460	3	68403	68414	2	6	12	TG	16	3
27466	3	80495	80516	2	11	22	GT	19	9
27467	3	87149	87174	2	13	26	CA	19	16
27472	3	104665	104678	2	7	14	AC	19	4
27473	3	104681	104692	2	6	12	AC	26	7
27479	3	107520	107531	2	6	12	CA	25	4
27480	3	107668	107705	2	19	38	GT	11	12
27481	3	108365	108376	2	6	12	TG	17	7
27484	3	114693	114706	2	7	14	AC	26	9
27485	3	115632	115647	2	8	16	GT	21	3
27487	3	124835	124864	2	15	30	GT	10	13
27488	3	124972	125001	2	15	30	GT	19	12
27489	3	125271	125288	2	9	18	GA	19	10
27492	3	128382	128425	2	22	44	AC	13	14
27493	3	128809	128832	2	12	24	CA	10	5
27497	3	131255	131272	2	9	18	CA	21	8
27499	3	132206	132221	2	8	16	TG	6	10
27500	3	132363	132378	2	8	16	AC	8	6
27501	3	132383	132396	2	7	14	AC	7	5
27503	3	133794	133811	2	9	18	GT	23	6
27506	3	137719	137738	4	5	20	ATGG	33	4
27507	3	137782	137793	2	6	12	AG	29	3
27508	3	138098	138113	2	8	16	TC	19	3
27515	3	142609	142626	3	6	18	AGA	18	4
27516	3	142766	142793	2	14	28	CA	13	9
27519	3	144078	144091	2	7	14	CA	27	13
27520	3	144906	144947	2	21	42	AC	9	10
27521	3	150572	150589	2	9	18	GA	24	4
27523	3	152029	152040	2	6	12	TG	35	3
27524	3	153386	153419	2	17	34	CT	8	9
27525	3	154030	154043	2	7	14	GT	18	4
27526	3	154045	154056	2	6	12	TG	20	7
27527	3	155339	155356	2	9	18	CT	21	5
27528	3	155535	155564	3	10	30	GAA	34	8
27531	3	159773	159804	2	16	32	CA	14	13
27534	3	162914	162939	2	13	26	TG	15	8
27535	3	167918	167949	2	16	32	TG	9	10
27537	3	172953	172974	2	11	22	TG	24	11
27543	3	176681	176728	2	24	48	AC	11	15
27547	3	183325	183342	2	9	18	CA	16	4
27550	3	184105	184136	2	16	32	TG	10	14
27555	3	201081	201098	2	9	18	CA	17	9

27556	3	201258	201269	2	6	12	TG	22	3
27560	3	202309	202330	2	11	22	CT	23	5
27562	3	207108	207127	2	10	20	TG	10	13
27565	3	213177	213200	2	12	24	TG	22	5
27566	3	215328	215349	2	11	22	CA	10	3
27567	3	215435	215449	3	5	15	GAG	29	8
27568	3	216379	216412	2	17	34	TG	16	7
27569	3	216572	216591	2	10	20	AC	17	14
27572	3	221494	221529	2	18	36	GT	6	8
27573	3	221705	221720	2	8	16	CA	17	6
27578	3	233764	233778	3	5	15	TGG	15	5
27580	3	260569	260586	3	6	18	CAG	26	6
27581	3	265001	265064	4	16	64	AGAT	14	7
27582	3	268420	268453	2	17	34	GT	12	8
27584	3	270666	270686	3	7	21	TCC	17	4
27587	3	271834	271857	2	12	24	CT	19	4
27593	3	278340	278379	4	10	40	CATC	27	7
27594	3	280842	280853	2	6	12	TC	26	3
27595	3	283540	283553	2	7	14	TG	22	3
27596	3	283713	283738	2	13	26	AC	18	9
27601	3	304605	304632	4	7	28	TACA	16	6
27605	3	322740	322757	2	9	18	AC	20	14
27608	3	330681	330706	2	13	26	GT	20	15
27609	3	334902	334913	2	6	12	AC	27	5
27616	3	351926	351941	2	8	16	TA	22	4
27617	3	354429	354458	3	10	30	CTC	19	4
27622	3	374716	374749	2	17	34	TG	11	13
27625	3	378385	378408	2	12	24	AC	11	6
27626	3	378610	378631	2	11	22	CA	11	11
27627	3	378738	378755	2	9	18	AC	21	5
27631	3	380250	380273	2	12	24	TG	11	11
27634	3	381126	381145	2	10	20	TC	20	4
27639	3	389679	389690	2	6	12	GT	24	3
27641	3	391827	391848	2	11	22	AC	18	5
27642	3	391903	391928	2	13	26	AC	22	9
27643	3	393405	393420	2	8	16	TG	17	4
27646	3	397835	397880	2	23	46	AC	23	15
27647	3	397952	397987	2	18	36	TG	11	11
27649	3	402178	402193	2	8	16	TG	16	5
27655	3	407410	407424	3	5	15	TTG	24	5
27681	3	485668	485685	3	6	18	TAT	13	3
27682	3	488046	488065	4	5	20	AAAT	15	3
27690	3	532289	532302	2	7	14	GT	22	3
27691	3	533989	534003	3	5	15	AAT	23	5
27693	3	540101	540132	2	16	32	CA	6	5
27694	3	541019	541060	2	21	42	CA	14	18
27695	3	542938	542953	2	8	16	TG	22	10
27696	3	543597	543628	2	16	32	AC	13	9
27703	3	549378	549393	2	8	16	AC	25	5
27704	3	549990	550003	2	7	14	TG	28	4
27707	3	556752	556797	2	23	46	TG	16	10

27708	3	556977	557012	2	18	36	TG	10	7
27709	3	557223	557242	2	10	20	TG	22	6
27710	3	557490	557503	2	7	14	TG	22	3
27711	3	558264	558295	2	16	32	TG	18	13
27722	3	571216	571231	2	8	16	TC	22	5
27723	3	575580	575593	2	7	14	GA	26	4
27725	3	578536	578553	2	9	18	TG	15	5
27727	3	603573	603587	3	5	15	ACA	25	4
27729	3	616344	616369	2	13	26	CA	22	5
27730	3	618215	618226	2	6	12	GT	14	3
27731	3	618579	618590	2	6	12	AC	25	5
27735	3	625141	625158	2	9	18	GT	24	3
27736	3	631324	631341	2	9	18	AT	12	4
27740	3	639841	639866	2	13	26	GT	14	10
27742	3	642887	642934	2	24	48	TG	11	15
27743	3	646724	646745	2	11	22	CA	22	7
27744	3	646891	646922	2	16	32	TG	15	10
27745	3	647485	647508	2	12	24	TG	9	8
27751	3	653147	653170	2	12	24	GT	9	13
27755	3	654970	654981	2	6	12	AC	28	4
27757	3	656557	656577	3	7	21	CAG	15	4
27759	3	658316	658333	2	9	18	AG	11	8
27766	3	665262	665283	2	11	22	GT	8	4
27767	3	665544	665563	2	10	20	GT	18	6
27768	3	665728	665753	2	13	26	GT	6	6
27774	3	681086	681097	2	6	12	CA	15	8
27775	3	681099	681112	2	7	14	AC	17	7
27781	3	685274	685299	2	13	26	AC	13	11
27790	3	697433	697446	2	7	14	TA	20	5
27791	3	699441	699462	2	11	22	CA	22	13
27792	3	699940	699961	2	11	22	GT	21	7
27795	3	704509	704544	4	9	36	AGAC	19	10
27796	3	704648	704663	2	8	16	GA	16	6
27802	3	716057	716072	2	8	16	AC	23	4
27803	3	716265	716302	2	19	38	TG	11	12
27807	3	719121	719144	4	6	24	CAGA	9	4
27808	3	719682	719699	2	9	18	TG	18	11
27809	3	719806	719831	2	13	26	AC	17	15
27810	3	720178	720197	2	10	20	TC	22	4
27811	3	720259	720285	3	9	27	CTT	22	6
27815	3	720824	720851	2	14	28	GT	21	9
27818	3	722940	722969	2	15	30	GT	27	18
27819	3	723169	723188	2	10	20	CA	16	8
27822	3	725609	725636	2	14	28	CA	10	9
27823	3	729402	729415	2	7	14	AG	27	3
27824	3	729515	729526	2	6	12	TG	20	4
27825	3	730269	730286	3	6	18	TCC	14	6
27828	3	734473	734502	3	10	30	CTG	28	7
27829	3	736197	736244	2	24	48	GT	10	15
27830	3	739691	739704	2	7	14	GA	21	3
27831	3	742352	742369	3	6	18	AGG	23	4

27834	3	748605	748624	2	10	20	TG	25	6
27836	3	750937	750976	4	10	40	TGGA	29	12
27843	3	754410	754437	2	14	28	AC	13	8
27847	3	755597	755648	2	26	52	GT	9	10
27848	3	755771	755800	2	15	30	TG	12	7
27850	3	757780	757815	2	18	36	CA	11	15
27851	3	762803	762824	2	11	22	AC	21	7
27854	3	764149	764206	2	29	58	TG	18	14
27861	3	769086	769099	2	7	14	GT	27	9
27862	3	771883	771903	3	7	21	GAG	23	3
27869	3	778162	778182	3	7	21	AGG	26	7
27872	3	779179	779194	2	8	16	AC	29	4
27880	3	791264	791277	2	7	14	TG	28	3
27882	3	794874	794893	2	10	20	GT	23	9
27885	3	795443	795464	2	11	22	GT	14	7
27887	3	800624	800637	2	7	14	AG	25	4
27888	3	800753	800768	2	8	16	TG	24	4
27890	3	804042	804057	4	4	16	AAAT	31	4
27894	3	806868	806881	2	7	14	CT	31	11
27895	3	808666	808691	2	13	26	CA	15	10
27897	3	809880	809905	2	13	26	TG	20	8
27898	3	810066	810085	2	10	20	AC	30	6
27899	3	810831	810850	2	10	20	TG	9	10
27902	3	811391	811404	2	7	14	CA	29	6
27912	3	844125	844144	4	5	20	TTTC	27	3
27916	3	850528	850559	2	16	32	AC	19	9
27917	3	856274	856287	2	7	14	CT	24	4
27919	3	856863	856894	2	16	32	CA	13	6
27922	3	860104	860119	2	8	16	GT	27	6
27924	3	870789	870804	2	8	16	CA	20	12
27925	3	870983	871010	2	14	28	AC	9	6
27926	3	874542	874573	4	8	32	TATT	19	9
27927	3	874718	874743	2	13	26	GT	21	6
27929	3	876046	876079	2	17	34	CA	17	10
27931	3	877133	877166	2	17	34	TG	13	8
27936	3	877747	877760	2	7	14	CT	9	5
27937	3	878116	878143	2	14	28	AC	19	8
27938	3	880165	880180	2	8	16	CT	24	7
27939	3	880383	880394	2	6	12	TG	13	6
27941	3	881502	881527	2	13	26	CA	16	12
27945	3	888611	888624	2	7	14	AG	28	3
27946	3	890339	890354	4	4	16	GACA	20	9
27947	3	890359	890374	4	4	16	GACA	8	3
27949	3	890466	890481	2	8	16	AC	29	3
27952	3	896057	896080	3	8	24	CTG	30	9
27953	3	899889	899920	2	16	32	TG	21	14
27954	3	901030	901061	2	16	32	TG	14	8
27958	3	907686	907705	4	5	20	AAAT	11	5
27959	3	909209	909222	2	7	14	AC	26	5
27961	3	920548	920561	2	7	14	AC	32	6
27962	3	923609	923634	2	13	26	GT	17	8

27963	3	925834	925849	2	8	16	GT	23	5
27965	3	927565	927586	2	11	22	AC	13	4
27971	3	941463	941490	2	14	28	TG	7	8
27972	3	941647	941658	2	6	12	GT	21	7
27974	3	945453	945470	2	9	18	CA	28	3
27976	3	962075	962089	3	5	15	CGC	25	3
27978	3	968703	968722	2	10	20	AC	20	8
27979	3	969779	969790	2	6	12	TG	32	3
27982	3	972712	972725	2	7	14	AC	17	5
27985	3	974148	974171	2	12	24	AC	15	9
27986	3	974925	974950	2	13	26	GT	21	5
27987	3	975204	975227	2	12	24	CA	20	8
27988	3	975421	975432	2	6	12	CA	25	5
27990	3	978110	978151	2	21	42	TG	14	7
27992	3	978258	978279	2	11	22	CA	10	4
27993	3	978398	978415	2	9	18	TG	12	3
27998	3	983879	983914	2	18	36	GT	9	11
27999	3	987269	987298	2	15	30	TG	16	11
28002	3	996618	996631	2	7	14	TG	19	3
28005	3	1008721	1008738	3	6	18	GCT	30	8
28010	3	1016435	1016458	4	6	24	TTTA	18	9
28011	3	1018951	1018978	2	14	28	GT	12	7
28013	3	1027605	1027618	2	7	14	CA	12	5
28014	3	1028879	1028906	2	14	28	CA	22	18
28026	3	1086448	1086473	2	13	26	GT	15	11
28027	3	1087096	1087121	2	13	26	GT	16	10
28036	3	1097354	1097377	4	6	24	AAAG	14	4
28039	3	1100834	1100849	4	4	16	AAAG	21	5
28040	3	1101176	1101191	2	8	16	TG	21	8
28041	3	1101997	1102038	3	14	42	GGA	6	5
28044	3	1102967	1103012	2	23	46	TG	8	8
28045	3	1105507	1105536	2	15	30	TG	12	7
28046	3	1105760	1105789	2	15	30	TG	6	7
28055	3	1116812	1116829	3	6	18	CAC	21	8
28059	3	1118567	1118584	2	9	18	TG	19	5
28060	3	1118641	1118652	2	6	12	AC	34	3
28061	3	1118860	1118873	2	7	14	TG	29	5
28062	3	1119023	1119038	2	8	16	AC	22	7
28068	3	1125119	1125154	2	18	36	GT	15	10
28069	3	1125751	1125786	2	18	36	CA	11	6
28076	3	1126529	1126558	2	15	30	TG	7	6
28078	3	1126908	1126921	2	7	14	TC	15	4
28080	3	1127070	1127084	3	5	15	TCC	20	4
28082	3	1127872	1127887	2	8	16	TG	23	4
28084	3	1129886	1129905	2	10	20	CA	16	8
28085	3	1130133	1130148	2	8	16	GT	25	3
28086	3	1130255	1130278	2	12	24	AC	20	10
28087	3	1131622	1131633	2	6	12	AC	17	3
28090	3	1133649	1133662	2	7	14	TC	17	3
28091	3	1133676	1133687	2	6	12	CT	11	4
28094	3	1133981	1134012	2	16	32	AC	9	7

28095	3	1134918	1134951	2	17	34	TG	18	9
28099	3	1136573	1136590	2	9	18	GT	6	6
28101	3	1143116	1143136	3	7	21	CTC	14	6
28103	3	1145807	1145820	2	7	14	TG	29	3
28104	3	1146579	1146604	2	13	26	AC	12	7
28105	3	1146649	1146674	2	13	26	AC	11	10
28106	3	1148525	1148536	2	6	12	AT	19	3
28110	3	1169071	1169108	2	19	38	GT	12	9
28112	3	1174470	1174483	2	7	14	AC	28	5
28113	3	1178177	1178192	2	8	16	AC	28	11
28119	3	1187384	1187401	3	6	18	GCT	16	4
28120	3	1191496	1191515	2	10	20	CA	12	13
28121	3	1192966	1192979	2	7	14	CA	18	5
28122	3	1196840	1196851	2	6	12	AC	18	3
28123	3	1197092	1197115	2	12	24	GT	7	8
28132	3	1197947	1197966	2	10	20	CA	14	9
28135	3	1200952	1200969	2	9	18	CA	20	9
28136	3	1201938	1201963	2	13	26	CA	8	6
28138	3	1203948	1203963	2	8	16	CA	19	8
28145	3	1221883	1221898	2	8	16	TG	16	5
28148	3	1234236	1234287	2	26	52	AC	9	9
28149	3	1237922	1237933	2	6	12	TG	27	3
28153	3	1247093	1247104	2	6	12	TG	27	9
28157	3	1259700	1259715	2	8	16	CA	20	4
28158	3	1260605	1260624	4	5	20	AAAT	8	3
28163	3	1269338	1269349	2	6	12	AG	12	4
28164	3	1269351	1269368	2	9	18	GA	22	8
28169	3	1276830	1276845	2	8	16	AC	20	4
28170	3	1278878	1278895	2	9	18	TG	17	4
28172	3	1282476	1282489	2	7	14	CT	17	3
28175	3	1283730	1283761	2	16	32	CA	28	10
28176	3	1284922	1284939	2	9	18	AC	21	10
28177	3	1285839	1285854	2	8	16	CA	14	15
28183	3	1293202	1293219	2	9	18	CA	22	6
28184	3	1294101	1294118	2	9	18	GT	7	7
28188	3	1294393	1294428	2	18	36	TG	11	11
28193	3	1300735	1300748	2	7	14	TG	22	7
28195	3	1301114	1301145	2	16	32	AC	12	12
28196	3	1301304	1301325	2	11	22	AC	13	13
28198	3	1301555	1301570	2	8	16	CA	20	9
28199	3	1302741	1302756	4	4	16	AAAG	25	5
28200	3	1310139	1310168	2	15	30	CA	6	7
28202	3	1312949	1312960	2	6	12	GT	7	4
28207	3	1328458	1328471	2	7	14	GT	27	5
28213	3	1344244	1344257	2	7	14	GA	22	3
28214	3	1345053	1345070	2	9	18	GT	23	15
28216	3	1346636	1346663	2	14	28	TG	17	12
28218	3	1348032	1348043	2	6	12	TG	26	10
28219	3	1349401	1349421	3	7	21	TGA	23	4
28220	3	1350502	1350513	2	6	12	AC	19	10
28221	3	1350516	1350531	2	8	16	AC	19	10

28222	3	1354289	1354302	2	7	14	TG	14	7
28226	3	1357431	1357446	2	8	16	GT	9	3
28227	3	1357492	1357517	2	13	26	CA	10	12
28231	3	1373765	1373798	2	17	34	CA	10	7
28232	3	1375144	1375163	2	10	20	GA	15	7
28233	3	1375289	1375320	2	16	32	AC	17	7
28237	3	1380588	1380605	2	9	18	TG	20	7
28239	3	1385155	1385168	2	7	14	TG	31	7
28241	3	1385442	1385457	2	8	16	AC	25	3
28242	3	1385530	1385547	2	9	18	AC	26	8
28243	3	1385803	1385820	2	9	18	AG	24	6
28246	3	1386309	1386330	2	11	22	AC	19	5
28247	3	1391882	1391903	2	11	22	TG	16	10
28249	3	1394321	1394340	2	10	20	GT	16	11
28254	3	1395306	1395323	3	6	18	GCT	30	3
28256	3	1397933	1397947	3	5	15	GGA	12	3
28258	3	1400757	1400780	2	12	24	TG	11	11
28260	3	1406369	1406384	2	8	16	TG	23	3
28261	3	1406413	1406442	2	15	30	GT	11	10
28262	3	1406595	1406618	2	12	24	CA	13	11
28265	3	1410119	1410136	2	9	18	TG	20	4
28268	3	1410687	1410712	2	13	26	CA	8	7
28269	3	1410952	1410973	2	11	22	CA	16	6
28274	3	1418858	1418885	2	14	28	CA	11	16
28275	3	1418986	1419011	2	13	26	TG	21	10
28277	3	1419238	1419267	2	15	30	GA	12	12
28278	3	1419526	1419561	2	18	36	CA	15	7
28279	3	1423414	1423445	2	16	32	GA	8	7
28282	3	1425490	1425507	2	9	18	GA	22	4
28285	3	1435021	1435046	2	13	26	AC	13	7
28289	3	1441436	1441469	2	17	34	TG	22	12
28292	3	1448548	1448561	2	7	14	TG	19	6
28295	3	1451407	1451434	2	14	28	CA	15	11
28296	3	1452714	1452735	2	11	22	TG	17	7
28297	3	1453385	1453396	2	6	12	AC	19	6
28306	3	1462298	1462315	3	6	18	GGA	18	4
28308	3	1469166	1469193	2	14	28	TG	19	11
28317	3	1476289	1476314	2	13	26	TG	14	13
28318	3	1476473	1476484	2	6	12	TG	19	9
28319	3	1476712	1476727	2	8	16	CA	19	9
28326	3	1487023	1487036	2	7	14	AC	25	8
28327	3	1487038	1487053	2	8	16	CA	18	8
28328	3	1488058	1488077	2	10	20	TG	12	7
28329	3	1491844	1491863	2	10	20	TG	10	7
28330	3	1492633	1492648	2	8	16	GT	25	5
28331	3	1492855	1492882	2	14	28	TG	16	8
28332	3	1493194	1493221	2	14	28	TG	7	6
28333	3	1495524	1495535	2	6	12	CA	16	11
28334	3	1496240	1496255	2	8	16	AC	12	5
28335	3	1496257	1496276	2	10	20	CA	10	7
28336	3	1496390	1496427	2	19	38	CA	8	9

28338	3	1498834	1498849	2	8	16	CT	15	6
28342	3	1499214	1499227	2	7	14	GA	15	9
28343	3	1499362	1499387	2	13	26	AC	18	11
28344	3	1505757	1505768	2	6	12	AT	19	4
28347	3	1506210	1506225	2	8	16	GT	21	11
28351	3	1510573	1510596	2	12	24	GA	7	4
28352	3	1510622	1510643	2	11	22	AG	10	4
28354	3	1510818	1510831	2	7	14	TC	14	6
28358	3	1511300	1511321	2	11	22	GT	11	7
28359	3	1511418	1511445	2	14	28	TG	11	10
28364	3	1512163	1512176	2	7	14	AC	8	11
28365	3	1512178	1512195	2	9	18	CA	11	12
28372	3	1524837	1524866	2	15	30	CA	13	9
28373	3	1524973	1525000	2	14	28	TG	17	18
28374	3	1525856	1525893	2	19	38	AC	12	7
28375	3	1527986	1528009	2	12	24	CA	13	15
28379	3	1534314	1534335	2	11	22	CA	15	6
28381	3	1535980	1535993	2	7	14	TG	21	5
28384	3	1543023	1543036	2	7	14	AC	20	4
28390	3	1566590	1566603	2	7	14	GT	19	5
28391	3	1566714	1566747	2	17	34	TG	8	10
28401	3	1594985	1595028	2	22	44	AC	9	13
28404	3	1600757	1600770	2	7	14	AC	28	3
28406	3	1604271	1604290	4	5	20	AAAG	25	3
28407	3	1607392	1607403	2	6	12	AT	12	3
28409	3	1608025	1608036	2	6	12	GT	13	8
28412	3	1613330	1613351	2	11	22	GT	27	10
28415	3	1617325	1617336	2	6	12	AC	18	3
28416	3	1617869	1617882	2	7	14	CA	25	4
28417	3	1617890	1617901	2	6	12	AT	23	5
28418	3	1620843	1620864	2	11	22	TG	7	4
28420	3	1622516	1622527	2	6	12	GT	25	3
28427	3	1626067	1626086	2	10	20	AC	10	8
28439	3	1635154	1635173	2	10	20	TG	13	7
28442	3	1638499	1638522	2	12	24	CT	21	10
28445	3	1641819	1641830	2	6	12	TG	33	4
28449	3	1644494	1644511	3	6	18	CTA	15	3
28450	3	1648148	1648165	2	9	18	AC	28	4
28452	3	1652628	1652645	2	9	18	TG	27	3
28454	3	1656825	1656842	2	9	18	GA	14	5
28456	3	1660367	1660381	3	5	15	CGG	30	5
28457	3	1660690	1660743	3	18	54	GAA	7	9
28464	3	1663161	1663172	2	6	12	TG	25	4
28466	3	1667813	1667828	2	8	16	AC	15	11
28467	3	1667864	1667877	2	7	14	CA	19	7
28470	3	1678527	1678558	2	16	32	TG	10	11
28471	3	1682203	1682230	2	14	28	TG	11	7
28472	3	1682454	1682479	2	13	26	CA	14	14
28476	3	1683297	1683326	2	15	30	TG	26	19
28483	3	1699343	1699366	2	12	24	GT	22	13
28484	3	1699669	1699680	2	6	12	GT	27	3

28485	3	1699911	1699950	2	20	40	CA	12	7
28486	3	1700065	1700082	2	9	18	CA	16	8
28491	3	1707043	1707062	2	10	20	TG	7	6
28492	3	1707166	1707183	2	9	18	GT	21	4
28495	3	1714504	1714539	2	18	36	TG	13	9
28499	3	1716257	1716272	2	8	16	GT	27	10
28501	3	1724981	1725004	2	12	24	AC	9	6
28505	3	1727949	1727970	2	11	22	GT	15	10
28507	3	1728445	1728480	2	18	36	CA	6	10
28508	3	1728594	1728619	2	13	26	CA	13	12
28509	3	1729385	1729402	3	6	18	ATC	27	4
28513	3	1732954	1732969	4	4	16	TGTC	23	4
28514	3	1733126	1733137	2	6	12	AC	27	6
28515	3	1733477	1733500	3	8	24	TCC	21	7
28517	3	1733948	1733983	2	18	36	CA	14	11
28518	3	1734108	1734121	2	7	14	CA	15	11
28520	3	1735925	1735940	2	8	16	TG	23	4
28521	3	1737085	1737106	2	11	22	CA	15	10
28527	3	1738752	1738778	3	9	27	GAG	17	4
28528	3	1738959	1738978	2	10	20	GT	20	11
28530	3	1743167	1743196	2	15	30	GT	6	5
28531	3	1745407	1745432	2	13	26	TG	13	10
28533	3	1747667	1747684	2	9	18	TG	19	9
28534	3	1747817	1747842	2	13	26	TG	14	8
28537	3	1749691	1749716	2	13	26	TG	12	8
28540	3	1752394	1752423	2	15	30	GT	25	13
28543	3	1754156	1754181	2	13	26	CA	15	5
28545	3	1754986	1755007	2	11	22	CA	12	6
28546	3	1758328	1758349	2	11	22	CA	19	8
28550	3	1763769	1763788	4	5	20	ATTT	23	3
28551	3	1765722	1765745	2	12	24	GT	15	11
28552	3	1765869	1765898	2	15	30	TG	15	9
28555	3	1769884	1769895	2	6	12	GT	22	6
28556	3	1772749	1772776	2	14	28	CA	16	13
28558	3	1775775	1775786	2	6	12	GT	22	8
28559	3	1776044	1776065	2	11	22	CA	26	5
28560	3	1781083	1781094	2	6	12	GT	30	3
28562	3	1781423	1781434	2	6	12	GT	9	7
28563	3	1781538	1781557	2	10	20	CT	9	4
28567	3	1782928	1782959	2	16	32	TG	9	7
28568	3	1783147	1783180	2	17	34	CA	11	7
28569	3	1783282	1783297	2	8	16	TG	25	12
28571	3	1783970	1783997	2	14	28	AG	14	6
28572	3	1784391	1784414	2	12	24	AC	17	11
28576	3	1787843	1787860	2	9	18	CA	21	9
28577	3	1788038	1788065	2	14	28	CA	13	11
28579	3	1789855	1789866	2	6	12	TG	21	3
28581	3	1790749	1790772	2	12	24	AC	19	8
28583	3	1790989	1791016	2	14	28	CA	9	8
28587	3	1791355	1791380	2	13	26	AC	13	5
28588	3	1794586	1794625	4	10	40	AGAC	16	10

28593	3	1802540	1802561	2	11	22	AC	11	8
28595	3	1802629	1802654	2	13	26	AC	7	7
28596	3	1802832	1802847	2	8	16	AG	22	3
28598	3	1804004	1804019	2	8	16	AC	24	5
28599	3	1804743	1804762	4	5	20	AAAT	8	4
28602	3	1805338	1805359	2	11	22	CA	16	9
28603	3	1812244	1812259	2	8	16	CA	24	8
28605	3	1818768	1818803	2	18	36	AC	13	10
28606	3	1821560	1821589	2	15	30	CA	19	7
28612	3	1827647	1827674	2	14	28	TG	12	13
28621	3	1830886	1830905	2	10	20	GT	23	15
28622	3	1830907	1830918	2	6	12	TG	20	14
28626	3	1838046	1838085	2	20	40	AC	10	11
28627	3	1838303	1838328	2	13	26	CA	14	6
28630	3	1839044	1839055	2	6	12	AC	22	5
28632	3	1842917	1842928	2	6	12	AT	18	12
28633	3	1847484	1847497	2	7	14	GT	17	7
28637	3	1849496	1849517	2	11	22	CT	15	13
28639	3	1850149	1850180	2	16	32	AC	13	6
28641	3	1850591	1850608	2	9	18	TC	20	3
28642	3	1850730	1850757	2	14	28	CA	17	5
28648	3	1859546	1859559	2	7	14	TG	18	3
28649	3	1859652	1859665	2	7	14	CT	18	5
28653	3	1863091	1863105	3	5	15	CAT	28	7
28656	3	1865745	1865774	2	15	30	CA	8	4
28659	3	1874919	1874948	2	15	30	CA	16	11
28661	3	1875062	1875073	2	6	12	CA	15	4
28662	3	1875193	1875204	2	6	12	CA	19	5
28663	3	1875336	1875349	2	7	14	CA	16	9
28664	3	1875487	1875500	2	7	14	CA	19	4
28669	3	1876907	1876922	2	8	16	AC	19	4
28670	3	1877082	1877099	2	9	18	TG	20	11
28671	3	1878726	1878755	2	15	30	GA	16	15
28672	3	1881697	1881720	3	8	24	GGA	21	5
28674	3	1887298	1887309	2	6	12	TC	25	3
28678	3	1902596	1902615	4	5	20	ATTG	12	8
28679	3	1902688	1902699	2	6	12	CT	20	3
28686	3	1917248	1917265	2	9	18	GT	23	4
28688	3	1928361	1928372	2	6	12	TG	15	4
28689	3	1930576	1930597	2	11	22	TG	29	10
28697	3	1941467	1941482	2	8	16	AC	25	5
28698	3	1942009	1942042	2	17	34	TG	15	14
28699	3	1942366	1942395	2	15	30	TG	20	9
28705	3	1956274	1956307	2	17	34	AC	9	12
28706	3	1958448	1958477	2	15	30	AC	10	9
28707	3	1962778	1962797	2	10	20	AC	21	8
28709	3	1963102	1963137	2	18	36	CT	6	5
28712	3	1963447	1963466	2	10	20	GT	20	9
28713	3	1963585	1963598	2	7	14	AC	26	3
28714	3	1965136	1965185	2	25	50	GT	11	13
28715	3	1965559	1965582	2	12	24	GT	15	13

28716	3	1968038	1968052	3	5	15	GGA	12	3
28722	3	1974283	1974298	4	4	16	TGTT	22	5
28723	3	1974495	1974508	2	7	14	GA	29	4
28725	3	1975559	1975573	3	5	15	TTG	23	3
28732	3	1977800	1977814	3	5	15	CAT	31	4
28737	3	1985027	1985040	2	7	14	GT	8	3
28738	3	1985437	1985458	2	11	22	GT	20	7
28742	3	1988668	1988739	4	18	72	TCTT	10	7
28747	3	2002642	2002683	2	21	42	GT	11	12
28748	3	2002936	2002956	3	7	21	TCC	22	4
28749	3	2003052	2003065	2	7	14	CA	21	6
28750	3	2003164	2003179	2	8	16	CA	20	3
28751	3	2008471	2008508	2	19	38	TG	10	13
28754	3	2009429	2009460	2	16	32	AC	16	8
28755	3	2009674	2009689	2	8	16	TG	13	10
28757	3	2011899	2011920	2	11	22	GT	20	12
28758	3	2012094	2012121	2	14	28	CA	12	6
28762	3	2017297	2017316	2	10	20	TG	9	5
28764	3	2017501	2017512	2	6	12	GT	10	5
28765	3	2017514	2017531	2	9	18	TG	23	5
28766	3	2020005	2020040	2	18	36	AC	8	5
28767	3	2021185	2021200	2	8	16	AC	31	4
28769	3	2022831	2022858	2	14	28	CT	15	5
28770	3	2022974	2022987	2	7	14	CA	22	3
28771	3	2032975	2032995	3	7	21	GAG	17	9
28774	3	2034610	2034635	2	13	26	GT	13	11
28778	3	2043862	2043877	2	8	16	GT	26	5
28782	3	2050325	2050346	2	11	22	TG	13	9
28784	3	2050523	2050568	2	23	46	CA	13	13
28787	3	2053298	2053321	2	12	24	TG	8	9
28788	3	2054804	2054830	3	9	27	AAT	12	10
28791	3	2056452	2056465	2	7	14	GT	23	4
28792	3	2057181	2057194	2	7	14	TG	23	4
28795	3	2060245	2060278	2	17	34	TG	11	10
28797	3	2062175	2062190	2	8	16	AC	16	3
28798	3	2062497	2062514	2	9	18	GT	20	8
28809	3	2101027	2101038	2	6	12	AC	27	6
28835	3	2153059	2153082	2	12	24	CA	10	7
28841	3	2163581	2163596	4	4	16	TTTA	13	6
28844	3	2168895	2168912	2	9	18	AC	19	3
28847	3	2172800	2172815	4	4	16	CAAA	20	6
28851	3	2176428	2176445	2	9	18	AC	17	5
28860	3	2191548	2191559	2	6	12	GT	12	4
28866	3	2202643	2202670	2	14	28	CA	11	8
28867	3	2202987	2203006	2	10	20	GT	19	4
28869	3	2205111	2205128	3	6	18	GGT	26	4
28872	3	2208578	2208599	2	11	22	AC	18	6
28874	3	2209580	2209599	2	10	20	TG	13	8
28879	3	2217858	2217873	2	8	16	TG	23	4
28885	3	2229242	2229275	2	17	34	TG	18	11
28886	3	2230777	2230788	2	6	12	AC	14	4

28887	3	2231268	2231285	2	9	18	AC	19	4
28888	3	2233846	2233859	2	7	14	AC	23	3
28890	3	2234093	2234104	2	6	12	CA	7	4
28892	3	2234967	2234978	2	6	12	TG	23	4
28895	3	2247192	2247225	2	17	34	GA	7	10
28897	3	2254572	2254585	2	7	14	AC	26	3
28903	3	2255936	2255965	2	15	30	CA	7	9
28904	3	2256101	2256116	2	8	16	GT	18	12
28907	3	2260056	2260079	2	12	24	GT	12	7
28909	3	2260328	2260371	2	22	44	TG	7	7
28910	3	2263259	2263274	2	8	16	AC	18	4
28914	3	2271114	2271129	2	8	16	GT	18	3
28920	3	2287865	2287890	2	13	26	GT	14	10
28921	3	2288104	2288123	2	10	20	AC	17	7
28923	3	2290906	2290929	4	6	24	GATG	33	3
28924	3	2292092	2292103	2	6	12	CA	20	3
28926	3	2308100	2308119	4	5	20	AAAT	27	3
28927	3	2311233	2311258	2	13	26	TC	6	5
28930	3	2311614	2311639	2	13	26	AC	8	8
28932	3	2311982	2312001	2	10	20	TG	8	5
28938	3	2319165	2319186	2	11	22	CA	14	8
28940	3	2323517	2323552	3	12	36	GAG	8	4
28946	3	2327133	2327152	2	10	20	GT	22	10
28948	3	2329192	2329206	3	5	15	AAT	23	4
28950	3	2334176	2334191	2	8	16	TG	25	11
28953	3	2340593	2340608	2	8	16	GT	13	4
28954	3	2343808	2343827	2	10	20	TC	10	9
28955	3	2343966	2343997	2	16	32	CA	6	9
28956	3	2344369	2344388	2	10	20	AC	25	5
28960	3	2351488	2351502	3	5	15	GAA	24	4
28963	3	2356796	2356825	2	15	30	TG	13	10
28969	3	2363189	2363206	2	9	18	CA	19	10
28972	3	2363825	2363844	2	10	20	GT	15	12
28973	3	2364311	2364338	2	14	28	TG	15	12
28974	3	2366622	2366643	2	11	22	GT	17	8
28979	3	2368321	2368352	4	8	32	AGAA	24	9
28988	3	2375569	2375604	2	18	36	TG	8	9
28997	3	2390648	2390681	2	17	34	CA	18	10
28999	3	2392541	2392564	2	12	24	CA	17	15
29000	3	2392707	2392726	2	10	20	AC	15	8
29004	3	2395620	2395631	2	6	12	CA	14	3
29008	3	2422121	2422140	2	10	20	TG	25	6
29015	3	2437858	2437871	2	7	14	TG	15	4
29016	3	2437910	2437923	2	7	14	GT	26	5
29019	3	2441477	2441492	2	8	16	CA	25	11
29020	3	2445372	2445399	2	14	28	GT	12	11
29021	3	2445557	2445572	2	8	16	CA	20	5
29024	3	2448652	2448675	2	12	24	TG	20	12
29032	3	2489197	2489211	3	5	15	GTG	31	5
29045	3	2514611	2514624	2	7	14	TG	15	5
29048	3	2519813	2519844	2	16	32	TC	17	11

29055	3	2542615	2542626	2	6	12	GT	18	3
29057	3	2542653	2542664	2	6	12	TG	13	3
29058	3	2544675	2544701	3	9	27	GAG	14	4
29059	3	2544800	2544817	3	6	18	TGC	20	7
29062	3	2545291	2545316	2	13	26	CT	20	7
29063	3	2545485	2545500	2	8	16	GT	24	7
29064	3	2547004	2547039	3	12	36	TGA	23	8
29065	3	2547397	2547436	2	20	40	AC	8	10
29068	3	2553273	2553290	2	9	18	GT	17	4
29076	3	2565276	2565299	2	12	24	TG	18	10
29077	3	2568353	2568364	2	6	12	TG	19	11
29078	3	2571960	2572005	2	23	46	TG	11	11
29079	3	2573631	2573645	3	5	15	TTG	27	3
29081	3	2578360	2578389	2	15	30	CA	20	12
29086	3	2589425	2589440	2	8	16	AC	26	6
29087	3	2592430	2592445	4	4	16	AACG	29	3
29088	3	2594493	2594518	2	13	26	TG	22	6
29089	3	2594919	2594958	2	20	40	CA	12	10
29092	3	2597714	2597735	2	11	22	CA	12	8
29096	3	2607414	2607445	2	16	32	GT	8	11
29106	3	2629698	2629715	2	9	18	TG	17	9
29107	3	2629842	2629881	2	20	40	GT	7	8
29108	3	2630322	2630353	2	16	32	CA	20	13
29113	3	2636827	2636847	3	7	21	CCT	24	3
29114	3	2639510	2639524	3	5	15	TTC	30	3
29115	3	2639528	2639542	3	5	15	TTA	30	3
29121	3	2647431	2647444	2	7	14	AC	21	3
29122	3	2647677	2647688	2	6	12	GT	22	8
29123	3	2650268	2650287	2	10	20	AC	15	7
29124	3	2650427	2650440	2	7	14	CA	28	3
29127	3	2654114	2654127	2	7	14	CT	20	11
29129	3	2654261	2654274	2	7	14	AC	19	5
29131	3	2658402	2658419	2	9	18	AC	21	9
29132	3	2658421	2658434	2	7	14	CA	17	9
29133	3	2659761	2659774	2	7	14	GA	20	8
29134	3	2662425	2662436	2	6	12	TG	26	5
29135	3	2663384	2663413	2	15	30	TG	18	7
29140	3	2674960	2674973	2	7	14	AC	23	7
29144	3	2682789	2682812	2	12	24	CA	26	11
29149	3	2696124	2696155	2	16	32	TG	10	7
29153	3	2709437	2709448	2	6	12	GT	26	3
29154	3	2710196	2710225	2	15	30	TG	16	11
29156	3	2712939	2712990	2	26	52	CA	9	11
29166	3	2731867	2731882	2	8	16	AT	23	5
29170	3	2740884	2740909	2	13	26	GT	11	10
29176	3	2755018	2755065	2	24	48	GT	7	10
29177	3	2756478	2756503	2	13	26	GT	16	14
29185	3	2772733	2772766	2	17	34	GT	9	8
29191	3	2795769	2795782	2	7	14	CA	28	5
29193	3	2829630	2829653	3	8	24	TGC	24	8
29201	3	2863967	2863986	2	10	20	CA	11	11

29208	3	2917395	2917408	2	7	14	TC	26	9
29209	3	2917411	2917424	2	7	14	TC	28	6
29227	3	2953877	2953896	2	10	20	GA	23	7
29228	3	2959191	2959214	2	12	24	CA	21	7
29229	3	2960379	2960396	3	6	18	AGC	24	3
29234	3	2974595	2974606	2	6	12	TG	22	3
29237	3	2980143	2980162	2	10	20	AC	17	8
29248	3	2999931	2999948	2	9	18	CT	20	6
29249	3	3000390	3000416	3	9	27	AGG	14	5
29250	3	3000531	3000548	2	9	18	CT	10	7
29251	3	3000551	3000566	2	8	16	CT	6	5
29252	3	3001318	3001331	2	7	14	AC	12	7
29253	3	3001337	3001350	2	7	14	GA	8	3
29255	3	3006108	3006128	3	7	21	TTC	26	4
29264	3	3026421	3026450	2	15	30	GT	9	6
29270	3	3037066	3037117	4	13	52	CCAT	27	13
29273	3	3039409	3039429	3	7	21	AAT	14	4
29298	3	3094964	3094997	2	17	34	CA	10	12
29308	3	3114967	3114978	2	6	12	AC	16	5
29315	3	3137989	3138004	4	4	16	TTAT	18	8
29320	3	3157605	3157620	2	8	16	CA	22	3
29321	3	3159956	3159975	4	5	20	TATT	21	3
29331	3	3177725	3177766	2	21	42	TG	12	12
29332	3	3178338	3178357	2	10	20	TG	15	4
29333	3	3178566	3178587	2	11	22	TG	25	4
29352	3	3214058	3214081	4	6	24	AAAT	27	5
29359	3	3225019	3225034	2	8	16	AT	7	3
29372	3	3257396	3257416	3	7	21	ACA	23	4
29382	3	3286833	3286872	4	10	40	TCCA	30	11
29386	3	3297292	3297341	2	25	50	TG	7	7
29391	3	3308992	3309003	2	6	12	AG	27	3
29398	3	3329039	3329066	2	14	28	CA	11	7
29404	3	3339174	3339197	2	12	24	AC	18	6
29406	3	3347975	3348000	2	13	26	AC	9	12
29410	3	3354970	3354983	2	7	14	GT	19	9
29411	3	3354984	3355001	2	9	18	GA	20	11
29413	3	3362746	3362766	3	7	21	TAA	15	8
29414	3	3363222	3363237	2	8	16	TC	18	4
29415	3	3365680	3365691	2	6	12	AT	23	4
29421	3	3371889	3371906	2	9	18	AC	17	4
29426	3	3385790	3385803	2	7	14	CA	21	7
29429	3	3387738	3387763	2	13	26	TG	20	8
29433	3	3396215	3396230	2	8	16	CA	18	4
29436	3	3398526	3398543	2	9	18	CA	14	6
29438	3	3399611	3399626	2	8	16	TG	10	3
29441	3	3400724	3400753	2	15	30	TC	7	8
29443	3	3400930	3400950	3	7	21	CTG	26	3
29458	3	3462835	3462850	2	8	16	CA	6	7
29461	3	3467488	3467505	3	6	18	GCT	30	3
29466	3	3485538	3485553	4	4	16	ATTT	23	6
29474	3	3511648	3511668	3	7	21	CTG	21	5

29475	3	3514003	3514029	3	9	27	AAG	31	6
29484	3	3543635	3543654	4	5	20	GTTT	20	3
29493	3	3577803	3577820	3	6	18	TCC	12	3
29494	3	3578411	3578436	2	13	26	TG	12	7
29495	3	3580844	3580859	2	8	16	CA	21	9
29497	3	3582306	3582325	2	10	20	AC	10	9
29527	3	3671487	3671504	2	9	18	CA	22	6
29531	3	3688841	3688896	2	28	56	GT	8	10
29535	3	3709449	3709464	2	8	16	AC	6	6
29536	3	3709467	3709482	2	8	16	AC	9	9
29537	3	3710812	3710831	2	10	20	GT	14	4
29539	3	3720841	3720856	2	8	16	AC	27	7
29543	3	3729261	3729276	4	4	16	GCAG	21	5
29544	3	3729288	3729303	4	4	16	GGCA	23	5
29546	3	3733947	3733970	2	12	24	AC	6	7
29552	3	3776848	3776863	2	8	16	AT	14	5
29559	3	3798916	3798947	2	16	32	CA	17	6
29568	3	3834166	3834217	4	13	52	TATC	10	6
29574	3	3853930	3853949	4	5	20	TATT	11	3
29575	3	3855284	3855315	2	16	32	TG	9	8
29580	3	3865287	3865307	3	7	21	CCT	22	4
29584	3	3879090	3879105	2	8	16	CA	21	5
29597	3	3906312	3906329	2	9	18	AC	26	5
29605	3	3916666	3916685	2	10	20	CT	19	8
29608	3	3918767	3918780	2	7	14	AG	18	4
29609	3	3919019	3919036	2	9	18	GT	14	8
29614	3	3927730	3927747	3	6	18	GTT	25	5
29615	3	3927783	3927800	3	6	18	TGT	20	5
29616	3	3927932	3927976	3	15	45	CTG	19	11
29617	3	3928177	3928200	3	8	24	GCT	25	8
29618	3	3928231	3928248	3	6	18	GCT	18	4
29622	3	3931178	3931217	4	10	40	TGGA	28	6
29623	3	3931616	3931631	4	4	16	ATGA	20	3
29635	3	3960893	3960908	2	8	16	AC	21	5
29636	3	3963578	3963593	4	4	16	AAAT	28	4
29644	3	3974623	3974636	2	7	14	AC	27	5
29647	3	3980208	3980219	2	6	12	GA	15	6
29649	3	3984385	3984405	3	7	21	TTC	15	5
29658	3	3991634	3991655	2	11	22	CT	15	3
29662	3	3994830	3994851	2	11	22	TG	21	5
29665	3	3997779	3997804	2	13	26	GT	15	16
29666	3	3998158	3998183	2	13	26	GT	11	6
29667	3	3998787	3998816	3	10	30	GCT	32	12
29668	3	3999425	3999440	2	8	16	AT	18	8
29675	3	4006182	4006201	2	10	20	TG	10	6
29680	3	4008144	4008157	2	7	14	GT	16	11
29681	3	4008159	4008176	2	9	18	TG	14	10
29688	3	4011293	4011316	4	6	24	TAAA	19	6
29690	3	4022458	4022497	2	20	40	TG	12	9
29694	3	4042427	4042440	2	7	14	TA	15	12
29698	3	4051433	4051447	3	5	15	ATG	22	9

29699	3	4061199	4061214	2	8	16	AC	17	9
29702	3	4070195	4070215	3	7	21	AAC	26	4
29705	3	4077383	4077403	3	7	21	CTT	27	5
29709	3	4082161	4082180	4	5	20	CATT	20	3
29711	3	4090768	4090783	2	8	16	GT	19	3
29712	3	4090938	4090951	2	7	14	CA	17	8
29718	3	4095884	4095901	3	6	18	CAT	25	3
29729	3	4119404	4119423	4	5	20	TTGA	29	3
29730	3	4119987	4120018	2	16	32	CA	19	10
29733	3	4130322	4130345	4	6	24	GTCA	30	4
29738	3	4145096	4145115	2	10	20	AC	20	11
29739	3	4147125	4147138	2	7	14	TA	15	4
29744	3	4164206	4164231	2	13	26	CA	7	6
29752	3	4189179	4189190	2	6	12	TG	22	3
29778	3	4249689	4249716	2	14	28	AC	6	7
29783	3	4260117	4260132	4	4	16	TTTG	14	3
29823	3	4357564	4357578	3	5	15	GCT	24	4
29833	3	4381277	4381298	2	11	22	AT	9	10
29848	3	4426979	4426998	2	10	20	AC	16	8
29849	3	4427146	4427173	2	14	28	GT	11	7
29852	3	4428580	4428601	2	11	22	CA	23	4
29857	3	4432307	4432332	2	13	26	TG	15	6
29861	3	4457170	4457211	2	21	42	TG	7	8
29862	3	4457706	4457721	2	8	16	CT	13	7
29864	3	4457823	4457842	2	10	20	GA	14	4
29874	3	4473854	4473873	2	10	20	AC	12	4
29885	3	4500591	4500605	3	5	15	TAT	16	4
29888	3	4502253	4502268	2	8	16	AC	24	4
29890	3	4503754	4503769	2	8	16	TG	22	3
29892	3	4510539	4510552	2	7	14	TG	14	11
29901	3	4531446	4531461	2	8	16	CT	27	3
29902	3	4531646	4531665	4	5	20	CAGG	29	3
29903	3	4531772	4531789	2	9	18	GA	24	4
29904	3	4535318	4535345	2	14	28	CA	8	8
29909	3	4545217	4545234	2	9	18	TG	20	5
29910	3	4545735	4545746	2	6	12	AC	20	5
29916	3	4551821	4551836	4	4	16	GACA	17	3
29917	3	4552871	4552888	2	9	18	GT	20	8
29926	3	4573486	4573527	2	21	42	GT	17	20
29932	3	4595650	4595665	4	4	16	TTAG	31	3
29933	3	4596897	4596908	2	6	12	TG	31	3
29935	3	4599293	4599304	2	6	12	TC	30	6
29944	3	4638597	4638622	2	13	26	CA	13	6
29946	3	4642295	4642330	2	18	36	CA	8	9
29949	3	4648389	4648400	2	6	12	TA	15	5
29963	3	4689623	4689668	2	23	46	AC	7	9
29982	3	4722486	4722513	2	14	28	GT	11	15
29985	3	4729098	4729125	2	14	28	GT	15	8
29992	3	4747087	4747106	4	5	20	CTGT	16	3
29993	3	4749550	4749561	2	6	12	GA	33	4
29997	3	4776000	4776017	2	9	18	AT	10	3

29999	3	4783683	4783720	2	19	38	AC	8	8
30016	3	4839493	4839504	2	6	12	AC	26	3
30028	3	4867820	4867833	2	7	14	GT	29	5
30030	3	4880718	4880747	2	15	30	CA	7	6
30040	3	4890655	4890682	2	14	28	TC	17	11
30042	3	4890710	4890727	2	9	18	GT	15	4
30047	3	4904032	4904045	2	7	14	AG	18	4
30056	3	4926309	4926320	2	6	12	CA	17	4
30057	3	4926538	4926559	2	11	22	CA	18	7
30064	3	4945626	4945645	2	10	20	TG	6	5
30070	3	4962517	4962530	2	7	14	CA	28	3
30075	3	4978494	4978508	3	5	15	GAG	18	3
30076	3	4980753	4980764	2	6	12	CA	33	3
30077	3	4981647	4981682	2	18	36	TG	9	8
30082	3	4987335	4987366	2	16	32	TG	20	11
30095	3	5033956	5033971	2	8	16	TG	20	10
30102	3	5047395	5047426	2	16	32	GT	11	9
30112	3	5069031	5069052	2	11	22	AC	16	10
30117	3	5069984	5070007	3	8	24	CCT	13	5
30121	3	5095357	5095376	4	5	20	TTAA	20	5
30124	3	5102695	5102708	2	7	14	AT	8	7
30133	3	5116098	5116142	3	15	45	TAC	11	7
30137	3	5129483	5129502	2	10	20	GT	17	8
30141	3	5155540	5155569	2	15	30	TG	7	6
30144	3	5161196	5161239	2	22	44	TG	6	8
30153	3	5187335	5187348	2	7	14	CA	14	3
30159	3	5200415	5200434	2	10	20	TG	16	8
30163	3	5206790	5206825	2	18	36	CT	12	7
30164	3	5208194	5208227	2	17	34	GT	10	9
30167	3	5208833	5208846	2	7	14	AC	21	3
30185	3	5279799	5279814	2	8	16	AC	30	5
30188	3	5283831	5283884	3	18	54	CAG	23	12
30191	3	5287151	5287184	2	17	34	AC	11	12
30197	3	5314935	5314952	3	6	18	GCT	27	4
30201	3	5317158	5317181	2	12	24	TG	22	6
30213	3	5339164	5339207	2	22	44	AC	8	7
30220	3	5364135	5364180	2	23	46	TG	8	8
30231	3	5385559	5385579	3	7	21	ATA	15	4
30233	3	5389694	5389711	2	9	18	GA	18	3
30241	3	5416783	5416808	2	13	26	GT	7	5
30243	3	5417161	5417186	2	13	26	AC	15	4
30245	3	5425095	5425140	2	23	46	TG	6	6
30252	3	5438581	5438610	2	15	30	AC	16	6
30258	3	5446435	5446482	3	16	48	CTG	26	7
30264	3	5458324	5458361	2	19	38	TG	12	8
30266	3	5458662	5458693	2	16	32	TG	7	8
30274	3	5473083	5473102	4	5	20	GTGC	23	3
30289	3	5509895	5509910	2	8	16	GT	16	6
30290	3	5509912	5509923	2	6	12	TG	9	4
30293	3	5518893	5518907	3	5	15	GCA	17	3
30300	3	5539379	5539404	2	13	26	TG	18	5

30302	3	5541005	5541036	2	16	32	AC	10	6
30303	3	5541066	5541083	2	9	18	AG	22	3
30309	3	5577912	5577926	3	5	15	CAG	24	4
30322	3	5609088	5609101	2	7	14	GT	29	3
30325	3	5615787	5615810	4	6	24	CTGT	16	3
30334	3	5635056	5635070	3	5	15	CTG	21	3
30336	3	5639527	5639544	2	9	18	TG	19	6
30344	3	5662193	5662208	2	8	16	GT	16	5
30384	3	5754553	5754572	2	10	20	CA	10	6
30432	3	5905372	5905391	2	10	20	CA	9	5
30433	3	5907476	5907497	2	11	22	GT	17	5
30446	3	5943026	5943051	2	13	26	TG	19	3
30449	3	5943733	5943762	2	15	30	TG	6	3
30454	3	5964764	5964777	2	7	14	CA	32	3
30455	3	5965814	5965841	2	14	28	GT	16	6
30466	3	5990293	5990316	4	6	24	TAGA	14	4
30469	3	6005078	6005107	2	15	30	AC	9	4
30486	3	6063976	6063993	2	9	18	CA	24	5
30489	3	6068649	6068676	4	7	28	AAAG	27	6
30523	3	6145028	6145043	2	8	16	CA	23	3
30528	3	6150380	6150401	2	11	22	CA	25	3
30531	3	6160219	6160236	2	9	18	TG	13	3
30542	3	6210269	6210280	2	6	12	CT	25	3
30553	3	6271873	6271900	2	14	28	CA	16	5
30559	3	6274480	6274527	2	24	48	TG	6	8
30560	3	6275768	6275782	3	5	15	CTG	26	4
30564	3	6295219	6295236	2	9	18	TG	20	5
30588	3	6362960	6362975	4	4	16	ATTT	9	3
30631	3	6510331	6510352	2	11	22	AC	17	7
30634	3	6528760	6528777	2	9	18	AC	16	3
30635	3	6530591	6530605	3	5	15	AGC	21	3
30642	3	6538846	6538861	4	4	16	TAGC	18	5
30647	3	6558262	6558291	2	15	30	CA	13	8
30651	3	6561449	6561464	4	4	16	TTTA	21	4
30653	3	6563187	6563220	2	17	34	TG	10	11
30664	3	6571531	6571544	2	7	14	CA	27	3
30665	3	6573905	6573928	2	12	24	AC	12	9
30666	3	6574010	6574021	2	6	12	CT	17	6
30667	3	6574023	6574038	2	8	16	TC	16	7
30669	3	6575773	6575804	2	16	32	GT	6	7
30676	3	6582537	6582550	2	7	14	CA	23	6
30677	3	6586943	6586954	2	6	12	CA	17	4
30678	3	6586958	6586973	2	8	16	AC	18	7
30686	3	6598614	6598643	2	15	30	TG	13	11
30687	3	6598908	6598925	3	6	18	GGA	24	5
30688	3	6599432	6599447	2	8	16	CA	16	5
30691	3	6601298	6601317	4	5	20	AAAC	18	3
30692	3	6603020	6603033	2	7	14	TG	19	5
30694	3	6603228	6603261	2	17	34	TG	7	8
30699	3	6606234	6606261	2	14	28	AC	10	9
30701	3	6609566	6609581	2	8	16	AC	20	11

30702	3	6609688	6609705	2	9	18	AC	22	6
30706	3	6623303	6623330	4	7	28	GTGC	25	10
30717	3	6642179	6642210	2	16	32	GT	10	10
30726	3	6650285	6650300	2	8	16	AC	15	8
30728	3	6656215	6656226	2	6	12	AC	27	3
30729	3	6657814	6657843	2	15	30	TG	20	12
30733	3	6665691	6665722	2	16	32	TG	12	8
30734	3	6668085	6668120	2	18	36	TG	7	5
30735	3	6668244	6668273	2	15	30	AC	14	15
30736	3	6668382	6668393	2	6	12	CA	31	4
30738	3	6672543	6672562	2	10	20	CA	12	7
30741	3	6673067	6673078	2	6	12	AG	20	3
30743	3	6677258	6677281	2	12	24	CA	8	7
30744	3	6679060	6679075	4	4	16	AAAC	22	3
30746	3	6682135	6682160	2	13	26	CA	16	9
30747	3	6682268	6682289	2	11	22	TG	21	8
30748	3	6685651	6685662	2	6	12	CA	30	4
30755	3	6692511	6692528	2	9	18	AC	16	7
30759	3	6703276	6703293	2	9	18	CA	15	6
30761	3	6705077	6705094	2	9	18	GT	17	11
30763	3	6714091	6714104	2	7	14	AC	28	4
30767	3	6727484	6727495	2	6	12	CA	34	4
30770	3	6731046	6731087	2	21	42	AC	18	13
30774	3	6736067	6736094	2	14	28	AC	13	9
30780	3	6738989	6739003	3	5	15	CCT	23	4
30785	3	6742412	6742423	2	6	12	GA	24	3
30787	3	6742538	6742559	2	11	22	GT	8	8
30789	3	6745407	6745432	2	13	26	AC	8	10
30793	3	6762456	6762473	2	9	18	CA	14	5
30800	3	6779886	6779900	3	5	15	CAG	33	3
30806	3	6795072	6795089	3	6	18	GCA	30	4
30808	3	6798880	6798899	2	10	20	CA	16	8
30810	3	6802654	6802689	4	9	36	AGAA	12	12
30819	3	6828126	6828141	4	4	16	AACG	29	6
30820	3	6828879	6828894	4	4	16	AAAC	30	3
30825	3	6843633	6843660	2	14	28	CT	26	5
30828	3	6849668	6849679	2	6	12	AG	18	4
30832	3	6857770	6857795	2	13	26	TG	16	8
30836	3	6863345	6863356	2	6	12	CA	26	3
30837	3	6863556	6863579	2	12	24	AC	16	6
30839	3	6864037	6864057	3	7	21	GCT	20	7
30841	3	6864478	6864497	2	10	20	AC	15	7
30842	3	6864749	6864766	2	9	18	CA	11	3
30843	3	6864795	6864810	2	8	16	CA	24	3
30847	3	6874046	6874071	2	13	26	AG	21	6
30848	3	6877571	6877598	2	14	28	AC	16	5
30849	3	6877723	6877742	2	10	20	CA	14	12
30851	3	6879762	6879777	2	8	16	CA	8	3
30852	3	6881860	6881881	2	11	22	CA	25	9
30855	3	6882186	6882221	2	18	36	GT	9	13
30857	3	6882571	6882584	2	7	14	CA	17	5

30858	3	6882792	6882813	2	11	22	AC	20	17
30863	3	6899921	6899952	2	16	32	TC	10	10
30868	3	6907405	6907430	2	13	26	GT	7	9
30870	3	6910088	6910099	2	6	12	GT	34	4
30871	3	6913720	6913735	2	8	16	CA	21	7
30872	3	6920531	6920544	2	7	14	GT	17	3
30873	3	6920677	6920722	2	23	46	TG	6	8
30874	3	6920752	6920775	2	12	24	GA	7	7
30877	3	6929891	6929908	2	9	18	AC	29	8
30879	3	6931019	6931052	2	17	34	CA	12	10
30880	3	6932428	6932447	2	10	20	GT	18	12
30881	3	6932601	6932644	2	22	44	TG	7	7
30882	3	6938436	6938449	2	7	14	AC	30	3
30884	3	6944791	6944820	2	15	30	TG	13	11
30887	3	6945299	6945314	2	8	16	AC	16	4
30888	3	6945467	6945480	2	7	14	GT	19	4
30892	3	6953343	6953396	2	27	54	TG	10	13
30897	3	6957229	6957244	2	8	16	GT	26	7
30902	3	6961744	6961755	2	6	12	TG	25	5
30903	3	6962058	6962083	2	13	26	GT	13	11
30906	3	6968853	6968880	2	14	28	GT	15	10
30907	3	6969522	6969535	2	7	14	TG	13	6
30908	3	6972144	6972155	2	6	12	AC	22	9
30909	3	6972497	6972516	2	10	20	CT	20	5
30910	3	6972656	6972687	2	16	32	GT	16	10
30912	3	6975367	6975394	2	14	28	TG	29	12
30918	3	6980426	6980445	2	10	20	GT	16	5
30921	3	6983370	6983387	2	9	18	AC	12	8
30932	3	6997491	6997512	2	11	22	TG	19	9
30933	3	7000446	7000459	2	7	14	TG	19	5
30945	3	7040408	7040423	2	8	16	AG	24	6
30946	3	7041328	7041345	3	6	18	CTC	22	3
30949	3	7044616	7044633	2	9	18	AC	25	3
30951	3	7047479	7047520	2	21	42	TG	10	11
30955	3	7058095	7058121	3	9	27	AGG	28	4
30959	3	7073670	7073691	2	11	22	AC	6	8
30965	3	7085490	7085507	2	9	18	CA	24	7
30967	3	7096170	7096187	2	9	18	TG	16	5
30968	3	7099469	7099483	3	5	15	TTC	23	3
30969	3	7099690	7099717	2	14	28	GT	14	5
30970	3	7100311	7100328	2	9	18	GT	25	10
30971	3	7100381	7100402	2	11	22	GT	19	7
30972	3	7104934	7104947	2	7	14	GT	15	9
30973	3	7104950	7104961	2	6	12	GT	12	9
30974	3	7105060	7105079	2	10	20	TG	19	4
30979	3	7134700	7134713	2	7	14	CA	22	9
30981	3	7158124	7158157	2	17	34	TG	18	4
30983	3	7167857	7167876	4	5	20	TTTG	22	8
30984	3	7173355	7173366	2	6	12	CA	31	4
30996	3	7195618	7195629	2	6	12	AC	24	3
31000	3	7205703	7205738	2	18	36	CA	14	7

31001	3	7211844	7211857	2	7	14	AC	7	4
31002	3	7211860	7211873	2	7	14	AC	17	11
31003	3	7212065	7212104	2	20	40	TG	8	9
31004	3	7213736	7213771	2	18	36	AG	11	12
31006	3	7214212	7214249	2	19	38	AG	8	11
31007	3	7217323	7217338	2	8	16	TG	21	4
31009	3	7220705	7220738	2	17	34	CA	6	7
31012	3	7222820	7222840	3	7	21	TGG	22	4
31013	3	7223614	7223625	2	6	12	TG	28	3
31016	3	7228015	7228044	2	15	30	TG	6	6
31019	3	7229873	7229886	2	7	14	TG	18	10
31021	3	7233923	7233934	2	6	12	GT	25	10
31022	3	7235719	7235754	2	18	36	CA	8	9
31023	3	7238259	7238294	2	18	36	AC	14	9
31024	3	7238854	7238875	2	11	22	CA	21	6
31031	3	7251334	7251347	2	7	14	AC	29	3
31032	3	7255727	7255766	2	20	40	CT	8	6
31033	3	7255957	7255976	2	10	20	TG	15	7
31034	3	7257330	7257363	2	17	34	GT	11	7
31040	3	7265164	7265183	4	5	20	TGTA	8	8
31042	3	7266319	7266352	2	17	34	TG	11	10
31043	3	7266996	7267019	2	12	24	TG	16	8
31047	3	7269174	7269189	2	8	16	AC	19	9
31048	3	7270208	7270235	2	14	28	CA	8	4
31049	3	7270464	7270489	2	13	26	AC	10	5
31050	3	7270676	7270687	2	6	12	GA	30	5
31052	3	7279394	7279419	2	13	26	GT	21	9
31054	3	7281648	7281671	2	12	24	GT	18	12
31055	3	7283332	7283355	2	12	24	TG	19	10
31056	3	7285101	7285112	2	6	12	AC	20	3
31062	3	7303560	7303585	2	13	26	GT	11	7
31066	3	7308079	7308090	2	6	12	CA	27	8
31071	3	7317664	7317685	2	11	22	TG	14	5
31074	3	7325068	7325093	2	13	26	TG	19	6
31077	3	7325319	7325350	4	8	32	CACG	8	7
31079	3	7326077	7326106	2	15	30	TG	17	6
31081	3	7334811	7334842	2	16	32	TG	15	8
31089	3	7339941	7339958	2	9	18	TA	16	4
31091	3	7341078	7341107	2	15	30	GT	21	9
31092	3	7341373	7341390	2	9	18	GT	17	9
31093	3	7341489	7341522	2	17	34	TG	6	5
31101	3	7350373	7350396	2	12	24	TG	19	7
31105	3	7357924	7357950	3	9	27	ATG	15	6
31113	3	7360026	7360037	2	6	12	TG	29	3
31116	3	7370586	7370603	3	6	18	TGC	6	3
31118	3	7370805	7370819	3	5	15	GCT	31	4
31121	3	7375805	7375844	2	20	40	CA	12	7
31125	3	7379024	7379053	2	15	30	CA	15	5
31127	3	7388636	7388667	2	16	32	TG	10	9
31128	3	7394351	7394362	2	6	12	CA	32	3
31129	3	7399550	7399564	3	5	15	TAC	27	4

31134	3	7418191	7418211	3	7	21	GGA	23	4
31142	3	7436674	7436687	2	7	14	AC	22	4
31144	3	7437654	7437671	3	6	18	ATC	24	6
31147	3	7448140	7448163	2	12	24	TG	18	8
31149	3	7455477	7455514	2	19	38	CA	17	9
31151	3	7463190	7463215	2	13	26	GA	19	11
31152	3	7463607	7463634	2	14	28	GT	15	6
31156	3	7491040	7491055	2	8	16	TG	26	4
31159	3	7492050	7492069	2	10	20	GT	20	7
31160	3	7500968	7501003	2	18	36	AC	12	11
31166	3	7521103	7521134	2	16	32	AC	6	6
31167	3	7522339	7522356	2	9	18	CA	26	8
31168	3	7522789	7522820	4	8	32	ATGG	10	3
31170	3	7528572	7528589	3	6	18	TAT	7	4
31172	3	7530032	7530063	2	16	32	GA	10	10
31175	3	7533749	7533772	3	8	24	TCT	27	5
31176	3	7534559	7534572	2	7	14	CA	22	5
31179	3	7536529	7536552	2	12	24	TC	15	7
31182	3	7538651	7538666	2	8	16	CA	24	8
31183	3	7540235	7540256	2	11	22	TC	20	11
31186	3	7544858	7544875	3	6	18	CTG	20	3
31187	3	7546183	7546203	3	7	21	GCA	20	4
31194	3	7550605	7550646	2	21	42	TG	11	6
31195	3	7551979	7552006	4	7	28	CAGG	15	7
31196	3	7552157	7552172	4	4	16	ATTT	15	6
31198	3	7553400	7553413	2	7	14	CA	11	6
31200	3	7556042	7556079	2	19	38	AC	10	11
31203	3	7558613	7558632	2	10	20	AC	13	10
31204	3	7562577	7562591	3	5	15	CAT	31	4
31205	3	7562760	7562777	2	9	18	GT	22	10
31206	3	7563039	7563052	2	7	14	AC	24	11
31207	3	7563103	7563114	2	6	12	TG	22	3
31208	3	7564945	7564974	3	10	30	ATG	27	4
31209	3	7568155	7568176	2	11	22	CT	16	10
31215	3	7583437	7583448	2	6	12	CA	14	4
31217	3	7584785	7584808	2	12	24	CA	17	10
31218	3	7586158	7586195	2	19	38	CA	19	11
31221	3	7593344	7593369	2	13	26	CA	15	7
31227	3	7611689	7611700	2	6	12	GT	28	3
31228	3	7611744	7611759	2	8	16	AG	25	3
31229	3	7612106	7612117	2	6	12	AC	27	4
31238	3	7639352	7639366	3	5	15	ATT	14	4
31241	3	7642814	7642835	2	11	22	AC	18	13
31247	3	7659501	7659516	4	4	16	AAAC	22	3
31254	3	7673591	7673606	2	8	16	CA	11	3
31256	3	7675601	7675621	3	7	21	GCA	21	3
31261	3	7677416	7677427	2	6	12	TG	11	3
31265	3	7689096	7689123	4	7	28	TGAA	25	5
31269	3	7695931	7695944	2	7	14	TG	22	3
31274	3	7701863	7701878	2	8	16	TG	25	5
31275	3	7702794	7702815	2	11	22	GT	13	11

31279	3	7717560	7717583	2	12	24	CA	9	5
31280	3	7717792	7717805	2	7	14	AC	26	8
31286	3	7738112	7738132	3	7	21	GCA	23	9
31291	3	7747958	7747969	2	6	12	TG	20	3
31296	3	7758043	7758062	2	10	20	TG	20	11
31297	3	7760202	7760213	2	6	12	AG	30	5
31298	3	7760515	7760530	2	8	16	AG	11	10
31302	3	7777903	7777948	2	23	46	TG	8	10
31303	3	7778327	7778342	4	4	16	TCCA	28	3
31313	3	7808818	7808835	2	9	18	CA	21	12
31320	3	7832439	7832452	2	7	14	CT	25	4
31321	3	7835331	7835348	2	9	18	AC	23	11
31324	3	7838798	7838821	2	12	24	AG	12	12
31325	3	7838946	7838971	2	13	26	TG	14	14
31328	3	7844865	7844876	2	6	12	AT	29	5
31331	3	7850774	7850809	3	12	36	GCA	19	13
31333	3	7854400	7854411	2	6	12	CA	20	4
31335	3	7858256	7858269	2	7	14	AC	25	5
31340	3	7883764	7883783	2	10	20	CA	10	8
31342	3	7894658	7894672	3	5	15	TCA	26	6
31344	3	7907558	7907572	3	5	15	CAG	22	7
31348	3	7914093	7914116	2	12	24	AG	17	11
31349	3	7914219	7914258	2	20	40	TG	6	10
31350	3	7919838	7919861	4	6	24	AAAC	21	4
31352	3	7923330	7923377	2	24	48	AG	14	11
31354	3	7923822	7923839	2	9	18	CA	25	9
31355	3	7926909	7926924	2	8	16	AC	30	4
31363	3	7945597	7945616	2	10	20	CA	8	5
31366	3	7950598	7950631	2	17	34	GT	10	11
31373	3	7965389	7965428	4	10	40	CTTT	19	12
31384	3	7994865	7994896	2	16	32	GT	21	7
31387	3	8011732	8011769	2	19	38	AC	14	14
31401	3	8042959	8042991	3	11	33	AAC	17	10
31412	3	8063780	8063797	2	9	18	TG	24	9
31417	3	8071615	8071628	2	7	14	CA	31	7
31425	3	8108793	8108804	2	6	12	TC	31	4
31430	3	8122617	8122637	3	7	21	TTA	21	5
31431	3	8123492	8123506	3	5	15	AGC	27	4
31446	3	8159342	8159355	2	7	14	GT	25	4
31448	3	8164883	8164912	2	15	30	CA	8	9
31449	3	8167340	8167353	2	7	14	GA	28	3
31460	3	8181960	8181979	2	10	20	AT	12	3
31474	3	8206342	8206353	2	6	12	TA	21	7
31477	3	8208249	8208263	3	5	15	TTA	27	3
31490	3	8244545	8244558	2	7	14	AT	7	9
31493	3	8250761	8250774	2	7	14	TG	29	4
31494	3	8250816	8250845	2	15	30	GT	17	11
31497	3	8257231	8257250	2	10	20	CA	21	22
31498	3	8262341	8262358	2	9	18	GT	21	5
31504	3	8274839	8274856	2	9	18	AC	20	9
31505	3	8275787	8275798	2	6	12	AC	15	6

31510	3	8280350	8280363	2	7	14	CA	30	3
31513	3	8283536	8283547	2	6	12	AC	20	3
31515	3	8284999	8285020	2	11	22	AC	17	13
31517	3	8292817	8292836	2	10	20	CA	18	7
31530	3	8304035	8304052	2	9	18	TG	12	4
31533	3	8304224	8304237	2	7	14	CA	19	5
31535	3	8304423	8304446	2	12	24	CT	12	7
31536	3	8304508	8304522	3	5	15	GGA	21	5
31537	3	8305889	8305908	2	10	20	AC	17	6
31540	3	8308805	8308816	2	6	12	AC	19	5
31541	3	8308922	8308957	2	18	36	AC	15	10
31542	3	8309125	8309160	2	18	36	TG	10	7
31548	3	8314208	8314241	2	17	34	CA	10	7
31550	3	8314716	8314735	2	10	20	TG	22	5
31553	3	8316443	8316454	2	6	12	CT	22	3
31554	3	8316552	8316569	2	9	18	GT	23	4
31556	3	8317395	8317422	2	14	28	AC	6	6
31557	3	8317964	8317991	2	14	28	AC	14	11
31558	3	8319170	8319195	2	13	26	CA	12	8
31571	3	8348296	8348311	2	8	16	CA	26	10
31573	3	8350708	8350731	3	8	24	TGA	32	5
31574	3	8353605	8353618	2	7	14	CT	28	4
31576	3	8355594	8355605	2	6	12	TG	13	5
31584	3	8365852	8365867	2	8	16	GA	27	3
31590	3	8398021	8398048	2	14	28	TC	8	11
31610	3	8457676	8457691	2	8	16	AG	11	4
31611	3	8457694	8457705	2	6	12	AG	16	4
31617	3	8461582	8461605	2	12	24	AC	22	5
31622	3	8468124	8468143	2	10	20	CA	18	6
31626	3	8469927	8469946	2	10	20	TG	19	9
31627	3	8470100	8470133	2	17	34	CT	8	5
31628	3	8472534	8472547	2	7	14	GT	30	3
31629	3	8474655	8474666	2	6	12	GT	27	3
31637	3	8482225	8482238	2	7	14	TC	13	4
31641	3	8488099	8488120	2	11	22	GT	19	4
31644	3	8491753	8491780	2	14	28	TG	15	8
31645	3	8491935	8491962	2	14	28	AC	23	11
31649	3	8495709	8495728	2	10	20	AC	16	7
31657	3	8501627	8501648	2	11	22	GA	17	9
31661	3	8515720	8515733	2	7	14	AG	19	3
31665	3	8521330	8521349	2	10	20	AC	20	4
31666	3	8521497	8521518	2	11	22	AC	22	7
31670	3	8529807	8529830	2	12	24	AG	28	10
31678	3	8539553	8539568	2	8	16	CA	26	3
31680	3	8541500	8541511	2	6	12	GA	7	3
31683	3	8544112	8544137	2	13	26	TG	7	5
31690	3	8555518	8555531	2	7	14	GT	22	3
31691	3	8557825	8557848	3	8	24	AAG	19	5
31695	3	8561335	8561352	2	9	18	AC	28	5
31696	3	8561959	8561974	2	8	16	GT	24	9
31697	3	8562606	8562623	2	9	18	CA	27	9

31698	3	8564007	8564028	2	11	22	AC	24	11
31699	3	8565118	8565141	2	12	24	TG	21	8
31700	3	8565940	8565955	2	8	16	AC	17	4
31702	3	8568034	8568051	2	9	18	CA	18	7
31704	3	8571661	8571690	2	15	30	TG	21	9
31706	3	8576745	8576768	4	6	24	AAAC	18	5
31725	3	8603717	8603732	4	4	16	GCTA	32	3
31731	3	8613665	8613692	4	7	28	AAAG	23	7
31732	3	8615110	8615125	2	8	16	CA	19	3
31734	3	8616350	8616363	2	7	14	GT	32	4
31735	3	8618943	8618958	2	8	16	AC	24	5
31738	3	8624203	8624218	4	4	16	CTTC	23	3
31739	3	8628367	8628398	2	16	32	CA	10	6
31742	3	8645724	8645737	2	7	14	TG	26	3
31745	3	8648421	8648448	2	14	28	TG	15	14
31746	3	8648593	8648616	2	12	24	CA	21	11
31747	3	8652690	8652713	4	6	24	ATGG	19	12
31756	3	8667018	8667029	2	6	12	AC	23	6
31768	3	8699123	8699148	2	13	26	CA	17	9
31769	3	8699303	8699316	2	7	14	AG	20	3
31772	3	8708919	8708940	2	11	22	GT	18	9
31776	3	8712217	8712244	2	14	28	AC	12	10
31777	3	8713569	8713588	2	10	20	CA	17	11
31782	3	8722412	8722438	3	9	27	TCC	21	5
31783	3	8729124	8729135	2	6	12	TG	23	6
31784	3	8729136	8729147	2	6	12	TC	25	7
31785	3	8730983	8731012	3	10	30	GAG	8	7
31791	3	8735922	8735937	2	8	16	AC	14	8
31795	3	8739323	8739338	4	4	16	CTGT	19	6
31797	3	8740424	8740445	2	11	22	CA	14	12
31805	3	8749332	8749346	3	5	15	AAT	23	5
31806	3	8750603	8750614	2	6	12	TC	27	3
31808	3	8757210	8757223	2	7	14	TG	22	4
31809	3	8760737	8760772	2	18	36	AC	21	13
31823	3	8794429	8794456	2	14	28	GT	16	9
31826	3	8796500	8796517	2	9	18	AC	25	4
31829	3	8798925	8798946	2	11	22	AC	21	10
31830	3	8800770	8800787	3	6	18	AAG	19	3
31831	3	8801632	8801651	2	10	20	AC	17	6
31832	3	8801818	8801855	2	19	38	CA	8	8
31839	3	8811566	8811577	2	6	12	AC	17	4
31842	3	8818910	8818921	2	6	12	AC	10	6
31846	3	8825540	8825555	2	8	16	TC	31	3
31848	3	8830792	8830815	2	12	24	GT	16	14
31856	3	8864620	8864637	2	9	18	GT	17	3
31863	3	8871768	8871781	2	7	14	GT	15	3
31868	3	8876634	8876649	2	8	16	GT	25	3
31869	3	8876799	8876814	2	8	16	GT	23	5
31871	3	8877778	8877793	2	8	16	GT	19	6
31876	3	8882197	8882220	2	12	24	TG	16	10
31877	3	8883502	8883523	2	11	22	TC	11	4

31879	3	8884577	8884590	2	7	14	CT	21	6
31880	3	8887945	8887968	2	12	24	TG	9	6
31882	3	8890905	8890940	2	18	36	GT	17	8
31883	3	8891178	8891191	2	7	14	TC	23	4
31885	3	8893524	8893535	2	6	12	TG	16	9
31890	3	8897362	8897403	2	21	42	TG	14	11
31891	3	8897610	8897621	2	6	12	TG	27	4
31892	3	8897626	8897637	2	6	12	TG	25	4
31893	3	8898779	8898802	2	12	24	GT	10	9
31894	3	8900460	8900480	3	7	21	CAT	22	3
31896	3	8902123	8902137	3	5	15	TGC	22	3
31897	3	8902207	8902222	2	8	16	CA	17	6
31900	3	8906323	8906352	2	15	30	TG	14	7
31902	3	8912948	8912971	3	8	24	GCT	24	5
31903	3	8914633	8914654	2	11	22	TC	11	7
31911	3	8927541	8927586	2	23	46	CA	8	10
31913	3	8929212	8929229	2	9	18	CT	12	6
31914	3	8929230	8929245	2	8	16	CA	16	7
31917	3	8932542	8932555	2	7	14	GT	18	9
31918	3	8932558	8932575	2	9	18	GT	10	8
31920	3	8936208	8936225	2	9	18	TG	22	9
31921	3	8938291	8938320	2	15	30	CA	12	6
31923	3	8938593	8938618	2	13	26	CA	23	6
31925	3	8942765	8942785	3	7	21	GGA	11	4
31930	3	8947935	8947964	2	15	30	GT	19	9
31932	3	8953370	8953381	2	6	12	AC	27	4
31933	3	8955983	8956012	3	10	30	ATT	14	5
31937	3	8960303	8960334	2	16	32	AC	12	14
31939	3	8961570	8961591	2	11	22	CA	17	22
31946	3	8965927	8965948	2	11	22	GT	15	4
31948	3	8968379	8968399	3	7	21	AGA	23	5
31950	3	8970729	8970743	3	5	15	GCA	30	4
31963	3	8989015	8989036	2	11	22	GT	16	11
31970	3	9004946	9004965	4	5	20	TTTA	17	7
31975	3	9016859	9016870	2	6	12	TG	26	4
31977	3	9028208	9028229	2	11	22	CA	24	5
31979	3	9032078	9032116	3	13	39	TCT	11	9
31981	3	9036445	9036472	2	14	28	CA	14	7
31982	3	9037099	9037110	2	6	12	AC	26	4
31984	3	9041235	9041255	3	7	21	AGA	21	6
31986	3	9044592	9044603	2	6	12	CA	24	3
31991	3	9054160	9054199	4	10	40	ATGG	28	9
31992	3	9055062	9055083	2	11	22	GT	24	6
31996	3	9057213	9057232	2	10	20	TC	9	8
31998	3	9057442	9057463	2	11	22	AC	14	11
32001	3	9070958	9070969	2	6	12	AC	21	3
32003	3	9071339	9071352	2	7	14	TG	25	6
32005	3	9075239	9075258	4	5	20	TGTT	24	3
32006	3	9080341	9080374	2	17	34	TG	20	10
32012	3	9110657	9110672	4	4	16	AAAT	26	12
32019	3	9146197	9146228	2	16	32	AC	10	9

32027	3	9166638	9166695	2	29	58	AG	9	6
32029	3	9168902	9168925	2	12	24	CA	13	7
32033	3	9175625	9175638	2	7	14	AC	13	5
32035	3	9179471	9179484	2	7	14	CA	24	8
32036	3	9186499	9186516	2	9	18	TG	16	6
32039	3	9188618	9188631	2	7	14	TG	31	3
32054	3	9234327	9234348	2	11	22	TG	19	17
32056	3	9244328	9244343	2	8	16	TG	20	8
32057	3	9244576	9244601	2	13	26	GT	11	10
32060	3	9246809	9246830	2	11	22	AC	12	5
32061	3	9246855	9246866	2	6	12	AC	20	7
32062	3	9249278	9249305	2	14	28	AC	9	11
32066	3	9251434	9251449	2	8	16	CT	18	6
32067	3	9251617	9251650	2	17	34	TG	10	6
32069	3	9251679	9251692	2	7	14	TG	14	3
32071	3	9252107	9252122	2	8	16	CA	19	6
32075	3	9254659	9254682	2	12	24	TG	14	9
32076	3	9255709	9255726	3	6	18	ATC	21	6
32080	3	9256329	9256346	3	6	18	ATG	24	7
32082	3	9257315	9257348	2	17	34	AC	10	7
32087	3	9261377	9261400	2	12	24	TG	23	10
32094	3	9266939	9266950	2	6	12	GT	13	5
32095	3	9266953	9266968	2	8	16	GT	11	3
32096	3	9269097	9269146	2	25	50	TG	9	10
32098	3	9269781	9269830	2	25	50	AC	9	11
32107	3	9277367	9277382	2	8	16	GA	20	3
32108	3	9277594	9277608	3	5	15	TTG	9	3
32119	3	9282906	9282932	3	9	27	TCC	9	10
32122	3	9284343	9284366	3	8	24	AAC	15	5
32123	3	9284398	9284429	2	16	32	AG	11	10
32127	3	9285196	9285219	3	8	24	TGA	17	6
32130	3	9285903	9285920	2	9	18	CT	7	5
32131	3	9286113	9286142	2	15	30	CA	13	9
32134	3	9291289	9291300	2	6	12	GA	18	3
32137	3	9292058	9292077	4	5	20	GGAG	9	3
32138	3	9292419	9292432	2	7	14	GT	24	5
32139	3	9292552	9292565	2	7	14	AC	27	7
32146	3	9302091	9302104	2	7	14	GT	11	7
32152	3	9305501	9305516	2	8	16	CA	28	4
32155	3	9309747	9309782	2	18	36	TG	12	8
32158	3	9313050	9313069	2	10	20	AT	7	5
32161	3	9314702	9314723	2	11	22	GA	11	3
32164	3	9321723	9321750	2	14	28	GT	11	11
32173	3	9345185	9345214	2	15	30	TG	16	8
32178	3	9355537	9355560	3	8	24	ATT	11	5
32179	3	9356886	9356900	3	5	15	AGC	29	3
32199	3	9387085	9387102	2	9	18	AC	21	5
32200	3	9387246	9387265	2	10	20	AC	28	4
32205	3	9392945	9392956	2	6	12	AC	14	3
32206	3	9392996	9393029	2	17	34	CA	10	6
32211	3	9394746	9394765	2	10	20	TG	11	7

32213	3	9400116	9400136	3	7	21	AGC	21	7
32214	3	9400187	9400216	2	15	30	GT	13	11
32218	3	9404039	9404062	3	8	24	GGA	27	4
32219	3	9404364	9404379	2	8	16	AT	13	6
32222	3	9406326	9406337	2	6	12	TA	21	7
32225	3	9410437	9410456	2	10	20	GT	16	8
32226	3	9410458	9410471	2	7	14	TG	18	12
32229	3	9413719	9413738	2	10	20	CT	13	8
32232	3	9418864	9418877	2	7	14	CA	26	3
32235	3	9421141	9421152	2	6	12	AC	27	4
32238	3	9425433	9425448	2	8	16	CA	25	7
32241	3	9427898	9427917	2	10	20	AC	30	9
32244	3	9431964	9431975	2	6	12	GT	34	3
32247	3	9435765	9435788	2	12	24	CA	19	6
32255	3	9441750	9441765	2	8	16	AC	19	4
32260	3	9442891	9442914	2	12	24	TG	18	14
32262	3	9444220	9444237	2	9	18	CA	24	4
32264	3	9449494	9449527	2	17	34	AC	10	7
32268	3	9461971	9461988	2	9	18	CT	14	7
32272	3	9471878	9471895	2	9	18	AC	19	10
32273	3	9473360	9473385	2	13	26	CA	20	12
32280	3	9483496	9483519	2	12	24	TG	25	9
32283	3	9487459	9487472	2	7	14	AG	31	3
32289	3	9505547	9505560	2	7	14	GT	19	7
32291	3	9507561	9507578	2	9	18	CT	8	3
32299	3	9510627	9510650	2	12	24	GT	16	8
32313	3	9514508	9514522	3	5	15	CAC	23	14
32315	3	9515152	9515177	2	13	26	AC	9	10
32320	3	9517261	9517288	2	14	28	GT	15	8
32321	3	9517935	9517946	2	6	12	AC	23	5
32324	3	9519471	9519492	2	11	22	AC	18	9
32327	3	9520077	9520096	2	10	20	AC	19	11
32329	3	9520257	9520274	2	9	18	TG	17	11
32330	3	9520435	9520450	2	8	16	TG	23	5
32331	3	9521049	9521064	2	8	16	GT	17	12
32332	3	9521067	9521082	2	8	16	GT	24	12
32335	3	9522293	9522308	2	8	16	TG	18	5
32339	3	9522794	9522811	2	9	18	AC	12	4
32344	3	9523729	9523740	2	6	12	AC	25	6
32345	3	9524135	9524160	2	13	26	AC	13	9
32351	3	9531913	9531938	2	13	26	CA	17	5
32352	3	9532037	9532054	2	9	18	AC	15	6
32353	3	9533871	9533892	2	11	22	TG	25	8
32355	3	9537027	9537046	2	10	20	CA	23	4
32360	3	9546866	9546889	2	12	24	GT	11	10
32363	3	9548082	9548126	3	15	45	GAG	20	8
32365	3	9550624	9550641	2	9	18	TG	24	8
32366	3	9550854	9550869	2	8	16	GT	15	4
32367	3	9551068	9551089	2	11	22	GT	15	4
32369	3	9551325	9551340	2	8	16	CA	19	10
32370	3	9552546	9552573	4	7	28	CAGA	26	5

32371	3	9552730	9552743	2	7	14	GT	21	6
32372	3	9552792	9552811	2	10	20	TG	24	7
32374	3	9554252	9554275	2	12	24	AC	15	8
32375	3	9554450	9554467	2	9	18	AC	20	7
32376	3	9556057	9556084	2	14	28	AC	14	11
32377	3	9556142	9556159	2	9	18	AC	25	3
32379	3	9557479	9557500	2	11	22	CT	28	7
32381	3	9558115	9558134	2	10	20	CA	17	6
32382	3	9558266	9558287	2	11	22	CA	18	9
32383	3	9559601	9559614	2	7	14	AG	32	3
32384	3	9559761	9559778	2	9	18	AC	15	3
32385	3	9559906	9559937	2	16	32	TG	21	9
32386	3	9560067	9560078	2	6	12	GT	32	6
32387	3	9562013	9562026	2	7	14	AC	15	3
32389	3	9563844	9563855	2	6	12	CA	23	10
32390	3	9564152	9564166	3	5	15	AGA	31	3
32391	3	9565341	9565366	2	13	26	AC	9	7
32392	3	9565672	9565693	2	11	22	CA	18	6
32393	3	9565931	9565958	2	14	28	GT	20	14
32394	3	9566072	9566089	2	9	18	CA	30	4
32395	3	9566288	9566313	2	13	26	AC	16	5
32397	3	9567912	9567939	2	14	28	GT	6	9
32398	3	9569173	9569240	2	34	68	TG	14	11
32399	3	9569310	9569329	2	10	20	CA	22	4
32401	3	9571984	9572007	2	12	24	CA	12	9
32402	3	9572223	9572264	2	21	42	AC	11	10
32406	3	9574372	9574397	2	13	26	TG	16	13
32408	3	9576091	9576110	2	10	20	CA	12	10
32411	3	9577446	9577489	2	22	44	AC	7	8
32413	3	9578758	9578797	2	20	40	CA	17	15
32415	3	9579404	9579423	2	10	20	CA	19	7
32417	3	9581483	9581494	2	6	12	GT	21	3
32418	3	9581686	9581719	2	17	34	TG	11	7
32420	3	9584166	9584185	2	10	20	CA	14	6
32428	3	9590710	9590727	3	6	18	AGG	23	3
32429	3	9590825	9590856	2	16	32	GT	23	9
32430	3	9593899	9593914	2	8	16	TG	21	4
32433	3	9597791	9597802	2	6	12	GT	24	3
32434	3	9600624	9600637	2	7	14	CA	22	9
32435	3	9603284	9603297	2	7	14	GT	25	4
32436	3	9609095	9609110	2	8	16	AC	20	7
32438	3	9618114	9618143	2	15	30	CA	11	7
32439	3	9619086	9619099	2	7	14	GT	27	7
32445	3	9625762	9625779	2	9	18	CA	14	6
32446	3	9627601	9627626	2	13	26	CA	14	13
32447	3	9631331	9631346	4	4	16	AATT	22	9
32448	3	9632121	9632144	2	12	24	GT	25	10
32449	3	9632295	9632308	2	7	14	GT	19	8
32450	3	9632310	9632321	2	6	12	TG	20	6
32451	3	9632696	9632725	2	15	30	TG	18	6
32452	3	9633200	9633213	2	7	14	AC	26	3

32453	3	9633545	9633564	2	10	20	TG	19	4
32461	3	9637211	9637270	3	20	60	TTC	7	8
32467	3	9643266	9643287	2	11	22	AG	22	5
32470	3	9648432	9648445	2	7	14	TG	16	4
32471	3	9648535	9648550	2	8	16	CA	24	5
32474	3	9658688	9658729	2	21	42	CA	7	10
32478	3	9666453	9666464	2	6	12	TG	22	10
32479	3	9667062	9667082	3	7	21	TTG	26	3
32480	3	9667170	9667181	2	6	12	CA	24	3
32482	3	9672793	9672828	3	12	36	CTT	11	9
32483	3	9672936	9672949	2	7	14	TC	18	3
32484	3	9673000	9673025	2	13	26	CA	11	10
32488	3	9676062	9676077	2	8	16	CA	27	3
32491	3	9679980	9679993	2	7	14	CA	22	4
32494	3	9686382	9686419	2	19	38	GT	6	7
32497	3	9690410	9690421	2	6	12	CT	26	3
32498	3	9691700	9691721	2	11	22	GT	14	7
32499	3	9691905	9691926	2	11	22	AC	20	7
32519	3	9714160	9714183	3	8	24	GAG	16	6
32520	3	9716585	9716610	2	13	26	TG	11	6
32521	3	9716734	9716771	2	19	38	AC	9	10
32523	3	9718756	9718791	2	18	36	TG	8	10
32525	3	9722328	9722359	2	16	32	TG	9	10
32526	3	9722666	9722683	2	9	18	TG	24	6
32530	3	9734360	9734371	2	6	12	CT	27	4
32531	3	9735282	9735297	2	8	16	CA	22	7
32532	3	9735480	9735493	2	7	14	AC	22	3
32533	3	9736423	9736434	2	6	12	CA	36	4
32537	3	9746145	9746159	3	5	15	GGA	21	6
32538	3	9746429	9746440	2	6	12	GT	20	14
32544	3	9758234	9758267	2	17	34	AC	9	8
32548	3	9763452	9763469	3	6	18	AAC	16	5
32549	3	9763947	9763962	2	8	16	AC	27	3
32550	3	9766926	9766959	2	17	34	AC	12	9
32551	3	9772912	9772939	2	14	28	AC	10	8
32555	3	9780702	9780719	2	9	18	CA	21	16
32560	3	9782720	9782751	2	16	32	CA	15	9
32561	3	9787187	9787218	2	16	32	TC	7	5
32564	3	9797406	9797427	2	11	22	TG	16	7
32576	3	9824027	9824056	2	15	30	AC	17	8
32582	3	9833166	9833195	2	15	30	CT	10	5
32584	3	9847157	9847172	2	8	16	AG	13	15
32585	3	9847769	9847786	3	6	18	GCT	26	8
32586	3	9849088	9849107	2	10	20	CA	17	6
32590	3	9851017	9851028	2	6	12	CT	24	5
32591	3	9851215	9851244	2	15	30	AC	7	5
32592	3	9851353	9851386	2	17	34	TG	13	8
32594	3	9857778	9857797	2	10	20	GT	18	8
32595	3	9862363	9862404	2	21	42	GT	7	8
32599	3	9865219	9865232	2	7	14	GT	20	6
32606	3	9894487	9894514	2	14	28	CA	19	13

32610	3	9905093	9905118	2	13	26	TG	20	17
32613	3	9930971	9930994	2	12	24	TG	24	8
32617	3	9937901	9937926	2	13	26	AC	13	6
32624	3	9949947	9949958	2	6	12	TG	22	4
32628	3	9950674	9950697	2	12	24	TG	17	7
32637	3	9957322	9957339	2	9	18	CA	15	13
32638	3	9957474	9957507	2	17	34	TG	8	9
32639	3	9959954	9959975	2	11	22	TG	21	7
32640	3	9961166	9961187	2	11	22	TG	19	3
32662	3	9987230	9987269	2	20	40	GA	7	6
32664	3	9988311	9988325	3	5	15	TTC	12	3
32665	3	9988809	9988832	3	8	24	TTA	13	4
32666	3	9988918	9988931	2	7	14	TG	20	6
32668	3	9989697	9989736	2	20	40	TG	6	5
32670	3	9991072	9991085	2	7	14	GT	17	6
32679	3	9992686	9992697	2	6	12	GT	8	10
32682	3	9993093	9993122	2	15	30	AC	8	9
32690	3	9996876	9996917	2	21	42	TG	7	9
32692	3	9999454	9999469	2	8	16	CT	17	3
32693	3	10000400	10000429	2	15	30	TG	16	5
32698	3	10004475	10004502	2	14	28	GT	18	12
32702	3	10025790	10025809	4	5	20	AAAC	10	6
32703	3	10026403	10026422	2	10	20	CA	26	14
32705	3	10027079	10027090	2	6	12	GT	21	7
32706	3	10027094	10027109	2	8	16	TG	14	4
32707	3	10027213	10027224	2	6	12	TG	13	9
32708	3	10027226	10027239	2	7	14	GT	17	14
32711	3	10028884	10028913	2	15	30	CA	13	8
32714	3	10031105	10031138	2	17	34	AC	6	5
32715	3	10033098	10033111	2	7	14	GA	10	4
32716	3	10033491	10033518	2	14	28	TG	13	12
32719	3	10034492	10034511	2	10	20	AC	16	9
32720	3	10035695	10035710	4	4	16	AAAT	19	4
32722	3	10036039	10036050	2	6	12	GT	35	3
32723	3	10036272	10036289	2	9	18	CA	20	5
32726	3	10044082	10044097	2	8	16	AT	18	3
32727	3	10044392	10044409	2	9	18	AC	10	6
32730	3	10046245	10046282	2	19	38	CA	9	9
32733	3	10049253	10049280	4	7	28	TGTC	28	5
32734	3	10049943	10049956	2	7	14	AG	21	6
32735	3	10053785	10053812	2	14	28	AC	8	7
32738	3	10065231	10065252	2	11	22	TG	32	13
32745	3	10081852	10081865	2	7	14	GT	25	8
32748	3	10089986	10090001	2	8	16	GT	15	3
32759	3	10145476	10145499	3	8	24	AAC	26	5
32770	3	10183924	10183953	2	15	30	TG	10	7
32773	3	10186029	10186042	2	7	14	TG	23	3
32774	3	10186692	10186703	2	6	12	AG	26	4
32776	3	10189783	10189796	2	7	14	CA	25	4
32777	3	10190410	10190423	2	7	14	TG	20	5
32781	3	10196083	10196102	2	10	20	GT	11	10

32782	3	10196227	10196252	2	13	26	GT	16	14
32783	3	10196365	10196391	3	9	27	CAT	24	4
32788	3	10213478	10213491	2	7	14	TG	25	3
32791	3	10219434	10219453	2	10	20	GA	18	6
32792	3	10220089	10220100	2	6	12	AC	34	3
32793	3	10220276	10220317	2	21	42	AC	8	9
32798	3	10228772	10228789	2	9	18	CT	20	3
32799	3	10230314	10230331	2	9	18	TG	25	6
32800	3	10230905	10230931	3	9	27	GTA	21	5
32801	3	10234110	10234127	2	9	18	CA	25	5
32802	3	10234291	10234310	2	10	20	GT	10	10
32803	3	10236561	10236584	4	6	24	CAGG	26	7
32805	3	10241741	10241768	2	14	28	AC	13	12
32808	3	10251284	10251305	2	11	22	AC	17	6
32820	3	10278363	10278377	3	5	15	TCC	26	4
32827	3	10288100	10288119	4	5	20	TTTC	20	9
32832	3	10296330	10296345	4	4	16	TTCA	18	3
32838	3	10316706	10316739	2	17	34	AC	12	10
32839	3	10317081	10317098	2	9	18	CA	21	5
32841	3	10322229	10322254	2	13	26	GT	24	7
32842	3	10322320	10322333	2	7	14	GT	16	12
32857	3	10342196	10342209	2	7	14	AC	28	3
32858	3	10346527	10346540	2	7	14	TA	12	7
32863	3	10373099	10373110	2	6	12	AC	27	3
32866	3	10373731	10373750	4	5	20	TATT	12	3
32867	3	10374653	10374710	2	29	58	TG	11	12
32871	3	10387832	10387851	2	10	20	AC	18	12
32874	3	10391917	10391931	3	5	15	GCA	27	3
32880	3	10404104	10404143	2	20	40	GT	14	11
32882	3	10417960	10417986	3	9	27	GAA	24	9
32885	3	10430519	10430545	3	9	27	ATC	15	5
32889	3	10436508	10436521	2	7	14	CA	19	3
32890	3	10445761	10445778	2	9	18	AC	15	3
32897	3	10459751	10459762	2	6	12	TC	13	3
32901	3	10461916	10461947	2	16	32	AC	10	5
32906	3	10478193	10478210	3	6	18	TAT	19	3
32907	3	10480198	10480215	2	9	18	GT	15	8
32908	3	10480217	10480228	2	6	12	TG	16	8
32909	3	10480592	10480627	4	9	36	TGAA	36	9
32917	3	10508301	10508324	2	12	24	CA	15	10
32918	3	10508365	10508382	2	9	18	TG	18	12
32919	3	10508761	10508774	2	7	14	CA	8	5
32920	3	10508904	10508929	2	13	26	TG	18	9
32929	3	10514080	10514106	3	9	27	CTG	23	7
32937	3	10516636	10516649	2	7	14	CA	21	11
32938	3	10516873	10516890	3	6	18	TCT	21	7
32943	3	10517782	10517803	2	11	22	CT	9	6
32945	3	10518203	10518214	2	6	12	GT	17	5
32946	3	10518216	10518227	2	6	12	TG	11	3
32949	3	10518676	10518715	2	20	40	AC	14	13
32951	3	10520454	10520471	3	6	18	CTG	17	3

32953	3	10522273	10522287	3	5	15	AAC	20	7
32954	3	10523705	10523720	4	4	16	CAGG	27	3
32960	3	10546092	10546103	2	6	12	AC	27	6
32961	3	10553426	10553449	3	8	24	TTG	19	4
32964	3	10560729	10560742	2	7	14	GT	27	3
32966	3	10563851	10563910	4	15	60	ATAG	6	5
32967	3	10566668	10566688	3	7	21	CAG	22	6
32969	3	10568310	10568329	2	10	20	TC	13	6
32973	3	10571137	10571152	2	8	16	AC	20	4
32981	3	10584256	10584270	3	5	15	CTG	23	4
32984	3	10589146	10589157	2	6	12	CA	20	10
32986	3	10591284	10591301	3	6	18	CCA	19	3
32991	3	10626216	10626233	2	9	18	TG	23	6
32992	3	10628788	10628799	2	6	12	AT	21	9
32999	3	10668796	10668815	4	5	20	AAAC	10	4
33004	3	10689281	10689312	2	16	32	CA	20	15
33006	3	10696829	10696840	2	6	12	TG	22	3
33013	3	10722112	10722133	2	11	22	AC	19	4
33016	3	10727102	10727113	2	6	12	GT	29	3
33017	3	10732323	10732334	2	6	12	CT	15	7
33018	3	10732336	10732347	2	6	12	TC	12	5
33022	3	10738405	10738431	3	9	27	GAG	19	5
33023	3	10738722	10738747	2	13	26	CA	9	13
33029	3	10751118	10751143	2	13	26	CA	9	9
33030	3	10751788	10751811	4	6	24	GAAG	25	5
33033	3	10759943	10759956	2	7	14	CT	33	3
33039	3	10772586	10772605	2	10	20	GT	14	8
33040	3	10775337	10775354	3	6	18	CCT	26	4
33049	3	10791118	10791133	2	8	16	AC	21	8
33052	3	10794888	10794901	2	7	14	CA	17	9
33053	3	10795089	10795100	2	6	12	CT	15	6
33055	3	10797733	10797752	2	10	20	CA	18	7
33059	3	10811713	10811730	2	9	18	CA	19	7
33060	3	10812148	10812161	2	7	14	AC	26	5
33064	3	10822031	10822045	3	5	15	GCA	27	3
33065	3	10832373	10832412	4	10	40	AAAG	20	9
33068	3	10839740	10839755	2	8	16	AG	29	4
33070	3	10842180	10842199	2	10	20	AG	17	5
33071	3	10845441	10845454	2	7	14	CA	19	3
33073	3	10845806	10845831	2	13	26	TC	12	8
33074	3	10847266	10847295	3	10	30	GAG	24	7
33076	3	10860140	10860159	2	10	20	GA	26	9
33077	3	10863261	10863282	2	11	22	GT	20	18
33091	3	10907168	10907187	2	10	20	GT	23	5
33092	3	10909880	10909895	2	8	16	AC	21	3
33094	3	10921009	10921026	2	9	18	GA	9	4
33101	3	10937000	10937019	4	5	20	TCTG	28	4
33102	3	10947437	10947462	2	13	26	AC	12	12
33107	3	10960611	10960655	3	15	45	AGA	19	17
33111	3	10969224	10969247	4	6	24	ATGG	27	4
33113	3	10976728	10976749	2	11	22	AC	21	10

33115	3	10979366	10979380	3	5	15	CTC	18	4
33116	3	10981664	10981701	2	19	38	GT	6	8
33117	3	10981955	10981981	3	9	27	TCC	22	8
33119	3	10983282	10983293	2	6	12	CA	24	5
33120	3	10984136	10984159	4	6	24	ATCC	21	12
33122	3	10985485	10985499	3	5	15	ACA	31	3
33123	3	10986149	10986172	2	12	24	TG	15	14
33133	3	11009154	11009173	2	10	20	CT	7	5
33136	3	11009497	11009514	2	9	18	CT	18	3
33137	3	11009646	11009663	2	9	18	AC	11	10
33139	3	11011204	11011233	2	15	30	CT	16	8
33141	3	11011747	11011760	2	7	14	AC	25	3
33143	3	11012292	11012305	2	7	14	TC	15	4
33145	3	11012811	11012848	2	19	38	CA	6	7
33150	3	11014110	11014127	3	6	18	CAG	20	3
33152	3	11014564	11014595	2	16	32	TG	6	8
33153	3	11014819	11014846	2	14	28	CA	21	8
33160	3	11030347	11030360	2	7	14	AC	28	5
33161	3	11031125	11031151	3	9	27	AGA	6	3
33168	3	11061529	11061549	3	7	21	TGA	14	5
33183	3	11104147	11104172	2	13	26	TG	17	8
33184	3	11104399	11104422	4	6	24	TTTA	17	5
33185	3	11110071	11110082	2	6	12	CA	18	4
33190	3	11117426	11117453	4	7	28	TATT	23	4
33195	3	11123817	11123838	2	11	22	TG	21	13
33212	3	11158410	11158427	3	6	18	TAG	18	3
33219	3	11168378	11168395	3	6	18	TCC	24	4
33223	3	11175447	11175461	3	5	15	CAA	30	4
33235	3	11200028	11200045	2	9	18	AT	11	3
33236	3	11207647	11207666	2	10	20	AG	14	4
33240	3	11209199	11209230	2	16	32	AC	21	13
33241	3	11210244	11210269	2	13	26	GA	18	7
33249	3	11231294	11231315	2	11	22	AC	20	6
33253	3	11235568	11235585	3	6	18	CGC	15	3
33254	3	11235766	11235783	3	6	18	CTG	21	5
33255	3	11235850	11235871	2	11	22	AC	15	8
33256	3	11236038	11236061	4	6	24	GTTT	18	3
33258	3	11242723	11242746	3	8	24	TCA	8	8
33259	3	11243916	11243941	2	13	26	CA	6	7
33273	3	11286308	11286323	4	4	16	AATA	27	3
33279	3	11307855	11307872	2	9	18	AC	15	7
33280	3	11312728	11312741	2	7	14	CA	26	4
33281	3	11313118	11313135	3	6	18	CAG	23	4
33282	3	11313260	11313277	3	6	18	CAG	32	4
33289	3	11324777	11324790	2	7	14	AC	24	6
33290	3	11324969	11325004	3	12	36	CTC	22	6
33291	3	11336141	11336158	2	9	18	TG	27	3
33308	3	11379591	11379602	2	6	12	AG	14	4
33309	3	11379605	11379616	2	6	12	AG	19	9
33316	3	11392998	11393023	2	13	26	AC	17	10
33322	3	11409802	11409829	4	7	28	AGAA	14	12

33328	3	11419379	11419412	2	17	34	TG	14	15
33329	3	11422923	11422946	3	8	24	TGT	19	7
33340	3	11440385	11440396	2	6	12	TG	22	6
33341	3	11440398	11440409	2	6	12	GT	19	5
33352	3	11494087	11494100	2	7	14	AC	30	4
33355	3	11498008	11498047	4	10	40	ATCT	20	10
33357	3	11507996	11508017	2	11	22	AC	20	5
33359	3	11508705	11508728	2	12	24	AG	8	9
33362	3	11510950	11510961	2	6	12	CA	6	5
33364	3	11510999	11511019	3	7	21	TCA	14	4
33370	3	11528596	11528609	2	7	14	GT	33	4
33374	3	11536634	11536663	3	10	30	TGG	7	3
33375	3	11537012	11537032	3	7	21	TGC	31	6
33376	3	11541433	11541448	4	4	16	TTTC	17	5
33379	3	11542911	11542934	3	8	24	GGT	25	5
33380	3	11544849	11544862	2	7	14	AC	23	3
33382	3	11552578	11552591	2	7	14	CA	16	7
33383	3	11552596	11552611	2	8	16	CA	20	8
33388	3	11562859	11562874	4	4	16	AAAT	12	3
33398	3	11588278	11588303	2	13	26	TG	16	8
33400	3	11603503	11603526	2	12	24	AC	11	8
33401	3	11608185	11608204	2	10	20	CA	21	4
33408	3	11621510	11621523	2	7	14	AC	23	3
33422	3	11640407	11640430	2	12	24	CA	8	8
33440	3	11706036	11706049	2	7	14	TG	18	6
33441	3	11706673	11706684	2	6	12	AT	24	3
33443	3	11722664	11722677	2	7	14	TG	18	5
33444	3	11729644	11729667	2	12	24	AC	23	14
33445	3	11729753	11729782	2	15	30	CA	15	8
33446	3	11730267	11730290	3	8	24	GAG	22	8
33448	3	11733772	11733783	2	6	12	TG	26	6
33449	3	11734059	11734088	2	15	30	CA	9	10
33451	3	11736842	11736871	2	15	30	TG	10	7
33452	3	11737271	11737291	3	7	21	AGA	25	3
33453	3	11738042	11738059	2	9	18	CA	24	4
33456	3	11740785	11740816	2	16	32	GA	8	9
33457	3	11741143	11741154	2	6	12	GT	29	5
33459	3	11741637	11741650	2	7	14	AC	21	6
33460	3	11741653	11741666	2	7	14	AC	18	5
33463	3	11746280	11746297	2	9	18	CT	14	3
33464	3	11746611	11746630	2	10	20	GT	9	5
33473	3	11766898	11766939	2	21	42	CA	17	12
33478	3	11787234	11787249	2	8	16	CA	17	8
33479	3	11788396	11788409	2	7	14	TG	16	4
33485	3	11795623	11795640	2	9	18	TG	16	4
33487	3	11797007	11797034	2	14	28	TG	12	4
33497	3	11809129	11809140	2	6	12	GA	25	6
33499	3	11814481	11814504	2	12	24	TG	20	8
33503	3	11821628	11821651	3	8	24	GGA	6	5
33504	3	11822742	11822756	3	5	15	AAC	31	8
33507	3	11824619	11824648	3	10	30	CAG	20	3

33516	3	11838223	11838242	2	10	20	TG	18	3
33518	3	11838771	11838800	2	15	30	TG	10	4
33519	3	11839191	11839214	4	6	24	AGAC	31	4
33520	3	11840068	11840085	3	6	18	GCA	18	6
33521	3	11844127	11844147	3	7	21	CAT	26	3
33531	3	11853794	11853811	2	9	18	GA	22	4
33533	3	11855374	11855395	2	11	22	GT	19	10
33534	3	11855629	11855654	2	13	26	TG	15	5
33535	3	11858090	11858105	2	8	16	CA	30	5
33536	3	11858211	11858238	2	14	28	TG	18	8
33539	3	11861002	11861027	2	13	26	TG	21	9
33542	3	11861572	11861591	2	10	20	GT	18	9
33545	3	11864194	11864207	2	7	14	GT	36	3
33546	3	11865339	11865354	2	8	16	CT	15	7
33551	3	11883576	11883589	2	7	14	CA	30	3
33552	3	11883999	11884022	2	12	24	CA	18	15
33561	3	11899092	11899115	2	12	24	GA	19	5
33565	3	11907440	11907459	2	10	20	AG	8	6
33566	3	11908126	11908141	2	8	16	AC	10	3
33571	3	11912585	11912608	3	8	24	AGA	24	5
33574	3	11918431	11918442	2	6	12	TG	18	4
33576	3	11923267	11923298	2	16	32	GT	9	7
33578	3	11923438	11923463	2	13	26	CA	9	9
33581	3	11926424	11926441	2	9	18	CA	24	11
33582	3	11930081	11930094	2	7	14	TG	20	4
33583	3	11932852	11932869	3	6	18	GAA	29	3
33587	3	11934180	11934191	2	6	12	CA	28	6
33612	3	11963869	11963882	2	7	14	AC	27	4
33614	3	11965997	11966016	2	10	20	CT	17	4
33621	3	11975106	11975129	2	12	24	CA	17	10
33631	3	11991549	11991566	2	9	18	GT	15	14
33638	3	12003881	12003892	2	6	12	GA	27	4
33639	3	12005567	12005580	2	7	14	TG	31	6
33651	3	12020885	12020908	3	8	24	ACA	19	5
33657	3	12028783	12028800	3	6	18	TTG	29	3
33669	3	12046516	12046531	2	8	16	GT	17	10
33672	3	12050289	12050304	2	8	16	TG	19	10
33673	3	12050483	12050494	2	6	12	CA	20	4
33681	3	12062765	12062776	2	6	12	GT	23	5
33684	3	12064087	12064100	2	7	14	AC	21	5
33688	3	12078565	12078597	3	11	33	TCC	10	6
33690	3	12099112	12099123	2	6	12	AG	17	4
33691	3	12100444	12100459	2	8	16	CA	10	10
33692	3	12100515	12100542	2	14	28	AC	11	5
33698	3	12101274	12101313	2	20	40	CT	8	8
33699	3	12101518	12101531	2	7	14	AC	17	5
33701	3	12101555	12101586	2	16	32	CA	10	7
33706	3	12108899	12108918	2	10	20	CA	20	3
33710	3	12112592	12112603	2	6	12	GA	27	3
33713	3	12116565	12116582	3	6	18	GCT	22	5
33716	3	12122087	12122110	2	12	24	GT	11	7

33717	3	12122602	12122616	3	5	15	CCA	19	8
33722	3	12128110	12128166	3	19	57	GAA	14	14
33729	3	12146436	12146447	2	6	12	GT	8	6
33730	3	12146448	12146467	2	10	20	AT	6	7
33732	3	12149606	12149641	3	12	36	CTT	12	3
33736	3	12171266	12171277	2	6	12	CT	30	6
33737	3	12171297	12171310	2	7	14	TG	24	4
33740	3	12175195	12175208	2	7	14	CA	23	4
33744	3	12178137	12178152	2	8	16	GT	25	5
33753	3	12201683	12201714	2	16	32	CA	13	9
33758	3	12218398	12218439	2	21	42	AC	8	8
33765	3	12240461	12240476	4	4	16	AACC	26	5
33767	3	12246679	12246692	2	7	14	AT	20	6
33768	3	12248562	12248581	4	5	20	AAAT	15	3
33770	3	12254510	12254525	4	4	16	TTTC	13	7
33772	3	12254784	12254797	2	7	14	GA	19	4
33784	3	12276913	12276926	2	7	14	CA	40	3
33785	3	12279528	12279545	3	6	18	GTC	24	6
33788	3	12282356	12282369	2	7	14	AT	15	7
33789	3	12284283	12284298	2	8	16	CA	20	4
33793	3	12291617	12291630	2	7	14	TG	28	4
33809	3	12316843	12316896	2	27	54	AC	13	11
33813	3	12321975	12321992	3	6	18	AAG	34	6
33827	3	12360747	12360761	3	5	15	GCA	25	3
33828	3	12375753	12375796	4	11	44	TATC	14	8
33830	3	12381863	12381878	2	8	16	AC	26	3
33835	3	12384756	12384779	2	12	24	TG	21	8
33838	3	12387787	12387806	4	5	20	CACT	26	3
33840	3	12395794	12395808	3	5	15	AAG	19	3
33843	3	12408384	12408405	2	11	22	TG	8	9
33846	3	12412777	12412804	2	14	28	TG	17	5
33855	3	12427846	12427860	3	5	15	AAC	24	4
33857	3	12429913	12429924	2	6	12	TG	21	3
33859	3	12431915	12431952	2	19	38	CA	7	7
33860	3	12433607	12433626	2	10	20	GA	14	3
33886	3	12459049	12459087	3	13	39	TAC	20	19
33890	3	12461370	12461391	2	11	22	GA	17	15
33892	3	12461572	12461593	2	11	22	CA	13	11
33894	3	12461716	12461737	2	11	22	CA	14	16
33898	3	12464975	12464990	2	8	16	TC	7	5
33901	3	12466149	12466162	2	7	14	CA	17	8
33908	3	12482045	12482090	2	23	46	AC	9	7
33911	3	12493902	12493923	2	11	22	TG	8	12
33914	3	12497893	12497906	2	7	14	AT	14	5
33917	3	12499064	12499081	2	9	18	AC	7	8
33921	3	12502101	12502120	2	10	20	AG	6	8
33923	3	12502451	12502464	2	7	14	AG	12	3
33927	3	12503803	12503818	4	4	16	AAAT	24	3
33932	3	12506706	12506725	4	5	20	GAGC	18	9
33937	3	12507526	12507540	3	5	15	GAT	13	3
33939	3	12508644	12508661	3	6	18	CAT	20	8

33940	3	12508740	12508751	2	6	12	GT	21	3
33945	3	12513074	12513093	2	10	20	AG	12	7
33954	3	12516719	12516738	2	10	20	GT	16	4
33957	3	12517489	12517502	2	7	14	CA	11	8
33961	3	12522980	12523005	2	13	26	TG	6	3
33965	3	12524220	12524237	2	9	18	GT	28	3
33966	3	12526259	12526288	2	15	30	GT	9	15
33967	3	12529872	12529883	2	6	12	GT	21	5
33968	3	12530140	12530157	2	9	18	TG	12	4
33969	3	12534622	12534636	3	5	15	TTC	23	6
33970	3	12536051	12536068	2	9	18	TG	21	3
33971	3	12536115	12536128	2	7	14	TG	27	3
33975	3	12544653	12544688	2	18	36	TG	6	7
33979	3	12547025	12547040	2	8	16	CA	14	4
33980	3	12547369	12547386	2	9	18	TG	28	4
33984	3	12551645	12551686	2	21	42	CA	8	13
33987	3	12554539	12554558	4	5	20	TCTG	27	5
33992	3	12562611	12562628	2	9	18	AC	28	3
33993	3	12572221	12572272	2	26	52	CA	12	9
33997	3	12576271	12576290	2	10	20	TG	22	4
34000	3	12577997	12578020	2	12	24	GT	23	6
34003	3	12580646	12580663	2	9	18	TG	14	11
34008	3	12585815	12585850	2	18	36	CA	11	9
34011	3	12591196	12591221	2	13	26	GT	20	4
34012	3	12592953	12592976	2	12	24	AC	18	9
34014	3	12593765	12593778	2	7	14	GT	27	3
34017	3	12596714	12596725	2	6	12	CA	15	4
34018	3	12596806	12596831	2	13	26	CA	21	12
34019	3	12597087	12597106	4	5	20	CCAT	19	4
34037	3	12626944	12626963	2	10	20	GT	19	7
34039	3	12629582	12629603	2	11	22	CA	16	13
34040	3	12632645	12632666	2	11	22	AC	15	6
34048	3	12640371	12640384	2	7	14	AC	20	4
34049	3	12640947	12640976	3	10	30	TTC	24	4
34050	3	12641127	12641146	2	10	20	AC	19	5
34051	3	12641365	12641390	2	13	26	CA	9	8
34056	3	12652090	12652101	2	6	12	GT	25	4
34058	3	12653012	12653049	2	19	38	TG	9	8
34059	3	12654225	12654242	2	9	18	AC	26	7
34060	3	12657837	12657874	2	19	38	GT	10	9
34062	3	12658742	12658761	2	10	20	GT	20	7
34064	3	12661439	12661460	2	11	22	AC	21	3
34065	3	12664119	12664130	2	6	12	TG	22	9
34066	3	12664134	12664147	2	7	14	GA	20	7
34067	3	12665364	12665375	2	6	12	AT	11	4
34087	3	12693519	12693530	2	6	12	TG	10	6
34089	3	12695683	12695706	2	12	24	GA	12	5
34091	3	12695852	12695873	2	11	22	TG	20	14
34092	3	12695931	12695952	2	11	22	CA	18	12
34104	3	12707271	12707296	2	13	26	AC	8	10
34105	3	12707547	12707570	2	12	24	CA	19	9

34107	3	12714725	12714752	2	14	28	CA	16	13
34108	3	12734830	12734871	2	21	42	GA	6	8
34109	3	12734983	12734994	2	6	12	GA	18	6
34113	3	12740285	12740320	2	18	36	GT	8	9
34118	3	12742985	12743028	2	22	44	TG	18	7
34124	3	12747283	12747306	2	12	24	AC	21	9
34126	3	12747635	12747664	3	10	30	TCT	16	5
34130	3	12750797	12750826	2	15	30	GT	13	8
34133	3	12751627	12751654	2	14	28	GT	13	5
34144	3	12760920	12760945	2	13	26	CA	22	7
34145	3	12763527	12763544	2	9	18	GA	22	5
34146	3	12764146	12764173	2	14	28	AG	16	5
34150	3	12768233	12768276	2	22	44	CT	8	6
34151	3	12768736	12768755	2	10	20	AC	11	3
34155	3	12772160	12772173	2	7	14	CA	23	8
34158	3	12776368	12776383	2	8	16	GT	30	5
34160	3	12781839	12781852	2	7	14	CA	21	9
34163	3	12787339	12787356	2	9	18	CA	17	6
34164	3	12787359	12787372	2	7	14	CA	16	6
34167	3	12793593	12793620	2	14	28	CA	11	6
34172	3	12798436	12798451	2	8	16	TG	10	5
34179	3	12802780	12802807	2	14	28	GT	9	9
34184	3	12804791	12804824	2	17	34	TC	6	9
34188	3	12809483	12809500	2	9	18	GA	19	6
34189	3	12811198	12811209	2	6	12	GT	17	7
34190	3	12811211	12811224	2	7	14	TG	25	11
34200	3	12826917	12826932	4	4	16	AAAT	30	4
34202	3	12835179	12835208	2	15	30	AC	24	13
34206	3	12839386	12839415	2	15	30	CA	12	7
34209	3	12840285	12840300	2	8	16	AC	23	4
34210	3	12841280	12841295	2	8	16	AC	18	4
34213	3	12846965	12846976	2	6	12	AC	14	14
34214	3	12846979	12846994	2	8	16	AC	16	13
34222	3	12861616	12861645	2	15	30	CA	8	10
34224	3	12863011	12863026	2	8	16	TC	13	3
34225	3	12863228	12863247	2	10	20	GT	24	9
34227	3	12863460	12863483	2	12	24	AG	26	6
34230	3	12864146	12864163	2	9	18	GT	25	9
34233	3	12869611	12869636	2	13	26	TG	10	12
34235	3	12871721	12871740	4	5	20	AAAT	8	5
34238	3	12872212	12872223	2	6	12	AC	20	4
34248	3	12879473	12879492	2	10	20	TG	26	6
34252	3	12883568	12883611	2	22	44	TG	12	13
34256	3	12883824	12883838	3	5	15	GAG	17	8
34257	3	12884034	12884073	2	20	40	CA	15	17
34260	3	12886738	12886771	2	17	34	AC	14	7
34268	3	12891399	12891412	2	7	14	CA	29	6
34269	3	12891786	12891823	2	19	38	TG	10	7
34270	3	12892677	12892696	2	10	20	AC	14	4
34273	3	12894191	12894212	2	11	22	GT	16	9
34277	3	12906901	12906915	3	5	15	ATT	8	3

34288	3	12925151	12925168	2	9	18	GT	17	8
34290	3	12928590	12928619	2	15	30	TG	21	9
34291	3	12930735	12930780	2	23	46	TG	6	7
34292	3	12933229	12933242	2	7	14	CA	24	4
34293	3	12935347	12935374	2	14	28	CA	18	14
34300	3	12945476	12945491	2	8	16	AC	22	13
34307	3	12956141	12956162	2	11	22	GT	17	6
34309	3	12961736	12961749	2	7	14	TA	7	3
34312	3	12963741	12963760	4	5	20	TTTA	30	17
34314	3	12979036	12979051	4	4	16	AAAT	19	3
34319	3	12997815	12997838	4	6	24	TCCA	15	3
34325	3	13002860	13002873	2	7	14	AT	18	3
34326	3	13006659	13006676	2	9	18	AC	18	14
34328	3	13015052	13015067	4	4	16	AACA	24	3
34329	3	13016567	13016586	4	5	20	AAAT	32	6
34332	3	13018263	13018280	3	6	18	ATA	14	9
34335	3	13019409	13019423	3	5	15	AAG	27	9
34337	3	13020605	13020622	3	6	18	AGA	27	13
34341	3	13021114	13021125	2	6	12	TC	21	3
34342	3	13021170	13021199	2	15	30	TG	22	19
34343	3	13023866	13023880	3	5	15	GCT	26	6
34344	3	13031988	13032003	2	8	16	TG	16	4
34347	3	13032314	13032325	2	6	12	AC	18	13
34352	3	13040458	13040481	2	12	24	TC	12	7
34358	3	13047975	13047988	2	7	14	CA	25	4
34359	3	13049283	13049318	2	18	36	TG	21	13
34363	3	13051755	13051769	3	5	15	CTC	23	3
34367	3	13061972	13062003	2	16	32	TG	24	13
34368	3	13062980	13062993	2	7	14	TA	11	5
34370	3	13064374	13064399	2	13	26	CA	7	3
34371	3	13065902	13065939	2	19	38	CA	11	10
34374	3	13069250	13069261	2	6	12	GT	12	3
34380	3	13083127	13083162	4	9	36	ATCT	19	9
34394	3	13118400	13118417	3	6	18	AGG	31	3
34399	3	13122810	13122849	2	20	40	CA	15	16
34400	3	13122992	13123019	2	14	28	GA	15	7
34403	3	13123594	13123653	3	20	60	TCT	21	15
34404	3	13124168	13124193	2	13	26	AG	19	6
34406	3	13131889	13131900	2	6	12	GT	25	4
34409	3	13132985	13133020	3	12	36	GAG	11	8
34412	3	13134468	13134479	2	6	12	CA	8	3
34418	3	13144059	13144090	2	16	32	CT	17	13
34420	3	13144892	13144911	4	5	20	CCTG	25	3
34421	3	13145392	13145405	2	7	14	TG	10	3
34435	3	13155445	13155468	3	8	24	TGA	27	3
34437	3	13156116	13156129	2	7	14	TG	15	11
34442	3	13157704	13157721	2	9	18	TG	10	10
34444	3	13157882	13157895	2	7	14	GT	19	3
34449	3	13161126	13161157	2	16	32	AC	16	6
34451	3	13161403	13161424	2	11	22	TG	15	12
34453	3	13161905	13161928	2	12	24	CA	16	12

34454	3	13163390	13163409	2	10	20	AC	21	11
34456	3	13164049	13164068	2	10	20	TG	13	8
34461	3	13167756	13167769	2	7	14	GT	19	4
34462	3	13167770	13167781	2	6	12	GA	17	4
34465	3	13169776	13169791	4	4	16	ATTT	17	6
34467	3	13170145	13170166	2	11	22	TG	12	5
34468	3	13170269	13170310	2	21	42	AC	10	7
34478	3	13171471	13171488	2	9	18	TG	12	12
34480	3	13171691	13171732	2	21	42	TC	8	10
34483	3	13172171	13172186	2	8	16	TC	23	8
34489	3	13177874	13177891	3	6	18	CCT	26	4
34502	3	13217322	13217345	2	12	24	AC	10	6
34504	3	13226630	13226641	2	6	12	CA	13	4
34508	3	13230106	13230123	2	9	18	GT	20	3
34511	3	13233927	13233940	2	7	14	CA	18	3
34513	3	13236925	13236940	4	4	16	TGCG	14	5
34514	3	13237224	13237267	2	22	44	GT	8	9
34527	3	13259443	13259474	2	16	32	TG	12	10
34528	3	13260653	13260682	3	10	30	GGA	21	4
34529	3	13268517	13268540	2	12	24	TG	18	8
34531	3	13270960	13270989	2	15	30	TG	10	7
34532	3	13275707	13275746	2	20	40	CA	9	10
34534	3	13280495	13280506	2	6	12	TG	18	3
34569	3	13317495	13317514	2	10	20	TG	22	5
34570	3	13323233	13323252	2	10	20	AC	26	5
34572	3	13325255	13325274	2	10	20	TG	11	11
34575	3	13325895	13325910	2	8	16	GT	22	7
34578	3	13328791	13328818	2	14	28	GT	15	9
34579	3	13328901	13328916	2	8	16	CA	25	5
34580	3	13329051	13329076	2	13	26	TC	6	9
34583	3	13333167	13333178	2	6	12	GT	24	7
34591	3	13353979	13354004	2	13	26	TG	9	7
34592	3	13356236	13356279	2	22	44	CA	7	7
34593	3	13356792	13356805	2	7	14	AG	23	3
34595	3	13358592	13358629	2	19	38	CA	8	11
34597	3	13363211	13363238	2	14	28	CA	16	7
34599	3	13364560	13364571	2	6	12	GT	8	9
34600	3	13364573	13364588	2	8	16	TG	7	8
34604	3	13375222	13375259	2	19	38	TG	15	13
34605	3	13376043	13376064	2	11	22	GT	12	3
34606	3	13378420	13378443	3	8	24	TCC	10	3
34607	3	13379080	13379095	2	8	16	TG	22	5
34613	3	13388131	13388146	4	4	16	TGTT	28	3
34626	3	13411394	13411415	2	11	22	TG	23	6
34639	3	13430160	13430187	2	14	28	GT	12	6
34642	3	13442943	13442958	2	8	16	AC	20	5
34651	3	13460229	13460248	2	10	20	GT	11	4
34657	3	13461659	13461682	4	6	24	TTTG	31	3
34659	3	13466468	13466485	2	9	18	CA	24	4
34663	3	13469265	13469280	4	4	16	TTCT	23	5
34667	3	13471327	13471340	2	7	14	GT	20	8

34686	3	13506244	13506259	2	8	16	GT	18	8
34690	3	13507103	13507126	2	12	24	GT	12	6
34691	3	13509063	13509086	2	12	24	AC	24	6
34693	3	13510833	13510846	2	7	14	GT	11	3
34704	3	13526481	13526496	2	8	16	TG	16	4
34710	3	13536049	13536080	4	8	32	AAAG	21	6
34719	3	13545581	13545606	2	13	26	TG	16	4
34722	3	13554118	13554145	2	14	28	TG	12	14
34725	3	13558642	13558659	2	9	18	GT	22	3
34729	3	13569572	13569583	2	6	12	AC	14	5
34730	3	13569586	13569599	2	7	14	AC	17	7
34731	3	13569852	13569863	2	6	12	TC	29	5
34736	3	13579396	13579419	2	12	24	GT	13	4
34739	3	13584098	13584119	2	11	22	TC	22	4
34761	3	13613557	13613574	3	6	18	AAC	18	5
34764	3	13618325	13618340	2	8	16	CA	16	6
34765	3	13622112	13622141	2	15	30	CA	12	11
34767	3	13634132	13634157	2	13	26	GT	22	8
34772	3	13637567	13637584	2	9	18	CA	19	4
34786	3	13654426	13654461	2	18	36	GT	11	10
34787	3	13654858	13654879	2	11	22	CA	18	8
34788	3	13655053	13655068	2	8	16	TG	15	6
34789	3	13657642	13657667	2	13	26	TG	17	6
34793	3	13667952	13667969	3	6	18	ATT	22	3
34794	3	13670292	13670306	3	5	15	TGC	29	3
34797	3	13678861	13678908	4	12	48	GAAA	20	9
34815	3	13699692	13699713	2	11	22	GT	18	8
34817	3	13704287	13704300	2	7	14	TG	20	4
34852	3	13818685	13818704	4	5	20	AAAC	18	4
34868	3	13844171	13844192	2	11	22	AC	17	6
34875	3	13859671	13859682	2	6	12	AT	24	8
34889	3	13876799	13876818	2	10	20	TG	17	13
34901	3	13911329	13911348	2	10	20	GT	9	10
34903	3	13911749	13911762	2	7	14	TG	23	7
34906	3	13915388	13915405	2	9	18	AC	21	6
34910	3	13932333	13932348	4	4	16	GCAG	22	3
34915	3	13935254	13935269	2	8	16	TG	12	4
34917	3	13937166	13937186	3	7	21	CTC	26	11
34921	3	13938765	13938779	3	5	15	CCT	16	3
34924	3	13945815	13945854	2	20	40	TC	12	11
34940	3	13972008	13972033	2	13	26	AC	26	9
34953	3	14000604	14000623	2	10	20	AC	28	4
34961	3	14043701	14043712	2	6	12	AC	29	3
34963	3	14047504	14047537	2	17	34	TG	12	7
34970	3	14061424	14061439	2	8	16	CA	19	3
35002	3	14151116	14151131	4	4	16	TATT	19	7
35003	3	14152293	14152322	2	15	30	GT	22	11
35007	3	14169700	14169726	3	9	27	TAA	17	6
35018	3	14195786	14195797	2	6	12	CA	13	4
35021	3	14200061	14200080	4	5	20	AAAT	23	3
35033	3	14226552	14226603	2	26	52	AG	12	12

35059	3	14292588	14292611	2	12	24	GT	14	11
35061	3	14293585	14293604	4	5	20	AAAT	31	3
35063	3	14304350	14304363	2	7	14	AG	24	4
35065	3	14306861	14306888	2	14	28	AC	21	16
35074	3	14328480	14328527	2	24	48	CA	15	10
35082	3	14346497	14346510	2	7	14	GT	20	7
35084	3	14348821	14348836	2	8	16	AT	7	7
35095	3	14360904	14360939	4	9	36	ACAG	21	4
35097	3	14365451	14365466	2	8	16	CA	10	3
35103	3	14376552	14376567	2	8	16	CA	25	3
35104	3	14376747	14376758	2	6	12	TG	23	10
35110	3	14397253	14397284	2	16	32	AC	17	8
35112	3	14409300	14409315	2	8	16	AT	22	3
35116	3	14421121	14421147	3	9	27	ATT	22	4
35118	3	14429092	14429111	4	5	20	TGGT	22	4
35126	3	14448168	14448188	3	7	21	AAC	22	4
35129	3	14457889	14457906	2	9	18	AC	21	10
35138	3	14475150	14475165	2	8	16	CT	19	3
35140	3	14475929	14475944	2	8	16	AG	22	3
35145	3	14502167	14502182	4	4	16	CCTG	19	4
35158	3	14534301	14534322	2	11	22	AC	10	4
35159	3	14535105	14535116	2	6	12	AG	30	3
35161	3	14539269	14539288	2	10	20	AC	14	8
35168	3	14544376	14544407	2	16	32	GT	13	15
35170	3	14546209	14546224	4	4	16	TATT	13	4
35174	3	14548353	14548378	2	13	26	AC	17	9
35177	3	14555110	14555136	3	9	27	ATG	26	6
35178	3	14555768	14555791	2	12	24	TG	16	10
35181	3	14559445	14559472	2	14	28	TG	6	7
35182	3	14563090	14563109	2	10	20	TG	10	6
35186	3	14566892	14566911	2	10	20	AC	17	8
35198	3	14578850	14578863	2	7	14	GT	8	4
35200	3	14589816	14589835	2	10	20	GT	16	17
35201	3	14590005	14590020	4	4	16	AAAT	28	12
35203	3	14592649	14592666	2	9	18	TG	18	3
35217	3	14610064	14610075	2	6	12	TG	16	12
35230	3	14624738	14624752	3	5	15	AAC	14	4
35237	3	14625951	14625983	3	11	33	TCT	16	7
35239	3	14626678	14626691	2	7	14	GT	23	3
35240	3	14627165	14627196	2	16	32	TG	7	6
35241	3	14629437	14629474	2	19	38	TC	17	7
35242	3	14629621	14629634	2	7	14	TC	14	3
35243	3	14629639	14629650	2	6	12	TC	8	3
35244	3	14629760	14629785	2	13	26	TG	10	6
35250	3	14642869	14642882	2	7	14	TG	24	3
35253	3	14643262	14643289	2	14	28	AC	17	8
35257	3	14645276	14645291	2	8	16	CT	23	4
35259	3	14645907	14645927	3	7	21	AGA	18	5
35260	3	14646092	14646112	3	7	21	CTG	19	4
35267	3	14647381	14647398	3	6	18	TTC	19	8
35270	3	14650481	14650512	2	16	32	CA	20	7

35271	3	14650669	14650680	2	6	12	TG	20	4
35274	3	14655299	14655326	2	14	28	CA	24	5
35286	3	14676328	14676345	2	9	18	TG	21	8
35291	3	14693620	14693635	2	8	16	TG	21	6
35294	3	14701360	14701371	2	6	12	GA	33	3
35300	3	14706806	14706833	2	14	28	AC	13	8
35301	3	14707144	14707173	2	15	30	CA	22	7
35303	3	14711131	14711152	2	11	22	GT	11	5
35311	3	14719506	14719523	2	9	18	AG	19	6
35313	3	14721340	14721363	3	8	24	CTG	26	3
35314	3	14722003	14722017	3	5	15	GGA	25	4
35316	3	14723511	14723524	2	7	14	GT	16	4
35317	3	14723526	14723537	2	6	12	TG	18	5
35337	3	14780470	14780487	3	6	18	TGT	31	4
35342	3	14787620	14787643	2	12	24	CA	10	16
35343	3	14789797	14789812	2	8	16	AC	14	5
35345	3	14790199	14790210	2	6	12	AC	19	3
35346	3	14796725	14796758	2	17	34	TG	18	8
35349	3	14797079	14797112	2	17	34	AC	6	7
35357	3	14813365	14813382	2	9	18	CT	18	6
35358	3	14813527	14813558	2	16	32	TG	8	10
35360	3	14816520	14816533	2	7	14	GT	19	8
35361	3	14816536	14816547	2	6	12	GT	22	11
35364	3	14817255	14817292	2	19	38	CA	6	9
35366	3	14819543	14819610	2	34	68	CA	11	11
35370	3	14821500	14821523	2	12	24	GT	13	5
35372	3	14822317	14822332	2	8	16	AC	26	4
35374	3	14824209	14824224	4	4	16	TCTT	14	6
35376	3	14824839	14824872	2	17	34	GT	11	9
35377	3	14824970	14824997	2	14	28	AC	13	11
35378	3	14827060	14827093	2	17	34	AC	15	18
35379	3	14829066	14829081	2	8	16	CA	20	10
35380	3	14829850	14829877	2	14	28	CA	15	8
35382	3	14831700	14831717	3	6	18	GAG	25	4
35384	3	14836358	14836425	2	34	68	AC	14	12
35387	3	14840418	14840447	2	15	30	AC	6	6
35391	3	14848177	14848203	3	9	27	AAC	22	12
35393	3	14851867	14851878	2	6	12	GT	26	3
35397	3	14863287	14863302	2	8	16	TG	23	5
35399	3	14863515	14863530	2	8	16	AC	12	5
35400	3	14864290	14864309	2	10	20	CA	18	7
35401	3	14866708	14866725	3	6	18	CCT	25	4
35403	3	14867464	14867487	2	12	24	AC	17	7
35407	3	14874042	14874055	2	7	14	TG	21	4
35408	3	14875552	14875565	2	7	14	CA	30	5
35420	3	14890503	14890520	2	9	18	GA	26	4
35421	3	14893342	14893355	2	7	14	GT	30	3
35426	3	14896069	14896084	2	8	16	GT	11	3
35434	3	14900805	14900816	2	6	12	AC	9	3
35437	3	14903759	14903779	3	7	21	ATA	9	4
35438	3	14904525	14904545	3	7	21	GAA	27	4

35439	3	14904860	14904871	2	6	12	AC	20	6
35446	3	14919153	14919204	4	13	52	ATCT	9	13
35447	3	14919314	14919335	2	11	22	TA	11	6
35449	3	14920014	14920028	3	5	15	GAA	22	5
35450	3	14920146	14920173	4	7	28	GCCT	20	4
35453	3	14920600	14920627	2	14	28	AC	18	14
35457	3	14921137	14921163	3	9	27	CTT	14	8
35461	3	14921627	14921640	2	7	14	GA	12	13
35462	3	14921983	14921998	2	8	16	TG	18	3
35470	3	14926817	14926854	2	19	38	GA	13	11
35487	3	14979986	14980009	3	8	24	CTG	16	3
35488	3	14980126	14980147	2	11	22	AG	10	8
35490	3	14984440	14984489	2	25	50	CA	6	5
35508	3	15021362	15021377	2	8	16	GT	17	4
35513	3	15033751	15033780	2	15	30	GT	14	10
35514	3	15035278	15035301	2	12	24	TC	19	8
35519	3	15042510	15042541	2	16	32	GT	6	5
35520	3	15043865	15043886	2	11	22	GT	14	9
35522	3	15045102	15045127	2	13	26	TG	21	8
35529	3	15054279	15054310	2	16	32	TG	8	8
35533	3	15060959	15060974	4	4	16	GTGC	29	4
35535	3	15069670	15069695	2	13	26	TG	13	7
35537	3	15074341	15074356	2	8	16	GT	19	9
35538	3	15074447	15074491	3	15	45	CTT	17	13
35539	3	15074551	15074564	2	7	14	TC	21	4
35542	3	15075026	15075055	2	15	30	TG	12	7
35544	3	15080423	15080442	2	10	20	CA	15	10
35545	3	15081592	15081611	2	10	20	CA	18	15
35548	3	15085340	15085357	3	6	18	GTT	31	5
35554	3	15103035	15103052	3	6	18	GTA	26	5
35556	3	15116007	15116052	2	23	46	CA	16	5
35557	3	15123181	15123214	2	17	34	GT	12	12
35558	3	15123993	15124008	2	8	16	GT	24	4
35559	3	15129166	15129177	2	6	12	AC	23	3
35560	3	15129370	15129383	2	7	14	TG	28	3
35561	3	15132202	15132215	2	7	14	CA	21	5
35562	3	15133969	15133992	4	6	24	ATGA	23	5
35564	3	15138642	15138655	2	7	14	AG	18	3
35567	3	15143881	15143902	2	11	22	AG	15	5
35568	3	15145017	15145032	2	8	16	TG	28	6
35586	3	15181946	15181961	2	8	16	AC	26	3
35589	3	15187120	15187135	4	4	16	GGCA	26	4
35590	3	15187243	15187257	3	5	15	CAG	27	3
35591	3	15187441	15187461	3	7	21	CAG	27	4
35593	3	15190676	15190690	3	5	15	GCT	33	3
35595	3	15193014	15193039	2	13	26	TG	12	8
35597	3	15195267	15195284	2	9	18	GT	18	14
35599	3	15198352	15198366	3	5	15	GAG	28	3
35602	3	15201761	15201775	3	5	15	TTC	10	4
35619	3	15221216	15221237	2	11	22	AC	15	6
35622	3	15226413	15226424	2	6	12	CA	24	4

35624	3	15232741	15232760	2	10	20	CA	19	9
35626	3	15234286	15234315	3	10	30	CTC	16	4
35628	3	15241844	15241859	2	8	16	TG	16	7
35630	3	15243793	15243842	2	25	50	CA	14	9
35635	3	15255866	15255885	2	10	20	AT	20	10
35636	3	15257058	15257097	4	10	40	AGTT	16	4
35643	3	15260724	15260745	2	11	22	GT	22	7
35644	3	15261413	15261428	2	8	16	GT	15	4
35646	3	15264378	15264395	2	9	18	TA	16	4
35647	3	15268601	15268627	3	9	27	AGG	19	5
35651	3	15275133	15275150	2	9	18	AC	12	7
35654	3	15276187	15276202	2	8	16	GT	25	11
35656	3	15278373	15278390	2	9	18	TG	21	9
35659	3	15278962	15278983	2	11	22	CA	14	11
35661	3	15280745	15280759	3	5	15	AGA	23	8
35662	3	15281275	15281286	2	6	12	GT	19	5
35663	3	15281288	15281303	2	8	16	TG	16	4
35664	3	15281453	15281478	2	13	26	TG	19	9
35665	3	15283409	15283424	2	8	16	GT	22	3
35667	3	15283547	15283574	2	14	28	AC	10	7
35668	3	15283794	15283807	2	7	14	AC	21	5
35671	3	15285326	15285347	2	11	22	CA	16	9
35672	3	15285504	15285529	2	13	26	CA	11	7
35676	3	15288575	15288594	4	5	20	AAAT	27	3
35677	3	15289905	15289928	2	12	24	GT	25	8
35686	3	15304556	15304575	2	10	20	TG	17	3
35687	3	15307080	15307103	2	12	24	GT	11	6
35695	3	15316476	15316491	2	8	16	TG	20	3
35699	3	15322869	15322896	2	14	28	CA	21	6
35704	3	15328597	15328608	2	6	12	GT	13	10
35705	3	15330442	15330453	2	6	12	TG	18	7
35710	3	15335759	15335776	3	6	18	GGA	6	4
35713	3	15347553	15347570	2	9	18	CA	15	11
35714	3	15348775	15348796	2	11	22	AC	23	5
35716	3	15349689	15349706	3	6	18	GAG	20	4
35721	3	15355835	15355856	2	11	22	GT	20	6
35722	3	15356092	15356105	2	7	14	AC	31	5
35723	3	15358488	15358499	2	6	12	CT	13	3
35724	3	15362588	15362603	2	8	16	GT	26	3
35725	3	15363011	15363028	2	9	18	GT	20	4
35726	3	15366567	15366578	2	6	12	GT	30	3
35728	3	15367845	15367870	2	13	26	CA	8	10
35732	3	15371785	15371805	3	7	21	CCT	21	3
35733	3	15374616	15374631	2	8	16	GA	22	3
35749	3	15405142	15405157	2	8	16	AC	19	5
35750	3	15407890	15407907	2	9	18	TC	25	4
35755	3	15412161	15412186	2	13	26	TG	16	8
35756	3	15412343	15412362	2	10	20	CT	21	3
35758	3	15413202	15413223	2	11	22	CA	29	5
35759	3	15414204	15414225	2	11	22	AC	18	7
35760	3	15414406	15414427	2	11	22	CA	7	6

35761	3	15414492	15414503	2	6	12	CA	17	7
35763	3	15416546	15416575	2	15	30	AC	12	8
35769	3	15419289	15419318	2	15	30	CA	14	5
35770	3	15419538	15419559	2	11	22	TC	25	5
35771	3	15419855	15419878	3	8	24	GGA	23	3
35775	3	15420504	15420518	3	5	15	GGA	19	5
35778	3	15421226	15421241	2	8	16	TC	24	3
35779	3	15421768	15421823	2	28	56	TG	7	7
35780	3	15421920	15421945	2	13	26	CA	14	10
35781	3	15422529	15422552	3	8	24	GCT	19	4
35782	3	15424182	15424227	2	23	46	TG	11	16
35785	3	15425690	15425719	2	15	30	CA	7	10
35788	3	15426039	15426078	2	20	40	AC	12	16
35789	3	15426368	15426381	2	7	14	AG	18	5
35797	3	15427201	15427216	4	4	16	AAAG	26	3
35798	3	15427275	15427286	2	6	12	GA	16	14
35799	3	15427287	15427306	2	10	20	GT	16	18
35805	3	15429299	15429318	4	5	20	AAAT	20	4
35807	3	15430139	15430176	2	19	38	GA	15	12
35808	3	15430297	15430318	2	11	22	AC	12	7
35809	3	15430910	15430939	3	10	30	AGG	22	5
35812	3	15432159	15432178	4	5	20	GAAT	24	5
35813	3	15433756	15433769	2	7	14	AC	30	3
35814	3	15433829	15433843	3	5	15	AAG	27	3
35816	3	15436782	15436801	4	5	20	AAAC	21	3
35819	3	15441109	15441152	4	11	44	GAAA	14	12
35826	3	15452523	15452544	2	11	22	TG	15	7
35835	3	15465754	15465769	4	4	16	TTTG	19	3
35839	3	15467062	15467089	4	7	28	AAAC	23	3
35840	3	15468045	15468060	2	8	16	AC	15	3
35841	3	15469795	15469812	2	9	18	GA	23	5
35845	3	15470623	15470670	2	24	48	CA	11	11
35849	3	15473193	15473207	3	5	15	GCT	30	5
35853	3	15478580	15478621	2	21	42	AC	6	6
35855	3	15483151	15483162	2	6	12	GC	13	6
35856	3	15483624	15483643	2	10	20	TG	13	10
35857	3	15483705	15483746	3	14	42	AGA	10	8
35860	3	15485567	15485590	2	12	24	AC	13	10
35862	3	15488174	15488187	2	7	14	TG	28	3
35863	3	15497610	15497633	2	12	24	AC	14	9
35869	3	15525302	15525337	4	9	36	GACA	24	16
35873	3	15551085	15551100	2	8	16	GA	24	9
35877	3	15554594	15554613	2	10	20	GT	20	11
35889	3	15576556	15576567	2	6	12	CA	25	3
35891	3	15578258	15578281	2	12	24	GT	17	7
35892	3	15580401	15580416	4	4	16	TTTA	17	4
35895	3	15580868	15580882	3	5	15	GCT	24	3
35897	3	15583189	15583216	2	14	28	TG	8	8
35899	3	15584672	15584691	2	10	20	TG	21	6
35900	3	15589934	15589969	2	18	36	AC	6	8
35902	3	15596264	15596284	3	7	21	TGC	22	5

35904	3	15614651	15614662	2	6	12	AG	20	3
35910	3	15634495	15634524	3	10	30	GGA	20	3
35911	3	15634705	15634722	3	6	18	GGA	12	3
35912	3	15634925	15634946	2	11	22	GT	21	9
35913	3	15635004	15635015	2	6	12	AC	31	3
35914	3	15635182	15635193	2	6	12	CA	25	5
35915	3	15636484	15636499	2	8	16	GT	9	10
35916	3	15637092	15637105	2	7	14	TG	23	6
35918	3	15638931	15638942	2	6	12	AC	31	3
35919	3	15639147	15639172	2	13	26	TG	15	6
35924	3	15645065	15645078	2	7	14	GT	18	5
35934	3	15666181	15666194	2	7	14	AC	18	4
35935	3	15668556	15668589	2	17	34	CA	21	17
35941	3	15674080	15674093	2	7	14	AC	21	5
35944	3	15690729	15690742	2	7	14	TA	14	3
35946	3	15696535	15696549	3	5	15	ATT	28	5
35949	3	15700945	15700978	2	17	34	TG	9	6
35951	3	15701915	15701932	2	9	18	GT	19	8
35955	3	15710661	15710688	2	14	28	TG	25	9
35956	3	15711198	15711223	2	13	26	CA	20	18
35960	3	15716149	15716182	2	17	34	TG	10	10
35961	3	15718988	15719001	2	7	14	AT	13	7
35966	3	15719428	15719441	2	7	14	TC	13	4
35968	3	15720399	15720414	2	8	16	TG	10	7
35971	3	15722484	15722513	2	15	30	TG	19	7
35973	3	15724431	15724446	2	8	16	TG	32	3
35977	3	15731626	15731647	2	11	22	AC	10	8
35978	3	15733502	15733519	2	9	18	CA	33	3
35979	3	15733688	15733713	2	13	26	CA	20	7
35980	3	15735266	15735283	2	9	18	AG	21	3
35983	3	15736630	15736663	2	17	34	TG	7	8
35990	3	15740696	15740719	2	12	24	AG	16	9
35996	3	15747267	15747294	2	14	28	GT	13	8
36001	3	15756265	15756284	4	5	20	TAGA	17	8
36003	3	15757065	15757100	2	18	36	CA	10	7
36004	3	15757249	15757266	2	9	18	GT	21	4
36006	3	15757715	15757740	2	13	26	CA	18	9
36019	3	15772875	15772910	3	12	36	TAA	14	8
36020	3	15776047	15776060	2	7	14	TG	18	4
36021	3	15776227	15776264	2	19	38	AC	14	7
36027	3	15785352	15785381	2	15	30	CA	19	12
36029	3	15787659	15787694	2	18	36	CA	6	7
36030	3	15798999	15799043	3	15	45	CCT	9	5
36037	3	15802795	15802816	2	11	22	CA	13	7
36040	3	15809937	15809952	2	8	16	GT	32	4
36064	3	15890128	15890147	2	10	20	AC	22	7
36070	3	15910590	15910613	4	6	24	TTTG	32	3
36076	3	15924852	15924907	2	28	56	AC	11	14
36077	3	15925453	15925492	2	20	40	CA	14	11
36086	3	15949136	15949151	2	8	16	CA	21	3
36087	3	15949472	15949501	2	15	30	GT	17	4

36093	3	15954643	15954664	2	11	22	GT	15	11
36105	3	15984679	15984692	2	7	14	GT	22	8
36106	3	15985408	15985419	2	6	12	GT	18	9
36108	3	15986271	15986300	2	15	30	GA	18	6
36118	3	16003051	16003080	2	15	30	TG	17	8
36120	3	16003643	16003674	2	16	32	TG	10	8
36123	3	16012119	16012144	2	13	26	AC	21	6
36124	3	16012494	16012507	2	7	14	CA	21	3
36127	3	16018624	16018639	4	4	16	GAGG	36	3
36129	3	16021241	16021256	2	8	16	CA	24	11
36132	3	16021760	16021779	2	10	20	CT	13	9
36137	3	16025976	16025989	2	7	14	AC	17	6
36138	3	16027472	16027491	2	10	20	CA	16	9
36139	3	16028043	16028056	2	7	14	TG	19	5
36141	3	16028298	16028313	2	8	16	AC	17	4
36143	3	16039776	16039789	2	7	14	GT	26	3
36149	3	16050175	16050198	4	6	24	ATCC	23	3
36152	3	16051288	16051307	2	10	20	TG	10	4
36155	3	16054162	16054201	2	20	40	TG	16	10
36158	3	16058978	16059005	2	14	28	CA	7	5
36159	3	16059168	16059193	2	13	26	CA	10	7
36162	3	16062756	16062767	2	6	12	GA	10	3
36163	3	16064183	16064214	2	16	32	CA	12	5
36167	3	16069845	16069872	2	14	28	CA	21	10
36170	3	16073293	16073310	2	9	18	GT	12	5
36172	3	16074151	16074162	2	6	12	AC	17	4
36177	3	16076466	16076487	2	11	22	TG	14	10
36180	3	16080102	16080125	2	12	24	TG	10	7
36185	3	16089424	16089439	4	4	16	AAAT	26	3
36194	3	16093620	16093651	2	16	32	CA	9	6
36195	3	16095854	16095889	2	18	36	GT	12	8
36197	3	16096870	16096883	2	7	14	CA	20	3
36200	3	16105175	16105194	2	10	20	GT	14	5
36202	3	16105601	16105624	2	12	24	GT	17	11
36211	3	16124838	16124849	2	6	12	GT	19	3
36212	3	16126170	16126193	2	12	24	AC	24	6
36214	3	16132941	16132956	2	8	16	GT	17	8
36216	3	16136412	16136425	2	7	14	GT	26	4
36218	3	16147431	16147444	2	7	14	GT	28	5
36224	3	16161379	16161406	2	14	28	CA	7	6
36225	3	16161572	16161585	2	7	14	TG	17	10
36228	3	16174370	16174385	2	8	16	AC	26	6
36230	3	16179666	16179687	2	11	22	TG	18	11
36233	3	16196005	16196048	4	11	44	TTCT	9	4
36246	3	16229054	16229087	2	17	34	GT	19	10
36249	3	16232394	16232417	2	12	24	TG	15	10
36251	3	16253512	16253531	4	5	20	ATAA	7	3
36252	3	16258112	16258123	2	6	12	TA	20	4
36263	3	16287350	16287363	2	7	14	AC	29	3
36264	3	16287575	16287588	2	7	14	TG	29	3
36271	3	16295550	16295571	2	11	22	GA	17	4

36272	3	16297186	16297201	2	8	16	AG	17	3
36274	3	16299212	16299241	2	15	30	CA	19	9
36280	3	16307843	16307854	2	6	12	AC	21	8
36287	3	16317734	16317757	2	12	24	GT	25	8
36288	3	16319156	16319167	2	6	12	TC	26	3
36289	3	16324517	16324528	2	6	12	AC	30	3
36290	3	16327587	16327614	2	14	28	AC	15	11
36293	3	16332525	16332536	2	6	12	AC	24	3
36294	3	16332540	16332557	2	9	18	CA	21	4
36296	3	16332923	16332934	2	6	12	GT	21	3
36298	3	16335384	16335399	4	4	16	AATG	33	3
36299	3	16338315	16338338	4	6	24	TTTC	23	5
36307	3	16373921	16373950	2	15	30	CA	27	10
36310	3	16385431	16385445	3	5	15	CAG	27	5
36325	3	16400758	16400771	2	7	14	AC	20	5
36329	3	16409425	16409444	2	10	20	GT	19	7
36333	3	16414842	16414855	2	7	14	AC	22	8
36334	3	16414857	16414872	2	8	16	CA	21	10
36335	3	16415200	16415215	4	4	16	AAGA	11	5
36336	3	16415394	16415429	2	18	36	CA	11	8
36338	3	16417094	16417117	2	12	24	TG	24	11
36339	3	16419169	16419208	2	20	40	GT	8	14
36343	3	16441429	16441456	2	14	28	GT	24	6
36345	3	16446042	16446069	2	14	28	AC	16	11
36353	3	16463947	16463958	2	6	12	TG	29	3
36361	3	16473754	16473793	2	20	40	AC	11	13
36375	3	16511379	16511390	2	6	12	GA	20	5
36376	3	16514404	16514421	3	6	18	GGT	22	4
36378	3	16519374	16519399	2	13	26	GT	20	12
36379	3	16529668	16529683	4	4	16	GTCT	27	6
36384	3	16551739	16551762	2	12	24	AC	18	8
36387	3	16562281	16562340	4	15	60	TATC	14	12
36403	3	16583861	16583886	2	13	26	CA	16	12
36410	3	16591136	16591151	2	8	16	AT	10	4
36411	3	16597140	16597159	4	5	20	CTGT	28	5
36413	3	16607201	16607236	2	18	36	CA	9	7
36414	3	16611928	16611943	2	8	16	CA	16	4
36416	3	16614973	16614988	4	4	16	CAAA	23	3
36420	3	16616904	16616927	2	12	24	GA	13	4
36423	3	16617424	16617453	3	10	30	GGA	23	8
36424	3	16617594	16617609	4	4	16	AAAG	20	5
36425	3	16618052	16618071	4	5	20	TTTG	24	3
36428	3	16618976	16618999	2	12	24	AG	18	9
36432	3	16627879	16627892	2	7	14	CA	24	3
36433	3	16630928	16630941	2	7	14	TC	20	7
36450	3	16661358	16661373	2	8	16	GT	26	4
36457	3	16669071	16669084	2	7	14	AT	18	5
36459	3	16675486	16675545	2	30	60	TG	16	9
36461	3	16677114	16677137	2	12	24	TC	22	5
36474	3	16703732	16703743	2	6	12	GA	28	4
36476	3	16704350	16704367	2	9	18	TG	22	6

36481	3	16716206	16716231	2	13	26	CT	10	6
36483	3	16721898	16721931	2	17	34	AC	6	7
36485	3	16724691	16724723	3	11	33	TCT	23	10
36488	3	16726191	16726211	3	7	21	ACA	20	8
36491	3	16730904	16730924	3	7	21	CTT	26	7
36492	3	16733122	16733148	3	9	27	CCT	25	6
36496	3	16737661	16737678	2	9	18	AG	20	6
36497	3	16737756	16737773	2	9	18	GT	21	8
36498	3	16738078	16738089	2	6	12	GT	12	6
36500	3	16738181	16738206	2	13	26	TG	9	9
36501	3	16738473	16738494	2	11	22	CA	20	7
36503	3	16741715	16741744	3	10	30	CCA	9	4
36508	3	16752347	16752414	4	17	68	GATA	17	14
36509	3	16753645	16753664	2	10	20	TG	22	8
36510	3	16753718	16753729	2	6	12	AC	23	3
36512	3	16754352	16754389	2	19	38	AC	11	10
36516	3	16758879	16758898	2	10	20	GT	18	6
36517	3	16760180	16760203	2	12	24	CT	10	4
36521	3	16761692	16761715	2	12	24	TG	11	12
36522	3	16763607	16763624	2	9	18	GA	23	6
36525	3	16769037	16769060	2	12	24	AC	16	7
36532	3	16775131	16775181	3	17	51	CTG	25	14
36534	3	16777631	16777664	2	17	34	CA	7	9
36537	3	16781202	16781233	2	16	32	CA	17	12
36539	3	16787516	16787547	2	16	32	CA	21	10
36548	3	16800594	16800608	3	5	15	GAA	25	4
36550	3	16804751	16804768	3	6	18	AGT	21	4
36552	3	16809464	16809489	2	13	26	TG	22	11
36558	3	16826798	16826815	2	9	18	AC	25	5
36559	3	16827504	16827515	2	6	12	GT	29	3
36560	3	16827844	16827873	2	15	30	AC	19	11
36561	3	16829843	16829854	2	6	12	AC	17	3
36563	3	16838171	16838188	3	6	18	GGA	8	3
36564	3	16838573	16838590	2	9	18	CA	13	3
36565	3	16843504	16843543	4	10	40	TTGG	23	4
36568	3	16845287	16845316	2	15	30	AC	11	11
36574	3	16867010	16867041	2	16	32	GT	17	10
36576	3	16867519	16867542	3	8	24	CTC	22	3
36579	3	16871787	16871820	2	17	34	TG	9	10
36586	3	16890223	16890242	2	10	20	CA	25	10
36590	3	16896530	16896547	2	9	18	TG	19	8
36591	3	16896789	16896803	3	5	15	GGT	20	4
36596	3	16905149	16905164	2	8	16	TC	21	3
36601	3	16910247	16910260	2	7	14	AC	21	5
36608	3	16914555	16914578	2	12	24	TC	13	6
36615	3	16917387	16917400	2	7	14	GT	19	5
36616	3	16917402	16917415	2	7	14	TG	15	3
36617	3	16917498	16917515	2	9	18	GT	16	9
36618	3	16918570	16918607	2	19	38	AC	21	16
36619	3	16918779	16918802	2	12	24	CA	17	7
36628	3	16936803	16936820	2	9	18	GT	22	11

36629	3	16936965	16936986	2	11	22	GA	21	6
36633	3	16941569	16941586	2	9	18	TG	20	4
36634	3	16947495	16947506	2	6	12	AG	33	4
36635	3	16951474	16951491	2	9	18	AC	24	6
36636	3	16953405	16953416	2	6	12	TG	25	3
36637	3	16959623	16959658	2	18	36	TG	13	9
36638	3	16964294	16964319	2	13	26	AG	24	5
36640	3	16964511	16964528	2	9	18	CA	10	9
36644	3	16971771	16971803	3	11	33	GGA	10	7
36647	3	16973090	16973113	2	12	24	GT	15	4
36649	3	16981404	16981427	2	12	24	CA	19	11
36650	3	16981565	16981606	3	14	42	CTT	14	9
36656	3	16987670	16987695	2	13	26	AC	12	5
36658	3	16990299	16990316	2	9	18	CT	14	5
36661	3	16995326	16995363	2	19	38	TG	8	8
36663	3	17002437	17002457	3	7	21	TGT	22	5
36672	3	17013657	17013674	2	9	18	GT	15	8
36675	3	17022261	17022286	2	13	26	TG	9	11
36679	3	17051589	17051600	2	6	12	TG	25	3
36680	3	17051700	17051713	2	7	14	GA	23	5
36686	3	17071606	17071617	2	6	12	AC	26	5
36687	3	17071618	17071632	3	5	15	CAG	25	5
36688	3	17077281	17077295	3	5	15	GCT	7	4
36694	3	17087944	17087961	2	9	18	TG	14	4
36700	3	17099671	17099682	2	6	12	AC	26	4
36701	3	17101410	17101425	2	8	16	CA	24	4
36703	3	17106589	17106644	2	28	56	TG	9	8
36708	3	17121176	17121187	2	6	12	TC	24	15
36709	3	17121188	17121203	2	8	16	AC	26	17
36718	3	17132971	17133008	2	19	38	TC	9	11
36726	3	17148838	17148861	3	8	24	GCA	23	17
36729	3	17149229	17149252	3	8	24	ATG	18	3
36731	3	17150507	17150520	2	7	14	TG	29	5
36732	3	17153088	17153099	2	6	12	GA	20	3
36733	3	17158359	17158379	3	7	21	TGT	27	5
36734	3	17166782	17166802	3	7	21	CCT	30	7
36735	3	17170895	17170918	2	12	24	GT	11	8
36736	3	17173952	17173975	2	12	24	TG	11	7
36737	3	17174636	17174649	2	7	14	AC	15	7
36747	3	17206206	17206232	3	9	27	TGC	23	5
36750	3	17213744	17213785	2	21	42	AG	6	6
36763	3	17257136	17257159	2	12	24	TG	25	9
36764	3	17257570	17257589	4	5	20	AAAG	24	12
36771	3	17291655	17291682	4	7	28	TATG	13	11
36773	3	17294533	17294558	2	13	26	AC	22	6
36777	3	17303605	17303636	4	8	32	TGTT	11	5
36778	3	17304023	17304049	3	9	27	TGC	20	4
36780	3	17312654	17312681	4	7	28	TTTG	24	8
36781	3	17312872	17312891	4	5	20	AAAT	25	3
36782	3	17313212	17313227	2	8	16	AG	11	3
36792	3	17332339	17332354	2	8	16	TG	13	5

36803	3	17375775	17375788	2	7	14	AG	12	3
36805	3	17384577	17384590	2	7	14	CA	18	3
36810	3	17410585	17410596	2	6	12	AT	18	3
36811	3	17411623	17411666	2	22	44	TC	9	11
36813	3	17411933	17411950	3	6	18	AAG	22	6
36820	3	17421019	17421030	2	6	12	GA	29	3
36821	3	17423865	17423894	2	15	30	TG	12	9
36822	3	17424846	17424865	2	10	20	AT	8	4
36834	3	17454375	17454386	2	6	12	GT	29	3
36841	3	17475105	17475120	4	4	16	CCAT	28	7
36844	3	17475795	17475818	2	12	24	GA	6	10
36846	3	17475860	17475871	2	6	12	GA	14	3
36851	3	17481244	17481271	2	14	28	AC	15	6
36854	3	17488304	17488319	2	8	16	TC	18	5
36855	3	17495350	17495367	2	9	18	GA	12	4
36861	3	17505602	17505622	3	7	21	AAG	25	5
36869	3	17517721	17517738	3	6	18	CTG	28	3
36872	3	17530651	17530664	2	7	14	GT	30	5
36874	3	17533344	17533361	2	9	18	AC	17	6
36877	3	17541287	17541318	2	16	32	TG	17	7
36882	3	17571639	17571656	2	9	18	TA	16	7
36884	3	17589347	17589370	4	6	24	ATAA	20	5
36886	3	17593039	17593052	2	7	14	AC	10	8
36887	3	17593054	17593069	2	8	16	CA	14	11
36888	3	17593370	17593393	4	6	24	TAAA	24	6
36889	3	17596046	17596061	2	8	16	CA	14	10
36890	3	17596237	17596256	2	10	20	CA	23	5
36899	3	17612874	17612894	3	7	21	CTT	26	4
36901	3	17620123	17620138	4	4	16	AAAT	19	3
36908	3	17624962	17624983	2	11	22	TG	17	11
36910	3	17625184	17625197	2	7	14	AC	19	5
36911	3	17625281	17625304	2	12	24	AC	26	10
36913	3	17625634	17625647	2	7	14	CT	13	5
36915	3	17625835	17625850	2	8	16	AG	16	4
36916	3	17626089	17626112	3	8	24	CTG	28	8
36917	3	17626259	17626279	3	7	21	CAA	29	5
36926	3	17640081	17640096	2	8	16	GT	11	13
36928	3	17643732	17643751	2	10	20	CA	19	8
36930	3	17644427	17644444	2	9	18	GT	18	10
36939	3	17657062	17657077	2	8	16	TC	14	6
36940	3	17657276	17657315	2	20	40	AC	10	6
36949	3	17665012	17665023	2	6	12	CT	22	6
36950	3	17665025	17665036	2	6	12	TC	26	5
36951	3	17665093	17665114	2	11	22	GA	27	7
36952	3	17666742	17666765	2	12	24	CA	11	11
36953	3	17668403	17668418	2	8	16	AC	29	5
36957	3	17687772	17687811	2	20	40	CA	6	10
36964	3	17705424	17705441	2	9	18	AT	11	3
36968	3	17712838	17712852	3	5	15	CAG	9	4
36969	3	17713159	17713173	3	5	15	GCG	25	6
36971	3	17714018	17714033	2	8	16	AC	10	3

36980	3	17722737	17722762	2	13	26	CA	22	8
36981	3	17723652	17723671	2	10	20	AC	8	7
36983	3	17729627	17729652	2	13	26	AC	7	3
36985	3	17730593	17730610	2	9	18	CT	21	4
36986	3	17731883	17731912	2	15	30	CA	17	7
36989	3	17738511	17738530	2	10	20	GT	7	8
36995	3	17744279	17744306	2	14	28	GT	14	5
36996	3	17744859	17744902	2	22	44	AG	11	12
37004	3	17767319	17767352	2	17	34	TG	8	7
37022	3	17816781	17816795	3	5	15	TTA	14	10
37024	3	17817487	17817526	2	20	40	CT	6	5
37025	3	17817590	17817611	2	11	22	TC	7	8
37028	3	17818672	17818699	4	7	28	TAAC	13	8
37029	3	17819817	17819831	3	5	15	CCT	13	6
37031	3	17821598	17821615	2	9	18	GT	18	15
37034	3	17830813	17830852	4	10	40	AAAG	18	14
37044	3	17852708	17852725	3	6	18	ATA	24	4
37045	3	17858848	17858863	4	4	16	CCAA	28	3
37047	3	17863237	17863248	2	6	12	CA	21	6
37049	3	17865968	17866005	2	19	38	TG	14	12
37050	3	17868625	17868638	2	7	14	AC	8	4
37051	3	17872178	17872195	2	9	18	AG	21	5
37054	3	17879722	17879743	2	11	22	GT	18	8
37055	3	17880836	17880877	2	21	42	TG	15	10
37056	3	17882178	17882201	4	6	24	TATG	19	3
37060	3	17884600	17884615	2	8	16	TG	21	9
37061	3	17885539	17885554	2	8	16	GT	13	9
37062	3	17890909	17890930	2	11	22	GT	21	5
37065	3	17892333	17892360	2	14	28	AG	14	11
37067	3	17894925	17894944	2	10	20	AC	17	9
37074	3	17908087	17908106	2	10	20	CA	20	9
37095	3	17964119	17964132	2	7	14	GT	22	6
37097	3	17965414	17965461	2	24	48	CA	9	11
37103	3	17990011	17990025	3	5	15	TCC	24	5
37110	3	18004911	18004930	2	10	20	TG	21	10
37111	3	18005664	18005679	4	4	16	GCTA	29	3
37114	3	18012198	18012243	2	23	46	GT	13	14
37115	3	18012413	18012428	2	8	16	AC	20	5
37119	3	18017728	18017761	2	17	34	CA	9	11
37125	3	18024400	18024441	2	21	42	GT	12	11
37128	3	18027664	18027675	2	6	12	CA	24	3
37138	3	18048689	18048704	2	8	16	CA	20	10
37141	3	18055030	18055067	2	19	38	TG	8	10
37143	3	18056957	18056968	2	6	12	AC	23	3
37156	3	18079402	18079445	2	22	44	AC	7	9
37170	3	18125736	18125751	4	4	16	CCTC	10	3
37178	3	18145433	18145447	3	5	15	TCC	9	4
37182	3	18151716	18151736	3	7	21	GGA	21	3
37183	3	18153236	18153259	2	12	24	AC	17	9
37184	3	18153436	18153457	2	11	22	TG	13	9
37186	3	18156890	18156905	2	8	16	AC	30	3

37193	3	18169116	18169131	2	8	16	TG	20	3
37194	3	18169564	18169584	3	7	21	CTG	29	8
37198	3	18181099	18181118	2	10	20	CT	25	3
37199	3	18182882	18182917	2	18	36	GT	7	10
37201	3	18183099	18183130	2	16	32	TG	8	7
37205	3	18191112	18191126	3	5	15	CTT	18	5
37209	3	18192976	18193009	2	17	34	AC	16	9
37214	3	18198462	18198481	2	10	20	CA	13	10
37223	3	18221812	18221841	2	15	30	CT	15	9
37231	3	18250025	18250072	3	16	48	CTT	18	16
37239	3	18285296	18285323	2	14	28	CT	12	7
37240	3	18286002	18286021	2	10	20	TG	19	9
37243	3	18320049	18320064	4	4	16	TTAC	14	8
37248	3	18332929	18332940	2	6	12	TG	27	3
37252	3	18339616	18339630	3	5	15	CTG	26	3
37254	3	18343427	18343450	2	12	24	CT	20	5
37255	3	18344521	18344540	2	10	20	TC	18	8
37256	3	18344834	18344851	2	9	18	GA	20	9
37261	3	18362194	18362208	3	5	15	AGC	23	6
37262	3	18362369	18362383	3	5	15	AAC	30	5
37267	3	18370011	18370030	2	10	20	CT	23	3
37270	3	18373630	18373644	3	5	15	CAG	27	7
37271	3	18381699	18381714	4	4	16	TATT	17	3
37276	3	18385011	18385025	3	5	15	CAA	25	4
37277	3	18385495	18385510	2	8	16	CA	24	7
37278	3	18387657	18387678	2	11	22	CT	24	5
37280	3	18390732	18390753	2	11	22	CA	15	11
37281	3	18392990	18393011	2	11	22	CA	23	5
37287	3	18431364	18431381	3	6	18	CAA	19	4
37292	3	18447729	18447749	3	7	21	TGC	17	6
37296	3	18461662	18461689	4	7	28	AATC	16	5
37313	3	18527897	18527944	4	12	48	ATCT	13	11
37322	3	18537311	18537328	3	6	18	ATC	23	3
37324	3	18547598	18547612	3	5	15	TGC	30	3
37329	3	18555116	18555131	4	4	16	TTTA	20	6
37330	3	18556555	18556584	2	15	30	GT	16	12
37333	3	18561686	18561699	2	7	14	TC	26	5
37342	3	18593443	18593470	2	14	28	CA	13	12
37343	3	18594127	18594154	2	14	28	CA	7	8
37345	3	18601518	18601532	3	5	15	AGG	13	3
37360	3	18626572	18626585	2	7	14	AC	25	4
37361	3	18629592	18629619	2	14	28	GT	19	8
37362	3	18629704	18629715	2	6	12	AC	31	3
37370	3	18649890	18649907	2	9	18	CT	15	3
37375	3	18662905	18662922	2	9	18	TA	16	4
37376	3	18664966	18664997	4	8	32	GTCT	22	16
37378	3	18669156	18669173	2	9	18	CT	17	7
37379	3	18669174	18669185	2	6	12	CA	13	8
37382	3	18670569	18670604	2	18	36	TG	17	12
37387	3	18673400	18673415	4	4	16	AAAC	27	3
37392	3	18686757	18686772	2	8	16	AC	20	9

37401	3	18703834	18703851	2	9	18	TG	16	10
37413	3	18728170	18728189	2	10	20	CA	20	10
37422	3	18741606	18741617	2	6	12	AC	25	3
37424	3	18742445	18742468	4	6	24	AGAC	30	4
37426	3	18747365	18747384	2	10	20	GA	24	8
37434	3	18762740	18762759	2	10	20	AG	8	9
37436	3	18763549	18763566	2	9	18	AT	15	6
37437	3	18765303	18765326	2	12	24	AC	17	7
37445	3	18768479	18768494	4	4	16	TTTC	18	3
37452	3	18774540	18774557	2	9	18	TG	30	7
37454	3	18775428	18775442	3	5	15	CTT	17	4
37458	3	18776765	18776782	2	9	18	AC	16	14
37460	3	18780479	18780492	2	7	14	CT	13	9
37463	3	18782728	18782755	2	14	28	GT	20	9
37465	3	18783368	18783394	3	9	27	AGA	20	8
37468	3	18796520	18796541	2	11	22	TC	15	5
37470	3	18803080	18803097	2	9	18	GT	21	3
37473	3	18805365	18805380	2	8	16	GT	26	6
37474	3	18805487	18805506	2	10	20	GA	23	4
37476	3	18807596	18807653	2	29	58	CA	6	8
37477	3	18808042	18808063	2	11	22	AC	14	16
37480	3	18808842	18808855	2	7	14	GT	9	5
37483	3	18810786	18810799	2	7	14	TG	23	6
37485	3	18814571	18814588	2	9	18	AC	23	8
37490	3	18823619	18823639	3	7	21	CTC	21	8
37493	3	18827611	18827624	2	7	14	TC	23	10
37496	3	18847526	18847545	2	10	20	AC	8	10
37502	3	18854306	18854317	2	6	12	TC	25	4
37503	3	18855105	18855119	3	5	15	CTG	24	4
37506	3	18857047	18857060	2	7	14	GT	26	4
37511	3	18862317	18862328	2	6	12	TG	15	6
37513	3	18862555	18862574	2	10	20	AG	15	5
37515	3	18862729	18862740	2	6	12	TC	19	7
37516	3	18868742	18868755	2	7	14	GA	27	6
37521	3	18872166	18872177	2	6	12	AC	29	3
37522	3	18875371	18875384	2	7	14	TG	26	3
37537	3	18908229	18908252	2	12	24	GT	21	11
37543	3	18932644	18932667	2	12	24	TG	16	11
37544	3	18933938	18933955	2	9	18	AC	20	4
37549	3	18943399	18943416	3	6	18	CAG	31	5
37550	3	18944261	18944278	3	6	18	AGG	30	3
37563	3	18968489	18968516	4	7	28	GTTT	26	4
37568	3	18978249	18978300	2	26	52	AC	12	14
37572	3	19007215	19007229	3	5	15	GAA	18	3
37573	3	19013815	19013840	2	13	26	TG	18	9
37578	3	19025706	19025743	2	19	38	GT	14	9
37599	3	19074504	19074517	2	7	14	TC	27	4
37604	3	19078338	19078353	2	8	16	TG	22	6
37606	3	19082851	19082864	2	7	14	GT	21	5
37611	3	19101822	19101837	4	4	16	TTTG	25	3
37612	3	19102109	19102123	3	5	15	TTA	11	3

37614	3	19106645	19106662	3	6	18	TCT	12	10
37616	3	19114875	19114886	2	6	12	AC	23	7
37618	3	19119698	19119713	2	8	16	AC	13	15
37621	3	19129466	19129480	3	5	15	GTG	24	5
37624	3	19137983	19137998	4	4	16	AAAT	27	8
37634	3	19154835	19154848	2	7	14	TG	21	4
37637	3	19169396	19169411	4	4	16	ATTC	30	3
37639	3	19187748	19187761	2	7	14	TG	23	3
37646	3	19206114	19206125	2	6	12	AC	26	3
37658	3	19228369	19228386	2	9	18	GT	24	9
37660	3	19232533	19232550	2	9	18	TG	16	9
37667	3	19245895	19245910	4	4	16	TTCA	35	3
37668	3	19248145	19248176	2	16	32	AC	10	10
37677	3	19269025	19269050	2	13	26	TG	16	9
37680	3	19271127	19271138	2	6	12	TG	11	4
37681	3	19271140	19271155	2	8	16	GT	12	4
37683	3	19273222	19273237	2	8	16	AC	18	6
37691	3	19280931	19280942	2	6	12	CT	22	5
37696	3	19284209	19284226	2	9	18	GT	12	15
37698	3	19284519	19284536	2	9	18	AC	9	3
37699	3	19284754	19284769	2	8	16	CA	20	7
37700	3	19284942	19284973	2	16	32	AC	6	8
37701	3	19285116	19285129	2	7	14	AC	16	10
37708	3	19287994	19288007	2	7	14	CT	7	4
37715	3	19303047	19303103	3	19	57	TTC	17	12
37720	3	19311818	19311831	2	7	14	GT	18	7
37721	3	19312018	19312031	2	7	14	TG	27	8
37724	3	19315011	19315034	3	8	24	CTC	25	5
37725	3	19315592	19315609	3	6	18	GGA	14	3
37727	3	19316043	19316058	4	4	16	CGCT	21	6
37731	3	19317838	19317863	2	13	26	TG	20	8
37735	3	19321072	19321111	2	20	40	AC	10	8
37736	3	19321243	19321256	2	7	14	AC	9	3
37739	3	19321286	19321315	2	15	30	CA	8	6
37747	3	19324417	19324442	2	13	26	AC	11	7
37750	3	19326426	19326453	2	14	28	GA	23	11
37752	3	19327117	19327131	3	5	15	GAG	6	4
37756	3	19329453	19329466	2	7	14	CT	6	3
37757	3	19330087	19330102	4	4	16	ATCT	15	6
37759	3	19331673	19331696	3	8	24	CTG	19	10
37760	3	19332420	19332439	2	10	20	GT	13	10
37762	3	19334167	19334198	2	16	32	AC	22	7
37763	3	19334749	19334770	2	11	22	TG	9	6
37765	3	19335236	19335268	3	11	33	CCT	14	10
37768	3	19337668	19337681	2	7	14	CT	32	9
37771	3	19340139	19340165	3	9	27	TCC	14	8
37776	3	19349879	19349906	4	7	28	GAAA	19	11
37778	3	19352573	19352612	4	10	40	GATA	17	9
37779	3	19353427	19353442	2	8	16	CT	27	4
37780	3	19353573	19353586	2	7	14	AC	22	12
37781	3	19354104	19354127	2	12	24	AC	19	13

37785	3	19356802	19356831	2	15	30	CA	12	13
37786	3	19357246	19357257	2	6	12	AT	16	3
37788	3	19357504	19357531	2	14	28	GT	10	8
37794	3	19383892	19383903	2	6	12	CA	30	4
37796	3	19387660	19387675	2	8	16	TG	24	5
37798	3	19399050	19399065	2	8	16	AC	29	3
37799	3	19400604	19400635	2	16	32	TG	24	14
37801	3	19405078	19405093	4	4	16	AATT	16	3
37804	3	19408384	19408409	2	13	26	GT	9	6
37809	3	19417733	19417758	2	13	26	AC	19	10
37812	3	19419833	19419884	2	26	52	AC	9	11
37822	3	19436252	19436263	2	6	12	TG	23	3
37823	3	19439701	19439720	2	10	20	CA	12	13
37825	3	19442657	19442671	3	5	15	CGC	21	5
37831	3	19450600	19450633	2	17	34	AC	23	13
37833	3	19453821	19453835	3	5	15	GAT	11	5
37834	3	19453839	19453853	3	5	15	GAT	8	3
37835	3	19456780	19456795	2	8	16	GA	19	5
37839	3	19459316	19459333	2	9	18	CA	21	18
37840	3	19460025	19460036	2	6	12	CA	23	3
37841	3	19461776	19461789	2	7	14	AC	8	3
37842	3	19461836	19461855	2	10	20	AC	6	6
37843	3	19462066	19462105	2	20	40	AC	13	11
37852	3	19480824	19480839	4	4	16	TCCA	23	3
37860	3	19490918	19490933	2	8	16	AC	17	8
37861	3	19491030	19491061	2	16	32	TG	14	12
37862	3	19491450	19491475	2	13	26	TG	13	11
37863	3	19491907	19491932	2	13	26	GT	13	14
37865	3	19496079	19496093	3	5	15	CAC	23	3
37866	3	19496346	19496365	2	10	20	AC	20	10
37867	3	19497601	19497638	2	19	38	GT	10	15
37868	3	19497812	19497837	2	13	26	AC	21	7
37869	3	19505778	19505793	2	8	16	TC	27	3
37870	3	19510085	19510116	2	16	32	CA	13	10
37875	3	19528066	19528083	2	9	18	TG	14	5
37876	3	19528728	19528743	4	4	16	TTTC	23	4
37880	3	19534319	19534330	2	6	12	TG	20	3
37881	3	19534758	19534785	4	7	28	TTTC	15	6
37882	3	19535198	19535217	2	10	20	AG	13	5
37883	3	19535449	19535466	3	6	18	TGC	32	3
37885	3	19536072	19536085	2	7	14	TG	17	7
37886	3	19536205	19536218	2	7	14	AT	13	4
37889	3	19537706	19537723	3	6	18	AAG	13	4
37893	3	19549222	19549239	2	9	18	CA	23	11
37894	3	19552097	19552112	2	8	16	CT	25	4
37898	3	19563222	19563239	3	6	18	TTA	23	4
37908	3	19598042	19598059	2	9	18	TG	15	5
37909	3	19600331	19600364	2	17	34	AC	15	8
37910	3	19600753	19600766	2	7	14	TG	25	3
37911	3	19604071	19604086	2	8	16	TG	21	5
37913	3	19605939	19605954	2	8	16	GA	24	7

37915	3	19606879	19606894	2	8	16	TG	26	3
37918	3	19613462	19613476	3	5	15	GCA	25	4
37923	3	19618019	19618042	3	8	24	CCT	22	6
37931	3	19622821	19622853	3	11	33	CCT	18	9
37932	3	19622928	19622953	2	13	26	GT	12	7
37935	3	19624231	19624254	3	8	24	CCT	18	4
37936	3	19628310	19628323	2	7	14	CA	21	7
37937	3	19629335	19629366	4	8	32	AACA	25	9
37941	3	19638474	19638493	2	10	20	GT	20	7
37949	3	19656405	19656416	2	6	12	CT	24	4
37950	3	19660240	19660255	4	4	16	TAGT	32	4
37954	3	19679669	19679680	2	6	12	TA	23	4
37958	3	19695188	19695251	4	16	64	ATAG	11	8
37962	3	19699671	19699684	2	7	14	TG	27	3
37969	3	19722385	19722396	2	6	12	TG	19	4
37976	3	19736024	19736039	2	8	16	CA	19	3
37978	3	19737256	19737270	3	5	15	GGT	11	3
37982	3	19739187	19739210	2	12	24	TG	31	11
37985	3	19740566	19740581	4	4	16	TTCT	19	5
37989	3	19742836	19742851	2	8	16	GT	18	4
37991	3	19744949	19744972	2	12	24	CA	10	7
37999	3	19752537	19752556	2	10	20	AG	17	7
38003	3	19762969	19762986	3	6	18	CCA	14	10
38013	3	19773000	19773017	3	6	18	TCC	19	12
38014	3	19773266	19773304	3	13	39	CAT	13	9
38018	3	19781177	19781194	2	9	18	CA	25	3
38019	3	19781287	19781301	3	5	15	GAA	11	4
38021	3	19786537	19786568	2	16	32	GT	14	11
38022	3	19792597	19792610	2	7	14	AC	17	12
38023	3	19793883	19793896	2	7	14	AT	25	5
38026	3	19804611	19804643	3	11	33	CTC	7	4
38028	3	19805386	19805403	3	6	18	CCT	24	9
38034	3	19809547	19809560	2	7	14	AG	21	3
38035	3	19812624	19812635	2	6	12	CA	27	5
38037	3	19815606	19815631	2	13	26	TG	15	12
38039	3	19816646	19816667	2	11	22	GT	10	9
38041	3	19819446	19819466	3	7	21	GCT	18	6
38043	3	19821810	19821827	2	9	18	AG	27	7
38045	3	19822515	19822530	2	8	16	TG	18	6
38047	3	19824951	19824965	3	5	15	CTG	26	3
38048	3	19827472	19827487	4	4	16	CATC	28	7
38060	3	19843784	19843799	2	8	16	AC	21	17
38063	3	19849777	19849796	4	5	20	ATGT	17	7
38065	3	19853923	19853934	2	6	12	AC	19	7
38067	3	19856221	19856236	2	8	16	CA	25	7
38068	3	19859224	19859245	2	11	22	TG	19	15
38069	3	19859745	19859764	2	10	20	TG	24	8
38072	3	19860916	19860933	2	9	18	GT	14	7
38073	3	19860935	19860954	2	10	20	TG	24	13
38075	3	19862380	19862391	2	6	12	GT	21	3
38085	3	19878801	19878826	2	13	26	CT	24	7

38088	3	19882494	19882519	2	13	26	GT	21	6
38102	3	19889820	19889831	2	6	12	AC	33	3
38106	3	19905190	19905213	2	12	24	TG	14	6
38107	3	19906098	19906129	4	8	32	CCTT	28	13
38112	3	19909322	19909339	3	6	18	TCA	23	12
38114	3	19911719	19911750	2	16	32	AC	9	12
38116	3	19913305	19913370	3	22	66	TCT	12	14
38117	3	19913682	19913705	3	8	24	CCT	7	5
38118	3	19913942	19913971	2	15	30	TG	14	10
38119	3	19914448	19914463	2	8	16	CT	22	10
38130	3	19925289	19925303	3	5	15	TAT	26	5
38133	3	19934499	19934512	2	7	14	TA	26	6
38135	3	19937533	19937547	3	5	15	CCA	21	3
38136	3	19937548	19937562	3	5	15	CCT	17	4
38137	3	19937809	19937826	3	6	18	TGC	19	11
38138	3	19937943	19937956	2	7	14	TG	20	10
38139	3	19937958	19937969	2	6	12	GT	21	8
38140	3	19938146	19938173	2	14	28	TG	11	9
38147	3	19939941	19939954	2	7	14	AT	21	5
38148	3	19940818	19940829	2	6	12	GA	11	4
38149	3	19943897	19943922	2	13	26	TG	13	5
38151	3	19944599	19944622	4	6	24	TGAA	14	5
38154	3	19945039	19945068	2	15	30	GT	8	7
38162	3	19952017	19952044	2	14	28	GT	21	16
38163	3	19952249	19952272	3	8	24	GGA	22	5
38166	3	19962216	19962233	2	9	18	AG	23	4
38171	3	19969828	19969861	2	17	34	TG	15	11
38172	3	19970082	19970103	2	11	22	TC	21	3
38173	3	19970222	19970235	2	7	14	TC	24	4
38175	3	19974628	19974645	2	9	18	CT	26	7
38188	3	19992138	19992171	2	17	34	GT	8	7
38195	3	20002754	20002768	3	5	15	TCA	26	3
38196	3	20003910	20003943	2	17	34	GT	7	11
38197	3	20004002	20004015	2	7	14	AC	25	3
38203	3	20020378	20020393	2	8	16	GT	22	6
38204	3	20022568	20022587	2	10	20	AC	21	3
38205	3	20024098	20024115	2	9	18	AC	20	7
38208	3	20032065	20032094	3	10	30	GCT	17	10
38210	3	20036495	20036510	2	8	16	GT	14	9
38212	3	20038661	20038676	2	8	16	TG	21	4
38215	3	20042080	20042094	3	5	15	ACC	22	4
38216	3	20042291	20042302	2	6	12	AT	15	5
38219	3	20046933	20046950	3	6	18	TCA	30	3
38221	3	20052098	20052115	2	9	18	GT	24	5
38223	3	20053701	20053718	2	9	18	TG	8	7
38224	3	20053720	20053739	2	10	20	TG	8	7
38241	3	20083768	20083785	3	6	18	CTT	22	14
38249	3	20092706	20092717	2	6	12	TC	33	3
38252	3	20097703	20097742	2	20	40	GT	11	11
38254	3	20106661	20106684	2	12	24	GA	19	11
38255	3	20106768	20106783	4	4	16	AAAG	14	5

38260	3	20119314	20119329	4	4	16	AGCT	25	4
38261	3	20123153	20123170	3	6	18	TTA	24	6
38266	3	20134757	20134783	3	9	27	CAT	19	4
38267	3	20135251	20135265	3	5	15	AAG	17	7
38286	3	20164250	20164271	2	11	22	AC	21	11
38300	3	20174255	20174287	3	11	33	AGA	19	10
38302	3	20174637	20174654	3	6	18	GAA	19	7
38304	3	20175383	20175400	2	9	18	GA	23	5
38305	3	20176017	20176046	2	15	30	CA	14	10
38306	3	20176247	20176264	2	9	18	CA	18	8
38309	3	20179832	20179855	2	12	24	AC	22	11
38310	3	20179900	20179913	2	7	14	GT	23	5
38314	3	20183264	20183275	2	6	12	AC	30	4
38320	3	20192626	20192647	2	11	22	CA	10	7
38326	3	20197485	20197520	2	18	36	TG	10	10
38327	3	20198129	20198150	2	11	22	GT	15	15
38332	3	20202545	20202556	2	6	12	CT	32	4
38336	3	20207750	20207767	2	9	18	AG	19	7
38338	3	20211930	20211947	2	9	18	TG	19	9
38345	3	20225644	20225671	2	14	28	AC	17	10
38347	3	20230421	20230438	2	9	18	AG	21	6
38348	3	20231959	20231984	2	13	26	CA	17	9
38350	3	20236217	20236230	2	7	14	AC	18	5
38354	3	20244372	20244403	2	16	32	CA	13	8
38355	3	20246494	20246509	2	8	16	AC	30	5
38360	3	20254302	20254331	2	15	30	TG	13	7
38364	3	20257556	20257569	2	7	14	TG	18	3
38365	3	20259147	20259162	2	8	16	GT	27	3
38370	3	20266291	20266302	2	6	12	CA	20	4
38371	3	20269138	20269167	2	15	30	CA	15	12
38372	3	20269423	20269434	2	6	12	TG	8	5
38373	3	20269437	20269452	2	8	16	TG	20	9
38376	3	20274087	20274110	2	12	24	AC	16	15
38379	3	20274552	20274589	2	19	38	AC	9	15
38384	3	20286501	20286524	2	12	24	AC	13	10
38387	3	20293143	20293162	2	10	20	CA	14	9
38388	3	20298878	20298893	2	8	16	AC	25	4
38389	3	20299584	20299599	2	8	16	TG	7	5
38390	3	20299613	20299626	2	7	14	GT	7	6
38392	3	20299854	20299883	2	15	30	TG	13	7
38393	3	20300115	20300132	2	9	18	GT	19	4
38397	3	20305929	20305946	2	9	18	AC	17	8
38400	3	20308962	20309013	4	13	52	TATC	14	7
38401	3	20311634	20311659	2	13	26	TG	31	9
38406	3	20316408	20316419	2	6	12	CA	21	4
38409	3	20321327	20321340	2	7	14	TA	9	9
38410	3	20321341	20321360	2	10	20	CA	9	11
38411	3	20328778	20328791	2	7	14	AC	29	8
38414	3	20331295	20331310	4	4	16	AAAT	30	4
38415	3	20337619	20337630	2	6	12	CA	28	9
38418	3	20344242	20344281	2	20	40	TC	21	9

38437	3	20373133	20373174	2	21	42	AC	16	12
38438	3	20373733	20373747	3	5	15	GCT	23	8
38442	3	20382205	20382226	2	11	22	GT	23	12
38446	3	20392766	20392783	2	9	18	TG	21	6
38448	3	20396616	20396633	3	6	18	CAA	22	4
38449	3	20397057	20397072	2	8	16	GT	17	4
38456	3	20402455	20402470	2	8	16	AC	29	4
38459	3	20402755	20402782	2	14	28	AC	11	9
38461	3	20409038	20409065	2	14	28	GT	18	9
38462	3	20409417	20409448	2	16	32	CA	10	9
38466	3	20414341	20414368	2	14	28	AC	6	9
38477	3	20419356	20419373	3	6	18	GAG	27	4
38480	3	20428768	20428783	2	8	16	AC	23	7
38482	3	20431027	20431038	2	6	12	GT	10	4
38488	3	20438940	20438953	2	7	14	AG	24	5
38489	3	20439279	20439294	2	8	16	AC	18	11
38503	3	20452235	20452256	2	11	22	AG	14	5
38506	3	20459385	20459412	4	7	28	GCTG	24	5
38515	3	20476411	20476430	2	10	20	TA	10	13
38520	3	20483557	20483586	2	15	30	GT	19	10
38526	3	20487411	20487436	2	13	26	GT	20	10
38531	3	20493131	20493168	2	19	38	GT	12	10
38537	3	20498175	20498188	2	7	14	CT	23	4
38539	3	20498563	20498582	2	10	20	GT	21	6
38540	3	20498785	20498806	2	11	22	AC	20	5
38542	3	20501127	20501154	2	14	28	AG	20	7
38546	3	20507981	20508000	2	10	20	AC	21	5
38548	3	20510187	20510200	2	7	14	AC	14	5
38550	3	20512509	20512532	3	8	24	CAT	23	5
38551	3	20512836	20512847	2	6	12	CA	19	5
38556	3	20521983	20522004	2	11	22	AC	13	10
38557	3	20522037	20522054	2	9	18	AC	13	8
38558	3	20523065	20523080	4	4	16	ATAC	14	3
38560	3	20524381	20524394	2	7	14	AC	10	12
38561	3	20524396	20524409	2	7	14	CA	12	12
38562	3	20525125	20525142	2	9	18	CA	9	10
38563	3	20526738	20526755	2	9	18	TG	19	11
38568	3	20531193	20531214	2	11	22	CA	16	11
38569	3	20531372	20531386	3	5	15	CTT	33	9
38570	3	20531388	20531405	3	6	18	TTC	26	6
38571	3	20531836	20531847	2	6	12	CA	25	5
38572	3	20532643	20532680	2	19	38	TG	8	10
38576	3	20537185	20537199	3	5	15	ATT	8	7
38577	3	20537504	20537530	3	9	27	TCC	18	6
38584	3	20549977	20549992	2	8	16	TG	16	12
38585	3	20550212	20550247	2	18	36	GT	9	13
38588	3	20550702	20550719	3	6	18	CTG	29	3
38594	3	20553847	20553866	2	10	20	TG	13	11
38604	3	20566343	20566357	3	5	15	ACA	16	9
38608	3	20574953	20574968	2	8	16	TG	15	5
38611	3	20578630	20578649	2	10	20	CA	12	12

38616	3	20582376	20582399	2	12	24	CA	15	15
38618	3	20584822	20584851	2	15	30	GT	15	11
38624	3	20596737	20596750	2	7	14	AG	18	7
38639	3	20636083	20636100	3	6	18	GGT	23	6
38640	3	20636841	20636855	3	5	15	ACA	26	5
38641	3	20637484	20637499	4	4	16	GAGC	23	5
38642	3	20637581	20637618	2	19	38	GT	8	10
38646	3	20643913	20643942	2	15	30	GT	19	11
38657	3	20673692	20673705	2	7	14	GT	23	3
38658	3	20674385	20674398	2	7	14	GT	18	10
38666	3	20682860	20682889	2	15	30	TG	16	9
38668	3	20686262	20686277	4	4	16	TTGG	26	3
38670	3	20690457	20690468	2	6	12	TG	7	6
38674	3	20692808	20692828	3	7	21	GCT	26	5
38675	3	20698541	20698564	2	12	24	TG	21	7
38676	3	20700274	20700309	4	9	36	GATG	29	12
38677	3	20702241	20702268	2	14	28	CA	13	8
38678	3	20708164	20708199	2	18	36	TC	6	8
38681	3	20719588	20719619	2	16	32	CA	14	6
38683	3	20722573	20722587	3	5	15	CCG	27	5
38684	3	20723847	20723860	2	7	14	TC	19	4
38695	3	20741537	20741566	3	10	30	TCC	8	9
38697	3	20746167	20746188	2	11	22	TG	10	6
38699	3	20749487	20749500	2	7	14	TG	7	4
38706	3	20758705	20758720	2	8	16	AT	15	10
38707	3	20758721	20758736	2	8	16	GT	17	8
38728	3	20780018	20780029	2	6	12	GT	20	4
38731	3	20782118	20782133	2	8	16	TG	19	5
38733	3	20786820	20786835	2	8	16	CA	28	6
38771	3	20827484	20827499	2	8	16	TG	16	4
38772	3	20827500	20827515	4	4	16	AGTG	18	5
38773	3	20827670	20827693	2	12	24	CA	19	9
38774	3	20829402	20829415	2	7	14	AC	14	5
38775	3	20830138	20830161	2	12	24	AC	19	7
38776	3	20835501	20835514	2	7	14	TG	26	3
38782	3	20838780	20838791	2	6	12	TC	27	4
38787	3	20842129	20842148	4	5	20	CCTT	29	3
38789	3	20845408	20845421	2	7	14	AC	25	5
38794	3	20848913	20848930	2	9	18	AC	21	5
38795	3	20849078	20849093	4	4	16	TTTA	14	9
38796	3	20849398	20849413	2	8	16	GT	25	3
38803	3	20863473	20863492	2	10	20	GT	21	8
38813	3	20890184	20890204	3	7	21	CTC	28	5
38815	3	20892391	20892402	2	6	12	TG	25	3
38816	3	20895103	20895122	2	10	20	TG	14	16
38817	3	20895226	20895249	2	12	24	CA	16	12
38818	3	20898493	20898508	4	4	16	TATT	11	10
38819	3	20901407	20901430	2	12	24	CT	26	8
38822	3	20906010	20906035	2	13	26	CA	16	5
38826	3	20917006	20917020	3	5	15	GAG	27	3
38827	3	20920083	20920102	2	10	20	GT	14	8

38829	3	20920846	20920867	2	11	22	TG	12	7
38830	3	20922300	20922345	2	23	46	GT	11	12
38831	3	20925785	20925802	3	6	18	TCT	23	5
38834	3	20932832	20932847	2	8	16	CA	19	9
38835	3	20935373	20935416	2	22	44	CA	18	13
38841	3	20950235	20950256	2	11	22	AC	15	6
38844	3	20952961	20952978	2	9	18	TC	9	5
38845	3	20953050	20953065	4	4	16	CCCT	21	3
38846	3	20953220	20953246	3	9	27	AGC	25	4
38849	3	20955240	20955267	2	14	28	AC	13	9
38850	3	20955504	20955517	2	7	14	AC	14	4
38851	3	20958014	20958025	2	6	12	GT	22	5
38853	3	20963751	20963764	2	7	14	AC	26	4
38854	3	20969637	20969648	2	6	12	TC	28	9
38855	3	20969650	20969665	2	8	16	CT	26	9
38859	3	20977226	20977243	2	9	18	GT	19	4
38863	3	20982611	20982625	3	5	15	TTA	13	3
38864	3	20983497	20983522	2	13	26	GT	16	8
38866	3	20989041	20989052	2	6	12	AC	15	4
38867	3	20989061	20989074	2	7	14	AT	16	6
38869	3	20989844	20989855	2	6	12	CA	13	3
38871	3	20991197	20991214	2	9	18	AC	23	11
38875	3	20993276	20993290	3	5	15	CAA	20	3
38879	3	21000935	21000974	2	20	40	AC	6	8
38882	3	21002036	21002051	4	4	16	AACC	36	4
38883	3	21003554	21003581	2	14	28	GT	11	7
38884	3	21004494	21004505	2	6	12	CT	23	3
38885	3	21007788	21007805	3	6	18	CCT	29	6
38888	3	21038109	21038150	2	21	42	AC	10	7
38910	4	46922	46939	3	6	18	GGA	22	5
38911	4	47094	47108	3	5	15	GCA	17	4
38913	4	53928	53939	2	6	12	GA	28	4
38919	4	70957	70972	2	8	16	TG	17	7
38925	4	72725	72738	2	7	14	AC	19	3
38926	4	75468	75488	3	7	21	TTC	22	7
38928	4	77198	77215	3	6	18	TTG	23	4
38931	4	81764	81777	2	7	14	CA	26	9
38932	4	83179	83190	2	6	12	GT	15	9
38933	4	83405	83422	2	9	18	AC	16	9
38935	4	83721	83735	3	5	15	CTG	18	4
38936	4	85275	85310	3	12	36	GAA	18	7
38937	4	85578	85591	2	7	14	TG	24	3
38938	4	85674	85697	2	12	24	AC	17	5
38940	4	87105	87128	3	8	24	GCA	31	6
38941	4	88317	88336	2	10	20	CA	20	8
38942	4	88408	88429	2	11	22	CA	17	6
38944	4	90375	90398	2	12	24	GT	8	4
38949	4	91588	91605	2	9	18	TG	22	6
38957	4	99286	99301	4	4	16	TTTG	23	10
38967	4	140525	140542	3	6	18	TCA	29	5
38970	4	146197	146211	3	5	15	TTC	14	4

38980	4	174314	174327	2	7	14	AC	25	3
38984	4	183902	183916	3	5	15	TCC	27	3
38989	4	209751	209792	2	21	42	GT	13	10
39006	4	243534	243547	2	7	14	CA	16	3
39007	4	244608	244619	2	6	12	CA	19	4
39008	4	246175	246190	4	4	16	CATC	25	3
39015	4	268179	268194	2	8	16	TG	26	5
39016	4	274701	274714	2	7	14	CA	22	4
39017	4	275983	275997	3	5	15	GAA	17	5
39019	4	288559	288572	2	7	14	AG	20	5
39024	4	295950	295981	2	16	32	GT	19	11
39027	4	305386	305397	2	6	12	CA	24	4
39033	4	311052	311063	2	6	12	GT	26	3
39034	4	311624	311655	2	16	32	AC	26	6
39040	4	316448	316471	2	12	24	CA	16	13
39041	4	316606	316619	2	7	14	CT	27	6
39063	4	362191	362230	2	20	40	CA	11	12
39067	4	366375	366404	2	15	30	CA	15	8
39068	4	368166	368183	3	6	18	GTT	34	4
39093	4	422048	422065	2	9	18	GT	21	9
39094	4	424625	424638	2	7	14	AC	25	3
39101	4	458376	458393	2	9	18	CA	23	4
39108	4	476783	476796	2	7	14	GA	12	4
39118	4	512904	512927	2	12	24	AC	25	14
39119	4	513157	513172	2	8	16	CT	24	5
39134	4	572964	572977	2	7	14	GT	18	5
39140	4	580219	580234	2	8	16	TG	30	9
39156	4	588522	588537	2	8	16	AG	25	4
39160	4	589741	589758	2	9	18	TC	14	6
39168	4	591123	591136	2	7	14	TC	28	3
39170	4	593178	593195	2	9	18	GT	23	5
39171	4	593493	593520	2	14	28	CA	14	6
39175	4	598064	598083	2	10	20	GT	17	6
39176	4	598112	598129	2	9	18	GT	17	5
39180	4	600117	600150	2	17	34	GT	7	8
39184	4	604202	604213	2	6	12	TG	22	4
39185	4	606271	606284	2	7	14	CA	26	8
39186	4	606317	606328	2	6	12	CA	17	4
39192	4	626642	626661	2	10	20	GT	18	7
39193	4	626720	626731	2	6	12	GT	14	10
39195	4	628383	628404	2	11	22	AG	24	4
39199	4	631597	631608	2	6	12	AC	29	4
39200	4	632765	632800	2	18	36	GT	6	7
39204	4	636855	636878	2	12	24	TG	13	7
39205	4	637642	637661	2	10	20	CA	8	3
39228	4	664513	664526	2	7	14	CA	29	3
39230	4	664714	664725	2	6	12	CA	18	7
39231	4	665035	665046	2	6	12	TG	20	4
39238	4	670373	670388	4	4	16	TTTC	15	3
39240	4	674336	674381	2	23	46	CA	7	10
39251	4	683117	683136	2	10	20	TG	19	7

39257	4	687231	687254	2	12	24	GT	15	12
39277	4	723874	723888	3	5	15	TTA	20	3
39291	4	733925	733950	2	13	26	CA	15	9
39303	4	744084	744105	2	11	22	GT	11	7
39306	4	748825	748866	2	21	42	TC	8	7
39309	4	752426	752449	2	12	24	CA	18	9
39315	4	756476	756507	2	16	32	CA	15	10
39317	4	760683	760696	2	7	14	TC	26	7
39319	4	763176	763197	2	11	22	AC	21	6
39324	4	765132	765145	2	7	14	CT	26	3
39326	4	765490	765513	2	12	24	TC	14	4
39327	4	767040	767061	2	11	22	CT	20	9
39333	4	768685	768704	2	10	20	TG	18	6
39334	4	769016	769037	2	11	22	AC	14	5
39335	4	769934	769949	2	8	16	CA	19	5
39336	4	771028	771043	2	8	16	GT	23	3
39337	4	771571	771582	2	6	12	AC	20	7
39338	4	772386	772401	2	8	16	GT	21	5
39340	4	776992	777017	2	13	26	GT	15	9
39346	4	786904	786919	2	8	16	AC	23	4
39353	4	800004	800033	2	15	30	TG	11	10
39357	4	803058	803091	2	17	34	AC	15	9
39359	4	807397	807416	2	10	20	TG	9	8
39363	4	808838	808851	2	7	14	AG	31	4
39365	4	820861	820884	3	8	24	CCT	20	6
39367	4	824816	824849	2	17	34	TG	7	9
39371	4	830217	830234	2	9	18	TC	20	3
39372	4	830400	830421	2	11	22	CA	20	6
39375	4	832490	832519	2	15	30	CA	12	10
39376	4	834763	834778	2	8	16	TC	23	5
39383	4	843685	843700	2	8	16	CA	22	3
39384	4	844941	844952	2	6	12	AC	32	3
39385	4	846754	846769	2	8	16	AC	14	12
39387	4	856756	856777	2	11	22	GT	16	7
39390	4	858393	858422	2	15	30	CA	16	7
39398	4	873264	873297	2	17	34	AC	14	13
39403	4	884845	884890	2	23	46	GT	7	6
39405	4	886749	886770	2	11	22	GT	20	13
39407	4	889477	889490	2	7	14	AG	28	4
39414	4	908725	908748	2	12	24	GT	13	11
39416	4	909360	909381	2	11	22	AT	11	5
39420	4	918038	918077	2	20	40	TG	9	12
39421	4	918281	918294	2	7	14	AC	32	4
39424	4	928359	928376	2	9	18	GT	7	4
39425	4	928826	928839	2	7	14	AT	13	6
39432	4	956017	956032	2	8	16	AC	22	8
39436	4	959782	959793	2	6	12	TG	26	3
39439	4	970879	970894	2	8	16	CA	19	4
39440	4	972342	972357	2	8	16	TG	19	4
39445	4	987586	987607	2	11	22	TG	15	7
39446	4	988418	988437	2	10	20	GT	18	7

39447	4	988867	988878	2	6	12	AC	24	3
39448	4	991436	991455	4	5	20	TAGA	8	3
39462	4	1012940	1012953	2	7	14	AC	23	3
39465	4	1023739	1023758	2	10	20	TG	14	8
39466	4	1024141	1024164	2	12	24	CA	6	6
39468	4	1042025	1042040	2	8	16	TG	9	4
39478	4	1056821	1056836	2	8	16	GT	24	6
39483	4	1065647	1065670	4	6	24	TGGA	13	3
39491	4	1098407	1098436	2	15	30	GT	11	9
39497	4	1108140	1108159	2	10	20	TG	18	9
39498	4	1108314	1108325	2	6	12	GT	15	7
39502	4	1109748	1109762	3	5	15	GCA	19	3
39503	4	1109773	1109787	3	5	15	CCT	15	3
39504	4	1110066	1110083	2	9	18	TG	12	8
39510	4	1113863	1113880	3	6	18	GCT	27	6
39513	4	1114196	1114213	3	6	18	GAG	28	4
39515	4	1116561	1116580	2	10	20	CA	20	6
39516	4	1118526	1118537	2	6	12	AC	22	3
39518	4	1121032	1121043	2	6	12	AT	26	4
39525	4	1127832	1127855	2	12	24	GT	19	12
39528	4	1130080	1130103	2	12	24	GT	11	12
39530	4	1134070	1134087	3	6	18	CCT	9	3
39533	4	1140376	1140396	3	7	21	CCT	26	4
39540	4	1149382	1149409	2	14	28	GT	8	10
39549	4	1153571	1153588	2	9	18	GT	14	11
39551	4	1155182	1155203	2	11	22	TG	17	7
39554	4	1158130	1158141	2	6	12	GT	9	7
39555	4	1160401	1160418	2	9	18	TC	24	5
39557	4	1160832	1160857	2	13	26	GT	12	9
39558	4	1161001	1161032	2	16	32	CA	12	15
39559	4	1162449	1162484	3	12	36	TCC	6	4
39564	4	1165470	1165485	2	8	16	TG	22	6
39566	4	1167767	1167790	2	12	24	CA	11	14
39570	4	1168575	1168590	2	8	16	CA	20	4
39572	4	1169242	1169253	2	6	12	GA	24	3
39584	4	1192247	1192262	4	4	16	AAAT	21	8
39595	4	1229065	1229088	4	6	24	ATGA	28	5
39596	4	1236322	1236339	3	6	18	CCA	19	5
39597	4	1242680	1242693	2	7	14	TG	20	9
39600	4	1244030	1244047	2	9	18	GT	19	6
39601	4	1247402	1247413	2	6	12	GT	22	10
39603	4	1247618	1247638	3	7	21	CCT	21	8
39606	4	1259845	1259860	2	8	16	TC	9	6
39608	4	1261216	1261241	2	13	26	AC	15	12
39612	4	1267473	1267487	3	5	15	CTG	32	3
39613	4	1276486	1276511	2	13	26	GT	15	5
39614	4	1279926	1279971	2	23	46	AC	6	8
39615	4	1280436	1280467	2	16	32	AC	8	9
39619	4	1295192	1295212	3	7	21	TGC	32	4
39621	4	1300405	1300424	2	10	20	CA	16	10
39622	4	1300426	1300441	2	8	16	AC	15	10

39626	4	1306290	1306317	2	14	28	TG	7	7
39634	4	1326301	1326318	2	9	18	AC	22	5
39636	4	1327350	1327371	2	11	22	GT	15	7
39646	4	1347466	1347477	2	6	12	TG	28	5
39647	4	1349195	1349230	2	18	36	AC	18	16
39648	4	1350812	1350847	2	18	36	TG	18	9
39654	4	1368360	1368387	2	14	28	CA	23	20
39655	4	1370606	1370617	2	6	12	AG	26	4
39658	4	1372068	1372097	2	15	30	CA	12	10
39661	4	1376187	1376212	2	13	26	CA	8	8
39664	4	1380038	1380049	2	6	12	AT	13	5
39666	4	1382755	1382796	2	21	42	TG	18	14
39667	4	1392075	1392086	2	6	12	GA	15	3
39680	4	1405473	1405496	3	8	24	AGG	18	7
39683	4	1407261	1407272	2	6	12	AC	29	3
39685	4	1414699	1414758	2	30	60	AC	10	12
39687	4	1418036	1418065	2	15	30	GT	16	7
39689	4	1422592	1422607	4	4	16	TAAA	27	3
39701	4	1455785	1455806	2	11	22	CA	17	5
39703	4	1460435	1460464	3	10	30	GAG	13	4
39708	4	1464580	1464599	4	5	20	AAAT	17	3
39710	4	1464908	1464931	3	8	24	TGA	11	5
39716	4	1466364	1466383	4	5	20	CACG	12	5
39719	4	1466679	1466704	2	13	26	TG	16	10
39722	4	1466875	1466895	3	7	21	ATG	18	14
39725	4	1468327	1468348	2	11	22	AC	15	9
39729	4	1470254	1470283	3	10	30	AAT	12	4
39730	4	1470962	1470981	2	10	20	AC	11	9
39731	4	1471194	1471217	2	12	24	GT	17	10
39733	4	1472156	1472173	3	6	18	ATA	6	3
39739	4	1474817	1474834	2	9	18	AT	19	10
39744	4	1488763	1488780	2	9	18	CA	17	4
39749	4	1493565	1493588	2	12	24	TG	17	4
39753	4	1540015	1540034	2	10	20	AG	19	5
39754	4	1540457	1540482	2	13	26	GT	19	9
39755	4	1540591	1540606	2	8	16	GT	18	11
39756	4	1540744	1540757	2	7	14	TG	22	3
39762	4	1556826	1556839	2	7	14	AC	30	6
39763	4	1560401	1560422	2	11	22	CA	16	6
39766	4	1563059	1563078	2	10	20	AC	19	4
39768	4	1565959	1565982	2	12	24	TG	16	5
39769	4	1573837	1573848	2	6	12	TG	10	4
39770	4	1573850	1573865	2	8	16	GT	9	8
39780	4	1574719	1574736	3	6	18	AGG	12	3
39781	4	1576883	1576906	2	12	24	CA	20	5
39783	4	1577490	1577501	2	6	12	TG	23	5
39786	4	1578293	1578330	2	19	38	GT	14	14
39787	4	1578876	1578889	2	7	14	CA	24	5
39791	4	1584864	1584877	2	7	14	AT	17	7
39792	4	1585854	1585881	4	7	28	TTTG	21	8
39793	4	1587473	1587488	2	8	16	TG	32	10

39794	4	1588072	1588097	2	13	26	GT	11	9
39796	4	1589915	1589926	2	6	12	AC	16	3
39802	4	1593765	1593780	2	8	16	CA	9	4
39805	4	1593960	1593975	2	8	16	GT	11	4
39806	4	1594463	1594480	2	9	18	GT	20	4
39809	4	1596080	1596123	2	22	44	CA	9	10
39811	4	1597080	1597100	3	7	21	TGA	6	5
39830	4	1642383	1642394	2	6	12	TG	21	4
39831	4	1642396	1642413	2	9	18	GT	20	4
39832	4	1642445	1642458	2	7	14	TG	16	4
39834	4	1642792	1642815	2	12	24	AC	11	9
39836	4	1644956	1644971	2	8	16	CT	21	3
39838	4	1645348	1645363	2	8	16	TG	20	3
39842	4	1647214	1647234	3	7	21	TCT	19	5
39844	4	1648545	1648564	2	10	20	CT	8	3
39848	4	1650276	1650293	2	9	18	CA	18	6
39849	4	1650532	1650545	2	7	14	AT	23	5
39850	4	1650546	1650559	2	7	14	AC	27	6
39851	4	1651120	1651153	2	17	34	CA	17	7
39853	4	1651465	1651500	2	18	36	TG	10	8
39854	4	1651621	1651652	2	16	32	AC	6	9
39855	4	1651745	1651762	2	9	18	TG	12	6
39866	4	1652873	1652898	2	13	26	AC	7	8
39868	4	1653019	1653050	2	16	32	AC	15	10
39871	4	1653350	1653367	2	9	18	TG	25	3
39873	4	1653505	1653522	2	9	18	GT	27	7
39874	4	1653666	1653691	2	13	26	AC	17	6
39876	4	1654055	1654074	2	10	20	TC	9	3
39878	4	1657549	1657572	2	12	24	GT	12	9
39880	4	1659720	1659735	4	4	16	TCTT	17	4
39881	4	1659829	1659862	2	17	34	CA	7	7
39882	4	1659983	1659994	2	6	12	CG	27	9
39883	4	1660141	1660180	2	20	40	AC	11	8
39889	4	1665746	1665765	2	10	20	TG	13	7
39891	4	1665901	1665914	2	7	14	TG	21	4
39894	4	1669768	1669785	3	6	18	TCT	22	4
39896	4	1669955	1669978	2	12	24	AC	8	6
39899	4	1672773	1672790	2	9	18	TG	21	4
39900	4	1674641	1674664	2	12	24	AC	7	3
39901	4	1674782	1674795	2	7	14	GT	23	7
39903	4	1676713	1676726	2	7	14	AT	6	3
39908	4	1682070	1682081	2	6	12	GT	31	3
39913	4	1700527	1700553	3	9	27	TGA	20	4
39915	4	1702370	1702405	4	9	36	ATAG	20	8
39918	4	1712147	1712174	2	14	28	AC	14	11
39920	4	1714598	1714625	2	14	28	AC	17	7
39921	4	1714772	1714791	2	10	20	CT	22	6
39923	4	1715429	1715444	2	8	16	GA	17	5
39926	4	1716427	1716470	2	22	44	TG	8	8
39927	4	1716778	1716789	2	6	12	CA	19	4
39928	4	1716791	1716802	2	6	12	AC	17	5

39930	4	1718311	1718338	2	14	28	TG	10	9
39931	4	1718480	1718505	2	13	26	GT	16	6
39934	4	1720021	1720032	2	6	12	GA	21	5
39939	4	1728021	1728038	2	9	18	GT	23	6
39941	4	1732831	1732848	2	9	18	CT	19	3
39942	4	1735555	1735570	4	4	16	TTAT	16	4
39944	4	1740006	1740023	2	9	18	TG	9	12
39945	4	1740025	1740042	2	9	18	GT	6	9
39950	4	1744184	1744198	3	5	15	CCT	20	3
39951	4	1744422	1744465	2	22	44	AG	7	6
39952	4	1746369	1746380	2	6	12	TG	27	3
39955	4	1748725	1748738	2	7	14	GT	19	9
39957	4	1748927	1748946	2	10	20	AC	18	4
39958	4	1749149	1749178	2	15	30	TC	9	6
39961	4	1749796	1749815	4	5	20	GGCT	13	4
39964	4	1750263	1750282	2	10	20	TG	8	3
39966	4	1754834	1754859	2	13	26	AC	18	6
39968	4	1756527	1756546	2	10	20	GT	22	13
39969	4	1756755	1756780	2	13	26	TG	23	10
39971	4	1758428	1758442	3	5	15	GGA	16	4
39972	4	1758794	1758835	2	21	42	GT	6	7
39973	4	1758979	1758996	2	9	18	TG	19	10
39976	4	1760120	1760131	2	6	12	TG	19	3
39981	4	1764223	1764238	2	8	16	AC	18	7
39993	4	1801941	1801964	3	8	24	CTG	28	6
39994	4	1802971	1802996	2	13	26	TG	10	10
39996	4	1806020	1806039	2	10	20	CA	17	4
39997	4	1806281	1806296	2	8	16	GT	27	3
40004	4	1813155	1813174	2	10	20	GT	16	15
40006	4	1815295	1815321	3	9	27	CTG	23	5
40007	4	1815411	1815428	3	6	18	TGT	26	5
40008	4	1815429	1815443	3	5	15	TGA	28	7
40009	4	1817685	1817700	2	8	16	CA	16	3
40010	4	1823837	1823848	2	6	12	TC	24	3
40011	4	1824118	1824135	3	6	18	TCT	25	3
40013	4	1842335	1842362	4	7	28	GTCT	24	3
40018	4	1856549	1856566	3	6	18	TCC	20	10
40020	4	1860030	1860061	2	16	32	TG	20	7
40023	4	1878870	1878899	2	15	30	TG	17	12
40030	4	1888149	1888178	2	15	30	GT	15	9
40034	4	1902898	1902936	3	13	39	GAG	12	6
40038	4	1908960	1908977	2	9	18	GT	25	6
40042	4	1916569	1916600	2	16	32	AC	7	8
40043	4	1917375	1917395	3	7	21	CTC	10	4
40046	4	1920001	1920030	2	15	30	CA	15	15
40049	4	1927573	1927602	2	15	30	AC	15	13
40050	4	1928040	1928069	2	15	30	GT	7	10
40055	4	1933585	1933606	2	11	22	CA	12	6
40057	4	1933735	1933762	2	14	28	GT	10	8
40059	4	1934339	1934365	3	9	27	CCT	25	8
40060	4	1936428	1936447	2	10	20	CA	25	15

40064	4	1945507	1945522	2	8	16	TG	27	4
40065	4	1952859	1952874	2	8	16	CA	23	6
40076	4	1979406	1979425	2	10	20	AG	27	5
40077	4	1982695	1982730	2	18	36	TG	18	16
40078	4	1984778	1984813	3	12	36	GAG	12	7
40082	4	2002408	2002429	2	11	22	CA	21	7
40088	4	2009598	2009621	3	8	24	AAT	7	4
40091	4	2016902	2016913	2	6	12	TG	23	3
40093	4	2019210	2019225	4	4	16	AAGA	17	4
40094	4	2019378	2019395	2	9	18	GA	12	7
40096	4	2019734	2019745	2	6	12	GT	11	3
40101	4	2021077	2021100	2	12	24	GA	16	12
40102	4	2025537	2025548	2	6	12	TG	22	4
40109	4	2035723	2035754	2	16	32	GT	16	8
40111	4	2038287	2038300	2	7	14	GA	25	3
40114	4	2043806	2043821	2	8	16	GA	10	5
40116	4	2049653	2049686	2	17	34	TG	7	12
40119	4	2056879	2056912	2	17	34	TG	7	10
40121	4	2062359	2062374	2	8	16	TG	20	14
40135	4	2075924	2075947	2	12	24	AC	9	9
40143	4	2086563	2086592	3	10	30	CTC	10	7
40144	4	2086749	2086762	2	7	14	TG	10	6
40145	4	2086768	2086781	2	7	14	GT	14	7
40153	4	2104989	2105010	2	11	22	GT	21	7
40154	4	2107902	2107933	2	16	32	CA	11	12
40156	4	2110694	2110709	4	4	16	AAAG	21	6
40159	4	2132263	2132276	2	7	14	TG	22	10
40160	4	2132278	2132291	2	7	14	GT	19	8
40187	4	2208239	2208250	2	6	12	TA	21	3
40194	4	2224984	2225005	2	11	22	CA	20	8
40197	4	2234373	2234387	3	5	15	AAG	7	4
40198	4	2234398	2234409	2	6	12	AG	11	7
40199	4	2234461	2234488	2	14	28	CA	16	9
40206	4	2241249	2241266	2	9	18	CT	19	4
40211	4	2250712	2250727	4	4	16	GTGA	32	3
40212	4	2251128	2251143	2	8	16	AC	20	8
40214	4	2265626	2265649	4	6	24	AATA	18	11
40217	4	2280421	2280444	2	12	24	AC	12	8
40218	4	2281808	2281822	3	5	15	ATA	20	4
40227	4	2322339	2322364	2	13	26	GT	17	17
40235	4	2363889	2363918	2	15	30	CA	12	12
40239	4	2379067	2379084	2	9	18	CA	10	11
40240	4	2379252	2379267	2	8	16	TG	22	12
40242	4	2385395	2385414	2	10	20	CT	11	7
40244	4	2385786	2385803	3	6	18	TCA	26	11
40245	4	2387153	2387173	3	7	21	GCA	32	12
40250	4	2399390	2399401	2	6	12	TA	24	4
40262	4	2429916	2429931	2	8	16	AC	22	7
40263	4	2430056	2430067	2	6	12	AG	25	5
40264	4	2430281	2430314	2	17	34	TC	7	9
40266	4	2431066	2431077	2	6	12	CA	15	3

40268	4	2431298	2431327	3	10	30	GAG	13	7
40271	4	2431911	2431924	2	7	14	GT	13	9
40283	4	2435952	2435972	3	7	21	GAG	23	6
40284	4	2436412	2436435	3	8	24	TCT	17	12
40288	4	2441794	2441805	2	6	12	GT	32	3
40297	4	2452739	2452758	2	10	20	GA	29	3
40299	4	2460323	2460354	2	16	32	TG	8	7
40313	4	2503420	2503457	2	19	38	TG	11	14
40315	4	2504693	2504704	2	6	12	GT	21	3
40318	4	2510151	2510174	3	8	24	TTG	14	4
40326	4	2530084	2530105	2	11	22	CA	22	10
40329	4	2543663	2543680	3	6	18	CCT	24	4
40337	4	2563826	2563857	2	16	32	CA	20	8
40338	4	2563996	2564009	2	7	14	GT	19	3
40342	4	2570441	2570454	2	7	14	GT	17	7
40348	4	2576120	2576137	2	9	18	TG	28	5
40350	4	2591963	2591977	3	5	15	CTG	15	3
40361	4	2607679	2607700	2	11	22	TG	23	3
40362	4	2608187	2608202	2	8	16	TC	25	6
40363	4	2609614	2609633	2	10	20	CA	23	4
40379	4	2663585	2663616	2	16	32	GT	9	6
40382	4	2666243	2666260	2	9	18	GT	11	7
40383	4	2666487	2666508	2	11	22	TG	21	6
40384	4	2667021	2667056	2	18	36	CA	7	11
40388	4	2670352	2670366	3	5	15	CTG	27	3
40392	4	2675221	2675235	3	5	15	CTT	30	3
40395	4	2683978	2684017	2	20	40	TG	16	8
40398	4	2686493	2686508	4	4	16	TTAT	26	5
40400	4	2690328	2690341	2	7	14	TC	8	7
40404	4	2695617	2695644	2	14	28	TG	7	7
40412	4	2704928	2704957	2	15	30	AC	10	9
40413	4	2705930	2705941	2	6	12	TA	26	3
40418	4	2710699	2710724	2	13	26	TG	19	14
40421	4	2713484	2713517	2	17	34	CA	11	6
40431	4	2727528	2727561	2	17	34	AC	11	9
40439	4	2754783	2754798	2	8	16	GA	22	4
40452	4	2778428	2778445	2	9	18	GT	22	5
40454	4	2781898	2781925	2	14	28	CA	14	6
40456	4	2782703	2782714	2	6	12	GT	11	12
40457	4	2783036	2783075	2	20	40	CA	18	11
40462	4	2786234	2786275	3	14	42	AGC	28	4
40466	4	2795253	2795282	2	15	30	TG	22	17
40468	4	2808784	2808797	2	7	14	CA	17	7
40469	4	2808800	2808813	2	7	14	CA	20	4
40471	4	2810172	2810199	2	14	28	CA	13	4
40474	4	2817921	2817940	2	10	20	GT	6	6
40475	4	2818054	2818071	2	9	18	CA	14	3
40476	4	2818334	2818355	2	11	22	TC	17	5
40477	4	2819843	2819864	2	11	22	GT	11	10
40480	4	2825879	2825894	2	8	16	GT	18	3
40481	4	2825898	2825909	2	6	12	TG	22	6

40482	4	2826252	2826275	2	12	24	CA	20	10
40496	4	2842784	2842811	2	14	28	GT	6	7
40497	4	2843410	2843461	2	26	52	TG	9	7
40499	4	2846454	2846469	2	8	16	GT	18	10
40500	4	2847950	2847961	2	6	12	TG	27	3
40505	4	2856809	2856846	2	19	38	AC	10	10
40512	4	2865222	2865253	2	16	32	TG	13	16
40513	4	2868018	2868041	3	8	24	CAT	31	11
40524	4	2897078	2897093	2	8	16	TC	24	5
40531	4	2901666	2901703	2	19	38	GT	13	14
40532	4	2902919	2902940	2	11	22	CA	16	8
40539	4	2933019	2933030	2	6	12	AC	18	3
40540	4	2933301	2933314	2	7	14	GA	14	3
40542	4	2935777	2935792	2	8	16	GT	25	4
40543	4	2937979	2937994	2	8	16	CA	18	6
40544	4	2938220	2938253	2	17	34	AC	12	8
40546	4	2941864	2941887	2	12	24	AC	12	8
40551	4	2945992	2946031	2	20	40	AC	21	13
40552	4	2946206	2946235	2	15	30	AC	10	11
40553	4	2949818	2949829	2	6	12	AC	27	5
40554	4	2953165	2953192	2	14	28	AC	21	7
40556	4	2965479	2965498	2	10	20	GT	17	5
40557	4	2967735	2967754	2	10	20	TG	7	3
40558	4	2968397	2968408	2	6	12	TC	11	3
40559	4	2968410	2968423	2	7	14	CT	10	4
40561	4	2968522	2968533	2	6	12	CT	12	4
40568	4	2984904	2984925	2	11	22	AC	7	5
40569	4	2987480	2987506	3	9	27	TCT	30	4
40570	4	2990018	2990033	4	4	16	TGTT	26	3
40571	4	2990263	2990276	2	7	14	TC	25	4
40573	4	2991692	2991718	3	9	27	CTC	9	4
40578	4	2998144	2998163	2	10	20	CA	16	6
40589	4	3017027	3017042	2	8	16	AC	24	3
40591	4	3019505	3019518	2	7	14	GT	31	3
40597	4	3031065	3031082	2	9	18	TG	22	3
40598	4	3031186	3031201	2	8	16	AC	26	7
40599	4	3031771	3031800	2	15	30	GT	10	8
40603	4	3039680	3039691	2	6	12	TC	18	7
40613	4	3096293	3096320	2	14	28	CA	16	10
40616	4	3108870	3108881	2	6	12	AC	32	6
40617	4	3109084	3109103	2	10	20	CA	16	7
40618	4	3109180	3109227	2	24	48	TG	13	14
40620	4	3110312	3110335	4	6	24	CCTT	13	4
40630	4	3136475	3136496	2	11	22	CA	8	3
40635	4	3142439	3142464	2	13	26	GT	15	11
40641	4	3150943	3150958	4	4	16	TGTC	27	4
40642	4	3151167	3151208	2	21	42	CA	15	12
40643	4	3151441	3151454	2	7	14	CA	17	3
40644	4	3153336	3153371	2	18	36	GA	8	5
40648	4	3155460	3155483	3	8	24	GCT	15	7
40650	4	3155616	3155635	4	5	20	AGAC	18	11

40652	4	3155852	3155875	2	12	24	GA	15	8
40674	4	3181363	3181382	2	10	20	GT	20	4
40675	4	3182219	3182236	2	9	18	AC	23	9
40677	4	3188532	3188547	4	4	16	GACA	20	7
40680	4	3190557	3190586	3	10	30	GAG	6	5
40681	4	3191929	3191946	3	6	18	ATG	34	4
40685	4	3194151	3194170	2	10	20	AC	11	9
40686	4	3194299	3194320	2	11	22	AC	18	3
40693	4	3208740	3208757	3	6	18	TGC	20	8
40695	4	3211498	3211527	2	15	30	GA	12	17
40697	4	3224723	3224734	2	6	12	AT	15	6
40700	4	3235171	3235188	3	6	18	TAT	26	8
40701	4	3235698	3235733	2	18	36	AC	8	9
40705	4	3243339	3243360	2	11	22	GT	22	13
40708	4	3246205	3246216	2	6	12	CA	26	3
40709	4	3246360	3246380	3	7	21	CTT	27	3
40721	4	3263125	3263142	2	9	18	AC	21	10
40723	4	3265720	3265733	2	7	14	CA	23	4
40725	4	3272064	3272087	4	6	24	TATT	12	4
40727	4	3275255	3275282	2	14	28	TG	12	7
40728	4	3275361	3275378	2	9	18	GT	16	7
40735	4	3291786	3291824	3	13	39	TCT	22	6
40737	4	3292139	3292158	2	10	20	GT	21	5
40738	4	3292999	3293016	2	9	18	TG	31	7
40739	4	3293114	3293145	2	16	32	AC	14	5
40740	4	3294292	3294309	2	9	18	TG	14	12
40741	4	3294530	3294543	2	7	14	TC	16	6
40742	4	3294698	3294713	2	8	16	TG	11	3
40747	4	3297687	3297712	2	13	26	GT	11	6
40748	4	3299446	3299469	2	12	24	CA	21	6
40751	4	3301489	3301522	2	17	34	TG	10	7
40761	4	3307723	3307734	2	6	12	AC	20	3
40762	4	3307819	3307848	2	15	30	AC	22	11
40768	4	3310130	3310177	3	16	48	AAG	12	12
40769	4	3310627	3310646	2	10	20	GA	16	10
40770	4	3310782	3310802	3	7	21	CCA	23	4
40771	4	3311260	3311274	3	5	15	GCC	12	3
40774	4	3312936	3312961	2	13	26	AC	6	7
40779	4	3313847	3313867	3	7	21	CTG	25	6
40780	4	3314006	3314031	2	13	26	GT	18	5
40781	4	3315530	3315544	3	5	15	ATA	25	3
40782	4	3315813	3315848	2	18	36	AC	6	6
40784	4	3317125	3317154	2	15	30	TG	14	10
40786	4	3319360	3319371	2	6	12	TG	26	3
40787	4	3319584	3319611	2	14	28	GT	14	12
40788	4	3323029	3323044	2	8	16	AC	22	3
40797	4	3323758	3323777	2	10	20	GA	16	9
40809	4	3353036	3353063	2	14	28	TG	24	4
40820	4	3384109	3384148	2	20	40	AC	24	13
40821	4	3385050	3385069	4	5	20	TGAT	29	8
40825	4	3393114	3393127	2	7	14	TG	25	3

40830	4	3397734	3397763	2	15	30	CA	14	9
40833	4	3403044	3403057	2	7	14	AG	28	5
40835	4	3412263	3412312	2	25	50	CA	8	7
40840	4	3426616	3426627	2	6	12	GT	32	3
40844	4	3434254	3434271	2	9	18	GT	22	5
40845	4	3434988	3435002	3	5	15	GGA	21	7
40849	4	3438227	3438246	4	5	20	TTAT	13	6
40850	4	3438248	3438263	4	4	16	TATT	13	8
40853	4	3446956	3446983	2	14	28	GT	13	6
40862	4	3463007	3463028	2	11	22	CA	14	6
40866	4	3467071	3467090	2	10	20	AC	16	4
40871	4	3470798	3470811	2	7	14	CA	15	6
40873	4	3471233	3471256	3	8	24	CTT	13	12
40876	4	3472401	3472440	2	20	40	CA	17	9
40877	4	3473687	3473704	2	9	18	AC	11	5
40878	4	3473706	3473725	2	10	20	CA	9	6
40879	4	3473896	3473945	2	25	50	TG	16	14
40882	4	3474253	3474278	2	13	26	AC	14	6
40884	4	3476457	3476474	2	9	18	CA	12	11
40885	4	3476934	3476945	2	6	12	TG	16	3
40887	4	3477085	3477108	2	12	24	AC	21	5
40888	4	3479925	3479944	2	10	20	AC	21	3
40890	4	3482420	3482435	2	8	16	CA	23	4
40891	4	3482461	3482475	3	5	15	CAG	31	4
40897	4	3483105	3483124	2	10	20	TG	11	6
40900	4	3483505	3483518	2	7	14	CA	18	5
40904	4	3485122	3485143	2	11	22	CA	15	5
40907	4	3487256	3487267	2	6	12	AC	31	3
40912	4	3491639	3491658	2	10	20	CA	16	6
40915	4	3497256	3497283	2	14	28	GA	10	5
40916	4	3498191	3498210	2	10	20	TC	26	7
40923	4	3499862	3499900	3	13	39	AAG	22	11
40924	4	3500014	3500027	2	7	14	AC	21	3
40925	4	3501455	3501474	4	5	20	ATTT	22	3
40927	4	3503986	3504001	2	8	16	TG	27	4
40931	4	3507603	3507617	3	5	15	TAC	27	4
40932	4	3508808	3508827	2	10	20	GT	20	7
40933	4	3508957	3508986	2	15	30	AC	10	10
40934	4	3509127	3509160	2	17	34	TG	13	14
40935	4	3509313	3509338	2	13	26	TG	10	6
40939	4	3510749	3510776	2	14	28	CA	8	5
40945	4	3514559	3514574	2	8	16	AG	18	5
40949	4	3517197	3517224	2	14	28	AG	9	6
40951	4	3517670	3517732	3	21	63	GCT	20	20
40955	4	3522804	3522819	2	8	16	GT	27	5
40956	4	3524500	3524527	2	14	28	CA	9	6
40969	4	3533590	3533603	2	7	14	CA	28	3
40970	4	3534228	3534249	2	11	22	TG	19	4
40973	4	3542028	3542043	4	4	16	GGCT	21	4
40975	4	3542469	3542492	4	6	24	CAGA	18	4
40978	4	3547160	3547171	2	6	12	AC	27	3

40979	4	3547266	3547301	2	18	36	TG	11	8
40983	4	3550304	3550327	2	12	24	GT	16	8
40984	4	3550490	3550523	2	17	34	AC	11	9
40985	4	3553483	3553500	2	9	18	AC	25	9
40987	4	3554511	3554522	2	6	12	CA	24	5
41000	4	3569372	3569421	2	25	50	CA	6	9
41001	4	3569542	3569573	2	16	32	AC	6	8
41003	4	3571856	3571867	2	6	12	AC	31	5
41004	4	3572031	3572090	2	30	60	AC	13	13
41006	4	3575203	3575214	2	6	12	AC	22	3
41024	4	3591010	3591021	2	6	12	AC	20	4
41025	4	3591207	3591246	2	20	40	TG	6	7
41026	4	3591375	3591390	2	8	16	GT	8	4
41029	4	3595371	3595398	4	7	28	AAAT	18	5
41031	4	3597837	3597854	2	9	18	GT	21	4
41033	4	3598292	3598303	2	6	12	AG	29	3
41034	4	3598697	3598714	2	9	18	TG	21	8
41038	4	3605018	3605029	2	6	12	TG	14	7
41039	4	3605031	3605048	2	9	18	GT	12	8
41041	4	3605236	3605255	2	10	20	CA	20	11
41042	4	3605557	3605589	3	11	33	GAG	15	6
41046	4	3608665	3608698	2	17	34	AC	8	10
41058	4	3613231	3613260	2	15	30	TG	13	10
41059	4	3613950	3613969	2	10	20	CA	16	14
41060	4	3614128	3614153	2	13	26	GT	9	5
41064	4	3615852	3615871	2	10	20	CA	23	7
41065	4	3616985	3617004	2	10	20	AC	19	6
41069	4	3619645	3619662	2	9	18	AC	16	3
41079	4	3634561	3634596	4	9	36	TGGA	28	8
41080	4	3636572	3636585	2	7	14	CA	21	3
41081	4	3637310	3637323	2	7	14	CA	20	4
41083	4	3639850	3639873	2	12	24	GT	19	3
41087	4	3643146	3643165	2	10	20	TG	19	6
41090	4	3643535	3643548	2	7	14	TG	23	3
41097	4	3653108	3653135	2	14	28	GT	12	11
41098	4	3653274	3653289	4	4	16	TTTG	12	5
41099	4	3655767	3655780	2	7	14	AG	28	4
41100	4	3655836	3655867	2	16	32	AC	23	12
41101	4	3656259	3656284	2	13	26	AC	6	9
41104	4	3658458	3658489	4	8	32	AGAC	22	7
41108	4	3663408	3663419	2	6	12	TG	18	4
41109	4	3663421	3663432	2	6	12	GT	25	5
41111	4	3665982	3665993	2	6	12	TG	16	6
41120	4	3679886	3679899	2	7	14	GT	24	10
41122	4	3682470	3682489	2	10	20	AC	16	4
41123	4	3683248	3683277	2	15	30	AG	13	4
41126	4	3687060	3687087	2	14	28	TG	17	11
41127	4	3687776	3687793	3	6	18	CCA	16	3
41128	4	3687975	3687988	2	7	14	CT	28	3
41129	4	3688086	3688101	2	8	16	GA	22	4
41133	4	3699243	3699266	3	8	24	TTA	18	6

41134	4	3699342	3699369	2	14	28	GA	8	8
41135	4	3701772	3701801	2	15	30	CA	20	17
41138	4	3704429	3704452	4	6	24	TCTT	20	3
41139	4	3711118	3711137	2	10	20	GT	22	4
41141	4	3713549	3713576	4	7	28	CAGT	20	3
41144	4	3718043	3718054	2	6	12	TG	23	9
41148	4	3724122	3724137	2	8	16	GA	14	9
41150	4	3729536	3729559	2	12	24	AC	19	10
41153	4	3731324	3731335	2	6	12	TC	23	4
41159	4	3738193	3738230	2	19	38	GT	6	9
41160	4	3738369	3738390	2	11	22	CA	18	7
41161	4	3738564	3738585	2	11	22	AG	23	7
41165	4	3744006	3744017	2	6	12	AG	25	3
41172	4	3758892	3758933	2	21	42	CA	14	12
41174	4	3774640	3774657	2	9	18	CT	11	4
41181	4	3792127	3792150	4	6	24	GTGC	17	10
41185	4	3799652	3799669	2	9	18	AG	25	3
41201	4	3829470	3829489	4	5	20	TGTC	28	3
41204	4	3837629	3837650	2	11	22	GT	13	11
41209	4	3856286	3856297	2	6	12	TG	27	4
41218	4	3883037	3883050	2	7	14	AG	27	3
41225	4	3893830	3893847	2	9	18	CA	18	5
41230	4	3908858	3908873	2	8	16	CA	16	8
41235	4	3911165	3911180	2	8	16	CA	26	4
41236	4	3911279	3911292	2	7	14	TG	11	8
41237	4	3911294	3911311	2	9	18	GT	16	10
41238	4	3913038	3913049	2	6	12	TG	15	5
41239	4	3913250	3913283	2	17	34	CA	23	15
41240	4	3919087	3919114	2	14	28	CA	10	14
41243	4	3922858	3922883	2	13	26	TG	7	8
41246	4	3925718	3925733	4	4	16	TTTA	12	7
41247	4	3926141	3926160	2	10	20	TG	23	5
41248	4	3929286	3929305	2	10	20	CA	14	6
41253	4	3938569	3938582	2	7	14	GT	13	9
41255	4	3939253	3939264	2	6	12	AC	28	3
41259	4	3943931	3943950	2	10	20	CA	28	5
41260	4	3946914	3946927	2	7	14	CA	25	6
41261	4	3947011	3947038	2	14	28	CA	20	9
41262	4	3947214	3947227	2	7	14	AC	23	10
41264	4	3948569	3948584	2	8	16	CA	11	9
41266	4	3949378	3949407	2	15	30	GT	20	8
41267	4	3950138	3950171	2	17	34	TG	14	7
41268	4	3951232	3951245	2	7	14	GT	29	3
41269	4	3951331	3951354	2	12	24	TG	7	3
41271	4	3951535	3951556	2	11	22	CA	14	11
41278	4	3960738	3960783	2	23	46	GT	9	9
41279	4	3960977	3960998	2	11	22	GT	22	8
41282	4	3971759	3971796	2	19	38	TG	6	6
41284	4	3973004	3973023	2	10	20	GT	18	6
41285	4	3976297	3976324	2	14	28	TG	13	10
41286	4	3978339	3978362	4	6	24	CCAT	28	6

41287	4	3979343	3979358	2	8	16	AC	17	8
41293	4	3979751	3979770	2	10	20	AG	11	8
41296	4	3980188	3980201	2	7	14	TG	16	6
41301	4	3982586	3982613	2	14	28	GT	23	6
41305	4	3984583	3984596	2	7	14	AC	12	4
41306	4	3985835	3985850	2	8	16	AC	19	3
41307	4	4001383	4001398	2	8	16	TC	8	8
41316	4	4006424	4006435	2	6	12	AC	17	3
41320	4	4008456	4008473	2	9	18	AC	11	4
41322	4	4010358	4010373	2	8	16	CT	18	4
41323	4	4012322	4012345	2	12	24	TG	20	9
41327	4	4022738	4022773	2	18	36	CA	13	11
41334	4	4031426	4031453	2	14	28	AC	17	16
41337	4	4035881	4035904	2	12	24	GT	14	12
41339	4	4036620	4036651	2	16	32	GT	11	7
41341	4	4037233	4037244	2	6	12	AT	11	3
41342	4	4038635	4038650	2	8	16	GT	27	5
41345	4	4043551	4043566	2	8	16	TG	19	5
41346	4	4043568	4043587	2	10	20	GT	11	5
41347	4	4044285	4044300	2	8	16	CA	10	4
41348	4	4044478	4044503	2	13	26	GT	6	7
41350	4	4045525	4045536	2	6	12	TG	20	11
41353	4	4048317	4048336	2	10	20	CA	18	3
41355	4	4051529	4051548	2	10	20	TG	31	7
41359	4	4052820	4052851	2	16	32	GT	12	12
41364	4	4054048	4054067	2	10	20	TG	13	6
41368	4	4056598	4056619	2	11	22	TG	18	7
41369	4	4058139	4058160	2	11	22	GT	18	4
41371	4	4058233	4058250	2	9	18	AC	18	8
41372	4	4058460	4058477	2	9	18	GT	16	4
41376	4	4061582	4061613	2	16	32	TG	10	12
41378	4	4061752	4061773	2	11	22	GT	13	9
41379	4	4062008	4062019	2	6	12	GT	23	3
41383	4	4063915	4063932	2	9	18	AC	14	6
41384	4	4063964	4063981	2	9	18	GT	32	6
41386	4	4066175	4066186	2	6	12	CA	23	4
41387	4	4066328	4066357	2	15	30	GT	18	6
41390	4	4066635	4066664	2	15	30	TG	13	11
41391	4	4066711	4066732	2	11	22	AC	15	4
41392	4	4066947	4066970	2	12	24	TG	20	4
41394	4	4067433	4067450	2	9	18	GA	17	3
41395	4	4067475	4067488	2	7	14	AG	12	13
41399	4	4073259	4073278	2	10	20	TG	19	8
41403	4	4075385	4075402	2	9	18	GT	11	3
41407	4	4084581	4084602	2	11	22	TG	10	5
41414	4	4089458	4089475	2	9	18	AC	14	3
41418	4	4091160	4091177	2	9	18	AC	18	3
41419	4	4091452	4091465	2	7	14	TG	14	4
41425	4	4097012	4097035	2	12	24	GA	17	8
41426	4	4100226	4100275	2	25	50	TG	7	10
41427	4	4102150	4102189	2	20	40	GT	7	9

41429	4	4104083	4104124	2	21	42	TG	8	10
41433	4	4107321	4107334	2	7	14	CA	6	4
41437	4	4109626	4109646	3	7	21	CTC	19	4
41441	4	4110189	4110218	2	15	30	CA	9	8
41461	4	4119652	4119685	2	17	34	TG	9	9
41466	4	4121722	4121737	2	8	16	CA	21	9
41469	4	4126106	4126119	2	7	14	TG	25	6
41472	4	4128407	4128420	2	7	14	GT	23	5
41474	4	4128999	4129022	2	12	24	AG	11	7
41477	4	4130543	4130560	2	9	18	AC	16	8
41481	4	4132291	4132302	2	6	12	CA	27	4
41482	4	4134297	4134314	2	9	18	TG	21	6
41483	4	4139484	4139503	2	10	20	TG	10	6
41484	4	4140952	4140963	2	6	12	GT	18	5
41495	4	4147294	4147315	2	11	22	TG	21	4
41496	4	4147539	4147550	2	6	12	CA	12	4
41499	4	4148634	4148657	2	12	24	AG	6	10
41501	4	4149326	4149337	2	6	12	CA	29	4
41502	4	4149339	4149354	2	8	16	AC	26	5
41503	4	4154972	4154991	2	10	20	CA	9	5
41504	4	4155271	4155284	2	7	14	AG	20	3
41507	4	4158842	4158867	2	13	26	CA	8	6
41508	4	4159296	4159333	2	19	38	CA	21	12
41515	4	4162246	4162259	2	7	14	TG	24	3
41517	4	4168012	4168025	2	7	14	CT	21	3
41518	4	4168173	4168198	2	13	26	GA	11	8
41519	4	4169224	4169235	2	6	12	TG	20	4
41520	4	4169409	4169424	4	4	16	CCTC	20	3
41527	4	4177395	4177418	2	12	24	GA	21	9
41542	4	4187451	4187478	2	14	28	CA	13	15
41544	4	4188486	4188515	2	15	30	GT	6	7
41548	4	4198300	4198317	2	9	18	GT	18	6
41549	4	4198456	4198479	2	12	24	AC	8	7
41552	4	4200625	4200650	2	13	26	CA	18	6
41553	4	4200924	4200937	2	7	14	GA	18	3
41555	4	4201365	4201385	3	7	21	CCT	19	5
41557	4	4203086	4203111	2	13	26	GT	10	11
41558	4	4203209	4203234	2	13	26	CA	18	6
41560	4	4204572	4204587	2	8	16	GA	27	3
41561	4	4204717	4204738	2	11	22	AC	6	8
41562	4	4205901	4205938	2	19	38	GT	12	7
41563	4	4208734	4208745	2	6	12	GA	29	4
41565	4	4209263	4209282	2	10	20	TG	22	7
41568	4	4216549	4216562	2	7	14	AC	30	7
41569	4	4216694	4216705	2	6	12	GT	22	7
41570	4	4216707	4216718	2	6	12	TG	26	6
41572	4	4219492	4219511	2	10	20	AC	12	4
41574	4	4225690	4225717	2	14	28	CA	12	11
41575	4	4227223	4227240	3	6	18	CCT	12	4
41578	4	4232502	4232529	2	14	28	GT	9	8
41580	4	4232969	4232980	2	6	12	GT	33	3

41584	4	4234896	4234933	2	19	38	CA	18	10
41585	4	4235246	4235279	2	17	34	CA	7	8
41589	4	4236467	4236496	2	15	30	CA	17	8
41591	4	4238298	4238319	2	11	22	AC	6	9
41593	4	4238463	4238486	2	12	24	CA	16	9
41638	4	4274145	4274162	2	9	18	GA	17	9
41643	4	4277117	4277130	2	7	14	AG	16	4
41646	4	4279884	4279909	2	13	26	CA	13	9
41650	4	4285726	4285773	4	12	48	AATC	16	9
41651	4	4286324	4286337	2	7	14	AG	13	5
41652	4	4286339	4286350	2	6	12	GA	13	7
41672	4	4297161	4297176	4	4	16	CACG	15	5
41673	4	4297223	4297240	2	9	18	CA	27	10
41674	4	4297421	4297434	2	7	14	TG	19	7
41676	4	4297679	4297704	2	13	26	TG	12	6
41679	4	4300393	4300420	2	14	28	GA	7	8
41680	4	4302336	4302353	2	9	18	CA	20	7
41683	4	4307105	4307118	2	7	14	CA	16	6
41689	4	4314638	4314665	2	14	28	CA	13	8
41693	4	4316232	4316243	2	6	12	AT	23	3
41694	4	4316370	4316387	2	9	18	CA	17	6
41695	4	4316965	4316992	2	14	28	GT	21	5
41697	4	4318858	4318875	2	9	18	GT	12	5
41698	4	4319821	4319850	3	10	30	CAG	21	10
41699	4	4320512	4320526	3	5	15	AAT	20	3
41702	4	4321372	4321391	2	10	20	AC	11	8
41704	4	4322824	4322841	2	9	18	GT	21	7
41705	4	4323143	4323176	2	17	34	GT	22	8
41709	4	4332058	4332089	2	16	32	GT	7	8
41715	4	4336671	4336682	2	6	12	GT	18	5
41716	4	4337166	4337183	2	9	18	TG	19	4
41717	4	4337205	4337218	2	7	14	TG	14	5
41718	4	4337338	4337367	2	15	30	TG	12	6
41720	4	4339128	4339149	2	11	22	GT	19	13
41721	4	4341819	4341834	4	4	16	AAAT	22	4
41723	4	4343205	4343232	2	14	28	CA	16	15
41727	4	4348136	4348171	2	18	36	TC	10	9
41728	4	4348356	4348381	2	13	26	TG	9	8
41733	4	4350219	4350236	2	9	18	GT	14	3
41743	4	4367811	4367824	2	7	14	GT	16	5
41745	4	4369381	4369416	2	18	36	TC	10	10
41748	4	4370289	4370302	2	7	14	TC	21	4
41751	4	4370494	4370513	2	10	20	GT	19	9
41752	4	4370708	4370723	2	8	16	AC	18	8
41753	4	4371444	4371455	2	6	12	AC	27	3
41755	4	4373398	4373427	2	15	30	TG	14	4
41760	4	4378026	4378057	2	16	32	TG	14	9
41761	4	4378080	4378094	3	5	15	ATG	19	3
41763	4	4378186	4378211	2	13	26	GT	10	9
41765	4	4378405	4378416	2	6	12	CA	11	4
41767	4	4380311	4380336	2	13	26	GT	19	11

41769	4	4383484	4383499	2	8	16	TG	27	6
41771	4	4383927	4383952	2	13	26	AC	8	11
41772	4	4384102	4384115	2	7	14	CA	15	5
41773	4	4385334	4385353	2	10	20	TG	20	3
41780	4	4391272	4391285	2	7	14	TG	28	4
41788	4	4396955	4396976	2	11	22	AC	22	13
41797	4	4403112	4403125	2	7	14	AC	25	5
41801	4	4409396	4409409	2	7	14	AC	23	4
41808	4	4419825	4419840	2	8	16	GT	23	4
41811	4	4422310	4422329	2	10	20	TG	17	5
41815	4	4427607	4427628	2	11	22	TG	13	7
41818	4	4429530	4429557	4	7	28	TGGA	17	7
41823	4	4433800	4433811	2	6	12	AG	18	3
41826	4	4440631	4440656	2	13	26	CA	24	9
41827	4	4442112	4442145	2	17	34	AC	14	9
41828	4	4442447	4442478	2	16	32	TC	12	9
41830	4	4444107	4444118	2	6	12	CA	8	3
41833	4	4450677	4450690	2	7	14	CA	19	4
41834	4	4451990	4452005	2	8	16	AC	14	6
41835	4	4452045	4452080	2	18	36	AC	6	5
41836	4	4452208	4452229	2	11	22	CA	12	13
41838	4	4452551	4452572	2	11	22	AC	12	8
41839	4	4456458	4456469	2	6	12	GT	24	7
41840	4	4456704	4456719	2	8	16	AC	24	5
41854	4	4486978	4487003	2	13	26	TG	20	13
41859	4	4493991	4494010	4	5	20	ATGC	32	6
41862	4	4501127	4501158	2	16	32	TG	8	9
41863	4	4501315	4501338	2	12	24	TG	17	9
41864	4	4501424	4501447	2	12	24	GA	9	5
41867	4	4503923	4503940	2	9	18	CA	18	11
41868	4	4510620	4510639	2	10	20	CA	16	5
41869	4	4512705	4512742	2	19	38	AC	7	8
41870	4	4513357	4513368	2	6	12	CA	23	3
41877	4	4516424	4516479	4	14	56	CTAT	9	9
41880	4	4518716	4518733	2	9	18	AC	24	5
41881	4	4519020	4519033	2	7	14	CT	25	3
41883	4	4521813	4521830	2	9	18	CA	14	8
41884	4	4524930	4524951	2	11	22	CA	19	5
41885	4	4525060	4525079	2	10	20	AC	17	8
41886	4	4526049	4526072	2	12	24	AC	11	9
41887	4	4526431	4526454	2	12	24	TG	22	13
41888	4	4529214	4529231	2	9	18	CA	21	3
41889	4	4534101	4534121	3	7	21	TAT	29	4
41890	4	4534879	4534892	2	7	14	CT	15	4
41891	4	4537675	4537702	2	14	28	CA	13	8
41897	4	4546677	4546694	2	9	18	TC	17	8
41899	4	4553832	4553861	2	15	30	AC	8	10
41900	4	4558975	4558988	2	7	14	GT	28	4
41906	4	4585712	4585727	4	4	16	TCAG	33	3
41908	4	4599748	4599765	2	9	18	TG	17	9
41912	4	4604024	4604037	2	7	14	GT	16	3

41915	4	4615147	4615162	2	8	16	TG	11	6
41919	4	4627237	4627250	2	7	14	GT	23	5
41925	4	4642103	4642122	2	10	20	AC	19	4
41926	4	4642223	4642252	2	15	30	CA	15	8
41927	4	4646312	4646323	2	6	12	AC	29	4
41928	4	4647519	4647530	2	6	12	GT	27	3
41929	4	4647556	4647569	2	7	14	GT	11	3
41941	4	4668086	4668103	2	9	18	CT	10	5
41943	4	4669575	4669598	2	12	24	GA	15	7
41946	4	4670551	4670564	2	7	14	TG	14	4
41948	4	4680434	4680447	2	7	14	AC	19	11
41950	4	4680925	4680936	2	6	12	AC	25	13
41951	4	4681402	4681416	3	5	15	TCC	27	4
41952	4	4681532	4681549	2	9	18	GA	16	4
41953	4	4685518	4685529	2	6	12	CA	17	3
41958	4	4697333	4697346	2	7	14	TG	15	3
41973	4	4728840	4728881	2	21	42	CA	23	11
41974	4	4734361	4734380	2	10	20	AC	19	7
41978	4	4735227	4735240	2	7	14	AC	19	3
41981	4	4737526	4737537	2	6	12	TG	21	5
41983	4	4738694	4738717	2	12	24	AC	19	7
41984	4	4739078	4739089	2	6	12	AC	15	10
41987	4	4745649	4745672	2	12	24	TG	24	7
41997	4	4757508	4757523	2	8	16	TG	19	3
41998	4	4763770	4763783	2	7	14	GT	12	6
41999	4	4766781	4766796	2	8	16	TG	27	6
42004	4	4798851	4798874	4	6	24	TCTG	23	6
42006	4	4808348	4808362	3	5	15	GAG	22	3
42011	4	4834203	4834220	2	9	18	GT	18	5
42013	4	4843662	4843673	2	6	12	CT	28	6
42023	4	4868219	4868242	2	12	24	CA	21	6
42031	4	4890990	4891004	3	5	15	ATC	19	4
42032	4	4892110	4892129	2	10	20	TG	19	7
42033	4	4893613	4893638	2	13	26	AC	18	9
42037	4	4899194	4899207	2	7	14	TG	20	4
42040	4	4901906	4901937	2	16	32	CA	12	9
42041	4	4902232	4902251	2	10	20	AT	9	7
42049	4	4934477	4934498	2	11	22	TG	15	3
42051	4	4937546	4937572	3	9	27	AAG	24	8
42052	4	4938372	4938392	3	7	21	AAT	13	6
42053	4	4939223	4939242	4	5	20	CATC	20	5
42056	4	4941376	4941389	2	7	14	CA	14	10
42058	4	4943060	4943073	2	7	14	AC	29	3
42059	4	4943304	4943319	2	8	16	AC	19	3
42068	4	4964856	4964881	2	13	26	GT	22	10
42069	4	4966198	4966209	2	6	12	GT	28	3
42070	4	4970573	4970584	2	6	12	CA	32	3
42075	4	4973270	4973285	2	8	16	GT	13	3
42081	4	4982646	4982673	2	14	28	AC	19	6
42083	4	4987752	4987765	2	7	14	CA	32	3
42090	4	5005776	5005799	2	12	24	GT	17	9

42091	4	5005969	5005980	2	6	12	TG	22	4
42092	4	5006106	5006125	2	10	20	GT	23	8
42094	4	5009430	5009441	2	6	12	TG	26	3
42096	4	5017586	5017603	3	6	18	TTA	24	5
42099	4	5022628	5022655	2	14	28	TG	7	6
42100	4	5024650	5024665	2	8	16	AT	19	5
42103	4	5026501	5026512	2	6	12	CA	17	5
42105	4	5036296	5036323	2	14	28	GA	14	17
42107	4	5040248	5040269	2	11	22	CA	14	5
42108	4	5044352	5044409	2	29	58	CA	8	13
42112	4	5047913	5047932	2	10	20	AC	16	8
42119	4	5056679	5056720	3	14	42	ATT	11	5
42121	4	5057534	5057549	2	8	16	TG	24	5
42123	4	5057752	5057775	2	12	24	TG	14	7
42124	4	5057894	5057921	2	14	28	TG	17	9
42125	4	5064171	5064190	2	10	20	GT	12	4
42137	4	5081941	5081976	2	18	36	GT	11	9
42141	4	5084694	5084717	2	12	24	AC	17	9
42142	4	5088222	5088243	2	11	22	GT	26	12
42143	4	5093940	5093973	2	17	34	AC	10	10
42150	4	5133233	5133278	2	23	46	TG	7	10
42158	4	5145748	5145779	2	16	32	CA	15	11
42159	4	5146847	5146876	2	15	30	AC	13	6
42165	4	5158639	5158653	3	5	15	CAG	26	3
42166	4	5161839	5161866	2	14	28	AC	10	3
42168	4	5162251	5162270	2	10	20	TC	16	5
42170	4	5163688	5163739	4	13	52	TATC	21	8
42172	4	5166356	5166395	2	20	40	TG	7	7
42173	4	5166524	5166551	2	14	28	GT	11	4
42174	4	5166677	5166702	2	13	26	CA	13	5
42177	4	5168122	5168151	2	15	30	CA	20	11
42178	4	5172542	5172567	2	13	26	AC	16	7
42183	4	5180616	5180629	2	7	14	TG	21	5
42184	4	5182207	5182220	2	7	14	AC	26	5
42186	4	5192980	5193001	2	11	22	CT	19	8
42191	4	5195788	5195815	2	14	28	CA	17	15
42194	4	5207608	5207629	2	11	22	TG	6	6
42197	4	5210723	5210744	2	11	22	GT	7	4
42199	4	5217304	5217341	2	19	38	TG	7	7
42201	4	5218592	5218619	2	14	28	CA	10	9
42204	4	5223702	5223731	2	15	30	TC	10	9
42205	4	5224033	5224060	2	14	28	TG	21	10
42210	4	5238877	5238897	3	7	21	GAG	32	9
42213	4	5240245	5240276	2	16	32	GT	15	8
42214	4	5241009	5241026	3	6	18	CTT	31	3
42217	4	5247110	5247127	2	9	18	TG	13	11
42218	4	5247129	5247144	2	8	16	GT	20	15
42221	4	5256834	5256847	2	7	14	TG	28	5
42235	4	5282963	5282990	2	14	28	AC	7	10
42239	4	5289825	5289840	2	8	16	AC	29	6
42246	4	5312542	5312573	2	16	32	AC	10	8

42260	4	5363468	5363481	2	7	14	TG	22	3
42263	4	5368448	5368483	2	18	36	GT	7	9
42264	4	5374192	5374206	3	5	15	TGT	26	5
42265	4	5378575	5378586	2	6	12	AC	29	4
42266	4	5382876	5382891	4	4	16	CATT	21	4
42270	4	5391397	5391408	2	6	12	GT	29	3
42272	4	5392126	5392137	2	6	12	GT	20	3
42273	4	5394941	5394954	2	7	14	GT	25	5
42277	4	5400386	5400403	2	9	18	AC	26	5
42278	4	5401056	5401073	2	9	18	GA	27	7
42279	4	5401410	5401427	2	9	18	GA	15	4
42280	4	5402916	5402931	2	8	16	AT	18	7
42286	4	5406975	5406992	2	9	18	AC	20	8
42289	4	5407428	5407445	2	9	18	TG	15	8
42290	4	5407589	5407600	2	6	12	AC	22	10
42291	4	5407604	5407621	2	9	18	CA	23	8
42292	4	5410110	5410121	2	6	12	CA	9	5
42293	4	5418628	5418657	2	15	30	CA	21	16
42296	4	5420852	5420887	2	18	36	AC	8	8
42297	4	5423038	5423077	4	10	40	ATCT	12	11
42298	4	5423776	5423799	3	8	24	GAT	28	4
42299	4	5424429	5424444	2	8	16	CA	13	3
42301	4	5426491	5426516	2	13	26	AC	10	11
42303	4	5428823	5428846	2	12	24	CA	14	7
42304	4	5428971	5428982	2	6	12	GT	20	5
42305	4	5428984	5429001	2	9	18	TG	23	5
42307	4	5432001	5432012	2	6	12	TC	22	4
42310	4	5434520	5434543	2	12	24	TG	15	15
42312	4	5434742	5434759	2	9	18	AC	23	6
42316	4	5442170	5442215	2	23	46	AC	19	11
42321	4	5454319	5454334	2	8	16	AC	30	5
42323	4	5455643	5455698	4	14	56	TCAA	11	8
42324	4	5455959	5455970	2	6	12	AC	25	6
42326	4	5456891	5456916	2	13	26	CA	7	9
42327	4	5457701	5457718	2	9	18	TG	19	5
42333	4	5460981	5460996	2	8	16	AC	20	7
42335	4	5468366	5468391	2	13	26	AG	18	7
42336	4	5469415	5469440	2	13	26	TG	15	12
42337	4	5469519	5469538	4	5	20	CGCA	19	16
42338	4	5469687	5469712	2	13	26	AC	14	13
42344	4	5480892	5480906	3	5	15	CAG	15	5
42345	4	5482202	5482223	2	11	22	TG	9	8
42349	4	5490442	5490455	2	7	14	CA	10	10
42350	4	5490456	5490467	2	6	12	AC	9	8
42358	4	5505971	5505994	2	12	24	TG	13	9
42360	4	5507254	5507281	2	14	28	CA	19	12
42361	4	5507821	5507832	2	6	12	GT	18	4
42362	4	5507902	5507913	2	6	12	TG	13	5
42363	4	5508073	5508100	2	14	28	AC	16	8
42364	4	5508222	5508245	2	12	24	TG	21	7
42365	4	5508429	5508442	2	7	14	AC	17	6

42367	4	5511946	5511973	2	14	28	AG	6	9
42371	4	5516999	5517024	2	13	26	TG	12	6
42372	4	5517149	5517168	2	10	20	TC	13	13
42375	4	5519750	5519773	2	12	24	AC	14	10
42379	4	5522189	5522212	2	12	24	TG	16	5
42381	4	5528617	5528632	4	4	16	CCTT	27	3
42386	4	5543424	5543445	2	11	22	CA	12	5
42387	4	5543490	5543509	2	10	20	AG	22	8
42389	4	5546576	5546591	2	8	16	TC	27	5
42391	4	5564224	5564255	2	16	32	CA	10	11
42394	4	5574826	5574849	4	6	24	AGAC	25	13
42398	4	5578781	5578798	2	9	18	CA	13	7
42401	4	5590366	5590385	2	10	20	GT	14	4
42402	4	5590904	5590917	2	7	14	AC	25	8
42403	4	5590918	5590931	2	7	14	CA	20	3
42407	4	5594283	5594312	2	15	30	CT	8	11
42409	4	5594390	5594405	2	8	16	GT	8	5
42410	4	5594735	5594750	2	8	16	TG	19	8
42411	4	5594754	5594765	2	6	12	GT	20	9
42414	4	5595668	5595697	2	15	30	AG	10	14
42422	4	5602315	5602338	2	12	24	CA	13	6
42425	4	5606862	5606873	2	6	12	CA	11	4
42426	4	5606876	5606887	2	6	12	CA	14	7
42433	4	5629033	5629048	2	8	16	TG	21	4
42435	4	5637397	5637412	4	4	16	AGTC	29	3
42437	4	5638302	5638313	2	6	12	CG	14	11
42440	4	5645310	5645353	2	22	44	CA	14	11
42445	4	5653141	5653158	2	9	18	CT	17	5
42449	4	5654458	5654481	2	12	24	GA	26	10
42450	4	5660264	5660275	2	6	12	TG	17	3
42455	4	5662143	5662174	2	16	32	TC	8	6
42456	4	5662755	5662806	4	13	52	CTTT	17	8
42457	4	5662990	5663035	2	23	46	CA	7	7
42458	4	5663169	5663202	2	17	34	GT	13	8
42459	4	5663360	5663401	2	21	42	TG	10	10
42466	4	5671300	5671329	3	10	30	GCA	21	5
42469	4	5672354	5672374	3	7	21	TGG	24	3
42474	4	5673978	5673995	2	9	18	CA	24	9
42479	4	5677870	5677903	2	17	34	GT	25	10
42480	4	5678338	5678357	2	10	20	CA	7	5
42484	4	5679784	5679799	2	8	16	GT	23	4
42488	4	5681544	5681579	2	18	36	TG	6	6
42495	4	5692963	5692984	2	11	22	TG	20	8
42496	4	5693249	5693276	2	14	28	GT	24	7
42498	4	5694206	5694233	2	14	28	AC	16	10
42500	4	5694628	5694661	2	17	34	GT	6	6
42503	4	5694871	5694896	2	13	26	CA	11	12
42512	4	5716903	5716922	2	10	20	GT	10	3
42515	4	5719063	5719082	2	10	20	TC	23	3
42517	4	5720627	5720638	2	6	12	TG	30	3
42520	4	5724199	5724212	2	7	14	CA	24	10

42521	4	5725157	5725208	4	13	52	TACA	14	6
42522	4	5725937	5725958	2	11	22	TG	14	7
42529	4	5732505	5732526	2	11	22	CA	17	10
42533	4	5737259	5737282	2	12	24	CA	13	10
42534	4	5737399	5737416	2	9	18	AC	19	9
42543	4	5743717	5743738	2	11	22	GT	10	7
42551	4	5749274	5749299	2	13	26	GT	14	10
42552	4	5749984	5750003	2	10	20	GT	18	12
42553	4	5750942	5750980	3	13	39	GTT	23	14
42554	4	5753177	5753196	2	10	20	CA	21	7
42556	4	5756285	5756320	2	18	36	TG	20	8
42569	4	5776110	5776123	2	7	14	GT	13	7
42573	4	5777536	5777553	2	9	18	TG	22	18
42574	4	5779569	5779584	2	8	16	AC	14	3
42577	4	5779901	5779924	2	12	24	TG	17	10
42582	4	5783306	5783325	2	10	20	AC	17	5
42584	4	5783700	5783721	2	11	22	AC	11	4
42585	4	5787442	5787455	2	7	14	AT	25	3
42592	4	5792017	5792044	4	7	28	TTTA	11	4
42594	4	5795758	5795785	2	14	28	CT	15	8
42596	4	5796328	5796353	2	13	26	AC	14	8
42597	4	5796529	5796558	2	15	30	CA	11	7
42599	4	5798906	5798927	2	11	22	AC	15	4
42600	4	5799005	5799024	2	10	20	GT	15	6
42602	4	5802193	5802210	3	6	18	CAC	26	6
42611	4	5817515	5817574	4	15	60	TTTC	14	13
42612	4	5818126	5818141	2	8	16	CA	8	7
42614	4	5822593	5822610	2	9	18	GT	25	10
42616	4	5825633	5825668	2	18	36	TG	9	7
42617	4	5826005	5826038	2	17	34	GT	12	10
42618	4	5826138	5826161	2	12	24	CA	20	9
42620	4	5826369	5826418	2	25	50	AC	7	7
42631	4	5829824	5829835	2	6	12	TG	15	6
42634	4	5831384	5831401	2	9	18	GT	12	8
42635	4	5831404	5831421	2	9	18	GT	15	9
42636	4	5835331	5835354	2	12	24	AC	24	7
42637	4	5835524	5835541	2	9	18	TG	23	7
42640	4	5836796	5836833	2	19	38	TG	6	6
42641	4	5837474	5837499	2	13	26	TG	19	7
42643	4	5838889	5838916	2	14	28	GT	8	9
42644	4	5839043	5839072	2	15	30	CA	7	12
42645	4	5839161	5839190	2	15	30	TG	9	7
42646	4	5839379	5839402	2	12	24	AG	11	8
42657	4	5844426	5844443	2	9	18	TG	24	8
42659	4	5844620	5844637	2	9	18	TG	9	3
42664	4	5846339	5846350	2	6	12	AC	23	4
42669	4	5855836	5855851	2	8	16	TG	23	3
42670	4	5857952	5857989	2	19	38	CA	13	8
42671	4	5858117	5858156	2	20	40	CA	9	10
42674	4	5858386	5858415	2	15	30	GT	8	9
42675	4	5859927	5859960	2	17	34	CA	17	7

42681	4	5861138	5861161	3	8	24	AAT	28	5
42682	4	5862940	5862993	2	27	54	AG	9	13
42683	4	5864112	5864139	2	14	28	CA	18	6
42686	4	5869919	5869938	2	10	20	TC	20	3
42689	4	5872497	5872517	3	7	21	TTC	19	9
42690	4	5872977	5872990	2	7	14	GT	18	3
42698	4	5879171	5879198	2	14	28	AC	9	11
42701	4	5881208	5881225	2	9	18	AC	20	6
42702	4	5881228	5881243	2	8	16	AC	20	10
42706	4	5883590	5883629	2	20	40	TG	11	10
42707	4	5883953	5883966	2	7	14	TA	13	4
42710	4	5887691	5887708	3	6	18	TGT	30	8
42712	4	5889676	5889697	2	11	22	CA	8	9
42728	4	5914442	5914453	2	6	12	CT	24	3
42730	4	5914529	5914540	2	6	12	TG	24	3
42732	4	5918214	5918249	2	18	36	GT	17	11
42736	4	5925368	5925383	2	8	16	TG	17	5
42738	4	5929118	5929143	2	13	26	TG	10	11
42746	4	5939443	5939470	2	14	28	GT	11	8
42747	4	5941029	5941058	2	15	30	AC	10	3
42749	4	5945241	5945296	4	14	56	CACG	20	10
42754	4	5949706	5949743	2	19	38	CA	12	10
42756	4	5953039	5953054	2	8	16	CA	15	4
42759	4	5953426	5953439	2	7	14	TG	17	3
42760	4	5953691	5953704	2	7	14	GT	21	7
42770	4	5967445	5967462	2	9	18	AT	12	5
42772	4	5968333	5968352	2	10	20	TG	28	10
42778	4	5974560	5974575	2	8	16	AG	21	4
42780	4	5976449	5976462	2	7	14	AC	24	3
42786	4	5981960	5981977	3	6	18	ATT	8	6
42789	4	5985959	5985974	2	8	16	GT	21	4
42791	4	5988668	5988681	2	7	14	AC	14	10
42797	4	5989903	5989918	2	8	16	TG	20	5
42799	4	5991901	5991918	2	9	18	GT	24	5
42805	4	5994701	5994724	3	8	24	CTC	24	4
42807	4	5995105	5995128	2	12	24	AC	10	10
42808	4	5995719	5995746	2	14	28	CA	25	8
42811	4	5996963	5996992	2	15	30	TG	16	8
42814	4	5997941	5997952	2	6	12	GT	30	4
42815	4	5998124	5998151	2	14	28	GT	19	6
42819	4	6002200	6002219	2	10	20	CT	19	5
42821	4	6002368	6002389	2	11	22	CA	9	9
42824	4	6002887	6002932	2	23	46	GT	11	10
42826	4	6003319	6003352	2	17	34	CA	16	17
42827	4	6003402	6003415	2	7	14	GT	26	4
42831	4	6006078	6006093	4	4	16	TTCT	15	3
42833	4	6006287	6006316	2	15	30	AC	11	5
42836	4	6006464	6006481	3	6	18	GGT	24	4
42839	4	6009973	6009987	3	5	15	ATA	20	3
42841	4	6010537	6010564	2	14	28	GT	12	11
42842	4	6012346	6012363	2	9	18	TG	16	5

42845	4	6014022	6014039	2	9	18	AC	19	4
42847	4	6016550	6016567	2	9	18	CA	18	3
42864	4	6042211	6042224	2	7	14	TC	27	3
42873	4	6047191	6047208	3	6	18	CTT	15	5
42875	4	6048756	6048779	4	6	24	GGCA	25	6
42877	4	6053092	6053125	2	17	34	AC	9	7
42880	4	6055594	6055613	2	10	20	GA	6	6
42881	4	6055916	6055927	2	6	12	AC	24	3
42883	4	6059742	6059765	2	12	24	GT	18	11
42884	4	6059862	6059885	2	12	24	AC	15	6
42893	4	6070098	6070127	2	15	30	TG	12	7
42895	4	6070231	6070266	2	18	36	CA	11	9
42915	4	6085913	6085932	2	10	20	CA	17	7
42916	4	6086067	6086088	2	11	22	GT	14	14
42918	4	6086261	6086272	2	6	12	CA	27	5
42919	4	6088418	6088435	2	9	18	GT	19	8
42920	4	6088526	6088559	2	17	34	CA	13	8
42930	4	6101086	6101097	2	6	12	TG	16	3
42931	4	6101110	6101121	2	6	12	TG	25	4
42933	4	6101372	6101397	2	13	26	TG	11	8
42941	4	6103713	6103766	2	27	54	CA	9	10
42944	4	6112394	6112439	2	23	46	GT	16	9
42955	4	6118872	6118911	2	20	40	AC	7	11
42970	4	6134737	6134756	2	10	20	GT	14	12
42974	4	6138726	6138737	2	6	12	GA	15	3
42977	4	6138969	6138988	2	10	20	TG	11	4
42980	4	6139683	6139712	2	15	30	CA	16	12
42983	4	6141333	6141346	2	7	14	TG	22	3
42985	4	6141601	6141612	2	6	12	CA	32	3
42986	4	6141687	6141706	2	10	20	TG	25	6
42988	4	6141915	6141928	2	7	14	GT	24	4
42989	4	6142048	6142081	2	17	34	TG	9	11
42991	4	6146470	6146489	2	10	20	CA	16	6
42992	4	6146536	6146549	2	7	14	CA	30	4
42994	4	6148148	6148171	2	12	24	GT	14	4
42997	4	6150513	6150542	2	15	30	GT	13	10
42998	4	6152066	6152077	2	6	12	TG	22	6
43010	4	6159661	6159672	2	6	12	GA	14	8
43016	4	6163167	6163178	2	6	12	AT	24	5
43018	4	6163790	6163811	2	11	22	AG	6	7
43021	4	6173495	6173514	4	5	20	AAAG	22	5
43022	4	6173850	6173875	2	13	26	AC	13	10
43023	4	6174102	6174113	2	6	12	TG	22	3
43027	4	6176572	6176601	2	15	30	AC	22	9
43029	4	6185421	6185432	2	6	12	GT	23	5
43030	4	6185435	6185450	2	8	16	GT	14	4
43031	4	6188627	6188659	3	11	33	CCT	12	6
43034	4	6189257	6189268	2	6	12	CA	15	5
43047	4	6213708	6213741	2	17	34	CA	7	11
43048	4	6213930	6213951	2	11	22	TG	20	7
43052	4	6216933	6216950	2	9	18	TG	7	8

43056	4	6220781	6220792	2	6	12	TG	20	3
43058	4	6224315	6224326	2	6	12	TG	19	3
43062	4	6227048	6227067	2	10	20	CA	8	7
43064	4	6227207	6227224	2	9	18	AC	19	3
43067	4	6230218	6230249	4	8	32	GTGC	12	15
43073	4	6233533	6233552	2	10	20	CA	21	11
43078	4	6235748	6235769	2	11	22	AC	21	6
43080	4	6236012	6236039	2	14	28	CA	12	7
43082	4	6237288	6237307	2	10	20	AC	15	5
43083	4	6237384	6237403	2	10	20	GT	19	3
43085	4	6237861	6237888	2	14	28	AC	8	4
43086	4	6238005	6238018	2	7	14	AC	17	3
43089	4	6241555	6241570	2	8	16	CA	28	5
43094	4	6247671	6247690	2	10	20	GT	15	5
43095	4	6250716	6250729	2	7	14	AC	21	5
43096	4	6250824	6250843	2	10	20	CA	10	7
43097	4	6250994	6251007	2	7	14	AC	19	6
43098	4	6254075	6254088	2	7	14	CA	12	6
43099	4	6255323	6255336	2	7	14	TG	25	6
43102	4	6256162	6256207	2	23	46	TG	6	9
43105	4	6258421	6258456	4	9	36	ATCA	27	3
43106	4	6259296	6259323	2	14	28	TC	19	8
43112	4	6264338	6264353	4	4	16	TTTG	22	4
43113	4	6264784	6264831	2	24	48	TG	6	9
43116	4	6265661	6265674	2	7	14	AC	15	3
43126	4	6280621	6280634	2	7	14	GT	11	6
43127	4	6280636	6280653	2	9	18	TG	11	5
43128	4	6280752	6280773	2	11	22	AC	19	4
43131	4	6281135	6281160	2	13	26	AC	11	6
43132	4	6282562	6282587	2	13	26	AC	15	11
43134	4	6282789	6282812	2	12	24	TG	8	7
43135	4	6282913	6282938	2	13	26	CA	18	8
43138	4	6283641	6283658	3	6	18	GGA	18	4
43142	4	6286729	6286740	2	6	12	TG	17	4
43145	4	6288909	6288926	2	9	18	CA	17	4
43148	4	6290772	6290799	4	7	28	AGAA	11	5
43153	4	6293848	6293859	2	6	12	GT	26	4
43154	4	6294108	6294123	2	8	16	GT	25	5
43156	4	6296062	6296077	2	8	16	AC	15	4
43157	4	6296136	6296151	2	8	16	CA	18	8
43159	4	6296270	6296317	2	24	48	GT	8	9
43163	4	6297526	6297541	2	8	16	GA	14	6
43166	4	6299329	6299354	2	13	26	GT	17	4
43168	4	6300632	6300679	2	24	48	GT	24	15
43170	4	6301652	6301667	2	8	16	GT	23	8
43171	4	6301807	6301834	2	14	28	CA	9	5
43176	4	6302688	6302705	2	9	18	AC	13	3
43179	4	6303707	6303746	2	20	40	GT	9	12
43180	4	6303832	6303849	2	9	18	CA	13	5
43182	4	6304605	6304632	2	14	28	AG	9	5
43184	4	6304898	6304917	2	10	20	CA	21	5

43186	4	6305221	6305238	3	6	18	TGC	18	8
43187	4	6305268	6305291	2	12	24	TG	12	3
43193	4	6307397	6307410	2	7	14	TG	22	6
43194	4	6307511	6307534	2	12	24	CA	12	7
43195	4	6307766	6307793	2	14	28	TG	11	11
43200	4	6310881	6310900	2	10	20	TG	19	9
43203	4	6311914	6311937	2	12	24	CA	27	7
43205	4	6312758	6312771	2	7	14	AC	20	7
43208	4	6313031	6313068	2	19	38	CA	17	7
43209	4	6314243	6314254	2	6	12	GT	13	3
43210	4	6314257	6314268	2	6	12	GT	19	5
43211	4	6315147	6315166	2	10	20	CA	20	7
43212	4	6315291	6315308	2	9	18	CA	22	5
43213	4	6315604	6315619	2	8	16	CA	20	5
43214	4	6315843	6315862	2	10	20	TG	20	5
43215	4	6315973	6316006	2	17	34	AC	15	9
43216	4	6316078	6316095	2	9	18	AC	17	9
43217	4	6318024	6318055	2	16	32	GT	11	8
43221	4	6319242	6319259	2	9	18	GT	21	7
43222	4	6319505	6319516	2	6	12	CA	20	3
43224	4	6319944	6319958	3	5	15	GCT	24	3
43231	4	6321639	6321650	2	6	12	GT	14	4
43232	4	6321788	6321799	2	6	12	AG	11	13
43233	4	6321801	6321818	2	9	18	GA	13	11
43234	4	6322684	6322704	3	7	21	TGG	20	4
43240	4	6324174	6324197	4	6	24	ATAA	7	4
43242	4	6324951	6324971	3	7	21	CTC	19	3
43250	4	6327014	6327037	2	12	24	AC	17	9
43266	4	6341993	6342012	4	5	20	CTGT	25	4
43268	4	6345174	6345191	2	9	18	AC	22	3
43276	4	6349919	6349946	4	7	28	GATG	7	4
43278	4	6350151	6350164	2	7	14	TG	17	3
43279	4	6350304	6350337	2	17	34	AC	10	10
43281	4	6357024	6357049	2	13	26	CA	18	9
43287	4	6362429	6362446	2	9	18	GT	10	5
43288	4	6362636	6362653	2	9	18	TG	23	7
43296	4	6373598	6373625	2	14	28	AC	6	8
43297	4	6373727	6373740	2	7	14	AC	15	3
43301	4	6382291	6382305	3	5	15	GCA	28	4
43302	4	6382715	6382730	4	4	16	GAAT	24	7
43308	4	6386654	6386665	2	6	12	CA	17	3
43309	4	6386689	6386706	3	6	18	CAG	18	5
43314	4	6389306	6389317	2	6	12	GT	17	9
43315	4	6389318	6389331	2	7	14	GA	14	8
43324	4	6401095	6401118	2	12	24	CA	8	7
43328	4	6404722	6404733	2	6	12	CA	17	6
43329	4	6405017	6405034	2	9	18	TG	21	3
43336	4	6408576	6408591	2	8	16	AG	17	3
43339	4	6416040	6416057	2	9	18	AC	26	11
43341	4	6421822	6421835	2	7	14	CA	18	4
43342	4	6421837	6421850	2	7	14	AC	20	3

43344	4	6422387	6422407	3	7	21	GAG	9	5
43346	4	6423160	6423191	2	16	32	AC	14	10
43348	4	6423339	6423350	2	6	12	TC	11	11
43349	4	6423351	6423370	2	10	20	AC	14	13
43362	4	6433209	6433222	2	7	14	CA	29	3
43363	4	6436312	6436337	2	13	26	GT	10	8
43365	4	6441305	6441318	2	7	14	GT	29	6
43370	4	6447283	6447296	2	7	14	CA	25	4
43372	4	6450404	6450437	2	17	34	AG	15	13
43374	4	6451786	6451807	2	11	22	AC	18	11
43377	4	6452115	6452126	2	6	12	TG	30	4
43378	4	6452271	6452302	2	16	32	GT	13	11
43382	4	6455448	6455471	4	6	24	AGGA	24	5
43385	4	6458814	6458835	2	11	22	GT	21	7
43390	4	6474671	6474698	2	14	28	GT	10	10
43392	4	6474873	6474900	2	14	28	TG	12	10
43393	4	6475109	6475120	2	6	12	GT	27	4
43406	4	6485432	6485452	3	7	21	AGG	13	9
43407	4	6492134	6492147	2	7	14	GA	33	3
43409	4	6493111	6493126	2	8	16	CA	31	8
43410	4	6494111	6494126	2	8	16	TG	22	5
43412	4	6499081	6499095	3	5	15	ACT	15	6
43413	4	6500342	6500353	2	6	12	CT	17	3
43414	4	6500449	6500470	2	11	22	CT	8	4
43415	4	6500782	6500802	3	7	21	AGG	20	6
43428	4	6523053	6523064	2	6	12	CA	14	8
43429	4	6523066	6523079	2	7	14	AC	11	6
43430	4	6525190	6525207	2	9	18	CA	21	6
43431	4	6527321	6527334	2	7	14	GT	32	5
43432	4	6528172	6528189	2	9	18	CA	21	8
43433	4	6528330	6528343	2	7	14	TC	18	9
43434	4	6528723	6528736	2	7	14	TG	28	3
43439	4	6539521	6539536	2	8	16	AC	25	3
43441	4	6546051	6546068	2	9	18	TG	26	8
43444	4	6559244	6559257	2	7	14	CA	10	3
43445	4	6568527	6568560	2	17	34	AC	9	12
43446	4	6568699	6568726	2	14	28	TG	14	15
43447	4	6568848	6568859	2	6	12	AC	24	5
43449	4	6569360	6569375	2	8	16	AG	24	3
43450	4	6572290	6572305	2	8	16	CA	8	6
43452	4	6574117	6574158	2	21	42	GA	16	9
43453	4	6574370	6574387	2	9	18	TG	25	5
43455	4	6579109	6579134	2	13	26	GT	18	11
43456	4	6579218	6579235	2	9	18	GT	22	6
43457	4	6581155	6581166	2	6	12	TG	22	4
43459	4	6583375	6583400	2	13	26	AG	8	8
43463	4	6596730	6596747	2	9	18	AC	18	7
43464	4	6597059	6597072	2	7	14	AC	19	6
43465	4	6597195	6597208	2	7	14	AC	25	6
43466	4	6597211	6597226	2	8	16	AC	29	3
43468	4	6597397	6597418	2	11	22	AC	16	6

43469	4	6600406	6600451	2	23	46	TG	7	9
43470	4	6600726	6600737	2	6	12	GT	28	5
43477	4	6606789	6606800	2	6	12	CA	18	13
43478	4	6609601	6609630	2	15	30	AC	12	9
43479	4	6609764	6609793	2	15	30	CA	8	4
43489	4	6628681	6628696	2	8	16	AC	16	6
43491	4	6630807	6630834	2	14	28	CA	18	9
43492	4	6631047	6631060	2	7	14	GT	33	4
43495	4	6636684	6636695	2	6	12	TC	7	6
43496	4	6636696	6636709	2	7	14	AC	12	6
43497	4	6637876	6637897	2	11	22	CT	27	5
43499	4	6640150	6640165	2	8	16	CA	23	4
43502	4	6644679	6644704	2	13	26	CA	23	11
43503	4	6647442	6647465	2	12	24	TG	21	17
43505	4	6658856	6658873	2	9	18	GT	19	13
43507	4	6666345	6666370	2	13	26	GT	20	10
43508	4	6670327	6670370	2	22	44	AC	14	11
43516	4	6680879	6680893	3	5	15	GGA	14	4
43517	4	6682672	6682683	2	6	12	CA	12	6
43518	4	6684256	6684287	2	16	32	GT	11	10
43522	4	6687337	6687356	2	10	20	AC	9	5
43524	4	6692732	6692745	2	7	14	AC	22	5
43527	4	6694396	6694419	2	12	24	AC	10	9
43531	4	6698190	6698217	2	14	28	TG	10	9
43535	4	6701373	6701394	2	11	22	AC	16	5
43536	4	6701466	6701493	2	14	28	CA	11	10
43537	4	6701669	6701682	2	7	14	CA	7	4
43540	4	6704653	6704702	2	25	50	CA	8	10
43541	4	6706253	6706266	2	7	14	TG	24	4
43542	4	6707423	6707434	2	6	12	AC	15	4
43543	4	6707599	6707630	2	16	32	CA	12	8
43545	4	6709598	6709623	2	13	26	CT	19	6
43548	4	6712118	6712131	2	7	14	AC	26	7
43549	4	6712209	6712228	2	10	20	GT	28	9
43551	4	6715650	6715669	4	5	20	ATTT	16	6
43552	4	6717214	6717229	4	4	16	TTGT	28	7
43570	4	6778967	6779010	2	22	44	GT	14	16
43576	4	6784603	6784622	2	10	20	GT	8	7
43589	4	6818200	6818211	2	6	12	GT	29	9
43597	4	6830750	6830769	2	10	20	CA	14	6
43598	4	6831010	6831029	2	10	20	GT	17	5
43604	4	6840136	6840159	2	12	24	TG	23	5
43606	4	6842505	6842524	2	10	20	TC	17	9
43610	4	6853001	6853012	2	6	12	CA	24	11
43611	4	6853046	6853061	2	8	16	CA	12	13
43616	4	6876905	6876950	2	23	46	TG	14	13
43617	4	6877969	6877984	2	8	16	TG	14	3
43620	4	6885411	6885426	4	4	16	ATAA	6	4
43621	4	6885924	6885935	2	6	12	AC	16	10
43622	4	6887518	6887533	2	8	16	CA	16	6
43623	4	6887535	6887546	2	6	12	AC	17	6

43625	4	6889725	6889742	2	9	18	GT	24	6
43627	4	6892756	6892787	2	16	32	GT	17	7
43628	4	6894320	6894341	2	11	22	GT	20	6
43630	4	6894578	6894597	2	10	20	AC	26	8
43631	4	6894732	6894757	2	13	26	CA	21	12
43632	4	6894901	6894924	2	12	24	TG	16	6
43633	4	6897010	6897021	2	6	12	AC	27	7
43636	4	6901583	6901602	2	10	20	AC	18	5
43637	4	6901788	6901801	2	7	14	AC	15	15
43639	4	6902410	6902431	2	11	22	TG	19	9
43641	4	6904405	6904426	2	11	22	TG	24	11
43643	4	6906392	6906407	4	4	16	GCAC	27	10
43646	4	6912805	6912824	2	10	20	TG	8	5
43648	4	6913001	6913024	3	8	24	ATC	21	4
43651	4	6918161	6918192	2	16	32	TG	7	6
43655	4	6919537	6919592	2	28	56	CA	9	14
43661	4	6921376	6921417	2	21	42	GA	9	10
43662	4	6921575	6921592	2	9	18	AC	12	6
43663	4	6921767	6921778	2	6	12	AC	21	4
43664	4	6924770	6924829	2	30	60	AC	8	11
43665	4	6925126	6925137	2	6	12	TG	25	3
43666	4	6925456	6925469	2	7	14	TG	22	3
43670	4	6931970	6931985	2	8	16	CA	24	7
43685	4	6944073	6944090	3	6	18	GGA	23	3
43686	4	6944720	6944747	2	14	28	TG	15	10
43688	4	6948946	6948981	2	18	36	CA	10	11
43690	4	6954022	6954033	2	6	12	GT	29	3
43691	4	6954620	6954635	2	8	16	TG	21	3
43692	4	6957362	6957395	2	17	34	TG	15	8
43696	4	6961592	6961617	2	13	26	AC	6	9
43697	4	6962989	6963004	2	8	16	AC	22	4
43701	4	6966724	6966741	2	9	18	TG	17	5
43702	4	6967012	6967027	2	8	16	CA	25	5
43703	4	6968042	6968077	2	18	36	CA	10	6
43704	4	6968127	6968172	2	23	46	CA	9	10
43709	4	6974423	6974450	2	14	28	TG	22	9
43710	4	6976049	6976063	3	5	15	TTC	15	5
43711	4	6976644	6976673	2	15	30	CA	9	5
43713	4	6976797	6976816	2	10	20	TG	17	9
43716	4	6979890	6979901	2	6	12	TG	26	3
43717	4	6981799	6981810	2	6	12	AC	25	4
43718	4	6982240	6982263	4	6	24	ACAG	31	3
43719	4	6983924	6983939	2	8	16	GT	33	4
43720	4	6984109	6984124	2	8	16	CA	26	3
43723	4	6984664	6984697	2	17	34	CT	9	6
43724	4	6984876	6984895	2	10	20	GT	15	9
43725	4	6985008	6985037	2	15	30	AC	15	8
43726	4	6985262	6985293	2	16	32	CA	14	11
43728	4	6985461	6985474	2	7	14	TG	17	3
43731	4	6990734	6990757	2	12	24	TG	19	7
43732	4	6990985	6991028	2	22	44	TG	9	7

43733	4	6991381	6991414	2	17	34	TG	20	7
43736	4	6994906	6994925	2	10	20	TG	19	8
43739	4	6997098	6997127	2	15	30	TG	21	7
43740	4	6997274	6997285	2	6	12	AC	28	4
43742	4	6999981	6999996	2	8	16	CA	14	9
43753	4	7018198	7018215	3	6	18	TCC	26	3
43754	4	7018983	7019004	2	11	22	GT	23	4
43756	4	7021043	7021064	2	11	22	TG	21	5
43757	4	7023033	7023064	2	16	32	GT	19	11
43759	4	7024946	7024959	2	7	14	CA	22	6
43760	4	7025049	7025064	4	4	16	ACTC	27	4
43762	4	7028030	7028047	2	9	18	CA	25	6
43769	4	7038960	7038973	2	7	14	TG	27	11
43774	4	7050896	7050929	2	17	34	TG	6	8
43776	4	7055551	7055566	2	8	16	CA	22	4
43777	4	7057496	7057523	2	14	28	CA	9	4
43781	4	7058593	7058610	2	9	18	AC	19	9
43784	4	7060467	7060488	2	11	22	GT	12	7
43785	4	7060754	7060779	2	13	26	GT	6	7
43790	4	7065945	7065968	2	12	24	GT	15	9
43807	4	7084038	7084053	2	8	16	CA	20	10
43809	4	7085634	7085659	2	13	26	CA	16	13
43817	4	7100258	7100269	2	6	12	GT	20	3
43818	4	7100772	7100789	2	9	18	TG	20	7
43820	4	7104206	7104239	2	17	34	GT	16	9
43825	4	7110709	7110720	2	6	12	CA	28	4
43826	4	7110968	7110985	2	9	18	AC	28	6
43827	4	7111388	7111401	2	7	14	TG	29	3
43828	4	7111573	7111592	2	10	20	CA	13	8
43835	4	7114060	7114091	2	16	32	AC	17	7
43836	4	7115710	7115733	2	12	24	CA	16	3
43838	4	7116956	7116977	2	11	22	AC	13	4
43839	4	7117009	7117034	2	13	26	AC	20	7
43840	4	7119102	7119125	4	6	24	AGAC	21	4
43842	4	7122357	7122392	2	18	36	TG	17	11
43847	4	7125673	7125688	2	8	16	CT	15	3
43848	4	7125714	7125737	2	12	24	AC	21	8
43852	4	7128246	7128257	2	6	12	AC	20	5
43854	4	7131319	7131330	2	6	12	CA	25	5
43859	4	7137040	7137065	2	13	26	GT	17	6
43861	4	7141000	7141015	4	4	16	CTTC	24	3
43862	4	7141347	7141378	2	16	32	AC	17	8
43864	4	7143121	7143142	2	11	22	TG	25	7
43865	4	7143591	7143608	2	9	18	AC	15	3
43870	4	7151724	7151738	3	5	15	GCT	26	5
43874	4	7152965	7152988	4	6	24	TATG	6	4
43875	4	7153302	7153316	3	5	15	GCA	26	4
43877	4	7154724	7154755	2	16	32	AG	18	9
43879	4	7159576	7159591	2	8	16	TG	16	5
43881	4	7159673	7159686	2	7	14	GT	20	7
43882	4	7159785	7159800	2	8	16	GT	20	4

43886	4	7166656	7166673	3	6	18	TGG	23	4
43887	4	7167403	7167416	2	7	14	TG	22	3
43891	4	7170641	7170654	2	7	14	GT	17	5
43892	4	7170780	7170825	2	23	46	AC	9	6
43894	4	7172185	7172220	2	18	36	AC	15	8
43895	4	7173960	7173971	2	6	12	CA	26	3
43896	4	7174132	7174151	2	10	20	CT	23	6
43897	4	7175819	7175838	2	10	20	AC	15	9
43898	4	7177780	7177815	2	18	36	AC	13	11
43899	4	7178008	7178023	2	8	16	AG	31	4
43903	4	7178545	7178571	3	9	27	CCT	31	5
43910	4	7183686	7183709	2	12	24	GT	23	9
43911	4	7185198	7185229	2	16	32	CA	11	8
43916	4	7191327	7191354	4	7	28	TGGC	23	13
43919	4	7197563	7197586	2	12	24	AC	7	7
43921	4	7200289	7200314	2	13	26	TG	21	8
43923	4	7203883	7203896	2	7	14	CA	10	3
43927	4	7220094	7220107	2	7	14	AC	25	3
43930	4	7220453	7220470	3	6	18	GAG	11	10
43931	4	7221892	7221903	2	6	12	AC	31	5
43932	4	7228433	7228444	2	6	12	GT	13	3
43936	4	7239105	7239118	2	7	14	AC	12	8
43941	4	7258692	7258727	3	12	36	ATC	25	7
43945	4	7261109	7261134	2	13	26	TG	11	7
43950	4	7280767	7280782	4	4	16	AACA	21	3
43951	4	7281308	7281325	2	9	18	AC	17	10
43954	4	7284156	7284170	3	5	15	TAT	15	3
43957	4	7303710	7303735	2	13	26	GT	13	13
43958	4	7309809	7309828	2	10	20	CA	19	10
43965	4	7318772	7318839	4	17	68	ATAG	17	10
43969	4	7328485	7328496	2	6	12	TC	18	3
43976	4	7335588	7335605	2	9	18	TG	18	3
43978	4	7342474	7342505	2	16	32	TG	10	8
43980	4	7343272	7343289	2	9	18	TG	12	9
43984	4	7343946	7343973	2	14	28	TG	11	8
43987	4	7346834	7346855	2	11	22	TG	16	7
43990	4	7347192	7347217	2	13	26	AC	17	9
43991	4	7347339	7347362	2	12	24	AC	13	14
43992	4	7347708	7347737	2	15	30	AC	10	11
43998	4	7352734	7352753	2	10	20	CT	14	3
44002	4	7352956	7352997	2	21	42	GA	8	6
44014	4	7358566	7358581	2	8	16	TG	19	6
44019	4	7361143	7361164	2	11	22	GT	20	4
44022	4	7365761	7365786	2	13	26	GT	8	10
44023	4	7365867	7365906	2	20	40	GA	12	8
44025	4	7366583	7366608	2	13	26	GT	16	11
44035	4	7370818	7370833	4	4	16	CGTG	19	5
44036	4	7370952	7370963	2	6	12	AC	26	6
44037	4	7371414	7371425	2	6	12	GA	25	4
44041	4	7373153	7373174	2	11	22	CT	20	9
44046	4	7375829	7375868	2	20	40	CA	18	12

44049	4	7377542	7377556	3	5	15	CTG	26	3
44050	4	7379165	7379184	2	10	20	GT	21	9
44053	4	7379757	7379772	2	8	16	CA	24	5
44056	4	7382985	7383004	2	10	20	GT	22	11
44061	4	7394347	7394362	4	4	16	TTTG	23	4
44065	4	7401042	7401059	2	9	18	AC	16	13
44067	4	7405973	7405990	2	9	18	TG	9	4
44068	4	7408210	7408233	2	12	24	AC	16	6
44085	4	7446926	7446939	2	7	14	GA	21	3
44092	4	7466856	7466881	2	13	26	AC	15	7
44093	4	7468478	7468517	2	20	40	TG	9	11
44097	4	7470564	7470587	2	12	24	TG	10	5
44101	4	7475168	7475193	2	13	26	CA	11	7
44103	4	7489427	7489444	2	9	18	TG	9	12
44104	4	7492477	7492488	2	6	12	TG	24	3
44109	4	7502116	7502139	2	12	24	CA	10	8
44112	4	7509672	7509685	2	7	14	AC	24	10
44113	4	7511144	7511167	2	12	24	CA	26	9
44116	4	7515537	7515554	3	6	18	CTT	26	6
44121	4	7521341	7521360	2	10	20	CA	17	3
44125	4	7524897	7524926	2	15	30	TG	28	6
44126	4	7525098	7525119	2	11	22	AC	6	7
44128	4	7530476	7530495	2	10	20	GT	16	10
44132	4	7538143	7538168	2	13	26	GA	16	9
44134	4	7545286	7545299	2	7	14	GA	15	4
44136	4	7555785	7555808	4	6	24	ACAG	9	6
44140	4	7566494	7566509	2	8	16	GT	30	4
44142	4	7568589	7568602	2	7	14	AC	23	3
44143	4	7569541	7569592	2	26	52	CA	13	12
44144	4	7569785	7569796	2	6	12	CA	21	3
44147	4	7573085	7573100	2	8	16	TG	8	8
44150	4	7585113	7585136	2	12	24	TG	21	6
44154	4	7596389	7596410	2	11	22	CA	13	15
44164	4	7627124	7627153	2	15	30	TG	15	8
44165	4	7627386	7627411	2	13	26	GT	18	8
44166	4	7633338	7633351	2	7	14	AC	28	3
44167	4	7633739	7633768	2	15	30	CA	16	10
44169	4	7635504	7635517	2	7	14	GT	24	4
44173	4	7647705	7647732	2	14	28	CA	15	12
44174	4	7648137	7648150	2	7	14	AC	22	4
44177	4	7650326	7650363	2	19	38	AC	7	8
44179	4	7654656	7654670	3	5	15	CCG	23	4
44180	4	7655109	7655132	2	12	24	GT	17	6
44194	4	7691465	7691476	2	6	12	CA	24	3
44200	4	7698888	7698907	2	10	20	TG	12	8
44201	4	7701919	7701933	3	5	15	ATT	26	3
44203	4	7702633	7702654	2	11	22	AG	15	6
44206	4	7710478	7710495	2	9	18	AC	21	4
44207	4	7711901	7711921	3	7	21	ATT	18	6
44208	4	7712863	7712883	3	7	21	CCT	26	3
44209	4	7713383	7713394	2	6	12	CA	28	4

44213	4	7717997	7718012	4	4	16	TTTG	17	4
44219	4	7732714	7732725	2	6	12	AC	25	3
44222	4	7740230	7740259	2	15	30	CA	8	8
44236	4	7760154	7760165	2	6	12	AC	28	3
44237	4	7765886	7765911	2	13	26	AC	7	8
44246	4	7781742	7781755	2	7	14	CA	20	3
44247	4	7782764	7782783	2	10	20	AC	22	6
44248	4	7783567	7783592	2	13	26	GT	23	9
44252	4	7788631	7788648	2	9	18	TG	24	9
44253	4	7788966	7788977	2	6	12	TG	14	7
44254	4	7789030	7789057	2	14	28	GT	11	4
44256	4	7792442	7792465	2	12	24	TG	8	10
44259	4	7801665	7801690	2	13	26	AC	10	7
44262	4	7805569	7805582	2	7	14	GT	14	3
44265	4	7808111	7808124	2	7	14	TG	20	4
44279	4	7832905	7832930	2	13	26	TC	12	12
44280	4	7835639	7835666	2	14	28	AC	7	8
44283	4	7843267	7843293	3	9	27	GCA	19	7
44284	4	7844163	7844182	4	5	20	TTTA	28	7
44292	4	7871086	7871099	2	7	14	AT	19	4
44306	4	7927141	7927158	2	9	18	TG	22	10
44307	4	7927161	7927174	2	7	14	TG	25	12
44310	4	7936648	7936677	3	10	30	TGT	25	5
44321	4	7986688	7986701	2	7	14	TG	25	3
44328	4	8002645	8002664	2	10	20	TG	18	9
44331	4	8004827	8004854	4	7	28	TCTT	10	8
44332	4	8007639	8007664	2	13	26	GT	24	5
44333	4	8008210	8008225	2	8	16	AG	22	6
44337	4	8021382	8021403	2	11	22	TG	8	7
44340	4	8033683	8033712	2	15	30	TG	9	13
44349	4	8057325	8057338	2	7	14	CA	24	4
44353	4	8065559	8065576	2	9	18	CT	13	8
44361	4	8086330	8086341	2	6	12	CA	14	3
44363	4	8087188	8087209	2	11	22	TG	13	14
44365	4	8087360	8087373	2	7	14	AC	18	4
44371	4	8094202	8094215	2	7	14	CA	25	7
44377	4	8126119	8126144	2	13	26	TG	14	7
44378	4	8127131	8127142	2	6	12	TG	37	3
44379	4	8128400	8128427	2	14	28	GT	20	5
44384	4	8130346	8130387	2	21	42	CA	13	9
44385	4	8130938	8130969	2	16	32	TG	15	10
44390	4	8137820	8137837	2	9	18	TG	19	12
44393	4	8144569	8144588	2	10	20	AC	15	11
44395	4	8148312	8148343	4	8	32	AAGA	27	3
44397	4	8150799	8150810	2	6	12	AT	14	5
44400	4	8152855	8152878	2	12	24	TG	23	9
44402	4	8153877	8153906	2	15	30	TG	25	11
44405	4	8159754	8159771	3	6	18	GGT	6	3
44408	4	8160835	8160854	2	10	20	CA	7	9
44413	4	8173191	8173202	2	6	12	GT	34	3
44418	4	8187994	8188026	3	11	33	GGA	12	5

44423	4	8195394	8195408	3	5	15	TCT	29	5
44424	4	8203022	8203037	2	8	16	GA	23	9
44427	4	8206445	8206480	2	18	36	AC	14	8
44433	4	8232338	8232357	2	10	20	CA	14	9
44441	4	8253054	8253071	3	6	18	GCT	24	3
44449	4	8258147	8258166	2	10	20	TG	15	4
44451	4	8265724	8265755	4	8	32	GATG	20	9
44456	4	8279657	8279702	2	23	46	AC	6	6
44457	4	8284893	8284907	3	5	15	TGC	24	5
44458	4	8287250	8287265	2	8	16	TG	21	4
44462	4	8293708	8293721	2	7	14	AG	13	3
44472	4	8303750	8303781	2	16	32	GT	13	11
44474	4	8305287	8305298	2	6	12	GT	22	3
44475	4	8308068	8308083	4	4	16	GCCA	19	5
44477	4	8310242	8310259	2	9	18	AC	21	6
44478	4	8312644	8312661	2	9	18	TG	21	6
44483	4	8318995	8319016	2	11	22	TG	13	9
44484	4	8319197	8319214	3	6	18	CCT	15	3
44485	4	8321146	8321160	3	5	15	TGC	32	3
44487	4	8334260	8334281	2	11	22	AG	21	6
44500	4	8373499	8373516	2	9	18	TG	18	13
44502	4	8375482	8375505	2	12	24	GT	6	8
44508	4	8391207	8391234	2	14	28	TG	26	11
44509	4	8391407	8391422	2	8	16	CA	22	8
44512	4	8394980	8395001	2	11	22	TG	11	11
44515	4	8402081	8402098	2	9	18	TG	24	3
44516	4	8402282	8402307	2	13	26	GT	17	14
44522	4	8411454	8411477	4	6	24	GTTG	25	5
44523	4	8416202	8416217	2	8	16	AC	20	6
44529	4	8428484	8428507	4	6	24	TGAA	33	3
44531	4	8433447	8433473	3	9	27	GCT	24	3
44533	4	8434953	8434982	2	15	30	AC	11	6
44540	4	8453583	8453598	4	4	16	TTTA	20	4
44552	4	8479963	8479988	2	13	26	TG	11	6
44553	4	8480188	8480213	2	13	26	AC	11	9
44556	4	8484214	8484225	2	6	12	AG	20	6
44557	4	8484319	8484336	2	9	18	CA	12	6
44558	4	8484338	8484353	2	8	16	AC	20	5
44559	4	8485674	8485685	2	6	12	CA	25	5
44560	4	8485687	8485700	2	7	14	AC	24	6
44565	4	8493635	8493662	2	14	28	GA	14	5
44573	4	8520746	8520759	2	7	14	CA	21	6
44574	4	8520979	8521008	2	15	30	CA	18	8
44576	4	8524357	8524410	2	27	54	TG	11	12
44578	4	8534214	8534227	2	7	14	AC	22	3
44579	4	8538395	8538420	2	13	26	CT	18	6
44580	4	8543913	8543934	2	11	22	CA	16	7
44581	4	8544101	8544116	2	8	16	AC	25	3
44585	4	8557302	8557325	3	8	24	TTG	21	4
44589	4	8565370	8565387	2	9	18	AC	15	8
44595	4	8573315	8573340	2	13	26	AC	14	7

44602	4	8580147	8580160	2	7	14	AC	25	6
44606	4	8582576	8582599	2	12	24	CA	16	6
44608	4	8595120	8595137	2	9	18	GT	27	9
44615	4	8614331	8614350	2	10	20	AG	11	7
44619	4	8614679	8614700	2	11	22	AC	19	11
44623	4	8617780	8617797	2	9	18	TA	13	6
44624	4	8622849	8622869	3	7	21	CAG	23	12
44629	4	8644538	8644549	2	6	12	TG	21	5
44630	4	8645406	8645419	2	7	14	GT	26	4
44631	4	8656787	8656824	2	19	38	AC	7	12
44638	4	8664307	8664328	2	11	22	GT	15	6
44640	4	8665579	8665590	2	6	12	TG	24	4
44648	4	8686194	8686205	2	6	12	CA	24	6
44653	4	8692796	8692821	2	13	26	AC	19	8
44656	4	8703214	8703234	3	7	21	GCT	21	7
44659	4	8705896	8705907	2	6	12	CA	32	7
44661	4	8715101	8715128	2	14	28	GT	7	10
44664	4	8735173	8735186	2	7	14	AC	13	6
44667	4	8737199	8737210	2	6	12	CA	26	3
44668	4	8738850	8738864	3	5	15	ATT	10	7
44671	4	8746304	8746315	2	6	12	TG	26	5
44675	4	8755930	8755962	3	11	33	ATC	6	4
44678	4	8757352	8757369	2	9	18	AG	21	7
44680	4	8763186	8763205	4	5	20	CAAA	9	3
44681	4	8764229	8764243	3	5	15	AAT	23	4
44685	4	8768140	8768153	2	7	14	CA	28	5
44703	4	8850737	8850760	2	12	24	GT	6	3
44708	4	8854835	8854852	2	9	18	CT	12	5
44709	4	8854876	8854897	2	11	22	CT	12	5
44710	4	8855814	8855827	2	7	14	TG	19	4
44722	4	8897339	8897374	2	18	36	GA	8	8
44733	4	8928552	8928563	2	6	12	GT	20	6
44734	4	8928653	8928692	2	20	40	GT	6	10
44741	4	8929169	8929184	4	4	16	TGTC	11	4
44755	4	8949590	8949629	2	20	40	AC	7	10
44757	4	8952325	8952350	2	13	26	TG	16	10
44758	4	8952644	8952671	2	14	28	TG	11	11
44768	4	8965381	8965402	2	11	22	CA	24	13
44772	4	8980819	8980842	3	8	24	TTC	19	8
44773	4	8983530	8983544	3	5	15	ATC	16	5
44774	4	8987480	8987493	2	7	14	CA	28	3
44776	4	8990244	8990264	3	7	21	TTA	25	4
44779	4	9004790	9004819	3	10	30	GGA	22	7
44794	4	9043615	9043634	4	5	20	GCGT	27	4
44795	4	9045895	9045932	2	19	38	GA	7	7
44800	4	9080159	9080198	2	20	40	GT	9	11
44807	4	9103500	9103521	2	11	22	GA	32	3
44813	4	9132899	9132916	2	9	18	CA	24	8
44815	4	9158477	9158496	2	10	20	TG	20	10
44822	4	9186240	9186255	2	8	16	AG	12	6
44829	4	9210888	9210911	2	12	24	CA	15	9

44844	4	9258831	9258858	4	7	28	TATG	8	3
44857	4	9308825	9308848	2	12	24	TC	14	9
44860	4	9316650	9316661	2	6	12	TG	20	3
44863	4	9324402	9324421	2	10	20	GT	20	5
44868	4	9333883	9333900	2	9	18	GA	26	3
44876	4	9358497	9358520	2	12	24	TG	15	9
44877	4	9364574	9364591	3	6	18	CAT	23	5
44879	4	9386938	9386949	2	6	12	GA	23	5
44882	4	9399617	9399640	4	6	24	TTTG	14	4
44889	4	9409333	9409364	2	16	32	AC	16	9
44890	4	9414555	9414570	2	8	16	CA	15	7
44893	4	9429478	9429493	2	8	16	TG	17	6
44894	4	9431366	9431379	2	7	14	GT	15	3
44897	4	9436511	9436530	2	10	20	AC	13	4
44902	4	9442314	9442337	3	8	24	GAA	10	5
44949	4	9524302	9524317	2	8	16	GA	32	5
44951	4	9526033	9526054	2	11	22	TG	15	8
44962	4	9558985	9559014	2	15	30	TG	13	12
44976	4	9584877	9584900	3	8	24	GCA	22	5
44985	4	9596614	9596639	2	13	26	CA	18	10
44988	4	9599150	9599197	2	24	48	TG	11	12
44996	4	9608247	9608262	2	8	16	AC	26	3
44997	4	9608444	9608489	2	23	46	AC	8	12
45003	4	9612140	9612189	2	25	50	CA	7	9
45011	4	9656938	9656953	2	8	16	AC	18	8
45012	4	9660397	9660414	2	9	18	AG	29	4
45016	4	9674663	9674682	4	5	20	ATAA	25	10
45018	4	9676727	9676748	2	11	22	AG	10	8
45024	4	9717532	9717575	4	11	44	TCTT	11	6
45027	4	9725163	9725174	2	6	12	CT	18	3
45029	4	9725531	9725548	2	9	18	TC	14	5
45032	4	9729315	9729328	2	7	14	CA	29	3
45034	4	9730041	9730056	4	4	16	CTGT	24	4
45039	4	9742831	9742852	2	11	22	GT	23	7
45042	4	9756632	9756653	2	11	22	AG	7	7
45044	4	9766121	9766160	2	20	40	CA	9	7
45048	4	9784668	9784681	2	7	14	GT	21	5
45050	4	9792571	9792594	4	6	24	GGTA	27	4
45051	4	9792845	9792864	2	10	20	AC	19	12
45053	4	9819372	9819401	2	15	30	GA	8	7
45055	4	9826206	9826233	2	14	28	CA	12	11
45056	4	9830960	9830989	2	15	30	GT	18	9
45071	4	9853088	9853115	2	14	28	TG	15	9
45073	4	9857206	9857225	2	10	20	GT	21	9
45074	4	9857453	9857468	2	8	16	TG	30	6
45077	4	9859475	9859512	2	19	38	TG	10	10
45078	4	9859831	9859856	2	13	26	CA	17	10
45082	4	9867590	9867605	2	8	16	GT	13	7
45083	4	9867608	9867621	2	7	14	GT	16	7
45091	4	9875852	9875863	2	6	12	CA	24	4
45098	4	9886974	9887005	2	16	32	TG	8	10

45102	4	9895759	9895798	4	10	40	CACG	13	19
45104	4	9895974	9895987	2	7	14	CA	12	7
45105	4	9895989	9896004	4	4	16	GCAC	16	7
45108	4	9900080	9900097	2	9	18	CA	21	8
45109	4	9901926	9901941	2	8	16	TG	12	9
45110	4	9903408	9903421	2	7	14	TG	17	3
45119	4	9936465	9936478	2	7	14	AC	26	3
45121	4	9937697	9937716	2	10	20	TG	22	12
45126	4	9952491	9952520	2	15	30	CA	15	8
45129	4	9955580	9955603	2	12	24	CT	12	9
45132	4	9957291	9957302	2	6	12	AC	23	3
45142	4	9976957	9976998	2	21	42	GT	12	10
45151	4	9990371	9990403	3	11	33	ATA	8	7
45153	4	9994573	9994584	2	6	12	AT	7	5
45159	4	10008148	10008171	4	6	24	AAAT	23	8
45162	4	10008806	10008825	2	10	20	TG	17	6
45163	4	10008899	10008919	3	7	21	CAT	16	6
45165	4	10014089	10014100	2	6	12	GT	19	3
45167	4	10018653	10018676	2	12	24	TG	8	8
45169	4	10024262	10024275	2	7	14	GT	26	3
45178	4	10050283	10050312	2	15	30	GT	16	12
45179	4	10051468	10051479	2	6	12	AC	18	4
45197	4	10114864	10114878	3	5	15	TGC	25	4
45205	4	10156765	10156788	2	12	24	AC	21	8
45208	4	10158386	10158409	2	12	24	TG	11	12
45210	4	10163655	10163692	2	19	38	AC	14	12
45212	4	10169293	10169314	2	11	22	CA	18	4
45220	4	10192671	10192684	2	7	14	TG	27	4
45221	4	10195885	10195914	2	15	30	AC	15	13
45222	4	10197410	10197423	2	7	14	GT	8	3
45226	4	10201726	10201739	2	7	14	AC	17	6
45229	4	10203270	10203285	2	8	16	AT	29	3
45230	4	10203751	10203774	2	12	24	CA	8	5
45234	4	10208282	10208303	2	11	22	AC	9	8
45237	4	10214097	10214108	2	6	12	AC	18	4
45243	4	10226804	10226818	3	5	15	GCT	28	3
45244	4	10227157	10227178	2	11	22	AC	24	7
45245	4	10229785	10229796	2	6	12	TC	23	4
45246	4	10229797	10229808	2	6	12	AC	24	4
45248	4	10236147	10236170	2	12	24	CA	13	5
45250	4	10239079	10239094	2	8	16	GA	16	9
45252	4	10245562	10245609	2	24	48	CT	8	6
45255	4	10249629	10249649	3	7	21	GGA	6	3
45256	4	10250557	10250574	2	9	18	GT	10	7
45257	4	10257988	10258005	2	9	18	CA	13	4
45258	4	10258837	10258854	2	9	18	CA	23	6
45259	4	10260170	10260181	2	6	12	GT	20	4
45262	4	10260665	10260702	2	19	38	GT	14	15
45267	4	10268851	10268862	2	6	12	CA	19	3
45271	4	10278954	10278981	2	14	28	TG	18	6
45272	4	10279147	10279174	2	14	28	CA	16	9

45273	4	10280415	10280438	2	12	24	GA	12	7
45274	4	10280764	10280777	2	7	14	AG	23	3
45275	4	10282141	10282164	2	12	24	CA	9	6
45277	4	10284584	10284625	2	21	42	TG	9	8
45286	4	10293861	10293872	2	6	12	AC	26	3
45288	4	10295377	10295396	2	10	20	AC	30	8
45289	4	10295725	10295740	2	8	16	AC	20	9
45291	4	10296249	10296268	2	10	20	AC	20	5
45295	4	10296708	10296722	3	5	15	AGA	20	7
45296	4	10297085	10297126	2	21	42	TC	8	7
45299	4	10299323	10299340	2	9	18	CA	20	6
45301	4	10300941	10300952	2	6	12	TG	27	3
45312	4	10306152	10306171	4	5	20	CCAA	17	3
45317	4	10310316	10310331	2	8	16	CA	14	4
45324	4	10320214	10320231	2	9	18	CA	31	3
45326	4	10330372	10330383	2	6	12	CA	24	4
45327	4	10330515	10330538	2	12	24	AC	19	11
45328	4	10332446	10332475	2	15	30	TG	22	14
45329	4	10336625	10336645	3	7	21	AGA	22	3
45339	4	10364863	10364882	2	10	20	GT	21	6
45341	4	10367395	10367406	2	6	12	TG	21	4
45342	4	10367590	10367603	2	7	14	AC	32	3
45353	4	10376814	10376867	2	27	54	GT	8	8
45354	4	10380262	10380276	3	5	15	AAG	10	3
45357	4	10403688	10403709	2	11	22	CA	17	4
45369	4	10446163	10446226	4	16	64	AAGA	6	9
45371	4	10447968	10447991	2	12	24	AC	19	10
45374	4	10449891	10449902	2	6	12	CA	31	3
45375	4	10450927	10450950	4	6	24	TGTA	11	3
45376	4	10454952	10454973	2	11	22	AG	23	4
45377	4	10459705	10459730	2	13	26	CA	19	7
45378	4	10462112	10462135	2	12	24	CA	22	8
45379	4	10468015	10468040	2	13	26	GT	14	12
45380	4	10470401	10470412	2	6	12	GA	31	3
45388	4	10473803	10473824	2	11	22	AC	26	4
45391	4	10477975	10477998	3	8	24	CCT	20	6
45392	4	10478255	10478270	2	8	16	AC	19	7
45400	4	10485111	10485124	2	7	14	AG	25	4
45402	4	10490235	10490248	2	7	14	CT	28	4
45403	4	10493864	10493883	2	10	20	AC	13	7
45407	4	10499897	10499912	2	8	16	TG	20	4
45408	4	10500043	10500054	2	6	12	AT	6	3
45415	4	10522146	10522169	4	6	24	AAAC	24	5
45416	4	10522817	10522830	2	7	14	CA	25	10
45417	4	10523408	10523427	2	10	20	GT	17	6
45419	4	10526097	10526112	4	4	16	TGTC	20	5
45420	4	10526167	10526188	2	11	22	TG	23	7
45421	4	10528174	10528187	2	7	14	TG	20	17
45426	4	10540113	10540160	2	24	48	CA	11	14
45429	4	10548354	10548393	2	20	40	CA	8	10
45430	4	10549395	10549412	2	9	18	AC	21	12

45431	4	10559157	10559172	2	8	16	GT	20	5
45435	4	10575717	10575732	2	8	16	AC	22	3
45444	4	10613901	10613912	2	6	12	CA	20	3
45447	4	10620752	10620771	4	5	20	ACAA	31	6
45448	4	10628846	10628861	4	4	16	TAAA	21	4
45449	4	10637724	10637751	4	7	28	TCTA	19	3
45453	4	10655637	10655674	2	19	38	TG	8	10
45457	4	10672874	10672887	2	7	14	CT	22	7
45459	4	10689916	10689949	2	17	34	CA	11	7
45462	4	10708799	10708824	2	13	26	TG	13	8
45471	4	10737304	10737319	2	8	16	TG	9	8
45480	4	10752964	10752983	2	10	20	CA	14	10
45482	4	10755746	10755757	2	6	12	CA	24	5
45483	4	10755760	10755771	2	6	12	CA	26	5
45485	4	10758292	10758303	2	6	12	TG	24	3
45489	4	10763204	10763223	2	10	20	CT	7	3
45492	4	10764817	10764840	3	8	24	TCC	17	7
45494	4	10768088	10768115	4	7	28	AAAG	11	8
45501	4	10786751	10786764	2	7	14	CA	12	5
45504	4	10793978	10794001	2	12	24	GT	14	5
45506	4	10795074	10795088	3	5	15	CCA	30	4
45512	4	10826740	10826760	3	7	21	TGC	31	3
45518	4	10837897	10837912	4	4	16	TTAT	18	3
45524	4	10858910	10858925	2	8	16	TG	28	3
45525	4	10866056	10866075	2	10	20	TG	19	8
45528	4	10871330	10871347	2	9	18	GT	15	10
45533	4	10879704	10879715	2	6	12	AC	10	7
45534	4	10879754	10879773	4	5	20	AAAT	27	5
45535	4	10879984	10879998	3	5	15	GAA	20	12
45536	4	10880564	10880579	4	4	16	TTGT	17	4
45537	4	10880714	10880729	4	4	16	ATTC	28	5
45541	4	10885948	10885979	2	16	32	GT	8	10
45543	4	10892664	10892679	2	8	16	CA	22	4
45548	4	10900141	10900156	2	8	16	CA	24	5
45551	4	10928275	10928318	2	22	44	AG	6	7
45558	4	10948879	10948924	2	23	46	TG	15	12
45566	4	10975155	10975168	2	7	14	TG	19	4
45575	4	10989032	10989045	2	7	14	TG	19	6
45576	4	10989047	10989062	2	8	16	GT	25	5
45580	4	10997831	10997846	4	4	16	ACTT	29	3
45588	4	11038845	11038870	2	13	26	TC	8	7
45589	4	11040241	11040258	3	6	18	GGA	29	3
45593	4	11044464	11044477	2	7	14	GT	29	4
45598	4	11052299	11052313	3	5	15	GAT	28	3
45600	4	11055167	11055182	4	4	16	TTCC	25	3
45604	4	11068396	11068411	2	8	16	GT	29	9
45606	4	11072278	11072291	2	7	14	GT	22	3
45608	4	11074166	11074189	2	12	24	TG	10	8
45621	4	11097584	11097601	2	9	18	GT	21	6
45642	4	11133283	11133308	2	13	26	TG	6	9
45647	4	11151149	11151176	2	14	28	AC	21	7

45653	4	11159312	11159351	2	20	40	CA	9	9
45658	4	11166048	11166073	2	13	26	CA	21	5
45662	4	11180904	11180931	4	7	28	TATC	24	3
45663	4	11181608	11181623	4	4	16	GGAA	22	5
45664	4	11184434	11184463	3	10	30	TCC	17	4
45667	4	11186302	11186323	2	11	22	GT	11	9
45668	4	11186728	11186755	2	14	28	CA	15	8
45670	4	11186932	11186971	4	10	40	ATCT	7	10
45680	4	11227075	11227088	2	7	14	TG	21	6
45682	4	11230633	11230654	2	11	22	TC	12	4
45702	4	11331659	11331704	2	23	46	TG	9	11
45704	4	11342684	11342699	4	4	16	AAAT	28	4
45709	4	11365928	11365948	3	7	21	GCA	20	6
45710	4	11366526	11366539	2	7	14	TC	8	9
45721	4	11387659	11387682	2	12	24	CT	16	8
45723	4	11391300	11391315	2	8	16	TG	27	4
45724	4	11392984	11393001	2	9	18	AC	12	5
45725	4	11393838	11393875	2	19	38	TG	14	8
45726	4	11393979	11393994	4	4	16	CAGG	26	4
45728	4	11397113	11397150	2	19	38	TG	10	9
45734	4	11404748	11404765	3	6	18	CCT	22	5
45738	4	11405749	11405768	2	10	20	GA	7	4
45743	4	11411498	11411521	2	12	24	CA	25	6
45744	4	11412193	11412216	2	12	24	GT	13	8
45745	4	11414474	11414487	2	7	14	GT	18	4
45746	4	11414488	11414499	2	6	12	GA	19	3
45748	4	11416600	11416623	2	12	24	CA	19	3
45749	4	11416991	11417006	2	8	16	AC	18	3
45750	4	11417009	11417022	2	7	14	AC	25	8
45753	4	11423569	11423596	2	14	28	GT	11	8
45756	4	11425210	11425227	2	9	18	CA	18	4
45759	4	11434255	11434278	2	12	24	TG	12	7
45760	4	11434719	11434739	3	7	21	CAG	17	8
45767	4	11439342	11439383	2	21	42	GT	20	13
45771	4	11446210	11446235	2	13	26	CA	22	8
45775	4	11450669	11450688	2	10	20	CA	14	8
45777	4	11451799	11451812	2	7	14	AC	27	4
45778	4	11452154	11452167	2	7	14	TG	18	3
45782	4	11455751	11455770	2	10	20	AC	12	13
45785	4	11457128	11457139	2	6	12	AC	26	3
45786	4	11459551	11459586	4	9	36	AGAA	15	8
45797	4	11480442	11480473	2	16	32	TG	13	10
45801	4	11487548	11487561	2	7	14	CA	25	3
45805	4	11494635	11494646	2	6	12	AC	25	4
45806	4	11502970	11502987	2	9	18	TG	22	4
45808	4	11509641	11509664	3	8	24	GAT	23	10
45809	4	11516162	11516200	3	13	39	TCT	24	18
45811	4	11521286	11521299	2	7	14	TG	6	7
45812	4	11521438	11521469	2	16	32	CA	6	8
45814	4	11522191	11522206	2	8	16	GT	32	4
45816	4	11524685	11524710	2	13	26	TG	18	7

45819	4	11529975	11530008	2	17	34	TG	8	6
45820	4	11550160	11550183	2	12	24	CA	11	9
45821	4	11550598	11550612	3	5	15	GCA	21	11
45822	4	11550613	11550633	3	7	21	GGA	11	12
45824	4	11550742	11550755	2	7	14	AC	21	7
45825	4	11551199	11551226	2	14	28	GT	25	14
45826	4	11553192	11553203	2	6	12	AC	32	4
45827	4	11553382	11553393	2	6	12	GT	20	15
45828	4	11555079	11555098	2	10	20	TG	10	8
45829	4	11555233	11555246	2	7	14	TG	21	4
45834	4	11555563	11555600	2	19	38	GT	15	11
45836	4	11556153	11556166	2	7	14	TC	25	5
45840	4	11560974	11560997	3	8	24	AAG	21	8
45841	4	11565314	11565363	2	25	50	CA	8	12
45842	4	11565879	11565902	3	8	24	GGA	16	4
45843	4	11569419	11569442	3	8	24	AGC	25	5
45845	4	11570045	11570068	2	12	24	TG	11	14
45846	4	11570173	11570190	2	9	18	CA	23	7
45849	4	11570302	11570313	2	6	12	AC	11	6
45853	4	11578552	11578581	2	15	30	TG	14	11
45856	4	11582263	11582286	2	12	24	GT	21	5
45858	4	11583184	11583205	2	11	22	AC	15	10
45859	4	11583255	11583270	2	8	16	AG	19	3
45860	4	11586804	11586831	2	14	28	CT	21	10
45862	4	11588419	11588442	4	6	24	ACAG	26	3
45863	4	11589516	11589547	2	16	32	GT	14	11
45864	4	11589678	11589693	2	8	16	GT	19	8
45874	4	11597211	11597226	2	8	16	CA	22	4
45876	4	11597538	11597561	2	12	24	TG	18	7
45877	4	11599094	11599119	2	13	26	TG	16	3
45879	4	11599275	11599295	3	7	21	GAG	12	3
45881	4	11599584	11599601	2	9	18	CT	22	4
45883	4	11600168	11600185	3	6	18	AGG	23	5
45885	4	11602912	11602927	2	8	16	GT	22	4
45888	4	11619754	11619768	3	5	15	AAC	29	3
45892	4	11627172	11627207	2	18	36	AC	18	12
45893	4	11628868	11628881	2	7	14	TC	27	3
45894	4	11632837	11632848	2	6	12	TG	27	5
45896	4	11648085	11648096	2	6	12	TG	29	4
45903	4	11664096	11664117	2	11	22	AG	24	4
45908	4	11672401	11672412	2	6	12	TG	25	3
45909	4	11674777	11674792	2	8	16	TC	21	4
45920	4	11691294	11691305	2	6	12	CA	23	3
45922	4	11691643	11691672	2	15	30	CA	8	12
45928	4	11705235	11705266	2	16	32	AC	14	6
45931	4	11715907	11715927	3	7	21	TAG	11	3
45936	4	11729967	11729982	2	8	16	AT	18	5
45937	4	11735078	11735107	2	15	30	AC	14	10
45939	4	11744281	11744296	2	8	16	TG	24	7
45949	4	11764697	11764712	2	8	16	CA	24	6
45952	4	11772003	11772017	3	5	15	CAT	22	4

45957	4	11790327	11790372	2	23	46	TG	11	13
45960	4	11802448	11802459	2	6	12	GA	24	4
45963	4	11810823	11810836	2	7	14	CA	25	6
45966	4	11817694	11817707	2	7	14	TG	31	3
45968	4	11820746	11820795	2	25	50	AG	8	7
45969	4	11821901	11821934	2	17	34	GT	9	5
45970	4	11823800	11823821	2	11	22	TG	22	4
45971	4	11825109	11825130	2	11	22	GA	24	4
45974	4	11830116	11830141	2	13	26	CA	14	8
45977	4	11834479	11834492	2	7	14	TG	17	3
45978	4	11835693	11835710	2	9	18	CA	25	4
45979	4	11837210	11837225	2	8	16	AC	24	3
45982	4	11841903	11841926	3	8	24	TGA	32	3
45996	4	11865638	11865665	2	14	28	TG	8	8
45997	4	11867005	11867022	2	9	18	AC	12	8
46000	4	11869158	11869185	2	14	28	GT	13	8
46002	4	11870049	11870068	2	10	20	GT	19	9
46009	4	11875645	11875670	2	13	26	GT	8	11
46012	4	11886294	11886309	2	8	16	TC	21	3
46013	4	11892087	11892112	2	13	26	AC	10	6
46016	4	11895870	11895895	2	13	26	GT	26	6
46018	4	11898446	11898459	2	7	14	TG	12	6
46022	4	11901114	11901167	2	27	54	CA	12	12
46025	4	11902290	11902309	2	10	20	CA	14	3
46027	4	11902346	11902366	3	7	21	GCT	18	3
46028	4	11902398	11902421	2	12	24	CA	20	6
46032	4	11909040	11909067	2	14	28	AC	23	9
46037	4	11916686	11916709	2	12	24	GT	16	8
46041	4	11918709	11918720	2	6	12	TC	25	5
46044	4	11925930	11925949	2	10	20	GT	20	7
46046	4	11926743	11926754	2	6	12	CA	23	6
46051	4	11933017	11933054	2	19	38	TG	13	9
46054	4	11935669	11935684	2	8	16	GT	17	6
46058	4	11938198	11938223	2	13	26	AC	15	8
46059	4	11938361	11938376	2	8	16	CA	20	4
46062	4	11944849	11944864	4	4	16	AAAC	26	3
46063	4	11948785	11948816	2	16	32	AC	17	11
46080	4	11985700	11985717	3	6	18	ATC	23	4
46083	4	11993033	11993056	2	12	24	AC	18	11
46084	4	11995723	11995740	3	6	18	TCA	25	4
46087	4	12014857	12014892	4	9	36	TATC	12	11
46089	4	12022484	12022501	2	9	18	TG	21	3
46092	4	12025643	12025678	2	18	36	CA	7	11
46093	4	12026556	12026567	2	6	12	TG	20	5
46100	4	12040760	12040777	2	9	18	AC	23	15
46104	4	12051138	12051161	2	12	24	TG	20	6
46105	4	12054211	12054240	3	10	30	GAA	23	9
46107	4	12055971	12055984	2	7	14	AC	20	7
46108	4	12056007	12056020	2	7	14	AC	21	7
46111	4	12060295	12060336	2	21	42	TG	16	11
46112	4	12061595	12061622	2	14	28	AC	17	11

46116	4	12068332	12068345	2	7	14	GT	17	3
46124	4	12079109	12079132	2	12	24	TC	23	9
46125	4	12082129	12082140	2	6	12	CA	26	5
46128	4	12084751	12084765	3	5	15	TTC	31	3
46129	4	12087320	12087335	2	8	16	AC	14	4
46130	4	12087384	12087403	2	10	20	AC	9	3
46131	4	12088265	12088284	4	5	20	AATT	12	3
46135	4	12092722	12092737	2	8	16	GT	22	9
46137	4	12096032	12096043	2	6	12	TG	29	4
46138	4	12101186	12101201	2	8	16	CA	15	11
46142	4	12104245	12104264	2	10	20	AC	22	10
46143	4	12106504	12106527	2	12	24	CA	16	9
46144	4	12106685	12106698	2	7	14	AC	27	4
46145	4	12107000	12107023	2	12	24	AC	21	8
46146	4	12107095	12107114	2	10	20	GT	19	10
46150	4	12114297	12114310	2	7	14	AC	24	9
46151	4	12116825	12116860	2	18	36	CA	9	11
46155	4	12122655	12122674	2	10	20	GT	23	5
46167	4	12132014	12132029	2	8	16	GT	24	4
46168	4	12133683	12133706	2	12	24	CA	16	7
46169	4	12133878	12133893	2	8	16	AC	26	3
46181	4	12151741	12151780	4	10	40	CAAT	25	8
46188	4	12159087	12159102	2	8	16	TC	27	4
46193	4	12163639	12163680	2	21	42	AC	19	17
46194	4	12163839	12163856	2	9	18	AC	21	4
46195	4	12164017	12164042	2	13	26	TG	17	8
46197	4	12164825	12164836	2	6	12	TA	31	3
46207	4	12176831	12176850	2	10	20	CA	21	9
46208	4	12177019	12177054	2	18	36	GT	16	6
46214	4	12184864	12184887	2	12	24	GT	24	13
46215	4	12186648	12186695	2	24	48	TG	10	11
46217	4	12188861	12188882	2	11	22	TG	13	8
46218	4	12190481	12190536	2	28	56	TG	13	11
46219	4	12190643	12190670	2	14	28	AC	16	12
46220	4	12192408	12192421	2	7	14	TG	28	3
46221	4	12193210	12193229	2	10	20	GA	17	6
46223	4	12194823	12194844	2	11	22	CA	14	5
46224	4	12197433	12197462	2	15	30	AC	8	5
46226	4	12206350	12206379	2	15	30	CA	16	11
46227	4	12206590	12206601	2	6	12	TG	31	3
46228	4	12206756	12206769	2	7	14	GT	28	4
46234	4	12211248	12211285	2	19	38	GT	8	8
46235	4	12211387	12211440	2	27	54	TG	8	10
46239	4	12223135	12223182	2	24	48	CA	8	8
46240	4	12223214	12223227	2	7	14	AC	24	4
46243	4	12223794	12223805	2	6	12	CA	24	6
46244	4	12224365	12224378	2	7	14	AT	9	10
46245	4	12224379	12224392	2	7	14	AG	12	9
46253	4	12228167	12228202	2	18	36	TG	7	9
46270	4	12242022	12242035	2	7	14	TG	22	10
46272	4	12249102	12249125	2	12	24	GA	12	6

46273	4	12249419	12249448	2	15	30	TC	21	11
46283	4	12261986	12262005	2	10	20	GT	9	7
46285	4	12263023	12263048	2	13	26	CA	25	10
46289	4	12266902	12266919	3	6	18	GGA	23	8
46295	4	12277543	12277564	2	11	22	CA	16	7
46297	4	12279449	12279493	3	15	45	TGA	17	11
46298	4	12279938	12279965	4	7	28	AGTT	23	4
46302	4	12288847	12288874	2	14	28	AC	7	3
46305	4	12291457	12291482	2	13	26	CA	17	13
46307	4	12292339	12292354	2	8	16	AG	24	4
46309	4	12304281	12304304	2	12	24	CA	19	5
46313	4	12309560	12309583	2	12	24	CA	18	5
46316	4	12310865	12310890	2	13	26	TG	22	11
46317	4	12310928	12310941	2	7	14	TG	26	3
46319	4	12314743	12314780	2	19	38	AC	15	12
46320	4	12316151	12316172	2	11	22	AC	16	9
46326	4	12322520	12322535	2	8	16	CA	24	8
46332	4	12336765	12336786	2	11	22	CT	13	5
46343	4	12365144	12365161	3	6	18	CAG	14	4
46346	4	12368424	12368437	2	7	14	GT	22	5
46347	4	12368505	12368522	2	9	18	CT	16	6
46350	4	12370407	12370421	3	5	15	AAT	7	6
46351	4	12371170	12371201	2	16	32	TG	14	13
46352	4	12373450	12373475	2	13	26	CT	18	7
46354	4	12388146	12388159	2	7	14	AC	11	9
46355	4	12388160	12388173	2	7	14	CA	13	8
46358	4	12396278	12396293	2	8	16	CA	25	6
46360	4	12400665	12400680	2	8	16	GT	6	7
46365	4	12404782	12404799	2	9	18	CA	17	6
46372	4	12407606	12407629	2	12	24	CA	8	7
46378	4	12410837	12410850	2	7	14	CG	24	14
46379	4	12410851	12410864	2	7	14	CA	24	15
46385	4	12418318	12418349	2	16	32	AC	15	10
46386	4	12419209	12419228	2	10	20	GT	14	9
46394	4	12423215	12423242	2	14	28	GT	14	6
46398	4	12427233	12427258	2	13	26	TG	13	5
46402	4	12432307	12432320	2	7	14	GT	24	4
46404	4	12435456	12435473	3	6	18	AAC	12	3
46407	4	12438013	12438052	2	20	40	AC	11	11
46409	4	12447524	12447563	2	20	40	TG	6	7
46411	4	12451303	12451324	2	11	22	AC	16	9
46412	4	12452357	12452368	2	6	12	GT	27	5
46414	4	12452591	12452618	2	14	28	AC	12	7
46418	4	12459962	12459983	2	11	22	CA	7	8
46419	4	12466164	12466179	2	8	16	GA	18	6
46422	4	12471456	12471467	2	6	12	CA	30	4
46423	4	12474989	12475000	2	6	12	CA	16	6
46424	4	12478392	12478417	2	13	26	TG	12	8
46426	4	12487973	12487984	2	6	12	GT	24	4
46441	4	12525281	12525324	2	22	44	TG	18	13
46444	4	12526822	12526837	4	4	16	CTCA	27	3

46448	4	12533930	12533955	2	13	26	CA	14	8
46450	4	12539819	12539832	2	7	14	AG	25	3
46451	4	12543100	12543117	3	6	18	GCT	27	3
46453	4	12543620	12543631	2	6	12	GT	31	5
46454	4	12543702	12543723	2	11	22	CA	19	7
46461	4	12554430	12554451	2	11	22	TG	10	10
46464	4	12567172	12567192	3	7	21	GAA	28	4
46466	4	12576576	12576589	2	7	14	TG	16	7
46474	4	12593539	12593550	2	6	12	TC	18	8
46478	4	12598135	12598154	2	10	20	TG	22	8
46479	4	12600567	12600584	2	9	18	TG	22	9
46480	4	12600856	12600879	2	12	24	TG	14	7
46481	4	12601864	12601877	2	7	14	GT	23	7
46482	4	12603537	12603558	2	11	22	CA	16	6
46484	4	12604037	12604052	2	8	16	GT	14	7
46486	4	12604469	12604492	2	12	24	AC	15	6
46487	4	12607236	12607249	2	7	14	GT	22	5
46488	4	12607405	12607444	2	20	40	CA	15	13
46500	4	12614353	12614366	2	7	14	AG	25	6
46503	4	12615837	12615856	2	10	20	GT	19	3
46505	4	12616408	12616419	2	6	12	GT	28	4
46506	4	12616962	12616977	2	8	16	GT	22	4
46507	4	12619340	12619357	3	6	18	CTG	25	3
46513	4	12625618	12625633	2	8	16	GT	16	11
46529	4	12638028	12638043	2	8	16	GT	24	7
46531	4	12640085	12640100	2	8	16	AC	21	4
46539	4	12645794	12645823	2	15	30	GT	13	8
46542	4	12646110	12646160	3	17	51	CAT	22	7
46543	4	12647143	12647170	2	14	28	AC	14	11
46549	4	12649483	12649514	2	16	32	CA	11	15
46551	4	12651780	12651793	2	7	14	TG	19	6
46553	4	12652701	12652718	2	9	18	CA	15	9
46554	4	12653312	12653325	2	7	14	TG	20	7
46555	4	12653472	12653501	3	10	30	GGA	9	9
46556	4	12653621	12653635	3	5	15	GCG	21	7
46557	4	12653954	12653971	2	9	18	AC	18	10
46558	4	12654083	12654114	2	16	32	GT	14	11
46559	4	12654278	12654307	2	15	30	CA	15	16
46560	4	12655756	12655771	2	8	16	TG	18	6
46561	4	12655935	12655966	2	16	32	TG	19	8
46565	4	12658540	12658551	2	6	12	AC	30	4
46566	4	12658857	12658870	2	7	14	CA	21	6
46571	4	12660825	12660836	2	6	12	AC	22	4
46572	4	12661222	12661237	2	8	16	CA	25	3
46573	4	12661954	12661974	3	7	21	AAT	19	5
46576	4	12663463	12663486	2	12	24	AC	17	10
46577	4	12665007	12665033	3	9	27	CTC	31	3
46578	4	12665104	12665117	2	7	14	AC	25	4
46580	4	12667518	12667541	2	12	24	TG	16	12
46581	4	12667663	12667674	2	6	12	GT	27	4
46586	4	12668758	12668775	2	9	18	CA	21	6

46587	4	12668931	12668946	2	8	16	AC	27	4
46595	4	12673482	12673511	2	15	30	AC	12	9
46597	4	12675258	12675269	2	6	12	AT	31	4
46601	4	12677137	12677156	2	10	20	AC	25	7
46603	4	12677519	12677536	2	9	18	CA	15	4
46608	4	12679359	12679376	2	9	18	CA	24	12
46619	4	12686025	12686038	2	7	14	AC	18	3
46620	4	12687277	12687314	2	19	38	CA	17	10
46621	4	12687603	12687626	2	12	24	AC	9	9
46623	4	12688597	12688612	2	8	16	CA	27	13
46624	4	12688732	12688745	2	7	14	CA	22	8
46625	4	12688798	12688815	2	9	18	AC	18	9
46626	4	12688934	12688963	2	15	30	CA	18	7
46628	4	12689461	12689476	2	8	16	AC	16	9
46629	4	12690034	12690049	2	8	16	CA	26	6
46632	4	12691328	12691345	2	9	18	AC	25	9
46634	4	12691790	12691810	3	7	21	CTT	21	7
46635	4	12692255	12692272	2	9	18	TC	11	12
46636	4	12692659	12692670	2	6	12	GT	20	15
46637	4	12692845	12692862	2	9	18	AC	23	17
46638	4	12694550	12694563	2	7	14	AG	34	8
46660	4	12735878	12735889	2	6	12	CA	23	3
46661	4	12735892	12735903	2	6	12	CA	26	6
46664	4	12743292	12743311	4	5	20	CAGA	28	7
46665	4	12751070	12751081	2	6	12	AC	31	3
46667	4	12755349	12755372	2	12	24	AC	22	8
46669	4	12757558	12757573	4	4	16	AATA	29	11
46676	4	12771348	12771379	2	16	32	TG	14	8
46677	4	12771507	12771536	2	15	30	CA	15	12
46678	4	12771634	12771649	2	8	16	AG	27	6
46679	4	12772202	12772229	2	14	28	GT	12	12
46682	4	12774272	12774321	2	25	50	TG	10	9
46683	4	12776106	12776119	2	7	14	GT	21	4
46691	4	12777442	12777463	2	11	22	GT	25	6
46692	4	12779601	12779632	2	16	32	AC	18	9
46694	4	12782505	12782540	2	18	36	GA	9	11
46696	4	12783981	12784012	2	16	32	GT	16	7
46699	4	12787601	12787642	2	21	42	CA	6	5
46701	4	12790050	12790093	2	22	44	CA	14	12
46708	4	12797758	12797784	3	9	27	TCT	18	4
46711	4	12799063	12799077	3	5	15	CTT	14	3
46714	4	12803969	12803998	2	15	30	CA	10	9
46717	4	12804741	12804758	2	9	18	CA	24	3
46720	4	12806639	12806656	3	6	18	TGC	29	3
46721	4	12807112	12807129	3	6	18	GAG	15	6
46722	4	12807394	12807411	2	9	18	AC	21	5
46724	4	12808009	12808042	2	17	34	CT	12	8
46725	4	12808204	12808243	2	20	40	GT	6	9
46726	4	12809066	12809079	2	7	14	CA	24	4
46727	4	12811735	12811762	2	14	28	AC	21	9
46728	4	12813796	12813816	3	7	21	TGT	33	3

46734	4	12814809	12814824	2	8	16	GT	17	15
46746	4	12827411	12827432	2	11	22	TG	13	5
46747	4	12830639	12830662	2	12	24	TG	11	13
46752	4	12836166	12836183	2	9	18	AC	20	6
46755	4	12839570	12839595	2	13	26	CA	8	5
46758	4	12841151	12841166	2	8	16	TG	24	4
46759	4	12841664	12841697	2	17	34	CA	19	8
46760	4	12843605	12843620	2	8	16	AC	10	9
46762	4	12844007	12844028	2	11	22	AC	20	9
46764	4	12845828	12845863	2	18	36	AC	10	7
46768	4	12849268	12849293	2	13	26	GT	19	10
46775	4	12852957	12852988	2	16	32	TG	16	11
46777	4	12854901	12854918	2	9	18	CA	18	6
46778	4	12855446	12855463	2	9	18	CT	14	6
46780	4	12855959	12855986	2	14	28	CA	18	7
46781	4	12857257	12857278	2	11	22	CA	16	6
46784	4	12857745	12857762	2	9	18	AG	19	5
46786	4	12862944	12862961	2	9	18	GT	16	9
46787	4	12864199	12864219	3	7	21	TCC	18	4
46789	4	12865449	12865472	2	12	24	CA	27	7
46792	4	12869795	12869812	2	9	18	CA	21	8
46794	4	12875395	12875414	2	10	20	CA	17	6
46796	4	12876035	12876074	2	20	40	CA	10	13
46800	4	12880720	12880737	3	6	18	CTG	24	6
46808	4	12890778	12890799	2	11	22	CA	18	8
46809	4	12891265	12891296	4	8	32	TTCT	21	14
46810	4	12893435	12893452	2	9	18	GT	6	7
46813	4	12897574	12897585	2	6	12	CA	19	7
46822	4	12902371	12902404	2	17	34	AC	22	17
46824	4	12904836	12904853	2	9	18	GT	21	5
46829	4	12916445	12916480	2	18	36	GT	19	10
46830	4	12919833	12919846	2	7	14	GT	26	5
46834	4	12922508	12922539	2	16	32	CA	15	10
46841	4	12932134	12932147	2	7	14	CA	25	8
46843	4	12932337	12932364	2	14	28	CA	15	10
46849	4	12947052	12947063	2	6	12	CA	24	4
46851	4	12956716	12956729	2	7	14	GT	18	15
46854	4	12958821	12958882	2	31	62	GA	11	11
46855	4	12960826	12960843	2	9	18	GA	25	4
46858	4	12966246	12966261	2	8	16	AC	19	8
46859	4	12966397	12966424	2	14	28	AC	14	8
46864	4	12976323	12976334	2	6	12	GT	29	4
46867	4	12993350	12993373	2	12	24	CT	13	6
46874	4	12998648	12998661	2	7	14	AC	21	3
46881	4	13024220	13024231	2	6	12	AT	24	3
46885	4	13030147	13030178	2	16	32	AC	7	11
46886	4	13031057	13031074	2	9	18	TG	24	11
46887	4	13033770	13033797	2	14	28	GT	23	11
46888	4	13034660	13034681	2	11	22	AC	19	8
46889	4	13036792	13036819	2	14	28	TG	18	9
46890	4	13036981	13036992	2	6	12	TG	25	8

46891	4	13037982	13038007	2	13	26	AC	14	16
46894	4	13038595	13038616	2	11	22	CA	17	9
46895	4	13038653	13038670	2	9	18	AC	19	10
46896	4	13039104	13039130	3	9	27	CCT	23	8
46898	4	13048630	13048645	2	8	16	TG	20	3
46900	4	13049748	13049765	2	9	18	TG	14	10
46901	4	13051424	13051447	2	12	24	TG	18	9
46904	4	13058380	13058395	2	8	16	GT	9	4
46916	4	13080247	13080264	2	9	18	AC	19	7
46924	4	13103509	13103520	2	6	12	CA	7	9
46925	4	13103807	13103822	2	8	16	CA	19	7
46927	4	13104683	13104702	2	10	20	CA	15	9
46929	4	13106774	13106787	2	7	14	AC	22	6
46932	4	13111616	13111635	2	10	20	TG	11	11
46933	4	13113313	13113340	2	14	28	CA	21	5
46935	4	13114590	13114617	2	14	28	TG	18	11
46936	4	13114845	13114866	2	11	22	GT	17	9
46937	4	13114991	13115010	2	10	20	GT	19	11
46939	4	13117193	13117246	2	27	54	TG	10	14
46942	4	13122839	13122858	2	10	20	TG	27	10
46943	4	13123079	13123098	2	10	20	TG	17	12
46946	4	13125360	13125381	2	11	22	CT	17	6
46950	4	13129128	13129157	2	15	30	GT	7	7
46951	4	13129555	13129570	2	8	16	GA	16	3
46955	4	13134887	13134910	3	8	24	CAG	18	5
46961	4	13145603	13145632	2	15	30	GT	12	14
46963	4	13145788	13145809	2	11	22	TG	19	7
46964	4	13146103	13146114	2	6	12	GT	19	12
46965	4	13146117	13146132	2	8	16	GT	15	11
46966	4	13148037	13148052	2	8	16	TG	25	9
46968	4	13149576	13149601	2	13	26	GT	17	6
46969	4	13150118	13150137	4	5	20	GACT	29	5
46970	4	13150219	13150240	2	11	22	GT	15	6
46971	4	13154424	13154441	2	9	18	GT	17	7
46974	4	13158974	13158989	4	4	16	TACT	29	5
46977	4	13164553	13164574	2	11	22	AC	21	6
46983	4	13180083	13180112	2	15	30	TG	12	12
46988	4	13186999	13187016	2	9	18	GT	21	5
46989	4	13187543	13187564	2	11	22	GT	16	9
46990	4	13194177	13194194	2	9	18	TG	25	6
46991	4	13195799	13195824	2	13	26	TG	18	10
46992	4	13195942	13195955	2	7	14	AC	27	3
46993	4	13198037	13198054	3	6	18	TCA	23	3
46997	4	13204758	13204787	3	10	30	ATC	21	7
46999	4	13211699	13211710	2	6	12	AT	8	4
47000	4	13212766	13212785	2	10	20	CA	24	8
47002	4	13218073	13218108	4	9	36	AGAT	22	8
47004	4	13219119	13219142	2	12	24	GT	23	10
47005	4	13219581	13219602	2	11	22	AC	17	8
47006	4	13220470	13220481	2	6	12	TG	29	3
47007	4	13225587	13225608	2	11	22	TG	16	6

47008	4	13225697	13225716	2	10	20	TG	20	4
47015	4	13240503	13240514	2	6	12	AG	23	3
47016	4	13240628	13240641	2	7	14	CT	25	3
47017	4	13242839	13242856	2	9	18	AC	20	7
47018	4	13243830	13243873	2	22	44	GT	17	15
47021	4	13244857	13244874	2	9	18	AC	15	8
47025	4	13245143	13245158	2	8	16	CT	21	5
47031	4	13268435	13268454	2	10	20	TG	26	15
47034	4	13271161	13271188	2	14	28	TG	13	11
47038	4	13284557	13284570	2	7	14	CA	34	3
47039	4	13288015	13288030	2	8	16	GT	21	3
47040	4	13288123	13288146	2	12	24	GT	19	5
47048	4	13303487	13303522	2	18	36	CT	15	12
47050	4	13308979	13309008	2	15	30	CA	15	18
47051	4	13311028	13311045	2	9	18	CA	30	9
47054	4	13315455	13315475	3	7	21	TTG	29	3
47055	4	13316252	13316267	2	8	16	CT	20	6
47066	4	13340049	13340066	2	9	18	GT	14	6
47067	4	13340136	13340147	2	6	12	GA	27	7
47068	4	13340150	13340161	2	6	12	GA	23	4
47069	4	13340631	13340654	4	6	24	TTCT	6	7
47076	4	13342975	13343002	2	14	28	GT	10	6
47077	4	13343827	13343856	2	15	30	GT	12	12
47081	4	13350193	13350206	2	7	14	CA	23	5
47084	4	13358936	13358963	4	7	28	ATAG	22	6
47092	4	13382202	13382216	3	5	15	TCC	22	5
47101	4	13398139	13398152	2	7	14	AC	19	5
47103	4	13400451	13400466	4	4	16	AATT	12	4
47105	4	13401286	13401311	2	13	26	CA	18	13
47106	4	13401885	13401900	2	8	16	TG	14	12
47107	4	13402015	13402050	2	18	36	GT	20	12
47112	4	13403405	13403418	2	7	14	CA	13	3
47116	4	13411439	13411464	2	13	26	AC	14	11
47120	4	13433042	13433061	2	10	20	GT	13	9
47121	4	13434371	13434394	2	12	24	TG	20	10
47125	4	13440718	13440751	2	17	34	AC	20	13
47129	4	13446339	13446352	2	7	14	TG	25	3
47140	4	13459720	13459737	2	9	18	TG	21	6
47141	4	13459865	13459880	2	8	16	AC	25	7
47143	4	13462907	13462922	2	8	16	GT	20	5
47144	4	13465195	13465226	2	16	32	GT	19	10
47152	4	13487615	13487632	3	6	18	TCC	33	3
47156	4	13504909	13504920	2	6	12	AC	27	4
47162	4	13518557	13518574	2	9	18	GT	16	7
47167	4	13527676	13527687	2	6	12	CT	28	4
47170	4	13535845	13535876	2	16	32	TG	21	12
47177	4	13548620	13548657	2	19	38	TG	13	13
47182	4	13564384	13564395	2	6	12	AT	28	3
47183	4	13571597	13571618	2	11	22	AC	8	10
47185	4	13578327	13578344	2	9	18	TG	19	10
47186	4	13578536	13578567	2	16	32	GT	11	11

47195	4	13591176	13591199	2	12	24	GT	15	7
47196	4	13591344	13591373	2	15	30	TG	10	6
47198	4	13593322	13593357	2	18	36	CA	11	5
47199	4	13594410	13594435	2	13	26	AC	8	10
47203	4	13598617	13598646	2	15	30	TG	15	9
47208	4	13606608	13606635	2	14	28	AC	9	7
47209	4	13607063	13607082	4	5	20	CTGC	30	3
47212	4	13613244	13613258	3	5	15	TAA	10	4
47213	4	13616175	13616198	3	8	24	GTT	27	11
47220	4	13629631	13629652	2	11	22	TG	16	10
47221	4	13630838	13630857	2	10	20	CA	14	5
47226	4	13635589	13635612	3	8	24	CTG	21	5
47228	4	13640120	13640149	3	10	30	TTA	6	8
47229	4	13643957	13643968	2	6	12	AT	26	3
47232	4	13662819	13662830	2	6	12	GT	19	5
47241	4	13703990	13704005	2	8	16	TC	29	4
47245	4	13713031	13713048	2	9	18	GT	19	9
47265	4	13763612	13763659	4	12	48	AGAT	13	10
47267	4	13764710	13764725	4	4	16	AAAT	19	4
47268	4	13764979	13764992	2	7	14	CA	22	4
47271	4	13771564	13771587	4	6	24	AAAT	18	4
47275	4	13786633	13786647	3	5	15	AAG	29	3
47276	4	13787264	13787283	4	5	20	CTTT	25	9
47283	4	13807132	13807183	4	13	52	TGTA	9	7
47289	4	13824150	13824167	3	6	18	GGA	10	9
47294	4	13837307	13837327	3	7	21	CAG	28	3
47302	4	13849573	13849602	2	15	30	GT	21	9
47305	4	13856410	13856429	4	5	20	AATC	17	4
47311	4	13872727	13872744	2	9	18	GT	23	4
47315	4	13875056	13875071	2	8	16	GT	33	3
47316	4	13881532	13881552	3	7	21	CAG	25	6
47337	4	13937677	13937694	2	9	18	TA	7	6
47338	4	13938023	13938038	4	4	16	ATTG	12	5
47346	4	13953339	13953356	2	9	18	AC	12	11
47348	4	13956415	13956447	3	11	33	AGG	16	4
47362	4	13979313	13979327	3	5	15	GAG	24	4
47369	4	13990407	13990424	2	9	18	TG	21	17
47371	4	13991462	13991477	2	8	16	TA	7	4
47375	4	14026435	14026452	2	9	18	CA	22	4
47379	4	14040675	14040692	2	9	18	AC	15	7
47380	4	14040694	14040711	2	9	18	CA	18	9
47381	4	14046308	14046343	2	18	36	CA	24	12
47387	4	14065206	14065227	2	11	22	AC	10	9
47395	4	14114397	14114414	3	6	18	GCC	29	10
47397	4	14118545	14118558	2	7	14	AG	32	4
47403	4	14139078	14139101	4	6	24	GCCT	18	4
47405	4	14140838	14140864	3	9	27	TGC	28	8
47406	4	14145194	14145209	2	8	16	CA	18	3
47409	4	14148145	14148164	2	10	20	GT	25	16
47410	4	14152707	14152718	2	6	12	AG	25	7
47415	4	14154958	14154979	2	11	22	GA	25	7

47416	4	14155251	14155266	2	8	16	GA	24	5
47421	4	14174889	14174910	2	11	22	TG	24	13
47433	4	14218115	14218126	2	6	12	GT	24	6
47434	4	14218219	14218232	2	7	14	CA	7	4
47441	4	14228051	14228092	3	14	42	TGT	27	14
47452	4	14242710	14242721	2	6	12	TG	30	3
47472	4	14274944	14274961	2	9	18	AC	21	5
47473	4	14274989	14275006	2	9	18	GT	26	4
47477	4	14280453	14280470	2	9	18	TA	7	6
47480	4	14283722	14283742	3	7	21	CAG	24	7
47481	4	14285096	14285113	2	9	18	GT	19	9
47482	4	14285276	14285303	2	14	28	AC	9	6
47485	4	14298594	14298620	3	9	27	TCC	9	5
47489	4	14311391	14311417	3	9	27	GCT	23	5
47495	4	14321529	14321544	4	4	16	TTAT	22	5
47499	4	14338219	14338232	2	7	14	AC	19	7
47502	4	14341474	14341495	2	11	22	AC	10	9
47503	4	14343261	14343280	4	5	20	ATTT	20	3
47504	4	14343563	14343578	2	8	16	GT	7	3
47505	4	14343583	14343598	2	8	16	GT	26	14
47507	4	14345568	14345581	2	7	14	TA	28	3
47509	4	14353104	14353121	2	9	18	AC	24	8
47510	4	14355169	14355194	2	13	26	TG	20	12
47519	4	14369962	14369973	2	6	12	AG	28	3
47521	4	14370495	14370510	2	8	16	CA	25	4
47526	4	14383463	14383476	2	7	14	TC	19	6
47532	4	14397160	14397181	2	11	22	TG	9	8
47533	4	14397555	14397576	2	11	22	TG	16	4
47538	4	14409680	14409699	2	10	20	CA	19	3
47539	4	14413939	14413962	3	8	24	AAC	11	4
47553	4	14443354	14443380	3	9	27	AGG	20	3
47555	4	14451109	14451124	2	8	16	GT	20	6
47569	4	14489917	14489940	3	8	24	ATC	13	8
47570	4	14491482	14491521	4	10	40	TCTG	6	5
47579	4	14512730	14512744	3	5	15	CAG	26	7
47581	4	14515332	14515353	2	11	22	CA	12	5
47591	4	14525478	14525505	2	14	28	GT	16	9
47599	4	14539636	14539653	2	9	18	AG	25	3
47600	4	14548412	14548423	2	6	12	TG	19	4
47608	4	14565112	14565135	2	12	24	CT	14	9
47618	4	14584802	14584821	2	10	20	GT	18	7
47619	4	14585045	14585082	2	19	38	CA	16	7
47625	4	14588554	14588565	2	6	12	TC	16	4
47630	4	14588985	14589000	4	4	16	TTTC	11	7
47632	4	14589401	14589414	2	7	14	TC	17	3
47633	4	14589774	14589807	2	17	34	CA	14	11
47634	4	14589902	14589935	2	17	34	TC	15	8
47635	4	14589981	14589994	2	7	14	AC	18	5
47641	4	14594130	14594149	2	10	20	CA	17	10
47645	4	14605893	14605926	2	17	34	TG	12	11
47646	4	14607238	14607255	2	9	18	AC	23	5

47664	4	14673114	14673127	2	7	14	AC	29	4
47666	4	14673472	14673509	2	19	38	AC	7	7
47679	4	14694786	14694803	3	6	18	CTT	27	3
47682	4	14695236	14695249	2	7	14	TG	21	6
47689	4	14711435	14711462	4	7	28	TCAT	32	6
47696	4	14721754	14721771	3	6	18	GAG	25	3
47697	4	14722998	14723013	4	4	16	TTTC	10	3
47703	4	14734405	14734419	3	5	15	TTC	22	3
47707	4	14755899	14755942	2	22	44	TG	6	8
47710	4	14774297	14774310	2	7	14	TG	25	3
47712	4	14788385	14788404	4	5	20	ATTC	19	3
47714	4	14792274	14792318	3	15	45	TTA	6	7
47715	4	14800720	14800743	4	6	24	AAAC	23	9
47717	4	14804163	14804214	2	26	52	GT	15	10
47722	4	14816316	14816331	2	8	16	CT	33	3
47742	4	14870244	14870261	2	9	18	GT	24	8
47743	4	14889033	14889046	2	7	14	AC	11	3
47753	4	14919589	14919640	2	26	52	TG	7	10
47759	4	14938150	14938165	2	8	16	AT	19	5
47763	4	14942482	14942507	2	13	26	CA	20	8
47769	4	14964801	14964812	2	6	12	TA	16	3
47776	4	14978163	14978177	3	5	15	CCT	26	3
47777	4	14978749	14978768	4	5	20	TTAT	28	5
47780	4	14990781	14990795	3	5	15	GCT	17	4
47784	4	14998329	14998352	2	12	24	TG	14	11
47795	4	15005487	15005518	2	16	32	CA	22	8
47796	4	15007763	15007784	2	11	22	CA	27	8
47798	4	15008320	15008335	2	8	16	AG	8	3
47800	4	15010229	15010252	2	12	24	AC	17	8
47804	4	15017515	15017544	2	15	30	TG	14	10
47805	4	15023071	15023097	3	9	27	TTG	25	4
47807	4	15027174	15027185	2	6	12	AC	34	3
47816	4	15039681	15039698	2	9	18	CA	28	11
47817	4	15042360	15042380	3	7	21	CAG	29	3
47823	4	15059606	15059629	4	6	24	TGCC	19	10
47827	4	15066713	15066728	2	8	16	GT	21	3
47829	4	15082001	15082012	2	6	12	GA	30	5
47830	4	15096970	15096997	2	14	28	TG	22	12
47836	4	15111685	15111696	2	6	12	CT	8	4
47855	4	15139020	15139051	2	16	32	CA	9	9
47857	4	15141167	15141194	2	14	28	TG	13	9
47862	4	15147792	15147818	3	9	27	GAG	24	14
47863	4	15152766	15152777	2	6	12	AC	29	3
47866	4	15159548	15159565	2	9	18	AT	11	5
47874	4	15196054	15196085	4	8	32	AAGA	22	5
47875	4	15199287	15199316	2	15	30	AG	10	10
47883	4	15225435	15225462	2	14	28	GT	17	8
47888	4	15231461	15231472	2	6	12	GT	26	3
47897	4	15248152	15248163	2	6	12	AC	24	3
47901	4	15251973	15252004	2	16	32	CA	20	10
47902	4	15254199	15254213	3	5	15	TGC	24	3

47903	4	15261793	15261810	2	9	18	CT	15	13
47923	4	15305083	15305094	2	6	12	TG	8	3
47924	4	15305230	15305244	3	5	15	AGC	22	3
47934	4	15343856	15343879	3	8	24	CTG	29	5
47943	4	15386208	15386219	2	6	12	TG	23	6
47944	4	15386221	15386232	2	6	12	GT	26	4
47946	4	15404849	15404872	2	12	24	TG	9	3
47956	4	15419068	15419085	3	6	18	AGG	26	3
47957	4	15419724	15419739	4	4	16	AGAC	24	3
47963	4	15427331	15427360	2	15	30	AG	16	5
47966	4	15437883	15437904	2	11	22	GT	28	12
47969	4	15444788	15444813	2	13	26	CA	21	7
47971	4	15449412	15449431	2	10	20	TG	23	4
47985	4	15481886	15481925	2	20	40	GT	13	6
47988	4	15482378	15482393	4	4	16	AAAT	32	3
47992	4	15491671	15491686	2	8	16	CT	25	4
48001	4	15509899	15509912	2	7	14	AT	23	5
48010	4	15517290	15517301	2	6	12	CA	24	8
48014	4	15519260	15519297	2	19	38	AG	7	7
48019	4	15534365	15534396	2	16	32	TG	12	11
48023	4	15548603	15548626	3	8	24	TTC	18	8
48024	4	15549275	15549298	2	12	24	TG	8	5
48033	4	15577779	15577794	2	8	16	AC	22	5
48035	4	15582766	15582801	2	18	36	GT	9	8
48051	4	15599827	15599844	2	9	18	GA	23	3
48054	4	15603035	15603072	2	19	38	CA	8	7
48055	4	15606473	15606486	2	7	14	AC	20	5
48060	4	15620034	15620049	2	8	16	GT	22	9
48061	4	15620924	15620939	2	8	16	TG	22	6
48062	4	15620940	15620959	2	10	20	AG	14	6
48069	4	15628750	15628764	3	5	15	TCA	19	3
48075	4	15633862	15633876	3	5	15	TAA	25	3
48083	4	15645904	15645945	2	21	42	AC	9	9
48084	4	15646773	15646796	2	12	24	AG	17	12
48087	4	15648273	15648304	2	16	32	GT	7	8
48092	4	15659925	15659942	2	9	18	GT	30	3
48093	4	15661445	15661462	2	9	18	CT	22	4
48094	4	15670636	15670657	2	11	22	GT	22	8
48099	4	15682926	15682943	2	9	18	CA	13	9
48100	4	15683495	15683506	2	6	12	TA	8	13
48105	4	15706965	15706982	2	9	18	TC	20	4
48109	4	15714479	15714498	4	5	20	AAAT	23	3
48114	4	15728606	15728637	2	16	32	GT	8	8
48118	4	15744340	15744357	3	6	18	TTG	20	3
48124	4	15758678	15758693	4	4	16	AAAC	15	7
48151	4	15808221	15808238	2	9	18	TG	15	9
48153	4	15808972	15808998	3	9	27	CTG	27	10
48154	4	15811188	15811203	2	8	16	AC	23	6
48155	4	15813652	15813671	4	5	20	ATAA	16	3
48164	4	15820110	15820127	3	6	18	CTC	15	7
48166	4	15820303	15820314	2	6	12	AC	13	4

48168	4	15828956	15828975	2	10	20	CA	18	8
48169	4	15830887	15830900	2	7	14	GT	21	4
48170	4	15834762	15834783	2	11	22	GT	13	9
48177	4	15853139	15853154	4	4	16	CCAT	23	3
48178	4	15853434	15853448	3	5	15	AAT	16	5
48186	4	15881510	15881524	3	5	15	AGT	19	3
48188	4	15882423	15882440	2	9	18	AC	24	5
48189	4	15882519	15882534	2	8	16	TG	20	4
48191	4	15888122	15888136	3	5	15	AAT	10	3
48199	4	15902208	15902257	2	25	50	AC	6	8
48200	4	15908577	15908590	2	7	14	AC	24	5
48204	4	15913216	15913247	2	16	32	TG	14	8
48205	4	15914235	15914254	2	10	20	AC	20	13
48221	4	15959011	15959026	4	4	16	ACAT	24	4
48222	4	15960814	15960831	2	9	18	TA	9	4
48225	4	15967801	15967822	2	11	22	CA	9	6
48230	4	15976329	15976361	3	11	33	AGG	17	6
48233	4	15984772	15984789	2	9	18	AC	16	6
48235	4	15986149	15986178	2	15	30	TG	11	8
48245	4	16019278	16019301	3	8	24	CTC	24	3
48246	4	16026344	16026376	3	11	33	TGT	19	13
48250	4	16038329	16038342	2	7	14	TG	26	3
48251	4	16038389	16038404	2	8	16	GT	26	6
48253	4	16041301	16041314	2	7	14	CA	13	15
48258	4	16043430	16043481	4	13	52	TAGA	17	10
48261	4	16053269	16053292	2	12	24	TA	19	6
48262	4	16053738	16053757	2	10	20	GT	18	9
48269	4	16082536	16082551	2	8	16	GT	27	9
48276	4	16115173	16115187	3	5	15	ACA	19	4
48285	4	16158470	16158485	4	4	16	GACT	29	5
48291	4	16186371	16186394	2	12	24	AC	13	7
48293	4	16194274	16194294	3	7	21	GAG	19	6
48294	4	16194939	16194950	2	6	12	AC	13	10
48295	4	16194953	16194966	2	7	14	AC	13	11
48298	4	16207480	16207509	2	15	30	TG	20	14
48304	4	16215874	16215897	3	8	24	GAG	16	6
48315	4	16224510	16224535	2	13	26	TG	11	8
48322	4	16245533	16245550	3	6	18	GGA	19	5
48323	4	16245859	16245882	4	6	24	CAAA	25	6
48334	4	16258501	16258518	2	9	18	AC	20	5
48341	4	16263077	16263096	2	10	20	CA	19	9
48346	4	16269747	16269761	3	5	15	AGA	26	4
48350	4	16278525	16278552	4	7	28	GTCT	22	6
48355	4	16285095	16285108	2	7	14	CA	21	6
48356	4	16285610	16285639	2	15	30	TG	14	13
48359	4	16289284	16289325	3	14	42	TTA	8	5
48361	4	16290896	16290921	2	13	26	TG	6	8
48368	4	16297044	16297064	3	7	21	TGT	28	6
48369	4	16304183	16304194	2	6	12	TG	28	5
48375	4	16323526	16323540	3	5	15	AAC	22	5
48376	4	16324549	16324560	2	6	12	GT	25	12

48378	4	16324957	16324988	2	16	32	CA	14	11
48381	4	16326892	16326906	3	5	15	TCA	17	4
48385	4	16329863	16329916	2	27	54	GT	15	16
48390	4	16335918	16335933	2	8	16	GT	31	4
48393	4	16336510	16336523	2	7	14	GT	10	9
48399	4	16341228	16341265	2	19	38	CA	21	6
48405	4	16355440	16355461	2	11	22	CA	14	12
48406	4	16358296	16358307	2	6	12	CA	7	3
48407	4	16358447	16358462	4	4	16	ACAG	29	3
48408	4	16358762	16358789	2	14	28	TG	7	7
48410	4	16361787	16361804	2	9	18	CA	24	3
48412	4	16366740	16366757	2	9	18	GT	13	10
48419	4	16378325	16378336	2	6	12	CA	26	10
48422	4	16388548	16388577	2	15	30	TG	13	10
48423	4	16388620	16388651	2	16	32	AC	8	8
48424	4	16388728	16388743	2	8	16	AC	18	5
48425	4	16388746	16388757	2	6	12	AC	23	4
48426	4	16388791	16388806	2	8	16	AG	24	3
48431	4	16392654	16392668	3	5	15	AAT	23	3
48432	4	16396215	16396258	4	11	44	AAGA	13	8
48434	4	16399794	16399808	3	5	15	ATA	27	3
48436	4	16422899	16422910	2	6	12	TG	23	3
48440	4	16432012	16432079	4	17	68	ATCC	20	18
48441	4	16434202	16434225	3	8	24	CTC	24	4
48447	4	16440507	16440530	2	12	24	GT	18	6
48450	4	16456695	16456709	3	5	15	TGT	15	13
48451	4	16457911	16457926	4	4	16	TTTA	16	6
48452	4	16458475	16458501	3	9	27	TTG	25	13
48453	4	16460779	16460794	2	8	16	CA	29	7
48456	4	16465283	16465298	4	4	16	CATC	19	4
48457	4	16466852	16466881	2	15	30	GT	22	11
48463	4	16472617	16472648	2	16	32	AC	20	15
48466	4	16478262	16478277	2	8	16	CA	14	5
48467	4	16479549	16479564	2	8	16	TG	28	4
48468	4	16482370	16482391	2	11	22	CA	13	6
48469	4	16483428	16483445	3	6	18	TAA	33	4
48473	4	16485878	16485893	2	8	16	TC	30	4
48474	4	16487540	16487565	2	13	26	GT	6	8
48477	4	16488989	16489012	2	12	24	AC	19	10
48478	4	16489250	16489267	2	9	18	CA	10	4
48480	4	16490558	16490571	2	7	14	GT	19	6
48482	4	16492434	16492455	2	11	22	GT	18	10
48483	4	16493619	16493632	2	7	14	AC	22	3
48503	4	16506185	16506196	2	6	12	CA	23	3
48504	4	16506431	16506458	2	14	28	AC	7	12
48505	4	16507210	16507229	2	10	20	TG	8	8
48507	4	16507526	16507551	2	13	26	TG	16	9
48508	4	16509786	16509805	2	10	20	AC	22	9
48509	4	16510720	16510731	2	6	12	GT	23	3
48510	4	16511077	16511092	2	8	16	AC	17	6
48511	4	16511093	16511108	2	8	16	CA	21	7

48513	4	16514237	16514256	2	10	20	CT	21	7
48516	4	16515441	16515452	2	6	12	CA	28	3
48523	4	16523058	16523085	2	14	28	AC	12	6
48525	4	16525282	16525307	2	13	26	CA	17	9
48526	4	16525469	16525496	2	14	28	CA	21	11
48527	4	16525994	16526019	2	13	26	GT	7	6
48531	4	16527107	16527118	2	6	12	CA	8	3
48536	4	16528781	16528804	2	12	24	GT	24	11
48538	4	16531199	16531216	3	6	18	CTC	24	3
48539	4	16531725	16531744	2	10	20	CA	15	10
48540	4	16532127	16532154	2	14	28	TG	19	9
48543	4	16534053	16534079	3	9	27	CAG	31	4
48547	4	16537605	16537628	4	6	24	ACAG	23	5
48560	4	16549562	16549597	2	18	36	TG	10	13
48561	4	16550880	16550909	3	10	30	GCT	22	4
48570	4	16570285	16570298	2	7	14	AC	22	5
48573	4	16576441	16576460	2	10	20	GA	11	9
48575	4	16579209	16579256	2	24	48	TG	16	14
48579	4	16585155	16585166	2	6	12	TG	26	6
48587	4	16610473	16610490	2	9	18	AC	20	7
48590	4	16620218	16620235	2	9	18	GT	15	7
48594	4	16637030	16637041	2	6	12	AC	23	3
48597	4	16641630	16641647	2	9	18	CA	18	6
48600	4	16660548	16660559	2	6	12	TC	18	7
48605	4	16672186	16672212	3	9	27	GGA	21	4
48608	4	16674816	16674835	2	10	20	AC	12	6
48609	4	16675231	16675266	2	18	36	AC	9	10
48615	4	16692188	16692208	3	7	21	TCC	17	10
48620	4	16697885	16697908	3	8	24	ATC	20	6
48622	4	16698286	16698301	2	8	16	CT	28	3
48623	4	16702968	16702988	3	7	21	ATG	31	4
48624	4	16703159	16703173	3	5	15	CAA	29	3
48632	4	16717238	16717255	3	6	18	GAG	22	6
48633	4	16717896	16717910	3	5	15	TCT	24	9
48638	4	16737250	16737264	3	5	15	TAG	23	4
48658	4	16798186	16798199	2	7	14	TG	21	4
48671	4	16816857	16816876	2	10	20	AC	15	8
48680	4	16855761	16855776	4	4	16	GACT	12	6
48682	4	16857239	16857254	2	8	16	TG	22	9
48689	4	16901124	16901135	2	6	12	TG	17	7
48691	4	16907918	16907931	2	7	14	GT	25	10
48697	4	16923660	16923671	2	6	12	AC	23	3
48701	4	16929949	16929962	2	7	14	TC	20	4
48702	4	16938706	16938717	2	6	12	AC	27	3
48708	4	16972581	16972594	2	7	14	GT	20	3
48722	4	17006529	17006544	2	8	16	AC	23	4
48740	4	17049903	17049923	3	7	21	GTA	16	3
48741	4	17050582	17050597	2	8	16	GT	16	5
48756	4	17061377	17061400	2	12	24	TG	19	4
48761	4	17069568	17069581	2	7	14	CA	22	3
48763	4	17074635	17074649	3	5	15	ATT	13	4

48764	4	17074737	17074750	2	7	14	AC	12	3
48783	4	17115624	17115644	3	7	21	CAA	18	8
48788	4	17130378	17130395	2	9	18	GT	24	4
48792	4	17139707	17139728	2	11	22	AC	19	7
48794	4	17146022	17146037	2	8	16	AC	25	4
48797	4	17150092	17150103	2	6	12	CA	33	3
48805	4	17172332	17172347	4	4	16	TGTC	23	4
48811	4	17191041	17191067	3	9	27	AAC	21	12
48814	4	17201583	17201632	2	25	50	CA	7	7
48816	4	17203660	17203671	2	6	12	AG	16	4
48817	4	17203691	17203702	2	6	12	GA	20	4
48819	4	17205200	17205217	2	9	18	AC	29	5
48822	4	17210115	17210146	2	16	32	GT	18	9
48837	4	17224027	17224048	2	11	22	CA	24	11
48845	4	17247046	17247065	4	5	20	AGAT	19	3
48847	4	17262991	17263010	2	10	20	CA	22	8
48848	4	17263198	17263241	2	22	44	GA	14	12
48852	4	17271367	17271382	2	8	16	AT	9	8
48856	4	17286223	17286240	2	9	18	TG	9	8
48857	4	17293081	17293096	2	8	16	AC	18	13
48858	4	17293098	17293111	2	7	14	CA	23	11
48870	4	17337743	17337774	2	16	32	AG	9	12
48874	4	17344990	17345001	2	6	12	AC	18	3
48875	4	17354898	17354936	3	13	39	CAT	23	7
48877	4	17360679	17360705	3	9	27	AAT	15	13
48879	4	17382961	17382988	4	7	28	TTTC	6	4
48886	4	17397375	17397404	2	15	30	GA	12	7
48895	4	17407640	17407685	2	23	46	CA	9	11
48896	4	17407871	17407897	3	9	27	GAG	8	7
48900	4	17418606	17418625	4	5	20	AAAT	18	8
48903	4	17426813	17426830	2	9	18	GT	12	5
48914	4	17463642	17463657	2	8	16	TG	18	9
48916	4	17466498	17466539	2	21	42	CA	9	9
48921	4	17488481	17488520	2	20	40	AG	7	7
48935	4	17530701	17530728	2	14	28	TC	9	7
48936	4	17532060	17532074	3	5	15	TTG	17	4
48939	4	17536397	17536420	2	12	24	TC	16	7
48951	4	17572997	17573012	2	8	16	TG	27	5
48952	4	17577373	17577388	2	8	16	TG	18	7
48953	4	17577390	17577401	2	6	12	GT	10	5
48954	4	17580487	17580498	2	6	12	TG	23	5
48955	4	17580864	17580879	2	8	16	CA	12	9
48957	4	17583742	17583789	2	24	48	GT	11	9
48966	4	17608572	17608585	2	7	14	TC	34	3
48971	4	17615950	17615961	2	6	12	GT	29	5
48972	4	17616674	17616701	2	14	28	CA	10	9
48974	4	17621211	17621248	2	19	38	GT	7	11
48977	4	17622501	17622520	4	5	20	CAGT	25	6
48978	4	17622614	17622629	4	4	16	AAAC	8	4
48987	4	17640952	17640967	2	8	16	CT	22	5
48988	4	17643199	17643214	2	8	16	GT	12	5

48990	4	17647702	17647739	2	19	38	CA	12	12
48994	4	17660181	17660202	2	11	22	AG	15	6
48995	4	17675386	17675435	2	25	50	CA	7	9
49000	4	17682816	17682831	2	8	16	GT	22	4
49006	4	17701704	17701733	2	15	30	GT	8	7
49009	4	17710996	17711011	2	8	16	AC	25	7
49010	4	17712354	17712377	4	6	24	CAGA	28	7
49013	4	17746169	17746186	3	6	18	GCA	20	4
49019	4	17756000	17756011	2	6	12	CA	29	4
49020	4	17756785	17756804	4	5	20	TTTC	24	6
49025	4	17758565	17758586	2	11	22	GT	24	6
49048	4	17834120	17834143	3	8	24	ATC	14	5
49049	4	17834353	17834370	2	9	18	AT	22	8
49051	4	17855186	17855201	4	4	16	TATT	23	5
49052	4	17862249	17862298	2	25	50	CA	11	11
49054	4	17870845	17870880	2	18	36	CA	15	8
49061	4	17885607	17885636	2	15	30	TG	10	10
49068	4	17900485	17900516	2	16	32	AC	13	9
49069	4	17900726	17900745	2	10	20	CT	16	6
49070	4	17900990	17901007	2	9	18	TG	21	5
49073	4	17903971	17903982	2	6	12	AG	26	3
49077	4	17910077	17910096	2	10	20	GA	21	3
49089	4	17933791	17933804	2	7	14	AG	18	3
49090	4	17938256	17938279	4	6	24	CAGC	24	6
49091	4	17938341	17938354	2	7	14	AC	25	3
49094	4	17939415	17939434	2	10	20	TC	26	3
49097	4	17942404	17942415	2	6	12	CA	23	3
49100	4	17943254	17943281	2	14	28	CA	15	9
49101	4	17948283	17948306	2	12	24	CA	21	8
49106	4	17956968	17956985	3	6	18	ACA	34	3
49108	4	17961424	17961443	2	10	20	CT	12	4
49112	4	17962485	17962514	2	15	30	GT	9	10
49114	4	17963292	17963319	2	14	28	AC	19	5
49115	4	17964150	17964171	2	11	22	CT	10	7
49130	4	17975553	17975564	2	6	12	TC	17	7
49131	4	17976013	17976032	2	10	20	CA	28	5
49133	4	17980329	17980350	2	11	22	AC	24	7
49134	4	17980591	17980612	2	11	22	GT	18	10
49137	4	17984007	17984024	3	6	18	GGT	27	3
49139	4	17984692	17984705	2	7	14	AC	27	5
49144	4	17986354	17986381	2	14	28	GT	17	7
49145	4	17986685	17986702	2	9	18	CT	15	5
49149	4	17994928	17994939	2	6	12	CA	6	3
49152	4	17995879	17995902	2	12	24	GT	16	14
49154	4	17997086	17997099	2	7	14	GC	19	19
49156	4	17998661	17998672	2	6	12	CA	20	3
49157	4	18000406	18000417	2	6	12	TG	19	3
49159	4	18004933	18004947	3	5	15	GAG	28	3
49172	4	18021697	18021716	2	10	20	TG	13	4
49176	4	18037375	18037390	2	8	16	AC	24	9
49177	4	18039660	18039683	2	12	24	CT	19	8

49178	4	18039802	18039815	2	7	14	CA	22	3
49183	4	18054088	18054101	2	7	14	TA	19	5
49184	4	18055751	18055766	2	8	16	CA	27	7
49185	4	18055928	18055941	2	7	14	CA	23	7
49191	4	18075951	18075980	2	15	30	GA	21	7
49194	4	18089017	18089038	2	11	22	TG	24	6
49200	4	18098778	18098797	4	5	20	CTGA	26	5
49202	4	18100614	18100625	2	6	12	AC	29	3
49204	4	18108027	18108046	4	5	20	TTAT	15	5
49210	4	18109285	18109296	2	6	12	CG	15	12
49215	4	18112328	18112342	3	5	15	GAG	21	5
49216	4	18113188	18113207	4	5	20	TTTC	31	3
49219	4	18117100	18117111	2	6	12	CA	17	4
49220	4	18117614	18117643	2	15	30	AC	10	10
49233	5	62694	62708	3	5	15	TAA	12	3
49237	5	83062	83075	2	7	14	TG	23	5
49243	5	92450	92471	2	11	22	TG	18	6
49248	5	98454	98473	2	10	20	GT	14	4
49249	5	98630	98655	2	13	26	AC	12	8
49253	5	101094	101114	3	7	21	AAG	21	3
49255	5	102127	102142	4	4	16	AAAC	26	3
49259	5	106055	106082	2	14	28	TG	12	8
49260	5	107737	107752	4	4	16	AATG	29	3
49268	5	114730	114763	2	17	34	CA	12	6
49273	5	127983	128022	2	20	40	CA	9	11
49277	5	136559	136578	2	10	20	GT	18	11
49281	5	144186	144201	2	8	16	AC	16	8
49285	5	168327	168365	3	13	39	GCA	25	9
49286	5	168386	168400	3	5	15	GCT	17	3
49288	5	174274	174293	2	10	20	TG	21	8
49291	5	182907	182922	2	8	16	AC	24	5
49293	5	193795	193818	2	12	24	TG	17	7
49300	5	243020	243035	2	8	16	AC	18	3
49301	5	243355	243366	2	6	12	TA	17	4
49305	5	249439	249478	2	20	40	GT	14	9
49316	5	260972	260986	3	5	15	GCT	28	4
49323	5	278767	278784	2	9	18	AC	23	8
49328	5	291489	291512	3	8	24	GCT	27	3
49334	5	308682	308693	2	6	12	CA	27	3
49336	5	311166	311189	3	8	24	TCC	23	7
49337	5	313519	313534	2	8	16	AC	28	5
49339	5	315217	315228	2	6	12	GT	18	7
49340	5	315232	315243	2	6	12	TG	14	5
49341	5	315345	315356	2	6	12	TG	8	7
49344	5	318910	318921	2	6	12	GT	23	7
49348	5	325226	325255	3	10	30	ATC	26	10
49353	5	347453	347464	2	6	12	AC	27	3
49354	5	347842	347859	3	6	18	CTC	16	6
49355	5	347861	347875	3	5	15	TCC	17	9
49363	5	363070	363081	2	6	12	CA	18	6
49366	5	368523	368558	3	12	36	AGG	18	6

49373	5	381923	381934	2	6	12	CA	21	3
49376	5	388162	388173	2	6	12	AC	23	4
49380	5	391987	392024	2	19	38	TG	9	10
49390	5	398394	398421	2	14	28	TG	16	13
49396	5	402959	402985	3	9	27	ATA	16	5
49398	5	406690	406701	2	6	12	TG	32	4
49403	5	412271	412312	2	21	42	AC	9	12
49404	5	412440	412457	2	9	18	GT	19	7
49405	5	418898	418923	2	13	26	GT	21	17
49420	5	436188	436203	2	8	16	TG	22	10
49422	5	438030	438049	4	5	20	TTTG	18	16
49426	5	446529	446540	2	6	12	TA	21	4
49427	5	447484	447509	2	13	26	TG	17	11
49428	5	448542	448557	4	4	16	TTTA	28	5
49430	5	449790	449815	2	13	26	TG	15	12
49432	5	451567	451592	2	13	26	AC	7	9
49433	5	451727	451756	2	15	30	GT	19	10
49434	5	451900	451927	2	14	28	TG	10	8
49443	5	460343	460354	2	6	12	CA	23	3
49444	5	460449	460476	2	14	28	CA	21	10
49454	5	478530	478551	2	11	22	CA	16	6
49456	5	483728	483741	2	7	14	TG	23	4
49457	5	484099	484110	2	6	12	AC	23	7
49460	5	488675	488686	2	6	12	TG	24	3
49463	5	490035	490052	2	9	18	AC	20	5
49465	5	491331	491346	2	8	16	CA	20	4
49468	5	495177	495200	2	12	24	GT	17	11
49470	5	496275	496304	2	15	30	AC	13	10
49484	5	511521	511548	2	14	28	AC	11	11
49485	5	511578	511589	2	6	12	TG	21	4
49486	5	511715	511728	2	7	14	CA	24	3
49491	5	526071	526084	2	7	14	AT	20	5
49492	5	530537	530556	2	10	20	TG	17	8
49493	5	531485	531502	2	9	18	CA	16	6
49495	5	535085	535102	2	9	18	TG	18	11
49499	5	541045	541058	2	7	14	AT	14	4
49508	5	544949	544960	2	6	12	GT	17	9
49511	5	545950	545971	2	11	22	AC	8	13
49513	5	546273	546300	2	14	28	GT	6	6
49516	5	548772	548787	2	8	16	CT	28	5
49518	5	554320	554367	2	24	48	GT	12	11
49519	5	554426	554457	2	16	32	GT	14	10
49520	5	554583	554620	2	19	38	CA	12	9
49521	5	554795	554832	2	19	38	TC	8	6
49526	5	567438	567451	2	7	14	TC	15	3
49530	5	577077	577096	2	10	20	AC	18	7
49531	5	577738	577755	3	6	18	CCT	15	5
49532	5	577759	577773	3	5	15	CCT	18	3
49536	5	583429	583444	4	4	16	GCCT	22	7
49540	5	585334	585363	2	15	30	TG	20	8
49543	5	589177	589192	2	8	16	AC	16	5

49544	5	589982	590003	2	11	22	AT	8	5
49545	5	590745	590756	2	6	12	CA	31	6
49552	5	597478	597489	2	6	12	CA	22	3
49554	5	598216	598229	2	7	14	GA	15	4
49555	5	598261	598282	2	11	22	GA	10	4
49559	5	601598	601615	2	9	18	CA	19	5
49568	5	616410	616425	4	4	16	TGGA	34	3
49569	5	616632	616643	2	6	12	TC	19	3
49571	5	618015	618038	4	6	24	ATGG	22	4
49572	5	619448	619475	2	14	28	GT	20	8
49576	5	627788	627831	2	22	44	CA	8	7
49577	5	627944	627955	2	6	12	CA	28	8
49578	5	628203	628214	2	6	12	GT	19	3
49585	5	651199	651224	2	13	26	GA	10	12
49590	5	660452	660465	2	7	14	CA	25	4
49591	5	660821	660840	2	10	20	GT	14	4
49593	5	660980	661023	2	22	44	AG	7	8
49595	5	670143	670170	2	14	28	TG	21	6
49605	5	689144	689159	4	4	16	CAGG	30	3
49609	5	698339	698354	2	8	16	GT	24	7
49610	5	698545	698558	2	7	14	GT	27	3
49611	5	698652	698675	2	12	24	TG	8	10
49614	5	705893	705912	4	5	20	ATTT	23	3
49620	5	721556	721567	2	6	12	TG	24	3
49626	5	738238	738253	2	8	16	TG	22	8
49629	5	743405	743422	2	9	18	CA	24	7
49649	5	797853	797878	2	13	26	TG	12	10
49650	5	798027	798048	2	11	22	GT	23	4
49651	5	799000	799021	2	11	22	GA	13	4
49654	5	812147	812168	2	11	22	GA	21	4
49657	5	816864	816875	2	6	12	CA	18	4
49666	5	843616	843637	2	11	22	TG	12	5
49668	5	846910	846924	3	5	15	AAT	25	8
49669	5	847896	847907	2	6	12	CT	28	3
49670	5	848324	848345	2	11	22	GT	12	7
49673	5	859687	859714	4	7	28	ATGG	20	6
49674	5	862956	862977	2	11	22	CA	18	13
49676	5	865639	865652	2	7	14	TG	13	10
49678	5	865918	865933	4	4	16	TTTG	17	3
49679	5	868764	868791	2	14	28	GT	23	5
49681	5	871834	871881	4	12	48	TCAA	16	13
49682	5	875409	875422	2	7	14	GA	16	5
49691	5	892284	892295	2	6	12	CT	28	3
49694	5	894656	894676	3	7	21	AGA	26	3
49697	5	900119	900134	4	4	16	TTTA	22	4
49702	5	913620	913649	2	15	30	CA	6	6
49703	5	914178	914189	2	6	12	CT	16	6
49705	5	916022	916035	2	7	14	TG	30	4
49706	5	917935	917950	4	4	16	TTTA	26	3
49707	5	921563	921576	2	7	14	TG	23	4
49711	5	937983	938010	4	7	28	AATA	16	6

49719	5	959556	959571	2	8	16	TC	25	3
49720	5	959683	959698	2	8	16	CT	25	5
49725	5	969048	969071	3	8	24	GAA	15	7
49728	5	974639	974654	2	8	16	AG	20	4
49733	5	992301	992314	2	7	14	AC	22	13
49754	5	1030068	1030085	3	6	18	AGC	23	6
49760	5	1033640	1033663	2	12	24	TG	17	5
49763	5	1034948	1034961	2	7	14	AC	20	3
49766	5	1041230	1041261	2	16	32	GA	8	11
49771	5	1044796	1044811	4	4	16	AGAT	9	11
49774	5	1046789	1046804	2	8	16	CA	18	8
49784	5	1063034	1063073	4	10	40	ATCT	18	11
49786	5	1069616	1069659	2	22	44	CA	12	11
49788	5	1071169	1071190	2	11	22	GT	10	12
49800	5	1098596	1098615	4	5	20	CAAA	34	3
49801	5	1098754	1098780	3	9	27	GAA	19	4
49808	5	1107178	1107209	2	16	32	CA	20	11
49814	5	1115674	1115695	2	11	22	CA	12	6
49816	5	1116866	1116913	2	24	48	GA	10	9
49817	5	1117152	1117169	2	9	18	CA	22	12
49818	5	1120663	1120677	3	5	15	GCT	25	4
49820	5	1126729	1126746	3	6	18	GAA	13	9
49822	5	1130153	1130168	4	4	16	TTTG	27	3
49825	5	1131510	1131533	2	12	24	CA	17	3
49829	5	1139285	1139310	2	13	26	CA	18	7
49834	5	1152682	1152699	2	9	18	TC	17	5
49836	5	1155174	1155203	2	15	30	GT	23	7
49840	5	1157969	1157984	4	4	16	GGAA	9	3
49854	5	1173324	1173335	2	6	12	GA	21	4
49873	5	1215273	1215290	2	9	18	AG	21	7
49877	5	1218328	1218354	3	9	27	GGA	16	9
49878	5	1218665	1218696	2	16	32	CA	12	4
49885	5	1222752	1222765	2	7	14	CA	24	9
49886	5	1222807	1222824	2	9	18	AC	31	3
49887	5	1224183	1224203	3	7	21	AAG	20	5
49888	5	1228301	1228342	2	21	42	AG	11	9
49889	5	1230138	1230153	2	8	16	CA	25	3
49897	5	1240755	1240788	2	17	34	CA	24	18
49898	5	1240813	1240824	2	6	12	CA	21	5
49900	5	1241024	1241041	3	6	18	AGG	17	4
49902	5	1241453	1241468	2	8	16	AG	19	3
49907	5	1242369	1242388	2	10	20	CA	12	5
49909	5	1242764	1242781	3	6	18	GTG	17	5
49913	5	1255594	1255633	2	20	40	TG	12	13
49927	5	1279144	1279163	4	5	20	CTGG	27	5
49935	5	1312844	1312871	4	7	28	CTGT	24	5
49940	5	1328330	1328344	3	5	15	CCA	20	5
49949	5	1339175	1339192	3	6	18	CAG	29	4
49950	5	1341100	1341111	2	6	12	CA	26	3
49951	5	1341166	1341197	2	16	32	TG	22	11
49955	5	1370070	1370081	2	6	12	TA	19	5

49965	5	1422604	1422637	2	17	34	GT	8	9
49966	5	1425055	1425066	2	6	12	TG	23	4
49970	5	1434112	1434133	2	11	22	GT	10	5
49971	5	1434592	1434607	2	8	16	GT	17	4
49980	5	1470610	1470630	3	7	21	AGA	27	11
49981	5	1471027	1471042	4	4	16	AAAG	19	3
49982	5	1471512	1471531	2	10	20	CT	18	5
49985	5	1482773	1482802	2	15	30	GT	14	7
49989	5	1492860	1492891	4	8	32	ATCA	27	5
49991	5	1494994	1495015	2	11	22	AC	16	4
49992	5	1497509	1497542	2	17	34	TG	9	7
49993	5	1497739	1497756	2	9	18	TG	11	3
50000	5	1503190	1503201	2	6	12	CA	33	7
50001	5	1505225	1505244	2	10	20	TG	17	7
50003	5	1505711	1505728	3	6	18	CTT	20	3
50004	5	1511272	1511289	2	9	18	GT	18	17
50005	5	1512310	1512325	2	8	16	CT	30	3
50011	5	1524602	1524631	2	15	30	AC	21	11
50012	5	1529962	1529976	3	5	15	AGC	24	3
50026	5	1566724	1566739	2	8	16	CA	26	3
50030	5	1573724	1573737	2	7	14	GT	21	4
50031	5	1573947	1573980	2	17	34	CA	15	15
50047	5	1593592	1593617	2	13	26	TG	14	8
50048	5	1595661	1595680	2	10	20	AC	15	14
50049	5	1595682	1595697	2	8	16	CA	12	11
50051	5	1601221	1601244	4	6	24	ATCT	20	11
50066	5	1642570	1642625	2	28	56	AC	16	17
50075	5	1654955	1654974	2	10	20	CA	23	10
50081	5	1673540	1673555	4	4	16	TGAT	28	4
50082	5	1673980	1673995	2	8	16	TC	24	3
50086	5	1693980	1693994	3	5	15	AGC	30	3
50087	5	1697194	1697207	2	7	14	TA	11	5
50088	5	1697975	1697988	2	7	14	AT	8	3
50089	5	1698402	1698417	4	4	16	TTGG	22	4
50092	5	1710109	1710122	2	7	14	CA	28	7
50093	5	1735707	1735746	2	20	40	AC	7	9
50096	5	1744935	1744950	2	8	16	AC	15	3
50097	5	1751015	1751028	2	7	14	CA	30	3
50098	5	1758982	1758995	2	7	14	AC	20	3
50099	5	1758997	1759010	2	7	14	CA	20	3
50100	5	1760356	1760381	2	13	26	AC	19	5
50101	5	1768200	1768213	2	7	14	GT	15	4
50102	5	1771051	1771083	3	11	33	TCC	8	5
50117	5	1808363	1808386	2	12	24	CA	11	9
50127	5	1823991	1824036	2	23	46	GT	6	9
50128	5	1824136	1824147	2	6	12	CA	16	5
50129	5	1824149	1824166	2	9	18	AC	11	6
50130	5	1824286	1824309	2	12	24	CA	13	4
50131	5	1826568	1826581	2	7	14	AC	22	6
50139	5	1833531	1833556	2	13	26	CA	19	8
50140	5	1839231	1839260	2	15	30	CA	12	12

50148	5	1869096	1869111	2	8	16	TG	22	8
50150	5	1881876	1881919	2	22	44	TG	14	11
50155	5	1900885	1900922	2	19	38	TG	12	12
50162	5	1909322	1909349	2	14	28	GT	17	8
50172	5	1929567	1929594	2	14	28	AC	16	12
50177	5	1938747	1938762	4	4	16	ATCC	16	5
50179	5	1939637	1939651	3	5	15	GAG	21	4
50180	5	1942162	1942181	2	10	20	CA	12	6
50181	5	1944775	1944786	2	6	12	GT	11	9
50182	5	1944788	1944807	2	10	20	TG	15	10
50185	5	1946957	1946998	3	14	42	TCT	6	7
50188	5	1947780	1947795	4	4	16	AAAT	16	3
50190	5	1948855	1948870	2	8	16	TG	18	4
50194	5	1951016	1951030	3	5	15	GCT	24	4
50197	5	1956458	1956475	2	9	18	TG	22	5
50198	5	1959754	1959775	2	11	22	TG	11	5
50203	5	1976322	1976333	2	6	12	GT	13	4
50206	5	1980296	1980319	3	8	24	AAG	22	4
50207	5	1985656	1985673	3	6	18	TTA	6	6
50209	5	1985840	1985875	2	18	36	AC	8	6
50216	5	2021065	2021098	2	17	34	TG	25	8
50217	5	2022953	2022982	3	10	30	TCT	14	5
50219	5	2030605	2030622	3	6	18	CTC	7	7
50224	5	2038877	2038922	2	23	46	GT	15	10
50226	5	2039946	2039965	4	5	20	CTAT	27	4
50228	5	2050060	2050075	4	4	16	AGAT	24	3
50233	5	2079163	2079180	2	9	18	CA	11	13
50239	5	2097914	2097943	2	15	30	TG	12	11
50243	5	2122819	2122846	2	14	28	GT	16	6
50244	5	2129818	2129841	2	12	24	AG	11	6
50248	5	2138472	2138487	2	8	16	CA	11	10
50252	5	2147002	2147021	4	5	20	AAAT	19	4
50254	5	2150477	2150534	2	29	58	AC	13	9
50257	5	2151587	2151602	2	8	16	CA	26	13
50269	5	2171277	2171306	2	15	30	AC	8	10
50270	5	2172001	2172018	2	9	18	GT	17	12
50271	5	2172019	2172030	2	6	12	GC	14	7
50272	5	2172223	2172248	2	13	26	GT	13	9
50273	5	2174226	2174240	3	5	15	CCA	34	3
50275	5	2177054	2177067	2	7	14	GT	27	3
50277	5	2178997	2179011	3	5	15	CAC	27	6
50278	5	2185513	2185572	3	20	60	TCT	11	9
50279	5	2186225	2186254	2	15	30	CT	12	7
50282	5	2192429	2192462	2	17	34	AC	14	9
50285	5	2212493	2212542	2	25	50	GT	11	14
50300	5	2236637	2236678	2	21	42	TG	19	11
50301	5	2242669	2242680	2	6	12	CT	24	4
50304	5	2245450	2245481	2	16	32	GT	20	14
50310	5	2268804	2268818	3	5	15	ATA	24	3
50311	5	2272889	2272932	2	22	44	GT	15	5
50313	5	2289297	2289317	3	7	21	GCT	25	7

50318	5	2302144	2302179	2	18	36	GT	17	15
50321	5	2303104	2303121	2	9	18	GT	17	4
50333	5	2330700	2330711	2	6	12	AC	16	7
50334	5	2330895	2330952	2	29	58	AC	13	12
50335	5	2331212	2331231	2	10	20	GT	17	4
50336	5	2347024	2347037	2	7	14	CA	31	4
50341	5	2358683	2358700	3	6	18	TCT	24	4
50342	5	2358826	2358843	2	9	18	CA	18	4
50343	5	2359018	2359033	2	8	16	AC	15	3
50347	5	2362447	2362461	3	5	15	GCC	25	5
50369	5	2387043	2387060	2	9	18	CA	17	5
50371	5	2387321	2387332	2	6	12	GT	25	3
50372	5	2387390	2387417	2	14	28	CA	18	5
50373	5	2387728	2387741	2	7	14	TG	14	8
50405	5	2404187	2404214	2	14	28	CA	21	8
50408	5	2407006	2407027	2	11	22	TC	22	6
50409	5	2407496	2407522	3	9	27	GAT	16	6
50414	5	2413354	2413375	2	11	22	GT	14	9
50415	5	2415735	2415746	2	6	12	GT	19	3
50417	5	2417654	2417687	2	17	34	TG	11	9
50421	5	2429859	2429890	2	16	32	AC	12	11
50439	5	2460359	2460386	2	14	28	CA	17	5
50441	5	2464518	2464547	2	15	30	GT	13	9
50443	5	2465688	2465717	3	10	30	GCT	27	9
50446	5	2468086	2468103	2	9	18	CA	22	9
50447	5	2468106	2468119	2	7	14	CA	21	7
50449	5	2468225	2468238	2	7	14	AC	19	3
50452	5	2475446	2475463	2	9	18	CA	21	3
50455	5	2477432	2477451	2	10	20	TG	21	5
50457	5	2477790	2477843	3	18	54	TCT	18	14
50472	5	2493541	2493556	2	8	16	CA	20	6
50474	5	2504946	2504969	2	12	24	TG	17	3
50480	5	2513108	2513123	2	8	16	CT	19	6
50487	5	2523903	2523936	2	17	34	AC	7	10
50488	5	2524239	2524259	3	7	21	TTC	23	4
50489	5	2526984	2527001	3	6	18	TTC	17	8
50493	5	2527973	2527994	2	11	22	CA	12	5
50502	5	2537945	2537959	3	5	15	TTC	15	5
50504	5	2539131	2539154	2	12	24	TG	12	4
50506	5	2544598	2544613	2	8	16	TG	18	3
50516	5	2555457	2555476	4	5	20	TTTC	13	5
50519	5	2556236	2556251	2	8	16	GT	15	5
50521	5	2563540	2563555	2	8	16	TG	21	4
50523	5	2563861	2563872	2	6	12	AC	16	10
50532	5	2569872	2569903	2	16	32	CA	10	5
50533	5	2569932	2569947	2	8	16	CA	19	5
50536	5	2571414	2571453	2	20	40	TG	6	7
50548	5	2581745	2581760	2	8	16	AC	13	3
50550	5	2586627	2586642	2	8	16	CT	11	3
50562	5	2596203	2596218	2	8	16	GT	13	3
50568	5	2601622	2601639	2	9	18	CA	13	4

50581	5	2613549	2613572	2	12	24	CT	10	5
50582	5	2614110	2614127	2	9	18	GA	19	6
50584	5	2616743	2616780	2	19	38	GT	12	6
50586	5	2618151	2618180	2	15	30	TG	15	7
50593	5	2619653	2619688	2	18	36	GA	12	10
50602	5	2623938	2623965	2	14	28	TG	7	9
50606	5	2629691	2629708	3	6	18	TTG	27	3
50612	5	2638607	2638622	2	8	16	TG	27	5
50613	5	2639155	2639180	2	13	26	GT	19	7
50614	5	2639361	2639390	2	15	30	CA	18	8
50617	5	2641780	2641795	2	8	16	TG	17	4
50623	5	2652238	2652273	2	18	36	TG	19	8
50625	5	2655704	2655719	4	4	16	ATGT	14	4
50627	5	2656441	2656460	2	10	20	TG	8	5
50629	5	2659784	2659817	2	17	34	TG	7	5
50630	5	2660331	2660350	4	5	20	CAAA	21	3
50637	5	2662231	2662244	2	7	14	GT	15	3
50639	5	2663307	2663324	2	9	18	GT	19	3
50641	5	2664881	2664898	2	9	18	GT	12	7
50643	5	2666001	2666020	2	10	20	AG	12	6
50646	5	2667444	2667465	2	11	22	CA	17	6
50651	5	2672015	2672028	2	7	14	CA	13	4
50652	5	2672030	2672043	2	7	14	AC	16	5
50659	5	2690056	2690067	2	6	12	TC	19	3
50662	5	2693933	2693958	2	13	26	TG	10	6
50663	5	2694070	2694093	2	12	24	GA	13	7
50665	5	2698051	2698066	2	8	16	TG	29	3
50669	5	2706357	2706394	2	19	38	GT	16	7
50672	5	2711546	2711557	2	6	12	AC	22	10
50673	5	2711562	2711577	2	8	16	AC	18	9
50678	5	2729583	2729594	2	6	12	GT	24	4
50681	5	2730024	2730035	2	6	12	GT	30	4
50682	5	2730273	2730308	2	18	36	AC	7	10
50687	5	2735577	2735588	2	6	12	AC	20	3
50689	5	2736840	2736851	2	6	12	TG	33	4
50691	5	2741407	2741422	4	4	16	TTTA	21	3
50692	5	2742971	2742982	2	6	12	CA	34	4
50700	5	2748780	2748791	2	6	12	CT	12	3
50703	5	2749180	2749191	2	6	12	AC	11	7
50713	5	2762346	2762365	2	10	20	CT	15	4
50721	5	2770306	2770337	2	16	32	CT	8	5
50729	5	2795133	2795184	4	13	52	GATA	24	15
50732	5	2801143	2801163	3	7	21	ATA	7	4
50734	5	2803706	2803735	2	15	30	GT	6	10
50748	5	2842045	2842070	2	13	26	CA	14	10
50749	5	2842570	2842591	2	11	22	CA	20	16
50764	5	2892603	2892654	4	13	52	AGAT	17	12
50767	5	2897517	2897530	2	7	14	TG	12	4
50768	5	2897686	2897727	2	21	42	TG	10	10
50770	5	2899093	2899118	2	13	26	AC	11	4
50771	5	2899774	2899793	2	10	20	AG	31	9

50780	5	2933977	2933990	2	7	14	CA	26	3
50781	5	2936296	2936329	2	17	34	AC	12	7
50782	5	2936732	2936755	2	12	24	AC	19	16
50783	5	2938926	2938939	2	7	14	CA	24	4
50794	5	2969441	2969454	2	7	14	CT	28	4
50798	5	2979591	2979608	3	6	18	AAT	19	5
50820	5	3015483	3015498	2	8	16	AC	15	7
50828	5	3021700	3021719	2	10	20	CT	25	4
50831	5	3033562	3033594	3	11	33	CTT	24	5
50833	5	3043335	3043352	2	9	18	TA	18	3
50834	5	3045496	3045510	3	5	15	CTT	16	8
50835	5	3046021	3046034	2	7	14	CT	15	7
50839	5	3054643	3054654	2	6	12	GT	27	3
50843	5	3062689	3062742	3	18	54	AGA	22	12
50850	5	3072073	3072104	2	16	32	CA	15	6
50853	5	3083492	3083521	2	15	30	AC	21	9
50864	5	3114935	3114955	3	7	21	CCT	9	3
50871	5	3126794	3126832	3	13	39	TCA	26	5
50872	5	3135714	3135739	2	13	26	CA	17	7
50875	5	3153138	3153159	2	11	22	CA	14	8
50876	5	3153754	3153785	2	16	32	TG	19	8
50880	5	3168986	3169017	2	16	32	AC	17	12
50881	5	3171607	3171626	4	5	20	ACCA	28	5
50884	5	3177764	3177781	2	9	18	TG	16	11
50888	5	3199230	3199263	2	17	34	CA	7	8
50890	5	3218698	3218712	3	5	15	ATT	9	8
50899	5	3239123	3239138	2	8	16	AT	22	4
50912	5	3261547	3261572	2	13	26	AC	18	10
50913	5	3265268	3265287	2	10	20	CA	18	4
50932	5	3304385	3304396	2	6	12	CA	28	3
50941	5	3328079	3328094	2	8	16	AC	12	3
50942	5	3328133	3328158	2	13	26	CA	11	6
50944	5	3328919	3328932	2	7	14	AC	23	8
50948	5	3332718	3332747	2	15	30	CA	21	13
50951	5	3334877	3334894	3	6	18	TGT	24	4
50958	5	3345989	3346020	2	16	32	GT	8	7
50962	5	3350994	3351019	2	13	26	GT	12	7
50963	5	3363841	3363861	3	7	21	TAG	29	5
50967	5	3374145	3374160	2	8	16	TG	21	5
50968	5	3380445	3380459	3	5	15	GAT	22	5
50975	5	3394806	3394817	2	6	12	GT	26	3
50976	5	3397765	3397780	4	4	16	TTAT	17	4
50982	5	3423048	3423063	2	8	16	CA	10	3
50986	5	3425404	3425424	3	7	21	GCT	20	7
50988	5	3434196	3434209	2	7	14	CA	16	3
50992	5	3437203	3437223	3	7	21	CTC	18	3
50998	5	3447242	3447262	3	7	21	AGG	6	3
51003	5	3448829	3448840	2	6	12	CT	31	3
51005	5	3451972	3451989	2	9	18	AC	20	8
51006	5	3451991	3452004	2	7	14	CA	17	7
51012	5	3465647	3465678	2	16	32	GT	15	7

51013	5	3465815	3465836	2	11	22	GA	23	9
51014	5	3466140	3466166	3	9	27	CCT	15	4
51015	5	3466251	3466266	2	8	16	CT	30	3
51021	5	3470635	3470652	2	9	18	AC	18	8
51029	5	3473350	3473387	2	19	38	GA	9	5
51031	5	3477259	3477270	2	6	12	AC	9	4
51036	5	3485347	3485366	2	10	20	AC	28	6
51043	5	3491489	3491514	2	13	26	CA	16	7
51047	5	3495152	3495175	2	12	24	CT	18	6
51049	5	3501679	3501700	2	11	22	AC	22	7
51051	5	3503130	3503155	2	13	26	GA	15	7
51058	5	3516621	3516660	2	20	40	AC	12	8
51061	5	3527633	3527660	2	14	28	AG	9	8
51065	5	3532635	3532646	2	6	12	AC	16	3
51068	5	3533370	3533390	3	7	21	AAG	18	3
51070	5	3536698	3536719	2	11	22	GT	10	11
51075	5	3544582	3544597	2	8	16	AC	15	9
51081	5	3549379	3549390	2	6	12	CA	26	8
51082	5	3549430	3549450	3	7	21	CAA	19	6
51083	5	3551137	3551160	3	8	24	CAG	26	3
51084	5	3551228	3551257	2	15	30	CA	15	9
51085	5	3555446	3555472	3	9	27	GAG	10	9
51087	5	3558390	3558425	2	18	36	CA	13	11
51089	5	3566338	3566353	2	8	16	TG	19	5
51092	5	3568093	3568106	2	7	14	GT	12	4
51094	5	3574758	3574783	2	13	26	AC	20	4
51099	5	3590503	3590514	2	6	12	AC	24	8
51101	5	3592268	3592291	2	12	24	AC	12	11
51106	5	3595000	3595017	3	6	18	CCT	21	3
51108	5	3598646	3598661	4	4	16	TAGC	21	4
51111	5	3612342	3612362	3	7	21	CCA	23	3
51116	5	3626974	3626988	3	5	15	ATT	6	3
51117	5	3628484	3628517	2	17	34	TG	20	16
51150	5	3688354	3688386	3	11	33	GCA	14	7
51159	5	3702050	3702070	3	7	21	CTG	24	6
51170	5	3716164	3716187	4	6	24	AATA	6	4
51173	5	3719173	3719186	2	7	14	TC	18	9
51183	5	3733580	3733594	3	5	15	AAC	22	4
51186	5	3741236	3741253	3	6	18	TTA	24	3
51187	5	3743639	3743664	2	13	26	AC	25	8
51188	5	3745264	3745290	3	9	27	AAT	10	6
51190	5	3748595	3748618	3	8	24	GAG	28	4
51191	5	3751446	3751460	3	5	15	TTA	13	8
51192	5	3751812	3751837	2	13	26	AC	8	9
51194	5	3754071	3754086	2	8	16	CA	27	6
51196	5	3757626	3757646	3	7	21	GAT	30	4
51206	5	3772691	3772714	2	12	24	AC	14	10
51208	5	3775387	3775404	3	6	18	GAG	20	4
51209	5	3779371	3779382	2	6	12	GT	17	4
51210	5	3779767	3779778	2	6	12	AT	27	10
51220	5	3811080	3811097	3	6	18	CAC	27	3

51225	5	3823878	3823889	2	6	12	CA	31	3
51228	5	3843364	3843377	2	7	14	AC	25	3
51230	5	3846997	3847014	3	6	18	GAG	17	5
51232	5	3849285	3849296	2	6	12	TA	7	3
51236	5	3860611	3860622	2	6	12	GA	10	8
51237	5	3860624	3860637	2	7	14	AG	10	11
51238	5	3860764	3860779	2	8	16	CA	19	5
51244	5	3883622	3883648	3	9	27	CTC	20	5
51245	5	3884335	3884367	3	11	33	CAG	25	6
51252	5	3902354	3902373	4	5	20	TTTG	29	3
51255	5	3904607	3904620	2	7	14	AC	11	9
51257	5	3908241	3908260	2	10	20	TG	21	8
51272	5	3969316	3969341	2	13	26	TG	25	4
51279	5	3977194	3977213	4	5	20	TTTC	19	6
51284	5	3984917	3984931	3	5	15	AGA	28	3
51288	5	3990774	3990787	2	7	14	CT	15	3
51290	5	3991158	3991175	3	6	18	CTT	15	3
51299	5	4000079	4000099	3	7	21	AAC	17	7
51313	5	4013666	4013679	2	7	14	AG	19	4
51315	5	4014506	4014519	2	7	14	TG	19	3
51326	5	4027466	4027497	2	16	32	GT	17	12
51327	5	4027649	4027666	2	9	18	CA	18	17
51328	5	4027822	4027837	2	8	16	CA	21	5
51334	5	4029594	4029613	2	10	20	CT	11	3
51335	5	4029635	4029655	3	7	21	CAC	26	3
51338	5	4032466	4032486	3	7	21	TGG	25	3
51339	5	4034235	4034270	4	9	36	GCAG	26	6
51340	5	4034706	4034735	2	15	30	AC	11	9
51349	5	4038662	4038676	3	5	15	AGC	14	5
51355	5	4040212	4040229	3	6	18	GCA	20	3
51357	5	4040981	4040992	2	6	12	AC	11	3
51359	5	4041892	4041915	2	12	24	CA	21	12
51360	5	4042790	4042813	3	8	24	AAT	10	4
51372	5	4076976	4076989	2	7	14	CT	26	4
51375	5	4087009	4087052	2	22	44	GA	7	11
51376	5	4090586	4090629	2	22	44	GA	12	13
51383	5	4113190	4113203	2	7	14	GA	32	3
51391	5	4127994	4128009	2	8	16	TG	18	5
51392	5	4133565	4133590	2	13	26	TG	15	8
51394	5	4134698	4134711	2	7	14	TC	31	5
51405	5	4204506	4204523	2	9	18	AC	22	7
51409	5	4206358	4206373	4	4	16	TTTG	30	4
51411	5	4212347	4212366	4	5	20	AGGA	28	5
51414	5	4216665	4216684	4	5	20	AAAT	13	4
51417	5	4218781	4218792	2	6	12	AC	23	3
51420	5	4221498	4221515	3	6	18	GAA	31	3
51427	5	4229736	4229753	3	6	18	GAG	26	5
51436	5	4287680	4287694	3	5	15	TCT	26	4
51449	5	4320700	4320731	4	8	32	TCCA	22	7
51460	5	4338511	4338538	2	14	28	GT	9	7
51461	5	4344894	4344923	3	10	30	CCT	12	5

51462	5	4347047	4347062	2	8	16	GT	20	4
51463	5	4350050	4350061	2	6	12	TC	20	4
51468	5	4364958	4364971	2	7	14	TG	23	7
51483	5	4407650	4407669	2	10	20	AC	14	6
51484	5	4407695	4407706	2	6	12	TG	30	11
51485	5	4408464	4408489	2	13	26	TG	22	12
51488	5	4418215	4418238	2	12	24	AC	18	9
51495	5	4431906	4431945	2	20	40	AC	8	9
51497	5	4445490	4445507	2	9	18	AC	20	4
51500	5	4457686	4457701	4	4	16	GTCT	28	3
51503	5	4465801	4465816	4	4	16	CAGG	25	4
51504	5	4466496	4466516	3	7	21	GCT	23	4
51510	5	4474328	4474341	2	7	14	AG	21	3
51517	5	4478898	4478924	3	9	27	TCA	15	9
51519	5	4480457	4480477	3	7	21	ATG	28	3
51522	5	4481942	4481965	2	12	24	TG	18	15
51525	5	4493443	4493454	2	6	12	CA	19	7
51526	5	4493457	4493470	2	7	14	CA	15	4
51532	5	4507194	4507223	2	15	30	TG	19	11
51539	5	4522905	4522922	3	6	18	CTC	14	3
51550	5	4543715	4543730	4	4	16	TTTG	25	4
51551	5	4544314	4544327	2	7	14	AT	28	9
51554	5	4559514	4559537	4	6	24	CCAG	25	7
51558	5	4574393	4574410	2	9	18	CT	21	5
51560	5	4576579	4576605	3	9	27	AGC	20	7
51563	5	4588568	4588585	2	9	18	GT	9	5
51566	5	4597895	4597910	2	8	16	AC	22	6
51568	5	4601736	4601750	3	5	15	AGG	18	5
51570	5	4607460	4607479	2	10	20	GT	26	9
51584	5	4624032	4624055	2	12	24	CA	16	11
51588	5	4628226	4628243	2	9	18	CT	10	3
51593	5	4632548	4632565	2	9	18	AC	19	7
51594	5	4639227	4639244	2	9	18	TG	20	3
51595	5	4641883	4641898	4	4	16	GACA	21	4
51597	5	4646266	4646281	2	8	16	TG	17	5
51598	5	4647028	4647045	2	9	18	TG	16	5
51599	5	4647522	4647536	3	5	15	GCT	26	7
51609	5	4651131	4651182	2	26	52	AC	9	10
51610	5	4651480	4651505	2	13	26	AC	15	8
51612	5	4651726	4651751	2	13	26	GT	13	3
51619	5	4656645	4656660	2	8	16	AG	26	5
51622	5	4659513	4659534	2	11	22	AC	22	4
51623	5	4660276	4660287	2	6	12	TC	17	6
51624	5	4660288	4660307	2	10	20	AC	19	7
51625	5	4662076	4662113	2	19	38	CA	13	7
51630	5	4678201	4678214	2	7	14	AG	14	3
51633	5	4679104	4679133	2	15	30	TG	11	3
51660	5	4710159	4710179	3	7	21	GAG	20	4
51661	5	4710217	4710230	2	7	14	GT	16	3
51671	5	4730374	4730393	2	10	20	GT	14	4
51672	5	4731553	4731570	3	6	18	GGA	21	4

51673	5	4733036	4733047	2	6	12	TG	27	3
51678	5	4743529	4743542	2	7	14	AC	29	7
51679	5	4743685	4743710	2	13	26	CA	11	9
51680	5	4743930	4743963	2	17	34	GT	23	10
51685	5	4744849	4744863	3	5	15	CCT	19	5
51689	5	4745551	4745571	3	7	21	CCT	22	3
51690	5	4745888	4745905	3	6	18	AAG	23	4
51692	5	4749270	4749285	2	8	16	CA	24	7
51693	5	4752639	4752656	2	9	18	TA	12	10
51707	5	4767806	4767821	2	8	16	GT	10	3
51726	5	4793076	4793093	2	9	18	AC	15	5
51727	5	4793558	4793578	3	7	21	TGC	23	3
51729	5	4796101	4796120	2	10	20	GT	21	7
51730	5	4796396	4796411	2	8	16	TG	21	5
51731	5	4797147	4797164	3	6	18	TCT	20	4
51732	5	4797997	4798020	3	8	24	AAC	27	7
51733	5	4799568	4799583	2	8	16	AT	7	4
51735	5	4808603	4808616	2	7	14	AC	23	6
51740	5	4822086	4822111	2	13	26	CA	9	9
51751	5	4842610	4842633	2	12	24	TG	16	7
51754	5	4847484	4847499	2	8	16	TG	20	3
51755	5	4851870	4851893	4	6	24	AAAC	22	3
51765	5	4893566	4893613	4	12	48	TGGT	16	8
51767	5	4897263	4897276	2	7	14	TA	13	4
51768	5	4900708	4900739	2	16	32	CA	14	11
51769	5	4900870	4900881	2	6	12	CA	22	6
51771	5	4903551	4903577	3	9	27	CTG	22	8
51772	5	4904812	4904845	2	17	34	AC	13	11
51773	5	4906920	4906940	3	7	21	CCA	11	5
51776	5	4922098	4922109	2	6	12	TG	29	4
51785	5	4945242	4945255	2	7	14	TC	14	7
51786	5	4945435	4945460	2	13	26	GT	16	12
51787	5	4945632	4945643	2	6	12	CT	22	4
51788	5	4945707	4945721	3	5	15	CTC	27	6
51791	5	4954344	4954369	2	13	26	AC	20	8
51794	5	4956347	4956386	2	20	40	GT	13	14
51798	5	4960335	4960360	2	13	26	TG	14	7
51803	5	4968013	4968034	2	11	22	GT	22	11
51804	5	4968196	4968229	2	17	34	GT	11	11
51806	5	4975203	4975230	4	7	28	CTTC	12	5
51812	5	4980715	4980740	2	13	26	TC	7	5
51815	5	4988135	4988146	2	6	12	GT	32	3
51820	5	4996676	4996697	2	11	22	GT	20	4
51826	5	5009068	5009103	2	18	36	CA	11	16
51828	5	5015620	5015637	2	9	18	CA	17	8
51831	5	5025429	5025472	2	22	44	TG	11	8
51833	5	5025644	5025655	2	6	12	GT	25	5
51834	5	5027662	5027679	2	9	18	TC	13	4
51839	5	5038820	5038833	2	7	14	TC	19	3
51840	5	5039012	5039026	3	5	15	CTT	14	8
51841	5	5041570	5041599	2	15	30	AC	7	11

51853	5	5060174	5060197	2	12	24	AC	19	11
51854	5	5062130	5062169	2	20	40	CA	18	7
51860	5	5064615	5064626	2	6	12	CA	23	5
51862	5	5064798	5064811	2	7	14	GA	16	4
51863	5	5064838	5064869	2	16	32	GA	12	10
51864	5	5070374	5070411	2	19	38	GT	9	10
51865	5	5074685	5074714	2	15	30	CA	7	10
51866	5	5082189	5082208	2	10	20	AC	16	10
51871	5	5104499	5104510	2	6	12	AC	30	3
51879	5	5125093	5125107	3	5	15	CAG	22	4
51887	5	5139405	5139432	2	14	28	GT	8	9
51890	5	5140376	5140403	2	14	28	CA	25	8
51892	5	5141092	5141119	2	14	28	CT	17	7
51905	5	5170770	5170783	2	7	14	TG	16	3
51906	5	5171317	5171344	2	14	28	TG	26	17
51926	5	5217993	5218012	2	10	20	CT	15	3
51934	5	5227577	5227609	3	11	33	CTG	26	17
51935	5	5228922	5228945	2	12	24	TC	17	7
51936	5	5229125	5229140	2	8	16	GT	17	7
51940	5	5230598	5230627	2	15	30	AC	14	13
51941	5	5231064	5231091	2	14	28	AG	19	15
51943	5	5233720	5233731	2	6	12	AC	33	3
51944	5	5234035	5234062	2	14	28	TG	17	12
51945	5	5234257	5234270	2	7	14	CA	14	3
51946	5	5235135	5235148	2	7	14	GT	23	8
51950	5	5236774	5236794	3	7	21	TAA	10	6
51951	5	5238065	5238088	2	12	24	AC	20	6
51952	5	5238227	5238248	2	11	22	TG	18	4
51955	5	5244776	5244793	2	9	18	CA	24	3
51956	5	5245076	5245091	4	4	16	ACAA	17	4
51957	5	5245633	5245666	2	17	34	CA	10	9
51960	5	5267189	5267208	4	5	20	TTAT	21	3
51961	5	5274348	5274361	2	7	14	GA	12	5
51963	5	5274677	5274696	2	10	20	CA	22	3
51972	5	5287046	5287069	2	12	24	CA	15	9
51973	5	5287169	5287192	2	12	24	CA	10	6
51974	5	5287672	5287683	2	6	12	GT	22	3
51981	5	5305685	5305700	2	8	16	GT	22	8
51990	5	5320279	5320296	3	6	18	ACC	17	7
51991	5	5321935	5321962	2	14	28	GT	14	8
51994	5	5322761	5322778	2	9	18	TG	13	5
51997	5	5323427	5323480	3	18	54	AGA	15	7
51998	5	5325693	5325707	3	5	15	GGT	14	4
52002	5	5336288	5336311	2	12	24	TG	19	10
52003	5	5336412	5336435	2	12	24	AC	25	6
52005	5	5336894	5336909	2	8	16	TG	21	5
52007	5	5338055	5338072	2	9	18	TG	25	3
52011	5	5343511	5343526	2	8	16	GT	23	3
52012	5	5343707	5343744	2	19	38	AC	13	10
52016	5	5346815	5346842	2	14	28	TG	18	11
52017	5	5347499	5347514	2	8	16	TG	20	10

52018	5	5347517	5347532	2	8	16	TG	16	5
52019	5	5347752	5347765	2	7	14	AC	21	4
52020	5	5349092	5349115	2	12	24	AG	23	7
52027	5	5356388	5356403	2	8	16	CA	9	8
52029	5	5356490	5356503	2	7	14	TG	15	4
52031	5	5359336	5359365	2	15	30	CT	8	6
52035	5	5362176	5362203	2	14	28	AC	13	6
52047	5	5371859	5371874	4	4	16	GACT	24	4
52054	5	5386531	5386542	2	6	12	GA	10	9
52058	5	5395754	5395767	2	7	14	CA	14	3
52069	5	5408411	5408434	3	8	24	AGC	29	6
52071	5	5409611	5409626	4	4	16	TATC	24	4
52072	5	5410858	5410887	3	10	30	TCT	26	5
52073	5	5411202	5411215	2	7	14	CA	11	5
52076	5	5411382	5411409	2	14	28	GA	10	12
52077	5	5411761	5411780	2	10	20	GT	21	7
52079	5	5412046	5412067	2	11	22	GT	13	10
52082	5	5412670	5412681	2	6	12	GT	15	7
52083	5	5412682	5412693	2	6	12	GA	16	5
52085	5	5412836	5412847	2	6	12	AC	22	3
52087	5	5414251	5414272	2	11	22	CA	10	9
52090	5	5415719	5415742	2	12	24	TG	18	8
52091	5	5416230	5416245	2	8	16	TG	26	8
52096	5	5419879	5419896	2	9	18	GT	23	5
52098	5	5420421	5420432	2	6	12	TC	24	3
52100	5	5421166	5421191	2	13	26	CA	17	6
52101	5	5421265	5421290	2	13	26	CA	18	6
52104	5	5423491	5423506	4	4	16	GTGC	22	6
52105	5	5424207	5424220	2	7	14	TG	16	3
52107	5	5425210	5425241	2	16	32	AC	13	5
52108	5	5425374	5425391	2	9	18	GT	19	6
52109	5	5425562	5425581	2	10	20	CA	22	5
52110	5	5425711	5425728	2	9	18	CA	23	7
52111	5	5428719	5428730	2	6	12	CA	31	3
52114	5	5429100	5429139	2	20	40	GT	7	5
52116	5	5431081	5431098	3	6	18	AAC	27	8
52117	5	5431453	5431464	2	6	12	TG	24	4
52121	5	5432493	5432514	2	11	22	TC	14	7
52122	5	5432629	5432652	2	12	24	AC	16	6
52123	5	5432694	5432713	2	10	20	TG	22	8
52124	5	5432809	5432826	2	9	18	CT	20	3
52130	5	5436759	5436773	3	5	15	TGG	15	4
52131	5	5436855	5436868	2	7	14	TG	24	4
52133	5	5438842	5438857	2	8	16	GA	27	3
52135	5	5441594	5441609	2	8	16	CA	23	4
52138	5	5444706	5444729	3	8	24	GAG	31	5
52140	5	5449485	5449514	2	15	30	CA	13	5
52143	5	5451752	5451763	2	6	12	TG	18	5
52144	5	5452039	5452056	2	9	18	CA	10	8
52145	5	5452167	5452186	2	10	20	GA	19	7
52147	5	5452238	5452249	2	6	12	GA	24	3

52153	5	5455387	5455398	2	6	12	AG	17	3
52155	5	5455862	5455891	2	15	30	TG	16	9
52156	5	5456024	5456043	2	10	20	AG	24	4
52158	5	5457066	5457087	2	11	22	TG	9	4
52161	5	5461026	5461051	2	13	26	CT	17	8
52162	5	5461152	5461179	2	14	28	TC	28	9
52167	5	5471866	5471881	2	8	16	CA	25	3
52170	5	5474252	5474263	2	6	12	CA	21	7
52171	5	5474265	5474280	2	8	16	AC	16	6
52174	5	5477508	5477525	2	9	18	AC	19	4
52175	5	5478132	5478149	2	9	18	CA	27	4
52176	5	5478634	5478653	4	5	20	GTTT	22	3
52178	5	5479399	5479416	2	9	18	CT	24	4
52181	5	5483462	5483483	2	11	22	CA	16	8
52182	5	5483627	5483642	2	8	16	GT	18	11
52183	5	5483710	5483725	2	8	16	AC	25	3
52185	5	5483867	5483884	2	9	18	TG	10	8
52188	5	5485668	5485691	2	12	24	CA	13	8
52190	5	5490128	5490157	3	10	30	GAG	28	4
52192	5	5494364	5494377	2	7	14	TC	17	4
52199	5	5497267	5497288	2	11	22	GT	20	10
52201	5	5499398	5499427	2	15	30	AC	17	9
52202	5	5499606	5499621	2	8	16	AC	19	4
52208	5	5516329	5516340	2	6	12	AC	9	3
52210	5	5525880	5525903	3	8	24	CAT	24	6
52216	5	5533590	5533609	2	10	20	CA	23	10
52219	5	5535519	5535530	2	6	12	CA	22	3
52224	5	5545600	5545623	4	6	24	TGTA	8	3
52226	5	5548396	5548409	2	7	14	TG	23	8
52227	5	5548590	5548601	2	6	12	CA	24	12
52230	5	5554779	5554808	2	15	30	TG	17	6
52233	5	5567931	5567957	3	9	27	GAG	12	5
52234	5	5571496	5571509	2	7	14	TA	16	8
52238	5	5580630	5580649	2	10	20	AC	23	7
52244	5	5593116	5593130	3	5	15	TTG	29	3
52245	5	5593461	5593474	2	7	14	TG	22	3
52251	5	5602073	5602084	2	6	12	GT	23	4
52254	5	5603999	5604019	3	7	21	ACA	14	5
52255	5	5606817	5606836	2	10	20	GT	13	6
52256	5	5607276	5607305	2	15	30	TG	7	7
52263	5	5608327	5608350	2	12	24	AC	12	5
52265	5	5609224	5609237	2	7	14	CA	30	5
52269	5	5619244	5619275	2	16	32	GT	14	10
52273	5	5624975	5625010	2	18	36	CA	12	13
52274	5	5625321	5625341	3	7	21	CTG	24	3
52277	5	5635291	5635302	2	6	12	GT	23	5
52279	5	5635577	5635590	2	7	14	TG	18	3
52280	5	5635742	5635761	2	10	20	AC	12	11
52283	5	5638556	5638569	2	7	14	AC	6	3
52285	5	5638739	5638758	2	10	20	GT	19	5
52287	5	5640627	5640646	2	10	20	TG	15	8

52290	5	5642860	5642883	2	12	24	CA	19	6
52291	5	5644056	5644069	2	7	14	CA	18	4
52293	5	5651168	5651189	2	11	22	GT	21	10
52294	5	5654901	5654914	2	7	14	TG	14	6
52295	5	5654979	5655014	2	18	36	AC	12	8
52297	5	5655093	5655104	2	6	12	CA	13	9
52298	5	5655106	5655119	2	7	14	TC	7	3
52301	5	5656010	5656025	2	8	16	AC	14	4
52303	5	5656453	5656474	2	11	22	TG	14	8
52305	5	5660414	5660443	2	15	30	GA	11	9
52306	5	5663263	5663284	2	11	22	AC	13	6
52307	5	5663791	5663814	4	6	24	TAGA	15	9
52308	5	5664853	5664866	2	7	14	TG	24	4
52309	5	5664919	5664936	2	9	18	AC	23	6
52310	5	5665336	5665351	2	8	16	TG	23	14
52311	5	5665352	5665363	2	6	12	AG	18	12
52313	5	5665511	5665526	2	8	16	GT	25	4
52317	5	5670506	5670525	2	10	20	TC	21	5
52318	5	5673934	5673953	2	10	20	CT	30	5
52321	5	5675595	5675610	2	8	16	GT	24	4
52326	5	5681152	5681169	2	9	18	TG	20	7
52335	5	5684915	5684934	2	10	20	CT	24	6
52337	5	5687394	5687419	2	13	26	AC	16	8
52338	5	5688550	5688571	2	11	22	TG	29	3
52339	5	5688742	5688759	2	9	18	AC	21	7
52340	5	5688839	5688874	2	18	36	CA	15	15
52341	5	5689023	5689048	2	13	26	CA	15	12
52342	5	5689121	5689136	2	8	16	CA	23	6
52343	5	5689138	5689153	2	8	16	AC	21	6
52348	5	5690199	5690214	2	8	16	CA	6	4
52350	5	5690266	5690307	2	21	42	CA	13	10
52353	5	5694168	5694209	2	21	42	GT	11	11
52355	5	5696787	5696798	2	6	12	AC	24	3
52357	5	5697648	5697663	2	8	16	GT	29	4
52360	5	5698259	5698278	2	10	20	CA	17	5
52362	5	5700589	5700603	3	5	15	TTC	25	4
52363	5	5704635	5704660	2	13	26	CA	11	9
52365	5	5706388	5706403	2	8	16	CT	12	4
52367	5	5713813	5713848	2	18	36	TG	9	13
52369	5	5722340	5722353	2	7	14	AC	20	3
52375	5	5724348	5724381	2	17	34	AC	12	15
52376	5	5725827	5725856	2	15	30	CA	20	4
52377	5	5725949	5725964	2	8	16	CA	16	5
52378	5	5726191	5726220	2	15	30	TG	12	8
52384	5	5731424	5731453	2	15	30	AC	11	8
52385	5	5732962	5732987	2	13	26	CA	19	6
52386	5	5733106	5733129	3	8	24	TCT	32	7
52395	5	5735990	5736019	3	10	30	GAG	20	6
52397	5	5743102	5743121	2	10	20	AT	16	5
52399	5	5746844	5746869	2	13	26	TG	16	10
52400	5	5749860	5749875	2	8	16	AC	23	7

52401	5	5751484	5751501	2	9	18	CA	10	15
52403	5	5752705	5752732	2	14	28	CT	19	10
52405	5	5754054	5754077	2	12	24	CA	7	9
52408	5	5755183	5755196	2	7	14	AC	20	6
52412	5	5759632	5759645	2	7	14	AC	29	5
52414	5	5761764	5761775	2	6	12	CA	16	5
52415	5	5761777	5761790	2	7	14	AC	18	9
52419	5	5765261	5765280	2	10	20	TG	20	3
52420	5	5768662	5768688	3	9	27	ATC	23	16
52425	5	5778776	5778789	2	7	14	CA	16	8
52426	5	5778792	5778807	2	8	16	CA	9	4
52427	5	5782333	5782364	2	16	32	AC	21	10
52428	5	5782443	5782480	2	19	38	TG	10	14
52448	5	5818373	5818398	2	13	26	CA	10	6
52470	5	5873008	5873041	2	17	34	AC	14	7
52471	5	5873419	5873442	2	12	24	GT	12	3
52472	5	5874688	5874719	2	16	32	AC	14	10
52477	5	5877132	5877153	2	11	22	TG	6	3
52478	5	5877363	5877378	2	8	16	AC	24	4
52479	5	5879162	5879173	2	6	12	AC	23	4
52490	5	5893732	5893763	2	16	32	TG	19	9
52492	5	5895811	5895840	2	15	30	GT	19	8
52494	5	5896439	5896458	2	10	20	CA	19	8
52496	5	5898702	5898715	2	7	14	CA	14	8
52500	5	5902134	5902147	2	7	14	GT	7	5
52504	5	5902498	5902533	2	18	36	GT	8	8
52505	5	5903007	5903022	2	8	16	GT	21	8
52506	5	5903129	5903156	2	14	28	TG	10	3
52507	5	5903266	5903293	2	14	28	AC	12	7
52509	5	5906126	5906141	4	4	16	TTGT	24	3
52511	5	5907925	5907948	2	12	24	AC	19	8
52512	5	5907992	5908023	2	16	32	GT	16	10
52513	5	5908258	5908275	3	6	18	TCC	25	3
52514	5	5913183	5913208	2	13	26	CA	21	4
52515	5	5914140	5914173	2	17	34	CA	13	12
52517	5	5914457	5914483	3	9	27	CTC	13	3
52527	5	5922703	5922726	2	12	24	AC	21	10
52528	5	5922819	5922836	2	9	18	TC	18	11
52534	5	5926942	5926963	2	11	22	GT	17	13
52535	5	5927330	5927369	4	10	40	TTCT	19	6
52549	5	5940542	5940565	2	12	24	TG	15	7
52550	5	5940766	5940793	2	14	28	TG	17	8
52554	5	5948824	5948849	2	13	26	GT	30	7
52555	5	5949061	5949086	2	13	26	CA	19	11
52556	5	5949348	5949375	2	14	28	TG	13	16
52561	5	5954226	5954243	3	6	18	CTC	29	5
52562	5	5956631	5956662	2	16	32	CA	14	11
52564	5	5956819	5956852	2	17	34	GT	16	23
52570	5	5970946	5970965	2	10	20	CA	27	3
52572	5	5974002	5974021	2	10	20	CA	25	5
52574	5	5977227	5977242	2	8	16	GT	11	7

52576	5	5978595	5978606	2	6	12	TG	13	3
52578	5	5978706	5978721	2	8	16	AC	17	3
52597	5	6008834	6008855	2	11	22	AC	18	5
52598	5	6009061	6009078	2	9	18	CA	21	6
52599	5	6009665	6009676	2	6	12	GT	30	3
52603	5	6010350	6010363	2	7	14	GT	23	5
52604	5	6010797	6010814	2	9	18	CT	13	6
52608	5	6013730	6013741	2	6	12	TG	25	5
52609	5	6013742	6013757	2	8	16	AG	21	6
52612	5	6014455	6014474	4	5	20	AAGG	14	4
52615	5	6020673	6020690	2	9	18	CA	27	7
52617	5	6024201	6024212	2	6	12	GT	24	5
52618	5	6027084	6027099	2	8	16	AC	15	9
52619	5	6027102	6027117	2	8	16	AC	12	10
52620	5	6027304	6027321	2	9	18	TG	29	9
52623	5	6029598	6029613	2	8	16	TC	21	4
52624	5	6032404	6032417	2	7	14	TG	22	7
52625	5	6033023	6033036	2	7	14	CA	28	4
52626	5	6035570	6035589	2	10	20	GT	20	7
52628	5	6043381	6043408	2	14	28	GT	19	10
52630	5	6048697	6048710	2	7	14	AC	22	5
52634	5	6052961	6052974	2	7	14	AG	24	4
52635	5	6055330	6055341	2	6	12	TG	18	14
52636	5	6055344	6055357	2	7	14	TG	19	11
52643	5	6060812	6060829	3	6	18	TAT	22	4
52644	5	6061806	6061831	2	13	26	CA	18	10
52646	5	6062597	6062616	4	5	20	TTTG	6	7
52651	5	6067883	6067938	4	14	56	AGAT	11	12
52653	5	6070268	6070301	2	17	34	GT	7	7
52654	5	6070488	6070523	2	18	36	AC	8	6
52656	5	6076845	6076865	3	7	21	GAG	26	4
52658	5	6078088	6078131	2	22	44	AC	6	10
52659	5	6078214	6078225	2	6	12	AC	20	3
52660	5	6083176	6083195	2	10	20	GT	23	5
52661	5	6083505	6083528	2	12	24	CA	16	10
52663	5	6084233	6084250	3	6	18	AGG	24	3
52664	5	6085999	6086028	2	15	30	CA	15	7
52665	5	6086151	6086164	2	7	14	AC	23	6
52667	5	6086310	6086339	2	15	30	AC	14	9
52668	5	6086509	6086544	2	18	36	AC	10	9
52670	5	6089802	6089822	3	7	21	ATC	24	4
52672	5	6090164	6090187	2	12	24	CA	9	6
52680	5	6101666	6101685	2	10	20	GT	15	7
52682	5	6106752	6106781	2	15	30	TG	24	9
52686	5	6139069	6139095	3	9	27	TCT	19	8
52688	5	6144832	6144847	4	4	16	ACTG	33	3
52695	5	6153858	6153883	2	13	26	TG	16	6
52697	5	6154322	6154337	2	8	16	AC	13	5
52698	5	6154483	6154498	2	8	16	AC	17	6
52699	5	6157585	6157608	2	12	24	CA	22	8
52701	5	6158362	6158385	2	12	24	GT	12	12

52702	5	6160293	6160320	2	14	28	GT	17	14
52705	5	6161270	6161283	2	7	14	TG	25	3
52706	5	6161440	6161451	2	6	12	CA	23	5
52707	5	6161632	6161647	2	8	16	TG	21	8
52709	5	6163417	6163428	2	6	12	GT	28	5
52710	5	6163430	6163441	2	6	12	TG	25	5
52712	5	6163612	6163639	2	14	28	CA	10	5
52720	5	6165914	6165943	2	15	30	AC	20	12
52721	5	6167944	6167958	3	5	15	CTG	21	4
52728	5	6169361	6169392	2	16	32	CT	12	6
52732	5	6180377	6180402	2	13	26	GT	21	11
52738	5	6187298	6187311	2	7	14	CT	8	3
52739	5	6187543	6187556	2	7	14	TG	16	6
52740	5	6187812	6187837	2	13	26	CA	10	14
52741	5	6188009	6188028	2	10	20	AC	19	6
52742	5	6193395	6193406	2	6	12	CA	7	10
52743	5	6193408	6193421	2	7	14	AC	11	12
52745	5	6194735	6194768	2	17	34	CA	8	8
52746	5	6195927	6195944	2	9	18	CA	27	6
52749	5	6198461	6198472	2	6	12	CT	20	6
52750	5	6198476	6198489	2	7	14	AC	18	7
52754	5	6206055	6206078	4	6	24	CAGA	27	3
52756	5	6207857	6207872	2	8	16	AC	8	3
52761	5	6215489	6215528	4	10	40	ATCT	17	17
52764	5	6219248	6219261	2	7	14	AC	22	11
52765	5	6227246	6227263	3	6	18	ATC	28	9
52766	5	6228972	6228987	2	8	16	GA	19	4
52770	5	6235618	6235633	2	8	16	TG	11	5
52772	5	6235790	6235801	2	6	12	TC	10	9
52774	5	6237272	6237295	2	12	24	AC	9	9
52775	5	6237831	6237854	2	12	24	AC	16	5
52776	5	6239910	6239921	2	6	12	AC	24	5
52781	5	6241536	6241556	3	7	21	CCT	22	3
52785	5	6260726	6260740	3	5	15	TCT	28	4
52788	5	6262395	6262412	2	9	18	GT	16	8
52789	5	6262587	6262610	2	12	24	TG	13	12
52790	5	6262747	6262766	2	10	20	AG	17	9
52791	5	6266159	6266184	2	13	26	TG	15	13
52792	5	6266291	6266314	2	12	24	TG	16	9
52794	5	6270016	6270039	3	8	24	ATC	24	5
52795	5	6270207	6270228	2	11	22	AC	14	15
52798	5	6274639	6274650	2	6	12	CA	22	4
52799	5	6275348	6275359	2	6	12	TC	21	10
52807	5	6304285	6304298	2	7	14	TG	31	6
52808	5	6307471	6307491	3	7	21	CTC	30	3
52810	5	6311267	6311287	3	7	21	CTC	14	3
52813	5	6311823	6311840	3	6	18	GAG	27	8
52816	5	6325849	6325876	2	14	28	GT	16	9
52818	5	6330920	6330935	2	8	16	GT	24	6
52820	5	6331377	6331392	2	8	16	CA	23	8
52835	5	6341148	6341193	2	23	46	GT	16	9

52842	5	6348720	6348735	2	8	16	TG	23	4
52843	5	6354787	6354801	3	5	15	TAT	17	5
52846	5	6356457	6356480	2	12	24	CA	12	12
52848	5	6362360	6362371	2	6	12	TA	23	7
52865	5	6438140	6438155	2	8	16	TG	10	3
52874	5	6467562	6467576	3	5	15	TCA	29	3
52875	5	6467833	6467846	2	7	14	CT	26	5
52879	5	6480183	6480196	2	7	14	TG	27	3
52880	5	6490813	6490828	4	4	16	ATCC	20	3
52887	5	6527091	6527108	3	6	18	GAA	26	6
52892	5	6552264	6552311	2	24	48	GA	6	7
52894	5	6552466	6552481	2	8	16	AC	22	3
52895	5	6552870	6552893	3	8	24	GAG	18	3
52897	5	6556117	6556136	2	10	20	GT	18	9
52908	5	6613622	6613635	2	7	14	AC	28	5
52912	5	6624150	6624163	2	7	14	GT	23	3
52918	5	6640738	6640759	2	11	22	TG	23	11
52920	5	6646751	6646770	2	10	20	TG	18	5
52921	5	6648404	6648423	2	10	20	GT	18	7
52922	5	6648648	6648673	2	13	26	TG	13	10
52932	5	6675952	6675969	3	6	18	AAG	26	3
52933	5	6676046	6676075	3	10	30	TGA	20	4
52935	5	6676710	6676737	2	14	28	AC	20	10
52938	5	6680555	6680580	2	13	26	AC	15	11
52939	5	6684581	6684604	3	8	24	GCA	20	5
52942	5	6690895	6690909	3	5	15	CAC	14	5
52943	5	6691155	6691168	2	7	14	AC	14	6
52944	5	6691216	6691247	2	16	32	GA	9	8
52948	5	6703122	6703153	2	16	32	GT	13	7
52949	5	6704730	6704749	2	10	20	AC	20	8
52950	5	6704853	6704879	3	9	27	TCT	22	7
52956	5	6712079	6712104	2	13	26	GT	23	8
52957	5	6712699	6712722	4	6	24	TTCT	11	6
52964	5	6722957	6722976	2	10	20	TG	7	7
52965	5	6723000	6723031	2	16	32	TG	7	9
52966	5	6724240	6724257	2	9	18	AC	8	9
52969	5	6724641	6724670	2	15	30	TG	11	9
52971	5	6728527	6728538	2	6	12	GT	14	3
52976	5	6730589	6730608	4	5	20	TTTG	6	3
52977	5	6730713	6730768	4	14	56	CTTT	13	8
52979	5	6733495	6733509	3	5	15	CCT	16	5
52982	5	6735432	6735447	2	8	16	CA	20	8
52988	5	6755135	6755158	3	8	24	GCA	31	5
52989	5	6755187	6755200	2	7	14	AC	20	4
52995	5	6764870	6764901	2	16	32	GT	11	11
52996	5	6768172	6768198	3	9	27	ATT	17	6
52997	5	6770594	6770625	2	16	32	CA	11	11
53006	5	6781314	6781331	2	9	18	AC	20	5
53009	5	6794356	6794373	2	9	18	GT	19	7
53015	5	6808285	6808304	2	10	20	TG	25	7
53021	5	6838067	6838096	2	15	30	GA	23	9

53053	5	6887810	6887837	2	14	28	TG	11	11
53055	5	6896209	6896232	2	12	24	GT	19	8
53057	5	6905294	6905325	4	8	32	ATCT	14	10
53062	5	6922389	6922409	3	7	21	CAG	21	6
53063	5	6926902	6926916	3	5	15	GCA	20	5
53065	5	6931449	6931460	2	6	12	CA	20	9
53067	5	6942660	6942704	3	15	45	AGA	24	8
53070	5	6954774	6954793	2	10	20	TG	20	7
53075	5	6963248	6963271	3	8	24	TCA	19	5
53076	5	6963308	6963323	4	4	16	GTTT	25	5
53080	5	6972804	6972823	4	5	20	TTAT	10	3
53082	5	6982187	6982198	2	6	12	GT	23	3
53094	5	7000085	7000099	3	5	15	AAT	17	3
53095	5	7001641	7001658	2	9	18	CA	28	4
53098	5	7002709	7002723	3	5	15	TGT	26	4
53099	5	7003896	7003941	2	23	46	CA	17	15
53100	5	7006372	7006401	2	15	30	CA	11	5
53104	5	7015636	7015667	2	16	32	AC	13	13
53106	5	7017863	7017888	2	13	26	AC	14	10
53107	5	7019472	7019483	2	6	12	TC	22	3
53110	5	7024244	7024267	3	8	24	CCT	19	3
53111	5	7024457	7024474	2	9	18	TG	16	5
53113	5	7026456	7026467	2	6	12	GA	32	3
53114	5	7026541	7026590	2	25	50	TG	8	11
53115	5	7026992	7027015	2	12	24	CA	17	5
53116	5	7028941	7028952	2	6	12	GT	28	4
53118	5	7031086	7031099	2	7	14	AC	8	8
53119	5	7031101	7031118	2	9	18	CA	13	11
53125	5	7033490	7033537	2	24	48	AC	7	14
53126	5	7033686	7033707	2	11	22	AC	19	15
53131	5	7036902	7036925	2	12	24	GT	23	23
53133	5	7040817	7040838	2	11	22	AC	12	13
53140	5	7048497	7048518	2	11	22	TC	12	5
53143	5	7060851	7060865	3	5	15	CAA	26	8
53144	5	7061430	7061443	2	7	14	AT	15	10
53147	5	7075979	7075993	3	5	15	CAG	27	4
53156	5	7101365	7101379	3	5	15	GCT	34	5
53157	5	7110562	7110576	3	5	15	AGC	25	4
53158	5	7111766	7111779	2	7	14	AC	27	6
53161	5	7126166	7126195	2	15	30	TC	9	10
53162	5	7132286	7132297	2	6	12	CA	21	3
53163	5	7138229	7138240	2	6	12	GT	25	5
53170	5	7150486	7150497	2	6	12	CA	28	5
53171	5	7155808	7155819	2	6	12	CT	30	3
53175	5	7160838	7160861	2	12	24	TG	12	7
53183	5	7177472	7177497	2	13	26	CA	18	9
53184	5	7182360	7182380	3	7	21	TTG	9	4
53186	5	7183780	7183797	2	9	18	AC	14	3
53188	5	7187435	7187476	2	21	42	CA	20	14
53190	5	7191541	7191564	3	8	24	AGG	28	6
53193	5	7196362	7196385	2	12	24	TG	20	13

53194	5	7198462	7198485	2	12	24	GT	22	12
53200	5	7216153	7216173	3	7	21	TCC	21	11
53211	5	7233231	7233254	2	12	24	GT	11	6
53221	5	7261429	7261443	3	5	15	TGA	29	3
53223	5	7261799	7261818	2	10	20	TG	17	11
53227	5	7265500	7265515	2	8	16	AC	15	4
53230	5	7266057	7266068	2	6	12	CA	13	8
53231	5	7266071	7266084	2	7	14	CA	16	9
53233	5	7266213	7266224	2	6	12	GT	15	8
53237	5	7287609	7287620	2	6	12	AC	27	4
53238	5	7287656	7287673	2	9	18	CA	23	9
53239	5	7293010	7293024	3	5	15	CCG	27	6
53240	5	7293281	7293298	2	9	18	AC	16	8
53241	5	7293301	7293316	2	8	16	AC	22	9
53243	5	7298097	7298117	3	7	21	GAG	9	4
53245	5	7298624	7298659	3	12	36	CCT	21	10
53246	5	7301208	7301221	2	7	14	CT	10	6
53247	5	7301505	7301520	2	8	16	TC	22	10
53263	5	7308426	7308455	2	15	30	AC	9	9
53266	5	7312814	7312839	2	13	26	AC	24	8
53267	5	7313412	7313423	2	6	12	CA	12	5
53274	5	7337021	7337052	4	8	32	CAAA	32	3
53275	5	7338362	7338383	2	11	22	TG	14	8
53277	5	7338831	7338845	3	5	15	CCA	20	7
53278	5	7338892	7338907	2	8	16	AG	16	4
53279	5	7339764	7339803	2	20	40	GT	9	8
53280	5	7341230	7341256	3	9	27	TTC	17	8
53282	5	7347596	7347609	2	7	14	TG	23	14
53291	5	7355808	7355821	2	7	14	GT	24	6
53297	5	7359455	7359475	3	7	21	CAG	25	6
53298	5	7367007	7367021	3	5	15	CCG	26	7
53303	5	7371180	7371213	2	17	34	TG	8	7
53305	5	7371455	7371478	4	6	24	TTTA	17	4
53309	5	7383092	7383105	2	7	14	CA	22	11
53310	5	7385052	7385077	2	13	26	TG	20	13
53311	5	7392845	7392864	2	10	20	GT	29	4
53320	5	7414991	7415014	2	12	24	AC	24	3
53322	5	7417537	7417560	3	8	24	CAT	23	8
53325	5	7422559	7422570	2	6	12	TC	12	4
53328	5	7429078	7429098	3	7	21	TGC	23	4
53329	5	7429099	7429119	3	7	21	TGG	22	6
53330	5	7432930	7432955	2	13	26	CA	8	8
53331	5	7433094	7433107	2	7	14	AG	22	8
53334	5	7440204	7440223	2	10	20	CA	16	7
53335	5	7443909	7443924	2	8	16	CT	18	3
53337	5	7445238	7445253	2	8	16	AG	23	4
53338	5	7446264	7446290	3	9	27	GAG	19	8
53339	5	7446460	7446480	3	7	21	TGC	27	5
53342	5	7446963	7446986	3	8	24	GGA	23	4
53344	5	7447200	7447220	3	7	21	AAT	17	9
53348	5	7452350	7452379	2	15	30	AC	9	8

53349	5	7453109	7453126	3	6	18	GGA	28	3
53351	5	7456441	7456452	2	6	12	TG	30	4
53352	5	7458992	7459017	2	13	26	TG	15	9
53355	5	7465889	7465908	2	10	20	GT	17	8
53360	5	7482125	7482156	2	16	32	GT	9	12
53367	5	7502643	7502663	3	7	21	TGC	27	3
53368	5	7503739	7503770	2	16	32	TG	8	9
53369	5	7504381	7504396	2	8	16	GT	15	5
53370	5	7505475	7505495	3	7	21	CCT	30	5
53374	5	7512978	7512989	2	6	12	AC	25	9
53375	5	7513231	7513248	2	9	18	AC	21	10
53377	5	7513367	7513380	2	7	14	CA	18	9
53378	5	7513381	7513396	2	8	16	AC	17	10
53380	5	7518433	7518456	2	12	24	CA	19	8
53382	5	7522807	7522822	2	8	16	TG	14	8
53383	5	7533268	7533289	2	11	22	TC	32	4
53389	5	7540007	7540048	2	21	42	TG	6	8
53393	5	7542058	7542069	2	6	12	AG	13	3
53395	5	7549379	7549396	2	9	18	CA	21	10
53397	5	7550165	7550178	2	7	14	TC	20	3
53401	5	7555434	7555447	2	7	14	CA	11	3
53402	5	7557521	7557536	4	4	16	CTTC	31	3
53404	5	7558704	7558735	2	16	32	GT	12	11
53405	5	7558868	7558885	2	9	18	AG	9	5
53408	5	7559748	7559771	2	12	24	TG	21	11
53410	5	7565743	7565766	2	12	24	CA	29	9
53413	5	7566175	7566194	2	10	20	GA	28	4
53414	5	7566564	7566579	2	8	16	TC	17	7
53417	5	7569437	7569454	2	9	18	GT	17	18
53418	5	7569457	7569470	2	7	14	GT	15	15
53419	5	7569625	7569644	2	10	20	CA	25	6
53420	5	7569717	7569734	3	6	18	GGT	26	6
53429	5	7573728	7573757	2	15	30	CA	10	11
53434	5	7610779	7610806	4	7	28	TCAT	28	9
53451	5	7657079	7657092	2	7	14	GT	16	5
53463	5	7675479	7675498	2	10	20	TA	9	6
53464	5	7686488	7686503	2	8	16	CA	24	6
53470	5	7708850	7708861	2	6	12	GA	26	4
53471	5	7708863	7708874	2	6	12	AG	26	5
53472	5	7713465	7713498	2	17	34	AC	10	13
53475	5	7715058	7715073	4	4	16	AAAC	23	5
53477	5	7720479	7720494	4	4	16	AAAC	6	4
53482	5	7729042	7729063	2	11	22	CA	7	8
53484	5	7739036	7739047	2	6	12	TG	20	3
53488	5	7748574	7748589	2	8	16	AT	17	3
53492	5	7752804	7752825	2	11	22	CA	17	12
53493	5	7754080	7754095	2	8	16	AC	13	5
53495	5	7759715	7759732	2	9	18	AG	18	5
53499	5	7769132	7769151	2	10	20	TG	15	10
53501	5	7771038	7771073	2	18	36	AC	16	12
53502	5	7771215	7771232	2	9	18	AC	21	7

53507	5	7776415	7776442	2	14	28	AC	6	4
53510	5	7781113	7781142	2	15	30	CA	18	8
53513	5	7783878	7783927	2	25	50	AC	8	7
53516	5	7786298	7786315	2	9	18	AC	24	6
53518	5	7790030	7790051	2	11	22	CA	17	15
53524	5	7802802	7802815	2	7	14	CA	17	13
53526	5	7804916	7804933	2	9	18	CA	17	7
53528	5	7808328	7808375	2	24	48	CA	14	12
53532	5	7810541	7810558	3	6	18	GCA	12	5
53535	5	7811356	7811379	3	8	24	TTC	12	4
53536	5	7811403	7811417	3	5	15	TTC	12	3
53547	5	7852485	7852541	3	19	57	AAT	16	9
53551	5	7875565	7875576	2	6	12	GT	28	6
53566	5	7938402	7938425	3	8	24	ATG	22	3
53570	5	7944344	7944359	4	4	16	AAAG	10	4
53573	5	7946593	7946622	3	10	30	GCA	22	7
53575	5	7947014	7947029	2	8	16	GT	16	8
53577	5	7947613	7947639	3	9	27	GAA	12	3
53578	5	7947764	7947777	2	7	14	CT	26	3
53580	5	7948146	7948163	3	6	18	ATT	12	5
53584	5	7950495	7950522	2	14	28	GT	10	7
53585	5	7954534	7954549	2	8	16	GT	22	5
53588	5	7958852	7958871	2	10	20	CA	18	9
53614	5	7996552	7996563	2	6	12	TG	25	8
53615	5	8001536	8001559	2	12	24	CA	11	7
53616	5	8003615	8003650	2	18	36	TG	10	8
53624	5	8012581	8012612	2	16	32	GT	6	8
53625	5	8016609	8016626	3	6	18	GCT	27	4
53628	5	8028383	8028408	2	13	26	TG	20	8
53636	5	8059395	8059408	2	7	14	GT	19	3
53640	5	8064506	8064521	4	4	16	AAAT	31	4
53651	5	8098692	8098713	2	11	22	TG	27	10
53661	5	8111791	8111812	2	11	22	AC	20	11
53672	5	8117757	8117780	2	12	24	CA	20	9
53673	5	8121880	8121897	3	6	18	AGC	28	3
53674	5	8129820	8129859	4	10	40	TATC	14	11
53676	5	8130431	8130452	2	11	22	GT	17	6
53677	5	8130590	8130604	3	5	15	AGG	20	11
53683	5	8133668	8133693	2	13	26	AG	21	6
53686	5	8136280	8136321	2	21	42	TG	9	8
53687	5	8141356	8141383	2	14	28	GT	20	10
53692	5	8143165	8143178	2	7	14	TG	18	3
53697	5	8151833	8151848	2	8	16	CA	26	3
53698	5	8155789	8155806	2	9	18	TA	16	6
53699	5	8157246	8157266	3	7	21	CAG	27	3
53711	5	8173864	8173877	2	7	14	GT	19	9
53718	5	8180597	8180642	2	23	46	GT	13	8
53722	5	8188351	8188371	3	7	21	AGA	27	4
53727	5	8202778	8202803	2	13	26	CA	13	11
53739	5	8212648	8212675	2	14	28	CA	17	9
53744	5	8214638	8214658	3	7	21	TGC	19	3

53745	5	8214873	8214888	2	8	16	TG	20	9
53746	5	8214986	8214997	2	6	12	AC	20	6
53747	5	8215092	8215121	2	15	30	GT	12	13
53751	5	8218059	8218076	2	9	18	CA	16	6
53753	5	8220200	8220229	2	15	30	GT	15	10
53754	5	8224831	8224858	2	14	28	TG	16	9
53756	5	8227269	8227280	2	6	12	TA	23	6
53758	5	8230096	8230115	4	5	20	AAAT	21	3
53760	5	8230652	8230665	2	7	14	AC	13	8
53761	5	8230798	8230827	3	10	30	GGA	6	9
53763	5	8231231	8231248	3	6	18	ATA	13	8
53766	5	8235877	8235896	2	10	20	TC	18	8
53770	5	8237800	8237819	4	5	20	AAAT	27	3
53773	5	8256161	8256193	3	11	33	CAG	24	14
53778	5	8264649	8264676	2	14	28	TG	12	9
53781	5	8265656	8265676	3	7	21	GAG	28	5
53782	5	8267664	8267677	2	7	14	TG	26	5
53784	5	8267871	8267892	2	11	22	CA	26	10
53785	5	8268055	8268090	2	18	36	GT	10	10
53789	5	8273743	8273782	2	20	40	GT	10	8
53795	5	8292174	8292188	3	5	15	CCG	24	9
53796	5	8292425	8292440	4	4	16	TTCA	23	4
53797	5	8293631	8293644	2	7	14	GT	28	3
53798	5	8296089	8296106	2	9	18	AC	23	5
53799	5	8296192	8296229	2	19	38	GT	11	9
53803	5	8297807	8297838	2	16	32	CA	16	11
53804	5	8297947	8297961	3	5	15	CTC	24	3
53805	5	8300226	8300245	2	10	20	AG	24	8
53807	5	8302886	8302909	2	12	24	AC	17	6
53809	5	8303035	8303061	3	9	27	CCT	12	10
53810	5	8303627	8303638	2	6	12	AC	16	3
53811	5	8306194	8306205	2	6	12	TA	22	4
53812	5	8306693	8306708	2	8	16	CA	21	4
53814	5	8310301	8310321	3	7	21	GGA	29	6
53815	5	8311597	8311620	3	8	24	CCT	12	3
53823	5	8313396	8313419	2	12	24	GT	30	7
53824	5	8313558	8313571	2	7	14	CT	20	3
53827	5	8316755	8316766	2	6	12	AG	29	3
53828	5	8318829	8318844	2	8	16	GA	25	4
53830	5	8321621	8321636	2	8	16	TG	25	6
53833	5	8325088	8325117	2	15	30	CA	21	13
53834	5	8327390	8327405	4	4	16	TTTC	24	3
53839	5	8335112	8335143	2	16	32	TG	11	9
53841	5	8337103	8337120	2	9	18	GT	20	8
53842	5	8337982	8338003	2	11	22	GT	9	6
53843	5	8340730	8340741	2	6	12	GA	21	8
53847	5	8341743	8341792	2	25	50	AC	11	11
53848	5	8341911	8341922	2	6	12	GT	17	8
53849	5	8342139	8342156	2	9	18	CA	19	6
53860	5	8355646	8355679	2	17	34	AC	6	7
53862	5	8356101	8356120	2	10	20	TG	10	8

53863	5	8358245	8358264	2	10	20	TG	16	8
53864	5	8358323	8358344	2	11	22	AC	15	9
53865	5	8358505	8358520	2	8	16	TC	16	5
53868	5	8359902	8359927	2	13	26	GT	17	7
53869	5	8360111	8360131	3	7	21	CTG	23	4
53870	5	8361169	8361200	2	16	32	GT	11	8
53871	5	8361386	8361415	3	10	30	CCT	21	6
53874	5	8365171	8365192	2	11	22	CA	19	9
53877	5	8367383	8367415	3	11	33	GTT	20	10
53879	5	8367830	8367847	3	6	18	CCA	16	3
53882	5	8370251	8370262	2	6	12	AC	21	5
53883	5	8370293	8370306	2	7	14	CA	21	3
53884	5	8370397	8370418	2	11	22	GA	11	6
53891	5	8371501	8371532	2	16	32	AC	12	11
53894	5	8379611	8379624	2	7	14	AT	26	5
53902	5	8399831	8399842	2	6	12	TG	27	3
53903	5	8401668	8401683	2	8	16	AC	6	6
53904	5	8405192	8405209	2	9	18	CA	14	8
53905	5	8405374	8405388	3	5	15	CCT	7	4
53906	5	8406217	8406237	3	7	21	GAG	23	10
53909	5	8408072	8408091	2	10	20	GT	16	9
53922	5	8437957	8437990	2	17	34	GT	14	9
53929	5	8451706	8451741	3	12	36	TTC	20	6
53932	5	8455807	8455818	2	6	12	CA	30	14
53934	5	8459232	8459243	2	6	12	TG	27	7
53938	5	8464129	8464164	3	12	36	CCT	14	6
53939	5	8464335	8464366	2	16	32	CT	10	7
53941	5	8465129	8465164	2	18	36	GT	11	12
53943	5	8467240	8467255	2	8	16	TG	23	4
53944	5	8469789	8469806	2	9	18	TG	22	4
53947	5	8476210	8476223	2	7	14	CA	11	6
53949	5	8481501	8481515	3	5	15	CAG	28	5
53955	5	8483670	8483699	3	10	30	TTC	19	7
53957	5	8486211	8486232	2	11	22	AC	11	6
53958	5	8488091	8488108	3	6	18	CAG	25	8
53964	5	8496440	8496465	2	13	26	GT	21	8
53967	5	8501961	8502000	2	20	40	CA	6	9
53969	5	8510449	8510462	2	7	14	GT	20	7
53970	5	8517672	8517685	2	7	14	CA	28	3
53972	5	8519274	8519291	3	6	18	AAG	12	6
53975	5	8522352	8522369	3	6	18	AGG	24	5
53978	5	8522975	8522995	3	7	21	GAG	24	4
53979	5	8523231	8523254	2	12	24	CA	21	12
53980	5	8523361	8523386	2	13	26	TG	11	9
53983	5	8525693	8525713	3	7	21	CCT	12	4
53984	5	8525966	8526007	2	21	42	GA	8	8
53985	5	8527679	8527694	4	4	16	ATCT	7	3
53989	5	8529994	8530019	2	13	26	TG	18	9
53991	5	8532829	8532866	2	19	38	GT	17	16
53992	5	8533940	8533957	2	9	18	TG	27	5
53993	5	8534107	8534120	2	7	14	TG	20	3

53994	5	8536752	8536765	2	7	14	AC	24	5
53998	5	8537411	8537426	2	8	16	TG	6	3
54001	5	8544263	8544282	2	10	20	TG	22	8
54012	5	8558761	8558804	2	22	44	CA	11	12
54017	5	8568163	8568176	2	7	14	AC	22	5
54018	5	8569876	8569891	2	8	16	GT	13	4
54020	5	8570009	8570026	2	9	18	AG	21	5
54021	5	8570220	8570235	2	8	16	TG	20	12
54023	5	8573211	8573230	2	10	20	CA	10	9
54032	5	8581238	8581261	2	12	24	AC	13	6
54037	5	8588063	8588094	2	16	32	CA	12	11
54039	5	8590756	8590779	2	12	24	CA	13	8
54040	5	8591002	8591017	2	8	16	TC	18	5
54046	5	8594486	8594505	2	10	20	CA	17	9
54048	5	8596968	8596985	3	6	18	TCC	26	5
54049	5	8597037	8597068	2	16	32	GT	9	9
54050	5	8597094	8597123	2	15	30	TG	15	4
54051	5	8597676	8597709	2	17	34	CA	16	11
54060	5	8614012	8614043	2	16	32	TG	15	13
54061	5	8614437	8614480	2	22	44	TG	9	8
54063	5	8615613	8615632	2	10	20	AC	24	6
54064	5	8616281	8616298	2	9	18	AC	23	6
54065	5	8616948	8616969	2	11	22	GA	18	6
54085	5	8627104	8627149	2	23	46	GT	11	12
54086	5	8632115	8632138	2	12	24	GT	18	19
54096	5	8647834	8647848	3	5	15	CTC	17	3
54098	5	8648124	8648147	2	12	24	AC	19	3
54099	5	8648190	8648209	2	10	20	TG	14	7
54100	5	8649014	8649031	3	6	18	TCT	10	6
54102	5	8650663	8650694	2	16	32	AC	7	10
54103	5	8650790	8650821	2	16	32	AC	6	6
54108	5	8652892	8652915	2	12	24	TC	19	3
54109	5	8653031	8653050	2	10	20	CT	18	7
54110	5	8654609	8654644	2	18	36	CA	15	12
54113	5	8660268	8660291	2	12	24	AC	20	8
54121	5	8666511	8666528	2	9	18	CA	21	6
54122	5	8667128	8667145	2	9	18	AC	26	4
54125	5	8667516	8667545	2	15	30	CA	12	6
54132	5	8671621	8671632	2	6	12	TG	30	4
54133	5	8672094	8672111	3	6	18	GAA	27	3
54134	5	8673341	8673352	2	6	12	GT	19	4
54136	5	8674111	8674132	2	11	22	CA	15	5
54142	5	8682170	8682187	3	6	18	TCC	17	6
54143	5	8686732	8686755	2	12	24	CA	8	7
54145	5	8686903	8686920	3	6	18	TTC	17	3
54147	5	8688656	8688691	2	18	36	GT	8	12
54149	5	8694781	8694800	2	10	20	TG	23	5
54152	5	8703532	8703555	3	8	24	ACA	24	4
54154	5	8714033	8714056	3	8	24	GAG	20	5
54157	5	8715083	8715097	3	5	15	GAG	13	13
54161	5	8723223	8723240	3	6	18	CCA	24	4

54162	5	8725360	8725375	2	8	16	TG	17	5
54163	5	8725857	8725886	2	15	30	CA	9	10
54164	5	8726488	8726499	2	6	12	TG	22	3
54165	5	8726577	8726604	2	14	28	TG	17	10
54167	5	8732660	8732685	2	13	26	TG	10	8
54168	5	8732732	8732751	2	10	20	AG	20	11
54169	5	8733932	8733949	3	6	18	GGA	21	10
54170	5	8735742	8735771	2	15	30	CA	15	9
54173	5	8737114	8737133	2	10	20	TG	21	9
54175	5	8738541	8738561	3	7	21	TAA	18	3
54177	5	8740252	8740272	3	7	21	CCT	11	6
54179	5	8740514	8740533	2	10	20	CT	22	7
54185	5	8744863	8744874	2	6	12	TC	9	4
54191	5	8746680	8746700	3	7	21	GGA	23	7
54193	5	8750133	8750156	3	8	24	GCT	18	6
54194	5	8750737	8750754	2	9	18	GT	22	22
54198	5	8751915	8751942	2	14	28	CA	15	11
54199	5	8754392	8754403	2	6	12	TC	22	9
54200	5	8754692	8754707	2	8	16	CA	27	11
54202	5	8755079	8755099	3	7	21	GGA	19	4
54207	5	8760980	8761003	2	12	24	TC	30	7
54209	5	8765476	8765493	2	9	18	CA	16	13
54220	5	8780202	8780228	3	9	27	GGA	6	4
54222	5	8780435	8780449	3	5	15	TCC	16	11
54223	5	8782114	8782139	2	13	26	GT	10	7
54225	5	8782756	8782773	3	6	18	TGA	31	4
54229	5	8796173	8796198	2	13	26	GT	18	14
54231	5	8796739	8796766	2	14	28	AC	11	11
54235	5	8801212	8801237	2	13	26	TG	7	7
54236	5	8803582	8803615	2	17	34	GT	10	11
54240	5	8808503	8808522	2	10	20	TG	16	10
54243	5	8809764	8809775	2	6	12	GT	20	4
54245	5	8810721	8810738	2	9	18	GT	19	7
54246	5	8810918	8810937	2	10	20	TG	9	6
54247	5	8811121	8811146	2	13	26	GT	22	7
54248	5	8813705	8813720	2	8	16	GT	16	7
54263	5	8846063	8846078	4	4	16	GTCA	28	3
54264	5	8847839	8847860	2	11	22	AC	17	5
54272	5	8864494	8864511	2	9	18	AC	20	7
54276	5	8873634	8873653	4	5	20	CTAT	6	3
54279	5	8877946	8877960	3	5	15	GAA	16	4
54280	5	8879919	8879942	2	12	24	TG	20	12
54283	5	8880959	8880992	2	17	34	CA	13	8
54284	5	8881171	8881191	3	7	21	GGA	18	9
54285	5	8882261	8882284	3	8	24	AGG	8	9
54286	5	8883387	8883410	3	8	24	CCT	13	8
54290	5	8886607	8886672	3	22	66	AGA	16	15
54291	5	8888904	8888931	2	14	28	TG	15	10
54296	5	8900041	8900060	4	5	20	TTTC	7	7
54304	5	8903338	8903373	3	12	36	TGT	18	12
54305	5	8903516	8903539	3	8	24	CCT	17	10

54307	5	8906893	8906919	3	9	27	TCT	18	9
54309	5	8908803	8908820	2	9	18	GA	24	8
54311	5	8914144	8914165	2	11	22	CA	21	8
54314	5	8917201	8917232	2	16	32	TC	13	7
54315	5	8918723	8918737	3	5	15	AAG	31	6
54316	5	8920517	8920530	2	7	14	TG	20	5
54317	5	8922825	8922858	2	17	34	AC	17	11
54318	5	8923747	8923770	2	12	24	CA	22	12
54322	5	8929156	8929170	3	5	15	GCT	24	3
54324	5	8932944	8932967	2	12	24	AC	23	11
54326	5	8938238	8938252	3	5	15	CGC	28	3
54331	5	8940476	8940499	4	6	24	TATC	13	4
54332	5	8941015	8941052	2	19	38	CA	14	13
54333	5	8942327	8942344	2	9	18	TG	25	9
54335	5	8946875	8946889	3	5	15	GAG	17	3
54337	5	8949515	8949530	2	8	16	TG	22	3
54339	5	8949727	8949756	2	15	30	CA	13	8
54341	5	8953181	8953196	4	4	16	AATA	22	3
54342	5	8955722	8955733	2	6	12	GA	22	3
54345	5	8964984	8964995	2	6	12	AC	19	4
54351	5	8966731	8966754	2	12	24	AC	16	12
54358	5	8980404	8980415	2	6	12	CA	16	8
54359	5	8982316	8982339	3	8	24	CCT	23	5
54360	5	8984817	8984832	2	8	16	TA	14	5
54361	5	8986143	8986156	2	7	14	CA	12	8
54363	5	8989423	8989438	4	4	16	AAAT	30	4
54364	5	8994692	8994706	3	5	15	CTC	18	4
54367	5	9010980	9011003	2	12	24	TG	12	6
54370	5	9013533	9013546	2	7	14	TG	24	5
54376	5	9020655	9020676	2	11	22	GA	15	7
54379	5	9023429	9023444	4	4	16	GAGG	9	3
54383	5	9032690	9032711	2	11	22	AC	16	5
54385	5	9033917	9033928	2	6	12	AC	15	6
54391	5	9047575	9047586	2	6	12	AT	20	4
54394	5	9055905	9055919	3	5	15	AGG	26	3
54399	5	9062591	9062611	3	7	21	ACA	19	4
54401	5	9063912	9063931	2	10	20	AC	11	12
54402	5	9066821	9066841	3	7	21	CAG	24	3
54405	5	9067397	9067416	2	10	20	GT	21	14
54406	5	9067929	9067946	2	9	18	AT	15	10
54407	5	9068801	9068822	2	11	22	GT	24	10
54412	5	9075580	9075611	2	16	32	AC	13	8
54413	5	9075802	9075821	2	10	20	AC	20	4
54416	5	9077693	9077724	2	16	32	CA	15	13
54418	5	9077977	9077998	2	11	22	CT	15	5
54422	5	9082874	9082899	2	13	26	TG	12	8
54423	5	9083066	9083077	2	6	12	AG	28	6
54435	5	9085796	9085822	3	9	27	GGA	17	11
54436	5	9088078	9088099	2	11	22	GT	13	5
54437	5	9088202	9088231	2	15	30	CA	6	10
54439	5	9089092	9089166	3	25	75	GAA	15	14

54442	5	9091335	9091367	3	11	33	CTC	19	8
54443	5	9092028	9092051	2	12	24	TG	11	6
54446	5	9092241	9092264	2	12	24	TG	12	9
54447	5	9093161	9093172	2	6	12	GT	30	3
54450	5	9093255	9093266	2	6	12	CA	23	3
54451	5	9095327	9095344	3	6	18	AAG	18	5
54453	5	9096243	9096272	2	15	30	CA	7	12
54483	5	9123212	9123239	2	14	28	CA	14	11
54500	5	9135136	9135147	2	6	12	TC	22	7
54502	5	9135588	9135609	2	11	22	CA	19	5
54508	5	9136280	9136315	3	12	36	CCT	12	4
54510	5	9137195	9137224	3	10	30	AGG	9	4
54514	5	9142179	9142190	2	6	12	CA	24	3
54516	5	9146314	9146334	3	7	21	GGA	23	5
54517	5	9146638	9146651	2	7	14	GA	21	4
54518	5	9146761	9146776	4	4	16	GTCT	28	3
54519	5	9148271	9148284	2	7	14	CA	26	5
54520	5	9149682	9149719	2	19	38	GT	9	7
54521	5	9149941	9149962	2	11	22	AC	19	8
54522	5	9151634	9151648	3	5	15	GGA	21	3
54523	5	9152177	9152214	2	19	38	AG	11	12
54525	5	9154213	9154226	2	7	14	TC	26	4
54526	5	9157930	9157971	2	21	42	CA	11	12
54528	5	9159682	9159713	2	16	32	GT	12	10
54530	5	9160027	9160048	2	11	22	TG	17	5
54536	5	9163630	9163647	2	9	18	GT	19	11
54542	5	9165831	9165846	2	8	16	TG	11	10
54543	5	9166001	9166026	2	13	26	CA	7	7
54544	5	9167587	9167620	2	17	34	CA	11	12
54545	5	9168149	9168174	2	13	26	TG	16	11
54547	5	9170337	9170370	2	17	34	CA	12	6
54548	5	9170896	9170913	3	6	18	AAG	25	4
54549	5	9171177	9171196	4	5	20	TATT	27	5
54550	5	9172244	9172257	2	7	14	CA	22	3
54553	5	9174507	9174526	4	5	20	AGTC	27	3
54554	5	9174653	9174674	2	11	22	TG	24	17
54561	5	9192157	9192174	2	9	18	AC	20	9
54564	5	9197725	9197762	2	19	38	GT	20	11
54566	5	9199026	9199043	3	6	18	ACA	27	4
54572	5	9213387	9213410	2	12	24	AG	11	10
54580	5	9222609	9222626	2	9	18	GT	9	8
54582	5	9229191	9229217	3	9	27	AGG	23	6
54586	5	9231822	9231833	2	6	12	TG	21	5
54592	5	9244498	9244513	4	4	16	CTGT	24	3
54594	5	9248806	9248843	2	19	38	AC	8	9
54614	5	9272912	9272957	2	23	46	AC	12	12
54616	5	9278645	9278682	2	19	38	AC	9	8
54617	5	9279111	9279128	3	6	18	CAG	22	5
54619	5	9283557	9283568	2	6	12	AC	17	4
54620	5	9284262	9284277	2	8	16	AC	24	6
54621	5	9288361	9288400	2	20	40	GT	18	8

54624	5	9296786	9296809	4	6	24	TGTT	10	7
54625	5	9298029	9298044	2	8	16	CA	10	3
54628	5	9299485	9299508	3	8	24	TCT	13	9
54632	5	9302944	9302975	4	8	32	GTTA	20	6
54638	5	9325777	9325788	2	6	12	AT	11	3
54639	5	9336734	9336759	2	13	26	GT	14	11
54640	5	9338106	9338121	4	4	16	TTCT	25	3
54641	5	9341016	9341030	3	5	15	ACT	28	3
54643	5	9357224	9357239	2	8	16	CA	22	7
54645	5	9358954	9358971	3	6	18	CAT	22	8
54651	5	9371561	9371587	3	9	27	CCT	10	4
54652	5	9378199	9378210	2	6	12	TG	23	5
54655	5	9388975	9388992	2	9	18	TG	27	4
54658	5	9392365	9392384	4	5	20	AAAT	12	4
54666	5	9429753	9429768	2	8	16	AT	16	5
54670	5	9433689	9433720	2	16	32	AC	13	14
54671	5	9434617	9434666	2	25	50	TG	7	7
54674	5	9438588	9438625	2	19	38	GT	7	7
54677	5	9443016	9443031	2	8	16	CA	20	11
54679	5	9443170	9443199	2	15	30	GT	15	10
54680	5	9443313	9443340	2	14	28	CA	23	9
54681	5	9443426	9443443	2	9	18	TG	26	12
54682	5	9443664	9443681	2	9	18	AC	10	9
54685	5	9447731	9447742	2	6	12	CA	23	4
54686	5	9448348	9448359	2	6	12	CT	25	5
54690	5	9455853	9455876	2	12	24	CA	12	7
54692	5	9460112	9460127	2	8	16	CA	21	7
54706	5	9483571	9483582	2	6	12	TC	21	4
54707	5	9483666	9483691	2	13	26	AC	11	11
54710	5	9487313	9487328	2	8	16	AC	17	6
54723	5	9510200	9510223	3	8	24	CAG	30	4
54725	5	9520363	9520410	4	12	48	ATAG	19	11
54727	5	9535182	9535226	3	15	45	CCT	11	6
54734	5	9546778	9546798	3	7	21	GAG	27	3
54740	5	9562345	9562364	2	10	20	CA	12	8
54743	5	9565819	9565833	3	5	15	AGC	26	6
54746	5	9570734	9570748	3	5	15	CAG	10	3
54752	5	9621550	9621582	3	11	33	GAG	12	7
54753	5	9621645	9621660	2	8	16	GT	15	8
54754	5	9621663	9621680	2	9	18	GT	15	10
54756	5	9621991	9622026	4	9	36	TGGA	23	6
54757	5	9622374	9622393	2	10	20	TG	14	7
54764	5	9635702	9635727	2	13	26	TG	20	8
54770	5	9654728	9654751	3	8	24	TCC	20	3
54774	5	9667137	9667168	2	16	32	GT	18	11
54778	5	9670756	9670769	2	7	14	TG	20	5
54779	5	9670801	9670814	2	7	14	TG	22	7
54782	5	9673651	9673678	2	14	28	GT	8	9
54783	5	9676408	9676435	2	14	28	CA	23	8
54785	5	9688469	9688483	3	5	15	CAG	29	3
54787	5	9691151	9691168	2	9	18	CA	27	5

54789	5	9694199	9694214	2	8	16	CT	27	5
54792	5	9696290	9696307	3	6	18	CTC	20	6
54793	5	9697000	9697035	2	18	36	TG	11	11
54797	5	9707864	9707877	2	7	14	CA	18	8
54798	5	9707880	9707891	2	6	12	CA	18	5
54800	5	9710194	9710220	3	9	27	CTG	28	11
54801	5	9710295	9710309	3	5	15	GGA	27	4
54803	5	9711492	9711512	3	7	21	TTC	16	14
54805	5	9717148	9717163	4	4	16	TCCT	12	3
54806	5	9720235	9720248	2	7	14	AC	14	4
54807	5	9720263	9720274	2	6	12	AC	13	4
54809	5	9722712	9722727	2	8	16	GT	20	5
54811	5	9724653	9724666	2	7	14	AT	17	4
54814	5	9730406	9730425	4	5	20	TTCA	24	6
54816	5	9753535	9753550	4	4	16	AAAC	22	4
54818	5	9765628	9765645	2	9	18	AC	16	5
54829	5	9796948	9796965	2	9	18	TG	23	4
54833	5	9806133	9806174	3	14	42	GAA	19	12
54834	5	9807920	9807931	2	6	12	AC	27	3
54835	5	9808178	9808201	2	12	24	CA	21	6
54836	5	9808405	9808424	2	10	20	GA	19	3
54837	5	9811929	9811946	3	6	18	GCA	11	7
54840	5	9814179	9814199	3	7	21	GAG	19	8
54841	5	9814320	9814337	2	9	18	AG	21	3
54844	5	9815946	9815969	3	8	24	GGA	24	4
54854	5	9817571	9817582	2	6	12	AG	22	4
54863	5	9822637	9822652	2	8	16	TG	12	9
54866	5	9827818	9827841	2	12	24	GT	19	16
54867	5	9828713	9828726	2	7	14	CA	28	4
54872	5	9846911	9846924	2	7	14	TG	22	3
54873	5	9851175	9851189	3	5	15	CCT	28	3
54877	5	9873550	9873565	2	8	16	AG	30	6
54881	5	9875984	9876010	3	9	27	AGG	26	6
54882	5	9882070	9882083	2	7	14	AG	26	11
54883	5	9885935	9885955	3	7	21	CCT	27	6
54886	5	9894862	9894876	3	5	15	TTG	23	6
54887	5	9898557	9898580	3	8	24	TTC	18	6
54888	5	9898606	9898626	3	7	21	CTC	23	6
54891	5	9903075	9903100	2	13	26	AC	15	6
54892	5	9903428	9903451	3	8	24	GGA	13	4
54893	5	9909338	9909349	2	6	12	AC	20	8
54894	5	9910795	9910809	3	5	15	AGC	28	4
54897	5	9915712	9915723	2	6	12	GT	30	3
54898	5	9917328	9917357	2	15	30	TG	10	13
54899	5	9917576	9917593	2	9	18	GA	14	7
54900	5	9920041	9920078	2	19	38	AC	22	11
54903	5	9924814	9924829	2	8	16	AG	24	3
54906	5	9927776	9927789	2	7	14	GT	27	10
54907	5	9927791	9927802	2	6	12	TG	26	8
54908	5	9927947	9927984	2	19	38	CA	9	10
54909	5	9929116	9929135	4	5	20	ATCA	22	4

54911	5	9930974	9930994	3	7	21	ATA	8	3
54914	5	9940011	9940024	2	7	14	GA	28	5
54918	5	9941290	9941307	2	9	18	TC	15	5
54919	5	9941534	9941566	3	11	33	GGA	11	4
54923	5	9946809	9946826	2	9	18	AG	19	10
54924	5	9946828	9946845	2	9	18	GA	21	14
54927	5	9949572	9949604	3	11	33	GAG	7	4
54929	5	9951813	9951830	3	6	18	CTG	23	4
54930	5	9951949	9951972	2	12	24	TG	14	6
54931	5	9954083	9954112	3	10	30	TCC	20	3
54932	5	9955328	9955355	4	7	28	GAAG	24	10
54933	5	9956440	9956451	2	6	12	CA	32	4
54934	5	9956495	9956514	2	10	20	TG	18	9
54935	5	9956647	9956664	2	9	18	AG	22	10
54937	5	9959422	9959451	2	15	30	TG	8	7
54939	5	9961346	9961377	2	16	32	CA	19	15
54941	5	9967141	9967161	3	7	21	GGA	26	5
54942	5	9967637	9967650	2	7	14	TC	15	3
54946	5	9979468	9979483	2	8	16	CA	17	5
54948	5	9980942	9980971	2	15	30	AC	10	8
54949	5	9984228	9984251	2	12	24	CA	19	7
54952	5	9987994	9988009	2	8	16	TC	23	9
54953	5	9988010	9988025	2	8	16	AC	22	8
54954	5	9988584	9988597	2	7	14	GT	23	5
54957	5	9992638	9992653	2	8	16	CT	18	9
54962	5	9996616	9996630	3	5	15	CTC	25	6
54965	5	9999955	9999988	2	17	34	CA	15	9
54966	5	10002337	10002368	4	8	32	AAAG	12	10
54967	5	10003004	10003027	2	12	24	GT	15	9
54972	5	10007836	10007859	2	12	24	CA	22	14
54973	5	10014756	10014775	2	10	20	GT	14	8
54976	5	10019317	10019346	2	15	30	CA	13	9
54977	5	10023098	10023118	3	7	21	AGC	26	5
54981	5	10033414	10033429	4	4	16	AGGC	23	5
54982	5	10033973	10033994	2	11	22	AC	15	9
54992	5	10044182	10044201	2	10	20	AC	25	6
54993	5	10046280	10046317	2	19	38	CA	13	9
55011	5	10067024	10067051	2	14	28	GA	9	4
55016	5	10080109	10080124	2	8	16	CA	19	5
55019	5	10100280	10100291	2	6	12	GT	27	3
55022	5	10104045	10104076	2	16	32	AC	18	6
55026	5	10121715	10121770	2	28	56	TG	6	9
55028	5	10127033	10127054	2	11	22	GT	11	13
55032	5	10136301	10136312	2	6	12	CA	19	3
55034	5	10139792	10139815	3	8	24	TGC	31	3
55036	5	10142259	10142273	3	5	15	CCG	12	5
55041	5	10149949	10149976	2	14	28	GT	15	6
55042	5	10150176	10150191	2	8	16	AC	24	5
55043	5	10150332	10150357	2	13	26	TC	15	5
55044	5	10153027	10153046	2	10	20	GT	22	6
55045	5	10155127	10155140	2	7	14	CA	26	3

55046	5	10155321	10155344	2	12	24	AC	24	10
55048	5	10158286	10158299	2	7	14	GT	24	3
55049	5	10158375	10158422	2	24	48	TG	7	12
55053	5	10160775	10160796	2	11	22	CA	16	10
55055	5	10163179	10163210	2	16	32	AC	12	8
55056	5	10164141	10164162	2	11	22	TG	16	8
55060	5	10166279	10166314	2	18	36	GA	17	4
55063	5	10172230	10172247	2	9	18	TG	23	6
55064	5	10172452	10172479	2	14	28	GA	17	8
55065	5	10173445	10173456	2	6	12	AT	21	3
55071	5	10192317	10192350	2	17	34	TG	16	15
55073	5	10197071	10197118	4	12	48	TAGA	20	16
55076	5	10197646	10197657	2	6	12	AG	22	5
55078	5	10200503	10200526	2	12	24	CA	14	5
55079	5	10200578	10200629	2	26	52	AC	7	9
55083	5	10222352	10222367	2	8	16	CA	25	4
55085	5	10222724	10222735	2	6	12	CA	24	5
55087	5	10226750	10226769	2	10	20	TG	24	4
55097	5	10239039	10239060	2	11	22	GT	21	7
55098	5	10249571	10249590	2	10	20	TC	11	5
55100	5	10255184	10255231	2	24	48	TG	6	4
55105	5	10268286	10268301	2	8	16	GT	6	3
55106	5	10270933	10270968	2	18	36	TG	6	7
55108	5	10282576	10282607	4	8	32	AGAT	18	7
55123	5	10307618	10307632	3	5	15	TGT	26	3
55124	5	10308860	10308873	2	7	14	CA	11	12
55125	5	10319165	10319184	4	5	20	TTTG	8	6
55127	5	10323764	10323779	2	8	16	TG	23	4
55129	5	10324600	10324615	2	8	16	TG	26	3
55130	5	10330226	10330237	2	6	12	TG	26	3
55131	5	10336166	10336189	3	8	24	TTC	12	10
55134	5	10340720	10340747	2	14	28	AC	7	7
55137	5	10344683	10344706	3	8	24	GGA	13	8
55139	5	10355551	10355566	4	4	16	AAAC	29	3
55140	5	10356349	10356364	2	8	16	TG	19	4
55145	5	10370167	10370182	2	8	16	TA	16	3
55146	5	10372660	10372675	2	8	16	AT	13	4
55147	5	10373283	10373316	2	17	34	TG	13	10
55151	5	10377774	10377801	2	14	28	TG	14	6
55155	5	10382863	10382888	2	13	26	CA	7	10
55156	5	10383312	10383331	4	5	20	AAAT	15	3
55158	5	10387852	10387885	2	17	34	CA	20	12
55165	5	10403222	10403247	2	13	26	GT	19	11
55167	5	10406504	10406517	2	7	14	AT	14	3
55168	5	10414601	10414626	2	13	26	AC	23	5
55170	5	10424816	10424829	2	7	14	AC	21	5
55180	5	10451112	10451125	2	7	14	GT	17	8
55185	5	10455930	10455945	4	4	16	CAGT	30	3
55197	5	10494656	10494667	2	6	12	TC	29	3
55201	5	10502804	10502818	3	5	15	TTG	23	3
55202	5	10502900	10502931	2	16	32	AC	10	9

55205	5	10506304	10506315	2	6	12	GT	32	5
55206	5	10507735	10507768	2	17	34	AC	12	11
55208	5	10509045	10509068	2	12	24	AC	14	10
55216	5	10519840	10519857	3	6	18	AAC	36	3
55217	5	10522621	10522644	2	12	24	GT	19	4
55226	5	10546930	10546953	2	12	24	GT	14	11
55232	5	10555993	10556014	2	11	22	TC	17	10
55233	5	10556215	10556232	2	9	18	TG	6	3
55241	5	10563625	10563640	2	8	16	GT	27	6
55246	5	10575184	10575197	2	7	14	TG	6	5
55247	5	10575402	10575433	2	16	32	AC	25	8
55252	5	10583014	10583028	3	5	15	TTG	24	3
55253	5	10590360	10590374	3	5	15	TGC	27	3
55256	5	10601206	10601241	2	18	36	CA	16	11
55257	5	10601575	10601616	2	21	42	AG	8	10
55265	5	10617303	10617320	3	6	18	AAC	29	3
55268	5	10622347	10622374	2	14	28	GT	15	6
55270	5	10629789	10629802	2	7	14	AC	8	3
55271	5	10629831	10629858	2	14	28	CA	9	6
55277	5	10632683	10632700	2	9	18	AC	20	7
55283	5	10644492	10644513	2	11	22	TG	19	11
55287	5	10650018	10650035	2	9	18	AC	9	8
55288	5	10650950	10650961	2	6	12	GT	13	10
55289	5	10650963	10650978	2	8	16	TG	9	10
55294	5	10655146	10655173	2	14	28	AC	15	6
55297	5	10658942	10658965	2	12	24	TG	20	9
55301	5	10661703	10661720	3	6	18	GGA	18	3
55303	5	10664584	10664595	2	6	12	TA	26	3
55304	5	10665144	10665159	4	4	16	AAAT	30	3
55308	5	10666673	10666686	2	7	14	AC	20	4
55310	5	10667203	10667240	2	19	38	GA	13	11
55312	5	10667710	10667723	2	7	14	AC	30	4
55313	5	10670020	10670059	4	10	40	ATGG	23	11
55315	5	10672807	10672824	2	9	18	GT	22	5
55319	5	10676325	10676370	2	23	46	AG	14	11
55320	5	10676505	10676524	2	10	20	AG	8	5
55324	5	10680205	10680218	2	7	14	AC	15	4
55325	5	10680478	10680501	3	8	24	TCC	21	5
55330	5	10685115	10685126	2	6	12	AG	13	6
55331	5	10685129	10685140	2	6	12	AG	8	9
55332	5	10687231	10687256	2	13	26	AC	12	12
55333	5	10687555	10687568	2	7	14	GT	13	9
55334	5	10690757	10690774	3	6	18	AAT	22	5
55363	5	10761648	10761661	2	7	14	CA	14	3
55366	5	10768215	10768232	2	9	18	TG	24	4
55373	5	10781391	10781426	4	9	36	GAAA	15	9
55379	5	10794674	10794687	2	7	14	AT	12	15
55381	5	10796362	10796389	2	14	28	AC	16	10
55387	5	10817132	10817147	2	8	16	TG	14	5
55388	5	10821050	10821063	2	7	14	AC	21	5
55393	5	10829961	10829978	3	6	18	AAG	18	3

55397	5	10834577	10834626	2	25	50	GT	10	9
55400	5	10837905	10837920	2	8	16	AC	19	7
55413	5	10864971	10864986	2	8	16	TG	28	3
55415	5	10871476	10871487	2	6	12	GT	25	3
55416	5	10874464	10874487	3	8	24	ACA	20	4
55417	5	10874834	10874865	2	16	32	TG	16	10
55418	5	10879913	10879928	4	4	16	AAAT	31	5
55421	5	10885288	10885299	2	6	12	TA	19	5
55433	5	10901074	10901089	4	4	16	AAAT	10	5
55434	5	10901468	10901487	2	10	20	TG	17	5
55437	5	10905215	10905230	2	8	16	CA	23	4
55438	5	10905544	10905563	4	5	20	CAGA	26	3
55443	5	10911564	10911579	2	8	16	TG	7	5
55450	5	10919602	10919617	2	8	16	TG	15	5
55451	5	10919618	10919631	2	7	14	AG	13	5
55456	5	10925256	10925267	2	6	12	AC	29	4
55464	5	10939086	10939100	3	5	15	ATT	22	8
55473	5	10957413	10957442	2	15	30	AG	8	8
55475	5	10964147	10964176	3	10	30	TAT	15	4
55476	5	10966275	10966286	2	6	12	GA	29	3
55477	5	10966469	10966486	2	9	18	GT	19	5
55478	5	10966581	10966614	2	17	34	CA	16	17
55487	5	10993595	10993610	4	4	16	GAGT	22	5
55498	5	11008058	11008097	2	20	40	CA	17	13
55504	5	11031370	11031383	2	7	14	CA	12	4
55505	5	11033074	11033089	4	4	16	TAAA	14	7
55522	5	11085335	11085348	2	7	14	CA	26	5
55527	5	11100417	11100440	2	12	24	AC	18	7
55540	5	11133405	11133436	2	16	32	AC	6	8
55542	5	11134076	11134093	2	9	18	CA	25	5
55554	5	11144875	11144902	2	14	28	GT	15	7
55555	5	11145651	11145668	3	6	18	GGA	22	5
55556	5	11146146	11146177	2	16	32	TG	13	7
55558	5	11147890	11147907	2	9	18	TC	17	7
55559	5	11147908	11147923	2	8	16	AC	9	4
55560	5	11148243	11148266	2	12	24	AC	18	8
55563	5	11152309	11152324	2	8	16	TG	21	7
55564	5	11154963	11154980	2	9	18	TG	16	7
55565	5	11159070	11159089	2	10	20	GT	10	5
55567	5	11167262	11167279	2	9	18	TG	20	5
55569	5	11167831	11167842	2	6	12	AC	18	9
55575	5	11188121	11188177	3	19	57	CTT	18	9
55577	5	11191743	11191756	2	7	14	AC	26	4
55583	5	11204077	11204097	3	7	21	CCT	24	4
55584	5	11204414	11204425	2	6	12	CT	23	3
55586	5	11205014	11205025	2	6	12	AT	7	3
55589	5	11207776	11207805	3	10	30	TTA	10	11
55590	5	11210425	11210440	2	8	16	AC	21	4
55594	5	11215079	11215100	2	11	22	TG	12	9
55597	5	11221672	11221685	2	7	14	GT	22	5
55598	5	11221688	11221699	2	6	12	GT	24	4

55606	5	11238037	11238068	2	16	32	AC	14	6
55608	5	11251300	11251331	2	16	32	GT	14	5
55609	5	11262924	11262947	2	12	24	GT	10	10
55614	5	11271652	11271669	2	9	18	TG	14	6
55615	5	11273464	11273493	2	15	30	CA	6	7
55621	5	11275705	11275720	2	8	16	GA	6	5
55626	5	11292251	11292265	3	5	15	CTA	6	5
55630	5	11296047	11296060	2	7	14	GT	6	5
55631	5	11296123	11296142	2	10	20	TG	20	11
55636	5	11306132	11306155	4	6	24	TTAG	32	7
55650	5	11334213	11334228	2	8	16	TG	10	6
55651	5	11334231	11334244	2	7	14	TG	11	3
55652	5	11334435	11334446	2	6	12	TA	18	7
55659	5	11340243	11340260	2	9	18	TG	25	5
55663	5	11344493	11344514	2	11	22	TG	14	12
55666	5	11345018	11345069	4	13	52	GAAA	9	9
55668	5	11347401	11347418	2	9	18	CA	16	3
55669	5	11351430	11351455	2	13	26	TC	6	7
55671	5	11352731	11352744	2	7	14	CA	18	8
55672	5	11352746	11352759	2	7	14	AC	18	7
55674	5	11356283	11356302	2	10	20	TG	20	8
55676	5	11358627	11358654	2	14	28	TC	10	10
55677	5	11365813	11365834	2	11	22	CA	9	3
55678	5	11366006	11366019	2	7	14	CT	20	3
55679	5	11368337	11368351	3	5	15	TCC	19	3
55692	5	11379847	11379864	2	9	18	AC	17	8
55700	5	11387639	11387650	2	6	12	TG	15	10
55701	5	11387653	11387668	2	8	16	TG	12	6
55703	5	11390837	11390860	4	6	24	TAGA	25	6
55704	5	11396135	11396146	2	6	12	CA	24	4
55706	5	11398928	11398959	2	16	32	CA	9	10
55710	5	11410901	11410918	2	9	18	CA	20	4
55712	5	11414747	11414764	2	9	18	CA	24	5
55716	5	11421552	11421563	2	6	12	GA	18	5
55717	5	11421794	11421807	2	7	14	GT	27	4
55720	5	11425958	11425984	3	9	27	CCT	16	4
55723	5	11428050	11428085	2	18	36	AC	11	10
55726	5	11429592	11429607	2	8	16	GT	13	4
55727	5	11429609	11429622	2	7	14	GT	14	3
55728	5	11429776	11429807	2	16	32	AC	7	7
55729	5	11431250	11431265	4	4	16	TTTA	22	3
55730	5	11431559	11431578	2	10	20	CA	23	6
55734	5	11435647	11435668	2	11	22	CA	13	4
55739	5	11446859	11446890	2	16	32	TG	17	7
55742	5	11448132	11448159	2	14	28	AC	9	3
55745	5	11450078	11450109	2	16	32	TG	7	8
55747	5	11453756	11453771	2	8	16	GT	15	4
55750	5	11458220	11458257	2	19	38	GT	11	9
55753	5	11459178	11459201	2	12	24	AC	7	5
55755	5	11466242	11466256	3	5	15	CAG	20	3
55756	5	11466355	11466382	2	14	28	GT	8	9

55761	5	11472684	11472698	3	5	15	GGT	28	3
55771	5	11490678	11490699	2	11	22	AC	16	15
55774	5	11497390	11497405	4	4	16	GTCT	28	4
55779	5	11503577	11503590	2	7	14	TC	23	10
55780	5	11504165	11504194	2	15	30	CA	10	8
55784	5	11508399	11508416	2	9	18	AC	21	6
55786	5	11512546	11512565	2	10	20	TG	17	10
55792	5	11517070	11517096	3	9	27	CAG	31	4
55794	5	11519133	11519164	2	16	32	TG	18	11
55797	5	11523957	11523974	3	6	18	ACA	29	5
55803	5	11531290	11531313	4	6	24	TTTA	16	6
55809	5	11549855	11549872	2	9	18	TG	27	7
55810	5	11549874	11549885	2	6	12	GT	25	8
55817	5	11554978	11554993	2	8	16	AC	17	6
55828	5	11577688	11577707	2	10	20	CA	19	4
55830	5	11578704	11578731	2	14	28	GA	11	5
55844	5	11604912	11604941	3	10	30	TTG	16	9
55847	5	11613457	11613468	2	6	12	AC	24	3
55854	5	11630784	11630798	3	5	15	ATA	23	5
55862	5	11637855	11637882	2	14	28	TG	14	12
55871	5	11647763	11647777	3	5	15	CAA	24	3
55873	5	11649513	11649548	2	18	36	CT	12	7
55876	5	11650520	11650541	2	11	22	GT	15	9
55879	5	11651023	11651034	2	6	12	CT	23	3
55880	5	11651210	11651236	3	9	27	GGA	15	6
55881	5	11652425	11652442	3	6	18	AGA	20	3
55882	5	11653622	11653657	3	12	36	GAA	20	5
55884	5	11655550	11655569	2	10	20	AC	13	10
55888	5	11659618	11659647	3	10	30	TCT	22	9
55898	5	11667048	11667061	2	7	14	TC	28	3
55899	5	11667465	11667486	2	11	22	CA	18	6
55901	5	11668681	11668698	2	9	18	CA	17	5
55902	5	11668955	11668966	2	6	12	TG	28	5
55904	5	11670870	11670883	2	7	14	TG	30	5
55905	5	11676069	11676090	2	11	22	AG	21	7
55909	5	11680908	11680919	2	6	12	TG	13	11
55911	5	11681963	11681986	2	12	24	GT	18	10
55913	5	11683351	11683382	2	16	32	CA	19	6
55918	5	11687401	11687430	2	15	30	CA	20	15
55919	5	11689615	11689630	2	8	16	TC	20	13
55921	5	11690386	11690397	2	6	12	CA	23	17
55927	5	11694977	11694994	3	6	18	AGC	25	5
55930	5	11700155	11700174	2	10	20	AG	15	6
55933	5	11708245	11708262	2	9	18	TG	20	9
55938	5	11710588	11710627	2	20	40	AC	12	12
55940	5	11714342	11714377	2	18	36	GT	12	7
55945	5	11728617	11728632	2	8	16	GT	25	7
55947	5	11732201	11732240	2	20	40	AC	19	10
55950	5	11744156	11744203	2	24	48	TG	6	8
55953	5	11746377	11746392	4	4	16	TGTT	26	3
55956	5	11749660	11749671	2	6	12	CA	8	6

55957	5	11749943	11749976	2	17	34	CA	13	9
55968	5	11764949	11764974	2	13	26	AC	12	9
55972	5	11769111	11769122	2	6	12	AC	25	3
55975	5	11772987	11773016	2	15	30	AC	14	8
55976	5	11773753	11773764	2	6	12	CT	24	4
55977	5	11773766	11773777	2	6	12	TC	24	5
55981	5	11795358	11795381	3	8	24	TCC	18	5
55982	5	11797450	11797473	2	12	24	GT	23	6
55985	5	11802126	11802167	2	21	42	CA	13	9
55987	5	11812516	11812527	2	6	12	CA	14	4
55988	5	11812529	11812546	2	9	18	AC	14	3
56008	5	11861957	11861974	2	9	18	AC	21	3
56009	5	11862281	11862308	2	14	28	TG	21	11
56014	5	11876167	11876182	2	8	16	TG	16	10
56018	5	11879681	11879694	2	7	14	TG	31	4
56025	5	11888943	11888954	2	6	12	AC	29	4
56026	5	11889117	11889148	2	16	32	GT	13	9
56029	5	11904127	11904146	2	10	20	CA	18	7
56031	5	11904763	11904782	2	10	20	CA	7	5
56032	5	11905787	11905798	2	6	12	GT	28	3
56035	5	11909389	11909408	4	5	20	AACA	28	5
56038	5	11911379	11911394	2	8	16	AC	12	3
56040	5	11913923	11913950	2	14	28	TG	15	11
56042	5	11914192	11914215	2	12	24	AC	11	8
56047	5	11915755	11915778	2	12	24	GA	7	5
56050	5	11916679	11916704	2	13	26	TG	10	10
56055	5	11927913	11927928	2	8	16	GT	10	3
56058	5	11941741	11941766	2	13	26	TC	12	8
56059	5	11946529	11946552	2	12	24	GT	10	5
56060	5	11957257	11957284	2	14	28	AC	21	8
56061	5	11958177	11958194	3	6	18	TTG	22	5
56063	5	11965536	11965550	3	5	15	TGC	15	3
56078	5	12022215	12022236	2	11	22	AG	23	7
56081	5	12028371	12028396	2	13	26	AC	18	6
56083	5	12035025	12035045	3	7	21	TGC	28	8
56085	5	12037068	12037081	2	7	14	GT	34	3
56086	5	12038160	12038185	2	13	26	AC	12	14
56100	5	12053581	12053598	2	9	18	GT	11	9
56105	5	12071663	12071680	3	6	18	AGC	24	4
56108	5	12072165	12072178	2	7	14	AC	18	4
56113	5	12093488	12093501	2	7	14	CA	22	6
56114	5	12093567	12093584	2	9	18	AG	23	7
56117	5	12096164	12096201	2	19	38	AC	11	8
56121	5	12097767	12097786	4	5	20	CAAA	27	8
56123	5	12098946	12098981	2	18	36	CA	17	9
56128	5	12106707	12106724	3	6	18	TCA	29	8
56129	5	12107153	12107166	2	7	14	GT	30	7
56130	5	12110973	12110998	2	13	26	CA	13	9
56131	5	12111461	12111480	2	10	20	GT	9	6
56135	5	12128785	12128799	3	5	15	TCC	20	4
56136	5	12128803	12128820	3	6	18	TCC	18	5

56137	5	12131613	12131640	2	14	28	TG	22	8
56140	5	12133688	12133699	2	6	12	AT	20	9
56141	5	12133700	12133717	2	9	18	GT	19	9
56142	5	12133884	12133897	2	7	14	TG	18	11
56143	5	12133982	12133997	2	8	16	GT	23	5
56146	5	12139663	12139686	2	12	24	TG	16	9
56147	5	12142907	12142920	2	7	14	CA	17	5
56156	5	12160591	12160620	3	10	30	GCT	25	5
56158	5	12165691	12165706	4	4	16	CATA	29	3
56161	5	12179796	12179807	2	6	12	AC	27	3
56165	5	12188042	12188061	2	10	20	TG	20	6
56166	5	12188645	12188656	2	6	12	AC	31	3
56167	5	12189203	12189244	2	21	42	CA	10	8
56174	5	12191338	12191357	2	10	20	AG	11	6
56175	5	12191400	12191413	2	7	14	TG	16	5
56186	5	12212716	12212742	3	9	27	TGC	24	6
56190	5	12230898	12230911	2	7	14	AC	26	3
56191	5	12234758	12234797	2	20	40	AC	14	12
56193	5	12243452	12243469	2	9	18	AC	24	6
56195	5	12253401	12253421	3	7	21	AGG	16	4
56196	5	12253448	12253471	2	12	24	AG	12	3
56198	5	12254772	12254801	2	15	30	TG	16	13
56199	5	12254919	12254939	3	7	21	CCT	22	5
56201	5	12258076	12258089	2	7	14	GT	9	5
56202	5	12258092	12258109	2	9	18	GT	16	7
56204	5	12273745	12273758	2	7	14	GT	26	3
56205	5	12278231	12278258	2	14	28	GT	8	7
56213	5	12290840	12290857	2	9	18	TG	17	11
56214	5	12291273	12291302	3	10	30	GCT	21	8
56215	5	12291481	12291494	2	7	14	TG	27	7
56219	5	12297687	12297712	2	13	26	AC	13	3
56221	5	12303803	12303817	3	5	15	CAG	28	5
56223	5	12308963	12308978	2	8	16	CA	29	3
56226	5	12323022	12323039	2	9	18	TG	28	12
56228	5	12330873	12330900	2	14	28	CA	24	15
56232	5	12342282	12342319	2	19	38	AC	28	9
56235	5	12344849	12344860	2	6	12	TC	21	3
56236	5	12345220	12345237	3	6	18	GAA	22	6
56240	5	12356471	12356484	2	7	14	GT	25	11
56242	5	12358106	12358119	2	7	14	CA	26	4
56245	5	12360062	12360077	2	8	16	CT	26	10
56246	5	12360222	12360236	3	5	15	AAG	15	5
56247	5	12372575	12372596	2	11	22	CA	16	9
56253	5	12380307	12380333	3	9	27	CAT	21	7
56258	5	12399065	12399078	2	7	14	CA	24	8
56259	5	12399137	12399160	2	12	24	AC	20	7
56261	5	12399347	12399358	2	6	12	TC	12	10
56267	5	12400986	12400997	2	6	12	GA	10	10
56268	5	12401399	12401431	3	11	33	GAA	24	10
56283	5	12432972	12432989	3	6	18	CTC	26	5
56285	5	12443602	12443631	3	10	30	ACA	27	8

56287	5	12447012	12447038	3	9	27	CTC	29	6
56288	5	12448597	12448623	3	9	27	AAG	23	3
56290	5	12452116	12452127	2	6	12	GA	23	5
56293	5	12461276	12461291	2	8	16	TA	8	4
56295	5	12462051	12462062	2	6	12	CA	16	6
56298	5	12463992	12464015	2	12	24	TG	9	8
56300	5	12466986	12467019	2	17	34	TG	13	15
56301	5	12468698	12468711	2	7	14	AG	30	3
56307	5	12473409	12473420	2	6	12	TG	15	6
56310	5	12478477	12478528	4	13	52	TCTA	18	13
56312	5	12490062	12490079	3	6	18	GCA	25	5
56314	5	12492373	12492384	2	6	12	AC	25	3
56318	5	12497321	12497336	4	4	16	TCTT	27	5
56320	5	12497646	12497663	3	6	18	CCG	15	5
56321	5	12497709	12497726	3	6	18	GCT	23	3
56322	5	12498122	12498137	2	8	16	TC	23	6
56323	5	12498182	12498205	4	6	24	ATGG	25	6
56324	5	12498470	12498483	2	7	14	GT	20	8
56329	5	12505183	12505197	3	5	15	GGA	9	6
56332	5	12511942	12511953	2	6	12	TG	29	7
56333	5	12513317	12513331	3	5	15	TGT	31	5
56336	5	12518832	12518859	4	7	28	ATTT	20	4
56337	5	12521884	12521899	4	4	16	TGAA	30	8
56340	5	12524733	12524756	2	12	24	GT	11	8
56342	5	12530833	12530856	4	6	24	AATC	31	6
56351	5	12542130	12542147	2	9	18	GT	11	6
56353	5	12545242	12545267	2	13	26	TG	19	8
56355	5	12547228	12547243	2	8	16	CT	17	4
56361	5	12556047	12556060	2	7	14	TG	26	11
56362	5	12556124	12556135	2	6	12	TG	15	5
56365	5	12562964	12562978	3	5	15	CTG	21	3
56366	5	12563003	12563020	3	6	18	CCT	6	3
56367	5	12563327	12563348	2	11	22	CA	28	16
56368	5	12568011	12568036	2	13	26	GT	16	5
56370	5	12571794	12571833	2	20	40	GT	16	10
56371	5	12572163	12572182	2	10	20	TG	21	6
56375	5	12582756	12582776	3	7	21	CAT	31	5
56376	5	12585026	12585041	4	4	16	CCAT	24	4
56379	5	12589357	12589368	2	6	12	CA	26	3
56380	5	12591421	12591452	2	16	32	GT	6	10
56381	5	12591561	12591622	2	31	62	GT	12	13
56382	5	12591773	12591800	2	14	28	CA	20	11
56383	5	12601228	12601239	2	6	12	TG	28	3
56384	5	12601381	12601396	2	8	16	GT	12	10
56385	5	12601398	12601409	2	6	12	TG	17	11
56386	5	12601573	12601608	2	18	36	TG	10	12
56387	5	12603870	12603883	2	7	14	AC	20	7
56393	5	12608439	12608454	4	4	16	TTTA	11	7
56394	5	12612127	12612148	2	11	22	TG	22	6
56397	5	12615986	12616015	2	15	30	CA	16	9
56399	5	12621340	12621351	2	6	12	CA	21	4

56401	5	12623962	12623975	2	7	14	TG	29	3
56402	5	12624021	12624038	2	9	18	TG	18	5
56406	5	12629239	12629268	3	10	30	CCT	13	7
56412	5	12634744	12634775	2	16	32	TG	12	9
56413	5	12635954	12635965	2	6	12	GT	32	3
56414	5	12641852	12641881	2	15	30	CA	8	8
56415	5	12645284	12645325	2	21	42	TG	14	10
56423	5	12653904	12653923	2	10	20	TG	19	9
56424	5	12654079	12654102	2	12	24	GT	25	15
56434	5	12668690	12668701	2	6	12	TG	12	3
56437	5	12674306	12674339	2	17	34	CA	6	6
56438	5	12676851	12676864	2	7	14	AC	29	13
56439	5	12677146	12677165	2	10	20	AC	18	7
56440	5	12677924	12677935	2	6	12	AG	24	3
56441	5	12678339	12678380	2	21	42	TC	15	6
56442	5	12679260	12679299	2	20	40	TG	9	13
56444	5	12680639	12680656	2	9	18	TG	20	7
56445	5	12680912	12680929	2	9	18	CT	17	6
56446	5	12684323	12684346	4	6	24	CTTC	26	3
56448	5	12686675	12686686	2	6	12	TG	19	4
56452	5	12688143	12688156	2	7	14	GT	27	4
56453	5	12688394	12688419	2	13	26	TG	12	7
56455	5	12688565	12688600	2	18	36	TG	8	9
56456	5	12689058	12689073	2	8	16	TG	27	5
56458	5	12692547	12692560	2	7	14	GT	25	6
56459	5	12694212	12694229	2	9	18	AC	11	7
56461	5	12695812	12695825	2	7	14	GA	17	6
56462	5	12695827	12695838	2	6	12	AG	16	7
56463	5	12703215	12703236	2	11	22	CA	23	12
56467	5	12704976	12704996	3	7	21	GAA	31	4
56470	5	12708852	12708869	3	6	18	CTT	23	3
56475	5	12712537	12712548	2	6	12	CA	23	3
56477	5	12720139	12720156	3	6	18	CTC	25	4
56478	5	12721235	12721252	2	9	18	GT	20	13
56481	5	12733113	12733160	2	24	48	TG	9	10
56486	5	12737863	12737896	2	17	34	AC	7	9
56489	5	12745063	12745076	2	7	14	TC	21	3
56490	5	12745268	12745285	2	9	18	TG	11	11
56492	5	12749595	12749614	2	10	20	TC	13	8
56494	5	12754214	12754253	2	20	40	GT	13	13
56497	5	12754669	12754682	2	7	14	AC	20	9
56498	5	12754684	12754699	2	8	16	AC	17	4
56500	5	12756654	12756675	2	11	22	AC	16	15
56512	5	12770841	12770858	3	6	18	CCT	23	9
56525	5	12801334	12801353	2	10	20	CA	17	9
56534	5	12808938	12808981	2	22	44	TG	11	11
56542	5	12820459	12820478	4	5	20	GTTT	28	5
56544	5	12821399	12821418	2	10	20	TG	11	8
56554	5	12838698	12838713	4	4	16	CAAA	23	3
56555	5	12840026	12840041	2	8	16	AC	27	9
56556	5	12843113	12843124	2	6	12	AC	26	3

56568	5	12883431	12883442	2	6	12	GA	22	4
56569	5	12883478	12883491	2	7	14	AC	28	4
56572	5	12885384	12885401	2	9	18	AC	16	4
56578	5	12903706	12903721	2	8	16	TC	28	3
56580	5	12906239	12906259	3	7	21	TAT	14	12
56582	5	12907557	12907580	2	12	24	AT	14	7
56583	5	12910002	12910013	2	6	12	AC	17	9
56584	5	12910016	12910027	2	6	12	AC	13	7
56589	5	12911101	12911120	4	5	20	AATA	20	3
56594	5	12920774	12920791	2	9	18	GA	20	9
56596	5	12927386	12927407	2	11	22	GA	14	4
56597	5	12928057	12928076	2	10	20	TC	20	3
56598	5	12928611	12928646	2	18	36	CA	10	11
56599	5	12936868	12936882	3	5	15	AAC	8	11
56606	5	12945167	12945192	2	13	26	GT	10	9
56607	5	12946963	12946980	2	9	18	CA	17	4
56610	5	12949328	12949363	3	12	36	CAG	26	9
56612	5	12953053	12953084	2	16	32	CA	8	5
56615	5	12956367	12956398	2	16	32	TG	7	9
56630	5	12985462	12985477	2	8	16	GT	17	3
56632	5	12987789	12987826	2	19	38	CA	15	14
56633	5	12988794	12988825	2	16	32	TG	12	11
56634	5	12989162	12989173	2	6	12	TG	27	3
56636	5	12991172	12991195	2	12	24	GA	16	11
56654	5	12998710	12998727	2	9	18	AC	26	15
56655	5	12999899	12999962	4	16	64	TCTA	15	11
56657	5	13001256	13001277	2	11	22	GA	17	5
56658	5	13001873	13001904	2	16	32	GT	13	9
56659	5	13002060	13002077	3	6	18	TTG	25	4
56667	5	13005451	13005468	3	6	18	TTC	17	4
56668	5	13006597	13006617	3	7	21	TGT	10	3
56669	5	13006668	13006697	2	15	30	GT	8	6
56674	5	13007760	13007771	2	6	12	AC	20	4
56675	5	13007803	13007818	2	8	16	AC	18	8
56684	5	13011480	13011503	2	12	24	TG	15	12
56694	5	13013773	13013800	2	14	28	TG	13	7
56695	5	13013834	13013857	2	12	24	TG	15	4
56697	5	13014647	13014674	4	7	28	TCTG	19	3
56716	5	13030480	13030500	3	7	21	TCC	16	6
56724	5	13037835	13037852	2	9	18	GT	25	3
56725	5	13041497	13041508	2	6	12	TG	20	12
56726	5	13041511	13041524	2	7	14	TG	17	8
56727	5	13041919	13041930	2	6	12	TC	20	3
56732	5	13043749	13043772	4	6	24	GTTG	26	3
56736	5	13049979	13050004	2	13	26	CA	11	16
56747	5	13061033	13061050	2	9	18	TG	15	8
56757	5	13076058	13076089	2	16	32	AC	6	9
56758	5	13077285	13077300	2	8	16	TG	13	3
56759	5	13077386	13077405	4	5	20	TTTC	17	3
56760	5	13077622	13077636	3	5	15	CTC	29	6
56761	5	13078026	13078043	3	6	18	AAG	25	3

56762	5	13078334	13078369	2	18	36	TG	25	14
56764	5	13079053	13079072	2	10	20	GT	7	6
56765	5	13079106	13079125	4	5	20	TTTC	23	5
56766	5	13079725	13079739	3	5	15	TAT	14	4
56768	5	13083234	13083247	2	7	14	GA	30	4
56769	5	13085558	13085573	2	8	16	AG	24	5
56775	5	13089164	13089189	2	13	26	GT	17	8
56779	5	13094536	13094555	2	10	20	CA	24	5
56780	5	13094920	13094937	2	9	18	CA	17	4
56781	5	13095350	13095361	2	6	12	AC	19	4
56782	5	13095364	13095377	2	7	14	AC	24	5
56783	5	13095925	13095952	4	7	28	CTTT	20	7
56786	5	13098527	13098550	2	12	24	TG	19	4
56789	5	13098723	13098754	2	16	32	CT	7	4
56790	5	13099582	13099615	2	17	34	AC	9	9
56803	5	13117651	13117664	2	7	14	AG	10	3
56804	5	13117720	13117737	3	6	18	AGA	19	4
56807	5	13121430	13121459	3	10	30	AGG	11	7
56808	5	13122068	13122094	3	9	27	GAG	12	5
56811	5	13129580	13129597	2	9	18	TC	14	6
56812	5	13129667	13129692	2	13	26	CT	15	7
56813	5	13130269	13130292	3	8	24	GCA	21	10
56819	5	13137283	13137298	4	4	16	TCAC	26	3
56824	5	13143838	13143865	2	14	28	TC	12	10
56826	5	13146226	13146237	2	6	12	TG	14	4
56831	5	13168390	13168403	2	7	14	CT	22	3
56838	5	13178030	13178044	3	5	15	GCA	15	3
56840	5	13179779	13179808	2	15	30	GT	13	9
56841	5	13182374	13182407	2	17	34	AC	11	14
56842	5	13182528	13182543	2	8	16	GT	19	10
56844	5	13185682	13185703	2	11	22	GT	20	17
56846	5	13193641	13193670	2	15	30	TG	8	10
56847	5	13196161	13196172	2	6	12	CA	23	11
56849	5	13196214	13196227	2	7	14	TG	26	6
56850	5	13196334	13196355	2	11	22	CA	27	7
56851	5	13198107	13198126	2	10	20	AG	8	3
56852	5	13200548	13200561	2	7	14	TG	12	6
56856	5	13204972	13204987	2	8	16	TG	14	4
56857	5	13208923	13208934	2	6	12	TG	26	7
56861	5	13216845	13216870	2	13	26	CA	11	15
56863	5	13221256	13221267	2	6	12	AG	28	3
56864	5	13224504	13224525	2	11	22	CA	15	12
56865	5	13230367	13230384	2	9	18	CA	20	3
56868	5	13230739	13230762	2	12	24	AC	18	9
56869	5	13235305	13235320	2	8	16	CA	26	6
56870	5	13236085	13236108	2	12	24	AC	16	5
56871	5	13236735	13236746	2	6	12	TG	20	5
56872	5	13238574	13238595	2	11	22	TG	12	4
56873	5	13239307	13239326	2	10	20	GT	15	13
56874	5	13239672	13239685	2	7	14	AC	32	3
56876	5	13243183	13243196	2	7	14	AG	31	5

56878	5	13245781	13245796	4	4	16	AGAC	37	3
56882	5	13250201	13250216	2	8	16	GA	16	10
56884	5	13253272	13253283	2	6	12	TG	30	6
56885	5	13254892	13254921	2	15	30	AC	15	11
56888	5	13256775	13256808	2	17	34	CA	12	13
56901	5	13271062	13271073	2	6	12	AG	25	3
56904	5	13271823	13271837	3	5	15	TTG	16	5
56908	5	13282286	13282303	2	9	18	TA	25	5
56909	5	13284217	13284231	3	5	15	ATA	29	3
56915	5	13292748	13292759	2	6	12	TC	22	5
56919	5	13309882	13309899	3	6	18	GCT	21	3
56925	5	13315175	13315190	2	8	16	TG	20	10
56926	5	13318969	13318986	3	6	18	GCT	26	5
56931	5	13321609	13321620	2	6	12	TG	27	4
56934	5	13326788	13326813	2	13	26	GT	11	8
56940	5	13343738	13343752	3	5	15	TGC	27	4
56941	5	13344565	13344578	2	7	14	AC	16	6
56944	5	13356901	13356914	2	7	14	TG	23	5
56945	5	13361388	13361403	4	4	16	CCTC	22	3
56958	5	13390150	13390170	3	7	21	GAG	16	6
56959	5	13390545	13390558	2	7	14	CA	31	5
56960	5	13391267	13391288	2	11	22	AC	11	5
56961	5	13394171	13394210	4	10	40	GCCT	25	9
56965	5	13404684	13404699	2	8	16	GT	18	4
56973	5	13412106	13412121	2	8	16	CA	11	5
56982	5	13426462	13426475	2	7	14	CA	18	11
56988	5	13430789	13430810	2	11	22	GA	14	6
56999	5	13436925	13436956	2	16	32	AG	9	7
57010	5	13448601	13448614	2	7	14	TG	31	4
57018	5	13457641	13457654	2	7	14	TA	16	8
57021	5	13459543	13459558	4	4	16	TTTG	17	3
57027	5	13486122	13486141	2	10	20	TG	26	3
57029	5	13488223	13488256	2	17	34	GT	13	8
57033	5	13499482	13499493	2	6	12	GT	30	4
57034	5	13500022	13500049	4	7	28	TTCT	30	3
57045	5	13523984	13523999	2	8	16	GT	19	7
57046	5	13524104	13524119	2	8	16	CA	25	5
57047	5	13524451	13524462	2	6	12	GT	19	10
57049	5	13526249	13526266	2	9	18	TC	13	9
57050	5	13526450	13526463	2	7	14	GT	19	6
57051	5	13526699	13526734	2	18	36	GT	8	12
57055	5	13533546	13533573	2	14	28	AC	26	5
57057	5	13536863	13536880	3	6	18	TGC	30	3
57061	5	13543596	13543625	2	15	30	CA	9	8
57066	5	13553561	13553581	3	7	21	GAA	13	9
57070	5	13566830	13566849	2	10	20	GT	13	7
57071	5	13572115	13572132	2	9	18	TG	23	3
57072	5	13573897	13573914	2	9	18	GT	16	3
57075	5	13574329	13574342	2	7	14	TC	11	5
57076	5	13574451	13574480	2	15	30	CT	10	4
57082	5	13579790	13579807	2	9	18	CT	25	3

57086	5	13587301	13587318	2	9	18	AC	18	4
57093	5	13602816	13602847	2	16	32	CA	12	11
57100	5	13613669	13613710	2	21	42	AC	7	7
57102	5	13613985	13614002	2	9	18	GT	25	5
57103	5	13615441	13615454	2	7	14	CA	19	5
57104	5	13615602	13615629	2	14	28	GT	17	11
57105	5	13621079	13621094	2	8	16	TG	27	3
57106	5	13626084	13626111	2	14	28	TG	22	8
57109	5	13629708	13629731	2	12	24	GT	26	7
57111	5	13631064	13631077	2	7	14	TG	27	5
57112	5	13634425	13634446	2	11	22	CA	25	11
57113	5	13639299	13639320	2	11	22	AC	18	13
57114	5	13644100	13644117	3	6	18	TTC	20	5
57122	5	13662808	13662831	2	12	24	AC	17	13
57125	5	13669268	13669291	2	12	24	TG	7	6
57127	5	13672048	13672063	2	8	16	GA	30	6
57129	5	13673982	13673997	4	4	16	TTCC	35	3
57131	5	13677274	13677291	2	9	18	TG	23	3
57135	5	13707075	13707086	2	6	12	CA	28	3
57140	5	13721339	13721366	2	14	28	TG	19	13
57141	5	13721554	13721571	3	6	18	GAA	18	12
57142	5	13726461	13726502	3	14	42	AAG	14	10
57145	5	13733162	13733182	3	7	21	TGA	22	6
57151	5	13756820	13756837	2	9	18	GT	22	3
57154	5	13760971	13760982	2	6	12	AC	26	4
57155	5	13761847	13761864	3	6	18	AGA	20	14
57157	5	13766495	13766524	2	15	30	CA	14	9
57159	5	13775714	13775725	2	6	12	GT	28	5
57162	5	13783529	13783555	3	9	27	GGA	30	8
57164	5	13788627	13788652	2	13	26	GT	23	13
57171	5	13816715	13816736	2	11	22	TC	15	3
57173	5	13816969	13816980	2	6	12	CT	15	4
57176	5	13828580	13828591	2	6	12	GT	31	4
57201	5	13841114	13841135	2	11	22	GT	23	7
57209	5	13846535	13846549	3	5	15	TCC	18	3
57210	5	13846953	13846972	2	10	20	CA	18	7
57211	5	13849142	13849159	2	9	18	AC	24	4
57212	5	13849192	13849219	2	14	28	CA	28	14
57216	5	13859200	13859220	3	7	21	TTG	24	8
57228	5	13873222	13873233	2	6	12	TC	20	5
57237	5	13875665	13875700	2	18	36	TG	7	12
57246	5	13885215	13885242	2	14	28	CA	17	10
57248	5	13885993	13886032	2	20	40	AC	10	6
57250	5	13892482	13892511	2	15	30	CT	18	6
57251	5	13893011	13893031	3	7	21	AAG	23	4
57253	5	13893196	13893235	2	20	40	CA	19	15
57257	5	13897359	13897372	2	7	14	TC	20	5
57264	5	13903188	13903207	4	5	20	CTGT	35	3
57265	5	13904579	13904590	2	6	12	CA	28	7
57266	5	13907645	13907658	2	7	14	CA	25	4
57273	5	13926381	13926410	3	10	30	CTG	26	7

57279	5	13928984	13929007	2	12	24	CA	19	10
57288	5	13943220	13943245	2	13	26	GT	6	6
57293	5	13959889	13959918	2	15	30	CA	19	11
57294	5	13963949	13963960	2	6	12	TG	27	3
57299	5	13972526	13972551	2	13	26	TG	22	8
57305	5	13984857	13984872	2	8	16	AG	27	3
57309	5	13990963	13990982	4	5	20	TTAT	16	4
57311	5	13995150	13995165	2	8	16	GT	14	7
57316	5	14001690	14001705	4	4	16	TAGT	23	3
57318	5	14003223	14003240	2	9	18	CT	19	3
57320	5	14007908	14007925	3	6	18	TCC	18	12
57339	5	14034894	14034909	2	8	16	TG	21	7
57340	5	14038520	14038537	3	6	18	CAG	24	4
57348	5	14054758	14054769	2	6	12	TG	23	4
57355	5	14071225	14071240	2	8	16	AC	12	10
57357	5	14077153	14077166	2	7	14	CA	13	4
57359	5	14080904	14080919	2	8	16	AG	21	3
57360	5	14084747	14084768	2	11	22	TG	18	8
57362	5	14091542	14091571	2	15	30	GT	17	9
57364	5	14101628	14101643	4	4	16	TCCC	11	3
57368	5	14107248	14107259	2	6	12	GT	13	4
57381	5	14133738	14133752	3	5	15	AGG	29	3
57382	5	14136018	14136037	2	10	20	TG	22	7
57384	5	14150765	14150784	2	10	20	AC	23	7
57390	5	14155435	14155448	2	7	14	CA	16	4
57391	5	14155906	14155939	2	17	34	GT	13	14
57402	5	14171794	14171809	2	8	16	TG	20	3
57404	5	14179012	14179035	3	8	24	GTT	11	7
57409	5	14183627	14183642	4	4	16	CTTA	15	3
57410	5	14184939	14184956	2	9	18	GT	27	5
57412	5	14187893	14187908	4	4	16	ATCA	32	3
57413	5	14198355	14198380	2	13	26	AC	10	8
57414	5	14204215	14204235	3	7	21	GGA	25	5
57416	5	14206628	14206647	2	10	20	GT	8	5
57424	5	14225145	14225160	4	4	16	GAGT	30	5
57426	5	14225300	14225317	2	9	18	GT	18	10
57427	5	14225523	14225552	2	15	30	AC	23	11
57431	5	14228123	14228134	2	6	12	GT	14	5
57433	5	14229062	14229076	3	5	15	TCC	31	3
57434	5	14233014	14233031	2	9	18	CA	21	10
57440	5	14241324	14241353	2	15	30	CA	18	10
57441	5	14242955	14242981	3	9	27	CAT	10	6
57442	5	14243724	14243737	2	7	14	TC	22	10
57443	5	14243902	14243915	2	7	14	AC	24	5
57444	5	14247699	14247710	2	6	12	AT	21	8
57451	5	14264738	14264755	2	9	18	GT	13	8
57452	5	14265377	14265416	2	20	40	GT	8	13
57455	5	14267910	14267927	3	6	18	AAC	31	3
57456	5	14269307	14269327	3	7	21	TCC	25	6
57460	5	14293874	14293888	3	5	15	AGC	16	7
57466	5	14312924	14312957	2	17	34	TG	12	7

57474	5	14332760	14332771	2	6	12	AC	19	3
57475	5	14341503	14341538	2	18	36	AC	13	14
57478	5	14344276	14344311	4	9	36	ATAC	19	9
57479	5	14345870	14345905	4	9	36	CACT	27	8
57481	5	14357015	14357030	4	4	16	CAGA	28	4
57486	5	14368716	14368741	2	13	26	GT	14	14
57487	5	14371405	14371436	2	16	32	AC	11	9
57488	5	14371750	14371761	2	6	12	AG	26	3
57489	5	14378721	14378740	4	5	20	GCTC	28	3
57491	5	14387743	14387782	2	20	40	GT	17	8
57492	5	14388280	14388293	2	7	14	TG	20	3
57493	5	14388524	14388539	2	8	16	AC	23	3
57494	5	14390803	14390852	2	25	50	CA	11	10
57495	5	14390909	14390920	2	6	12	AC	26	3
57497	5	14392898	14392917	2	10	20	TG	19	5
57498	5	14393262	14393285	2	12	24	GA	14	5
57499	5	14393316	14393330	3	5	15	GGA	21	3
57500	5	14393451	14393480	2	15	30	AC	17	9
57502	5	14394337	14394364	2	14	28	AC	10	9
57503	5	14394537	14394556	2	10	20	CT	23	6
57504	5	14394666	14394695	2	15	30	AC	10	9
57505	5	14394799	14394810	2	6	12	TG	23	3
57508	5	14397051	14397064	2	7	14	AC	25	4
57509	5	14403340	14403351	2	6	12	TC	25	3
57510	5	14403542	14403561	2	10	20	GT	20	8
57513	5	14403784	14403803	2	10	20	AC	23	4
57515	5	14404078	14404099	2	11	22	CA	15	8
57516	5	14404290	14404301	2	6	12	TG	23	4
57522	5	14404995	14405008	2	7	14	TG	20	13
57523	5	14405150	14405165	2	8	16	CA	26	9
57527	5	14409985	14410000	2	8	16	GA	16	3
57531	5	14411006	14411033	2	14	28	AC	20	10
57534	5	14411361	14411384	2	12	24	TG	16	5
57535	5	14413438	14413452	3	5	15	GTT	24	3
57540	5	14420050	14420061	2	6	12	GT	22	3
57541	5	14420213	14420234	2	11	22	TG	20	10
57543	5	14420448	14420463	2	8	16	TG	21	5
57544	5	14420843	14420868	2	13	26	AC	23	7
57546	5	14424765	14424782	2	9	18	TG	28	7
57547	5	14424851	14424868	2	9	18	GT	17	6
57551	5	14430099	14430136	2	19	38	TG	18	12
57553	5	14430652	14430671	2	10	20	GT	20	5
57555	5	14430741	14430770	2	15	30	AC	17	7
57556	5	14430798	14430811	2	7	14	CA	25	4
57562	5	14437198	14437209	2	6	12	TG	25	3
57568	5	14447675	14447701	3	9	27	GAG	14	7
57569	5	14447821	14447832	2	6	12	GA	22	6
57575	5	14455816	14455863	2	24	48	TG	13	9
57581	5	14483518	14483551	2	17	34	GA	19	8
57584	5	14487120	14487171	2	26	52	TG	6	11
57587	5	14488636	14488659	2	12	24	AC	10	5

57589	5	14489066	14489089	2	12	24	CA	17	11
57590	5	14489265	14489282	2	9	18	CA	21	9
57593	5	14494126	14494147	2	11	22	GT	18	10
57594	5	14494759	14494774	2	8	16	TG	17	3
57600	5	14498101	14498120	2	10	20	GA	23	4
57601	5	14498195	14498212	2	9	18	TC	24	4
57602	5	14498969	14498994	2	13	26	GT	10	7
57603	5	14502040	14502053	2	7	14	TG	22	3
57625	5	14517890	14517913	4	6	24	TCTG	14	4
57629	5	14520924	14520935	2	6	12	CA	14	4
57631	5	14523957	14523982	2	13	26	TG	18	6
57633	5	14531180	14531217	2	19	38	TG	6	8
57634	5	14533837	14533870	2	17	34	AC	11	8
57637	5	14538578	14538599	2	11	22	GT	14	6
57639	5	14540474	14540489	2	8	16	CA	17	8
57640	5	14543099	14543120	2	11	22	GT	18	6
57641	5	14543335	14543346	2	6	12	TA	28	6
57654	5	14553487	14553508	2	11	22	TC	12	4
57655	5	14554332	14554351	2	10	20	TG	16	3
57656	5	14554537	14554570	2	17	34	AC	6	7
57657	5	14554672	14554705	2	17	34	GT	10	8
57658	5	14555953	14555992	2	20	40	CA	16	13
57660	5	14560064	14560075	2	6	12	AT	16	4
57663	5	14567762	14567777	2	8	16	CA	24	5
57665	5	14570555	14570570	2	8	16	AC	22	5
57666	5	14570573	14570584	2	6	12	AC	19	6
57673	5	14590414	14590435	2	11	22	CA	20	7
57678	5	14604380	14604397	3	6	18	AGC	26	5
57679	5	14610557	14610572	4	4	16	TATT	22	5
57680	5	14610942	14610957	4	4	16	GCTA	31	3
57681	5	14611338	14611358	3	7	21	TCA	26	7
57682	5	14611688	14611705	2	9	18	GT	18	6
57683	5	14614863	14614878	2	8	16	TC	21	3
57690	5	14672418	14672432	3	5	15	GAA	21	8
57692	5	14676654	14676677	2	12	24	TG	14	9
57694	5	14678558	14678571	2	7	14	AG	22	3
57695	5	14685195	14685209	3	5	15	GCC	18	4
57700	5	14690904	14690924	3	7	21	GGA	32	8
57703	5	14693179	14693211	3	11	33	TCC	21	9
57706	5	14693405	14693416	2	6	12	GA	34	3
57707	5	14693604	14693631	4	7	28	TTCA	16	5
57708	5	14693792	14693806	3	5	15	TGG	29	3
57710	5	14694184	14694204	3	7	21	AAT	20	6
57711	5	14697079	14697093	3	5	15	CCT	18	6
57713	5	14699350	14699365	2	8	16	CA	28	3
57714	5	14701834	14701851	3	6	18	GAT	31	8
57717	5	14707047	14707061	3	5	15	AAG	30	5
57718	5	14711055	14711069	3	5	15	AGC	27	3
57721	5	14722578	14722597	2	10	20	TG	10	5
57722	5	14722725	14722750	2	13	26	CA	9	6
57723	5	14725687	14725706	4	5	20	TTGG	23	5

57726	5	14732431	14732442	2	6	12	CT	9	4
57727	5	14732711	14732726	4	4	16	CTCC	8	3
57732	5	14748609	14748641	3	11	33	TCT	15	9
57736	5	14759422	14759457	2	18	36	AC	16	9
57740	5	14763339	14763350	2	6	12	TA	22	5
57741	5	14772142	14772163	2	11	22	CA	15	8
57750	5	14794637	14794654	3	6	18	CAC	32	8
57751	5	14794716	14794778	3	21	63	CTT	19	22
57755	5	14800123	14800134	2	6	12	GA	17	6
57760	5	14809497	14809526	3	10	30	TCC	17	5
57765	5	14812448	14812468	3	7	21	TGT	28	11
57766	5	14813748	14813775	2	14	28	CA	25	12
57767	5	14816177	14816196	2	10	20	GT	25	4
57768	5	14816234	14816248	3	5	15	TGC	31	4
57771	5	14826921	14826966	2	23	46	TG	6	8
57773	5	14827105	14827132	2	14	28	CA	15	18
57774	5	14828135	14828156	2	11	22	AC	15	7
57784	5	14843090	14843111	2	11	22	TG	13	7
57785	5	14843321	14843332	2	6	12	GT	19	7
57788	5	14844591	14844612	2	11	22	GT	20	9
57789	5	14850933	14850956	3	8	24	ACA	24	7
57790	5	14851473	14851487	3	5	15	GGA	16	5
57794	5	14855909	14855924	4	4	16	GCGA	21	7
57798	5	14863053	14863068	2	8	16	TG	20	3
57805	5	14881579	14881592	2	7	14	AC	18	3
57816	5	14895795	14895809	3	5	15	TTG	8	5
57827	5	14903292	14903315	2	12	24	AG	11	6
57829	5	14903781	14903798	3	6	18	AAT	17	4
57840	5	14916406	14916423	2	9	18	TG	14	14
57841	5	14916425	14916436	2	6	12	GT	15	13
57842	5	14916630	14916643	2	7	14	AC	23	6
57843	5	14916646	14916661	2	8	16	AC	21	6
57844	5	14916784	14916831	2	24	48	AC	10	11
57845	5	14916940	14916965	2	13	26	CA	18	14
57848	5	14926351	14926372	2	11	22	TG	13	7
57849	5	14935515	14935535	3	7	21	GAG	22	8
57850	5	14937919	14937939	3	7	21	GCA	29	6
57855	5	14946686	14946701	2	8	16	CT	27	3
57858	5	14952877	14952894	3	6	18	AAG	31	3
57860	5	14954280	14954291	2	6	12	TG	14	7
57861	5	14954460	14954485	2	13	26	GT	19	9
57865	5	14963871	14963886	2	8	16	TG	15	12
57867	5	14966316	14966363	4	12	48	CCAT	23	10
57868	5	14967806	14967823	3	6	18	AAC	11	3
57870	5	14969813	14969842	2	15	30	CA	10	10
57875	5	14979579	14979596	3	6	18	CAG	30	3
57876	5	14980050	14980076	3	9	27	AGC	27	6
57879	5	14980294	14980331	2	19	38	GT	9	12
57880	5	14980444	14980475	2	16	32	GT	15	14
57881	5	14981678	14981689	2	6	12	AC	20	3
57882	5	14983652	14983673	2	11	22	GT	19	14

57883	5	14983771	14983786	4	4	16	TATC	27	6
57884	5	14983891	14983906	2	8	16	CA	8	7
57885	5	14983909	14983922	2	7	14	CA	18	12
57886	5	14984083	14984103	3	7	21	TCT	21	7
57888	5	14985277	14985306	3	10	30	CTC	8	6
57892	5	14987883	14987912	3	10	30	TCT	20	8
57894	5	14991916	14991929	2	7	14	CT	19	5
57900	5	15000402	15000425	3	8	24	CCT	27	11
57901	5	15000599	15000616	3	6	18	AGG	21	13
57902	5	15005952	15005973	2	11	22	TG	15	13
57906	5	15007680	15007703	2	12	24	GT	17	11
57910	5	15009028	15009049	2	11	22	GT	19	7
57913	5	15011920	15011947	2	14	28	CA	18	10
57915	5	15012437	15012466	2	15	30	TG	20	6
57919	5	15015022	15015048	3	9	27	CTG	29	6
57920	5	15015208	15015239	2	16	32	AC	15	11
57921	5	15015440	15015459	2	10	20	CA	22	3
57923	5	15018568	15018585	3	6	18	AGA	15	4
57927	5	15021439	15021454	4	4	16	TTTA	27	6
57930	5	15026417	15026431	3	5	15	AAG	11	7
57937	5	15031725	15031742	2	9	18	TG	17	6
57938	5	15031797	15031816	2	10	20	CA	21	9
57941	5	15032517	15032536	2	10	20	GT	15	9
57946	5	15033681	15033694	2	7	14	CA	22	4
57947	5	15034389	15034408	2	10	20	CA	18	10
57951	5	15041205	15041216	2	6	12	TG	26	11
57952	5	15042111	15042126	2	8	16	GT	28	4
57954	5	15043518	15043550	3	11	33	CCT	23	9
57958	5	15044702	15044721	2	10	20	GT	10	5
57960	5	15048124	15048159	3	12	36	CTC	25	7
57968	5	15054743	15054763	3	7	21	CTC	16	3
57972	5	15060938	15060955	2	9	18	GT	21	10
57974	5	15065031	15065063	3	11	33	CAT	19	12
57975	5	15065092	15065113	2	11	22	TC	16	8
57978	5	15065632	15065649	2	9	18	TC	14	3
57979	5	15065771	15065790	2	10	20	TC	24	6
57980	5	15066151	15066176	2	13	26	CA	9	7
57981	5	15066252	15066271	2	10	20	TC	11	5
57983	5	15067401	15067422	2	11	22	GA	23	6
57984	5	15069479	15069496	2	9	18	CA	18	3
57991	5	15072665	15072679	3	5	15	GCC	20	5
57992	5	15072809	15072828	4	5	20	TTTA	18	6
57994	5	15074079	15074090	2	6	12	GA	16	4
57997	5	15075516	15075547	2	16	32	GT	7	7
57999	5	15076332	15076349	2	9	18	CA	8	6
58000	5	15079304	15079327	3	8	24	CTT	19	5
58001	5	15079518	15079535	3	6	18	CAG	31	10
58004	5	15081662	15081677	2	8	16	AC	17	11
58006	5	15083076	15083093	3	6	18	GCT	29	3
58007	5	15089527	15089544	2	9	18	GT	20	4
58010	5	15091453	15091468	2	8	16	AC	13	8

58011	5	15091745	15091762	2	9	18	AG	16	3
58012	5	15092378	15092392	3	5	15	AAG	19	4
58014	5	15099228	15099242	3	5	15	GCA	23	4
58015	5	15099407	15099420	2	7	14	CA	21	5
58023	5	15119267	15119304	2	19	38	AC	13	14
58027	5	15121568	15121585	2	9	18	CA	21	7
58029	5	15125738	15125761	2	12	24	GT	11	10
58030	5	15125794	15125805	2	6	12	GT	13	3
58031	5	15125833	15125848	2	8	16	TG	20	5
58034	5	15126072	15126089	2	9	18	TC	13	3
58035	5	15126152	15126185	2	17	34	AC	6	9
58044	5	15131821	15131832	2	6	12	GT	25	9
58045	5	15131834	15131853	2	10	20	TG	26	12
58050	5	15133100	15133123	2	12	24	AC	19	6
58051	5	15135808	15135829	2	11	22	CA	7	7
58058	5	15136919	15136942	3	8	24	TTG	16	3
58065	5	15156001	15156018	2	9	18	CA	23	5
58066	5	15157088	15157115	2	14	28	CA	18	18
58067	5	15158326	15158341	2	8	16	AG	21	3
58068	5	15158530	15158551	2	11	22	GT	18	12
58074	5	15161805	15161822	3	6	18	CTT	19	4
58075	5	15163343	15163354	2	6	12	CT	31	9
58076	5	15163499	15163524	2	13	26	GT	13	7
58078	5	15163585	15163614	2	15	30	CA	9	7
58080	5	15164079	15164112	2	17	34	TG	8	10
58087	5	15166022	15166049	2	14	28	GT	13	15
58088	5	15168184	15168201	3	6	18	GAT	25	5
58090	5	15168612	15168627	4	4	16	ATAA	26	4
58091	5	15169847	15169874	2	14	28	AC	18	15
58100	5	15172714	15172727	2	7	14	AT	13	6
58101	5	15173315	15173334	2	10	20	GT	15	14
58102	5	15173469	15173486	2	9	18	CA	19	11
58103	5	15173511	15173522	2	6	12	CA	31	5
58104	5	15173711	15173736	2	13	26	AC	15	6
58109	5	15180555	15180572	2	9	18	CA	11	4
58112	5	15181841	15181856	2	8	16	AC	13	6
58113	5	15181899	15181910	2	6	12	AG	23	3
58114	5	15182009	15182040	2	16	32	CA	9	7
58117	5	15184628	15184641	2	7	14	AC	30	3
58120	5	15186326	15186337	2	6	12	CA	12	14
58121	5	15186339	15186352	2	7	14	AC	13	13
58122	5	15186625	15186660	2	18	36	TG	6	7
58125	5	15188117	15188134	2	9	18	TG	16	9
58127	5	15198181	15198202	2	11	22	CA	17	7
58129	5	15198894	15198913	4	5	20	TTTC	19	9
58130	5	15199702	15199717	2	8	16	TG	14	8
58131	5	15201684	15201695	2	6	12	CA	32	6
58132	5	15202939	15202958	2	10	20	GT	24	6
58134	5	15205956	15205970	3	5	15	CTC	25	5
58135	5	15206291	15206306	2	8	16	TG	28	3
58136	5	15206892	15206913	2	11	22	CA	19	4

58141	5	15212458	15212473	2	8	16	TC	22	7
58142	5	15212927	15212941	3	5	15	GAT	20	3
58145	5	15216904	15216927	2	12	24	CA	14	11
58147	5	15217585	15217600	2	8	16	TG	19	3
58153	5	15226009	15226024	2	8	16	CT	6	6
58158	5	15232882	15232897	2	8	16	GA	24	6
58161	5	15237582	15237621	2	20	40	TC	18	16
58166	5	15248243	15248256	2	7	14	CT	26	3
58168	5	15250007	15250036	2	15	30	TG	15	13
58169	5	15253499	15253514	2	8	16	TG	9	3
58176	5	15262495	15262510	2	8	16	AG	22	7
58179	5	15264666	15264685	2	10	20	CA	24	5
58180	5	15268831	15268842	2	6	12	AT	6	5
58181	5	15269287	15269312	2	13	26	CA	9	5
58190	5	15277278	15277305	2	14	28	TG	11	11
58191	5	15277466	15277480	3	5	15	AGA	11	6
58198	5	15284129	15284149	3	7	21	GCC	21	8
58203	5	15288014	15288027	2	7	14	CT	25	4
58205	5	15296404	15296431	2	14	28	CA	16	10
58206	5	15298405	15298416	2	6	12	CA	26	5
58216	5	15306332	15306361	2	15	30	GT	19	12
58220	5	15310560	15310591	2	16	32	CA	8	9
58221	5	15310752	15310765	2	7	14	CA	23	4
58222	5	15311415	15311435	3	7	21	GAA	13	4
58223	5	15311688	15311699	2	6	12	TG	20	4
58224	5	15311788	15311825	2	19	38	GT	16	12
58225	5	15311925	15311944	2	10	20	AC	15	4
58227	5	15323521	15323538	2	9	18	CA	14	7
58229	5	15327858	15327869	2	6	12	GA	19	3
58230	5	15330621	15330638	2	9	18	GT	26	4
58232	5	15332850	15332889	2	20	40	AC	8	10
58238	5	15347295	15347314	2	10	20	AC	23	11
58239	5	15347812	15347829	3	6	18	GAG	18	6
58241	5	15347941	15347962	2	11	22	AC	13	4
58243	5	15348138	15348153	2	8	16	GT	7	5
58244	5	15348289	15348320	2	16	32	GT	17	12
58246	5	15349813	15349836	2	12	24	AC	12	7
58247	5	15352001	15352018	2	9	18	CA	7	4
58248	5	15352469	15352482	2	7	14	GT	21	14
58249	5	15352485	15352496	2	6	12	GT	17	12
58251	5	15357391	15357404	2	7	14	TC	27	3
58252	5	15358048	15358081	2	17	34	AC	8	7
58266	5	15362657	15362670	2	7	14	TG	21	4
58273	5	15364557	15364576	2	10	20	GT	17	8
58279	5	15367387	15367410	2	12	24	AC	10	11
58284	5	15367940	15367954	3	5	15	TCT	22	3
58289	5	15369211	15369242	2	16	32	CA	7	10
58291	5	15369630	15369656	3	9	27	TTC	8	4
58295	5	15370200	15370219	2	10	20	CA	14	9
58298	5	15371003	15371022	2	10	20	GT	9	9
58299	5	15371276	15371293	2	9	18	CA	24	6

58303	5	15372240	15372263	3	8	24	GGA	7	6
58307	5	15374884	15374899	2	8	16	GA	15	4
58313	5	15375523	15375548	2	13	26	AC	9	4
58315	5	15375725	15375754	3	10	30	CTC	6	6
58328	5	15391201	15391224	3	8	24	GAG	23	8
58330	5	15391557	15391574	2	9	18	CT	20	4
58332	5	15392376	15392403	2	14	28	TG	6	6
58334	5	15392912	15392932	3	7	21	GAG	18	6
58335	5	15393108	15393143	2	18	36	GT	15	7
58336	5	15393479	15393502	2	12	24	TC	6	3
58339	5	15394774	15394791	2	9	18	GT	22	5
58340	5	15396524	15396535	2	6	12	GT	8	6
58341	5	15396538	15396551	2	7	14	GT	21	6
58343	5	15397061	15397078	2	9	18	AC	15	7
58344	5	15397267	15397287	3	7	21	GCG	29	14
58348	5	15399433	15399447	3	5	15	CGC	19	7
58350	5	15401660	15401683	3	8	24	GAG	18	3
58351	5	15403410	15403430	3	7	21	AGG	23	5
58353	5	15403768	15403791	2	12	24	AC	16	9
58358	5	15405752	15405766	3	5	15	CCG	8	9
58359	5	15406946	15406981	2	18	36	GA	18	9
58360	5	15407007	15407020	2	7	14	GA	6	3
58361	5	15407443	15407458	4	4	16	TTTA	20	13
58365	5	15411435	15411454	2	10	20	AG	21	5
58366	5	15412580	15412597	3	6	18	GCC	17	10
58367	5	15412808	15412822	3	5	15	CCT	23	6
58370	5	15415060	15415087	2	14	28	GT	13	9
58372	5	15416975	15416990	2	8	16	AC	12	10
58383	5	15426465	15426476	2	6	12	TG	29	3
58384	5	15428270	15428293	2	12	24	TG	14	10
58385	5	15428345	15428364	2	10	20	CA	7	5
58386	5	15428366	15428379	2	7	14	AC	8	7
58387	5	15428446	15428489	2	22	44	TG	7	9
58390	5	15430570	15430591	2	11	22	AC	13	4
58391	5	15430651	15430692	2	21	42	CA	14	11
58393	5	15430837	15430860	2	12	24	CA	11	11
58394	5	15430994	15431031	2	19	38	GT	11	9
58395	5	15431176	15431201	2	13	26	GT	18	10
58398	5	15431283	15431312	3	10	30	GGA	20	5
58399	5	15431377	15431408	2	16	32	CA	12	9
58401	5	15432796	15432807	2	6	12	CA	18	4
58402	5	15432810	15432821	2	6	12	CA	17	5
58406	5	15433864	15433893	3	10	30	CTC	26	9
58407	5	15434742	15434771	2	15	30	CA	15	9
58409	5	15436395	15436422	2	14	28	AC	10	9
58410	5	15437214	15437234	3	7	21	GAG	19	4
58419	5	15440002	15440017	2	8	16	GT	16	10
58427	5	15446912	15446929	3	6	18	CTT	22	5
58429	5	15448170	15448193	2	12	24	TG	16	6
58430	5	15448399	15448434	2	18	36	TC	9	10
58431	5	15449000	15449013	2	7	14	TC	26	3

58433	5	15450267	15450298	2	16	32	GT	13	11
58449	5	15478421	15478447	3	9	27	TCC	21	5
58450	5	15479270	15479295	2	13	26	GT	17	10
58457	5	15482532	15482547	2	8	16	TG	26	11
58458	5	15482713	15482727	3	5	15	GGC	15	5
58460	5	15484176	15484193	3	6	18	GCA	25	11
58463	5	15489577	15489590	2	7	14	AG	21	7
58465	5	15491693	15491720	2	14	28	GT	28	12
58473	5	15500709	15500722	2	7	14	AC	19	5
58477	5	15507257	15507282	2	13	26	AC	15	5
58478	5	15507379	15507390	2	6	12	AC	29	9
58479	5	15507568	15507599	2	16	32	CA	9	8
58481	5	15522403	15522417	3	5	15	CAA	22	3
58482	5	15526007	15526020	2	7	14	GT	22	5
58483	5	15526050	15526073	2	12	24	GT	16	14
58486	5	15526931	15526963	3	11	33	TCT	24	6
58488	5	15528839	15528870	2	16	32	CA	11	10
58492	5	15530227	15530240	2	7	14	TA	7	4
58497	5	15535768	15535789	2	11	22	CA	27	9
58499	5	15536738	15536755	2	9	18	GT	8	5
58501	5	15542896	15542915	2	10	20	CA	18	13
58502	5	15544470	15544490	3	7	21	CTT	27	5
58504	5	15545981	15546010	2	15	30	TG	21	10
58505	5	15546060	15546087	2	14	28	GT	15	10
58507	5	15546627	15546641	3	5	15	AGG	21	11
58508	5	15546911	15546934	2	12	24	TG	19	8
58509	5	15546991	15547026	2	18	36	TG	8	11
58510	5	15548935	15548946	2	6	12	GA	29	5
58517	5	15554082	15554096	3	5	15	GGT	17	3
58518	5	15554258	15554271	2	7	14	GT	21	4
58519	5	15554295	15554306	2	6	12	TG	19	3
58524	5	15556202	15556229	2	14	28	GT	21	7
58525	5	15556369	15556388	2	10	20	GT	19	12
58527	5	15556589	15556606	2	9	18	AC	15	10
58528	5	15557923	15557938	2	8	16	CA	24	4
58530	5	15563837	15563869	3	11	33	TCT	12	7
58532	5	15564265	15564302	2	19	38	GT	6	9
58533	5	15564416	15564437	2	11	22	TG	15	13
58536	5	15564954	15564971	3	6	18	GCT	23	5
58539	5	15567238	15567249	2	6	12	CA	20	5
58542	5	15569799	15569838	2	20	40	GT	9	9
58545	5	15572433	15572453	3	7	21	CCT	21	13
58546	5	15573886	15573900	3	5	15	TCC	30	5
58550	5	15576942	15576956	3	5	15	GGT	14	5
58553	5	15578883	15578902	2	10	20	AC	19	7
58555	5	15579029	15579052	3	8	24	CCT	8	8
58556	5	15579854	15579869	4	4	16	TCTT	13	5
58562	5	15586997	15587012	2	8	16	TG	10	11
58567	5	15590524	15590535	2	6	12	AC	27	6
58571	5	15593132	15593143	2	6	12	TA	24	7
58579	5	15604557	15604590	2	17	34	CA	9	13

58582	5	15607574	15607588	3	5	15	CTT	27	8
58585	5	15613353	15613367	3	5	15	AGA	17	6
58586	5	15613368	15613382	3	5	15	GGA	15	5
58587	5	15619603	15619620	3	6	18	GCC	19	3
58592	5	15620857	15620889	3	11	33	CCT	15	7
58595	5	15622932	15622947	4	4	16	GGTT	15	6
58598	5	15626752	15626763	2	6	12	GA	26	4
58599	5	15628929	15628943	3	5	15	ACC	28	3
58602	5	15633402	15633428	3	9	27	GCT	26	8
58605	5	15635931	15635948	2	9	18	AC	12	5
58606	5	15636004	15636029	2	13	26	AC	21	10
58608	5	15639175	15639206	2	16	32	GT	13	9
58609	5	15639355	15639382	2	14	28	CA	15	15
58612	5	15640340	15640354	3	5	15	CAC	9	3
58613	5	15640587	15640628	2	21	42	CA	9	10
58614	5	15640757	15640794	2	19	38	GT	15	15
58617	5	15642034	15642065	2	16	32	CA	13	8
58618	5	15642233	15642254	2	11	22	TG	21	11
58620	5	15642318	15642343	2	13	26	GT	14	13
58621	5	15642921	15642968	2	24	48	TG	6	8
58624	5	15643257	15643276	2	10	20	AC	6	4
58625	5	15643313	15643354	2	21	42	GT	13	12
58643	5	15659518	15659538	3	7	21	CTT	10	6
58646	5	15664366	15664389	3	8	24	CCT	17	11
58648	5	15666174	15666203	2	15	30	AC	22	11
58652	5	15669121	15669138	3	6	18	GAA	25	7
58653	5	15670955	15670966	2	6	12	TG	17	3
58655	5	15670993	15671008	2	8	16	TG	20	4
58656	5	15672242	15672256	3	5	15	GGA	22	14
58658	5	15672685	15672705	3	7	21	AGA	21	6
58659	5	15674480	15674495	4	4	16	GAGG	11	3
58661	5	15675922	15675941	2	10	20	AC	17	3
58663	5	15677217	15677231	3	5	15	TAA	14	10
58665	5	15680958	15680984	3	9	27	CAT	20	7
58666	5	15681263	15681276	2	7	14	AC	17	4
58667	5	15681466	15681489	2	12	24	GT	22	8
58670	5	15682476	15682502	3	9	27	GAA	26	5
58676	5	15687479	15687512	2	17	34	AC	6	7
58677	5	15689098	15689112	3	5	15	GAT	24	3
58684	5	15693543	15693569	3	9	27	TCC	17	6
58686	5	15693937	15693957	3	7	21	GAG	14	9
58687	5	15695324	15695337	2	7	14	TC	29	7
58691	5	15698670	15698685	4	4	16	ACAG	25	5
58694	5	15701906	15701920	3	5	15	GAG	26	7
58695	5	15704122	15704137	4	4	16	CCTC	15	7
58699	5	15712550	15712561	2	6	12	GT	16	9
58703	5	15718067	15718093	3	9	27	CTT	16	7
58706	5	15718299	15718319	3	7	21	GAG	14	8
58707	5	15718481	15718495	3	5	15	GCG	24	4
58709	5	15720275	15720292	3	6	18	CTG	6	4
58710	5	15721809	15721838	3	10	30	CCT	21	9

58712	5	15726676	15726708	3	11	33	TCC	19	8
58714	5	15726970	15726989	2	10	20	AC	17	13
58716	5	15730825	15730858	2	17	34	AC	17	11
58719	5	15731091	15731118	2	14	28	TG	19	12
58720	5	15732049	15732076	2	14	28	TG	17	9
58721	5	15734588	15734607	2	10	20	CA	15	12
58735	6	42769	42789	3	7	21	ATA	25	3
58739	6	49905	49916	2	6	12	CA	20	5
58750	6	79944	79970	3	9	27	TTA	6	3
58761	6	97575	97588	2	7	14	CA	24	3
58771	6	117281	117292	2	6	12	AC	19	3
58774	6	129495	129506	2	6	12	CA	29	3
58775	6	129868	129885	2	9	18	AT	6	3
58781	6	146057	146096	2	20	40	GA	6	3
58792	6	171357	171384	2	14	28	TG	14	6
58793	6	174526	174543	2	9	18	TG	21	7
58798	6	180652	180687	4	9	36	CCAA	16	4
58822	6	238196	238239	4	11	44	GAAA	14	7
58826	6	256629	256661	3	11	33	AAC	20	4
58830	6	266229	266246	2	9	18	TG	16	3
58834	6	286546	286565	2	10	20	AC	13	3
58847	6	322019	322066	4	12	48	TATC	14	10
58862	6	354146	354175	3	10	30	AAT	12	7
58888	6	438156	438189	2	17	34	AC	12	5
58891	6	444016	444031	4	4	16	AAAC	23	6
58895	6	450007	450022	2	8	16	TG	21	5
58911	6	483968	484001	2	17	34	GT	16	7
58917	6	502737	502752	2	8	16	CT	28	3
58942	6	587094	587109	2	8	16	CA	11	3
58948	6	594345	594380	2	18	36	AG	10	7
58952	6	601602	601623	2	11	22	AC	22	3
58972	6	633477	633488	2	6	12	TG	15	4
58973	6	633503	633516	2	7	14	TG	22	3
59001	6	671284	671327	2	22	44	TG	10	9
59020	6	703392	703407	4	4	16	AAAT	19	5
59022	6	705718	705739	2	11	22	AC	17	3
59042	6	746859	746872	2	7	14	TG	20	3
59045	6	751120	751135	2	8	16	GT	8	4
59057	6	768307	768342	2	18	36	GA	12	6
59072	6	789498	789529	4	8	32	TGTC	17	4
59073	6	790226	790239	2	7	14	AT	8	3
59088	6	811714	811727	2	7	14	GT	32	3
59096	6	829477	829496	2	10	20	GT	27	3
59108	6	841050	841063	2	7	14	CA	23	3
59123	6	868983	868996	2	7	14	GT	24	4
59134	6	900169	900216	4	12	48	GATG	12	6
59135	6	900287	900318	2	16	32	TC	6	7
59137	6	902422	902449	2	14	28	AT	15	6
59145	6	914892	914907	2	8	16	GT	26	4
59150	6	930815	930828	2	7	14	AC	24	5
59154	6	942477	942508	4	8	32	TGTT	25	7

59155	6	945673	945698	2	13	26	AG	21	5
59164	6	963768	963783	4	4	16	AATG	12	3
59186	6	987146	987159	2	7	14	TA	8	4
59198	6	1020940	1020959	4	5	20	CATA	29	4
59209	6	1035073	1035086	2	7	14	AT	11	3
59227	6	1088769	1088780	2	6	12	TG	26	4
59235	6	1103101	1103118	2	9	18	AC	19	3
59245	6	1128937	1128958	2	11	22	CA	16	12
59248	6	1131153	1131174	2	11	22	CA	13	5
59251	6	1136540	1136569	2	15	30	AC	13	8
59252	6	1139701	1139714	2	7	14	GA	18	3
59257	6	1149670	1149691	2	11	22	TG	19	8
59265	6	1168864	1168893	2	15	30	CA	17	7
59277	6	1190134	1190148	3	5	15	GCA	21	4
59278	6	1190556	1190579	4	6	24	GACA	32	5
59289	6	1209284	1209311	4	7	28	AAAC	20	10
59298	6	1223621	1223640	2	10	20	CA	12	4
59316	6	1279986	1280005	2	10	20	TG	16	7
59325	6	1295986	1296019	2	17	34	GT	11	13
59340	6	1336263	1336278	2	8	16	AC	24	6
59346	6	1351075	1351090	2	8	16	AC	9	5
59347	6	1351093	1351108	2	8	16	AC	10	5
59349	6	1357016	1357035	2	10	20	CA	20	5
59353	6	1361518	1361543	2	13	26	AC	20	4
59365	6	1376179	1376202	2	12	24	AC	11	7
59377	6	1386417	1386438	2	11	22	GT	12	9
59386	6	1406775	1406802	2	14	28	AC	14	8
59392	6	1414899	1414916	2	9	18	TG	25	5
59420	6	1456982	1457001	4	5	20	ATGG	14	5
59426	6	1472297	1472308	2	6	12	GT	24	4
59431	6	1478987	1479004	2	9	18	CA	22	5
59432	6	1481016	1481033	3	6	18	ACT	25	5
59433	6	1481433	1481454	2	11	22	TG	22	5
59438	6	1491824	1491837	2	7	14	AC	11	3
59440	6	1494462	1494485	2	12	24	TG	14	4
59443	6	1500824	1500841	2	9	18	CT	25	5
59444	6	1505898	1505919	2	11	22	CA	17	6
59447	6	1507109	1507122	2	7	14	TG	24	6
59457	6	1515432	1515469	2	19	38	AC	11	8
59458	6	1515639	1515668	2	15	30	AC	25	7
59459	6	1517254	1517275	2	11	22	AC	19	5
59465	6	1528341	1528370	2	15	30	AC	9	10
59470	6	1541904	1541915	2	6	12	AC	7	4
59480	6	1560857	1560880	2	12	24	CA	16	13
59487	6	1566858	1566875	3	6	18	TGC	35	7
59495	6	1579269	1579288	4	5	20	TTTG	26	4
59500	6	1596734	1596753	2	10	20	TC	21	8
59505	6	1598432	1598451	2	10	20	CA	19	6
59506	6	1599110	1599139	2	15	30	CA	24	10
59508	6	1605898	1605911	2	7	14	TG	28	4
59512	6	1607446	1607459	2	7	14	AC	18	9

59513	6	1607462	1607473	2	6	12	AC	6	4
59514	6	1608770	1608781	2	6	12	TC	19	8
59515	6	1608782	1608793	2	6	12	AC	18	8
59517	6	1609396	1609407	2	6	12	TG	22	3
59518	6	1609410	1609421	2	6	12	TG	22	3
59520	6	1610798	1610815	2	9	18	AT	7	5
59529	6	1630054	1630065	2	6	12	TC	10	3
59530	6	1631550	1631575	2	13	26	AC	18	11
59531	6	1633940	1633953	2	7	14	CT	20	6
59532	6	1634159	1634206	2	24	48	TG	12	11
59537	6	1639043	1639070	2	14	28	TG	15	12
59538	6	1639192	1639227	2	18	36	AC	10	9
59539	6	1640729	1640750	2	11	22	GT	10	10
59540	6	1640879	1640914	2	18	36	TG	6	10
59543	6	1647100	1647111	2	6	12	CA	23	5
59544	6	1647114	1647125	2	6	12	CA	18	3
59545	6	1647327	1647344	2	9	18	AC	22	4
59548	6	1650293	1650332	4	10	40	GAAA	11	15
59549	6	1650397	1650418	2	11	22	GT	16	13
59550	6	1650538	1650563	2	13	26	AC	8	9
59553	6	1652285	1652300	2	8	16	TC	16	4
59554	6	1652461	1652494	2	17	34	AC	15	7
59557	6	1664906	1664937	2	16	32	CA	6	9
59559	6	1681861	1681878	2	9	18	AC	9	5
59562	6	1687292	1687318	3	9	27	CCT	7	4
59567	6	1701251	1701266	2	8	16	CT	20	7
59569	6	1703690	1703703	2	7	14	TG	10	5
59571	6	1712947	1712985	3	13	39	TCC	12	5
59573	6	1716453	1716464	2	6	12	AT	28	6
59579	6	1722621	1722634	2	7	14	CA	30	3
59580	6	1728637	1728652	4	4	16	ATTT	21	9
59583	6	1735562	1735575	2	7	14	TA	24	4
59589	6	1739665	1739676	2	6	12	TG	34	4
59591	6	1747983	1748010	2	14	28	AC	18	16
59601	6	1772215	1772230	2	8	16	AC	16	4
59604	6	1776958	1776975	2	9	18	AC	21	4
59627	6	1802957	1802972	2	8	16	TG	28	3
59630	6	1816009	1816072	2	32	64	GA	9	12
59633	6	1817088	1817107	2	10	20	GA	9	3
59641	6	1830221	1830250	3	10	30	ATC	30	5
59651	6	1868297	1868323	3	9	27	AGC	20	5
59654	6	1873206	1873223	2	9	18	GA	15	4
59656	6	1873883	1873902	2	10	20	GA	12	5
59658	6	1873929	1873942	2	7	14	GA	14	3
59659	6	1874070	1874083	2	7	14	AC	13	9
59660	6	1878782	1878819	2	19	38	CA	11	8
59665	6	1887108	1887123	2	8	16	TG	21	4
59674	6	1917755	1917794	2	20	40	GT	7	9
59683	6	1962526	1962543	2	9	18	TG	23	10
59687	6	1966921	1966936	4	4	16	AAAG	27	5
59689	6	1970755	1970778	3	8	24	TGT	23	5

59691	6	1972242	1972253	2	6	12	AT	28	4
59694	6	1976356	1976375	4	5	20	ATTT	18	7
59704	6	1995030	1995049	4	5	20	TTCT	13	3
59707	6	1996217	1996272	4	14	56	GAAA	10	8
59709	6	2001389	2001418	3	10	30	ATG	22	3
59714	6	2006887	2006900	2	7	14	AG	7	6
59723	6	2017065	2017106	2	21	42	GT	8	7
59725	6	2017173	2017194	2	11	22	GA	11	7
59727	6	2017916	2017933	3	6	18	AAG	25	5
59730	6	2021320	2021346	3	9	27	TCT	21	5
59739	6	2052302	2052313	2	6	12	TG	22	4
59741	6	2062281	2062296	2	8	16	GA	26	3
59748	6	2084998	2085017	2	10	20	TG	19	5
59750	6	2087975	2088018	2	22	44	AC	9	11
59757	6	2098572	2098589	2	9	18	GT	23	5
59759	6	2102794	2102811	3	6	18	TTC	12	10
59768	6	2141052	2141067	4	4	16	CCTG	23	3
59769	6	2141240	2141253	2	7	14	TG	30	3
59771	6	2146059	2146082	2	12	24	TG	7	8
59776	6	2155801	2155816	2	8	16	TG	23	13
59779	6	2158782	2158797	2	8	16	CA	31	3
59782	6	2163902	2163921	2	10	20	TG	31	7
59785	6	2178238	2178253	2	8	16	TG	26	4
59787	6	2212157	2212168	2	6	12	GT	28	3
59795	6	2236377	2236418	2	21	42	AC	6	6
59800	6	2247818	2247835	3	6	18	AGC	26	5
59803	6	2253154	2253167	2	7	14	AT	14	4
59815	6	2264804	2264823	2	10	20	AC	20	5
59816	6	2266113	2266124	2	6	12	CA	15	6
59817	6	2266362	2266377	2	8	16	GT	27	3
59820	6	2268838	2268877	4	10	40	TGTA	16	9
59832	6	2286212	2286227	2	8	16	GA	7	3
59836	6	2292696	2292723	4	7	28	TCCA	22	5
59841	6	2318232	2318249	2	9	18	TG	10	6
59842	6	2318252	2318263	2	6	12	TG	12	7
59844	6	2327209	2327241	3	11	33	ATT	8	5
59851	6	2342766	2342777	2	6	12	CA	31	3
59852	6	2344153	2344180	2	14	28	AC	6	9
59853	6	2345089	2345106	2	9	18	CA	18	11
59855	6	2345631	2345668	2	19	38	GT	15	11
59857	6	2347216	2347231	2	8	16	CA	20	9
59858	6	2347441	2347468	4	7	28	TTCT	12	9
59862	6	2348839	2348854	2	8	16	GT	19	3
59881	6	2387093	2387108	4	4	16	TGTC	27	4
59886	6	2400096	2400139	2	22	44	TG	17	10
59888	6	2401021	2401041	3	7	21	CCT	18	4
59890	6	2402137	2402180	4	11	44	ATCT	17	15
59892	6	2403161	2403178	3	6	18	GCA	31	3
59902	6	2422053	2422067	3	5	15	CCT	30	3
59904	6	2425298	2425309	2	6	12	CA	14	6
59910	6	2437783	2437796	2	7	14	TA	17	3

59924	6	2495665	2495692	2	14	28	TG	12	9
59925	6	2496448	2496461	2	7	14	TC	19	3
59926	6	2499218	2499241	4	6	24	TTTA	20	3
59929	6	2505896	2505931	2	18	36	GT	22	12
59932	6	2508647	2508662	4	4	16	GTCT	28	3
59935	6	2524485	2524506	2	11	22	TC	24	7
59944	6	2545977	2545988	2	6	12	AC	18	3
59946	6	2556653	2556666	2	7	14	CA	27	3
59947	6	2561911	2561936	2	13	26	AG	20	8
59948	6	2563606	2563623	2	9	18	TC	25	6
59950	6	2565940	2565959	4	5	20	ATAA	20	4
59953	6	2585730	2585744	3	5	15	GCT	29	3
59962	6	2603536	2603550	3	5	15	GAA	14	6
59965	6	2606017	2606028	2	6	12	GT	19	3
59969	6	2612230	2612247	2	9	18	GA	15	3
59970	6	2612470	2612495	2	13	26	AG	8	6
59973	6	2612944	2612955	2	6	12	AT	13	4
59974	6	2613148	2613171	2	12	24	CA	15	10
59986	6	2626475	2626490	4	4	16	TTAT	23	4
59995	6	2638913	2638928	2	8	16	TG	14	8
59996	6	2639181	2639196	2	8	16	TG	19	6
59998	6	2645624	2645639	4	4	16	ATGG	10	6
60002	6	2657091	2657112	2	11	22	CA	13	5
60003	6	2657228	2657255	2	14	28	AC	18	12
60006	6	2660032	2660073	3	14	42	GAA	21	10
60007	6	2660387	2660401	3	5	15	CGG	30	3
60010	6	2662916	2662961	2	23	46	AC	10	9
60023	6	2671022	2671057	2	18	36	CA	8	10
60025	6	2671533	2671544	2	6	12	TG	10	4
60026	6	2671546	2671561	2	8	16	GT	11	3
60035	6	2682078	2682095	2	9	18	AC	27	6
60043	6	2700280	2700291	2	6	12	TA	27	3
60046	6	2702480	2702499	2	10	20	AC	15	4
60053	6	2726370	2726395	2	13	26	CA	14	11
60068	6	2755419	2755444	2	13	26	GT	10	7
60083	6	2787451	2787466	2	8	16	GT	10	12
60086	6	2794762	2794773	2	6	12	AC	31	7
60089	6	2805364	2805409	2	23	46	TG	8	9
60094	6	2818065	2818088	2	12	24	AC	22	12
60096	6	2823905	2823922	2	9	18	GA	15	5
60098	6	2825184	2825205	2	11	22	CA	15	8
60100	6	2825367	2825388	2	11	22	TG	18	8
60102	6	2829194	2829214	3	7	21	CAA	20	8
60121	6	2845201	2845220	2	10	20	CA	20	10
60122	6	2845222	2845237	2	8	16	AC	13	9
60124	6	2845665	2845688	2	12	24	TG	23	9
60126	6	2846679	2846696	3	6	18	CTG	24	3
60128	6	2846777	2846794	3	6	18	ACA	20	8
60131	6	2851241	2851270	2	15	30	TG	17	11
60136	6	2855292	2855315	2	12	24	CT	18	13
60138	6	2856832	2856853	2	11	22	CA	26	11

60140	6	2858387	2858410	4	6	24	ATCC	21	3
60141	6	2858548	2858563	2	8	16	GT	23	6
60142	6	2862158	2862179	2	11	22	AC	18	9
60143	6	2862200	2862229	3	10	30	CCT	19	5
60144	6	2868717	2868744	2	14	28	TC	13	4
60147	6	2870824	2870879	4	14	56	ATCT	16	12
60149	6	2872586	2872600	3	5	15	CCT	14	5
60153	6	2884175	2884195	3	7	21	GCA	26	3
60156	6	2889293	2889326	2	17	34	TG	17	13
60157	6	2889525	2889540	2	8	16	GT	18	10
60163	6	2917284	2917307	2	12	24	AG	19	7
60164	6	2917647	2917666	2	10	20	GT	17	8
60167	6	2938879	2938894	2	8	16	AC	20	12
60172	6	2964055	2964070	2	8	16	TG	25	15
60183	6	2991437	2991448	2	6	12	CA	15	5
60184	6	2991451	2991462	2	6	12	CA	22	6
60188	6	2996749	2996762	2	7	14	CA	11	10
60219	6	3073411	3073425	3	5	15	TTG	7	5
60235	6	3103087	3103101	3	5	15	GCA	9	4
60238	6	3104024	3104038	3	5	15	TAT	18	4
60244	6	3119942	3119971	2	15	30	TG	12	9
60247	6	3124295	3124310	4	4	16	CAGG	29	3
60248	6	3131267	3131278	2	6	12	TG	16	3
60255	6	3138239	3138258	2	10	20	TG	16	5
60263	6	3144437	3144458	2	11	22	TG	21	3
60278	6	3167407	3167418	2	6	12	CA	20	3
60281	6	3168732	3168749	2	9	18	AG	8	6
60283	6	3174971	3174982	2	6	12	AC	14	3
60285	6	3179948	3179977	2	15	30	GT	13	8
60301	6	3220803	3220826	2	12	24	CA	15	11
60302	6	3223484	3223498	3	5	15	CTA	21	3
60307	6	3241985	3242018	2	17	34	TG	13	10
60308	6	3242570	3242590	3	7	21	GGT	11	5
60310	6	3243476	3243493	2	9	18	AC	22	10
60312	6	3248039	3248056	2	9	18	AC	20	9
60325	6	3273831	3273856	2	13	26	AC	14	10
60326	6	3278392	3278407	2	8	16	CA	21	5
60328	6	3278714	3278725	2	6	12	GT	24	8
60332	6	3314540	3314561	2	11	22	AC	14	5
60338	6	3322214	3322233	2	10	20	GT	18	4
60339	6	3323658	3323679	2	11	22	AG	13	6
60340	6	3323745	3323760	4	4	16	TTTA	18	6
60341	6	3324057	3324076	2	10	20	TA	10	5
60344	6	3326109	3326134	2	13	26	GT	8	8
60346	6	3327702	3327713	2	6	12	GT	20	7
60347	6	3330141	3330166	2	13	26	GT	22	11
60352	6	3340345	3340356	2	6	12	AT	12	4
60357	6	3343010	3343025	2	8	16	GA	9	5
60365	6	3354843	3354860	2	9	18	GT	17	7
60367	6	3355583	3355626	2	22	44	GT	6	9
60371	6	3364144	3364161	2	9	18	TC	7	4

60380	6	3425532	3425546	3	5	15	TTA	22	8
60386	6	3433088	3433105	2	9	18	TG	22	10
60389	6	3434727	3434742	4	4	16	AAAT	23	9
60390	6	3435878	3435891	2	7	14	AT	14	3
60391	6	3437296	3437317	2	11	22	CT	10	6
60392	6	3437534	3437545	2	6	12	CA	12	5
60393	6	3437548	3437559	2	6	12	CA	13	6
60394	6	3438391	3438404	2	7	14	GT	17	5
60396	6	3442601	3442622	2	11	22	AC	11	4
60400	6	3446415	3446428	2	7	14	AT	18	3
60402	6	3448139	3448168	3	10	30	CTC	15	5
60405	6	3450852	3450867	2	8	16	TG	17	8
60409	6	3452418	3452431	2	7	14	TG	13	3
60413	6	3455642	3455665	2	12	24	CA	20	16
60416	6	3459655	3459684	2	15	30	TG	10	8
60417	6	3460320	3460337	2	9	18	TC	17	5
60419	6	3463967	3463990	2	12	24	CT	10	5
60423	6	3481938	3481949	2	6	12	CA	12	11
60424	6	3481952	3481969	2	9	18	CA	14	11
60425	6	3482921	3482940	2	10	20	CA	24	4
60442	6	3510619	3510638	2	10	20	TG	12	3
60446	6	3513409	3513438	2	15	30	CA	15	7
60451	6	3518929	3518944	2	8	16	CA	28	8
60453	6	3525444	3525455	2	6	12	GT	14	3
60454	6	3525780	3525791	2	6	12	AC	27	5
60456	6	3532546	3532561	2	8	16	AC	23	6
60457	6	3532596	3532611	2	8	16	AC	15	6
60458	6	3534594	3534611	2	9	18	CA	15	6
60466	6	3563061	3563092	2	16	32	CA	6	5
60469	6	3564846	3564877	2	16	32	CA	12	7
60478	6	3597167	3597186	2	10	20	CA	18	11
60479	6	3597431	3597450	2	10	20	GT	20	6
60480	6	3598094	3598115	2	11	22	AC	9	7
60481	6	3607564	3607575	2	6	12	CA	23	3
60487	6	3620931	3620944	2	7	14	CA	24	5
60490	6	3627604	3627627	2	12	24	GT	6	6
60500	6	3635183	3635200	2	9	18	AC	16	4
60503	6	3639366	3639389	2	12	24	AC	19	10
60508	6	3661393	3661418	2	13	26	AG	22	5
60515	6	3694636	3694661	2	13	26	AC	11	6
60516	6	3695269	3695298	2	15	30	GT	22	14
60525	6	3724638	3724679	2	21	42	TG	7	6
60532	6	3735083	3735108	2	13	26	GT	23	9
60533	6	3739676	3739701	2	13	26	GT	18	6
60538	6	3744608	3744655	2	24	48	GT	13	10
60539	6	3746966	3746989	4	6	24	TATC	14	5
60547	6	3756854	3756867	2	7	14	AC	11	3
60548	6	3757879	3757898	2	10	20	AC	17	7
60554	6	3761769	3761780	2	6	12	AT	13	3
60559	6	3764031	3764044	2	7	14	TG	7	7
60564	6	3787103	3787114	2	6	12	AC	25	4

60571	6	3800073	3800086	2	7	14	TG	26	3
60572	6	3803472	3803486	3	5	15	AAT	20	4
60573	6	3806428	3806448	3	7	21	GGA	21	12
60575	6	3821187	3821202	2	8	16	GA	28	3
60578	6	3828800	3828813	2	7	14	TG	23	3
60580	6	3838314	3838325	2	6	12	CA	28	5
60590	6	3856166	3856195	2	15	30	TG	17	9
60595	6	3864088	3864105	3	6	18	TGT	29	3
60597	6	3878467	3878478	2	6	12	TG	29	3
60599	6	3885573	3885604	2	16	32	TG	19	11
60600	6	3891880	3891897	3	6	18	CAT	22	3
60606	6	3904161	3904180	2	10	20	TG	19	13
60608	6	3911730	3911747	3	6	18	AAG	19	6
60611	6	3916891	3916902	2	6	12	AC	17	3
60612	6	3921429	3921444	2	8	16	AC	11	4
60614	6	3924395	3924414	4	5	20	ACAG	16	5
60615	6	3926504	3926519	2	8	16	TG	19	17
60616	6	3926522	3926533	2	6	12	TG	23	14
60623	6	3937545	3937562	2	9	18	GT	18	9
60626	6	3940496	3940509	2	7	14	CT	25	3
60629	6	3946388	3946399	2	6	12	AC	6	5
60634	6	3962490	3962523	2	17	34	CA	8	11
60657	6	4051680	4051700	3	7	21	TAG	21	4
60659	6	4053335	4053356	2	11	22	GT	29	5
60670	6	4085755	4085770	2	8	16	GT	21	3
60675	6	4094387	4094406	2	10	20	TG	6	6
60678	6	4102375	4102389	3	5	15	AAT	11	6
60680	6	4108221	4108254	2	17	34	CA	22	14
60685	6	4125926	4125945	2	10	20	AC	11	11
60686	6	4126206	4126229	2	12	24	CA	20	10
60692	6	4151418	4151431	2	7	14	CA	28	6
60697	6	4163381	4163404	4	6	24	AGAT	21	3
60701	6	4170434	4170448	3	5	15	ACC	24	5
60705	6	4177097	4177118	2	11	22	AC	19	7
60710	6	4190559	4190570	2	6	12	AT	15	4
60716	6	4206048	4206063	2	8	16	GT	18	5
60718	6	4221920	4221937	3	6	18	CAG	24	3
60719	6	4224652	4224677	2	13	26	CA	11	13
60724	6	4230909	4230932	2	12	24	CA	11	6
60727	6	4243133	4243176	2	22	44	TG	6	8
60731	6	4253556	4253587	4	8	32	ATGG	22	4
60734	6	4258131	4258162	4	8	32	GACA	23	5
60737	6	4268438	4268459	2	11	22	AG	24	3
60738	6	4271791	4271816	2	13	26	GT	24	18
60741	6	4283058	4283069	2	6	12	GA	30	4
60742	6	4291454	4291505	2	26	52	GT	10	11
60750	6	4314993	4315018	2	13	26	CA	14	9
60751	6	4317707	4317718	2	6	12	TG	28	3
60755	6	4323736	4323777	2	21	42	AC	11	15
60756	6	4325623	4325636	2	7	14	AC	24	4
60757	6	4329738	4329753	2	8	16	AC	19	4

60786	6	4387007	4387022	2	8	16	AG	29	4
60789	6	4390110	4390127	2	9	18	CA	18	6
60790	6	4390361	4390378	2	9	18	AC	17	6
60795	6	4400250	4400273	2	12	24	TG	19	5
60797	6	4402017	4402036	4	5	20	ATTT	27	5
60798	6	4403739	4403758	2	10	20	TG	13	7
60799	6	4403831	4403850	4	5	20	TAAA	21	8
60803	6	4405181	4405202	2	11	22	GA	11	6
60804	6	4407382	4407407	2	13	26	TG	14	9
60805	6	4407735	4407760	2	13	26	CA	19	11
60809	6	4418701	4418726	2	13	26	AG	13	5
60810	6	4419504	4419519	4	4	16	AAAT	18	3
60811	6	4420476	4420487	2	6	12	CA	16	3
60812	6	4421111	4421156	2	23	46	CA	7	9
60816	6	4427908	4427928	3	7	21	CCT	24	8
60820	6	4435593	4435608	2	8	16	TC	25	3
60823	6	4439167	4439178	2	6	12	AC	28	3
60824	6	4439911	4439931	3	7	21	TGC	24	7
60830	6	4455421	4455434	2	7	14	TG	27	4
60836	6	4465167	4465188	2	11	22	CA	13	3
60840	6	4475972	4475986	3	5	15	TGG	26	6
60845	6	4476665	4476682	2	9	18	CA	12	12
60848	6	4477608	4477643	2	18	36	CA	6	8
60849	6	4477708	4477719	2	6	12	AC	17	3
60850	6	4481382	4481397	2	8	16	TG	30	5
60852	6	4483571	4483584	2	7	14	AC	28	4
60856	6	4487257	4487286	2	15	30	CA	19	8
60857	6	4493736	4493775	4	10	40	TCAA	12	11
60858	6	4494210	4494260	3	17	51	AGA	12	12
60859	6	4497955	4497970	2	8	16	TG	8	5
60864	6	4509765	4509784	4	5	20	AAAG	15	4
60865	6	4516843	4516866	3	8	24	ATA	20	3
60879	6	4526509	4526528	2	10	20	CA	19	8
60884	6	4539071	4539086	2	8	16	CA	15	3
60893	6	4544548	4544563	2	8	16	TG	21	5
60894	6	4544731	4544770	2	20	40	CA	15	6
60902	6	4558269	4558280	2	6	12	TG	25	3
60904	6	4558734	4558753	2	10	20	CA	21	16
60908	6	4562129	4562140	2	6	12	CA	20	4
60910	6	4562653	4562670	2	9	18	AC	11	5
60912	6	4562696	4562709	2	7	14	CA	12	4
60913	6	4567696	4567715	2	10	20	GT	7	6
60920	6	4571488	4571499	2	6	12	GT	21	3
60924	6	4576415	4576426	2	6	12	CA	26	4
60925	6	4578565	4578590	2	13	26	AC	12	8
60927	6	4580382	4580397	2	8	16	CA	19	5
60930	6	4584800	4584827	2	14	28	TG	12	7
60931	6	4584956	4585013	2	29	58	CA	8	10
60936	6	4594971	4594986	2	8	16	AT	15	10
60937	6	4599389	4599428	2	20	40	CA	15	14
60940	6	4608174	4608187	2	7	14	GT	27	3

60945	6	4622554	4622569	2	8	16	AC	29	6
60951	6	4644375	4644386	2	6	12	TC	19	6
60953	6	4644587	4644610	2	12	24	TC	15	7
60958	6	4659223	4659242	2	10	20	CA	19	10
60965	6	4676371	4676382	2	6	12	GT	26	3
60978	6	4700059	4700079	3	7	21	CCT	23	4
60985	6	4703281	4703295	3	5	15	AAC	15	4
60996	6	4714431	4714444	2	7	14	AC	21	4
60997	6	4715187	4715210	2	12	24	TG	20	9
60999	6	4717032	4717046	3	5	15	TGC	25	6
61001	6	4724931	4724958	2	14	28	TG	14	12
61003	6	4725415	4725435	3	7	21	TCT	17	5
61008	6	4731103	4731128	2	13	26	AG	21	6
61011	6	4732496	4732509	2	7	14	AC	24	4
61013	6	4736207	4736232	2	13	26	AG	8	10
61015	6	4737483	4737496	2	7	14	TC	22	7
61016	6	4737637	4737662	2	13	26	AC	10	10
61017	6	4742547	4742574	2	14	28	AC	13	5
61019	6	4746193	4746204	2	6	12	AC	22	6
61020	6	4746206	4746219	2	7	14	CA	28	8
61021	6	4746370	4746383	2	7	14	GT	24	3
61023	6	4746852	4746871	2	10	20	AC	19	8
61026	6	4747600	4747623	2	12	24	GT	7	8
61028	6	4749919	4749932	2	7	14	TG	23	4
61032	6	4770096	4770113	2	9	18	AC	27	4
61037	6	4774016	4774041	2	13	26	TG	19	9
61038	6	4774741	4774752	2	6	12	TA	9	3
61039	6	4777297	4777334	2	19	38	TG	11	9
61040	6	4778028	4778045	2	9	18	GT	15	3
61048	6	4785777	4785802	2	13	26	AC	20	13
61050	6	4785963	4785980	3	6	18	GCT	10	4
61052	6	4786314	4786329	2	8	16	AC	20	3
61055	6	4786890	4786903	2	7	14	GA	16	7
61057	6	4787304	4787327	4	6	24	TATT	18	3
61059	6	4787632	4787645	2	7	14	AC	8	3
61066	6	4807083	4807104	2	11	22	CA	27	5
61068	6	4808189	4808200	2	6	12	AT	16	7
61070	6	4814130	4814147	3	6	18	TTA	24	3
61078	6	4869094	4869107	2	7	14	TG	24	3
61088	6	4890801	4890812	2	6	12	TG	23	8
61089	6	4890814	4890825	2	6	12	GT	23	8
61090	6	4891028	4891039	2	6	12	GT	7	3
61091	6	4892950	4892967	3	6	18	CTC	23	3
61093	6	4894725	4894762	2	19	38	TG	6	10
61095	6	4897157	4897174	3	6	18	TTG	24	3
61097	6	4897809	4897826	2	9	18	CA	20	10
61100	6	4899268	4899287	4	5	20	TGAT	30	3
61103	6	4900940	4900961	2	11	22	CA	26	22
61104	6	4901444	4901455	2	6	12	TA	26	4
61106	6	4903813	4903838	2	13	26	CA	9	7
61108	6	4908077	4908094	2	9	18	TG	17	10

61111	6	4911929	4911944	4	4	16	TCTT	16	6
61114	6	4918041	4918056	2	8	16	AC	19	12
61116	6	4918394	4918419	2	13	26	TG	21	15
61120	6	4919079	4919090	2	6	12	AT	6	3
61126	6	4920618	4920651	2	17	34	CT	10	8
61127	6	4920685	4920712	2	14	28	TG	17	10
61129	6	4921365	4921382	3	6	18	GCT	26	5
61130	6	4921573	4921608	3	12	36	TCC	23	6
61137	6	4928033	4928046	2	7	14	CT	23	5
61139	6	4937769	4937784	2	8	16	TG	20	8
61141	6	4938453	4938474	2	11	22	TG	8	11
61142	6	4940928	4940941	2	7	14	TC	27	3
61145	6	4945006	4945029	2	12	24	GT	20	7
61146	6	4945161	4945198	2	19	38	CA	14	8
61148	6	4951841	4951868	2	14	28	TG	7	9
61149	6	4952080	4952101	2	11	22	AC	18	7
61150	6	4952271	4952282	2	6	12	GT	20	7
61151	6	4952418	4952431	2	7	14	AC	28	4
61157	6	4971009	4971028	2	10	20	TG	13	6
61161	6	4972182	4972195	2	7	14	GT	25	3
61167	6	4984706	4984725	2	10	20	TG	25	5
61168	6	4984875	4984892	2	9	18	GT	19	8
61169	6	4985112	4985127	2	8	16	GT	20	11
61172	6	4987690	4987707	2	9	18	TG	9	9
61181	6	5005155	5005170	2	8	16	CA	12	8
61182	6	5005368	5005395	2	14	28	CA	6	4
61195	6	5024262	5024289	2	14	28	AC	13	10
61201	6	5031717	5031732	4	4	16	TGAA	32	5
61203	6	5034502	5034515	2	7	14	TG	29	4
61206	6	5039170	5039193	3	8	24	TTG	23	9
61207	6	5044066	5044099	2	17	34	AC	8	7
61211	6	5049414	5049434	3	7	21	TCC	22	3
61212	6	5053005	5053020	2	8	16	GT	7	6
61213	6	5053022	5053039	2	9	18	TG	13	7
61214	6	5053189	5053200	2	6	12	AT	16	4
61221	6	5067540	5067551	2	6	12	AC	14	4
61224	6	5069247	5069268	2	11	22	AC	12	3
61226	6	5070985	5071028	2	22	44	TG	18	13
61227	6	5071125	5071144	2	10	20	AC	22	6
61230	6	5072503	5072526	3	8	24	AAT	18	3
61236	6	5090975	5090992	3	6	18	AAT	14	9
61237	6	5091096	5091119	2	12	24	AC	15	11
61238	6	5091237	5091254	3	6	18	GGA	10	7
61254	6	5121802	5121829	2	14	28	TG	14	8
61260	6	5129113	5129148	2	18	36	TG	7	9
61264	6	5131563	5131610	2	24	48	GT	12	16
61265	6	5131732	5131749	2	9	18	TC	13	4
61267	6	5143654	5143669	2	8	16	AC	23	3
61270	6	5145709	5145735	3	9	27	CTC	19	3
61271	6	5145890	5145901	2	6	12	TG	15	5
61272	6	5153424	5153441	3	6	18	GCA	22	7

61277	6	5163406	5163423	3	6	18	GGA	31	3
61279	6	5166670	5166689	2	10	20	AC	15	12
61281	6	5167594	5167619	2	13	26	TG	25	13
61284	6	5174921	5174938	2	9	18	GT	9	6
61285	6	5176710	5176737	2	14	28	CA	23	7
61286	6	5177331	5177350	2	10	20	CA	19	13
61288	6	5182843	5182854	2	6	12	AG	11	11
61290	6	5186365	5186388	3	8	24	CTC	22	8
61292	6	5189779	5189810	2	16	32	CA	16	7
61306	6	5198134	5198149	2	8	16	CT	15	6
61309	6	5204876	5204901	2	13	26	TG	17	12
61310	6	5206301	5206326	2	13	26	GT	12	8
61311	6	5209297	5209316	2	10	20	GT	19	4
61313	6	5214163	5214190	2	14	28	AC	10	6
61318	6	5218375	5218400	2	13	26	TC	11	11
61320	6	5218523	5218538	2	8	16	CT	20	6
61321	6	5225192	5225223	4	8	32	TCTG	25	12
61326	6	5238846	5238867	2	11	22	AC	12	9
61330	6	5252385	5252400	2	8	16	CA	13	7
61334	6	5256172	5256195	2	12	24	TG	12	11
61339	6	5279135	5279150	2	8	16	TG	24	5
61341	6	5282530	5282553	2	12	24	GT	12	15
61343	6	5292254	5292268	3	5	15	GAG	21	5
61345	6	5308671	5308686	2	8	16	GT	17	4
61346	6	5315420	5315441	2	11	22	TG	10	12
61349	6	5328672	5328683	2	6	12	GA	23	4
61353	6	5333598	5333615	3	6	18	AAC	22	7
61354	6	5335982	5336009	2	14	28	GT	21	11
61357	6	5339162	5339179	3	6	18	CTG	32	6
61358	6	5353033	5353046	2	7	14	AT	24	11
61360	6	5357110	5357147	2	19	38	TG	7	9
61361	6	5360687	5360700	2	7	14	AC	22	6
61363	6	5361242	5361255	2	7	14	AC	20	6
61365	6	5363539	5363562	2	12	24	TG	19	11
61366	6	5363599	5363616	2	9	18	GT	22	7
61368	6	5367766	5367781	2	8	16	CA	23	5
61373	6	5372039	5372058	2	10	20	AT	6	3
61374	6	5372930	5372949	2	10	20	AC	23	5
61375	6	5373338	5373355	2	9	18	AC	23	5
61377	6	5379013	5379032	2	10	20	CA	16	6
61387	6	5391633	5391652	2	10	20	AC	16	7
61389	6	5394676	5394690	3	5	15	GCA	26	7
61392	6	5397096	5397117	2	11	22	CA	22	11
61393	6	5397480	5397493	2	7	14	TG	15	3
61394	6	5397760	5397775	2	8	16	TG	14	5
61396	6	5398450	5398463	2	7	14	GT	13	5
61398	6	5401197	5401208	2	6	12	AG	28	3
61401	6	5404281	5404310	2	15	30	GT	16	8
61403	6	5406937	5406952	2	8	16	TC	6	5
61408	6	5414808	5414837	2	15	30	AC	18	9
61423	6	5428110	5428139	2	15	30	GT	14	7

61424	6	5428418	5428435	2	9	18	CA	18	7
61425	6	5434673	5434690	2	9	18	CA	17	4
61433	6	5449463	5449474	2	6	12	GT	16	3
61434	6	5449494	5449523	2	15	30	GT	11	4
61437	6	5462281	5462298	2	9	18	TG	17	5
61438	6	5463124	5463139	2	8	16	CA	20	4
61442	6	5486674	5486701	4	7	28	TTCT	20	4
61448	6	5495481	5495501	3	7	21	GAG	24	5
61451	6	5502901	5502922	2	11	22	TG	24	8
61477	6	5545548	5545595	2	24	48	TG	9	9
61478	6	5547310	5547333	2	12	24	TG	22	11
61479	6	5547878	5547893	2	8	16	AC	19	5
61480	6	5548444	5548464	3	7	21	CTC	28	5
61486	6	5563692	5563707	4	4	16	TTTA	12	6
61489	6	5566703	5566718	4	4	16	AATC	22	5
61505	6	5591977	5591988	2	6	12	AG	24	4
61507	6	5593148	5593163	4	4	16	AGCT	17	5
61510	6	5605841	5605860	2	10	20	AC	16	13
61512	6	5617344	5617361	2	9	18	TC	25	5
61514	6	5621042	5621055	2	7	14	AC	27	3
61522	6	5645048	5645061	2	7	14	GT	25	6
61525	6	5653488	5653503	4	4	16	TTTG	24	4
61532	6	5674886	5674903	3	6	18	GAA	25	3
61542	6	5694225	5694236	2	6	12	TG	21	9
61543	6	5694248	5694263	4	4	16	TTTA	19	3
61544	6	5694783	5694797	3	5	15	GAG	25	8
61547	6	5700052	5700063	2	6	12	GT	20	5
61550	6	5702811	5702828	2	9	18	TC	25	3
61552	6	5704262	5704282	3	7	21	AGT	33	7
61553	6	5705005	5705034	3	10	30	AAT	7	3
61555	6	5706061	5706074	2	7	14	GA	22	6
61570	6	5716864	5716879	2	8	16	CT	13	8
61571	6	5717039	5717054	2	8	16	CA	19	4
61574	6	5734221	5734241	3	7	21	CAA	28	3
61575	6	5741379	5741402	2	12	24	AC	19	7
61576	6	5741870	5741887	2	9	18	AT	8	4
61579	6	5749984	5749997	2	7	14	AC	23	7
61588	6	5765364	5765431	4	17	68	TAGA	19	14
61589	6	5766609	5766628	4	5	20	GACA	24	4
61595	6	5789922	5789949	2	14	28	TG	21	6
61596	6	5790478	5790501	2	12	24	TG	8	9
61598	6	5793005	5793022	3	6	18	GAG	12	4
61607	6	5820718	5820729	2	6	12	GA	17	4
61612	6	5830382	5830411	2	15	30	TC	23	5
61620	6	5861123	5861148	2	13	26	AC	21	6
61624	6	5883050	5883067	2	9	18	CT	21	4
61625	6	5883226	5883261	2	18	36	CA	8	7
61627	6	5885434	5885451	2	9	18	AC	21	11
61628	6	5887939	5887954	2	8	16	AC	24	6
61638	6	5899675	5899714	2	20	40	AC	18	10
61646	6	5915827	5915838	2	6	12	TG	23	3

61657	6	5943310	5943329	2	10	20	GT	18	4
61659	6	5958462	5958475	2	7	14	AG	28	4
61678	6	6000124	6000157	2	17	34	GT	21	10
61680	6	6000728	6000739	2	6	12	AG	19	3
61681	6	6000742	6000759	2	9	18	AG	16	3
61682	6	6005676	6005721	2	23	46	GT	8	9
61684	6	6027765	6027780	2	8	16	AC	6	3
61697	6	6048554	6048585	2	16	32	CA	12	7
61699	6	6057181	6057192	2	6	12	GT	25	4
61700	6	6060292	6060311	2	10	20	CA	11	5
61701	6	6060461	6060496	2	18	36	TG	16	9
61703	6	6060876	6060903	2	14	28	TG	15	8
61704	6	6064972	6064983	2	6	12	TG	26	3
61706	6	6067121	6067150	2	15	30	AC	21	8
61713	6	6081207	6081220	2	7	14	CA	19	3
61714	6	6082940	6082959	2	10	20	CA	24	7
61715	6	6083890	6083937	2	24	48	AC	9	11
61719	6	6089402	6089427	2	13	26	AC	20	6
61721	6	6092590	6092611	2	11	22	CT	21	3
61722	6	6092638	6092657	2	10	20	CT	13	3
61724	6	6093268	6093289	2	11	22	AC	24	6
61725	6	6093566	6093579	2	7	14	AC	27	6
61726	6	6093624	6093643	2	10	20	GT	15	7
61728	6	6094957	6094972	2	8	16	AC	23	4
61729	6	6095128	6095147	2	10	20	CA	26	6
61732	6	6095680	6095697	2	9	18	TG	23	7
61733	6	6096328	6096357	2	15	30	TG	15	16
61734	6	6098042	6098053	2	6	12	CA	35	3
61736	6	6098289	6098300	2	6	12	AC	22	4
61738	6	6099985	6099998	2	7	14	AC	24	3
61739	6	6103062	6103087	2	13	26	CA	23	10
61745	6	6127326	6127349	3	8	24	AAC	20	6
61748	6	6131519	6131542	3	8	24	CTT	21	4
61749	6	6132006	6132032	3	9	27	TTC	23	7
61755	6	6156157	6156190	2	17	34	TG	8	14
61757	6	6158908	6158929	2	11	22	TG	11	9
61764	6	6162017	6162036	2	10	20	GA	12	4
61765	6	6163006	6163021	4	4	16	AAAT	25	6
61771	6	6171672	6171683	2	6	12	GT	22	3
61772	6	6172624	6172639	2	8	16	AG	28	6
61774	6	6176012	6176029	2	9	18	AC	28	4
61777	6	6180672	6180687	2	8	16	GT	22	3
61779	6	6180848	6180867	2	10	20	CA	30	3
61783	6	6188048	6188063	2	8	16	AC	20	5
61789	6	6194372	6194401	2	15	30	AC	15	8
61794	6	6201159	6201190	2	16	32	AC	9	5
61795	6	6204563	6204580	2	9	18	AC	15	14
61806	6	6217455	6217466	2	6	12	AC	29	3
61808	6	6219314	6219329	2	8	16	TG	12	7
61814	6	6237393	6237426	2	17	34	AC	15	11
61821	6	6245327	6245346	2	10	20	TG	15	10

61836	6	6279389	6279404	2	8	16	GT	7	7
61838	6	6284421	6284440	2	10	20	CA	16	12
61840	6	6295782	6295799	2	9	18	GT	17	11
61843	6	6304234	6304257	2	12	24	CA	20	6
61856	6	6319298	6319321	2	12	24	TG	16	7
61857	6	6321179	6321198	2	10	20	GA	12	8
61863	6	6332584	6332599	4	4	16	ATTT	11	4
61865	6	6344235	6344276	2	21	42	AC	6	10
61869	6	6350553	6350568	2	8	16	TG	27	5
61872	6	6355707	6355722	2	8	16	CA	21	3
61873	6	6355832	6355855	2	12	24	TG	11	14
61879	6	6361250	6361265	2	8	16	CT	13	3
61881	6	6361469	6361484	2	8	16	AC	14	3
61882	6	6362562	6362582	3	7	21	TGT	32	4
61886	6	6365555	6365570	2	8	16	CA	22	6
61910	6	6420197	6420212	2	8	16	TA	15	9
61911	6	6423894	6423920	3	9	27	GAT	21	5
61914	6	6435796	6435807	2	6	12	GT	15	10
61916	6	6445102	6445135	2	17	34	TG	11	12
61918	6	6452084	6452104	3	7	21	GAG	25	5
61919	6	6453419	6453438	4	5	20	TTGT	20	4
61921	6	6454791	6454812	2	11	22	AG	19	8
61922	6	6456404	6456415	2	6	12	AC	20	16
61923	6	6456572	6456586	3	5	15	CCT	18	3
61924	6	6457796	6457813	3	6	18	TTA	22	6
61929	6	6473523	6473538	2	8	16	AC	25	4
61936	6	6481233	6481248	2	8	16	TG	19	5
61937	6	6481830	6481843	2	7	14	TG	29	4
61941	6	6490198	6490239	2	21	42	AC	7	9
61942	6	6493101	6493124	2	12	24	TG	11	12
61943	6	6493266	6493279	2	7	14	TG	25	3
61944	6	6495403	6495417	3	5	15	GTT	28	4
61946	6	6496708	6496719	2	6	12	CA	6	6
61947	6	6497415	6497426	2	6	12	AG	22	4
61953	6	6508842	6508859	2	9	18	AG	17	4
61954	6	6509466	6509479	2	7	14	CT	25	7
61961	6	6521190	6521219	2	15	30	GT	13	10
61963	6	6522404	6522427	3	8	24	CAG	15	5
61964	6	6523674	6523707	2	17	34	TG	8	7
61965	6	6524106	6524123	2	9	18	AG	23	5
61969	6	6526980	6527011	2	16	32	CA	6	8
61973	6	6539536	6539575	2	20	40	TG	7	8
61974	6	6544165	6544176	2	6	12	AT	25	3
61976	6	6547394	6547415	2	11	22	CA	10	4
61977	6	6549427	6549456	2	15	30	GA	15	4
61978	6	6549645	6549658	2	7	14	GT	26	7
61979	6	6550270	6550281	2	6	12	GT	23	4
61987	6	6556041	6556056	2	8	16	AC	25	7
61989	6	6558330	6558359	3	10	30	TAT	16	5
61991	6	6559782	6559801	2	10	20	AG	18	4
61996	6	6565928	6565949	2	11	22	CA	23	7

61999	6	6570611	6570622	2	6	12	TG	25	5
62000	6	6570808	6570829	2	11	22	CA	15	9
62004	6	6576547	6576570	2	12	24	GT	22	10
62008	6	6578298	6578321	2	12	24	AC	22	7
62016	6	6585876	6585899	2	12	24	GT	16	14
62017	6	6586074	6586101	2	14	28	TG	11	7
62018	6	6586219	6586242	2	12	24	TG	14	13
62019	6	6586394	6586405	2	6	12	TC	17	3
62021	6	6591113	6591140	2	14	28	TG	14	12
62030	6	6597478	6597505	2	14	28	CA	7	6
62032	6	6603148	6603173	2	13	26	TG	19	14
62033	6	6603929	6603942	2	7	14	TG	21	5
62034	6	6604254	6604277	2	12	24	AC	18	11
62035	6	6606480	6606523	2	22	44	CT	7	7
62039	6	6618466	6618483	2	9	18	GT	20	7
62040	6	6618822	6618841	2	10	20	GT	23	8
62043	6	6633097	6633110	2	7	14	CA	25	11
62046	6	6639146	6639159	2	7	14	GT	21	4
62048	6	6640965	6641000	4	9	36	TGAG	25	6
62051	6	6646438	6646459	2	11	22	CA	18	13
62052	6	6648229	6648244	2	8	16	AC	17	11
62056	6	6655298	6655317	2	10	20	GT	13	3
62067	6	6671453	6671470	2	9	18	TG	20	3
62071	6	6673550	6673567	3	6	18	TCC	9	5
62075	6	6674040	6674055	2	8	16	CT	14	4
62078	6	6676099	6676113	3	5	15	GGC	27	4
62083	6	6677864	6677881	2	9	18	CA	18	10
62093	6	6704432	6704443	2	6	12	AC	22	4
62095	6	6714008	6714025	3	6	18	GGA	9	4
62096	6	6714104	6714127	2	12	24	CA	16	18
62098	6	6714276	6714289	2	7	14	CT	23	3
62112	6	6723336	6723349	2	7	14	GA	26	4
62114	6	6728433	6728448	2	8	16	TG	23	6
62117	6	6730088	6730101	2	7	14	CA	17	6
62118	6	6730172	6730187	2	8	16	CA	20	4
62119	6	6730351	6730370	2	10	20	CA	19	7
62120	6	6730966	6730977	2	6	12	CA	16	4
62122	6	6733198	6733236	3	13	39	TCT	23	10
62124	6	6736105	6736118	2	7	14	TG	24	3
62126	6	6739255	6739278	2	12	24	TG	12	4
62131	6	6741750	6741771	2	11	22	TG	10	4
62132	6	6741833	6741858	2	13	26	GT	13	9
62137	6	6746819	6746864	2	23	46	CT	15	5
62139	6	6747144	6747164	3	7	21	ATC	16	3
62144	6	6748792	6748811	2	10	20	CT	15	4
62145	6	6748998	6749027	2	15	30	CA	14	9
62159	6	6756788	6756819	2	16	32	GT	17	12
62171	6	6764725	6764738	2	7	14	GA	12	3
62174	6	6770083	6770097	3	5	15	GAG	19	3
62180	6	6775253	6775278	2	13	26	TG	19	13
62182	6	6776875	6776889	3	5	15	GCA	29	3

62185	6	6785110	6785125	4	4	16	AGAA	12	4
62188	6	6786357	6786370	2	7	14	GT	16	7
62189	6	6787313	6787339	3	9	27	GCA	27	6
62191	6	6788595	6788630	4	9	36	CAGG	28	18
62197	6	6799727	6799742	2	8	16	CA	17	5
62199	6	6800884	6800898	3	5	15	GGA	20	3
62202	6	6804952	6804971	2	10	20	CA	31	6
62210	6	6820842	6820855	2	7	14	AT	22	3
62212	6	6822047	6822066	2	10	20	CA	24	12
62215	6	6828767	6828798	2	16	32	TG	10	7
62217	6	6830149	6830164	4	4	16	TTTA	14	8
62219	6	6841768	6841779	2	6	12	GT	21	3
62220	6	6842797	6842832	2	18	36	CA	9	10
62222	6	6846395	6846410	4	4	16	TTTG	31	3
62223	6	6847246	6847265	2	10	20	TG	24	9
62225	6	6850019	6850038	2	10	20	AC	18	9
62238	6	6889108	6889119	2	6	12	TG	26	5
62243	6	6895066	6895083	2	9	18	TC	22	3
62251	6	6897335	6897346	2	6	12	TC	11	4
62256	6	6902730	6902751	2	11	22	GA	16	8
62257	6	6903643	6903666	3	8	24	GCT	27	9
62258	6	6905013	6905030	2	9	18	CA	32	3
62269	6	6929409	6929428	2	10	20	CA	19	7
62272	6	6930385	6930400	2	8	16	AG	15	8
62280	6	6946478	6946503	2	13	26	GT	8	9
62283	6	6950473	6950490	3	6	18	CAA	24	3
62293	6	6966768	6966787	4	5	20	CTGT	13	6
62297	6	6971112	6971127	2	8	16	GT	11	5
62298	6	6971335	6971346	2	6	12	TC	30	4
62301	6	6983380	6983394	3	5	15	TTG	12	15
62306	6	6997022	6997057	2	18	36	CA	21	15
62308	6	6999464	6999483	2	10	20	CT	22	5
62309	6	7003065	7003082	2	9	18	TG	16	10
62324	6	7027698	7027711	2	7	14	CA	25	4
62328	6	7031240	7031263	2	12	24	TG	21	5
62331	6	7035153	7035174	2	11	22	CA	13	7
62332	6	7040663	7040683	3	7	21	GAT	19	5
62338	6	7046045	7046078	2	17	34	TG	11	11
62339	6	7050162	7050173	2	6	12	AC	26	3
62340	6	7052641	7052670	2	15	30	TC	10	11
62346	6	7061732	7061751	2	10	20	AC	25	10
62351	6	7071115	7071130	2	8	16	TG	17	6
62352	6	7071199	7071230	2	16	32	GT	12	8
62353	6	7071330	7071357	2	14	28	TG	21	13
62362	6	7083890	7083905	4	4	16	TTTA	13	3
62369	6	7092276	7092313	2	19	38	GA	6	7
62373	6	7095334	7095355	2	11	22	AC	17	8
62374	6	7097515	7097536	2	11	22	AC	17	8
62376	6	7104069	7104088	2	10	20	CA	20	12
62377	6	7106035	7106064	2	15	30	CA	11	11
62380	6	7108261	7108286	2	13	26	TG	12	9

62382	6	7109783	7109798	4	4	16	TCCC	13	3
62383	6	7111428	7111453	2	13	26	CA	13	6
62384	6	7111520	7111531	2	6	12	CA	20	4
62385	6	7111780	7111797	2	9	18	CA	18	6
62395	6	7119952	7119975	2	12	24	GT	14	8
62398	6	7120841	7120866	2	13	26	GT	14	4
62399	6	7121028	7121051	2	12	24	CT	14	5
62402	6	7122458	7122469	2	6	12	GT	15	3
62403	6	7123646	7123669	2	12	24	TG	21	18
62404	6	7124782	7124801	4	5	20	TATC	14	6
62407	6	7129538	7129561	3	8	24	CTC	20	6
62410	6	7133584	7133598	3	5	15	GGT	13	7
62411	6	7134684	7134701	3	6	18	CAA	24	5
62416	6	7145458	7145473	2	8	16	AC	24	5
62422	6	7157552	7157583	2	16	32	AC	15	14
62424	6	7159972	7159987	2	8	16	AC	20	5
62425	6	7160165	7160194	2	15	30	CA	17	11
62429	6	7166308	7166333	2	13	26	CA	16	10
62433	6	7173513	7173566	2	27	54	CA	15	9
62440	6	7183594	7183621	2	14	28	TG	16	10
62442	6	7187114	7187149	2	18	36	TG	7	7
62444	6	7188686	7188725	2	20	40	TG	10	10
62446	6	7189064	7189081	2	9	18	TC	17	3
62452	6	7206125	7206150	2	13	26	GT	18	10
62455	6	7216770	7216817	2	24	48	CA	6	11
62461	6	7223503	7223518	2	8	16	TC	21	4
62467	6	7229426	7229445	2	10	20	TG	21	11
62471	6	7234897	7234910	2	7	14	AC	9	6
62475	6	7243011	7243032	2	11	22	TG	15	11
62477	6	7246241	7246252	2	6	12	GT	13	6
62478	6	7246267	7246282	2	8	16	GT	7	3
62483	6	7248389	7248402	2	7	14	GT	6	4
62485	6	7256298	7256313	2	8	16	GT	26	3
62487	6	7259870	7259881	2	6	12	GT	21	10
62488	6	7260340	7260351	2	6	12	GT	26	3
62489	6	7261578	7261589	2	6	12	CA	20	10
62492	6	7266819	7266870	2	26	52	AC	11	13
62493	6	7269005	7269020	2	8	16	GT	24	4
62496	6	7272458	7272475	2	9	18	AC	17	7
62505	6	7282151	7282182	2	16	32	CA	11	7
62508	6	7285976	7285990	3	5	15	GGA	30	4
62515	6	7299618	7299645	2	14	28	GT	23	15
62516	6	7300354	7300365	2	6	12	CA	20	7
62524	6	7316457	7316474	2	9	18	AC	7	3
62527	6	7321715	7321728	2	7	14	AC	25	3
62530	6	7329888	7329908	3	7	21	GAT	27	5
62531	6	7335580	7335591	2	6	12	GT	29	4
62533	6	7346384	7346401	2	9	18	GT	26	7
62538	6	7358519	7358545	3	9	27	TCC	24	9
62560	6	7388133	7388146	2	7	14	GT	26	6
62564	6	7394727	7394764	2	19	38	AG	9	6

62565	6	7395959	7395978	2	10	20	GT	20	5
62567	6	7398137	7398152	2	8	16	AC	15	6
62571	6	7414530	7414551	2	11	22	TG	19	9
62575	6	7422914	7422955	2	21	42	CA	17	15
62581	6	7439402	7439417	4	4	16	GACA	21	5
62584	6	7441442	7441455	2	7	14	GT	32	6
62588	6	7442733	7442756	2	12	24	CA	20	8
62589	6	7442972	7442985	2	7	14	AC	26	3
62591	6	7443203	7443224	2	11	22	AG	23	5
62594	6	7443592	7443605	2	7	14	GT	26	5
62595	6	7444249	7444290	2	21	42	AG	7	9
62596	6	7444404	7444433	2	15	30	TG	12	10
62598	6	7445947	7445978	2	16	32	TG	14	9
62600	6	7446901	7446928	2	14	28	TG	17	9
62601	6	7448323	7448346	4	6	24	TTCC	20	3
62602	6	7448558	7448577	2	10	20	GT	15	6
62607	6	7451472	7451489	2	9	18	GT	6	6
62609	6	7455446	7455463	3	6	18	GCA	24	4
62610	6	7458671	7458690	2	10	20	TG	29	7
62611	6	7459944	7459961	2	9	18	GA	26	6
62613	6	7460765	7460782	2	9	18	TG	17	5
62615	6	7461794	7461819	2	13	26	GT	14	12
62617	6	7465973	7466006	2	17	34	AC	13	12
62623	6	7472839	7472858	2	10	20	TG	19	10
62625	6	7474656	7474679	2	12	24	AC	13	6
62626	6	7484136	7484151	2	8	16	TC	15	3
62627	6	7484236	7484249	2	7	14	CA	21	4
62629	6	7487052	7487065	2	7	14	TG	24	4
62630	6	7487103	7487116	2	7	14	TG	24	9
62631	6	7488916	7488936	3	7	21	TCC	20	5
62636	6	7494194	7494231	2	19	38	GA	7	9
62637	6	7498469	7498482	2	7	14	TG	11	6
62639	6	7499445	7499462	2	9	18	AC	31	4
62641	6	7503868	7503882	3	5	15	TCT	28	3
62648	6	7514201	7514224	2	12	24	CA	18	5
62651	6	7517772	7517789	3	6	18	ATT	17	8
62653	6	7525160	7525179	2	10	20	TC	18	11
62654	6	7525389	7525414	2	13	26	TG	19	6
62656	6	7547970	7547990	3	7	21	TTG	21	4
62657	6	7548208	7548223	4	4	16	TTTA	21	3
62659	6	7551662	7551677	4	4	16	ATTT	20	5
62660	6	7551984	7552009	2	13	26	TG	20	7
62664	6	7557050	7557065	2	8	16	TA	14	5
62666	6	7558008	7558019	2	6	12	AC	24	4
62674	6	7567226	7567246	3	7	21	GCA	21	7
62676	6	7570200	7570211	2	6	12	AT	26	5
62681	6	7573161	7573180	2	10	20	CA	18	9
62683	6	7573680	7573691	2	6	12	GT	25	6
62684	6	7578701	7578730	2	15	30	TG	21	10
62686	6	7579459	7579478	2	10	20	TG	13	12
62690	6	7585325	7585338	2	7	14	GT	20	4

62692	6	7591600	7591614	3	5	15	ATT	18	11
62694	6	7591967	7591982	2	8	16	GT	8	6
62695	6	7592228	7592241	2	7	14	AC	28	6
62700	6	7596488	7596503	2	8	16	CA	22	3
62702	6	7600374	7600400	3	9	27	GAT	23	3
62703	6	7601256	7601275	2	10	20	CT	9	3
62706	6	7605029	7605042	2	7	14	CA	30	3
62711	6	7609212	7609245	2	17	34	TG	14	12
62713	6	7611645	7611664	4	5	20	AAAG	6	5
62714	6	7612217	7612242	2	13	26	AC	13	11
62716	6	7612680	7612693	2	7	14	CA	12	12
62725	6	7634185	7634198	2	7	14	AT	24	3
62726	6	7635561	7635584	2	12	24	AC	16	8
62729	6	7637855	7637868	2	7	14	GT	27	7
62730	6	7638146	7638165	2	10	20	AC	14	4
62735	6	7647001	7647018	3	6	18	TGT	17	8
62739	6	7655477	7655488	2	6	12	TC	20	3
62742	6	7662339	7662354	4	4	16	TTTA	24	7
62748	6	7681473	7681514	2	21	42	CT	22	10
62751	6	7687397	7687414	2	9	18	TG	8	9
62771	6	7714546	7714565	2	10	20	TG	25	10
62772	6	7714915	7714948	2	17	34	TC	7	7
62776	6	7715985	7715999	3	5	15	CCT	10	3
62777	6	7716601	7716627	3	9	27	TGA	28	6
62780	6	7720725	7720748	2	12	24	AC	11	11
62784	6	7723382	7723399	2	9	18	GT	23	6
62791	6	7726103	7726122	2	10	20	GA	27	4
62793	6	7726900	7726917	2	9	18	GA	22	7
62794	6	7730410	7730431	2	11	22	GT	16	9
62798	6	7735435	7735450	2	8	16	AG	26	6
62801	6	7739696	7739713	2	9	18	AG	15	6
62803	6	7744268	7744283	2	8	16	TA	10	3
62807	6	7750063	7750086	2	12	24	CT	16	7
62808	6	7750918	7750935	2	9	18	TG	19	4
62817	6	7758825	7758840	2	8	16	AT	11	4
62822	6	7776892	7776921	2	15	30	TG	16	12
62828	6	7786227	7786260	2	17	34	GT	10	12
62835	6	7801850	7801873	3	8	24	CTG	23	8
62838	6	7805877	7805890	2	7	14	AC	28	3
62846	6	7825439	7825460	2	11	22	CA	15	9
62860	6	7840412	7840423	2	6	12	TG	24	3
62868	6	7848970	7848984	3	5	15	CAA	23	4
62870	6	7861624	7861635	2	6	12	CT	18	4
62874	6	7874857	7874871	3	5	15	GTT	6	3
62891	6	7908523	7908536	2	7	14	AG	21	5
62893	6	7910373	7910384	2	6	12	CA	13	7
62902	6	7922930	7922965	2	18	36	AC	6	6
62907	6	7925907	7925934	2	14	28	CA	10	8
62910	6	7934946	7934969	2	12	24	CA	6	8
62913	6	7938365	7938380	4	4	16	AAAC	31	4
62914	6	7938651	7938674	3	8	24	GCT	22	7

62915	6	7939313	7939328	2	8	16	TG	18	4
62916	6	7939994	7940031	2	19	38	AC	11	10
62917	6	7942118	7942141	2	12	24	GA	12	9
62918	6	7942312	7942331	2	10	20	TG	18	6
62936	6	7957788	7957807	2	10	20	TG	12	5
62937	6	7958326	7958349	2	12	24	AC	21	10
62938	6	7959674	7959688	3	5	15	AAC	27	11
62939	6	7959948	7959965	3	6	18	CTG	13	6
62942	6	7960444	7960464	3	7	21	TCC	17	4
62948	6	7971186	7971199	2	7	14	AC	23	3
62950	6	7976919	7976948	2	15	30	AC	10	8
62953	6	7981397	7981422	2	13	26	CA	14	17
62954	6	7984498	7984511	2	7	14	TG	27	4
62955	6	7987820	7987835	2	8	16	CA	24	12
62957	6	7995047	7995062	4	4	16	ATAG	19	10
62959	6	8000762	8000773	2	6	12	CA	25	3
62969	6	8019672	8019697	2	13	26	TG	18	8
62971	6	8030005	8030040	2	18	36	AC	8	11
62972	6	8030634	8030647	2	7	14	GA	19	4
62983	6	8040980	8040999	2	10	20	GT	9	9
62985	6	8048766	8048783	3	6	18	ATA	22	4
62988	6	8053160	8053183	2	12	24	TG	14	9
62992	6	8054484	8054497	2	7	14	TG	24	7
62993	6	8056218	8056265	2	24	48	TC	7	11
62995	6	8062673	8062708	4	9	36	TATT	13	5
63002	6	8079008	8079019	2	6	12	AC	15	11
63003	6	8079159	8079182	2	12	24	AC	14	19
63005	6	8080693	8080716	2	12	24	AC	10	6
63006	6	8082450	8082473	2	12	24	GT	15	6
63012	6	8090789	8090812	2	12	24	CA	23	7
63015	6	8094715	8094730	2	8	16	CA	6	7
63016	6	8097259	8097270	2	6	12	CA	17	3
63017	6	8098165	8098180	4	4	16	TTTG	18	4
63018	6	8099426	8099443	2	9	18	AC	19	12
63023	6	8102451	8102468	2	9	18	AG	11	3
63025	6	8104410	8104427	2	9	18	CA	22	11
63026	6	8104430	8104443	2	7	14	CA	19	12
63027	6	8104554	8104571	2	9	18	TG	16	4
63031	6	8111113	8111156	4	11	44	GATA	13	6
63032	6	8111315	8111342	2	14	28	CA	19	8
63041	6	8119575	8119604	2	15	30	TG	13	8
63043	6	8120286	8120313	2	14	28	GT	22	10
63044	6	8123275	8123292	2	9	18	AC	17	8
63045	6	8126799	8126834	2	18	36	TG	19	12
63048	6	8129226	8129257	2	16	32	AC	8	8
63049	6	8129384	8129401	2	9	18	AC	7	3
63051	6	8132619	8132638	2	10	20	CA	16	5
63053	6	8132891	8132902	2	6	12	GT	13	6
63055	6	8136718	8136729	2	6	12	TG	29	6
63059	6	8139035	8139074	2	20	40	CA	10	7
63062	6	8147898	8147925	2	14	28	AC	7	6

63064	6	8149662	8149681	4	5	20	TATC	23	3
63066	6	8152297	8152318	2	11	22	AC	21	6
63067	6	8153014	8153041	2	14	28	CA	7	7
63081	6	8172273	8172302	2	15	30	CA	18	8
63085	6	8175941	8175958	2	9	18	TG	16	4
63090	6	8197322	8197335	2	7	14	AC	18	3
63091	6	8198315	8198370	2	28	56	TG	6	7
63092	6	8198664	8198677	2	7	14	GC	23	7
63093	6	8201879	8201904	2	13	26	CA	23	12
63100	6	8206123	8206148	2	13	26	CA	11	10
63101	6	8206331	8206366	3	12	36	ATC	15	6
63108	6	8209424	8209439	2	8	16	TG	17	10
63110	6	8210056	8210079	2	12	24	CA	8	6
63113	6	8210808	8210835	2	14	28	TC	7	4
63120	6	8214069	8214080	2	6	12	TG	20	5
63122	6	8216881	8216906	2	13	26	TG	16	10
63124	6	8218467	8218482	2	8	16	TG	12	5
63127	6	8218945	8218962	2	9	18	CA	23	8
63131	6	8225648	8225661	2	7	14	CT	28	3
63134	6	8227160	8227201	3	14	42	GAA	6	7
63136	6	8227721	8227754	2	17	34	AC	9	10
63142	6	8234690	8234709	2	10	20	GT	22	4
63143	6	8235254	8235273	2	10	20	GT	14	6
63146	6	8247252	8247281	2	15	30	CT	6	6
63147	6	8248644	8248661	2	9	18	GT	14	8
63150	6	8248923	8248950	2	14	28	TG	25	9
63153	6	8250666	8250681	4	4	16	TACA	16	4
63154	6	8253418	8253445	2	14	28	CA	19	8
63162	6	8259002	8259023	2	11	22	GT	17	9
63164	6	8262491	8262506	2	8	16	CA	20	6
63165	6	8262847	8262864	2	9	18	AC	8	4
63168	6	8263511	8263526	2	8	16	TC	28	3
63169	6	8265657	8265672	2	8	16	AC	18	3
63173	6	8268965	8269002	2	19	38	GT	12	8
63174	6	8269181	8269202	2	11	22	AC	21	8
63176	6	8271001	8271026	2	13	26	CA	20	14
63183	6	8279060	8279085	2	13	26	GA	22	13
63185	6	8279636	8279663	2	14	28	TG	6	8
63188	6	8282708	8282723	4	4	16	TTTC	15	3
63190	6	8285289	8285304	2	8	16	AC	20	5
63192	6	8286660	8286673	2	7	14	AC	30	4
63197	6	8291058	8291069	2	6	12	AC	8	3
63201	6	8293091	8293134	2	22	44	TG	17	14
63202	6	8293813	8293828	4	4	16	TTAT	25	4
63206	6	8295652	8295677	2	13	26	TG	11	12
63209	6	8296160	8296187	2	14	28	TG	13	7
63210	6	8296483	8296502	2	10	20	CA	13	4
63219	6	8302039	8302068	2	15	30	TG	14	12
63220	6	8306239	8306264	2	13	26	TG	23	9
63222	6	8309887	8309900	2	7	14	CA	22	5
63230	6	8318413	8318432	4	5	20	TGGT	18	3

63232	6	8319065	8319090	2	13	26	TG	6	9
63234	6	8319667	8319686	2	10	20	AC	17	6
63235	6	8320105	8320158	2	27	54	AC	6	7
63239	6	8324249	8324262	2	7	14	TG	22	9
63242	6	8337774	8337797	2	12	24	TG	19	7
63243	6	8338351	8338365	3	5	15	CCA	19	3
63244	6	8338668	8338679	2	6	12	AC	30	4
63246	6	8344407	8344422	2	8	16	CA	26	4
63249	6	8347332	8347343	2	6	12	TG	7	3
63250	6	8347756	8347773	2	9	18	TC	20	4
63260	6	8366861	8366896	2	18	36	AC	8	9
63264	6	8370318	8370341	2	12	24	TC	13	7
63266	6	8371916	8371941	2	13	26	TG	15	5
63271	6	8378213	8378230	2	9	18	GT	30	8
63273	6	8378913	8378948	2	18	36	GT	12	5
63275	6	8381418	8381441	2	12	24	TG	14	15
63277	6	8382460	8382489	2	15	30	CA	17	14
63282	6	8383763	8383774	2	6	12	AG	27	3
63283	6	8383777	8383788	2	6	12	AG	28	3
63287	6	8389551	8389576	2	13	26	AC	13	13
63289	6	8394790	8394807	2	9	18	CA	20	8
63290	6	8395530	8395544	3	5	15	CAG	24	3
63293	6	8397265	8397298	2	17	34	CA	14	7
63294	6	8397613	8397628	2	8	16	GT	17	7
63297	6	8403958	8403979	2	11	22	AC	24	7
63300	6	8407677	8407688	2	6	12	CA	24	5
63301	6	8407848	8407863	2	8	16	GA	22	6
63303	6	8410762	8410783	2	11	22	TG	25	6
63308	6	8419950	8419961	2	6	12	GT	31	6
63314	6	8429324	8429335	2	6	12	AC	16	6
63315	6	8429338	8429353	2	8	16	AC	20	10
63319	6	8433292	8433309	3	6	18	TTG	16	4
63323	6	8443137	8443148	2	6	12	AC	18	8
63326	6	8446976	8446995	2	10	20	TG	16	10
63327	6	8447197	8447218	2	11	22	TG	8	5
63329	6	8453906	8453923	3	6	18	TTG	15	3
63330	6	8454308	8454325	3	6	18	TGT	21	3
63332	6	8455285	8455308	3	8	24	TCC	16	5
63339	6	8458002	8458019	3	6	18	TCT	21	3
63342	6	8462027	8462050	2	12	24	AC	22	10
63345	6	8464321	8464334	2	7	14	GT	13	4
63347	6	8464479	8464506	2	14	28	AC	11	9
63354	6	8469583	8469596	2	7	14	CA	15	3
63355	6	8469793	8469808	2	8	16	TG	27	6
63356	6	8470957	8470974	2	9	18	CA	19	7
63360	6	8475299	8475322	2	12	24	AC	13	8
63371	6	8499985	8499996	2	6	12	CA	12	4
63372	6	8500311	8500340	2	15	30	GT	8	8
63374	6	8506721	8506735	3	5	15	TTG	24	3
63376	6	8508981	8508998	3	6	18	TTA	14	7
63382	6	8521950	8521965	4	4	16	TTGT	30	3

63384	6	8522820	8522843	2	12	24	AC	25	14
63385	6	8524059	8524102	2	22	44	GT	12	10
63387	6	8532422	8532449	2	14	28	CA	6	5
63390	6	8534111	8534134	2	12	24	AC	19	7
63394	6	8536350	8536367	3	6	18	GCT	31	3
63395	6	8536954	8536967	2	7	14	CA	22	3
63397	6	8538049	8538066	2	9	18	TG	19	3
63398	6	8538463	8538494	2	16	32	TG	19	13
63399	6	8540147	8540164	2	9	18	AC	21	3
63400	6	8540412	8540423	2	6	12	TG	16	3
63402	6	8542409	8542426	2	9	18	TG	20	6
63403	6	8542429	8542442	2	7	14	TG	20	9
63409	6	8544099	8544114	2	8	16	TC	7	6
63411	6	8549818	8549841	2	12	24	CA	24	5
63413	6	8555280	8555295	2	8	16	AC	22	4
63420	6	8568855	8568872	2	9	18	TG	10	6
63425	6	8578537	8578568	2	16	32	AC	8	10
63430	6	8583764	8583775	2	6	12	CA	24	4
63431	6	8586307	8586322	2	8	16	TC	26	6
63432	6	8587317	8587330	2	7	14	CT	21	4
63433	6	8587925	8587952	2	14	28	AC	14	10
63436	6	8591779	8591810	2	16	32	AG	24	5
63439	6	8596529	8596546	2	9	18	CA	28	7
63440	6	8596548	8596559	2	6	12	AC	28	7
63442	6	8597767	8597786	2	10	20	TG	25	7
63443	6	8603351	8603374	2	12	24	AC	12	7
63447	6	8614815	8614852	2	19	38	AC	13	9
63449	6	8617547	8617566	2	10	20	CA	16	4
63451	6	8620618	8620633	2	8	16	TG	23	4
63453	6	8624709	8624722	2	7	14	TG	27	3
63457	6	8630307	8630350	2	22	44	TG	8	6
63462	6	8634036	8634053	3	6	18	CCT	22	4
63467	6	8636608	8636631	4	6	24	AAAG	8	4
63468	6	8636900	8636927	2	14	28	CT	22	8
63475	6	8642130	8642147	2	9	18	TG	25	9
63477	6	8642795	8642816	2	11	22	GT	19	8
63478	6	8643101	8643134	2	17	34	AC	18	16
63480	6	8644678	8644691	2	7	14	TG	20	4
63482	6	8646926	8646967	3	14	42	CTT	25	9
63484	6	8647818	8647849	2	16	32	TG	16	11
63491	6	8658357	8658372	2	8	16	CA	23	8
63500	6	8662366	8662381	2	8	16	GT	8	7
63506	6	8665758	8665775	2	9	18	CT	13	6
63507	6	8665777	8665796	2	10	20	TC	10	6
63524	6	8703373	8703388	2	8	16	GT	10	11
63529	6	8710554	8710593	2	20	40	TC	11	11
63550	6	8749753	8749766	2	7	14	GT	23	7
63560	6	8789454	8789477	4	6	24	AAAT	18	5
63563	6	8796324	8796349	2	13	26	TG	14	11
63564	6	8796956	8796971	2	8	16	AC	11	7
63566	6	8800221	8800250	2	15	30	CA	15	12

63568	6	8803690	8803707	3	6	18	TAA	23	9
63577	6	8812705	8812716	2	6	12	TG	29	3
63578	6	8815726	8815765	2	20	40	CA	8	7
63581	6	8816611	8816622	2	6	12	AC	23	7
63582	6	8816624	8816639	2	8	16	CA	22	4
63583	6	8816806	8816825	2	10	20	CA	14	9
63584	6	8827691	8827708	2	9	18	TG	23	4
63593	6	8841684	8841697	2	7	14	CA	23	4
63597	6	8849330	8849347	2	9	18	GT	17	3
63601	6	8859201	8859216	2	8	16	TG	18	3
63602	6	8862261	8862280	4	5	20	AGGA	30	3
63606	6	8866872	8866891	2	10	20	TG	17	4
63608	6	8867954	8867979	2	13	26	GT	13	4
63610	6	8869704	8869731	2	14	28	TG	17	8
63614	6	8872833	8872856	4	6	24	AACC	21	4
63617	6	8880090	8880125	2	18	36	TG	12	11
63620	6	8884250	8884275	2	13	26	GT	19	7
63621	6	8885853	8885876	2	12	24	CA	17	7
63622	6	8902355	8902381	3	9	27	CAG	21	5
63623	6	8910464	8910477	2	7	14	AC	20	7
63625	6	8917561	8917572	2	6	12	TG	15	3
63628	6	8922458	8922471	2	7	14	TG	22	4
63630	6	8925019	8925032	2	7	14	TC	18	8
63633	6	8927445	8927462	3	6	18	TCC	18	3
63634	6	8927973	8927988	2	8	16	TG	16	7
63635	6	8931222	8931245	2	12	24	GT	20	8
63638	6	8939952	8939967	4	4	16	GCTA	23	6
63643	6	8951812	8951831	2	10	20	GT	14	15
63645	6	8952050	8952063	2	7	14	CA	29	4
63646	6	8957226	8957243	2	9	18	GT	27	5
63652	6	8962646	8962659	2	7	14	CT	21	3
63654	6	8962790	8962807	2	9	18	GT	16	8
63656	6	8965469	8965502	2	17	34	TG	20	9
63661	6	8979313	8979334	2	11	22	GT	15	9
63668	6	8999791	8999812	2	11	22	CA	18	12
63671	6	9003887	9003900	2	7	14	GT	30	8
63673	6	9005561	9005611	3	17	51	CAG	24	7
63675	6	9011558	9011577	2	10	20	GT	17	8
63677	6	9013900	9013915	2	8	16	CT	28	5
63679	6	9016762	9016789	4	7	28	ACAG	30	11
63681	6	9020593	9020607	3	5	15	CTC	25	6
63683	6	9026716	9026735	4	5	20	TTTA	24	4
63684	6	9027653	9027664	2	6	12	CA	28	5
63685	6	9028226	9028243	2	9	18	CA	25	4
63692	6	9034957	9034974	2	9	18	CA	13	7
63708	6	9070212	9070237	2	13	26	GA	6	9
63723	6	9115847	9115870	2	12	24	CA	26	6
63732	6	9118551	9118580	2	15	30	AC	10	11
63748	6	9145633	9145648	4	4	16	TCCA	27	4
63751	6	9150888	9150899	2	6	12	CA	20	3
63758	6	9164833	9164864	2	16	32	AC	14	11

63765	6	9172911	9172926	2	8	16	AT	23	14
63768	6	9185151	9185222	4	18	72	GATA	11	9
63771	6	9193957	9193970	2	7	14	AC	27	9
63777	6	9215663	9215686	4	6	24	AACC	26	6
63780	6	9228147	9228162	2	8	16	CA	20	6
63783	6	9230325	9230342	2	9	18	CA	19	4
63785	6	9232331	9232348	3	6	18	GCA	25	5
63795	6	9244965	9244992	2	14	28	AC	7	8
63800	6	9262466	9262483	3	6	18	CAG	29	5
63812	6	9277260	9277281	2	11	22	AC	18	10
63822	6	9295822	9295835	2	7	14	AC	12	5
63823	6	9297637	9297662	2	13	26	TG	22	9
63824	6	9298764	9298779	2	8	16	CA	24	6
63826	6	9300858	9300869	2	6	12	GA	8	3
63832	6	9305588	9305602	3	5	15	CTG	29	3
63833	6	9305941	9305958	2	9	18	AG	27	6
63838	6	9318973	9318990	2	9	18	TG	13	8
63845	6	9327746	9327765	4	5	20	GCAG	17	7
63850	6	9329167	9329184	3	6	18	AAG	13	4
63852	6	9332427	9332440	2	7	14	AG	12	4
63853	6	9332684	9332707	3	8	24	GCT	28	6
63860	6	9342986	9343003	2	9	18	AC	11	6
63865	6	9346546	9346560	3	5	15	GGC	29	4
63867	6	9348309	9348324	2	8	16	AC	20	7
63868	6	9348658	9348673	2	8	16	AG	15	3
63877	6	9350527	9350560	2	17	34	GT	8	6
63881	6	9353413	9353448	2	18	36	AC	6	5
63885	6	9360218	9360237	2	10	20	TG	21	6
63902	6	9373407	9373426	4	5	20	CAGC	27	4
63913	6	9384957	9384977	3	7	21	AAC	28	4
63914	6	9385159	9385174	2	8	16	AG	20	8
63919	6	9386221	9386238	2	9	18	GT	24	4
63920	6	9386472	9386486	3	5	15	CCA	27	3
63924	6	9391398	9391421	4	6	24	CTGT	15	6
63925	6	9392175	9392188	2	7	14	GT	18	6
63926	6	9392199	9392210	2	6	12	GT	18	3
63928	6	9393015	9393026	2	6	12	TG	24	4
63929	6	9393641	9393670	3	10	30	CAG	30	5
63931	6	9394074	9394085	2	6	12	CA	27	5
63932	6	9394268	9394283	2	8	16	GT	22	3
63936	6	9402385	9402422	2	19	38	GT	9	12
63937	6	9402469	9402482	2	7	14	GT	15	4
63939	6	9402990	9403011	2	11	22	TG	20	4
63940	6	9403115	9403148	2	17	34	TG	12	11
63941	6	9403368	9403399	2	16	32	CA	10	8
63947	6	9406008	9406025	3	6	18	TGT	18	4
63948	6	9407167	9407194	4	7	28	ATTC	18	4
63949	6	9409171	9409202	2	16	32	CA	15	9
63953	6	9409772	9409801	2	15	30	CA	6	5
63955	6	9410059	9410098	2	20	40	AC	10	11
63956	6	9410896	9410919	3	8	24	CCT	7	5

63964	6	9417241	9417274	2	17	34	GT	13	12
63971	6	9423300	9423327	2	14	28	CA	12	8
63973	6	9424055	9424078	3	8	24	TGA	21	5
63974	6	9426043	9426070	2	14	28	AC	19	8
63992	6	9455492	9455505	2	7	14	TG	17	4
63993	6	9456649	9456664	2	8	16	GT	15	5
63994	6	9457190	9457204	3	5	15	AAT	9	5
63997	6	9460788	9460805	3	6	18	CAC	10	4
63999	6	9462550	9462613	4	16	64	ATCT	8	11
64010	6	9476177	9476226	2	25	50	CA	6	7
64012	6	9480514	9480543	2	15	30	AC	9	9
64015	6	9485579	9485594	4	4	16	ACGG	21	5
64017	6	9486037	9486066	2	15	30	TG	17	11
64020	6	9487569	9487586	2	9	18	GT	17	5
64022	6	9487937	9487956	2	10	20	GA	16	6
64025	6	9490550	9490613	4	16	64	TCTA	10	8
64029	6	9497951	9497974	4	6	24	TATT	11	3
64032	6	9498549	9498569	3	7	21	CAG	7	3
64033	6	9498602	9498633	4	8	32	CCTG	9	4
64035	6	9499075	9499098	3	8	24	GCT	10	4
64038	6	9502734	9502769	3	12	36	GCT	27	10
64041	6	9505809	9505829	3	7	21	GCA	28	10
64042	6	9505982	9505995	2	7	14	AC	22	4
64043	6	9509327	9509348	2	11	22	TG	16	6
64044	6	9509427	9509462	2	18	36	TG	11	11
64046	6	9514491	9514514	4	6	24	ACAG	20	5
64053	6	9540768	9540819	2	26	52	TG	6	7
64054	6	9549257	9549274	2	9	18	TC	15	10
64057	6	9555180	9555191	2	6	12	CA	28	4
64076	6	9672858	9672885	2	14	28	TG	26	8
64078	6	9680634	9680657	4	6	24	TATT	12	4
64079	6	9683432	9683475	2	22	44	TG	8	9
64090	6	9712777	9712812	3	12	36	GAG	25	4
64092	6	9713936	9713947	2	6	12	CT	34	3
64093	6	9714011	9714022	2	6	12	GT	23	5
64100	6	9734264	9734289	2	13	26	TG	9	10
64102	6	9740368	9740387	2	10	20	CA	26	9
64112	6	9762067	9762078	2	6	12	CA	23	7
64119	6	9782119	9782142	4	6	24	TTTG	23	7
64126	6	9800389	9800434	2	23	46	AC	7	9
64127	6	9800996	9801016	3	7	21	GAG	31	14
64128	6	9811938	9811958	3	7	21	CTG	12	5
64134	6	9819588	9819605	2	9	18	AC	17	3
64137	6	9823497	9823510	2	7	14	AC	27	4
64140	6	9825720	9825735	4	4	16	ATTG	28	13
64142	6	9835961	9835976	2	8	16	CA	21	3
64144	6	9842943	9842968	2	13	26	AC	12	14
64149	6	9850073	9850098	2	13	26	AC	15	11
64150	6	9854874	9854891	2	9	18	AC	25	8
64153	6	9869875	9869898	2	12	24	AC	22	8
64161	6	9910045	9910058	2	7	14	CT	10	3

64163	6	9922319	9922352	2	17	34	CA	9	10
64165	6	9929121	9929150	2	15	30	AC	11	10
64171	6	9966276	9966289	2	7	14	TG	24	6
64172	6	9967290	9967313	2	12	24	GT	15	11
64175	6	9979030	9979053	4	6	24	GTCA	28	3
64184	6	9996897	9996928	2	16	32	TG	20	9
64186	6	10001544	10001564	3	7	21	CCT	25	6
64188	6	10004157	10004188	2	16	32	TG	14	15
64189	6	10008357	10008410	2	27	54	TG	11	12
64190	6	10008945	10008968	2	12	24	AC	17	10
64196	6	10013436	10013447	2	6	12	TC	24	3
64198	6	10019030	10019049	2	10	20	CA	23	8
64199	6	10019360	10019393	2	17	34	GT	7	6
64200	6	10026804	10026819	2	8	16	AC	26	3
64201	6	10030714	10030725	2	6	12	AG	26	3
64202	6	10032807	10032818	2	6	12	CA	13	4
64203	6	10033032	10033057	2	13	26	AC	11	7
64209	6	10043102	10043117	2	8	16	TC	28	3
64210	6	10043237	10043252	2	8	16	AG	22	3
64212	6	10046089	10046104	2	8	16	AC	28	4
64219	6	10057572	10057585	2	7	14	AG	22	4
64220	6	10058637	10058654	3	6	18	GTT	20	4
64224	6	10063046	10063073	2	14	28	GT	22	8
64231	6	10067778	10067813	2	18	36	TG	13	8
64232	6	10069081	10069106	2	13	26	AC	8	6
64236	6	10076384	10076407	2	12	24	CA	20	3
64245	6	10089861	10089880	2	10	20	CA	16	4
64249	6	10090824	10090841	2	9	18	CT	13	4
64259	6	10113686	10113713	2	14	28	TG	9	7
64261	6	10113852	10113881	2	15	30	AC	15	10
64265	6	10115678	10115717	2	20	40	AC	7	8
64270	6	10122442	10122493	2	26	52	AG	8	5
64271	6	10124931	10124946	2	8	16	GT	15	7
64272	6	10124948	10124959	2	6	12	TG	18	8
64274	6	10126112	10126127	2	8	16	GT	13	4
64285	6	10149707	10149724	2	9	18	AG	23	4
64286	6	10151196	10151227	4	8	32	GAAT	17	3
64289	6	10161864	10161875	2	6	12	TG	28	3
64296	6	10173854	10173874	3	7	21	ATT	15	3
64297	6	10175684	10175697	2	7	14	TG	18	4
64301	6	10187350	10187385	2	18	36	CA	14	12
64304	6	10188984	10189001	2	9	18	AC	14	4
64306	6	10207177	10207212	2	18	36	TG	12	14
64313	6	10228982	10229001	2	10	20	CA	13	5
64321	6	10245460	10245475	2	8	16	GT	16	6
64323	6	10247616	10247633	2	9	18	TC	27	4
64325	6	10251506	10251541	2	18	36	GT	9	10
64329	6	10256737	10256754	2	9	18	CA	32	5
64331	6	10260549	10260564	2	8	16	TG	22	5
64336	6	10261407	10261428	2	11	22	TG	6	4
64337	6	10261513	10261528	2	8	16	GT	16	4

64342	6	10272310	10272331	2	11	22	GA	22	9
64345	6	10275718	10275729	2	6	12	CT	25	3
64348	6	10279210	10279224	3	5	15	GAT	29	7
64352	6	10291427	10291441	3	5	15	TCT	34	9
64353	6	10298877	10298890	2	7	14	TA	21	9
64361	6	10308215	10308236	2	11	22	AG	19	3
64368	6	10316640	10316657	2	9	18	AC	18	4
64374	6	10331654	10331667	2	7	14	TG	20	5
64375	6	10335636	10335653	3	6	18	CCT	17	3
64376	6	10338760	10338779	2	10	20	CT	26	5
64382	6	10356126	10356159	2	17	34	TG	11	10
64383	6	10356409	10356440	2	16	32	CA	8	6
64384	6	10359417	10359436	2	10	20	TG	18	5
64389	6	10365542	10365562	3	7	21	CTC	20	3
64391	6	10366468	10366507	2	20	40	TG	8	9
64392	6	10366842	10366869	4	7	28	TGCC	23	6
64398	6	10383636	10383647	2	6	12	CT	33	3
64399	6	10385114	10385127	2	7	14	GT	9	7
64400	6	10385520	10385537	2	9	18	TG	25	4
64402	6	10387035	10387054	2	10	20	GT	15	3
64412	6	10405405	10405428	2	12	24	CA	21	15
64415	6	10410887	10410902	4	4	16	TCTT	22	3
64421	6	10421038	10421049	2	6	12	GT	26	4
64423	6	10424470	10424487	2	9	18	CT	7	4
64428	6	10432918	10432945	4	7	28	CCAT	23	5
64431	6	10434003	10434016	2	7	14	GT	23	8
64434	6	10443264	10443281	3	6	18	TGC	23	4
64435	6	10446661	10446684	4	6	24	CATC	26	3
64437	6	10449514	10449533	2	10	20	TG	17	9
64440	6	10452354	10452368	3	5	15	AGC	33	3
64458	6	10480128	10480153	2	13	26	AC	18	6
64463	6	10505236	10505263	2	14	28	CA	8	6
64465	6	10512246	10512265	4	5	20	CAGG	14	3
64468	6	10513316	10513336	3	7	21	GAT	17	4
64470	6	10516342	10516355	2	7	14	AC	25	11
64471	6	10516602	10516619	2	9	18	AC	22	9
64472	6	10517121	10517134	2	7	14	AC	18	7
64478	6	10539784	10539799	4	4	16	TTTG	24	4
64492	6	10568872	10568899	2	14	28	AG	12	4
64502	6	10596033	10596068	3	12	36	GTT	23	8
64505	6	10599995	10600014	2	10	20	TG	23	4
64511	6	10602155	10602182	2	14	28	TG	12	10
64514	6	10603120	10603135	4	4	16	TTTC	11	10
64515	6	10605630	10605647	2	9	18	GT	16	13
64516	6	10605818	10605845	4	7	28	AAAT	20	9
64521	6	10610249	10610266	2	9	18	GT	16	12
64528	6	10622310	10622327	3	6	18	AGC	21	5
64529	6	10622464	10622484	3	7	21	CTG	29	10
64531	6	10627571	10627592	2	11	22	TG	15	11
64534	6	10629365	10629388	2	12	24	CT	11	13
64539	6	10630245	10630259	3	5	15	CTG	25	3

64543	6	10648596	10648635	4	10	40	TTTC	8	6
64545	6	10649706	10649731	2	13	26	CA	7	9
64551	6	10662183	10662196	2	7	14	TA	10	4
64568	6	10703145	10703170	2	13	26	GT	19	7
64575	6	10716425	10716439	3	5	15	TTC	17	4
64576	6	10716470	10716481	2	6	12	AG	24	6
64578	6	10721524	10721550	3	9	27	ACT	13	6
64584	6	10728720	10728735	2	8	16	GT	20	3
64585	6	10731280	10731295	4	4	16	GCAG	20	3
64598	6	10769565	10769576	2	6	12	CA	23	4
64599	6	10769683	10769702	4	5	20	AAAT	21	3
64606	6	10797614	10797628	3	5	15	GAG	26	8
64609	6	10804847	10804864	2	9	18	CA	20	8
64610	6	10804866	10804881	2	8	16	AC	17	10
64611	6	10807029	10807040	2	6	12	AG	32	4
64615	6	10812398	10812415	2	9	18	GT	16	4
64617	6	10813213	10813228	2	8	16	AC	19	10
64618	6	10813508	10813541	2	17	34	AC	12	12
64619	6	10814017	10814043	3	9	27	TCC	16	9
64621	6	10814342	10814359	2	9	18	GT	18	8
64622	6	10816319	10816342	3	8	24	TCT	17	3
64625	6	10819743	10819758	2	8	16	TG	15	7
64628	6	10822417	10822436	4	5	20	TTGT	23	7
64631	6	10823635	10823648	2	7	14	AC	14	3
64632	6	10823880	10823891	2	6	12	GT	30	3
64635	6	10827552	10827577	2	13	26	GT	16	13
64637	6	10832559	10832582	3	8	24	TCT	18	7
64638	6	10833341	10833358	3	6	18	CCT	23	4
64639	6	10834857	10834876	2	10	20	AG	21	7
64640	6	10836159	10836172	2	7	14	AC	25	3
64641	6	10836444	10836461	2	9	18	GT	18	3
64642	6	10841194	10841211	3	6	18	CTG	30	3
64644	6	10842088	10842117	2	15	30	TC	21	10
64645	6	10842171	10842196	2	13	26	AC	14	10
64656	6	10861383	10861396	2	7	14	AC	25	8
64660	6	10862923	10862936	2	7	14	GT	25	4
64662	6	10870554	10870569	4	4	16	AAAT	9	4
64664	6	10877070	10877085	2	8	16	TG	24	6
64668	6	10880568	10880581	2	7	14	GT	20	6
64669	6	10880624	10880647	2	12	24	TG	13	8
64670	6	10880832	10880853	2	11	22	AG	14	5
64673	6	10884498	10884521	2	12	24	GA	16	8
64677	6	10891802	10891815	2	7	14	GT	22	3
64680	6	10894451	10894484	2	17	34	GT	7	12
64681	6	10895122	10895161	2	20	40	CA	19	15
64682	6	10895449	10895460	2	6	12	CA	24	3
64687	6	10896134	10896148	3	5	15	CAG	23	6
64688	6	10896672	10896687	2	8	16	GT	18	9
64689	6	10896691	10896704	2	7	14	TG	11	5
64690	6	10896947	10896964	2	9	18	CT	13	10
64691	6	10897021	10897040	2	10	20	CT	12	12

64694	6	10907039	10907054	2	8	16	AC	10	3
64697	6	10916850	10916873	2	12	24	GT	16	10
64698	6	10917388	10917411	2	12	24	TG	12	3
64701	6	10924657	10924674	2	9	18	AC	21	9
64702	6	10926918	10926937	2	10	20	AC	22	3
64703	6	10928432	10928471	2	20	40	CT	9	11
64708	6	10940792	10940809	2	9	18	GA	24	3
64709	6	10941035	10941062	2	14	28	TG	12	14
64723	6	10976578	10976603	2	13	26	AC	6	6
64727	6	10977398	10977415	2	9	18	TG	21	5
64736	6	10994796	10994810	3	5	15	AAG	19	3
64737	6	10995720	10995751	2	16	32	CA	7	8
64739	6	10997947	10997960	2	7	14	AC	24	6
64742	6	10999922	10999936	3	5	15	GAA	24	5
64744	6	11001197	11001217	3	7	21	TTG	21	4
64751	6	11029445	11029462	2	9	18	CA	18	4
64757	6	11036131	11036151	3	7	21	TCT	13	9
64758	6	11037896	11037907	2	6	12	CT	18	3
64759	6	11041125	11041144	4	5	20	GACA	31	4
64760	6	11053424	11053447	2	12	24	AC	15	7
64761	6	11054140	11054161	2	11	22	CA	6	6
64766	6	11083657	11083668	2	6	12	CA	27	4
64767	6	11083941	11083958	3	6	18	CAT	31	3
64772	6	11096815	11096844	2	15	30	AC	20	7
64779	6	11112884	11112907	2	12	24	GT	21	7
64783	6	11113729	11113746	2	9	18	CT	24	3
64784	6	11115240	11115253	2	7	14	AC	27	3
64786	6	11116251	11116282	2	16	32	AC	22	15
64794	6	11123459	11123472	2	7	14	GT	19	13
64797	6	11127450	11127461	2	6	12	AC	19	6
64803	6	11133674	11133705	4	8	32	CTGT	25	6
64810	6	11149208	11149225	2	9	18	TC	15	4
64811	6	11149227	11149238	2	6	12	CT	16	5
64813	6	11151638	11151649	2	6	12	AT	9	3
64814	6	11156824	11156837	2	7	14	AC	30	3
64817	6	11162934	11162945	2	6	12	CA	24	5
64829	6	11184121	11184144	2	12	24	GT	18	8
64836	6	11197772	11197791	2	10	20	GA	13	4
64841	6	11202558	11202581	2	12	24	AC	10	7
64843	6	11207685	11207700	2	8	16	TG	22	4
64846	6	11210465	11210492	2	14	28	CA	23	7
64849	6	11212187	11212201	3	5	15	GTG	24	5
64855	6	11222365	11222378	2	7	14	CT	30	4
64863	6	11235444	11235469	2	13	26	CA	13	10
64866	6	11246272	11246295	3	8	24	TGT	30	3
64872	6	11260711	11260740	3	10	30	GCT	17	6
64873	6	11280425	11280440	4	4	16	TCCA	30	3
64876	6	11287916	11287931	2	8	16	TC	23	5
64879	6	11301261	11301276	2	8	16	GT	15	5
64882	6	11313198	11313218	3	7	21	CTG	22	3
64884	6	11316456	11316475	4	5	20	AAAT	12	7

64885	6	11318450	11318473	2	12	24	TC	20	9
64895	6	11336339	11336356	3	6	18	TTG	25	3
64897	6	11337166	11337209	4	11	44	AAGA	17	8
64902	6	11352966	11352977	2	6	12	AC	13	4
64906	6	11357138	11357149	2	6	12	CT	25	3
64909	6	11368977	11368990	2	7	14	GT	27	5
64914	6	11373542	11373567	2	13	26	TG	7	7
64919	6	11383927	11383938	2	6	12	CA	29	4
64921	6	11386909	11386922	2	7	14	AC	18	4
64922	6	11386968	11386985	2	9	18	CA	19	3
64933	6	11393556	11393575	2	10	20	TG	12	5
64937	6	11394725	11394774	2	25	50	GT	16	14
64948	6	11403253	11403268	2	8	16	AC	10	3
64950	6	11403327	11403352	2	13	26	CA	15	12
64954	6	11417216	11417237	2	11	22	CA	18	10
64957	6	11424299	11424314	2	8	16	AC	8	6
64958	6	11432534	11432545	2	6	12	AC	24	3
64960	6	11441754	11441765	2	6	12	GA	22	3
64964	6	11454570	11454595	2	13	26	GT	13	7
64981	6	11484143	11484157	3	5	15	TGA	34	3
64987	6	11487451	11487482	2	16	32	CA	15	11
64989	6	11488885	11488908	3	8	24	GGT	15	3
64990	6	11489604	11489645	2	21	42	TG	12	8
64996	6	11499740	11499751	2	6	12	AC	17	4
64997	6	11499754	11499769	2	8	16	AC	21	5
65002	6	11508493	11508507	3	5	15	GAA	16	3
65015	6	11531483	11531510	2	14	28	CA	16	10
65016	6	11534178	11534213	3	12	36	AGG	13	6
65019	6	11537086	11537127	3	14	42	AGC	20	10
65024	6	11544650	11544661	2	6	12	AC	23	4
65035	6	11569051	11569064	2	7	14	AC	16	5
65043	6	11602021	11602044	2	12	24	CA	16	10
65044	6	11603472	11603485	2	7	14	GA	11	5
65047	6	11609296	11609319	2	12	24	TG	18	8
65049	6	11610487	11610508	2	11	22	AC	19	7
65052	6	11614372	11614407	2	18	36	CA	17	10
65058	6	11622384	11622407	3	8	24	CCT	26	9
65059	6	11622893	11622910	2	9	18	GT	14	9
65060	6	11624516	11624541	2	13	26	GT	17	6
65061	6	11625192	11625212	3	7	21	TGG	21	3
65062	6	11625399	11625414	2	8	16	TG	18	5
65068	6	11641524	11641549	2	13	26	TC	12	6
65071	6	11661908	11661931	2	12	24	AC	10	8
65073	6	11670082	11670101	2	10	20	GA	23	3
65074	6	11684617	11684642	2	13	26	TG	20	10
65076	6	11685496	11685515	2	10	20	TG	25	15
65081	6	11701369	11701388	2	10	20	CA	14	10
65086	6	11732572	11732585	2	7	14	TG	33	5
65088	6	11734397	11734422	2	13	26	GT	22	9
65089	6	11735126	11735153	4	7	28	AAAC	18	5
65091	6	11736123	11736136	2	7	14	AC	30	3

65098	6	11750559	11750590	2	16	32	CA	8	7
65099	6	11750865	11750888	4	6	24	TTTC	26	3
65101	6	11757222	11757241	2	10	20	CA	15	7
65102	6	11768685	11768696	2	6	12	AC	24	4
65107	6	11777153	11777172	2	10	20	GT	25	6
65109	6	11777441	11777468	2	14	28	GT	18	8
65117	6	11807545	11807560	4	4	16	ATAG	16	7
65119	6	11807667	11807680	2	7	14	AC	19	5
65123	6	11808024	11808047	3	8	24	CAG	8	7
65128	6	11808405	11808424	4	5	20	CCGA	28	4
65129	6	11809184	11809205	2	11	22	AC	18	15
65132	6	11814276	11814302	3	9	27	TCT	19	5
65139	6	11831673	11831706	2	17	34	GA	22	12
65148	6	11857478	11857507	2	15	30	AC	8	9
65149	6	11857913	11857934	2	11	22	AC	22	5
65151	6	11865520	11865535	4	4	16	ATAA	10	3
65158	6	11883950	11883961	2	6	12	AT	12	4
65163	6	11888981	11888998	2	9	18	AC	18	17
65167	6	11896778	11896791	2	7	14	GT	10	8
65171	6	11914702	11914716	3	5	15	TGC	29	4
65176	6	11936832	11936843	2	6	12	GT	30	10
65191	6	11960365	11960376	2	6	12	CT	19	6
65201	6	11982634	11982649	4	4	16	CTCA	29	3
65203	6	12002728	12002763	4	9	36	TATC	22	12
65208	6	12013076	12013099	2	12	24	GT	21	8
65211	6	12013648	12013663	2	8	16	TG	15	4
65218	6	12024701	12024726	2	13	26	AG	14	7
65225	6	12027073	12027092	2	10	20	GT	26	5
65226	6	12027667	12027682	2	8	16	TG	21	12
65230	6	12030075	12030090	2	8	16	AC	22	4
65231	6	12031110	12031121	2	6	12	TG	30	3
65233	6	12031427	12031440	2	7	14	GT	24	7
65239	6	12035197	12035220	2	12	24	TG	16	7
65241	6	12035386	12035403	2	9	18	CA	6	5
65242	6	12039129	12039162	2	17	34	TG	18	12
65243	6	12039429	12039442	2	7	14	CA	19	8
65246	6	12042348	12042369	2	11	22	AC	14	7
65247	6	12042540	12042559	2	10	20	CA	20	5
65248	6	12043938	12043961	2	12	24	GA	18	5
65264	6	12055261	12055288	2	14	28	CA	11	13
65270	6	12059520	12059543	2	12	24	CA	14	5
65275	6	12061306	12061320	3	5	15	CAG	17	4
65276	6	12061434	12061469	2	18	36	GT	12	13
65280	6	12070235	12070252	2	9	18	AG	15	3
65282	6	12071957	12071976	4	5	20	AACC	12	3
65283	6	12077301	12077326	2	13	26	AC	12	10
65286	6	12078057	12078071	3	5	15	GAG	11	3
65287	6	12082997	12083011	3	5	15	TCA	34	4
65288	6	12083177	12083194	2	9	18	CA	18	8
65290	6	12088388	12088405	3	6	18	AAT	18	5
65293	6	12097600	12097623	2	12	24	AC	19	6

65297	6	12110055	12110072	2	9	18	CA	27	6
65298	6	12110594	12110605	2	6	12	AC	34	5
65300	6	12114531	12114566	3	12	36	TGC	21	12
65303	6	12114834	12114867	2	17	34	AG	11	6
65304	6	12115145	12115166	2	11	22	TC	12	5
65308	6	12118129	12118143	3	5	15	AAG	25	4
65311	6	12122987	12123002	2	8	16	AG	8	3
65312	6	12125715	12125734	2	10	20	TG	32	13
65315	6	12135644	12135669	2	13	26	CA	28	7
65316	6	12135771	12135786	2	8	16	AC	25	4
65319	6	12141387	12141402	4	4	16	ATGT	13	3
65322	6	12142493	12142504	2	6	12	AT	18	3
65328	6	12147477	12147498	2	11	22	CA	15	6
65329	6	12147723	12147738	2	8	16	AC	18	4
65339	6	12165842	12165861	2	10	20	TG	15	16
65374	6	12229076	12229091	2	8	16	GT	29	3
65390	6	12261458	12261469	2	6	12	CA	20	4
65393	6	12268634	12268647	2	7	14	AT	14	4
65395	6	12277325	12277342	2	9	18	CA	20	6
65409	6	12327694	12327709	2	8	16	GT	28	4
65411	6	12329521	12329550	2	15	30	TG	22	7
65416	6	12344500	12344511	2	6	12	GT	17	6
65417	6	12351467	12351480	2	7	14	AG	29	3
65424	6	12358077	12358103	3	9	27	TCC	13	13
65426	6	12358224	12358241	3	6	18	TCC	11	6
65428	6	12368517	12368528	2	6	12	TA	14	4
65430	6	12377261	12377276	2	8	16	AC	22	7
65432	6	12377416	12377427	2	6	12	TG	30	3
65434	6	12382155	12382182	2	14	28	CA	16	15
65442	6	12406226	12406245	2	10	20	AT	12	7
65444	6	12413621	12413632	2	6	12	AT	21	3
65446	6	12416468	12416500	3	11	33	CAG	21	10
65447	6	12417346	12417402	3	19	57	CAG	16	12
65448	6	12419802	12419822	3	7	21	CAG	22	5
65449	6	12422745	12422760	4	4	16	AAAC	21	3
65451	6	12423692	12423721	2	15	30	TG	6	7
65457	6	12448021	12448034	2	7	14	TA	15	6
65460	6	12452142	12452155	2	7	14	CA	12	13
65462	6	12461481	12461492	2	6	12	AC	32	4
65467	6	12465450	12465473	4	6	24	ATTT	11	6
65468	6	12468448	12468467	2	10	20	TG	21	6
65469	6	12470907	12470926	2	10	20	TG	24	9
65472	6	12474054	12474095	2	21	42	GT	9	10
65476	6	12480972	12480995	2	12	24	AC	8	9
65477	6	12481967	12481984	2	9	18	TC	10	11
65479	6	12485112	12485125	2	7	14	TC	31	3
65480	6	12485665	12485676	2	6	12	TG	15	10
65481	6	12485899	12485916	2	9	18	TG	24	16
65488	6	12495341	12495356	4	4	16	ATGC	27	4
65498	6	12514736	12514759	3	8	24	AAT	22	7
65499	6	12516910	12516930	3	7	21	GCT	22	4

65508	6	12533494	12533511	2	9	18	GA	23	3
65516	6	12559456	12559469	2	7	14	AC	25	4
65521	6	12580574	12580589	4	4	16	ACGC	14	4
65522	6	12582603	12582629	3	9	27	TCA	22	10
65523	6	12583106	12583126	3	7	21	CAT	28	6
65524	6	12590019	12590032	2	7	14	CG	29	10
65525	6	12591654	12591665	2	6	12	AT	7	3
65552	6	12622420	12622435	4	4	16	CTTT	22	8
65560	6	12647801	12647820	2	10	20	GT	15	7
65566	6	12653458	12653493	2	18	36	GT	15	13
65569	6	12657543	12657574	2	16	32	AC	8	6
65571	6	12659792	12659809	2	9	18	AC	17	5
65573	6	12662639	12662666	4	7	28	AAAC	18	3
65575	6	12664769	12664788	2	10	20	GT	16	9
65576	6	12664971	12664988	3	6	18	CCT	19	5
65578	6	12665380	12665391	2	6	12	AT	11	6
65612	6	12697948	12697961	2	7	14	AT	10	5
65617	6	12704642	12704659	2	9	18	AG	24	4
65618	6	12706455	12706472	2	9	18	TG	24	4
65619	6	12706539	12706550	2	6	12	TC	22	4
65630	6	12729197	12729208	2	6	12	AC	25	6
65635	6	12734772	12734789	2	9	18	GT	23	4
65640	6	12742378	12742405	2	14	28	GT	8	10
65643	6	12744543	12744556	2	7	14	AG	27	4
65648	6	12757109	12757126	2	9	18	TC	17	7
65654	6	12763831	12763850	2	10	20	TG	19	3
65655	6	12765322	12765335	2	7	14	GT	25	4
65658	6	12767823	12767846	3	8	24	CTC	21	4
65659	6	12768419	12768442	2	12	24	AG	9	5
65661	6	12771327	12771342	2	8	16	CA	24	3
65663	6	12772431	12772448	2	9	18	CA	22	10
65665	6	12772890	12772904	3	5	15	GAG	26	3
65670	6	12778336	12778371	3	12	36	AAT	9	6
65682	6	12787176	12787197	2	11	22	TC	16	5
65685	6	12791525	12791536	2	6	12	TC	14	5
65688	6	12795961	12795976	2	8	16	TG	20	4
65698	6	12809576	12809607	2	16	32	TG	14	7
65701	6	12813626	12813637	2	6	12	GA	18	4
65702	6	12816119	12816132	2	7	14	AC	10	5
65703	6	12820192	12820206	3	5	15	GAG	29	3
65713	6	12835581	12835614	2	17	34	AC	11	11
65715	6	12836481	12836528	2	24	48	GT	7	12
65719	6	12840565	12840582	3	6	18	CTC	29	8
65721	6	12842216	12842239	2	12	24	CA	17	9
65722	6	12842735	12842770	2	18	36	AC	6	5
65727	6	12854107	12854118	2	6	12	CA	16	7
65728	6	12854864	12854875	2	6	12	GT	16	5
65738	6	12864082	12864093	2	6	12	AC	14	4
65739	6	12864095	12864108	2	7	14	CA	24	8
65745	6	12871325	12871362	2	19	38	GT	14	15
65748	6	12873544	12873559	2	8	16	TG	24	5

65749	6	12873993	12874010	2	9	18	AG	15	4
65751	6	12874280	12874293	2	7	14	TG	18	3
65756	6	12878677	12878698	2	11	22	AC	17	7
65757	6	12878860	12878871	2	6	12	GA	20	5
65778	6	12913065	12913082	2	9	18	AG	16	6
65781	6	12921193	12921226	2	17	34	AC	9	10
65782	6	12921283	12921296	2	7	14	CA	17	5
65783	6	12921298	12921313	2	8	16	AC	20	3
65786	6	12925303	12925317	3	5	15	GAG	8	3
65788	6	12927833	12927852	4	5	20	ATGT	30	3
65790	6	12929082	12929096	3	5	15	TGT	20	4
65794	6	12930593	12930608	2	8	16	AC	25	8
65795	6	12930761	12930776	4	4	16	CAGG	20	5
65808	6	12939236	12939249	2	7	14	AC	17	5
65809	6	12939480	12939507	2	14	28	GA	17	10
65812	6	12945474	12945487	2	7	14	CA	25	4
65813	6	12945637	12945652	2	8	16	AG	24	3
65824	6	12960063	12960074	2	6	12	AC	22	5
65826	6	12960985	12961000	2	8	16	TG	15	3
65828	6	12970501	12970522	2	11	22	AG	16	5
65829	6	12971207	12971226	2	10	20	AG	23	9
65830	6	12971497	12971508	2	6	12	TG	13	6
65832	6	12971680	12971703	2	12	24	GA	7	6
65835	6	12974526	12974553	2	14	28	GT	21	13
65846	6	12992377	12992404	2	14	28	GT	19	10
65848	6	12993238	12993255	2	9	18	CA	18	8
65850	6	12995065	12995076	2	6	12	CT	26	5
65874	6	13052288	13052305	2	9	18	CA	27	3
65875	6	13052624	13052643	2	10	20	AC	22	9
65878	6	13059387	13059418	2	16	32	AC	10	7
65879	6	13064134	13064157	2	12	24	GA	17	7
65880	6	13064729	13064752	2	12	24	GT	14	5
65890	6	13081449	13081470	2	11	22	TG	27	8
65893	6	13092999	13093022	4	6	24	AACC	23	6
65900	6	13108340	13108355	2	8	16	TC	21	5
65906	6	13130934	13130955	2	11	22	GT	19	9
65910	6	13133096	13133113	2	9	18	CA	12	3
65913	6	13135927	13135946	2	10	20	TG	13	3
65916	6	13136225	13136238	2	7	14	GA	21	3
65921	6	13136371	13136382	2	6	12	GA	22	4
65925	6	13138010	13138033	3	8	24	CCT	32	3
65938	6	13154391	13154402	2	6	12	TG	23	3
65948	6	13161130	13161143	2	7	14	TA	22	5
65956	6	13169306	13169337	2	16	32	TG	7	5
65964	6	13176673	13176718	2	23	46	GA	6	9
65967	6	13177446	13177469	2	12	24	AC	15	9
65971	6	13180953	13180988	3	12	36	AAC	13	7
65981	6	13197340	13197351	2	6	12	TC	22	3
65984	6	13205867	13205888	2	11	22	GT	19	5
65986	6	13210062	13210091	2	15	30	CA	13	7
65990	6	13221041	13221072	2	16	32	TG	12	8

65994	6	13226814	13226859	2	23	46	GT	13	8
66001	6	13242535	13242546	2	6	12	AC	23	3
66010	6	13266588	13266599	2	6	12	CA	23	7
66011	6	13268812	13268831	2	10	20	GT	17	6
66013	6	13269870	13269889	4	5	20	TGGA	25	3
66029	6	13300462	13300485	2	12	24	GT	13	7
66030	6	13300539	13300550	2	6	12	AC	26	4
66036	6	13313826	13313840	3	5	15	AAG	14	3
66045	6	13318079	13318096	3	6	18	CAC	27	5
66054	6	13328856	13328891	4	9	36	AGGC	31	3
66073	6	13366753	13366784	4	8	32	CCAT	25	5
66075	6	13371845	13371864	2	10	20	GA	15	12
66085	6	13388306	13388323	2	9	18	CT	20	4
66087	6	13395964	13395977	2	7	14	TG	24	5
66089	6	13398134	13398154	3	7	21	GAG	17	3
66095	6	13411835	13411864	2	15	30	AG	14	10
66101	6	13422650	13422663	2	7	14	TG	20	3
66103	6	13429569	13429595	3	9	27	CTG	28	12
66106	6	13443431	13443448	2	9	18	GA	20	6
66108	6	13445190	13445201	2	6	12	GT	30	3
66109	6	13447084	13447095	2	6	12	GT	16	4
66110	6	13447098	13447113	2	8	16	GT	16	7
66114	6	13455696	13455711	2	8	16	CA	20	4
66126	6	13479388	13479417	2	15	30	GT	14	9
66128	6	13481801	13481812	2	6	12	TG	29	5
66137	6	13489543	13489558	2	8	16	CT	22	8
66138	6	13493662	13493675	2	7	14	TA	19	6
66141	6	13497889	13497936	2	24	48	CA	9	12
66142	6	13497978	13497991	2	7	14	AC	14	4
66143	6	13501721	13501738	2	9	18	GA	8	4
66146	6	13516716	13516737	2	11	22	TG	24	9
66148	6	13519274	13519295	2	11	22	TG	15	8
66150	6	13519873	13519906	2	17	34	GT	17	13
66151	6	13520584	13520615	2	16	32	GT	12	8
66163	6	13535315	13535328	2	7	14	TA	10	6
66165	6	13536527	13536540	2	7	14	GT	31	6
66167	6	13538038	13538061	3	8	24	CCT	24	8
66173	6	13547242	13547253	2	6	12	GT	25	6
66176	6	13561484	13561505	2	11	22	TG	10	10
66179	6	13569847	13569868	2	11	22	TG	17	4
66184	6	13578437	13578462	2	13	26	GT	15	7
66186	6	13579217	13579232	4	4	16	AAAC	13	5
66187	6	13581083	13581094	2	6	12	GA	22	4
66188	6	13581267	13581292	2	13	26	AC	15	7
66189	6	13583546	13583569	2	12	24	CA	18	4
66194	6	13586882	13586897	2	8	16	CA	21	6
66196	6	13587703	13587714	2	6	12	CA	29	3
66200	6	13594384	13594395	2	6	12	CA	25	3
66201	6	13594670	13594685	4	4	16	AAAT	17	4
66203	6	13595590	13595615	2	13	26	GT	14	13
66207	6	13604207	13604220	2	7	14	TG	15	4

66209	6	13610137	13610160	2	12	24	GT	17	19
66211	6	13612363	13612374	2	6	12	AT	12	3
66213	6	13615237	13615254	3	6	18	GTG	15	6
66215	6	13615524	13615541	3	6	18	TTC	19	3
66216	6	13616539	13616556	2	9	18	TG	24	13
66217	6	13619406	13619427	2	11	22	GT	23	7
66218	6	13623620	13623635	2	8	16	AG	23	3
66227	6	13663284	13663299	2	8	16	CA	26	6
66249	6	13729468	13729485	2	9	18	GA	31	5
66250	6	13731428	13731447	2	10	20	GT	14	7
66258	6	13739743	13739758	2	8	16	TG	22	9
66266	6	13750057	13750078	2	11	22	CA	7	8
66267	6	13750243	13750266	2	12	24	AC	9	7
66269	6	13752301	13752318	2	9	18	CA	15	10
66270	6	13752320	13752333	2	7	14	AC	13	9
66280	6	13768087	13768098	2	6	12	TA	7	3
66281	6	13768553	13768570	3	6	18	TTA	14	6
66291	6	13783272	13783285	2	7	14	TC	22	3
66302	6	13795381	13795416	2	18	36	CA	15	10
66315	6	13811736	13811747	2	6	12	GT	23	3
66320	6	13829860	13829899	4	10	40	AGAT	24	11
66322	6	13830414	13830428	3	5	15	ATA	10	3
66323	6	13831513	13831530	3	6	18	CCT	22	3
66329	6	13833787	13833800	2	7	14	AG	17	7
66337	6	13852012	13852035	2	12	24	GA	14	5
66345	6	13864402	13864419	3	6	18	AAT	20	6
66347	6	13868943	13868975	3	11	33	TTC	16	6
66351	6	13874158	13874181	2	12	24	AC	16	10
66352	6	13878070	13878093	2	12	24	TG	8	10
66366	6	13914429	13914443	3	5	15	ACA	30	3
66375	6	13932458	13932481	2	12	24	CA	13	9
66376	6	13932517	13932528	2	6	12	AC	31	6
66379	6	13939623	13939638	2	8	16	TG	14	7
66381	6	13941210	13941241	2	16	32	AC	11	9
66385	6	13946461	13946474	2	7	14	TG	15	6
66386	6	13950028	13950041	2	7	14	AT	25	4
66388	6	13951812	13951823	2	6	12	GT	16	3
66390	6	13961022	13961045	2	12	24	GA	23	11
66396	6	13973392	13973407	4	4	16	TTTA	20	4
66408	6	13985244	13985279	2	18	36	AC	24	15
66412	6	14000977	14000990	2	7	14	AC	23	3
66413	6	14001010	14001021	2	6	12	AC	18	3
66414	6	14001387	14001408	2	11	22	CA	14	10
66416	6	14002402	14002421	2	10	20	AC	20	6
66417	6	14002586	14002597	2	6	12	CT	18	4
66421	6	14010026	14010037	2	6	12	GA	25	7
66422	6	14017777	14017792	4	4	16	TTTA	19	5
66424	6	14018686	14018707	2	11	22	GT	10	7
66427	6	14025863	14025886	2	12	24	CA	16	8
66430	6	14028968	14028979	2	6	12	CA	30	5
66440	6	14046332	14046353	2	11	22	GT	9	8

66443	6	14048444	14048461	2	9	18	AC	21	8
66449	6	14062784	14062809	2	13	26	AC	9	9
66451	6	14063666	14063677	2	6	12	GT	13	3
66454	6	14068944	14068958	3	5	15	TTC	25	3
66460	6	14089660	14089695	2	18	36	TG	14	12
66468	6	14098748	14098795	2	24	48	CA	23	15
66471	6	14106006	14106025	2	10	20	GA	26	4
66472	6	14108276	14108303	2	14	28	CA	21	10
66477	6	14114353	14114368	2	8	16	AC	25	5
66480	6	14118639	14118658	2	10	20	AC	23	10
66481	6	14119276	14119293	2	9	18	AT	24	5
66483	6	14122559	14122573	3	5	15	ATG	21	3
66491	6	14134161	14134188	2	14	28	AC	20	8
66494	6	14136870	14136893	2	12	24	GA	14	3
66504	6	14164720	14164739	2	10	20	CA	15	11
66505	6	14169337	14169348	2	6	12	CA	22	4
66506	6	14170326	14170359	2	17	34	AC	7	3
66522	6	14182438	14182469	2	16	32	AC	12	10
66524	6	14183980	14183997	3	6	18	GCT	28	4
66528	6	14190850	14190861	2	6	12	CA	19	4
66529	6	14197222	14197237	4	4	16	TGGT	15	4
66535	6	14214479	14214492	2	7	14	CT	21	3
66540	6	14218724	14218745	2	11	22	TG	12	6
66542	6	14222296	14222319	2	12	24	TG	19	11
66543	6	14222415	14222446	2	16	32	TG	12	9
66544	6	14223089	14223116	2	14	28	AC	18	6
66554	6	14237358	14237387	2	15	30	GT	23	11
66557	6	14240869	14240888	2	10	20	GT	20	14
66558	6	14241845	14241858	2	7	14	TG	16	11
66559	6	14245389	14245412	3	8	24	TCT	9	5
66564	6	14247604	14247627	3	8	24	CTC	22	4
66577	6	14263062	14263082	3	7	21	CCA	20	4
66578	6	14263314	14263331	2	9	18	GA	19	9
66579	6	14263443	14263462	2	10	20	AG	12	5
66580	6	14263524	14263537	2	7	14	GT	8	5
66583	6	14263903	14263920	2	9	18	TG	12	13
66585	6	14264877	14264894	2	9	18	CA	27	3
66586	6	14265738	14265759	2	11	22	TG	11	7
66589	6	14269148	14269183	2	18	36	TG	15	8
66603	6	14306068	14306083	2	8	16	GT	17	11
66604	6	14306697	14306710	2	7	14	GT	21	3
66607	6	14309626	14309639	2	7	14	CA	25	5
66611	6	14316226	14316285	4	15	60	AGAT	14	10
66618	6	14318562	14318573	2	6	12	GA	14	3
66620	6	14321857	14321876	2	10	20	CA	21	6
66622	6	14326988	14327007	2	10	20	AC	12	12
66624	6	14328173	14328190	3	6	18	TCC	23	10
66630	6	14334092	14334105	2	7	14	AT	24	4
66632	6	14336277	14336288	2	6	12	TG	29	9
66637	6	14352961	14353016	4	14	56	TATC	18	10
66638	6	14353628	14353655	2	14	28	GT	15	8

66639	6	14353723	14353755	3	11	33	CTC	22	10
66641	6	14354318	14354333	4	4	16	AAAT	28	5
66656	6	14374255	14374278	4	6	24	TGTT	20	5
66657	6	14376455	14376466	2	6	12	CA	29	3
66658	6	14380978	14380999	2	11	22	CA	16	10
66662	6	14385309	14385326	3	6	18	GCA	33	5
66664	6	14400426	14400445	4	5	20	AAAT	25	5
66667	6	14403372	14403393	2	11	22	AC	22	6
66668	6	14404862	14404879	3	6	18	GTG	28	5
66673	6	14414047	14414076	3	10	30	AAG	21	8
66677	6	14423203	14423214	2	6	12	TG	26	4
66678	6	14423628	14423641	2	7	14	AC	14	4
66680	6	14425935	14425958	3	8	24	ATG	17	7
66682	6	14427415	14427438	2	12	24	CT	8	4
66688	6	14428443	14428466	3	8	24	GAG	18	4
66689	6	14428759	14428773	3	5	15	TCA	22	4
66694	6	14434529	14434542	2	7	14	CT	14	3
66695	6	14434937	14434950	2	7	14	CA	26	3
66701	6	14445295	14445314	2	10	20	GT	11	4
66703	6	14450030	14450065	3	12	36	ATG	11	6
66704	6	14450778	14450795	2	9	18	TC	16	3
66708	6	14451991	14452004	2	7	14	TG	19	7
66709	6	14452215	14452241	3	9	27	TTA	15	5
66712	6	14458725	14458736	2	6	12	AT	7	5
66713	6	14459205	14459219	3	5	15	ACA	11	3
66715	6	14459782	14459809	2	14	28	CA	13	7
66717	6	14463008	14463033	2	13	26	GT	13	8
66723	6	14471684	14471715	2	16	32	GT	14	10
66724	6	14473194	14473221	2	14	28	TG	16	9
66728	6	14484055	14484068	2	7	14	GA	20	3
66729	6	14484099	14484125	3	9	27	CTG	18	5
66730	6	14484280	14484297	2	9	18	TG	18	7
66732	6	14484850	14484879	3	10	30	AAC	7	5
66733	6	14485033	14485068	2	18	36	TG	17	9
66746	6	14509983	14510003	3	7	21	TTG	32	6
66750	6	14527278	14527291	2	7	14	AG	17	9
66759	6	14541794	14541807	2	7	14	TG	19	7
66760	6	14542234	14542266	3	11	33	CCT	23	9
66761	6	14542872	14542927	2	28	56	GT	9	8
66762	6	14543855	14543870	2	8	16	CT	25	4
66763	6	14545654	14545673	2	10	20	GT	21	14
66774	6	14563570	14563589	2	10	20	TA	7	7
66785	6	14581136	14581153	2	9	18	TG	16	6
66786	6	14581900	14581913	2	7	14	CT	18	3
66787	6	14583069	14583108	2	20	40	CA	18	14
66791	6	14590042	14590062	3	7	21	ACA	23	3
66798	6	14602506	14602521	2	8	16	GT	23	8
66799	6	14605507	14605524	3	6	18	TTA	22	9
66801	6	14616566	14616585	2	10	20	AT	29	9
66802	6	14621399	14621414	2	8	16	TC	23	5
66806	6	14625270	14625305	2	18	36	GT	7	9

66810	6	14631891	14631924	2	17	34	TC	13	9
66811	6	14636067	14636088	2	11	22	TG	10	7
66814	6	14637313	14637326	2	7	14	TC	19	4
66815	6	14637564	14637578	3	5	15	GAA	19	5
66816	6	14637579	14637596	3	6	18	GGA	25	7
66818	6	14639310	14639330	3	7	21	GCA	28	8
66824	6	14650144	14650163	4	5	20	ATTT	21	7
66825	6	14655510	14655523	2	7	14	AT	24	4
66826	6	14661731	14661748	3	6	18	GAG	30	3
66828	6	14662698	14662712	3	5	15	CAT	24	6
66831	6	14667963	14667978	2	8	16	AT	17	11
66834	6	14674042	14674053	2	6	12	TA	8	6
66837	6	14681788	14681807	2	10	20	CA	19	8
66842	6	14686939	14686964	2	13	26	AC	15	7
66843	6	14687145	14687165	3	7	21	AGC	26	5
66844	6	14689405	14689420	2	8	16	GA	30	8
66846	6	14690695	14690722	2	14	28	GT	9	7
66848	6	14695788	14695803	4	4	16	GGAT	10	6
66857	6	14700742	14700771	2	15	30	AC	23	11
66858	6	14700947	14700961	3	5	15	CTT	31	3
66859	6	14701469	14701482	2	7	14	AT	15	7
66863	6	14714726	14714740	3	5	15	CCT	25	3
66871	6	14730933	14730958	2	13	26	CA	19	6
66872	6	14731798	14731811	2	7	14	AC	27	5
66874	6	14738140	14738153	2	7	14	AT	11	4
66877	6	14744096	14744115	2	10	20	CA	26	8
66880	6	14749154	14749171	2	9	18	AC	20	9
66885	6	14753308	14753325	2	9	18	GA	18	6
66886	6	14753718	14753753	2	18	36	CT	8	10
66888	6	14754205	14754228	3	8	24	ATA	15	5
66890	6	14758128	14758151	4	6	24	AAGA	21	4
66892	6	14760577	14760592	2	8	16	TG	12	10
66902	6	14767135	14767152	2	9	18	TG	14	6
66903	6	14767614	14767634	3	7	21	AAG	17	4
66906	6	14769122	14769153	2	16	32	CA	7	8
66911	6	14776713	14776730	2	9	18	CT	15	6
66913	6	14777120	14777149	3	10	30	GCA	22	7
66920	6	14784624	14784638	3	5	15	CTT	28	5
66924	6	14790307	14790321	3	5	15	AAG	24	5
66928	6	14791508	14791527	2	10	20	AC	23	8
66939	6	14806602	14806629	2	14	28	AC	9	8
66946	6	14812161	14812178	3	6	18	CTG	25	4
66949	6	14818491	14818512	2	11	22	CA	6	7
66951	6	14819115	14819135	3	7	21	GAG	19	6
66956	6	14820264	14820296	3	11	33	TAT	11	5
66957	6	14832177	14832191	3	5	15	AAC	29	6
66959	6	14846797	14846810	2	7	14	AT	21	9
66961	6	14852568	14852613	2	23	46	CA	8	11
66967	6	14883634	14883648	3	5	15	AAG	20	4
66969	6	14885347	14885360	2	7	14	TC	22	4
66973	6	14887631	14887646	2	8	16	AG	18	5

66974	6	14887849	14887872	2	12	24	GT	17	10
66976	6	14892486	14892505	2	10	20	AC	11	4
66984	6	14907328	14907342	3	5	15	GAG	17	7
66988	6	14921569	14921594	2	13	26	GT	19	8
66990	6	14924259	14924282	2	12	24	CA	19	12
66991	6	14924383	14924396	2	7	14	TG	15	5
66992	6	14924504	14924523	2	10	20	CA	10	8
66993	6	14924652	14924667	2	8	16	CA	14	11
66995	6	14926746	14926767	2	11	22	CA	13	3
66996	6	14929477	14929490	2	7	14	CA	11	3
66998	6	14935047	14935061	3	5	15	TGT	27	3
67004	6	14941339	14941360	2	11	22	CA	20	7
67005	6	14941806	14941835	3	10	30	GGA	18	4
67011	6	14943994	14944013	2	10	20	GA	7	8
67014	6	14944404	14944417	2	7	14	TC	9	7
67015	6	14944458	14944481	2	12	24	TG	23	5
67017	6	14954453	14954466	2	7	14	GA	22	3
67018	6	14954493	14954516	2	12	24	GA	17	9
67019	6	14954795	14954809	3	5	15	GCG	18	10
67021	6	14956674	14956687	2	7	14	TC	26	4
67022	6	14958920	14958937	2	9	18	CA	19	6
67023	6	14960195	14960254	3	20	60	AGA	17	13
67025	6	14983392	14983407	4	4	16	ATGA	19	8
67026	6	14983410	14983429	4	5	20	GAAT	25	13
67029	6	14987003	14987030	2	14	28	CA	22	20
67032	6	15013112	15013137	2	13	26	GT	19	12
67033	6	15013291	15013304	2	7	14	TG	21	11
67035	6	15014893	15014904	2	6	12	AG	25	3
67036	6	15016369	15016398	2	15	30	TG	19	7
67037	6	15017843	15017858	2	8	16	GT	24	7
67039	6	15018512	15018537	2	13	26	TG	17	4
67040	6	15018823	15018852	2	15	30	GT	16	12
67041	6	15019021	15019052	2	16	32	TG	12	6
67045	6	15021003	15021022	2	10	20	CA	21	12
67048	6	15021553	15021582	2	15	30	CA	14	14
67049	6	15022015	15022042	2	14	28	AC	8	10
67051	6	15023610	15023631	2	11	22	AC	19	7
67052	6	15023826	15023837	2	6	12	AC	21	6
67053	6	15023840	15023855	2	8	16	AC	15	3
67054	6	15026969	15027002	2	17	34	GT	17	11
67056	6	15027852	15027863	2	6	12	GT	28	4
67062	6	15033207	15033234	4	7	28	CAGA	11	9
67063	6	15033590	15033615	2	13	26	AC	9	5
67064	6	15033777	15033796	2	10	20	AG	9	4
67070	6	15040094	15040108	3	5	15	AGC	26	7
67073	6	15043127	15043138	2	6	12	AC	29	3
67074	6	15043345	15043362	2	9	18	AG	19	5
67076	6	15047921	15047935	3	5	15	GAG	26	6
67077	6	15048664	15048689	2	13	26	CA	21	9
67080	6	15049067	15049090	2	12	24	AC	13	6
67083	6	15051224	15051245	2	11	22	AC	8	16

67084	6	15051385	15051406	2	11	22	TG	15	11
67086	6	15053003	15053018	2	8	16	TG	11	7
67087	6	15053137	15053192	2	28	56	TG	6	7
67088	6	15053357	15053382	2	13	26	CA	8	6
67091	6	15054272	15054289	2	9	18	CA	9	11
67092	6	15054855	15054881	3	9	27	GAG	17	6
67094	6	15054972	15055011	2	20	40	TG	10	6
67095	6	15055212	15055235	2	12	24	AC	17	13
67102	6	15060914	15060947	2	17	34	GT	13	7
67103	6	15061227	15061238	2	6	12	GT	24	4
67104	6	15061242	15061255	2	7	14	TG	26	4
67106	6	15061593	15061614	2	11	22	AC	11	9
67107	6	15062680	15062697	3	6	18	AGA	23	3
67108	6	15062931	15062958	2	14	28	GT	9	6
67110	6	15063106	15063135	3	10	30	GAA	12	6
67115	6	15063470	15063511	2	21	42	AC	13	14
67116	6	15063667	15063694	2	14	28	CA	13	12
67117	6	15064710	15064755	2	23	46	TG	6	8
67119	6	15065725	15065758	2	17	34	AC	10	11
67122	6	15066866	15066895	2	15	30	TG	13	6
67125	6	15069272	15069299	2	14	28	TG	18	9
67126	6	15070495	15070524	2	15	30	CA	8	6
67128	6	15073150	15073177	2	14	28	AC	16	10
67130	6	15076011	15076044	2	17	34	TG	18	6
67131	6	15077627	15077640	2	7	14	TC	18	4
67132	6	15077986	15078003	2	9	18	AC	23	7
67133	6	15078298	15078317	2	10	20	AC	20	14
67134	6	15080626	15080641	2	8	16	AC	21	3
67135	6	15082940	15082957	3	6	18	GGA	26	10
67137	6	15087956	15087970	3	5	15	CCT	15	3
67139	6	15089876	15089899	4	6	24	AAAT	22	4
67140	6	15091225	15091242	2	9	18	GT	22	8
67142	6	15091426	15091449	4	6	24	GGAG	12	4
67143	6	15091756	15091779	2	12	24	GT	22	7
67144	6	15093858	15093878	3	7	21	CTG	19	6
67145	6	15094788	15094814	3	9	27	CCT	16	8
67147	6	15096314	15096329	4	4	16	ATTT	28	7
67148	6	15096379	15096396	2	9	18	GT	21	25
67152	6	15100596	15100613	2	9	18	CT	18	14
67153	6	15100614	15100629	2	8	16	CA	19	12
67155	6	15109489	15109506	2	9	18	AC	27	11
67168	6	15199432	15199451	2	10	20	AG	20	5
67170	6	15202746	15202785	2	20	40	GT	12	8
67172	6	15215849	15215866	3	6	18	CCT	25	4
67176	6	15221095	15221120	2	13	26	GT	16	14
67177	6	15221336	15221371	3	12	36	TCT	11	8
67180	6	15223765	15223806	2	21	42	GT	13	8
67183	6	15230093	15230116	2	12	24	GT	17	10
67189	6	15248024	15248053	2	15	30	TG	13	9
67190	6	15248099	15248116	2	9	18	TG	19	13
67191	6	15248277	15248302	2	13	26	TG	18	5

67197	6	15256327	15256342	4	4	16	TAAA	16	4
67201	6	15259112	15259129	2	9	18	GT	7	9
67206	6	15266723	15266740	3	6	18	GAG	29	5
67208	6	15270880	15270894	3	5	15	GAG	22	6
67211	6	15272646	15272665	4	5	20	AAAG	33	10
67212	6	15273126	15273155	2	15	30	TG	7	7
67213	6	15277025	15277068	2	22	44	TG	9	13
67218	6	15280768	15280787	2	10	20	GA	11	6
67221	6	15282888	15282911	2	12	24	CA	24	15
67223	6	15287400	15287429	2	15	30	TG	12	7
67226	6	15293249	15293276	2	14	28	TG	12	9
67231	6	15294734	15294757	2	12	24	GT	8	6
67233	6	15299100	15299121	2	11	22	AC	18	6
67234	6	15299327	15299348	2	11	22	CA	26	7
67236	6	15299886	15299925	2	20	40	GT	8	7
67242	6	15305714	15305731	2	9	18	CT	26	4
67244	6	15306435	15306446	2	6	12	AC	13	4
67253	6	15312657	15312690	2	17	34	AC	8	6
67256	6	15314630	15314645	2	8	16	AG	28	3
67263	6	15318603	15318630	4	7	28	ATAA	22	16
67268	6	15321186	15321211	2	13	26	CA	6	9
67270	6	15321435	15321452	2	9	18	CA	13	5
67272	6	15322135	15322160	2	13	26	CA	19	12
67275	6	15329069	15329080	2	6	12	AC	20	3
67278	6	15331910	15331925	2	8	16	CT	24	4
67281	6	15336080	15336103	3	8	24	AGG	23	5
67282	6	15337642	15337683	2	21	42	GT	7	6
67283	6	15342409	15342424	4	4	16	ATAG	32	3
67284	6	15344954	15344977	4	6	24	ATCA	23	4
67286	6	15345687	15345707	3	7	21	ATA	10	5
67288	6	15349763	15349802	2	20	40	AC	7	13
67289	6	15351220	15351251	2	16	32	AC	17	7
67291	6	15353398	15353413	2	8	16	AC	20	6
67295	6	15362718	15362749	2	16	32	TG	20	10
67298	6	15364762	15364787	2	13	26	TG	18	6
67299	6	15364991	15365006	2	8	16	TA	16	7
67302	6	15369594	15369625	2	16	32	GT	7	10
67304	6	15370202	15370219	2	9	18	AC	16	12
67305	6	15375197	15375212	2	8	16	AC	13	6
67306	6	15375623	15375654	2	16	32	TG	14	14
67307	6	15377683	15377696	2	7	14	GT	17	6
67308	6	15377723	15377736	2	7	14	AC	21	5
67309	6	15380074	15380091	3	6	18	ATA	14	4
67310	6	15381045	15381086	2	21	42	AC	9	7
67311	6	15381306	15381321	2	8	16	CA	22	12
67312	6	15381443	15381472	2	15	30	AC	19	12
67316	6	15386407	15386448	2	21	42	TG	7	12
67317	6	15386625	15386642	3	6	18	CTC	22	3
67318	6	15386957	15386986	2	15	30	TG	12	10
67319	6	15387207	15387230	3	8	24	TCC	28	16
67321	6	15387595	15387624	2	15	30	CA	6	7

67328	6	15387979	15387993	3	5	15	CAT	9	5
67339	6	15398780	15398791	2	6	12	CA	23	3
67341	6	15400484	15400503	2	10	20	AG	21	3
67344	6	15409314	15409325	2	6	12	TC	34	4
67345	6	15409833	15409856	2	12	24	AC	12	8
67351	6	15418330	15418353	3	8	24	TGT	24	8
67357	6	15427892	15427933	2	21	42	AG	11	11
67358	6	15429568	15429581	2	7	14	TG	13	3
67363	6	15438444	15438473	2	15	30	GT	16	5
67367	6	15439439	15439464	2	13	26	CA	20	7
67368	6	15439863	15439884	2	11	22	GT	20	7
67375	6	15443497	15443510	2	7	14	AC	28	5
67377	6	15451404	15451419	2	8	16	TA	12	3
67378	6	15456056	15456067	2	6	12	GT	27	3
67382	6	15460327	15460356	2	15	30	AC	17	11
67383	6	15461759	15461782	2	12	24	GT	18	8
67386	6	15465191	15465216	2	13	26	TG	19	11
67388	6	15471084	15471115	2	16	32	CA	10	7
67389	6	15473299	15473332	2	17	34	CA	10	8
67391	6	15473877	15473894	2	9	18	CA	26	14
67394	6	15479555	15479576	2	11	22	AC	16	15
67399	6	15483518	15483531	2	7	14	GT	6	5
67400	6	15486707	15486718	2	6	12	TA	12	3
67405	6	15513580	15513611	2	16	32	CA	20	8
67408	6	15524309	15524324	4	4	16	GTCA	26	4
67410	6	15527634	15527671	2	19	38	AC	9	9
67412	6	15529602	15529625	2	12	24	CA	27	8
67414	6	15529952	15529977	2	13	26	CA	16	11
67416	6	15531175	15531202	2	14	28	CA	16	11
67417	6	15536981	15536992	2	6	12	CA	23	6
67420	6	15539144	15539159	4	4	16	ATAC	11	3
67421	6	15539853	15539890	2	19	38	GT	18	18
67426	6	15546949	15546980	2	16	32	AC	16	12
67428	6	15548800	15548814	3	5	15	CTG	28	8
67433	6	15559026	15559041	2	8	16	TC	26	8
67434	6	15560129	15560156	4	7	28	TGTC	31	6
67441	6	15590661	15590680	2	10	20	TG	11	11
67442	6	15590750	15590767	2	9	18	GT	19	8
67443	6	15590840	15590861	2	11	22	AC	16	8
67445	6	15592801	15592812	2	6	12	CA	16	5
67446	6	15592815	15592826	2	6	12	CA	12	5
67447	6	15593464	15593475	2	6	12	TA	31	3
67448	6	15594130	15594153	2	12	24	GT	14	16
67449	6	15594681	15594700	2	10	20	CA	21	7
67450	6	15599313	15599330	3	6	18	CCT	21	4
67453	6	15613702	15613721	2	10	20	TG	15	7
67455	6	15622838	15622851	2	7	14	AT	16	3
67457	6	15628422	15628439	2	9	18	CA	22	15
67470	6	15639006	15639035	2	15	30	TG	11	9
67471	6	15641769	15641780	2	6	12	GT	25	3
67472	6	15644184	15644199	2	8	16	CT	25	6

67473	6	15644381	15644410	2	15	30	TG	15	10
67477	6	15646260	15646307	2	24	48	AC	6	7
67479	6	15647324	15647339	2	8	16	GA	12	7
67481	6	15648053	15648080	2	14	28	AC	19	9
67483	6	15648984	15648999	2	8	16	CA	26	14
67484	6	15649465	15649491	3	9	27	GAG	21	7
67486	6	15650083	15650109	3	9	27	GAG	9	12
67487	6	15650174	15650203	2	15	30	GT	13	9
67489	6	15650554	15650577	3	8	24	CTC	28	13
67490	6	15653018	15653038	3	7	21	TGA	25	8
67493	6	15653718	15653733	4	4	16	CTGA	31	4
67495	6	15656818	15656829	2	6	12	AT	23	7
67498	6	15658036	15658057	2	11	22	CA	13	8
67499	6	15658578	15658595	2	9	18	CA	8	3
67500	6	15658668	15658691	2	12	24	CA	9	7
67501	6	15658888	15658907	2	10	20	AC	10	7
67503	6	15663986	15664011	2	13	26	CA	16	9
67504	6	15664213	15664260	2	24	48	GT	9	14
67505	6	15664851	15664868	3	6	18	TTG	28	3
67506	6	15667612	15667639	2	14	28	CA	21	10
67507	6	15668027	15668048	2	11	22	AC	7	9
67511	6	15674463	15674476	2	7	14	AC	12	4
67521	6	15699757	15699772	2	8	16	TC	17	4
67523	6	15701868	15701885	2	9	18	TA	12	8
67530	6	15727671	15727700	2	15	30	CA	17	10
67532	6	15736253	15736268	2	8	16	AC	29	3
67535	6	15742896	15742915	2	10	20	TG	10	3
67538	6	15745161	15745188	2	14	28	CA	11	13
67564	6	15809654	15809674	3	7	21	GAA	23	7
67567	6	15821784	15821799	4	4	16	AAAT	18	12
67568	6	15827767	15827786	2	10	20	CT	17	11
67569	6	15827877	15827900	2	12	24	AC	14	10
67570	6	15830352	15830367	2	8	16	AC	23	4
67572	6	15833927	15833954	2	14	28	AC	11	8
67579	6	15867708	15867733	2	13	26	GA	12	6
67580	6	15878065	15878080	4	4	16	CTAG	23	3
67586	6	15894498	15894513	4	4	16	TTAT	9	3
67587	6	15894985	15895004	2	10	20	GT	24	3
67588	6	15895648	15895669	2	11	22	CA	19	10
67596	6	15936256	15936270	3	5	15	TTG	19	3
67605	6	15968313	15968336	3	8	24	CTC	18	3
67607	6	15970203	15970232	2	15	30	TG	18	7
67615	6	15989508	15989547	2	20	40	CA	6	7
67629	6	16004601	16004622	2	11	22	AC	22	5
67634	6	16010282	16010311	3	10	30	TTC	21	7
67635	6	16014393	16014408	2	8	16	TG	23	4
67636	6	16024335	16024348	2	7	14	CT	27	3
67647	6	16055065	16055098	2	17	34	TC	12	9
67652	6	16063724	16063735	2	6	12	CA	26	3
67674	6	16080643	16080656	2	7	14	GT	22	5
67675	6	16080658	16080673	2	8	16	TG	21	5

67684	6	16110221	16110240	2	10	20	TG	16	5
67690	6	16120553	16120570	2	9	18	TG	23	8
67693	6	16123833	16123860	2	14	28	TG	16	3
67695	6	16126429	16126454	2	13	26	AC	14	9
67697	6	16132046	16132077	2	16	32	AC	9	6
67704	6	16169391	16169402	2	6	12	CA	20	4
67710	6	16181565	16181604	2	20	40	GT	16	10
67711	6	16181801	16181812	2	6	12	GC	23	7
67712	6	16181813	16181828	2	8	16	GT	24	10
67715	6	16184183	16184200	2	9	18	AC	17	6
67721	6	16186961	16186996	2	18	36	CA	11	7
67723	6	16189342	16189357	2	8	16	GT	23	3
67731	6	16213973	16213987	3	5	15	GCA	27	3
67735	6	16220131	16220158	2	14	28	GT	13	6
67740	7	15639	15665	3	9	27	TAA	13	5
67743	7	24084	24105	2	11	22	GA	13	6
67747	7	38744	38775	2	16	32	CA	11	8
67756	7	69880	69894	3	5	15	ACA	26	4
67759	7	76972	76993	2	11	22	CA	7	5
67764	7	93370	93399	3	10	30	TCC	22	8
67776	7	116405	116440	2	18	36	AC	7	8
67778	7	118156	118169	2	7	14	TG	10	5
67779	7	118172	118187	2	8	16	TG	10	5
67781	7	121517	121534	3	6	18	TTG	22	7
67785	7	136980	137005	2	13	26	TG	11	7
67786	7	139914	139941	2	14	28	TG	18	9
67794	7	164955	164972	2	9	18	CA	21	7
67809	7	217832	217857	2	13	26	CA	11	10
67811	7	222328	222353	2	13	26	GT	10	10
67812	7	223935	223961	3	9	27	TCC	18	4
67813	7	224046	224060	3	5	15	TCC	17	3
67814	7	229240	229255	2	8	16	AC	25	7
67823	7	250027	250054	2	14	28	CA	19	18
67826	7	259620	259659	2	20	40	CA	16	14
67827	7	261487	261500	2	7	14	AT	20	4
67831	7	279583	279626	4	11	44	TATC	8	9
67848	7	311049	311064	4	4	16	CAAA	27	4
67852	7	322383	322406	3	8	24	TGG	19	9
67854	7	327441	327455	3	5	15	ATT	21	11
67856	7	337912	337939	4	7	28	TCTT	18	7
67857	7	338693	338704	2	6	12	TG	22	4
67858	7	339656	339669	2	7	14	TG	22	10
67861	7	362550	362569	2	10	20	AC	23	14
67862	7	364597	364623	3	9	27	CTG	32	6
67867	7	371949	371968	2	10	20	AG	19	4
67868	7	372089	372106	2	9	18	AC	17	8
67871	7	374557	374586	3	10	30	CCT	9	6
67873	7	375783	375797	3	5	15	TGC	34	4
67874	7	375999	376020	2	11	22	TG	17	16
67875	7	376126	376137	2	6	12	TG	28	4
67876	7	377995	378010	4	4	16	TATT	16	8

67877	7	380901	380920	2	10	20	TG	18	7
67880	7	384014	384049	4	9	36	TTCT	14	11
67884	7	390895	390910	4	4	16	TAGC	24	3
67890	7	402277	402296	4	5	20	ACTG	17	3
67895	7	407147	407178	2	16	32	CA	6	9
67903	7	430404	430421	2	9	18	TG	25	17
67904	7	433393	433407	3	5	15	ATA	19	3
67909	7	449721	449744	2	12	24	TG	9	12
67911	7	451298	451327	3	10	30	GAT	19	4
67913	7	452942	452955	2	7	14	TG	22	9
67916	7	466462	466479	2	9	18	TG	17	9
67919	7	476999	477019	3	7	21	AAT	21	5
67921	7	481870	481901	2	16	32	TG	15	10
67928	7	500222	500233	2	6	12	CA	18	3
67932	7	510901	510932	2	16	32	CA	7	6
67936	7	524329	524350	2	11	22	AG	14	12
67937	7	527889	527918	2	15	30	GT	10	10
67941	7	540667	540680	2	7	14	GA	16	4
67942	7	541693	541708	4	4	16	TCTG	19	3
67943	7	544818	544841	2	12	24	CA	10	5
67946	7	556457	556472	2	8	16	CA	27	6
67947	7	557909	557948	2	20	40	TG	11	13
67965	7	613905	613916	2	6	12	CA	30	5
67972	7	628336	628367	2	16	32	AC	8	10
67974	7	639053	639068	4	4	16	TTAT	22	4
67982	7	657262	657285	3	8	24	CTG	21	10
67986	7	666048	666063	2	8	16	AC	20	7
67987	7	666540	666551	2	6	12	GT	24	7
67989	7	674690	674703	2	7	14	GT	13	7
67990	7	674709	674720	2	6	12	TA	15	7
67996	7	702928	702941	2	7	14	AG	21	4
68000	7	708826	708839	2	7	14	CA	22	3
68003	7	711226	711263	2	19	38	AC	6	10
68004	7	714648	714661	2	7	14	CA	19	4
68018	7	732011	732026	2	8	16	AC	12	5
68019	7	732140	732169	2	15	30	GT	13	12
68020	7	738529	738542	2	7	14	TG	17	4
68022	7	743759	743776	3	6	18	ATA	20	5
68024	7	745814	745841	2	14	28	CA	14	14
68027	7	750765	750792	2	14	28	AC	16	11
68030	7	752424	752451	2	14	28	GT	16	7
68031	7	752635	752654	2	10	20	GA	22	6
68032	7	752850	752869	2	10	20	AC	17	7
68034	7	753010	753027	2	9	18	TG	20	10
68035	7	753186	753203	2	9	18	CA	24	7
68037	7	757621	757640	4	5	20	GTTG	24	3
68041	7	760210	760227	2	9	18	TG	23	8
68045	7	766644	766683	2	20	40	GT	6	9
68046	7	767508	767527	2	10	20	GT	24	7
68057	7	778838	778861	2	12	24	CA	15	14
68063	7	785070	785129	2	30	60	TG	7	8

68066	7	785322	785335	2	7	14	TG	9	5
68067	7	785431	785456	2	13	26	CA	13	6
68070	7	793479	793512	2	17	34	CA	9	8
68071	7	793697	793724	2	14	28	TG	16	7
68077	7	800787	800820	2	17	34	CA	7	7
68078	7	800978	801001	2	12	24	GT	28	9
68093	7	827916	827933	2	9	18	AC	12	6
68099	7	830292	830323	2	16	32	AC	7	6
68105	7	848672	848687	2	8	16	TG	7	4
68106	7	848688	848699	2	6	12	AG	16	6
68112	7	856800	856815	2	8	16	CA	15	10
68120	7	875364	875381	2	9	18	AC	25	10
68128	7	900155	900188	2	17	34	CA	8	9
68129	7	901799	901830	2	16	32	CA	17	12
68130	7	902883	902897	3	5	15	TCC	32	5
68132	7	906404	906417	2	7	14	TC	31	3
68136	7	907329	907344	4	4	16	GGAC	24	3
68138	7	913611	913630	2	10	20	AC	17	11
68139	7	913795	913820	2	13	26	TG	19	11
68140	7	923841	923856	2	8	16	AC	25	7
68141	7	924347	924376	2	15	30	GT	11	11
68142	7	924573	924604	2	16	32	AC	17	8
68153	7	932711	932732	2	11	22	GT	10	11
68160	7	943523	943552	2	15	30	GT	13	7
68162	7	952495	952518	2	12	24	GT	7	8
68163	7	963425	963446	2	11	22	TG	15	11
68164	7	963597	963632	2	18	36	GT	9	8
68167	7	964784	964817	2	17	34	GT	6	6
68171	7	972130	972151	2	11	22	AC	18	5
68172	7	974790	974807	2	9	18	GT	22	11
68177	7	981647	981674	2	14	28	AC	12	6
68180	7	985629	985644	4	4	16	TTAT	24	3
68186	7	1000997	1001012	4	4	16	GATC	34	4
68187	7	1001388	1001443	2	28	56	TG	10	12
68196	7	1013254	1013268	3	5	15	CTC	25	4
68198	7	1013839	1013850	2	6	12	AC	15	6
68202	7	1023705	1023720	2	8	16	AC	30	4
68208	7	1036110	1036137	4	7	28	GATG	19	11
68209	7	1038544	1038569	2	13	26	CA	6	8
68216	7	1048866	1048897	2	16	32	AC	6	7
68217	7	1052628	1052639	2	6	12	CT	12	6
68221	7	1060545	1060566	2	11	22	TG	23	9
68222	7	1065818	1065833	2	8	16	CA	7	10
68228	7	1095305	1095328	4	6	24	TGAG	24	5
68233	7	1108216	1108227	2	6	12	GT	29	3
68236	7	1114294	1114323	3	10	30	CAT	23	10
68242	7	1131079	1131102	3	8	24	AAT	15	10
68243	7	1131302	1131319	3	6	18	AGC	21	5
68244	7	1131678	1131689	2	6	12	CA	33	4
68250	7	1140717	1140728	2	6	12	GA	21	7
68252	7	1142792	1142815	4	6	24	AGAC	25	10

68255	7	1146282	1146302	3	7	21	AAT	15	4
68256	7	1146756	1146767	2	6	12	GT	25	4
68257	7	1148883	1148900	2	9	18	GT	12	9
68261	7	1155705	1155716	2	6	12	AT	15	3
68262	7	1160260	1160285	2	13	26	TG	27	6
68268	7	1169640	1169665	2	13	26	CA	18	8
68274	7	1182479	1182514	2	18	36	AC	16	10
68278	7	1193444	1193461	2	9	18	GT	21	10
68279	7	1196507	1196522	2	8	16	TA	12	9
68289	7	1214594	1214625	2	16	32	GT	7	8
68290	7	1217290	1217313	4	6	24	TCTA	26	9
68335	7	1342371	1342400	2	15	30	GT	25	12
68339	7	1346924	1346941	3	6	18	AGC	33	6
68341	7	1366836	1366847	2	6	12	CA	10	4
68344	7	1382283	1382297	3	5	15	GTA	17	4
68345	7	1401077	1401090	2	7	14	TG	10	8
68355	7	1421235	1421273	3	13	39	ATG	18	8
68359	7	1429489	1429510	2	11	22	GT	13	8
68360	7	1431200	1431219	2	10	20	GT	12	10
68362	7	1431604	1431641	2	19	38	GT	17	12
68367	7	1447542	1447557	4	4	16	ACAG	18	5
68371	7	1447815	1447842	2	14	28	TG	12	10
68373	7	1458292	1458312	3	7	21	GAG	21	4
68376	7	1461101	1461115	3	5	15	AGG	27	6
68381	7	1464506	1464525	2	10	20	TC	7	3
68383	7	1466402	1466443	2	21	42	GT	9	11
68385	7	1469108	1469125	2	9	18	AC	23	6
68386	7	1471152	1471165	2	7	14	GT	29	3
68391	7	1478172	1478187	4	4	16	ATTC	29	3
68392	7	1479901	1479918	3	6	18	TAC	24	4
68395	7	1486697	1486708	2	6	12	TG	26	3
68401	7	1514404	1514421	3	6	18	GCA	32	7
68402	7	1516094	1516107	2	7	14	TG	19	5
68404	7	1517362	1517375	2	7	14	AT	15	16
68405	7	1517804	1517821	2	9	18	TG	20	6
68413	7	1538240	1538255	4	4	16	AGAT	18	5
68416	7	1547286	1547305	4	5	20	ATGG	25	4
68417	7	1549056	1549070	3	5	15	AAC	15	6
68431	7	1638567	1638578	2	6	12	TG	27	5
68435	7	1648309	1648336	4	7	28	AATA	6	4
68436	7	1655564	1655599	4	9	36	TTTC	23	13
68439	7	1662411	1662426	4	4	16	TTCT	17	4
68440	7	1664710	1664729	2	10	20	GT	19	11
68441	7	1664933	1664954	2	11	22	TG	13	5
68451	7	1694144	1694171	2	14	28	AC	11	11
68457	7	1700363	1700386	4	6	24	GATA	12	10
68459	7	1703896	1703921	2	13	26	GT	6	7
68473	7	1733109	1733140	2	16	32	CA	10	11
68474	7	1734198	1734229	4	8	32	AATC	11	5
68480	7	1743891	1743911	3	7	21	ATC	20	5
68483	7	1745448	1745463	2	8	16	GA	17	6

68484	7	1745464	1745483	2	10	20	GT	21	6
68488	7	1751131	1751148	2	9	18	TG	16	9
68489	7	1751232	1751269	2	19	38	CA	10	9
68493	7	1756527	1756556	2	15	30	AC	6	6
68496	7	1759039	1759064	2	13	26	CA	8	7
68497	7	1761034	1761055	2	11	22	GT	11	11
68505	7	1764233	1764266	2	17	34	AC	10	8
68506	7	1764460	1764479	2	10	20	AC	6	7
68509	7	1783947	1783978	2	16	32	GT	8	10
68510	7	1784020	1784037	2	9	18	AG	19	3
68518	7	1798537	1798552	2	8	16	AC	13	7
68519	7	1798884	1798903	2	10	20	CA	14	8
68520	7	1799910	1799921	2	6	12	CA	23	6
68521	7	1801180	1801203	2	12	24	TC	8	3
68528	7	1809072	1809085	2	7	14	CA	21	4
68530	7	1811657	1811676	4	5	20	CACG	12	7
68531	7	1811677	1811694	2	9	18	CA	11	4
68534	7	1817571	1817584	2	7	14	TA	13	9
68535	7	1817585	1817600	2	8	16	TG	13	8
68537	7	1819070	1819087	2	9	18	CA	14	6
68538	7	1820171	1820190	2	10	20	GT	14	12
68539	7	1825222	1825236	3	5	15	AGT	31	3
68541	7	1828992	1829009	2	9	18	TG	16	8
68549	7	1847310	1847329	4	5	20	TGAG	17	4
68555	7	1854879	1854890	2	6	12	AC	25	5
68556	7	1854994	1855009	2	8	16	GT	24	5
68557	7	1855912	1855929	2	9	18	GT	11	5
68558	7	1857112	1857125	2	7	14	AC	26	3
68559	7	1864256	1864282	3	9	27	GGA	17	3
68563	7	1869493	1869510	2	9	18	GA	23	7
68564	7	1870144	1870207	2	32	64	AC	15	12
68577	7	1896179	1896192	2	7	14	GT	15	4
68578	7	1896839	1896850	2	6	12	GA	18	3
68579	7	1897744	1897775	2	16	32	GT	7	9
68589	7	1916872	1916885	2	7	14	TG	9	6
68591	7	1917099	1917128	2	15	30	AG	22	13
68592	7	1917271	1917282	2	6	12	TG	20	4
68597	7	1918773	1918786	2	7	14	TG	21	6
68599	7	1921296	1921325	2	15	30	GA	8	7
68600	7	1923612	1923645	2	17	34	AG	7	7
68601	7	1923949	1923960	2	6	12	GT	31	4
68603	7	1927556	1927607	4	13	52	TTGG	21	9
68616	7	1942017	1942036	4	5	20	ACAT	30	5
68617	7	1947586	1947613	2	14	28	TG	6	10
68629	7	1964305	1964324	4	5	20	CTGG	30	6
68630	7	1964531	1964548	3	6	18	CTG	20	3
68633	7	1965721	1965738	2	9	18	GT	14	6
68635	7	1965796	1965815	2	10	20	AG	14	6
68637	7	1965915	1965944	2	15	30	GA	17	12
68649	7	1992194	1992213	2	10	20	CT	10	6
68655	7	2001638	2001651	2	7	14	GT	20	6

68656	7	2001653	2001664	2	6	12	TG	18	7
68659	7	2003061	2003088	4	7	28	ACAT	19	9
68679	7	2022697	2022722	2	13	26	AC	20	11
68682	7	2034595	2034610	2	8	16	TG	19	10
68687	7	2062775	2062788	2	7	14	GT	12	10
68689	7	2069274	2069288	3	5	15	GCA	25	4
68690	7	2073827	2073854	2	14	28	AC	15	15
68692	7	2076151	2076180	2	15	30	TG	11	9
68694	7	2080858	2080885	2	14	28	GT	16	6
68696	7	2083310	2083335	2	13	26	TG	16	14
68697	7	2087770	2087793	2	12	24	AG	15	9
68701	7	2093154	2093181	2	14	28	AC	14	8
68704	7	2097104	2097127	2	12	24	GT	19	5
68707	7	2100298	2100325	2	14	28	AC	9	8
68714	7	2109539	2109558	2	10	20	AC	11	9
68715	7	2109788	2109801	2	7	14	GT	20	5
68716	7	2111286	2111331	2	23	46	AC	7	9
68717	7	2112479	2112498	2	10	20	CA	20	6
68718	7	2114245	2114262	2	9	18	CA	7	3
68719	7	2114492	2114527	2	18	36	TG	16	13
68728	7	2134845	2134880	2	18	36	TG	11	10
68732	7	2139296	2139311	2	8	16	AC	26	8
68733	7	2143062	2143089	2	14	28	TG	8	12
68763	7	2175944	2175967	2	12	24	CA	12	8
68769	7	2184020	2184041	2	11	22	AC	17	7
68778	7	2190999	2191024	2	13	26	TG	21	17
68779	7	2191825	2191844	4	5	20	AACC	23	5
68782	7	2193615	2193632	2	9	18	GA	23	6
68785	7	2194145	2194172	2	14	28	AC	18	9
68786	7	2194319	2194346	4	7	28	AACA	27	5
68790	7	2200467	2200492	2	13	26	TG	11	11
68792	7	2203226	2203239	2	7	14	AC	15	4
68799	7	2214321	2214338	2	9	18	TG	17	4
68803	7	2221161	2221178	2	9	18	AC	20	8
68814	7	2233796	2233839	2	22	44	AC	14	11
68815	7	2235700	2235743	2	22	44	GT	10	9
68818	7	2237066	2237087	2	11	22	TG	18	8
68820	7	2237657	2237684	2	14	28	CA	19	12
68821	7	2240139	2240166	4	7	28	AAAG	9	6
68824	7	2250416	2250448	3	11	33	ATT	14	8
68830	7	2261068	2261087	2	10	20	CA	27	10
68832	7	2261384	2261399	2	8	16	CA	19	7
68833	7	2261632	2261647	2	8	16	TG	13	6
68834	7	2262794	2262808	3	5	15	TAA	17	3
68837	7	2272903	2272922	4	5	20	ATAG	24	4
68846	7	2290880	2290900	3	7	21	ATT	11	3
68847	7	2291136	2291156	3	7	21	TCA	27	9
68857	7	2298952	2298979	2	14	28	AC	7	8
68858	7	2299084	2299103	2	10	20	GA	12	7
68861	7	2302334	2302363	2	15	30	CA	9	14
68863	7	2303496	2303513	2	9	18	GT	27	3

68879	7	2329750	2329777	2	14	28	AC	18	10
68882	7	2331681	2331704	2	12	24	TC	17	4
68883	7	2331820	2331837	2	9	18	TG	16	4
68884	7	2331934	2331969	2	18	36	GT	11	7
68885	7	2332087	2332108	2	11	22	TG	19	15
68888	7	2333868	2333891	2	12	24	GT	12	6
68890	7	2334025	2334048	2	12	24	AC	15	13
68894	7	2335950	2335969	2	10	20	AC	19	7
68895	7	2336127	2336152	2	13	26	CA	14	9
68897	7	2336285	2336296	2	6	12	GT	23	4
68898	7	2336430	2336451	2	11	22	AC	23	12
68900	7	2336506	2336525	2	10	20	GT	13	9
68902	7	2338774	2338801	2	14	28	AC	21	8
68903	7	2339445	2339462	2	9	18	TG	22	6
68904	7	2340586	2340617	2	16	32	TG	12	5
68907	7	2346264	2346279	2	8	16	TG	18	5
68908	7	2346448	2346485	2	19	38	TG	8	8
68913	7	2353147	2353182	2	18	36	CA	16	10
68914	7	2353882	2353909	2	14	28	GT	6	6
68915	7	2354450	2354461	2	6	12	GT	18	8
68917	7	2355302	2355329	2	14	28	CA	17	9
68922	7	2369626	2369651	2	13	26	TG	17	10
68923	7	2369683	2369696	2	7	14	AC	23	4
68926	7	2381868	2381883	2	8	16	TG	27	5
68931	7	2403084	2403099	4	4	16	TGTA	27	4
68951	7	2435525	2435542	3	6	18	TCT	18	3
68953	7	2438223	2438242	2	10	20	AC	22	6
68957	7	2442867	2442882	2	8	16	AT	19	7
68958	7	2443778	2443795	2	9	18	CA	13	9
68959	7	2443911	2443922	2	6	12	AC	25	3
68962	7	2450690	2450704	3	5	15	CTG	25	5
68963	7	2450706	2450723	3	6	18	TTA	28	5
68965	7	2454970	2454997	2	14	28	AC	17	6
68966	7	2456934	2456953	2	10	20	CA	15	5
68968	7	2463416	2463431	2	8	16	AC	30	4
68969	7	2463630	2463659	2	15	30	CA	12	10
68971	7	2466781	2466818	2	19	38	TG	6	5
68972	7	2472649	2472670	2	11	22	CA	15	5
68973	7	2474753	2474772	2	10	20	TG	19	15
68974	7	2474928	2474963	2	18	36	AC	14	12
68978	7	2479499	2479519	3	7	21	CTC	30	8
68980	7	2483984	2484015	4	8	32	TCTT	20	8
68993	7	2527256	2527273	3	6	18	CAC	30	3
68998	7	2537637	2537674	2	19	38	TG	18	6
69013	7	2563172	2563201	2	15	30	CA	23	12
69018	7	2579688	2579739	2	26	52	GT	13	18
69020	7	2582738	2582757	2	10	20	TG	18	6
69021	7	2582888	2582903	2	8	16	TG	21	5
69023	7	2589035	2589080	2	23	46	CA	10	9
69035	7	2629173	2629202	2	15	30	CA	9	9
69039	7	2631582	2631597	2	8	16	GT	20	3

69043	7	2634963	2634992	2	15	30	GT	11	9
69050	7	2643166	2643179	2	7	14	GA	13	5
69052	7	2643606	2643626	3	7	21	CCT	18	7
69054	7	2644434	2644447	2	7	14	CA	18	6
69055	7	2644754	2644775	2	11	22	AC	11	8
69056	7	2657699	2657716	3	6	18	GCA	22	3
69078	7	2697117	2697132	2	8	16	GT	18	3
69091	7	2716538	2716549	2	6	12	GT	25	4
69100	7	2731958	2731985	4	7	28	AACC	24	4
69106	7	2749820	2749831	2	6	12	GT	16	7
69107	7	2749834	2749851	2	9	18	GT	14	8
69109	7	2759964	2760021	2	29	58	GT	10	13
69112	7	2767587	2767600	2	7	14	GT	22	11
69113	7	2767780	2767795	2	8	16	TC	25	5
69122	7	2781554	2781567	2	7	14	AG	27	4
69126	7	2795921	2795942	2	11	22	AC	12	11
69127	7	2797898	2797912	3	5	15	CAG	35	5
69136	7	2804289	2804304	2	8	16	CA	25	8
69137	7	2805732	2805759	2	14	28	GT	10	10
69138	7	2805917	2805928	2	6	12	CA	22	7
69140	7	2811187	2811234	4	12	48	TCAA	28	8
69143	7	2820412	2820429	2	9	18	AC	20	8
69149	7	2837075	2837088	2	7	14	GT	15	4
69152	7	2842937	2842951	3	5	15	TCT	18	9
69155	7	2843965	2843996	2	16	32	AC	6	9
69158	7	2846002	2846015	2	7	14	AC	23	3
69165	7	2863213	2863226	2	7	14	TG	19	10
69166	7	2863522	2863541	2	10	20	GT	16	5
69169	7	2869731	2869748	2	9	18	AC	10	9
69170	7	2869750	2869763	2	7	14	CA	14	10
69172	7	2871985	2871996	2	6	12	CA	10	4
69173	7	2873434	2873453	2	10	20	CA	18	6
69175	7	2879267	2879292	2	13	26	GT	10	7
69177	7	2884722	2884745	2	12	24	CA	26	8
69179	7	2890758	2890777	2	10	20	AC	16	8
69180	7	2890800	2890825	2	13	26	CA	24	5
69181	7	2890909	2890938	2	15	30	CA	15	13
69184	7	2893905	2893918	2	7	14	GT	18	7
69186	7	2902582	2902595	2	7	14	CA	25	3
69187	7	2906593	2906610	2	9	18	TG	23	7
69192	7	2928289	2928306	2	9	18	GT	15	5
69194	7	2930196	2930213	2	9	18	CT	27	4
69200	7	2935436	2935473	2	19	38	AC	22	9
69202	7	2939522	2939537	2	8	16	CA	19	8
69204	7	2940134	2940153	2	10	20	TG	15	7
69210	7	2947772	2947813	3	14	42	GAA	20	15
69216	7	2956630	2956659	3	10	30	TCC	17	8
69230	7	3002693	3002707	3	5	15	TGA	17	3
69232	7	3010661	3010688	4	7	28	TTTG	20	7
69233	7	3013664	3013725	2	31	62	GT	13	10
69236	7	3020400	3020415	2	8	16	AC	26	9

69238	7	3036766	3036789	2	12	24	CA	22	5
69240	7	3051178	3051193	2	8	16	AT	18	13
69247	7	3064491	3064514	2	12	24	AC	18	7
69250	7	3074557	3074576	2	10	20	GA	25	5
69255	7	3082077	3082090	2	7	14	CA	22	12
69256	7	3082242	3082253	2	6	12	AT	20	3
69257	7	3083938	3083967	2	15	30	TG	11	11
69260	7	3089925	3089939	3	5	15	TAA	21	3
69261	7	3090851	3090880	2	15	30	GT	15	8
69264	7	3092950	3092961	2	6	12	CA	25	4
69265	7	3097866	3097885	2	10	20	AC	22	16
69266	7	3097887	3097898	2	6	12	CA	20	12
69267	7	3101527	3101542	2	8	16	CA	17	6
69272	7	3112128	3112139	2	6	12	CA	23	3
69274	7	3112486	3112517	2	16	32	AC	15	11
69275	7	3112627	3112642	2	8	16	AT	10	14
69277	7	3113567	3113588	2	11	22	GT	11	7
69278	7	3114811	3114826	4	4	16	TGGT	26	4
69287	7	3121859	3121900	2	21	42	GT	23	9
69288	7	3124132	3124151	2	10	20	AC	20	6
69293	7	3153561	3153580	2	10	20	GT	22	6
69296	7	3157058	3157075	3	6	18	GTG	25	6
69307	7	3177964	3178005	2	21	42	TG	6	7
69311	7	3208398	3208425	2	14	28	GT	19	11
69312	7	3211331	3211344	2	7	14	GT	34	5
69330	7	3273680	3273691	2	6	12	AC	12	6
69331	7	3273693	3273712	2	10	20	CA	16	8
69339	7	3298523	3298550	4	7	28	TATC	17	11
69341	7	3302733	3302752	2	10	20	TC	15	9
69342	7	3302945	3302968	2	12	24	TC	13	8
69344	7	3323373	3323384	2	6	12	GA	25	4
69348	7	3335182	3335193	2	6	12	AC	28	9
69350	7	3339344	3339361	3	6	18	CTC	18	3
69353	7	3341846	3341861	2	8	16	AG	17	4
69355	7	3363583	3363594	2	6	12	CA	32	3
69357	7	3371847	3371862	2	8	16	TG	12	5
69358	7	3372723	3372746	4	6	24	TCCA	32	5
69364	7	3375516	3375543	2	14	28	CA	9	7
69373	7	3392796	3392819	3	8	24	GTA	11	4
69378	7	3398988	3399003	2	8	16	TG	12	4
69382	7	3411549	3411562	2	7	14	CT	23	3
69385	7	3421696	3421713	3	6	18	ATT	18	3
69386	7	3422031	3422057	3	9	27	TAT	14	5
69387	7	3426365	3426379	3	5	15	GCA	25	4
69392	7	3436496	3436515	2	10	20	GT	12	3
69408	7	3463748	3463769	2	11	22	AG	10	7
69416	7	3472423	3472452	2	15	30	CA	8	12
69417	7	3473707	3473720	2	7	14	AC	25	5
69420	7	3482566	3482579	2	7	14	GA	15	3
69424	7	3486286	3486333	2	24	48	AC	10	10
69438	7	3505464	3505481	3	6	18	CTG	32	5

69440	7	3514275	3514296	2	11	22	TC	10	4
69441	7	3514343	3514366	2	12	24	CT	18	7
69442	7	3517943	3517960	2	9	18	CT	26	4
69443	7	3518319	3518336	2	9	18	CT	6	3
69445	7	3521957	3521996	2	20	40	TG	7	8
69447	7	3526254	3526267	2	7	14	AC	23	5
69452	7	3532615	3532630	4	4	16	AAAT	21	4
69457	7	3544218	3544233	2	8	16	GA	21	8
69458	7	3546783	3546826	2	22	44	CA	20	12
69466	7	3561246	3561269	2	12	24	TG	14	7
69471	7	3561800	3561832	3	11	33	CTC	26	3
69476	7	3562961	3562980	2	10	20	TG	9	4
69478	7	3563040	3563065	2	13	26	GT	18	8
69482	7	3565674	3565697	2	12	24	GT	10	5
69484	7	3566259	3566274	2	8	16	TG	12	10
69495	7	3577746	3577763	3	6	18	CCA	12	4
69496	7	3578114	3578129	2	8	16	AC	27	6
69497	7	3578340	3578359	4	5	20	CTCC	9	6
69499	7	3583567	3583594	2	14	28	CA	16	10
69506	7	3602085	3602124	2	20	40	AC	8	8
69510	7	3606062	3606073	2	6	12	CA	22	6
69527	7	3625941	3625952	2	6	12	TG	31	4
69528	7	3626013	3626040	2	14	28	AC	11	7
69545	7	3650271	3650304	2	17	34	GT	11	8
69555	7	3662864	3662891	2	14	28	CA	15	13
69558	7	3665158	3665172	3	5	15	TTG	21	3
69565	7	3670212	3670235	4	6	24	AAAT	23	6
69566	7	3671135	3671150	2	8	16	AC	22	6
69568	7	3671498	3671509	2	6	12	AC	27	3
69571	7	3673094	3673115	2	11	22	TG	11	8
69573	7	3674041	3674084	2	22	44	GT	11	11
69576	7	3674902	3674915	2	7	14	GT	22	6
69578	7	3680385	3680414	2	15	30	GT	13	11
69579	7	3683421	3683432	2	6	12	AC	23	6
69598	7	3700096	3700111	2	8	16	AT	6	6
69599	7	3702886	3702897	2	6	12	AT	18	3
69600	7	3703977	3703992	2	8	16	GT	27	13
69601	7	3704118	3704143	2	13	26	CA	16	7
69602	7	3705744	3705761	3	6	18	TCT	22	5
69608	7	3708976	3708989	2	7	14	GA	14	3
69612	7	3709642	3709673	2	16	32	TG	11	10
69614	7	3711309	3711330	2	11	22	TG	23	5
69618	7	3714462	3714485	2	12	24	GT	6	9
69619	7	3716102	3716113	2	6	12	GT	10	3
69625	7	3721940	3721966	3	9	27	CTC	22	7
69628	7	3726940	3726965	2	13	26	TG	12	8
69638	7	3734446	3734475	2	15	30	TG	17	17
69639	7	3734715	3734726	2	6	12	GT	25	3
69642	7	3735113	3735134	2	11	22	TG	16	5
69661	7	3751170	3751199	2	15	30	AC	11	6
69679	7	3770522	3770543	2	11	22	TG	11	6

69680	7	3771842	3771859	2	9	18	CA	8	5
69683	7	3773826	3773843	2	9	18	TG	21	4
69689	7	3779228	3779247	2	10	20	AC	15	12
69690	7	3779504	3779515	2	6	12	CA	23	20
69693	7	3780526	3780543	2	9	18	CA	15	6
69698	7	3780889	3780910	2	11	22	CA	13	7
69699	7	3782521	3782548	2	14	28	GT	16	16
69701	7	3783421	3783432	2	6	12	TG	25	5
69705	7	3786954	3786965	2	6	12	GT	24	5
69706	7	3788976	3788989	2	7	14	GT	22	6
69711	7	3796195	3796214	2	10	20	TG	25	10
69713	7	3797752	3797767	2	8	16	TG	24	7
69715	7	3806569	3806592	3	8	24	AGC	26	3
69717	7	3807682	3807709	2	14	28	GT	23	14
69722	7	3811695	3811718	2	12	24	TG	19	7
69723	7	3814807	3814827	3	7	21	CTC	17	4
69726	7	3815607	3815626	2	10	20	GT	13	12
69727	7	3818168	3818181	2	7	14	TG	20	3
69730	7	3825489	3825506	2	9	18	GT	20	6
69736	7	3842940	3842963	2	12	24	GA	13	9
69739	7	3845662	3845683	2	11	22	GT	7	4
69741	7	3845713	3845738	2	13	26	AG	12	4
69742	7	3845996	3846039	2	22	44	TG	6	9
69743	7	3846148	3846175	2	14	28	GT	6	6
69748	7	3852039	3852054	2	8	16	GT	29	3
69749	7	3852703	3852714	2	6	12	GT	23	5
69751	7	3855547	3855562	2	8	16	CA	20	10
69753	7	3857326	3857353	2	14	28	CA	13	12
69754	7	3857598	3857615	2	9	18	TG	17	5
69756	7	3859106	3859153	2	24	48	TC	8	6
69767	7	3888631	3888642	2	6	12	TG	23	3
69770	7	3900105	3900116	2	6	12	TG	28	3
69775	7	3910980	3911007	2	14	28	AC	19	9
69777	7	3918268	3918295	2	14	28	AC	13	6
69779	7	3921039	3921064	2	13	26	CA	12	11
69780	7	3921804	3921815	2	6	12	CA	32	4
69781	7	3923326	3923346	3	7	21	GAT	29	6
69782	7	3924934	3924949	2	8	16	GT	10	9
69783	7	3938026	3938041	2	8	16	AG	23	5
69790	7	3956927	3956938	2	6	12	TA	7	3
69794	7	3969666	3969681	2	8	16	GT	25	10
69804	7	3998114	3998125	2	6	12	AT	26	4
69805	7	3999095	3999114	4	5	20	AAAC	19	3
69806	7	4001043	4001060	2	9	18	CA	9	7
69814	7	4046568	4046583	2	8	16	TG	6	4
69815	7	4053988	4054021	2	17	34	AC	7	6
69817	7	4057375	4057394	2	10	20	CA	15	4
69827	7	4079411	4079426	4	4	16	GAAG	26	4
69829	7	4080893	4080916	2	12	24	AC	19	10
69831	7	4081470	4081495	2	13	26	GT	15	9
69833	7	4082064	4082081	2	9	18	CA	21	3

69839	7	4090695	4090706	2	6	12	AT	27	3
69840	7	4093704	4093717	2	7	14	AC	22	4
69845	7	4102062	4102089	4	7	28	AAAT	20	11
69848	7	4109767	4109786	2	10	20	GT	10	11
69849	7	4113949	4113962	2	7	14	GT	24	10
69852	7	4121464	4121483	2	10	20	GA	27	4
69854	7	4122528	4122542	3	5	15	CAG	26	4
69858	7	4127388	4127408	3	7	21	CTT	29	3
69859	7	4127791	4127812	2	11	22	CA	33	6
69869	7	4147958	4147989	2	16	32	GA	16	5
69872	7	4153622	4153645	4	6	24	AGTG	26	3
69874	7	4158916	4158939	2	12	24	AC	24	6
69877	7	4164021	4164042	2	11	22	AT	22	11
69880	7	4174305	4174330	2	13	26	GT	18	11
69881	7	4174589	4174620	2	16	32	AC	18	10
69890	7	4186376	4186431	4	14	56	ATCT	17	9
69891	7	4186557	4186578	2	11	22	GT	18	7
69896	7	4194495	4194514	2	10	20	CT	22	6
69897	7	4197991	4198008	2	9	18	GT	20	6
69898	7	4200385	4200405	3	7	21	CAG	27	7
69899	7	4200967	4200978	2	6	12	CA	26	4
69900	7	4201184	4201207	2	12	24	CA	14	8
69904	7	4205572	4205609	2	19	38	TG	13	8
69905	7	4206018	4206032	3	5	15	TCT	6	3
69906	7	4206135	4206146	2	6	12	GA	26	8
69907	7	4206682	4206697	2	8	16	CT	19	8
69908	7	4206698	4206717	2	10	20	CA	21	7
69911	7	4213993	4214006	2	7	14	TG	10	4
69914	7	4219699	4219714	2	8	16	TA	9	3
69929	7	4246096	4246117	2	11	22	AC	16	5
69933	7	4251705	4251746	2	21	42	TG	13	9
69934	7	4251880	4251899	2	10	20	GT	21	6
69937	7	4253499	4253518	2	10	20	GA	17	9
69938	7	4253520	4253535	2	8	16	AG	21	10
69940	7	4253729	4253754	2	13	26	GT	12	6
69945	7	4255295	4255310	2	8	16	TG	15	7
69955	7	4265269	4265286	2	9	18	AG	13	4
69960	7	4269028	4269047	4	5	20	TTTG	19	14
69961	7	4279544	4279571	2	14	28	AC	20	10
69963	7	4289953	4289974	2	11	22	GT	13	6
69967	7	4292563	4292582	2	10	20	CA	17	9
69968	7	4297152	4297169	3	6	18	CTG	28	5
69970	7	4299906	4299926	3	7	21	ATC	10	9
69975	7	4314743	4314760	2	9	18	GA	17	8
69987	7	4342392	4342411	4	5	20	TTTG	23	4
69988	7	4344072	4344087	2	8	16	AC	27	7
69989	7	4346965	4346976	2	6	12	TG	7	4
69993	7	4352494	4352511	3	6	18	AAT	9	3
69997	7	4359757	4359776	4	5	20	ATTT	20	5
70006	7	4365597	4365614	2	9	18	GT	8	8
70008	7	4367060	4367071	2	6	12	TA	26	4

70010	7	4368241	4368258	2	9	18	GT	20	5
70013	7	4374517	4374534	3	6	18	AGG	21	4
70016	7	4381982	4381995	2	7	14	AT	21	4
70017	7	4384466	4384481	4	4	16	TAAA	29	4
70019	7	4385240	4385257	2	9	18	AG	14	8
70021	7	4392722	4392739	2	9	18	GT	20	7
70030	7	4400400	4400437	2	19	38	TG	12	8
70034	7	4413608	4413622	3	5	15	GTT	30	3
70039	7	4427722	4427741	2	10	20	GT	21	15
70040	7	4429025	4429052	4	7	28	TTTC	10	9
70042	7	4437745	4437759	3	5	15	CAA	23	4
70051	7	4461667	4461680	2	7	14	AG	22	3
70056	7	4466017	4466038	2	11	22	CA	15	10
70058	7	4468106	4468123	2	9	18	CA	23	3
70059	7	4468806	4468827	2	11	22	AC	21	9
70060	7	4470077	4470100	2	12	24	GT	17	7
70062	7	4475922	4475945	2	12	24	GT	19	13
70063	7	4476399	4476434	2	18	36	AG	6	6
70064	7	4476626	4476651	2	13	26	CA	19	7
70065	7	4477844	4477859	2	8	16	AC	28	6
70071	7	4481728	4481779	4	13	52	AGAT	17	7
70072	7	4482464	4482487	3	8	24	ATG	29	7
70075	7	4484670	4484705	2	18	36	CA	6	10
70077	7	4484986	4485007	2	11	22	TG	22	10
70078	7	4485721	4485772	2	26	52	GT	8	12
70079	7	4486795	4486808	2	7	14	GA	27	4
70086	7	4493866	4493885	2	10	20	AC	9	9
70087	7	4495166	4495186	3	7	21	CAA	30	7
70091	7	4497737	4497754	2	9	18	GT	18	15
70098	7	4504553	4504576	2	12	24	GA	18	7
70099	7	4505157	4505186	3	10	30	TGT	20	5
70103	7	4513402	4513413	2	6	12	TG	25	3
70104	7	4513815	4513830	2	8	16	TG	25	5
70111	7	4526326	4526341	2	8	16	GT	23	6
70113	7	4530232	4530291	2	30	60	AC	10	12
70114	7	4530970	4530985	2	8	16	CA	12	14
70116	7	4532796	4532825	2	15	30	CA	18	10
70117	7	4533017	4533042	2	13	26	CA	11	6
70122	7	4542306	4542317	2	6	12	CT	21	3
70123	7	4546274	4546285	2	6	12	GT	25	4
70125	7	4547394	4547415	2	11	22	CA	10	10
70126	7	4547521	4547532	2	6	12	AC	21	5
70127	7	4547611	4547622	2	6	12	TG	24	3
70129	7	4548978	4549011	2	17	34	TG	7	5
70131	7	4549715	4549726	2	6	12	CT	36	3
70132	7	4549975	4550000	2	13	26	TG	19	8
70136	7	4557270	4557289	2	10	20	TG	14	10
70137	7	4557563	4557577	3	5	15	AGC	29	3
70138	7	4562454	4562481	2	14	28	GT	22	7
70147	7	4567360	4567373	2	7	14	GT	12	5
70148	7	4567375	4567390	2	8	16	TG	13	6

70151	7	4570718	4570733	2	8	16	CA	6	4
70153	7	4572844	4572863	2	10	20	TG	17	14
70159	7	4586287	4586326	2	20	40	AC	18	14
70160	7	4588770	4588785	4	4	16	CTGT	22	4
70162	7	4592440	4592453	2	7	14	AC	27	6
70170	7	4598164	4598181	2	9	18	CA	19	9
70172	7	4603510	4603525	2	8	16	TG	13	8
70175	7	4603846	4603871	2	13	26	AC	7	8
70178	7	4611461	4611472	2	6	12	TC	18	13
70182	7	4620288	4620315	2	14	28	GT	10	9
70184	7	4621760	4621792	3	11	33	GCT	26	19
70186	7	4624924	4624947	3	8	24	ATT	16	6
70188	7	4630683	4630726	2	22	44	GT	11	7
70189	7	4633268	4633285	2	9	18	GT	19	9
70190	7	4633287	4633302	2	8	16	TG	18	8
70193	7	4639980	4640001	2	11	22	CT	15	4
70197	7	4642455	4642484	2	15	30	AC	20	15
70207	7	4670900	4670911	2	6	12	TG	9	7
70208	7	4671770	4671785	2	8	16	GT	24	5
70213	7	4675423	4675436	2	7	14	CA	31	5
70214	7	4675490	4675519	2	15	30	GT	20	9
70221	7	4688093	4688114	2	11	22	TG	7	6
70225	7	4698692	4698713	2	11	22	AC	14	9
70230	7	4710323	4710336	2	7	14	TG	23	5
70231	7	4711376	4711389	2	7	14	GT	25	15
70234	7	4713973	4713992	2	10	20	CA	8	7
70238	7	4715547	4715574	2	14	28	TG	21	13
70239	7	4717483	4717508	2	13	26	GT	21	10
70244	7	4725204	4725215	2	6	12	GT	23	15
70245	7	4725358	4725387	2	15	30	GT	13	10
70246	7	4725481	4725492	2	6	12	TG	14	6
70247	7	4726781	4726810	2	15	30	GT	14	6
70248	7	4726917	4726944	2	14	28	GT	24	13
70252	7	4734774	4734787	2	7	14	GT	19	9
70254	7	4739200	4739213	2	7	14	GT	22	10
70256	7	4739443	4739456	2	7	14	GT	21	13
70259	7	4759094	4759109	2	8	16	GA	27	6
70261	7	4763217	4763234	2	9	18	TG	21	13
70264	7	4775472	4775487	2	8	16	GT	23	6
70265	7	4776118	4776129	2	6	12	CA	22	10
70267	7	4783554	4783583	2	15	30	TG	23	14
70269	7	4791108	4791121	2	7	14	CT	7	4
70278	7	4803935	4803956	2	11	22	GT	15	11
70279	7	4805176	4805187	2	6	12	AC	9	4
70286	7	4815742	4815761	2	10	20	AC	14	4
70288	7	4817151	4817168	3	6	18	AGG	18	4
70289	7	4817342	4817355	2	7	14	GT	7	3
70290	7	4821110	4821135	2	13	26	CA	9	8
70292	7	4822268	4822279	2	6	12	AT	22	3
70293	7	4827107	4827138	2	16	32	CA	16	7
70294	7	4828946	4828965	4	5	20	AAGA	15	6

70298	7	4831650	4831675	2	13	26	TG	17	15
70299	7	4831824	4831857	2	17	34	AC	19	14
70300	7	4832012	4832047	2	18	36	CA	16	10
70301	7	4838046	4838075	2	15	30	AC	8	6
70302	7	4838468	4838479	2	6	12	AC	24	4
70306	7	4840172	4840209	2	19	38	TG	13	11
70311	7	4843265	4843276	2	6	12	GT	24	4
70315	7	4850473	4850493	3	7	21	CAT	32	5
70323	7	4864910	4864925	4	4	16	TCAC	30	3
70328	7	4878849	4878872	2	12	24	CA	10	6
70329	7	4879352	4879377	2	13	26	GT	17	13
70331	7	4885768	4885787	2	10	20	GT	16	5
70334	7	4890928	4890949	2	11	22	CA	17	8
70335	7	4894669	4894696	2	14	28	AC	20	10
70338	7	4908313	4908342	2	15	30	AC	14	14
70361	7	4954429	4954443	3	5	15	TTC	25	6
70363	7	4956861	4956874	2	7	14	TG	24	9
70364	7	4957923	4957952	2	15	30	AC	9	5
70368	7	4960844	4960861	3	6	18	GAG	27	5
70373	7	4964308	4964331	2	12	24	GT	16	9
70378	7	4965715	4965734	2	10	20	CA	13	10
70382	7	4968081	4968110	2	15	30	TG	7	8
70384	7	4970231	4970248	2	9	18	CA	21	4
70385	7	4970380	4970399	2	10	20	GT	20	5
70389	7	4976314	4976325	2	6	12	CA	26	3
70390	7	4976650	4976667	2	9	18	AC	28	4
70391	7	4979430	4979443	2	7	14	GT	21	8
70392	7	4979446	4979457	2	6	12	GT	21	8
70393	7	4980446	4980463	2	9	18	CA	19	3
70394	7	4982210	4982229	2	10	20	CT	19	4
70396	7	4984370	4984393	2	12	24	TG	7	9
70398	7	4986307	4986342	2	18	36	AC	19	9
70402	7	4988955	4988976	2	11	22	GT	20	7
70405	7	4995785	4995808	2	12	24	TG	20	9
70406	7	4996037	4996050	2	7	14	AC	13	10
70407	7	4996053	4996070	2	9	18	AC	14	9
70408	7	4997834	4997871	2	19	38	AC	10	10
70411	7	5009069	5009088	2	10	20	TG	20	12
70416	7	5012961	5012982	2	11	22	CA	12	9
70417	7	5013086	5013111	2	13	26	TG	19	13
70419	7	5014484	5014539	2	28	56	TG	6	7
70420	7	5015641	5015652	2	6	12	TG	12	3
70421	7	5015657	5015670	2	7	14	TG	26	5
70432	7	5022019	5022046	2	14	28	AC	15	12
70447	7	5033710	5033725	2	8	16	CA	20	3
70448	7	5033765	5033786	2	11	22	AC	12	6
70449	7	5033864	5033895	2	16	32	CA	23	10
70450	7	5035492	5035519	2	14	28	AC	14	8
70451	7	5035635	5035654	2	10	20	GT	20	6
70455	7	5040146	5040169	2	12	24	TG	10	11
70456	7	5040350	5040367	2	9	18	GT	15	10

70458	7	5040507	5040530	2	12	24	AC	16	16
70460	7	5041333	5041350	3	6	18	AGA	17	6
70461	7	5042945	5042956	2	6	12	GT	22	11
70463	7	5043054	5043077	2	12	24	CA	15	6
70467	7	5048600	5048613	2	7	14	TG	23	6
70469	7	5050919	5050934	2	8	16	CA	21	3
70471	7	5057080	5057091	2	6	12	AT	15	4
70473	7	5058478	5058495	2	9	18	AC	6	5
70476	7	5060323	5060348	2	13	26	AC	30	4
70481	7	5070585	5070604	2	10	20	AG	28	6
70484	7	5079849	5079892	2	22	44	TG	6	9
70487	7	5081021	5081038	2	9	18	GT	9	3
70490	7	5082296	5082325	2	15	30	CA	8	7
70494	7	5087344	5087357	2	7	14	GT	28	4
70496	7	5087659	5087672	2	7	14	GT	19	11
70497	7	5090496	5090533	2	19	38	GT	9	6
70500	7	5093138	5093149	2	6	12	TC	24	6
70501	7	5093549	5093564	2	8	16	GT	16	4
70504	7	5094076	5094087	2	6	12	GT	29	3
70505	7	5095557	5095578	2	11	22	CA	18	6
70508	7	5097232	5097259	2	14	28	AC	13	8
70510	7	5105772	5105799	2	14	28	TG	12	5
70512	7	5109583	5109598	4	4	16	ATGT	27	4
70525	7	5137836	5137847	2	6	12	TG	21	3
70530	7	5145566	5145597	2	16	32	GT	13	9
70531	7	5145814	5145829	4	4	16	TTTA	21	4
70532	7	5146154	5146165	2	6	12	GT	27	3
70533	7	5146305	5146334	2	15	30	AC	16	14
70534	7	5146389	5146410	2	11	22	CA	13	7
70535	7	5146560	5146573	2	7	14	CG	7	5
70537	7	5146685	5146710	2	13	26	AC	7	5
70539	7	5157427	5157442	2	8	16	TG	33	4
70540	7	5159975	5160000	2	13	26	AC	12	6
70547	7	5201007	5201022	2	8	16	GT	31	3
70550	7	5206608	5206625	2	9	18	TG	17	4
70554	7	5237293	5237332	4	10	40	CTAT	13	11
70555	7	5239615	5239642	4	7	28	AATA	14	5
70562	7	5243723	5243748	2	13	26	CT	6	7
70564	7	5245925	5245964	2	20	40	GT	8	9
70565	7	5247800	5247813	2	7	14	CT	25	4
70568	7	5249709	5249732	2	12	24	TG	16	7
70572	7	5267920	5267933	2	7	14	AC	26	6
70573	7	5268208	5268223	4	4	16	TTTC	12	6
70575	7	5269812	5269837	2	13	26	CT	15	5
70579	7	5287683	5287703	3	7	21	CTT	27	3
70580	7	5293333	5293344	2	6	12	CT	16	4
70581	7	5293612	5293645	2	17	34	CA	13	16
70583	7	5299971	5299986	2	8	16	AC	26	6
70585	7	5302018	5302033	2	8	16	GA	18	4
70595	7	5319759	5319776	2	9	18	GA	19	3
70597	7	5327993	5328004	2	6	12	GA	25	3

70598	7	5329441	5329456	2	8	16	GT	31	3
70599	7	5337002	5337045	2	22	44	AC	12	12
70600	7	5338523	5338534	2	6	12	CA	29	4
70604	7	5351535	5351568	2	17	34	TG	18	17
70606	7	5352166	5352187	2	11	22	CA	18	6
70608	7	5352448	5352461	2	7	14	AT	21	8
70614	7	5369918	5369931	2	7	14	GT	24	6
70615	7	5370045	5370080	2	18	36	TG	15	8
70619	7	5392317	5392340	2	12	24	AC	17	8
70620	7	5395495	5395510	2	8	16	AG	26	3
70622	7	5409066	5409085	2	10	20	TG	23	5
70634	7	5432729	5432752	3	8	24	TCT	15	5
70641	7	5438740	5438759	4	5	20	GAAA	26	3
70643	7	5444370	5444393	2	12	24	AC	19	10
70644	7	5446863	5446882	4	5	20	AAAG	16	3
70647	7	5454998	5455013	2	8	16	TG	12	7
70650	7	5455459	5455491	3	11	33	CTT	20	10
70652	7	5456030	5456041	2	6	12	CA	21	7
70654	7	5460812	5460843	2	16	32	CA	11	15
70662	7	5466868	5466887	2	10	20	TG	17	9
70663	7	5466936	5466949	2	7	14	TC	23	3
70664	7	5467058	5467071	2	7	14	GT	20	4
70667	7	5471009	5471023	3	5	15	TCC	27	4
70669	7	5475932	5475949	3	6	18	CTC	20	6
70671	7	5477046	5477079	2	17	34	TG	11	10
70673	7	5481877	5481892	2	8	16	GT	27	4
70675	7	5486559	5486600	2	21	42	AC	12	10
70683	7	5501228	5501243	2	8	16	GT	19	14
70685	7	5511471	5511498	2	14	28	CA	22	9
70696	7	5526196	5526234	3	13	39	CAA	21	14
70725	7	5592000	5592031	2	16	32	CA	9	11
70730	7	5599762	5599785	2	12	24	CA	6	4
70732	7	5600646	5600661	4	4	16	CCTC	16	3
70740	7	5612659	5612682	2	12	24	GT	12	6
70742	7	5616597	5616610	2	7	14	GT	20	15
70749	7	5631365	5631376	2	6	12	TG	22	4
70751	7	5636914	5636928	3	5	15	GGT	26	4
70752	7	5638379	5638414	4	9	36	ATCT	26	15
70755	7	5641823	5641840	3	6	18	AAC	21	4
70763	7	5658133	5658154	2	11	22	AC	21	7
70765	7	5659506	5659517	2	6	12	AC	12	7
70767	7	5662303	5662346	2	22	44	CA	11	9
70768	7	5662503	5662534	2	16	32	TG	9	11
70772	7	5668737	5668760	2	12	24	CA	19	9
70773	7	5668952	5668977	2	13	26	GT	6	8
70774	7	5671725	5671739	3	5	15	ATT	12	3
70786	7	5699789	5699806	2	9	18	GT	24	9
70792	7	5708605	5708622	2	9	18	CA	18	6
70793	7	5714263	5714298	2	18	36	TG	11	10
70794	7	5714486	5714501	4	4	16	CCTC	7	3
70795	7	5714631	5714644	2	7	14	CA	20	13

70804	7	5735684	5735715	4	8	32	GGAT	23	6
70809	7	5741994	5742007	2	7	14	CA	18	3
70813	7	5748065	5748080	2	8	16	GT	13	7
70817	7	5753368	5753399	2	16	32	AC	17	12
70820	7	5756781	5756820	2	20	40	TG	9	8
70826	7	5776261	5776275	3	5	15	AGA	25	5
70827	7	5777701	5777732	2	16	32	AC	21	7
70830	7	5780883	5780906	4	6	24	CAGA	21	6
70831	7	5781178	5781201	4	6	24	TTTC	10	4
70832	7	5781417	5781450	2	17	34	AC	12	10
70837	7	5784241	5784252	2	6	12	AC	27	6
70838	7	5784255	5784266	2	6	12	AC	21	3
70844	7	5796095	5796130	2	18	36	AC	6	8
70846	7	5802047	5802058	2	6	12	CA	22	9
70847	7	5802061	5802072	2	6	12	CA	24	9
70856	7	5811718	5811741	2	12	24	CA	7	5
70859	7	5815307	5815330	3	8	24	GAG	20	4
70863	7	5824843	5824898	2	28	56	AC	7	12
70870	7	5839038	5839051	2	7	14	TG	24	11
70875	7	5845200	5845221	2	11	22	GT	18	6
70880	7	5859679	5859702	3	8	24	ATC	21	6
70883	7	5864640	5864661	2	11	22	TC	14	6
70886	7	5864700	5864719	2	10	20	AC	14	10
70889	7	5871764	5871815	2	26	52	TG	13	12
70895	7	5895780	5895806	3	9	27	TGC	29	8
70899	7	5908180	5908203	2	12	24	GT	10	8
70900	7	5908290	5908307	2	9	18	AC	16	5
70901	7	5909367	5909380	2	7	14	GT	24	3
70903	7	5916202	5916213	2	6	12	AC	27	5
70904	7	5916416	5916427	2	6	12	CA	25	3
70915	7	5929654	5929669	2	8	16	AC	29	5
70921	7	5942251	5942270	2	10	20	CA	28	6
70923	7	5945709	5945720	2	6	12	AG	25	3
70924	7	5945883	5945900	3	6	18	GAG	20	3
70927	7	5949387	5949400	2	7	14	CA	10	7
70928	7	5949403	5949416	2	7	14	CA	13	11
70929	7	5949529	5949550	2	11	22	AC	21	15
70937	7	5951621	5951648	2	14	28	TG	18	12
70938	7	5952594	5952613	4	5	20	TTTA	29	9
70940	7	5953537	5953552	2	8	16	CA	28	6
70941	7	5953844	5953859	4	4	16	TCTT	15	9
70945	7	5955674	5955693	4	5	20	CTGT	23	5
70946	7	5955777	5955822	2	23	46	AC	7	9
70949	7	5956868	5956879	2	6	12	TG	12	3
70950	7	5957030	5957069	2	20	40	CA	11	6
70951	7	5957174	5957187	2	7	14	TG	16	3
70954	7	5957583	5957596	2	7	14	GT	27	3
70955	7	5957667	5957682	2	8	16	TG	25	3
70956	7	5957733	5957744	2	6	12	TG	14	4
70957	7	5957747	5957760	2	7	14	TG	13	5
70958	7	5957906	5957923	2	9	18	TG	19	4

70960	7	5959589	5959616	2	14	28	TC	16	6
70961	7	5962775	5962790	2	8	16	TG	14	11
70962	7	5962908	5962922	3	5	15	CCA	21	5
70964	7	5964970	5964991	2	11	22	TG	12	7
70968	7	5969676	5969699	2	12	24	TG	25	5
70969	7	5969836	5969855	2	10	20	AC	16	10
70972	7	5973297	5973318	2	11	22	TG	19	6
70974	7	5976591	5976616	2	13	26	GT	15	10
70975	7	5976756	5976783	2	14	28	TC	27	5
70977	7	5980194	5980211	2	9	18	AC	21	3
70981	7	5983660	5983677	2	9	18	GT	18	6
70983	7	5986989	5987026	2	19	38	AC	8	6
70985	7	5988534	5988553	2	10	20	GT	17	11
70991	7	5994523	5994544	2	11	22	AG	17	4
70992	7	5994661	5994694	2	17	34	TG	14	8
70993	7	5999980	6000003	2	12	24	TG	18	4
70994	7	6003251	6003288	2	19	38	CA	7	3
70998	7	6006201	6006214	2	7	14	AC	18	3
70999	7	6007731	6007752	2	11	22	TG	12	9
71006	7	6015035	6015054	2	10	20	GT	17	3
71012	7	6023244	6023263	2	10	20	GT	17	5
71017	7	6028440	6028465	2	13	26	TG	14	6
71021	7	6030523	6030550	2	14	28	TG	14	8
71029	7	6037951	6037964	2	7	14	CA	14	4
71030	7	6038756	6038773	2	9	18	GT	17	5
71032	7	6043376	6043411	2	18	36	TG	13	7
71033	7	6043584	6043617	2	17	34	AC	15	11
71034	7	6043773	6043784	2	6	12	GT	18	3
71035	7	6043926	6043943	2	9	18	TG	20	7
71036	7	6043944	6043955	2	6	12	CG	17	7
71040	7	6054199	6054222	2	12	24	GT	11	8
71052	7	6067773	6067784	2	6	12	TG	24	5
71054	7	6072771	6072788	2	9	18	CA	7	5
71055	7	6072887	6072914	2	14	28	CA	17	4
71056	7	6073070	6073087	2	9	18	GT	16	11
71057	7	6073089	6073102	2	7	14	TG	22	14
71058	7	6073224	6073245	2	11	22	TG	24	7
71064	7	6081381	6081394	2	7	14	AG	22	5
71066	7	6091234	6091257	2	12	24	GT	10	5
71068	7	6096996	6097022	3	9	27	TTA	7	4
71070	7	6097675	6097692	2	9	18	TG	17	4
71072	7	6099252	6099271	2	10	20	TG	30	6
71073	7	6099731	6099752	2	11	22	GT	25	4
71075	7	6101800	6101823	2	12	24	GA	10	6
71077	7	6101998	6102017	2	10	20	TG	25	3
71079	7	6107558	6107573	2	8	16	GT	22	3
71085	7	6114933	6114968	2	18	36	GT	15	8
71088	7	6118976	6118989	2	7	14	GA	25	3
71089	7	6120296	6120313	2	9	18	GT	17	4
71090	7	6120585	6120614	2	15	30	AC	10	10
71094	7	6124178	6124199	2	11	22	AG	14	4

71095	7	6124389	6124406	2	9	18	CA	19	5
71097	7	6124610	6124621	2	6	12	GT	16	3
71098	7	6125524	6125539	2	8	16	AC	20	5
71101	7	6125990	6126025	2	18	36	GT	14	13
71102	7	6126184	6126216	3	11	33	TTC	11	5
71109	7	6129477	6129490	2	7	14	TG	22	3
71116	7	6130191	6130202	2	6	12	TG	27	3
71117	7	6130862	6130879	2	9	18	AG	15	5
71118	7	6130945	6130970	2	13	26	AC	16	7
71123	7	6134646	6134669	2	12	24	AC	11	4
71139	7	6150426	6150437	2	6	12	CA	13	6
71140	7	6151626	6151643	2	9	18	CT	19	6
71143	7	6152211	6152228	2	9	18	GA	20	3
71144	7	6156737	6156758	2	11	22	CA	8	3
71146	7	6157097	6157120	2	12	24	AC	8	8
71148	7	6157298	6157309	2	6	12	AC	29	3
71149	7	6157564	6157603	2	20	40	GT	17	12
71150	7	6157755	6157772	2	9	18	CA	20	4
71153	7	6159189	6159200	2	6	12	CA	30	3
71155	7	6159839	6159866	2	14	28	CA	16	9
71161	7	6166599	6166614	2	8	16	AC	15	3
71162	7	6166617	6166630	2	7	14	AC	17	8
71164	7	6169037	6169052	2	8	16	AC	18	5
71165	7	6169393	6169422	2	15	30	TG	17	8
71167	7	6171343	6171372	2	15	30	CA	21	8
71173	7	6176064	6176091	2	14	28	TC	12	6
71178	7	6178432	6178449	2	9	18	TG	23	5
71181	7	6178835	6178848	2	7	14	TG	16	5
71183	7	6178995	6179012	2	9	18	GT	17	4
71189	7	6181418	6181429	2	6	12	TG	24	3
71192	7	6183184	6183207	2	12	24	AC	15	4
71193	7	6183684	6183695	2	6	12	CA	25	3
71194	7	6184006	6184029	2	12	24	CA	14	9
71196	7	6185657	6185668	2	6	12	AC	18	4
71198	7	6187486	6187505	2	10	20	AC	19	3
71201	7	6188352	6188367	2	8	16	GT	25	6
71202	7	6189224	6189241	2	9	18	CA	22	6
71205	7	6190218	6190259	2	21	42	TG	10	7
71206	7	6190369	6190396	2	14	28	TC	17	7
71207	7	6191735	6191748	2	7	14	CA	25	3
71209	7	6192671	6192684	2	7	14	AC	18	9
71211	7	6194500	6194515	2	8	16	GT	21	3
71215	7	6197531	6197542	2	6	12	TC	16	3
71217	7	6203509	6203542	2	17	34	CA	17	9
71218	7	6203598	6203613	2	8	16	CA	16	3
71219	7	6203615	6203628	2	7	14	AC	18	5
71220	7	6203915	6203938	2	12	24	AC	16	8
71222	7	6204213	6204230	2	9	18	GT	19	5
71223	7	6204611	6204628	3	6	18	CAG	29	3
71224	7	6205248	6205287	2	20	40	AG	12	9
71228	7	6208326	6208341	2	8	16	CA	25	3

71229	7	6209039	6209064	2	13	26	AC	16	10
71230	7	6209119	6209138	2	10	20	AC	20	4
71232	7	6210186	6210213	2	14	28	CA	8	8
71233	7	6210394	6210409	4	4	16	GCAC	28	6
71234	7	6210459	6210472	2	7	14	AC	21	5
71236	7	6211008	6211027	2	10	20	CA	28	3
71237	7	6212551	6212574	3	8	24	CTG	21	5
71246	7	6220426	6220439	2	7	14	CA	18	10
71247	7	6221195	6221222	2	14	28	AC	17	11
71248	7	6222011	6222024	2	7	14	AC	23	3
71249	7	6222135	6222150	2	8	16	AC	21	7
71254	7	6225044	6225055	2	6	12	CA	25	4
71258	7	6227761	6227790	2	15	30	CA	17	5
71260	7	6230045	6230059	3	5	15	CGG	27	6
71262	7	6235614	6235629	2	8	16	TG	7	6
71266	7	6239948	6239959	2	6	12	TG	33	3
71267	7	6240044	6240061	2	9	18	TG	18	9
71269	7	6241294	6241321	2	14	28	GT	8	6
71273	7	6241829	6241840	2	6	12	GT	10	7
71274	7	6241961	6241992	2	16	32	GT	13	10
71276	7	6245347	6245378	2	16	32	AC	16	19
71281	7	6251558	6251573	2	8	16	GT	25	4
71283	7	6253393	6253410	3	6	18	AGC	25	4
71284	7	6253563	6253576	2	7	14	TC	21	5
71290	7	6266338	6266377	2	20	40	CA	10	10
71298	7	6293011	6293030	2	10	20	AT	14	4
71302	7	6310961	6310986	2	13	26	AC	21	6
71305	7	6315375	6315395	3	7	21	TCT	19	3
71307	7	6324712	6324726	3	5	15	GTT	27	3
71312	7	6347299	6347325	3	9	27	TGT	22	3
71314	7	6352686	6352719	2	17	34	CA	15	14
71318	7	6354401	6354424	4	6	24	TTTA	26	3
71320	7	6359185	6359199	3	5	15	AAT	24	4
71321	7	6362669	6362683	3	5	15	TTA	20	4
71322	7	6362885	6362899	3	5	15	CCG	18	5
71329	7	6374448	6374459	2	6	12	GT	9	3
71330	7	6375799	6375818	2	10	20	AC	15	8
71334	7	6390408	6390421	2	7	14	TG	20	10
71337	7	6394918	6394929	2	6	12	TG	26	3
71338	7	6396479	6396518	4	10	40	ATCC	21	5
71343	7	6398946	6398969	2	12	24	GT	18	6
71345	7	6401102	6401117	2	8	16	GT	9	7
71346	7	6401119	6401132	2	7	14	TG	16	8
71347	7	6401288	6401313	2	13	26	TG	11	10
71348	7	6403006	6403017	2	6	12	TG	30	3
71349	7	6403098	6403117	2	10	20	GT	26	7
71352	7	6409344	6409357	2	7	14	TG	16	10
71357	7	6412460	6412471	2	6	12	TG	24	4
71358	7	6412901	6412938	2	19	38	CA	22	19
71362	7	6417509	6417530	2	11	22	TG	15	9
71373	7	6440066	6440077	2	6	12	GT	24	7

71374	7	6440172	6440183	2	6	12	TC	14	3
71376	7	6441639	6441654	2	8	16	AC	29	5
71378	7	6445966	6445981	2	8	16	GT	23	5
71379	7	6445983	6445998	2	8	16	TG	21	4
71383	7	6456977	6456998	2	11	22	TG	15	14
71384	7	6458245	6458292	4	12	48	TTCT	23	10
71385	7	6460166	6460177	2	6	12	TG	24	3
71389	7	6462049	6462062	2	7	14	TG	26	12
71390	7	6462068	6462083	4	4	16	GTGC	24	9
71391	7	6462496	6462521	2	13	26	GT	9	9
71393	7	6464855	6464882	2	14	28	TG	17	13
71397	7	6472447	6472464	2	9	18	AC	16	7
71401	7	6476986	6477035	2	25	50	AC	13	9
71407	7	6487133	6487159	3	9	27	TTC	25	9
71408	7	6491644	6491659	2	8	16	CA	30	5
71411	7	6500783	6500802	2	10	20	TG	9	6
71414	7	6501100	6501123	2	12	24	CA	10	6
71415	7	6501281	6501304	2	12	24	CA	24	5
71417	7	6502724	6502743	4	5	20	TTTG	27	3
71420	7	6503307	6503320	2	7	14	GA	23	5
71422	7	6503715	6503734	4	5	20	TATT	28	4
71425	7	6508027	6508038	2	6	12	AC	22	3
71427	7	6515134	6515147	2	7	14	AC	28	4
71429	7	6516149	6516164	2	8	16	TG	26	3
71430	7	6516269	6516280	2	6	12	TG	18	4
71433	7	6518705	6518726	2	11	22	AG	10	3
71434	7	6521482	6521493	2	6	12	GT	25	6
71435	7	6527777	6527796	4	5	20	CACG	23	14
71437	7	6530285	6530306	2	11	22	TG	18	8
71439	7	6534868	6534881	2	7	14	CA	24	4
71442	7	6545448	6545463	2	8	16	TG	22	3
71444	7	6547075	6547118	2	22	44	GT	6	8
71449	7	6557492	6557509	2	9	18	AC	21	3
71462	7	6561500	6561531	2	16	32	CA	12	7
71464	7	6563573	6563592	2	10	20	TG	32	4
71465	7	6563795	6563822	2	14	28	GT	20	8
71471	7	6573384	6573401	2	9	18	TG	24	7
71477	7	6584276	6584289	2	7	14	AC	18	4
71480	7	6587703	6587722	4	5	20	TCTG	24	3
71482	7	6592503	6592547	3	15	45	ATT	11	8
71483	7	6593565	6593580	2	8	16	TG	13	11
71485	7	6596382	6596425	2	22	44	TC	8	9
71487	7	6598446	6598475	2	15	30	CA	8	5
71493	7	6601494	6601517	2	12	24	TG	17	9
71494	7	6601658	6601677	2	10	20	TG	21	5
71501	7	6622843	6622866	3	8	24	AGG	20	6
71502	7	6629302	6629333	2	16	32	CA	10	10
71503	7	6631108	6631129	2	11	22	TG	16	6
71505	7	6633596	6633622	3	9	27	TCC	11	5
71506	7	6634752	6634777	2	13	26	TG	18	10
71507	7	6635493	6635507	3	5	15	GGT	15	3

71509	7	6639205	6639234	2	15	30	GT	18	13
71512	7	6645244	6645257	2	7	14	TG	21	5
71513	7	6645369	6645394	2	13	26	AC	11	12
71520	7	6655786	6655817	2	16	32	AC	10	8
71524	7	6658695	6658718	2	12	24	GT	27	7
71526	7	6660688	6660705	2	9	18	TG	25	6
71527	7	6663527	6663566	2	20	40	CA	13	15
71529	7	6667086	6667153	4	17	68	TTCT	9	11
71530	7	6669804	6669831	2	14	28	AC	10	8
71531	7	6670447	6670470	2	12	24	TG	24	12
71534	7	6671745	6671792	2	24	48	TG	11	13
71535	7	6676257	6676276	2	10	20	TG	18	5
71543	7	6681254	6681275	2	11	22	CA	13	8
71547	7	6684888	6684931	2	22	44	GT	9	10
71548	7	6685250	6685265	2	8	16	AC	20	3
71549	7	6685267	6685286	2	10	20	CA	18	5
71551	7	6685883	6685924	2	21	42	TG	12	15
71553	7	6696337	6696376	2	20	40	GT	8	12
71558	7	6705766	6705785	2	10	20	AC	25	12
71560	7	6707181	6707198	2	9	18	TG	17	11
71563	7	6708011	6708022	2	6	12	CA	17	4
71564	7	6710756	6710771	2	8	16	GT	24	4
71565	7	6710925	6710938	2	7	14	AC	21	4
71566	7	6715150	6715165	4	4	16	TCCC	17	4
71567	7	6715782	6715819	2	19	38	CA	13	9
71569	7	6719085	6719112	2	14	28	TG	7	7
71571	7	6721018	6721037	2	10	20	GT	18	5
71578	7	6734703	6734720	3	6	18	CTC	27	4
71581	7	6739929	6739944	2	8	16	AC	18	5
71586	7	6745314	6745345	2	16	32	GT	13	6
71587	7	6750707	6750718	2	6	12	CA	27	4
71589	7	6752636	6752657	2	11	22	AG	18	5
71594	7	6756636	6756695	2	30	60	GT	10	11
71595	7	6761063	6761080	2	9	18	TG	16	5
71600	7	6770815	6770826	2	6	12	GT	21	4
71606	7	6778769	6778788	4	5	20	AACC	32	5
71608	7	6780471	6780490	2	10	20	AC	15	7
71611	7	6781802	6781833	2	16	32	AC	13	4
71614	7	6783782	6783807	2	13	26	CA	8	12
71615	7	6783895	6783916	2	11	22	GT	20	10
71616	7	6786170	6786195	2	13	26	GT	23	9
71617	7	6786890	6786913	2	12	24	GT	20	16
71619	7	6789115	6789130	4	4	16	ATCC	26	4
71620	7	6791072	6791089	2	9	18	TG	21	12
71622	7	6794841	6794852	2	6	12	TG	26	8
71624	7	6795687	6795698	2	6	12	CA	8	7
71625	7	6795839	6795864	2	13	26	AC	12	10
71628	7	6798932	6798959	2	14	28	GT	24	10
71630	7	6799150	6799175	2	13	26	CA	17	9
71631	7	6799253	6799268	2	8	16	TG	17	4
71632	7	6799322	6799347	2	13	26	GT	22	9

71633	7	6799837	6799866	2	15	30	CT	12	10
71639	7	6803862	6803891	3	10	30	GGA	15	8
71640	7	6805487	6805506	2	10	20	AC	23	8
71642	7	6806712	6806737	2	13	26	AC	14	12
71643	7	6807023	6807048	2	13	26	AC	18	6
71647	7	6818109	6818124	2	8	16	AC	21	7
71648	7	6819448	6819473	2	13	26	CA	17	8
71655	7	6828808	6828843	2	18	36	AG	6	10
71656	7	6829074	6829103	2	15	30	CA	13	12
71661	7	6832648	6832691	2	22	44	AC	9	8
71663	7	6834454	6834465	2	6	12	GT	21	4
71667	7	6850477	6850494	2	9	18	AC	13	6
71683	7	6879414	6879433	2	10	20	TG	19	6
71687	7	6880448	6880459	2	6	12	GT	29	3
71692	7	6888589	6888608	2	10	20	AC	15	7
71701	7	6901519	6901538	2	10	20	GT	22	4
71711	7	6916935	6916946	2	6	12	GT	34	3
71715	7	6917833	6917850	2	9	18	TG	19	6
71717	7	6920296	6920317	2	11	22	TC	10	6
71721	7	6926377	6926388	2	6	12	GT	22	6
71726	7	6934986	6934997	2	6	12	GT	9	4
71727	7	6935000	6935013	2	7	14	GT	24	4
71729	7	6938282	6938299	2	9	18	AC	17	8
71733	7	6940501	6940518	2	9	18	AC	22	8
71743	7	6946540	6946561	2	11	22	TG	16	9
71745	7	6947808	6947822	3	5	15	TCC	26	4
71749	7	6952747	6952764	2	9	18	GT	19	7
71758	7	6969034	6969061	2	14	28	TC	15	6
71765	7	6989798	6989811	2	7	14	TA	23	7
71766	7	6990685	6990714	3	10	30	CCT	22	12
71769	7	6991137	6991148	2	6	12	AT	23	5
71773	7	6994249	6994282	2	17	34	AC	10	7
71774	7	6994328	6994347	2	10	20	AC	11	8
71775	7	6994399	6994418	2	10	20	CA	26	10
71776	7	6995119	6995134	2	8	16	TG	22	7
71778	7	6995372	6995401	2	15	30	CA	17	8
71782	7	6999021	6999036	4	4	16	TTTG	17	3
71784	7	7000418	7000445	2	14	28	TG	6	6
71785	7	7000596	7000611	2	8	16	GT	18	7
71791	7	7012982	7013003	2	11	22	GA	13	10
71794	7	7017148	7017169	2	11	22	GA	14	9
71796	7	7023848	7023867	2	10	20	AC	12	8
71797	7	7024090	7024101	2	6	12	CA	25	3
71799	7	7025670	7025697	2	14	28	CA	17	4
71803	7	7035277	7035288	2	6	12	AC	26	4
71813	7	7059502	7059517	2	8	16	TG	28	5
71823	7	7070017	7070030	2	7	14	AC	19	3
71824	7	7070034	7070045	2	6	12	CA	24	4
71825	7	7070385	7070412	2	14	28	TG	14	9
71854	7	7123929	7123942	2	7	14	GT	23	4
71863	7	7137156	7137171	2	8	16	AC	20	3

71865	7	7140500	7140531	2	16	32	TG	20	6
71875	7	7156383	7156400	3	6	18	TAC	25	5
71876	7	7156743	7156760	3	6	18	CTT	20	3
71883	7	7157824	7157845	2	11	22	CA	20	14
71888	7	7159878	7159893	2	8	16	GT	8	6
71889	7	7160216	7160230	3	5	15	CCA	19	3
71895	7	7163048	7163073	2	13	26	GA	17	9
71905	7	7176338	7176355	2	9	18	GT	25	7
71907	7	7179408	7179437	2	15	30	CA	11	11
71908	7	7182500	7182527	2	14	28	CA	17	5
71918	7	7205092	7205115	2	12	24	GT	15	9
71921	7	7205710	7205725	2	8	16	GT	22	6
71934	7	7222190	7222205	4	4	16	GGAG	26	3
71936	7	7223895	7223914	2	10	20	TG	20	3
71939	7	7227282	7227313	2	16	32	TG	9	12
71940	7	7227966	7227989	2	12	24	AC	21	6
71941	7	7228034	7228053	2	10	20	AC	23	10
71944	7	7231960	7231971	2	6	12	AC	31	4
71945	7	7232099	7232110	2	6	12	GT	21	3
71946	7	7232142	7232155	2	7	14	TG	25	6
71947	7	7232306	7232329	2	12	24	CA	15	12
71954	7	7233220	7233235	2	8	16	TG	15	4
71955	7	7233445	7233464	2	10	20	AC	19	8
71960	7	7236134	7236147	2	7	14	GT	24	3
71974	7	7257861	7257918	2	29	58	TG	9	12
71975	7	7260878	7260923	2	23	46	TG	7	7
71978	7	7263240	7263275	2	18	36	TG	12	13
71979	7	7263392	7263431	2	20	40	TG	9	11
71990	7	7278102	7278121	2	10	20	TC	13	5
71992	7	7280504	7280533	2	15	30	AC	10	9
71994	7	7283801	7283822	2	11	22	TG	22	4
71996	7	7287611	7287622	2	6	12	GA	20	3
71998	7	7288221	7288242	2	11	22	GT	20	10
71999	7	7288431	7288452	2	11	22	TC	15	10
72003	7	7290300	7290311	2	6	12	TG	18	3
72008	7	7296686	7296705	2	10	20	GT	10	4
72010	7	7299314	7299346	3	11	33	CTC	18	3
72011	7	7300371	7300390	2	10	20	CA	23	3
72015	7	7308174	7308191	2	9	18	CA	22	5
72016	7	7311053	7311078	2	13	26	AC	10	5
72018	7	7313987	7314008	2	11	22	GT	22	4
72021	7	7314454	7314485	2	16	32	TG	15	11
72023	7	7315738	7315757	2	10	20	AC	16	9
72024	7	7315991	7316004	2	7	14	GT	23	5
72026	7	7319381	7319396	2	8	16	GT	22	7
72028	7	7324625	7324638	2	7	14	TC	21	3
72029	7	7325951	7325966	2	8	16	AT	10	7
72030	7	7327197	7327216	2	10	20	CA	27	12
72031	7	7327447	7327470	2	12	24	TG	13	10
72032	7	7327584	7327595	2	6	12	CA	19	4
72034	7	7328033	7328064	2	16	32	TG	14	9

72042	7	7346633	7346650	2	9	18	GA	13	7
72046	7	7355954	7355965	2	6	12	GT	25	5
72050	7	7365337	7365352	2	8	16	CA	17	4
72052	7	7372916	7372947	2	16	32	CA	11	10
72058	7	7380862	7380877	2	8	16	TC	21	5
72081	7	7405567	7405582	2	8	16	AC	25	7
72082	7	7405734	7405745	2	6	12	TG	26	3
72095	7	7429119	7429152	2	17	34	CA	7	13
72096	7	7430838	7430853	2	8	16	TG	28	4
72097	7	7433690	7433703	2	7	14	CA	17	7
72102	7	7435964	7435977	2	7	14	AC	26	3
72103	7	7439812	7439835	2	12	24	AC	14	12
72104	7	7446489	7446502	2	7	14	CA	9	4
72106	7	7446879	7446898	4	5	20	AAAT	20	6
72117	7	7468778	7468795	2	9	18	TG	21	8
72118	7	7469709	7469728	2	10	20	CA	20	11
72121	7	7480867	7480892	2	13	26	GT	11	10
72124	7	7483595	7483612	2	9	18	AC	15	7
72131	7	7502729	7502752	2	12	24	GA	11	6
72135	7	7511932	7511947	2	8	16	GT	21	10
72146	7	7532279	7532290	2	6	12	GT	27	6
72147	7	7532597	7532620	2	12	24	GT	20	8
72148	7	7537180	7537195	2	8	16	GT	20	12
72157	7	7546090	7546109	2	10	20	CA	15	4
72159	7	7546257	7546276	2	10	20	AC	16	7
72164	7	7549439	7549460	2	11	22	CA	20	6
72167	7	7549958	7549972	3	5	15	AGG	22	3
72173	7	7556062	7556079	2	9	18	GA	10	3
72174	7	7557692	7557719	4	7	28	TGCC	28	5
72175	7	7558079	7558094	4	4	16	AGAC	30	3
72179	7	7567357	7567374	3	6	18	TCC	19	4
72184	7	7577487	7577504	3	6	18	GCA	17	5
72187	7	7579206	7579219	2	7	14	TG	17	3
72190	7	7587508	7587521	2	7	14	GT	20	13
72197	7	7597961	7597980	2	10	20	AC	15	6
72199	7	7601367	7601393	3	9	27	AGG	13	8
72203	7	7607872	7607885	2	7	14	CA	24	4
72206	7	7614155	7614180	2	13	26	TG	21	13
72208	7	7616955	7616966	2	6	12	TG	25	4
72210	7	7617369	7617384	4	4	16	ACGC	36	6
72212	7	7617808	7617821	2	7	14	AC	10	4
72215	7	7622292	7622311	2	10	20	CA	15	5
72217	7	7623958	7623973	2	8	16	TG	19	6
72218	7	7624125	7624142	3	6	18	GGA	13	7
72220	7	7624698	7624711	2	7	14	AG	22	4
72230	7	7637137	7637148	2	6	12	AC	14	4
72231	7	7638989	7639004	2	8	16	TA	13	3
72233	7	7640944	7640957	2	7	14	TG	8	5
72234	7	7640964	7640977	2	7	14	TG	11	3
72235	7	7641073	7641096	2	12	24	CA	16	3
72237	7	7643469	7643498	2	15	30	CT	10	6

72240	7	7650249	7650288	2	20	40	AC	8	7
72242	7	7653675	7653700	2	13	26	AC	17	10
72243	7	7653758	7653769	2	6	12	CA	21	3
72244	7	7653953	7653964	2	6	12	CA	20	5
72247	7	7660930	7660957	2	14	28	AC	18	11
72248	7	7661142	7661167	2	13	26	GT	21	11
72257	7	7672462	7672477	2	8	16	GT	12	3
72259	7	7674082	7674093	2	6	12	CT	19	3
72262	7	7676256	7676281	2	13	26	GT	7	6
72264	7	7676783	7676820	2	19	38	TG	20	12
72266	7	7679686	7679713	2	14	28	TG	15	8
72268	7	7681841	7681860	2	10	20	CT	20	5
72273	7	7694625	7694651	3	9	27	TGA	19	10
72283	7	7703255	7703278	2	12	24	CA	17	14
72288	7	7705874	7705887	2	7	14	TC	23	3
72292	7	7707786	7707805	2	10	20	CA	9	7
72293	7	7707909	7707928	2	10	20	CA	20	13
72307	7	7725319	7725334	2	8	16	CA	27	7
72309	7	7728935	7728962	2	14	28	CA	14	7
72310	7	7729068	7729103	2	18	36	GT	9	10
72311	7	7729213	7729224	2	6	12	TG	9	3
72313	7	7732049	7732076	2	14	28	GT	10	7
72314	7	7732313	7732324	2	6	12	GT	24	5
72315	7	7732470	7732481	2	6	12	TG	25	9
72316	7	7732482	7732493	2	6	12	AG	18	3
72319	7	7739193	7739222	2	15	30	CA	10	11
72321	7	7739577	7739598	2	11	22	GT	14	8
72322	7	7739785	7739820	2	18	36	TG	6	4
72323	7	7740343	7740354	2	6	12	AC	18	4
72324	7	7740466	7740477	2	6	12	CA	33	3
72325	7	7741462	7741491	2	15	30	GT	20	13
72326	7	7741902	7741929	2	14	28	TG	19	8
72327	7	7748729	7748748	2	10	20	TG	23	7
72328	7	7750537	7750558	2	11	22	GT	15	5
72329	7	7750890	7750919	2	15	30	AC	18	8
72334	7	7757475	7757508	2	17	34	TG	17	10
72335	7	7757618	7757633	2	8	16	CA	25	3
72336	7	7759543	7759564	2	11	22	AC	12	6
72337	7	7759723	7759746	2	12	24	AC	22	11
72338	7	7759895	7759912	2	9	18	TG	17	6
72341	7	7762743	7762760	2	9	18	AC	22	6
72342	7	7765332	7765347	2	8	16	AC	25	9
72344	7	7767941	7767960	2	10	20	AC	7	8
72345	7	7768105	7768124	2	10	20	TG	12	3
72346	7	7768527	7768544	2	9	18	GT	21	6
72347	7	7768597	7768612	2	8	16	GT	20	3
72348	7	7768715	7768728	2	7	14	GT	30	4
72350	7	7775038	7775073	2	18	36	CA	19	12
72351	7	7776253	7776278	2	13	26	GT	15	12
72352	7	7776603	7776616	2	7	14	AG	22	3
72354	7	7777234	7777263	2	15	30	CA	22	14

72355	7	7777399	7777418	2	10	20	CA	18	6
72361	7	7784388	7784403	2	8	16	AC	24	6
72366	7	7786005	7786036	2	16	32	TG	20	15
72367	7	7788136	7788151	2	8	16	TG	24	3
72368	7	7790075	7790094	2	10	20	CA	19	6
72373	7	7797076	7797091	2	8	16	CA	28	4
72376	7	7797384	7797407	2	12	24	TG	22	15
72377	7	7799506	7799529	2	12	24	CA	15	6
72378	7	7800955	7800970	2	8	16	AC	21	3
72380	7	7804758	7804773	2	8	16	CA	18	5
72383	7	7805083	7805102	2	10	20	AC	8	4
72384	7	7805256	7805271	2	8	16	AC	16	3
72385	7	7806620	7806637	2	9	18	GT	31	12
72387	7	7807052	7807073	2	11	22	AG	20	5
72388	7	7807292	7807307	2	8	16	CA	23	4
72396	7	7825357	7825372	4	4	16	AAGT	22	3
72398	7	7834472	7834519	4	12	48	TATC	13	9
72401	7	7837078	7837098	3	7	21	GAT	20	7
72405	7	7842877	7842900	2	12	24	CA	18	7
72406	7	7843633	7843646	2	7	14	AC	22	7
72411	7	7859685	7859716	2	16	32	AC	12	9
72412	7	7859792	7859807	2	8	16	GT	20	7
72422	7	7877298	7877312	3	5	15	GCA	32	6
72423	7	7877656	7877669	2	7	14	AT	20	5
72424	7	7877823	7877834	2	6	12	AC	20	7
72425	7	7877836	7877851	2	8	16	CA	15	5
72427	7	7881516	7881553	2	19	38	TG	7	8
72433	7	7891988	7892023	2	18	36	AC	9	12
72435	7	7901820	7901834	3	5	15	TTG	18	3
72438	7	7905677	7905690	2	7	14	GT	20	3
72439	7	7906303	7906318	2	8	16	TG	29	5
72443	7	7913516	7913543	4	7	28	AAAG	14	5
72444	7	7914177	7914191	3	5	15	TGC	29	3
72446	7	7927909	7927936	4	7	28	AATA	20	8
72447	7	7934959	7934978	2	10	20	TG	20	6
72448	7	7936293	7936336	2	22	44	TG	18	7
72462	7	7981704	7981721	2	9	18	CT	16	6
72464	7	7989753	7989764	2	6	12	CA	28	4
72467	7	7993023	7993040	3	6	18	CTC	20	3
72469	7	7994046	7994073	2	14	28	GT	13	15
72472	7	7994522	7994579	2	29	58	CA	17	15
72473	7	7995965	7995979	3	5	15	AAT	30	4
72474	7	8000105	8000118	2	7	14	GA	24	5
72478	7	8005688	8005707	4	5	20	AGAT	23	7
72486	7	8013100	8013131	2	16	32	CA	11	5
72490	7	8020937	8020956	2	10	20	CT	9	8
72491	7	8022038	8022051	2	7	14	GT	22	3
72493	7	8024201	8024232	2	16	32	AC	9	8
72495	7	8024874	8024897	2	12	24	GT	10	7
72496	7	8025203	8025214	2	6	12	GC	12	5
72498	7	8025765	8025792	2	14	28	TG	7	7

72499	7	8025820	8025837	2	9	18	TG	15	4
72500	7	8026054	8026067	2	7	14	GT	22	4
72504	7	8048080	8048113	2	17	34	GT	7	5
72506	7	8054131	8054146	4	4	16	CTTT	22	5
72507	7	8054310	8054330	3	7	21	GGA	15	3
72513	7	8062724	8062744	3	7	21	GAA	14	3
72514	7	8063032	8063057	2	13	26	AC	16	11
72515	7	8063160	8063171	2	6	12	GT	25	7
72517	7	8068231	8068244	2	7	14	CA	23	3
72518	7	8068442	8068469	2	14	28	GA	8	5
72519	7	8072252	8072267	2	8	16	CA	24	4
72527	7	8099935	8099949	3	5	15	TTA	6	3
72532	7	8105843	8105882	4	10	40	TTTC	9	6
72534	7	8106473	8106488	2	8	16	GA	21	3
72535	7	8106959	8106970	2	6	12	AC	19	4
72536	7	8106973	8106986	2	7	14	AC	22	6
72537	7	8107471	8107488	2	9	18	AC	17	6
72539	7	8108159	8108176	2	9	18	TG	13	8
72540	7	8108396	8108421	2	13	26	TC	28	8
72541	7	8112917	8112928	2	6	12	AC	26	3
72560	7	8133984	8134003	2	10	20	CT	14	12
72564	7	8137252	8137267	2	8	16	GT	20	3
72568	7	8144074	8144105	2	16	32	CA	6	6
72573	7	8153063	8153089	3	9	27	CCT	15	7
72575	7	8176385	8176398	2	7	14	TG	23	6
72580	7	8182939	8182964	2	13	26	TC	11	12
72585	7	8183402	8183429	2	14	28	TG	18	10
72588	7	8195574	8195594	3	7	21	CAG	30	6
72591	7	8210522	8210539	2	9	18	AG	19	8
72596	7	8218868	8218881	2	7	14	GT	25	3
72600	7	8221767	8221778	2	6	12	TG	24	4
72601	7	8221888	8221903	4	4	16	TGAA	32	3
72602	7	8225318	8225332	3	5	15	CAT	31	4
72604	7	8239456	8239477	2	11	22	GT	28	5
72605	7	8240025	8240036	2	6	12	TC	24	4
72607	7	8240126	8240161	2	18	36	GT	9	9
72608	7	8240745	8240772	2	14	28	GT	13	9
72610	7	8245538	8245559	2	11	22	GT	19	13
72614	7	8251393	8251406	2	7	14	AT	8	3
72615	7	8252317	8252346	2	15	30	AC	13	11
72617	7	8256994	8257008	3	5	15	GGA	28	3
72618	7	8257322	8257345	4	6	24	ATGT	14	5
72620	7	8259183	8259196	2	7	14	GT	24	8
72621	7	8259690	8259701	2	6	12	GA	22	5
72626	7	8269521	8269534	2	7	14	AC	30	5
72628	7	8270421	8270440	2	10	20	CA	22	4
72631	7	8272006	8272055	2	25	50	TC	7	9
72634	7	8278357	8278394	2	19	38	TG	12	12
72636	7	8280984	8281011	2	14	28	TG	20	14
72637	7	8281171	8281190	2	10	20	AC	21	5
72641	7	8284736	8284747	2	6	12	TG	29	3

72643	7	8285516	8285531	2	8	16	CA	31	5
72646	7	8290578	8290593	2	8	16	GT	23	5
72647	7	8291663	8291680	2	9	18	CT	26	4
72650	7	8306914	8306927	2	7	14	TC	19	3
72651	7	8311026	8311040	3	5	15	CCT	12	4
72653	7	8313275	8313306	4	8	32	GATG	19	11
72655	7	8316041	8316058	2	9	18	CT	24	12
72658	7	8324597	8324610	2	7	14	AC	21	11
72659	7	8326987	8327018	2	16	32	CA	19	11
72665	7	8331196	8331219	2	12	24	AC	19	14
72666	7	8331769	8331784	2	8	16	TC	17	11
72667	7	8332976	8332990	3	5	15	GGT	25	6
72668	7	8333414	8333433	2	10	20	TC	22	5
72682	7	8354732	8354745	2	7	14	CA	21	6
72692	7	8369392	8369405	2	7	14	GA	20	3
72693	7	8371000	8371013	2	7	14	CA	21	4
72694	7	8371096	8371107	2	6	12	AC	17	3
72698	7	8392712	8392735	2	12	24	AG	23	5
72717	7	8435344	8435387	2	22	44	AC	8	9
72722	7	8438466	8438483	2	9	18	AC	20	3
72728	7	8443387	8443410	2	12	24	TG	22	12
72729	7	8445919	8445946	2	14	28	AC	19	8
72732	7	8453869	8453898	2	15	30	TG	19	15
72736	7	8468088	8468115	2	14	28	GT	14	7
72737	7	8470379	8470400	2	11	22	TG	16	3
72750	7	8496877	8496892	2	8	16	GT	13	6
72753	7	8498469	8498506	2	19	38	CA	13	10
72755	7	8500922	8500933	2	6	12	GT	27	5
72766	7	8530833	8530852	2	10	20	CA	24	8
72767	7	8533488	8533499	2	6	12	CT	17	4
72769	7	8533973	8533986	2	7	14	TG	24	4
72776	7	8552305	8552360	4	14	56	CTTT	15	12
72780	7	8560677	8560688	2	6	12	AC	21	3
72781	7	8561065	8561094	2	15	30	TG	20	12
72783	7	8561302	8561313	2	6	12	CA	20	8
72784	7	8561534	8561545	2	6	12	CT	25	3
72792	7	8583215	8583248	2	17	34	AC	11	8
72795	7	8595512	8595557	2	23	46	CA	9	8
72797	7	8596909	8596924	2	8	16	CA	22	4
72799	7	8598096	8598123	4	7	28	ATCC	27	10
72800	7	8598302	8598317	4	4	16	TCCA	25	3
72802	7	8601958	8601981	2	12	24	TG	13	14
72803	7	8605203	8605220	2	9	18	AC	22	9
72805	7	8607173	8607199	3	9	27	TCC	7	4
72812	7	8616667	8616684	3	6	18	GAG	24	4
72813	7	8617849	8617886	2	19	38	CA	12	12
72814	7	8618089	8618104	2	8	16	GT	19	5
72815	7	8618518	8618541	2	12	24	CA	13	5
72816	7	8618602	8618625	2	12	24	AC	13	7
72821	7	8626986	8627001	2	8	16	AT	17	7
72824	7	8630809	8630820	2	6	12	AC	26	3

72825	7	8631646	8631673	2	14	28	CA	9	8
72830	7	8646201	8646221	3	7	21	GCA	24	6
72833	7	8649205	8649220	4	4	16	AACT	19	3
72848	7	8677845	8677858	2	7	14	GT	8	3
72850	7	8678021	8678034	2	7	14	CA	22	6
72851	7	8678110	8678121	2	6	12	AC	27	3
72858	7	8699960	8699973	2	7	14	GA	30	3
72859	7	8701835	8701848	2	7	14	TG	25	4
72865	7	8706848	8706863	2	8	16	GT	19	8
72869	7	8711480	8711497	2	9	18	GT	26	7
72870	7	8711761	8711774	2	7	14	AC	7	4
72875	7	8716118	8716151	2	17	34	CA	6	8
72877	7	8718422	8718435	2	7	14	AC	19	6
72883	7	8725954	8725977	4	6	24	CAGA	33	4
72890	7	8736459	8736486	4	7	28	TGAT	30	17
72891	7	8737094	8737115	2	11	22	GT	21	11
72892	7	8740214	8740229	2	8	16	GT	22	11
72903	7	8751520	8751535	2	8	16	GA	25	3
72905	7	8752210	8752227	2	9	18	AC	16	8
72919	7	8772955	8772968	2	7	14	AC	28	4
72927	7	8780987	8781004	2	9	18	AC	8	4
72929	7	8786247	8786258	2	6	12	GT	26	3
72934	7	8789147	8789166	2	10	20	AT	6	6
72938	7	8796178	8796195	2	9	18	AC	22	4
72942	7	8799401	8799416	2	8	16	CA	25	15
72945	7	8803063	8803074	2	6	12	TG	18	4
72946	7	8803076	8803087	2	6	12	TG	16	4
72950	7	8808781	8808796	2	8	16	TG	22	7
72951	7	8808941	8808998	2	29	58	AC	6	7
72953	7	8816311	8816330	2	10	20	TG	26	4
72957	7	8820590	8820609	2	10	20	GT	6	6
72958	7	8820735	8820748	2	7	14	AC	18	3
72959	7	8820771	8820800	2	15	30	CA	19	8
72960	7	8825708	8825747	2	20	40	TG	6	7
72962	7	8827252	8827265	2	7	14	AC	22	3
72964	7	8827437	8827460	2	12	24	AC	13	9
72971	7	8837788	8837799	2	6	12	TG	10	4
72972	7	8837933	8837956	2	12	24	AC	19	6
72973	7	8838046	8838061	2	8	16	TG	23	6
72975	7	8845335	8845362	2	14	28	CA	19	15
72984	7	8850767	8850780	2	7	14	TG	23	3
72987	7	8853114	8853133	2	10	20	CA	15	10
72991	7	8856471	8856492	2	11	22	GT	15	7
72994	7	8859202	8859227	2	13	26	TG	17	9
72995	7	8859371	8859386	2	8	16	CA	19	9
72997	7	8859467	8859478	2	6	12	CA	26	4
73000	7	8863553	8863566	2	7	14	GT	23	4
73001	7	8864168	8864203	2	18	36	AC	25	17
73003	7	8864959	8864984	2	13	26	GT	11	11
73008	7	8868348	8868365	2	9	18	GA	20	3
73014	7	8874062	8874089	2	14	28	GA	21	9

73015	7	8874472	8874491	2	10	20	GA	8	5
73016	7	8884772	8884791	2	10	20	CT	27	9
73023	7	8893646	8893673	2	14	28	AC	12	9
73025	7	8897820	8897833	2	7	14	GT	30	6
73026	7	8898449	8898474	2	13	26	AC	18	9
73031	7	8899470	8899483	2	7	14	AC	22	6
73033	7	8899878	8899895	3	6	18	GCT	16	3
73036	7	8901144	8901183	4	10	40	AGAA	7	8
73039	7	8903168	8903183	4	4	16	AAAT	23	3
73040	7	8907067	8907080	2	7	14	CA	24	4
73045	7	8912273	8912288	2	8	16	AG	14	9
73050	7	8916349	8916364	2	8	16	CA	25	9
73053	7	8917914	8917931	2	9	18	TG	21	7
73054	7	8918071	8918082	2	6	12	CA	27	7
73055	7	8918970	8918996	3	9	27	CTT	16	5
73057	7	8921227	8921252	2	13	26	CA	8	9
73059	7	8924677	8924698	2	11	22	TG	15	7
73060	7	8926965	8926982	2	9	18	CT	14	6
73061	7	8926985	8926998	2	7	14	CT	14	6
73062	7	8927482	8927496	3	5	15	AGC	27	6
73065	7	8931131	8931144	2	7	14	CA	15	5
73066	7	8932503	8932518	4	4	16	TTTG	15	11
73069	7	8941530	8941551	2	11	22	GT	21	8
73070	7	8941719	8941738	2	10	20	AC	25	7
73071	7	8941890	8941909	4	5	20	CCTT	28	4
73072	7	8942325	8942348	2	12	24	CA	14	15
73075	7	8944968	8944991	3	8	24	CCT	26	7
73078	7	8947253	8947268	2	8	16	TG	29	4
73079	7	8947378	8947397	2	10	20	CA	14	5
73080	7	8948038	8948061	2	12	24	GT	12	7
73085	7	8957889	8957908	2	10	20	AC	21	12
73086	7	8958565	8958582	2	9	18	AC	22	6
73088	7	8959649	8959669	3	7	21	CCT	18	3
73090	7	8960059	8960085	3	9	27	AAT	7	4
73091	7	8962042	8962073	2	16	32	GT	27	7
73092	7	8962499	8962512	2	7	14	GT	13	9
73094	7	8962896	8962907	2	6	12	TA	15	3
73099	7	8967359	8967374	4	4	16	AAAT	19	6
73101	7	8967659	8967672	2	7	14	AC	14	3
73102	7	8969273	8969286	2	7	14	CT	25	4
73109	7	8974781	8974794	2	7	14	GT	22	6
73112	7	8982631	8982650	2	10	20	CA	23	10
73115	7	8983551	8983566	2	8	16	TG	24	6
73122	7	9000709	9000732	3	8	24	CAT	16	3
73123	7	9000875	9000898	2	12	24	CA	19	8
73129	7	9008626	9008641	2	8	16	GA	22	7
73133	7	9013546	9013557	2	6	12	AG	11	3
73134	7	9014205	9014222	2	9	18	CA	13	3
73136	7	9018405	9018426	2	11	22	CA	20	12
73143	7	9031136	9031161	2	13	26	CA	19	13
73147	7	9034999	9035013	3	5	15	TCC	10	3

73149	7	9035759	9035782	2	12	24	TG	14	8
73157	7	9042612	9042627	4	4	16	GCAC	14	4
73158	7	9042683	9042700	2	9	18	CA	12	10
73159	7	9042703	9042716	2	7	14	CA	15	7
73180	7	9051897	9051914	3	6	18	CTC	24	5
73181	7	9053188	9053203	2	8	16	TC	10	4
73182	7	9054443	9054466	2	12	24	TG	12	9
73184	7	9057185	9057200	2	8	16	CA	16	6
73189	7	9065907	9065930	2	12	24	TG	11	6
73192	7	9067290	9067309	4	5	20	TCCC	9	3
73194	7	9072052	9072071	2	10	20	GT	23	5
73199	7	9074957	9074992	2	18	36	GT	13	12
73203	7	9075745	9075768	2	12	24	AC	9	7
73204	7	9075948	9075961	2	7	14	GA	22	3
73207	7	9078753	9078780	2	14	28	GT	15	9
73208	7	9080589	9080612	2	12	24	TG	30	8
73212	7	9085161	9085176	2	8	16	TA	12	3
73214	7	9100143	9100154	2	6	12	AT	12	6
73215	7	9100634	9100651	3	6	18	GAA	10	5
73217	7	9101790	9101817	2	14	28	GT	14	15
73219	7	9102028	9102055	2	14	28	AG	9	5
73226	7	9111036	9111075	2	20	40	CA	8	8
73245	7	9143554	9143573	2	10	20	TC	12	3
73251	7	9148075	9148092	2	9	18	CA	11	4
73266	7	9163664	9163689	2	13	26	AG	10	7
73267	7	9164640	9164677	2	19	38	TG	6	5
73270	7	9165627	9165642	2	8	16	CT	7	4
73271	7	9165895	9165908	2	7	14	AC	8	9
73278	7	9172386	9172397	2	6	12	TG	31	5
73279	7	9172602	9172619	2	9	18	AC	17	3
73280	7	9172641	9172652	2	6	12	CA	29	7
73281	7	9172654	9172665	2	6	12	AC	27	5
73282	7	9176925	9176944	2	10	20	CA	21	6
73287	7	9185990	9186015	2	13	26	TG	13	5
73288	7	9186057	9186106	2	25	50	AC	7	8
73291	7	9186691	9186706	2	8	16	TG	20	4
73294	7	9191858	9191873	2	8	16	CA	25	4
73295	7	9192708	9192729	2	11	22	CT	13	4
73298	7	9199058	9199075	2	9	18	AC	20	6
73305	7	9205389	9205400	2	6	12	CA	23	5
73306	7	9205460	9205485	2	13	26	AC	14	4
73310	7	9208085	9208096	2	6	12	CA	30	3
73312	7	9209706	9209731	2	13	26	GT	13	9
73318	7	9213787	9213800	2	7	14	GT	26	3
73320	7	9214506	9214527	2	11	22	AC	14	6
73324	7	9218829	9218846	2	9	18	CA	21	9
73325	7	9219012	9219023	2	6	12	AC	20	4
73326	7	9222212	9222229	2	9	18	AC	26	5
73339	7	9234359	9234394	2	18	36	AC	6	6
73347	7	9239206	9239221	2	8	16	TG	26	3
73348	7	9239350	9239371	2	11	22	AC	19	7

73350	7	9239403	9239422	2	10	20	CA	26	4
73351	7	9239644	9239661	2	9	18	TC	29	7
73352	7	9239759	9239782	3	8	24	GAA	18	6
73354	7	9240656	9240676	3	7	21	AAG	16	3
73360	7	9243337	9243351	3	5	15	CTT	9	6
73366	7	9249611	9249628	2	9	18	AG	11	3
73367	7	9249658	9249685	2	14	28	AG	6	8
73368	7	9249797	9249814	2	9	18	GT	19	7
73371	7	9255137	9255150	2	7	14	GT	23	5
73372	7	9257634	9257651	2	9	18	GT	16	8
73373	7	9261001	9261024	2	12	24	GT	21	7
73374	7	9264338	9264353	2	8	16	TG	17	5
73378	7	9267294	9267305	2	6	12	TG	16	5
73379	7	9271100	9271123	2	12	24	TG	10	8
73380	7	9272603	9272616	2	7	14	TG	27	3
73381	7	9275920	9275931	2	6	12	AC	29	3
73383	7	9280825	9280848	2	12	24	GT	12	8
73387	7	9289161	9289182	2	11	22	AG	26	5
73390	7	9293617	9293638	2	11	22	AC	16	4
73392	7	9297730	9297744	3	5	15	TCT	27	4
73394	7	9300912	9300927	2	8	16	AC	20	3
73395	7	9301112	9301123	2	6	12	TG	27	5
73396	7	9302939	9302962	3	8	24	AGG	24	4
73406	7	9312557	9312568	2	6	12	CT	21	3
73413	7	9315542	9315561	2	10	20	AC	19	11
73419	7	9319724	9319737	2	7	14	TA	11	3
73420	7	9320284	9320315	2	16	32	CA	13	7
73430	7	9329246	9329263	2	9	18	TG	27	8
73431	7	9329539	9329578	2	20	40	TG	9	9
73434	7	9335030	9335047	2	9	18	TG	17	10
73435	7	9338223	9338240	2	9	18	AG	16	4
73440	7	9342482	9342519	2	19	38	AC	14	11
73441	7	9342853	9342870	3	6	18	AGG	26	5
73450	7	9353588	9353605	2	9	18	GT	15	14
73451	7	9353926	9353957	2	16	32	CA	6	7
73452	7	9357735	9357762	2	14	28	GT	15	11
73456	7	9360957	9360992	2	18	36	GT	11	10
73461	7	9365620	9365631	2	6	12	CA	22	5
73462	7	9365819	9365832	2	7	14	AC	22	3
73469	7	9372583	9372598	2	8	16	TC	23	8
73473	7	9373165	9373176	2	6	12	GT	32	3
73474	7	9373688	9373713	2	13	26	TG	17	8
73477	7	9375723	9375734	2	6	12	TG	21	6
73478	7	9377171	9377196	2	13	26	CA	12	4
73482	7	9379523	9379544	2	11	22	AG	12	3
73486	7	9382594	9382607	2	7	14	AG	13	4
73489	7	9383081	9383098	3	6	18	AGA	17	4
73490	7	9383907	9383918	2	6	12	GA	17	4
73491	7	9383923	9383938	2	8	16	GA	10	3
73509	7	9404825	9404842	2	9	18	CA	10	7
73531	7	9430614	9430637	3	8	24	TCC	15	3

73535	7	9433001	9433014	2	7	14	CA	24	3
73536	7	9433161	9433174	2	7	14	AC	22	6
73538	7	9446761	9446778	3	6	18	AGC	33	3
73542	7	9451713	9451734	2	11	22	AC	22	8
73543	7	9456157	9456174	3	6	18	CCT	15	4
73547	7	9458414	9458441	2	14	28	CA	13	7
73548	7	9458686	9458718	3	11	33	CCT	17	5
73550	7	9459212	9459265	2	27	54	CA	10	12
73556	7	9461555	9461578	2	12	24	GT	22	12
73557	7	9461881	9461920	2	20	40	CA	9	8
73559	7	9463258	9463291	2	17	34	CA	12	11
73567	7	9474418	9474433	2	8	16	GT	15	8
73569	7	9477172	9477195	2	12	24	GA	24	7
73570	7	9477450	9477483	2	17	34	TG	7	8
73573	7	9481131	9481160	2	15	30	TG	7	8
73574	7	9481358	9481383	2	13	26	AC	10	10
73575	7	9481524	9481541	2	9	18	CA	23	4
73577	7	9482284	9482303	4	5	20	TCCT	29	3
73579	7	9485392	9485406	3	5	15	TAA	8	8
73580	7	9486848	9486862	3	5	15	AGG	8	6
73583	7	9488754	9488781	2	14	28	AC	17	7
73588	7	9490131	9490146	2	8	16	CA	17	3
73590	7	9490511	9490530	2	10	20	TG	16	9
73601	7	9512859	9512885	3	9	27	TGT	25	9
73604	7	9521468	9521491	3	8	24	CTG	31	5
73605	7	9524172	9524207	2	18	36	AC	10	9
73607	7	9525615	9525642	2	14	28	GT	11	11
73610	7	9530649	9530664	4	4	16	ACAG	28	4
73614	7	9539638	9539653	2	8	16	CT	21	3
73620	7	9555175	9555194	2	10	20	AC	23	3
73622	7	9562816	9562849	2	17	34	CA	19	10
73630	7	9580866	9580886	3	7	21	CCT	27	5
73634	7	9592901	9592912	2	6	12	CG	22	9
73635	7	9594724	9594738	3	5	15	GAA	27	3
73637	7	9597886	9597919	2	17	34	TG	7	7
73640	7	9607292	9607313	2	11	22	AC	20	4
73642	7	9609016	9609037	2	11	22	CA	12	9
73643	7	9609872	9609898	3	9	27	TTC	14	7
73645	7	9611344	9611365	2	11	22	TG	22	8
73653	7	9624618	9624641	2	12	24	TG	21	9
73666	7	9643132	9643155	2	12	24	CT	14	6
73670	7	9649058	9649079	2	11	22	CA	17	5
73671	7	9651107	9651130	2	12	24	TG	14	9
73674	7	9657349	9657360	2	6	12	TG	21	5
73675	7	9657794	9657815	2	11	22	GT	11	9
73676	7	9658274	9658305	2	16	32	AC	23	11
73680	7	9674747	9674774	2	14	28	GT	7	6
73683	7	9676963	9676986	2	12	24	GT	17	6
73685	7	9680018	9680031	2	7	14	TG	21	3
73687	7	9681277	9681309	3	11	33	GGA	21	11
73696	7	9717197	9717226	2	15	30	TG	13	14

73701	7	9724181	9724196	2	8	16	GT	18	3
73703	7	9727471	9727502	2	16	32	CA	23	8
73704	7	9729746	9729763	2	9	18	AC	13	5
73709	7	9731183	9731197	3	5	15	CCT	30	6
73714	7	9738593	9738614	2	11	22	CA	16	10
73717	7	9741635	9741668	2	17	34	CA	9	7
73718	7	9742806	9742823	2	9	18	AC	23	4
73723	7	9751329	9751350	2	11	22	TG	16	7
73727	7	9763446	9763469	2	12	24	TG	16	8
73731	7	9773463	9773480	2	9	18	CA	24	12
73733	7	9775864	9775875	2	6	12	CA	10	5
73734	7	9785151	9785168	2	9	18	CA	24	6
73736	7	9789477	9789500	2	12	24	GT	8	8
73738	7	9791683	9791694	2	6	12	AG	30	3
73746	7	9819473	9819510	2	19	38	GT	15	16
73747	7	9819792	9819805	2	7	14	CA	13	4
73750	7	9824675	9824690	2	8	16	GT	27	3
73752	7	9824933	9824952	2	10	20	CA	19	9
73754	7	9827732	9827747	2	8	16	CA	26	6
73756	7	9829863	9829886	2	12	24	CA	16	5
73757	7	9830162	9830181	2	10	20	CA	17	6
73760	7	9831167	9831186	2	10	20	GA	10	5
73773	7	9854168	9854183	2	8	16	CA	24	3
73778	7	9872199	9872222	2	12	24	CA	13	6
73781	7	9873376	9873391	2	8	16	CA	6	4
73783	7	9878731	9878746	2	8	16	AC	20	11
73784	7	9879345	9879362	2	9	18	TG	24	8
73785	7	9879442	9879473	2	16	32	CA	14	8
73786	7	9882429	9882456	2	14	28	CA	20	7
73787	7	9883728	9883763	2	18	36	CA	7	9
73789	7	9887512	9887543	2	16	32	TG	16	12
73790	7	9887686	9887715	2	15	30	AC	15	11
73792	7	9893280	9893305	2	13	26	GT	23	8
73799	7	9895466	9895483	2	9	18	CA	22	4
73800	7	9896814	9896833	2	10	20	TC	8	7
73802	7	9897403	9897424	2	11	22	CA	8	5
73812	7	9912133	9912146	2	7	14	GT	27	4
73813	7	9912280	9912303	2	12	24	TG	17	9
73815	7	9918547	9918576	2	15	30	TG	12	10
73816	7	9918889	9918900	2	6	12	TG	24	4
73817	7	9920260	9920299	4	10	40	GATA	16	9
73829	7	9942234	9942265	2	16	32	TG	20	7
73830	7	9943964	9944003	2	20	40	TG	8	10
73834	7	9953135	9953174	4	10	40	TTTC	17	7
73837	7	9961396	9961413	2	9	18	AC	19	11
73838	7	9961443	9961462	2	10	20	AC	22	7
73839	7	9962139	9962158	2	10	20	TC	17	3
73841	7	9972391	9972410	4	5	20	AAAT	32	3
73845	7	9977016	9977031	4	4	16	GTGC	6	5
73847	7	9978054	9978077	4	6	24	TATC	23	8
73852	7	9993518	9993543	2	13	26	AC	16	8

73855	7	9997725	9997766	2	21	42	AG	7	6
73857	7	9998819	9998842	2	12	24	GT	6	10
73858	7	10001568	10001597	2	15	30	TG	23	15
73861	7	10010570	10010593	2	12	24	CA	19	7
73871	7	10027901	10027936	2	18	36	TG	7	10
73872	7	10031303	10031316	2	7	14	AC	31	3
73873	7	10032876	10032890	3	5	15	GCA	24	8
73875	7	10034691	10034716	2	13	26	TG	12	8
73876	7	10035134	10035151	2	9	18	AC	23	3
73877	7	10035454	10035469	2	8	16	CA	18	4
73881	7	10037107	10037118	2	6	12	CT	12	3
73882	7	10037253	10037274	2	11	22	AC	10	14
73883	7	10037431	10037448	2	9	18	TG	18	4
73890	7	10048048	10048077	3	10	30	TTG	20	5
73894	7	10054388	10054423	2	18	36	GT	17	15
73895	7	10054626	10054653	2	14	28	CA	17	10
73900	7	10058832	10058847	2	8	16	AC	19	5
73905	7	10081608	10081627	2	10	20	CA	11	7
73909	7	10090880	10090903	3	8	24	TGA	27	4
73911	7	10096324	10096338	3	5	15	ATT	23	3
73912	7	10096975	10096989	3	5	15	AAC	33	4
73920	7	10113778	10113793	2	8	16	TG	17	13
73926	7	10123436	10123449	2	7	14	GT	22	3
73929	7	10124197	10124208	2	6	12	CA	29	6
73934	7	10132774	10132785	2	6	12	GT	28	8
73938	7	10138106	10138135	2	15	30	AG	6	4
73948	7	10148935	10148964	2	15	30	GT	6	7
73954	7	10163997	10164011	3	5	15	GAG	31	4
73956	7	10170132	10170143	2	6	12	GT	33	3
73958	7	10174627	10174659	3	11	33	TGA	27	11
73961	7	10176169	10176182	2	7	14	AC	27	5
73963	7	10179236	10179251	2	8	16	AC	28	3
73964	7	10180300	10180311	2	6	12	GT	33	3
73966	7	10182250	10182271	2	11	22	AC	19	14
73968	7	10185811	10185822	2	6	12	CA	9	6
73972	7	10188838	10188851	2	7	14	GT	21	8
73978	7	10192463	10192484	2	11	22	CA	23	9
73979	7	10194305	10194319	3	5	15	GCT	20	3
73980	7	10194552	10194571	4	5	20	AAGG	31	4
73981	7	10195056	10195089	2	17	34	GT	13	13
73986	7	10201726	10201741	2	8	16	GA	30	3
73988	7	10203907	10203920	2	7	14	GA	9	6
73994	7	10210182	10210197	2	8	16	AC	28	4
73996	7	10212458	10212475	2	9	18	CA	16	8
73997	7	10212606	10212625	2	10	20	CA	17	14
74000	7	10215960	10215983	4	6	24	CAGG	29	5
74002	7	10216714	10216737	2	12	24	AC	9	11
74003	7	10216864	10216877	2	7	14	AC	23	3
74004	7	10217141	10217192	2	26	52	CA	10	10
74005	7	10217940	10217957	2	9	18	TG	23	3
74008	7	10231519	10231538	2	10	20	TC	8	7

74010	7	10239140	10239155	4	4	16	TTAT	25	11
74012	7	10242077	10242094	3	6	18	TTG	12	3
74016	7	10248458	10248475	2	9	18	GT	28	8
74018	7	10251121	10251132	2	6	12	TG	18	4
74019	7	10259162	10259215	2	27	54	TG	6	9
74028	7	10276963	10276982	2	10	20	TG	10	13
74033	7	10287986	10288003	3	6	18	CTG	28	7
74034	7	10288319	10288334	4	4	16	TATT	17	3
74037	7	10291558	10291585	2	14	28	TG	22	7
74040	7	10293408	10293429	2	11	22	TG	16	8
74041	7	10293901	10293918	2	9	18	TG	17	9
74043	7	10294205	10294224	2	10	20	GT	24	4
74044	7	10294374	10294389	2	8	16	TG	18	13
74045	7	10294558	10294571	2	7	14	AC	25	4
74046	7	10294697	10294708	2	6	12	GT	21	5
74048	7	10296619	10296634	2	8	16	GT	22	5
74049	7	10296838	10296857	2	10	20	AC	18	5
74052	7	10299236	10299249	2	7	14	AC	31	6
74053	7	10299403	10299416	2	7	14	GT	28	3
74054	7	10301783	10301800	2	9	18	AC	20	8
74056	7	10304550	10304565	2	8	16	AG	20	5
74058	7	10308570	10308585	2	8	16	GT	28	5
74065	7	10317408	10317419	2	6	12	AC	31	4
74066	7	10317549	10317560	2	6	12	TG	25	4
74067	7	10317699	10317742	2	22	44	GT	8	11
74070	7	10322908	10322925	2	9	18	AC	26	8
74071	7	10325162	10325173	2	6	12	GT	23	4
74074	7	10327745	10327764	2	10	20	CA	9	5
74075	7	10327934	10327947	2	7	14	AC	26	8
74077	7	10330639	10330674	2	18	36	CA	6	6
74080	7	10331608	10331631	2	12	24	GT	22	8
74082	7	10332724	10332737	2	7	14	AG	24	7
74083	7	10333868	10333897	2	15	30	TG	13	11
74084	7	10335996	10336021	2	13	26	TG	16	8
74085	7	10336106	10336121	2	8	16	TG	24	6
74086	7	10336163	10336178	2	8	16	TG	21	3
74087	7	10336275	10336302	2	14	28	AC	17	11
74088	7	10336442	10336457	2	8	16	TG	20	7
74089	7	10336516	10336533	2	9	18	TG	21	8
74106	7	10345280	10345301	2	11	22	AC	20	8
74108	7	10345585	10345616	2	16	32	TG	14	15
74109	7	10345819	10345836	2	9	18	AC	14	5
74110	7	10345969	10345984	2	8	16	CA	15	3
74116	7	10350580	10350597	2	9	18	AC	13	7
74122	7	10359301	10359314	2	7	14	CA	24	8
74123	7	10359552	10359563	2	6	12	TG	23	6
74124	7	10361863	10361878	2	8	16	GT	19	4
74126	7	10362803	10362848	2	23	46	TG	9	11
74134	7	10375055	10375090	2	18	36	AC	7	9
74137	7	10377393	10377412	2	10	20	TG	19	5
74139	7	10377478	10377491	2	7	14	GT	18	4

74140	7	10385127	10385166	2	20	40	GT	13	10
74143	7	10386603	10386616	2	7	14	TG	30	4
74145	7	10390731	10390752	2	11	22	TG	28	6
74146	7	10390880	10390895	4	4	16	CAGT	26	3
74150	7	10396964	10396981	2	9	18	TG	19	5
74156	7	10408412	10408427	2	8	16	TC	21	4
74159	7	10410034	10410061	2	14	28	CA	9	14
74165	7	10412552	10412572	3	7	21	CTG	23	4
74167	7	10419722	10419735	2	7	14	GT	14	5
74168	7	10419869	10419896	2	14	28	TG	6	9
74170	7	10420222	10420247	2	13	26	GT	19	11
74175	7	10421360	10421399	2	20	40	TG	7	8
74176	7	10421883	10421897	3	5	15	TGG	28	4
74179	7	10426357	10426378	2	11	22	GA	22	5
74183	7	10428605	10428632	2	14	28	TG	18	7
74186	7	10432191	10432210	2	10	20	AC	16	9
74189	7	10433324	10433349	2	13	26	CT	15	10
74193	7	10438117	10438156	2	20	40	GT	7	6
74195	7	10440673	10440708	2	18	36	TG	17	10
74196	7	10443817	10443838	2	11	22	CA	20	7
74197	7	10446016	10446053	2	19	38	TG	7	7
74198	7	10446155	10446168	2	7	14	TG	19	5
74205	7	10453522	10453543	2	11	22	GT	14	11
74211	7	10455052	10455063	2	6	12	AC	25	9
74213	7	10457279	10457312	2	17	34	AC	11	13
74214	7	10457634	10457647	2	7	14	GT	30	3
74219	7	10459561	10459572	2	6	12	GT	15	8
74223	7	10466907	10466920	2	7	14	GT	33	4
74226	7	10470417	10470448	2	16	32	AC	10	9
74228	7	10471708	10471721	2	7	14	AC	25	4
74231	7	10473821	10473836	4	4	16	ATTT	12	10
74236	7	10477558	10477579	2	11	22	AC	9	11
74241	7	10482958	10482969	2	6	12	GT	34	6
74242	7	10489927	10489960	2	17	34	AC	12	5
74244	7	10492201	10492220	4	5	20	GAGG	13	4
74246	7	10496740	10496751	2	6	12	GT	27	3
74253	7	10502298	10502311	2	7	14	CA	27	3
74254	7	10503887	10503912	2	13	26	CA	21	7
74256	7	10504690	10504707	2	9	18	GA	26	8
74257	7	10504709	10504720	2	6	12	AG	20	9
74260	7	10506097	10506110	2	7	14	GT	26	3
74261	7	10506482	10506509	2	14	28	CA	13	7
74264	7	10514600	10514613	2	7	14	TG	15	5
74270	7	10518314	10518329	2	8	16	AC	11	9
74271	7	10518864	10518879	2	8	16	CA	30	3
74282	7	10536574	10536594	3	7	21	AGA	6	5
74286	7	10545007	10545032	2	13	26	TG	26	7
74287	7	10545148	10545161	2	7	14	AC	18	6
74289	7	10545720	10545746	3	9	27	AGG	19	4
74295	7	10562477	10562504	2	14	28	TG	14	5
74298	7	10569474	10569491	2	9	18	GT	19	8

74304	7	10602898	10602913	2	8	16	CT	9	6
74308	7	10607800	10607833	2	17	34	AC	12	11
74312	7	10610318	10610335	2	9	18	GT	22	4
74316	7	10621104	10621115	2	6	12	TG	28	3
74320	7	10624998	10625037	2	20	40	TG	11	12
74325	7	10631179	10631194	4	4	16	AAAT	19	8
74327	7	10634375	10634392	2	9	18	GA	17	10
74328	7	10634490	10634505	4	4	16	GAGC	13	3
74331	7	10637032	10637053	2	11	22	TG	9	13
74341	7	10648214	10648235	2	11	22	CA	19	7
74346	7	10656499	10656522	2	12	24	TG	8	8
74350	7	10661205	10661226	2	11	22	CA	20	9
74351	7	10661537	10661566	2	15	30	GT	26	10
74362	7	10683998	10684017	2	10	20	TC	20	3
74363	7	10687042	10687057	4	4	16	CTTC	35	6
74370	7	10694515	10694534	2	10	20	AC	17	11
74372	7	10696397	10696412	2	8	16	AC	9	3
74373	7	10697906	10697919	2	7	14	TG	17	13
74377	7	10701387	10701404	2	9	18	TG	15	8
74379	7	10708704	10708723	2	10	20	CA	20	5
74382	7	10714984	10715017	2	17	34	CA	8	10
74383	7	10716790	10716815	2	13	26	TG	11	10
74386	7	10718280	10718293	2	7	14	GT	25	12
74391	7	10724802	10724815	2	7	14	TC	22	3
74402	7	10735310	10735323	2	7	14	TC	12	9
74403	7	10735324	10735341	2	9	18	AC	13	14
74409	7	10737792	10737813	2	11	22	TG	28	8
74410	7	10738838	10738853	2	8	16	GT	21	7
74414	7	10743518	10743535	2	9	18	TG	17	12
74415	7	10743538	10743549	2	6	12	TG	6	5
74416	7	10743640	10743661	2	11	22	GT	16	12
74421	7	10758533	10758548	2	8	16	AC	25	6
74425	7	10761132	10761143	2	6	12	GT	21	4
74430	7	10772217	10772242	2	13	26	TG	16	8
74431	7	10772873	10772896	3	8	24	TTG	18	4
74432	7	10773319	10773333	3	5	15	CCT	21	3
74435	7	10774905	10774919	3	5	15	CTC	24	6
74437	7	10782434	10782449	2	8	16	CA	18	4
74443	7	10785363	10785386	3	8	24	GGA	16	6
74445	7	10785701	10785715	3	5	15	CTG	24	4
74446	7	10787727	10787744	2	9	18	CA	22	9
74447	7	10789269	10789280	2	6	12	GT	21	6
74450	7	10791438	10791463	2	13	26	CA	18	11
74452	7	10791672	10791693	2	11	22	CA	28	11
74456	7	10795161	10795172	2	6	12	CT	30	3
74458	7	10797002	10797033	2	16	32	GT	18	10
74459	7	10797409	10797422	2	7	14	CA	6	5
74462	7	10797761	10797786	2	13	26	TG	14	9
74463	7	10797827	10797846	2	10	20	CA	21	5
74468	7	10802912	10802931	2	10	20	TC	20	6
74469	7	10806149	10806166	2	9	18	GA	24	3

74470	7	10806641	10806656	2	8	16	GT	22	6
74471	7	10806841	10806852	2	6	12	GT	22	7
74472	7	10811172	10811191	2	10	20	GT	17	9
74475	7	10819339	10819374	2	18	36	AC	12	12
74476	7	10820305	10820320	2	8	16	CA	27	4
74479	7	10822915	10822930	2	8	16	CA	18	8
74481	7	10823119	10823140	2	11	22	CA	12	10
74482	7	10825227	10825250	2	12	24	TG	16	7
74489	7	10832506	10832529	2	12	24	CA	14	8
74490	7	10832640	10832671	2	16	32	AC	14	6
74491	7	10832754	10832771	2	9	18	TG	15	9
74492	7	10832827	10832844	2	9	18	GT	22	7
74494	7	10833309	10833328	4	5	20	AGAT	25	3
74497	7	10835252	10835271	2	10	20	AC	31	8
74498	7	10837006	10837021	2	8	16	GA	18	7
74500	7	10837380	10837399	2	10	20	TG	17	7
74502	7	10838893	10838914	2	11	22	TG	19	12
74503	7	10839054	10839065	2	6	12	CA	31	6
74505	7	10845476	10845487	2	6	12	CA	24	10
74507	7	10848980	10848991	2	6	12	GT	19	5
74508	7	10850770	10850797	2	14	28	CA	16	8
74513	7	10855768	10855803	2	18	36	GT	14	12
74514	7	10855915	10855926	2	6	12	GA	17	8
74521	7	10868124	10868155	2	16	32	AC	9	10
74522	7	10871439	10871458	2	10	20	CA	15	9
74523	7	10872795	10872806	2	6	12	AC	27	3
74524	7	10873234	10873281	4	12	48	ATCT	11	10
74527	7	10879685	10879700	2	8	16	GT	24	4
74529	7	10885224	10885238	3	5	15	GAA	28	5
74531	7	10890737	10890758	2	11	22	TG	10	14
74532	7	10896886	10896903	2	9	18	TG	14	7
74533	7	10897738	10897749	2	6	12	CA	24	10
74534	7	10900189	10900202	2	7	14	TG	17	9
74535	7	10900205	10900218	2	7	14	TG	16	9
74536	7	10901217	10901230	2	7	14	GT	24	7
74537	7	10903092	10903113	2	11	22	TG	21	10
74539	7	10907811	10907826	2	8	16	TG	32	5
74540	7	10908973	10908984	2	6	12	TG	24	5
74541	7	10910785	10910802	2	9	18	GT	23	9
74555	7	10931599	10931613	3	5	15	GTT	31	4
74556	7	10933414	10933431	2	9	18	CT	24	6
74557	7	10933434	10933445	2	6	12	CT	21	8
74558	7	10941704	10941718	3	5	15	TCC	10	3
74559	7	10946408	10946419	2	6	12	GT	24	3
74562	7	10951292	10951307	4	4	16	TTTG	22	3
74570	7	10960267	10960280	2	7	14	TG	26	16
74574	7	10978483	10978497	3	5	15	ACA	21	5
74578	7	10997479	10997494	4	4	16	TAAA	10	3
74582	7	11007131	11007146	2	8	16	CT	26	7
74583	7	11007149	11007160	2	6	12	CT	24	6
74587	7	11018627	11018644	2	9	18	TG	20	11

74588	7	11021914	11021925	2	6	12	CA	14	5
74589	7	11043259	11043276	3	6	18	GTG	28	3
74590	7	11045654	11045671	3	6	18	TGA	25	4
74592	7	11049514	11049546	3	11	33	ATT	14	12
74596	7	11055335	11055355	3	7	21	CCT	17	5
74598	7	11055895	11055916	2	11	22	TG	16	7
74599	7	11056065	11056076	2	6	12	TG	24	6
74600	7	11056078	11056091	2	7	14	GT	20	6
74603	7	11063263	11063280	2	9	18	TG	19	3
74611	7	11092514	11092537	2	12	24	TG	20	10
74615	7	11106374	11106400	3	9	27	AAT	6	4
74616	7	11107679	11107690	2	6	12	GA	29	3
74625	7	11117163	11117182	4	5	20	GATG	27	4
74630	7	11122846	11122865	2	10	20	GT	12	6
74631	7	11126054	11126079	2	13	26	CA	20	7
74635	7	11131189	11131230	2	21	42	AC	14	10
74636	7	11132224	11132237	2	7	14	AT	6	5
74640	7	11139560	11139571	2	6	12	TA	20	5
74641	7	11140358	11140369	2	6	12	GT	15	3
74642	7	11141810	11141835	2	13	26	CA	14	7
74645	7	11144329	11144348	2	10	20	TG	20	9
74646	7	11144530	11144561	2	16	32	CA	15	10
74647	7	11149827	11149838	2	6	12	TG	20	6
74650	7	11153456	11153473	3	6	18	GAA	24	3
74651	7	11153630	11153644	3	5	15	CAG	20	4
74654	7	11155663	11155684	2	11	22	TG	18	8
74655	7	11159542	11159567	2	13	26	AC	20	12
74658	7	11166943	11166966	2	12	24	GT	14	6
74659	7	11170946	11170959	2	7	14	TG	23	7
74661	7	11171134	11171153	2	10	20	AC	6	3
74663	7	11171473	11171496	2	12	24	CA	20	5
74666	7	11181219	11181232	2	7	14	CA	15	4
74667	7	11181361	11181386	2	13	26	AC	17	5
74669	7	11185753	11185770	2	9	18	TG	10	7
74674	7	11194160	11194207	4	12	48	GATA	15	13
74675	7	11194606	11194627	2	11	22	CA	10	7
74679	7	11197177	11197190	2	7	14	TA	9	6
74680	7	11197778	11197801	2	12	24	GA	18	12
74684	7	11211583	11211610	2	14	28	CA	22	10
74688	7	11217553	11217568	2	8	16	CA	14	10
74689	7	11217630	11217647	2	9	18	AC	17	15
74692	7	11224870	11224887	3	6	18	TAA	11	4
74695	7	11231001	11231015	3	5	15	AGC	24	6
74698	7	11236533	11236556	3	8	24	CTG	32	5
74699	7	11241357	11241372	2	8	16	GT	21	7
74705	7	11247630	11247649	4	5	20	ATGG	19	5
74707	7	11249637	11249660	2	12	24	GT	15	7
74711	7	11252522	11252553	2	16	32	AC	11	8
74721	7	11261157	11261170	2	7	14	TA	19	3
74722	7	11264812	11264829	2	9	18	GT	29	4
74723	7	11265017	11265032	2	8	16	CA	24	3

74725	7	11266661	11266674	2	7	14	TG	14	3
74732	7	11269370	11269385	2	8	16	CA	20	3
74734	7	11270810	11270829	2	10	20	TG	21	6
74735	7	11271193	11271230	2	19	38	TG	12	11
74739	7	11279115	11279138	2	12	24	GT	25	6
74740	7	11281407	11281424	2	9	18	AC	15	10
74747	7	11289848	11289863	2	8	16	TG	26	6
74749	7	11295549	11295572	2	12	24	AC	17	9
74751	7	11301325	11301338	2	7	14	CT	34	3
74753	7	11301785	11301806	2	11	22	GT	21	3
74758	7	11306063	11306110	2	24	48	TC	6	7
74759	7	11306653	11306676	3	8	24	ATG	15	6
74762	7	11308258	11308279	2	11	22	AC	20	6
74766	7	11316947	11316960	2	7	14	GT	27	3
74770	7	11322372	11322383	2	6	12	AG	26	3
74772	7	11322767	11322786	2	10	20	TG	7	11
74776	7	11326319	11326362	2	22	44	AC	8	10
74777	7	11328814	11328835	2	11	22	TG	7	6
74786	7	11333002	11333025	2	12	24	AC	24	8
74788	7	11334975	11334996	2	11	22	AC	9	6
74789	7	11335079	11335108	2	15	30	AC	17	5
74790	7	11341607	11341620	2	7	14	TC	23	5
74791	7	11341622	11341639	2	9	18	CT	21	5
74792	7	11341966	11341997	2	16	32	TG	13	10
74794	7	11346000	11346020	3	7	21	AGG	19	5
74798	7	11351202	11351221	2	10	20	CA	13	7
74799	7	11352657	11352672	2	8	16	CA	13	5
74800	7	11352673	11352687	3	5	15	CTG	12	4
74803	7	11361262	11361291	2	15	30	CA	7	7
74804	7	11361433	11361446	2	7	14	GT	32	4
74805	7	11362757	11362776	2	10	20	TG	18	9
74808	7	11365466	11365477	2	6	12	TG	15	10
74810	7	11367590	11367605	2	8	16	CA	26	11
74812	7	11370643	11370657	3	5	15	CAG	34	3
74813	7	11371301	11371324	2	12	24	TG	14	10
74816	7	11375917	11375938	2	11	22	CT	22	6
74817	7	11378359	11378388	2	15	30	TG	17	16
74827	7	11384159	11384174	2	8	16	CA	25	4
74828	7	11384321	11384342	2	11	22	CA	16	12
74829	7	11384984	11385045	2	31	62	GT	24	11
74832	7	11391496	11391545	2	25	50	GT	13	11
74833	7	11392561	11392600	2	20	40	GT	13	13
74834	7	11392886	11392903	3	6	18	TGT	24	7
74841	7	11400100	11400121	2	11	22	TG	19	3
74842	7	11400957	11400984	2	14	28	GT	7	10
74843	7	11403227	11403244	3	6	18	GAG	25	5
74845	7	11403638	11403661	2	12	24	AC	13	9
74846	7	11403756	11403767	2	6	12	GT	29	5
74847	7	11403971	11403998	2	14	28	TG	12	12
74848	7	11404061	11404072	2	6	12	CA	24	6
74861	7	11418792	11418805	2	7	14	CT	22	4

74863	7	11422839	11422852	2	7	14	AC	24	6
74864	7	11431112	11431127	2	8	16	AC	20	11
74866	7	11431976	11432003	2	14	28	GT	7	7
74873	7	11440914	11440943	2	15	30	GT	10	12
74876	7	11442326	11442341	2	8	16	TG	18	7
74877	7	11442766	11442777	2	6	12	TG	12	9
74880	7	11443141	11443170	2	15	30	GT	27	8
74881	7	11443411	11443450	2	20	40	TG	13	11
74882	7	11444640	11444665	2	13	26	AC	14	5
74884	7	11446547	11446562	4	4	16	CAGA	33	5
74887	7	11452096	11452107	2	6	12	GT	32	3
74891	7	11453390	11453415	2	13	26	CA	6	6
74895	7	11459459	11459502	2	22	44	AC	11	10
74903	7	11461950	11461977	2	14	28	AC	11	8
74906	7	11464287	11464310	2	12	24	CA	21	7
74907	7	11464542	11464553	2	6	12	CT	25	3
74909	7	11464790	11464821	2	16	32	CA	10	4
74910	7	11466470	11466511	2	21	42	CT	7	7
74914	7	11466739	11466762	2	12	24	AC	9	5
74915	7	11466893	11466920	2	14	28	AC	14	5
74916	7	11468079	11468092	2	7	14	CT	19	6
74917	7	11468587	11468612	2	13	26	TG	16	8
74924	7	11476510	11476533	4	6	24	TGTC	20	3
74933	7	11481824	11481837	2	7	14	AC	25	7
74934	7	11481969	11481980	2	6	12	AC	23	4
74935	7	11483798	11483811	2	7	14	AC	19	3
74937	7	11486042	11486061	2	10	20	CA	13	5
74938	7	11487045	11487064	2	10	20	TG	24	9
74945	7	11492150	11492191	2	21	42	CA	12	12
74946	7	11492690	11492710	3	7	21	CTC	18	5
74947	7	11492940	11492957	2	9	18	TC	14	4
74949	7	11493143	11493174	2	16	32	CA	8	11
74954	7	11496219	11496240	2	11	22	AC	22	9
74955	7	11499434	11499485	2	26	52	GT	10	12
74958	7	11499969	11499984	2	8	16	GT	12	8
74959	7	11501656	11501679	4	6	24	TCTT	24	5
74961	7	11501929	11501960	2	16	32	CT	12	9
74964	7	11502455	11502475	3	7	21	TCC	25	3
74966	7	11503971	11504006	3	12	36	TTC	15	11
74967	7	11504059	11504079	3	7	21	TCC	8	3
74981	7	11511949	11511974	2	13	26	AC	14	8
74982	7	11512618	11512633	2	8	16	TG	22	8
74983	7	11514802	11514827	2	13	26	TG	11	7
74986	7	11516725	11516742	2	9	18	AC	21	11
74990	7	11517379	11517396	2	9	18	CA	18	16
74992	7	11518034	11518049	4	4	16	CAGG	21	5
74996	7	11521834	11521847	2	7	14	CA	22	13
74997	7	11522441	11522466	2	13	26	AC	25	7
74999	7	11522868	11522891	3	8	24	GAG	14	4
75006	7	11528422	11528435	2	7	14	GT	20	8
75007	7	11529201	11529226	2	13	26	TG	9	8

75010	7	11530086	11530100	3	5	15	AAT	22	4
75013	7	11532171	11532184	2	7	14	AC	13	3
75014	7	11532199	11532210	2	6	12	AC	19	7
75015	7	11532436	11532449	2	7	14	GT	26	5
75018	7	11536036	11536049	2	7	14	GT	24	6
75019	7	11536219	11536234	2	8	16	GA	21	6
75020	7	11538312	11538343	2	16	32	GT	18	6
75022	7	11540223	11540248	2	13	26	TG	12	10
75027	7	11544571	11544582	2	6	12	AC	31	3
75029	7	11546881	11546892	2	6	12	GT	28	3
75030	7	11549536	11549549	2	7	14	CG	13	12
75034	7	11550860	11550879	2	10	20	CA	14	5
75035	7	11551052	11551075	2	12	24	AC	21	8
75040	7	11553823	11553840	3	6	18	AAT	22	3
75041	7	11556762	11556787	2	13	26	AC	10	6
75043	7	11557330	11557349	2	10	20	TG	22	5
75044	7	11557478	11557491	2	7	14	GT	30	4
75045	7	11557827	11557838	2	6	12	AC	26	4
75049	7	11566215	11566248	2	17	34	TG	12	8
75055	7	11572853	11572866	2	7	14	CA	22	4
75057	7	11573013	11573024	2	6	12	AC	17	7
75059	7	11573204	11573225	2	11	22	CA	10	9
75064	7	11574459	11574492	2	17	34	GT	15	12
75070	7	11577541	11577558	2	9	18	AC	33	6
75071	7	11577706	11577721	4	4	16	TGTC	24	6
75072	7	11577722	11577735	2	7	14	TG	24	5
75073	7	11577883	11577898	2	8	16	TG	27	8
75074	7	11581917	11581932	2	8	16	AC	23	6
75076	7	11582074	11582109	2	18	36	GT	7	6
75077	7	11582281	11582294	2	7	14	AC	27	5
75078	7	11584177	11584192	2	8	16	AC	22	7
75079	7	11586008	11586025	2	9	18	AC	24	4
75083	7	11588489	11588514	2	13	26	CA	18	12
75086	7	11589096	11589107	2	6	12	TG	22	3
75089	7	11596191	11596202	2	6	12	GA	28	4
75095	7	11604511	11604534	2	12	24	GA	13	5
75097	7	11605613	11605628	2	8	16	CT	20	3
75104	7	11612352	11612367	2	8	16	AC	9	8
75106	7	11612501	11612514	2	7	14	AC	17	4
75108	7	11615080	11615112	3	11	33	GGA	11	8
75109	7	11615436	11615481	2	23	46	TG	11	11
75110	7	11616998	11617011	2	7	14	GT	25	3
75111	7	11617928	11617945	2	9	18	CA	19	9
75112	7	11618104	11618115	2	6	12	GT	30	3
75113	7	11618671	11618688	2	9	18	CA	21	3
75122	7	11631166	11631177	2	6	12	CA	28	5
75123	7	11631346	11631361	2	8	16	CA	24	3
75129	7	11634774	11634801	2	14	28	CT	7	7
75141	7	11641900	11641911	2	6	12	AC	34	4
75143	7	11642582	11642607	2	13	26	AC	10	6
75145	7	11644845	11644874	2	15	30	GT	13	7

75146	7	11645044	11645057	2	7	14	CA	21	3
75149	7	11651202	11651215	2	7	14	CA	26	3
75150	7	11651505	11651520	2	8	16	AG	23	4
75152	7	11651818	11651839	2	11	22	GT	16	12
75157	7	11659287	11659314	2	14	28	CA	26	7
75162	7	11667198	11667217	2	10	20	CA	20	9
75166	7	11673798	11673813	2	8	16	CA	18	8
75169	7	11676405	11676430	2	13	26	CA	14	12
75171	7	11680380	11680393	2	7	14	GT	22	4
75173	7	11680638	11680653	2	8	16	AC	17	7
75174	7	11682011	11682042	2	16	32	AC	12	8
75175	7	11682421	11682434	2	7	14	CT	25	4
75176	7	11683735	11683754	2	10	20	CA	19	10
75177	7	11684617	11684654	2	19	38	TG	11	10
75178	7	11686245	11686274	2	15	30	TG	14	9
75179	7	11686402	11686419	2	9	18	TG	18	10
75180	7	11690818	11690837	2	10	20	TG	16	8
75181	7	11690839	11690852	2	7	14	GT	15	9
75182	7	11692983	11693006	2	12	24	AC	22	13
75183	7	11693055	11693066	2	6	12	CA	22	3
75185	7	11696304	11696319	2	8	16	CA	21	7
75186	7	11696411	11696428	2	9	18	AC	23	4
75188	7	11700870	11700881	2	6	12	AC	19	3
75191	7	11704474	11704495	2	11	22	CA	11	9
75192	7	11704643	11704664	2	11	22	GT	18	8
75193	7	11705394	11705411	2	9	18	AC	24	3
75201	7	11723177	11723200	2	12	24	CA	21	16
75203	7	11725784	11725803	2	10	20	AG	27	3
75207	7	11727872	11727895	2	12	24	AC	16	7
75211	7	11738274	11738293	2	10	20	GT	15	7
75212	7	11738384	11738417	2	17	34	AC	7	7
75214	7	11741198	11741235	2	19	38	GT	7	9
75217	7	11741753	11741764	2	6	12	TG	27	3
75218	7	11741963	11741976	2	7	14	TC	24	3
75219	7	11744516	11744537	2	11	22	TG	6	5
75221	7	11745733	11745746	2	7	14	TG	16	4
75222	7	11749143	11749168	2	13	26	AG	14	4
75226	7	11750444	11750459	2	8	16	GA	22	5
75229	7	11756402	11756417	2	8	16	CA	23	5
75232	7	11756701	11756714	2	7	14	TG	8	3
75233	7	11761336	11761347	2	6	12	TG	23	4
75238	7	11766296	11766337	2	21	42	CA	8	11
75240	7	11766978	11766995	3	6	18	GCC	26	4
75244	7	11770228	11770255	2	14	28	TG	14	8
75245	7	11770995	11771014	2	10	20	AC	22	4
75256	7	11783593	11783606	2	7	14	GT	26	4
75261	7	11788400	11788414	3	5	15	GCA	25	6
75262	7	11788415	11788432	3	6	18	ACA	12	3
75269	7	11808478	11808495	2	9	18	AC	24	4
75270	7	11812132	11812149	2	9	18	TG	24	4
75271	7	11812307	11812322	2	8	16	TG	13	5

75279	7	11821395	11821406	2	6	12	AC	24	3
75282	7	11824902	11824925	2	12	24	AC	9	9
75290	7	11829906	11829925	2	10	20	TG	14	4
75291	7	11833169	11833194	2	13	26	CA	16	10
75292	7	11833681	11833692	2	6	12	AC	29	3
75293	7	11834532	11834545	2	7	14	AC	21	3
75299	7	11835485	11835496	2	6	12	TG	27	3
75303	7	11839775	11839788	2	7	14	GT	20	3
75304	7	11854835	11854856	2	11	22	AC	20	8
75311	7	11865494	11865513	2	10	20	GA	18	11
75313	7	11873042	11873063	2	11	22	GT	14	9
75320	7	11907131	11907160	2	15	30	AC	8	9
75323	7	11911237	11911250	2	7	14	AC	26	5
75326	7	11912760	11912775	4	4	16	AAAT	7	6
75327	7	11916426	11916437	2	6	12	CA	23	3
75333	7	11925891	11925922	2	16	32	CA	6	9
75334	7	11926047	11926102	2	28	56	CA	10	9
75337	7	11927902	11927945	2	22	44	AC	9	8
75341	7	11933415	11933444	2	15	30	GT	17	9
75342	7	11935473	11935508	2	18	36	CA	6	9
75343	7	11935823	11935846	2	12	24	TG	21	6
75344	7	11937386	11937417	2	16	32	AC	19	9
75346	7	11937918	11937933	2	8	16	AC	21	3
75348	7	11938194	11938223	2	15	30	TC	14	5
75349	7	11938259	11938272	2	7	14	CA	16	3
75350	7	11938668	11938679	2	6	12	AT	9	5
75351	7	11939847	11939858	2	6	12	AT	11	3
75354	7	11947410	11947423	2	7	14	AC	20	3
75355	7	11950219	11950232	2	7	14	TG	18	4
75356	7	11951255	11951266	2	6	12	AC	23	6
75358	7	11951867	11951880	2	7	14	TG	32	3
75360	7	11952697	11952724	2	14	28	TG	15	6
75363	7	11957588	11957605	2	9	18	CA	14	4
75367	7	11969898	11969917	2	10	20	GT	20	3
75368	7	11970544	11970571	2	14	28	AC	19	12
75371	7	11978812	11978835	4	6	24	TATT	18	3
75372	7	11980027	11980050	2	12	24	CA	24	10
75373	7	11981703	11981718	2	8	16	TG	18	7
75375	7	11985070	11985083	2	7	14	AC	19	4
75378	7	11986675	11986688	2	7	14	AC	24	15
75380	7	11987497	11987508	2	6	12	TG	30	3
75382	7	11989793	11989822	2	15	30	AC	11	13
75383	7	11993254	11993277	2	12	24	AC	16	6
75385	7	11994141	11994156	2	8	16	CT	16	3
75386	7	11994821	11994858	2	19	38	CA	13	18
75387	7	11995826	11995845	2	10	20	CA	18	4
75388	7	11995968	11995979	2	6	12	CA	11	8
75391	7	11997677	11997708	4	8	32	TTCT	19	7
75396	7	12002047	12002061	3	5	15	TCT	15	5
75399	7	12004222	12004237	2	8	16	GA	29	3
75400	7	12004437	12004454	3	6	18	TCC	8	3

75403	7	12011401	12011424	2	12	24	GT	20	5
75407	7	12016739	12016752	2	7	14	AC	12	7
75409	7	12019430	12019463	2	17	34	GT	16	7
75411	7	12019659	12019688	2	15	30	CA	7	10
75412	7	12019785	12019800	2	8	16	CA	17	7
75413	7	12019859	12019885	3	9	27	CAT	13	3
75414	7	12019958	12019983	2	13	26	CA	14	8
75417	7	12020161	12020184	4	6	24	CAGA	22	13
75420	7	12021144	12021157	2	7	14	CT	15	4
75423	7	12022665	12022686	2	11	22	GT	18	5
75424	7	12022794	12022814	3	7	21	CAT	32	5
75426	7	12024872	12024883	2	6	12	CT	33	3
75427	7	12025295	12025314	2	10	20	CA	15	9
75432	7	12032416	12032437	2	11	22	TG	21	5
75433	7	12033034	12033057	2	12	24	AC	18	8
75434	7	12033165	12033184	2	10	20	TG	21	4
75440	7	12037538	12037557	2	10	20	GA	13	3
75447	7	12046871	12046890	2	10	20	CA	19	6
75449	7	12047314	12047345	2	16	32	GT	21	6
75452	7	12051049	12051064	2	8	16	AC	19	6
75454	7	12051394	12051409	2	8	16	TG	26	4
75456	7	12051667	12051682	2	8	16	GT	25	4
75460	7	12053490	12053503	2	7	14	TC	20	3
75462	7	12054396	12054413	2	9	18	GT	18	3
75467	7	12057746	12057765	2	10	20	TG	20	10
75468	7	12058407	12058428	2	11	22	GT	17	3
75470	7	12058734	12058755	2	11	22	TG	15	8
75471	7	12058843	12058862	2	10	20	CA	13	7
75472	7	12059108	12059121	2	7	14	GT	25	4
75473	7	12060272	12060287	2	8	16	GT	22	4
75475	7	12060493	12060506	2	7	14	AG	22	3
75476	7	12060614	12060629	2	8	16	AC	20	9
75477	7	12061191	12061206	2	8	16	GT	20	4
75479	7	12061455	12061472	2	9	18	AC	21	3
75481	7	12063493	12063520	2	14	28	CA	26	6
75483	7	12066422	12066439	2	9	18	TG	21	6
75486	7	12068728	12068739	2	6	12	AC	25	4
75488	7	12070002	12070033	2	16	32	AG	13	9
75489	7	12070188	12070208	3	7	21	TCC	7	6
75490	7	12070278	12070301	3	8	24	TCC	12	3
75491	7	12070345	12070358	2	7	14	TC	18	6
75495	7	12072997	12073010	2	7	14	AG	8	4
75499	7	12075697	12075718	2	11	22	CA	22	5
75506	7	12078000	12078021	2	11	22	CT	14	5
75508	7	12083110	12083141	2	16	32	TG	14	8
75509	7	12083338	12083349	2	6	12	CA	32	3
75510	7	12092422	12092435	2	7	14	GT	25	3
75519	7	12100358	12100369	2	6	12	GT	25	3
75520	7	12101287	12101318	2	16	32	TG	16	6
75521	7	12101434	12101455	2	11	22	AC	17	4
75525	7	12106509	12106542	2	17	34	CA	11	8

75531	7	12112354	12112371	2	9	18	GT	22	4
75532	7	12112933	12112952	2	10	20	GT	21	10
75535	7	12115149	12115164	2	8	16	GT	20	3
75538	7	12119976	12120011	2	18	36	CA	14	9
75539	7	12120136	12120151	2	8	16	CA	25	5
75548	7	12132182	12132207	2	13	26	GT	20	3
75549	7	12132282	12132297	2	8	16	CA	12	7
75550	7	12137864	12137887	2	12	24	AC	19	8
75552	7	12138453	12138470	2	9	18	GT	17	5
75556	7	12138737	12138750	2	7	14	AC	18	4
75557	7	12139276	12139287	2	6	12	GT	26	4
75562	7	12140381	12140392	2	6	12	GT	18	3
75564	7	12142484	12142497	2	7	14	AC	23	5
75565	7	12142697	12142732	2	18	36	CT	7	3
75567	7	12143728	12143739	2	6	12	AC	25	4
75568	7	12143744	12143755	2	6	12	AC	12	3
75570	7	12143907	12143938	2	16	32	TG	7	7
75571	7	12144044	12144079	2	18	36	AC	16	12
75574	7	12148115	12148130	2	8	16	AC	27	4
75575	7	12149628	12149665	2	19	38	GT	13	9
75577	7	12155714	12155735	2	11	22	CA	12	5
75579	7	12161248	12161259	2	6	12	CA	28	3
75582	7	12165215	12165228	2	7	14	CA	24	5
75587	7	12187472	12187501	3	10	30	CCT	15	5
75605	7	12201764	12201775	2	6	12	TG	28	5
75607	7	12206285	12206300	2	8	16	TG	14	4
75613	7	12211132	12211147	2	8	16	AC	22	3
75614	7	12212735	12212768	2	17	34	GT	12	17
75616	7	12213009	12213028	2	10	20	TG	16	5
75618	7	12213070	12213081	2	6	12	GT	18	3
75623	7	12221535	12221552	3	6	18	TTC	18	3
75628	7	12232645	12232656	2	6	12	GT	20	4
75635	7	12235780	12235801	2	11	22	CA	32	10
75636	7	12235974	12235997	2	12	24	AC	17	14
75639	7	12236409	12236426	3	6	18	TGT	10	9
75641	7	12246935	12246955	3	7	21	CCT	18	9
75650	7	12275869	12275880	2	6	12	CA	24	3
75651	7	12281290	12281315	2	13	26	CT	17	10
75653	7	12283486	12283499	2	7	14	TG	21	6
75656	7	12297813	12297826	2	7	14	CA	9	7
75663	7	12304700	12304719	4	5	20	ATGG	6	3
75669	7	12306581	12306596	4	4	16	TAAA	13	4
75676	7	12312714	12312739	2	13	26	CA	21	7
75678	7	12314736	12314751	4	4	16	AGAC	28	3
75680	7	12315425	12315440	4	4	16	TTTA	17	4
75683	7	12322776	12322796	3	7	21	ACA	30	6
75688	7	12354128	12354153	2	13	26	GT	18	10
75690	7	12359919	12359952	2	17	34	TG	9	9
75691	7	12365174	12365223	2	25	50	GT	7	7
75692	7	12372915	12372944	2	15	30	CA	14	9
75693	7	12375422	12375433	2	6	12	GT	26	3

75702	7	12409607	12409618	2	6	12	AC	25	3
75703	7	12409740	12409751	2	6	12	CA	25	4
75705	7	12417424	12417451	2	14	28	GT	12	9
75707	7	12427610	12427627	2	9	18	AC	22	5
75708	7	12439350	12439377	2	14	28	AC	11	8
75724	7	12459336	12459357	2	11	22	TG	17	4
75725	7	12464687	12464708	2	11	22	TC	23	6
75726	7	12467706	12467717	2	6	12	TA	16	3
75727	7	12471733	12471752	2	10	20	TG	18	3
75728	7	12473181	12473196	2	8	16	GT	26	7
75730	7	12478155	12478182	2	14	28	TG	13	12
75737	7	12490740	12490751	2	6	12	CT	27	4
75738	7	12494390	12494403	2	7	14	AC	20	3
75741	7	12496930	12496943	2	7	14	CA	22	4
75744	7	12504157	12504170	2	7	14	AC	29	4
75750	7	12509537	12509552	2	8	16	GT	19	6
75751	7	12513711	12513724	2	7	14	AC	12	4
75760	7	12517528	12517539	2	6	12	TG	26	3
75762	7	12521504	12521517	2	7	14	TG	26	4
75763	7	12521816	12521827	2	6	12	AC	21	8
75770	7	12533564	12533577	2	7	14	CA	34	4
75772	7	12541705	12541764	2	30	60	TG	10	16
75773	7	12543141	12543180	2	20	40	TG	7	9
75774	7	12543299	12543310	2	6	12	CA	31	3
75776	7	12543756	12543793	2	19	38	GT	14	8
75778	7	12547997	12548010	2	7	14	AG	24	3
75779	7	12548537	12548590	2	27	54	GT	8	12
75780	7	12548862	12548877	2	8	16	TG	25	7
75781	7	12550122	12550137	2	8	16	TG	21	8
75782	7	12550140	12550153	2	7	14	TG	18	5
75783	7	12550824	12550851	2	14	28	CA	13	9
75785	7	12554338	12554349	2	6	12	CA	31	3
75787	7	12557177	12557198	2	11	22	TG	12	9
75789	7	12557989	12558030	2	21	42	TC	17	8
75792	7	12566442	12566457	2	8	16	CA	21	6
75799	7	12568805	12568834	2	15	30	CA	10	14
75800	7	12573530	12573553	2	12	24	TG	16	5
75802	7	12579883	12579904	2	11	22	TG	20	9
75807	7	12582679	12582692	2	7	14	TG	19	5
75809	7	12586619	12586642	2	12	24	CA	22	10
75812	7	12586903	12586962	2	30	60	GA	11	11
75814	7	12587361	12587380	2	10	20	AC	21	13
75815	7	12592553	12592566	2	7	14	CT	18	7
75820	7	12603173	12603216	2	22	44	GT	15	13
75821	7	12604093	12604104	2	6	12	GT	25	10
75822	7	12609373	12609406	2	17	34	GT	24	8
75823	7	12612349	12612368	2	10	20	GT	22	4
75832	7	12624171	12624204	2	17	34	GT	7	7
75833	7	12624621	12624638	2	9	18	AC	21	5
75838	7	12630633	12630652	2	10	20	CA	13	7
75840	7	12636308	12636331	2	12	24	TG	15	4

75842	7	12638657	12638671	3	5	15	CAG	30	3
75844	7	12644408	12644437	2	15	30	AC	13	9
75845	7	12644575	12644588	2	7	14	AC	20	5
75846	7	12644848	12644867	2	10	20	AC	7	5
75847	7	12646061	12646075	3	5	15	GCT	15	3
75853	7	12651933	12651992	4	15	60	TGAA	15	12
75855	7	12655338	12655359	2	11	22	AC	20	7
75856	7	12656490	12656507	2	9	18	AC	20	14
75859	7	12662612	12662629	2	9	18	GA	17	6
75860	7	12664149	12664162	2	7	14	GA	29	3
75865	7	12674280	12674297	2	9	18	TG	21	8
75866	7	12675824	12675839	4	4	16	AAAT	28	7
75868	7	12677480	12677495	4	4	16	AATA	29	3
75869	7	12680539	12680554	2	8	16	TG	13	8
75874	7	12682762	12682781	2	10	20	AC	13	3
75875	7	12682910	12682925	2	8	16	AC	24	5
75876	7	12683108	12683141	2	17	34	AC	21	14
75877	7	12684794	12684829	2	18	36	CA	8	8
75878	7	12685322	12685339	2	9	18	GT	25	9
75880	7	12685534	12685559	2	13	26	TG	23	5
75881	7	12687237	12687258	2	11	22	CA	14	7
75882	7	12687444	12687461	2	9	18	CA	23	6
75883	7	12688116	12688129	2	7	14	CA	22	6
75885	7	12690554	12690575	2	11	22	TG	6	3
75893	7	12696265	12696278	2	7	14	TG	16	12
75894	7	12696419	12696440	2	11	22	CA	13	6
75897	7	12699027	12699038	2	6	12	TG	21	10
75898	7	12702286	12702331	2	23	46	AC	7	7
75900	7	12702604	12702635	2	16	32	CT	12	8
75901	7	12702706	12702721	2	8	16	CA	23	12
75902	7	12703160	12703197	2	19	38	AC	8	10
75915	7	12717478	12717493	2	8	16	TG	22	5
75916	7	12719820	12719839	4	5	20	TTTC	16	5
75918	7	12722412	12722447	2	18	36	GT	14	9
75925	7	12735519	12735542	2	12	24	AC	11	9
75930	7	12743152	12743177	2	13	26	AC	22	7
75933	7	12752029	12752044	2	8	16	TG	21	4
75936	7	12758007	12758020	2	7	14	CA	18	11
75937	7	12758137	12758148	2	6	12	TG	18	5
75938	7	12759117	12759128	2	6	12	CA	28	4
75939	7	12762140	12762151	2	6	12	GA	24	3
75942	7	12767682	12767693	2	6	12	TG	29	6
75943	7	12767697	12767708	2	6	12	GT	25	5
75950	7	12780343	12780363	3	7	21	AGG	18	4
75951	7	12780615	12780632	3	6	18	GAG	23	3
75957	7	12794156	12794181	2	13	26	AC	8	9
75959	7	12796668	12796679	2	6	12	TG	23	3
75963	7	12801506	12801519	2	7	14	GT	19	4
75971	7	12818454	12818479	2	13	26	AC	9	9
75972	7	12822453	12822479	3	9	27	GAG	24	6
75974	7	12824811	12824828	2	9	18	CA	16	10

75975	7	12824831	12824844	2	7	14	CA	7	6
75976	7	12829799	12829820	2	11	22	GT	21	7
75977	7	12830040	12830061	2	11	22	AC	21	8
75978	7	12830546	12830569	2	12	24	GA	15	5
75979	7	12831638	12831681	2	22	44	AC	12	11
75985	7	12840918	12840931	2	7	14	AC	29	4
75987	7	12843025	12843048	4	6	24	ATTT	20	3
75992	7	12846016	12846059	2	22	44	AC	9	11
75993	7	12846096	12846115	2	10	20	CA	13	4
75994	7	12850419	12850432	2	7	14	AG	20	3
75995	7	12852593	12852604	2	6	12	GT	33	4
75996	7	12853414	12853427	2	7	14	GT	23	3
75998	7	12855309	12855320	2	6	12	AC	23	4
75999	7	12855459	12855470	2	6	12	CA	26	4
76001	7	12857355	12857384	2	15	30	GT	9	8
76003	7	12859788	12859835	2	24	48	AC	17	17
76005	7	12862930	12862945	4	4	16	TATT	21	3
76009	7	12869844	12869863	2	10	20	CA	23	5
76010	7	12870261	12870272	2	6	12	TG	23	3
76013	7	12878032	12878055	2	12	24	CA	21	10
76014	7	12884066	12884079	2	7	14	GT	25	4
76016	7	12885582	12885593	2	6	12	CA	18	3
76018	7	12888868	12888891	2	12	24	AC	14	10
76021	7	12893107	12893122	2	8	16	TG	28	6
76023	7	12895806	12895827	2	11	22	TG	20	10
76032	7	12901106	12901117	2	6	12	AC	6	5
76039	7	12908802	12908817	2	8	16	GT	12	12
76040	7	12908819	12908832	2	7	14	TG	25	20
76046	7	12920840	12920851	2	6	12	TG	15	8
76047	7	12922695	12922710	2	8	16	CA	22	4
76049	7	12930592	12930611	2	10	20	CA	14	8
76050	7	12930716	12930735	2	10	20	TG	10	11
76058	7	12942161	12942208	2	24	48	CT	6	6
76061	7	12967903	12967930	4	7	28	TTTG	26	4
76069	7	12993133	12993166	2	17	34	AC	7	8
76079	7	13006096	13006113	3	6	18	TCC	32	6
76088	7	13017927	13017948	2	11	22	AC	24	11
76096	7	13031990	13032003	2	7	14	AG	19	4
76098	7	13033185	13033200	2	8	16	AT	8	4
76101	7	13042747	13042770	3	8	24	CTG	29	5
76105	7	13065417	13065438	2	11	22	TC	20	11
76112	7	13077415	13077428	2	7	14	TG	27	4
76114	7	13081452	13081465	2	7	14	TG	20	3
76115	7	13086086	13086097	2	6	12	GC	33	6
76116	7	13089735	13089778	4	11	44	AAAG	9	8
76117	7	13091335	13091360	2	13	26	GT	8	6
76118	7	13091595	13091616	2	11	22	AC	10	12
76119	7	13091827	13091858	2	16	32	TG	14	6
76121	7	13094913	13094926	2	7	14	TA	15	4
76122	7	13097652	13097669	3	6	18	AGC	24	3
76125	7	13103056	13103075	2	10	20	AC	14	5

76126	7	13103158	13103175	2	9	18	TG	15	5
76132	7	13105457	13105470	2	7	14	AC	16	5
76135	7	13113901	13113916	2	8	16	AC	17	5
76136	7	13114720	13114737	2	9	18	TG	27	9
76137	7	13120374	13120415	2	21	42	GA	9	9
76149	7	13144901	13144920	2	10	20	GT	10	8
76150	7	13145855	13145872	2	9	18	AG	21	6
76151	7	13147937	13147968	2	16	32	TG	14	12
76155	7	13153932	13153949	2	9	18	TG	19	4
76159	7	13160750	13160771	2	11	22	TC	23	3
76160	7	13160886	13160901	2	8	16	AC	21	5
76161	7	13161242	13161271	3	10	30	TGC	23	6
76163	7	13161912	13161929	2	9	18	CT	12	5
76165	7	13163442	13163459	2	9	18	GA	15	4
76172	7	13172122	13172159	2	19	38	AC	8	10
76174	7	13175688	13175707	2	10	20	AC	14	14
76175	7	13176843	13176868	2	13	26	CA	24	11
76176	7	13176958	13176971	2	7	14	CA	22	3
76180	7	13185283	13185298	2	8	16	GT	20	9
76181	7	13185481	13185494	2	7	14	AC	19	3
76183	7	13188370	13188385	2	8	16	TG	17	15
76184	7	13188576	13188593	2	9	18	CA	18	6
76186	7	13195535	13195555	3	7	21	AAT	9	3
76190	7	13203139	13203164	2	13	26	CA	12	10
76194	7	13208212	13208225	2	7	14	CA	29	5
76196	7	13211385	13211400	2	8	16	TC	19	4
76197	7	13214180	13214207	2	14	28	AC	9	11
76201	7	13220787	13220801	3	5	15	TCT	14	10
76203	7	13224410	13224441	2	16	32	GT	12	9
76205	7	13226281	13226295	3	5	15	CCT	34	3
76210	7	13232744	13232781	2	19	38	GT	12	14
76220	7	13242984	13243015	4	8	32	TAAA	16	6
76222	7	13243828	13243842	3	5	15	CAC	17	4
76225	7	13244059	13244073	3	5	15	CAC	19	8
76226	7	13248109	13248124	4	4	16	GCTC	25	6
76230	7	13256572	13256583	2	6	12	GT	22	4
76231	7	13258286	13258297	2	6	12	TC	16	9
76234	7	13260944	13260958	3	5	15	GCT	23	5
76240	7	13267511	13267532	2	11	22	CA	21	10
76243	7	13272302	13272329	2	14	28	CA	24	8
76245	7	13276395	13276424	2	15	30	CA	6	6
76246	7	13278889	13278916	2	14	28	CA	11	6
76247	7	13279488	13279503	4	4	16	TTTA	19	4
76249	7	13281497	13281522	2	13	26	TG	26	8
76250	7	13289665	13289676	2	6	12	CA	23	3
76253	7	13295029	13295048	2	10	20	TG	23	6
76259	7	13307688	13307708	3	7	21	GCA	25	4
76262	7	13314198	13314233	2	18	36	GA	18	9
76266	7	13326692	13326707	4	4	16	TTTG	8	3
76267	7	13327297	13327342	2	23	46	AC	7	9
76268	7	13327807	13327830	2	12	24	TG	18	8

76269	7	13329182	13329197	2	8	16	TG	23	3
76271	7	13329569	13329620	2	26	52	AC	11	13
76275	7	13342658	13342685	2	14	28	GT	14	9
76279	7	13348144	13348155	2	6	12	CA	14	3
76280	7	13348496	13348510	3	5	15	CAC	20	7
76281	7	13348511	13348528	3	6	18	CAA	21	8
76284	7	13349453	13349464	2	6	12	TG	28	4
76285	7	13349659	13349674	4	4	16	TTTG	35	5
76289	7	13355285	13355296	2	6	12	GT	38	3
76292	7	13361354	13361392	3	13	39	ATC	24	7
76296	7	13373794	13373813	2	10	20	AC	15	3
76297	7	13390365	13390406	2	21	42	AG	9	5
76304	7	13398065	13398094	2	15	30	CA	12	9
76305	7	13403141	13403156	2	8	16	CT	27	4
76306	7	13404168	13404213	2	23	46	TG	8	10
76313	7	13430970	13430981	2	6	12	GT	25	4
76319	7	13456267	13456292	2	13	26	CA	20	10
76321	7	13457951	13457964	2	7	14	AC	29	5
76322	7	13458208	13458239	2	16	32	AC	16	13
76323	7	13459227	13459246	4	5	20	ATTC	31	3
76324	7	13459991	13460002	2	6	12	CA	21	3
76326	7	13462094	13462121	2	14	28	TG	12	6
76328	7	13463758	13463777	2	10	20	TC	34	9
76330	7	13466392	13466417	2	13	26	TG	7	12
76337	7	13486310	13486333	2	12	24	CA	7	7
76345	7	13505614	13505641	2	14	28	GT	18	12
76350	7	13507583	13507597	3	5	15	CTC	25	5
76351	7	13508536	13508549	2	7	14	AC	25	5
76353	7	13510897	13510911	3	5	15	GAT	25	5
76354	7	13512079	13512112	2	17	34	TG	13	13
76356	7	13521384	13521401	2	9	18	TG	18	7
76359	7	13530597	13530608	2	6	12	AC	10	5
76360	7	13530611	13530624	2	7	14	AC	25	7
76362	7	13530824	13530861	2	19	38	TG	10	10
76365	7	13539527	13539547	3	7	21	GAA	11	5
76368	7	13539941	13539955	3	5	15	TAT	30	3
76376	7	13556999	13557026	2	14	28	TG	13	12
76380	7	13568212	13568223	2	6	12	AC	21	3
76383	7	13578346	13578373	2	14	28	CA	8	13
76391	7	13608789	13608808	2	10	20	CA	23	3
76395	7	13619701	13619728	2	14	28	TG	25	9
76404	7	13633848	13633861	2	7	14	TG	14	6
76405	7	13633864	13633879	2	8	16	TG	21	7
76407	7	13638621	13638648	2	14	28	GA	14	18
76410	7	13643206	13643219	2	7	14	CA	27	3
76421	7	13651917	13651930	2	7	14	GT	13	3
76422	7	13651935	13651948	2	7	14	TG	17	4
76428	7	13655296	13655307	2	6	12	TG	18	3
76429	7	13656227	13656246	2	10	20	AC	14	4
76432	7	13658122	13658141	2	10	20	TG	23	6
76433	7	13658268	13658283	2	8	16	GT	15	3

76434	7	13658443	13658460	2	9	18	GT	23	3
76435	7	13658594	13658615	2	11	22	GT	22	4
76437	7	13659608	13659621	2	7	14	AC	21	3
76443	7	13668195	13668216	2	11	22	CA	11	8
76446	7	13675689	13675702	2	7	14	AC	17	3
76447	7	13675883	13675920	2	19	38	GT	11	9
76464	7	13687801	13687812	2	6	12	TG	29	3
76465	7	13689178	13689199	2	11	22	TG	14	8
76474	7	13699919	13699932	2	7	14	GT	19	10
76475	7	13700326	13700349	3	8	24	GAG	26	5
76478	7	13703519	13703542	3	8	24	CTT	23	8
76479	7	13703863	13703878	2	8	16	TG	29	4
76484	7	13704573	13704600	2	14	28	AC	15	13
76485	7	13704812	13704823	2	6	12	TG	16	3
76487	7	13712793	13712814	2	11	22	TG	21	6
76488	7	13714896	13714913	2	9	18	AC	24	7
76494	7	13725229	13725276	2	24	48	TG	8	11
76501	7	13760481	13760494	2	7	14	GT	26	3
76502	7	13760652	13760701	2	25	50	CA	15	18
76504	7	13762375	13762392	2	9	18	CA	18	3
76513	7	13776684	13776701	2	9	18	AC	23	19
76516	7	13781811	13781832	2	11	22	CA	22	11
76519	7	13787027	13787040	2	7	14	CT	22	3
76522	7	13789376	13789415	2	20	40	TG	8	10
76525	7	13800668	13800679	2	6	12	CA	24	3
76527	7	13802045	13802058	2	7	14	CA	26	3
76529	7	13809556	13809581	2	13	26	TG	15	9
76532	7	13813189	13813204	2	8	16	CA	22	5
76535	7	13815652	13815675	2	12	24	GT	27	6
76540	7	13821985	13822000	2	8	16	GT	13	6
76541	7	13822806	13822817	2	6	12	TG	25	4
76544	7	13827052	13827083	2	16	32	TG	14	9
76545	7	13827584	13827599	2	8	16	GT	18	3
76548	7	13830013	13830024	2	6	12	AG	22	3
76550	7	13832753	13832768	2	8	16	CA	18	8
76551	7	13832771	13832784	2	7	14	CA	6	5
76552	7	13834757	13834772	2	8	16	CA	9	3
76554	7	13837783	13837794	2	6	12	AC	22	3
76555	7	13840412	13840447	2	18	36	GT	8	11
76560	7	13849053	13849074	2	11	22	TG	13	6
76562	7	13849117	13849140	2	12	24	TG	8	7
76567	7	13857049	13857080	2	16	32	TG	7	7
76568	7	13857246	13857281	2	18	36	GT	8	10
76569	7	13857465	13857504	2	20	40	CA	7	11
76570	7	13857665	13857684	2	10	20	AC	17	5
76571	7	13859574	13859589	2	8	16	TC	26	4
76573	7	13861046	13861077	2	16	32	AC	18	9
76584	7	13877377	13877400	4	6	24	GGAT	25	5
76586	7	13880917	13880932	2	8	16	CA	19	9
76588	7	13881273	13881298	2	13	26	TG	19	6
76589	7	13882304	13882321	2	9	18	GT	22	4

76590	7	13882589	13882604	2	8	16	GT	21	9
76591	7	13882607	13882620	2	7	14	GT	20	8
76592	7	13882680	13882709	2	15	30	GT	11	8
76593	7	13890007	13890020	2	7	14	CA	27	6
76595	7	13890296	13890333	2	19	38	AC	9	9
76596	7	13895116	13895145	2	15	30	GT	14	7
76605	7	13912613	13912632	2	10	20	AC	15	6
76608	7	13922973	13922992	2	10	20	CA	24	5
76617	7	13944103	13944126	3	8	24	AGC	27	11
76622	7	13954058	13954089	2	16	32	AC	12	8
76623	7	13954442	13954467	2	13	26	GT	22	4
76627	7	13960709	13960720	2	6	12	GT	23	3
76636	7	13985066	13985101	2	18	36	TG	17	9
76638	7	13987210	13987227	2	9	18	GA	23	7
76640	7	13989355	13989370	2	8	16	CA	6	11
76651	7	13999391	13999434	2	22	44	TG	15	10
76666	7	14034084	14034121	2	19	38	CT	6	5
76667	7	14035325	14035338	2	7	14	TG	23	6
76668	7	14035343	14035356	2	7	14	TG	18	3
76671	7	14037573	14037588	2	8	16	CA	27	6
76672	7	14037705	14037716	2	6	12	AC	21	4
76691	7	14060873	14060892	2	10	20	TG	20	5
76698	7	14080945	14080992	2	24	48	AC	7	8
76699	7	14081895	14081910	2	8	16	CT	16	3
76701	7	14084401	14084430	2	15	30	AC	7	5
76702	7	14086034	14086049	2	8	16	GT	23	8
76704	7	14092254	14092269	4	4	16	TTTA	26	6
76711	7	14107635	14107652	2	9	18	CA	26	5
76717	7	14115568	14115581	2	7	14	TG	22	4
76721	7	14118343	14118358	2	8	16	CA	14	3
76722	7	14118510	14118539	2	15	30	CA	10	10
76723	7	14118726	14118751	2	13	26	GT	12	11
76725	7	14122683	14122698	4	4	16	CCCT	18	6
76726	7	14124732	14124751	4	5	20	TTTA	17	4
76728	7	14126041	14126054	2	7	14	AC	22	4
76729	7	14126123	14126152	2	15	30	AC	14	6
76734	7	14131197	14131212	4	4	16	AAAG	9	4
76740	7	14135342	14135365	2	12	24	TG	12	9
76751	7	14147405	14147430	2	13	26	AC	15	6
76758	7	14167630	14167649	2	10	20	CA	20	10
76759	7	14167830	14167847	2	9	18	GT	17	9
76760	7	14167850	14167863	2	7	14	GT	17	12
76763	7	14174423	14174438	2	8	16	TG	23	3
76773	7	14188134	14188161	2	14	28	GT	9	9
76775	7	14192537	14192548	2	6	12	CA	28	3
76777	7	14193948	14193973	2	13	26	GT	21	9
76778	7	14199820	14199857	2	19	38	CA	18	7
76779	7	14202041	14202098	2	29	58	AC	9	14
76780	7	14204103	14204116	2	7	14	TG	23	3
76783	7	14206833	14206844	2	6	12	CT	31	3
76802	7	14240818	14240845	4	7	28	AGTC	29	5

76803	7	14241384	14241403	2	10	20	AC	10	6
76804	7	14243052	14243067	2	8	16	CA	22	7
76805	7	14243162	14243185	2	12	24	AC	15	12
76806	7	14243354	14243373	2	10	20	CA	7	5
76811	7	14256431	14256446	4	4	16	GAGG	12	4
76814	7	14258559	14258576	2	9	18	AC	22	5
76816	7	14260853	14260876	2	12	24	AG	11	12
76833	7	14293122	14293149	2	14	28	CA	16	8
76836	7	14296958	14296973	2	8	16	AC	14	6
76838	7	14301416	14301427	2	6	12	GT	25	7
76841	7	14304363	14304394	2	16	32	GT	6	5
76845	7	14315061	14315096	4	9	36	AGGA	17	11
76857	7	14330500	14330513	2	7	14	GA	14	5
76862	7	14336974	14336999	2	13	26	GT	14	17
76865	7	14342686	14342705	2	10	20	CA	23	16
76867	7	14344215	14344226	2	6	12	TC	24	4
76868	7	14345597	14345610	2	7	14	AC	20	4
76869	7	14347090	14347115	2	13	26	AC	13	8
76870	7	14347582	14347613	2	16	32	TG	11	11
76879	7	14357210	14357225	2	8	16	CA	27	8
76880	7	14358367	14358382	2	8	16	TG	19	7
76881	7	14359411	14359446	3	12	36	GAA	28	8
76882	7	14361375	14361390	4	4	16	CACG	16	7
76885	7	14362314	14362340	3	9	27	GAG	6	5
76887	7	14362921	14362944	3	8	24	AAC	23	9
76891	7	14372007	14372018	2	6	12	AT	17	5
76895	7	14374099	14374118	2	10	20	TG	6	3
76896	7	14374162	14374176	3	5	15	AGA	16	7
76897	7	14374225	14374248	2	12	24	CA	14	10
76900	7	14374570	14374593	4	6	24	TCCA	13	3
76903	7	14375462	14375479	3	6	18	TCT	32	3
76909	7	14399319	14399342	2	12	24	AC	18	4
76913	7	14406467	14406478	2	6	12	CA	14	3
76916	7	14406608	14406627	2	10	20	GT	22	7
76917	7	14406815	14406826	2	6	12	GT	13	8
76923	7	14419333	14419352	4	5	20	AAAG	30	4
76928	7	14427092	14427103	2	6	12	AC	16	4
76929	7	14432347	14432364	2	9	18	CA	19	9
76930	7	14432407	14432418	2	6	12	AC	26	4
76935	7	14445142	14445162	3	7	21	CTC	16	3
76936	7	14448395	14448406	2	6	12	GT	21	11
76937	7	14450669	14450690	2	11	22	GT	32	6
76940	7	14456324	14456344	3	7	21	GCT	28	7
76943	7	14467651	14467680	2	15	30	AC	15	11
76944	7	14467826	14467877	2	26	52	TG	8	6
76946	7	14468507	14468536	2	15	30	CA	10	10
76949	7	14485985	14486012	2	14	28	CA	11	7
76950	7	14486439	14486468	2	15	30	CA	7	10
76952	7	14487771	14487786	2	8	16	TG	20	4
76954	7	14489594	14489605	2	6	12	GT	24	7
76955	7	14489780	14489817	2	19	38	CA	16	12

76956	7	14491778	14491803	2	13	26	AC	21	7
76957	7	14491970	14491985	2	8	16	CA	20	10
76958	7	14493640	14493661	2	11	22	TG	20	8
76960	7	14502433	14502458	2	13	26	TG	16	12
76962	7	14504533	14504560	2	14	28	TC	9	10
76964	7	14506964	14506987	2	12	24	CA	22	10
76968	7	14519838	14519859	2	11	22	GT	18	9
76969	7	14521145	14521164	2	10	20	GT	14	8
76971	7	14522822	14522837	2	8	16	TG	23	6
76972	7	14522884	14522907	3	8	24	CCT	19	5
76983	7	14538752	14538783	2	16	32	TG	25	7
76988	7	14543846	14543867	2	11	22	CA	11	13
76989	7	14543908	14543921	2	7	14	TG	29	3
76992	7	14546974	14546993	2	10	20	CA	15	10
77002	7	14560178	14560191	2	7	14	AG	30	5
77006	7	14564577	14564598	2	11	22	AG	9	7
77012	7	14570484	14570503	2	10	20	GA	21	6
77013	7	14570911	14570922	2	6	12	GT	29	4
77017	7	14575150	14575161	2	6	12	GT	11	11
77019	7	14575376	14575403	2	14	28	TG	14	7
77020	7	14575598	14575612	3	5	15	TTG	24	3
77022	7	14576415	14576430	4	4	16	GCGG	24	5
77023	7	14577218	14577231	2	7	14	TG	20	8
77026	7	14579040	14579059	2	10	20	AC	16	9
77028	7	14579491	14579512	2	11	22	GT	16	5
77029	7	14579733	14579748	2	8	16	AC	17	5
77033	7	14581523	14581558	2	18	36	GA	11	12
77034	7	14581970	14581989	2	10	20	CA	10	7
77036	7	14583996	14584015	2	10	20	AC	17	8
77037	7	14585665	14585682	2	9	18	GT	25	11
77040	7	14590741	14590768	2	14	28	AC	13	9
77042	7	14592780	14592803	4	6	24	CAGA	23	9
77043	7	14593197	14593226	3	10	30	CCT	15	8
77049	7	14598628	14598663	2	18	36	GT	16	13
77050	7	14598861	14598882	2	11	22	CA	19	6
77051	7	14603170	14603187	3	6	18	CCA	25	3
77053	7	14608762	14608777	2	8	16	AC	17	5
77055	7	14610199	14610222	3	8	24	AGA	24	6
77056	7	14611286	14611315	2	15	30	CA	24	11
77059	7	14613452	14613471	2	10	20	AC	17	8
77060	7	14618086	14618111	2	13	26	CA	21	10
77061	7	14618461	14618498	2	19	38	TG	24	12
77063	7	14621761	14621782	2	11	22	AC	20	7
77067	7	14624476	14624497	2	11	22	CA	22	10
77068	7	14624726	14624741	2	8	16	GT	30	4
77069	7	14626914	14626928	3	5	15	CTT	25	3
77073	7	14637861	14637875	3	5	15	TAG	28	3
77074	7	14642134	14642163	2	15	30	CA	14	10
77075	7	14642546	14642571	2	13	26	AC	16	11
77079	7	14652763	14652776	2	7	14	TG	30	12
77080	7	14655597	14655623	3	9	27	TCA	26	4

77083	7	14659127	14659148	2	11	22	TG	21	9
77084	7	14659356	14659385	2	15	30	TG	8	11
77088	7	14664165	14664185	3	7	21	CTT	18	4
77090	7	14665330	14665345	4	4	16	GAGG	21	3
77091	7	14665533	14665546	2	7	14	TC	12	5
77094	7	14665920	14665931	2	6	12	CA	24	4
77099	7	14685166	14685197	2	16	32	CA	17	10
77102	7	14689359	14689396	2	19	38	TG	16	12
77107	7	14715082	14715143	2	31	62	TG	16	13
77108	7	14715629	14715648	2	10	20	TG	17	3
77109	7	14715682	14715695	2	7	14	TG	20	6
77110	7	14715834	14715851	2	9	18	AC	17	5
77111	7	14716266	14716289	2	12	24	GT	13	7
77113	7	14717155	14717174	2	10	20	GT	21	7
77117	7	14727111	14727131	3	7	21	GAG	31	5
77119	7	14730244	14730259	4	4	16	CCTC	31	3
77120	7	14731702	14731717	2	8	16	AC	15	3
77132	7	14757850	14757864	3	5	15	TTA	24	3
77138	7	14793612	14793637	2	13	26	AG	22	6
77158	7	14818048	14818063	4	4	16	AAAT	19	3
77159	7	14820152	14820175	4	6	24	TTTC	22	7
77161	7	14822074	14822093	2	10	20	TC	10	3
77164	7	14824725	14824744	2	10	20	TG	8	3
77167	7	14830107	14830128	2	11	22	TG	17	7
77168	7	14830791	14830804	2	7	14	GT	22	3
77169	7	14831940	14831955	2	8	16	GT	28	4
77172	7	14836765	14836782	2	9	18	CA	17	7
77174	7	14837008	14837041	2	17	34	AC	11	11
77176	7	14843829	14843862	2	17	34	TG	15	10
77179	7	14848767	14848784	2	9	18	AC	24	6
77182	7	14849707	14849748	2	21	42	CA	6	9
77191	7	14855942	14855959	2	9	18	GT	17	7
77192	7	14858130	14858145	2	8	16	TG	31	7
77194	7	14859191	14859210	2	10	20	GT	22	5
77196	7	14861654	14861669	2	8	16	AC	13	8
77198	7	14861972	14861999	2	14	28	AC	6	4
77199	7	14862177	14862192	2	8	16	AC	26	5
77200	7	14862334	14862383	2	25	50	TG	6	10
77204	7	14869854	14869865	2	6	12	TC	27	4
77206	7	14870684	14870723	2	20	40	GT	12	9
77211	7	14878952	14878969	2	9	18	GT	22	3
77212	7	14883556	14883583	2	14	28	GT	19	6
77213	7	14884943	14884954	2	6	12	CA	29	3
77217	7	14891464	14891481	2	9	18	CA	15	12
77218	7	14893887	14893900	2	7	14	TG	24	6
77221	7	14901563	14901574	2	6	12	GT	33	4
77222	7	14904320	14904340	3	7	21	CAG	16	3
77225	7	14912374	14912417	2	22	44	AC	15	18
77227	7	14912894	14912929	2	18	36	CA	8	9
77228	7	14913098	14913115	2	9	18	TG	19	4
77231	7	14916337	14916356	2	10	20	CT	10	7

77232	7	14916393	14916407	3	5	15	TCC	20	3
77235	7	14918919	14918946	2	14	28	AC	12	11
77236	7	14919142	14919169	2	14	28	AC	25	7
77238	7	14922875	14922890	2	8	16	AC	19	3
77239	7	14922932	14922963	2	16	32	TG	8	12
77242	7	14923265	14923278	2	7	14	TC	21	4
77243	7	14923341	14923352	2	6	12	CA	28	3
77245	7	14924556	14924567	2	6	12	AC	27	6
77246	7	14927871	14927884	2	7	14	CA	24	3
77248	7	14930462	14930479	2	9	18	AC	22	3
77263	7	14941977	14941996	2	10	20	CT	15	9
77264	7	14942196	14942241	2	23	46	CA	12	11
77268	7	14951428	14951453	2	13	26	GT	10	3
77270	7	14951818	14951839	2	11	22	TG	9	8
77271	7	14956256	14956273	2	9	18	TG	20	6
77272	7	14957597	14957620	2	12	24	TG	14	8
77276	7	14963543	14963564	2	11	22	AC	17	3
77278	7	14968070	14968089	2	10	20	GA	22	3
77280	7	14970875	14970886	2	6	12	TG	27	4
77281	7	14974391	14974424	2	17	34	TG	8	10
77282	7	14976358	14976371	2	7	14	GT	8	8
77285	7	14976587	14976604	2	9	18	AC	8	8
77286	7	14979977	14980004	2	14	28	AC	17	9
77287	7	14981297	14981312	2	8	16	GT	22	8
77292	7	14982959	14982986	2	14	28	AC	9	11
77293	7	14984952	14984979	2	14	28	AC	14	10
77297	7	14985575	14985592	2	9	18	TG	20	10
77298	7	14985654	14985669	2	8	16	GT	20	11
77299	7	14985670	14985685	2	8	16	GA	23	12
77300	7	14991800	14991811	2	6	12	AG	24	4
77308	7	15001244	15001283	2	20	40	GT	18	10
77313	7	15007239	15007260	2	11	22	CA	19	7
77314	7	15009131	15009150	2	10	20	CT	18	5
77316	7	15009529	15009552	2	12	24	AC	14	13
77317	7	15010225	15010252	2	14	28	GA	14	5
77319	7	15010649	15010674	2	13	26	AC	13	7
77326	7	15014561	15014574	2	7	14	CA	23	5
77327	7	15014632	15014643	2	6	12	GT	23	7
77334	7	15017939	15017950	2	6	12	TG	15	4
77335	7	15019882	15019899	2	9	18	CT	15	4
77337	7	15023513	15023528	2	8	16	AC	18	13
77340	7	15026898	15026917	2	10	20	AC	12	5
77343	7	15027892	15027905	2	7	14	CT	16	3
77344	7	15027916	15027927	2	6	12	CA	7	4
77345	7	15027929	15027944	2	8	16	AC	25	8
77348	7	15030154	15030169	2	8	16	CA	20	3
77349	7	15030309	15030324	2	8	16	AC	21	8
77351	7	15032521	15032544	3	8	24	TCC	23	8
77352	7	15032659	15032674	2	8	16	AC	24	10
77356	7	15040758	15040775	3	6	18	GAG	26	3
77357	7	15041247	15041288	2	21	42	CA	11	11

77359	7	15043813	15043832	2	10	20	CA	21	6
77362	7	15046023	15046050	2	14	28	GT	7	5
77370	7	15061653	15061674	2	11	22	AC	20	5
77374	7	15070642	15070662	3	7	21	TGG	23	3
77377	7	15076378	15076397	2	10	20	AC	23	11
77378	7	15076421	15076432	2	6	12	GT	28	6
77379	7	15079880	15079905	2	13	26	TG	16	7
77386	7	15088237	15088248	2	6	12	AC	36	3
77390	7	15091872	15091919	2	24	48	TG	9	9
77393	7	15092080	15092095	2	8	16	GT	8	11
77395	7	15094642	15094653	2	6	12	GA	20	3
77396	7	15097858	15097869	2	6	12	AC	28	3
77398	7	15102712	15102735	2	12	24	CA	18	3
77399	7	15102844	15102857	2	7	14	AC	21	3
77402	7	15104909	15104940	2	16	32	TG	17	10
77408	7	15106857	15106892	2	18	36	CA	7	10
77409	7	15107892	15107905	2	7	14	TG	25	7
77410	7	15108042	15108055	2	7	14	CA	22	6
77411	7	15108291	15108324	2	17	34	AC	17	10
77413	7	15109979	15110016	2	19	38	GT	8	11
77415	7	15114195	15114212	3	6	18	TGC	29	8
77416	7	15115113	15115136	4	6	24	TTTG	9	7
77419	7	15117282	15117309	2	14	28	AC	14	5
77420	7	15117434	15117454	3	7	21	CAG	23	8
77422	7	15119290	15119331	3	14	42	AGG	8	8
77427	7	15122344	15122355	2	6	12	AT	20	4
77428	7	15124198	15124227	2	15	30	GA	16	10
77429	7	15124491	15124510	2	10	20	CA	7	6
77432	7	15125067	15125080	2	7	14	GA	24	7
77433	7	15125463	15125480	2	9	18	CA	21	20
77437	7	15129482	15129507	2	13	26	AC	19	14
77438	7	15129737	15129748	2	6	12	AG	21	3
77446	7	15159541	15159572	2	16	32	AC	9	10
77447	7	15163419	15163446	2	14	28	AC	14	12
77449	7	15166655	15166672	3	6	18	AGC	27	8
77450	7	15166751	15166768	3	6	18	TGC	8	7
77457	7	15197694	15197713	2	10	20	AC	22	3
77460	7	15204791	15204802	2	6	12	AC	25	8
77461	7	15204806	15204821	2	8	16	CA	23	8
77466	7	15227114	15227129	2	8	16	CT	23	8
77473	7	15242550	15242573	2	12	24	TC	25	7
77479	7	15269845	15269856	2	6	12	AC	27	3
77482	7	15275908	15275955	2	24	48	CA	7	7
77498	7	15333818	15333829	2	6	12	GT	22	5
77499	7	15334610	15334649	4	10	40	CTAT	18	14
77500	7	15334794	15334813	2	10	20	GT	14	9
77502	7	15344445	15344456	2	6	12	GT	33	3
77506	7	15350430	15350455	2	13	26	CA	16	15
77512	7	15381717	15381730	2	7	14	TG	30	3
77516	7	15386869	15386895	3	9	27	CTC	13	9
77517	7	15387923	15387940	2	9	18	AC	20	10

77519	7	15388266	15388301	2	18	36	AC	8	9
77523	7	15401722	15401735	2	7	14	AT	13	8
77527	7	15411169	15411182	2	7	14	AC	22	6
77528	7	15412200	15412211	2	6	12	CT	22	4
77529	7	15417228	15417248	3	7	21	GCA	34	5
77533	7	15426202	15426216	3	5	15	CCT	13	3
77544	7	15451260	15451274	3	5	15	TCA	28	3
77547	7	15463913	15463926	2	7	14	TA	17	3
77549	7	15486706	15486731	2	13	26	GT	19	13
77552	7	15493772	15493791	2	10	20	GT	15	16
77558	7	15511607	15511622	4	4	16	AATA	32	3
77559	7	15512124	15512141	2	9	18	CA	8	7
77560	7	15512707	15512736	2	15	30	GT	6	5
77567	7	15522848	15522863	2	8	16	TG	15	8
77568	7	15523129	15523144	2	8	16	GT	21	4
77570	7	15524881	15524894	2	7	14	GA	17	5
77571	7	15525508	15525522	3	5	15	GCA	25	9
77573	7	15527887	15527904	2	9	18	GT	23	5
77574	7	15528129	15528152	2	12	24	CA	13	11
77575	7	15530306	15530329	2	12	24	CA	22	12
77580	7	15538472	15538491	2	10	20	AG	7	5
77581	7	15538705	15538726	2	11	22	GT	26	22
77584	7	15539792	15539806	3	5	15	CAG	13	3
77591	7	15548361	15548381	3	7	21	GCT	8	3
77618	7	15634242	15634273	2	16	32	CA	11	11
77620	7	15637137	15637152	2	8	16	GT	28	3
77621	7	15639382	15639409	2	14	28	TG	12	4
77622	7	15640869	15640892	2	12	24	GA	6	3
77627	7	15645835	15645862	4	7	28	AATA	12	10
77632	7	15656082	15656101	2	10	20	AG	24	11
77633	7	15657268	15657279	2	6	12	AT	23	3
77634	7	15658953	15658972	2	10	20	CA	20	5
77635	7	15660728	15660742	3	5	15	CCT	27	4
77637	7	15660937	15660952	2	8	16	GT	20	4
77639	7	15661307	15661338	2	16	32	AC	6	8
77640	7	15663715	15663732	2	9	18	CA	18	11
77647	7	15674332	15674345	2	7	14	CA	20	9
77649	7	15675648	15675677	2	15	30	CA	21	12
77651	7	15676063	15676074	2	6	12	CA	15	13
77654	7	15686033	15686046	2	7	14	AC	26	4
77655	7	15686362	15686375	2	7	14	TC	22	6
77660	7	15694272	15694287	4	4	16	TTCC	38	3
77661	7	15698191	15698218	4	7	28	CCAT	25	5
77663	7	15700542	15700559	2	9	18	GA	25	5
77667	7	15706398	15706411	2	7	14	TG	28	3
77668	7	15706640	15706669	2	15	30	GA	11	9
77678	7	15735760	15735771	2	6	12	CA	24	7
77679	7	15735774	15735787	2	7	14	CA	21	4
77681	7	15736513	15736524	2	6	12	TG	29	4
77682	7	15737779	15737790	2	6	12	CA	11	4
77684	7	15738227	15738250	2	12	24	TC	22	6

77686	7	15741381	15741394	2	7	14	AC	31	4
77688	7	15743084	15743105	2	11	22	AC	13	8
77689	7	15744452	15744477	2	13	26	CA	10	7
77691	7	15750218	15750237	2	10	20	TG	18	7
77692	7	15750969	15750995	3	9	27	GGA	9	5
77693	7	15755059	15755076	2	9	18	AG	19	4
77696	7	15765012	15765037	2	13	26	AC	9	11
77698	7	15765844	15765857	2	7	14	AC	24	3
77699	7	15774185	15774218	2	17	34	GT	17	12
77700	7	15774682	15774713	2	16	32	GT	11	13
77704	7	15777101	15777112	2	6	12	AG	30	3
77706	7	15779008	15779022	3	5	15	AAG	26	5
77710	7	15785392	15785421	2	15	30	GT	15	6
77713	7	15795171	15795198	4	7	28	CAAC	18	5
77714	7	15795224	15795247	4	6	24	CAAC	19	6
77716	7	15803998	15804017	2	10	20	AC	26	10
77721	7	15822508	15822525	3	6	18	TGC	29	3
77722	7	15825221	15825244	2	12	24	CA	26	5
77724	7	15833068	15833085	2	9	18	CT	29	3
77726	7	15838906	15838917	2	6	12	TC	28	4
77731	7	15844762	15844777	2	8	16	CA	22	5
77739	7	15852075	15852090	2	8	16	CA	9	6
77741	7	15855510	15855557	2	24	48	CA	11	10
77742	7	15855963	15855982	4	5	20	CCAG	21	4
77746	7	15856242	15856257	2	8	16	AG	24	5
77748	7	15857277	15857288	2	6	12	GT	13	6
77751	7	15859612	15859629	2	9	18	GT	26	3
77756	7	15879155	15879180	2	13	26	TG	7	6
77758	7	15887509	15887522	2	7	14	TG	25	5
77759	7	15889369	15889388	4	5	20	GCCT	23	3
77765	7	15913914	15913925	2	6	12	AG	30	3
77768	7	15922479	15922490	2	6	12	TA	11	6
77770	7	15922780	15922797	3	6	18	GGA	15	4
77776	7	15938747	15938778	2	16	32	AC	13	9
77779	7	15953925	15953936	2	6	12	AC	24	3
77780	7	15954128	15954141	2	7	14	AC	17	5
77781	7	15954785	15954812	4	7	28	ACAG	20	10
77782	7	15956142	15956171	2	15	30	AC	12	11
77785	7	15959705	15959722	2	9	18	TG	25	9
77798	7	15971793	15971816	3	8	24	GGA	11	7
77801	7	15981166	15981205	2	20	40	GT	11	10
77802	7	15981676	15981691	2	8	16	GT	25	4
77803	7	15984246	15984269	2	12	24	CA	6	8
77806	7	15989403	15989424	2	11	22	CA	25	12
77809	7	15994599	15994614	2	8	16	TG	13	13
77810	7	15994617	15994630	2	7	14	TG	17	12
77811	7	16004578	16004591	2	7	14	CT	24	3
77821	7	16017931	16017942	2	6	12	CA	22	3
77824	7	16021368	16021393	2	13	26	CA	10	9
77825	7	16028222	16028259	2	19	38	TG	6	5
77826	7	16034905	16034916	2	6	12	AC	34	5

77833	7	16061737	16061757	3	7	21	GAG	29	3
77835	7	16066488	16066503	4	4	16	TCCA	20	7
77839	7	16078368	16078397	2	15	30	CA	6	7
77840	7	16078803	16078814	2	6	12	TG	8	3
77841	7	16079749	16079762	2	7	14	AC	29	7
77847	7	16086723	16086742	2	10	20	TG	16	8
77852	7	16092071	16092086	4	4	16	GCTC	21	3
77854	7	16093757	16093774	3	6	18	GGT	23	4
77856	7	16099360	16099387	2	14	28	GT	11	8
77858	7	16104368	16104387	4	5	20	CTAT	27	6
77860	7	16107190	16107205	2	8	16	TG	22	9
77864	7	16130083	16130104	2	11	22	CA	7	7
77868	7	16137932	16137961	2	15	30	TC	21	7
77869	7	16139471	16139485	3	5	15	AAT	8	4
77870	7	16141317	16141344	2	14	28	GT	21	8
77875	7	16148569	16148592	2	12	24	TG	7	5
77881	7	16159959	16159970	2	6	12	TG	25	3
77882	7	16163423	16163440	2	9	18	AC	20	6
77887	7	16169643	16169658	2	8	16	GT	28	9
77888	7	16171860	16171879	2	10	20	TG	16	5
77890	7	16174408	16174431	2	12	24	GT	18	8
77892	7	16176007	16176032	2	13	26	TG	18	7
77897	7	16188122	16188133	2	6	12	GT	27	3
77898	7	16190552	16190565	2	7	14	GT	22	4
77901	7	16194600	16194623	2	12	24	CA	9	7
77902	7	16196752	16196778	3	9	27	GGA	21	8
77903	7	16196960	16197001	2	21	42	GT	11	12
77911	7	16220427	16220442	4	4	16	AAAT	22	8
77915	7	16231373	16231394	2	11	22	AC	15	5
77925	7	16250536	16250549	2	7	14	TC	11	13
77926	7	16250550	16250565	2	8	16	AC	12	13
77927	7	16250860	16250880	3	7	21	GAG	20	6
77928	7	16251391	16251408	2	9	18	TC	6	8
77933	7	16254165	16254178	2	7	14	AC	20	19
77934	7	16256060	16256085	2	13	26	CA	15	7
77939	7	16270300	16270335	3	12	36	ATC	22	8
77949	7	16288121	16288156	2	18	36	CA	15	17
77959	7	16293914	16293927	2	7	14	TC	25	3
77963	7	16300693	16300707	3	5	15	GCA	25	3
77970	7	16307808	16307823	2	8	16	GT	23	9
77975	7	16312112	16312127	2	8	16	GT	26	11
77977	7	16313149	16313170	2	11	22	GT	18	5
77981	7	16333575	16333590	2	8	16	GT	7	10
77992	7	16340614	16340635	2	11	22	GT	16	12
77997	7	16350997	16351010	2	7	14	GA	17	7
78001	7	16353498	16353529	2	16	32	GT	7	12
78005	7	16363480	16363493	2	7	14	GA	16	3
78006	7	16366961	16366978	2	9	18	CA	18	4
78008	7	16367538	16367555	3	6	18	AAC	26	4
78012	7	16370602	16370619	2	9	18	TC	20	7
78027	7	16388171	16388185	3	5	15	TGT	26	3

78033	7	16393848	16393861	2	7	14	GA	21	3
78036	7	16399811	16399838	2	14	28	AC	26	10
78045	7	16410440	16410453	2	7	14	TG	10	8
78046	7	16410997	16411008	2	6	12	AC	18	8
78048	7	16412210	16412227	2	9	18	GT	19	4
78052	7	16419924	16419939	4	4	16	GTCA	25	3
78053	7	16420676	16420687	2	6	12	TA	19	7
78054	7	16422889	16422902	2	7	14	TG	29	5
78058	7	16428943	16428968	2	13	26	GT	19	7
78059	7	16429155	16429172	2	9	18	TA	11	8
78064	7	16431732	16431753	2	11	22	GA	25	3
78065	7	16433154	16433171	2	9	18	CA	21	7
78071	7	16439341	16439354	2	7	14	AG	22	3
78072	7	16439622	16439637	2	8	16	AC	31	3
78082	7	16457076	16457099	4	6	24	TGTA	16	7
78086	7	16470540	16470551	2	6	12	AC	19	4
78087	7	16471411	16471444	2	17	34	CA	18	12
78094	7	16478487	16478514	2	14	28	CA	14	12
78095	7	16482110	16482161	2	26	52	AC	7	12
78104	7	16507524	16507538	3	5	15	CTG	25	5
78106	7	16510721	16510732	2	6	12	CT	11	5
78107	7	16510733	16510746	2	7	14	CA	11	5
78109	7	16511224	16511253	2	15	30	CA	8	6
78111	7	16511418	16511431	2	7	14	TG	8	3
78113	7	16511662	16511695	2	17	34	TG	8	11
78114	7	16511797	16511826	3	10	30	AGG	17	4
78115	7	16511861	16511878	2	9	18	CA	19	11
78118	7	16512573	16512604	2	16	32	AC	14	8
78123	7	16516687	16516704	3	6	18	GCT	30	5
78124	7	16521079	16521092	2	7	14	CA	25	4
78126	7	16524839	16524856	2	9	18	TG	22	9
78128	7	16533048	16533063	4	4	16	GATG	16	4
78129	7	16533997	16534017	3	7	21	TGC	27	7
78135	7	16543190	16543215	2	13	26	GT	15	4
78136	7	16543389	16543404	4	4	16	CCAA	12	3
78139	7	16549418	16549439	2	11	22	TG	17	8
78140	7	16550558	16550593	2	18	36	CA	12	10
78143	7	16551160	16551171	2	6	12	TC	28	3
78146	7	16552990	16553005	2	8	16	AC	18	4
78148	7	16563215	16563242	2	14	28	CA	17	11
78150	7	16568441	16568464	3	8	24	GCT	26	3
78151	7	16570328	16570351	2	12	24	CA	22	9
78157	7	16575285	16575299	3	5	15	TCT	21	5
78162	7	16582560	16582577	2	9	18	TG	12	5
78166	7	16588503	16588518	2	8	16	CA	28	6
78167	7	16591917	16591932	4	4	16	AAAT	29	4
78173	7	16605742	16605756	3	5	15	CAG	27	4
78175	7	16611554	16611569	4	4	16	GTCT	24	5
78177	7	16614289	16614302	2	7	14	GA	24	3
78180	7	16625095	16625106	2	6	12	TG	29	4
78183	7	16630336	16630350	3	5	15	TCT	27	3

78185	7	16634291	16634308	3	6	18	GTT	28	8
78189	7	16637199	16637225	3	9	27	GGT	8	5
78191	7	16639206	16639227	2	11	22	TG	14	9
78200	7	16659851	16659864	2	7	14	TG	21	4
78204	7	16660632	16660651	2	10	20	CT	13	3
78209	7	16661663	16661688	2	13	26	TG	11	5
78210	7	16662760	16662773	2	7	14	TG	15	4
78212	7	16662996	16663015	2	10	20	AC	24	7
78217	7	16670692	16670703	2	6	12	CT	27	3
78222	7	16677431	16677446	2	8	16	AG	16	8
78226	7	16686200	16686214	3	5	15	GAG	27	4
78228	7	16691629	16691646	2	9	18	AC	18	16
78231	7	16695292	16695305	2	7	14	AC	18	12
78233	7	16697940	16697953	2	7	14	TG	24	3
78237	7	16705588	16705607	2	10	20	AC	35	8
78240	7	16713721	16713760	2	20	40	TG	12	10
78242	7	16726758	16726781	2	12	24	GT	7	7
78244	7	16729097	16729117	3	7	21	CTG	28	6
78245	7	16732942	16732965	4	6	24	GAGG	6	4
78248	7	16736828	16736859	2	16	32	GT	21	15
78250	7	16740050	16740070	3	7	21	GCA	28	8
78255	7	16750696	16750713	2	9	18	TC	9	4
78265	7	16769712	16769731	2	10	20	AG	20	11
78266	7	16769786	16769801	4	4	16	GAGG	7	4
78269	7	16775097	16775108	2	6	12	TC	16	3
78272	7	16781106	16781117	2	6	12	CT	29	5
78281	7	16794918	16794947	2	15	30	AC	16	9
78282	7	16796230	16796245	4	4	16	AAAG	26	3
78283	7	16798352	16798387	2	18	36	TG	15	10
78292	7	16815531	16815554	2	12	24	CA	16	5
78293	7	16818501	16818524	2	12	24	CA	17	6
78294	7	16818755	16818775	3	7	21	GGA	28	7
78295	7	16819106	16819125	2	10	20	GT	14	12
78296	7	16820596	16820615	2	10	20	AG	25	5
78297	7	16826904	16826921	2	9	18	AG	21	9
78298	7	16827112	16827137	2	13	26	TC	23	5
78307	7	16848867	16848914	2	24	48	TG	10	11
78309	7	16855293	16855306	2	7	14	AT	10	5
78312	7	16857176	16857193	2	9	18	GT	19	9
78313	7	16857354	16857373	2	10	20	CA	16	8
78319	7	16865440	16865457	2	9	18	GT	23	8
78322	7	16871918	16871935	3	6	18	CCT	25	4
78325	7	16889096	16889123	4	7	28	TATT	16	7
78328	7	16891215	16891230	2	8	16	TG	27	8
78330	7	16892287	16892316	3	10	30	CCT	13	7
78331	7	16892500	16892511	2	6	12	TG	26	5
78380	7	16921044	16921077	2	17	34	GT	14	15
78385	7	16929320	16929335	4	4	16	GGAG	17	4
78387	7	16935143	16935164	2	11	22	AC	30	6
78390	7	16941146	16941175	2	15	30	CT	13	6
78391	7	16944520	16944537	2	9	18	TC	24	5

78399	7	16989711	16989724	2	7	14	GA	24	14
78404	7	16996695	16996714	2	10	20	AC	20	13
78406	7	16996967	16996986	2	10	20	CA	16	9
78408	7	16997860	16997883	3	8	24	CCT	19	10
78409	7	16997979	16997994	2	8	16	CA	27	5
78410	7	16998322	16998342	3	7	21	GAG	29	5
78412	7	16999845	16999872	2	14	28	TG	12	13
78415	7	17018273	17018294	2	11	22	TG	7	8
78416	7	17018889	17018914	2	13	26	TC	7	4
78418	7	17018953	17018984	2	16	32	TC	7	6
78421	7	17038806	17038825	4	5	20	TGCC	27	5
78422	7	17046419	17046468	2	25	50	AC	6	5
78424	7	17049077	17049112	2	18	36	TG	7	7
78430	7	17066628	17066643	2	8	16	TG	16	5
78432	7	17081649	17081660	2	6	12	AC	29	3
78434	7	17089142	17089153	2	6	12	GA	24	3
78435	7	17095127	17095150	2	12	24	AG	12	5
78436	7	17095509	17095520	2	6	12	GT	26	3
78437	7	17101414	17101429	2	8	16	TC	23	3
78440	7	17113073	17113084	2	6	12	AC	30	4
78442	7	17124500	17124517	3	6	18	GAA	25	3
78447	7	17136366	17136383	2	9	18	AC	23	3
78448	7	17141372	17141389	2	9	18	CA	16	7
78451	7	17148752	17148769	2	9	18	TG	19	12
78452	7	17148771	17148786	2	8	16	GT	20	12
78453	7	17154244	17154257	2	7	14	CT	17	3
78455	7	17161818	17161845	2	14	28	CA	20	14
78458	7	17172158	17172179	2	11	22	AC	18	6
78468	7	17240479	17240498	2	10	20	AC	21	5
78471	7	17251699	17251728	2	15	30	TC	18	7
78475	7	17265831	17265850	4	5	20	TTTA	23	4
78485	7	17291425	17291448	2	12	24	GT	11	6
78488	7	17294862	17294882	3	7	21	CCT	27	3
78493	7	17303111	17303134	2	12	24	TG	11	8
78500	7	17324638	17324663	2	13	26	CA	17	12
78502	7	17331581	17331600	2	10	20	AC	23	12
78503	7	17332133	17332148	4	4	16	ATCC	20	4
78507	7	17368925	17368944	2	10	20	GT	7	7
78513	7	17377392	17377411	2	10	20	AC	14	7
78521	7	17386410	17386429	2	10	20	AG	20	4
78526	7	17407041	17407058	3	6	18	CCT	17	7
78528	7	17407569	17407586	2	9	18	TG	23	6
78529	7	17412907	17412918	2	6	12	TC	22	3
78533	7	17423014	17423025	2	6	12	GA	24	5
78538	7	17433426	17433437	2	6	12	TA	12	7
78539	7	17436153	17436190	2	19	38	AC	10	7
78541	7	17442148	17442165	2	9	18	TG	12	16
78551	7	17448215	17448250	2	18	36	AC	9	12
78552	7	17451024	17451037	2	7	14	AG	16	5
78553	7	17453090	17453103	2	7	14	AC	23	7
78561	7	17475014	17475041	2	14	28	TG	19	14

78566	7	17482973	17482996	2	12	24	CA	11	10
78568	7	17484992	17485009	2	9	18	CT	22	8
78569	7	17486385	17486428	4	11	44	TAGA	15	9
78573	7	17489355	17489376	2	11	22	TG	12	11
78575	7	17492175	17492194	2	10	20	GA	20	6
78576	7	17492395	17492412	2	9	18	TG	20	9
78587	7	17506042	17506068	3	9	27	TCT	25	5
78590	7	17510674	17510685	2	6	12	CA	18	4
78596	7	17525788	17525811	2	12	24	TG	8	8
78598	7	17526655	17526681	3	9	27	TCT	14	13
78603	7	17530255	17530270	4	4	16	TCTT	6	6
78605	7	17535609	17535620	2	6	12	TC	16	7
78606	7	17535622	17535635	2	7	14	CT	15	5
78609	7	17538178	17538235	2	29	58	TC	9	7
78616	7	17550651	17550666	2	8	16	CT	26	5
78624	7	17559188	17559211	3	8	24	AGC	22	11
78625	7	17559490	17559501	2	6	12	GT	23	3
78628	7	17564072	17564087	4	4	16	AGGA	24	3
78629	7	17568517	17568536	2	10	20	GT	19	7
78631	7	17569627	17569646	4	5	20	AGCT	9	4
78632	7	17571784	17571795	2	6	12	TG	25	5
78635	7	17578475	17578494	2	10	20	TG	18	11
78644	7	17600278	17600292	3	5	15	TCC	13	3
78646	7	17603283	17603306	2	12	24	AC	8	9
78647	7	17603668	17603679	2	6	12	CA	24	11
78648	7	17603682	17603695	2	7	14	CA	22	8
78653	7	17618948	17618974	3	9	27	TCC	20	8
78657	7	17645421	17645442	2	11	22	AC	24	4
78659	7	17647388	17647399	2	6	12	TG	27	3
78661	7	17650963	17650996	2	17	34	GT	13	9
78662	7	17651383	17651414	2	16	32	CA	10	7
78663	7	17657546	17657573	2	14	28	AC	20	15
78664	7	17658124	17658141	2	9	18	TG	23	3
78665	7	17659558	17659585	2	14	28	GT	12	11
78666	7	17660096	17660113	3	6	18	GAA	12	3
78670	7	17672282	17672299	2	9	18	GT	24	4
78672	7	17673984	17673997	2	7	14	GT	27	4
78673	7	17674571	17674586	2	8	16	AC	17	4
78675	7	17677135	17677146	2	6	12	TG	31	3
78676	7	17678775	17678800	2	13	26	GT	11	9
78680	7	17681895	17681912	3	6	18	TGA	30	6
78689	7	17700740	17700765	2	13	26	GT	23	9
78692	7	17701308	17701321	2	7	14	TG	21	5
78693	7	17701322	17701333	2	6	12	AG	17	6
78694	7	17701716	17701736	3	7	21	GCT	28	6
78697	7	17716022	17716033	2	6	12	GT	23	5
78698	7	17716911	17716964	2	27	54	CA	7	8
78699	7	17720487	17720504	2	9	18	CT	25	6
78700	7	17720570	17720593	2	12	24	TG	20	4
78705	7	17734415	17734430	2	8	16	CT	31	6
78713	7	17742047	17742061	3	5	15	GGC	17	6

78715	7	17744920	17744961	2	21	42	CA	14	13
78716	7	17745918	17745941	3	8	24	TGC	37	3
78717	7	17746579	17746590	2	6	12	AT	21	9
78718	7	17750346	17750369	2	12	24	TC	12	9
78720	7	17750417	17750430	2	7	14	TC	18	5
78727	7	17760994	17761005	2	6	12	AC	27	3
78730	7	17765165	17765204	2	20	40	TG	10	9
78732	7	17768745	17768766	2	11	22	AC	17	17
78737	7	17787385	17787396	2	6	12	TG	28	3
78738	7	17787560	17787575	2	8	16	AC	13	13
78740	7	17791488	17791503	2	8	16	GT	24	5
78744	7	17800961	17800978	3	6	18	AGC	15	3
78747	7	17801256	17801279	3	8	24	TCA	11	8
78751	7	17806399	17806414	2	8	16	TG	7	8
78753	7	17806545	17806570	2	13	26	AC	8	7
78754	7	17806596	17806621	2	13	26	CA	9	10
78755	7	17808646	17808661	2	8	16	AC	22	4
78756	7	17808901	17808920	4	5	20	GAGG	10	6
78762	7	17816607	17816640	2	17	34	AC	9	10
78766	7	17818712	17818741	2	15	30	GT	18	5
78767	7	17818919	17818932	2	7	14	AC	17	5
78768	7	17821989	17822016	2	14	28	GT	11	15
78769	7	17822229	17822258	2	15	30	TG	13	7
78771	7	17824320	17824335	2	8	16	AC	25	4
78772	7	17824404	17824433	2	15	30	AC	9	9
78773	7	17824545	17824560	2	8	16	CA	14	3
78779	7	17826889	17826916	2	14	28	TG	9	9
78782	7	17827044	17827083	2	20	40	GT	8	9
78784	7	17827258	17827277	2	10	20	CA	21	6
78786	7	17828557	17828590	2	17	34	CA	13	9
78788	7	17828726	17828747	2	11	22	CA	11	4
78797	7	17835955	17835976	2	11	22	GT	15	11
78798	7	17836154	17836173	2	10	20	CA	16	6
78799	7	17836482	17836493	2	6	12	TG	15	4
78801	7	17838295	17838321	3	9	27	ACA	7	3
78805	7	17843197	17843236	2	20	40	CA	16	11
78807	7	17844220	17844237	2	9	18	GT	20	9
78809	7	17848195	17848209	3	5	15	GAG	20	3
78810	7	17848593	17848612	2	10	20	GT	21	9
78815	7	17867165	17867186	2	11	22	TG	25	5
78822	7	17883010	17883027	3	6	18	GAA	24	4
78823	7	17888269	17888282	2	7	14	TG	19	3
78824	7	17888726	17888745	2	10	20	GT	13	6
78825	7	17888880	17888897	2	9	18	GT	17	6
78831	7	17908019	17908046	2	14	28	CA	13	12
78835	7	17915209	17915226	2	9	18	TC	19	4
78836	7	17919160	17919189	2	15	30	GT	9	9
78837	7	17919310	17919349	2	20	40	TG	6	7
78838	7	17919955	17919988	2	17	34	TC	10	10
78840	7	17932236	17932255	2	10	20	GT	14	5
78841	7	17932348	17932359	2	6	12	TG	13	7

78848	7	17945264	17945285	2	11	22	GT	18	10
78850	7	17948841	17948856	2	8	16	AG	23	13
78863	7	17973902	17973916	3	5	15	AGA	7	6
78867	7	17977476	17977513	2	19	38	TG	7	7
78869	7	17979634	17979659	2	13	26	GT	8	5
78872	7	17983707	17983722	2	8	16	GT	33	5
78873	7	17985052	17985085	2	17	34	TG	22	20
78875	7	17988635	17988652	3	6	18	AGG	9	4
78876	7	17988808	17988829	2	11	22	GT	14	13
78877	7	17991363	17991374	2	6	12	GT	23	4
78879	7	17993162	17993189	2	14	28	GT	12	12
78882	7	17994928	17994957	3	10	30	CTT	16	8
78883	7	17995054	17995077	3	8	24	CAG	27	8
78889	7	17996910	17996927	2	9	18	TG	21	11
78892	7	17997618	17997631	2	7	14	GT	25	4
78897	7	18000322	18000353	2	16	32	GT	14	11
78901	7	18014148	18014165	2	9	18	GT	6	6
78903	7	18023438	18023459	2	11	22	CA	18	11
78907	7	18028068	18028081	2	7	14	GT	19	3
78908	7	18028717	18028730	2	7	14	GT	28	5
78910	7	18031434	18031451	3	6	18	TCC	26	3
78913	7	18038005	18038016	2	6	12	CA	33	3
78914	7	18038173	18038186	2	7	14	TC	34	3
78918	7	18042167	18042199	3	11	33	GAT	16	8
78923	7	18047160	18047185	2	13	26	AC	18	12
78924	7	18049145	18049176	2	16	32	TG	12	8
78925	7	18049338	18049361	2	12	24	TG	8	7
78927	7	18050354	18050375	2	11	22	GT	16	4
78928	7	18053712	18053735	2	12	24	GT	11	11
78932	7	18060440	18060455	2	8	16	GT	7	8
78935	7	18065573	18065590	2	9	18	TG	22	11
78938	7	18068509	18068534	2	13	26	GT	9	8
78939	7	18070077	18070092	2	8	16	AG	19	3
78943	7	18070795	18070814	4	5	20	CTGT	24	3
78944	7	18071191	18071218	2	14	28	GA	29	11
78945	7	18072638	18072663	2	13	26	TG	13	11
78948	7	18073331	18073369	3	13	39	TCC	8	6
78950	7	18074189	18074203	3	5	15	TTC	21	3
78953	7	18075153	18075188	2	18	36	TG	8	8
78954	7	18075884	18075907	2	12	24	TG	9	8
78956	7	18076189	18076232	2	22	44	CA	8	11
78959	7	18080366	18080377	2	6	12	AC	22	3
78965	7	18082509	18082523	3	5	15	TTA	18	3
78966	7	18082781	18082795	3	5	15	TTC	24	6
78968	7	18083111	18083149	3	13	39	AGG	18	10
78969	7	18083343	18083370	2	14	28	AC	14	10
78973	7	18087855	18087884	2	15	30	TG	7	11
78974	7	18087995	18088018	2	12	24	GT	14	9
78975	7	18088177	18088192	2	8	16	TG	21	5
78981	7	18088739	18088770	2	16	32	CA	12	9
78982	7	18089247	18089264	2	9	18	AC	19	5

78983	7	18089323	18089344	2	11	22	CA	6	4
78991	7	18095327	18095340	2	7	14	AC	15	6
78996	7	18097113	18097134	2	11	22	AC	19	11
78999	7	18097316	18097333	2	9	18	TG	12	6
79000	7	18097541	18097556	2	8	16	TG	21	5
79001	7	18101202	18101219	2	9	18	TG	25	3
79003	7	18107747	18107762	2	8	16	GT	24	3
79005	7	18113019	18113036	2	9	18	CA	11	6
79008	7	18117441	18117464	3	8	24	GAT	25	9
79010	7	18118331	18118348	2	9	18	AC	23	8
79013	7	18119977	18119988	2	6	12	GT	19	7
79016	7	18121371	18121392	2	11	22	CA	23	7
79017	7	18121524	18121545	2	11	22	TG	18	17
79018	7	18122897	18122920	2	12	24	CA	12	9
79021	7	18125592	18125605	2	7	14	CA	32	4
79024	7	18127982	18128002	3	7	21	GGA	13	3
79025	7	18128159	18128182	3	8	24	GAG	16	6
79026	7	18129377	18129410	2	17	34	TG	19	12
79027	7	18129535	18129560	2	13	26	TG	12	10
79028	7	18129748	18129773	2	13	26	CA	13	10
79029	7	18129868	18129887	2	10	20	GT	12	7
79031	7	18130666	18130683	2	9	18	GT	22	10
79032	7	18130807	18130818	2	6	12	TG	19	4
79033	7	18130972	18131001	2	15	30	CA	13	9
79035	7	18131702	18131719	2	9	18	GA	24	4
79036	7	18131913	18131926	2	7	14	GA	26	3
79037	7	18134709	18134750	2	21	42	AC	13	11
79039	7	18136702	18136735	2	17	34	TG	15	16
79040	7	18138687	18138700	2	7	14	CA	6	5
79041	7	18138704	18138721	2	9	18	AC	8	4
79045	7	18140554	18140585	2	16	32	TG	9	8
79049	7	18145710	18145754	3	15	45	TCT	25	15
79052	7	18150112	18150125	2	7	14	CT	19	4
79054	7	18153673	18153700	2	14	28	CA	10	8
79055	7	18153972	18153986	3	5	15	AAG	28	5
79058	7	18155114	18155125	2	6	12	TG	28	3
79059	7	18155425	18155440	4	4	16	ATAA	25	3
79060	7	18156752	18156785	2	17	34	GT	10	9
79061	7	18157084	18157098	3	5	15	AGA	29	3
79064	7	18166023	18166054	2	16	32	TG	6	5
79066	7	18166207	18166234	2	14	28	GT	12	8
79072	7	18171536	18171547	2	6	12	TG	11	6
79073	7	18180098	18180115	2	9	18	GT	23	7
79074	7	18181423	18181444	2	11	22	TC	24	4
79075	7	18181821	18181844	2	12	24	TG	11	8
79076	7	18181897	18181918	2	11	22	AC	18	9
79077	7	18182046	18182069	2	12	24	TG	20	9
79078	7	18182679	18182706	2	14	28	GT	11	11
79079	7	18182876	18182905	2	15	30	CA	19	17
79080	7	18183784	18183795	2	6	12	GT	10	11
79081	7	18183797	18183812	2	8	16	TG	12	16

79083	7	18184155	18184174	2	10	20	TG	13	12
79085	7	18185287	18185314	2	14	28	AC	19	18
79086	7	18185753	18185784	2	16	32	AC	18	14
79087	7	18185923	18185944	2	11	22	GT	11	11
79089	7	18187336	18187367	2	16	32	AC	24	12
79090	7	18187721	18187742	2	11	22	TG	14	12
79094	7	18190136	18190153	2	9	18	CA	15	5
79095	7	18190261	18190274	2	7	14	GT	27	5
79096	7	18191992	18192013	2	11	22	AG	22	17
79099	7	18194358	18194381	2	12	24	CA	18	13
79100	7	18194490	18194503	2	7	14	AC	10	12
79101	7	18198243	18198254	2	6	12	TG	29	5
79102	7	18198434	18198475	2	21	42	AC	10	10
79103	7	18198671	18198685	3	5	15	GGA	25	7
79104	7	18198763	18198786	2	12	24	TG	20	18
79106	7	18201551	18201578	2	14	28	AC	16	5
79111	7	18204925	18204936	2	6	12	TG	23	4
79112	7	18205105	18205116	2	6	12	TG	29	3
79114	7	18206851	18206865	3	5	15	CAG	31	4
79115	7	18208481	18208502	2	11	22	CA	14	5
79116	7	18211878	18211919	2	21	42	CA	13	10
79121	7	18216643	18216660	2	9	18	TG	21	5
79122	7	18216973	18216984	2	6	12	TG	26	3
79123	7	18220206	18220217	2	6	12	AC	22	7
79124	7	18222487	18222502	2	8	16	CA	7	11
79126	7	18224934	18224961	2	14	28	CA	26	11
79130	7	18229438	18229470	3	11	33	CAG	28	18
79131	7	18231805	18231822	3	6	18	TCC	17	11
79132	7	18231824	18231838	3	5	15	CCT	14	11
79133	7	18233493	18233508	2	8	16	AC	20	11
79139	7	18237968	18237985	3	6	18	GAT	21	7
79141	7	18239763	18239786	3	8	24	TGT	22	8
79144	7	18241224	18241253	3	10	30	TTG	27	8
79147	7	18242735	18242761	3	9	27	CCT	16	6
79148	7	18243348	18243359	2	6	12	TA	20	5
79149	7	18245455	18245480	2	13	26	AC	13	18
79151	7	18247021	18247036	2	8	16	AG	25	4
79152	7	18247166	18247191	2	13	26	AC	20	12
79154	7	18248472	18248501	2	15	30	TG	20	15
79157	7	18250693	18250713	3	7	21	TCA	24	5
79158	7	18251406	18251420	3	5	15	AAC	19	5
79160	7	18254593	18254616	2	12	24	TG	9	8
79165	7	18269734	18269754	3	7	21	GCA	27	12
79168	7	18272201	18272252	4	13	52	CCTT	6	7
79169	7	18272607	18272621	3	5	15	GAG	25	3
79174	7	18293347	18293378	2	16	32	AC	21	13
79175	7	18295007	18295021	3	5	15	AGG	27	3
79176	7	18299410	18299433	3	8	24	AAG	30	5
79177	7	18300067	18300084	2	9	18	GT	24	5
79178	7	18300261	18300296	2	18	36	AC	21	7
79183	7	18308516	18308533	3	6	18	CAA	14	12

79184	7	18310197	18310220	3	8	24	GGA	27	5
79185	7	18310297	18310314	2	9	18	GA	24	3
79189	7	18311804	18311817	2	7	14	GT	28	3
79190	7	18311851	18311888	2	19	38	GT	16	8
79193	7	18315277	18315306	2	15	30	AC	17	14
79194	7	18315424	18315461	2	19	38	GT	12	13
79195	7	18315493	18315504	2	6	12	AG	17	6
79197	7	18318152	18318172	3	7	21	AGA	25	6
79202	7	18323830	18323850	3	7	21	CTT	6	9
79204	7	18324501	18324526	2	13	26	AC	18	8
79205	7	18324761	18324774	2	7	14	CA	20	7
79209	7	18329818	18329829	2	6	12	TG	16	9
79211	7	18332925	18332954	2	15	30	TG	9	9
79212	7	18333102	18333113	2	6	12	AC	14	12
79216	7	18337691	18337714	2	12	24	TG	19	6
79217	7	18337773	18337794	2	11	22	AC	14	7
79225	7	18354601	18354632	2	16	32	AG	12	8
79226	7	18354652	18354701	2	25	50	CA	12	11
79233	7	18360617	18360638	2	11	22	AC	19	18
79235	7	18365218	18365235	3	6	18	GCT	25	6
79237	7	18365841	18365852	2	6	12	CT	20	8
79238	7	18367556	18367587	2	16	32	CA	10	6
79240	7	18367769	18367784	2	8	16	CA	23	3
79242	7	18370491	18370506	2	8	16	TG	23	3
79244	7	18370773	18370792	2	10	20	TG	21	5
79245	7	18370892	18370924	3	11	33	GAG	16	7
79249	7	18373191	18373220	2	15	30	TG	9	10
79266	7	18386972	18386983	2	6	12	TA	12	5
79267	7	18388222	18388233	2	6	12	TG	15	6
79268	7	18388963	18388980	3	6	18	AAG	21	5
79278	7	18398267	18398298	2	16	32	AC	7	10
79280	7	18401799	18401822	3	8	24	TTG	25	10
79284	7	18408288	18408315	2	14	28	AC	13	9
79285	7	18409326	18409343	3	6	18	CTG	29	3
79288	7	18412212	18412225	2	7	14	TG	19	5
79289	7	18416721	18416742	2	11	22	GT	20	6
79290	7	18416933	18416950	2	9	18	AG	24	5
79292	7	18421145	18421162	2	9	18	AC	9	8
79316	7	18486710	18486727	2	9	18	TG	24	5
79317	7	18487702	18487721	2	10	20	CA	20	4
79318	7	18493402	18493421	4	5	20	GTGC	7	9
79321	7	18497290	18497339	2	25	50	CA	18	8
79324	7	18508813	18508824	2	6	12	GT	22	7
79325	7	18509211	18509246	2	18	36	GT	11	10
79326	7	18509925	18509940	4	4	16	AGCT	25	3
79328	7	18512437	18512457	3	7	21	ATA	24	3
79334	7	18518911	18518926	2	8	16	AG	29	3
79336	7	18521426	18521465	2	20	40	AC	21	15
79339	7	18530938	18530959	2	11	22	GT	18	8
79342	7	18537465	18537485	3	7	21	GCA	23	4
79347	7	18546602	18546625	3	8	24	ACA	21	3

79357	7	18562502	18562513	2	6	12	CA	30	6
79362	7	18563427	18563446	2	10	20	GT	8	7
79364	7	18568863	18568878	2	8	16	AC	21	4
79374	7	18581982	18581997	4	4	16	AAAC	15	3
79375	7	18591832	18591855	2	12	24	AC	12	7
79376	7	18592248	18592301	2	27	54	TG	10	11
79377	7	18596485	18596502	2	9	18	TG	22	3
79381	7	18597574	18597589	2	8	16	CA	10	3
79384	7	18600381	18600395	3	5	15	CTG	32	3
79386	7	18602617	18602650	2	17	34	AC	13	9
79388	7	18608252	18608265	2	7	14	CT	22	6
79391	7	18612407	18612430	2	12	24	TG	13	10
79396	7	18617292	18617311	2	10	20	AC	20	10
79399	7	18621056	18621103	2	24	48	AC	10	11
79401	7	18621406	18621441	2	18	36	AC	17	8
79403	7	18623203	18623232	2	15	30	AC	13	6
79405	7	18623679	18623702	4	6	24	GATG	25	3
79410	7	18629830	18629861	2	16	32	AC	15	8
79411	7	18629978	18630009	2	16	32	GT	9	7
79412	7	18630823	18630836	2	7	14	GT	21	4
79414	7	18631074	18631097	2	12	24	CA	18	6
79415	7	18631220	18631233	2	7	14	CA	22	3
79416	7	18633293	18633316	2	12	24	GT	16	13
79417	7	18633787	18633814	2	14	28	GT	22	8
79423	7	18640324	18640339	2	8	16	AC	25	4
79424	7	18640432	18640447	2	8	16	AC	24	7
79426	7	18643727	18643784	2	29	58	GT	6	9
79428	7	18645051	18645076	2	13	26	AC	14	8
79436	7	18662946	18662969	3	8	24	TGC	17	6
79437	7	18663062	18663087	2	13	26	CA	15	8
79438	7	18663413	18663440	2	14	28	GT	16	9
79439	7	18669733	18669758	2	13	26	CA	15	8
79443	7	18682160	18682191	4	8	32	ATAG	17	10
79445	7	18686144	18686175	2	16	32	TG	9	7
79447	7	18695370	18695383	2	7	14	TG	25	4
79454	7	18725252	18725279	2	14	28	AG	26	7
79467	7	18769034	18769087	2	27	54	AC	6	7
79472	7	18773274	18773297	2	12	24	GT	16	4
79473	7	18773411	18773422	2	6	12	GT	19	5
79476	7	18775884	18775899	2	8	16	CA	13	13
79478	7	18776809	18776826	3	6	18	CTC	26	3
79479	7	18777018	18777029	2	6	12	AC	20	7
79492	7	18796858	18796885	2	14	28	TG	17	11
79494	7	18802441	18802455	3	5	15	ATT	19	11
79502	7	18811532	18811559	2	14	28	AC	10	8
79503	7	18814674	18814689	2	8	16	CA	15	4
79505	7	18818015	18818040	2	13	26	AC	13	10
79507	7	18819953	18819978	2	13	26	CA	8	6
79508	7	18820493	18820512	4	5	20	TGAC	29	5
79513	7	18830610	18830629	2	10	20	GA	7	3
79521	7	18841745	18841762	2	9	18	CA	10	9

79524	7	18844357	18844370	2	7	14	GT	26	3
79540	7	18866610	18866645	2	18	36	CA	13	11
79542	7	18871438	18871470	3	11	33	TCT	31	3
79543	7	18873902	18873927	2	13	26	CA	9	8
79548	7	18888663	18888674	2	6	12	TG	17	4
79554	7	18890858	18890869	2	6	12	GT	9	4
79557	7	18896065	18896086	2	11	22	AC	19	6
79561	7	18902226	18902253	2	14	28	CA	17	12
79562	7	18907971	18907998	2	14	28	CA	14	8
79564	7	18917878	18917921	2	22	44	CA	10	13
79578	7	18940715	18940728	2	7	14	AC	22	5
79583	7	18955777	18955816	2	20	40	AC	7	9
79587	7	18958967	18958987	3	7	21	AAT	7	3
79588	7	18960548	18960559	2	6	12	AC	24	10
79590	7	18971101	18971115	3	5	15	ATT	30	3
79605	7	19023514	19023529	4	4	16	TTGT	13	3
79607	7	19027110	19027125	4	4	16	GGGA	16	3
79611	7	19037013	19037026	2	7	14	CA	16	4
79612	7	19037116	19037151	2	18	36	CA	17	14
79619	7	19052668	19052697	2	15	30	AC	17	18
79622	7	19057125	19057139	3	5	15	TTA	21	4
79624	7	19060643	19060660	2	9	18	TG	9	6
79625	7	19061835	19061849	3	5	15	GGT	20	5
79638	7	19092706	19092727	2	11	22	AC	6	9
79639	7	19092883	19092914	2	16	32	CA	6	8
79641	7	19095329	19095360	2	16	32	AC	11	13
79643	7	19097765	19097790	2	13	26	TG	16	11
79646	7	19103817	19103838	2	11	22	GT	14	9
79648	7	19110543	19110556	2	7	14	AC	31	3
79649	7	19111708	19111733	2	13	26	GT	6	6
79650	7	19117611	19117622	2	6	12	AT	8	11
79651	7	19118459	19118480	2	11	22	GT	24	11
79661	7	19142432	19142443	2	6	12	GT	19	16
79662	7	19144501	19144512	2	6	12	AT	21	4
79669	7	19176165	19176185	3	7	21	CTC	23	4
79670	7	19177075	19177088	2	7	14	TG	13	5
79672	7	19182647	19182658	2	6	12	AC	23	10
79673	7	19183136	19183147	2	6	12	TG	29	4
79674	7	19184329	19184343	3	5	15	AAT	8	4
79682	7	19197123	19197136	2	7	14	AC	16	5
79688	7	19220446	19220460	3	5	15	TGT	26	3
79692	7	19228587	19228602	4	4	16	TTTG	24	3
79696	7	19232722	19232757	2	18	36	CA	12	8
79700	7	19242301	19242324	2	12	24	GT	8	3
79701	7	19244552	19244599	2	24	48	TG	13	15
79702	7	19247188	19247199	2	6	12	GT	27	3
79703	7	19248889	19248914	2	13	26	GT	14	4
79704	7	19249047	19249072	2	13	26	GT	6	3
79705	7	19253159	19253172	2	7	14	AG	29	3
79719	7	19287857	19287878	2	11	22	GA	19	8
79724	7	19332775	19332786	2	6	12	TG	26	4

79726	7	19361537	19361550	2	7	14	GT	26	4
79727	7	19363722	19363736	3	5	15	AAT	26	4
79728	7	19365153	19365168	4	4	16	GATG	28	3
79729	7	19365600	19365629	2	15	30	AC	12	9
79730	7	19366729	19366748	2	10	20	AC	18	7
79734	7	19379566	19379579	2	7	14	AC	23	3
79744	7	19395651	19395668	3	6	18	TTG	25	4
79754	7	19406998	19407039	2	21	42	GT	8	12
79765	7	19425297	19425310	2	7	14	GT	16	9
79766	7	19425605	19425616	2	6	12	GT	25	7
79768	7	19430933	19430956	3	8	24	ATA	11	9
79770	7	19440169	19440198	2	15	30	TG	14	11
79771	7	19442720	19442739	4	5	20	TCTG	32	3
79777	7	19461515	19461538	2	12	24	GA	17	6
79779	7	19461610	19461627	2	9	18	GA	13	9
79784	7	19462851	19462864	2	7	14	CA	22	7
79785	7	19462965	19462985	3	7	21	GAG	26	10
79789	7	19469581	19469595	3	5	15	CAG	27	4
79790	7	19470239	19470256	3	6	18	TGC	28	4
79791	7	19474586	19474605	2	10	20	CT	10	10
79796	7	19482908	19482935	2	14	28	GT	14	12
79807	7	19496823	19496852	2	15	30	AC	9	8
79808	7	19502825	19502838	2	7	14	TG	31	3
79810	7	19503972	19503995	2	12	24	TG	20	15
79812	7	19504530	19504565	2	18	36	TG	11	14
79813	7	19504668	19504679	2	6	12	AC	16	8
79814	7	19504682	19504695	2	7	14	AC	12	7
79824	7	19524435	19524452	3	6	18	ATT	9	4
79834	7	19541418	19541437	4	5	20	TTTC	8	3
79835	7	19549365	19549382	3	6	18	CAT	21	3
79836	7	19561036	19561053	2	9	18	AC	19	6
79837	7	19561223	19561246	2	12	24	TG	19	5
79841	7	19566444	19566487	2	22	44	CA	15	10
79844	7	19573982	19573999	3	6	18	ATC	25	11
79856	7	19588512	19588523	2	6	12	TA	18	3
79863	7	19602452	19602466	3	5	15	TTG	28	3
79873	7	19638403	19638416	2	7	14	TC	24	3
79878	7	19647601	19647624	2	12	24	TC	11	10
79881	7	19657520	19657555	2	18	36	AC	9	10
79885	7	19663677	19663702	2	13	26	TG	6	5
79892	7	19671067	19671082	2	8	16	GT	11	4
79893	7	19671092	19671103	2	6	12	TG	15	8
79898	7	19695371	19695382	2	6	12	CA	24	3
79899	7	19701742	19701767	2	13	26	CA	15	5
79902	7	19701990	19702001	2	6	12	CA	22	3
79904	7	19702721	19702760	2	20	40	AC	15	8
79905	7	19703446	19703477	2	16	32	AC	10	10
79908	7	19706397	19706416	2	10	20	TG	14	9
79912	7	19710899	19710924	2	13	26	AC	13	11
79913	7	19712292	19712303	2	6	12	GT	25	3
79914	7	19712473	19712486	2	7	14	GT	18	5

79918	7	19712897	19712920	2	12	24	CA	17	8
79919	7	19712984	19713001	2	9	18	AC	17	9
79920	7	19713828	19713863	2	18	36	TG	6	7
79921	7	19715144	19715161	3	6	18	ATT	18	3
79926	7	19718230	19718259	2	15	30	CA	16	9
79927	7	19720628	19720643	2	8	16	TC	24	3
79931	7	19728028	19728047	2	10	20	CA	14	7
79933	7	19728201	19728216	2	8	16	GT	28	4
79934	7	19729043	19729068	2	13	26	TG	11	10
79935	7	19729269	19729302	2	17	34	AC	10	7
79939	7	19738409	19738422	2	7	14	GT	35	6
79941	7	19745923	19745936	2	7	14	AG	24	5
79942	7	19745939	19745952	2	7	14	AG	22	5
79946	7	19753772	19753783	2	6	12	CA	12	13
79947	7	19753786	19753801	2	8	16	CA	16	10
79949	7	19757923	19757948	2	13	26	AC	19	10
79950	7	19760082	19760095	2	7	14	TG	25	8
79951	7	19761571	19761592	2	11	22	CA	15	7
79952	7	19768215	19768228	2	7	14	AC	26	10
79953	7	19768407	19768420	2	7	14	CA	7	10
79954	7	19768423	19768438	2	8	16	CA	8	8
79956	7	19771666	19771679	2	7	14	CT	24	3
79957	7	19772609	19772628	2	10	20	AC	17	9
79959	7	19774242	19774255	2	7	14	CA	18	12
79960	7	19774257	19774272	2	8	16	AC	10	8
79961	7	19776312	19776327	2	8	16	TG	33	5
79962	7	19776983	19776998	2	8	16	TG	17	6
79963	7	19777164	19777197	2	17	34	AC	16	9
79964	7	19777996	19778027	2	16	32	GT	17	11
79966	7	19779053	19779080	2	14	28	GT	14	5
79967	7	19780467	19780478	2	6	12	TG	19	3
79969	7	19789724	19789745	2	11	22	AC	19	11
79982	7	19797326	19797357	2	16	32	AC	8	10
79983	7	19797585	19797608	2	12	24	GT	19	6
79984	7	19797657	19797670	2	7	14	TG	20	6
79985	7	19797895	19797914	2	10	20	GT	10	9
79986	7	19799209	19799220	2	6	12	CA	28	5
79987	7	19799619	19799642	2	12	24	CA	24	6
79990	7	19802524	19802539	4	4	16	TTTC	6	4
79992	7	19811068	19811095	2	14	28	CA	9	8
79997	7	19817671	19817700	2	15	30	GT	6	6
79999	7	19817881	19817896	2	8	16	CA	16	5
80002	7	19819735	19819762	2	14	28	AG	12	12
80003	7	19819914	19819925	2	6	12	AC	16	3
80007	7	19826956	19826981	2	13	26	AC	8	8
80008	7	19827069	19827088	2	10	20	CT	7	6
80009	7	19827089	19827106	2	9	18	CA	15	12
80012	7	19829055	19829069	3	5	15	GCA	29	3
80015	7	19831475	19831489	3	5	15	AGG	23	4
80016	7	19831895	19831926	2	16	32	GT	20	14
80021	7	19851368	19851379	2	6	12	TG	29	4

80029	7	19854377	19854404	4	7	28	TTTC	29	3
80031	7	19862472	19862501	2	15	30	GT	22	12
80033	7	19870071	19870092	2	11	22	CA	12	8
80034	7	19872174	19872199	2	13	26	AC	21	9
80037	7	19876596	19876629	2	17	34	AC	19	9
80038	7	19877347	19877366	2	10	20	AC	25	8
80040	7	19879957	19880000	2	22	44	GT	7	10
80041	7	19880611	19880626	2	8	16	AC	18	3
80056	7	19914680	19914699	4	5	20	TCCA	27	3
80060	7	19920391	19920410	2	10	20	GT	26	3
80061	7	19922825	19922846	2	11	22	GT	24	5
80065	7	19930546	19930569	2	12	24	CA	28	10
80069	7	19935362	19935381	4	5	20	TATC	14	3
80070	7	19936256	19936267	2	6	12	GT	25	4
80072	7	19960401	19960415	3	5	15	GAG	28	4
80075	7	19969960	19969979	2	10	20	GT	7	8
80080	7	19975500	19975511	2	6	12	AT	9	3
80085	7	20003424	20003438	3	5	15	GTT	26	5
80087	7	20010324	20010341	2	9	18	TG	21	16
80107	7	20061031	20061050	2	10	20	GT	19	8
80109	7	20061428	20061453	2	13	26	AC	14	9
80110	7	20061647	20061664	2	9	18	CA	7	7
80114	7	20075790	20075805	2	8	16	TC	17	4
80117	7	20076632	20076653	2	11	22	AC	10	10
80121	7	20084560	20084591	2	16	32	TG	14	14
80128	7	20097718	20097733	4	4	16	TTTG	33	4
80133	7	20102327	20102350	2	12	24	TG	12	9
80141	7	20127842	20127859	3	6	18	TGG	12	4
80143	7	20137280	20137303	3	8	24	GGA	6	5
80146	7	20139541	20139578	2	19	38	AC	6	9
80148	7	20143433	20143453	3	7	21	TGC	20	4
80149	7	20147156	20147179	2	12	24	AC	7	9
80153	7	20150814	20150839	2	13	26	TG	12	13
80155	7	20151189	20151200	2	6	12	TG	21	5
80156	7	20154351	20154364	2	7	14	CA	24	6
80158	7	20160312	20160327	2	8	16	GT	25	8
80160	7	20161318	20161344	3	9	27	CAA	22	4
80168	7	20176892	20176917	2	13	26	GT	6	6
80169	7	20178021	20178036	4	4	16	AAAT	26	3
80170	7	20180617	20180652	2	18	36	GT	15	6
80171	7	20182621	20182644	2	12	24	GT	11	8
80172	7	20182867	20182888	2	11	22	CA	10	6
80173	7	20183521	20183536	4	4	16	ATTT	11	8
80181	7	20201396	20201421	2	13	26	TG	7	8
80182	7	20203180	20203239	2	30	60	AC	10	10
80184	7	20204679	20204730	4	13	52	AAAC	25	7
80185	7	20206782	20206815	2	17	34	AC	8	7
80191	7	20214229	20214248	2	10	20	AC	26	8
80199	7	20225193	20225226	2	17	34	TG	10	10
80200	7	20240462	20240495	2	17	34	AC	13	10
80203	7	20241589	20241618	2	15	30	TG	16	12

80205	7	20248228	20248247	2	10	20	CA	18	6
80206	7	20248992	20249011	2	10	20	AC	22	7
80207	7	20254639	20254652	2	7	14	AG	21	4
80208	7	20256190	20256209	4	5	20	AAAC	25	4
80216	7	20277663	20277676	2	7	14	TG	9	3
80217	7	20278116	20278155	2	20	40	AG	7	7
80223	7	20303749	20303768	4	5	20	TCCC	9	3
80225	7	20308693	20308710	3	6	18	CAG	23	3
80227	7	20313424	20313439	2	8	16	AT	6	5
80237	7	20332871	20332908	2	19	38	GT	9	11
80238	7	20334119	20334132	2	7	14	TC	8	4
80239	7	20334134	20334147	2	7	14	CA	15	8
80241	7	20349869	20349896	2	14	28	TG	16	16
80242	7	20369042	20369067	2	13	26	GT	26	7
80243	7	20369615	20369638	3	8	24	AAT	24	9
80244	7	20378252	20378263	2	6	12	AC	23	8
80248	7	20386975	20387008	2	17	34	GT	9	9
80251	7	20388806	20388823	2	9	18	GT	11	11
80256	7	20396198	20396239	2	21	42	TG	8	11
80257	7	20401281	20401312	2	16	32	TG	18	5
80258	7	20402639	20402650	2	6	12	GT	21	3
80262	7	20407425	20407436	2	6	12	TG	11	3
80263	7	20411829	20411848	2	10	20	GT	22	14
80265	7	20413009	20413038	2	15	30	TG	13	10
80267	7	20413756	20413773	2	9	18	CT	16	6
80270	7	20415947	20415962	2	8	16	TC	21	8
80273	7	20423180	20423195	4	4	16	CACG	14	9
80275	7	20424648	20424659	2	6	12	AC	31	4
80277	7	20428731	20428752	2	11	22	AC	11	8
80279	7	20428920	20428933	2	7	14	CA	25	4
80283	7	20432234	20432257	2	12	24	AC	10	10
80286	7	20437467	20437510	2	22	44	CA	8	8
80288	7	20437661	20437684	2	12	24	CA	8	3
80294	7	20464229	20464240	2	6	12	TG	14	5
80298	7	20465819	20465846	2	14	28	AC	11	14
80300	7	20466575	20466588	2	7	14	AC	6	4
80304	7	20470205	20470220	2	8	16	GT	21	14
80305	7	20470663	20470690	2	14	28	CA	14	10
80306	7	20470841	20470860	2	10	20	AC	17	9
80317	7	20482900	20482935	2	18	36	GT	15	12
80318	7	20483262	20483283	2	11	22	CA	16	8
80320	7	20487286	20487313	2	14	28	AC	18	14
80330	7	20493149	20493164	4	4	16	TTTA	24	3
80332	7	20495325	20495358	2	17	34	TG	13	10
80333	7	20496196	20496219	2	12	24	AC	20	7
80334	7	20496395	20496408	2	7	14	CA	23	11
80337	7	20497957	20497986	2	15	30	TG	13	6
80342	7	20500114	20500125	2	6	12	GT	7	3
80351	7	20525436	20525449	2	7	14	CT	7	4
80353	7	20525587	20525612	2	13	26	AC	13	11
80354	7	20528093	20528110	2	9	18	TG	10	7

80355	7	20528244	20528255	2	6	12	TC	16	6
80356	7	20528258	20528269	2	6	12	TC	15	5
80358	7	20529839	20529878	4	10	40	CAGA	17	13
80361	7	20551116	20551155	2	20	40	CA	12	12
80362	7	20551419	20551439	3	7	21	GAG	18	3
80363	7	20553505	20553522	2	9	18	GT	15	6
80365	7	20565075	20565098	2	12	24	CA	19	9
80366	7	20570205	20570246	2	21	42	CA	10	9
80369	7	20576738	20576755	2	9	18	GT	6	6
80370	7	20576845	20576860	2	8	16	GT	23	9
80375	7	20583812	20583823	2	6	12	GA	13	4
80377	7	20586866	20586885	4	5	20	TCTG	24	6
80380	7	20590672	20590683	2	6	12	TG	21	6
80383	7	20598418	20598431	2	7	14	CT	27	4
80384	7	20604772	20604801	2	15	30	AC	13	8
80390	7	20611277	20611300	4	6	24	TAGG	25	3
80399	7	20624019	20624042	2	12	24	CA	21	8
80403	7	20625538	20625557	2	10	20	GT	29	5
80404	7	20626926	20626959	2	17	34	TG	11	7
80406	7	20628064	20628107	2	22	44	CA	9	9
80416	7	20638138	20638151	2	7	14	GT	21	5
80417	7	20639464	20639513	2	25	50	CA	13	15
80418	7	20639611	20639630	2	10	20	AC	19	8
80419	7	20641035	20641068	2	17	34	TG	6	8
80420	7	20642131	20642158	2	14	28	GT	12	10
80421	7	20642241	20642258	2	9	18	GT	19	6
80428	7	20655926	20655963	2	19	38	GT	14	12
80432	7	20661541	20661556	2	8	16	GT	16	8
80440	7	20671280	20671299	2	10	20	CA	14	12
80441	7	20673227	20673244	3	6	18	ATC	18	14
80445	7	20680973	20680988	2	8	16	AT	9	4
80446	7	20681501	20681520	2	10	20	AC	27	5
80451	7	20686578	20686593	4	4	16	AAAT	15	4
80454	7	20699891	20699906	2	8	16	GT	20	6
80455	7	20703928	20703941	2	7	14	GT	26	4
80457	7	20705662	20705673	2	6	12	TG	26	5
80460	7	20721283	20721300	2	9	18	CA	7	6
80465	7	20744518	20744563	2	23	46	GT	8	11
80478	7	20769441	20769452	2	6	12	AC	15	4
80492	7	20788007	20788020	2	7	14	AC	8	7
80493	7	20788022	20788039	2	9	18	CA	12	8
80497	7	20796141	20796154	2	7	14	AC	23	6
80501	7	20799234	20799248	3	5	15	GGA	23	9
80504	7	20804880	20804891	2	6	12	AC	31	3
80505	7	20805130	20805153	3	8	24	AGG	32	4
80509	7	20810137	20810176	2	20	40	GT	12	12
80512	7	20817363	20817382	2	10	20	CA	18	6
80513	7	20819580	20819599	2	10	20	GT	18	9
80515	7	20825439	20825459	3	7	21	AAC	24	3
80516	7	20826128	20826147	2	10	20	CA	19	8
80527	7	20864872	20864885	2	7	14	GT	24	5

80528	7	20869045	20869068	4	6	24	TATC	24	4
80530	7	20875089	20875118	2	15	30	TG	7	7
80538	7	20880899	20880930	2	16	32	AC	9	9
80544	7	20893926	20893941	2	8	16	AC	17	7
80545	7	20893943	20893958	2	8	16	CA	25	11
80550	7	20904185	20904212	4	7	28	ATCT	17	8
80554	7	20914929	20914942	2	7	14	TC	26	4
80555	7	20923795	20923818	2	12	24	CA	23	6
80558	7	20926437	20926448	2	6	12	CA	24	7
80563	7	20931393	20931418	2	13	26	TG	25	9
80569	7	20938426	20938457	2	16	32	GT	9	14
80570	7	20940840	20940871	2	16	32	GT	10	9
80571	7	20941020	20941061	2	21	42	GT	12	7
80578	7	20949796	20949825	2	15	30	AC	8	9
80593	7	21014630	21014653	3	8	24	ATA	14	4
80595	8	36056	36067	2	6	12	AC	18	3
80596	8	36092	36103	2	6	12	AT	8	6
80597	8	38014	38049	4	9	36	TGTA	13	12
80598	8	43508	43527	4	5	20	CTTA	27	6
80603	8	51240	51259	4	5	20	GAAT	12	5
80606	8	66261	66276	2	8	16	CA	23	10
80614	8	69632	69649	2	9	18	GT	16	5
80618	8	78908	78928	3	7	21	GAT	29	3
80622	8	84852	84865	2	7	14	AC	10	7
80624	8	89530	89541	2	6	12	TC	16	9
80626	8	103063	103078	2	8	16	GT	25	6
80632	8	112674	112687	2	7	14	AC	23	4
80633	8	112804	112825	2	11	22	TG	15	9
80636	8	118796	118825	2	15	30	CA	22	9
80644	8	129438	129469	2	16	32	TG	15	9
80646	8	133577	133588	2	6	12	AC	31	3
80647	8	136911	136924	2	7	14	CA	16	6
80648	8	137125	137140	2	8	16	GT	22	5
80649	8	137143	137156	2	7	14	GT	18	4
80651	8	143914	143937	2	12	24	TG	15	9
80655	8	154972	155011	2	20	40	GT	10	13
80656	8	155218	155237	2	10	20	TG	16	8
80660	8	158787	158808	2	11	22	CA	19	14
80662	8	166913	166945	3	11	33	AGA	15	10
80663	8	170207	170240	2	17	34	GT	13	7
80665	8	172835	172882	2	24	48	AC	11	10
80666	8	178588	178599	2	6	12	GT	20	12
80668	8	195993	196016	2	12	24	AC	23	6
80672	8	200669	200716	2	24	48	TG	7	8
80674	8	201086	201111	2	13	26	CA	21	6
80675	8	201521	201541	3	7	21	GCA	23	7
80677	8	202014	202031	2	9	18	GT	10	8
80680	8	218881	218894	2	7	14	TA	9	4
80682	8	223663	223678	2	8	16	AC	10	8
80684	8	226495	226532	2	19	38	CA	9	11
80690	8	237302	237331	2	15	30	AC	21	11

80691	8	245146	245169	2	12	24	AG	18	8
80692	8	247811	247834	4	6	24	GTCT	26	3
80693	8	249459	249494	2	18	36	AC	12	10
80696	8	250634	250647	2	7	14	GT	6	4
80698	8	250840	250859	2	10	20	CA	21	12
80699	8	252029	252073	3	15	45	AGA	11	11
80704	8	267249	267266	2	9	18	TG	18	10
80705	8	267269	267280	2	6	12	TG	20	14
80709	8	277007	277026	4	5	20	TGTT	15	3
80710	8	280461	280472	2	6	12	AT	32	3
80715	8	292326	292339	2	7	14	AC	31	3
80721	8	318251	318286	2	18	36	GT	9	9
80725	8	325988	326005	3	6	18	GGA	7	3
80726	8	326702	326717	2	8	16	AC	16	8
80730	8	334996	335016	3	7	21	CTG	28	6
80731	8	336143	336157	3	5	15	GAA	32	3
80735	8	341273	341326	2	27	54	CA	7	12
80736	8	344094	344111	3	6	18	ATA	9	3
80740	8	346402	346415	2	7	14	AC	23	12
80741	8	346417	346432	2	8	16	CA	26	10
80742	8	346520	346531	2	6	12	GT	25	9
80743	8	346534	346545	2	6	12	GT	23	8
80744	8	351203	351220	2	9	18	AC	23	8
80748	8	356125	356148	2	12	24	GT	19	6
80749	8	356193	356220	2	14	28	TG	16	6
80761	8	401604	401627	2	12	24	TG	17	5
80769	8	439155	439170	2	8	16	GT	13	5
80779	8	464171	464196	2	13	26	CA	12	8
80781	8	464571	464604	2	17	34	TG	9	9
80786	8	471262	471285	2	12	24	TG	9	8
80788	8	478935	478958	2	12	24	TG	17	9
80789	8	479307	479318	2	6	12	GT	24	8
80791	8	479873	479892	2	10	20	GT	14	4
80801	8	493350	493367	2	9	18	TG	18	5
80810	8	497504	497517	2	7	14	CA	26	3
80811	8	499402	499422	3	7	21	ACA	19	3
80816	8	514880	514907	2	14	28	TG	17	5
80817	8	515007	515034	2	14	28	AC	18	9
80819	8	518207	518248	2	21	42	TG	8	9
80821	8	520625	520654	2	15	30	GT	10	7
80822	8	521129	521154	2	13	26	TG	10	9
80827	8	534841	534866	2	13	26	TG	11	12
80829	8	538616	538633	3	6	18	CAG	16	4
80831	8	542432	542449	2	9	18	GT	23	7
80833	8	544828	544841	2	7	14	AC	22	3
80837	8	550355	550368	2	7	14	TG	25	3
80844	8	570559	570578	4	5	20	TGAT	27	3
80846	8	575150	575171	2	11	22	CA	20	10
80848	8	578327	578359	3	11	33	TAT	13	6
80851	8	582967	582990	2	12	24	AC	20	9
80852	8	586706	586717	2	6	12	AC	32	3

80854	8	592144	592167	3	8	24	ATT	9	3
80859	8	596667	596694	2	14	28	TG	13	8
80861	8	597992	598027	2	18	36	TG	6	8
80865	8	607406	607425	2	10	20	TG	15	6
80866	8	607450	607479	2	15	30	AC	12	6
80869	8	610375	610394	2	10	20	AC	14	7
80870	8	612387	612414	2	14	28	AC	18	8
80871	8	612658	612669	2	6	12	CA	24	5
80872	8	612754	612767	2	7	14	AC	14	7
80873	8	612770	612785	2	8	16	AC	14	9
80877	8	616179	616200	2	11	22	TG	23	8
80878	8	618654	618673	4	5	20	TTTG	24	3
80880	8	621550	621563	2	7	14	GT	22	5
80881	8	622522	622536	3	5	15	GTG	20	3
80882	8	622907	622922	2	8	16	GT	26	6
80886	8	625590	625627	2	19	38	AC	24	12
80887	8	628791	628808	2	9	18	GT	23	13
80888	8	635508	635527	4	5	20	CAAT	28	4
80891	8	648073	648090	2	9	18	AC	23	6
80904	8	689512	689527	2	8	16	TG	29	3
80906	8	704211	704222	2	6	12	AT	11	6
80908	8	708310	708345	2	18	36	TG	7	9
80909	8	708472	708503	2	16	32	GT	6	5
80913	8	720321	720334	2	7	14	TG	23	4
80917	8	728742	728795	2	27	54	CA	8	10
80923	8	736276	736287	2	6	12	TC	23	3
80928	8	744865	744878	2	7	14	CA	26	4
80930	8	747998	748021	2	12	24	TG	11	6
80931	8	749977	749996	2	10	20	TG	22	5
80936	8	757058	757081	2	12	24	CA	18	9
80938	8	757220	757241	2	11	22	AC	15	10
80944	8	766227	766272	2	23	46	AC	6	8
80945	8	766321	766338	2	9	18	GT	17	4
80947	8	775393	775418	2	13	26	GT	10	7
80948	8	780635	780662	2	14	28	GT	11	11
80950	8	781656	781675	2	10	20	AC	24	5
80958	8	787683	787696	2	7	14	TG	26	4
80967	8	795606	795621	2	8	16	TG	25	5
80986	8	821413	821430	2	9	18	TG	20	8
80989	8	828653	828668	4	4	16	GGCA	25	4
80997	8	854680	854697	3	6	18	CCT	20	6
81000	8	858792	858805	2	7	14	CA	27	6
81002	8	862071	862084	2	7	14	AC	17	3
81008	8	870849	870888	2	20	40	GT	7	7
81009	8	871007	871022	2	8	16	GT	19	4
81025	8	922955	922972	2	9	18	GT	23	8
81028	8	940952	940969	3	6	18	GCA	19	3
81034	8	947303	947322	2	10	20	AC	18	10
81038	8	948885	948902	2	9	18	TC	22	3
81039	8	950101	950112	2	6	12	GT	27	5
81050	8	976344	976365	2	11	22	TG	14	8

81052	8	977050	977061	2	6	12	AG	33	3
81054	8	993505	993518	2	7	14	TC	14	4
81056	8	993983	994002	2	10	20	TG	17	8
81059	8	994126	994145	2	10	20	TG	9	3
81066	8	1002760	1002775	2	8	16	CA	19	5
81070	8	1006267	1006302	2	18	36	TG	11	7
81071	8	1006409	1006424	2	8	16	CA	12	4
81076	8	1021081	1021112	2	16	32	CA	10	8
81077	8	1023441	1023466	2	13	26	TG	14	6
81084	8	1031778	1031792	3	5	15	CTG	31	4
81092	8	1052744	1052761	2	9	18	CA	16	9
81106	8	1068645	1068706	2	31	62	AC	8	11
81108	8	1078186	1078207	2	11	22	AC	18	10
81109	8	1083997	1084022	2	13	26	AC	16	10
81113	8	1094236	1094249	2	7	14	GT	21	5
81119	8	1104055	1104072	3	6	18	CAT	21	3
81124	8	1108837	1108874	2	19	38	AC	8	9
81125	8	1108943	1108968	2	13	26	AC	13	8
81127	8	1110366	1110383	3	6	18	AAT	22	9
81136	8	1123244	1123263	2	10	20	GA	18	5
81139	8	1137141	1137168	2	14	28	TG	7	7
81149	8	1183104	1183123	2	10	20	CA	26	6
81151	8	1187387	1187401	3	5	15	TTG	25	3
81158	8	1204171	1204182	2	6	12	TG	21	7
81159	8	1204184	1204195	2	6	12	GT	21	6
81160	8	1204259	1204273	3	5	15	GCC	30	5
81164	8	1207799	1207858	2	30	60	TC	12	9
81171	8	1240492	1240509	3	6	18	ATT	8	4
81179	8	1277542	1277553	2	6	12	TG	29	3
81183	8	1279835	1279854	2	10	20	TG	24	11
81186	8	1286007	1286030	3	8	24	AAT	9	3
81192	8	1313248	1313269	2	11	22	CA	16	5
81203	8	1393030	1393053	2	12	24	TG	11	5
81204	8	1395057	1395090	2	17	34	CA	9	13
81210	8	1414328	1414349	2	11	22	CA	17	6
81212	8	1417470	1417499	2	15	30	TG	13	15
81216	8	1421493	1421504	2	6	12	TG	6	7
81219	8	1431554	1431581	4	7	28	TAGA	22	4
81222	8	1446874	1446907	2	17	34	CA	14	13
81228	8	1453820	1453833	2	7	14	AT	22	3
81229	8	1454195	1454220	2	13	26	AC	16	4
81230	8	1454343	1454376	2	17	34	TC	8	8
81234	8	1459755	1459794	4	10	40	AATC	13	12
81246	8	1484198	1484215	2	9	18	AC	21	9
81247	8	1488879	1488896	2	9	18	CA	26	5
81248	8	1488898	1488915	2	9	18	AC	21	7
81249	8	1492348	1492361	2	7	14	AT	18	5
81255	8	1505173	1505190	3	6	18	TGT	19	4
81257	8	1518651	1518674	2	12	24	AC	18	16
81265	8	1536633	1536644	2	6	12	TA	8	7
81269	8	1542271	1542288	2	9	18	TG	24	9

81291	8	1579524	1579535	2	6	12	AT	15	7
81299	8	1596322	1596349	2	14	28	AC	18	8
81303	8	1600805	1600818	2	7	14	TG	22	4
81306	8	1601551	1601570	4	5	20	TTTG	11	4
81309	8	1606080	1606097	2	9	18	TG	17	3
81314	8	1623360	1623385	2	13	26	AC	13	10
81319	8	1635502	1635529	2	14	28	CA	15	7
81321	8	1641952	1641993	2	21	42	GA	15	8
81328	8	1660357	1660370	2	7	14	AC	8	11
81348	8	1699717	1699734	2	9	18	GA	29	4
81353	8	1714740	1714759	4	5	20	GTTG	27	6
81355	8	1721975	1721998	4	6	24	ATCC	25	8
81360	8	1732482	1732495	2	7	14	GT	7	4
81364	8	1734902	1734921	2	10	20	AC	24	8
81365	8	1737679	1737716	2	19	38	TG	11	12
81374	8	1746039	1746086	4	12	48	TCTT	21	9
81388	8	1774210	1774221	2	6	12	CA	26	3
81397	8	1803557	1803592	3	12	36	GAT	24	7
81399	8	1809266	1809325	4	15	60	GAAA	10	6
81403	8	1817778	1817793	2	8	16	GT	8	3
81406	8	1818254	1818283	2	15	30	TG	7	8
81408	8	1821025	1821062	2	19	38	GT	15	7
81415	8	1861918	1861935	3	6	18	TCC	28	3
81434	8	1908643	1908658	2	8	16	TG	19	3
81441	8	1943888	1943905	2	9	18	TG	11	13
81455	8	1948945	1948959	3	5	15	GTT	27	4
81456	8	1955145	1955165	3	7	21	CTC	28	4
81457	8	1956725	1956742	3	6	18	GCT	30	4
81458	8	1957370	1957381	2	6	12	AC	20	7
81459	8	1957537	1957562	2	13	26	AC	10	6
81460	8	1957710	1957727	2	9	18	AC	14	5
81461	8	1959850	1959865	2	8	16	CA	17	3
81462	8	1960262	1960273	2	6	12	CA	18	7
81465	8	1968795	1968808	2	7	14	TG	17	4
81466	8	1968889	1968916	2	14	28	GT	17	6
81470	8	1972365	1972382	2	9	18	CA	12	10
81472	8	1972545	1972576	2	16	32	CT	14	7
81475	8	1980042	1980055	2	7	14	CA	18	3
81480	8	1983912	1983935	2	12	24	TC	15	10
81492	8	2017621	2017636	4	4	16	TTAT	21	6
81497	8	2033175	2033194	2	10	20	AC	22	9
81503	8	2035123	2035146	3	8	24	TAA	12	4
81504	8	2041246	2041257	2	6	12	AT	11	3
81513	8	2087749	2087766	3	6	18	AAT	26	4
81515	8	2092760	2092789	2	15	30	AG	15	12
81524	8	2111218	2111256	3	13	39	CTC	8	6
81528	8	2128044	2128055	2	6	12	TA	13	4
81529	8	2129970	2129983	2	7	14	AC	18	12
81534	8	2136744	2136759	2	8	16	TG	10	4
81535	8	2136762	2136773	2	6	12	TG	23	6
81545	8	2214423	2214449	3	9	27	TCA	24	13

81549	8	2220184	2220207	2	12	24	AC	11	13
81554	8	2226116	2226137	2	11	22	TG	11	10
81561	8	2247702	2247741	4	10	40	ATAG	31	11
81563	8	2271045	2271056	2	6	12	TG	27	4
81569	8	2306269	2306286	3	6	18	AAG	22	3
81570	8	2310567	2310590	3	8	24	TAT	12	9
81573	8	2323099	2323130	2	16	32	TG	11	9
81574	8	2324023	2324050	2	14	28	TG	15	7
81586	8	2357159	2357186	2	14	28	AC	13	11
81599	8	2372246	2372260	3	5	15	CTT	23	6
81602	8	2399332	2399345	2	7	14	CT	19	3
81615	8	2416311	2416334	2	12	24	CA	8	6
81617	8	2423833	2423852	2	10	20	TG	25	3
81618	8	2427189	2427216	4	7	28	GAAA	18	3
81621	8	2431677	2431710	2	17	34	TG	11	11
81624	8	2440019	2440036	2	9	18	CA	15	6
81630	8	2444768	2444785	2	9	18	AC	16	11
81637	8	2447158	2447173	2	8	16	TC	20	4
81647	8	2480757	2480770	2	7	14	AC	17	4
81652	8	2512812	2512825	2	7	14	AC	26	7
81666	8	2529450	2529483	2	17	34	AC	18	7
81667	8	2529537	2529558	2	11	22	CA	15	4
81668	8	2530416	2530487	4	18	72	TGAT	14	13
81670	8	2533199	2533216	2	9	18	AC	28	4
81676	8	2544254	2544285	4	8	32	TCTG	25	4
81692	8	2552761	2552796	3	12	36	GAG	16	9
81696	8	2555563	2555576	2	7	14	AC	21	7
81719	8	2582065	2582078	2	7	14	CA	29	5
81720	8	2585997	2586008	2	6	12	AG	26	4
81732	8	2623609	2623632	2	12	24	CA	21	9
81735	8	2624704	2624737	2	17	34	GT	9	7
81741	8	2628281	2628294	2	7	14	TG	26	6
81743	8	2630032	2630070	3	13	39	TGT	18	9
81744	8	2630090	2630140	3	17	51	TTC	7	9
81746	8	2633629	2633640	2	6	12	AC	19	10
81751	8	2645112	2645141	2	15	30	TG	21	9
81765	8	2671166	2671183	2	9	18	GT	11	8
81768	8	2672821	2672836	4	4	16	TGTT	26	3
81772	8	2676929	2676944	2	8	16	CA	27	4
81778	8	2682890	2682901	2	6	12	AC	8	3
81791	8	2699981	2700008	2	14	28	TG	10	9
81792	8	2700138	2700169	2	16	32	TG	17	13
81813	8	2758837	2758850	2	7	14	AG	24	3
81825	8	2784259	2784292	2	17	34	CA	17	9
81826	8	2791417	2791436	2	10	20	AC	25	3
81833	8	2807880	2807909	2	15	30	GT	6	6
81847	8	2836491	2836508	2	9	18	AC	19	6
81853	8	2855810	2855821	2	6	12	GA	33	5
81857	8	2879745	2879756	2	6	12	GT	24	3
81858	8	2882922	2882935	2	7	14	GT	20	7
81864	8	2913148	2913167	4	5	20	GTTT	30	6

81867	8	2931212	2931229	3	6	18	TTG	22	7
81868	8	2933799	2933825	3	9	27	GAT	22	5
81871	8	2938267	2938286	4	5	20	CAGC	18	7
81872	8	2938325	2938340	4	4	16	CGCA	19	3
81883	8	2948302	2948315	2	7	14	CA	20	4
81890	8	2953079	2953098	2	10	20	CG	8	7
81901	8	2965737	2965757	3	7	21	GCA	22	3
81907	8	2968401	2968416	2	8	16	AG	26	4
81910	8	2980522	2980557	2	18	36	TG	13	9
81911	8	2980731	2980754	2	12	24	CA	17	7
81912	8	2980891	2980916	2	13	26	GT	12	9
81914	8	2983868	2983883	2	8	16	AT	19	5
81915	8	2984094	2984113	4	5	20	ATGG	12	8
81917	8	2986902	2986916	3	5	15	TTC	25	3
81922	8	2988947	2988968	2	11	22	AC	16	9
81924	8	2989284	2989295	2	6	12	AC	22	3
81930	8	2994527	2994542	2	8	16	TG	19	4
81933	8	3000091	3000102	2	6	12	GT	22	5
81934	8	3000130	3000141	2	6	12	TG	19	4
81938	8	3003125	3003156	2	16	32	TG	18	7
81941	8	3003740	3003757	2	9	18	GT	17	4
81945	8	3004415	3004456	2	21	42	GT	12	8
81947	8	3005258	3005285	2	14	28	TG	9	11
81951	8	3007617	3007646	2	15	30	GT	6	6
81952	8	3007716	3007727	2	6	12	GT	16	5
81953	8	3007728	3007739	2	6	12	GA	14	4
81954	8	3008259	3008279	3	7	21	GTT	26	4
81955	8	3008829	3008852	2	12	24	GT	9	10
81960	8	3013293	3013306	2	7	14	CA	22	4
81961	8	3014166	3014181	2	8	16	GT	27	5
81962	8	3016058	3016071	2	7	14	TG	16	3
81963	8	3016749	3016763	3	5	15	GCA	24	3
81966	8	3017814	3017839	2	13	26	AC	16	12
81967	8	3022506	3022521	2	8	16	GT	26	4
81975	8	3034225	3034242	2	9	18	GT	15	7
81976	8	3043145	3043156	2	6	12	AT	15	3
81979	8	3054412	3054427	2	8	16	AT	27	3
81985	8	3070758	3070772	3	5	15	CAT	29	4
81993	8	3106497	3106508	2	6	12	TG	18	3
82000	8	3124860	3124873	2	7	14	GA	28	5
82007	8	3143189	3143210	2	11	22	AC	12	5
82022	8	3197726	3197747	2	11	22	GT	21	9
82024	8	3201386	3201415	2	15	30	AC	17	6
82027	8	3215106	3215127	2	11	22	TG	16	13
82029	8	3218571	3218584	2	7	14	AG	29	4
82032	8	3222489	3222509	3	7	21	AAG	24	6
82035	8	3225773	3225786	2	7	14	GT	26	5
82049	8	3267165	3267178	2	7	14	GT	28	11
82055	8	3273308	3273327	2	10	20	TA	8	3
82058	8	3283651	3283674	3	8	24	CCT	18	6
82059	8	3289694	3289713	4	5	20	TTTC	30	7

82063	8	3293760	3293783	2	12	24	GA	22	4
82064	8	3300273	3300284	2	6	12	AG	16	5
82066	8	3302342	3302355	2	7	14	CA	29	4
82081	8	3339841	3339860	2	10	20	TG	16	7
82083	8	3343837	3343856	2	10	20	AC	23	7
82084	8	3344065	3344078	2	7	14	CA	17	4
82086	8	3344603	3344620	3	6	18	CAT	31	3
82088	8	3347542	3347557	2	8	16	GT	23	4
82089	8	3350014	3350039	2	13	26	CT	12	10
82090	8	3350699	3350713	3	5	15	ATC	22	5
82094	8	3359738	3359751	2	7	14	GA	25	3
82098	8	3367789	3367800	2	6	12	TG	18	3
82099	8	3367803	3367814	2	6	12	TG	24	3
82105	8	3377934	3377945	2	6	12	CA	26	3
82106	8	3382495	3382528	2	17	34	CA	10	9
82128	8	3432043	3432070	2	14	28	TG	9	6
82129	8	3432193	3432207	3	5	15	CAA	19	12
82132	8	3457585	3457614	3	10	30	GAA	18	3
82133	8	3457908	3457919	2	6	12	CA	25	5
82136	8	3460761	3460793	3	11	33	CTC	16	6
82137	8	3461160	3461181	2	11	22	CA	19	10
82139	8	3475155	3475168	2	7	14	CA	27	3
82140	8	3478557	3478574	3	6	18	GCT	22	4
82142	8	3483068	3483082	3	5	15	TCC	25	9
82145	8	3492070	3492083	2	7	14	AC	31	3
82146	8	3492273	3492288	2	8	16	AG	24	3
82147	8	3495236	3495257	2	11	22	GT	13	4
82149	8	3495292	3495325	2	17	34	GT	7	6
82158	8	3504323	3504350	2	14	28	GT	12	6
82160	8	3508336	3508347	2	6	12	GT	19	7
82161	8	3508551	3508562	2	6	12	TG	19	4
82163	8	3511750	3511779	2	15	30	TG	20	8
82166	8	3522705	3522720	2	8	16	AC	27	6
82167	8	3529548	3529571	2	12	24	CA	12	11
82177	8	3531664	3531687	3	8	24	AAG	18	8
82178	8	3532480	3532505	2	13	26	TG	17	8
82179	8	3536365	3536382	2	9	18	TG	22	6
82180	8	3536566	3536587	2	11	22	GT	21	12
82182	8	3537528	3537551	2	12	24	CA	17	12
82183	8	3538172	3538185	2	7	14	AT	10	7
82188	8	3540291	3540310	2	10	20	CT	14	12
82190	8	3541854	3541891	2	19	38	GT	15	11
82193	8	3552523	3552538	4	4	16	AACC	25	3
82204	8	3569407	3569424	2	9	18	AG	26	4
82210	8	3576501	3576524	2	12	24	CA	10	8
82212	8	3577655	3577682	2	14	28	GT	8	10
82214	8	3578925	3578942	2	9	18	GT	31	7
82222	8	3589429	3589444	2	8	16	AC	10	4
82235	8	3605596	3605613	2	9	18	CA	15	6
82237	8	3608280	3608303	4	6	24	GGCT	27	3
82238	8	3614413	3614428	2	8	16	AC	27	9

82239	8	3614513	3614530	3	6	18	GCA	21	3
82246	8	3615579	3615594	4	4	16	CTGC	20	3
82247	8	3615881	3615892	2	6	12	AC	15	8
82248	8	3615895	3615906	2	6	12	AC	19	11
82250	8	3617028	3617043	4	4	16	GTTT	19	3
82251	8	3618075	3618098	2	12	24	TG	28	8
82269	8	3651880	3651893	2	7	14	CA	32	3
82274	8	3668437	3668452	2	8	16	CA	25	5
82275	8	3669287	3669310	4	6	24	AAAG	20	8
82276	8	3682748	3682767	4	5	20	ATTG	26	3
82277	8	3689718	3689729	2	6	12	AT	11	4
82278	8	3690338	3690351	2	7	14	TG	28	4
82279	8	3692252	3692271	2	10	20	GT	18	8
82287	8	3716651	3716662	2	6	12	AC	26	3
82288	8	3716883	3716896	2	7	14	GT	30	3
82290	8	3733988	3734002	3	5	15	CCT	36	3
82313	8	3820571	3820590	2	10	20	CA	23	10
82316	8	3825033	3825052	2	10	20	GT	10	10
82319	8	3843561	3843574	2	7	14	GT	31	3
82321	8	3852220	3852251	2	16	32	GT	7	9
82326	8	3870701	3870728	2	14	28	CA	13	10
82333	8	3899583	3899610	2	14	28	CA	6	8
82334	8	3901906	3901925	2	10	20	TC	6	6
82335	8	3902548	3902563	4	4	16	AAAC	23	5
82336	8	3907487	3907516	3	10	30	ACT	30	15
82343	8	3936040	3936065	2	13	26	CA	11	11
82349	8	3953865	3953882	2	9	18	AC	13	10
82350	8	3963197	3963208	2	6	12	AC	36	4
82368	8	4030409	4030428	2	10	20	CA	15	11
82371	8	4038766	4038780	3	5	15	TTG	26	4
82373	8	4052312	4052329	2	9	18	CA	19	13
82374	8	4056645	4056656	2	6	12	TG	26	3
82395	8	4097447	4097458	2	6	12	AT	28	5
82396	8	4097849	4097866	3	6	18	GAG	18	3
82398	8	4103232	4103259	4	7	28	TCAT	23	5
82399	8	4103882	4103897	2	8	16	CT	26	5
82404	8	4117736	4117751	2	8	16	GT	23	16
82405	8	4118913	4118927	3	5	15	TGT	28	11
82413	8	4130871	4130884	2	7	14	AC	15	3
82416	8	4131298	4131319	2	11	22	TG	20	8
82418	8	4137519	4137530	2	6	12	AG	22	5
82420	8	4154955	4154990	2	18	36	CA	7	7
82424	8	4158395	4158409	3	5	15	TGT	15	5
82425	8	4158429	4158450	2	11	22	GA	16	7
82429	8	4165820	4165831	2	6	12	TG	32	4
82433	8	4175367	4175380	2	7	14	AT	15	6
82437	8	4186805	4186837	3	11	33	TGA	26	7
82447	8	4221397	4221423	3	9	27	TTA	10	3
82449	8	4222006	4222033	2	14	28	AC	6	12
82450	8	4222312	4222347	2	18	36	TG	12	10
82451	8	4223431	4223464	2	17	34	GT	14	14

82461	8	4276186	4276197	2	6	12	TA	30	3
82462	8	4281948	4281975	4	7	28	AAAT	16	3
82473	8	4289490	4289501	2	6	12	AG	19	3
82475	8	4299657	4299676	2	10	20	GT	10	3
82476	8	4300481	4300500	2	10	20	TC	23	4
82477	8	4301606	4301617	2	6	12	CA	27	4
82484	8	4313963	4313980	2	9	18	CA	24	8
82489	8	4323421	4323432	2	6	12	CA	33	4
82516	8	4404574	4404587	2	7	14	AC	18	10
82517	8	4404728	4404751	2	12	24	GA	10	12
82530	8	4406884	4406901	2	9	18	TG	20	7
82532	8	4407206	4407232	3	9	27	AGC	23	7
82536	8	4411680	4411699	4	5	20	TCTG	25	3
82539	8	4417948	4417969	2	11	22	CA	21	5
82548	8	4441425	4441484	4	15	60	ATTG	24	10
82552	8	4451925	4451944	2	10	20	AC	9	8
82568	8	4497983	4498000	2	9	18	CA	23	8
82570	8	4507100	4507149	2	25	50	CA	15	12
82571	8	4507652	4507667	2	8	16	GA	21	7
82594	8	4559508	4559545	2	19	38	GT	15	10
82596	8	4571178	4571203	2	13	26	CA	7	7
82597	8	4571953	4571972	4	5	20	GGAT	13	4
82598	8	4578064	4578090	3	9	27	GCT	17	5
82599	8	4579939	4579950	2	6	12	TG	20	3
82600	8	4580354	4580401	3	16	48	TTA	12	9
82601	8	4583876	4583901	2	13	26	TG	16	6
82610	8	4616045	4616058	2	7	14	GT	25	6
82611	8	4618300	4618315	2	8	16	CA	27	5
82614	8	4623357	4623377	3	7	21	TCC	22	4
82615	8	4627677	4627710	2	17	34	GT	15	7
82621	8	4634075	4634094	2	10	20	GT	19	8
82636	8	4657602	4657615	2	7	14	TG	15	14
82637	8	4657617	4657630	2	7	14	GT	20	18
82638	8	4659237	4659260	2	12	24	CA	13	10
82639	8	4669954	4669968	3	5	15	TTA	11	10
82647	8	4689417	4689438	2	11	22	AC	13	7
82656	8	4703161	4703186	2	13	26	GT	19	5
82674	8	4775645	4775658	2	7	14	CA	24	3
82675	8	4783883	4783896	2	7	14	GT	33	3
82678	8	4790245	4790265	3	7	21	GTA	18	3
82680	8	4792540	4792554	3	5	15	GAG	31	5
82685	8	4800469	4800500	2	16	32	TG	8	10
82686	8	4800778	4800807	3	10	30	TCC	21	7
82687	8	4800902	4800919	3	6	18	CCT	14	6
82689	8	4801050	4801064	3	5	15	TCC	16	4
82690	8	4801205	4801228	3	8	24	CTC	28	7
82693	8	4803245	4803276	2	16	32	CA	12	6
82704	8	4824970	4824989	2	10	20	GT	13	8
82714	8	4884358	4884381	2	12	24	CA	20	8
82720	8	4896463	4896482	2	10	20	TA	11	7
82721	8	4905079	4905096	2	9	18	TG	30	3

82727	8	4910736	4910750	3	5	15	AGG	20	5
82731	8	4914087	4914120	2	17	34	CA	8	10
82733	8	4914205	4914232	2	14	28	TG	9	10
82734	8	4914508	4914541	2	17	34	GT	13	9
82737	8	4915551	4915580	2	15	30	GT	15	10
82738	8	4918681	4918698	3	6	18	CTG	27	6
82739	8	4919869	4919900	2	16	32	AC	16	10
82740	8	4920032	4920075	2	22	44	AC	7	7
82741	8	4921331	4921358	2	14	28	GT	16	11
82742	8	4922151	4922168	3	6	18	CAA	21	3
82749	8	4931860	4931877	2	9	18	AC	18	5
82753	8	4937184	4937197	2	7	14	GT	27	8
82754	8	4938322	4938339	2	9	18	TG	18	6
82755	8	4938341	4938352	2	6	12	GT	25	4
82763	8	4948815	4948852	2	19	38	CA	13	13
82766	8	4950914	4950943	2	15	30	CA	20	10
82767	8	4954315	4954344	2	15	30	GT	7	10
82770	8	4963777	4963794	2	9	18	AC	18	5
82772	8	4967694	4967735	2	21	42	GT	9	8
82776	8	4976167	4976188	2	11	22	TG	19	9
82778	8	4978609	4978620	2	6	12	GT	25	4
82779	8	4979919	4979934	4	4	16	ATTT	9	6
82783	8	4987111	4987122	2	6	12	CA	16	3
82785	8	4987734	4987747	2	7	14	GT	13	8
82787	8	4989711	4989736	2	13	26	GT	14	10
82789	8	4993150	4993161	2	6	12	GT	15	4
82790	8	4993164	4993177	2	7	14	GT	14	4
82792	8	4993626	4993646	3	7	21	GAA	26	9
82794	8	4998265	4998282	2	9	18	AC	17	7
82797	8	5004229	5004240	2	6	12	TG	29	3
82799	8	5004638	5004649	2	6	12	AC	28	4
82802	8	5009460	5009497	2	19	38	AC	11	14
82806	8	5012672	5012685	2	7	14	AC	15	11
82817	8	5021461	5021476	4	4	16	CACG	16	13
82819	8	5025528	5025543	2	8	16	AC	10	5
82820	8	5040633	5040644	2	6	12	TC	20	5
82831	8	5066640	5066663	4	6	24	AAAT	8	4
82840	8	5091054	5091089	2	18	36	TG	12	10
82855	8	5113493	5113530	2	19	38	CA	7	9
82862	8	5137767	5137787	3	7	21	TCA	28	5
82863	8	5144729	5144740	2	6	12	TA	20	4
82866	8	5173312	5173323	2	6	12	TC	22	3
82871	8	5187833	5187858	2	13	26	AC	13	5
82873	8	5189685	5189720	4	9	36	AAAC	14	7
82878	8	5213483	5213504	2	11	22	TG	20	8
82881	8	5219221	5219238	3	6	18	AAG	19	3
82883	8	5222565	5222624	4	15	60	CTTT	18	15
82889	8	5237318	5237335	2	9	18	CA	28	6
82890	8	5241011	5241026	2	8	16	TG	11	5
82891	8	5241028	5241039	2	6	12	GT	10	5
82893	8	5243261	5243290	2	15	30	GT	15	13

82895	8	5245907	5245920	2	7	14	TG	28	5
82901	8	5257679	5257706	2	14	28	CA	15	7
82903	8	5259857	5259886	2	15	30	GT	7	8
82904	8	5260074	5260095	2	11	22	TG	18	5
82906	8	5262860	5262879	2	10	20	CA	13	6
82908	8	5262967	5263008	2	21	42	TG	7	8
82913	8	5268232	5268247	4	4	16	TCCC	7	3
82914	8	5268591	5268606	2	8	16	GT	15	4
82918	8	5279337	5279362	2	13	26	CA	21	11
82919	8	5281005	5281026	2	11	22	CA	24	5
82928	8	5295201	5295212	2	6	12	AG	11	6
82930	8	5301512	5301557	2	23	46	CA	16	14
82934	8	5317161	5317200	2	20	40	GT	20	13
82938	8	5332270	5332287	2	9	18	AG	19	4
82944	8	5342741	5342764	3	8	24	TCA	23	4
82945	8	5344983	5344998	2	8	16	AT	11	11
82946	8	5347856	5347869	2	7	14	AC	19	4
82947	8	5347871	5347882	2	6	12	CA	13	4
82948	8	5352526	5352543	3	6	18	AAG	13	3
82964	8	5386552	5386579	2	14	28	TG	15	13
82965	8	5386763	5386774	2	6	12	GT	20	6
82974	8	5401101	5401124	3	8	24	TCT	18	3
82975	8	5402369	5402396	2	14	28	AC	8	7
82983	8	5413139	5413166	2	14	28	GT	15	9
82984	8	5421787	5421802	2	8	16	TG	15	11
82988	8	5428678	5428691	2	7	14	GT	29	4
82991	8	5439145	5439182	2	19	38	AC	7	9
82992	8	5443015	5443029	3	5	15	CCA	31	3
82993	8	5445154	5445175	2	11	22	TG	22	12
82995	8	5447540	5447557	2	9	18	TG	20	8
83006	8	5473763	5473786	3	8	24	GAA	16	3
83011	8	5480131	5480166	2	18	36	TG	21	8
83013	8	5490636	5490655	2	10	20	TA	19	11
83020	8	5511821	5511842	2	11	22	AC	10	5
83021	8	5515571	5515596	2	13	26	GA	7	6
83022	8	5521831	5521842	2	6	12	CA	14	3
83023	8	5523371	5523392	2	11	22	AC	21	11
83024	8	5523685	5523720	2	18	36	AC	18	10
83027	8	5528193	5528238	2	23	46	CA	16	19
83029	8	5540686	5540699	2	7	14	TG	18	6
83030	8	5540700	5540715	2	8	16	AG	20	6
83035	8	5542589	5542615	3	9	27	CCT	18	5
83038	8	5549018	5549035	2	9	18	CA	21	16
83040	8	5553230	5553253	3	8	24	TTC	9	3
83042	8	5560872	5560899	2	14	28	TG	14	6
83049	8	5570190	5570205	4	4	16	AAAG	24	5
83051	8	5570614	5570637	3	8	24	ACA	6	3
83053	8	5572875	5572904	2	15	30	GT	15	6
83055	8	5574736	5574763	2	14	28	GT	15	10
83057	8	5576880	5576906	3	9	27	ATG	25	10
83060	8	5580609	5580623	3	5	15	TTG	13	7

83065	8	5586464	5586481	3	6	18	TGA	27	3
83071	8	5594337	5594354	2	9	18	TG	22	12
83075	8	5595042	5595071	2	15	30	CT	11	8
83078	8	5595834	5595847	2	7	14	CT	25	5
83080	8	5598511	5598532	2	11	22	TG	15	6
83084	8	5603784	5603805	2	11	22	TC	21	6
83089	8	5611746	5611773	4	7	28	GTTG	25	8
83090	8	5612198	5612239	2	21	42	GT	8	8
83092	8	5615763	5615796	2	17	34	AC	15	9
83094	8	5622420	5622443	3	8	24	CCT	23	5
83095	8	5623361	5623380	2	10	20	CT	24	5
83096	8	5630560	5630587	2	14	28	CA	19	11
83101	8	5644751	5644782	2	16	32	TG	14	10
83105	8	5654339	5654358	2	10	20	CA	15	14
83109	8	5658935	5658962	2	14	28	TG	19	10
83112	8	5664294	5664314	3	7	21	CCT	16	5
83113	8	5664752	5664785	2	17	34	TG	10	9
83114	8	5664844	5664865	2	11	22	GA	16	5
83115	8	5664976	5665015	2	20	40	GT	14	18
83120	8	5666891	5666906	2	8	16	GA	14	4
83122	8	5667856	5667885	2	15	30	GT	16	9
83134	8	5675037	5675066	2	15	30	TG	19	10
83135	8	5675102	5675122	3	7	21	ATG	14	3
83141	8	5680067	5680090	3	8	24	AGC	31	5
83142	8	5682276	5682329	2	27	54	CA	8	13
83148	8	5691019	5691036	2	9	18	CA	17	6
83153	8	5691464	5691493	2	15	30	CA	10	6
83155	8	5693141	5693166	2	13	26	CA	7	12
83160	8	5694509	5694548	2	20	40	AG	7	7
83163	8	5696710	5696725	2	8	16	TG	21	4
83165	8	5700933	5700948	2	8	16	AC	17	13
83172	8	5709879	5709898	2	10	20	TG	14	8
83178	8	5715294	5715305	2	6	12	GT	27	3
83183	8	5719133	5719160	2	14	28	CA	14	13
83184	8	5721594	5721611	2	9	18	CT	24	6
83188	8	5728431	5728456	2	13	26	AC	11	7
83198	8	5741072	5741101	2	15	30	GT	7	7
83199	8	5743967	5743992	2	13	26	CA	23	12
83204	8	5748179	5748216	2	19	38	TG	7	6
83215	8	5768127	5768146	2	10	20	AC	22	7
83217	8	5770478	5770513	2	18	36	TG	11	9
83218	8	5770904	5770929	2	13	26	TG	10	8
83219	8	5771070	5771081	2	6	12	CA	19	10
83221	8	5773117	5773138	2	11	22	GT	8	8
83222	8	5773415	5773430	2	8	16	TG	29	3
83223	8	5774791	5774818	2	14	28	TG	10	9
83224	8	5776345	5776358	2	7	14	GA	22	3
83229	8	5780310	5780336	3	9	27	AAG	18	4
83231	8	5781114	5781125	2	6	12	AG	28	3
83234	8	5784763	5784780	2	9	18	TG	25	5
83238	8	5787220	5787246	3	9	27	CTC	11	6

83240	8	5789445	5789478	2	17	34	TG	8	8
83241	8	5789539	5789550	2	6	12	GT	13	3
83242	8	5789719	5789742	2	12	24	CA	18	11
83244	8	5790229	5790248	2	10	20	TC	14	6
83250	8	5797589	5797604	2	8	16	TG	12	9
83256	8	5808076	5808091	4	4	16	AAAC	25	3
83260	8	5812996	5813007	2	6	12	GT	29	6
83262	8	5814730	5814755	2	13	26	TC	24	5
83263	8	5817395	5817418	2	12	24	AC	14	8
83265	8	5819331	5819350	2	10	20	AC	15	7
83267	8	5824127	5824170	2	22	44	TG	14	13
83269	8	5825489	5825503	3	5	15	TTG	20	14
83282	8	5837338	5837369	4	8	32	ACAG	27	13
83286	8	5840292	5840307	2	8	16	GT	16	4
83290	8	5841215	5841232	2	9	18	AG	18	4
83293	8	5843496	5843509	2	7	14	CA	10	7
83296	8	5846065	5846076	2	6	12	AC	23	3
83299	8	5853048	5853073	2	13	26	AC	18	5
83302	8	5858273	5858290	2	9	18	AG	16	8
83303	8	5858293	5858304	2	6	12	AG	10	6
83307	8	5874737	5874788	2	26	52	CA	7	12
83308	8	5876973	5876990	2	9	18	TG	15	6
83310	8	5880323	5880336	2	7	14	AC	21	4
83313	8	5894739	5894754	4	4	16	AAAT	18	3
83314	8	5895829	5895852	3	8	24	ATT	11	4
83317	8	5905283	5905303	3	7	21	GTG	22	6
83327	8	5928800	5928819	2	10	20	CA	22	5
83330	8	5933131	5933164	2	17	34	TC	21	13
83333	8	5937834	5937865	2	16	32	AC	11	10
83337	8	5944504	5944521	2	9	18	CA	20	8
83338	8	5944612	5944623	2	6	12	AC	28	4
83339	8	5944725	5944742	2	9	18	AC	17	9
83343	8	5947960	5947979	2	10	20	CA	10	12
83378	8	5982511	5982526	4	4	16	TATC	17	3
83384	8	5999455	5999478	3	8	24	AGG	16	4
83393	8	6011649	6011678	2	15	30	TG	11	10
83394	8	6013534	6013561	2	14	28	AC	14	10
83396	8	6015584	6015597	2	7	14	GT	23	4
83401	8	6019679	6019690	2	6	12	TA	16	9
83430	8	6053270	6053293	2	12	24	GT	18	6
83433	8	6055882	6055903	2	11	22	GT	17	8
83434	8	6056071	6056082	2	6	12	GT	11	6
83435	8	6056085	6056102	2	9	18	GT	13	12
83436	8	6056211	6056234	2	12	24	TG	12	6
83447	8	6075831	6075846	2	8	16	CA	12	11
83448	8	6075929	6075962	2	17	34	CA	7	8
83452	8	6087077	6087114	2	19	38	AC	15	9
83453	8	6089586	6089601	2	8	16	GT	21	6
83454	8	6089753	6089768	2	8	16	TG	17	3
83456	8	6090566	6090589	3	8	24	CCT	19	3
83459	8	6091427	6091448	2	11	22	TG	15	3

83461	8	6095497	6095522	2	13	26	CA	11	8
83479	8	6115096	6115119	2	12	24	AC	18	8
83481	8	6118108	6118139	2	16	32	GT	15	9
83486	8	6125856	6125877	2	11	22	CA	17	11
83491	8	6138614	6138643	2	15	30	TG	12	10
83496	8	6150230	6150243	2	7	14	AG	25	11
83497	8	6150246	6150259	2	7	14	AG	16	6
83498	8	6150561	6150586	2	13	26	GT	16	14
83499	8	6150744	6150759	2	8	16	TG	22	3
83502	8	6155797	6155824	2	14	28	AC	13	7
83503	8	6155940	6155959	2	10	20	CA	26	3
83513	8	6175417	6175428	2	6	12	AT	22	4
83514	8	6178844	6178861	2	9	18	CA	21	6
83516	8	6183758	6183775	2	9	18	GT	20	13
83521	8	6214643	6214658	2	8	16	AG	25	3
83525	8	6226683	6226694	2	6	12	CA	23	5
83526	8	6230067	6230086	2	10	20	TG	18	12
83533	8	6243033	6243058	2	13	26	TG	14	3
83534	8	6246796	6246821	2	13	26	GT	14	7
83536	8	6249796	6249809	2	7	14	AC	22	8
83537	8	6249947	6249958	2	6	12	TG	27	4
83538	8	6249961	6249972	2	6	12	TG	29	3
83544	8	6261856	6261869	2	7	14	AG	31	4
83548	8	6276571	6276585	3	5	15	GTT	27	3
83556	8	6311328	6311347	2	10	20	CA	13	8
83559	8	6313094	6313119	2	13	26	CT	16	11
83564	8	6321586	6321601	2	8	16	GT	27	5
83571	8	6326008	6326019	2	6	12	GT	22	3
83575	8	6328027	6328038	2	6	12	TG	28	5
83577	8	6329289	6329320	2	16	32	TG	14	15
83578	8	6329470	6329485	2	8	16	TC	16	6
83579	8	6330470	6330481	2	6	12	GA	21	3
83580	8	6330835	6330852	2	9	18	GT	23	8
83583	8	6334659	6334688	2	15	30	CA	12	11
83584	8	6335576	6335609	2	17	34	CA	14	9
83585	8	6336372	6336389	2	9	18	CT	17	6
83594	8	6348582	6348613	2	16	32	TC	6	8
83597	8	6352603	6352623	3	7	21	CCT	19	5
83601	8	6375013	6375040	4	7	28	ATGT	20	3
83602	8	6375835	6375852	2	9	18	CA	22	9
83603	8	6376575	6376592	2	9	18	GT	15	10
83607	8	6382701	6382716	2	8	16	TG	14	7
83613	8	6387361	6387380	4	5	20	AAAT	25	7
83616	8	6393007	6393026	2	10	20	AC	11	6
83620	8	6399855	6399872	3	6	18	TAT	16	3
83621	8	6406863	6406878	2	8	16	TC	22	5
83624	8	6418605	6418626	2	11	22	GT	17	4
83625	8	6418687	6418722	2	18	36	TG	10	12
83628	8	6422421	6422436	2	8	16	AC	29	7
83635	8	6439129	6439150	2	11	22	CT	19	6
83637	8	6440082	6440097	2	8	16	GT	30	3

83638	8	6440302	6440319	2	9	18	TG	27	6
83640	8	6444096	6444111	2	8	16	AC	19	4
83642	8	6451532	6451547	2	8	16	TG	26	5
83643	8	6452195	6452226	4	8	32	GCCT	25	5
83644	8	6465518	6465533	2	8	16	AC	26	4
83645	8	6468129	6468144	2	8	16	TG	12	4
83647	8	6491282	6491319	2	19	38	GT	7	7
83649	8	6496433	6496446	2	7	14	AC	25	4
83653	8	6504845	6504864	4	5	20	TTTA	15	3
83657	8	6511666	6511681	2	8	16	GT	30	5
83658	8	6512697	6512726	2	15	30	GT	16	11
83659	8	6513296	6513335	2	20	40	GT	13	13
83662	8	6518355	6518368	2	7	14	TG	26	3
83663	8	6518387	6518400	2	7	14	TG	20	4
83666	8	6522288	6522311	2	12	24	TC	21	5
83670	8	6536417	6536430	2	7	14	GT	18	3
83681	8	6558255	6558292	2	19	38	CA	6	9
83682	8	6558593	6558612	2	10	20	GT	21	3
83686	8	6571178	6571195	2	9	18	AC	9	10
83688	8	6582482	6582493	2	6	12	CA	31	3
83690	8	6585957	6585977	3	7	21	CAG	23	5
83694	8	6591895	6591910	2	8	16	TG	30	4
83695	8	6592022	6592041	2	10	20	CA	20	6
83697	8	6595810	6595827	3	6	18	ATG	30	3
83703	8	6605245	6605262	2	9	18	GT	24	6
83705	8	6606056	6606067	2	6	12	CT	20	4
83708	8	6608432	6608445	2	7	14	TG	25	4
83709	8	6608579	6608593	3	5	15	AAT	16	6
83710	8	6608961	6608978	3	6	18	CTT	24	4
83715	8	6623475	6623500	2	13	26	AC	8	9
83717	8	6645285	6645298	2	7	14	TG	19	7
83718	8	6645829	6645848	4	5	20	ATTT	19	4
83720	8	6648387	6648420	2	17	34	GA	19	8
83727	8	6652354	6652365	2	6	12	AG	25	4
83728	8	6652743	6652760	2	9	18	AG	21	6
83729	8	6652916	6652931	4	4	16	CTGC	27	4
83730	8	6653119	6653132	2	7	14	TG	8	3
83732	8	6653613	6653636	2	12	24	TG	14	15
83739	8	6663255	6663272	2	9	18	TG	22	5
83742	8	6670729	6670740	2	6	12	GT	27	3
83744	8	6673669	6673702	2	17	34	GT	7	11
83746	8	6677304	6677318	3	5	15	TGC	29	4
83747	8	6678082	6678102	3	7	21	CAT	25	3
83750	8	6679697	6679724	2	14	28	AC	16	6
83752	8	6684314	6684347	2	17	34	TG	21	5
83755	8	6686390	6686405	2	8	16	TC	28	4
83756	8	6687010	6687041	2	16	32	TG	12	9
83757	8	6688847	6688858	2	6	12	TG	18	5
83758	8	6689063	6689074	2	6	12	CA	25	3
83779	8	6715518	6715535	3	6	18	AGC	24	4
83780	8	6720349	6720366	2	9	18	TG	17	5

83782	8	6721082	6721095	2	7	14	TG	23	3
83799	8	6758053	6758064	2	6	12	AC	14	11
83800	8	6758066	6758083	2	9	18	CA	17	13
83802	8	6764586	6764609	3	8	24	AAG	25	7
83807	8	6768876	6768889	2	7	14	CA	20	5
83812	8	6778331	6778357	3	9	27	AAC	19	5
83818	8	6792495	6792528	2	17	34	AG	15	8
83828	8	6799776	6799805	2	15	30	AC	12	13
83831	8	6805809	6805826	2	9	18	AC	15	12
83832	8	6805881	6805904	2	12	24	AC	10	5
83837	8	6808694	6808713	2	10	20	AC	21	11
83841	8	6813384	6813411	2	14	28	AC	8	5
83865	8	6829715	6829732	2	9	18	TG	21	7
83867	8	6831388	6831399	2	6	12	TA	25	5
83871	8	6837469	6837480	2	6	12	GT	12	3
83874	8	6840994	6841029	2	18	36	AC	6	9
83880	8	6858184	6858205	2	11	22	CA	9	7
83882	8	6860469	6860486	2	9	18	TG	26	6
83889	8	6879102	6879125	3	8	24	GAT	19	5
83892	8	6893696	6893740	3	15	45	CTC	10	7
83896	8	6900036	6900059	2	12	24	AC	15	7
83903	8	6917429	6917444	2	8	16	TG	6	4
83910	8	6931436	6931459	2	12	24	GT	15	6
83912	8	6932980	6933015	2	18	36	TG	10	11
83913	8	6935047	6935082	2	18	36	CA	9	10
83914	8	6936374	6936389	2	8	16	CA	19	6
83916	8	6936758	6936769	2	6	12	CA	28	3
83917	8	6939421	6939448	2	14	28	AC	8	4
83918	8	6944156	6944169	2	7	14	TG	23	3
83919	8	6946359	6946373	3	5	15	TTA	7	3
83938	8	6989193	6989218	2	13	26	TG	9	11
83941	8	6994273	6994306	2	17	34	GT	13	13
83946	8	7010364	7010395	4	8	32	AGAC	21	12
83949	8	7018278	7018289	2	6	12	GT	29	4
83950	8	7018610	7018623	2	7	14	AT	12	8
83953	8	7031958	7031969	2	6	12	AC	13	4
83954	8	7031971	7031984	2	7	14	CA	23	6
83961	8	7046316	7046357	2	21	42	TG	14	18
83965	8	7049718	7049737	2	10	20	AC	21	5
83974	8	7079189	7079224	4	9	36	GGAT	19	13
83976	8	7104661	7104678	2	9	18	AC	14	7
83977	8	7104681	7104694	2	7	14	AC	18	7
83982	8	7119193	7119222	2	15	30	CT	12	9
83984	8	7122016	7122047	4	8	32	TCTA	11	9
83995	8	7160646	7160663	2	9	18	AC	10	4
84005	8	7172197	7172208	2	6	12	GT	27	3
84006	8	7176102	7176135	2	17	34	CA	8	8
84008	8	7179561	7179581	3	7	21	CCT	25	3
84017	8	7202733	7202752	2	10	20	TC	26	5
84026	8	7213219	7213236	3	6	18	GTG	14	5
84027	8	7214008	7214025	3	6	18	CAG	35	3

84028	8	7214073	7214086	2	7	14	AC	28	4
84029	8	7214513	7214526	2	7	14	CA	20	4
84030	8	7219373	7219386	2	7	14	AC	20	8
84031	8	7219388	7219399	2	6	12	CA	22	7
84034	8	7228194	7228217	2	12	24	GT	7	5
84037	8	7239848	7239859	2	6	12	AC	32	5
84038	8	7244005	7244022	2	9	18	TG	24	5
84042	8	7251318	7251333	2	8	16	CA	26	4
84043	8	7251388	7251409	2	11	22	TG	25	4
84044	8	7252110	7252130	3	7	21	ATC	26	5
84045	8	7254759	7254776	2	9	18	AG	18	9
84046	8	7254778	7254797	2	10	20	GA	16	8
84049	8	7262062	7262079	2	9	18	TC	20	4
84052	8	7268265	7268284	2	10	20	GT	11	7
84053	8	7268286	7268301	2	8	16	TG	13	7
84054	8	7268629	7268652	2	12	24	AC	12	6
84055	8	7268848	7268861	2	7	14	CA	19	4
84057	8	7271077	7271091	3	5	15	AAG	20	3
84062	8	7287112	7287127	4	4	16	TATC	7	3
84068	8	7305700	7305715	4	4	16	AAAT	25	7
84078	8	7320683	7320720	2	19	38	CA	12	12
84079	8	7325674	7325713	2	20	40	TG	12	8
84082	8	7329968	7329991	2	12	24	TC	14	5
84083	8	7330412	7330437	2	13	26	AC	7	5
84093	8	7355951	7355964	2	7	14	CA	19	3
84094	8	7356040	7356061	2	11	22	CA	23	10
84095	8	7356325	7356336	2	6	12	CA	27	4
84097	8	7357645	7357666	2	11	22	CA	12	14
84098	8	7358992	7359011	2	10	20	CA	26	6
84100	8	7361330	7361347	2	9	18	AT	20	4
84102	8	7364820	7364843	2	12	24	GT	10	7
84105	8	7372527	7372550	2	12	24	TG	13	9
84109	8	7381766	7381789	2	12	24	AC	12	8
84111	8	7383729	7383746	2	9	18	AT	11	5
84115	8	7389460	7389473	2	7	14	AC	23	4
84116	8	7391799	7391838	2	20	40	CA	9	10
84117	8	7399282	7399293	2	6	12	AC	9	5
84118	8	7405725	7405736	2	6	12	CA	35	3
84120	8	7410886	7410917	2	16	32	GA	7	8
84121	8	7419217	7419228	2	6	12	CA	15	6
84123	8	7421827	7421842	2	8	16	CT	21	3
84129	8	7433871	7433926	2	28	56	GT	12	14
84133	8	7442003	7442016	2	7	14	CA	23	5
84134	8	7444245	7444264	2	10	20	CA	23	6
84135	8	7444424	7444439	2	8	16	TG	23	4
84138	8	7446497	7446512	2	8	16	GA	29	4
84144	8	7462897	7462910	2	7	14	TG	26	6
84147	8	7467273	7467288	4	4	16	CACG	19	5
84162	8	7518865	7518882	3	6	18	AAC	27	3
84163	8	7520067	7520078	2	6	12	GA	32	3
84166	8	7523630	7523645	2	8	16	GT	34	3

84181	8	7579000	7579011	2	6	12	TC	31	3
84193	8	7611627	7611640	2	7	14	GA	11	5
84194	8	7619485	7619502	3	6	18	GAG	22	4
84196	8	7619800	7619826	3	9	27	GGA	7	3
84201	8	7625670	7625693	3	8	24	TGC	27	3
84203	8	7628609	7628636	2	14	28	GT	12	7
84214	8	7661588	7661599	2	6	12	GT	15	3
84217	8	7664650	7664687	2	19	38	AC	8	7
84222	8	7685722	7685745	4	6	24	TATT	13	7
84223	8	7686401	7686414	2	7	14	GT	20	4
84231	8	7715924	7715944	3	7	21	AGA	30	3
84232	8	7718038	7718049	2	6	12	AT	26	5
84233	8	7718206	7718223	3	6	18	CTG	30	7
84239	8	7724255	7724274	2	10	20	AG	25	3
84240	8	7724409	7724422	2	7	14	TG	25	5
84241	8	7724530	7724545	2	8	16	AG	28	3
84243	8	7724962	7724994	3	11	33	TCT	26	8
84250	8	7738484	7738519	2	18	36	AG	11	8
84257	8	7754166	7754181	4	4	16	TATC	13	8
84266	8	7790847	7790870	3	8	24	GAT	24	4
84274	8	7803020	7803037	2	9	18	TG	19	6
84277	8	7819795	7819816	2	11	22	TC	17	4
84284	8	7841039	7841065	3	9	27	GAG	22	6
84288	8	7841567	7841596	2	15	30	TG	22	12
84290	8	7843483	7843503	3	7	21	TCC	33	3
84295	8	7854410	7854433	2	12	24	AC	12	6
84298	8	7855653	7855673	3	7	21	TCT	16	3
84300	8	7859415	7859446	4	8	32	ATAG	15	7
84301	8	7870760	7870791	2	16	32	AC	13	13
84302	8	7870972	7870995	2	12	24	GT	9	9
84307	8	7881753	7881786	2	17	34	GT	7	7
84308	8	7886421	7886452	2	16	32	CA	9	13
84313	8	7891503	7891518	2	8	16	CA	22	5
84318	8	7899446	7899473	4	7	28	TTGG	24	4
84319	8	7899550	7899575	2	13	26	AG	17	8
84321	8	7904884	7904901	2	9	18	CT	17	9
84325	8	7909545	7909566	2	11	22	GT	14	6
84362	8	7939641	7939656	2	8	16	TC	22	3
84363	8	7945778	7945804	3	9	27	AGC	24	3
84376	8	7983097	7983122	2	13	26	AC	22	9
84378	8	7983754	7983775	2	11	22	TG	17	13
84381	8	7984062	7984079	2	9	18	GT	8	8
84385	8	7985055	7985066	2	6	12	TC	13	3
84387	8	7985977	7985992	4	4	16	GTTT	23	3
84395	8	8000450	8000469	4	5	20	CTTA	29	7
84396	8	8001146	8001160	3	5	15	CCT	19	5
84401	8	8029192	8029205	2	7	14	CA	25	11
84402	8	8029270	8029281	2	6	12	GT	24	4
84403	8	8029656	8029673	2	9	18	AC	23	6
84406	8	8033690	8033707	2	9	18	TG	13	9
84408	8	8043686	8043706	3	7	21	GCA	22	3

84409	8	8044387	8044398	2	6	12	AC	22	3
84411	8	8047577	8047592	4	4	16	GACA	31	3
84412	8	8047742	8047763	2	11	22	TG	18	9
84413	8	8047905	8047920	2	8	16	CA	18	6
84420	8	8055859	8055902	2	22	44	GA	6	8
84422	8	8056176	8056191	2	8	16	TC	11	4
84423	8	8056199	8056212	2	7	14	CT	9	3
84424	8	8056391	8056404	2	7	14	GT	24	3
84425	8	8056408	8056419	2	6	12	TG	18	3
84429	8	8057854	8057873	2	10	20	TC	23	3
84435	8	8070443	8070488	2	23	46	AC	10	10
84436	8	8071304	8071333	2	15	30	TG	12	8
84437	8	8074860	8074881	2	11	22	CA	12	7
84438	8	8075014	8075029	2	8	16	TA	19	3
84442	8	8085600	8085627	2	14	28	CA	24	8
84444	8	8088020	8088045	2	13	26	AC	17	5
84453	8	8106902	8106927	2	13	26	CA	8	6
84457	8	8113103	8113118	2	8	16	AC	16	14
84458	8	8113121	8113136	2	8	16	AC	14	11
84459	8	8114648	8114661	2	7	14	CA	26	4
84464	8	8121079	8121104	2	13	26	CA	19	7
84465	8	8121733	8121746	2	7	14	CA	21	3
84468	8	8127136	8127147	2	6	12	GT	22	8
84474	8	8134253	8134284	4	8	32	AAAT	12	6
84476	8	8134959	8134980	2	11	22	CA	12	11
84477	8	8135118	8135137	2	10	20	TC	9	4
84479	8	8135352	8135383	2	16	32	AG	10	11
84481	8	8137481	8137494	2	7	14	AC	22	3
84486	8	8138175	8138195	3	7	21	TCC	18	7
84487	8	8138350	8138371	2	11	22	GT	15	4
84501	8	8164027	8164044	2	9	18	GT	21	6
84510	8	8190771	8190802	4	8	32	ATTG	25	6
84512	8	8201126	8201161	3	12	36	GTA	12	12
84519	8	8212110	8212124	3	5	15	CAG	23	4
84521	8	8214716	8214729	2	7	14	TG	9	7
84524	8	8215126	8215143	2	9	18	AT	21	4
84525	8	8218543	8218558	2	8	16	GT	22	4
84528	8	8222493	8222508	4	4	16	CATT	25	3
84533	8	8235936	8235953	3	6	18	AAC	21	3
84545	8	8256405	8256440	2	18	36	CA	9	9
84548	8	8266332	8266351	2	10	20	CA	23	7
84555	8	8281467	8281482	2	8	16	TG	19	6
84558	8	8289165	8289188	2	12	24	AC	20	7
84562	8	8305874	8305901	2	14	28	AC	19	7
84569	8	8319384	8319401	2	9	18	TG	10	3
84571	8	8321827	8321846	4	5	20	AAAC	26	3
84572	8	8323161	8323186	2	13	26	AC	22	4
84577	8	8326669	8326692	4	6	24	CTGT	18	5
84587	8	8360230	8360261	2	16	32	GT	18	10
84589	8	8362294	8362317	4	6	24	AAAT	18	18
84591	8	8366065	8366090	2	13	26	AC	14	12

84592	8	8371021	8371044	2	12	24	AC	6	6
84598	8	8379804	8379835	2	16	32	TG	8	10
84614	8	8397183	8397206	2	12	24	CA	12	4
84615	8	8397661	8397676	2	8	16	AC	11	7
84618	8	8401498	8401515	2	9	18	TG	27	3
84620	8	8404616	8404639	2	12	24	TG	23	8
84623	8	8406447	8406476	2	15	30	TG	11	9
84624	8	8406907	8406934	2	14	28	AC	6	8
84625	8	8409350	8409361	2	6	12	AC	21	5
84626	8	8409363	8409378	2	8	16	CA	9	4
84628	8	8410955	8410966	2	6	12	CA	28	4
84629	8	8411374	8411403	2	15	30	AC	18	7
84635	8	8418639	8418654	4	4	16	AAAT	21	7
84641	8	8423079	8423098	2	10	20	AC	16	5
84642	8	8430070	8430105	4	9	36	TGAT	18	13
84645	8	8437844	8437858	3	5	15	AAC	27	3
84647	8	8439191	8439220	3	10	30	GAG	10	6
84648	8	8440226	8440253	2	14	28	CA	19	9
84652	8	8448397	8448412	2	8	16	GT	15	6
84653	8	8448533	8448560	2	14	28	AG	14	6
84656	8	8458607	8458632	2	13	26	TG	11	5
84657	8	8459371	8459394	2	12	24	GT	6	5
84658	8	8460343	8460358	2	8	16	AG	24	3
84660	8	8470993	8471012	2	10	20	AC	25	5
84661	8	8471136	8471161	2	13	26	CA	11	9
84663	8	8472182	8472199	2	9	18	CA	21	14
84664	8	8474559	8474572	2	7	14	GT	30	5
84665	8	8485302	8485315	2	7	14	CA	21	3
84666	8	8489134	8489147	2	7	14	TG	30	4
84672	8	8492702	8492745	4	11	44	TATC	16	8
84673	8	8493422	8493451	3	10	30	CAA	23	7
84676	8	8504095	8504130	2	18	36	AC	11	9
84692	8	8548072	8548083	2	6	12	CA	22	3
84696	8	8581775	8581804	2	15	30	CA	12	13
84698	8	8597002	8597027	2	13	26	GT	11	10
84701	8	8606827	8606848	2	11	22	AC	20	5
84712	8	8621513	8621533	3	7	21	GAG	28	3
84713	8	8623543	8623558	2	8	16	CT	24	3
84715	8	8623710	8623727	2	9	18	GA	9	10
84719	8	8625930	8625957	2	14	28	TG	16	9
84720	8	8629954	8630011	2	29	58	GA	12	11
84722	8	8631834	8631863	2	15	30	CA	14	13
84724	8	8632572	8632583	2	6	12	GT	23	4
84725	8	8632765	8632782	2	9	18	AG	16	5
84729	8	8643385	8643400	2	8	16	AC	23	7
84753	8	8682405	8682440	2	18	36	TG	8	6
84754	8	8682603	8682620	2	9	18	AC	23	8
84764	8	8696160	8696179	2	10	20	GA	16	5
84769	8	8702355	8702378	2	12	24	TC	13	5
84776	8	8709018	8709057	2	20	40	GT	6	8
84777	8	8711201	8711228	2	14	28	CA	12	5

84778	8	8711401	8711412	2	6	12	CA	12	4
84781	8	8714914	8714925	2	6	12	CA	29	4
84784	8	8716658	8716681	2	12	24	AC	11	6
84786	8	8720104	8720135	2	16	32	TG	21	11
84787	8	8720271	8720282	2	6	12	GT	27	6
84789	8	8722621	8722648	2	14	28	CA	23	10
84790	8	8722793	8722804	2	6	12	CA	23	3
84793	8	8724061	8724082	2	11	22	TG	19	10
84794	8	8724811	8724836	2	13	26	TG	14	6
84802	8	8731553	8731564	2	6	12	AT	19	6
84803	8	8732107	8732138	2	16	32	TG	16	14
84806	8	8735664	8735681	2	9	18	TG	27	6
84807	8	8737473	8737520	2	24	48	CA	8	10
84810	8	8738050	8738065	2	8	16	AC	14	8
84814	8	8739483	8739514	2	16	32	CA	18	11
84818	8	8753617	8753650	2	17	34	GT	13	9
84825	8	8758286	8758303	2	9	18	CA	10	6
84827	8	8758847	8758864	2	9	18	TG	20	7
84836	8	8766729	8766768	2	20	40	TG	11	9
84838	8	8767250	8767267	3	6	18	CTC	30	3
84844	8	8781844	8781861	2	9	18	AC	14	5
84849	8	8788796	8788809	2	7	14	AT	12	9
84857	8	8805381	8805410	2	15	30	GT	23	11
84861	8	8829768	8829787	2	10	20	AC	14	9
84863	8	8834450	8834482	3	11	33	TTG	17	9
84879	8	8885150	8885183	2	17	34	TG	23	14
84881	8	8892467	8892482	2	8	16	TG	20	8
84882	8	8896332	8896347	4	4	16	TTTG	9	3
84893	8	8932556	8932571	2	8	16	TG	31	6
84895	8	8932878	8932899	2	11	22	CA	12	4
84898	8	8935702	8935719	2	9	18	GT	27	8
84899	8	8936117	8936136	2	10	20	TG	6	5
84900	8	8939833	8939854	2	11	22	CA	19	7
84902	8	8941291	8941326	3	12	36	CCT	23	7
84903	8	8941957	8941976	2	10	20	TG	10	9
84905	8	8942144	8942187	2	22	44	TG	14	11
84906	8	8942264	8942275	2	6	12	TC	25	3
84907	8	8942277	8942288	2	6	12	CT	24	4
84909	8	8952561	8952572	2	6	12	AC	31	4
84911	8	8954903	8954929	3	9	27	GAG	22	5
84913	8	8957997	8958020	2	12	24	TG	11	8
84917	8	8961932	8961949	3	6	18	GTT	26	5
84918	8	8963644	8963677	2	17	34	TG	13	8
84921	8	8972173	8972184	2	6	12	AT	21	3
84926	8	8994299	8994326	2	14	28	GT	13	9
84927	8	8996809	8996824	2	8	16	TG	12	8
84936	8	9018382	9018408	3	9	27	TAT	11	5
84937	8	9019410	9019431	2	11	22	AC	20	11
84940	8	9024000	9024013	2	7	14	GT	21	11
84944	8	9030577	9030590	2	7	14	CA	11	3
84961	8	9041037	9041078	2	21	42	TG	9	6

84963	8	9043408	9043431	2	12	24	CA	27	4
84964	8	9058783	9058800	3	6	18	TGC	29	4
84979	8	9102904	9102919	4	4	16	AAAC	28	4
84989	8	9143307	9143324	3	6	18	GCT	17	4
84990	8	9145423	9145438	2	8	16	AC	24	6
85000	8	9178963	9178980	3	6	18	AAT	14	3
85003	8	9182010	9182033	2	12	24	CA	21	15
85007	8	9195953	9195970	2	9	18	CA	10	3
85008	8	9198234	9198251	2	9	18	TA	11	4
85011	8	9208430	9208461	2	16	32	AC	6	6
85012	8	9208780	9208815	2	18	36	TC	9	6
85015	8	9210824	9210841	3	6	18	GCT	18	6
85026	8	9237143	9237166	3	8	24	AAT	18	4
85032	8	9247213	9247230	2	9	18	CA	17	5
85034	8	9261038	9261055	2	9	18	AC	24	6
85035	8	9268940	9268959	2	10	20	CA	7	5
85038	8	9276547	9276561	3	5	15	AAT	16	4
85048	8	9303808	9303851	4	11	44	GATG	7	5
85049	8	9309417	9309430	2	7	14	GT	23	8
85051	8	9324983	9324998	2	8	16	CA	22	8
85052	8	9324999	9325014	4	4	16	CGCA	21	6
85053	8	9325062	9325073	2	6	12	TC	28	3
85054	8	9325191	9325218	4	7	28	TTTA	19	10
85055	8	9327078	9327089	2	6	12	TG	17	4
85060	8	9355769	9355786	2	9	18	GT	24	5
85069	8	9420516	9420529	2	7	14	CA	15	8
85070	8	9433136	9433156	3	7	21	GCA	25	4
85076	8	9455701	9455716	2	8	16	CA	18	10
85077	8	9456264	9456293	3	10	30	GCT	22	9
85080	8	9460779	9460799	3	7	21	GAG	23	3
85085	8	9463971	9463982	2	6	12	CA	18	3
85087	8	9468594	9468625	2	16	32	CA	8	9
85090	8	9470214	9470243	2	15	30	GT	15	10
85091	8	9470475	9470492	2	9	18	CA	16	8
85093	8	9474352	9474403	4	13	52	AAAG	9	8
85097	8	9483781	9483810	2	15	30	AC	7	11
85100	8	9493662	9493689	2	14	28	TG	18	9
85103	8	9502329	9502340	2	6	12	TG	32	3
85116	8	9539881	9539904	3	8	24	TCC	27	3
85136	8	9579260	9579289	3	10	30	AAG	27	6
85139	8	9581935	9581954	2	10	20	TG	19	12
85140	8	9582289	9582304	4	4	16	AAAC	27	8
85142	8	9582808	9582827	4	5	20	TGTT	21	3
85144	8	9585013	9585028	2	8	16	AT	13	3
85147	8	9595659	9595676	3	6	18	CAG	10	3
85152	8	9601941	9601952	2	6	12	AC	28	7
85154	8	9617537	9617558	2	11	22	TG	8	3
85161	8	9628208	9628237	2	15	30	AC	10	12
85162	8	9631980	9631999	2	10	20	AC	25	14
85167	8	9658224	9658241	3	6	18	CCT	16	4
85168	8	9659020	9659035	2	8	16	TG	13	12

85171	8	9662270	9662290	3	7	21	CCT	24	3
85174	8	9684861	9684876	2	8	16	CA	27	4
85193	8	9731732	9731745	2	7	14	TC	23	7
85194	8	9731746	9731759	2	7	14	AC	23	9
85204	8	9781743	9781757	3	5	15	CCT	22	8
85211	8	9809951	9809966	4	4	16	GTAA	27	4
85212	8	9814287	9814304	3	6	18	AGG	27	4
85214	8	9817167	9817178	2	6	12	AT	28	3
85215	8	9818729	9818770	3	14	42	AAG	11	9
85217	8	9818994	9819011	3	6	18	GCT	31	8
85218	8	9819440	9819453	2	7	14	GT	17	9
85226	8	9837304	9837335	2	16	32	CA	9	12
85237	8	9848035	9848068	2	17	34	GT	11	13
85238	8	9848982	9849005	3	8	24	TAT	11	4
85240	8	9849669	9849712	4	11	44	ATCT	16	17
85246	8	9866039	9866054	4	4	16	TTGA	25	5
85247	8	9868824	9868837	2	7	14	AT	16	12
85248	8	9888316	9888333	2	9	18	GT	23	7
85257	8	9913623	9913658	2	18	36	CA	10	9
85267	8	9931133	9931144	2	6	12	TA	24	4
85271	8	9939691	9939714	3	8	24	TGA	23	10
85275	8	9942278	9942301	4	6	24	AAAG	23	7
85290	8	9951779	9951814	2	18	36	CA	9	11
85293	8	9956143	9956158	4	4	16	AAAC	9	3
85295	8	9962816	9962829	2	7	14	AC	30	4
85323	8	10087940	10087971	2	16	32	AC	22	11
85328	8	10101721	10101748	2	14	28	AC	11	7
85335	8	10132462	10132476	3	5	15	AGC	27	6
85339	8	10151829	10151842	2	7	14	CT	23	4
85340	8	10151876	10151891	4	4	16	GGAT	30	3
85343	8	10156049	10156060	2	6	12	AC	24	5
85350	8	10189799	10189819	3	7	21	TCC	31	4
85353	8	10202848	10202861	2	7	14	GT	23	5
85354	8	10209519	10209542	4	6	24	ATTT	26	3
85369	8	10226972	10226987	2	8	16	GA	11	5
85370	8	10227386	10227400	3	5	15	GCG	21	7
85371	8	10227401	10227418	3	6	18	GCA	20	7
85374	8	10234358	10234381	2	12	24	GT	9	7
85380	8	10243771	10243800	2	15	30	CA	12	12
85381	8	10245988	10246011	2	12	24	TG	6	6
85392	8	10260272	10260287	2	8	16	AC	28	6
85395	8	10263455	10263472	2	9	18	GT	15	5
85398	8	10267123	10267138	4	4	16	CTTT	10	8
85412	8	10306500	10306525	2	13	26	TG	11	7
85414	8	10310644	10310665	2	11	22	TG	14	12
85415	8	10310995	10311018	2	12	24	GT	12	9
85416	8	10311402	10311413	2	6	12	TG	21	3
85417	8	10312772	10312783	2	6	12	CA	33	3
85421	8	10314770	10314783	2	7	14	AC	17	3
85424	8	10321306	10321319	2	7	14	GT	16	11
85439	8	10349421	10349448	2	14	28	CA	17	10

85440	8	10351754	10351773	2	10	20	AC	21	7
85441	8	10352013	10352026	2	7	14	GT	29	3
85446	8	10364369	10364384	4	4	16	AAAT	13	4
85455	8	10373821	10373832	2	6	12	AT	17	7
85457	8	10374805	10374818	2	7	14	AC	29	3
85458	8	10376907	10376922	2	8	16	AT	6	4
85468	8	10392005	10392018	2	7	14	CA	26	6
85469	8	10393145	10393174	2	15	30	AC	15	7
85473	8	10396971	10397010	2	20	40	GT	7	7
85475	8	10397886	10397903	2	9	18	AG	24	4
85476	8	10404921	10404940	2	10	20	TG	23	4
85478	8	10415117	10415132	4	4	16	TTTG	20	5
85485	8	10433611	10433622	2	6	12	TA	17	3
85487	8	10438615	10438634	4	5	20	AGCT	32	3
85501	8	10509229	10509242	2	7	14	TG	27	3
85503	8	10509893	10509916	2	12	24	GT	16	5
85513	8	10531597	10531611	3	5	15	GGT	22	5
85515	8	10536419	10536430	2	6	12	CA	18	3
85518	8	10541923	10541937	3	5	15	AAC	34	3
85519	8	10542983	10542997	3	5	15	TAC	31	3
85528	8	10575194	10575213	2	10	20	GT	15	5
85534	8	10592304	10592317	2	7	14	CA	19	3
85536	8	10596273	10596290	2	9	18	TC	20	5
85543	8	10604984	10604997	2	7	14	CA	26	3
85544	8	10611224	10611237	2	7	14	AC	21	4
85545	8	10613598	10613617	2	10	20	GA	14	3
85547	8	10616926	10616943	2	9	18	TC	6	7
85549	8	10618113	10618124	2	6	12	AC	15	9
85550	8	10618126	10618143	2	9	18	CA	15	9
85551	8	10618434	10618447	2	7	14	AG	15	4
85552	8	10618450	10618467	2	9	18	AG	29	5
85555	8	10620127	10620138	2	6	12	AG	26	3
85556	8	10620882	10620905	3	8	24	CTC	18	5
85559	8	10627064	10627099	2	18	36	CA	11	12
85562	8	10635190	10635223	2	17	34	AC	7	7
85567	8	10660689	10660704	4	4	16	AAAC	29	6
85574	8	10665471	10665491	3	7	21	TCC	22	3
85583	8	10669616	10669641	2	13	26	GT	7	7
85588	8	10672202	10672219	2	9	18	CA	28	6
85605	8	10729128	10729153	2	13	26	TG	20	5
85606	8	10729291	10729318	2	14	28	AC	12	7
85609	8	10747818	10747829	2	6	12	CA	34	4
85610	8	10750389	10750412	4	6	24	CTGC	9	3
85615	8	10755337	10755352	2	8	16	TG	12	5
85619	8	10761676	10761693	2	9	18	AG	25	3
85624	8	10764093	10764116	2	12	24	TG	7	4
85630	8	10770480	10770497	3	6	18	GCA	23	4
85635	8	10774954	10774971	2	9	18	CA	18	7
85637	8	10777182	10777209	2	14	28	GT	17	10
85643	8	10793731	10793752	2	11	22	AG	20	5
85662	8	10857656	10857671	2	8	16	AG	14	4

85669	8	10864844	10864864	3	7	21	GGA	29	5
85672	8	10866843	10866884	2	21	42	GT	12	14
85678	8	10892746	10892775	2	15	30	AC	18	9
85679	8	10893138	10893153	2	8	16	CA	20	4
85682	8	10897870	10897887	2	9	18	TC	23	3
85690	8	10904429	10904460	2	16	32	TG	7	8
85692	8	10905207	10905226	2	10	20	CA	15	11
85704	8	10930552	10930569	2	9	18	TG	10	9
85705	8	10930572	10930585	2	7	14	TG	8	7
85709	8	10934771	10934790	2	10	20	TG	15	5
85710	8	10936589	10936604	2	8	16	AC	29	13
85711	8	10936892	10936929	2	19	38	GT	20	14
85716	8	10954088	10954127	2	20	40	CA	13	12
85722	8	10971356	10971375	2	10	20	GT	22	11
85730	8	10984737	10984750	2	7	14	GA	27	3
85735	8	11000936	11000949	2	7	14	GT	15	5
85745	8	11043864	11043877	2	7	14	AC	21	5
85750	8	11053719	11053754	4	9	36	TCTA	10	8
85753	8	11058596	11058613	2	9	18	CA	10	7
85770	8	11110622	11110633	2	6	12	TA	19	4
85779	8	11173249	11173272	2	12	24	AC	13	10
85780	8	11173356	11173381	2	13	26	AC	25	7
85782	8	11178759	11178776	2	9	18	GT	19	6
85802	8	11232565	11232576	2	6	12	TA	22	4
85806	8	11245901	11245912	2	6	12	CA	19	4
85808	8	11255049	11255060	2	6	12	AT	24	5
85809	8	11257192	11257207	4	4	16	TTTC	26	3
85811	8	11263735	11263746	2	6	12	AC	31	3
85820	8	11284403	11284442	4	10	40	TAGA	10	11
85822	8	11287961	11287980	2	10	20	GT	14	3
85827	8	11300705	11300722	3	6	18	CCA	25	3
85828	8	11301659	11301674	2	8	16	CT	30	5
85831	8	11305010	11305039	3	10	30	GCA	16	6
85835	8	11309928	11309941	2	7	14	TG	20	3
85841	8	11315245	11315259	3	5	15	AAT	23	4
85842	8	11315284	11315317	2	17	34	GT	7	7
85846	8	11318147	11318162	2	8	16	TG	23	5
85847	8	11320062	11320073	2	6	12	TC	17	3
85848	8	11320276	11320297	2	11	22	CA	17	7
85849	8	11320457	11320474	2	9	18	TC	14	3
85855	8	11330365	11330379	3	5	15	GCC	29	3
85859	8	11338256	11338279	2	12	24	GA	20	6
85860	8	11342161	11342176	2	8	16	AC	12	7
85862	8	11351486	11351497	2	6	12	CA	23	5
85863	8	11351499	11351510	2	6	12	AC	22	4
85867	8	11356269	11356288	4	5	20	TCTT	31	3
85871	8	11365967	11365984	2	9	18	TG	24	7
85872	8	11366164	11366225	2	31	62	AC	11	11
85874	8	11369460	11369475	2	8	16	CT	21	5
85875	8	11370831	11370852	2	11	22	AC	18	5
85876	8	11378502	11378513	2	6	12	AC	22	3

85878	8	11385416	11385431	4	4	16	CCAA	26	3
85880	8	11386641	11386661	3	7	21	TGC	25	9
85881	8	11386662	11386676	3	5	15	TGT	28	8
85882	8	11386800	11386811	2	6	12	AG	26	3
85886	8	11389665	11389688	2	12	24	GT	16	11
85888	8	11405244	11405259	4	4	16	CGAG	19	4
85889	8	11405260	11405279	4	5	20	CAAG	25	8
85890	8	11410970	11410989	4	5	20	GTTT	25	12
85893	8	11414746	11414761	2	8	16	AT	11	3
85895	8	11426043	11426057	3	5	15	GGA	36	3
85896	8	11426904	11426921	2	9	18	CA	22	4
85899	8	11430581	11430598	3	6	18	CTG	24	4
85902	8	11436081	11436102	2	11	22	AT	11	6
85905	8	11438289	11438321	3	11	33	AGG	13	6
85914	8	11447434	11447448	3	5	15	AAT	10	3
85919	8	11453449	11453468	2	10	20	AG	13	10
85926	8	11458442	11458456	3	5	15	CAC	23	3
85951	8	11491222	11491241	2	10	20	TG	20	6
85954	8	11496325	11496342	2	9	18	GA	28	3
85955	8	11502683	11502698	2	8	16	TG	21	3
85957	8	11504420	11504435	4	4	16	TGTC	29	5
85969	8	11525685	11525702	3	6	18	GCA	24	3
85970	8	11525766	11525783	2	9	18	GT	15	11
85976	8	11532806	11532826	3	7	21	CCT	31	7
85985	8	11544997	11545026	3	10	30	AAG	30	4
85988	8	11548797	11548817	3	7	21	TCC	26	4
85992	8	11558973	11558986	2	7	14	AC	22	5
85994	8	11565853	11565876	3	8	24	ATT	18	7
86014	8	11629394	11629407	2	7	14	TG	26	4
86015	8	11633865	11633886	2	11	22	CA	9	8
86016	8	11634019	11634042	4	6	24	ATAG	16	3
86039	8	11648835	11648846	2	6	12	GT	28	4
86045	8	11660238	11660265	2	14	28	TG	14	8
86050	8	11665972	11666021	2	25	50	CA	11	9
86052	8	11669743	11669757	3	5	15	CCT	28	4
86054	8	11670827	11670853	3	9	27	TCT	25	6
86059	8	11671776	11671795	2	10	20	TG	10	6
86061	8	11673675	11673713	3	13	39	GCA	23	8
86067	8	11687604	11687627	4	6	24	TAAA	22	6
86070	8	11692587	11692598	2	6	12	GA	24	3
86074	8	11696657	11696670	2	7	14	CT	20	6
86077	8	11701256	11701271	4	4	16	TTTA	23	6
86079	8	11702826	11702855	3	10	30	GGT	16	5
86080	8	11704968	11704985	2	9	18	TG	6	6
86085	8	11708135	11708164	2	15	30	GT	16	11
86088	8	11711637	11711656	4	5	20	GCCT	28	6
86095	8	11724702	11724719	2	9	18	AC	18	15
86096	8	11728071	11728096	2	13	26	TC	16	11
86108	8	11743733	11743746	2	7	14	TC	17	4
86121	8	11755131	11755158	2	14	28	TG	10	10
86122	8	11756863	11756884	2	11	22	TG	9	6

86123	8	11757113	11757130	2	9	18	TA	18	4
86128	8	11760872	11760904	3	11	33	TAA	9	3
86149	8	11797283	11797322	4	10	40	CTGG	25	20
86161	8	11811604	11811636	3	11	33	TCC	15	6
86181	8	11836946	11836965	2	10	20	TG	11	4
86187	8	11838928	11838951	4	6	24	TTTA	25	7
86190	8	11849660	11849675	2	8	16	AC	18	4
86193	8	11851750	11851771	2	11	22	GT	13	14
86195	8	11851825	11851838	2	7	14	AC	29	3
86200	8	11860676	11860689	2	7	14	AG	15	3
86202	8	11862938	11862957	2	10	20	AG	16	3
86204	8	11865861	11865888	2	14	28	AC	16	10
86220	8	11898164	11898189	2	13	26	TG	18	10
86222	8	11898643	11898654	2	6	12	AC	18	3
86223	8	11898677	11898694	2	9	18	AC	11	6
86224	8	11899851	11899871	3	7	21	CAT	19	3
86241	8	11937725	11937752	2	14	28	AC	9	6
86245	8	11945116	11945127	2	6	12	GT	15	3
86252	8	11964310	11964325	2	8	16	TG	21	6
86255	8	11964947	11964964	3	6	18	TTA	13	3
86265	8	11990788	11990801	2	7	14	GT	22	8
86266	8	11992995	11993012	3	6	18	ATT	11	3
86270	8	12000638	12000649	2	6	12	TC	25	3
86273	8	12003474	12003497	2	12	24	TG	15	6
86276	8	12006108	12006139	2	16	32	CA	10	11
86278	8	12007077	12007092	2	8	16	AC	20	4
86279	8	12009901	12009936	2	18	36	GA	15	11
86281	8	12013249	12013269	3	7	21	CTC	17	3
86292	8	12039895	12039918	2	12	24	TG	11	12
86294	8	12040159	12040200	2	21	42	CA	19	13
86299	8	12048369	12048383	3	5	15	AAC	34	3
86309	8	12069065	12069079	3	5	15	ACA	32	3
86313	8	12079555	12079572	2	9	18	CA	22	3
86316	8	12084517	12084530	2	7	14	GT	23	10
86319	8	12086954	12086981	2	14	28	CA	8	13
86323	8	12098890	12098939	2	25	50	TG	16	21
86342	8	12130564	12130583	2	10	20	GT	18	6
86343	8	12134408	12134435	4	7	28	ACTG	24	6
86349	8	12152371	12152391	3	7	21	GAA	17	3
86359	8	12164147	12164168	2	11	22	TG	7	7
86367	8	12179802	12179827	2	13	26	AC	13	20
86373	8	12193443	12193456	2	7	14	CT	22	3
86385	8	12243185	12243199	3	5	15	AAT	25	12
86386	8	12243991	12244006	4	4	16	TCTT	15	7
86401	8	12302313	12302336	2	12	24	CA	15	7
86404	8	12306805	12306828	2	12	24	AG	11	7
86405	8	12307023	12307037	3	5	15	AAC	22	4
86406	8	12308093	12308122	2	15	30	AG	9	7
86408	8	12312351	12312368	2	9	18	GT	15	5
86410	8	12327327	12327359	3	11	33	TCT	16	6
86412	8	12328238	12328267	3	10	30	AGC	21	7

86415	8	12333155	12333170	2	8	16	TG	20	8
86416	8	12346543	12346556	2	7	14	CA	28	3
86417	8	12348843	12348856	2	7	14	AG	27	5
86426	8	12361263	12361282	2	10	20	TG	22	9
86428	8	12362517	12362532	2	8	16	CA	11	5
86432	8	12368461	12368472	2	6	12	TC	20	3
86434	8	12388028	12388060	3	11	33	ATC	25	5
86438	8	12394196	12394211	4	4	16	GACA	27	6
86439	8	12397160	12397187	2	14	28	AC	12	10
86445	8	12404435	12404452	3	6	18	ACA	27	5
86446	8	12407661	12407674	2	7	14	CT	27	3
86448	8	12409174	12409189	2	8	16	AC	10	9
86450	8	12409923	12409952	3	10	30	ATT	8	3
86454	8	12413893	12413912	2	10	20	CT	23	5
86459	8	12414971	12414984	2	7	14	CA	22	9
86470	8	12431028	12431049	2	11	22	AC	25	7
86472	8	12440830	12440847	3	6	18	CAT	28	3
86475	8	12443481	12443498	3	6	18	CAG	23	3
86476	8	12443943	12443960	2	9	18	AG	20	5
86477	8	12445188	12445205	2	9	18	GT	19	6
86478	8	12452578	12452598	3	7	21	GGA	14	3
86481	8	12456074	12456087	2	7	14	AT	21	10
86488	8	12463824	12463841	3	6	18	TCA	25	3
86492	8	12473271	12473282	2	6	12	TG	22	5
86508	8	12496148	12496197	2	25	50	GT	7	11
86509	8	12496346	12496369	4	6	24	AAAT	17	4
86512	8	12498720	12498737	2	9	18	GT	13	5
86519	8	12510372	12510413	2	21	42	GA	8	7
86521	8	12512360	12512375	4	4	16	TTTA	8	3
86523	8	12512897	12512920	2	12	24	GT	23	14
86533	8	12552249	12552264	2	8	16	TG	15	9
86534	8	12552266	12552283	2	9	18	GT	19	12
86537	8	12564656	12564667	2	6	12	GT	33	3
86539	8	12565970	12566035	3	22	66	ATC	23	11
86540	8	12575470	12575489	4	5	20	AAAT	12	6
86542	8	12583139	12583154	2	8	16	TG	25	4
86559	8	12630128	12630155	2	14	28	TG	12	6
86560	8	12633750	12633777	2	14	28	AC	12	7
86565	8	12645751	12645766	4	4	16	TGCG	31	3
86566	8	12646039	12646064	2	13	26	TG	16	8
86572	8	12654476	12654490	3	5	15	TTG	23	3
86574	8	12658382	12658401	2	10	20	AC	16	7
86576	8	12660990	12661005	4	4	16	AAAG	23	4
86585	8	12690326	12690339	2	7	14	CA	25	5
86590	8	12714260	12714275	4	4	16	ATCC	30	3
86603	8	12752032	12752058	3	9	27	CTC	15	7
86604	8	12752647	12752660	2	7	14	TA	19	5
86605	8	12753681	12753704	2	12	24	GT	14	6
86609	8	12764288	12764313	2	13	26	CA	14	5
86613	8	12767755	12767781	3	9	27	AGG	17	3
86614	8	12767904	12767927	3	8	24	GAA	24	5

86618	8	12774079	12774108	2	15	30	GA	12	6
86623	8	12778015	12778040	2	13	26	CA	13	8
86632	8	12788739	12788754	2	8	16	TC	29	4
86639	8	12806960	12806987	2	14	28	TG	16	6
86640	8	12807187	12807200	2	7	14	TG	32	3
86644	8	12814393	12814422	2	15	30	CA	23	8
86645	8	12814735	12814746	2	6	12	AG	19	3
86646	8	12814806	12814821	4	4	16	CACT	15	12
86647	8	12814822	12814835	2	7	14	CA	16	11
86651	8	12815534	12815553	2	10	20	GT	11	8
86653	8	12815726	12815757	2	16	32	CA	14	10
86656	8	12816687	12816702	2	8	16	CA	21	3
86659	8	12818903	12818936	2	17	34	GT	14	11
86661	8	12820481	12820502	2	11	22	GT	13	7
86666	8	12833686	12833700	3	5	15	AAT	19	3
86669	8	12840486	12840503	3	6	18	AAC	29	4
86679	8	12877875	12877902	2	14	28	CA	12	13
86680	8	12880821	12880834	2	7	14	CA	23	6
86682	8	12884911	12884922	2	6	12	AG	31	3
86684	8	12888399	12888422	2	12	24	TC	12	5
86686	8	12889526	12889546	3	7	21	GCT	21	7
86687	8	12889682	12889699	2	9	18	CA	22	9
86695	8	12911056	12911067	2	6	12	TG	19	4
86698	8	12919206	12919217	2	6	12	AC	31	3
86714	8	12963355	12963368	2	7	14	AC	8	3
86716	8	12964335	12964376	2	21	42	CA	6	5
86717	8	12967659	12967674	2	8	16	AC	26	3
86721	8	12971902	12971921	2	10	20	CA	12	8
86724	8	12980711	12980724	2	7	14	AG	28	5
86725	8	12981072	12981095	2	12	24	GA	14	7
86730	8	12992254	12992271	3	6	18	TTA	8	9
86741	8	13008127	13008140	2	7	14	GT	20	4
86742	8	13009490	13009501	2	6	12	TG	26	3
86743	8	13012291	13012306	2	8	16	TC	28	4
86749	8	13027706	13027717	2	6	12	TG	7	3
86750	8	13028003	13028020	2	9	18	AC	18	4
86751	8	13030875	13030888	2	7	14	TG	6	6
86762	8	13049121	13049132	2	6	12	GT	26	3
86766	8	13054634	13054647	2	7	14	GT	31	3
86769	8	13059090	13059122	3	11	33	TAC	14	8
86774	8	13072235	13072258	4	6	24	GCAC	20	7
86775	8	13075218	13075249	2	16	32	AC	13	9
86777	8	13084572	13084593	2	11	22	AG	11	3
86780	8	13097609	13097620	2	6	12	TG	13	3
86785	8	13114978	13115001	4	6	24	TAGA	26	4
86792	8	13125567	13125581	3	5	15	ATT	25	3
86794	8	13130225	13130248	2	12	24	AG	12	3
86823	8	13188615	13188632	2	9	18	TA	7	3
86824	8	13189347	13189366	2	10	20	TG	24	4
86826	8	13190013	13190032	4	5	20	TATT	23	3
86833	8	13208370	13208389	2	10	20	AT	7	5

86840	8	13211717	13211736	2	10	20	GT	17	10
86841	8	13211819	13211848	3	10	30	TCC	9	3
86842	8	13212672	13212691	2	10	20	GT	20	9
86843	8	13213687	13213710	2	12	24	CA	19	8
86864	8	13220103	13220114	2	6	12	AC	28	5
86865	8	13220173	13220200	2	14	28	GT	20	5
86866	8	13220299	13220326	2	14	28	CT	15	9
86868	8	13220572	13220586	3	5	15	ATT	18	3
86870	8	13220768	13220787	2	10	20	CA	11	11
86871	8	13221761	13221798	2	19	38	AC	9	7
86874	8	13222495	13222506	2	6	12	TG	18	7
86875	8	13222508	13222519	2	6	12	GT	20	7
86876	8	13222525	13222536	2	6	12	GT	11	3
86877	8	13222786	13222813	4	7	28	CATC	24	5
86878	8	13222886	13222903	2	9	18	GT	14	8
86885	8	13224488	13224511	3	8	24	CCT	27	3
86886	8	13224709	13224780	3	24	72	GAA	9	10
86893	8	13226575	13226606	2	16	32	GT	15	13
86902	8	13231593	13231607	3	5	15	GGT	15	3
86904	8	13234170	13234205	2	18	36	AC	9	9
86919	8	13245557	13245574	3	6	18	CTC	17	5
86920	8	13246467	13246502	3	12	36	AAC	17	5
86928	8	13265250	13265267	2	9	18	TC	23	8
86932	8	13269818	13269839	2	11	22	TG	16	9
86934	8	13270652	13270681	2	15	30	TG	10	7
86944	8	13283064	13283075	2	6	12	CT	30	3
86948	8	13292365	13292379	3	5	15	GCA	28	5
86951	8	13295842	13295869	2	14	28	AC	11	10
86953	8	13297014	13297043	2	15	30	AG	13	7
86975	8	13342856	13342869	2	7	14	AC	6	3
86986	8	13360354	13360367	2	7	14	GT	12	3
86988	8	13360496	13360527	2	16	32	AC	7	7
86992	8	13370764	13370797	2	17	34	CA	12	8
86996	8	13375767	13375794	2	14	28	TG	21	11
86998	8	13381177	13381200	2	12	24	TG	20	5
86999	8	13381328	13381345	2	9	18	GT	14	3
87008	8	13406426	13406440	3	5	15	GCG	24	3
87013	8	13416647	13416667	3	7	21	AGC	30	6
87032	8	13451433	13451444	2	6	12	AG	26	3
87038	8	13460785	13460806	2	11	22	CA	23	10
87039	8	13460927	13460940	2	7	14	GT	23	6
87044	8	13474377	13474409	3	11	33	TTG	21	6
87062	8	13500845	13500856	2	6	12	TC	31	3
87063	8	13502877	13502888	2	6	12	TC	32	3
87070	8	13523316	13523339	2	12	24	TC	21	5
87073	8	13526521	13526556	2	18	36	CA	12	8
87076	8	13529757	13529784	2	14	28	GT	9	7
87106	8	13558008	13558028	3	7	21	CTG	24	4
87111	8	13563880	13563891	2	6	12	AT	24	5
87117	8	13565893	13565916	2	12	24	CT	11	9
87132	8	13594554	13594591	2	19	38	CA	10	7

87135	8	13596611	13596642	2	16	32	GT	16	7
87136	8	13600603	13600617	3	5	15	CTG	29	3
87147	8	13618129	13618144	2	8	16	GT	21	6
87148	8	13625221	13625253	3	11	33	AGG	8	5
87157	8	13641723	13641736	2	7	14	AC	22	3
87169	8	13666460	13666477	2	9	18	CA	24	3
87178	8	13671732	13671773	2	21	42	AC	8	8
87179	8	13678187	13678213	3	9	27	CCT	14	6
87181	8	13678432	13678453	2	11	22	AC	12	6
87186	8	13685214	13685245	2	16	32	AC	13	6
87192	8	13688432	13688463	2	16	32	CA	21	10
87201	8	13703039	13703058	2	10	20	CA	27	5
87202	8	13703153	13703166	2	7	14	GT	18	4
87203	8	13703168	13703179	2	6	12	TG	21	4
87204	8	13703333	13703356	2	12	24	CA	11	7
87209	8	13705728	13705739	2	6	12	AC	31	3
87218	8	13713435	13713452	2	9	18	CA	17	9
87223	8	13716576	13716608	3	11	33	CCT	13	4
87224	8	13719455	13719474	2	10	20	AC	19	5
87225	8	13720204	13720231	2	14	28	CT	22	8
87227	8	13721100	13721113	2	7	14	CA	34	3
87233	8	13725716	13725730	3	5	15	ATT	15	7
87236	8	13729511	13729544	2	17	34	AG	13	7
87238	8	13731495	13731506	2	6	12	CA	20	4
87241	8	13743761	13743774	2	7	14	TC	14	5
87242	8	13743777	13743790	2	7	14	TC	12	4
87243	8	13745899	13745918	2	10	20	GT	18	9
87245	8	13748032	13748043	2	6	12	AC	21	3
87247	8	13751133	13751164	2	16	32	CA	10	8
87249	8	13754687	13754716	2	15	30	AC	19	8
87250	8	13761907	13761928	2	11	22	AC	19	6
87255	8	13770642	13770677	2	18	36	TG	13	9
87256	8	13773281	13773316	4	9	36	AATG	32	7
87261	8	13779874	13779901	2	14	28	TG	11	11
87263	8	13784719	13784734	2	8	16	CA	7	6
87264	8	13786291	13786312	2	11	22	TG	25	7
87271	8	13803266	13803279	2	7	14	TG	21	5
87275	8	13804838	13804857	2	10	20	AC	25	7
87276	8	13807320	13807343	2	12	24	GT	22	15
87282	8	13823211	13823236	2	13	26	CA	15	5
87284	8	13827717	13827734	2	9	18	CA	26	5
87285	8	13828182	13828197	4	4	16	TTTA	20	5
87286	8	13828459	13828472	2	7	14	AT	16	12
87288	8	13828770	13828787	2	9	18	TG	21	5
87289	8	13829848	13829867	2	10	20	CA	20	5
87291	8	13837122	13837147	2	13	26	CA	19	8
87298	8	13847250	13847265	2	8	16	CA	23	7
87301	8	13855336	13855365	2	15	30	TC	12	10
87303	8	13865322	13865349	4	7	28	TTTG	27	3
87304	8	13866683	13866718	2	18	36	AG	20	9
87305	8	13868351	13868368	2	9	18	AC	31	7

87306	8	13868473	13868506	2	17	34	AC	9	9
87309	8	13870369	13870384	2	8	16	GT	23	5
87311	8	13876342	13876368	3	9	27	ACA	21	7
87314	8	13881141	13881158	2	9	18	AC	16	7
87323	8	13905351	13905362	2	6	12	AG	12	5
87324	8	13905365	13905378	2	7	14	AG	14	4
87327	8	13908178	13908193	2	8	16	AC	21	9
87329	8	13908998	13909011	2	7	14	CA	19	6
87330	8	13910313	13910338	2	13	26	GT	15	11
87332	8	13917267	13917284	3	6	18	CCT	26	4
87334	8	13922150	13922165	4	4	16	GACA	26	3
87335	8	13922398	13922413	2	8	16	CA	21	5
87336	8	13922519	13922530	2	6	12	GA	25	3
87337	8	13922894	13922911	2	9	18	TG	19	8
87340	8	13926279	13926294	2	8	16	TG	8	3
87347	8	13933784	13933799	2	8	16	GT	19	10
87348	8	13933987	13933998	2	6	12	CT	16	3
87349	8	13934000	13934015	2	8	16	TC	17	4
87350	8	13934235	13934248	2	7	14	AC	22	4
87351	8	13934375	13934396	2	11	22	CA	19	12
87353	8	13938545	13938574	2	15	30	CA	13	8
87354	8	13939303	13939316	2	7	14	GA	10	3
87367	8	13946660	13946683	2	12	24	TG	21	7
87374	8	13960407	13960440	2	17	34	TG	7	8
87375	8	13960607	13960627	3	7	21	TCA	22	8
87393	8	13984686	13984701	4	4	16	AAAC	18	7
87396	8	13988934	13988945	2	6	12	GT	31	4
87397	8	13990614	13990631	2	9	18	GT	21	4
87408	8	14025364	14025385	2	11	22	TG	9	7
87415	8	14040383	14040398	2	8	16	TG	22	3
87423	8	14051078	14051092	3	5	15	AGG	9	3
87427	8	14056998	14057027	2	15	30	TC	7	5
87428	8	14057702	14057721	2	10	20	CA	22	6
87429	8	14057827	14057846	2	10	20	GT	15	10
87432	8	14060315	14060334	2	10	20	GT	21	13
87433	8	14061817	14061838	2	11	22	TG	21	4
87436	8	14063587	14063610	2	12	24	GT	18	13
87440	8	14066115	14066132	3	6	18	GGA	22	4
87442	8	14068740	14068771	2	16	32	GT	21	10
87444	8	14073633	14073644	2	6	12	CA	23	3
87446	8	14076988	14076999	2	6	12	AC	23	4
87447	8	14077161	14077172	2	6	12	CA	9	4
87448	8	14077477	14077494	3	6	18	AAC	32	5
87451	8	14078005	14078022	2	9	18	CA	18	4
87452	8	14079482	14079515	2	17	34	TG	17	9
87453	8	14079741	14079762	2	11	22	CA	21	7
87454	8	14080050	14080071	2	11	22	AC	21	11
87456	8	14081880	14081897	2	9	18	TG	20	5
87458	8	14083940	14083967	2	14	28	CA	18	9
87472	8	14110150	14110171	2	11	22	GT	10	14
87473	8	14112864	14112875	2	6	12	AC	34	3

87474	8	14113226	14113255	2	15	30	AC	16	7
87479	8	14125053	14125066	2	7	14	GT	24	3
87481	8	14137125	14137138	2	7	14	TC	31	3
87482	8	14139101	14139118	2	9	18	TC	18	3
87483	8	14140876	14140901	2	13	26	GT	12	5
87490	8	14157483	14157510	2	14	28	CA	15	11
87494	8	14160059	14160082	2	12	24	CT	8	8
87495	8	14161079	14161118	2	20	40	AC	8	10
87496	8	14161158	14161181	2	12	24	AC	12	14
87498	8	14161773	14161787	3	5	15	AGG	14	6
87500	8	14185256	14185267	2	6	12	TG	27	3
87501	8	14189882	14189897	2	8	16	TG	16	3
87503	8	14190217	14190250	2	17	34	GT	17	10
87505	8	14199017	14199032	4	4	16	AAAT	26	9
87506	8	14203053	14203070	2	9	18	TG	23	6
87507	8	14205796	14205813	2	9	18	GT	24	5
87511	8	14210637	14210660	2	12	24	CA	13	5
87512	8	14211365	14211400	3	12	36	TCA	16	5
87513	8	14212707	14212742	2	18	36	AC	14	13
87515	8	14213847	14213864	2	9	18	TG	21	6
87517	8	14215942	14215953	2	6	12	CA	26	3
87518	8	14216774	14216791	2	9	18	GT	24	5
87519	8	14216912	14216929	2	9	18	GT	16	6
87520	8	14218633	14218658	2	13	26	AC	25	13
87521	8	14218847	14218860	2	7	14	AC	19	7
87522	8	14218861	14218874	2	7	14	CA	13	3
87523	8	14218932	14218947	2	8	16	GA	29	3
87526	8	14219255	14219268	2	7	14	CA	18	4
87528	8	14220264	14220277	2	7	14	AC	16	3
87529	8	14220492	14220503	2	6	12	CA	16	4
87532	8	14226302	14226313	2	6	12	AT	21	3
87533	8	14226790	14226809	4	5	20	GTCC	30	8
87534	8	14229937	14229954	2	9	18	TG	13	9
87535	8	14230082	14230111	2	15	30	AC	17	7
87538	8	14231370	14231403	2	17	34	GT	16	14
87542	8	14233068	14233106	3	13	39	CAG	23	14
87544	8	14233660	14233677	2	9	18	GA	13	3
87548	8	14237590	14237607	3	6	18	GTG	17	6
87549	8	14238942	14238971	2	15	30	TG	14	16
87550	8	14239134	14239153	2	10	20	TG	19	11
87554	8	14249432	14249449	2	9	18	GT	16	6
87562	8	14255062	14255073	2	6	12	GT	23	4
87567	8	14256826	14256840	3	5	15	CAG	19	4
87574	8	14263923	14263938	2	8	16	TG	33	3
87576	8	14267627	14267646	2	10	20	GT	12	8
87583	8	14272387	14272402	2	8	16	GT	15	9
87584	8	14272806	14272825	2	10	20	TG	20	12
87585	8	14273016	14273035	2	10	20	GT	20	8
87588	8	14277708	14277727	2	10	20	AC	17	8
87589	8	14278425	14278438	2	7	14	GA	31	3
87590	8	14278595	14278620	2	13	26	AC	17	11

87592	8	14278990	14279007	3	6	18	AGG	20	3
87594	8	14279420	14279437	2	9	18	CA	24	3
87600	8	14285539	14285558	2	10	20	GT	13	6
87601	8	14285833	14285856	2	12	24	GT	18	6
87603	8	14288305	14288326	2	11	22	TG	15	12
87604	8	14288786	14288801	2	8	16	GT	25	8
87607	8	14294004	14294017	2	7	14	GT	14	10
87608	8	14294090	14294115	2	13	26	GT	14	9
87612	8	14298479	14298496	2	9	18	CA	11	3
87613	8	14298523	14298548	2	13	26	CA	8	4
87615	8	14298927	14298942	2	8	16	CA	20	5
87616	8	14299124	14299147	2	12	24	AC	18	5
87619	8	14300032	14300065	2	17	34	GT	12	10
87621	8	14300140	14300154	3	5	15	CTC	18	4
87622	8	14300282	14300301	4	5	20	ATTT	23	6
87624	8	14300979	14301000	2	11	22	GA	12	11
87626	8	14301639	14301682	2	22	44	AC	11	13
87627	8	14301707	14301730	2	12	24	TC	11	5
87628	8	14301790	14301825	2	18	36	CA	12	10
87631	8	14302423	14302456	2	17	34	AC	14	12
87632	8	14304722	14304751	2	15	30	GT	6	6
87633	8	14305825	14305840	4	4	16	CACT	34	15
87635	8	14309019	14309036	2	9	18	CA	9	3
87638	8	14310681	14310702	2	11	22	TG	17	4
87640	8	14312433	14312458	2	13	26	GT	19	7
87641	8	14314114	14314139	2	13	26	TG	19	8
87643	8	14315335	14315364	2	15	30	CA	9	12
87644	8	14316971	14316994	2	12	24	GT	26	4
87645	8	14317577	14317588	2	6	12	TG	26	5
87646	8	14317902	14317941	4	10	40	ATAG	13	8
87650	8	14318951	14318972	2	11	22	GA	16	6
87652	8	14321098	14321109	2	6	12	AC	31	3
87653	8	14321239	14321250	2	6	12	AC	30	3
87654	8	14321287	14321312	2	13	26	AC	13	13
87656	8	14323996	14324017	2	11	22	GT	14	16
87657	8	14324330	14324357	2	14	28	AC	26	9
87661	8	14326255	14326266	2	6	12	GA	22	5
87665	8	14328676	14328687	2	6	12	TG	18	3
87666	8	14329315	14329328	2	7	14	GT	26	3
87667	8	14330184	14330199	2	8	16	AC	6	6
87668	8	14330940	14330963	2	12	24	CA	12	9
87669	8	14331157	14331172	2	8	16	CA	11	9
87673	8	14333119	14333142	2	12	24	TG	14	4
87675	8	14336864	14336883	2	10	20	GT	23	5
87684	8	14348189	14348222	2	17	34	GT	7	6
87685	8	14349987	14350002	2	8	16	AC	20	8
87686	8	14351551	14351570	2	10	20	GA	17	8
87689	8	14367937	14367952	2	8	16	CA	18	4
87690	8	14368218	14368231	2	7	14	AG	21	3
87695	8	14378875	14378890	2	8	16	GA	20	6
87696	8	14378892	14378905	2	7	14	AG	12	3

87698	8	14379350	14379395	2	23	46	TG	7	7
87700	8	14383503	14383530	2	14	28	TG	17	10
87703	8	14385184	14385205	2	11	22	GT	30	5
87704	8	14385916	14385931	2	8	16	TC	27	3
87705	8	14386147	14386172	2	13	26	TG	12	15
87706	8	14386831	14386866	2	18	36	CA	9	10
87707	8	14386995	14387008	2	7	14	AC	19	10
87708	8	14387012	14387025	2	7	14	AC	17	9
87727	8	14407579	14407614	2	18	36	CA	10	7
87729	8	14408043	14408062	2	10	20	TG	15	5
87732	8	14409462	14409495	2	17	34	TG	15	14
87733	8	14409962	14409977	2	8	16	TG	19	3
87734	8	14410230	14410241	2	6	12	CA	27	9
87735	8	14411409	14411424	2	8	16	TG	28	5
87737	8	14413794	14413817	2	12	24	TG	20	5
87738	8	14415692	14415705	2	7	14	TG	20	3
87741	8	14422387	14422398	2	6	12	AC	17	3
87743	8	14422644	14422673	2	15	30	AC	11	7
87744	8	14424771	14424800	2	15	30	TG	17	11
87747	8	14426921	14426940	2	10	20	CA	17	7
87748	8	14426942	14426955	2	7	14	AC	20	8
87750	8	14431068	14431093	2	13	26	CA	6	6
87752	8	14431253	14431270	2	9	18	AC	20	10
87754	8	14433071	14433082	2	6	12	CA	23	4
87761	8	14447848	14447875	2	14	28	AC	13	5
87762	8	14448665	14448688	2	12	24	CA	20	6
87763	8	14449134	14449157	2	12	24	AC	22	9
87765	8	14451348	14451381	2	17	34	AC	9	7
87766	8	14451509	14451534	2	13	26	TG	14	13
87769	8	14453532	14453547	2	8	16	GA	20	5
87773	8	14468217	14468240	2	12	24	GT	12	11
87775	8	14468590	14468616	3	9	27	GCT	22	5
87776	8	14470373	14470402	3	10	30	CAG	23	5
87780	8	14472675	14472695	3	7	21	TGC	21	3
87782	8	14472720	14472734	3	5	15	TGA	19	3
87789	8	14486902	14486937	2	18	36	CA	12	11
87802	8	14501829	14501848	4	5	20	TTAT	32	5
87806	8	14504599	14504626	2	14	28	GT	8	12
87810	8	14509944	14509979	2	18	36	TG	7	9
87811	8	14510137	14510178	2	21	42	GT	12	13
87813	8	14511575	14511602	2	14	28	TG	19	9
87814	8	14514699	14514730	2	16	32	AC	9	12
87815	8	14516344	14516363	2	10	20	CA	29	6
87816	8	14517075	14517090	2	8	16	TG	29	5
87819	8	14530749	14530766	2	9	18	GT	32	5
87828	8	14543481	14543514	2	17	34	AC	10	12
87836	8	14582443	14582462	4	5	20	ATTT	14	5
87837	8	14597112	14597135	2	12	24	AC	19	13
87840	8	14601630	14601689	4	15	60	TAGA	18	11
87847	8	14612531	14612542	2	6	12	CA	12	7
87848	8	14612587	14612630	2	22	44	CA	6	8

87849	8	14613139	14613156	2	9	18	TG	26	6
87850	8	14613534	14613563	2	15	30	AG	9	6
87854	8	14617884	14617901	2	9	18	TG	24	4
87855	8	14618538	14618549	2	6	12	AC	22	5
87862	8	14628508	14628519	2	6	12	TC	31	4
87863	8	14628591	14628620	2	15	30	GT	10	9
87871	8	14637416	14637449	2	17	34	TG	11	9
87876	8	14641837	14641857	3	7	21	AAC	23	4
87878	8	14648291	14648316	2	13	26	TG	11	9
87879	8	14650117	14650134	2	9	18	GT	20	5
87880	8	14650761	14650778	2	9	18	CA	25	7
87891	8	14659928	14659948	3	7	21	CAG	17	5
87901	8	14671593	14671616	3	8	24	TTC	22	4
87902	8	14676930	14676944	3	5	15	GCT	26	5
87903	8	14677369	14677382	2	7	14	TG	23	4
87907	8	14687125	14687150	2	13	26	AC	13	7
87908	8	14690308	14690319	2	6	12	AC	21	5
87912	8	14699099	14699112	2	7	14	GA	21	5
87913	8	14699488	14699501	2	7	14	TG	27	8
87915	8	14700637	14700664	2	14	28	TG	6	7
87917	8	14702615	14702646	2	16	32	GT	15	14
87918	8	14704237	14704268	2	16	32	AC	21	8
87919	8	14705019	14705042	2	12	24	CA	14	5
87921	8	14706488	14706511	2	12	24	TG	14	8
87925	8	14708285	14708300	2	8	16	AC	25	4
87926	8	14708884	14708899	2	8	16	CT	24	4
87928	8	14714024	14714037	2	7	14	AC	24	3
87931	8	14717834	14717855	2	11	22	TG	21	5
87932	8	14720971	14720984	2	7	14	TG	26	4
87933	8	14721736	14721759	2	12	24	CA	20	16
87935	8	14722186	14722199	2	7	14	AC	10	3
87936	8	14722202	14722213	2	6	12	AC	22	5
87937	8	14722401	14722418	2	9	18	CA	31	5
87938	8	14722551	14722576	2	13	26	TG	14	14
87941	8	14723542	14723565	2	12	24	GT	13	6
87944	8	14730435	14730470	2	18	36	GT	9	11
87945	8	14730533	14730550	2	9	18	TG	17	8
87949	8	14734992	14735031	2	20	40	AC	13	9
87951	8	14736845	14736860	4	4	16	AAAT	22	3
87952	8	14738143	14738160	2	9	18	GT	24	6
87955	8	14743522	14743551	2	15	30	AC	12	12
87956	8	14743666	14743693	2	14	28	TG	7	8
87960	8	14744115	14744129	3	5	15	CAT	17	3
87961	8	14744638	14744659	2	11	22	GT	11	5
87963	8	14745004	14745033	2	15	30	TG	14	7
87964	8	14745158	14745173	2	8	16	TG	17	3
87965	8	14745269	14745282	2	7	14	AC	16	5
87967	8	14745418	14745437	2	10	20	CA	15	5
87970	8	14748222	14748249	2	14	28	CA	17	16
87972	8	14748782	14748807	2	13	26	TG	13	7
87973	8	14749078	14749093	2	8	16	CA	22	3

87976	8	14758086	14758101	2	8	16	AC	19	4
87982	8	14766521	14766540	2	10	20	GA	14	6
87983	8	14766862	14766885	2	12	24	AG	9	3
87984	8	14766947	14766984	2	19	38	GA	15	15
87985	8	14767799	14767834	2	18	36	TG	6	8
87987	8	14768341	14768382	3	14	42	CTC	11	10
87991	8	14772206	14772237	2	16	32	TG	11	10
87992	8	14772902	14772915	2	7	14	AG	28	4
87994	8	14776504	14776519	2	8	16	TG	20	7
88002	8	14786095	14786120	2	13	26	GT	9	6
88009	8	14796807	14796838	2	16	32	AC	18	8
88011	8	14801196	14801209	2	7	14	CA	18	6
88012	8	14801279	14801308	2	15	30	AC	14	8
88013	8	14801442	14801485	2	22	44	GT	13	16
88014	8	14804712	14804739	2	14	28	CA	12	7
88022	8	14812514	14812549	2	18	36	GT	10	15
88023	8	14813724	14813739	2	8	16	AC	29	3
88026	8	14816543	14816558	2	8	16	TG	22	4
88027	8	14816923	14816958	2	18	36	CT	12	8
88031	8	14823131	14823144	2	7	14	TC	22	3
88038	8	14827754	14827775	2	11	22	TG	21	7
88041	8	14829577	14829616	2	20	40	GT	8	9
88050	8	14844187	14844206	2	10	20	AC	18	5
88051	8	14844733	14844746	2	7	14	CA	20	3
88054	8	14847506	14847521	2	8	16	CA	17	9
88063	8	14860886	14860905	2	10	20	CT	7	3
88064	8	14863040	14863061	2	11	22	GT	20	15
88065	8	14866391	14866420	2	15	30	GT	11	9
88066	8	14866566	14866579	2	7	14	AC	22	4
88068	8	14867309	14867324	4	4	16	AAAT	27	4
88069	8	14869708	14869723	2	8	16	GT	26	4
88070	8	14870240	14870261	2	11	22	CA	21	6
88071	8	14870415	14870430	2	8	16	TG	15	5
88074	8	14879418	14879447	2	15	30	CA	14	9
88075	8	14879557	14879598	2	21	42	TG	12	9
88079	8	14883490	14883505	4	4	16	GTAC	29	5
88080	8	14884700	14884713	2	7	14	AC	27	3
88092	8	14887737	14887762	2	13	26	TG	8	9
88096	8	14888557	14888586	2	15	30	AC	7	5
88107	8	14901778	14901805	2	14	28	AC	8	5
88110	8	14907727	14907744	3	6	18	TCT	16	6
88121	8	14935536	14935561	2	13	26	TG	18	7
88122	8	14935747	14935762	2	8	16	CA	17	3
88123	8	14937756	14937769	2	7	14	GT	27	5
88131	8	14958515	14958529	3	5	15	ACA	8	3
88134	9	7607	7636	2	15	30	GT	9	5
88136	9	10589	10600	2	6	12	GT	27	5
88137	9	10869	10888	2	10	20	TG	22	6
88139	9	11649	11664	2	8	16	CA	17	9
88144	9	22408	22419	2	6	12	GT	14	4
88145	9	22422	22435	2	7	14	GT	21	6

88147	9	22752	22779	2	14	28	CA	11	12
88149	9	22949	22966	2	9	18	AC	16	12
88153	9	23299	23324	2	13	26	GT	18	15
88156	9	29497	29512	2	8	16	AC	21	11
88157	9	29628	29651	2	12	24	TG	11	7
88158	9	30060	30073	2	7	14	GT	14	9
88159	9	30241	30284	2	22	44	TG	8	7
88160	9	32661	32684	2	12	24	GT	11	8
88166	9	38607	38622	2	8	16	AC	21	3
88167	9	38771	38802	2	16	32	TG	8	8
88169	9	40156	40179	2	12	24	GT	19	12
88174	9	49182	49199	3	6	18	CTT	9	6
88179	9	50825	50850	2	13	26	GT	11	9
88180	9	50923	50937	3	5	15	GCT	20	7
88182	9	52654	52665	2	6	12	TA	6	3
88185	9	55432	55446	3	5	15	TTC	25	4
88186	9	56327	56338	2	6	12	TA	16	3
88187	9	56339	56352	2	7	14	CA	18	8
88192	9	60763	60798	2	18	36	TC	9	10
88194	9	61044	61070	3	9	27	CTC	17	7
88205	9	70666	70689	4	6	24	AAGG	7	5
88208	9	71004	71023	2	10	20	CA	10	8
88211	9	76608	76623	4	4	16	TGGA	11	12
88213	9	77419	77433	3	5	15	CTG	27	3
88218	9	83568	83585	2	9	18	GA	17	4
88220	9	85392	85419	2	14	28	GT	15	16
88221	9	88220	88237	2	9	18	GT	22	12
88222	9	88336	88347	2	6	12	GT	17	4
88223	9	88378	88425	2	24	48	GT	8	11
88224	9	88551	88586	2	18	36	TG	8	6
88225	9	89224	89255	2	16	32	GT	13	8
88226	9	89428	89479	2	26	52	CA	15	12
88230	9	91227	91253	3	9	27	TTC	20	9
88239	9	94020	94045	2	13	26	AC	11	9
88240	9	94978	95005	2	14	28	GT	10	9
88241	9	95145	95168	4	6	24	ATTT	12	6
88242	9	98348	98383	2	18	36	GT	10	13
88243	9	98490	98541	2	26	52	TG	9	10
88244	9	101044	101061	3	6	18	GGA	15	5
88248	9	101780	101793	2	7	14	GA	13	4
88257	9	105717	105770	2	27	54	TG	8	9
88260	9	107042	107063	2	11	22	CT	25	4
88262	9	108960	108989	2	15	30	GT	11	12
88264	9	110046	110063	2	9	18	AC	18	10
88269	9	112964	112985	2	11	22	AC	7	4
88276	9	115372	115413	2	21	42	TG	10	12
88279	9	118500	118523	3	8	24	TTG	23	4
88280	9	119130	119143	2	7	14	CT	27	3
88284	9	120349	120382	2	17	34	AC	8	8
88286	9	121727	121760	2	17	34	GT	11	12
88288	9	121942	121961	2	10	20	TG	12	4

88290	9	129114	129127	2	7	14	AG	19	3
88293	9	135952	135969	3	6	18	TTA	20	3
88299	9	141325	141346	2	11	22	AC	16	10
88302	9	144336	144361	2	13	26	GT	17	7
88314	9	157611	157640	2	15	30	AC	8	8
88315	9	157743	157776	2	17	34	AC	8	10
88326	9	176253	176264	2	6	12	TC	31	5
88340	9	192958	192971	2	7	14	GA	7	3
88341	9	192972	192985	2	7	14	GT	10	3
88343	9	201282	201295	2	7	14	CA	29	6
88345	9	212567	212578	2	6	12	CA	26	3
88346	9	215194	215217	2	12	24	TG	7	7
88347	9	223055	223069	3	5	15	ACA	16	3
88349	9	233565	233579	3	5	15	TCT	35	3
88356	9	251262	251279	2	9	18	CA	21	4
88357	9	252749	252776	2	14	28	CA	13	9
88358	9	253506	253521	2	8	16	AC	27	11
88360	9	261216	261233	3	6	18	TTA	19	6
88362	9	262186	262213	4	7	28	GAAG	21	7
88363	9	262541	262572	2	16	32	AC	11	5
88365	9	263658	263669	2	6	12	GT	25	18
88368	9	266839	266874	2	18	36	CA	9	8
88369	9	266995	267036	2	21	42	AC	9	12
88370	9	269466	269489	2	12	24	CA	22	12
88378	9	284655	284681	3	9	27	GAA	27	10
88383	9	286887	286906	2	10	20	TG	8	9
88385	9	286945	286962	2	9	18	TG	12	3
88391	9	289459	289500	2	21	42	GT	8	9
88392	9	290001	290016	2	8	16	TG	23	10
88393	9	290397	290412	2	8	16	AG	23	5
88397	9	293753	293790	2	19	38	TG	15	11
88398	9	293928	293939	2	6	12	GT	25	3
88400	9	296263	296276	2	7	14	GA	19	5
88402	9	297012	297027	2	8	16	AT	7	3
88403	9	298149	298162	2	7	14	AC	23	4
88404	9	298360	298371	2	6	12	GA	11	4
88405	9	299729	299748	2	10	20	AC	20	6
88406	9	299903	299934	2	16	32	GT	13	10
88408	9	300992	301021	2	15	30	TG	14	13
88409	9	301092	301105	2	7	14	CT	18	3
88414	9	305176	305189	2	7	14	CA	19	3
88417	9	307389	307412	2	12	24	CA	17	13
88418	9	307512	307523	2	6	12	GT	19	6
88419	9	307555	307584	2	15	30	TG	10	7
88420	9	307688	307701	2	7	14	CA	22	10
88421	9	307806	307853	2	24	48	GT	6	8
88422	9	307911	307936	2	13	26	GT	18	8
88423	9	309246	309281	2	18	36	GT	6	6
88424	9	309376	309393	2	9	18	AC	18	10
88426	9	309454	309471	2	9	18	TG	6	5
88427	9	309639	309654	2	8	16	GT	19	10

88435	9	315691	315710	2	10	20	CA	10	3
88437	9	320337	320360	2	12	24	AC	21	5
88452	9	329956	329973	2	9	18	TC	12	3
88454	9	332363	332384	2	11	22	GT	19	8
88455	9	334200	334217	2	9	18	TG	24	7
88458	9	339592	339612	3	7	21	GCT	28	8
88465	9	343467	343488	2	11	22	TG	8	5
88466	9	343663	343694	2	16	32	CA	10	8
88467	9	343883	343898	2	8	16	AC	28	4
88468	9	343943	343964	2	11	22	GT	22	3
88469	9	345612	345627	4	4	16	CTGT	31	4
88472	9	346035	346052	2	9	18	GT	11	8
88478	9	346690	346707	3	6	18	GGT	12	4
88479	9	346798	346815	2	9	18	AC	15	6
88486	9	356309	356350	2	21	42	TG	10	11
88487	9	358690	358713	3	8	24	GAA	26	5
88488	9	361038	361052	3	5	15	GGT	14	4
88496	9	375992	376005	2	7	14	TG	14	6
88497	9	376149	376176	2	14	28	GT	8	8
88498	9	377926	377953	4	7	28	TAGA	13	14
88501	9	383349	383370	2	11	22	TG	10	12
88502	9	383596	383607	2	6	12	GT	14	6
88503	9	388197	388216	2	10	20	AC	12	12
88512	9	391074	391097	3	8	24	AGG	11	10
88517	9	395909	395923	3	5	15	CAG	25	11
88524	9	403887	403906	4	5	20	AAAG	18	4
88528	9	415335	415349	3	5	15	GAT	31	7
88529	9	416896	416917	2	11	22	TG	24	9
88531	9	418540	418561	2	11	22	GT	22	13
88532	9	420479	420506	2	14	28	GT	20	13
88533	9	421699	421718	2	10	20	GT	20	4
88534	9	422720	422749	2	15	30	GT	11	12
88537	9	426014	426041	2	14	28	TG	16	9
88538	9	426553	426584	4	8	32	CTTT	28	7
88541	9	426912	426925	2	7	14	AC	19	18
88544	9	430668	430687	2	10	20	AC	8	9
88545	9	430689	430702	2	7	14	CA	8	9
88546	9	430826	430849	2	12	24	AC	22	8
88547	9	432612	432649	2	19	38	CA	10	10
88548	9	432740	432783	2	22	44	CA	16	15
88549	9	433795	433814	2	10	20	AC	22	11
88550	9	433969	433988	2	10	20	CA	18	13
88551	9	434091	434130	2	20	40	GT	9	8
88552	9	435534	435561	2	14	28	TG	19	10
88553	9	435723	435752	2	15	30	TG	11	8
88555	9	438765	438786	2	11	22	GT	18	12
88556	9	439240	439273	2	17	34	TG	15	9
88560	9	445315	445329	3	5	15	GAA	25	3
88561	9	446154	446179	2	13	26	AC	21	10
88562	9	446254	446273	4	5	20	GTCA	29	3
88565	9	450331	450350	2	10	20	GT	23	4

88566	9	450579	450598	2	10	20	CA	14	8
88568	9	450789	450812	2	12	24	CT	15	7
88569	9	450900	450917	2	9	18	TC	19	6
88570	9	451554	451587	2	17	34	GT	23	10
88571	9	452099	452113	3	5	15	CTG	33	7
88573	9	454058	454081	4	6	24	GGAG	6	4
88575	9	454608	454634	3	9	27	GAG	27	8
88576	9	457660	457687	2	14	28	TG	22	8
88578	9	460225	460240	2	8	16	TG	24	10
88579	9	461425	461438	2	7	14	CT	24	6
88580	9	463295	463322	2	14	28	AC	18	8
88582	9	464417	464430	2	7	14	TC	31	3
88585	9	467408	467427	4	5	20	CTGT	21	5
88586	9	469368	469397	2	15	30	CA	10	9
88587	9	472672	472705	2	17	34	GT	11	10
88588	9	473062	473079	3	6	18	AGA	34	8
88589	9	473294	473305	2	6	12	TG	25	3
88590	9	473536	473561	2	13	26	GT	13	7
88600	9	476536	476567	2	16	32	GT	21	9
88601	9	478088	478111	2	12	24	TG	25	4
88602	9	478498	478517	2	10	20	CA	12	7
88603	9	479433	479466	2	17	34	AC	12	11
88608	9	486669	486724	4	14	56	TTTC	9	7
88609	9	486767	486786	4	5	20	TCTT	13	6
88611	9	488109	488140	2	16	32	GT	10	12
88612	9	488427	488442	2	8	16	TG	22	5
88613	9	489395	489421	3	9	27	TTC	20	8
88615	9	489783	489804	2	11	22	AC	17	8
88620	9	501252	501267	2	8	16	TC	12	3
88623	9	501520	501535	2	8	16	CA	22	11
88624	9	504504	504525	2	11	22	GT	16	5
88625	9	506464	506487	3	8	24	TCC	15	6
88628	9	510949	510982	2	17	34	GT	11	7
88629	9	513138	513167	2	15	30	TG	15	10
88630	9	514737	514748	2	6	12	AT	24	11
88631	9	514789	514804	4	4	16	GATT	24	3
88633	9	517254	517273	2	10	20	AC	16	8
88634	9	517529	517546	2	9	18	AC	16	13
88635	9	519469	519484	2	8	16	AG	19	3
88637	9	519540	519569	2	15	30	GT	11	8
88638	9	519669	519690	2	11	22	GT	19	13
88640	9	521661	521678	3	6	18	GCA	23	6
88645	9	530759	530780	2	11	22	GT	23	8
88647	9	532341	532356	4	4	16	GATA	18	5
88649	9	534886	534937	4	13	52	GATA	21	9
88652	9	545148	545159	2	6	12	AC	21	3
88653	9	545775	545794	2	10	20	GT	23	4
88654	9	545952	545977	2	13	26	CA	14	10
88657	9	547713	547750	2	19	38	GT	11	14
88658	9	551146	551165	2	10	20	GT	9	8
88659	9	552004	552017	2	7	14	CA	31	3

88660	9	552042	552097	2	28	56	TG	15	11
88661	9	557709	557722	2	7	14	GA	21	3
88667	9	569662	569695	2	17	34	GT	24	15
88668	9	570403	570426	2	12	24	CA	12	15
88669	9	575531	575564	2	17	34	GT	14	7
88672	9	582746	582781	2	18	36	TG	15	16
88673	9	582867	582886	4	5	20	GATG	10	3
88674	9	582955	582984	2	15	30	CA	31	9
88675	9	585318	585335	2	9	18	GT	15	4
88676	9	586126	586140	3	5	15	TCT	31	3
88678	9	592393	592418	2	13	26	TG	20	7
88679	9	594636	594647	2	6	12	TG	30	3
88686	9	626162	626175	2	7	14	GT	21	3
88688	9	628257	628283	3	9	27	AGG	20	4
88690	9	637720	637733	2	7	14	CT	19	3
88697	9	643477	643494	3	6	18	CAG	24	11
88701	9	647748	647779	2	16	32	GT	11	9
88713	9	654190	654231	2	21	42	GT	6	6
88714	9	654273	654292	2	10	20	CA	18	8
88724	9	662853	662890	2	19	38	TG	7	10
88725	9	662977	662988	2	6	12	AC	9	4
88729	9	663180	663199	2	10	20	AC	20	9
88730	9	663361	663378	2	9	18	AC	19	6
88733	9	670744	670757	2	7	14	AT	18	6
88750	9	681841	681855	3	5	15	TTA	19	5
88753	9	682674	682697	4	6	24	AATA	15	6
88758	9	694813	694844	2	16	32	AC	18	8
88762	9	695231	695254	2	12	24	CA	21	7
88763	9	695411	695472	2	31	62	CA	8	11
88764	9	698219	698234	2	8	16	GT	19	6
88766	9	698486	698505	2	10	20	TG	23	6
88770	9	704253	704276	3	8	24	GCT	21	7
88771	9	707268	707291	2	12	24	GT	17	5
88772	9	707468	707485	2	9	18	AC	17	14
88774	9	710848	710861	2	7	14	AC	25	4
88776	9	716579	716612	2	17	34	CA	15	8
88777	9	716722	716753	2	16	32	CA	6	7
88778	9	716871	716884	2	7	14	CA	10	13
88779	9	717117	717144	2	14	28	GT	17	6
88782	9	718956	718989	2	17	34	CA	7	9
88785	9	720177	720194	2	9	18	TG	19	10
88793	9	725096	725113	2	9	18	TG	13	8
88797	9	732664	732685	2	11	22	CA	20	11
88799	9	732958	732973	2	8	16	TG	17	13
88800	9	736028	736055	2	14	28	AC	13	10
88801	9	736244	736281	2	19	38	CA	8	10
88806	9	742123	742144	2	11	22	AC	22	7
88808	9	742304	742327	2	12	24	CA	16	16
88809	9	744054	744081	2	14	28	TG	9	8
88810	9	745906	745921	2	8	16	AC	20	12
88815	9	746305	746342	2	19	38	CA	8	12

88817	9	746643	746668	2	13	26	CA	14	9
88818	9	746726	746739	2	7	14	GT	31	3
88819	9	751520	751531	2	6	12	TG	27	4
88821	9	751873	751886	2	7	14	CA	8	5
88822	9	752034	752051	2	9	18	AC	16	6
88833	9	759012	759029	2	9	18	CT	24	4
88835	9	762629	762654	2	13	26	CA	10	7
88836	9	762955	762980	2	13	26	CA	18	9
88838	9	764835	764848	2	7	14	AG	19	3
88839	9	764891	764906	2	8	16	GA	22	3
88844	9	769787	769800	2	7	14	CT	17	4
88861	9	783232	783245	2	7	14	AG	13	5
88862	9	785229	785248	2	10	20	GT	18	13
88864	9	787361	787380	2	10	20	CT	19	11
88865	9	791314	791358	3	15	45	TGA	17	8
88867	9	793114	793129	4	4	16	TCTT	32	12
88868	9	793236	793251	2	8	16	CA	19	13
88869	9	797303	797316	2	7	14	CT	25	3
88871	9	797917	797940	4	6	24	TCCA	19	10
88872	9	798082	798105	2	12	24	CA	17	5
88884	9	815444	815457	2	7	14	TA	22	3
88887	9	823619	823656	2	19	38	AC	12	11
88888	9	824617	824632	2	8	16	CA	21	13
88889	9	824658	824677	4	5	20	CATA	7	5
88890	9	824772	824797	2	13	26	AC	10	8
88893	9	825052	825063	2	6	12	AC	20	4
88895	9	827145	827168	3	8	24	GAA	17	6
88896	9	827225	827254	2	15	30	CA	9	6
88897	9	827316	827331	2	8	16	CA	24	5
88898	9	827384	827397	2	7	14	CA	15	5
88899	9	827399	827418	2	10	20	AC	19	7
88903	9	827661	827687	3	9	27	TCT	21	5
88904	9	827732	827745	2	7	14	GA	11	3
88906	9	827974	828009	2	18	36	TC	12	7
88908	9	833002	833019	2	9	18	TA	16	5
88912	9	834266	834293	2	14	28	AC	12	14
88913	9	834401	834412	2	6	12	AC	20	3
88916	9	835199	835220	2	11	22	TG	26	11
88917	9	835439	835484	2	23	46	AC	11	12
88919	9	838251	838270	2	10	20	AC	20	8
88920	9	838272	838287	2	8	16	CA	18	6
88924	9	838892	838919	2	14	28	GT	7	8
88936	9	858859	858872	2	7	14	CA	16	5
88939	9	862529	862560	2	16	32	AC	12	12
88940	9	862764	862783	2	10	20	AC	16	10
88942	9	863510	863529	2	10	20	TC	6	3
88946	9	864268	864281	2	7	14	CA	10	3
88947	9	864342	864377	2	18	36	CA	9	10
88949	9	868814	868827	2	7	14	CA	22	7
88951	9	871730	871757	2	14	28	AC	15	9
88953	9	873713	873736	2	12	24	CA	17	12

88954	9	873857	873868	2	6	12	GT	11	8
88956	9	875321	875346	2	13	26	TG	20	5
88957	9	876431	876464	2	17	34	TG	12	10
88958	9	876564	876593	2	15	30	GT	10	10
88961	9	887981	888002	2	11	22	AC	26	8
88964	9	889612	889645	2	17	34	GT	6	7
88968	9	893721	893756	2	18	36	AC	10	9
88969	9	893838	893871	2	17	34	AC	12	10
88971	9	896968	896979	2	6	12	AG	31	3
88972	9	897355	897375	3	7	21	TCC	22	6
88974	9	897547	897560	2	7	14	AC	15	4
88976	9	898319	898362	2	22	44	CA	10	9
88977	9	898398	898411	2	7	14	AC	20	4
88978	9	898516	898563	2	24	48	CA	15	15
88980	9	901485	901508	3	8	24	CAA	18	10
88981	9	903611	903656	2	23	46	CA	9	12
88983	9	908434	908453	4	5	20	TCTT	25	8
88985	9	910518	910529	2	6	12	CA	8	6
88990	9	911449	911504	2	28	56	TG	13	15
88995	9	912011	912048	2	19	38	AC	13	13
88996	9	912100	912111	2	6	12	TC	10	4
88998	9	912198	912213	2	8	16	CT	18	7
88999	9	912255	912280	2	13	26	AC	17	6
89000	9	912392	912445	2	27	54	AC	20	13
89002	9	915070	915115	2	23	46	AC	6	9
89006	9	919470	919502	3	11	33	CTC	11	5
89007	9	919604	919621	2	9	18	CA	21	11
89010	9	927717	927752	2	18	36	GT	6	7
89012	9	930150	930173	2	12	24	TG	16	8
89014	9	932191	932204	2	7	14	GT	23	5
89015	9	932380	932395	2	8	16	TG	28	8
89016	9	932485	932498	2	7	14	TG	21	3
89017	9	932526	932549	3	8	24	GCC	23	5
89020	9	934501	934520	2	10	20	TG	19	12
89021	9	936021	936060	2	20	40	CA	11	11
89027	9	942385	942414	2	15	30	AC	11	14
89030	9	943692	943717	2	13	26	CA	15	6
89032	9	948106	948129	4	6	24	AATA	27	5
89033	9	948242	948271	2	15	30	GT	10	10
89035	9	951322	951341	2	10	20	AC	13	10
89036	9	951674	951703	2	15	30	AC	11	6
89037	9	951870	951887	2	9	18	CA	25	13
89038	9	954783	954804	2	11	22	TC	19	5
89039	9	954843	954870	2	14	28	AC	14	10
89045	9	960104	960121	2	9	18	CA	18	7
89047	9	961275	961286	2	6	12	AC	20	5
89051	9	966081	966095	3	5	15	CCT	24	7
89053	9	967519	967533	3	5	15	CCA	27	7
89054	9	967726	967741	2	8	16	CT	19	3
89055	9	967796	967807	2	6	12	CA	15	4
89056	9	967871	967894	2	12	24	AC	18	6

89060	9	973008	973022	3	5	15	GGT	26	3
89061	9	973147	973167	3	7	21	TCT	20	10
89066	9	976901	976920	2	10	20	CA	16	6
89067	9	978223	978248	2	13	26	CA	16	9
89068	9	978427	978440	2	7	14	AC	23	9
89070	9	978538	978567	2	15	30	CA	13	13
89074	9	981407	981420	2	7	14	CT	13	4
89079	9	995939	995954	2	8	16	TG	22	5
89081	9	998060	998083	2	12	24	AC	10	5
89086	9	1003054	1003077	2	12	24	TG	6	7
89088	9	1009327	1009340	2	7	14	CA	13	3
89090	9	1012129	1012155	3	9	27	AGA	26	8
89091	9	1012279	1012296	2	9	18	AC	10	9
89095	9	1015274	1015309	3	12	36	GAG	17	7
89098	9	1015682	1015713	2	16	32	TG	13	11
89099	9	1015794	1015807	2	7	14	GT	18	6
89100	9	1015871	1015890	2	10	20	TG	10	11
89101	9	1016960	1016999	2	20	40	AC	6	9
89107	9	1025892	1025912	3	7	21	AGC	23	11
89108	9	1026231	1026242	2	6	12	TG	7	5
89109	9	1026245	1026256	2	6	12	TG	6	6
89111	9	1029496	1029525	2	15	30	GT	16	7
89112	9	1029600	1029637	2	19	38	TG	7	9
89115	9	1030186	1030203	2	9	18	GT	20	8
89121	9	1033531	1033572	2	21	42	CA	8	8
89126	9	1037723	1037742	2	10	20	AC	16	12
89129	9	1043437	1043451	3	5	15	GCT	26	3
89131	9	1044044	1044067	2	12	24	AC	14	4
89132	9	1044197	1044218	2	11	22	TG	11	11
89133	9	1044636	1044651	4	4	16	ACAA	22	3
89143	9	1049073	1049086	2	7	14	CA	24	6
89145	9	1049622	1049659	2	19	38	TG	8	7
89147	9	1050935	1050952	3	6	18	GCA	23	5
89149	9	1051606	1051629	2	12	24	GT	19	18
89161	9	1054519	1054539	3	7	21	GAG	21	6
89162	9	1054738	1054777	2	20	40	TG	10	10
89164	9	1054990	1055003	2	7	14	TG	7	7
89165	9	1055073	1055096	2	12	24	AG	6	7
89166	9	1055159	1055174	2	8	16	GT	20	7
89170	9	1059112	1059135	2	12	24	AG	13	6
89175	9	1060693	1060710	2	9	18	CT	6	6
89176	9	1061379	1061393	3	5	15	GAG	22	3
89180	9	1065942	1065961	2	10	20	AG	15	9
89181	9	1066118	1066139	2	11	22	TG	15	13
89183	9	1066304	1066315	2	6	12	TC	21	3
89185	9	1066462	1066491	2	15	30	GT	23	9
89186	9	1067070	1067089	4	5	20	AAAT	19	3
89187	9	1068454	1068467	2	7	14	AC	27	6
89188	9	1068575	1068604	2	15	30	AC	13	13
89189	9	1068716	1068727	2	6	12	TG	24	4
89193	9	1069230	1069249	2	10	20	GT	6	7

89194	9	1069330	1069363	2	17	34	GT	14	11
89200	9	1071966	1072001	2	18	36	GT	9	4
89201	9	1072041	1072058	2	9	18	TG	17	9
89202	9	1072258	1072289	2	16	32	AC	14	8
89203	9	1073120	1073143	2	12	24	CT	19	6
89205	9	1073342	1073369	2	14	28	TG	16	9
89207	9	1074693	1074726	2	17	34	AC	11	7
89209	9	1075608	1075633	2	13	26	GT	8	6
89210	9	1075719	1075746	2	14	28	GT	25	8
89212	9	1078657	1078674	2	9	18	CA	17	7
89213	9	1078750	1078767	2	9	18	GT	24	5
89216	9	1081147	1081168	2	11	22	TG	6	7
89218	9	1081279	1081308	2	15	30	TG	14	10
89221	9	1081675	1081686	2	6	12	AC	23	6
89222	9	1081921	1081960	2	20	40	CA	8	8
89223	9	1082087	1082100	2	7	14	CA	14	12
89226	9	1083780	1083819	2	20	40	AC	9	10
89227	9	1083941	1083968	2	14	28	AC	11	11
89229	9	1084190	1084213	2	12	24	AC	13	10
89230	9	1084343	1084362	2	10	20	GA	15	12
89231	9	1084501	1084520	2	10	20	GT	21	9
89232	9	1085458	1085485	2	14	28	TG	16	13
89233	9	1085525	1085546	2	11	22	GT	24	5
89234	9	1087217	1087234	2	9	18	AC	20	6
89235	9	1087378	1087419	2	21	42	CA	18	11
89236	9	1088190	1088207	2	9	18	AC	20	10
89237	9	1089470	1089490	3	7	21	AGG	21	7
89238	9	1089685	1089708	3	8	24	AGG	9	4
89240	9	1090601	1090632	2	16	32	TG	11	8
89241	9	1090713	1090746	2	17	34	TG	6	8
89242	9	1090885	1090904	2	10	20	GT	11	7
89245	9	1091339	1091360	2	11	22	GT	18	11
89247	9	1092544	1092559	2	8	16	GT	22	9
89248	9	1092752	1092781	2	15	30	GT	6	9
89249	9	1092936	1092963	2	14	28	AC	13	8
89250	9	1093816	1093835	2	10	20	AC	15	4
89251	9	1094405	1094426	2	11	22	GT	18	7
89252	9	1094940	1094953	2	7	14	CA	28	4
89254	9	1096986	1097001	4	4	16	TTAA	24	3
89255	9	1097338	1097355	2	9	18	TG	20	8
89257	9	1097602	1097615	2	7	14	AC	31	5
89258	9	1097617	1097628	2	6	12	CA	30	5
89259	9	1097803	1097832	2	15	30	AC	22	10
89260	9	1099854	1099873	2	10	20	TC	7	5
89263	9	1102396	1102413	2	9	18	AC	17	3
89266	9	1106120	1106157	2	19	38	CA	12	9
89267	9	1106343	1106356	2	7	14	AG	12	4
89271	9	1110641	1110688	2	24	48	GT	6	9
89273	9	1114703	1114716	2	7	14	AC	30	4
89274	9	1114951	1114962	2	6	12	TG	28	6
89275	9	1115280	1115305	2	13	26	GT	16	11

89276	9	1119045	1119078	2	17	34	GT	13	10
89277	9	1119197	1119224	2	14	28	AC	7	7
89279	9	1119592	1119611	2	10	20	TG	8	5
89280	9	1119752	1119775	2	12	24	AC	12	7
89281	9	1120035	1120052	2	9	18	CA	14	3
89282	9	1121022	1121037	2	8	16	AC	25	4
89284	9	1122073	1122124	2	26	52	AC	20	15
89287	9	1123674	1123699	2	13	26	CA	17	10
89289	9	1125796	1125833	2	19	38	GT	14	12
89290	9	1125909	1125944	3	12	36	TGA	22	10
89291	9	1126001	1126020	2	10	20	TG	25	9
89293	9	1126537	1126556	2	10	20	TG	21	6
89294	9	1126634	1126661	2	14	28	CA	18	10
89295	9	1127909	1127924	2	8	16	TG	23	4
89298	9	1129597	1129628	2	16	32	GT	10	9
89299	9	1130193	1130224	2	16	32	GT	9	13
89304	9	1133405	1133430	2	13	26	AC	15	12
89307	9	1135160	1135183	2	12	24	GT	17	14
89308	9	1136060	1136073	2	7	14	AC	26	3
89310	9	1137017	1137030	2	7	14	GT	27	3
89312	9	1138551	1138578	4	7	28	TCTT	17	8
89313	9	1138691	1138714	2	12	24	TG	13	11
89314	9	1144586	1144601	2	8	16	TA	10	7
89317	9	1144828	1144843	2	8	16	TG	16	5
89318	9	1144845	1144856	2	6	12	GT	17	6
89319	9	1145337	1145368	2	16	32	TG	13	10
89320	9	1147058	1147083	2	13	26	CA	9	10
89323	9	1147495	1147530	2	18	36	GT	13	5
89324	9	1147644	1147661	2	9	18	TG	23	4
89327	9	1148897	1148912	2	8	16	GT	24	9
89328	9	1150549	1150562	2	7	14	TG	26	4
89329	9	1150718	1150741	4	6	24	TTTC	20	7
89334	9	1152213	1152230	2	9	18	TG	12	3
89337	9	1154230	1154279	2	25	50	AC	11	16
89339	9	1159553	1159570	2	9	18	TG	23	14
89340	9	1159733	1159748	4	4	16	CTTC	25	4
89342	9	1160445	1160456	2	6	12	TG	22	4
89343	9	1161338	1161365	2	14	28	GT	13	10
89346	9	1166771	1166798	2	14	28	GT	26	8
89348	9	1168505	1168516	2	6	12	CA	14	12
89350	9	1168799	1168856	2	29	58	CA	7	9
89351	9	1169035	1169046	2	6	12	AC	22	5
89352	9	1169362	1169383	2	11	22	CA	13	6
89354	9	1170545	1170572	2	14	28	TG	12	11
89356	9	1172893	1172908	2	8	16	AC	29	3
89359	9	1176148	1176163	2	8	16	AC	26	9
89360	9	1176322	1176335	2	7	14	AC	23	8
89365	9	1178362	1178373	2	6	12	AC	32	4
89369	9	1182316	1182331	2	8	16	GT	18	9
89371	9	1182499	1182528	2	15	30	TG	6	7
89372	9	1182701	1182712	2	6	12	TG	12	8

89373	9	1182715	1182726	2	6	12	TG	16	10
89378	9	1183494	1183515	2	11	22	AG	19	12
89380	9	1190503	1190526	2	12	24	TG	28	5
89381	9	1194265	1194286	2	11	22	AC	13	4
89386	9	1198485	1198514	2	15	30	AC	10	10
89388	9	1199725	1199736	2	6	12	AC	27	5
89391	9	1204161	1204200	2	20	40	GT	8	11
89393	9	1210115	1210172	2	29	58	GT	15	8
89394	9	1211013	1211048	2	18	36	AC	8	8
89395	9	1211120	1211131	2	6	12	AC	22	3
89399	9	1216458	1216477	2	10	20	GA	22	4
89401	9	1218358	1218377	2	10	20	TC	21	8
89404	9	1226060	1226089	2	15	30	AC	13	11
89408	9	1227187	1227204	2	9	18	AC	17	3
89413	9	1233084	1233097	2	7	14	CA	18	9
89417	9	1236856	1236885	2	15	30	CA	6	5
89423	9	1243626	1243639	2	7	14	AT	8	3
89424	9	1245854	1245873	4	5	20	AACC	22	3
89428	9	1249221	1249236	2	8	16	CA	19	6
89429	9	1250864	1250881	2	9	18	CA	16	8
89433	9	1261672	1261685	2	7	14	AG	19	5
89434	9	1263223	1263238	2	8	16	AC	28	3
89439	9	1271051	1271068	2	9	18	CA	21	4
89443	9	1272496	1272507	2	6	12	GT	15	5
89444	9	1272634	1272647	2	7	14	TG	17	11
89445	9	1272738	1272755	2	9	18	GT	13	5
89446	9	1274818	1274835	2	9	18	GT	7	4
89449	9	1274960	1274983	2	12	24	GT	19	8
89450	9	1275055	1275072	2	9	18	TG	26	4
89451	9	1275192	1275207	2	8	16	CA	22	3
89458	9	1291960	1291985	2	13	26	TG	23	6
89459	9	1293259	1293276	2	9	18	CA	15	8
89460	9	1294694	1294711	2	9	18	AC	29	4
89461	9	1294917	1294936	2	10	20	TG	22	6
89464	9	1297182	1297217	2	18	36	GT	13	15
89465	9	1297249	1297270	2	11	22	CA	20	8
89469	9	1301483	1301512	2	15	30	AC	20	15
89470	9	1303998	1304019	2	11	22	CA	7	9
89472	9	1304181	1304206	2	13	26	CA	14	5
89473	9	1304353	1304384	2	16	32	CA	18	13
89477	9	1306041	1306070	2	15	30	CA	14	8
89479	9	1309226	1309249	2	12	24	GT	10	9
89481	9	1310431	1310450	4	5	20	GTGA	13	6
89483	9	1311733	1311748	2	8	16	TG	23	6
89484	9	1311910	1311937	2	14	28	CA	9	9
89487	9	1312104	1312129	2	13	26	AC	8	5
89490	9	1313947	1313962	2	8	16	TG	24	3
89496	9	1318779	1318796	2	9	18	TG	22	10
89505	9	1325574	1325599	2	13	26	CA	18	11
89506	9	1325639	1325652	2	7	14	AC	28	3
89509	9	1329890	1329903	2	7	14	AG	10	3

89510	9	1334575	1334596	2	11	22	AC	14	15
89511	9	1334970	1334987	3	6	18	TTC	24	3
89515	9	1336447	1336470	2	12	24	GT	13	7
89517	9	1336621	1336644	2	12	24	TG	14	11
89521	9	1340256	1340267	2	6	12	TG	22	5
89522	9	1340271	1340282	2	6	12	GT	21	3
89523	9	1340371	1340390	2	10	20	AC	17	15
89525	9	1345704	1345715	2	6	12	CA	15	3
89526	9	1345829	1345843	3	5	15	CCA	13	3
89528	9	1346457	1346480	2	12	24	GT	18	7
89529	9	1347274	1347291	2	9	18	CT	18	6
89533	9	1349629	1349668	2	20	40	GT	10	14
89534	9	1349824	1349841	2	9	18	TG	21	5
89535	9	1349862	1349877	2	8	16	GT	15	5
89536	9	1350000	1350013	2	7	14	AG	27	6
89537	9	1350110	1350121	2	6	12	GA	8	3
89540	9	1354652	1354665	2	7	14	GA	22	4
89542	9	1355777	1355800	2	12	24	CA	20	8
89546	9	1362203	1362236	2	17	34	CA	11	7
89548	9	1363989	1364008	2	10	20	GT	17	3
89550	9	1364116	1364139	2	12	24	CA	11	7
89557	9	1369574	1369591	2	9	18	CA	20	6
89566	9	1378185	1378202	2	9	18	AG	18	12
89570	9	1379901	1379916	2	8	16	GT	20	8
89571	9	1380200	1380215	2	8	16	GT	23	6
89572	9	1380339	1380368	2	15	30	AC	6	7
89573	9	1380504	1380531	2	14	28	TG	13	10
89574	9	1380698	1380713	4	4	16	AAAG	26	8
89578	9	1381964	1382023	2	30	60	AC	8	11
89579	9	1382154	1382185	2	16	32	GT	6	8
89580	9	1382342	1382373	2	16	32	TG	8	7
89581	9	1385069	1385086	2	9	18	AC	16	9
89582	9	1385263	1385292	2	15	30	AC	12	15
89583	9	1385429	1385452	2	12	24	AC	21	7
89585	9	1390312	1390337	2	13	26	CA	18	12
89586	9	1392495	1392520	2	13	26	TG	13	6
89589	9	1395339	1395358	2	10	20	TC	14	10
89593	9	1398471	1398482	2	6	12	AC	25	10
89594	9	1399814	1399843	2	15	30	GT	14	13
89596	9	1399969	1399996	2	14	28	AC	10	12
89597	9	1400122	1400149	2	14	28	AC	17	11
89598	9	1402083	1402096	2	7	14	CA	22	3
89600	9	1403321	1403352	2	16	32	TG	9	10
89602	9	1405042	1405061	2	10	20	GT	13	12
89603	9	1405608	1405629	2	11	22	CT	15	8
89604	9	1405800	1405825	2	13	26	TC	14	4
89605	9	1406029	1406050	2	11	22	GT	19	9
89608	9	1407998	1408019	2	11	22	GT	14	5
89610	9	1408365	1408376	2	6	12	TG	30	5
89612	9	1409615	1409648	2	17	34	GT	6	8
89615	9	1413684	1413704	3	7	21	GAA	25	6

89616	9	1413930	1413949	2	10	20	AG	16	6
89617	9	1414299	1414324	2	13	26	GT	20	12
89618	9	1415509	1415532	4	6	24	TTTA	29	3
89620	9	1417261	1417276	2	8	16	AG	19	3
89622	9	1419018	1419051	2	17	34	AC	7	5
89626	9	1424737	1424750	2	7	14	CA	9	7
89627	9	1424843	1424858	2	8	16	CA	19	6
89628	9	1425002	1425037	2	18	36	TG	7	6
89629	9	1425254	1425273	2	10	20	AT	21	10
89632	9	1430971	1430994	4	6	24	AGGC	11	4
89646	9	1447843	1447868	2	13	26	CA	12	15
89647	9	1447919	1447970	2	26	52	AC	6	8
89650	9	1448329	1448340	2	6	12	TA	17	8
89651	9	1448472	1448501	2	15	30	AC	20	11
89652	9	1448695	1448718	2	12	24	CA	16	9
89653	9	1450857	1450868	2	6	12	GA	20	5
89654	9	1451951	1451962	2	6	12	TA	22	6
89655	9	1452077	1452092	4	4	16	CTGA	14	4
89657	9	1453979	1454002	4	6	24	GTGC	12	13
89659	9	1454105	1454140	2	18	36	GT	8	5
89662	9	1456019	1456034	2	8	16	CA	16	12
89667	9	1459634	1459647	2	7	14	CA	19	9
89669	9	1468051	1468064	2	7	14	AT	20	3
89670	9	1468146	1468157	2	6	12	CA	29	7
89676	9	1470310	1470325	2	8	16	GT	27	5
89677	9	1471479	1471490	2	6	12	GT	23	5
89679	9	1471794	1471807	2	7	14	AC	24	6
89681	9	1472477	1472504	2	14	28	AC	24	7
89686	9	1473769	1473789	3	7	21	CTC	24	6
89694	9	1481123	1481149	3	9	27	AAC	23	3
89695	9	1481347	1481373	3	9	27	CTC	23	7
89696	9	1481624	1481635	2	6	12	GT	9	6
89698	9	1483968	1484006	3	13	39	ATC	24	15
89704	9	1488795	1488814	2	10	20	AG	8	6
89705	9	1488910	1488927	2	9	18	AC	20	10
89708	9	1495171	1495192	2	11	22	AC	20	11
89709	9	1495289	1495318	2	15	30	CA	14	12
89712	9	1497702	1497713	2	6	12	TG	20	3
89714	9	1498534	1498547	2	7	14	AC	16	3
89715	9	1498670	1498685	2	8	16	CA	23	8
89717	9	1500260	1500277	3	6	18	CCT	19	5
89718	9	1501324	1501353	2	15	30	AC	18	16
89719	9	1501430	1501451	2	11	22	GT	24	13
89720	9	1501529	1501556	2	14	28	AC	16	10
89721	9	1503312	1503331	2	10	20	CA	24	9
89722	9	1503368	1503381	2	7	14	CA	24	6
89723	9	1503513	1503526	2	7	14	GT	21	9
89724	9	1503677	1503712	2	18	36	GT	16	15
89725	9	1505362	1505397	2	18	36	CA	8	7
89728	9	1510027	1510050	2	12	24	TG	19	15
89729	9	1510194	1510205	2	6	12	CA	18	4

89739	9	1524124	1524138	3	5	15	TGT	26	8
89740	9	1527270	1527293	2	12	24	CA	11	6
89742	9	1527545	1527586	2	21	42	CA	10	9
89743	9	1527654	1527671	2	9	18	TG	20	7
89744	9	1529842	1529859	2	9	18	GT	21	7
89745	9	1532808	1532829	2	11	22	TG	17	7
89747	9	1534224	1534239	2	8	16	CT	21	4
89748	9	1535499	1535514	2	8	16	AT	18	3
89749	9	1535673	1535700	2	14	28	CA	15	6
89753	9	1559085	1559108	4	6	24	AAAT	16	4
89754	9	1561687	1561706	2	10	20	TG	24	8
89755	9	1566010	1566024	3	5	15	CTG	19	4
89758	9	1569789	1569808	2	10	20	TG	24	12
89759	9	1571238	1571255	2	9	18	GT	24	15
89760	9	1571420	1571437	2	9	18	TG	18	12
89764	9	1577864	1577884	3	7	21	TTG	16	4
89765	9	1578680	1578703	3	8	24	TAA	19	7
89766	9	1580287	1580304	2	9	18	GA	18	5
89767	9	1580852	1580869	3	6	18	GAA	23	3
89768	9	1581322	1581336	3	5	15	AAC	29	5
89775	9	1585868	1585903	3	12	36	AAT	10	5
89791	9	1613249	1613268	2	10	20	AC	11	6
89801	9	1622354	1622368	3	5	15	ATC	32	3
89802	9	1623280	1623293	2	7	14	AG	29	3
89805	9	1629295	1629320	2	13	26	TG	16	5
89810	9	1642285	1642298	2	7	14	CA	19	4
89815	9	1651027	1651046	2	10	20	CA	22	7
89816	9	1651324	1651351	2	14	28	CA	19	7
89817	9	1656285	1656304	2	10	20	CA	17	6
89818	9	1656376	1656393	2	9	18	CA	7	7
89820	9	1656532	1656557	2	13	26	AC	8	6
89823	9	1658308	1658321	2	7	14	AC	6	4
89825	9	1663072	1663107	3	12	36	CAA	7	8
89826	9	1665935	1665980	2	23	46	GT	13	9
89831	9	1676092	1676103	2	6	12	GT	25	3
89836	9	1685016	1685031	2	8	16	AC	18	4
89840	9	1685239	1685254	4	4	16	AGCC	11	4
89841	9	1685398	1685415	2	9	18	TC	16	5
89843	9	1685580	1685597	3	6	18	GTT	20	7
89846	9	1689805	1689822	2	9	18	CA	22	6
89848	9	1693935	1693958	4	6	24	TAAA	18	5
89849	9	1695461	1695484	3	8	24	CCT	27	6
89850	9	1700573	1700606	2	17	34	AC	17	13
89851	9	1700724	1700761	2	19	38	GT	6	8
89852	9	1700873	1700912	2	20	40	TG	13	10
89854	9	1703051	1703070	2	10	20	GA	7	8
89860	9	1706745	1706778	2	17	34	GT	7	9
89862	9	1707153	1707172	2	10	20	GT	16	8
89863	9	1707728	1707748	3	7	21	AAT	8	4
89868	9	1709836	1709859	2	12	24	AC	16	13
89869	9	1716512	1716527	4	4	16	TATC	8	3

89870	9	1716888	1716921	2	17	34	AC	20	7
89877	9	1723212	1723243	2	16	32	AG	16	10
89879	9	1724951	1724966	2	8	16	GT	22	7
89880	9	1727325	1727360	2	18	36	TG	19	17
89884	9	1728206	1728231	2	13	26	AC	20	11
89885	9	1728802	1728817	2	8	16	CA	25	7
89886	9	1729024	1729061	2	19	38	TG	10	15
89889	9	1729361	1729384	2	12	24	AC	11	9
89890	9	1731837	1731851	3	5	15	CTC	28	5
89892	9	1733511	1733538	2	14	28	TG	19	8
89893	9	1737766	1737780	3	5	15	AAT	17	5
89895	9	1739996	1740017	2	11	22	CA	16	9
89896	9	1740365	1740382	3	6	18	CAT	30	3
89899	9	1745228	1745239	2	6	12	AT	22	5
89903	9	1747417	1747454	2	19	38	AC	19	14
89904	9	1748101	1748120	2	10	20	CA	24	8
89905	9	1748753	1748776	2	12	24	CA	7	8
89907	9	1748924	1748945	2	11	22	CA	18	8
89910	9	1751864	1751887	3	8	24	AGA	26	8
89912	9	1752810	1752825	2	8	16	CT	21	5
89913	9	1753808	1753823	2	8	16	GT	20	13
89915	9	1754016	1754033	3	6	18	ACA	11	3
89916	9	1754089	1754108	4	5	20	TCCT	24	3
89918	9	1755483	1755502	2	10	20	GT	21	7
89920	9	1758754	1758775	2	11	22	CA	18	7
89925	9	1771352	1771378	3	9	27	TTC	23	3
89926	9	1771867	1771878	2	6	12	GT	25	4
89927	9	1772854	1772877	2	12	24	TG	14	11
89928	9	1773042	1773061	2	10	20	CA	18	12
89931	9	1778090	1778128	3	13	39	TGA	24	9
89932	9	1780259	1780288	3	10	30	GGA	14	10
89933	9	1780347	1780364	2	9	18	CT	23	6
89935	9	1781945	1781959	3	5	15	ACA	25	3
89940	9	1785350	1785369	4	5	20	TATT	15	4
89944	9	1788924	1788938	3	5	15	AGG	33	3
89946	9	1791474	1791511	2	19	38	CA	22	13
89947	9	1792873	1792887	3	5	15	AGA	28	6
89948	9	1795938	1795955	3	6	18	CCT	21	3
89953	9	1804772	1804787	4	4	16	CAAA	26	5
89956	9	1809787	1809800	2	7	14	GT	19	4
89959	9	1811376	1811401	2	13	26	CA	19	7
89960	9	1811523	1811546	2	12	24	AG	23	15
89961	9	1811590	1811613	3	8	24	CTC	16	7
89962	9	1811948	1811965	3	6	18	GAG	19	5
89963	9	1812122	1812137	2	8	16	GT	28	7
89964	9	1812927	1812968	2	21	42	TG	9	8
89965	9	1813103	1813140	2	19	38	GT	7	6
89967	9	1814179	1814200	2	11	22	AC	17	11
89974	9	1819160	1819189	2	15	30	TG	12	7
89976	9	1819384	1819409	2	13	26	CA	24	10
89978	9	1825261	1825275	3	5	15	TGC	24	3

89979	9	1826925	1826936	2	6	12	TG	26	5
89980	9	1832502	1832522	3	7	21	CTG	25	7
89981	9	1834226	1834253	2	14	28	CA	16	17
89982	9	1834405	1834428	2	12	24	CA	15	11
89985	9	1835667	1835682	2	8	16	GA	12	10
89988	9	1836173	1836188	4	4	16	AAGA	13	4
89989	9	1836913	1836942	2	15	30	AC	9	8
89992	9	1845852	1845873	2	11	22	AC	30	7
89993	9	1851602	1851625	2	12	24	TG	15	7
89994	9	1852093	1852140	2	24	48	GA	10	11
89995	9	1853066	1853079	2	7	14	AC	26	5
89997	9	1854276	1854287	2	6	12	TG	26	8
90000	9	1869115	1869160	2	23	46	GT	12	13
90002	9	1871784	1871799	2	8	16	AC	22	12
90003	9	1872021	1872034	2	7	14	TG	29	4
90007	9	1894942	1894959	3	6	18	AGG	20	6
90011	9	1897278	1897319	3	14	42	AAG	13	7
90012	9	1897731	1897746	4	4	16	TTTA	16	6
90015	9	1906808	1906839	4	8	32	TTTA	18	9
90019	9	1919846	1919866	3	7	21	CCT	16	6
90020	9	1920732	1920761	2	15	30	TG	11	7
90030	9	1935723	1935744	2	11	22	GT	24	7
90033	9	1941747	1941772	2	13	26	AG	10	8
90034	9	1941820	1941837	2	9	18	GT	19	9
90036	9	1941960	1941983	2	12	24	TG	16	12
90037	9	1942118	1942149	2	16	32	CA	13	13
90039	9	1944878	1944897	2	10	20	GT	22	4
90040	9	1948154	1948173	2	10	20	GT	9	9
90041	9	1948345	1948372	2	14	28	AC	21	8
90043	9	1948832	1948847	2	8	16	GT	6	5
90044	9	1948941	1948962	2	11	22	TG	15	6
90052	9	1956143	1956176	2	17	34	GT	10	10
90053	9	1956274	1956289	2	8	16	TG	19	4
90054	9	1956360	1956375	2	8	16	CA	24	7
90055	9	1957924	1957937	2	7	14	CA	21	10
90057	9	1959716	1959747	2	16	32	AC	20	16
90059	9	1960803	1960818	2	8	16	TG	12	5
90062	9	1965016	1965051	2	18	36	CA	15	17
90069	9	1968730	1968745	2	8	16	AC	24	16
90073	9	1974900	1974915	2	8	16	AC	20	11
90074	9	1974986	1975009	3	8	24	CAT	27	8
90075	9	1977429	1977460	2	16	32	AC	9	11
90076	9	1978683	1978720	2	19	38	CA	10	10
90078	9	1979331	1979351	3	7	21	AGA	21	7
90080	9	1979702	1979727	2	13	26	CA	11	7
90082	9	1981899	1981916	2	9	18	TG	19	10
90087	9	1986968	1986983	2	8	16	AC	13	10
90089	9	1987122	1987133	2	6	12	CA	21	8
90090	9	1987404	1987439	2	18	36	CA	9	8
90095	9	1994515	1994542	2	14	28	AC	15	11
90096	9	1995375	1995400	2	13	26	AC	17	9

90097	9	1996478	1996495	3	6	18	CAT	23	5
90098	9	1996741	1996764	2	12	24	AC	10	9
90104	9	2000095	2000112	2	9	18	TG	22	6
90107	9	2003477	2003494	3	6	18	ATG	19	7
90116	9	2007601	2007628	2	14	28	AC	25	13
90117	9	2010866	2010883	2	9	18	TG	20	13
90118	9	2011318	2011357	2	20	40	CA	11	11
90120	9	2012115	2012132	2	9	18	CA	14	7
90121	9	2020186	2020200	3	5	15	AAC	29	4
90122	9	2021925	2021940	2	8	16	AG	26	4
90123	9	2027031	2027056	2	13	26	TG	8	5
90124	9	2027089	2027110	2	11	22	AC	22	7
90125	9	2031659	2031682	2	12	24	CA	15	9
90128	9	2034862	2034889	2	14	28	GT	15	7
90131	9	2040681	2040692	2	6	12	AC	33	3
90132	9	2040931	2040950	2	10	20	CA	20	10
90134	9	2041336	2041353	2	9	18	AC	25	6
90137	9	2055201	2055212	2	6	12	GT	20	4
90140	9	2066104	2066117	2	7	14	CT	30	3
90141	9	2066868	2066885	2	9	18	TG	15	9
90142	9	2066888	2066901	2	7	14	TG	14	7
90145	9	2068039	2068062	2	12	24	TG	22	6
90146	9	2068486	2068505	4	5	20	TCTG	25	9
90150	9	2072635	2072652	2	9	18	TG	17	5
90151	9	2073381	2073418	2	19	38	AC	15	12
90155	9	2076759	2076773	3	5	15	TTA	13	3
90165	9	2089124	2089157	2	17	34	AC	6	7
90169	9	2096226	2096249	2	12	24	TG	10	10
90178	9	2107756	2107773	2	9	18	TG	10	4
90183	9	2111734	2111771	2	19	38	AC	9	7
90189	9	2117299	2117328	2	15	30	AC	8	6
90191	9	2120500	2120545	2	23	46	AC	11	14
90192	9	2120574	2120585	2	6	12	GT	21	4
90193	9	2120637	2120670	2	17	34	GT	21	10
90195	9	2121998	2122021	3	8	24	TCC	10	8
90196	9	2122157	2122168	2	6	12	TG	21	4
90198	9	2122870	2122883	2	7	14	CT	12	3
90199	9	2123786	2123803	3	6	18	GAG	14	3
90200	9	2124389	2124418	3	10	30	CCT	18	11
90201	9	2124698	2124715	2	9	18	TC	15	5
90202	9	2125173	2125206	2	17	34	GT	15	14
90204	9	2125314	2125333	2	10	20	GT	8	10
90206	9	2127500	2127513	2	7	14	TC	22	3
90211	9	2136316	2136351	2	18	36	AC	8	8
90216	9	2138369	2138388	4	5	20	CTGT	29	5
90217	9	2138472	2138495	2	12	24	AG	15	8
90218	9	2138581	2138610	2	15	30	CA	16	9
90219	9	2141436	2141479	2	22	44	TG	10	9
90220	9	2143887	2143920	2	17	34	AC	8	10
90221	9	2144016	2144031	2	8	16	TG	15	5
90222	9	2144057	2144100	2	22	44	GT	15	11

90224	9	2144260	2144283	2	12	24	CA	16	6
90225	9	2144498	2144511	2	7	14	GT	20	7
90226	9	2144518	2144529	2	6	12	GT	14	5
90227	9	2145299	2145331	3	11	33	TTC	21	10
90229	9	2147640	2147673	2	17	34	AC	13	9
90237	9	2155195	2155230	2	18	36	GT	13	8
90238	9	2155721	2155746	2	13	26	TG	23	7
90244	9	2163019	2163040	2	11	22	GT	10	11
90246	9	2168810	2168829	4	5	20	AAAG	24	4
90247	9	2169075	2169090	4	4	16	AAAC	30	7
90252	9	2199855	2199869	3	5	15	AAG	27	4
90253	9	2202240	2202283	2	22	44	GT	7	4
90254	9	2202384	2202404	3	7	21	GGA	24	6
90256	9	2203817	2203840	3	8	24	CCT	24	6
90257	9	2204624	2204637	2	7	14	AC	18	4
90264	9	2217406	2217423	3	6	18	GAT	13	6
90269	9	2222300	2222311	2	6	12	TG	17	3
90270	9	2222341	2222364	2	12	24	TG	12	8
90271	9	2222519	2222534	2	8	16	TG	21	4
90273	9	2226118	2226133	2	8	16	TG	19	6
90274	9	2228318	2228335	2	9	18	GT	21	6
90276	9	2233843	2233860	2	9	18	GT	20	6
90281	9	2241778	2241793	2	8	16	AC	29	6
90283	9	2244242	2244265	2	12	24	GT	17	5
90284	9	2246925	2246942	2	9	18	GA	25	7
90288	9	2249686	2249743	2	29	58	CA	13	6
90289	9	2249860	2249895	2	18	36	AC	8	11
90290	9	2260769	2260784	2	8	16	TG	21	4
90291	9	2263409	2263424	4	4	16	TCTG	22	3
90292	9	2266704	2266729	2	13	26	AC	14	12
90293	9	2281302	2281325	4	6	24	TGTC	29	4
90297	9	2291705	2291728	2	12	24	TG	7	5
90305	9	2304758	2304769	2	6	12	AC	22	3
90306	9	2308853	2308866	2	7	14	AT	21	10
90308	9	2316863	2316890	2	14	28	TG	19	11
90309	9	2319541	2319567	3	9	27	GAG	22	12
90310	9	2320077	2320100	2	12	24	TG	17	14
90311	9	2322321	2322344	2	12	24	CA	19	7
90313	9	2324815	2324832	2	9	18	TG	22	12
90324	9	2337322	2337341	2	10	20	TG	18	10
90325	9	2339197	2339210	2	7	14	TG	12	7
90327	9	2339955	2339974	2	10	20	AC	19	4
90328	9	2342837	2342848	2	6	12	GT	20	9
90329	9	2343426	2343459	2	17	34	TG	15	13
90330	9	2343646	2343689	2	22	44	AC	8	8
90331	9	2343766	2343795	2	15	30	TG	9	9
90334	9	2348763	2348798	4	9	36	TCTG	27	7
90342	9	2360700	2360739	2	20	40	TG	13	12
90343	9	2360891	2360904	2	7	14	TC	31	3
90344	9	2362115	2362134	2	10	20	TG	18	7
90345	9	2363779	2363796	2	9	18	AC	22	9

90346	9	2372288	2372299	2	6	12	AT	24	5
90353	9	2381157	2381182	2	13	26	GT	18	8
90363	9	2410144	2410158	3	5	15	CAA	26	9
90366	9	2414074	2414087	2	7	14	CA	14	7
90370	9	2422398	2422411	2	7	14	TG	20	5
90371	9	2424742	2424757	2	8	16	GT	11	4
90372	9	2424909	2424944	2	18	36	AC	14	12
90374	9	2426975	2426990	2	8	16	CA	8	3
90375	9	2429018	2429038	3	7	21	AAC	29	8
90378	9	2433145	2433164	4	5	20	GACA	33	3
90379	9	2437485	2437502	2	9	18	AC	12	8
90381	9	2438410	2438421	2	6	12	CA	17	3
90382	9	2439284	2439305	2	11	22	AG	16	4
90384	9	2440560	2440579	2	10	20	GT	15	8
90385	9	2442947	2442980	2	17	34	CA	18	15
90393	9	2453544	2453569	2	13	26	GT	11	7
90400	9	2462622	2462633	2	6	12	CA	31	4
90403	9	2465987	2466006	2	10	20	CA	19	8
90404	9	2470137	2470148	2	6	12	AT	13	4
90405	9	2472909	2472930	2	11	22	TG	16	6
90408	9	2478619	2478648	2	15	30	AC	9	11
90418	9	2493562	2493591	2	15	30	CA	18	9
90419	9	2495410	2495425	2	8	16	AG	27	3
90420	9	2496861	2496910	2	25	50	GT	13	10
90424	9	2504609	2504636	2	14	28	GT	13	11
90426	9	2507600	2507626	3	9	27	CCT	19	7
90427	9	2508087	2508110	3	8	24	TCC	20	3
90428	9	2508710	2508721	2	6	12	TA	18	10
90430	9	2511654	2511674	3	7	21	GAG	22	5
90431	9	2512210	2512223	2	7	14	CA	28	4
90433	9	2525914	2525937	3	8	24	AGC	20	8
90437	9	2531518	2531541	2	12	24	AC	23	12
90438	9	2533263	2533286	3	8	24	TGA	28	6
90443	9	2535512	2535531	2	10	20	CA	13	15
90445	9	2542639	2542677	3	13	39	CCT	7	6
90449	9	2552497	2552511	3	5	15	TTC	25	8
90450	9	2552960	2552979	2	10	20	CA	15	4
90455	9	2562451	2562468	3	6	18	TTA	18	4
90456	9	2564420	2564431	2	6	12	TG	24	4
90458	9	2571341	2571364	4	6	24	TTTA	22	6
90459	9	2573035	2573064	2	15	30	GT	25	12
90461	9	2574687	2574714	2	14	28	GT	17	7
90463	9	2578364	2578399	2	18	36	TG	15	17
90465	9	2581666	2581679	2	7	14	GT	24	4
90466	9	2586348	2586375	2	14	28	TG	17	12
90467	9	2586481	2586496	4	4	16	TTTC	21	8
90468	9	2588369	2588386	2	9	18	CT	26	13
90473	9	2590544	2590555	2	6	12	TC	26	8
90474	9	2592319	2592344	2	13	26	AC	17	9
90477	9	2599772	2599803	4	8	32	CTGT	28	8
90481	9	2615167	2615181	3	5	15	TTC	22	7

90484	9	2619253	2619266	2	7	14	TG	16	12
90485	9	2620088	2620103	2	8	16	AC	23	7
90487	9	2629276	2629287	2	6	12	AC	16	4
90491	9	2645225	2645240	2	8	16	AT	20	9
90492	9	2663367	2663382	2	8	16	CA	25	8
90496	9	2672890	2672905	2	8	16	GT	15	7
90497	9	2674056	2674076	3	7	21	CCT	25	5
90500	9	2682251	2682274	4	6	24	CAAT	21	6
90507	9	2695540	2695563	2	12	24	TG	12	7
90508	9	2695722	2695753	2	16	32	GT	10	9
90511	9	2697375	2697410	2	18	36	TG	10	9
90512	9	2697491	2697510	2	10	20	CT	12	10
90514	9	2698069	2698096	2	14	28	CT	19	10
90519	9	2702538	2702552	3	5	15	CAG	28	4
90520	9	2704706	2704725	2	10	20	GT	20	6
90523	9	2706987	2707010	3	8	24	CTT	15	3
90525	9	2707831	2707860	2	15	30	AC	19	9
90526	9	2708685	2708699	3	5	15	AAC	18	3
90532	9	2715661	2715708	2	24	48	AG	14	12
90535	9	2720519	2720530	2	6	12	TA	6	3
90537	9	2727255	2727284	2	15	30	GT	15	6
90539	9	2729833	2729844	2	6	12	AC	26	4
90540	9	2729846	2729863	2	9	18	CA	18	4
90541	9	2729995	2730012	2	9	18	AC	27	6
90543	9	2731741	2731768	2	14	28	GT	13	9
90547	9	2735830	2735851	2	11	22	TG	15	9
90548	9	2735969	2736002	2	17	34	TG	13	11
90549	9	2736129	2736162	2	17	34	CA	17	17
90550	9	2743545	2743564	4	5	20	ATTT	29	5
90558	9	2763205	2763228	3	8	24	GTT	25	8
90559	9	2763687	2763706	2	10	20	AC	14	5
90560	9	2765732	2765791	2	30	60	TG	20	18
90562	9	2767025	2767046	2	11	22	TG	10	9
90568	9	2781087	2781118	2	16	32	CA	13	9
90572	9	2783037	2783048	2	6	12	TG	19	6
90573	9	2784539	2784550	2	6	12	CA	15	7
90574	9	2784612	2784629	2	9	18	AC	15	6
90579	9	2786426	2786453	2	14	28	GT	20	19
90580	9	2786754	2786769	4	4	16	ATAA	8	7
90581	9	2789747	2789772	2	13	26	GT	17	13
90585	9	2792303	2792314	2	6	12	AC	21	9
90586	9	2792457	2792478	2	11	22	TG	11	15
90587	9	2794274	2794291	2	9	18	TG	25	3
90588	9	2794321	2794360	2	20	40	AC	15	8
90591	9	2796874	2796895	2	11	22	CA	9	9
90594	9	2808049	2808062	2	7	14	TC	28	3
90597	9	2809131	2809148	2	9	18	AC	21	6
90601	9	2824787	2824813	3	9	27	CAT	26	12
90603	9	2828795	2828820	2	13	26	CA	18	9
90606	9	2829234	2829247	2	7	14	CT	22	3
90607	9	2829272	2829301	2	15	30	CA	13	12

90611	9	2834109	2834132	2	12	24	GT	18	8
90612	9	2835191	2835216	2	13	26	GT	12	11
90613	9	2835814	2835840	3	9	27	CCT	16	10
90615	9	2839989	2840016	4	7	28	AAGA	26	9
90617	9	2841620	2841639	2	10	20	AC	15	13
90618	9	2841820	2841845	2	13	26	AC	8	9
90620	9	2842256	2842271	4	4	16	TTGA	17	9
90621	9	2843902	2843941	2	20	40	TG	6	9
90622	9	2843970	2843981	2	6	12	GT	12	5
90623	9	2844008	2844029	2	11	22	GT	16	8
90627	9	2846103	2846120	3	6	18	TCC	17	7
90629	9	2851009	2851020	2	6	12	CA	29	4
90631	9	2853566	2853577	2	6	12	TA	25	8
90634	9	2858878	2858891	2	7	14	TG	21	5
90635	9	2860072	2860098	3	9	27	CTG	25	8
90638	9	2864151	2864164	2	7	14	TG	7	5
90642	9	2873434	2873451	2	9	18	GT	18	6
90643	9	2873480	2873495	2	8	16	TG	22	4
90644	9	2883685	2883699	3	5	15	TCT	21	3
90646	9	2884927	2884941	3	5	15	TCT	37	4
90647	9	2887136	2887147	2	6	12	AG	24	3
90649	9	2889507	2889536	2	15	30	AC	13	9
90650	9	2890618	2890633	2	8	16	TC	21	3
90656	9	2909015	2909026	2	6	12	TG	20	7
90659	9	2913414	2913431	2	9	18	CA	28	7
90661	9	2915892	2915915	2	12	24	AC	17	10
90663	9	2919786	2919801	2	8	16	GT	17	13
90665	9	2919913	2919924	2	6	12	AC	24	3
90666	9	2919956	2919967	2	6	12	AC	28	3
90668	9	2921346	2921373	4	7	28	CTGT	31	7
90669	9	2921665	2921692	2	14	28	GT	13	9
90672	9	2923313	2923352	2	20	40	TG	9	9
90674	9	2923510	2923533	2	12	24	TG	6	5
90675	9	2923643	2923670	2	14	28	AC	18	6
90677	9	2924756	2924779	4	6	24	TCTG	29	5
90678	9	2928189	2928245	3	19	57	CAT	21	13
90679	9	2929234	2929257	4	6	24	TCTG	27	3
90681	9	2930938	2930963	2	13	26	CT	15	15
90687	9	2938102	2938119	2	9	18	CA	21	15
90688	9	2939721	2939760	2	20	40	TG	9	6
90689	9	2939874	2939907	2	17	34	AC	13	8
90691	9	2942360	2942373	2	7	14	AG	18	8
90715	9	2958727	2958750	3	8	24	ATC	17	7
90716	9	2958802	2958819	3	6	18	TCT	11	3
90717	9	2959075	2959098	2	12	24	TC	8	7
90718	9	2959267	2959298	2	16	32	TG	16	12
90722	9	2961393	2961416	4	6	24	TTGA	10	3
90745	9	2976442	2976455	2	7	14	GT	8	10
90746	9	2976865	2976880	4	4	16	TTTA	20	3
90748	9	2982090	2982115	2	13	26	CA	21	23
90750	9	2984410	2984429	2	10	20	AC	21	11

90755	9	2994430	2994449	2	10	20	GT	16	12
90756	9	2994546	2994567	2	11	22	CA	19	11
90764	9	3006454	3006487	2	17	34	AC	14	12
90765	9	3006922	3006937	4	4	16	GAGG	7	3
90768	9	3012821	3012850	3	10	30	TAA	15	6
90769	9	3013755	3013774	2	10	20	GT	14	8
90770	9	3015632	3015651	2	10	20	CA	22	7
90771	9	3016168	3016181	2	7	14	AC	13	6
90781	9	3026633	3026646	2	7	14	TG	15	5
90785	9	3028513	3028532	2	10	20	TG	11	3
90788	9	3032287	3032312	2	13	26	AC	19	12
90789	9	3036366	3036393	4	7	28	CTAA	22	6
90791	9	3037840	3037865	2	13	26	TG	9	6
90792	9	3037908	3037927	2	10	20	TG	18	7
90794	9	3040091	3040104	2	7	14	TA	19	3
90796	9	3043063	3043084	2	11	22	GT	17	5
90797	9	3043244	3043279	4	9	36	ATCA	20	8
90804	9	3047989	3048016	2	14	28	TG	9	8
90805	9	3048189	3048216	2	14	28	TG	10	7
90806	9	3048923	3048937	3	5	15	CAT	34	4
90809	9	3050928	3050941	2	7	14	GT	13	3
90810	9	3051086	3051129	2	22	44	TG	7	10
90815	9	3057602	3057635	2	17	34	GT	17	14
90816	9	3057917	3057938	2	11	22	GT	27	10
90817	9	3058004	3058023	2	10	20	CA	23	6
90822	9	3065457	3065492	2	18	36	AC	11	5
90824	9	3065722	3065767	2	23	46	TG	7	10
90826	9	3068782	3068843	2	31	62	CA	9	9
90827	9	3068949	3068980	2	16	32	GT	13	11
90828	9	3069095	3069106	2	6	12	AC	31	4
90830	9	3069292	3069325	2	17	34	CA	12	9
90835	9	3073209	3073228	4	5	20	GACA	25	3
90836	9	3073381	3073400	4	5	20	TTAA	28	4
90838	9	3074864	3074885	2	11	22	TG	15	11
90839	9	3075035	3075054	2	10	20	TG	25	12
90840	9	3075296	3075325	3	10	30	AGG	17	8
90841	9	3080411	3080424	2	7	14	GT	8	3
90843	9	3081186	3081203	2	9	18	GA	23	5
90844	9	3082005	3082036	2	16	32	GT	21	16
90846	9	3082476	3082497	2	11	22	GA	9	8
90847	9	3082952	3082963	2	6	12	CA	19	7
90848	9	3083026	3083043	2	9	18	GT	22	9
90849	9	3085150	3085163	2	7	14	AC	26	8
90851	9	3087884	3087897	2	7	14	TG	23	8
90852	9	3089189	3089202	2	7	14	TC	23	10
90853	9	3091192	3091251	3	20	60	TCT	15	22
90855	9	3100651	3100666	2	8	16	AC	26	3
90857	9	3100934	3100949	2	8	16	AC	8	3
90868	9	3110818	3110845	2	14	28	AC	20	11
90880	9	3205739	3205759	3	7	21	TAT	22	3
90881	9	3206059	3206078	2	10	20	CA	25	16

90883	9	3211618	3211631	2	7	14	CA	13	11
90884	9	3211907	3211922	4	4	16	AACA	17	3
90885	9	3212567	3212594	2	14	28	CA	6	5
90888	9	3213612	3213639	2	14	28	CA	15	9
90889	9	3213817	3213832	2	8	16	TC	15	5
90890	9	3213902	3213925	3	8	24	TCC	11	4
90891	9	3214052	3214071	2	10	20	TG	16	15
90899	9	3220345	3220356	2	6	12	AG	27	8
90901	9	3221190	3221222	3	11	33	CCT	11	7
90902	9	3221404	3221429	2	13	26	CA	12	9
90903	9	3221591	3221624	2	17	34	AC	8	12
90904	9	3221726	3221755	2	15	30	TG	14	10
90905	9	3224721	3224747	3	9	27	CCT	18	3
90911	9	3242208	3242223	4	4	16	CTAC	28	3
90912	9	3244574	3244601	4	7	28	GTCT	31	3
90918	9	3254852	3254872	3	7	21	TTC	15	9
90924	9	3261399	3261419	3	7	21	TCT	22	12
90925	9	3263465	3263491	3	9	27	AGA	16	7
90927	9	3267103	3267132	2	15	30	AC	7	7
90933	9	3274787	3274810	2	12	24	AC	17	8
90934	9	3275072	3275095	2	12	24	GA	14	4
90938	9	3281125	3281152	4	7	28	CAAA	31	5
90940	9	3288359	3288376	3	6	18	TAT	10	3
90941	9	3290206	3290217	2	6	12	GT	28	3
90957	9	3328872	3328887	2	8	16	GT	13	5
90958	9	3329016	3329029	2	7	14	CA	8	3
90960	9	3333414	3333445	2	16	32	AC	6	4
90961	9	3333564	3333583	4	5	20	TCTT	12	6
90965	9	3334726	3334747	2	11	22	GT	7	3
90966	9	3334774	3334791	3	6	18	TGG	6	3
90970	9	3335232	3335253	2	11	22	AC	10	6
90971	9	3335397	3335416	2	10	20	CA	15	8
90972	9	3335480	3335521	2	21	42	AC	7	7
90975	9	3339628	3339647	2	10	20	GT	12	8
90984	9	3360269	3360290	2	11	22	TG	14	11
90988	9	3366647	3366685	3	13	39	TAT	9	3
90990	9	3368285	3368302	2	9	18	AC	20	7
90991	9	3371296	3371311	2	8	16	AG	18	5
90992	9	3371812	3371826	3	5	15	GGA	7	3
90999	9	3386764	3386775	2	6	12	AT	7	4
91000	9	3386777	3386794	2	9	18	TA	9	6
91004	9	3389550	3389567	2	9	18	TG	12	3
91005	9	3390025	3390040	4	4	16	TCTT	24	7
91007	9	3391235	3391250	2	8	16	AC	15	3
91008	9	3392563	3392583	3	7	21	GCT	20	5
91010	9	3394303	3394323	3	7	21	CAG	23	6
91011	9	3394832	3394849	2	9	18	AC	16	8
91012	9	3394852	3394867	2	8	16	AC	21	10
91015	9	3396941	3396956	2	8	16	GT	30	9
91019	9	3400280	3400311	2	16	32	CA	12	9
91024	9	3403421	3403446	2	13	26	CA	14	8

91027	9	3411626	3411640	3	5	15	TCT	29	3
91028	9	3411905	3411924	2	10	20	AC	14	11
91031	9	3413602	3413627	2	13	26	TC	20	5
91032	9	3414122	3414136	3	5	15	TCT	25	5
91036	9	3422321	3422340	2	10	20	AC	17	8
91037	9	3424299	3424310	2	6	12	TG	30	4
91038	9	3425953	3425978	2	13	26	TC	15	3
91039	9	3428070	3428091	2	11	22	AG	21	7
91044	9	3435536	3435555	2	10	20	AC	18	5
91050	9	3449928	3449961	2	17	34	GT	11	10
91052	9	3450619	3450634	2	8	16	GT	9	6
91055	9	3451366	3451389	2	12	24	GT	13	5
91065	9	3463562	3463589	2	14	28	GT	16	9
91070	9	3471027	3471044	2	9	18	CA	24	4
91079	9	3479989	3480024	2	18	36	AC	19	10
91081	9	3484010	3484035	2	13	26	TG	12	8
91084	9	3487921	3487932	2	6	12	AG	28	3
91086	9	3491611	3491626	4	4	16	ATTT	21	4
91108	9	3537655	3537686	2	16	32	TG	11	10
91113	9	3557517	3557536	2	10	20	GT	25	5
91115	9	3561437	3561448	2	6	12	TG	18	6
91120	9	3574785	3574804	4	5	20	ATAA	17	6
91130	9	3595166	3595179	2	7	14	TC	21	6
91135	9	3603604	3603631	2	14	28	CT	10	6
91142	9	3614184	3614197	2	7	14	AG	14	5
91143	9	3616731	3616748	3	6	18	AAC	28	4
91150	9	3639592	3639615	3	8	24	CTC	24	4
91151	9	3643738	3643759	2	11	22	AC	14	10
91152	9	3645046	3645061	2	8	16	TG	7	3
91154	9	3648753	3648778	2	13	26	GT	7	7
91160	9	3662382	3662399	2	9	18	AC	27	4
91167	9	3668115	3668128	2	7	14	TG	24	7
91168	9	3668277	3668292	2	8	16	CA	8	4
91170	9	3669092	3669105	2	7	14	CA	18	3
91171	9	3669210	3669243	2	17	34	CA	17	11
91172	9	3669618	3669633	4	4	16	TTTG	31	3
91175	9	3673929	3673943	3	5	15	AGA	30	3
91188	9	3692853	3692868	2	8	16	AC	22	6
91192	9	3696316	3696329	2	7	14	TG	30	3
91200	9	3711831	3711890	4	15	60	AGAT	16	8
91201	9	3711990	3712013	4	6	24	ATGG	33	10
91202	9	3712584	3712609	2	13	26	AC	11	9
91213	9	3733843	3733854	2	6	12	TC	24	7
91219	9	3736494	3736511	2	9	18	GT	22	4
91230	9	3767986	3768005	4	5	20	TGGA	20	5
91234	9	3770909	3770928	2	10	20	AC	23	6
91236	9	3775221	3775232	2	6	12	GT	10	3
91242	9	3789635	3789656	2	11	22	CA	11	13
91247	9	3795384	3795411	2	14	28	CA	13	7
91254	9	3802207	3802250	4	11	44	ATCT	20	10
91265	9	3816960	3816987	4	7	28	GTTG	22	3

91266	9	3823586	3823601	4	4	16	AATA	23	3
91267	9	3826367	3826386	4	5	20	TAAA	13	4
91274	9	3834810	3834835	2	13	26	CA	21	8
91280	9	3854262	3854293	2	16	32	AC	11	5
91287	9	3858679	3858690	2	6	12	GT	29	3
91288	9	3863306	3863321	2	8	16	TG	21	10
91290	9	3865132	3865155	2	12	24	GT	16	10
91293	9	3867958	3867979	2	11	22	AC	20	6
91296	9	3876402	3876429	2	14	28	AC	16	10
91309	9	3897757	3897774	3	6	18	TTC	27	3
91310	9	3898259	3898270	2	6	12	CA	15	4
91311	9	3898272	3898285	2	7	14	AC	24	5
91312	9	3898637	3898648	2	6	12	GT	29	6
91315	9	3902620	3902631	2	6	12	CA	23	3
91317	9	3906375	3906394	2	10	20	TG	23	14
91319	9	3911973	3911994	2	11	22	AC	16	4
91333	9	3940803	3940820	3	6	18	TTG	21	5
91344	9	3962737	3962750	2	7	14	GT	26	3
91350	9	3983296	3983311	2	8	16	AC	17	6
91351	9	3983313	3983326	2	7	14	CA	12	4
91360	9	3999898	3999915	3	6	18	CCA	23	6
91362	9	4002491	4002506	4	4	16	GGGA	11	3
91370	9	4005735	4005766	2	16	32	GT	20	12
91373	9	4007401	4007416	2	8	16	AC	25	5
91374	9	4008506	4008523	3	6	18	AGA	12	3
91375	9	4008580	4008607	4	7	28	ATCT	17	5
91376	9	4012641	4012654	2	7	14	CT	29	3
91378	9	4024438	4024449	2	6	12	CA	26	3
91394	9	4057547	4057558	2	6	12	CA	17	6
91402	9	4094000	4094023	2	12	24	GA	17	5
91404	9	4099127	4099142	2	8	16	TC	26	4
91405	9	4099583	4099606	2	12	24	GT	16	13
91408	9	4108980	4108995	2	8	16	TG	24	4
91409	9	4115197	4115208	2	6	12	AT	24	12
91426	9	4152242	4152257	4	4	16	TTTG	27	3
91427	9	4153065	4153076	2	6	12	CA	27	3
91428	9	4153078	4153091	2	7	14	AC	26	3
91429	9	4155138	4155161	2	12	24	GT	11	7
91430	9	4155303	4155316	2	7	14	TG	12	6
91433	9	4158861	4158896	2	18	36	TG	12	12
91434	9	4159093	4159106	2	7	14	CA	20	8
91435	9	4160610	4160639	2	15	30	TG	12	11
91437	9	4162540	4162553	2	7	14	AG	17	4
91439	9	4165896	4165919	2	12	24	GT	14	9
91441	9	4168377	4168404	2	14	28	CA	8	11
91442	9	4168764	4168789	2	13	26	GT	17	12
91443	9	4168905	4168931	3	9	27	GCT	26	6
91445	9	4171578	4171593	4	4	16	AAGA	14	4
91446	9	4173835	4173868	2	17	34	TG	16	12
91449	9	4177859	4177876	2	9	18	GT	12	6
91451	9	4178262	4178279	2	9	18	AC	11	6

91457	9	4183189	4183212	2	12	24	GT	16	5
91468	9	4207931	4207946	2	8	16	AC	16	9
91471	9	4211313	4211328	4	4	16	ATTT	38	3
91472	9	4216349	4216370	2	11	22	TG	16	7
91482	9	4233914	4233935	2	11	22	GT	10	11
91486	9	4238969	4238986	2	9	18	CA	27	7
91488	9	4245096	4245111	2	8	16	AC	23	4
91489	9	4258112	4258123	2	6	12	CT	8	3
91494	9	4271056	4271081	2	13	26	GT	13	13
91495	9	4275012	4275065	2	27	54	AG	13	14
91497	9	4275431	4275448	3	6	18	TTG	16	10
91501	9	4281630	4281653	2	12	24	CA	18	7
91503	9	4297659	4297676	2	9	18	AT	10	6
91504	9	4298980	4299018	3	13	39	TTA	8	6
91505	9	4299605	4299620	4	4	16	TTTA	8	4
91506	9	4299801	4299820	4	5	20	AATA	7	3
91510	9	4307942	4307953	2	6	12	TG	22	9
91511	9	4307956	4307967	2	6	12	TG	19	6
91513	9	4320027	4320040	2	7	14	TG	10	4
91514	9	4320044	4320057	2	7	14	GT	20	9
91515	9	4324090	4324119	2	15	30	CA	22	9
91520	9	4341160	4341177	2	9	18	TG	6	6
91521	9	4341772	4341799	4	7	28	TCCT	24	4
91533	9	4376076	4376089	2	7	14	AC	20	3
91535	9	4377046	4377059	2	7	14	GT	22	11
91536	9	4377314	4377331	3	6	18	AGA	16	7
91539	9	4378872	4378885	2	7	14	TG	34	3
91540	9	4381068	4381083	2	8	16	GT	20	4
91541	9	4381692	4381715	4	6	24	TATG	28	4
91545	9	4390036	4390047	2	6	12	GT	25	3
91546	9	4393249	4393260	2	6	12	CA	10	4
91553	9	4417886	4417909	4	6	24	AACA	24	4
91558	9	4422818	4422833	4	4	16	AATA	11	3
91562	9	4425132	4425151	2	10	20	AC	26	8
91563	9	4427414	4427429	2	8	16	TC	21	9
91566	9	4434865	4434884	2	10	20	CT	19	7
91575	9	4457494	4457508	3	5	15	GAG	25	5
91587	9	4480742	4480781	4	10	40	GCAG	24	7
91589	9	4484723	4484758	4	9	36	AACC	16	7
91590	9	4485595	4485610	2	8	16	TG	14	8
91592	9	4488466	4488481	2	8	16	GT	26	5
91596	9	4491555	4491566	2	6	12	TG	28	3
91598	9	4492108	4492137	2	15	30	GA	9	7
91600	9	4493139	4493164	2	13	26	GT	11	6
91601	9	4493336	4493355	2	10	20	GA	18	5
91602	9	4495443	4495466	2	12	24	TG	19	7
91603	9	4495656	4495673	2	9	18	TC	24	4
91608	9	4499549	4499569	3	7	21	TTC	16	3
91615	9	4513052	4513067	2	8	16	AC	21	4
91619	9	4515317	4515338	2	11	22	CA	16	10
91624	9	4522287	4522301	3	5	15	TGA	27	4

91626	9	4523614	4523631	3	6	18	AGG	25	3
91628	9	4525401	4525424	2	12	24	CA	26	7
91636	9	4531864	4531877	2	7	14	TC	28	3
91657	9	4567344	4567361	2	9	18	TG	26	3
91662	9	4584462	4584487	2	13	26	TC	21	7
91665	9	4591513	4591530	2	9	18	TG	28	5
91670	9	4593146	4593169	2	12	24	GT	11	6
91671	9	4593322	4593337	2	8	16	AC	21	4
91676	9	4598468	4598493	2	13	26	AC	6	3
91677	9	4600288	4600303	2	8	16	AG	29	3
91678	9	4600713	4600730	3	6	18	CTT	35	7
91679	9	4602084	4602101	3	6	18	CTC	7	4
91680	9	4602273	4602292	2	10	20	TG	22	8
91692	9	4612989	4613006	2	9	18	GT	27	6
91693	9	4613196	4613217	2	11	22	TG	29	6
91695	9	4622595	4622622	2	14	28	CA	15	15
91714	9	4650295	4650306	2	6	12	AG	25	3
91715	9	4651127	4651141	3	5	15	GAT	25	4
91720	9	4655010	4655023	2	7	14	GT	11	6
91723	9	4670855	4670870	4	4	16	ATGG	27	3
91727	9	4679663	4679680	2	9	18	CT	24	4
91728	9	4680036	4680047	2	6	12	TC	12	5
91738	9	4693771	4693788	2	9	18	CA	13	4
91743	9	4703545	4703570	2	13	26	CA	16	8
91744	9	4703984	4703999	2	8	16	AG	14	3
91745	9	4705198	4705215	2	9	18	AC	28	9
91746	9	4705391	4705408	3	6	18	GTG	11	6
91747	9	4705789	4705806	3	6	18	CTG	27	4
91750	9	4707799	4707812	2	7	14	CA	27	3
91751	9	4710672	4710687	2	8	16	TG	19	7
91755	9	4716984	4717005	2	11	22	TC	14	5
91756	9	4717153	4717166	2	7	14	TC	15	7
91763	9	4729569	4729580	2	6	12	GT	16	7
91764	9	4729583	4729594	2	6	12	GT	16	8
91765	9	4729772	4729786	3	5	15	AAG	25	3
91766	9	4732064	4732085	2	11	22	GT	7	9
91771	9	4741055	4741082	2	14	28	TC	17	11
91772	9	4742027	4742046	2	10	20	AC	16	7
91780	9	4753563	4753578	2	8	16	CA	13	6
91781	9	4753580	4753595	2	8	16	AC	15	7
91783	9	4755043	4755066	2	12	24	AC	18	5
91785	9	4755666	4755679	2	7	14	CA	10	6
91786	9	4755692	4755705	2	7	14	CA	13	6
91787	9	4756358	4756377	2	10	20	AG	13	6
91788	9	4758299	4758318	4	5	20	CCAT	29	3
91790	9	4761234	4761249	2	8	16	TG	16	4
91796	9	4763309	4763354	2	23	46	TC	11	12
91799	9	4767924	4767947	2	12	24	AC	12	6
91800	9	4769694	4769709	2	8	16	AC	9	9
91803	9	4770206	4770235	2	15	30	CA	10	7
91810	9	4775493	4775508	4	4	16	TTAT	22	6

91813	9	4782256	4782267	2	6	12	AT	18	4
91820	9	4788141	4788172	2	16	32	AC	17	8
91823	9	4793609	4793628	4	5	20	TGTT	25	3
91827	9	4805453	4805468	2	8	16	CA	24	7
91828	9	4805657	4805688	2	16	32	GT	13	10
91829	9	4811679	4811710	4	8	32	ATGG	32	8
91836	9	4821369	4821384	2	8	16	CA	21	5
91837	9	4822228	4822247	2	10	20	CA	13	13
91838	9	4822396	4822449	2	27	54	GA	8	11
91840	9	4822831	4822846	2	8	16	GT	28	7
91843	9	4828248	4828265	2	9	18	TC	23	4
91844	9	4828362	4828375	2	7	14	AC	21	11
91854	9	4856029	4856060	2	16	32	AC	12	6
91858	9	4864087	4864104	2	9	18	GA	22	5
91860	9	4869371	4869388	2	9	18	AC	22	4
91865	9	4879996	4880009	2	7	14	AC	30	4
91867	9	4883910	4883937	2	14	28	TG	16	8
91872	9	4888324	4888343	4	5	20	AATA	15	5
91875	9	4889274	4889285	2	6	12	TG	20	5
91881	9	4897181	4897212	2	16	32	AC	7	7
91884	9	4900960	4900977	3	6	18	TTC	18	3
91885	9	4903438	4903469	4	8	32	CATC	25	5
91891	9	4911196	4911222	3	9	27	TCA	20	3
91892	9	4911612	4911639	2	14	28	CA	13	10
91894	9	4920925	4920942	3	6	18	CTG	32	5
91899	9	4924813	4924840	2	14	28	TG	18	13
91914	9	4956775	4956790	2	8	16	CT	25	6
91916	9	4956987	4957001	3	5	15	GAG	12	3
91919	9	4962179	4962192	2	7	14	AG	31	5
91920	9	4966721	4966744	3	8	24	GAG	23	8
91922	9	4970222	4970237	4	4	16	AAGA	28	4
91927	9	4986664	4986699	2	18	36	AC	12	12
91928	9	4990485	4990526	3	14	42	GAA	17	13
91929	9	4990654	4990675	2	11	22	GT	20	7
91932	9	4994334	4994353	4	5	20	CAGG	19	3
91933	9	4996521	4996532	2	6	12	TG	19	8
91939	9	5007150	5007165	2	8	16	TC	27	3
91942	9	5015652	5015669	2	9	18	GT	19	3
91944	9	5016753	5016764	2	6	12	AC	21	6
91945	9	5018933	5018944	2	6	12	AC	33	3
91948	9	5020169	5020192	2	12	24	TG	14	12
91952	9	5032369	5032398	3	10	30	ATA	14	8
91954	9	5035052	5035081	2	15	30	TG	9	10
91961	9	5050761	5050787	3	9	27	ACA	16	4
91963	9	5051223	5051249	3	9	27	GCT	28	7
91973	9	5108801	5108814	2	7	14	CT	23	3
91976	9	5118140	5118154	3	5	15	ATT	30	4
91977	9	5118414	5118427	2	7	14	TG	13	5
91978	9	5121603	5121647	3	15	45	AGC	26	9
91985	9	5142368	5142387	2	10	20	TC	22	7
91993	9	5147955	5147969	3	5	15	GGT	9	3

91995	9	5150419	5150456	2	19	38	GT	7	12
92005	9	5173134	5173149	2	8	16	TC	20	4
92008	9	5184179	5184193	3	5	15	ATA	19	4
92009	9	5184194	5184208	3	5	15	AGA	18	11
92010	9	5185689	5185728	2	20	40	AC	11	13
92020	9	5193109	5193132	4	6	24	AAAT	13	7
92031	9	5238189	5238230	2	21	42	CA	6	7
92033	9	5240963	5240986	2	12	24	TG	18	13
92036	9	5246674	5246691	2	9	18	CT	13	4
92041	9	5252367	5252392	2	13	26	TG	16	9
92052	9	5277816	5277831	2	8	16	AC	16	5
92060	9	5293894	5293914	3	7	21	CTG	24	5
92064	9	5297664	5297675	2	6	12	AT	15	4
92072	9	5320118	5320129	2	6	12	AC	19	7
92077	9	5325064	5325078	3	5	15	CCA	19	8
92084	9	5326660	5326677	3	6	18	GAG	13	3
92086	9	5328459	5328474	2	8	16	TG	16	5
92088	9	5333387	5333406	2	10	20	GT	13	3
92089	9	5335073	5335116	4	11	44	AGAT	18	7
92102	9	5373864	5373881	3	6	18	GCT	24	4
92114	9	5391998	5392011	2	7	14	TA	15	3
92120	9	5410416	5410430	3	5	15	CTT	27	3
92123	9	5418762	5418775	2	7	14	GT	17	4
92140	9	5489486	5489501	4	4	16	AACA	28	4
92164	9	5579262	5579281	2	10	20	GT	23	6
92165	9	5579602	5579613	2	6	12	TA	30	3
92168	9	5586714	5586731	3	6	18	CTG	21	3
92171	9	5602505	5602536	2	16	32	TG	24	10
92173	9	5608254	5608269	4	4	16	TTTG	27	4
92190	9	5671349	5671369	3	7	21	CTG	26	8
92192	9	5677325	5677368	2	22	44	CA	11	7
92202	9	5693071	5693122	2	26	52	TG	15	14
92207	9	5705181	5705204	2	12	24	TG	18	7
92208	9	5705327	5705346	2	10	20	CA	26	5
92213	9	5711034	5711061	2	14	28	CT	20	5
92221	9	5721323	5721342	2	10	20	AC	22	9
92223	9	5723515	5723528	2	7	14	GT	35	3
92233	9	5733665	5733712	3	16	48	TGC	26	9
92239	9	5746909	5746923	3	5	15	GAG	23	5
92243	9	5750954	5750969	2	8	16	TA	13	6
92248	9	5763845	5763859	3	5	15	TTC	26	3
92254	9	5780386	5780405	4	5	20	AAAT	18	6
92260	9	5787254	5787267	2	7	14	CT	7	4
92261	9	5787319	5787346	2	14	28	AC	16	9
92271	9	5814146	5814159	2	7	14	AC	8	4
92281	9	5833802	5833821	2	10	20	AC	15	7
92283	9	5836666	5836689	2	12	24	AC	9	4
92286	9	5839706	5839719	2	7	14	CA	19	4
92296	9	5854842	5854855	2	7	14	CA	20	3
92297	9	5856071	5856094	2	12	24	GT	17	5
92298	9	5867314	5867343	3	10	30	GGA	7	8

92307	9	5881081	5881094	2	7	14	GT	29	3
92312	9	5891499	5891526	2	14	28	AC	10	8
92326	9	5920265	5920284	2	10	20	CT	15	7
92328	9	5920857	5920882	2	13	26	GT	17	7
92330	9	5920989	5921012	2	12	24	AC	6	6
92334	9	5925197	5925226	2	15	30	TG	16	5
92339	9	5936494	5936509	2	8	16	AC	13	6
92341	9	5937078	5937089	2	6	12	CA	19	6
92342	9	5938143	5938154	2	6	12	AC	24	4
92343	9	5938226	5938251	2	13	26	CA	17	9
92344	9	5940397	5940432	4	9	36	AGAT	17	5
92345	9	5940735	5940760	2	13	26	TG	16	12
92348	9	5941967	5941980	2	7	14	GT	19	5
92351	9	5955205	5955220	2	8	16	AC	26	8
92355	9	5964737	5964752	2	8	16	GA	17	6
92360	9	5983973	5983993	3	7	21	CAA	20	4
92367	9	6009655	6009672	2	9	18	GA	9	3
92373	9	6027966	6027983	2	9	18	AC	28	4
92375	9	6030904	6030925	2	11	22	GT	20	6
92376	9	6032803	6032820	2	9	18	AC	15	5
92378	9	6033447	6033470	3	8	24	TCC	23	5
92380	9	6035304	6035319	2	8	16	TC	19	3
92386	9	6037632	6037643	2	6	12	GT	24	4
92388	9	6038533	6038548	2	8	16	AT	11	3
92389	9	6041344	6041370	3	9	27	TGC	23	10
92391	9	6041915	6041944	3	10	30	GGA	17	5
92393	9	6042208	6042219	2	6	12	TG	19	8
92396	9	6048561	6048586	2	13	26	AC	18	9
92397	9	6050088	6050120	3	11	33	ATC	25	7
92398	9	6055072	6055083	2	6	12	CA	27	5
92399	9	6055110	6055135	2	13	26	AC	26	9
92406	9	6066341	6066354	2	7	14	CA	24	4
92408	9	6070731	6070748	3	6	18	GAG	15	3
92411	9	6075423	6075438	2	8	16	AC	8	7
92412	9	6075440	6075459	2	10	20	CA	12	10
92420	9	6088316	6088345	2	15	30	CA	15	5
92422	9	6095633	6095653	3	7	21	CAA	27	4
92423	9	6096976	6096989	2	7	14	AT	17	4
92426	9	6108837	6108876	4	10	40	GGAT	17	3
92442	9	6186989	6187034	2	23	46	GT	9	11
92443	9	6188082	6188143	2	31	62	AC	18	16
92445	9	6197614	6197633	4	5	20	CCAT	27	8
92456	9	6213620	6213647	2	14	28	TG	14	11
92458	9	6221805	6221816	2	6	12	TG	17	3
92474	9	6253338	6253353	2	8	16	TA	24	7
92475	9	6257200	6257213	2	7	14	TG	8	3
92479	9	6262689	6262702	2	7	14	TG	22	8
92480	9	6266420	6266435	2	8	16	AC	24	4
92481	9	6266614	6266637	3	8	24	GAG	29	4
92484	9	6277679	6277696	3	6	18	GAA	18	4
92485	9	6280772	6280783	2	6	12	AC	23	3

92500	9	6326636	6326650	3	5	15	TAA	23	3
92511	9	6353569	6353586	2	9	18	CT	27	5
92512	9	6354709	6354723	3	5	15	AAT	29	3
92513	9	6356484	6356501	2	9	18	AC	8	7
92517	9	6372922	6372948	3	9	27	TGC	20	6
92519	9	6382719	6382734	4	4	16	AAAT	14	6
92521	9	6382873	6382910	2	19	38	GT	15	9
92525	9	6386011	6386032	2	11	22	CA	17	8
92527	9	6387785	6387812	2	14	28	CA	8	6
92529	9	6389598	6389637	2	20	40	AC	10	7
92533	9	6396360	6396373	2	7	14	GT	27	6
92542	9	6414169	6414188	4	5	20	TATT	18	3
92543	9	6415910	6415927	2	9	18	AC	21	5
92544	9	6416239	6416256	3	6	18	TCC	23	8
92545	9	6417144	6417159	4	4	16	TTTG	18	5
92547	9	6418224	6418267	4	11	44	TAGA	13	11
92549	9	6418574	6418597	3	8	24	CTG	24	7
92550	9	6418718	6418751	2	17	34	AC	12	8
92553	9	6422013	6422024	2	6	12	CA	29	3
92561	9	6429484	6429498	3	5	15	CCT	20	4
92568	9	6439056	6439069	2	7	14	CA	30	3
92570	9	6442032	6442067	2	18	36	TC	14	5
92571	9	6443751	6443776	2	13	26	TG	24	7
92574	9	6461384	6461401	3	6	18	AAT	9	3
92577	9	6462260	6462275	2	8	16	GT	19	5
92578	9	6462779	6462802	3	8	24	TTG	16	15
92582	9	6470677	6470691	3	5	15	GAA	24	3
92584	9	6476167	6476184	3	6	18	CCT	26	5
92602	9	6510061	6510074	2	7	14	TG	23	10
92608	9	6520133	6520150	3	6	18	CCT	22	5
92615	9	6537220	6537243	3	8	24	GAG	19	8
92619	9	6549854	6549883	2	15	30	AC	19	10
92620	9	6550050	6550097	2	24	48	TG	14	13
92622	9	6556273	6556290	2	9	18	GA	19	3
92623	9	6558652	6558669	2	9	18	AT	8	5
92624	9	6559346	6559361	2	8	16	AC	17	3
92630	9	6572063	6572078	2	8	16	AC	15	13
92631	9	6572080	6572099	2	10	20	CA	17	13
92639	9	6582231	6582248	2	9	18	GT	21	7
92643	9	6583260	6583283	2	12	24	TG	16	9
92653	9	6595093	6595138	2	23	46	GT	7	8
92660	9	6605673	6605688	2	8	16	GT	16	4
92666	9	6612182	6612225	4	11	44	GGAT	24	8
92674	9	6617319	6617364	2	23	46	GT	6	9
92675	9	6617569	6617588	2	10	20	CA	18	12
92681	9	6623318	6623359	2	21	42	AG	9	5
92683	9	6628708	6628725	2	9	18	TG	24	6
92686	9	6629169	6629189	3	7	21	GAG	24	3
92688	9	6638401	6638426	2	13	26	CA	14	5
92693	9	6639054	6639077	2	12	24	TG	12	7
92700	9	6651881	6651896	2	8	16	GT	22	6

92705	9	6659985	6660000	4	4	16	CACT	22	3
92707	9	6660077	6660092	2	8	16	CA	17	7
92711	9	6665598	6665621	4	6	24	GATG	25	4
92715	9	6672629	6672648	2	10	20	AC	23	3
92716	9	6673968	6673985	3	6	18	ATA	9	4
92730	9	6719472	6719493	2	11	22	CA	25	9
92738	9	6727173	6727190	2	9	18	AC	12	3
92741	9	6727412	6727427	2	8	16	AC	9	6
92746	9	6730051	6730072	2	11	22	AC	14	9
92747	9	6730225	6730236	2	6	12	CA	19	4
92757	9	6744056	6744067	2	6	12	AC	18	12
92758	9	6744068	6744085	2	9	18	GC	14	12
92769	9	6769079	6769098	4	5	20	AAAT	21	3
92770	9	6769271	6769292	2	11	22	TC	23	6
92771	9	6774583	6774596	2	7	14	TG	22	12
92773	9	6776742	6776759	2	9	18	TC	12	14
92774	9	6776760	6776773	2	7	14	AC	14	17
92778	9	6778756	6778769	2	7	14	CT	18	4
92782	9	6787957	6787974	2	9	18	TC	15	5
92784	9	6790770	6790799	2	15	30	AC	11	13
92789	9	6801345	6801370	2	13	26	GT	10	9
92790	9	6801558	6801573	2	8	16	CA	28	7
92791	9	6805370	6805397	2	14	28	CA	7	4
92792	9	6807657	6807684	2	14	28	CA	14	9
92793	9	6807979	6807996	2	9	18	AC	24	7
92794	9	6810144	6810177	2	17	34	GT	17	13
92796	9	6811493	6811519	3	9	27	GAG	25	6
92801	9	6816701	6816726	2	13	26	CA	20	9
92809	9	6826591	6826630	2	20	40	GT	14	8
92811	9	6830371	6830386	2	8	16	GT	12	9
92812	9	6830594	6830609	2	8	16	GT	18	6
92813	9	6830713	6830736	2	12	24	TG	16	8
92814	9	6833263	6833274	2	6	12	TG	32	3
92815	9	6834633	6834648	2	8	16	CA	16	9
92816	9	6834681	6834714	2	17	34	TC	18	8
92818	9	6834802	6834813	2	6	12	CA	27	3
92820	9	6834923	6834938	2	8	16	AG	26	9
92822	9	6840618	6840633	2	8	16	TG	20	8
92823	9	6842785	6842802	2	9	18	AC	12	7
92824	9	6843207	6843226	2	10	20	TG	17	7
92843	9	6883113	6883124	2	6	12	CT	20	5
92850	9	6902793	6902812	2	10	20	AC	14	7
92851	9	6912387	6912398	2	6	12	CT	20	3
92858	9	6920148	6920161	2	7	14	TG	29	6
92862	9	6929254	6929279	2	13	26	AC	15	8
92864	9	6944505	6944518	2	7	14	GT	22	13
92867	9	6950688	6950711	2	12	24	TC	21	12
92879	9	6977140	6977157	2	9	18	AC	24	5
92881	9	6978739	6978752	2	7	14	TC	11	4
92882	9	6978756	6978771	2	8	16	CT	8	5
92885	9	6979272	6979287	2	8	16	CA	16	4

92886	9	6979464	6979487	2	12	24	TG	17	7
92888	9	6982335	6982350	2	8	16	TC	29	12
92889	9	6987638	6987651	2	7	14	GA	21	3
92890	9	6987966	6987993	2	14	28	AG	25	6
92899	9	7017635	7017656	2	11	22	CA	13	4
92900	9	7018450	7018463	2	7	14	TC	23	5
92901	9	7024820	7024849	2	15	30	AC	14	8
92903	9	7031875	7031886	2	6	12	CA	23	4
92905	9	7035086	7035099	2	7	14	CT	27	9
92907	9	7039400	7039438	3	13	39	AGG	14	8
92912	9	7065377	7065408	2	16	32	GT	10	10
92915	9	7067264	7067287	2	12	24	AC	8	7
92919	9	7088767	7088800	2	17	34	TG	15	11
92921	9	7097209	7097220	2	6	12	CA	20	3
92922	9	7097341	7097360	2	10	20	AC	16	4
92923	9	7097799	7097822	2	12	24	TG	22	8
92927	9	7105838	7105853	2	8	16	AG	25	3
92929	9	7107728	7107777	2	25	50	AC	9	8
92931	9	7108749	7108772	2	12	24	CA	8	5
92935	9	7127648	7127669	2	11	22	CA	23	10
92939	9	7131458	7131483	2	13	26	CT	9	5
92946	9	7148571	7148594	2	12	24	CA	14	6
92959	9	7190298	7190333	2	18	36	GT	12	11
92961	9	7195753	7195778	2	13	26	TG	14	11
92963	9	7199051	7199066	2	8	16	AC	19	3
92970	9	7215542	7215561	4	5	20	AAGG	17	3
92974	9	7216999	7217014	2	8	16	CA	17	7
92983	9	7233787	7233818	2	16	32	TC	11	4
92984	9	7238853	7238896	2	22	44	AC	11	12
92987	9	7248757	7248771	3	5	15	AAT	25	4
92991	9	7262307	7262322	2	8	16	CA	24	4
92994	9	7268089	7268110	2	11	22	GT	13	8
92995	9	7268756	7268775	4	5	20	AAAC	26	4
93004	9	7282455	7282490	2	18	36	AC	16	14
93011	9	7285170	7285193	3	8	24	AGG	23	3
93012	9	7285481	7285498	2	9	18	GA	15	5
93017	9	7287221	7287236	4	4	16	AAAT	21	4
93019	9	7287502	7287519	3	6	18	GAA	20	6
93020	9	7287688	7287717	3	10	30	TCC	14	4
93021	9	7287753	7287782	2	15	30	TG	12	4
93022	9	7287829	7287879	3	17	51	TTC	14	16
93023	9	7288119	7288156	2	19	38	GT	18	15
93027	9	7302587	7302610	3	8	24	AAG	21	5
93028	9	7311462	7311489	2	14	28	AC	14	5
93029	9	7314050	7314064	3	5	15	GTG	11	4
93037	9	7320710	7320727	2	9	18	TG	20	5
93043	9	7335714	7335728	3	5	15	CAG	27	4
93047	9	7344379	7344396	2	9	18	GT	26	4
93049	9	7346726	7346745	2	10	20	AC	22	3
93057	9	7354307	7354338	2	16	32	AG	21	8
93058	9	7355027	7355050	4	6	24	TTTC	12	5

93080	9	7372434	7372448	3	5	15	CCT	12	4
93081	9	7374815	7374838	3	8	24	AGG	22	8
93083	9	7376203	7376214	2	6	12	AT	16	5
93090	9	7382319	7382346	2	14	28	TG	13	6
93093	9	7382563	7382578	2	8	16	AG	17	3
93094	9	7383024	7383037	2	7	14	GT	16	5
93100	9	7389249	7389284	2	18	36	AC	8	13
93101	9	7389364	7389377	2	7	14	AC	30	3
93102	9	7389759	7389778	2	10	20	CA	24	7
93103	9	7391678	7391719	2	21	42	TG	24	12
93109	9	7398688	7398703	2	8	16	GT	28	5
93117	9	7402653	7402702	2	25	50	CA	16	12
93119	9	7404237	7404284	2	24	48	AC	16	11
93120	9	7404780	7404803	2	12	24	GT	19	16
93122	9	7405502	7405519	2	9	18	GA	11	4
93124	9	7405656	7405679	2	12	24	GT	20	5
93129	9	7411871	7411908	2	19	38	CA	12	10
93130	9	7412086	7412103	2	9	18	GT	19	6
93132	9	7413312	7413329	2	9	18	GA	21	7
93134	9	7415358	7415381	2	12	24	GA	10	10
93139	9	7423081	7423116	2	18	36	AC	11	9
93144	9	7431018	7431037	2	10	20	CT	17	6
93145	9	7431039	7431050	2	6	12	TC	25	8
93149	9	7432117	7432146	2	15	30	AC	18	14
93150	9	7433806	7433819	2	7	14	GA	22	5
93155	9	7435326	7435347	2	11	22	GT	12	6
93177	9	7467859	7467876	2	9	18	AG	28	4
93179	9	7473024	7473065	2	21	42	TG	12	13
93181	9	7474133	7474178	2	23	46	CA	8	5
93188	9	7483697	7483712	4	4	16	TTTA	22	6
93189	9	7485312	7485331	4	5	20	CAAT	24	5
93191	9	7486337	7486354	2	9	18	AC	20	5
93198	9	7493656	7493691	2	18	36	AC	11	16
93200	9	7498501	7498522	2	11	22	CA	22	8
93202	9	7502646	7502667	2	11	22	AG	20	9
93212	9	7523412	7523427	4	4	16	ACCA	25	3
93223	9	7549569	7549620	2	26	52	CA	6	7
93235	9	7573211	7573230	2	10	20	GT	14	9
93238	9	7580401	7580428	2	14	28	GA	9	4
93245	9	7592267	7592280	2	7	14	TG	20	13
93246	9	7592282	7592301	2	10	20	GT	17	13
93248	9	7594238	7594261	2	12	24	GT	9	10
93252	9	7599370	7599385	2	8	16	AT	7	3
93253	9	7601910	7601927	2	9	18	CA	7	8
93257	9	7612411	7612464	2	27	54	TC	12	11
93258	9	7612724	7612737	2	7	14	GA	18	5
93261	9	7617019	7617044	2	13	26	GT	17	7
93263	9	7621346	7621359	2	7	14	GA	23	4
93273	9	7641094	7641127	2	17	34	AC	11	7
93276	9	7644705	7644718	2	7	14	AC	28	11
93287	9	7655922	7655935	2	7	14	AG	10	3

93294	9	7663468	7663497	2	15	30	CT	16	8
93295	9	7664233	7664264	2	16	32	AC	21	11
93304	9	7669155	7669170	2	8	16	AC	21	3
93308	9	7673037	7673050	2	7	14	GA	14	5
93313	9	7677589	7677612	3	8	24	AAT	12	6
93314	9	7681699	7681720	2	11	22	AC	13	8
93316	9	7685370	7685383	2	7	14	CA	25	3
93322	9	7694977	7695008	2	16	32	CA	19	9
93323	9	7695822	7695854	3	11	33	GTA	24	12
93328	9	7700695	7700708	2	7	14	AC	6	6
93334	9	7717582	7717613	2	16	32	CT	7	8
93336	9	7718116	7718145	2	15	30	TG	19	5
93344	9	7723965	7724000	4	9	36	AAGA	25	9
93346	9	7725706	7725735	2	15	30	AG	9	11
93348	9	7726901	7726928	2	14	28	CA	19	6
93350	9	7731870	7731899	2	15	30	CA	8	5
93352	9	7734720	7734737	3	6	18	AGG	30	6
93353	9	7735297	7735316	2	10	20	AG	16	5
93355	9	7736278	7736289	2	6	12	GT	21	3
93356	9	7736317	7736348	2	16	32	GT	13	8
93359	9	7741685	7741696	2	6	12	GT	23	14
93365	9	7742507	7742548	2	21	42	GT	6	9
93370	9	7744842	7744861	2	10	20	CA	18	6
93378	9	7758212	7758235	2	12	24	GT	17	6
93379	9	7760070	7760103	2	17	34	TG	9	10
93382	9	7763022	7763033	2	6	12	AC	21	3
93385	9	7766607	7766646	2	20	40	CA	6	8
93387	9	7772527	7772562	4	9	36	TAGA	27	11
93388	9	7773051	7773066	2	8	16	CA	25	3
93392	9	7776015	7776032	2	9	18	AG	20	4
93393	9	7777148	7777161	2	7	14	GT	25	3
93402	9	7790236	7790250	3	5	15	TCA	29	3
93403	9	7790833	7790856	2	12	24	AG	13	6
93406	9	7792051	7792070	2	10	20	TC	17	7
93410	9	7806586	7806609	4	6	24	ATAG	27	8
93411	9	7806674	7806689	2	8	16	CT	28	4
93412	9	7807354	7807369	2	8	16	TG	17	9
93413	9	7807801	7807822	2	11	22	AC	22	11
93414	9	7808048	7808061	2	7	14	GA	28	8
93419	9	7819250	7819283	2	17	34	TG	7	9
93431	9	7830454	7830475	2	11	22	TG	17	7
93433	9	7832999	7833034	2	18	36	TG	6	6
93435	9	7833585	7833602	2	9	18	GT	11	5
93437	9	7838106	7838119	2	7	14	GT	32	5
93438	9	7848404	7848429	2	13	26	AC	17	9
93446	9	7859966	7859977	2	6	12	TG	25	6
93451	9	7867904	7867925	2	11	22	AC	30	9
93452	9	7869526	7869540	3	5	15	TTG	25	3
93453	9	7870216	7870239	2	12	24	CA	10	8
93458	9	7873974	7873988	3	5	15	CAG	25	3
93459	9	7874265	7874292	2	14	28	GT	19	8

93460	9	7874431	7874448	2	9	18	CA	15	7
93461	9	7874477	7874506	2	15	30	AC	13	5
93463	9	7874617	7874634	2	9	18	AC	14	4
93464	9	7875143	7875158	4	4	16	TCTT	21	4
93467	9	7880774	7880791	2	9	18	CT	26	4
93471	9	7893457	7893476	2	10	20	TG	20	5
93474	9	7896422	7896439	2	9	18	TC	23	3
93478	9	7900296	7900309	2	7	14	AC	23	6
93479	9	7900312	7900323	2	6	12	AC	20	5
93481	9	7900572	7900589	2	9	18	TG	29	9
93485	9	7903056	7903069	2	7	14	TA	15	6
93487	9	7905524	7905543	2	10	20	CA	27	5
93490	9	7909648	7909675	2	14	28	CA	13	12
93492	9	7910351	7910374	2	12	24	TG	18	14
93496	9	7912519	7912534	2	8	16	TG	29	5
93509	9	7926525	7926548	2	12	24	CT	16	10
93510	9	7929806	7929825	4	5	20	CAGG	31	3
93511	9	7931707	7931736	2	15	30	CA	11	13
93512	9	7931849	7931868	2	10	20	AC	24	15
93513	9	7932135	7932160	2	13	26	CA	12	8
93515	9	7932359	7932374	2	8	16	TG	29	6
93516	9	7932448	7932471	4	6	24	GCCT	34	3
93517	9	7935237	7935272	2	18	36	AC	10	14
93524	9	7940855	7940868	2	7	14	AC	15	3
93526	9	7941076	7941105	2	15	30	CA	17	7
93528	9	7943372	7943389	2	9	18	AC	27	4
93531	9	7947213	7947238	2	13	26	CA	6	6
93532	9	7947378	7947393	2	8	16	GT	21	7
93533	9	7949336	7949349	2	7	14	CA	27	5
93538	9	7953501	7953512	2	6	12	TG	29	3
93544	9	7967550	7967581	2	16	32	TG	14	14
93545	9	7967663	7967684	2	11	22	CA	24	7
93555	9	7974305	7974330	2	13	26	CA	8	7
93561	9	7987632	7987649	3	6	18	CAA	27	3
93563	9	7992975	7992986	2	6	12	AG	23	4
93565	9	7996997	7997032	2	18	36	TG	9	7
93569	9	7998628	7998655	2	14	28	GA	11	12
93572	9	8012933	8012960	2	14	28	TC	19	6
93578	9	8020696	8020717	2	11	22	GT	15	9
93582	9	8024099	8024112	2	7	14	AG	19	3
93583	9	8024192	8024221	2	15	30	TC	14	5
93584	9	8024264	8024287	2	12	24	GA	25	5
93592	9	8031062	8031073	2	6	12	CT	21	5
93611	9	8043266	8043283	2	9	18	TG	17	6
93620	9	8048749	8048766	3	6	18	CTT	33	3
93621	9	8049286	8049299	2	7	14	CA	26	4
93622	9	8050644	8050663	2	10	20	TG	18	5
93627	9	8055889	8055900	2	6	12	GA	23	4
93646	9	8074570	8074613	2	22	44	TC	7	8
93654	9	8078946	8078957	2	6	12	CT	28	3
93656	9	8080766	8080791	2	13	26	TG	20	14

93659	9	8082987	8083010	2	12	24	GA	9	8
93666	9	8087462	8087473	2	6	12	GA	24	3
93667	9	8089112	8089131	4	5	20	TATC	13	3
93670	9	8091998	8092009	2	6	12	GA	32	3
93671	9	8092017	8092028	2	6	12	AG	24	4
93684	9	8097942	8097971	2	15	30	AG	16	14
93685	9	8098189	8098210	2	11	22	AG	22	3
93686	9	8098320	8098343	2	12	24	TG	13	7
93691	9	8099374	8099397	2	12	24	AC	21	11
93696	9	8104590	8104609	4	5	20	TTAT	14	3
93699	9	8105274	8105297	2	12	24	AG	15	5
93705	9	8110057	8110090	2	17	34	TG	15	9
93714	9	8121790	8121801	2	6	12	GT	20	3
93718	9	8124939	8124954	2	8	16	GT	22	7
93720	9	8125169	8125206	2	19	38	CA	13	7
93721	9	8125654	8125693	2	20	40	TG	18	8
93737	9	8141689	8141700	2	6	12	TC	11	3
93745	9	8146202	8146223	2	11	22	CT	8	4
93746	9	8146360	8146383	4	6	24	AGAC	22	7
93752	9	8151049	8151066	3	6	18	CAG	28	3
93753	9	8151316	8151335	2	10	20	TG	22	5
93754	9	8151529	8151554	2	13	26	GT	15	11
93759	9	8155735	8155764	2	15	30	AC	16	11
93760	9	8156078	8156111	2	17	34	TG	11	10
93761	9	8156952	8156963	2	6	12	GT	26	3
93762	9	8157212	8157229	2	9	18	CT	27	5
93765	9	8162251	8162268	2	9	18	GA	15	5
93767	9	8164259	8164286	4	7	28	TGTT	18	8
93771	9	8167989	8168012	2	12	24	TG	28	6
93775	9	8174466	8174481	2	8	16	GT	24	6
93785	9	8184576	8184591	4	4	16	AATT	9	4
93789	9	8191019	8191064	2	23	46	AC	8	5
93792	9	8203587	8203602	4	4	16	GATG	26	3
93796	9	8212480	8212495	2	8	16	TG	20	7
93800	9	8223167	8223187	3	7	21	AGG	23	11
93808	9	8239238	8239258	3	7	21	TAA	14	6
93813	9	8246063	8246100	2	19	38	CA	14	6
93818	9	8264684	8264699	2	8	16	TG	21	4
93825	9	8276389	8276404	2	8	16	GT	10	5
93833	9	8291865	8291878	2	7	14	GT	14	4
93835	9	8293505	8293522	2	9	18	AC	25	4
93836	9	8293864	8293885	2	11	22	CT	14	4
93839	9	8299841	8299860	2	10	20	AG	23	12
93840	9	8300118	8300141	2	12	24	TG	6	4
93842	9	8304714	8304735	2	11	22	GT	19	6
93851	9	8326504	8326523	2	10	20	GT	20	8
93852	9	8329277	8329300	2	12	24	GT	9	6
93853	9	8330408	8330431	4	6	24	AAAG	22	14
93864	9	8367008	8367045	2	19	38	CA	9	8
93867	9	8372683	8372696	2	7	14	CT	20	6
93868	9	8375493	8375508	2	8	16	AC	15	7

93876	9	8388346	8388365	2	10	20	CA	20	6
93879	9	8392431	8392444	2	7	14	AG	15	3
93880	9	8392492	8392507	2	8	16	AC	18	4
93886	9	8408195	8408232	2	19	38	AC	17	10
93888	9	8410342	8410371	2	15	30	TG	9	9
93891	9	8414872	8414887	2	8	16	AC	15	5
93892	9	8414945	8414982	2	19	38	CA	15	8
93895	9	8419251	8419274	3	8	24	GAG	20	8
93896	9	8419363	8419374	2	6	12	GT	22	4
93898	9	8427515	8427530	2	8	16	TG	32	5
93899	9	8427737	8427788	4	13	52	CTAT	21	15
93901	9	8427987	8428000	2	7	14	CA	17	9
93903	9	8429423	8429450	2	14	28	GT	12	11
93904	9	8437813	8437834	2	11	22	GT	17	11
93907	9	8444098	8444115	3	6	18	TGC	22	3
93909	9	8448599	8448610	2	6	12	AT	19	4
93912	9	8455589	8455604	4	4	16	TCAG	25	11
93913	9	8455609	8455624	4	4	16	TCAC	29	12
93915	9	8456047	8456073	3	9	27	ACT	16	13
93916	9	8461584	8461613	3	10	30	AAG	25	15
93921	9	8472288	8472319	2	16	32	GA	24	6
93923	9	8474304	8474335	4	8	32	AATC	22	5
93925	9	8479386	8479409	4	6	24	TATT	19	7
93929	9	8483228	8483239	2	6	12	TG	21	5
93934	9	8491879	8491898	2	10	20	GA	13	3
93937	9	8506295	8506320	2	13	26	GT	16	5
93938	9	8508818	8508832	3	5	15	ATG	24	4
93939	9	8508836	8508850	3	5	15	ATG	24	3
93942	9	8514953	8514972	2	10	20	TG	14	10
93944	9	8515827	8515844	2	9	18	GT	20	7
93945	9	8515926	8515941	2	8	16	TG	17	3
93946	9	8515943	8515954	2	6	12	GT	18	4
93948	9	8519405	8519418	2	7	14	CA	13	7
93950	9	8520904	8520921	2	9	18	TG	16	9
93953	9	8524507	8524540	2	17	34	AC	15	13
93954	9	8524593	8524614	2	11	22	AC	23	15
93955	9	8525203	8525224	2	11	22	CA	14	8
93957	9	8525772	8525801	2	15	30	AG	7	10
93963	9	8528665	8528679	3	5	15	AAC	31	3
93986	9	8550555	8550566	2	6	12	CT	27	5
93987	9	8550808	8550840	3	11	33	TTA	6	7
93989	9	8550917	8550937	3	7	21	AAG	11	3
93991	9	8551214	8551231	3	6	18	TGG	17	3
93995	9	8553991	8554004	2	7	14	AC	21	7
93996	9	8554006	8554019	2	7	14	CA	26	10
94005	9	8570186	8570203	3	6	18	TCA	22	3
94006	9	8570519	8570533	3	5	15	GCT	20	3
94007	9	8578542	8578559	2	9	18	TG	15	8
94011	9	8582734	8582753	4	5	20	TCAT	22	4
94013	9	8583738	8583758	3	7	21	CTC	16	5
94014	9	8585100	8585119	2	10	20	TG	24	10

94016	9	8585492	8585503	2	6	12	AG	12	3
94017	9	8585905	8585920	2	8	16	AG	17	3
94021	9	8588011	8588030	2	10	20	TG	33	3
94027	9	8596928	8596943	4	4	16	AAGA	21	3
94028	9	8599226	8599259	2	17	34	TG	20	9
94031	9	8599813	8599828	2	8	16	TG	14	9
94033	9	8599875	8599890	2	8	16	AG	24	4
94036	9	8600891	8600908	2	9	18	CT	16	4
94037	9	8601172	8601189	3	6	18	TTC	10	4
94038	9	8601442	8601461	4	5	20	TTCT	12	4
94040	9	8601963	8601990	2	14	28	AC	26	6
94041	9	8602081	8602116	2	18	36	TG	15	11
94044	9	8605998	8606015	3	6	18	GCT	21	5
94045	9	8607759	8607774	2	8	16	CA	15	9
94050	9	8614421	8614440	2	10	20	TG	14	4
94051	9	8614598	8614609	2	6	12	AC	20	3
94055	9	8638279	8638296	3	6	18	TTG	14	3
94063	9	8657626	8657639	2	7	14	AC	12	5
94065	9	8660035	8660048	2	7	14	AC	20	5
94069	9	8664795	8664816	2	11	22	GT	7	9
94071	9	8664996	8665033	2	19	38	GT	14	9
94075	9	8668896	8668915	2	10	20	GA	21	6
94084	9	8682887	8682908	2	11	22	CA	15	4
94085	9	8683054	8683067	2	7	14	TG	23	4
94086	9	8683724	8683735	2	6	12	AT	27	3
94093	9	8695364	8695383	2	10	20	TG	15	9
94095	9	8699177	8699202	2	13	26	TC	13	5
94097	9	8703654	8703671	2	9	18	TG	28	3
94098	9	8703796	8703833	2	19	38	TG	16	11
94104	9	8722769	8722780	2	6	12	CT	22	5
94111	9	8735665	8735688	2	12	24	AC	20	6
94117	9	8753352	8753369	2	9	18	AC	10	4
94118	9	8757800	8757845	2	23	46	AC	24	8
94122	9	8764980	8764993	2	7	14	TG	9	5
94149	9	8805364	8805385	2	11	22	GA	13	9
94150	9	8807957	8807974	3	6	18	ATC	16	4
94151	9	8810353	8810370	2	9	18	TG	10	5
94153	9	8810776	8810787	2	6	12	TG	32	3
94154	9	8811045	8811082	2	19	38	GT	16	9
94157	9	8811823	8811838	4	4	16	ATTT	24	3
94165	9	8827473	8827488	2	8	16	AC	25	4
94167	9	8829464	8829483	2	10	20	TG	21	6
94169	9	8837012	8837023	2	6	12	GT	21	3
94171	9	8838011	8838028	2	9	18	TG	27	7
94172	9	8840233	8840244	2	6	12	CT	15	3
94181	9	8849529	8849548	2	10	20	AC	18	17
94182	9	8849678	8849707	2	15	30	TG	16	11
94185	9	8865831	8865854	2	12	24	AC	13	12
94192	9	8884988	8885007	2	10	20	CA	18	13
94196	9	8893548	8893567	4	5	20	AAAG	30	3
94198	9	8899427	8899440	2	7	14	CA	14	7

94199	9	8900247	8900262	4	4	16	TGCC	29	6
94200	9	8900421	8900438	2	9	18	CA	15	11
94202	9	8902174	8902191	2	9	18	AC	21	7
94204	9	8903064	8903105	2	21	42	AC	9	10
94205	9	8904305	8904319	3	5	15	ACA	26	3
94210	9	8918005	8918019	3	5	15	AGA	26	3
94217	9	8926393	8926422	2	15	30	GT	16	9
94221	9	8937917	8937976	4	15	60	GATA	11	9
94225	9	8955719	8955736	2	9	18	TG	25	3
94231	9	8965789	8965802	2	7	14	AT	19	8
94233	9	8972491	8972524	2	17	34	AC	12	7
94240	9	8977850	8977861	2	6	12	CA	24	3
94254	9	9003788	9003835	4	12	48	TGTA	14	4
94257	9	9007560	9007589	2	15	30	AG	11	6
94264	9	9011221	9011272	2	26	52	TG	7	9
94265	9	9011481	9011530	2	25	50	AC	12	6
94266	9	9012045	9012082	2	19	38	TG	7	8
94268	9	9014017	9014036	2	10	20	AG	11	5
94271	9	9017484	9017499	4	4	16	AAAT	25	3
94273	9	9020537	9020574	2	19	38	AC	6	8
94278	9	9030295	9030322	2	14	28	AC	11	7
94280	9	9032814	9032833	2	10	20	TG	18	5
94285	9	9035445	9035468	2	12	24	TG	14	9
94293	9	9069135	9069152	3	6	18	GCT	24	4
94297	9	9073507	9073542	4	9	36	TATC	14	6
94299	9	9073981	9074016	2	18	36	TG	16	17
94302	9	9078905	9078922	2	9	18	CA	17	6
94305	9	9093978	9093989	2	6	12	TG	28	4
94306	9	9098313	9098328	2	8	16	AC	25	5
94307	9	9098819	9098830	2	6	12	GC	25	8
94311	9	9111770	9111781	2	6	12	TA	9	3
94313	9	9113298	9113323	2	13	26	CT	24	6
94315	9	9121301	9121315	3	5	15	ACA	19	6
94319	9	9122396	9122413	3	6	18	GTT	23	6
94320	9	9123301	9123326	2	13	26	AC	15	10
94321	9	9124106	9124120	3	5	15	TCC	14	4
94322	9	9124121	9124135	3	5	15	CTC	13	4
94329	9	9129326	9129359	2	17	34	GT	21	9
94331	9	9129763	9129776	2	7	14	TG	24	3
94333	9	9132315	9132332	2	9	18	AG	20	4
94335	9	9134097	9134114	3	6	18	CTT	27	3
94336	9	9135709	9135738	3	10	30	TCC	19	4
94337	9	9141009	9141026	2	9	18	CA	23	6
94341	9	9145306	9145323	2	9	18	GA	25	8
94347	9	9152465	9152478	2	7	14	GT	27	5
94348	9	9152619	9152646	2	14	28	TG	21	7
94349	9	9152719	9152734	4	4	16	GTGC	28	7
94350	9	9152735	9152748	2	7	14	GT	28	6
94351	9	9154883	9154914	2	16	32	GT	15	10
94357	9	9157906	9157935	2	15	30	TC	19	10
94362	9	9161172	9161191	2	10	20	GA	9	4

94363	9	9164881	9164898	2	9	18	CA	28	5
94367	9	9173917	9173934	3	6	18	GGA	33	3
94369	9	9182695	9182716	2	11	22	TG	27	6
94373	9	9185536	9185551	2	8	16	AG	26	16
94379	9	9196128	9196141	2	7	14	CA	27	3
94380	9	9196269	9196308	2	20	40	TG	10	10
94386	9	9201024	9201059	2	18	36	TG	10	8
94394	9	9209578	9209589	2	6	12	AC	21	3
94396	9	9214104	9214130	3	9	27	GGA	15	3
94397	9	9215602	9215627	2	13	26	AC	16	7
94398	9	9217581	9217620	4	10	40	AAGA	19	11
94400	9	9218865	9218878	2	7	14	TA	18	6
94416	9	9236751	9236765	3	5	15	TTG	26	3
94417	9	9237172	9237192	3	7	21	CAT	29	9
94418	9	9237193	9237207	3	5	15	CAG	31	9
94422	9	9243785	9243812	2	14	28	GT	7	6
94426	9	9246775	9246800	2	13	26	TG	22	12
94427	9	9247243	9247268	2	13	26	GT	6	6
94428	9	9247373	9247393	3	7	21	TCT	25	4
94433	9	9256606	9256621	2	8	16	CA	20	4
94437	9	9263952	9263973	2	11	22	AC	18	9
94441	9	9285599	9285610	2	6	12	GA	32	3
94444	9	9289519	9289552	2	17	34	AC	15	7
94445	9	9290770	9290787	3	6	18	CTG	19	3
94453	9	9299633	9299646	2	7	14	CA	21	4
94455	9	9300966	9300985	2	10	20	AC	13	14
94457	9	9302346	9302369	2	12	24	AC	24	8
94459	9	9305810	9305824	3	5	15	TTG	25	15
94460	9	9309140	9309163	4	6	24	TAAA	31	4
94463	9	9317617	9317634	2	9	18	GA	8	8
94464	9	9317637	9317652	2	8	16	GA	14	11
94473	9	9323987	9324018	2	16	32	GT	14	7
94476	9	9337187	9337200	2	7	14	AG	22	6
94479	9	9343890	9343907	2	9	18	AC	21	6
94485	9	9372986	9373007	2	11	22	CT	7	4
94486	9	9376058	9376087	2	15	30	TG	14	10
94487	9	9376251	9376262	2	6	12	TG	21	3
94491	9	9383420	9383461	2	21	42	AC	10	10
94492	9	9385786	9385819	2	17	34	TG	7	6
94495	9	9390941	9390954	2	7	14	TC	17	3
94499	9	9395075	9395098	3	8	24	CTG	30	11
94504	9	9401961	9401972	2	6	12	TG	21	4
94516	9	9424734	9424753	2	10	20	GT	22	4
94517	9	9425079	9425093	3	5	15	CCT	30	3
94518	9	9427483	9427494	2	6	12	AG	29	4
94521	9	9439497	9439530	2	17	34	TG	9	10
94522	9	9439751	9439772	2	11	22	CA	17	7
94523	9	9440107	9440124	2	9	18	TG	26	8
94527	9	9449430	9449441	2	6	12	TG	29	3
94534	9	9454164	9454185	2	11	22	TC	17	6
94536	9	9462274	9462285	2	6	12	CT	23	4

94552	9	9494525	9494539	3	5	15	GTG	25	4
94556	9	9499714	9499737	2	12	24	GT	14	9
94557	9	9499826	9499843	3	6	18	GCA	25	7
94558	9	9499978	9500003	2	13	26	AC	20	11
94573	9	9529666	9529686	3	7	21	GTA	7	8
94574	9	9532098	9532127	3	10	30	CAG	25	5
94576	9	9537827	9537844	2	9	18	AT	21	6
94578	9	9542810	9542824	3	5	15	ATA	11	6
94581	9	9546116	9546133	3	6	18	ACT	14	5
94589	9	9550546	9550560	3	5	15	TTG	25	3
94591	9	9556911	9556926	2	8	16	GT	23	5
94597	9	9565821	9565838	2	9	18	GT	25	5
94600	9	9568150	9568161	2	6	12	CA	21	3
94604	9	9569788	9569805	3	6	18	GAG	26	3
94605	9	9572871	9572900	2	15	30	TG	16	11
94608	9	9573345	9573360	2	8	16	TG	22	4
94609	9	9573638	9573655	2	9	18	CA	18	13
94611	9	9575924	9575937	2	7	14	AC	13	6
94612	9	9575940	9575955	2	8	16	AC	10	5
94613	9	9575968	9575983	2	8	16	CT	7	3
94623	9	9595153	9595164	2	6	12	TG	36	3
94625	9	9600444	9600455	2	6	12	GT	31	3
94629	9	9608470	9608485	2	8	16	AC	13	5
94633	9	9610389	9610404	2	8	16	TG	15	9
94635	9	9611941	9611964	4	6	24	CAGT	17	4
94637	9	9612635	9612656	2	11	22	AG	24	7
94644	9	9627570	9627583	2	7	14	CA	31	3
94647	9	9632331	9632346	4	4	16	AAAC	23	11
94651	9	9658340	9658357	2	9	18	CT	20	5
94652	9	9660103	9660122	2	10	20	GT	18	6
94659	9	9667287	9667322	3	12	36	CAG	24	4
94661	9	9670873	9670884	2	6	12	CT	26	5
94664	9	9680509	9680554	2	23	46	GA	6	4
94668	9	9692094	9692121	4	7	28	CAAC	20	9
94669	9	9694888	9694913	2	13	26	TG	11	8
94670	9	9697459	9697470	2	6	12	GT	21	4
94672	9	9699490	9699513	2	12	24	AC	13	5
94678	9	9724916	9724935	2	10	20	CA	16	4
94692	9	9755984	9756009	2	13	26	CA	20	6
94698	9	9771049	9771068	4	5	20	AAGA	22	5
94699	9	9771966	9771986	3	7	21	GGA	7	6
94701	9	9776151	9776178	4	7	28	ATGA	19	9
94703	9	9776802	9776817	4	4	16	CATA	17	5
94704	9	9779306	9779317	2	6	12	CA	11	5
94705	9	9780732	9780753	2	11	22	AC	12	13
94706	9	9786282	9786297	2	8	16	TG	23	14
94707	9	9786299	9786310	2	6	12	GT	21	9
94709	9	9788308	9788364	3	19	57	TAG	13	10
94710	9	9790723	9790749	3	9	27	TCA	9	9
94712	9	9793220	9793261	2	21	42	AC	12	12
94713	9	9796008	9796047	2	20	40	CA	7	11

94724	9	9805826	9805852	3	9	27	AGG	21	7
94725	9	9809537	9809568	2	16	32	CA	15	13
94726	9	9810244	9810259	2	8	16	CT	22	4
94728	9	9817599	9817618	2	10	20	GT	24	4
94729	9	9817758	9817783	2	13	26	AC	10	3
94733	9	9819218	9819268	3	17	51	TCT	19	10
94738	9	9833210	9833233	2	12	24	CA	13	5
94745	9	9851250	9851277	2	14	28	GT	16	9
94747	9	9852409	9852434	2	13	26	AC	7	3
94748	9	9853476	9853521	2	23	46	AC	11	11
94749	9	9853611	9853638	2	14	28	CA	17	4
94753	9	9854532	9854575	2	22	44	CA	7	8
94756	9	9856617	9856654	2	19	38	TG	14	10
94757	9	9856808	9856825	2	9	18	AG	19	4
94761	9	9863895	9863908	2	7	14	GA	25	3
94764	9	9865556	9865583	2	14	28	AC	21	5
94777	9	9884052	9884071	2	10	20	GT	18	7
94786	9	9905158	9905179	2	11	22	CA	17	8
94804	9	9927227	9927286	2	30	60	TG	8	9
94805	9	9930661	9930682	2	11	22	CA	10	10
94808	9	9934294	9934309	2	8	16	AC	23	3
94809	9	9935313	9935332	2	10	20	TA	13	5
94810	9	9938273	9938310	2	19	38	TG	14	11
94812	9	9939152	9939165	2	7	14	AG	21	3
94813	9	9939531	9939542	2	6	12	AC	30	4
94821	9	9952503	9952514	2	6	12	CA	23	3
94827	9	9961168	9961181	2	7	14	AC	19	6
94828	9	9961414	9961433	2	10	20	TG	11	11
94830	9	9961543	9961560	2	9	18	TG	23	3
94834	9	9963526	9963555	2	15	30	AC	9	7
94838	9	9965248	9965265	2	9	18	GT	17	7
94839	9	9967756	9967769	2	7	14	TG	27	8
94842	9	9970035	9970062	2	14	28	GT	11	5
94843	9	9972098	9972111	2	7	14	AC	36	3
94845	9	9976423	9976448	2	13	26	GT	13	8
94847	9	9981772	9981785	2	7	14	AC	12	4
94848	9	9981816	9981843	2	14	28	AG	22	9
94849	9	9982182	9982199	3	6	18	GCT	15	10
94851	9	9983061	9983076	4	4	16	AACA	20	3
94854	9	9987054	9987067	2	7	14	GA	20	8
94862	9	9997139	9997156	2	9	18	AT	8	5
94867	9	10008662	10008691	2	15	30	AC	13	7
94868	9	10010957	10010974	2	9	18	CA	24	3
94871	9	10015717	10015734	3	6	18	GCT	27	4
94873	9	10017640	10017651	2	6	12	GT	31	3
94880	9	10024969	10024994	2	13	26	TG	11	4
94881	9	10025134	10025153	2	10	20	TG	11	5
94886	9	10029109	10029134	2	13	26	CA	21	7
94887	9	10029231	10029254	2	12	24	CA	16	11
94889	9	10029636	10029667	4	8	32	CAGG	21	8
94890	9	10029740	10029755	2	8	16	AT	14	7

94893	9	10032697	10032711	3	5	15	TTG	32	3
94896	9	10036861	10036890	2	15	30	CA	11	11
94897	9	10037279	10037290	2	6	12	CT	34	3
94922	9	10094812	10094829	2	9	18	AT	6	5
94923	9	10100315	10100340	2	13	26	TG	9	9
94955	9	10153549	10153568	4	5	20	GCGT	24	11
94968	9	10181056	10181067	2	6	12	TG	22	4
94972	9	10192836	10192857	2	11	22	GT	12	5
94974	9	10193781	10193792	2	6	12	AG	27	3
94975	9	10199114	10199131	2	9	18	CA	14	9
94979	9	10200824	10200847	2	12	24	CA	19	11
94983	9	10204167	10204184	2	9	18	CT	20	3
94985	9	10205459	10205472	2	7	14	AC	25	3
94995	9	10221517	10221534	2	9	18	TG	16	6
95009	9	10233304	10233321	3	6	18	GGA	19	4
95011	9	10235773	10235786	2	7	14	TG	29	3
95013	9	10244079	10244098	2	10	20	GT	23	7
95014	9	10245515	10245538	2	12	24	CA	23	7
95018	9	10256031	10256054	3	8	24	ATG	24	4
95019	9	10256472	10256495	3	8	24	GAG	17	6
95026	9	10267307	10267330	2	12	24	GT	15	7
95037	9	10278106	10278137	2	16	32	TG	20	14
95042	9	10280890	10280903	2	7	14	TG	29	5
95043	9	10283235	10283254	4	5	20	AGAC	24	4
95044	9	10285342	10285357	2	8	16	GT	24	8
95051	9	10322672	10322691	2	10	20	CT	27	4
95052	9	10322872	10322893	2	11	22	TG	22	6
95058	9	10329815	10329838	2	12	24	CA	22	3
95059	9	10330078	10330107	2	15	30	TG	14	9
95071	9	10356017	10356034	2	9	18	AC	19	8
95078	9	10367788	10367805	2	9	18	AG	18	8
95082	9	10376271	10376285	3	5	15	ACA	22	4
95085	9	10388507	10388528	2	11	22	AC	16	4
95089	9	10393023	10393036	2	7	14	CT	22	3
95091	9	10395225	10395238	2	7	14	CA	25	5
95093	9	10407533	10407544	2	6	12	GA	35	3
95096	9	10413515	10413554	2	20	40	GT	11	7
95098	9	10421968	10421981	2	7	14	GT	15	3
95099	9	10422182	10422199	2	9	18	CA	21	5
95100	9	10422923	10422940	2	9	18	AT	8	6
95104	9	10425519	10425538	2	10	20	AG	14	9
95108	9	10430192	10430203	2	6	12	TG	6	4
95114	9	10433122	10433137	2	8	16	GA	12	7
95118	9	10453967	10453986	4	5	20	GTGC	20	4
95119	9	10454825	10454844	2	10	20	CA	10	10
95126	9	10472116	10472129	2	7	14	TG	23	5
95131	9	10476846	10476867	2	11	22	TG	12	6
95136	9	10479807	10479842	2	18	36	AC	13	8
95142	9	10489355	10489374	2	10	20	CA	22	7
95148	9	10497472	10497487	2	8	16	CA	25	3
95149	9	10499326	10499343	2	9	18	AC	26	6

95150	9	10505418	10505437	4	5	20	GTCT	31	5
95152	9	10509711	10509728	2	9	18	TG	20	4
95157	9	10519333	10519382	2	25	50	CA	10	9
95162	9	10523395	10523408	2	7	14	AT	21	3
95163	9	10524011	10524022	2	6	12	CA	21	4
95164	9	10524662	10524679	2	9	18	CA	19	3
95171	9	10528048	10528061	2	7	14	AG	16	8
95172	9	10528465	10528479	3	5	15	GAG	33	3
95173	9	10530148	10530159	2	6	12	AC	28	4
95178	9	10537753	10537767	3	5	15	TAA	22	6
95180	9	10539380	10539399	4	5	20	TTTA	8	3
95188	9	10548298	10548321	4	6	24	AGTG	31	13
95189	9	10550981	10550996	2	8	16	GT	21	3
95196	9	10558098	10558118	3	7	21	ACT	20	5
95198	9	10576565	10576584	2	10	20	AC	23	5
95200	9	10578460	10578473	2	7	14	TG	22	3
95202	9	10581998	10582013	2	8	16	AG	25	3
95203	9	10582791	10582808	3	6	18	TCC	32	5
95204	9	10588469	10588484	2	8	16	AC	19	3
95206	9	10604046	10604059	2	7	14	GT	14	4
95207	9	10604249	10604266	2	9	18	TG	20	6
95218	9	10616768	10616787	2	10	20	GA	20	6
95223	9	10631373	10631398	2	13	26	AC	18	4
95228	9	10650902	10650953	4	13	52	ATAG	22	15
95231	9	10656025	10656058	2	17	34	AC	9	8
95237	9	10673669	10673680	2	6	12	AT	13	3
95238	9	10675011	10675031	3	7	21	AGC	18	3
95244	9	10677773	10677798	2	13	26	TC	9	11
95260	9	10704553	10704576	2	12	24	TG	22	8
95262	9	10707903	10707922	2	10	20	AC	15	7
95263	9	10708072	10708097	2	13	26	CA	9	11
95271	9	10732420	10732433	2	7	14	AC	21	8
95274	9	10739324	10739339	2	8	16	TC	26	4
95280	9	10755617	10755634	2	9	18	AT	15	5
95281	9	10757844	10757859	2	8	16	GT	13	10
95282	9	10757861	10757878	2	9	18	TG	12	10
95285	9	10773837	10773848	2	6	12	TG	23	4
95294	9	10808040	10808059	2	10	20	GT	13	6
95296	9	10819414	10819433	4	5	20	ACAG	21	9
95307	9	10858777	10858788	2	6	12	TC	35	3
95314	9	10867857	10867868	2	6	12	AC	15	11
95319	9	10868865	10868888	3	8	24	AAG	19	12
95321	9	10871579	10871618	2	20	40	AC	12	15
95322	9	10872013	10872026	2	7	14	AC	23	4
95323	9	10872243	10872274	2	16	32	AC	8	6
95339	9	10892554	10892569	2	8	16	TG	20	7
95340	9	10893288	10893301	2	7	14	TG	18	3
95342	9	10895130	10895149	2	10	20	CA	18	5
95345	9	10899392	10899411	2	10	20	CA	15	11
95351	9	10915048	10915061	2	7	14	AC	24	6
95360	9	10933610	10933637	2	14	28	CT	21	6

95363	9	10944250	10944263	2	7	14	AC	30	4
95366	9	10955016	10955039	3	8	24	GCA	36	3
95374	9	10979409	10979436	2	14	28	TC	7	5
95376	9	10983164	10983199	2	18	36	GT	11	10
95386	9	11002190	11002209	2	10	20	GT	10	7
95389	9	11002402	11002421	2	10	20	GT	13	8
95403	9	11011078	11011099	2	11	22	GT	15	6
95409	9	11018689	11018708	4	5	20	TTAT	23	7
95411	9	11018945	11018960	2	8	16	CT	14	4
95422	9	11042056	11042070	3	5	15	CCT	29	4
95427	9	11043832	11043845	2	7	14	AC	18	4
95431	9	11047313	11047324	2	6	12	GT	24	6
95437	9	11052007	11052024	2	9	18	CA	14	9
95442	9	11053624	11053657	2	17	34	TC	11	12
95448	9	11056774	11056789	4	4	16	TTTC	24	3
95452	9	11061770	11061797	2	14	28	GT	12	8
95454	9	11068210	11068229	2	10	20	CA	22	3
95457	9	11075770	11075793	3	8	24	AAG	17	3
95459	9	11078889	11078906	2	9	18	AG	29	5
95466	9	11109609	11109624	2	8	16	CA	27	8
95468	9	11110658	11110685	2	14	28	CA	25	9
95469	9	11111615	11111642	4	7	28	GATG	19	5
95472	9	11117429	11117444	2	8	16	TG	25	4
95475	9	11125173	11125194	2	11	22	AC	25	8
95477	9	11126250	11126265	2	8	16	CT	27	4
95483	9	11130126	11130137	2	6	12	CA	27	3
95485	9	11133334	11133361	2	14	28	AC	10	10
95491	9	11143265	11143294	2	15	30	CA	24	8
95494	9	11151507	11151544	2	19	38	GT	9	9
95509	9	11180595	11180609	3	5	15	GCT	28	3
95514	9	11183932	11183945	2	7	14	GT	26	4
95515	9	11184588	11184627	2	20	40	CA	11	13
95521	9	11208033	11208046	2	7	14	AC	27	3
95522	9	11208231	11208246	2	8	16	AC	27	3
95523	9	11210331	11210356	2	13	26	GT	15	7
95526	9	11216558	11216585	2	14	28	TG	22	8
95527	9	11217448	11217465	2	9	18	TG	21	4
95532	9	11256723	11256744	2	11	22	AC	16	4
95535	9	11272696	11272707	2	6	12	CA	14	7
95536	9	11273281	11273292	2	6	12	AG	28	3
95556	9	11311901	11311920	2	10	20	GT	25	7
95558	9	11315596	11315613	3	6	18	TTC	9	5
95564	9	11323387	11323418	2	16	32	GT	17	13
95567	9	11333234	11333247	2	7	14	GA	6	3
95573	9	11337321	11337336	4	4	16	AAAC	29	3
95576	9	11339454	11339474	3	7	21	TGT	25	6
95584	9	11372323	11372336	2	7	14	TA	18	6
95586	9	11379649	11379668	4	5	20	CAAA	20	5
95587	9	11379670	11379689	4	5	20	AAAC	21	8
95599	9	11397704	11397724	3	7	21	AAC	26	4
95614	9	11439601	11439628	2	14	28	TG	10	7

95616	9	11442312	11442331	2	10	20	CA	14	3
95619	9	11449139	11449158	2	10	20	AC	13	3
95624	9	11453650	11453663	2	7	14	CA	21	4
95625	9	11453860	11453893	2	17	34	TG	11	9
95635	9	11472092	11472151	3	20	60	AGA	15	13
95639	9	11482392	11482407	2	8	16	CA	10	6
95646	9	11498743	11498758	4	4	16	TTTG	29	6
95649	9	11503610	11503625	2	8	16	TA	19	15
95650	9	11503626	11503641	2	8	16	CA	19	15
95651	9	11504221	11504250	2	15	30	TG	13	10
95653	9	11509568	11509581	2	7	14	GA	20	3
95663	9	11545100	11545119	2	10	20	GT	18	5
95668	9	11557345	11557360	4	4	16	GCTA	24	6
95671	9	11584265	11584290	2	13	26	GT	20	8
95679	9	11601779	11601802	2	12	24	GT	17	4
95687	9	11614395	11614424	2	15	30	AC	18	11
95690	9	11622156	11622173	3	6	18	TGG	26	5
95695	9	11632376	11632389	2	7	14	TG	20	9
95699	9	11642161	11642186	2	13	26	GT	7	11
95703	9	11645765	11645798	2	17	34	GA	6	7
95705	9	11647169	11647188	2	10	20	TG	12	8
95707	9	11647632	11647661	2	15	30	TG	11	5
95709	9	11647746	11647775	2	15	30	GT	11	9
95713	9	11655035	11655058	3	8	24	CTC	26	5
95715	9	11655254	11655283	2	15	30	AC	8	5
95723	9	11659954	11659971	3	6	18	AGA	22	4
95724	9	11660014	11660031	3	6	18	CAC	32	4
95729	9	11664772	11664793	2	11	22	AC	13	9
95731	9	11668270	11668285	2	8	16	CA	25	4
95732	9	11669026	11669045	2	10	20	TG	20	4
95733	9	11669418	11669465	2	24	48	AG	8	9
95734	9	11672776	11672791	2	8	16	GA	14	4
95736	9	11674965	11674992	2	14	28	AC	15	4
95737	9	11675760	11675781	2	11	22	CA	20	7
95744	9	11692549	11692568	4	5	20	AGAC	31	3
95748	9	11699046	11699061	2	8	16	CA	24	3
95756	9	11730947	11730962	2	8	16	AT	14	3
95757	9	11731258	11731277	4	5	20	GCTA	30	3
95758	9	11735288	11735301	2	7	14	CA	14	11
95766	9	11756286	11756299	2	7	14	TG	23	3
95768	9	11758570	11758589	2	10	20	TG	23	8
95769	9	11761750	11761765	4	4	16	CTTT	25	3
95771	9	11777482	11777502	3	7	21	AAG	23	4
95773	9	11792949	11792968	4	5	20	TCTT	33	9
95778	9	11808508	11808528	3	7	21	CAT	30	4
95780	9	11813309	11813334	2	13	26	GT	16	11
95789	9	11820105	11820124	2	10	20	AC	20	7
95790	9	11826495	11826518	2	12	24	TG	15	11
95794	9	11831652	11831663	2	6	12	TG	24	4
95801	9	11843611	11843626	2	8	16	TG	17	4
95803	9	11844851	11844872	2	11	22	CA	20	5

95804	9	11846161	11846178	2	9	18	TG	23	5
95809	9	11851974	11851995	2	11	22	AC	16	7
95812	9	11852832	11852843	2	6	12	GT	23	3
95814	9	11856121	11856152	2	16	32	TG	9	9
95821	9	11867807	11867836	2	15	30	AG	15	6
95827	9	11874045	11874062	2	9	18	GT	14	7
95828	9	11874064	11874075	2	6	12	TG	12	5
95829	9	11875836	11875861	2	13	26	TG	21	8
95831	9	11881374	11881389	2	8	16	TG	24	4
95833	9	11886193	11886204	2	6	12	TC	17	4
95834	9	11886207	11886220	2	7	14	TC	18	6
95837	9	11902499	11902514	4	4	16	TTTA	29	3
95842	9	11907800	11907811	2	6	12	TA	7	6
95843	9	11911547	11911588	2	21	42	TG	11	14
95850	9	11920714	11920737	2	12	24	CA	14	12
95851	9	11931725	11931744	2	10	20	CA	20	6
95852	9	11931892	11931911	2	10	20	CA	20	5
95857	9	11936441	11936467	3	9	27	TCC	14	5
95864	9	11947678	11947711	2	17	34	TG	11	10
95871	9	11954037	11954058	2	11	22	TG	7	8
95879	9	11974206	11974220	3	5	15	ACC	21	4
95880	9	11982398	11982419	2	11	22	GT	14	9
95888	9	12001581	12001608	2	14	28	AC	9	6
95891	9	12008435	12008474	2	20	40	CA	14	17
95894	9	12020952	12020979	2	14	28	CT	18	9
95897	9	12032384	12032395	2	6	12	AC	20	8
95899	9	12033360	12033379	2	10	20	TC	12	5
95900	9	12033690	12033745	2	28	56	TG	13	6
95911	9	12050845	12050895	3	17	51	TCT	16	11
95913	9	12051380	12051415	2	18	36	CT	15	13
95916	9	12055876	12055901	2	13	26	GT	17	6
95920	9	12066162	12066179	3	6	18	CTC	28	4
95921	9	12068157	12068168	2	6	12	TG	35	3
95929	9	12071500	12071520	3	7	21	TCT	12	6
95931	9	12071850	12071867	2	9	18	GA	10	7
95933	9	12071920	12071937	2	9	18	GA	13	4
95944	9	12091570	12091595	2	13	26	AC	9	4
95963	9	12143576	12143595	2	10	20	GT	19	9
95988	9	12219410	12219439	2	15	30	GT	12	8
95989	9	12222063	12222098	4	9	36	TAGA	17	7
95993	9	12229241	12229256	2	8	16	TG	14	4
95994	9	12229889	12229918	2	15	30	AG	14	4
95998	9	12246109	12246120	2	6	12	GT	15	4
96000	9	12253416	12253437	2	11	22	GT	12	11
96001	9	12254034	12254061	2	14	28	GA	12	7
96005	9	12258755	12258775	3	7	21	GCA	26	5
96008	9	12263895	12263912	2	9	18	TG	21	7
96009	9	12269508	12269529	2	11	22	CA	6	5
96014	9	12272947	12272966	4	5	20	GGAG	8	3
96018	9	12275150	12275173	2	12	24	TG	17	8
96023	9	12277698	12277715	2	9	18	CA	26	3

96024	9	12279693	12279710	2	9	18	TC	30	3
96026	9	12282671	12282684	2	7	14	TG	23	5
96028	9	12284470	12284485	2	8	16	TG	26	5
96035	9	12300360	12300379	2	10	20	CA	12	12
96036	9	12300881	12300916	2	18	36	AC	19	13
96038	9	12302329	12302358	3	10	30	GAG	15	7
96041	9	12306112	12306129	2	9	18	TG	21	5
96048	9	12329076	12329089	2	7	14	AG	28	5
96049	9	12332106	12332137	2	16	32	AC	17	9
96050	9	12332288	12332301	2	7	14	GT	19	7
96052	9	12343957	12343986	3	10	30	GTT	21	8
96054	9	12362024	12362035	2	6	12	TC	22	3
96058	9	12366078	12366115	2	19	38	GT	11	10
96059	9	12366787	12366842	2	28	56	AG	7	7
96068	9	12419249	12419262	2	7	14	TG	28	3
96071	9	12420898	12420913	4	4	16	TAAA	28	3
96073	9	12421676	12421691	2	8	16	GT	14	8
96074	9	12421742	12421753	2	6	12	TG	13	4
96075	9	12422082	12422101	2	10	20	AG	25	13
96078	9	12436619	12436634	2	8	16	TG	15	3
96083	9	12455632	12455658	3	9	27	TTG	22	6
96084	9	12460947	12460958	2	6	12	TG	19	3
96085	9	12460959	12460970	2	6	12	AG	17	4
96087	9	12462264	12462297	2	17	34	AC	12	14
96093	9	12473647	12473660	2	7	14	AT	9	8
96101	9	12501010	12501035	2	13	26	TG	21	12
96103	9	12505198	12505211	2	7	14	TG	20	6
96104	9	12511160	12511174	3	5	15	ATC	20	6
96107	9	12513777	12513792	2	8	16	GT	23	6
96110	9	12520371	12520382	2	6	12	GT	20	3
96112	9	12524188	12524217	2	15	30	TG	20	11
96114	9	12547121	12547134	2	7	14	CT	18	8
96117	9	12550504	12550515	2	6	12	GT	16	3
96120	9	12553290	12553309	2	10	20	GT	7	8
96126	9	12561199	12561236	2	19	38	TG	8	10
96127	9	12564115	12564136	2	11	22	TG	13	6
96133	9	12583551	12583565	3	5	15	CAC	28	3
96139	9	12609168	12609231	4	16	64	TAGA	12	12
96140	9	12609382	12609399	2	9	18	AC	31	5
96141	9	12611604	12611621	2	9	18	GT	16	3
96142	9	12611850	12611877	2	14	28	GT	14	13
96159	9	12645659	12645694	4	9	36	CTTT	20	3
96160	9	12645901	12645912	2	6	12	TG	29	5
96161	9	12645915	12645928	2	7	14	TG	26	5
96163	9	12646972	12646989	3	6	18	AAG	26	4
96167	9	12650117	12650140	2	12	24	AC	12	5
96169	9	12650713	12650728	2	8	16	TG	23	7
96173	9	12656962	12656983	2	11	22	TG	19	5
96198	9	12731473	12731487	3	5	15	GGT	25	5
96211	9	12768263	12768294	2	16	32	AC	19	9
96215	9	12779043	12779068	2	13	26	CA	11	8

96216	9	12784973	12784992	4	5	20	GAGG	12	5
96219	9	12791276	12791295	2	10	20	AG	26	5
96221	9	12794667	12794694	2	14	28	CA	13	10
96222	9	12795016	12795027	2	6	12	CT	21	3
96223	9	12795100	12795120	3	7	21	CAG	22	4
96227	9	12795917	12795964	4	12	48	TCTA	19	14
96235	9	12814246	12814266	3	7	21	AGC	25	8
96238	9	12822711	12822730	2	10	20	GT	21	7
96241	9	12827211	12827228	2	9	18	AC	15	6
96250	9	12845100	12845113	2	7	14	GT	20	3
96252	9	12849143	12849156	2	7	14	TG	20	5
96253	9	12849158	12849169	2	6	12	GT	13	4
96255	9	12850038	12850061	4	6	24	AGAT	34	3
96256	9	12851763	12851794	2	16	32	GT	15	10
96264	9	12872672	12872683	2	6	12	TC	27	4
96268	9	12883567	12883592	2	13	26	AC	7	8
96288	9	12907703	12907740	2	19	38	GT	10	7
96291	9	12910932	12910949	2	9	18	TG	22	3
96294	9	12913075	12913106	2	16	32	TG	14	11
96297	9	12914276	12914303	2	14	28	TG	12	6
96299	9	12917041	12917060	2	10	20	TG	27	3
96300	9	12918616	12918642	3	9	27	GAG	22	4
96305	9	12923006	12923035	3	10	30	TAT	11	4
96308	9	12930145	12930172	2	14	28	TC	9	4
96311	9	12941628	12941649	2	11	22	CA	15	3
96313	9	12943225	12943238	2	7	14	AT	6	4
96322	9	12991051	12991074	2	12	24	TG	7	4
96326	9	12992953	12992970	2	9	18	TG	14	4
96335	9	13013569	13013588	4	5	20	AAAC	22	5
96336	9	13013745	13013760	2	8	16	GA	24	4
96338	9	13019443	13019460	3	6	18	CTG	14	6
96341	9	13027567	13027604	2	19	38	TG	16	11
96347	9	13036714	13036737	2	12	24	GT	22	10
96348	9	13038414	13038427	2	7	14	GT	22	3
96355	9	13047480	13047493	2	7	14	AG	26	11
96358	9	13052582	13052599	3	6	18	TTG	20	3
96362	9	13062562	13062589	2	14	28	AC	11	6
96388	9	13105900	13105914	3	5	15	CCA	16	9
96399	9	13130630	13130643	2	7	14	AT	21	6
96401	9	13135267	13135282	4	4	16	AACC	28	5
96402	9	13135697	13135708	2	6	12	GT	22	6
96404	9	13144625	13144644	2	10	20	TG	20	6
96408	9	13153450	13153473	4	6	24	TCCA	12	7
96424	9	13208422	13208435	2	7	14	GT	17	4
96435	9	13226958	13226977	2	10	20	TG	10	6
96441	9	13234765	13234776	2	6	12	TC	21	3
96444	9	13239976	13240009	2	17	34	AC	13	12
96455	9	13246577	13246594	2	9	18	AC	22	3
96465	9	13251842	13251856	3	5	15	AAT	13	7
96478	9	13277292	13277309	2	9	18	CA	14	3
96481	9	13278695	13278710	4	4	16	AAAT	27	5

96486	9	13288460	13288480	3	7	21	GCA	12	4
96498	9	13306523	13306534	2	6	12	TC	18	3
96500	9	13316847	13316861	3	5	15	ACC	24	7
96501	9	13317404	13317415	2	6	12	GT	23	8
96505	9	13321583	13321594	2	6	12	GT	21	3
96506	9	13321596	13321609	2	7	14	TG	19	3
96507	9	13322024	13322049	2	13	26	AC	16	8
96547	9	13393135	13393154	2	10	20	GT	20	18
96553	9	13402829	13402858	2	15	30	CA	16	12
96564	9	13448401	13448414	2	7	14	CA	11	9
96572	9	13454194	13454221	4	7	28	TTTG	19	6
96575	9	13462990	13463007	2	9	18	GT	14	3
96577	9	13463497	13463529	3	11	33	CTG	24	5
96582	9	13465018	13465031	2	7	14	AG	20	5
96589	9	13466330	13466341	2	6	12	AT	18	3
96600	9	13508050	13508065	2	8	16	CA	24	5
96604	9	13516318	13516338	3	7	21	AGC	14	5
96611	9	13525192	13525211	4	5	20	TGTC	29	4
96613	9	13532461	13532493	3	11	33	TCT	11	7
96651	9	13612457	13612482	2	13	26	GT	15	7
96652	9	13618251	13618265	3	5	15	AGC	36	3
96661	9	13626949	13626974	2	13	26	TG	28	8
96664	9	13637533	13637552	2	10	20	CA	17	6
96671	9	13666251	13666278	4	7	28	GATG	19	3
96679	9	13682962	13682985	2	12	24	AC	18	10
96688	9	13723086	13723105	4	5	20	ACCA	14	3
96689	9	13727386	13727399	2	7	14	GT	18	4
96692	9	13734736	13734750	3	5	15	TGC	24	5
96695	9	13746063	13746080	2	9	18	GT	22	8
96696	9	13746082	13746095	2	7	14	TG	14	5
96705	9	13783462	13783483	2	11	22	GT	14	9
96715	9	13820773	13820784	2	6	12	TA	23	4
96716	9	13821650	13821675	2	13	26	TG	24	14
96717	9	13825965	13825990	2	13	26	AG	11	9
96718	9	13826284	13826309	2	13	26	CT	14	6
96721	9	13831020	13831037	2	9	18	GA	14	10
96725	9	13842616	13842647	2	16	32	TG	6	5
96728	9	13852259	13852322	4	16	64	TCTA	8	6
96729	9	13852527	13852550	3	8	24	GAG	14	6
96733	9	13854244	13854255	2	6	12	CT	26	8
96734	9	13868145	13868162	3	6	18	ACA	23	7
96739	9	13882234	13882247	2	7	14	AC	27	4
96746	9	13903871	13903890	2	10	20	GT	23	5
96753	9	13926167	13926182	2	8	16	AC	24	6
96754	9	13929009	13929030	2	11	22	GA	18	4
96758	9	13931441	13931456	4	4	16	TCAG	30	4
96759	9	13932023	13932048	2	13	26	AC	10	12
96766	9	13943994	13944011	2	9	18	AG	14	6
96774	9	13952781	13952840	4	15	60	AGAT	18	7
96775	9	13955352	13955377	2	13	26	TG	14	8
96778	9	13963035	13963060	2	13	26	CA	13	8

96781	9	13975725	13975754	2	15	30	CA	18	11
96782	9	13977571	13977592	2	11	22	AC	10	5
96784	9	13982923	13982949	3	9	27	TCA	10	3
96800	9	14005107	14005134	4	7	28	TGTA	16	10
96803	9	14016697	14016716	4	5	20	CCAA	29	4
96804	9	14017062	14017079	3	6	18	CAG	25	10
96806	9	14022765	14022779	3	5	15	CAC	29	3
96813	9	14039348	14039375	2	14	28	TG	11	11
96815	9	14041126	14041153	2	14	28	TG	17	7
96818	9	14054901	14054912	2	6	12	AC	16	3
96819	9	14059517	14059528	2	6	12	CA	21	3
96829	9	14076406	14076425	2	10	20	AC	30	4
96830	9	14076577	14076590	2	7	14	AT	13	4
96835	9	14080571	14080594	4	6	24	TCTG	12	8
96839	9	14088713	14088724	2	6	12	GT	26	4
96842	9	14100383	14100394	2	6	12	TA	19	3
96844	9	14111927	14111946	2	10	20	AC	12	3
96849	9	14117242	14117269	4	7	28	AGAT	18	7
96853	9	14125461	14125478	3	6	18	GGA	24	5
96858	9	14137431	14137444	2	7	14	TG	22	3
96886	9	14184583	14184594	2	6	12	AC	20	4
96887	9	14187976	14188005	3	10	30	CCT	20	11
96890	9	14191899	14191919	3	7	21	GCA	22	4
96891	9	14193197	14193210	2	7	14	GT	21	9
96906	9	14230548	14230561	2	7	14	TG	11	3
96907	9	14231878	14231897	2	10	20	AC	14	6
96910	9	14232826	14232851	2	13	26	TG	9	8
96915	9	14240752	14240779	2	14	28	GT	6	6
96922	9	14246860	14246871	2	6	12	TC	21	3
96923	9	14250357	14250384	2	14	28	AC	14	7
96927	9	14260131	14260154	2	12	24	CA	17	7
96929	9	14260406	14260431	2	13	26	CT	10	4
96946	9	14290352	14290369	3	6	18	GGA	22	7
96951	9	14295936	14295961	2	13	26	GT	22	14
96954	9	14302622	14302635	2	7	14	TG	26	5
96960	9	14310939	14310956	2	9	18	CA	23	12
96961	9	14310959	14310972	2	7	14	CA	20	7
96963	9	14311420	14311431	2	6	12	CA	17	4
96965	9	14320452	14320469	2	9	18	TG	15	5
96970	9	14350351	14350364	2	7	14	GT	15	8
96976	9	14361795	14361808	2	7	14	AT	8	3
96980	9	14371367	14371378	2	6	12	AG	27	5
96981	9	14375683	14375710	2	14	28	GT	9	4
96983	9	14377183	14377197	3	5	15	AAG	29	4
96984	9	14383951	14383976	2	13	26	CA	18	9
96985	9	14388489	14388500	2	6	12	TG	13	8
96995	9	14425716	14425729	2	7	14	TG	24	12
97002	9	14454441	14454452	2	6	12	TG	14	11
97003	9	14454455	14454472	2	9	18	TG	17	15
97007	9	14460650	14460685	2	18	36	TC	7	5
97009	9	14464062	14464076	3	5	15	CAG	24	3

97010	9	14468403	14468420	3	6	18	GAA	22	3
97014	9	14475945	14475964	2	10	20	AC	21	4
97017	9	14490881	14490895	3	5	15	ATC	27	3
97027	9	14504881	14504894	2	7	14	CA	11	5
97043	9	14545719	14545736	2	9	18	AC	23	13
97046	9	14561357	14561383	3	9	27	CCA	17	4
97048	9	14576301	14576316	4	4	16	TTAT	11	4
97050	9	14581057	14581080	3	8	24	TCC	26	6
97051	9	14582567	14582580	2	7	14	TG	17	11
97053	9	14589790	14589809	4	5	20	CTTT	29	4
97054	9	14599379	14599400	2	11	22	AC	20	7
97056	9	14604501	14604524	2	12	24	TG	13	6
97058	9	14607400	14607413	2	7	14	AC	25	6
97085	9	14642372	14642403	4	8	32	AATA	22	11
97087	9	14644136	14644153	2	9	18	TA	7	4
97092	9	14647253	14647274	2	11	22	CT	25	4
97093	9	14648098	14648113	4	4	16	AACC	33	3
97095	9	14655882	14655893	2	6	12	TG	21	3
97096	9	14659914	14659933	2	10	20	AC	11	9
97098	9	14666619	14666634	2	8	16	TG	17	8
97100	9	14671349	14671360	2	6	12	GT	29	3
97102	9	14672123	14672134	2	6	12	GT	36	3
97105	9	14682497	14682512	2	8	16	AC	23	8
97108	9	14685013	14685036	2	12	24	TG	12	6
97109	9	14687823	14687838	2	8	16	AC	14	4
97110	9	14687840	14687851	2	6	12	CA	11	4
97111	9	14687874	14687887	2	7	14	CA	23	13
97115	9	14702834	14702849	2	8	16	TG	27	4
97116	9	14705844	14705857	2	7	14	GA	30	3
97117	9	14705985	14705996	2	6	12	AC	27	3
97124	9	14714440	14714453	2	7	14	GT	18	6
97128	9	14722950	14722965	2	8	16	AT	12	9
97129	9	14722966	14722985	2	10	20	GT	12	10
97131	9	14728695	14728706	2	6	12	TC	14	3
97136	9	14745395	14745414	2	10	20	TC	12	5
97143	9	14770170	14770207	2	19	38	GT	9	10
97147	9	14781394	14781411	3	6	18	GAG	22	5
97152	9	14804823	14804834	2	6	12	AC	27	6
97153	9	14806437	14806448	2	6	12	CT	22	3
97155	9	14809098	14809131	2	17	34	AC	8	9
97165	9	14875632	14875667	3	12	36	ATT	6	6
97166	9	14876846	14876869	3	8	24	TTG	22	6
97168	9	14877574	14877585	2	6	12	AT	13	4
97173	9	14882802	14882834	3	11	33	CCT	12	9
97176	9	14885261	14885296	2	18	36	AC	12	11
97179	9	14886864	14886890	3	9	27	CAA	15	11
97181	9	14887235	14887261	3	9	27	TCC	11	3
97187	9	14890225	14890236	2	6	12	AC	18	4
97189	9	14898949	14898960	2	6	12	AC	21	5
97195	9	14931541	14931574	2	17	34	CA	15	10
97199	9	14936399	14936414	2	8	16	AC	13	5

97200	9	14936679	14936690	2	6	12	CA	26	9
97202	9	14940734	14940745	2	6	12	TG	21	3
97203	9	14941734	14941765	2	16	32	AC	9	9
97204	9	14944124	14944181	2	29	58	CA	12	16
97207	9	14946221	14946284	4	16	64	AGAT	25	11
97208	9	14946748	14946762	3	5	15	TTG	20	3
97220	9	14977573	14977600	2	14	28	GT	24	6
97222	9	14980856	14980885	2	15	30	GT	12	7
97224	9	14985626	14985657	2	16	32	CA	23	10
97225	9	14985974	14985991	3	6	18	CAG	18	7
97226	9	14986138	14986151	2	7	14	CT	21	6
97227	9	14986153	14986170	2	9	18	TC	25	8
97230	9	14994888	14994903	2	8	16	CA	22	7
97233	9	15002052	15002063	2	6	12	AT	12	3
97239	9	15016636	15016671	2	18	36	TG	8	11
97255	9	15069191	15069236	2	23	46	CA	6	9
97263	9	15095737	15095756	4	5	20	TCCA	11	3
97265	9	15103730	15103741	2	6	12	CA	13	4
97271	9	15124580	15124615	2	18	36	CA	10	10
97273	9	15129134	15129149	2	8	16	GT	12	7
97276	9	15133890	15133925	2	18	36	GT	20	8
97284	9	15142835	15142854	2	10	20	TG	19	5
97292	9	15168526	15168547	2	11	22	GT	23	10
97294	9	15179877	15179892	4	4	16	TTTG	11	10
97295	9	15179893	15179908	4	4	16	TTTC	7	5
97300	9	15184597	15184620	2	12	24	AC	12	5
97302	9	15187828	15187843	2	8	16	GT	26	4
97303	9	15188455	15188468	2	7	14	GA	24	10
97309	9	15189360	15189379	2	10	20	GT	24	7
97311	9	15189797	15189820	4	6	24	GGAT	23	3
97313	9	15193690	15193711	2	11	22	CA	19	4
97320	9	15212199	15212212	2	7	14	CA	12	6
97321	9	15214165	15214182	2	9	18	GT	23	7
97325	9	15223770	15223803	2	17	34	TG	8	7
97328	9	15233330	15233347	3	6	18	GGA	19	7
97336	9	15252581	15252618	2	19	38	TG	13	10
97337	9	15258863	15258874	2	6	12	CA	26	3
97339	9	15260719	15260750	4	8	32	AAAG	25	5
97344	9	15280698	15280743	2	23	46	AC	8	13
97352	9	15288779	15288806	4	7	28	TATG	17	5
97354	9	15294521	15294532	2	6	12	CA	32	3
97376	9	15343135	15343154	4	5	20	AGTT	14	5
97381	9	15348664	15348695	2	16	32	GT	11	12
97382	9	15350615	15350632	3	6	18	AGG	26	4
97384	9	15352675	15352690	2	8	16	AC	7	3
97386	9	15352726	15352749	2	12	24	AC	6	5
97392	9	15359925	15359944	2	10	20	TG	17	15
97393	9	15361258	15361279	2	11	22	GT	10	3
97401	9	15397759	15397782	3	8	24	TGC	25	8
97405	9	15403848	15403871	2	12	24	CA	19	13
97418	9	15412990	15413007	2	9	18	CT	18	9

97419	9	15413010	15413025	2	8	16	CT	6	4
97423	9	15425195	15425209	3	5	15	GAT	22	5
97424	9	15433400	15433429	3	10	30	AGA	21	6
97425	9	15439058	15439087	2	15	30	CA	12	10
97426	9	15450889	15450906	2	9	18	CA	25	10
97427	9	15451063	15451078	2	8	16	AC	16	6
97434	9	15477303	15477322	2	10	20	CA	30	4
97442	9	15494134	15494165	4	8	32	AAAG	12	9
97454	9	15520119	15520134	4	4	16	TAAA	20	6
97457	9	15520784	15520815	2	16	32	AC	14	13
97462	9	15543946	15543961	4	4	16	AAAT	28	3
97467	9	15564921	15564932	2	6	12	TG	7	6
97468	9	15565470	15565484	3	5	15	TGG	19	4
97473	9	15579124	15579135	2	6	12	TG	10	3
97476	9	15580436	15580453	2	9	18	CA	17	5
97477	9	15581559	15581600	3	14	42	CTT	16	13
97480	9	15592524	15592535	2	6	12	AG	16	3
97482	9	15594308	15594355	2	24	48	GT	9	10
97489	9	15603024	15603055	2	16	32	TC	22	6
97494	9	15607353	15607368	4	4	16	TTAA	20	3
97498	9	15608943	15608963	3	7	21	TGC	16	4
97509	9	15633289	15633330	2	21	42	TG	8	12
97512	9	15652726	15652747	2	11	22	TG	9	11
97513	9	15654765	15654798	2	17	34	TG	22	11
97514	9	15661783	15661802	4	5	20	TCTG	25	4
97521	9	15683670	15683693	4	6	24	ATCT	21	10
97523	9	15685259	15685274	4	4	16	TTTC	25	3
97538	9	15714980	15715005	2	13	26	GT	10	7
97540	9	15718368	15718387	2	10	20	CT	22	6
97544	9	15757395	15757414	2	10	20	CT	28	7
97545	9	15757695	15757730	2	18	36	GT	14	10
97553	9	15778162	15778201	2	20	40	GT	8	9
97561	9	15791338	15791359	2	11	22	TG	10	3
97570	9	15802933	15802950	2	9	18	TG	9	3
97572	9	15803901	15803926	2	13	26	TC	6	9
97575	9	15804650	15804664	3	5	15	CCT	29	4
97577	9	15809253	15809270	2	9	18	TC	16	3
97583	9	15818571	15818590	2	10	20	AC	25	13
97584	9	15819080	15819091	2	6	12	GT	6	7
97588	9	15828344	15828389	2	23	46	TG	9	9
97591	9	15834957	15835004	2	24	48	AC	15	15
97592	9	15836310	15836324	3	5	15	GAA	26	5
97593	9	15837365	15837380	2	8	16	GT	15	8
97594	9	15837639	15837672	2	17	34	GA	11	9
97598	9	15842895	15842912	2	9	18	TG	10	6
97600	9	15860462	15860487	2	13	26	AC	12	11
97604	9	15862095	15862112	2	9	18	TG	18	4
97605	9	15868316	15868327	2	6	12	CA	32	3
97607	9	15875151	15875171	3	7	21	GAG	24	4
97608	9	15880316	15880327	2	6	12	AC	24	7
97614	9	15889373	15889390	3	6	18	AGA	16	5

97618	9	15910752	15910769	2	9	18	TC	21	7
97619	9	15911321	15911346	2	13	26	GT	17	11
97622	9	15924776	15924799	4	6	24	TGTT	16	6
97623	9	15934023	15934050	2	14	28	TG	17	9
97626	9	15943115	15943154	2	20	40	CA	6	9
97627	9	15948069	15948084	2	8	16	GT	10	3
97632	9	15973267	15973288	2	11	22	TG	14	9
97635	9	15975601	15975632	2	16	32	AC	8	6
97636	9	15976243	15976260	2	9	18	CA	7	7
97638	9	15978670	15978691	2	11	22	TG	15	6
97639	9	15978811	15978826	2	8	16	TG	7	4
97641	9	15983463	15983500	2	19	38	CA	7	8
97643	9	15983753	15983778	2	13	26	GT	11	8
97647	9	15985343	15985393	3	17	51	CAT	7	9
97648	9	15985778	15985807	3	10	30	ATC	15	6
97650	9	15986174	15986191	3	6	18	CCT	26	3
97651	9	15986327	15986346	4	5	20	ATGA	16	6
97652	9	15987314	15987343	2	15	30	AC	7	8
97657	9	15990189	15990210	2	11	22	AC	15	9
97661	9	15991035	15991060	2	13	26	TG	19	10
97664	9	15994854	15994880	3	9	27	GCA	22	6
97665	9	15995832	15995843	2	6	12	TG	26	5
97666	9	15997372	15997395	3	8	24	GCA	21	7
97680	9	16022075	16022086	2	6	12	GT	25	3
97683	9	16031680	16031697	2	9	18	CA	19	9
97684	9	16032446	16032481	4	9	36	CATC	23	10
97687	9	16035473	16035498	2	13	26	CA	13	7
97692	9	16039260	16039287	2	14	28	AC	7	8
97693	9	16042049	16042068	2	10	20	TG	18	5
97694	9	16043274	16043297	2	12	24	CA	27	5
97698	9	16058992	16059005	2	7	14	CA	19	4
97700	9	16060094	16060119	2	13	26	CT	13	10
97701	9	16062550	16062571	2	11	22	GT	20	7
97711	9	16102629	16102640	2	6	12	GA	25	6
97718	9	16104849	16104862	2	7	14	AC	23	4
97738	9	16180342	16180355	2	7	14	CA	7	5
97742	9	16188676	16188715	4	10	40	TATC	18	10
97747	9	16205454	16205477	3	8	24	GCT	26	7
97749	9	16215039	16215056	3	6	18	AAC	9	3
97752	9	16215455	16215474	2	10	20	CA	17	5
97756	9	16217134	16217163	2	15	30	GT	21	8
97759	9	16223843	16223858	2	8	16	TG	31	5
97765	9	16231762	16231800	3	13	39	TCC	16	7
97766	9	16233464	16233520	3	19	57	AGC	11	8
97768	9	16234377	16234388	2	6	12	TG	23	3
97775	9	16245972	16245999	2	14	28	TC	10	6
97778	9	16256233	16256254	2	11	22	CA	13	9
97783	9	16288418	16288473	4	14	56	ATCT	21	10
97788	9	16296889	16296908	2	10	20	AT	7	4
97793	9	16306516	16306529	2	7	14	CT	8	3
97799	9	16314462	16314487	2	13	26	CA	14	8

97800	9	16318257	16318270	2	7	14	AG	30	3
97803	9	16328763	16328782	2	10	20	TG	28	3
97808	9	16346028	16346043	4	4	16	CTTT	18	3
97809	9	16348517	16348534	3	6	18	GCA	25	5
97818	9	16372522	16372541	2	10	20	AC	21	7
97819	9	16373715	16373746	2	16	32	AC	18	6
97822	9	16392439	16392456	2	9	18	TG	6	8
97823	9	16392457	16392474	2	9	18	TA	7	9
97839	9	16467036	16467087	2	26	52	TG	7	8
97846	9	16480626	16480640	3	5	15	AGC	29	6
97849	9	16482326	16482349	2	12	24	TG	20	10
97850	9	16486431	16486442	2	6	12	CA	28	4
97851	9	16488905	16488928	2	12	24	CT	8	9
97857	9	16492613	16492626	2	7	14	GT	6	4
97861	9	16499301	16499316	2	8	16	TG	31	6
97863	9	16502650	16502689	2	20	40	AG	10	10
97866	9	16507420	16507451	2	16	32	TG	17	11
97869	9	16515032	16515059	2	14	28	GA	17	10
97870	9	16522718	16522733	2	8	16	GT	13	7
97873	9	16529953	16529968	2	8	16	AT	19	3
97881	9	16541599	16541622	2	12	24	GT	19	9
97882	9	16541817	16541834	2	9	18	TG	15	6
97888	9	16560079	16560100	2	11	22	AC	8	6
97889	9	16562640	16562667	2	14	28	AC	8	9
97896	9	16567578	16567593	2	8	16	TC	19	3
97904	9	16578816	16578841	2	13	26	GT	15	6
97905	9	16579911	16579922	2	6	12	AC	24	3
97906	9	16581888	16581899	2	6	12	AC	20	3
97907	9	16581902	16581915	2	7	14	AC	24	3
97914	9	16593258	16593295	2	19	38	AC	9	7
97917	9	16594279	16594298	2	10	20	TG	10	8
97918	9	16594300	16594317	2	9	18	GT	10	7
97919	9	16594450	16594463	2	7	14	AC	20	6
97920	9	16595755	16595776	2	11	22	GT	21	5
97921	9	16595936	16595955	2	10	20	TG	19	10
97930	9	16635548	16635562	3	5	15	AAC	19	7
97934	9	16644664	16644679	2	8	16	TG	15	10
97935	9	16644680	16644691	2	6	12	TA	7	6
97936	9	16646370	16646381	2	6	12	TG	23	4
97937	9	16650699	16650714	2	8	16	TC	15	3
97938	9	16654408	16654425	2	9	18	TG	20	6
97940	9	16656982	16657007	2	13	26	GT	17	13
97946	9	16666477	16666488	2	6	12	AT	7	5
97974	9	16755775	16755790	2	8	16	GT	16	3
97976	9	16758748	16758769	2	11	22	TG	11	11
97981	9	16787211	16787224	2	7	14	TG	22	6
97986	9	16807169	16807180	2	6	12	CT	25	4
97987	9	16807181	16807192	2	6	12	CA	25	3
97990	9	16812961	16813004	2	22	44	TC	13	13
97991	9	16813180	16813201	2	11	22	TG	17	8
97992	9	16813843	16813878	2	18	36	AC	8	6

97994	9	16814769	16814792	2	12	24	GT	11	12
97995	9	16814909	16814936	2	14	28	GA	12	9
97996	9	16816585	16816604	2	10	20	CA	27	13
97999	9	16832504	16832517	2	7	14	CT	27	9
98000	9	16832629	16832642	2	7	14	AC	19	3
98001	9	16833615	16833644	2	15	30	TG	21	9
98003	9	16837525	16837542	2	9	18	CA	21	7
98005	9	16839142	16839153	2	6	12	CA	19	6
98006	9	16839155	16839170	2	8	16	AC	19	6
98008	9	16840609	16840636	2	14	28	GT	16	8
98009	9	16840788	16840807	2	10	20	TG	14	6
98012	9	16844596	16844607	2	6	12	AC	21	6
98013	9	16850923	16850938	4	4	16	TTTG	26	3
98015	9	16853333	16853376	4	11	44	ATCT	22	17
98016	9	16853506	16853525	2	10	20	AC	31	9
98018	9	16854265	16854282	2	9	18	CA	17	7
98030	9	16878011	16878026	2	8	16	CA	11	9
98032	9	16882938	16882987	2	25	50	GT	6	8
98035	9	16886362	16886399	2	19	38	TG	6	7
98037	9	16890775	16890798	2	12	24	AC	10	7
98039	9	16896271	16896288	3	6	18	AGC	23	4
98047	9	16905759	16905772	2	7	14	CT	29	4
98053	9	16920280	16920297	2	9	18	AC	17	4
98061	9	16935204	16935235	2	16	32	TG	11	10
98063	9	16942941	16942972	2	16	32	AC	13	6
98064	9	16943777	16943804	2	14	28	AC	13	10
98065	9	16945890	16945903	2	7	14	GT	17	8
98073	9	16962329	16962354	2	13	26	GT	20	10
98076	9	16967201	16967215	3	5	15	ATT	14	4
98078	9	16969136	16969159	3	8	24	GAT	21	7
98079	9	16969528	16969543	2	8	16	TG	9	8
98080	9	16969902	16969916	3	5	15	TCC	28	3
98085	9	16973200	16973219	4	5	20	ATAA	8	5
98087	9	16985906	16985923	2	9	18	TG	14	5
98088	9	16987428	16987439	2	6	12	CA	22	7
98094	9	16994622	16994653	2	16	32	GT	9	8
98098	9	17002894	17002917	2	12	24	GT	14	11
98103	9	17030544	17030573	2	15	30	CA	10	7
98131	10	33154	33165	2	6	12	AC	6	3
98132	10	34892	34915	2	12	24	AC	14	8
98133	10	37961	37978	3	6	18	AAG	25	3
98135	10	40933	40965	3	11	33	CTG	20	10
98136	10	44097	44110	2	7	14	AC	28	3
98137	10	46508	46537	2	15	30	CA	15	12
98138	10	48351	48366	2	8	16	TG	23	3
98139	10	52307	52336	2	15	30	GT	12	9
98146	10	66586	66601	2	8	16	GA	19	3
98149	10	76811	76822	2	6	12	CA	33	3
98154	10	86402	86431	2	15	30	TG	17	8
98156	10	87363	87378	2	8	16	GT	27	3
98157	10	88950	88973	2	12	24	AG	27	3

98160	10	98236	98261	2	13	26	CT	7	9
98165	10	121562	121589	2	14	28	TG	16	10
98166	10	132114	132128	3	5	15	CTG	33	5
98171	10	144554	144567	2	7	14	GT	10	4
98172	10	144570	144585	2	8	16	GT	8	5
98180	10	151524	151561	2	19	38	GA	12	11
98182	10	152721	152734	2	7	14	AT	20	3
98188	10	156857	156874	2	9	18	CT	15	6
98195	10	163344	163355	2	6	12	AT	7	5
98196	10	167384	167407	3	8	24	TTC	27	4
98199	10	180605	180616	2	6	12	GT	27	3
98200	10	185900	185915	4	4	16	TTTG	24	3
98205	10	187382	187399	2	9	18	CA	16	3
98206	10	187626	187641	2	8	16	TG	21	3
98208	10	190618	190629	2	6	12	GA	29	3
98213	10	193169	193180	2	6	12	AG	25	3
98214	10	194971	194982	2	6	12	GT	25	3
98216	10	198045	198059	3	5	15	TTG	24	3
98221	10	201159	201197	3	13	39	GAT	25	8
98225	10	205398	205429	2	16	32	CA	8	8
98233	10	209849	209860	2	6	12	GT	19	5
98239	10	217852	217867	2	8	16	TG	25	9
98243	10	220140	220157	2	9	18	GA	24	3
98244	10	220237	220250	2	7	14	AG	23	3
98245	10	220252	220263	2	6	12	GA	21	3
98246	10	223589	223606	2	9	18	TG	22	3
98251	10	226624	226643	2	10	20	CA	19	10
98254	10	231690	231709	2	10	20	AC	23	7
98256	10	232369	232382	2	7	14	GT	15	4
98257	10	232385	232396	2	6	12	GT	17	6
98262	10	258094	258114	3	7	21	GAG	21	4
98264	10	260928	260951	2	12	24	AC	18	4
98267	10	262926	262961	2	18	36	AC	9	10
98268	10	263069	263080	2	6	12	AC	24	4
98271	10	266423	266454	2	16	32	CA	19	9
98273	10	267461	267484	3	8	24	CTC	20	4
98274	10	268072	268089	2	9	18	TG	17	5
98279	10	275882	275893	2	6	12	TA	13	3
98280	10	277746	277769	2	12	24	CA	15	6
98283	10	278937	278952	2	8	16	CA	16	4
98285	10	279580	279599	2	10	20	GA	9	5
98290	10	283376	283399	2	12	24	TG	21	6
98291	10	283537	283576	2	20	40	AC	10	10
98292	10	283616	283627	2	6	12	TG	19	3
98310	10	299791	299818	4	7	28	ATGG	28	8
98316	10	306719	306734	2	8	16	GA	6	6
98327	10	340372	340407	2	18	36	CA	8	9
98329	10	346285	346311	3	9	27	AGG	25	3
98331	10	352065	352084	2	10	20	CA	14	7
98336	10	364138	364152	3	5	15	AGA	20	4
98338	10	365957	365968	2	6	12	GT	27	4

98339	10	366227	366254	2	14	28	TC	14	8
98340	10	366590	366611	2	11	22	GT	20	6
98341	10	369637	369654	2	9	18	TA	11	6
98356	10	385132	385159	2	14	28	CA	18	7
98359	10	388007	388026	2	10	20	AC	13	7
98363	10	393367	393381	3	5	15	TGA	24	3
98364	10	399983	400000	2	9	18	CA	18	3
98366	10	403267	403286	2	10	20	GA	13	4
98367	10	403497	403547	3	17	51	GAA	17	11
98368	10	403795	403824	3	10	30	CCT	11	9
98374	10	406623	406646	2	12	24	TG	19	10
98385	10	413435	413464	2	15	30	CA	12	9
98386	10	413588	413613	2	13	26	AC	12	7
98387	10	415046	415065	2	10	20	GT	14	10
98388	10	415179	415192	2	7	14	CT	16	11
98389	10	415334	415351	2	9	18	TG	18	7
98393	10	416902	416916	3	5	15	GAG	19	6
98394	10	418141	418166	2	13	26	TG	20	6
98396	10	421253	421296	2	22	44	AC	6	7
98399	10	424020	424057	2	19	38	TG	7	9
98400	10	424651	424692	2	21	42	GA	7	7
98401	10	424791	424822	2	16	32	AC	11	10
98402	10	425327	425358	4	8	32	TGTC	19	6
98405	10	427975	427988	2	7	14	TC	17	3
98407	10	430680	430705	2	13	26	TG	8	5
98409	10	431271	431306	2	18	36	AC	9	5
98411	10	432229	432260	4	8	32	CCTG	30	8
98412	10	432612	432631	2	10	20	TG	27	5
98414	10	435894	435917	3	8	24	AGG	11	4
98416	10	436498	436519	2	11	22	AG	15	6
98418	10	437582	437609	2	14	28	GT	15	5
98420	10	438676	438695	2	10	20	TG	8	6
98422	10	441093	441122	3	10	30	TGC	28	4
98425	10	443919	443942	2	12	24	AC	6	5
98428	10	445370	445391	2	11	22	GT	15	6
98432	10	445694	445717	2	12	24	CA	18	8
98440	10	448601	448615	3	5	15	TGG	24	4
98453	10	457629	457656	2	14	28	TG	12	4
98454	10	457910	457927	2	9	18	AC	27	7
98457	10	460532	460545	2	7	14	TG	13	3
98458	10	460548	460561	2	7	14	TG	19	3
98460	10	462056	462079	2	12	24	GT	11	9
98461	10	462999	463028	2	15	30	TG	13	9
98465	10	464512	464549	2	19	38	CA	9	11
98468	10	469889	469922	2	17	34	TG	9	9
98469	10	471379	471394	2	8	16	AC	22	7
98475	10	494132	494151	2	10	20	CA	24	5
98476	10	494291	494308	2	9	18	CA	14	11
98477	10	494418	494433	2	8	16	AG	24	4
98478	10	498619	498654	2	18	36	CA	21	16
98483	10	512367	512386	4	5	20	TTTA	23	5

98484	10	512962	512975	2	7	14	TG	24	3
98485	10	513074	513088	3	5	15	GTG	28	3
98486	10	513319	513348	2	15	30	TG	13	6
98487	10	513474	513485	2	6	12	AC	27	3
98488	10	513578	513595	2	9	18	TG	18	7
98489	10	513971	513991	3	7	21	TTC	21	11
98491	10	520229	520250	2	11	22	AG	16	6
98492	10	522853	522866	2	7	14	TG	16	3
98496	10	526349	526374	2	13	26	TG	9	6
98498	10	526799	526813	3	5	15	GAC	36	5
98499	10	527817	527831	3	5	15	TCA	26	4
98500	10	530241	530256	2	8	16	GT	22	10
98501	10	533322	533336	3	5	15	CCT	27	3
98502	10	536597	536622	2	13	26	CT	18	6
98503	10	537682	537695	2	7	14	CA	23	6
98504	10	541662	541697	2	18	36	TC	17	8
98505	10	542604	542633	2	15	30	CA	8	5
98507	10	544933	544976	2	22	44	GT	17	8
98513	10	551195	551220	2	13	26	CA	15	6
98514	10	553247	553262	2	8	16	CA	28	7
98515	10	554057	554080	2	12	24	CA	16	6
98523	10	564137	564148	2	6	12	TG	23	4
98524	10	565508	565521	2	7	14	GT	20	12
98529	10	569739	569772	2	17	34	CA	14	16
98535	10	579443	579456	2	7	14	TG	29	7
98538	10	589972	589995	4	6	24	AGAT	17	7
98541	10	590696	590722	3	9	27	CAT	20	7
98547	10	603024	603039	2	8	16	GT	30	3
98548	10	603190	603217	2	14	28	AC	14	8
98551	10	612297	612326	2	15	30	TG	6	10
98556	10	640827	640840	2	7	14	GT	19	4
98560	10	643020	643031	2	6	12	CA	30	4
98563	10	646256	646267	2	6	12	TG	15	3
98567	10	649513	649550	2	19	38	CA	14	6
98569	10	652304	652325	2	11	22	AC	14	5
98572	10	653549	653582	2	17	34	TG	9	7
98574	10	654681	654700	2	10	20	AC	16	7
98575	10	655718	655741	2	12	24	TG	16	9
98576	10	657549	657582	2	17	34	GT	13	15
98577	10	657694	657713	2	10	20	GT	15	3
98581	10	659473	659488	2	8	16	AC	22	4
98590	10	662610	662629	4	5	20	GTCT	16	5
98591	10	662685	662702	2	9	18	TG	20	3
98596	10	670383	670402	2	10	20	AC	13	7
98599	10	671622	671645	2	12	24	AG	19	4
98604	10	678386	678411	2	13	26	AG	22	3
98605	10	678655	678680	2	13	26	TG	24	6
98607	10	678940	678965	2	13	26	TG	16	7
98612	10	682524	682549	2	13	26	CA	10	5
98618	10	690173	690188	2	8	16	GT	20	5
98625	10	699896	699907	2	6	12	CA	32	5

98630	10	701976	701996	3	7	21	CCT	26	4
98633	10	704825	704848	2	12	24	AC	14	11
98635	10	706332	706349	3	6	18	TTA	10	3
98637	10	708586	708619	2	17	34	GA	9	9
98639	10	708922	708947	2	13	26	GT	14	10
98642	10	712093	712128	2	18	36	CA	11	11
98643	10	712790	712813	2	12	24	TG	7	5
98644	10	714195	714230	4	9	36	CTGT	13	5
98645	10	715238	715273	4	9	36	TCTT	14	8
98648	10	721947	721960	2	7	14	AT	25	5
98649	10	722273	722287	3	5	15	TGA	27	3
98652	10	726517	726542	2	13	26	TG	18	5
98653	10	726706	726735	2	15	30	GT	10	11
98657	10	728676	728703	2	14	28	TC	18	9
98658	10	728906	728937	2	16	32	CA	14	7
98662	10	735769	735780	2	6	12	TC	22	6
98667	10	738884	738895	2	6	12	AT	18	5
98670	10	741177	741197	3	7	21	AAC	14	8
98677	10	743896	743909	2	7	14	CA	25	4
98680	10	745968	745989	2	11	22	GT	21	3
98681	10	746135	746156	2	11	22	AC	14	6
98686	10	748924	748944	3	7	21	TCC	25	3
98690	10	751819	751832	2	7	14	GT	24	4
98691	10	753276	753295	2	10	20	AC	19	6
98692	10	754079	754090	2	6	12	GA	29	3
98695	10	757441	757470	2	15	30	AC	17	11
98696	10	758174	758201	2	14	28	AC	12	6
98697	10	758338	758355	2	9	18	CA	23	9
98699	10	759263	759290	2	14	28	TG	12	10
98700	10	760459	760480	2	11	22	CA	17	12
98701	10	761227	761238	2	6	12	TG	10	4
98703	10	761482	761493	2	6	12	AC	20	5
98704	10	761738	761775	2	19	38	CA	12	8
98707	10	764080	764097	3	6	18	GAG	20	3
98709	10	764612	764623	2	6	12	CA	11	3
98713	10	767028	767039	2	6	12	GA	17	8
98716	10	770158	770171	2	7	14	TG	34	3
98717	10	770246	770269	2	12	24	GT	19	6
98719	10	775127	775159	3	11	33	ATC	19	5
98721	10	776448	776467	2	10	20	GA	19	3
98722	10	776857	776872	2	8	16	CA	11	3
98731	10	783037	783066	3	10	30	TTG	14	7
98733	10	784860	784877	2	9	18	GT	18	7
98735	10	786713	786742	2	15	30	GT	20	6
98736	10	787038	787053	2	8	16	AC	20	3
98737	10	787704	787715	2	6	12	CA	23	5
98739	10	791891	791908	2	9	18	GT	13	4
98745	10	801591	801606	2	8	16	CA	24	3
98746	10	801923	801934	2	6	12	GT	22	4
98747	10	801937	801948	2	6	12	GT	18	4
98748	10	802082	802103	2	11	22	TG	20	14

98758	10	822861	822882	2	11	22	CT	6	6
98762	10	823836	823847	2	6	12	AC	24	3
98763	10	824339	824354	4	4	16	AAAT	22	10
98765	10	825785	825812	2	14	28	AC	16	6
98766	10	825855	825880	2	13	26	TG	9	3
98767	10	825902	825937	2	18	36	GA	10	8
98769	10	826256	826293	2	19	38	TG	14	13
98771	10	830602	830653	2	26	52	AC	9	13
98777	10	840372	840387	2	8	16	GT	22	11
98786	10	854904	854929	2	13	26	GT	14	4
98788	10	855103	855116	2	7	14	TG	13	4
98793	10	881232	881269	2	19	38	AC	7	6
98794	10	881931	881945	3	5	15	AGC	28	3
98798	10	888801	888814	2	7	14	CA	28	5
98805	10	897499	897512	2	7	14	CA	23	3
98806	10	897757	897784	2	14	28	CA	16	4
98812	10	906215	906264	2	25	50	CA	6	7
98813	10	909773	909792	4	5	20	TTGA	27	3
98815	10	912906	912917	2	6	12	AC	29	3
98820	10	928148	928175	2	14	28	AC	11	7
98821	10	931058	931072	3	5	15	CCA	26	5
98824	10	934836	934851	2	8	16	AC	8	6
98829	10	973606	973639	2	17	34	TG	8	6
98831	10	974096	974109	2	7	14	GT	30	3
98846	10	988970	988985	2	8	16	AC	11	6
98851	10	997148	997163	2	8	16	CA	17	3
98854	10	999606	999619	2	7	14	GT	29	4
98856	10	1002144	1002169	2	13	26	GT	20	3
98872	10	1043799	1043820	2	11	22	CA	14	11
98881	10	1063058	1063078	3	7	21	AAT	9	5
98887	10	1081904	1081929	2	13	26	CA	8	7
98889	10	1093150	1093169	2	10	20	AC	12	11
98896	10	1104520	1104533	2	7	14	AC	26	5
98899	10	1110023	1110036	2	7	14	GA	32	3
98900	10	1110664	1110683	4	5	20	TTTA	23	6
98902	10	1111243	1111264	2	11	22	TG	19	8
98909	10	1117106	1117123	2	9	18	TG	24	7
98910	10	1117126	1117137	2	6	12	TG	11	3
98912	10	1118155	1118190	2	18	36	GA	12	6
98919	10	1122675	1122692	2	9	18	GA	18	6
98920	10	1124029	1124055	3	9	27	TTG	13	11
98925	10	1127302	1127319	2	9	18	AG	20	5
98931	10	1135248	1135263	2	8	16	AC	35	3
98942	10	1151525	1151542	2	9	18	CT	13	4
98945	10	1153156	1153173	3	6	18	GAG	11	4
98947	10	1155116	1155127	2	6	12	CA	22	4
98949	10	1155330	1155347	2	9	18	TG	16	5
98953	10	1159322	1159337	2	8	16	TC	21	3
98955	10	1163360	1163407	4	12	48	GATA	21	17
98957	10	1169954	1169968	3	5	15	GTA	20	5
98959	10	1172730	1172747	2	9	18	AC	17	10

98962	10	1175440	1175471	2	16	32	TG	12	8
98975	10	1188635	1188658	2	12	24	AG	7	6
98981	10	1189956	1189982	3	9	27	TCT	18	7
98985	10	1196885	1196899	3	5	15	CTG	17	3
98992	10	1200583	1200596	2	7	14	AC	26	6
98993	10	1200787	1200808	2	11	22	TG	18	11
98995	10	1206435	1206446	2	6	12	AC	13	3
99004	10	1210897	1210916	2	10	20	TG	10	13
99008	10	1216675	1216686	2	6	12	TG	13	4
99009	10	1216688	1216701	2	7	14	GT	7	4
99013	10	1222052	1222067	2	8	16	GT	21	5
99016	10	1228552	1228571	2	10	20	CA	20	4
99018	10	1232794	1232819	2	13	26	TG	16	7
99022	10	1236762	1236775	2	7	14	GA	25	5
99023	10	1236776	1236787	2	6	12	GT	21	3
99026	10	1240724	1240735	2	6	12	GT	22	9
99044	10	1261984	1262005	2	11	22	TG	25	4
99054	10	1276887	1276900	2	7	14	TG	17	6
99055	10	1277037	1277052	2	8	16	GT	24	5
99056	10	1277218	1277233	2	8	16	AC	13	4
99057	10	1277662	1277679	2	9	18	AG	26	7
99060	10	1280952	1280967	4	4	16	TTCT	25	4
99061	10	1281029	1281042	2	7	14	TC	27	4
99062	10	1281205	1281250	2	23	46	TG	12	13
99063	10	1281360	1281375	2	8	16	TG	23	4
99064	10	1282960	1282977	3	6	18	AAC	14	3
99065	10	1283695	1283738	4	11	44	GAAA	18	6
99066	10	1283792	1283807	2	8	16	TC	17	3
99068	10	1285168	1285195	2	14	28	TG	17	6
99070	10	1287457	1287488	2	16	32	GA	11	10
99074	10	1295578	1295605	2	14	28	AC	12	12
99078	10	1298798	1298825	2	14	28	CT	17	11
99081	10	1301787	1301798	2	6	12	CA	22	8
99083	10	1303100	1303117	2	9	18	CA	15	6
99085	10	1305126	1305139	2	7	14	TG	14	3
99088	10	1306205	1306220	4	4	16	GCCA	27	3
99094	10	1311584	1311623	2	20	40	AC	7	8
99095	10	1311674	1311685	2	6	12	CA	12	3
99097	10	1312659	1312670	2	6	12	TG	10	9
99104	10	1317960	1317971	2	6	12	TC	25	3
99107	10	1318584	1318599	2	8	16	AC	23	3
99110	10	1320973	1320988	2	8	16	GT	21	4
99113	10	1321236	1321253	2	9	18	AC	15	9
99114	10	1322599	1322612	2	7	14	TG	23	6
99116	10	1323744	1323783	2	20	40	GT	9	12
99124	10	1328663	1328680	2	9	18	GT	6	5
99125	10	1328812	1328831	2	10	20	AC	23	3
99126	10	1329445	1329482	2	19	38	CA	6	5
99128	10	1330215	1330238	2	12	24	TG	14	6
99142	10	1340078	1340101	2	12	24	CA	13	10
99144	10	1340532	1340557	2	13	26	GT	13	7

99146	10	1341489	1341518	2	15	30	TG	8	7
99147	10	1341639	1341662	2	12	24	CA	20	6
99150	10	1342131	1342158	2	14	28	CA	15	21
99155	10	1343167	1343186	2	10	20	CA	27	7
99159	10	1345180	1345207	2	14	28	CA	15	9
99160	10	1345422	1345435	2	7	14	TC	21	3
99161	10	1347462	1347473	2	6	12	GT	16	8
99162	10	1347477	1347494	2	9	18	TG	18	7
99166	10	1352541	1352560	2	10	20	AC	20	6
99167	10	1353933	1353952	2	10	20	CA	11	9
99173	10	1362381	1362394	2	7	14	TG	34	3
99174	10	1363360	1363373	2	7	14	GA	16	4
99179	10	1367866	1367899	2	17	34	CA	13	8
99180	10	1368270	1368287	2	9	18	CA	13	3
99182	10	1369512	1369527	2	8	16	CA	16	7
99187	10	1370588	1370607	2	10	20	AG	14	7
99189	10	1371499	1371520	2	11	22	AC	8	4
99192	10	1373242	1373267	2	13	26	TG	12	10
99193	10	1374255	1374266	2	6	12	GT	28	5
99194	10	1374694	1374711	2	9	18	GA	16	8
99195	10	1376693	1376726	2	17	34	AG	9	6
99196	10	1377535	1377550	2	8	16	AC	26	5
99199	10	1379120	1379149	2	15	30	CT	8	3
99200	10	1379333	1379360	2	14	28	AG	25	8
99210	10	1386007	1386036	3	10	30	CTC	13	5
99211	10	1389323	1389352	3	10	30	GAA	18	6
99215	10	1391959	1391997	3	13	39	ATT	8	11
99217	10	1392352	1392366	3	5	15	AGC	11	3
99225	10	1397045	1397060	4	4	16	TTTC	21	3
99226	10	1397718	1397743	2	13	26	AC	18	8
99244	10	1432111	1432126	4	4	16	GCAC	32	5
99246	10	1432600	1432617	2	9	18	GA	12	7
99247	10	1432748	1432759	2	6	12	CT	20	3
99248	10	1434270	1434297	2	14	28	CT	6	6
99256	10	1457792	1457811	2	10	20	TG	18	8
99259	10	1466493	1466506	2	7	14	AT	15	4
99262	10	1483160	1483179	2	10	20	TC	13	7
99265	10	1486195	1486208	2	7	14	AG	16	3
99268	10	1488424	1488441	2	9	18	AC	21	5
99274	10	1496151	1496166	4	4	16	TGCG	25	7
99278	10	1504712	1504735	3	8	24	TCC	14	4
99279	10	1505527	1505548	2	11	22	GT	16	6
99294	10	1529678	1529697	2	10	20	AC	18	7
99295	10	1529796	1529819	2	12	24	AC	13	6
99299	10	1535119	1535136	2	9	18	TC	27	3
99300	10	1535831	1535844	2	7	14	GT	25	6
99301	10	1535957	1535988	2	16	32	GT	16	4
99302	10	1536108	1536127	2	10	20	GT	15	9
99304	10	1538713	1538762	2	25	50	TG	11	14
99338	10	1601358	1601375	3	6	18	AAC	9	5
99344	10	1615234	1615253	2	10	20	AC	21	6

99351	10	1626915	1626946	2	16	32	CA	7	8
99354	10	1628420	1628437	3	6	18	TAA	12	5
99355	10	1628441	1628455	3	5	15	TAA	14	8
99356	10	1633692	1633706	3	5	15	AAC	26	5
99357	10	1634492	1634507	2	8	16	AG	20	5
99359	10	1639862	1639877	2	8	16	CA	18	3
99363	10	1645781	1645800	2	10	20	GT	18	8
99366	10	1647539	1647554	4	4	16	AACA	26	4
99367	10	1647717	1647728	2	6	12	GT	26	4
99368	10	1647731	1647742	2	6	12	GT	25	6
99370	10	1648212	1648237	2	13	26	AG	25	8
99377	10	1655810	1655829	2	10	20	GT	25	4
99382	10	1667308	1667339	2	16	32	AC	23	17
99383	10	1670113	1670128	2	8	16	AC	18	5
99395	10	1711838	1711855	2	9	18	TG	24	5
99397	10	1713775	1713796	2	11	22	CA	11	10
99398	10	1714119	1714146	2	14	28	GT	22	7
99400	10	1715676	1715693	3	6	18	TTC	12	9
99401	10	1716179	1716211	3	11	33	TTC	16	6
99409	10	1725274	1725299	2	13	26	CT	13	4
99412	10	1727783	1727804	2	11	22	AC	13	7
99413	10	1727841	1727860	2	10	20	TG	15	5
99415	10	1728192	1728227	3	12	36	TCT	15	8
99418	10	1728473	1728506	2	17	34	GT	11	10
99420	10	1729508	1729531	2	12	24	GT	14	5
99433	10	1732917	1732936	4	5	20	AATA	6	3
99441	10	1734104	1734117	2	7	14	GT	6	6
99442	10	1734535	1734546	2	6	12	GT	29	4
99443	10	1734866	1734880	3	5	15	CCA	19	4
99444	10	1735563	1735580	2	9	18	AC	21	5
99447	10	1738749	1738762	2	7	14	CT	14	3
99448	10	1738763	1738776	2	7	14	CA	11	3
99452	10	1740062	1740077	4	4	16	GAGG	27	5
99456	10	1741616	1741633	3	6	18	TCC	24	7
99458	10	1742762	1742775	2	7	14	TG	16	3
99461	10	1743522	1743549	2	14	28	AC	23	11
99464	10	1745285	1745310	2	13	26	TG	12	7
99467	10	1746119	1746138	4	5	20	AAAT	23	3
99469	10	1746819	1746836	3	6	18	AAT	17	11
99470	10	1747629	1747654	2	13	26	GT	11	8
99474	10	1752203	1752240	2	19	38	CA	7	7
99477	10	1752571	1752586	2	8	16	CA	19	6
99480	10	1755029	1755040	2	6	12	GA	32	3
99488	10	1778784	1778799	2	8	16	TG	29	3
99490	10	1786919	1786930	2	6	12	TG	27	3
99500	10	1826653	1826690	2	19	38	CA	7	10
99517	10	1843414	1843427	2	7	14	GT	27	7
99518	10	1849974	1850007	2	17	34	AC	6	9
99519	10	1850383	1850410	2	14	28	GT	13	9
99522	10	1850924	1850935	2	6	12	GT	15	4
99523	10	1850961	1850978	2	9	18	GT	16	9

99524	10	1851030	1851051	2	11	22	CT	9	6
99528	10	1852216	1852239	4	6	24	ATAA	17	7
99532	10	1859752	1859772	3	7	21	GGA	24	3
99533	10	1860019	1860030	2	6	12	GT	34	3
99541	10	1867773	1867792	2	10	20	GT	10	7
99542	10	1868309	1868322	2	7	14	GA	25	3
99543	10	1869461	1869490	2	15	30	TG	8	8
99549	10	1878303	1878316	2	7	14	GT	19	3
99553	10	1885264	1885281	3	6	18	GAA	25	8
99554	10	1891882	1891917	2	18	36	TG	7	8
99556	10	1902954	1902981	2	14	28	TG	9	9
99561	10	1910815	1910826	2	6	12	TA	22	4
99563	10	1913349	1913364	2	8	16	CA	20	4
99569	10	1917472	1917492	3	7	21	GAG	21	3
99570	10	1917679	1917693	3	5	15	GAG	19	3
99575	10	1939658	1939689	4	8	32	GAAA	26	8
99577	10	1940925	1940940	2	8	16	CA	22	6
99580	10	1946756	1946776	3	7	21	ATA	26	4
99581	10	1950926	1950937	2	6	12	GT	28	4
99588	10	1973618	1973637	4	5	20	GTGC	22	9
99596	10	1992207	1992226	2	10	20	GT	11	7
99604	10	1998014	1998039	2	13	26	GT	13	8
99609	10	2003544	2003563	2	10	20	GA	20	4
99610	10	2004201	2004226	2	13	26	GT	13	9
99615	10	2006677	2006698	2	11	22	AC	6	5
99616	10	2008044	2008073	2	15	30	CA	7	6
99620	10	2017268	2017282	3	5	15	CAG	27	3
99621	10	2017688	2017703	2	8	16	GT	27	3
99622	10	2017784	2017797	2	7	14	AC	21	4
99625	10	2018516	2018527	2	6	12	AG	31	6
99626	10	2018643	2018670	2	14	28	AC	14	7
99628	10	2018802	2018815	2	7	14	AC	12	5
99632	10	2021204	2021215	2	6	12	GT	28	3
99633	10	2021679	2021698	2	10	20	GT	15	6
99637	10	2023953	2023964	2	6	12	GT	24	5
99641	10	2025865	2025886	2	11	22	AC	13	7
99642	10	2026049	2026060	2	6	12	TG	28	4
99645	10	2027650	2027677	2	14	28	CA	8	6
99646	10	2027704	2027737	2	17	34	AC	7	6
99648	10	2029548	2029561	2	7	14	GT	25	5
99650	10	2029756	2029777	2	11	22	AC	14	7
99662	10	2035419	2035456	2	19	38	TG	14	11
99672	10	2038630	2038657	2	14	28	TG	16	8
99673	10	2038838	2038877	2	20	40	AC	6	8
99677	10	2041248	2041263	2	8	16	CA	16	6
99681	10	2041777	2041800	3	8	24	AGG	18	3
99682	10	2041911	2041928	2	9	18	TG	15	4
99683	10	2042104	2042119	2	8	16	AC	23	5
99684	10	2042262	2042275	2	7	14	AC	21	3
99687	10	2044623	2044650	2	14	28	CA	8	6
99688	10	2045160	2045191	2	16	32	CA	13	9

99691	10	2045570	2045585	2	8	16	AG	10	3
99694	10	2050072	2050095	2	12	24	CA	7	8
99695	10	2051417	2051430	2	7	14	GT	21	5
99696	10	2051855	2051870	2	8	16	TG	14	3
99697	10	2051907	2051938	2	16	32	TG	9	10
99700	10	2056173	2056212	2	20	40	CA	6	7
99702	10	2059078	2059099	2	11	22	GT	13	3
99704	10	2060023	2060056	2	17	34	AC	16	6
99707	10	2062367	2062378	2	6	12	TG	26	3
99708	10	2063368	2063381	2	7	14	AC	24	4
99709	10	2063590	2063613	2	12	24	CA	11	9
99712	10	2068200	2068213	2	7	14	GT	24	3
99716	10	2081205	2081226	2	11	22	CA	20	5
99717	10	2081350	2081361	2	6	12	CA	23	4
99718	10	2083165	2083176	2	6	12	GT	28	5
99720	10	2084000	2084011	2	6	12	GT	21	4
99721	10	2084099	2084114	2	8	16	GT	22	5
99727	10	2093206	2093265	2	30	60	TG	8	10
99730	10	2096391	2096410	2	10	20	AC	16	11
99731	10	2097123	2097140	2	9	18	AC	19	6
99733	10	2097636	2097651	2	8	16	AC	10	6
99739	10	2098469	2098489	3	7	21	GGA	10	6
99742	10	2103138	2103161	4	6	24	ATGT	29	8
99746	10	2106252	2106266	3	5	15	AAG	32	3
99747	10	2106318	2106341	2	12	24	AG	16	7
99749	10	2106470	2106485	2	8	16	CA	23	3
99768	10	2123457	2123470	2	7	14	GT	18	4
99772	10	2132998	2133013	2	8	16	CA	9	3
99829	10	2180116	2180141	2	13	26	TG	21	14
99832	10	2186643	2186670	2	14	28	CA	18	8
99833	10	2186814	2186829	2	8	16	GA	24	4
99835	10	2190720	2190734	3	5	15	TCC	25	3
99836	10	2191106	2191129	2	12	24	AC	21	8
99837	10	2191168	2191195	2	14	28	CA	20	8
99838	10	2193673	2193688	2	8	16	AC	19	4
99842	10	2199413	2199428	2	8	16	GT	23	8
99843	10	2200585	2200604	2	10	20	AC	17	6
99844	10	2200774	2200801	2	14	28	CA	10	6
99845	10	2201264	2201275	2	6	12	AG	31	3
99848	10	2207455	2207490	4	9	36	CAGA	23	6
99849	10	2208121	2208138	3	6	18	GAG	24	7
99850	10	2208729	2208740	2	6	12	CA	19	15
99851	10	2208743	2208754	2	6	12	CA	17	11
99852	10	2209009	2209032	3	8	24	GCT	20	11
99853	10	2209064	2209078	3	5	15	TGC	17	5
99856	10	2209181	2209212	2	16	32	AC	16	7
99868	10	2216982	2217005	2	12	24	CA	21	4
99870	10	2220327	2220338	2	6	12	CT	21	3
99874	10	2225821	2225844	4	6	24	CTGA	24	4
99876	10	2227196	2227213	2	9	18	TG	20	8
99878	10	2229492	2229511	2	10	20	CA	26	9

99880	10	2233799	2233832	2	17	34	AC	16	9
99881	10	2238552	2238585	2	17	34	AC	7	6
99884	10	2242467	2242478	2	6	12	TG	17	3
99888	10	2258773	2258794	2	11	22	CA	14	6
99891	10	2267137	2267168	2	16	32	TG	12	9
99895	10	2285047	2285073	3	9	27	CTC	17	3
99899	10	2296535	2296558	3	8	24	GAG	28	3
99900	10	2298880	2298911	2	16	32	TG	14	8
99905	10	2304543	2304563	3	7	21	TCT	25	4
99909	10	2311523	2311544	2	11	22	AC	15	5
99914	10	2317733	2317760	4	7	28	AAGA	25	7
99925	10	2333744	2333757	2	7	14	CT	19	4
99926	10	2334038	2334055	3	6	18	GTT	18	3
99928	10	2334173	2334196	2	12	24	GT	8	7
99929	10	2334336	2334361	2	13	26	AC	15	8
99930	10	2334511	2334528	3	6	18	CTG	21	7
99935	10	2335317	2335331	3	5	15	ATC	28	3
99937	10	2335676	2335689	2	7	14	TG	25	6
99938	10	2336032	2336051	2	10	20	TG	10	7
99940	10	2341772	2341783	2	6	12	TG	29	4
99941	10	2344255	2344276	2	11	22	GT	16	10
99945	10	2349027	2349044	3	6	18	ATG	29	3
99953	10	2354918	2354945	2	14	28	TG	11	10
99960	10	2356025	2356036	2	6	12	TG	28	4
99961	10	2356255	2356282	2	14	28	CA	12	9
99962	10	2356423	2356448	2	13	26	GT	14	9
99965	10	2358888	2358909	2	11	22	AC	21	5
99967	10	2359842	2359859	2	9	18	CA	25	3
99968	10	2360191	2360226	2	18	36	GT	19	9
99969	10	2361772	2361803	2	16	32	TG	11	14
99975	10	2364639	2364659	3	7	21	GAA	27	5
99977	10	2365345	2365368	2	12	24	AC	22	10
99978	10	2366930	2366949	2	10	20	AG	18	9
99979	10	2367128	2367147	2	10	20	TG	23	6
99981	10	2369442	2369457	2	8	16	GT	20	5
99982	10	2369667	2369684	2	9	18	CA	19	4
99987	10	2375621	2375638	2	9	18	GT	28	6
99990	10	2384857	2384884	2	14	28	AC	9	7
99993	10	2387822	2387845	2	12	24	GT	22	5
99994	10	2387959	2387984	2	13	26	GT	10	5
99995	10	2388307	2388320	2	7	14	TG	24	3
99997	10	2391060	2391077	2	9	18	TG	20	4
99998	10	2392862	2392881	2	10	20	TG	15	6
99999	10	2393035	2393058	2	12	24	CA	19	11
100003	10	2400658	2400671	2	7	14	CA	26	3
100004	10	2400796	2400809	2	7	14	AC	26	4
100005	10	2400925	2400952	2	14	28	CA	18	9
100006	10	2402300	2402343	2	22	44	TC	6	8
100007	10	2402741	2402752	2	6	12	TA	16	5
100008	10	2402753	2402766	2	7	14	TG	15	6
100012	10	2408213	2408236	2	12	24	GT	15	5

100014	10	2408268	2408279	2	6	12	TG	14	4
100015	10	2408285	2408296	2	6	12	GT	23	3
100016	10	2408403	2408416	2	7	14	AC	25	3
100019	10	2414946	2414957	2	6	12	TA	17	3
100021	10	2415840	2415866	3	9	27	AGG	18	6
100022	10	2415923	2415950	2	14	28	AG	18	6
100024	10	2421061	2421088	4	7	28	AAAC	21	6
100029	10	2423171	2423200	3	10	30	TGT	26	6
100033	10	2426389	2426415	3	9	27	GAG	14	9
100035	10	2437641	2437652	2	6	12	GT	30	3
100036	10	2440348	2440379	2	16	32	GT	14	5
100037	10	2440592	2440621	2	15	30	AC	14	8
100044	10	2459653	2459668	2	8	16	AC	27	4
100051	10	2468824	2468857	2	17	34	TG	10	10
100057	10	2482662	2482699	2	19	38	CA	7	8
100067	10	2498585	2498598	2	7	14	GA	15	9
100068	10	2498626	2498637	2	6	12	TG	15	3
100069	10	2498835	2498852	3	6	18	CTT	11	3
100070	10	2498967	2498986	2	10	20	TG	12	7
100079	10	2501803	2501832	2	15	30	GT	11	17
100081	10	2504456	2504475	2	10	20	CA	11	13
100083	10	2504516	2504527	2	6	12	CA	18	4
100086	10	2505504	2505527	2	12	24	AC	14	4
100087	10	2506789	2506814	2	13	26	CA	8	10
100095	10	2511126	2511137	2	6	12	TG	28	4
100096	10	2511352	2511369	2	9	18	CA	13	9
100097	10	2511854	2511869	2	8	16	GT	22	6
100100	10	2514417	2514428	2	6	12	GT	25	3
100101	10	2515735	2515752	2	9	18	TG	24	8
100103	10	2516949	2516980	2	16	32	TG	16	9
100104	10	2517513	2517526	2	7	14	TG	10	3
100105	10	2517532	2517545	2	7	14	GT	22	3
100106	10	2519190	2519219	2	15	30	AC	15	8
100109	10	2521807	2521822	2	8	16	TG	20	5
100113	10	2523711	2523728	2	9	18	TC	22	5
100114	10	2525991	2526018	2	14	28	CA	18	5
100116	10	2529292	2529305	2	7	14	AC	28	5
100120	10	2536874	2536893	2	10	20	TC	14	4
100123	10	2538194	2538229	3	12	36	CCT	14	7
100124	10	2538440	2538459	2	10	20	CA	16	5
100127	10	2543471	2543492	2	11	22	TG	16	5
100132	10	2553582	2553597	2	8	16	TC	19	6
100135	10	2559289	2559300	2	6	12	AC	16	3
100137	10	2559841	2559862	2	11	22	AC	14	13
100139	10	2561863	2561880	2	9	18	TG	22	6
100140	10	2562452	2562493	2	21	42	CT	9	8
100141	10	2563322	2563351	2	15	30	CA	18	7
100147	10	2572582	2572601	2	10	20	GT	29	6
100155	10	2577231	2577252	2	11	22	AC	19	9
100160	10	2579273	2579286	2	7	14	AC	19	3
100161	10	2580200	2580229	2	15	30	AC	9	9

100162	10	2580417	2580448	2	16	32	CA	9	7
100163	10	2580645	2580658	2	7	14	AC	24	3
100165	10	2580944	2580967	2	12	24	CA	15	5
100166	10	2584706	2584729	2	12	24	TG	15	15
100167	10	2586040	2586065	2	13	26	TG	6	8
100174	10	2591673	2591694	2	11	22	AG	11	5
100175	10	2595056	2595071	2	8	16	TG	23	5
100176	10	2597544	2597555	2	6	12	AC	17	10
100178	10	2600026	2600037	2	6	12	AC	16	3
100180	10	2602383	2602412	2	15	30	GT	11	4
100182	10	2605882	2605897	2	8	16	AT	20	5
100183	10	2608195	2608230	2	18	36	AC	17	10
100184	10	2609125	2609138	2	7	14	GT	18	6
100187	10	2612749	2612760	2	6	12	CT	27	4
100192	10	2616314	2616331	2	9	18	TG	18	8
100196	10	2622428	2622459	2	16	32	AC	22	13
100199	10	2625156	2625171	2	8	16	AC	13	3
100201	10	2625515	2625548	2	17	34	TG	10	10
100213	10	2635805	2635828	2	12	24	AC	15	9
100215	10	2638247	2638270	3	8	24	GGA	14	5
100226	10	2655983	2656004	2	11	22	TG	15	14
100231	10	2663354	2663377	4	6	24	TTAT	12	3
100232	10	2664884	2664899	4	4	16	AGGG	29	3
100233	10	2665309	2665334	2	13	26	TG	11	11
100235	10	2669440	2669459	2	10	20	GA	19	6
100236	10	2673206	2673221	2	8	16	AC	16	7
100242	10	2694027	2694050	2	12	24	TG	19	12
100251	10	2700687	2700702	4	4	16	AAAT	14	5
100252	10	2701597	2701616	2	10	20	AC	14	7
100254	10	2711215	2711256	2	21	42	TG	6	7
100272	10	2731967	2731990	3	8	24	GCT	27	4
100273	10	2734658	2734675	2	9	18	AC	20	7
100277	10	2738940	2738957	3	6	18	TGC	22	4
100278	10	2739247	2739268	2	11	22	TG	20	9
100279	10	2740176	2740201	2	13	26	GT	22	11
100282	10	2745604	2745623	2	10	20	AC	6	3
100285	10	2752005	2752022	2	9	18	TG	21	3
100287	10	2752693	2752706	2	7	14	AC	23	5
100288	10	2753107	2753126	2	10	20	CA	20	6
100294	10	2766053	2766074	2	11	22	CA	25	5
100296	10	2778033	2778046	2	7	14	AC	27	5
100298	10	2789527	2789541	3	5	15	GAG	29	3
100305	10	2800641	2800678	2	19	38	AC	20	14
100307	10	2805711	2805732	2	11	22	CA	15	10
100311	10	2813847	2813860	2	7	14	TC	31	3
100315	10	2834146	2834161	2	8	16	TA	14	7
100316	10	2835157	2835168	2	6	12	CT	23	3
100317	10	2843022	2843037	2	8	16	TG	29	4
100325	10	2878162	2878181	2	10	20	CA	10	11
100329	10	2887912	2887925	2	7	14	GT	18	8
100333	10	2901940	2901975	2	18	36	AC	9	9

100334	10	2904976	2904993	3	6	18	ATC	25	8
100337	10	2914851	2914864	2	7	14	GT	16	6
100339	10	2926439	2926452	2	7	14	AC	24	3
100340	10	2927052	2927071	2	10	20	TG	16	4
100342	10	2929133	2929166	2	17	34	GT	18	14
100343	10	2929444	2929459	2	8	16	CA	18	10
100344	10	2929901	2929926	2	13	26	TG	12	11
100351	10	2932347	2932362	2	8	16	TC	9	6
100354	10	2943225	2943245	3	7	21	AAC	19	7
100360	10	2953727	2953744	2	9	18	TA	16	5
100364	10	2972012	2972027	2	8	16	TC	15	4
100366	10	2975471	2975484	2	7	14	TG	28	6
100370	10	2987209	2987220	2	6	12	CA	20	4
100378	10	3004700	3004735	2	18	36	TC	6	6
100383	10	3009456	3009481	2	13	26	CA	17	9
100389	10	3021015	3021036	2	11	22	TG	16	3
100394	10	3032486	3032499	2	7	14	AC	21	7
100396	10	3036944	3036959	2	8	16	GT	10	3
100398	10	3036984	3036999	4	4	16	GTGC	21	5
100400	10	3043711	3043722	2	6	12	AT	17	8
100411	10	3081592	3081609	2	9	18	CA	17	6
100412	10	3088663	3088680	2	9	18	GT	23	4
100420	10	3091941	3091952	2	6	12	GT	13	5
100421	10	3106305	3106324	2	10	20	CT	18	7
100425	10	3109379	3109400	2	11	22	CA	16	8
100426	10	3117697	3117724	2	14	28	TG	15	9
100430	10	3122053	3122070	2	9	18	CA	14	8
100431	10	3122270	3122305	2	18	36	GT	10	9
100436	10	3135452	3135469	3	6	18	GAG	13	5
100439	10	3136180	3136203	3	8	24	CCT	21	5
100445	10	3139063	3139078	2	8	16	AC	10	9
100446	10	3139080	3139091	2	6	12	CA	12	14
100447	10	3139979	3139992	2	7	14	CT	17	4
100450	10	3144403	3144418	2	8	16	TC	14	3
100454	10	3150110	3150121	2	6	12	GT	22	3
100461	10	3175749	3175760	2	6	12	AC	13	5
100469	10	3194236	3194251	2	8	16	GA	24	4
100476	10	3203137	3203160	4	6	24	AATA	24	4
100480	10	3209733	3209772	2	20	40	TG	22	14
100481	10	3210024	3210049	2	13	26	TG	9	7
100484	10	3224442	3224467	2	13	26	AC	18	8
100485	10	3224510	3224521	2	6	12	AC	28	4
100488	10	3225378	3225391	2	7	14	AC	18	6
100499	10	3266390	3266404	3	5	15	GCA	30	6
100518	10	3315344	3315367	2	12	24	GT	9	3
100521	10	3317781	3317800	2	10	20	TC	19	3
100527	10	3329328	3329351	4	6	24	ATCC	20	7
100530	10	3333421	3333452	2	16	32	GA	14	7
100536	10	3339879	3339910	2	16	32	GA	20	6
100538	10	3343901	3343918	2	9	18	TG	19	4
100546	10	3350360	3350379	2	10	20	TC	15	7

100551	10	3353575	3353589	3	5	15	TTC	29	3
100555	10	3355745	3355764	2	10	20	GT	22	4
100561	10	3373507	3373521	3	5	15	CAG	28	6
100563	10	3375428	3375467	4	10	40	TCTT	26	8
100576	10	3423057	3423096	2	20	40	AC	14	9
100581	10	3431337	3431364	2	14	28	GT	15	6
100586	10	3435575	3435594	2	10	20	TA	8	3
100592	10	3442375	3442396	2	11	22	TG	18	9
100593	10	3442522	3442539	3	6	18	GCA	27	3
100600	10	3444782	3444808	3	9	27	AGA	18	10
100603	10	3451706	3451721	2	8	16	GT	18	4
100606	10	3456799	3456826	2	14	28	TG	10	7
100607	10	3457026	3457039	2	7	14	AG	23	7
100610	10	3461997	3462018	2	11	22	AG	11	4
100611	10	3462505	3462524	2	10	20	AC	29	4
100612	10	3464976	3465003	2	14	28	TG	7	7
100617	10	3465716	3465737	2	11	22	TG	14	9
100619	10	3467419	3467432	2	7	14	GA	18	4
100620	10	3467891	3467902	2	6	12	AC	26	4
100621	10	3467998	3468041	2	22	44	TG	10	14
100622	10	3468532	3468549	2	9	18	TG	24	7
100624	10	3469940	3469955	2	8	16	AC	15	3
100625	10	3472547	3472568	2	11	22	GT	14	7
100633	10	3482995	3483018	2	12	24	AG	15	5
100634	10	3483446	3483465	2	10	20	AC	12	8
100635	10	3483732	3483753	2	11	22	TC	19	6
100637	10	3491618	3491637	2	10	20	GT	18	7
100642	10	3495195	3495232	2	19	38	CT	8	9
100646	10	3499462	3499477	2	8	16	AC	22	5
100649	10	3506140	3506163	2	12	24	CA	25	14
100650	10	3507564	3507579	2	8	16	CA	26	3
100654	10	3521773	3521792	2	10	20	CA	10	9
100655	10	3521909	3521928	2	10	20	GT	20	5
100656	10	3523206	3523223	2	9	18	CA	23	5
100665	10	3530411	3530438	2	14	28	CA	16	11
100667	10	3531405	3531430	2	13	26	TC	14	6
100670	10	3533687	3533701	3	5	15	CCT	17	3
100674	10	3536236	3536257	2	11	22	CA	7	4
100683	10	3541831	3541858	2	14	28	GA	12	3
100690	10	3544930	3544969	2	20	40	CT	7	9
100691	10	3545247	3545267	3	7	21	ACA	22	6
100699	10	3556346	3556357	2	6	12	TG	29	3
100701	10	3559855	3559892	2	19	38	CA	11	8
100702	10	3560075	3560098	3	8	24	TCC	14	4
100704	10	3561388	3561409	2	11	22	CA	21	7
100708	10	3564032	3564055	2	12	24	GT	9	9
100709	10	3565285	3565314	2	15	30	CA	9	9
100710	10	3567950	3567965	2	8	16	AC	20	6
100715	10	3573260	3573275	2	8	16	AC	16	10
100724	10	3582578	3582597	2	10	20	TC	18	3
100729	10	3587630	3587653	2	12	24	GA	12	5

100730	10	3587765	3587784	2	10	20	CA	22	3
100731	10	3587894	3587915	2	11	22	TC	26	7
100733	10	3592109	3592122	2	7	14	TC	20	3
100734	10	3592626	3592643	3	6	18	CCT	19	3
100746	10	3604808	3604839	2	16	32	TG	15	10
100753	10	3608962	3608985	2	12	24	TG	15	8
100758	10	3612595	3612614	2	10	20	CT	16	10
100761	10	3613973	3614000	2	14	28	TG	11	10
100762	10	3614167	3614196	2	15	30	AC	12	11
100779	10	3628416	3628447	2	16	32	CA	17	6
100780	10	3628552	3628563	2	6	12	CT	26	7
100781	10	3628564	3628577	2	7	14	CA	7	3
100782	10	3630348	3630361	2	7	14	GT	24	4
100785	10	3631009	3631034	2	13	26	AC	15	10
100789	10	3643615	3643630	2	8	16	AC	17	5
100791	10	3644226	3644239	2	7	14	GA	15	4
100796	10	3656053	3656080	2	14	28	TG	18	11
100798	10	3660889	3660910	2	11	22	GT	19	8
100799	10	3662476	3662493	2	9	18	GT	14	8
100801	10	3665329	3665358	2	15	30	GT	10	6
100806	10	3681670	3681681	2	6	12	TG	10	4
100809	10	3683802	3683825	4	6	24	GTGC	13	6
100810	10	3685280	3685291	2	6	12	GT	31	4
100813	10	3691959	3691974	2	8	16	GT	25	4
100814	10	3692658	3692673	2	8	16	TG	26	4
100817	10	3693646	3693661	2	8	16	AC	23	6
100818	10	3693726	3693749	2	12	24	CA	21	9
100820	10	3708290	3708303	2	7	14	AC	27	4
100823	10	3709691	3709728	2	19	38	AG	8	7
100824	10	3714439	3714454	2	8	16	CT	16	4
100829	10	3717897	3717912	2	8	16	CA	22	6
100835	10	3732511	3732538	2	14	28	AC	20	9
100839	10	3744013	3744024	2	6	12	TG	28	3
100846	10	3759623	3759642	2	10	20	AG	17	7
100848	10	3761829	3761852	2	12	24	TG	8	10
100849	10	3763514	3763535	2	11	22	TG	23	11
100850	10	3771661	3771682	2	11	22	AC	24	9
100852	10	3777260	3777271	2	6	12	TG	24	4
100853	10	3777437	3777448	2	6	12	CA	17	8
100858	10	3781300	3781323	2	12	24	AC	18	8
100870	10	3800292	3800305	2	7	14	TG	18	11
100877	10	3812206	3812219	2	7	14	AC	25	4
100878	10	3812764	3812775	2	6	12	CA	24	6
100884	10	3819385	3819426	2	21	42	AC	13	13
100886	10	3822507	3822522	2	8	16	AC	13	9
100888	10	3825541	3825560	2	10	20	GT	20	9
100889	10	3829480	3829499	2	10	20	AC	25	12
100891	10	3839852	3839877	2	13	26	TG	13	9
100893	10	3847139	3847154	4	4	16	AAAG	28	5
100894	10	3847483	3847497	3	5	15	AAG	24	3
100895	10	3847587	3847610	4	6	24	TTTG	29	4

100902	10	3859293	3859308	2	8	16	AG	28	6
100909	10	3873306	3873335	2	15	30	CA	22	11
100911	10	3876109	3876159	3	17	51	GAA	15	8
100940	10	3896320	3896363	2	22	44	CA	11	16
100943	10	3904219	3904238	2	10	20	GT	21	6
100946	10	3908272	3908301	2	15	30	TG	9	8
100947	10	3911895	3911948	2	27	54	CA	7	10
100948	10	3912083	3912098	2	8	16	GT	19	9
100950	10	3912413	3912442	2	15	30	GT	15	11
100951	10	3914393	3914426	2	17	34	TG	7	11
100957	10	3916753	3916780	2	14	28	CA	9	9
100960	10	3920179	3920192	2	7	14	GA	16	5
100961	10	3920328	3920343	4	4	16	CAGA	22	5
100964	10	3923031	3923056	2	13	26	AC	19	22
100977	10	3927537	3927562	2	13	26	TG	16	14
100978	10	3927629	3927640	2	6	12	TG	15	3
100979	10	3927790	3927823	2	17	34	AC	20	18
100985	10	3931704	3931721	3	6	18	CTC	19	4
100988	10	3935149	3935162	2	7	14	AC	25	7
100990	10	3939410	3939435	2	13	26	AC	18	6
100992	10	3941241	3941256	2	8	16	GT	26	5
100995	10	3942100	3942113	2	7	14	AG	21	9
101000	10	3945723	3945736	2	7	14	TG	21	3
101001	10	3945780	3945797	2	9	18	TG	18	3
101005	10	3947400	3947415	4	4	16	CCTC	27	3
101009	10	3949451	3949476	2	13	26	GT	16	7
101010	10	3952684	3952697	2	7	14	AG	27	3
101016	10	3958377	3958414	2	19	38	CA	13	14
101019	10	3961402	3961431	2	15	30	GA	18	8
101026	10	3965494	3965521	2	14	28	AC	12	10
101027	10	3968060	3968091	2	16	32	AC	16	6
101030	10	3973242	3973273	2	16	32	CA	10	9
101031	10	3973838	3973851	2	7	14	CT	17	15
101032	10	3973852	3973871	2	10	20	CA	17	13
101035	10	3975897	3975936	2	20	40	CA	10	12
101037	10	3980138	3980152	3	5	15	CCT	22	3
101038	10	3980406	3980421	2	8	16	AC	18	7
101039	10	3980423	3980438	2	8	16	CA	22	7
101047	10	3983337	3983368	2	16	32	CA	25	8
101051	10	3988160	3988174	3	5	15	GAG	9	6
101053	10	3988520	3988546	3	9	27	GAG	14	4
101054	10	3988631	3988664	2	17	34	TC	15	11
101055	10	3989244	3989255	2	6	12	CT	22	3
101056	10	3989729	3989746	3	6	18	CTC	13	3
101063	10	3997891	3997908	2	9	18	CA	23	8
101069	10	4002845	4002858	2	7	14	GT	16	7
101070	10	4002860	4002871	2	6	12	GT	10	3
101079	10	4010817	4010837	3	7	21	CAG	15	10
101086	10	4017435	4017452	2	9	18	AC	25	6
101092	10	4022199	4022212	2	7	14	AC	18	4
101093	10	4023983	4023996	2	7	14	AC	25	6

101096	10	4024987	4025006	4	5	20	TGTC	8	3
101098	10	4025780	4025803	2	12	24	CA	14	10
101100	10	4026643	4026662	2	10	20	GA	18	3
101101	10	4032773	4032804	2	16	32	CA	14	15
101105	10	4033030	4033045	2	8	16	AC	8	6
101106	10	4034672	4034709	2	19	38	CA	14	7
101108	10	4035110	4035123	2	7	14	GT	27	3
101112	10	4039323	4039354	2	16	32	GT	9	9
101113	10	4039481	4039508	2	14	28	AC	14	14
101114	10	4039550	4039585	2	18	36	GT	15	7
101115	10	4043199	4043210	2	6	12	AC	26	6
101116	10	4043321	4043342	2	11	22	AC	19	11
101120	10	4044225	4044252	2	14	28	TG	24	7
101121	10	4044494	4044513	2	10	20	GT	17	7
101122	10	4044641	4044670	2	15	30	TG	13	10
101123	10	4044778	4044801	4	6	24	TCCA	18	10
101125	10	4044942	4044957	4	4	16	CCAT	23	4
101126	10	4044990	4045005	4	4	16	CATC	29	3
101127	10	4047029	4047050	2	11	22	GT	23	14
101136	10	4051754	4051777	2	12	24	GT	10	4
101137	10	4051858	4051875	2	9	18	TG	19	8
101140	10	4053469	4053504	4	9	36	AAGA	16	8
101141	10	4053907	4053932	2	13	26	AG	11	9
101142	10	4054021	4054042	2	11	22	CA	10	8
101143	10	4054133	4054146	2	7	14	GT	13	4
101144	10	4054341	4054354	2	7	14	AG	14	4
101150	10	4057919	4057938	2	10	20	CA	16	13
101151	10	4058059	4058080	2	11	22	TG	23	10
101152	10	4059968	4059985	2	9	18	AC	13	6
101153	10	4061047	4061060	2	7	14	GT	23	5
101154	10	4061536	4061547	2	6	12	TG	31	5
101156	10	4066465	4066476	2	6	12	AC	20	4
101157	10	4066985	4067012	2	14	28	TG	19	16
101158	10	4067611	4067628	3	6	18	ATT	9	3
101163	10	4070693	4070704	2	6	12	AC	17	7
101164	10	4070798	4070809	2	6	12	TG	23	3
101167	10	4070971	4070986	2	8	16	TG	22	5
101168	10	4071052	4071065	2	7	14	GT	12	6
101175	10	4074791	4074824	2	17	34	AC	15	12
101176	10	4075033	4075066	2	17	34	GT	12	11
101178	10	4076793	4076812	2	10	20	TG	13	4
101181	10	4090017	4090030	2	7	14	CA	24	3
101185	10	4091778	4091799	2	11	22	GA	6	4
101191	10	4097036	4097049	2	7	14	AC	28	5
101192	10	4097100	4097123	2	12	24	GT	15	9
101195	10	4108563	4108576	2	7	14	GT	25	5
101196	10	4108609	4108620	2	6	12	CT	24	3
101202	10	4128024	4128051	2	14	28	TG	12	7
101204	10	4142917	4142944	2	14	28	CA	15	6
101206	10	4149286	4149337	4	13	52	CTTT	17	11
101207	10	4149733	4149762	2	15	30	AC	14	13

101216	10	4159965	4159982	2	9	18	TG	24	14
101217	10	4163173	4163192	2	10	20	AC	21	5
101218	10	4163527	4163538	2	6	12	TG	28	6
101222	10	4187477	4187490	2	7	14	TG	14	4
101227	10	4193917	4193934	2	9	18	AC	25	3
101228	10	4195431	4195442	2	6	12	AT	23	3
101231	10	4199228	4199247	4	5	20	CTTT	12	4
101233	10	4199636	4199655	4	5	20	TGTT	23	4
101237	10	4204502	4204527	2	13	26	CA	11	7
101240	10	4206098	4206109	2	6	12	TC	22	5
101243	10	4209245	4209258	2	7	14	TC	30	3
101249	10	4222737	4222751	3	5	15	AAC	29	5
101251	10	4223705	4223716	2	6	12	AC	21	3
101252	10	4223721	4223734	2	7	14	AC	22	4
101253	10	4226268	4226283	2	8	16	GT	24	6
101254	10	4229075	4229104	2	15	30	CA	17	10
101255	10	4229522	4229537	2	8	16	CA	26	5
101262	10	4238258	4238271	2	7	14	AC	24	10
101263	10	4240891	4240920	3	10	30	AGA	20	6
101265	10	4244419	4244436	3	6	18	TAT	31	3
101266	10	4245066	4245083	2	9	18	TG	19	6
101267	10	4245881	4245898	2	9	18	CA	20	4
101273	10	4272499	4272513	3	5	15	CTC	28	3
101275	10	4283220	4283247	2	14	28	GT	12	7
101279	10	4288877	4288924	4	12	48	ATAG	15	7
101280	10	4291733	4291750	3	6	18	TTA	18	8
101282	10	4299212	4299235	2	12	24	TG	15	11
101284	10	4311793	4311810	3	6	18	GCA	14	4
101285	10	4312144	4312155	2	6	12	TA	17	5
101298	10	4357706	4357721	2	8	16	AC	22	4
101301	10	4360702	4360731	2	15	30	GT	22	10
101302	10	4361002	4361031	2	15	30	AC	17	6
101308	10	4370119	4370132	2	7	14	CA	17	5
101311	10	4377645	4377672	2	14	28	CA	12	7
101312	10	4377716	4377743	2	14	28	AC	15	7
101313	10	4379981	4379992	2	6	12	AC	27	3
101314	10	4383350	4383361	2	6	12	TC	23	4
101315	10	4383362	4383373	2	6	12	TG	25	6
101316	10	4387794	4387819	2	13	26	CA	10	9
101317	10	4387872	4387897	2	13	26	TG	14	9
101319	10	4389479	4389490	2	6	12	AG	16	3
101323	10	4400468	4400483	2	8	16	TC	15	3
101325	10	4401799	4401810	2	6	12	AC	28	3
101335	10	4410661	4410681	3	7	21	AGT	25	4
101338	10	4420217	4420249	3	11	33	TTA	7	5
101341	10	4427520	4427535	2	8	16	TG	10	9
101342	10	4436191	4436218	2	14	28	GT	16	9
101357	10	4477559	4477584	2	13	26	TG	24	13
101366	10	4496926	4496977	4	13	52	CTAT	24	10
101373	10	4521237	4521248	2	6	12	GT	29	5
101379	10	4527153	4527168	2	8	16	TC	24	4

101381	10	4536304	4536321	3	6	18	TGA	27	3
101383	10	4541104	4541125	2	11	22	AC	16	4
101385	10	4541493	4541516	2	12	24	AC	20	6
101394	10	4551270	4551285	2	8	16	GT	9	7
101397	10	4559684	4559701	2	9	18	AC	30	11
101402	10	4564035	4564048	2	7	14	CA	23	6
101403	10	4564384	4564405	2	11	22	CA	22	9
101404	10	4564604	4564615	2	6	12	AC	23	4
101406	10	4566198	4566215	2	9	18	AC	22	5
101408	10	4566422	4566433	2	6	12	CA	27	3
101409	10	4570840	4570865	2	13	26	TG	11	8
101411	10	4571860	4571903	2	22	44	AC	8	10
101418	10	4578781	4578792	2	6	12	TC	28	3
101419	10	4586239	4586258	2	10	20	TG	25	7
101420	10	4586412	4586427	2	8	16	AC	21	3
101421	10	4588493	4588518	2	13	26	AC	9	6
101426	10	4591949	4591968	2	10	20	GT	16	5
101429	10	4594814	4594831	2	9	18	TG	10	6
101430	10	4594833	4594844	2	6	12	GT	12	6
101435	10	4599829	4599840	2	6	12	TG	26	5
101439	10	4604384	4604413	3	10	30	GAG	8	5
101442	10	4604723	4604758	2	18	36	TG	16	13
101443	10	4608134	4608145	2	6	12	CT	14	3
101444	10	4608146	4608161	2	8	16	CA	24	7
101445	10	4608428	4608451	2	12	24	AC	17	9
101447	10	4612429	4612450	2	11	22	TG	29	11
101449	10	4615172	4615185	2	7	14	AT	25	4
101450	10	4616052	4616063	2	6	12	TG	14	7
101459	10	4645623	4645640	2	9	18	AC	18	4
101466	10	4652509	4652520	2	6	12	GC	18	13
101467	10	4652521	4652540	2	10	20	AC	6	7
101477	10	4674184	4674219	2	18	36	CT	11	6
101479	10	4677231	4677244	2	7	14	CA	20	3
101485	10	4693266	4693285	2	10	20	CA	21	6
101488	10	4697107	4697124	2	9	18	AC	23	6
101489	10	4697714	4697751	2	19	38	CA	13	8
101492	10	4700757	4700776	2	10	20	AC	11	3
101493	10	4701122	4701151	2	15	30	CA	8	5
101503	10	4704431	4704458	2	14	28	TG	13	9
101504	10	4708779	4708792	2	7	14	CA	21	3
101517	10	4719797	4719826	2	15	30	AC	9	7
101519	10	4720946	4720977	2	16	32	AC	12	8
101530	10	4733932	4733961	2	15	30	TG	11	8
101533	10	4739125	4739158	2	17	34	GT	14	7
101536	10	4740076	4740109	2	17	34	AC	23	7
101552	10	4762329	4762350	2	11	22	AC	14	3
101553	10	4762563	4762600	2	19	38	AC	10	14
101554	10	4763224	4763235	2	6	12	GT	30	3
101555	10	4767699	4767714	2	8	16	TG	20	7
101560	10	4771571	4771582	2	6	12	AG	29	5
101567	10	4777146	4777163	2	9	18	AG	16	4

101569	10	4782329	4782342	2	7	14	AG	22	4
101571	10	4790808	4790835	2	14	28	CA	18	6
101572	10	4793802	4793817	2	8	16	TG	26	3
101575	10	4797276	4797293	2	9	18	AG	26	3
101579	10	4808660	4808679	2	10	20	CA	23	3
101581	10	4813135	4813146	2	6	12	TC	22	3
101582	10	4813434	4813463	2	15	30	CA	12	9
101586	10	4816206	4816217	2	6	12	TG	20	4
101587	10	4816339	4816352	2	7	14	AC	21	3
101588	10	4817718	4817737	4	5	20	AATC	20	4
101593	10	4823374	4823399	2	13	26	AC	10	4
101594	10	4823707	4823720	2	7	14	TC	28	3
101614	10	4865843	4865862	2	10	20	GA	25	4
101620	10	4867397	4867422	2	13	26	GT	11	7
101625	10	4873818	4873843	2	13	26	TG	16	9
101627	10	4877409	4877452	2	22	44	GT	7	9
101633	10	4881102	4881123	2	11	22	TG	25	9
101635	10	4882547	4882576	2	15	30	GT	21	14
101637	10	4888327	4888366	2	20	40	CT	8	7
101642	10	4890855	4890866	2	6	12	AC	25	3
101655	10	4908847	4908864	3	6	18	CTC	9	5
101661	10	4919708	4919745	2	19	38	AC	11	12
101662	10	4923296	4923313	2	9	18	CA	16	11
101669	10	4931892	4931923	2	16	32	TG	15	13
101673	10	4938484	4938499	2	8	16	GA	20	5
101679	10	4947798	4947823	2	13	26	GT	16	8
101684	10	4957726	4957745	2	10	20	CA	19	4
101690	10	4969503	4969540	2	19	38	CA	13	11
101691	10	4969701	4969716	2	8	16	CA	13	13
101692	10	4969719	4969736	2	9	18	CA	11	12
101695	10	4977542	4977569	2	14	28	AG	15	5
101697	10	4979997	4980020	2	12	24	GT	19	5
101698	10	4984295	4984322	2	14	28	GA	15	8
101699	10	4987672	4987727	4	14	56	GTGC	24	11
101700	10	4993652	4993691	2	20	40	CA	8	9
101705	10	4994289	4994320	2	16	32	CA	17	12
101706	10	4994441	4994468	2	14	28	GT	12	5
101708	10	4999928	4999945	2	9	18	AC	25	3
101710	10	5003011	5003022	2	6	12	CA	20	6
101712	10	5003247	5003268	2	11	22	AC	8	6
101713	10	5003428	5003467	2	20	40	GT	18	14
101715	10	5010495	5010506	2	6	12	TA	26	6
101717	10	5011907	5011942	2	18	36	CA	20	11
101718	10	5012874	5012905	2	16	32	GT	9	10
101719	10	5012965	5012976	2	6	12	TG	23	6
101721	10	5013661	5013674	2	7	14	AC	26	6
101736	10	5033758	5033783	2	13	26	CA	8	4
101738	10	5034119	5034132	2	7	14	GC	12	7
101739	10	5034135	5034150	2	8	16	AG	8	3
101746	10	5037161	5037187	3	9	27	GGA	6	3
101748	10	5037365	5037376	2	6	12	AC	7	8

101749	10	5037914	5037933	4	5	20	TCCA	17	11
101751	10	5040210	5040229	4	5	20	TAAA	21	5
101759	10	5042242	5042263	2	11	22	TG	16	7
101761	10	5045853	5045890	2	19	38	TG	13	13
101774	10	5072939	5072954	4	4	16	AAAT	33	3
101775	10	5074463	5074484	2	11	22	GT	24	7
101782	10	5096307	5096320	2	7	14	AC	24	3
101785	10	5099079	5099092	2	7	14	CA	25	4
101794	10	5127435	5127474	4	10	40	TTCT	24	6
101796	10	5129781	5129804	2	12	24	TG	8	14
101803	10	5138802	5138825	2	12	24	CA	20	15
101805	10	5145796	5145819	2	12	24	AC	24	14
101806	10	5148318	5148337	2	10	20	CT	19	8
101807	10	5148460	5148491	2	16	32	CA	14	15
101810	10	5160399	5160419	3	7	21	TTG	16	3
101813	10	5163564	5163578	3	5	15	AAC	34	3
101821	10	5176116	5176127	2	6	12	AG	15	3
101823	10	5176281	5176292	2	6	12	AC	23	3
101825	10	5181414	5181427	2	7	14	TG	19	6
101829	10	5182913	5182930	2	9	18	GT	25	10
101835	10	5186572	5186589	3	6	18	CAA	35	3
101841	10	5200422	5200447	2	13	26	TG	16	8
101844	10	5206487	5206508	2	11	22	AC	16	7
101854	10	5242326	5242339	2	7	14	AC	23	6
101857	10	5255154	5255165	2	6	12	AC	26	3
101859	10	5257422	5257435	2	7	14	TG	20	7
101861	10	5258947	5258960	2	7	14	CA	28	4
101868	10	5266603	5266620	2	9	18	AC	22	7
101871	10	5272475	5272495	3	7	21	TGC	22	12
101876	10	5277577	5277591	3	5	15	ACC	22	4
101877	10	5278654	5278668	3	5	15	AGG	26	3
101878	10	5283003	5283016	2	7	14	GA	29	4
101885	10	5322093	5322106	2	7	14	CA	8	4
101894	10	5341215	5341236	2	11	22	AC	15	9
101897	10	5354534	5354551	3	6	18	TCC	32	8
101901	10	5359979	5359993	3	5	15	TCT	30	3
101902	10	5362324	5362345	2	11	22	CT	23	5
101904	10	5365332	5365379	4	12	48	GAAA	12	10
101907	10	5374945	5375008	4	16	64	AAAG	27	12
101908	10	5380418	5380447	2	15	30	AC	22	10
101911	10	5387425	5387442	2	9	18	GT	26	7
101923	10	5399339	5399354	4	4	16	GAGG	21	4
101927	10	5402988	5403003	2	8	16	CA	19	5
101928	10	5403297	5403348	2	26	52	TG	13	15
101929	10	5403449	5403492	2	22	44	AC	19	18
101933	10	5408657	5408682	2	13	26	TG	15	6
101937	10	5415631	5415658	2	14	28	TG	14	9
101939	10	5417109	5417120	2	6	12	GT	23	8
101942	10	5420349	5420372	2	12	24	TG	25	17
101946	10	5428134	5428157	2	12	24	CA	11	3
101951	10	5433793	5433808	2	8	16	CA	6	4

101954	10	5437011	5437026	4	4	16	GACA	23	3
101955	10	5439054	5439071	2	9	18	AC	22	5
101958	10	5448468	5448481	2	7	14	AC	12	7
101960	10	5456757	5456784	2	14	28	CA	24	22
101970	10	5483513	5483524	2	6	12	AC	30	3
101975	10	5494999	5495014	4	4	16	AATA	28	3
101976	10	5495545	5495577	3	11	33	ATT	16	5
101981	10	5502410	5502433	4	6	24	TGCC	8	4
101989	10	5514006	5514019	2	7	14	CA	17	4
101996	10	5521322	5521337	2	8	16	GT	30	5
101998	10	5523242	5523259	3	6	18	GAG	28	5
101999	10	5525706	5525735	2	15	30	AC	12	15
102000	10	5525862	5525887	2	13	26	CA	7	7
102007	10	5528231	5528246	2	8	16	TG	18	8
102009	10	5529743	5529776	2	17	34	GT	11	11
102010	10	5530848	5530865	3	6	18	CTG	23	4
102011	10	5530923	5530943	3	7	21	GAG	24	9
102017	10	5532312	5532351	4	10	40	ATGG	27	12
102025	10	5536409	5536420	2	6	12	GT	11	3
102027	10	5536781	5536804	3	8	24	GTT	20	3
102029	10	5537751	5537772	2	11	22	AC	20	11
102033	10	5538551	5538564	2	7	14	TG	25	5
102036	10	5539936	5539961	2	13	26	TG	14	11
102040	10	5541118	5541138	3	7	21	CTC	18	3
102053	10	5559917	5559938	2	11	22	TG	24	6
102062	10	5563233	5563252	2	10	20	GT	10	12
102065	10	5566994	5567015	2	11	22	TG	19	10
102067	10	5567527	5567550	2	12	24	CA	18	9
102076	10	5577251	5577286	2	18	36	GT	14	16
102077	10	5578921	5578932	2	6	12	GT	27	3
102079	10	5581149	5581174	2	13	26	TG	16	8
102082	10	5584716	5584727	2	6	12	AC	21	7
102084	10	5585678	5585689	2	6	12	CT	27	3
102086	10	5589235	5589248	2	7	14	CA	26	5
102088	10	5592229	5592264	2	18	36	CA	11	8
102089	10	5592396	5592407	2	6	12	GT	36	3
102090	10	5592572	5592589	2	9	18	CA	24	14
102093	10	5605165	5605185	3	7	21	GAG	18	5
102095	10	5608293	5608320	2	14	28	CA	11	10
102102	10	5624486	5624517	2	16	32	AC	14	15
102104	10	5628233	5628252	4	5	20	GTCT	22	4
102113	10	5641340	5641371	2	16	32	AC	12	9
102116	10	5648454	5648469	2	8	16	AC	17	4
102119	10	5657021	5657042	2	11	22	TG	11	9
102121	10	5657235	5657248	2	7	14	TG	14	5
102122	10	5657254	5657265	2	6	12	GT	14	4
102123	10	5660676	5660691	4	4	16	AGAC	24	4
102126	10	5663063	5663074	2	6	12	CA	25	7
102127	10	5663563	5663576	2	7	14	TG	20	9
102128	10	5663581	5663596	2	8	16	GT	17	7
102132	10	5669829	5669854	2	13	26	AC	14	5

102135	10	5674449	5674464	2	8	16	TG	25	6
102136	10	5674845	5674862	2	9	18	CA	15	9
102138	10	5679681	5679698	3	6	18	CTG	16	4
102143	10	5704314	5704365	4	13	52	AGAT	21	14
102144	10	5704511	5704538	4	7	28	TTTC	17	5
102146	10	5707315	5707330	2	8	16	TG	22	3
102150	10	5724214	5724229	2	8	16	AC	23	5
102158	10	5735767	5735790	3	8	24	AAC	23	8
102161	10	5736819	5736840	2	11	22	CA	7	10
102163	10	5741590	5741604	3	5	15	CCT	30	6
102164	10	5742578	5742593	2	8	16	TC	18	4
102170	10	5770118	5770165	2	24	48	TG	8	8
102171	10	5770621	5770644	3	8	24	CCT	12	9
102172	10	5771506	5771523	3	6	18	CCT	27	7
102173	10	5775898	5775927	2	15	30	TG	17	8
102178	10	5790116	5790129	2	7	14	CA	20	9
102187	10	5821417	5821434	2	9	18	TC	20	3
102191	10	5824272	5824299	2	14	28	AC	15	8
102192	10	5825019	5825034	2	8	16	TG	27	6
102193	10	5825516	5825537	2	11	22	TG	18	6
102196	10	5829260	5829273	2	7	14	AC	19	3
102198	10	5831262	5831279	3	6	18	CCA	20	4
102199	10	5834127	5834142	2	8	16	TG	33	3
102200	10	5837222	5837235	2	7	14	GA	23	3
102201	10	5837889	5837912	3	8	24	AGG	29	4
102205	10	5857275	5857290	2	8	16	AG	14	3
102217	10	5869504	5869529	2	13	26	CA	14	10
102218	10	5881394	5881411	2	9	18	AC	24	6
102219	10	5882772	5882793	2	11	22	TG	16	8
102224	10	5897500	5897519	2	10	20	AC	19	13
102226	10	5901934	5901945	2	6	12	GT	27	3
102229	10	5904742	5904753	2	6	12	TC	15	3
102236	10	5916886	5916909	2	12	24	GT	19	6
102237	10	5917482	5917493	2	6	12	TG	24	3
102238	10	5923302	5923319	2	9	18	GT	20	6
102239	10	5924873	5924914	2	21	42	CA	6	8
102243	10	5930459	5930498	2	20	40	AC	8	8
102244	10	5935910	5935924	3	5	15	ATT	15	3
102246	10	5945807	5945868	2	31	62	AC	9	10
102247	10	5946866	5946887	2	11	22	CA	13	9
102251	10	5954288	5954301	2	7	14	TG	24	3
102258	10	5986672	5986719	2	24	48	GT	7	8
102261	10	5994211	5994224	2	7	14	TG	21	3
102262	10	5994523	5994546	2	12	24	AC	18	9
102274	10	6024069	6024083	3	5	15	GTT	29	13
102275	10	6028610	6028625	4	4	16	TTTG	9	4
102277	10	6029423	6029446	4	6	24	GGCT	31	6
102278	10	6031058	6031073	2	8	16	TC	19	6
102282	10	6032968	6032985	2	9	18	CT	14	8
102285	10	6034240	6034269	2	15	30	TG	15	7
102289	10	6037873	6037890	2	9	18	GT	20	9

102295	10	6044093	6044118	2	13	26	TG	7	6
102299	10	6051062	6051075	2	7	14	AC	26	7
102300	10	6053812	6053823	2	6	12	GT	27	3
102302	10	6055607	6055624	2	9	18	AC	14	11
102305	10	6056630	6056657	2	14	28	CA	14	13
102314	10	6073407	6073434	2	14	28	GT	12	10
102317	10	6078012	6078047	2	18	36	CA	8	9
102319	10	6081512	6081529	2	9	18	CA	25	4
102320	10	6081783	6081800	2	9	18	TG	23	8
102321	10	6086352	6086365	2	7	14	TC	28	3
102322	10	6086976	6086989	2	7	14	CT	20	3
102327	10	6091892	6091905	2	7	14	CA	22	4
102330	10	6098019	6098044	2	13	26	GT	15	11
102334	10	6100329	6100342	2	7	14	GA	25	4
102335	10	6106502	6106521	2	10	20	TG	29	4
102336	10	6106577	6106590	2	7	14	GT	23	4
102337	10	6106853	6106867	3	5	15	AGG	26	4
102341	10	6111752	6111763	2	6	12	CA	29	4
102344	10	6113873	6113888	2	8	16	TG	21	9
102345	10	6114087	6114114	2	14	28	AC	17	11
102348	10	6116224	6116235	2	6	12	GT	13	5
102349	10	6116237	6116248	2	6	12	GT	15	7
102350	10	6121476	6121501	2	13	26	GT	13	10
102361	10	6136216	6136257	2	21	42	CA	6	10
102362	10	6137687	6137719	3	11	33	TCC	13	11
102368	10	6140763	6140780	3	6	18	TCC	19	3
102371	10	6146268	6146297	2	15	30	CA	14	14
102372	10	6148211	6148242	2	16	32	CA	12	7
102374	10	6152465	6152498	2	17	34	TG	20	5
102375	10	6154322	6154355	2	17	34	GT	9	12
102377	10	6156678	6156695	2	9	18	TA	20	4
102378	10	6157062	6157076	3	5	15	GGC	30	3
102382	10	6161124	6161135	2	6	12	CT	21	4
102383	10	6161141	6161152	2	6	12	CT	20	4
102384	10	6161318	6161347	3	10	30	TTG	25	6
102390	10	6175087	6175104	2	9	18	AC	6	8
102392	10	6181622	6181673	2	26	52	AC	14	12
102394	10	6192028	6192039	2	6	12	AC	18	4
102396	10	6192366	6192381	2	8	16	GT	19	4
102398	10	6200109	6200128	2	10	20	CA	28	13
102399	10	6200343	6200366	2	12	24	TG	16	5
102404	10	6205611	6205648	2	19	38	AC	12	10
102407	10	6213790	6213821	2	16	32	GT	7	7
102410	10	6220374	6220387	2	7	14	GT	31	6
102418	10	6239560	6239571	2	6	12	AC	30	5
102419	10	6240320	6240357	2	19	38	TG	8	11
102421	10	6246197	6246216	4	5	20	ATGA	29	5
102422	10	6247498	6247509	2	6	12	CA	18	4
102424	10	6248970	6248985	2	8	16	AG	19	5
102425	10	6257694	6257713	2	10	20	GA	22	6
102426	10	6257955	6257968	2	7	14	GT	22	3

102427	10	6258043	6258054	2	6	12	GT	23	5
102431	10	6261719	6261742	2	12	24	AC	15	9
102432	10	6262354	6262371	3	6	18	CCA	6	4
102437	10	6262586	6262606	3	7	21	CAT	14	5
102441	10	6279009	6279024	2	8	16	GT	27	6
102458	10	6297606	6297633	4	7	28	GGCT	26	8
102459	10	6299361	6299374	2	7	14	AC	20	5
102461	10	6300654	6300665	2	6	12	AG	11	5
102468	10	6304914	6304925	2	6	12	TG	28	5
102469	10	6305308	6305321	2	7	14	CA	10	8
102470	10	6308299	6308319	3	7	21	TCC	28	4
102474	10	6316869	6316894	2	13	26	CA	12	7
102475	10	6317189	6317202	2	7	14	CA	20	3
102478	10	6317520	6317549	2	15	30	CA	9	6
102479	10	6319446	6319461	4	4	16	AGAC	23	4
102480	10	6323845	6323864	4	5	20	TCTT	18	4
102482	10	6327100	6327117	2	9	18	CA	11	6
102483	10	6327298	6327315	2	9	18	CA	20	5
102485	10	6329089	6329112	2	12	24	CA	20	8
102488	10	6337223	6337234	2	6	12	TA	8	3
102489	10	6339018	6339041	2	12	24	CA	17	9
102492	10	6341336	6341347	2	6	12	TG	23	3
102495	10	6344631	6344670	2	20	40	CA	15	13
102496	10	6348294	6348305	2	6	12	TC	20	6
102500	10	6355498	6355512	3	5	15	CAT	33	3
102501	10	6357676	6357689	2	7	14	GT	27	3
102508	10	6362784	6362817	2	17	34	TG	22	7
102509	10	6364385	6364398	2	7	14	TG	19	5
102515	10	6372371	6372394	4	6	24	AATG	12	7
102516	10	6373190	6373201	2	6	12	GT	18	6
102517	10	6373203	6373222	2	10	20	TG	15	6
102518	10	6373846	6373859	2	7	14	GA	31	4
102525	10	6381031	6381046	2	8	16	GT	15	6
102529	10	6383972	6383989	2	9	18	GT	17	6
102532	10	6392403	6392464	2	31	62	AC	9	9
102533	10	6394048	6394071	2	12	24	AC	11	14
102537	10	6402184	6402207	2	12	24	GT	8	3
102539	10	6406325	6406340	2	8	16	CA	21	6
102542	10	6421307	6421356	2	25	50	CA	12	11
102544	10	6423393	6423408	2	8	16	AG	23	6
102549	10	6429230	6429268	3	13	39	CTT	13	9
102550	10	6429469	6429480	2	6	12	AC	22	4
102551	10	6429482	6429495	2	7	14	CA	23	4
102553	10	6430145	6430174	2	15	30	TG	15	7
102554	10	6430356	6430385	2	15	30	TG	16	7
102555	10	6430482	6430493	2	6	12	TG	27	4
102556	10	6430745	6430786	2	21	42	AC	10	9
102557	10	6432617	6432642	2	13	26	GT	18	5
102562	10	6434517	6434550	2	17	34	TG	14	10
102565	10	6436440	6436473	2	17	34	CA	18	9
102566	10	6437401	6437428	4	7	28	GGCT	18	7

102569	10	6439486	6439503	2	9	18	AC	24	4
102572	10	6441511	6441534	2	12	24	CA	19	6
102573	10	6441989	6442006	2	9	18	AC	20	10
102581	10	6444680	6444699	2	10	20	AC	18	7
102584	10	6447343	6447356	2	7	14	GT	12	5
102586	10	6451457	6451484	2	14	28	TG	19	11
102587	10	6451647	6451666	2	10	20	GA	20	3
102588	10	6451780	6451793	2	7	14	CT	24	4
102589	10	6451848	6451871	2	12	24	GT	18	10
102590	10	6451916	6451943	2	14	28	AC	20	6
102592	10	6454029	6454056	2	14	28	AC	10	7
102593	10	6454115	6454138	2	12	24	AC	14	6
102595	10	6455819	6455862	2	22	44	AC	9	14
102596	10	6457234	6457247	2	7	14	GT	29	5
102599	10	6459414	6459441	2	14	28	AC	10	5
102601	10	6463195	6463206	2	6	12	GA	27	3
102603	10	6463480	6463513	2	17	34	CA	15	9
102604	10	6463855	6463868	2	7	14	GA	17	4
102607	10	6466611	6466628	2	9	18	CA	13	4
102608	10	6466769	6466800	2	16	32	TG	6	8
102609	10	6466974	6467017	2	22	44	TG	6	7
102611	10	6470381	6470401	3	7	21	GAG	23	5
102613	10	6474393	6474419	3	9	27	CAG	13	7
102614	10	6475432	6475471	2	20	40	TG	12	8
102615	10	6475622	6475655	2	17	34	CA	8	5
102617	10	6479320	6479333	2	7	14	AT	6	3
102618	10	6479555	6479570	2	8	16	AC	17	8
102623	10	6485662	6485675	2	7	14	AC	19	5
102629	10	6541680	6541694	3	5	15	AGA	26	7
102630	10	6542825	6542844	2	10	20	TC	26	3
102631	10	6550260	6550274	3	5	15	AAG	24	11
102632	10	6550896	6550915	2	10	20	GT	20	9
102633	10	6550986	6551013	2	14	28	GA	11	10
102639	10	6563000	6563013	2	7	14	AC	21	3
102642	10	6583009	6583026	3	6	18	AAC	25	8
102643	10	6583735	6583746	2	6	12	GC	24	5
102648	10	6593714	6593737	3	8	24	GTT	22	4
102649	10	6595091	6595108	2	9	18	AC	11	8
102656	10	6602720	6602740	3	7	21	AGG	28	8
102657	10	6604787	6604798	2	6	12	CA	24	5
102658	10	6604996	6605013	2	9	18	AC	17	9
102659	10	6605247	6605270	3	8	24	TGC	32	3
102660	10	6605471	6605486	2	8	16	AT	6	3
102679	10	6623307	6623348	2	21	42	CA	12	12
102686	10	6640583	6640616	2	17	34	AC	18	11
102689	10	6641760	6641791	2	16	32	TG	9	15
102699	10	6661799	6661812	2	7	14	TG	19	7
102714	10	6701789	6701804	4	4	16	AACA	28	3
102726	10	6719540	6719559	2	10	20	AG	21	6
102727	10	6724456	6724467	2	6	12	CA	31	11
102734	10	6739339	6739372	2	17	34	AC	6	9

102737	10	6746670	6746684	3	5	15	CAG	9	8
102743	10	6749553	6749566	2	7	14	CA	16	10
102745	10	6752014	6752025	2	6	12	AC	14	3
102750	10	6764878	6764901	4	6	24	CATA	16	4
102751	10	6772739	6772770	2	16	32	TG	16	11
102752	10	6773769	6773800	2	16	32	AC	10	11
102756	10	6777134	6777153	4	5	20	TATC	27	3
102757	10	6777694	6777708	3	5	15	TCT	19	3
102760	10	6778940	6778973	2	17	34	CA	8	9
102761	10	6780940	6780955	4	4	16	AAAT	19	3
102763	10	6787444	6787463	4	5	20	AGAC	17	5
102767	10	6791407	6791430	4	6	24	TTTA	20	4
102768	10	6791752	6791769	2	9	18	TG	21	12
102774	10	6810034	6810053	2	10	20	GT	22	11
102776	10	6813593	6813610	2	9	18	TG	19	10
102780	10	6820443	6820481	3	13	39	GTA	30	9
102784	10	6824166	6824213	2	24	48	CA	8	8
102786	10	6828502	6828525	3	8	24	CCT	12	5
102789	10	6831990	6832013	3	8	24	TGC	18	4
102791	10	6838750	6838773	4	6	24	TTGG	26	3
102792	10	6843555	6843582	2	14	28	GT	14	5
102806	10	6863539	6863562	3	8	24	GAG	24	7
102813	10	6887458	6887487	2	15	30	GT	10	9
102815	10	6887602	6887619	2	9	18	TG	19	3
102822	10	6905685	6905710	2	13	26	CA	21	7
102826	10	6911984	6911995	2	6	12	CA	22	3
102827	10	6912351	6912384	2	17	34	GT	9	10
102828	10	6912494	6912519	2	13	26	AC	10	6
102832	10	6927785	6927796	2	6	12	GA	28	3
102839	10	6958402	6958425	2	12	24	AG	14	14
102840	10	6958965	6958984	2	10	20	AC	17	8
102852	10	6997653	6997674	2	11	22	GT	9	3
102859	10	7006038	7006061	4	6	24	TTGA	20	4
102862	10	7008881	7008895	3	5	15	CTA	25	4
102865	10	7016350	7016364	3	5	15	CAA	27	3
102867	10	7029423	7029434	2	6	12	TG	24	5
102869	10	7038030	7038047	3	6	18	AGG	27	3
102870	10	7042249	7042269	3	7	21	AAG	28	4
102871	10	7042811	7042822	2	6	12	GT	35	3
102879	10	7050433	7050448	4	4	16	TCTG	25	3
102881	10	7053859	7053870	2	6	12	TG	31	3
102891	10	7068880	7068899	2	10	20	CT	6	8
102892	10	7068900	7068913	2	7	14	CA	13	16
102894	10	7070231	7070245	3	5	15	TTG	22	3
102896	10	7071345	7071360	4	4	16	TATC	20	3
102900	10	7075529	7075542	2	7	14	AG	24	4
102901	10	7077525	7077578	2	27	54	AC	18	15
102903	10	7081462	7081475	2	7	14	CA	26	5
102905	10	7082917	7082940	2	12	24	CA	14	11
102927	10	7150651	7150670	4	5	20	TCTG	17	7
102930	10	7157983	7158003	3	7	21	TCC	20	8

102936	10	7182027	7182042	4	4	16	TTTC	18	3
102942	10	7204569	7204608	4	10	40	AGAA	6	6
102944	10	7206284	7206319	4	9	36	CTAT	23	10
102952	10	7223506	7223529	2	12	24	CT	19	9
102959	10	7244875	7244892	2	9	18	AC	26	4
102962	10	7256318	7256341	2	12	24	GT	9	7
102965	10	7257268	7257295	2	14	28	TG	14	6
102966	10	7258262	7258287	2	13	26	GT	13	11
102968	10	7258429	7258440	2	6	12	CA	20	6
102971	10	7259707	7259746	2	20	40	CA	12	10
102972	10	7259899	7259922	2	12	24	CA	14	8
102977	10	7262610	7262631	2	11	22	TG	8	5
102986	10	7274439	7274456	2	9	18	TG	17	7
102987	10	7277308	7277345	2	19	38	CA	7	6
102989	10	7286388	7286413	2	13	26	TG	13	7
102992	10	7288341	7288364	2	12	24	TG	6	6
102993	10	7288573	7288586	2	7	14	CA	27	3
102997	10	7296983	7297042	2	30	60	CA	6	9
103003	10	7316936	7316953	2	9	18	GT	23	7
103004	10	7316955	7316968	2	7	14	TG	20	8
103005	10	7318784	7318805	2	11	22	CA	15	5
103006	10	7319239	7319258	2	10	20	CA	25	15
103007	10	7319443	7319470	2	14	28	CA	23	8
103010	10	7322291	7322302	2	6	12	GT	30	4
103011	10	7326954	7326968	3	5	15	GAG	36	3
103013	10	7331096	7331107	2	6	12	AG	18	11
103018	10	7338204	7338225	2	11	22	TG	19	10
103019	10	7340433	7340446	2	7	14	CT	25	3
103020	10	7340934	7340963	2	15	30	CA	8	7
103021	10	7341026	7341037	2	6	12	TC	18	4
103025	10	7343056	7343095	2	20	40	GA	13	6
103026	10	7343917	7343952	2	18	36	CA	6	10
103028	10	7345433	7345450	2	9	18	CT	19	3
103030	10	7347474	7347493	2	10	20	TG	22	5
103031	10	7348301	7348312	2	6	12	GA	12	3
103035	10	7349145	7349160	2	8	16	TC	14	3
103039	10	7350168	7350194	3	9	27	TCT	24	7
103040	10	7350534	7350579	2	23	46	TG	9	8
103046	10	7357104	7357117	2	7	14	AG	13	3
103066	10	7368342	7368359	2	9	18	AC	29	8
103067	10	7368699	7368714	2	8	16	CA	17	11
103068	10	7368716	7368727	2	6	12	AC	16	12
103071	10	7370999	7371014	2	8	16	AC	18	11
103072	10	7371473	7371492	2	10	20	AC	28	5
103074	10	7373207	7373220	2	7	14	GT	22	4
103079	10	7376714	7376725	2	6	12	GT	23	6
103081	10	7377476	7377493	2	9	18	AC	18	16
103085	10	7382073	7382084	2	6	12	CA	28	4
103090	10	7405205	7405222	2	9	18	AT	13	12
103093	10	7410811	7410832	2	11	22	CA	17	7
103097	10	7413862	7413875	2	7	14	AT	26	3

103105	10	7430039	7430054	2	8	16	CA	26	5
103107	10	7432029	7432054	2	13	26	TG	17	8
103122	10	7443867	7443880	2	7	14	CA	21	5
103123	10	7443883	7443896	2	7	14	CA	19	5
103131	10	7459855	7459904	2	25	50	CA	12	10
103133	10	7463326	7463351	2	13	26	TG	21	5
103139	10	7470419	7470438	4	5	20	GCCA	17	7
103142	10	7470884	7470895	2	6	12	GT	17	4
103143	10	7470903	7470916	2	7	14	TG	20	4
103148	10	7482264	7482275	2	6	12	AC	27	4
103149	10	7483452	7483471	4	5	20	ATGA	17	3
103162	10	7528513	7528536	2	12	24	CA	16	18
103183	10	7541728	7541745	2	9	18	GA	11	6
103186	10	7543149	7543162	2	7	14	TA	10	8
103190	10	7545313	7545330	2	9	18	AC	24	7
103191	10	7545867	7545882	2	8	16	AC	31	5
103221	10	7598602	7598619	2	9	18	TG	27	6
103228	10	7610359	7610374	2	8	16	GT	16	7
103250	10	7643666	7643683	2	9	18	TC	11	3
103257	10	7647089	7647108	4	5	20	AAAT	19	3
103280	10	7658761	7658778	2	9	18	TG	8	7
103283	10	7660154	7660181	2	14	28	GT	6	9
103288	10	7666141	7666158	3	6	18	GGA	28	5
103289	10	7666251	7666270	2	10	20	TC	17	5
103306	10	7694177	7694200	4	6	24	TCAT	24	4
103310	10	7695763	7695792	2	15	30	CA	12	13
103322	10	7719516	7719531	2	8	16	CA	16	10
103323	10	7719536	7719549	2	7	14	CA	11	7
103329	10	7731369	7731388	2	10	20	CA	8	4
103330	10	7732821	7732841	3	7	21	CAA	12	5
103338	10	7745692	7745709	2	9	18	AC	7	5
103350	10	7765597	7765636	2	20	40	CT	18	6
103373	10	7812447	7812474	2	14	28	AC	14	10
103395	10	7849106	7849125	2	10	20	TG	17	8
103396	10	7850257	7850288	4	8	32	TTCT	14	9
103399	10	7853597	7853610	2	7	14	AC	25	4
103401	10	7855618	7855645	2	14	28	AC	18	12
103402	10	7858301	7858344	2	22	44	AC	9	9
103419	10	7881695	7881716	2	11	22	AC	9	7
103428	10	7899147	7899164	2	9	18	TG	6	3
103431	10	7902226	7902245	4	5	20	AAAT	9	4
103439	10	7921665	7921696	2	16	32	AG	10	11
103440	10	7921791	7921832	2	21	42	GT	6	7
103441	10	7921980	7921995	2	8	16	GT	18	6
103447	10	7958141	7958158	2	9	18	GT	23	6
103451	10	7991323	7991366	2	22	44	TG	7	11
103459	10	8016164	8016175	2	6	12	CT	26	7
103464	10	8041080	8041127	2	24	48	GT	6	6
103465	10	8043878	8043891	2	7	14	TG	26	6
103474	10	8051250	8051261	2	6	12	AG	9	3
103476	10	8052148	8052175	2	14	28	TG	10	5

103489	10	8067207	8067221	3	5	15	AAT	16	5
103495	10	8086569	8086580	2	6	12	TA	20	4
103498	10	8090480	8090493	2	7	14	AC	28	4
103503	10	8113094	8113123	2	15	30	AC	16	11
103504	10	8115982	8115995	2	7	14	TA	13	6
103505	10	8116665	8116700	2	18	36	TG	10	10
103519	10	8147226	8147240	3	5	15	ATA	26	3
103523	10	8159525	8159554	3	10	30	CAT	14	7
103524	10	8160531	8160546	4	4	16	TCTG	18	3
103525	10	8173207	8173220	2	7	14	TA	12	4
103526	10	8174932	8174959	4	7	28	ATAA	11	3
103527	10	8184691	8184712	2	11	22	GT	17	10
103538	10	8239000	8239013	2	7	14	TG	17	7
103545	10	8250727	8250756	2	15	30	AC	8	9
103546	10	8251670	8251685	4	4	16	ATTC	23	4
103547	10	8251689	8251704	4	4	16	CATT	18	3
103550	10	8257010	8257021	2	6	12	AT	21	3
103551	10	8259752	8259763	2	6	12	TA	20	5
103559	10	8288257	8288268	2	6	12	TG	24	8
103562	10	8295890	8295925	2	18	36	CA	8	7
103563	10	8296395	8296408	2	7	14	CA	21	10
103564	10	8303931	8303942	2	6	12	CT	20	4
103568	10	8316901	8316928	2	14	28	AC	14	9
103570	10	8320764	8320791	2	14	28	TG	7	7
103578	10	8342670	8342689	2	10	20	TG	22	10
103583	10	8349912	8349923	2	6	12	AC	22	8
103584	10	8349924	8349937	2	7	14	AT	22	8
103585	10	8354160	8354177	2	9	18	CA	20	9
103587	10	8355714	8355725	2	6	12	GT	20	11
103588	10	8355728	8355741	2	7	14	GT	10	5
103590	10	8355868	8355885	2	9	18	AC	12	10
103591	10	8356027	8356038	2	6	12	AC	11	5
103592	10	8356263	8356288	2	13	26	GA	20	9
103594	10	8358836	8358847	2	6	12	TG	32	3
103595	10	8359361	8359375	3	5	15	GTT	29	4
103597	10	8367290	8367301	2	6	12	AC	27	4
103598	10	8367304	8367317	2	7	14	AC	21	3
103601	10	8368475	8368500	2	13	26	AC	7	8
103602	10	8369568	8369583	2	8	16	TG	22	8
103603	10	8370487	8370504	3	6	18	AGC	27	7
103605	10	8375672	8375701	2	15	30	AC	11	9
103606	10	8375755	8375793	3	13	39	GCT	23	6
103612	10	8380159	8380182	3	8	24	TTG	28	6
103622	10	8400685	8400700	4	4	16	TTTC	22	11
103625	10	8416572	8416617	2	23	46	CA	12	13
103633	10	8462005	8462036	2	16	32	AC	6	11
103642	10	8473249	8473274	2	13	26	GA	19	8
103644	10	8474120	8474143	3	8	24	TCC	24	3
103647	10	8478944	8478969	2	13	26	GT	24	6
103650	10	8491246	8491263	2	9	18	AC	21	9
103651	10	8491387	8491402	2	8	16	TG	9	10

103652	10	8491405	8491420	2	8	16	TG	11	9
103653	10	8492555	8492570	2	8	16	TG	20	5
103654	10	8494051	8494072	2	11	22	GT	19	12
103655	10	8495518	8495549	2	16	32	TG	14	7
103656	10	8496631	8496646	2	8	16	AC	18	5
103659	10	8496991	8497032	2	21	42	AC	15	11
103664	10	8506121	8506140	2	10	20	CA	20	5
103665	10	8507650	8507664	3	5	15	GGT	27	10
103666	10	8507741	8507760	4	5	20	GGGA	7	9
103673	10	8513303	8513324	2	11	22	AC	23	8
103678	10	8516646	8516667	2	11	22	CA	14	11
103679	10	8519009	8519022	2	7	14	AC	18	3
103680	10	8519160	8519183	4	6	24	TTTA	9	4
103681	10	8519706	8519733	4	7	28	AGTT	25	5
103682	10	8520102	8520117	4	4	16	GTTA	13	5
103690	10	8544469	8544496	4	7	28	AGTC	24	3
103691	10	8545067	8545082	4	4	16	CCAC	12	4
103692	10	8548566	8548595	2	15	30	AC	14	6
103699	10	8554212	8554223	2	6	12	GT	19	3
103700	10	8554763	8554782	2	10	20	TG	21	4
103706	10	8558307	8558328	2	11	22	TG	24	7
103707	10	8558451	8558482	2	16	32	CA	16	8
103708	10	8559211	8559226	2	8	16	GA	28	3
103715	10	8565898	8565909	2	6	12	AC	28	4
103716	10	8570014	8570057	2	22	44	TG	13	13
103721	10	8571673	8571690	2	9	18	GT	12	9
103722	10	8571693	8571708	2	8	16	GT	10	7
103724	10	8572057	8572082	2	13	26	AC	13	8
103726	10	8572872	8572921	2	25	50	CA	6	5
103727	10	8577180	8577201	2	11	22	GT	14	3
103731	10	8582890	8582903	2	7	14	TG	20	5
103732	10	8583017	8583036	2	10	20	TG	16	9
103733	10	8583345	8583360	2	8	16	TC	23	5
103734	10	8583361	8583374	2	7	14	AC	26	5
103736	10	8587050	8587061	2	6	12	TG	22	3
103737	10	8588036	8588050	3	5	15	CCG	9	3
103739	10	8591388	8591405	3	6	18	CAA	23	4
103745	10	8597562	8597577	2	8	16	TG	14	7
103746	10	8597578	8597589	2	6	12	AG	17	4
103748	10	8599712	8599727	2	8	16	GT	17	3
103751	10	8604477	8604498	2	11	22	GT	23	6
103752	10	8604543	8604570	2	14	28	TG	18	9
103761	10	8616849	8616862	2	7	14	TG	28	3
103762	10	8616895	8616906	2	6	12	TG	25	3
103764	10	8618383	8618394	2	6	12	GT	15	4
103765	10	8620006	8620021	2	8	16	TG	25	4
103770	10	8622012	8622037	2	13	26	AC	14	5
103771	10	8622482	8622499	2	9	18	AC	29	7
103774	10	8622819	8622832	2	7	14	TG	20	5
103777	10	8628341	8628376	2	18	36	TG	17	12
103782	10	8634687	8634710	2	12	24	CA	13	7

103785	10	8637161	8637188	2	14	28	CA	18	9
103786	10	8637265	8637278	2	7	14	GT	9	7
103789	10	8638536	8638565	2	15	30	GT	8	7
103791	10	8641362	8641385	2	12	24	CA	16	10
103792	10	8642471	8642486	2	8	16	TG	24	5
103793	10	8646554	8646575	2	11	22	CA	14	14
103800	10	8661984	8661998	3	5	15	TTG	23	4
103802	10	8697401	8697424	4	6	24	ATTT	25	3
103803	10	8698259	8698286	4	7	28	ATTT	20	6
103804	10	8699150	8699161	2	6	12	TG	21	9
103805	10	8700185	8700198	2	7	14	AC	27	3
103806	10	8703833	8703856	3	8	24	CCT	17	10
103807	10	8703987	8704018	2	16	32	GT	6	5
103808	10	8709559	8709572	2	7	14	AC	29	5
103812	10	8725152	8725163	2	6	12	GT	13	5
103813	10	8725168	8725181	2	7	14	GT	8	3
103814	10	8728831	8728852	2	11	22	AC	28	4
103821	10	8768924	8768938	3	5	15	ATG	31	3
103827	10	8779501	8779524	3	8	24	TCT	21	3
103832	10	8785277	8785291	3	5	15	ATC	26	5
103845	10	8817686	8817719	2	17	34	AC	9	8
103846	10	8820107	8820126	2	10	20	CA	18	9
103848	10	8820310	8820331	2	11	22	CA	14	6
103849	10	8820439	8820450	2	6	12	CA	13	3
103853	10	8820660	8820675	2	8	16	TG	16	4
103863	10	8827548	8827593	2	23	46	AC	24	11
103865	10	8831168	8831191	2	12	24	AC	11	7
103866	10	8831439	8831492	2	27	54	CA	11	14
103867	10	8831613	8831630	2	9	18	CA	26	9
103869	10	8833254	8833279	2	13	26	AC	11	7
103871	10	8836610	8836625	2	8	16	TG	19	4
103872	10	8840218	8840261	2	22	44	TG	9	10
103874	10	8842338	8842349	2	6	12	CA	29	8
103875	10	8843061	8843086	2	13	26	AC	20	5
103876	10	8845089	8845108	2	10	20	GA	17	6
103888	10	8867140	8867155	4	4	16	AAAT	28	3
103890	10	8871456	8871479	2	12	24	TG	16	12
103899	10	8896967	8896984	3	6	18	GTA	29	3
103906	10	8912151	8912165	3	5	15	GGA	24	3
103907	10	8912373	8912392	2	10	20	CA	10	7
103911	10	8920573	8920593	3	7	21	AAC	27	4
103923	10	8928646	8928671	2	13	26	TG	13	3
103926	10	8931810	8931841	2	16	32	AC	9	10
103928	10	8935976	8935999	2	12	24	CT	21	6
103930	10	8949119	8949136	2	9	18	TG	19	4
103931	10	8949458	8949473	2	8	16	TC	10	3
103936	10	8959127	8959140	2	7	14	GT	13	6
103941	10	8984237	8984257	3	7	21	TTA	11	4
103943	10	8990598	8990613	4	4	16	AACA	28	3
103949	10	9012762	9012781	4	5	20	TAAA	23	3
103956	10	9047847	9047876	2	15	30	AC	15	6

103976	10	9105128	9105157	2	15	30	CA	14	8
103978	10	9108774	9108785	2	6	12	TC	27	4
103981	10	9114380	9114393	2	7	14	TG	21	4
103986	10	9151203	9151232	2	15	30	CA	19	11
103990	10	9170378	9170399	2	11	22	TG	16	7
103998	10	9183714	9183733	2	10	20	AC	17	10
104000	10	9187505	9187530	2	13	26	AC	13	9
104001	10	9187593	9187608	2	8	16	CA	18	5
104007	10	9209247	9209274	2	14	28	CA	18	7
104009	10	9217539	9217552	2	7	14	AG	24	3
104018	10	9227899	9227912	2	7	14	AC	32	3
104020	10	9233355	9233374	2	10	20	GT	15	14
104022	10	9238916	9238933	2	9	18	AG	24	5
104023	10	9239023	9239076	2	27	54	AC	11	14
104029	10	9254972	9254989	2	9	18	GA	14	5
104035	10	9259885	9259898	2	7	14	TG	21	4
104041	10	9268134	9268157	2	12	24	AG	16	12
104044	10	9276051	9276090	4	10	40	TTGA	16	14
104053	10	9295872	9295885	2	7	14	AC	16	9
104054	10	9295887	9295898	2	6	12	CA	20	9
104067	10	9312861	9312884	4	6	24	CTTA	21	4
104070	10	9320677	9320688	2	6	12	TG	26	4
104071	10	9320690	9320705	2	8	16	GT	26	4
104072	10	9321012	9321026	3	5	15	CCA	25	4
104073	10	9322508	9322529	2	11	22	TG	24	3
104075	10	9330908	9330921	2	7	14	GT	30	3
104076	10	9332040	9332059	4	5	20	AAAC	28	5
104079	10	9334524	9334541	3	6	18	GAG	24	8
104083	10	9340404	9340437	2	17	34	TG	10	7
104084	10	9340789	9340803	3	5	15	AGC	22	4
104086	10	9345148	9345169	2	11	22	GT	15	7
104087	10	9347193	9347214	2	11	22	GT	16	5
104089	10	9353924	9353955	2	16	32	TG	13	6
104093	10	9361020	9361053	2	17	34	AG	9	10
104096	10	9361761	9361772	2	6	12	CT	16	5
104098	10	9365177	9365192	2	8	16	CA	13	10
104099	10	9365195	9365208	2	7	14	CA	16	10
104100	10	9365425	9365444	2	10	20	AC	13	8
104107	10	9378070	9378083	2	7	14	CA	23	3
104112	10	9380531	9380546	2	8	16	TG	19	16
104114	10	9381314	9381335	2	11	22	TG	20	13
104116	10	9384823	9384837	3	5	15	GGT	33	4
104121	10	9388409	9388434	2	13	26	AC	16	15
104122	10	9388562	9388585	2	12	24	GT	13	7
104123	10	9388639	9388658	2	10	20	GT	12	8
104126	10	9391189	9391204	2	8	16	AT	6	3
104130	10	9392919	9392930	2	6	12	AT	14	5
104132	10	9395569	9395583	3	5	15	TTG	23	3
104133	10	9396447	9396460	2	7	14	TC	9	3
104134	10	9396700	9396713	2	7	14	TC	24	4
104142	10	9398817	9398854	2	19	38	AG	9	12

104143	10	9399575	9399589	3	5	15	AGA	16	9
104144	10	9400121	9400146	2	13	26	GT	13	6
104145	10	9400397	9400440	4	11	44	ATCT	11	10
104149	10	9404965	9404988	2	12	24	CA	17	8
104150	10	9405577	9405596	2	10	20	AC	15	9
104154	10	9412829	9412844	2	8	16	CA	27	6
104155	10	9415698	9415715	2	9	18	AC	22	4
104156	10	9417057	9417076	2	10	20	AG	17	8
104159	10	9417289	9417314	2	13	26	TG	14	5
104161	10	9417735	9417766	2	16	32	AC	7	10
104162	10	9417968	9417988	3	7	21	AGG	20	4
104163	10	9418138	9418151	2	7	14	AC	20	3
104169	10	9419659	9419674	2	8	16	AC	25	4
104171	10	9420827	9420844	2	9	18	CA	16	6
104173	10	9423106	9423141	2	18	36	AC	13	14
104174	10	9423346	9423379	2	17	34	CA	17	16
104176	10	9423959	9423982	2	12	24	GT	17	11
104177	10	9424098	9424119	2	11	22	GT	17	8
104181	10	9426502	9426525	2	12	24	AC	23	5
104182	10	9426583	9426612	2	15	30	CA	15	8
104183	10	9426723	9426750	2	14	28	AC	21	7
104185	10	9426913	9426948	2	18	36	TG	10	6
104187	10	9428445	9428458	2	7	14	GT	29	5
104188	10	9428995	9429010	2	8	16	GT	7	10
104192	10	9430758	9430773	2	8	16	GT	25	5
104193	10	9432025	9432050	2	13	26	AC	12	9
104194	10	9435353	9435364	2	6	12	GT	25	4
104195	10	9435518	9435532	3	5	15	TTC	29	4
104196	10	9437105	9437118	2	7	14	GT	18	9
104198	10	9437815	9437828	2	7	14	CT	16	5
104199	10	9438526	9438537	2	6	12	CA	27	4
104211	10	9451785	9451810	2	13	26	CT	9	3
104214	10	9457671	9457685	3	5	15	CCA	24	3
104217	10	9459156	9459171	2	8	16	TG	19	8
104222	10	9463811	9463840	2	15	30	TG	15	9
104223	10	9463904	9463921	2	9	18	TG	22	6
104227	10	9466705	9466722	2	9	18	TG	21	8
104236	10	9482214	9482249	2	18	36	CA	16	15
104241	10	9490364	9490379	4	4	16	ACTC	30	3
104244	10	9493020	9493047	2	14	28	CA	12	4
104248	10	9497097	9497116	2	10	20	TG	16	7
104255	10	9507103	9507114	2	6	12	GA	34	3
104259	10	9512085	9512114	2	15	30	CA	14	17
104261	10	9519351	9519366	4	4	16	CAAA	27	6
104263	10	9526824	9526835	2	6	12	GT	26	5
104264	10	9528815	9528829	3	5	15	TGC	25	3
104265	10	9535028	9535048	3	7	21	GAG	15	6
104266	10	9539029	9539042	2	7	14	TG	23	8
104271	10	9546380	9546401	2	11	22	AC	17	13
104273	10	9547657	9547670	2	7	14	TG	20	3
104274	10	9547790	9547837	2	24	48	AC	6	7

104276	10	9550121	9550162	2	21	42	GT	14	12
104277	10	9550239	9550272	2	17	34	CT	19	8
104280	10	9552057	9552078	2	11	22	GT	17	9
104281	10	9554632	9554661	2	15	30	CA	15	10
104282	10	9554796	9554827	2	16	32	TG	14	12
104285	10	9560237	9560248	2	6	12	CT	11	4
104291	10	9568864	9568907	2	22	44	TG	6	10
104299	10	9576274	9576291	3	6	18	TCC	26	5
104300	10	9576604	9576629	2	13	26	GT	15	8
104310	10	9590886	9590925	2	20	40	CA	11	9
104312	10	9596042	9596059	3	6	18	AGA	17	4
104314	10	9596320	9596343	2	12	24	CA	18	11
104315	10	9596601	9596624	2	12	24	AC	15	8
104316	10	9596649	9596660	2	6	12	CA	23	4
104317	10	9596786	9596797	2	6	12	CA	17	5
104319	10	9597077	9597092	4	4	16	CACG	15	8
104320	10	9597093	9597110	2	9	18	CA	12	10
104325	10	9623856	9623881	2	13	26	AC	21	10
104326	10	9624798	9624833	2	18	36	AC	16	11
104327	10	9626115	9626128	2	7	14	AC	30	3
104328	10	9626451	9626468	3	6	18	ATT	16	4
104333	10	9650187	9650220	2	17	34	AG	12	7
104334	10	9650428	9650439	2	6	12	AC	17	9
104345	10	9665253	9665273	3	7	21	ACA	23	7
104349	10	9675885	9675906	2	11	22	CA	18	9
104350	10	9678735	9678766	4	8	32	TAGA	28	9
104356	10	9707273	9707290	2	9	18	GA	17	3
104357	10	9715405	9715424	2	10	20	AC	15	9
104359	10	9719585	9719608	2	12	24	AC	10	8
104363	10	9723889	9723916	2	14	28	AC	8	9
104369	10	9736423	9736442	2	10	20	CT	11	3
104374	10	9742485	9742498	2	7	14	CA	12	4
104376	10	9744552	9744593	2	21	42	GT	13	12
104377	10	9744915	9744930	2	8	16	TG	18	12
104381	10	9748738	9748763	2	13	26	GT	28	9
104400	10	9797445	9797498	2	27	54	AC	10	10
104403	10	9807956	9807977	2	11	22	GT	22	4
104408	10	9820995	9821015	3	7	21	TGA	15	5
104414	10	9828145	9828170	2	13	26	TG	15	8
104420	10	9845324	9845341	3	6	18	ATC	22	3
104427	10	9862142	9862156	3	5	15	CAA	27	3
104431	10	9878980	9878995	2	8	16	CT	16	5
104433	10	9880195	9880216	2	11	22	TG	13	3
104446	10	9927763	9927774	2	6	12	AC	23	5
104450	10	9937371	9937382	2	6	12	GT	15	10
104461	10	9952006	9952041	3	12	36	TCC	6	4
104465	10	9958304	9958347	2	22	44	TG	10	13
104467	10	9964049	9964062	2	7	14	CA	19	5
104472	10	9971341	9971372	4	8	32	GTCT	21	4
104479	10	9980200	9980225	2	13	26	TG	15	9
104481	10	9980358	9980371	2	7	14	GT	33	3

104486	10	10000722	10000748	3	9	27	GTA	11	10
104490	10	10015977	10016000	2	12	24	GA	10	7
104493	10	10017906	10017923	2	9	18	GT	15	8
104495	10	10018965	10018992	2	14	28	CT	14	11
104499	10	10021333	10021366	2	17	34	CA	6	9
104502	10	10022194	10022238	3	15	45	GAA	26	13
104504	10	10025105	10025116	2	6	12	TC	28	8
104505	10	10025926	10025937	2	6	12	AT	9	3
104508	10	10026876	10026921	2	23	46	CA	10	9
104511	10	10027716	10027742	3	9	27	TCC	20	9
104512	10	10029211	10029224	2	7	14	TG	23	8
104513	10	10029998	10030017	2	10	20	AC	32	5
104515	10	10035785	10035796	2	6	12	AC	18	4
104516	10	10036672	10036689	2	9	18	TG	21	4
104517	10	10036758	10036771	2	7	14	CA	22	3
104518	10	10036924	10036935	2	6	12	GT	22	3
104520	10	10037316	10037333	3	6	18	AGG	22	4
104521	10	10038011	10038026	2	8	16	AC	13	8
104523	10	10038361	10038380	2	10	20	TG	13	4
104524	10	10039069	10039080	2	6	12	CA	23	3
104536	10	10051239	10051250	2	6	12	GT	19	3
104537	10	10051328	10051351	2	12	24	CA	27	7
104538	10	10051464	10051481	3	6	18	AGA	22	4
104542	10	10053802	10053829	2	14	28	AC	22	7
104545	10	10056895	10056918	2	12	24	TG	13	6
104550	10	10063674	10063687	2	7	14	AC	26	3
104556	10	10067538	10067555	2	9	18	AC	10	3
104558	10	10070186	10070207	2	11	22	GA	14	6
104562	10	10074472	10074489	2	9	18	TG	13	7
104567	10	10089518	10089553	2	18	36	GT	19	14
104569	10	10091876	10091893	2	9	18	GT	15	9
104572	10	10093751	10093772	2	11	22	CA	22	9
104573	10	10094056	10094091	2	18	36	TG	13	10
104585	10	10110905	10110926	2	11	22	GT	6	5
104587	10	10111100	10111125	2	13	26	GT	18	16
104594	10	10120446	10120459	2	7	14	GT	17	3
104595	10	10120562	10120591	2	15	30	GT	13	7
104596	10	10121500	10121559	4	15	60	AGAA	9	7
104609	10	10130213	10130233	3	7	21	TAG	20	6
104618	10	10137233	10137264	2	16	32	GT	16	11
104621	10	10143974	10143989	2	8	16	CA	13	6
104631	10	10165409	10165438	2	15	30	GT	13	12
104636	10	10169897	10169914	2	9	18	TG	20	8
104642	10	10173607	10173620	2	7	14	GT	21	8
104643	10	10173805	10173838	2	17	34	CT	13	12
104645	10	10174052	10174063	2	6	12	CT	13	3
104646	10	10175014	10175033	2	10	20	TC	21	16
104648	10	10175791	10175810	2	10	20	CA	12	5
104657	10	10178777	10178798	2	11	22	CA	23	11
104659	10	10179453	10179467	3	5	15	TTC	14	4
104660	10	10180038	10180065	2	14	28	GT	12	8

104667	10	10180987	10181000	2	7	14	GT	27	5
104669	10	10181996	10182013	2	9	18	GT	13	5
104670	10	10182484	10182499	2	8	16	CA	21	6
104671	10	10182628	10182649	2	11	22	CA	14	9
104676	10	10187982	10188011	2	15	30	AC	10	14
104679	10	10189566	10189601	2	18	36	GT	14	14
104691	10	10198766	10198783	2	9	18	CA	6	6
104692	10	10199543	10199572	2	15	30	TG	19	7
104694	10	10200081	10200100	2	10	20	CA	14	6
104696	10	10200827	10200838	2	6	12	TG	16	4
104734	10	10233307	10233322	2	8	16	TG	18	6
104735	10	10234006	10234019	2	7	14	TG	15	6
104736	10	10234084	10234127	2	22	44	TG	16	9
104737	10	10234332	10234345	2	7	14	GT	17	10
104738	10	10235490	10235515	2	13	26	CA	17	9
104740	10	10237940	10237965	2	13	26	TG	13	8
104747	10	10246567	10246590	2	12	24	CA	7	6
104752	10	10248572	10248607	4	9	36	TCTA	6	6
104756	10	10250859	10250870	2	6	12	CA	27	11
104757	10	10251357	10251368	2	6	12	GT	10	4
104758	10	10251371	10251388	2	9	18	GT	7	5
104760	10	10252960	10252981	2	11	22	TC	16	8
104762	10	10256280	10256297	3	6	18	AAC	24	3
104768	10	10261258	10261287	2	15	30	TG	11	11
104769	10	10262983	10263012	2	15	30	GA	11	6
104773	10	10268209	10268230	2	11	22	TG	10	8
104775	10	10270243	10270256	2	7	14	CA	26	4
104776	10	10270394	10270409	2	8	16	TG	16	9
104778	10	10270646	10270673	2	14	28	CA	28	8
104794	10	10291751	10291768	2	9	18	GT	20	7
104805	10	10320864	10320887	2	12	24	AG	8	6
104811	10	10331595	10331616	2	11	22	TG	20	12
104813	10	10334770	10334785	2	8	16	CA	13	6
104822	10	10341532	10341565	2	17	34	AC	15	9
104828	10	10350765	10350776	2	6	12	TG	25	4
104837	10	10387229	10387252	2	12	24	GT	19	9
104845	10	10404225	10404238	2	7	14	CA	22	9
104849	10	10411500	10411517	2	9	18	GT	17	3
104852	10	10416150	10416163	2	7	14	TG	20	3
104856	10	10432942	10432981	2	20	40	TG	11	13
104861	10	10444378	10444392	3	5	15	TTC	6	5
104867	10	10453576	10453595	2	10	20	AC	21	9
104868	10	10459490	10459507	2	9	18	TG	24	6
104869	10	10459665	10459676	2	6	12	GT	23	6
104870	10	10461351	10461370	2	10	20	GT	18	9
104872	10	10464454	10464467	2	7	14	GT	19	7
104874	10	10468327	10468358	2	16	32	AC	19	20
104878	10	10488903	10488924	2	11	22	TC	15	15
104879	10	10489504	10489515	2	6	12	AT	11	6
104890	10	10533718	10533729	2	6	12	GT	32	3
104891	10	10533731	10533748	2	9	18	TG	26	3

104894	10	10535934	10535973	2	20	40	AC	14	12
104895	10	10537369	10537414	2	23	46	AC	8	7
104903	10	10551809	10551836	2	14	28	TG	19	9
104904	10	10552655	10552666	2	6	12	GT	29	3
104907	10	10555292	10555307	2	8	16	TG	23	6
104909	10	10558432	10558461	2	15	30	GT	14	11
104910	10	10558642	10558661	2	10	20	TG	6	5
104913	10	10561182	10561197	2	8	16	TG	22	4
104914	10	10561517	10561537	3	7	21	CTC	20	6
104916	10	10566600	10566645	2	23	46	AG	11	11
104923	10	10579050	10579065	2	8	16	GT	17	4
104935	10	10596725	10596754	2	15	30	TG	7	6
104947	10	10622862	10622887	2	13	26	TG	6	5
104959	10	10637175	10637212	2	19	38	CT	11	10
104960	10	10640589	10640628	4	10	40	CTTT	13	9
104976	10	10662212	10662237	2	13	26	CA	18	11
104979	10	10667878	10667897	2	10	20	TG	17	8
104982	10	10670310	10670327	2	9	18	AC	12	4
104983	10	10670477	10670488	2	6	12	TG	13	3
104984	10	10670491	10670502	2	6	12	TG	12	4
104989	10	10680258	10680269	2	6	12	CA	27	5
104993	10	10685516	10685537	2	11	22	TG	11	8
104994	10	10685713	10685728	2	8	16	GT	14	5
105002	10	10694690	10694711	2	11	22	TG	11	6
105005	10	10698640	10698685	2	23	46	AC	7	8
105007	10	10701480	10701509	2	15	30	AG	12	9
105012	10	10704481	10704500	2	10	20	GA	25	3
105018	10	10706780	10706823	2	22	44	CA	14	12
105019	10	10708689	10708718	2	15	30	CA	10	10
105020	10	10709069	10709086	2	9	18	CA	13	10
105021	10	10709088	10709103	2	8	16	AC	10	5
105023	10	10710661	10710672	2	6	12	AC	21	6
105024	10	10710674	10710691	2	9	18	CA	17	8
105025	10	10710865	10710882	2	9	18	TG	19	3
105027	10	10712527	10712548	2	11	22	GT	19	10
105030	10	10715178	10715215	2	19	38	GA	7	7
105031	10	10718190	10718201	2	6	12	CA	22	5
105033	10	10723345	10723382	2	19	38	GT	16	19
105035	10	10725056	10725071	2	8	16	AC	22	5
105038	10	10730322	10730345	3	8	24	TCC	22	3
105039	10	10730419	10730438	4	5	20	AAAT	16	4
105043	10	10736913	10736936	2	12	24	CA	19	7
105044	10	10739612	10739623	2	6	12	TG	35	4
105045	10	10739843	10739886	4	11	44	CTGC	29	18
105048	10	10744534	10744554	3	7	21	CTT	25	7
105051	10	10747445	10747471	3	9	27	GAG	12	7
105054	10	10751609	10751622	2	7	14	GT	24	8
105055	10	10752626	10752651	2	13	26	AC	12	10
105056	10	10752774	10752795	2	11	22	CA	21	5
105059	10	10755961	10755978	2	9	18	TA	24	5
105075	10	10774644	10774691	2	24	48	CT	10	7

105079	10	10776399	10776422	2	12	24	CA	14	8
105080	10	10777543	10777562	2	10	20	CA	19	10
105086	10	10788952	10788981	2	15	30	TG	13	13
105101	10	10796772	10796803	4	8	32	AGAA	17	11
105102	10	10797960	10797973	2	7	14	TA	21	6
105105	10	10803773	10803802	2	15	30	CA	20	13
105109	10	10810774	10810813	2	20	40	AC	8	12
105112	10	10823825	10823845	3	7	21	ACC	24	6
105115	10	10829062	10829095	2	17	34	AC	12	10
105116	10	10841450	10841473	4	6	24	AACA	25	4
105134	10	10870137	10870150	2	7	14	TA	7	3
105137	10	10875308	10875323	2	8	16	CA	23	9
105138	10	10876479	10876510	2	16	32	AC	17	8
105142	10	10884731	10884748	3	6	18	TGA	16	6
105144	10	10894519	10894539	3	7	21	CAA	21	4
105146	10	10909537	10909560	3	8	24	GAG	19	7
105150	10	10924881	10924894	2	7	14	GA	18	4
105156	10	10941738	10941763	2	13	26	AC	6	8
105158	10	10948727	10948742	2	8	16	AC	25	3
105160	10	10950560	10950577	2	9	18	TG	21	10
105161	10	10951456	10951467	2	6	12	GT	29	5
105166	10	10958167	10958182	2	8	16	CT	27	4
105169	10	10959656	10959669	2	7	14	GT	24	3
105173	10	10970145	10970164	2	10	20	CA	20	6
105175	10	10976066	10976105	2	20	40	GT	13	12
105179	10	10981247	10981264	3	6	18	GGT	25	8
105181	10	10981805	10981818	2	7	14	CA	23	7
105187	10	10990673	10990686	2	7	14	CA	22	3
105190	10	10997128	10997163	2	18	36	AC	11	6
105191	10	10997475	10997488	2	7	14	TG	29	4
105201	10	11007796	11007809	2	7	14	AC	16	7
105202	10	11007812	11007825	2	7	14	AC	15	7
105204	10	11019465	11019492	4	7	28	TACA	28	5
105215	10	11050322	11050339	3	6	18	CTC	21	3
105216	10	11050618	11050655	2	19	38	AC	14	14
105225	10	11067454	11067471	3	6	18	TAC	29	5
105227	10	11069316	11069329	2	7	14	AC	23	6
105231	10	11083397	11083440	2	22	44	GT	6	11
105236	10	11095283	11095314	4	8	32	ATCT	12	7
105240	10	11105164	11105179	2	8	16	TG	11	13
105241	10	11105180	11105195	4	4	16	CGTG	13	13
105242	10	11105225	11105236	2	6	12	GT	13	3
105247	10	11116329	11116344	2	8	16	GT	26	5
105277	10	11224963	11224984	2	11	22	CA	20	11
105281	10	11226956	11226971	2	8	16	GT	15	11
105282	10	11226974	11226989	2	8	16	GT	15	13
105284	10	11237464	11237478	3	5	15	GCA	29	6
105287	10	11240661	11240678	2	9	18	TG	21	10
105293	10	11248740	11248755	2	8	16	TG	23	14
105300	10	11256658	11256681	3	8	24	AAT	13	3
105304	10	11270915	11270930	2	8	16	CT	24	3

105306	10	11273177	11273200	3	8	24	GAA	14	12
105307	10	11276602	11276629	2	14	28	GT	23	3
105309	10	11278924	11278935	2	6	12	AG	31	6
105311	10	11294229	11294248	2	10	20	CA	19	5
105340	10	11359480	11359493	2	7	14	GT	16	5
105341	10	11359590	11359609	2	10	20	TG	17	4
105356	10	11423891	11423912	2	11	22	TG	12	9
105363	10	11432073	11432088	2	8	16	AC	24	4
105375	10	11461038	11461061	2	12	24	TG	20	15
105378	10	11466052	11466067	2	8	16	CA	18	3
105383	10	11477293	11477308	2	8	16	GT	23	7
105384	10	11477789	11477803	3	5	15	CTG	19	3
105386	10	11482703	11482718	2	8	16	CA	25	5
105389	10	11483752	11483772	3	7	21	GAA	20	3
105391	10	11484894	11484929	3	12	36	GAA	17	9
105392	10	11485212	11485231	2	10	20	TG	17	9
105393	10	11485370	11485385	4	4	16	GTGC	15	5
105394	10	11485389	11485406	2	9	18	TG	15	5
105396	10	11486387	11486412	2	13	26	TG	12	10
105398	10	11487501	11487520	4	5	20	AAAT	20	13
105401	10	11488413	11488424	2	6	12	AC	30	4
105408	10	11502339	11502350	2	6	12	AG	28	3
105410	10	11505289	11505308	2	10	20	TG	22	13
105411	10	11506376	11506405	2	15	30	TG	11	8
105412	10	11510118	11510133	4	4	16	AAAG	31	3
105413	10	11516170	11516205	2	18	36	AC	8	7
105416	10	11523256	11523275	4	5	20	TTTC	27	3
105417	10	11524455	11524478	2	12	24	AG	8	4
105420	10	11528061	11528081	3	7	21	TTG	21	5
105421	10	11528225	11528245	3	7	21	AAT	17	5
105425	10	11539202	11539217	4	4	16	AAAT	17	5
105432	10	11562364	11562377	2	7	14	CA	25	3
105433	10	11563658	11563673	4	4	16	ATTG	31	4
105436	10	11591388	11591403	4	4	16	TTCG	27	3
105437	10	11591732	11591759	4	7	28	TTTA	8	9
105444	10	11613459	11613474	4	4	16	GATT	27	4
105446	10	11617064	11617079	2	8	16	TG	16	11
105453	10	11631282	11631327	2	23	46	TG	12	8
105460	10	11651824	11651851	2	14	28	CA	26	7
105464	10	11666616	11666649	2	17	34	TG	17	14
105472	10	11724114	11724139	2	13	26	AC	12	9
105474	10	11726619	11726638	2	10	20	TC	16	9
105479	10	11756576	11756591	2	8	16	GT	19	5
105485	10	11763369	11763400	2	16	32	AC	8	7
105488	10	11765379	11765410	2	16	32	AC	17	9
105489	10	11768269	11768292	3	8	24	CAG	16	3
105494	10	11777934	11777960	3	9	27	TAG	19	12
105495	10	11779444	11779458	3	5	15	TAT	29	3
105500	10	11788751	11788774	3	8	24	CTC	19	6
105507	10	11808779	11808800	2	11	22	GT	14	8
105508	10	11813997	11814022	2	13	26	TG	15	6

105514	10	11818668	11818693	2	13	26	AC	7	5
105515	10	11821705	11821728	4	6	24	ATCC	27	4
105523	10	11834353	11834372	4	5	20	GATA	17	14
105525	10	11837826	11837861	2	18	36	AC	22	14
105531	10	11848237	11848260	4	6	24	TCAG	29	4
105540	10	11882844	11882861	2	9	18	AC	16	4
105542	10	11883784	11883803	2	10	20	CT	23	8
105545	10	11897645	11897658	2	7	14	GT	9	6
105546	10	11897839	11897852	2	7	14	TC	24	4
105548	10	11900770	11900793	2	12	24	TG	19	9
105556	10	11906138	11906163	2	13	26	CA	18	12
105558	10	11910410	11910421	2	6	12	CA	12	4
105559	10	11914505	11914516	2	6	12	AC	31	3
105566	10	11927081	11927100	2	10	20	AC	12	11
105567	10	11935204	11935235	2	16	32	GT	14	11
105575	10	11958662	11958699	2	19	38	TG	7	10
105577	10	11979464	11979475	2	6	12	AC	22	10
105580	10	11996391	11996404	2	7	14	AG	29	3
105585	10	12009650	12009664	3	5	15	GGA	18	4
105588	10	12013297	12013314	2	9	18	CA	21	7
105599	10	12032257	12032276	4	5	20	GCAC	22	15
105600	10	12032445	12032478	2	17	34	AC	10	15
105606	10	12041314	12041335	2	11	22	TG	16	12
105609	10	12047031	12047042	2	6	12	CT	25	4
105615	10	12063212	12063235	2	12	24	AG	21	6
105619	10	12069032	12069049	3	6	18	AGG	20	3
105621	10	12069882	12069897	2	8	16	TC	16	7
105622	10	12069898	12069913	4	4	16	GCTC	15	5
105625	10	12073914	12073933	2	10	20	GT	10	5
105626	10	12076481	12076496	2	8	16	AC	25	9
105629	10	12079476	12079491	2	8	16	GT	31	4
105630	10	12080681	12080696	2	8	16	CA	7	8
105634	10	12083112	12083125	2	7	14	TC	26	3
105636	10	12084736	12084761	2	13	26	CA	25	20
105643	10	12088835	12088854	4	5	20	ATAA	29	5
105644	10	12089819	12089862	2	22	44	GT	11	10
105646	10	12090482	12090507	2	13	26	TC	17	10
105649	10	12093741	12093770	2	15	30	TC	12	6
105652	10	12100838	12100859	2	11	22	AC	21	3
105653	10	12102519	12102552	2	17	34	GT	14	7
105654	10	12104178	12104199	2	11	22	AC	15	7
105656	10	12104317	12104350	2	17	34	AC	16	7
105657	10	12105664	12105687	2	12	24	TG	17	12
105658	10	12108226	12108237	2	6	12	CA	27	4
105664	10	12114770	12114817	2	24	48	AC	9	15
105667	10	12117035	12117070	2	18	36	CA	6	7
105675	10	12131072	12131098	3	9	27	AAG	23	7
105676	10	12135433	12135452	2	10	20	GT	23	9
105679	10	12140315	12140326	2	6	12	AC	22	3
105680	10	12140674	12140689	2	8	16	GT	21	10
105681	10	12140861	12140886	2	13	26	CA	19	6

105684	10	12146857	12146884	2	14	28	AC	13	7
105686	10	12154047	12154060	2	7	14	CA	21	4
105687	10	12155063	12155086	2	12	24	AG	21	13
105691	10	12159009	12159040	2	16	32	CA	14	11
105699	10	12166664	12166687	2	12	24	AC	10	10
105700	10	12166802	12166825	2	12	24	AC	9	7
105708	10	12181393	12181406	2	7	14	AC	27	6
105709	10	12182078	12182092	3	5	15	GAG	23	4
105712	10	12187754	12187773	2	10	20	TC	28	4
105713	10	12188725	12188750	2	13	26	AC	7	6
105723	10	12214705	12214722	2	9	18	GT	20	6
105724	10	12214918	12214955	2	19	38	AC	21	12
105726	10	12219049	12219062	2	7	14	CA	23	3
105728	10	12223099	12223122	4	6	24	AAAC	12	3
105729	10	12224512	12224547	2	18	36	GT	11	12
105732	10	12230302	12230317	2	8	16	AC	18	3
105734	10	12237978	12237992	3	5	15	AGG	31	6
105736	10	12240473	12240502	2	15	30	AG	8	11
105745	10	12249830	12249843	2	7	14	AC	29	7
105746	10	12250541	12250552	2	6	12	GT	25	5
105749	10	12250932	12250963	2	16	32	CA	12	6
105750	10	12251860	12251885	2	13	26	AC	16	8
105751	10	12252078	12252103	2	13	26	CA	18	8
105753	10	12254191	12254206	2	8	16	AC	26	6
105754	10	12255408	12255437	2	15	30	TG	13	9
105755	10	12255744	12255783	2	20	40	CA	14	10
105756	10	12258142	12258161	2	10	20	AC	23	11
105757	10	12259552	12259583	2	16	32	AC	11	6
105758	10	12260835	12260854	4	5	20	TGGT	29	6
105766	10	12284560	12284573	2	7	14	TG	23	7
105767	10	12286066	12286095	2	15	30	CA	22	8
105768	10	12286244	12286261	2	9	18	CA	21	8
105770	10	12291012	12291043	4	8	32	TCCA	20	4
105771	10	12292178	12292193	2	8	16	AC	18	5
105772	10	12292328	12292359	2	16	32	CA	13	12
105774	10	12295173	12295184	2	6	12	CT	17	4
105775	10	12296364	12296379	4	4	16	GTGC	23	5
105776	10	12297302	12297313	2	6	12	TG	26	4
105793	10	12309564	12309611	2	24	48	AC	8	6
105794	10	12312804	12312817	2	7	14	CA	19	4
105797	10	12314792	12314807	2	8	16	AC	17	3
105799	10	12314972	12314985	2	7	14	TG	17	3
105800	10	12315089	12315100	2	6	12	GT	22	3
105803	10	12317394	12317413	2	10	20	CA	18	7
105809	10	12321523	12321546	3	8	24	TCA	13	3
105822	10	12322300	12322335	3	12	36	TCA	13	7
105826	10	12325591	12325642	4	13	52	GAAA	19	6
105834	10	12339439	12339450	2	6	12	AC	18	5
105840	10	12352466	12352483	2	9	18	TG	20	5
105846	10	12365505	12365516	2	6	12	CA	22	4
105853	10	12381625	12381648	2	12	24	AC	22	9

105862	10	12392974	12393007	2	17	34	AC	8	9
105864	10	12396138	12396165	2	14	28	TG	7	9
105870	10	12401470	12401495	2	13	26	CT	8	3
105873	10	12403721	12403744	3	8	24	TTG	14	6
105876	10	12407063	12407074	2	6	12	CA	24	4
105887	10	12433759	12433784	2	13	26	GT	15	8
105894	10	12470618	12470637	2	10	20	GT	17	12
105898	10	12489825	12489844	4	5	20	ACTT	22	3
105903	10	12511844	12511859	2	8	16	GT	16	9
105904	10	12516633	12516650	3	6	18	CAG	30	3
105905	10	12517560	12517575	4	4	16	GAAA	18	4
105907	10	12518928	12518941	2	7	14	AC	10	10
105911	10	12519698	12519717	2	10	20	AC	23	6
105917	10	12542247	12542260	2	7	14	AC	15	4
105918	10	12544343	12544358	4	4	16	GCGT	23	6
105919	10	12544362	12544373	2	6	12	TG	23	6
105920	10	12544597	12544616	2	10	20	TG	25	6
105930	10	12567260	12567271	2	6	12	AC	17	3
105942	10	12600094	12600105	2	6	12	AT	23	5
105945	10	12605460	12605480	3	7	21	GAA	21	6
105948	10	12610919	12610936	3	6	18	ATT	28	10
105958	10	12646605	12646644	2	20	40	CA	15	9
105959	10	12660667	12660688	2	11	22	GT	20	17
105961	10	12662428	12662461	2	17	34	TG	7	4
105962	10	12664354	12664373	2	10	20	TG	15	4
105973	10	12686966	12686980	3	5	15	CCT	23	3
105975	10	12695786	12695812	3	9	27	GGA	27	4
105977	10	12705292	12705305	2	7	14	AC	15	5
105985	10	12722684	12722697	2	7	14	CA	20	3
105986	10	12728149	12728176	4	7	28	CTAT	15	8
105987	10	12730041	12730052	2	6	12	AC	21	6
105992	10	12735581	12735610	2	15	30	GT	19	12
105993	10	12735676	12735691	2	8	16	TG	16	3
105997	10	12741254	12741271	2	9	18	TG	30	5
106000	10	12754761	12754772	2	6	12	GT	22	3
106002	10	12758467	12758478	2	6	12	GT	27	3
106005	10	12762161	12762174	2	7	14	CA	16	6
106007	10	12766827	12766858	2	16	32	GA	12	9
106009	10	12770598	12770637	4	10	40	GGAT	24	9
106010	10	12775529	12775554	2	13	26	AC	16	8
106014	10	12788589	12788608	4	5	20	AAAC	21	5
106021	10	12803419	12803434	2	8	16	TG	22	5
106026	10	12820691	12820702	2	6	12	GT	28	3
106027	10	12820874	12820889	2	8	16	AC	29	4
106034	10	12830704	12830719	2	8	16	CG	17	15
106035	10	12830720	12830739	2	10	20	CA	16	13
106036	10	12831582	12831605	2	12	24	TG	24	7
106039	10	12842519	12842538	2	10	20	AC	19	6
106040	10	12843470	12843495	2	13	26	AC	7	10
106043	10	12843781	12843792	2	6	12	TG	20	4
106047	10	12845855	12845866	2	6	12	AC	9	3

106049	10	12848014	12848027	2	7	14	CT	14	5
106052	10	12861936	12861950	3	5	15	GCT	30	3
106053	10	12870293	12870318	2	13	26	AG	18	7
106059	10	12875348	12875369	2	11	22	CA	16	9
106062	10	12877676	12877695	2	10	20	GT	23	7
106065	10	12878677	12878696	2	10	20	TG	15	4
106066	10	12884746	12884761	4	4	16	AAAT	18	4
106069	10	12898568	12898587	2	10	20	TG	13	9
106078	10	12940241	12940266	2	13	26	TG	8	7
106079	10	12941317	12941338	2	11	22	TC	23	7
106081	10	12946722	12946748	3	9	27	CAA	27	6
106084	10	12948390	12948411	2	11	22	TG	19	7
106098	10	12997593	12997608	4	4	16	ACAG	33	3
106120	10	13017034	13017063	3	10	30	CTA	23	7
106132	10	13049147	13049160	2	7	14	TC	19	3
106133	10	13049806	13049817	2	6	12	AG	18	4
106134	10	13050350	13050381	2	16	32	CA	14	11
106137	10	13054578	13054595	2	9	18	GT	20	5
106153	10	13099079	13099123	3	15	45	GCA	15	17
106154	10	13100670	13100685	4	4	16	CTGC	30	5
106157	10	13102805	13102832	2	14	28	TG	8	8
106176	10	13145781	13145806	2	13	26	TG	11	5
106177	10	13145956	13145971	2	8	16	TC	14	3
106184	10	13151535	13151546	2	6	12	GA	25	3
106189	10	13160945	13160956	2	6	12	TG	26	3
106190	10	13165033	13165047	3	5	15	CAT	30	4
106191	10	13165583	13165598	2	8	16	CA	9	5
106195	10	13178715	13178738	4	6	24	ATTC	24	3
106207	10	13197368	13197379	2	6	12	CA	22	6
106215	10	13219817	13219831	3	5	15	TTC	26	4
106245	10	13249869	13249896	2	14	28	GT	17	11
106250	10	13253213	13253242	2	15	30	CA	12	9
106252	10	13253604	13253623	2	10	20	CA	10	11
106254	10	13257940	13257959	2	10	20	TG	16	8
106255	10	13258072	13258091	2	10	20	GT	15	8
106257	10	13258179	13258190	2	6	12	AC	26	3
106261	10	13265340	13265351	2	6	12	TG	28	3
106263	10	13265407	13265418	2	6	12	CA	28	3
106267	10	13267720	13267739	2	10	20	GT	11	4
106268	10	13268170	13268183	2	7	14	TG	30	4
106271	10	13283268	13283282	3	5	15	CAT	11	3
106274	10	13285718	13285749	2	16	32	TG	7	10
106275	10	13290048	13290085	2	19	38	TG	7	5
106278	10	13295970	13295989	2	10	20	TG	7	5
106289	10	13325621	13325658	2	19	38	GT	17	12
106290	10	13332201	13332222	2	11	22	TG	22	13
106296	10	13332785	13332796	2	6	12	CA	26	3
106299	10	13337231	13337252	2	11	22	GT	16	5
106302	10	13341715	13341736	2	11	22	GA	13	5
106311	10	13352628	13352643	2	8	16	AC	14	6
106317	10	13357503	13357516	2	7	14	CG	31	11

106336	10	13395813	13395838	2	13	26	AC	18	9
106337	10	13396037	13396062	2	13	26	AC	14	7
106341	10	13398651	13398672	2	11	22	TG	11	6
106342	10	13398771	13398803	3	11	33	TTG	21	4
106365	10	13412622	13412651	2	15	30	TG	9	8
106372	10	13417245	13417262	3	6	18	AGG	16	5
106375	10	13419956	13419967	2	6	12	TC	26	3
106379	10	13422083	13422114	4	8	32	AAAC	15	5
106382	10	13434158	13434201	4	11	44	AAAT	9	10
106384	10	13436253	13436268	4	4	16	AAAT	21	4
106390	10	13449797	13449810	2	7	14	AC	23	4
106393	10	13459361	13459372	2	6	12	GT	27	6
106394	10	13459929	13459940	2	6	12	TC	23	3
106396	10	13462925	13462942	2	9	18	CT	27	4
106398	10	13463036	13463051	4	4	16	GTGC	20	3
106399	10	13463244	13463271	2	14	28	TC	14	8
106403	10	13463529	13463552	3	8	24	CTC	19	3
106405	10	13465285	13465322	2	19	38	TG	9	9
106410	10	13467593	13467614	2	11	22	TC	9	7
106411	10	13470255	13470266	2	6	12	TG	24	3
106414	10	13472783	13472794	2	6	12	GT	16	3
106415	10	13472797	13472812	2	8	16	GT	18	5
106420	10	13478566	13478585	4	5	20	CTGC	17	3
106421	10	13479290	13479303	2	7	14	CA	31	4
106423	10	13481290	13481307	2	9	18	GT	24	8
106424	10	13481505	13481540	2	18	36	CA	12	11
106433	10	13485750	13485799	2	25	50	TG	7	7
106434	10	13486016	13486033	2	9	18	TC	12	3
106441	10	13506532	13506545	2	7	14	TG	8	7
106443	10	13510678	13510689	2	6	12	CT	33	4
106444	10	13512818	13512831	2	7	14	CA	23	5
106450	10	13514603	13514658	2	28	56	GT	11	8
106452	10	13519024	13519037	2	7	14	TG	28	6
106453	10	13519218	13519231	2	7	14	CA	20	5
106455	10	13520029	13520090	2	31	62	AC	9	10
106456	10	13520253	13520264	2	6	12	AC	25	5
106459	10	13524465	13524476	2	6	12	AC	21	3
106462	10	13525744	13525757	2	7	14	AC	18	4
106465	10	13536003	13536029	3	9	27	CTC	11	5
106469	10	13546290	13546307	2	9	18	TG	14	4
106470	10	13546415	13546448	2	17	34	CA	16	8
106471	10	13548256	13548271	2	8	16	AC	12	11
106472	10	13548429	13548452	2	12	24	TC	10	7
106478	10	13551603	13551616	2	7	14	GT	28	4
106480	10	13552324	13552349	2	13	26	TG	12	5
106488	10	13565311	13565354	4	11	44	TCTG	25	8
106491	10	13571053	13571070	2	9	18	CA	17	10
106494	10	13572194	13572213	2	10	20	CA	18	9
106495	10	13572342	13572359	2	9	18	TG	17	12
106497	10	13574573	13574590	2	9	18	AG	25	7
106499	10	13576297	13576322	2	13	26	CA	7	8

106500	10	13578326	13578343	3	6	18	AGA	14	3
106507	10	13591436	13591450	3	5	15	ATG	29	5
106508	10	13591824	13591855	2	16	32	CA	18	10
106509	10	13592785	13592810	2	13	26	CT	6	6
106511	10	13593333	13593360	4	7	28	TAGA	18	10
106516	10	13611346	13611361	2	8	16	AC	15	5
106518	10	13620755	13620766	2	6	12	AC	20	7
106519	10	13622688	13622701	2	7	14	AC	25	6
106532	10	13657071	13657091	3	7	21	AAG	24	8
106533	10	13658330	13658367	2	19	38	CA	7	10
106534	10	13659458	13659477	2	10	20	GT	21	7
106536	10	13670940	13670959	2	10	20	AC	26	6
106538	10	13672357	13672376	2	10	20	AG	16	4
106540	10	13674091	13674104	2	7	14	GT	25	3
106546	10	13678079	13678100	2	11	22	TG	17	11
106570	10	13729305	13729316	2	6	12	AT	13	10
106573	10	13739408	13739422	3	5	15	CCA	10	4
106575	10	13750575	13750604	2	15	30	GT	18	8
106576	10	13751348	13751399	4	13	52	GATA	20	7
106579	10	13761211	13761228	3	6	18	GGA	12	4
106583	10	13777297	13777332	2	18	36	CA	8	7
106584	10	13777435	13777450	2	8	16	TG	12	5
106585	10	13778870	13778889	2	10	20	TG	25	3
106587	10	13782862	13782877	2	8	16	TG	21	10
106591	10	13792611	13792624	2	7	14	TG	22	4
106598	10	13805934	13805957	2	12	24	GT	7	7
106599	10	13806717	13806728	2	6	12	CA	22	3
106604	10	13813403	13813432	2	15	30	TG	7	6
106605	10	13817874	13817889	2	8	16	TG	17	6
106635	11	23369	23380	2	6	12	GT	27	4
106636	11	24082	24093	2	6	12	GT	20	3
106639	11	31413	31446	2	17	34	TG	10	11
106642	11	32975	32990	2	8	16	TG	11	7
106645	11	37758	37771	2	7	14	GT	7	5
106649	11	40850	40867	3	6	18	TAT	11	3
106657	11	56864	56893	2	15	30	TG	22	8
106658	11	63609	63622	2	7	14	GT	19	6
106659	11	63752	63779	2	14	28	TC	10	6
106661	11	63835	63854	2	10	20	TG	7	8
106662	11	64026	64051	2	13	26	CA	22	8
106663	11	65045	65064	4	5	20	AGTT	33	3
106665	11	70605	70620	2	8	16	TG	21	8
106666	11	70622	70637	2	8	16	GT	24	10
106667	11	75000	75017	2	9	18	AG	22	5
106668	11	76207	76234	2	14	28	TG	21	14
106669	11	76363	76388	2	13	26	AC	14	9
106674	11	83215	83238	2	12	24	AC	12	7
106675	11	83569	83590	2	11	22	GT	17	7
106680	11	85748	85765	2	9	18	CT	18	3
106692	11	110363	110378	2	8	16	TG	16	4
106693	11	110464	110501	2	19	38	GT	7	11

106696	11	111445	111460	4	4	16	TTAA	22	3
106697	11	112186	112199	2	7	14	TC	8	3
106701	11	117344	117367	2	12	24	CA	9	6
106704	11	119358	119381	2	12	24	TG	8	9
106706	11	121046	121081	2	18	36	AC	18	10
106710	11	129772	129795	2	12	24	TG	17	7
106715	11	135128	135157	2	15	30	TG	9	13
106716	11	135489	135502	2	7	14	AC	26	3
106721	11	139107	139122	2	8	16	GA	14	6
106723	11	146165	146188	4	6	24	GATG	28	9
106726	11	147355	147366	2	6	12	GT	22	6
106731	11	149331	149350	2	10	20	AC	22	4
106733	11	154422	154433	2	6	12	AC	6	7
106738	11	158710	158727	2	9	18	GA	21	6
106740	11	159460	159495	4	9	36	GTTT	18	7
106741	11	164490	164507	2	9	18	CA	18	6
106745	11	170972	171007	4	9	36	GATA	12	9
106748	11	183994	184008	3	5	15	ATT	19	9
106758	11	196255	196286	2	16	32	GT	13	11
106765	11	220805	220822	3	6	18	ACT	25	4
106766	11	225811	225824	2	7	14	AC	21	4
106767	11	225953	225996	4	11	44	GAAA	11	9
106768	11	226220	226239	2	10	20	AC	19	13
106775	11	231463	231474	2	6	12	TC	7	6
106779	11	231720	231735	2	8	16	AC	9	6
106781	11	234702	234728	3	9	27	TCA	12	9
106782	11	235742	235761	2	10	20	AC	6	5
106783	11	240873	240884	2	6	12	CA	22	13
106784	11	240887	240904	2	9	18	CA	16	7
106786	11	242113	242132	2	10	20	TG	14	4
106787	11	243169	243202	2	17	34	GA	12	9
106789	11	243411	243438	2	14	28	GT	19	7
106791	11	243536	243555	2	10	20	TG	23	6
106792	11	245126	245143	2	9	18	GA	18	4
106793	11	245192	245219	2	14	28	GA	16	11
106794	11	245354	245375	2	11	22	GA	15	5
106797	11	246954	246967	2	7	14	GA	27	5
106798	11	247059	247084	2	13	26	AC	16	7
106799	11	247313	247326	2	7	14	AC	6	5
106800	11	249162	249181	2	10	20	GT	17	10
106801	11	249287	249316	2	15	30	TG	21	16
106806	11	256711	256730	2	10	20	TC	15	4
106809	11	256813	256826	2	7	14	GA	15	4
106810	11	256903	256946	2	22	44	TG	12	11
106811	11	257447	257473	3	9	27	TCT	12	9
106813	11	258722	258741	2	10	20	TG	22	8
106814	11	260071	260110	2	20	40	CT	6	6
106815	11	260307	260320	2	7	14	GT	24	4
106817	11	260480	260499	2	10	20	CA	15	14
106819	11	261764	261795	2	16	32	TG	11	10
106820	11	261964	261993	2	15	30	AC	19	13

106821	11	263238	263263	2	13	26	TG	23	10
106822	11	263353	263400	4	12	48	TCTT	21	9
106823	11	267106	267119	2	7	14	AC	18	13
106825	11	268154	268169	2	8	16	AG	25	6
106826	11	268537	268562	2	13	26	CA	17	15
106829	11	273411	273432	2	11	22	TG	10	7
106835	11	275973	275992	2	10	20	TG	6	5
106838	11	278305	278322	2	9	18	CA	7	5
106843	11	286123	286178	4	14	56	ACAT	18	8
106845	11	289136	289159	3	8	24	TGA	18	10
106849	11	297333	297352	4	5	20	TAGA	26	6
106850	11	301793	301807	3	5	15	AGT	31	4
106851	11	310998	311011	2	7	14	CA	22	12
106864	11	324214	324241	2	14	28	TG	7	7
106869	11	326235	326268	2	17	34	AC	11	7
106871	11	336647	336698	2	26	52	AC	13	15
106876	11	344898	344921	2	12	24	GT	12	9
106878	11	349409	349436	4	7	28	GTTT	17	3
106879	11	350261	350278	2	9	18	CA	24	9
106880	11	351963	351980	2	9	18	AC	24	11
106881	11	351982	351993	2	6	12	CA	21	8
106887	11	364332	364343	2	6	12	AC	28	4
106889	11	372994	373031	2	19	38	TG	7	9
106890	11	373259	373272	2	7	14	GT	29	5
106892	11	375423	375458	2	18	36	GT	10	6
106897	11	378728	378775	4	12	48	TTTC	8	6
106899	11	379740	379757	3	6	18	TTA	19	3
106900	11	380964	380995	2	16	32	CA	11	6
106903	11	381594	381623	2	15	30	AC	16	11
106907	11	385469	385488	2	10	20	AC	23	9
106909	11	385872	385889	2	9	18	AC	25	4
106913	11	397007	397022	2	8	16	TG	21	4
106920	11	402361	402380	2	10	20	CA	12	11
106921	11	402530	402559	2	15	30	TG	14	16
106922	11	402654	402703	2	25	50	TG	7	9
106930	11	420518	420543	2	13	26	TG	10	8
106942	11	429229	429252	3	8	24	CCT	6	3
106944	11	430453	430479	3	9	27	TGA	24	8
106945	11	432268	432295	4	7	28	AGGA	9	9
106948	11	433980	434001	2	11	22	CT	15	9
106954	11	443351	443368	3	6	18	CAG	35	4
106959	11	451178	451199	2	11	22	TG	22	11
106960	11	451343	451366	2	12	24	CA	14	9
106961	11	455486	455543	2	29	58	AC	8	7
106964	11	464837	464857	3	7	21	TTG	22	6
106970	11	476128	476149	2	11	22	AC	23	6
106974	11	486727	486742	2	8	16	AT	18	7
106975	11	486865	486878	2	7	14	GT	20	9
106976	11	487084	487105	2	11	22	TG	22	7
106983	11	490906	490926	3	7	21	GGA	11	4
106985	11	491823	491840	2	9	18	GT	11	5

106986	11	491878	491899	2	11	22	TG	25	9
106989	11	494274	494297	2	12	24	AC	34	8
106990	11	496188	496235	2	24	48	CA	8	8
106993	11	502319	502348	2	15	30	AC	9	9
106999	11	517241	517254	2	7	14	GT	21	8
107000	11	521984	522007	2	12	24	CA	12	8
107002	11	522236	522253	2	9	18	AC	19	15
107004	11	522281	522300	2	10	20	CA	7	6
107006	11	524797	524808	2	6	12	GT	30	3
107009	11	530739	530750	2	6	12	GT	25	3
107012	11	539499	539548	2	25	50	CA	10	5
107013	11	547385	547402	2	9	18	AC	18	8
107016	11	552584	552611	2	14	28	TG	18	13
107030	11	569394	569407	2	7	14	TG	17	3
107031	11	569695	569710	2	8	16	AC	23	9
107032	11	569712	569731	2	10	20	CA	22	8
107038	11	578658	578683	2	13	26	GT	8	10
107041	11	589364	589383	2	10	20	GT	16	5
107042	11	590339	590360	2	11	22	GT	19	3
107045	11	596127	596150	2	12	24	CA	9	11
107049	11	598220	598235	2	8	16	TC	10	3
107056	11	605007	605026	2	10	20	CA	14	3
107072	11	609800	609827	2	14	28	TG	11	8
107073	11	615185	615214	2	15	30	CA	11	6
107075	11	619351	619368	3	6	18	GGT	26	4
107082	11	628821	628832	2	6	12	AC	22	7
107083	11	628999	629016	2	9	18	TG	7	5
107084	11	631544	631571	2	14	28	AC	14	7
107086	11	635327	635340	2	7	14	CA	24	7
107087	11	635343	635360	2	9	18	CA	18	7
107090	11	637467	637480	2	7	14	TG	27	5
107093	11	640649	640669	3	7	21	ATG	22	3
107096	11	643297	643310	2	7	14	TG	34	4
107102	11	655373	655390	2	9	18	GT	12	11
107103	11	655760	655771	2	6	12	TG	29	3
107104	11	656336	656359	3	8	24	AGG	21	6
107109	11	658337	658350	2	7	14	CT	11	6
107114	11	664346	664373	2	14	28	TG	18	8
107117	11	667055	667068	2	7	14	GT	24	10
107122	11	672388	672399	2	6	12	CT	25	3
107129	11	684414	684443	2	15	30	AC	10	8
107133	11	686967	686986	4	5	20	TTTG	23	3
107139	11	703280	703299	2	10	20	CA	16	4
107140	11	706611	706638	2	14	28	GT	8	10
107145	11	729623	729644	2	11	22	GT	13	10
107146	11	730812	730859	2	24	48	GT	20	6
107151	11	747949	747966	3	6	18	TTG	24	3
107153	11	753364	753389	2	13	26	TG	17	11
107158	11	766266	766283	2	9	18	CA	24	15
107161	11	781644	781659	2	8	16	AC	19	8
107162	11	781996	782021	2	13	26	TG	16	15

107164	11	782107	782118	2	6	12	GT	27	3
107167	11	790065	790094	2	15	30	GT	10	10
107168	11	790798	790817	2	10	20	TG	29	13
107172	11	800151	800190	2	20	40	TG	14	12
107175	11	821751	821768	3	6	18	GCA	20	5
107179	11	833815	833836	2	11	22	GT	17	6
107183	11	838361	838388	2	14	28	AC	19	9
107186	11	846582	846617	2	18	36	AC	16	13
107187	11	848087	848107	3	7	21	AAT	13	6
107192	11	860484	860499	2	8	16	AC	8	4
107193	11	860509	860522	2	7	14	CA	11	8
107204	11	888880	888891	2	6	12	GT	29	6
107214	11	910282	910296	3	5	15	TTG	30	4
107217	11	922241	922256	4	4	16	ATTT	13	3
107220	11	927149	927160	2	6	12	TG	25	10
107221	11	927521	927532	2	6	12	TC	25	4
107228	11	934996	935031	2	18	36	AG	7	10
107229	11	935571	935582	2	6	12	GT	25	8
107230	11	935650	935661	2	6	12	TG	31	3
107231	11	935818	935845	2	14	28	GT	12	10
107243	11	971805	971830	2	13	26	AC	11	11
107249	11	976578	976591	2	7	14	AT	11	3
107256	11	981811	981824	2	7	14	GT	30	15
107257	11	982115	982144	2	15	30	AC	20	8
107260	11	983154	983185	2	16	32	GT	12	7
107261	11	983340	983385	2	23	46	TG	18	14
107266	11	991716	991727	2	6	12	TG	27	5
107268	11	994286	994315	2	15	30	AC	20	7
107271	11	996813	996840	2	14	28	CA	15	7
107273	11	997074	997089	2	8	16	CA	13	8
107275	11	998820	998839	2	10	20	CA	20	10
107277	11	1000177	1000192	2	8	16	AC	24	4
107279	11	1000855	1000874	2	10	20	GA	22	3
107281	11	1003258	1003269	2	6	12	TG	27	3
107286	11	1010743	1010762	2	10	20	AC	23	8
107287	11	1011588	1011603	2	8	16	TG	26	5
107290	11	1015575	1015586	2	6	12	GT	25	8
107295	11	1025290	1025303	2	7	14	CA	15	7
107297	11	1027198	1027224	3	9	27	GCA	25	7
107298	11	1028105	1028125	3	7	21	TGA	21	6
107300	11	1028538	1028549	2	6	12	AG	26	3
107305	11	1032271	1032284	2	7	14	GT	19	6
107306	11	1032286	1032303	2	9	18	TG	16	10
107307	11	1032414	1032441	2	14	28	AC	16	13
107312	11	1036839	1036854	2	8	16	CA	17	6
107313	11	1036954	1036971	2	9	18	GT	22	5
107314	11	1038389	1038406	2	9	18	TG	31	3
107315	11	1040454	1040473	2	10	20	AC	21	8
107316	11	1042945	1042958	2	7	14	TG	23	4
107317	11	1043092	1043113	2	11	22	CT	15	4
107319	11	1043273	1043292	2	10	20	TG	12	13

107321	11	1046052	1046071	2	10	20	AC	10	12
107326	11	1048317	1048330	2	7	14	AG	14	4
107327	11	1048467	1048512	2	23	46	TG	7	9
107329	11	1050750	1050765	2	8	16	AC	19	13
107330	11	1050766	1050779	2	7	14	CA	17	10
107332	11	1051925	1051944	2	10	20	AC	22	6
107337	11	1056449	1056462	2	7	14	TG	26	4
107339	11	1058463	1058474	2	6	12	GT	24	3
107342	11	1064116	1064131	2	8	16	TG	28	6
107343	11	1066415	1066438	2	12	24	CA	15	7
107344	11	1066658	1066703	2	23	46	GT	9	10
107353	11	1071994	1072015	2	11	22	AC	8	9
107354	11	1072116	1072135	2	10	20	TG	6	4
107357	11	1075400	1075421	2	11	22	AC	15	7
107360	11	1077360	1077373	2	7	14	CA	24	3
107368	11	1083429	1083464	2	18	36	TG	12	8
107370	11	1085962	1086007	2	23	46	CA	17	13
107371	11	1088708	1088723	2	8	16	CA	19	5
107373	11	1091574	1091585	2	6	12	GT	22	7
107375	11	1091888	1091901	2	7	14	AC	23	6
107378	11	1096314	1096325	2	6	12	TG	30	6
107379	11	1096469	1096494	2	13	26	AC	19	8
107380	11	1097026	1097045	2	10	20	GT	19	7
107385	11	1101668	1101695	4	7	28	ATGG	21	4
107391	11	1111413	1111436	2	12	24	GT	13	8
107399	11	1116665	1116680	2	8	16	GT	27	10
107400	11	1118004	1118015	2	6	12	GT	29	4
107403	11	1120486	1120497	2	6	12	TG	31	7
107404	11	1120928	1120939	2	6	12	TG	34	4
107406	11	1120987	1120998	2	6	12	GA	27	3
107408	11	1123000	1123029	3	10	30	GGA	14	5
107417	11	1126607	1126630	2	12	24	CA	14	11
107419	11	1127478	1127501	2	12	24	TC	11	4
107422	11	1128370	1128393	2	12	24	CA	19	8
107426	11	1132362	1132375	2	7	14	GT	19	3
107427	11	1135339	1135359	3	7	21	TAA	14	5
107429	11	1137440	1137451	2	6	12	TG	25	4
107431	11	1139017	1139030	2	7	14	CA	21	12
107434	11	1148824	1148835	2	6	12	CT	32	4
107439	11	1153770	1153789	2	10	20	CA	16	6
107443	11	1161521	1161532	2	6	12	AT	27	3
107444	11	1167187	1167204	3	6	18	CCT	24	4
107445	11	1173462	1173489	2	14	28	CA	7	6
107449	11	1179937	1179966	2	15	30	AC	14	7
107455	11	1187199	1187212	2	7	14	AC	24	4
107458	11	1193770	1193793	2	12	24	TG	15	8
107459	11	1195515	1195534	2	10	20	CA	26	11
107460	11	1195646	1195675	3	10	30	GAG	29	9
107461	11	1196935	1196954	4	5	20	TTTA	15	8
107462	11	1197526	1197551	2	13	26	AC	14	5
107464	11	1199105	1199118	2	7	14	CA	7	7

107472	11	1209358	1209369	2	6	12	TG	23	9
107475	11	1220930	1220956	3	9	27	GAG	18	4
107476	11	1221174	1221207	2	17	34	CA	7	8
107481	11	1225854	1225877	2	12	24	GT	18	7
107491	11	1238319	1238336	2	9	18	TG	23	5
107497	11	1245786	1245809	2	12	24	AC	14	6
107500	11	1246497	1246516	2	10	20	TG	14	8
107501	11	1246791	1246814	3	8	24	TCC	21	5
107505	11	1248887	1248900	2	7	14	AC	19	10
107506	11	1248902	1248919	2	9	18	CA	11	9
107509	11	1256717	1256730	2	7	14	TG	28	7
107510	11	1257370	1257397	2	14	28	CA	16	9
107512	11	1259667	1259678	2	6	12	CA	38	3
107514	11	1260106	1260131	2	13	26	AC	20	6
107523	11	1266151	1266168	3	6	18	GGA	10	4
107524	11	1266768	1266809	2	21	42	GT	7	9
107526	11	1268659	1268684	2	13	26	TG	20	8
107527	11	1268802	1268825	2	12	24	AC	16	7
107528	11	1269040	1269053	2	7	14	AG	24	3
107529	11	1269081	1269100	2	10	20	AG	17	8
107531	11	1270808	1270821	2	7	14	GT	26	5
107532	11	1271034	1271045	2	6	12	TG	26	3
107533	11	1271276	1271293	2	9	18	AC	18	10
107536	11	1274911	1274936	2	13	26	AC	15	10
107537	11	1275234	1275245	2	6	12	GT	21	4
107540	11	1279783	1279802	2	10	20	CT	15	4
107543	11	1282526	1282540	3	5	15	TCT	25	4
107545	11	1287840	1287867	2	14	28	TG	12	7
107546	11	1288002	1288021	2	10	20	AC	15	5
107547	11	1288231	1288242	2	6	12	GA	19	3
107552	11	1290756	1290773	2	9	18	TG	14	5
107567	11	1306934	1306955	2	11	22	CA	26	3
107569	11	1308675	1308696	2	11	22	CA	17	9
107570	11	1309010	1309021	2	6	12	CA	28	3
107576	11	1314960	1314977	2	9	18	GT	13	12
107580	11	1318276	1318302	3	9	27	GAA	15	13
107594	11	1328042	1328071	2	15	30	CA	10	12
107595	11	1328396	1328429	2	17	34	TC	22	7
107597	11	1329491	1329506	2	8	16	GT	26	3
107599	11	1330704	1330729	2	13	26	GT	12	11
107600	11	1330817	1330842	2	13	26	GT	15	12
107602	11	1331450	1331485	2	18	36	TG	9	6
107610	11	1333445	1333460	2	8	16	CA	11	7
107626	11	1348140	1348153	2	7	14	CA	18	9
107632	11	1356792	1356817	2	13	26	TC	20	6
107633	11	1357259	1357276	2	9	18	TG	14	8
107635	11	1357438	1357473	2	18	36	CA	8	8
107637	11	1359728	1359757	2	15	30	GT	11	7
107643	11	1360564	1360578	3	5	15	AGA	12	3
107644	11	1360689	1360700	2	6	12	GT	7	4
107647	11	1361145	1361184	4	10	40	TTTC	12	9

107648	11	1361569	1361596	2	14	28	CA	17	11
107655	11	1362014	1362039	2	13	26	AC	10	9
107659	11	1363868	1363899	2	16	32	AC	19	6
107660	11	1363941	1363968	2	14	28	AC	17	7
107661	11	1364123	1364146	2	12	24	CA	12	7
107666	11	1365306	1365325	4	5	20	AAAG	7	3
107668	11	1365858	1365897	2	20	40	CA	15	10
107669	11	1366338	1366365	2	14	28	CA	12	11
107670	11	1367690	1367709	2	10	20	GT	17	5
107671	11	1368847	1368868	2	11	22	AC	18	7
107673	11	1369562	1369577	2	8	16	AC	25	4
107674	11	1369731	1369750	2	10	20	CA	21	3
107675	11	1369969	1369986	2	9	18	CA	33	3
107676	11	1370954	1370981	2	14	28	TG	14	6
107680	11	1376333	1376344	2	6	12	CA	21	6
107681	11	1376346	1376361	2	8	16	AC	23	10
107685	11	1379008	1379029	2	11	22	CT	17	5
107686	11	1380102	1380117	2	8	16	TA	15	8
107688	11	1382783	1382797	3	5	15	GCT	28	4
107690	11	1384859	1384870	2	6	12	GT	26	5
107694	11	1385885	1385900	2	8	16	TC	12	3
107698	11	1394241	1394276	2	18	36	GT	8	7
107702	11	1397742	1397756	3	5	15	AGG	24	3
107704	11	1398460	1398481	2	11	22	TG	17	13
107705	11	1398883	1398914	2	16	32	TG	11	8
107710	11	1402758	1402775	2	9	18	CT	21	4
107726	11	1418003	1418018	2	8	16	AT	10	7
107727	11	1423879	1423890	2	6	12	TG	32	3
107742	11	1470327	1470338	2	6	12	TG	23	4
107746	11	1477222	1477236	3	5	15	GAA	32	3
107748	11	1479209	1479258	2	25	50	AC	8	10
107757	11	1501724	1501759	2	18	36	AC	6	6
107764	11	1514232	1514251	2	10	20	CA	24	11
107765	11	1517720	1517735	2	8	16	AC	17	9
107769	11	1521888	1521907	2	10	20	AG	18	7
107774	11	1532450	1532461	2	6	12	AC	31	4
107778	11	1542129	1542154	2	13	26	CA	10	11
107781	11	1546233	1546254	2	11	22	AC	18	11
107783	11	1548574	1548605	2	16	32	CA	15	8
107784	11	1548796	1548823	2	14	28	GT	16	11
107785	11	1549104	1549143	2	20	40	TG	8	10
107791	11	1552718	1552743	2	13	26	AC	10	12
107795	11	1557355	1557370	2	8	16	CT	24	3
107802	11	1561241	1561262	2	11	22	AC	23	9
107805	11	1562416	1562431	2	8	16	CA	21	15
107806	11	1562462	1562477	2	8	16	CA	18	5
107807	11	1563059	1563072	2	7	14	AG	29	3
107808	11	1563192	1563236	3	15	45	GAA	22	11
107815	11	1565224	1565239	2	8	16	CA	26	4
107816	11	1566307	1566336	3	10	30	GAG	9	4
107819	11	1571641	1571654	2	7	14	GT	30	12

107820	11	1571841	1571912	4	18	72	AGAT	6	6
107822	11	1572660	1572719	4	15	60	TAGA	6	10
107824	11	1574949	1574980	2	16	32	CA	18	12
107830	11	1579189	1579204	2	8	16	AC	23	6
107831	11	1579268	1579281	2	7	14	CA	22	5
107832	11	1580745	1580760	2	8	16	AT	7	3
107833	11	1581291	1581308	2	9	18	TG	17	8
107834	11	1581311	1581322	2	6	12	TG	20	9
107836	11	1582081	1582100	2	10	20	TG	18	5
107841	11	1599153	1599184	2	16	32	GA	8	7
107846	11	1602926	1602937	2	6	12	CA	27	5
107847	11	1603591	1603610	2	10	20	GA	17	4
107848	11	1603802	1603813	2	6	12	AG	21	3
107852	11	1628707	1628740	2	17	34	GT	14	10
107859	11	1636889	1636902	2	7	14	CA	20	4
107860	11	1637687	1637718	2	16	32	AC	12	12
107862	11	1638102	1638116	3	5	15	GCA	31	5
107872	11	1656589	1656602	2	7	14	CA	22	10
107874	11	1666698	1666709	2	6	12	TG	29	4
107883	11	1684812	1684827	4	4	16	AAAT	19	7
107886	11	1702938	1702963	2	13	26	CA	23	21
107888	11	1710004	1710015	2	6	12	CA	31	3
107890	11	1712056	1712076	3	7	21	GAT	15	3
107893	11	1715905	1715920	2	8	16	AC	18	4
107895	11	1718348	1718365	2	9	18	GT	8	4
107899	11	1722616	1722635	2	10	20	GT	7	3
107905	11	1725341	1725362	2	11	22	AG	18	5
107907	11	1732057	1732071	3	5	15	CAG	25	3
107911	11	1738033	1738059	3	9	27	GCT	22	5
107912	11	1739501	1739526	2	13	26	CA	22	7
107914	11	1740396	1740411	2	8	16	AC	15	9
107917	11	1741941	1741968	2	14	28	GT	13	6
107919	11	1746424	1746456	3	11	33	CTT	27	5
107920	11	1747726	1747755	2	15	30	CA	14	6
107922	11	1750285	1750312	2	14	28	CA	17	6
107923	11	1751521	1751548	2	14	28	GA	15	9
107930	11	1761814	1761835	2	11	22	GT	22	5
107931	11	1763941	1763960	2	10	20	CA	8	8
107940	11	1798390	1798404	3	5	15	AAT	30	6
107948	11	1819246	1819279	2	17	34	TC	6	9
107949	11	1821439	1821450	2	6	12	AT	26	5
107964	11	1856431	1856452	2	11	22	GT	20	5
107974	11	1890295	1890308	2	7	14	CA	15	4
107976	11	1891618	1891631	2	7	14	CA	9	5
107985	11	1905360	1905377	3	6	18	GGA	16	4
107986	11	1906240	1906254	3	5	15	GCT	26	4
107994	11	1926723	1926743	3	7	21	GAA	20	5
107999	11	1930553	1930580	4	7	28	ATGT	18	4
108003	11	1936996	1937019	2	12	24	AC	20	5
108008	11	1945293	1945304	2	6	12	GT	18	3
108009	11	1945308	1945319	2	6	12	GT	19	3

108017	11	1955402	1955413	2	6	12	CA	22	4
108019	11	1956597	1956614	2	9	18	TG	22	6
108020	11	1956616	1956627	2	6	12	GT	26	4
108021	11	1959284	1959295	2	6	12	AG	31	3
108022	11	1959386	1959419	2	17	34	CA	13	8
108024	11	1968291	1968318	2	14	28	GT	8	8
108030	11	1984744	1984757	2	7	14	AC	20	13
108036	11	2013272	2013283	2	6	12	GT	12	8
108037	11	2013285	2013300	2	8	16	TG	15	9
108038	11	2017142	2017171	2	15	30	GT	15	10
108041	11	2022820	2022833	2	7	14	AC	22	4
108042	11	2024982	2025007	2	13	26	TG	8	13
108046	11	2028571	2028594	2	12	24	GA	19	4
108047	11	2030530	2030569	2	20	40	CA	10	8
108050	11	2031806	2031833	2	14	28	TG	7	8
108054	11	2036891	2036904	2	7	14	AC	17	3
108055	11	2038678	2038701	2	12	24	TG	13	12
108056	11	2039174	2039191	2	9	18	GT	17	10
108057	11	2039318	2039333	4	4	16	ACAG	33	4
108063	11	2044954	2044968	3	5	15	AGG	35	3
108064	11	2047983	2047998	2	8	16	GT	21	3
108068	11	2052174	2052189	4	4	16	GTGC	17	8
108073	11	2059460	2059499	2	20	40	TG	16	9
108078	11	2077728	2077751	2	12	24	AC	21	9
108081	11	2085499	2085522	4	6	24	AATG	16	5
108083	11	2090422	2090433	2	6	12	CA	25	9
108086	11	2097689	2097710	2	11	22	GT	15	7
108089	11	2105441	2105472	2	16	32	TG	13	10
108093	11	2116143	2116178	4	9	36	ATTC	24	8
108099	11	2123034	2123053	2	10	20	AG	18	5
108100	11	2123475	2123492	2	9	18	GA	19	4
108105	11	2135759	2135782	4	6	24	GATG	23	9
108109	11	2152781	2152804	4	6	24	TTTG	19	6
108112	11	2164293	2164304	2	6	12	CA	6	3
108120	11	2180370	2180381	2	6	12	TG	23	4
108124	11	2192721	2192750	2	15	30	CA	21	11
108126	11	2193801	2193820	4	5	20	CTGC	27	5
108128	11	2194858	2194871	2	7	14	AG	35	4
108132	11	2214367	2214384	3	6	18	GAG	22	6
108141	11	2222278	2222299	2	11	22	CA	21	6
108142	11	2222503	2222526	3	8	24	TGC	24	7
108143	11	2223949	2223968	2	10	20	AC	10	12
108146	11	2230442	2230471	2	15	30	CA	12	10
108147	11	2233389	2233402	2	7	14	GT	28	4
108148	11	2233513	2233546	2	17	34	GT	11	8
108149	11	2233718	2233735	2	9	18	CA	21	4
108150	11	2238582	2238601	2	10	20	AC	19	9
108152	11	2241533	2241558	2	13	26	GT	18	6
108154	11	2242907	2242944	2	19	38	CA	8	9
108164	11	2266058	2266073	2	8	16	AT	8	6
108165	11	2266895	2266914	2	10	20	TG	18	14

108166	11	2266915	2266926	2	6	12	AG	19	11
108168	11	2267202	2267221	2	10	20	GT	17	9
108169	11	2267361	2267382	2	11	22	AC	10	13
108175	11	2281908	2281939	2	16	32	AC	6	7
108180	11	2285099	2285114	2	8	16	TG	25	3
108188	11	2291495	2291522	2	14	28	TC	12	14
108189	11	2291683	2291696	2	7	14	CT	7	3
108192	11	2292016	2292057	2	21	42	CT	7	6
108194	11	2298525	2298540	2	8	16	TG	21	4
108195	11	2300222	2300247	2	13	26	GT	20	6
108196	11	2302368	2302385	3	6	18	CAG	33	3
108197	11	2302481	2302500	2	10	20	AC	13	14
108198	11	2302626	2302641	2	8	16	GT	20	5
108199	11	2302751	2302774	3	8	24	TTG	27	4
108200	11	2304067	2304106	2	20	40	TG	19	15
108212	11	2315174	2315194	3	7	21	GTG	23	5
108213	11	2315557	2315582	2	13	26	GT	15	15
108214	11	2315819	2315860	3	14	42	CAT	23	12
108215	11	2317235	2317266	2	16	32	TG	6	7
108217	11	2317366	2317389	2	12	24	AC	10	13
108218	11	2322566	2322579	2	7	14	AC	33	4
108219	11	2322748	2322763	2	8	16	AC	26	5
108222	11	2326443	2326454	2	6	12	GT	8	7
108223	11	2326872	2326899	2	14	28	TG	19	15
108228	11	2330740	2330763	2	12	24	TG	18	8
108229	11	2330842	2330853	2	6	12	AC	18	4
108232	11	2332856	2332879	3	8	24	CTG	23	4
108233	11	2333173	2333190	2	9	18	TC	17	3
108238	11	2334129	2334152	2	12	24	TG	14	12
108240	11	2335928	2336003	4	19	76	TAGA	15	12
108241	11	2336398	2336409	2	6	12	TG	16	7
108242	11	2336412	2336427	2	8	16	TG	21	10
108244	11	2346827	2346846	2	10	20	TG	24	9
108247	11	2357419	2357439	3	7	21	TTC	7	7
108251	11	2375233	2375248	2	8	16	TG	15	13
108253	11	2390269	2390296	2	14	28	CA	13	7
108257	11	2393804	2393823	2	10	20	TG	9	7
108267	11	2437299	2437319	3	7	21	GGA	11	3
108268	11	2437754	2437767	2	7	14	CT	19	5
108280	11	2459953	2459966	2	7	14	TG	10	4
108282	11	2467525	2467540	2	8	16	AC	22	4
108284	11	2468862	2468879	2	9	18	TG	23	11
108290	11	2477536	2477557	2	11	22	CT	15	13
108291	11	2477941	2477973	3	11	33	CTT	6	9
[truncated: 487,605 more chars]
