# Supplementary material for: Microsatellite Genome-Wide Database Development for the Commercial Blackhead Seabream (Acanthopagrus schlegelii)
Source: Genes (Basel). 2023 Mar 1;14(3):620. doi: 10.3390/genes14030620 (PMC10048070; doi:10.3390/genes14030620)
Supplement: Supplementary file 1 [file genes-14-00620-s001.zip › Table S1. The number, length, frequency, and density of six different types of perfect SSRs.pdf]

Table S1. The number, length, frequency, and density of six different types of perfect SSRs.

Type	Counts	Length (bp)	Percent (%)	Average Length (bp)	Relative Abundance (loci/Mb)	Relative Density (bp/Mb)
Mono	93792	1431027	22.74	15.26	142.87	2179.84
Di	209890	4752212	50.9	22.64	319.72	7238.89
Tri	41486	933654	10.06	22.51	63.19	1422.2
Tetra	28444	686204	6.9	24.12	43.33	1045.27
Penta	29365	526310	7.12	17.92	44.73	801.71
Hexa	9404	184458	2.28	19.61	14.32	280.98
