# Supplementary material for: Microsatellite Genome-Wide Database Development for the Commercial Blackhead Seabream (Acanthopagrus schlegelii)
Source: Genes (Basel). 2023 Mar 1;14(3):620. doi: 10.3390/genes14030620 (PMC10048070; doi:10.3390/genes14030620)
Supplement: Supplementary file 1 [file genes-14-00620-s001.zip › Table S2. Frequency distribution of motif types of hexanucleotide SSR loci in the reference genome of blackhead seabream.pdf]

Table S2. Frequency distribution of motif types of hexanucleotide SSR loci in the reference genome of blackhead seabream

Motif	Type	Counts	Percent (%)
AATCCC	6	737	7.84
AATCAG	6	652	6.93
AAAAAT	6	592	6.3
AAAAAC	6	496	5.27
AAAAAG	6	468	4.98
AAGAGG	6	302	3.21
AACCAG	6	290	3.08
ACACGC	6	235	2.5
ACCTCC	6	231	2.46
AGAGGG	6	202	2.15
ATATAG	6	171	1.82
ACCAGG	6	119	1.27
ACGAGG	6	126	1.34
AAATGT	6	150	1.6
AAACAC	6	145	1.54
ACACAG	6	122	1.3
ACAGAG	6	120	1.28
ATATAC	6	109	1.16
AATAGT	6	105	1.12
ATACAC	6	103	1.1
AAATAT	6	98	1.04
AAAGAG	6	91	0.97
ACACTC	6	82	0.87
AAAATG	6	87	0.93
AAGATG	6	77	0.82
ACCTGG	6	55	0.58
AAATAC	6	69	0.73
AAACAG	6	67	0.71
ACCCCC	6	67	0.71
AATCGT	6	59	0.63
AACACC	6	53	0.56
ATCCTC	6	59	0.63
AAGGAG	6	56	0.6
AAAATC	6	59	0.63
ACCAGC	6	47	0.5
AATACT	6	48	0.51
AATAAC	6	52	0.55
ACTCAG	6	51	0.54
AAATTC	6	51	0.54
AAACTG	6	48	0.51
ATCAGG	6	39	0.41
ACACCC	6	44	0.47
AATATT	6	45	0.48

AAAATT	6	45	0.48
AACAAG	6	38	0.4
ATCACC	6	43	0.46
ATAGAG	6	41	0.44
AATCTG	6	41	0.44
AGGGGG	6	43	0.46
ACTCCG	6	38	0.4
AATAAG	6	33	0.35
AATTCT	6	42	0.45
AATATG	6	42	0.45
AATTAC	6	42	0.45
ATCTGG	6	35	0.37
AATATC	6	40	0.43
ATCTCC	6	39	0.41
AACATG	6	35	0.37
AAATCT	6	37	0.39
AACCTG	6	35	0.37
AAATAG	6	32	0.34
AACATC	6	35	0.37
ACCACG	6	28	0.3
AATGTG	6	34	0.36
AAATGG	6	33	0.35
ACTCTC	6	29	0.31
ATACAG	6	31	0.33
AAATCC	6	28	0.3
AGCTCC	6	29	0.31
AACAGG	6	23	0.24
AATGTC	6	30	0.32
ATGTGC	6	29	0.31
ACACCG	6	27	0.29
AATCAC	6	27	0.29
AACAGC	6	27	0.29
AATCAT	6	24	0.26
AAAGTT	6	27	0.29
ACCCTC	6	26	0.28
AGCCCG	6	20	0.21
AAGGTG	6	25	0.27
ACCTCG	6	19	0.2
ATCAGC	6	21	0.22
AAGTCT	6	24	0.26
ATGACC	6	20	0.21
AAAGTC	6	23	0.24
ACTGGG	6	21	0.22
AAATTG	6	21	0.22
ATACTG	6	22	0.23
ATGTCC	6	18	0.19
AAGACG	6	21	0.22

ACCCAG	6	20	0.21
AAAGTG	6	21	0.22
ACTCCC	6	18	0.19
AAAACC	6	19	0.2
ACGGAG	6	18	0.19
ATCTGC	6	18	0.19
AAGGAC	6	18	0.19
AGAGCG	6	18	0.19
ACAGGG	6	16	0.17
AAAGAT	6	18	0.19
ATCCAC	6	18	0.19
AAGTCG	6	17	0.18
AATGAG	6	16	0.17
ACAGTC	6	17	0.18
ACCTGC	6	14	0.15
AAAAGG	6	17	0.18
ACCGAG	6	17	0.18
AACTTG	6	16	0.17
AGAGGC	6	12	0.13
AATGAC	6	16	0.17
ACAGTG	6	16	0.17
ACGGCG	6	16	0.17
ACCGCC	6	15	0.16
AAAGCT	6	15	0.16
ATCGAC	6	15	0.16
AAATGC	6	15	0.16
AAAAGC	6	14	0.15
AGCCGG	6	9	0.1
AACTTC	6	14	0.15
ACTCTG	6	13	0.14
AGCCCC	6	14	0.15
AAATTT	6	14	0.15
ATGAGC	6	13	0.14
ATCGTC	6	12	0.13
AAGGGG	6	12	0.13
ACGTCG	6	12	0.13
ACTCGG	6	11	0.12
ATCCCC	6	12	0.13
AAGCAC	6	10	0.11
AATCTC	6	11	0.12
AAAGGG	6	10	0.11
AACCAT	6	10	0.11
ATGACG	6	11	0.12
AACAGT	6	11	0.12
AACTCC	6	11	0.12
ACTGAG	6	10	0.11
ATAGTG	6	11	0.12

AAACTC	6	10	0.11
AAGGCG	6	8	0.09
AGTCCG	6	7	0.07
AACTGC	6	9	0.1
AAGTGG	6	8	0.09
ATCTAC	6	8	0.09
AACCCC	6	9	0.1
AATCCT	6	9	0.1
AATTCC	6	9	0.1
AACCAC	6	8	0.09
AACTAG	6	8	0.09
AAGGAT	6	8	0.09
ACCCCG	6	7	0.07
AGGCCC	6	7	0.07
AACGTG	6	7	0.07
AATACC	6	8	0.09
ACCGGG	6	8	0.09
ATCACG	6	7	0.07
ATGTAC	6	7	0.07
AAAGCC	6	7	0.07
AGGCGG	6	7	0.07
ATACCG	6	7	0.07
ATGGAG	6	7	0.07
AACTCG	6	5	0.05
ACACGG	6	7	0.07
ACACTG	6	7	0.07
ATGCCC	6	6	0.06
AAACCC	6	6	0.06
ATCGCC	6	6	0.06
AACTAC	6	6	0.06
AATGGG	6	6	0.06
AATTAG	6	6	0.06
ACTGCC	6	6	0.06
AAGATC	6	6	0.06
AATAGC	6	5	0.05
AGGGGC	6	5	0.05
ATACTC	6	6	0.06
ATATCC	6	6	0.06
AACGCT	6	5	0.05
AATCGG	6	5	0.05
CCCCGG	6	4	0.04
ATCATG	6	4	0.04
AACACG	6	4	0.04
AACCGG	6	4	0.04
AAGCAT	6	4	0.04
AATGGT	6	4	0.04
ACGCAG	6	4	0.04

AGCCGC	6	4	0.04
ATCGGC	6	4	0.04
AAATCG	6	3	0.03
AACCCG	6	3	0.03
AATCCG	6	3	0.03
ACCGGC	6	3	0.03
ACCGTG	6	3	0.03
ATAGGG	6	3	0.03
AACTGG	6	2	0.02
AAGAGC	6	3	0.03
AAGGGC	6	3	0.03
ACGCGC	6	3	0.03
AGCGCC	6	3	0.03
ATGGGG	6	2	0.02
ACCCGC	6	2	0.02
ACGGGC	6	2	0.02
AACGGC	6	2	0.02
AAGCCG	6	2	0.02
AAGGTC	6	2	0.02
AATTCG	6	2	0.02
ACAGCG	6	2	0.02
ACCCGG	6	2	0.02
ACGGGG	6	2	0.02
ACGTCC	6	2	0.02
ACGTGC	6	2	0.02
ACTCGC	6	2	0.02
AGCGGG	6	2	0.02
ATCCGG	6	2	0.02
ATCGCG	6	2	0.02
ATCTCG	6	2	0.02
ACGAGC	6	1	0.01
ATGGCC	6	1	0.01
AAACGG	6	1	0.01
AACCGC	6	1	0.01
AATGCC	6	1	0.01
AATTGC	6	1	0.01
ACGCGG	6	1	0.01
ACGCTC	6	1	0.01
ACTGCG	6	1	0.01
ACTGGC	6	1	0.01
AGCTCG	6	1	0.01
AGGGCG	6	1	0.01
ATAGCG	6	1	0.01
ATATCG	6	1	0.01
ATATGC	6	1	0.01
ATCCGC	6	1	0.01
ATCTAG	6	1	0.01

ATGCGC	6	1	0.01
CCCCCG	6	1	0.01
CCCGGG	6	1	0.01
