# Supplementary material for: Microsatellite Genome-Wide Database Development for the Commercial Blackhead Seabream (Acanthopagrus schlegelii)
Source: Genes (Basel). 2023 Mar 1;14(3):620. doi: 10.3390/genes14030620 (PMC10048070; doi:10.3390/genes14030620)
Supplement: Supplementary file 1 [file genes-14-00620-s001.zip › Table S3. Distribution of main SSR repeat number for each SSR type in blackhead seabream.pdf]

Table S3. Distribution of main SSR repeat number for each SSR type in blackhead seabream

Repeat	All counts	Times	counts	Ratio(%)	times	counts	Ratio(%)
1	93792	12-23	89696	95.63	12	23600	25.16
2	209890	6-20	191786	91.37	6	45329	21.60
3	41486	5-12	38444	92.67	5	16281	39.24
4	28444	4-10	25798	90.70	4	13923	48.95
5	29365	3-6	28054	95.54	3	22574	76.87
6	9404	3-6	9301	98.90	3	7767	82.60
