# Supplementary material for: Microsatellite Genome-Wide Database Development for the Commercial Blackhead Seabream (Acanthopagrus schlegelii)
Source: Genes (Basel). 2023 Mar 1;14(3):620. doi: 10.3390/genes14030620 (PMC10048070; doi:10.3390/genes14030620)
Supplement: Supplementary file 1 [file genes-14-00620-s001.zip › Table S4. Distribution of called microsatellite loci on the scaffold of the genome.pdf]

Table S4. Distribution of called microsatellite loci on the scaffold of the genome

Scaffold	Number of microsatellites	Total(ratio)	Scaffold length(bp)
1	5851	44077(28.24%)	182,321,725
2	5588		
3	4660		
4	4304		
5	4040		
6	3387		
7	5703		
8	2823		
9	4244		
10	3477		
11	4014	28999(18.58%)	108,318,484
12	4332		
13	3122		
14	2845		
15	2636		
16	2914		
17	3014		
18	2185		
19	1795		
20	2142		
21	1915	20062(12.85%)	75,395,736
22	3505		
23	1923		
24	1910		
25	1444		
26	2818		
27	2091		
28	1161		
29	1742		
30	1553		
31	4094	16702(10.70%)	68,101,658
32	1279		
33	1435		
34	1562		
35	1923		
36	1599		
37	1156		
38	1437		
39	1007		
40	1210		
41	946		
42	743		

43	1135	10025	40,827,991
44	808		
45	865		
46	1415		
47	1485		
48	896		
49	1053		
50	679		
51	874	7292	32,876,149
52	986		
53	636		
54	920		
55	580		
56	540		
57	410		
58	1184		
59	610		
60	552		
61	503	6583	
62	537		
63	558		
64	688		
65	588		
66	675		
67	962		
68	802		
69	916		
70	354		
71	720	6577	
72	440		
73	693		
74	473		
75	417		
76	579		
77	1197		
78	550		
79	422		
80	1086		
81	702	5503	
82	364		
83	825		
84	249		
85	579		
86	525		
87	567		
88	469		
89	623		

90	600		
91	432		
92	260		
93	271		
94	425		
95	607		
96	258		
97	528		
98	590		
99	230		
100	457		
101	257		
102	489		
103	293		
104	405		
105	955		
106	329		
107	156		
108	133		
109	377		
110	195		
111	452		
112	768		
113	336		
114	225		
115	0		
116	421		
117	0		
118	138		
119	0		
120	0		
138	279		
Total	156086		
