# Supplementary material for: Microsatellite Genome-Wide Database Development for the Commercial Blackhead Seabream (Acanthopagrus schlegelii)
Source: Genes (Basel). 2023 Mar 1;14(3):620. doi: 10.3390/genes14030620 (PMC10048070; doi:10.3390/genes14030620)
Supplement: Supplementary file 1 [file genes-14-00620-s001.zip › Table S5. Distribution of repeats of called microsatellite loci.pdf]

Table S5. Distribution of repeats of called microsatellite loci

Repeat type	Times	HipSTR count	Genome count	Proportion (%)
di-	6	24953	45329	55.05
	7	16710	28271	59.11
	8	12094	20287	59.61
	9	9879	16650	59.33
	10	8004	13386	59.79
	11	6754	10983	61.50
	12	6317	10092	62.59
	13	5790	9263	62.51
	14	5224	8320	62.79
	15	4522	7302	61.93
	16	3801	6187	61.44
	17	3130	5238	59.76
	18	2448	4154	58.93
	19	1962	3451	56.85
	20	1638	2873	57.01
	total	113226	191786	59.04
Tri-	5	8917	16281	54.77
	6	4879	8311	58.71
	7	2963	5119	57.88
	8	1871	3173	58.97
	9	1211	2197	55.12
	10	800	1527	52.39
	11	505	1035	48.79
	12	331	801	41.32
	total	21477	38444	55.87
Tetra-	4	7375	13923	52.97
	5	3154	5647	55.85
	6	1545	2712	56.97
	7	785	1455	53.95
	8	479	939	51.01
	9	298	631	47.23
	10	237	491	48.27
	total	13873	25798	53.76
