# Supplementary material for: Microsatellite Genome-Wide Database Development for the Commercial Blackhead Seabream (Acanthopagrus schlegelii)
Source: Genes (Basel). 2023 Mar 1;14(3):620. doi: 10.3390/genes14030620 (PMC10048070; doi:10.3390/genes14030620)
Supplement: Supplementary file 1 [file genes-14-00620-s001.zip › Table S6. Distribution of genotyped individuals of called microsatellite loci.pdf]

Table S6. Distribution of genotyped individuals of called microsatellite loci

Number of genotyped individuals	counts	
1	2163	13505
2	2307	
3	2645	
4	2949	
5	3441	
6	3547	140777
7	4094	
8	4296	
9	4505	
10	4645	
11	5098	
12	5383	
13	5604	
14	5862	
15	6219	
16	6291	
17	6492	
18	6583	
19	6783	
20	6701	
21	6634	
22	6479	
23	6276	
24	5969	
25	5596	
26	5205	
27	4727	
28	4129	
29	3656	
30	3011	
31	2469	
32	1951	
33	1444	
34	1128	
35	780	1804
36	513	
37	282	
38	129	
39	70	
40	19	
41	9	
42	2	
Total	156086	
