# Supplementary material for: Microsatellite Genome-Wide Database Development for the Commercial Blackhead Seabream (Acanthopagrus schlegelii)
Source: Genes (Basel). 2023 Mar 1;14(3):620. doi: 10.3390/genes14030620 (PMC10048070; doi:10.3390/genes14030620)
Supplement: Supplementary file 1 [file genes-14-00620-s001.zip › Table S7. The alleles number of 58 known polymorphic SSRs identified by PCR and HipSTR.pdf]

Table S7. The alleles number of 58 known polymorphic SSRs identified by PCR and HipSTR

no.	loci	modif	scaf.	start	Na-PCR	Na-HipSTR	SSR ID	A.no.	N	Ref.
1	acs36	$(GT)_{10}$	71	674178	6	18	322302	GU206877	16	this study
2	acs146	$(CA)_{6}$	10	1940925	5	6	99577	GU206878	22	
3	acs12	$(GT)_{7}$	31	11907171	2	2	228328	GU206900	27	
4	acs145	$(AC)_{20}$	1	2580620	8	7	1847	GU206881	9	
5	acs133	$(CTC)_{7}$	37	2016884	7	9	250846	GU206883	21	
6	acs201	$(GT)_{9}$	11	491823	6	5	106985	GU206884	11	
7	acs173	$(TGA)_{6}$	1	11639371	2	3	6424	GU206882	26	
8	acs28	$(GT)_{7}$	11	9588433	3	4	112998	GU206885	20	
9	acse121	$(CA)_{10}$	79	440251	6	8	334041	GU206886	4	
10	acs199	$(GT)_{15}$	29	3536862	6	9	214683	GU206887	6	
11	acs107	$(AC)_{22}$	49	1363541	4	6	282692	GU206888	12	
12	acs16	$(CA)_{18}$	10	563205	9	4	98519	GU206893	3	
13	acs151	$(CA)_{6}$	51	1786036	2	2	286855	GU206880	29	
14	acs4	$(CA)_{15}$	48	277523	10	7	279624	GU206891	8	
15	acs75	$(AC)_{13}$	32	1905545	8	5	232573	GU206897	11	
16	acse138	$(GA)_{12}$	40	4068974	7	8	261164	GU206899	9	
17	acse141	$(TG)_{9}$	1	19443821	8	10	11392	GU206902	17	
18	acs3	$(GT)_{18}$	46	2369548	7	10	274857	GU206903	12	
19	acs78	$(GT)_{11}$	4	15914235	2	13	48205	GU206905	20	
20	acse165	$(GT)_{7}$	10	10950560	7	10	105160	GU206904	21	
21	acse76	$(GAA)_{15}$	8	2630032	11	9	81743	GU206906	18	
22	HJD-118	$(AC)_{20}$	42	2870242	3	2	265111	KT898198	16	Mao.et al. [11]
23	HJD-120	$(GT)_{11}$	7	9873376	4	4	73781	KT898199	6	
24	AC120	$(GT)_{15}$	36	1829946	12	13	247244	GU166141	22	Yang. et al. [10]
25	AC230	$(TG)_{13}$	22	7782175	9	3	185260	GU166147	2	
26	AC146	$(CA)_{13}$	35	3048985	19	8	243990	GU166148	10	
27	AC234	$(CA)_{17}$	105	672407	8	8	363852	GU166149	11	
28	AC221	$(CA)_{14}$	23	6991179	8	5	190163	GU166150	4	
29	AC123	$(GT)_{16}$	3	16419169	18	14	36339	GU166151	8	
30	AC176	$(CA)_{13}$	38	3110607	7	10	255032	GU166152	12	
31	AC87	$(GT)_{12}$	15	6781621	6	8	142664	GU166153	12	
32	AC103	$(CA)_{12}$	62	1948078	14	7	307256	GU166143	7	
33	AC229	$(TG)_{11}$	6	10449514	9	9	64437	GU166144	17	
34	*ACS3*	$(CA)_{16}$	12	12517366	14	8	123998	AB095008	12	Jeong.et al. [26]
35	*ACS4*	$(GT)_{12}$	23	6245505	7	7	189703	AB095009	18	
36	*ACS16*	$(CA)_{28}$	5	12733113	14	10	56481	AB095012	9	
37	*ACS17*	$(CA)_{24}$	4	10548354	11	10	45429	AB095013	8	
38	M414	$(TCTA)_{6}$	4	10637724	5	3	45449	MH782242	19	Wang et al. [8]
39	M448	$(GATA)_{7}$	5	6905294	15	10	53057	MH782245	14	
40	M473	$(TGTC)_{7}$	17	9189964	7	5	157893	MH782247	22	
41	M478	$(AAAC)_{4}$	98	774280	10	9	357636	MH782248	19	
42	M432	$(AAAG)_{8}$	7	7913516	9	5	72443	MH782244	14	
43	AS2-3	$(CA)_{10}$	102	84079	10	11	360499	MG892348	16	
44	AS2-6	$(AC)_{11}$	2	11235336	13	4	21022	MG892351	12	
45	AS2-7	$(AC)_{12}$	54	1364919	12	11	293105	MG892352	21	
46	AS2-9	$(GT)_{9}$	37	4849190	6	6	252389	MG892354	8	
47	AS2-10	$(TG)_{8}$	110	578725	5	5	368019	MG892355	16	
48	AS2-13	$(AC)_{9}$	11	6058827	8	9	110696	MG892358	20	

49	AS3-3	$(CTT)_{18}$	5	5323427	15	7	51997	MG892361	15	Wu et al. [13]
50	AS3-5	$(GAG)_7$	10	652695	2	2	98570	MG892362	27	
51	AS3-6	$(TTG)_8$	7	18401799	12	10	79280	MG892363	25	
52	AS3-7	$(TCA)_8$	5	6963248	6	5	53075	MG892364	19	
53	AS3-10	$(TCC)_8$	25	6897139	4	7	198684	MG892366	29	
54	AS3-12	$(TCC)_7$	84	573558	5	3	341982	MG892368	18	
55	AS3-14	$(AAT)_8$	55	1754052	9	5	295261	MG892369	7	
56	AS3-15	$(AGA)_{12}$	23	5954963	7	5	189508	MG892370	20	
57	AS4-4	$(GAAG)_5$	7	4079411	5	4	69827	MG892373	26	
58	AS4-5	$(AGAT)_5$	36	4281062	10	8	249113	MG892374	17	

scaf.:scaffold; Na-PCR:the number of alleles per locus idetified by PCR; Na-HipSTR:the number of alleles per locus called by HipSTR; A.no.: ncbi accession number; N: the number of genotyped individuals
