# Supplementary material for: Microsatellite Genome-Wide Database Development for the Commercial Blackhead Seabream (Acanthopagrus schlegelii)
Source: Genes (Basel). 2023 Mar 1;14(3):620. doi: 10.3390/genes14030620 (PMC10048070; doi:10.3390/genes14030620)
Supplement: Supplementary file 1 [file genes-14-00620-s001.zip › Table S8. Comparison of genotyping consistency between PCR and HipSTR.pdf]

Table S8. Comparison of genotyping consistency between PCR and HipSTR

loci	Same samples	heterozygotes	homozygotes	same genotype
AS3	6	4	2	6
AS26	6	4	2	4
AS31	10	2	8	7
AS27	5	4	1	4
AS20	9	3	6	8
AS9	6	5	1	5
AS8	11	10	1	11
AS34	10	7	3	10
SSR137989	12	10	2	11
SSR99580	8	6	2	8
SSR153041	10	8	2	9
SSR143492	11	10	1	9
SSR5381	8	5	3	7
SSR145663	11	9	2	8
SSR183796	12	6	6	11
SSR359082	13	8	5	12
SSR24062	9	9	0	8
SSR21025	11	3	8	11
SSR232706	13	8	5	10
SSR274142	11	11	0	11
SSR244447	12	10	2	12
SSR246842	9	4	5	9
Total	213	146	67	191
Proportion(%)	100	68.54	31.46	89.67
