# Supplementary material for: Microsatellite Genome-Wide Database Development for the Commercial Blackhead Seabream (Acanthopagrus schlegelii)
Source: Genes (Basel). 2023 Mar 1;14(3):620. doi: 10.3390/genes14030620 (PMC10048070; doi:10.3390/genes14030620)
Supplement: Supplementary file 1 [file genes-14-00620-s001.zip › Table S9. The polymorphic information content of 63 sites called by HipSTR.pdf]

Table S9. The polymorphic information content of 63 sites called by HipSTR

No.	SSR ID	motif	SCA.	START	END	N	Na	PIC
1	367719	$(GCT)_9$	109	1026512	1026538	32	3	0.5853
2	362903	$(TTG)_7$	104	1011345	1011365	33	4	0.5654
3	387045	$(CCA)_6$	138	583991	584008	34	4	0.4488
4	18688	$(ACA)_7$	2	8067101	8067121	28	4	0.3539
5	294823	$(GT)_7$	55	791498	791511	31	4	0.5404
6	101255	$(CA)_8$	10	4229522	4229537	26	4	0.5314
7	134297	$(TGT)_5$	14	3401208	3401222	31	4	0.4039
8	17392	$(TC)_6$	2	6312724	6312735	31	4	0.4375
9	10951	$(TC)_7$	1	18936689	18936702	32	4	0.5278
10	167153	$(CAG)_6$	19	7029399	7029416	31	4	0.5614
11	6587	$(TGC)_9$	1	11858611	11858637	27	5	0.5430
12	99580	$(ATA)_7$	10	1946756	1946776	26	5	0.6122
13	371520	$(TG)_8$	113	862987	863002	33	5	0.4666
14	17	$(TG)_7$	1	76103	76116	24	5	0.7072
15	3627	$(AC)_9$	1	5883024	5883041	24	5	0.6130
16	370685	$(TC)_7$	112	847454	847467	31	5	0.5784
17	371277	$(TG)_7$	113	598414	598427	25	5	0.6031
18	246813	$(GAAC)_5$	36	983344	983363	32	5	0.6045
19	372065	$(CAC)_5$	114	853290	853304	31	5	0.4229
20	387013	$(TGA)_{10}$	138	532540	532569	24	5	0.6674
21	107491	$(TG)_9$	11	1238319	1238336	23	5	0.5942
22	4462	$(CA)_8$	1	7344660	7344675	27	5	0.5902
23	6459	$(CA)_{11}$	1	11690349	11690370	25	5	0.6380
24	192591	$(CA)_9$	24	4099516	4099533	23	5	0.6037
25	69122	$(AG)_7$	7	2781554	2781567	27	5	0.4764
26	123864	$(TGC)_7$	12	12196300	12196320	30	5	0.5336
27	10641	$(CTC)_8$	1	18564276	18564299	34	5	0.5425
28	132112	$(TG)_9$	14	109879	109896	30	5	0.5716
29	142229	$(AC)_6$	15	5749929	5749940	32	5	0.5007
30	152575	$(GT)_9$	17	1680939	1680956	29	5	0.6303
31	169145	$(AC)_9$	20	1141452	1141469	26	5	0.5061
32	119237	$(GT)_7$	12	5606828	5606841	33	5	0.4740
33	121223	$(GAT)_6$	12	7989508	7989525	34	5	0.6324
34	2452	$(TG)_8$	1	3689054	3689069	27	6	0.6570
35	5381	$(TCT)_7$	1	9282706	9282726	25	6	0.5277
36	127689	$(CA)_9$	13	4042243	4042260	27	6	0.6481
37	127725	$(GT)_{10}$	13	4073487	4073506	28	6	0.4944
38	137989	$(TCC)_6$	14	9752334	9752351	34	6	0.5765
39	132180	$(AC)_9$	14	273760	273777	30	6	0.6691
40	140140	$(AC)_8$	15	2668284	2668299	31	6	0.6263
41	145663	$(TCA)_{11}$	16	552885	552917	26	6	0.7309

42	153448	$(CCT)_6$	17	2968836	2968853	30	6	0.6437
43	2049	$(TG)_8$	1	2992202	2992217	27	6	0.6933
44	149362	$(TTTC)_6$	16	6911061	6911084	25	6	0.6878
45	112919	$(GAT)_9$	11	9436954	9436980	27	6	0.6415
46	373027	$(TTGA)_9$	116	288350	288385	30	6	0.7138
47	24062	$(AGC)_8$	2	16536741	16536764	33	6	0.7431
48	3527	$(GT)_{13}$	1	5659359	5659384	26	6	0.5550
49	120557	$(CA)_9$	12	7008773	7008790	33	6	0.5147
50	101191	$(AC)_7$	10	4097036	4097049	28	6	0.5919
51	153041	$(TCCA)_8$	17	2392775	2392806	27	6	0.7312
52	166169	$(CT)_9$	19	5279101	5279118	31	6	0.6299
53	105284	$(GCA)_5$	10	11237464	11237478	29	6	0.4329
54	102192	$(TG)_8$	10	5825019	5825034	27	7	0.5697
55	106452	$(TG)_7$	10	13519024	13519037	28	7	0.4457
56	108603	$(CA)_8$	11	3118253	3118268	32	7	0.6176
57	133447	$(TC)_{11}$	14	2427150	2427171	27	7	0.6422
58	143492	$(AC)_7$	15	8220430	8220443	23	7	0.7380
59	155655	$(GA)_{10}$	17	5847037	5847056	22	7	0.6981
60	162790	$(TG)_6$	18	8354208	8354219	30	7	0.5604
61	20853	$(GT)_{10}$	2	10975086	10975105	27	7	0.6028
62	367354	$(TCC)_8$	109	585477	585500	29	7	0.6313
63	346526	$(CA)_{11}$	87	1274287	1274308	25	7	0.7936

SCAF: scaffold number; START: motif start point; END: motif end point;
N: the number of genotyped individuals; Na: the number of alleles per locus called by HipSTR；PIC: polymorphic information content.
